# *In Vitro* Assessment of the Interaction Potential of *Ocimum basilicum* (L.) Extracts on CYP2B6, 3A4, and Rifampicin Metabolism

**DOI:** 10.3389/fphar.2020.00517

**Published:** 2020-04-30

**Authors:** Saneesh Kumar, Patrick J. Bouic, Bernd Rosenkranz

**Affiliations:** ^1^Division of Clinical Pharmacology, Faculty of Medicine and Health Sciences, University of Stellenbosch, Cape Town, South Africa; ^2^Division of Medical Microbiology, Faculty of Medicine and Health Sciences, University of Stellenbosch, Cape Town, South Africa; ^3^Synexa Life Sciences, Cape Town, South Africa; ^4^Fundisa African Academy of Medicines Development, Cape Town, South Africa

**Keywords:** herb-drug interactions, LC-MS, basil, phytoconstituents, CYP450, time-dependent inhibition, HepG2, induction

## Abstract

*Ocimum basilicum* L. or basilicum is a common culinary herb, used as a traditional medicine for various medical conditions including HIV/AIDS and tuberculosis, in Africa. The objective of this study was to evaluate the effect of methanol, ethanol, aqueous and ethyl acetate extracts of the dried leaves and inflorescence of *O. basilicum*, on the activity of cytochrome P450 enzymes (CYPs) CYP2B6 and 3A4, as well as esterase-mediated metabolism of rifampicin to 25-*O*-desacetyl rifampicin (25ODESRIF). Human liver microsomes (HLM) were used to evaluate inhibition and CYP2B6/3A4 mRNA expression HepG2 assays were used to measure induction. Furthermore, the phytoconstituents likely involved in causing the observed effect were analyzed using biochemical tests and LC-MS. The aqueous and methanolic extracts showed reversible and time-dependent inhibition (TDI) of CYP2B6 with TDI-IC_50_s 33.35 μg/ml (IC_50_ shift-fold >1.5) and 4.93 μg/ml (IC_50_ shift-fold >7) respectively, while the methanolic and ethanolic extracts inhibited 25ODESRIF formation (IC_50_s 31 μg/ml, 8.94 μg/ml). In HepG2 assays, the methanolic and ethanolic extracts moderately induced CYP2B6, 3A4 mRNA with 38%-, 28%-fold shift, and 22%-, 44%-fold shift respectively. LC-MS full scans identified phenols rosmarinic acid [*m/z* 359 (M-H)^-^, approximately 2298 mg/L in aqueous extract] and caftaric acid along with flavones salvigenin [*m/z* 329 (M+H)^+^, approximately 1855 mg/L in ethanolic extract], eupatorin [*m/z* 345 (M+H)^+^, 668.772 mg/L in ethanolic extract], rutin [*m/z* 609 (M-H)^-^] and isoquercetin [*m/z* 463 (M-H)^-^] and other compounds—linalool [*m/z* 153 (M-H)^-^], hydroxyjasmonic acid [*m/z* 225 (M-H)^-^], eucommiol [*m/z* 187 (M-H)^-^] and trihydroxy octadecenoic acid [*m/z* 329 (M-H)^-^, 530 mg/L in ethanolic extract]. The putative gastrointestinal tract (GIT) concentration for all extracts was calculated as 2,400 μg/ml and hepatic circulation concentrations were estimated at 805.68 μg/ml for the aqueous extract, and 226.56 μg/ml for methanolic extract. Based on the putative GIT concentration, estimated hepatic circulation concentration [I] and inhibition constant K_i_, the predicted percentile of inhibition *in vivo* was highest for the aqueous extract on CYP2B6 (96.7%). The observations indicated that *O. basilicum* extracts may have the potential to cause clinically relevant herb-drug interactions (HDI) with CYP2B6 and rifampicin metabolism *in vivo*, if sufficient hepatic concentrations are reached in humans.

**Graphical Abstract:**
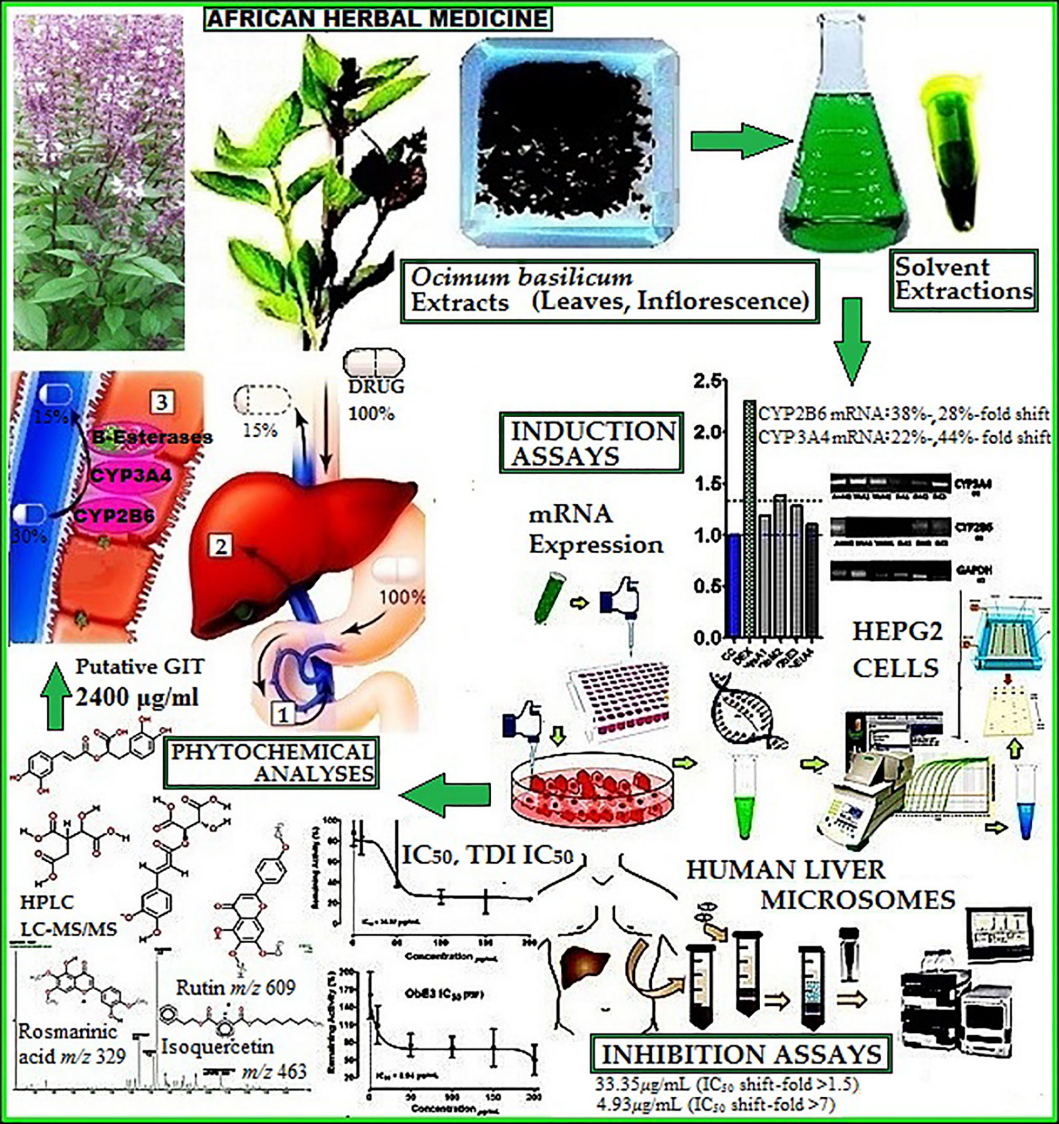


## Introduction

*Ocimum basilicum* L. (Lamiaceae) or sweet basil is a popular culinary and ornamental herb, used for its medicinal properties in Asia and Africa. The herb is native to India, Southeast Asia, New Guinea and many countries in Africa, and a famous ingredient in Ayurveda, Unani and Siddha system of traditional medicine. The distinct characteristic aroma, chemical composition and biological activity of the basil essential oil depend on factors such as morphological variability, topography and other environmental factors (Nurzynska-Wierdak et al., 2012). It is used as an Indian traditional medicine in the supplementary treatment of asthma, diabetes and stress (Duke, 2008). Ethnic communities in Africa and India use whole basil plant decoctions in patients with tuberculosis (TB) (Rai, 2016; Dzoyem et al., 2017). The volatile oil of basil comprises of components such as eugenol (Akgül, 1989), geraniol, eucalyptol, fenchone, and estragole (Muráriková et al., 2017), some of these compounds being used as a local antiseptic and anaesthetic (Jadhav et al., 2004). Previous studies have shown anti-TB activity of the crude methanolic extract from the aerial parts (leaves, fruits, and flowers) of basil (Siddiqui et al., 2012). *In vitro* studies have shown that phytocompounds in the oil have potent antioxidant, antiviral, and antimicrobial properties, and have been tested in cancer treatment (Chiang et al., 2005; Bozin et al., 2006; Manosroi et al., 2006; de Almeida et al., 2007). Esters and amides synthesized from dichloromethane extract of basil have been tested *in vitro* using an HIV-1 Reverse Transcriptase (RT)-associated RNase H inhibition assay; tetradecyl ferulate inhibited RNase H with IC_50_ 12.4 μM and N-oleylcaffeamide strongly inhibited the RT-associated activity of ribonuclease H and DNA polymerase (Sonar et al., 2017).

Due to the use of this herb as a common spice as well as its availability in the pharmacies as a liquid/powder extract and pure essential oils for various health conditions, there is a potential for concomitant administration with conventional drugs, hence potential for herb-drug interactions (HDI). The phytoconstituents within the herbs can potentially inhibit or induce the activity of drug-metabolizing enzymes and transport proteins. Recent studies have examined the inhibitory and inducing effects of various West African, Chinese, South American and Indian herbs and their extracts or formulations, such as *Uncaria tomentosa* (Willd. ex Schult.) DC. (Weiss, 2019), *Astragalus mongholicus* Bunge (Kumar et al., 2018), *Momordica charantia* L. (Fasinu et al., 2017), and *Curcuma longa* L. and *Phyllanthus emblica* L. (Shengule et al., 2018), on cytochrome P450 activities.

Previous studies showed the inhibitory effect of the methanolic extract of basil on the activity of CYP2D6, CYP3A4, CYP3A5, and CYP3A7 (Nguyen et al., 2014). Another study using MROD assay (7-methoxyresorufin dealkylation) showed the inhibitory effect of methanol-dibutyl ether extract from basil on the CYP1A2 mediated metabolism of methoxyresorufin to its fluorescent metabolite resorufin (Jeurissen et al., 2007). Other interactions reported include the induction of CYP2A6, 2C9, 2D6 and 2E1 by safrole and estragole present in basil extracts, to form carcinogenic 1′-hydroxy metabolites (Jeurissen et al., 2004; Jeurissen et al., 2007).

Systematic review studies have reported the high HIV/TB-burden in African countries where coinfected patients are treated with efavirenz and rifampicin-isoniazid regimens (Atwine et al., 2018). The current WHO TB-HIV treatment guideline for the effective dosage of efavirenz in patients during concomitant rifampicin-based anti-TB therapy is 600 mg/day; this being confirmed in previous studies in the sub-Saharan Africa (Bhatt et al., 2014; Habtewold et al., 2015). However, with the possibility of drug-drug interactions involving CYP2B6 and 3A4, it is necessary to analyze if the coadministration of basil in such scenarios can cause clinically significant herb-drug interactions. For example, artemisinin extract obtained from the Chinese herb *Artemisia annua* L., used in malarial treatment and metabolized by CYP2B6 and 3A4, has been studied to cause potential toxicities *in vitro* when coadministered with drugs such as orphenadrine (Hedrich et al., 2016), which could be attributed to CYP2B6/3A4 inhibition and incomplete metabolism.

CYP2B6 predominantly metabolizes efavirenz to its primary metabolite 8-hydroxy efavirenz with the involvement of CYP3A to a lesser extent, and CYP2A6-mediated metabolism to 7-Hydroxy efavirenz. CYP2B6 plays a critical role in efavirenz metabolism, forming the secondary metabolite 8,14-dihydroxy efavirenz from 8-hydroxy efavirenz (Ward et al., 2003). Previous studies have shown the significance of various factors such as ethnicity and pharmacogenetic variations (CYP2B6 alleles) in influencing the efavirenz pharmacokinetics in HIV/AIDS patients in Africa (Swart et al., 2012; Ngaimisi et al., 2013). CYP3A4 catalyzed metabolism pathway devises a major route for elimination of many drugs and the induction or the inhibition of its expression by other drugs or herbs is often implicated in clinically significant interactions. Many drug-drug interaction (DDI) studies related to drug-resistant TB and TB/HIV co-infection analyzed the involvement of CYP3A4 as a key enzyme (Kwara et al., 2010).

Research has been done to explore the effects of rifampicin as an inducer in herb and drug-drug interaction studies; however the effect of HDI on the β-esterase-mediated metabolism pathway of rifampicin to 25-*O*-desacetyl rifampicin has not been investigated. Assessing its metabolism profile and the potential of the herbs to induce or inhibit the formation of 25-*O*-desacetyl rifampicin is critical, because if the normal pharmacokinetics of this metabolism pathway is affected, incomplete metabolism of rifampicin may result in toxicity and fatal poisoning (Cheng et al., 1988; Sridhar et al., 2012). Case studies have reported reversible hepatic, renal damage and fatal poising with the ingestion of 9–12 g and 14–15 g of rifampicin (Plomp et al., 1981; Cheng et al., 1988; Marks et al., 2009). The high burden of rifampicin toxicities among HIV/TB co-infected patients (Gort et al., 1997; Yee et al., 2003) often contributes to morbidity and mortality; anti-TB drug induced liver injury in China being an example (Shang et al., 2011).

This research study investigated the relevant phyto-compounds present in the dried leaves and inflorescence of *O. basilicum* using various biochemical tests, LC-MS and their interactions with CYP2B6, 3A4, and rifampicin metabolism (β-esterases) in HLM and HepG2 cells. The potential clinical relevance of the findings was assessed by *in vivo* predictions of the inhibitory potential of the extracts in the GIT.

## Materials and Methods

### Reagents and Chemicals

The biochemical tests were performed using the following reagents:

- Dilute ammonia solution (Hopkin and Williams, England).
- Vanillin reagent: 1% vanillin in 70% concentrated sulphuric acid (BDH Chemicals, England).
- Wagner’s reagent: 2 g of iodine (BDH Chemicals, England) and 6 g of potassium iodide (Merck, Germany) dissolved in 100 ml of water.
- Neutral ferric chloride solution, 0.1% ferric chloride solution (Sigma-Aldrich, Steinheim, Germany).
- 10% Sodium hydroxide solution (BDH Chemicals, England).
- Magnesium solution (Hopkin and Williams, England).
- Glacial acetic acid, concentrated sulphuric acid (BDH Chemicals, England), ferric chloride (Sigma-Aldrich, Germany).
- Chloroform, acetic anhydride (BDH Chemicals, England).

Efavirenz, rifampicin, ticlopidine and nelfinavir mesylate hydrate were obtained from Sigma-Aldrich (Steinheim, Germany), while pure 8-hydroxy efavirenz, 25-*O*-desacetyl rifampicin and neostigmine methyl sulphate were obtained from Clearsynth Labs Ltd. (Mumbai, India).

HPLC-grade methanol (Sigma-Aldrich, Germany), ethanol (Merck KGaA, Darmstadt, Germany), purified HPLC-grade water (Adrona B30 purification systems, Adrona SIA, Latvia), and ethyl acetate (BDH Chemicals, England) were used for the extractions and LC-MS mobile phase solvents.

For the HLM inhibition assays, magnesium chloride, glucose-6-phosphate sodium salt, glucose-6-phosphate dehydrogenase, phosphate buffer solution 1 M, and β-nicotinamide adenine dinucleotide phosphate hydrate (NADPH) were purchased from Sigma-Aldrich (Steinheim, Germany). For the induction assays, microtiter 96–well U–bottom plates from Tarsons Products Pvt. Ltd., Kolkata, India were used. 25 cm^2^ cell culture flasks were purchased from Corning^®^ Inc. (New York, US). MTT (thiazolyl blue tetrazolium bromide), phosphate buffered saline pH 7.4 (PBS), nutrient mixture F-12 Ham, EDTA, Dulbecco’s modified Eagle’s medium—high glucose (DMEM), trypsin, and the antibiotics for cell culture were purchased from HiMedia Laboratories (Mumbai, India). Gibco™ Fetal bovine serum (FBS) was purchased from Thermofisher Scientific (MA, USA). Tri-Xtract™ for the RNA isolation was purchased from G-Biosciences Ltd. (MO, US). Dimethyl sulfoxide (DMSO) was purchased from Finar Ltd. (Gujarat, India).

### Plant Material

The dried leaves and inflorescence of *O. basilicum* (Lamiaceae) were obtained in powdered and packed form, from Pharma Germania, Benoni, South Africa (Certificate of Analysis# PFI-2645/08/2014, country of origin—Egypt).

### Preparation of Plant Extracts

The dried leaves and inflorescence of *O. basilicum* were weighed (4 g) andextracted exhaustively after boiling with purified water (Adrona B30, up to 500 ml for 9 days). Forthe other solvent extractions, 4 g of the herb was added to methanol, ethanol and ethyl acetate(HPLC grade), and extracted exhaustively using mechanical agitation (up to 500 ml for 9 days). The extract was filtered and evaporated at 50°C using a concentrator-freeze drier (miVac, England) to complete dryness and stored in sealed glass containers in a vacuum desiccator, at 2–4 °C. Percentage of yield was calculated as per equation (2.3.1):

Eq. (2.3.1) $$\mathrm{Extract}\%\mathrm{yield}=(W_{1}/W_{2})\times 100$$

Where, W_1_ is net weight of basil extract in grams after extraction and W_2_ is total weight of dried basil in grams taken for extraction.

### Human Liver Microsomes and HepG2 Cell Lines

The HLM assays were performed using H0630—pooled human liver microsomes (mixed gender, protein concentration: 20 mg/ml) obtained from Sekisui Xenotech LLC (Kansas, USA). HepG2 (human hepatocellular carcinoma cells) cell line was procured from National Centre for Cell Sciences (NCCS), Cell Repository, Pune, India.

### Analytical Instrumentation Settings

LC-MS phytochemical fingerprinting analyses were performed using Waters Synapt G2 Quadrupole time-of-flight (QTOF) mass spectrometer (MS) connected to a Waters Acquity ultra-performance liquid chromatograph (UPLC) (Waters, Milford, MA, USA). Waters HSS T3, 2.1 × 100 mm, 1.7 μm column was used for the separation. A cone voltage of 15 V for both positive and negative mode ionizations, desolvation temperature of 275°C, and desolvation gas at 650 L/h (Stander et al., 2017). Data were acquired by scanning all extracts, from 150 to 1,500 *m/z* in resolution mode as well as in MS^E^ mode. In MS^E^ mode two channels of MS data were acquired, one used low collision energy (4 V) and the second one at collision energy ramp in the range 40−100 V, to obtain fragmentation data as well. Sodium formate was used to calibrate the UPLC-MS and leucine enkaphalin was used as reference mass (lock mass) for accuracy in mass determination; Waters HSS T3, 2.1 × 100 mm, 1.7 μm column was used for the separation.

For the HLM assay sample analyses, Waters Alliance 2695 HPLC system coupled with 2996 PDA detector was used. A C-18 Phenomenex-Evo column (150 x 2.6 mm, 3.5 μm) and a C-18 Phenomenex Luna column (150 x 4.6 mm, 5 μm) was used for separating efavirenz and 8-hydroxy efavirenz, and rifampicin and its metabolite, respectively. PDA wavelength was set at 245 nm for efavirenz assay sample analyses and 254 n for rifampicin assay sample analyses. The gradient solvents elution program was set as (Time*_min_*_/_% solution B) at 0/10, 5/80, 10/95, 9/80, and 11.5/10 (Varghese et al., 2014; Kumar et al., 2017).

AE-series inverted microscope (Motic Asia, Hong Kong) was used for tissue culture inspection. BioTek Epoch automated microplate reader with Gen5 2005 software v1.10.8 (BioTek Instruments, Inc. USA) was used for plate incubations and readings. Polymerase chain reactions were done using the MJ mini thermocycler (Bio Rad, Hercules, CA, USA).

### Data Analysis

Non-linear regression graph plots for determining the IC_50_ and statistical analyses were performed using GraphPad Software Inc. (San Diego, CA; www.graphpad.com) Prism version 5.00 for Windows, was used.

For gel electrophoresis applications, inGenius—gel documentation system comprising of GeneTools analysis software (Syngene, MD, USA) was used for digital imaging and relative sample expression levels.

### Biochemical Phyto-Profiling

The following standard methodologies were followed for biochemical tests (Harborne, 1973; Raaman, 2006; Iqbal et al., 2015):

1. Test for alkaloids
  - Harborne Test
    About 170 μl of dilute ammonia solution was added to 200 μl of test solution of each basil extract followed by addition of few drops of concentrated sulphuric acid. Formation of yellow coloration indicated the presence of alkaloids.
  - Wagner’s Test
    To 500 μl of plant extract solution, equal amount of Wagner’s reagent was added. The test result was observed. Formation of reddish-brown coloration ascertained the presence of alkaloids.
2. Test for saponins
  About 200 μl of the plant extract was mixed with 170 μl of pure distilled water and shaken vigorously for a stable persistent froth, which indicated the presence of saponins.
3. Test for phenols
  To 200 μl of the plant extract solution, 150 μl of neutral ferric chloride solution was added. Formation of greenish colour showed the presence of polyphenols.
4. Test for tannins
  To 200 μl of the plant extract solution, 150 μl of 0.1% ferric chloride was added and observed for the formation of a bluish-black precipitate, which indicated the presence of tannins in the extract.
5. Test for glycosides (Keller-Kiliani Test)
  To 200 μl of the plant extract solution, 150 μl of glacial acetic acid was added. To the resultant, a pinch of ferric chloride along with 100 μl of sulphuric acid was added. Formation of a prominent brown ring showed the presence of glycosides.
6. Test for terpenoids (Salkowski Test)
  About 200 μl of the plant extract was mixed with 75 μl of chloroform, and 125 μl of concentrated sulphuric acid was carefully added from the sides of the test-tube to form a reddish-brown layer, which indicated the presence of terpenoids in the plant extract.
7. Test for flavonoids
  To 200 μl of the plant extract solution, equal amount of Vanillin reagent was added. Formation of reddish-brown colour precipitate indicated the presence of flavonoids in the extract.
8. Test for steroids (Leibermann-Burchard Test)
  To 200 μl plant extract solution, 150 μl of chloroform was added. Then 3-4 drops of acetic anhydride and three drops of concentrated sulphuric acid were added. Formation of a dark-bluish precipitate confirmed the presence of phytosterols in the extract.
9. Test for coumarins
  To 200 μl plant sample extract solution, equal quantity of 10% sodium hydroxide solution was added and heated at 100 °C for 5 min. Formation of yellow color indicated the presence of coumarins in the plant extract.

### Inhibition Assays

#### Validation of HLM Assays for Efavirenz and Rifampicin

**Figure 1:**
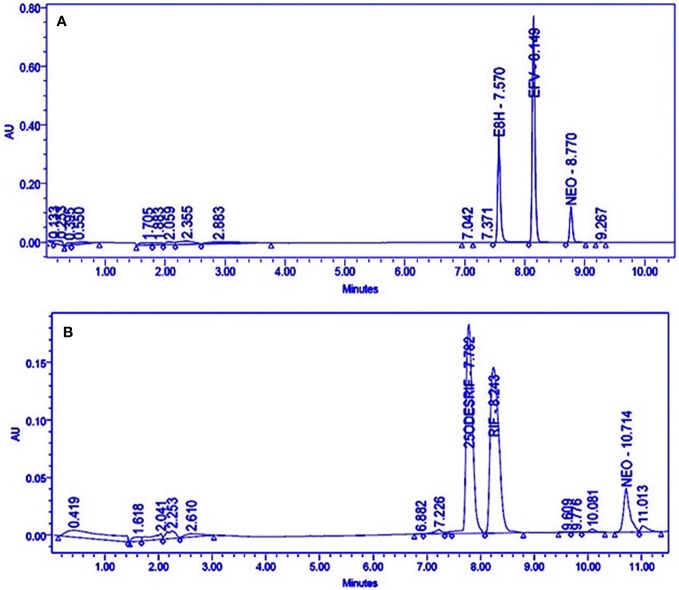
HPLC Chromatograms showing the separation of **(A)** Efavirenz (EFV), its metabolite (E8H), and internal standard (NEO), **(B)** Rifampicin (RIF), its metabolite (25ODESRIF), and the internal standard (NEO). EFV, efavirenz; E8H, 8-Hydroxy efavirenz; NEO, neostigmine; RIF, Rifampicin; 25ODESRIF, 25-*O*-desacetyl rifampicin (Kumar et al., 2019).

Satisfactory separation of the drug, its metabolite and the internal standard was achieved using gradient elution (**Figure 1**).

Method optimization was achieved by modifying various run parameters such as change in gradient elution and, inner column diameter (2.6 and 4.6mm), length (100, 150, and 250mm), and particle size (3.5 μm and 5 μm).

A linear response was obtained in the concentration range 0–200 μM for both efavirenz and its metabolite (R^2^ = 0.9930). LLOD and LLOQ were calculated at 7.57 μM and 22.95 μM for efavirenz and 7.99 μM and 24.24 μM for 8-hydroxy efavirenz, respectively. The peak area of the internal standard neostigmine was relatively constant for all time-point incubations. For the rifampicin method, a linear response was obtained in the concentration range 0–200 μM for both rifampicin and 25-*O*-desacetyl rifampicin (R^2^ = 0.9950). LLOD and LLOQ were calculated at 5.86 μM and 17.75 μM for rifampicin and 7.78 μM and 23.57 μM for 25-*O*-desacetyl rifampicin, respectively (**Table 1**). Linearity was established for both time-variant HLM assays with R^2^ = 0.9918 (Varghese et al., 2014; Kumar et al., 2017).

**Table 1:** HPLC method parameters for efavirenz and rifampicin.

Column	Phenomenex-Evo C-18 100A Column (150 x 4.6 mm, 2.6 μm)			Phenomenex Luna C-18 Column (150 x 4.6 mm, 5 μm)		
**Drug**	EFV	E8H	NEO	RIF	25ODESRIF	NEO
**Retention Time (min)**	7.57	8.15	8.77	7.70	8.25	10.70
**LLOD (μM)**	7.57	7.99	–	5.86	7.78	–
**LLOQ (μM)**	22.95	24.24	–	17.75	23.57	–
**Linear Correlation Coefficient (R^2^)**	0.9906	0.9948	–	0.9932	0.9976	–
**Overall Run Time (min)**	10.5			11.5		
**HLM Time-variant assay (15-60 min): Linear Correlation Coefficient (R^2^)**	0.9934			0.9901		

EFV, efavirenz; E8H, 8-hydroxy efavirenz; NEO, neostigmine; RIF, Rifampicin; 25ODESRIF, 25-O-desacetyl rifampicin (Kumar et al., 2019).

**Figure 2:**
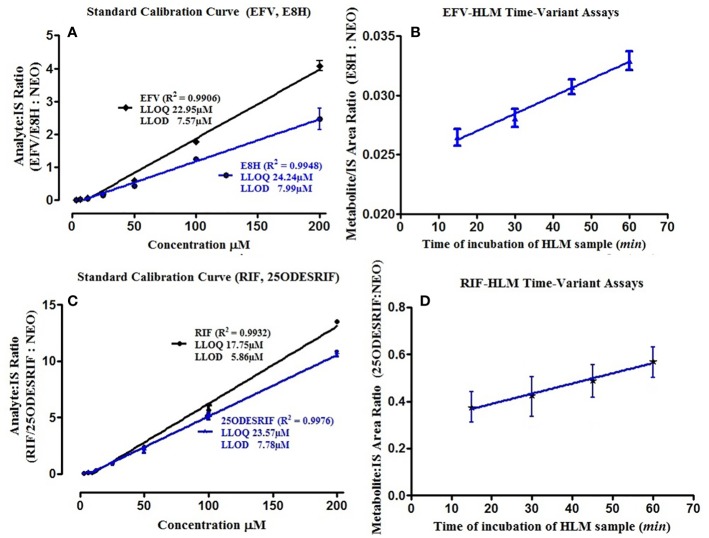
**(A)** Standard calibration curves for pure EFV and E8H standards; **(B)** HLM time variant incubation assay linearity showing the ratio of E8H to NEO, for the specific time of incubation (15, 30, 45, and 60 min), **(C)** Standard calibration curves for pure RIF and 25ODESRIF standards **(D)**; HLM time variant incubation assay linearity showing the ratio of 25ODESRIF to NEO, for the specific time of incubation (15, 30, 45, and 60 min). EFV, efavirenz; E8H, 8-hydroxy efavirenz; NEO, neostigmine; RIF, Rifampicin; 25ODESRIF, 25-*O*-desacetyl rifampicin.

Four incubation time points (15, 30, 45, and 60 min, in triplicates) were selected for the *in vitro* human liver microsomal incubation assays for efavirenz and rifampicin. The metabolites for both drugs were detected, separated and quantified (peak area) along with the internal standard neostigmine, at consistent retention times using the above method parameters; the peak area of neostigmine for the assays were relatively constant (**Figure 1A, B**). Linearity was attained for the 15–60 min time-point incubations based on the ratio of the metabolite to the internal standard, with R^2^ = 0.9934 for efavirenz and R^2^ = 0.9901 for rifampicin, respectively (**Figure 2**).

#### Kinetics of Efavirenz and Rifampicin

**Figure 3:**
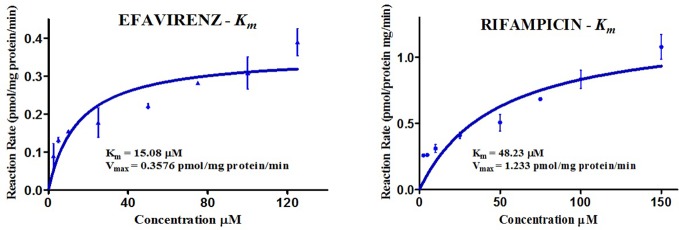
Michaelis-Menten kinetic plots (K*_m_*) of efavirenz and rifampicin in human liver microsomes.

The kinetics for the formation of 8-hydroxy efavirenz from efavirenz, and 25-*O*-desacetyl rifampicin from rifampicin were determined through several HLM incubations for concentrations in the range of 0–150 μM. Representative Michaelis-Menten kinetic plots from all assays were as illustrated below (**Figure 3**, **Table 2**).

**Table 2:** Kinetics of efavirenz and rifampicin metabolism in HLM.

HLMs	Enzyme	SUBSTRATE	K_m_^*^	V_max_	CL_int_ (V_max_/K_m_)
Pooled HLM—Mixed Gender, Xenotech (H0610, H0620, H0630, H0640)	CYP2B6	EFV	15.08	0.3576	0.0240
	β-esterases	RIF	48.23	1.2330	0.0260

*V_max_, pmol/min/mg protein or pmol/min/pmol P450; K_m_, μM; CL_int_, μl/min/mg protein or μl/min/pmol P450 (Kumar et al., 2017; Kumar et al., 2018; Kumar et al., 2019).

#### IC_50_ and TDI Assays

The inhibitory potential of each extract was assessed using two-point screening (20 and 200 μg/ml) and the activity potential was compared with the control incubates (without inhibitor). Neostigmine was used as the internal standard.

Briefly, a standard 200 μl incubation mixture containing the liver microsomes (0.5 mg/ml protein concentration for efavirenz, 0.25 mg/ml protein concentration rifampicin), efavirenz (15 μM)/rifampicin (48 μM) in 0.2M phosphate buffer (pH 7.4) and the basil herb extract (final concentration 10 or 200 μg/ml, dissolved in <1% solvent) was incubated at 37°C for 30 min, in triplicates, with master reaction mix comprising of NADPH (final concentration 1.3 mM) and other reaction co-factors such as glucose-6-phosphate (final concentration 1.3 mM), glucose-6-phosphate dehydrogenase (1 U/ml) and magnesium chloride (final concentration 3.3 mM). About 200 μl of chilled ice-cold acetonitrile spiked with neostigmine (20 μM) was used to terminate each reaction. These samples were then centrifuged at 13,000 rpm for 10 min and the supernatants were subjected to HPLC analysis. The mobile phase comprised of water (A): acetonitrile (B) at a flow rate of 0.7 ml min^-1^ for efavirenz samples and water (A): methanol (B) at a flow rate of 0.8 ml min^-1^ for rifampicin samples. Ticlopidine and nelfinavir were used as the standard inhibitors for CYP2B6 and rifampicin metabolism pathway (Polsky-Fisher et al., 2006; Flockhart, 2007). The percentage of remaining activity was expressed as in the equation below (equation 2.8.3.1):

Eq. (2.8.3.1) %remaining activity=(Test−test control)/(Control−control blank)×100%

A concentration range of 1–200 μg/ml of each active basil extract (six-point screening), in triplicates, was used to determine the IC_50_. Ticlopidine and nelfinavir were screened in within concentration ranges 1–100 μM (0–66.30 μg/ml) to determine their IC_50_ values. The percentage of remaining activity was plotted on GraphPad prism against the log-transformed concentrations of the herbal extract or the positive control, using non-linear dose-response inhibition regression analysis to obtain the sigmoidal curves for the IC_50_s.

For the TDI assays, the active basil extracts were pre-incubated with HLM and buffer (90 μl) and the co-factors (reaction master mix, 100 μl) at 37°C for 30 min prior to addition of 10 μl of the substrate (15 μM efavirenz or 48 μM rifampicin).

The TDI fold-shift was calculated using the ratio of the IC_50_s of the normal assay IC_50_(-) to that of the pre-incubation assay IC_50_(+), with NADPH (equation 2.8.3.2).

Eq. (2.8.3.2) $${\mathrm{TDI}}_{\text{fold-shift}}=\text{normal assay}{\mathrm{IC}}_{50}(-)/\text{pre-incubation assay}{\mathrm{IC}}_{50}(+)$$

Extracts with fold shifts ≥1.5 were classified positive for TDI (Nomeir et al., 2004).

The dose-response curves in sections *Preparation of Plant Extracts* and *Human Liver Microsomes and HepG2 Cell Lines* represent the IC_50_ (μM) calculated using non-linear regression (dose-response inhibition) v/s the actual IC_50_ plot curve-fit.

#### Hepatic Blood Concentrations—Prediction on Inhibition Percentage

The concentrations of each basil extract in the GIT and in hepatic blood were estimated using the percentage yield (% ^W^/_W_, section *Reagents and Chemicals*) on the basis of a basic model (F.D.A., 2017) where the maximal unbound plasma concentration of the interacting herb [I] was calculated using an estimated available GIT fluid of 250 ml (Mudie et al., 2014; Thomford et al., 2016) and the recommended single dosage of each extract obtained from various online sources (https://www.drugs.com; https://draxe.com/; http://naturimedica.com) and the label insert instructions obtained for the crude basil extracts obtained from Pharma Germania, Benoni (equation 2.8.4.1).

Eq. (2.8.4.1) $${\text{Putative GIT Conc. }}_{\left(\text{μ}g/\mathrm{ml}\right)}=({\text{Recommended single dose}}_{\left(\text{μ}g/\mathrm{ml}\right)})/250$$

The available hepatic blood concentration of the extract [I] was calculated using the putative GIT concentration value, based on the equation (2.8.4.2).

Eq. (2.8.4.2) $$\text{Estimated Hep. Blood Conc. }[I]{\;}_{\left(\text{μg}/\mathrm{ml}\right)}=(\%\text{yield}*{\text{Putative GIT Conc. }}_{\text{μ}g/\mathrm{ml})})/100$$

The inhibition constant (K_i_) for each extract was calculated based on the IC_50_ values on the assumption that most documented CYP inhibitions are competitive, as per the following equation (2.8.4.3):

Eq. (2.8.4.3) $${\mathrm{IC}}_{50}=K_{i}(1+[S]/K_{m}),\text{ when }[\text{S}]=K_{m},\;K_{i}={\mathrm{IC}}_{50}/2$$

S and K_m_ values denote the substrate concentration used in this study and the affinity constant for the metabolic activity, respectively [15]. Likely hepatic HDI predictions for the basil extracts were assessed and evaluated based on comparison of the estimated concentration hepatic blood to the IC_50_ value for each extract and the predicted percentage of inhibition was calculated using the inhibitory concentration [I] as per the following equation (2.8.4.4).

Eq. (2.8.4.4) $$\text{Predicted }\%\text{ inhibition }=([I]/([I]+K_{i}))*100$$

The herbs were ranked for their potential risk in causing HDI based on the inhibitory potency ([I]/K_i_ inhibitory ratio). According to the FDA guidelines, [I]/K_i_ > 1.0 is correlated to high risk of potential DDI, [I]/K_i_=0.1–1 is correlated to intermediate risk for DDI and [I]/K_i_ < 0.1 is unlikely to cause any significant interactions (Prueksaritanont et al., 2013). This study did not use static and dynamic mechanistic models to evaluate the plasma concentration-time curve ratio (AUCR) for the target drugs in the presence of the herbal extracts, as recommended in the FDA clinical pharmacology guidelines (F.D.A., 2017).

### Induction Assays

#### Cytotoxicity Testing and Determination of CC_50_

Stock solution of each basil extract was prepared in DMEM medium supplemented with 2% inactivated FBS (10% w/v concentration) and filtered using 0.22 µm syringe filter. Serial two-fold dilutions were prepared from this for carrying out the cytotoxicity studies. HepG2 cells were cultured in DMEM supplemented with 10% inactivated FBS, penicillin (100 IU/ml), streptomycin (100 μg/ml) and amphotericin B (5 μg/ml) in a humidified atmosphere (5% CO_2_) at 37°C until confluency was attained (Freimoser et al., 1999).

Cytotoxicity of the plant extracts was evaluated based on the method described in a previous study on *Plectranthus barbatus* Andrews (Nagarajappa et al., 2016). In brief, HepG2 cell suspension was added to 96-well microtitre plate and after 24 h, the supernatant was flicked off, the monolayer formed was washed with medium and 100 μl of each extract was added; the plates were incubated in 5% CO_2_ atmosphere at 37°C for 72 h. Post this, the solution in each well was discarded and 50 μl of tetrazolium dye (MTT) in PBS was added; plates were incubated for 3 h. Post this, 100 μl iso-propanol was added and absorbance was measured at 540 nm using plate reader. The growth inhibition percentage was calculated as per the following equation (2.9.1.1):

Eq. (2.9.1.1) $$\%\text{growth inhibition}=({\text{Control}}_{\text{absorbance}}-{\text{test}}_{\text{absorbance}})/{\text{Control}}_{\text{absorbance}}\times 100$$

The dose-response curves against cell lines were used to determine the half-cytotoxicity concentration (CC_50_) (Tukappa et al., 2015).

#### mRNA Expression for CYP2B6 and 3A4

CC_50_ concentration of each extract was added to 60 mm petridish comprising of the HepG2 cells cultured in DMEM medium, FBS and amphotericin (48 h) and incubated for 24 h. Total cellular RNA was isolated from the untreated (control) and treated cells using Tri-Xtract™ as per the protocol provided by the manufacturer (G-Biosciences Ltd). cDNA was synthesized from each isolated RNA by reverse transcriptase kit (Thermo Scientific Ltd. protocol). Primers for CYP3A4 and CYP2B6 were selected as per a method developed previously for analysing the modulation of CYPs (Park et al., 2009). 50 μl of the reaction mixture (1x cDNA synthesis buffer, dithiothreitol (0.5 M), RiboLock RNAse inhibitor (20 U), deoxynucleotide mix (1.6 mM), oligo dT (100 ng), reverse transcriptase (25 U), and total RNA) was subjected to PCR for amplification of hepatic cells. cDNAs using specifically designed primers (procured from Eurofins, India) were used. The house keeping gene glyceraldehyde 3-phosphate dehydrogenase (GAPDH) was co-amplified with each reaction as internal control. Rifampicin (50 μM) and dexamethasone (10 μM) were used as positive controls for CYP3A4 and CYP2B6, respectively (Nagarajappa et al., 2016). For CYP3A4 oligo dT primer was used for first strand synthesis and for second strand synthesis, 5′ ATTCAGCAAGAAGAACAAGGACA 3′ and 5′ TGGTGTTCTCAGGCACAGAT 3′ were used as the forward and reverse primers, respectively. For 2B6, oligo dT primer was used for first strand synthesis and for second strand synthesis, 5′ ATGGGGCACTGAAAAAGACTGA 3′ and 5′ AGAGGCGGGGACACTGAATGAC 3′ were used as the forward and reverse primers, respectively.

The amplified samples were further analyzed using agarose gel electrophoresis. The gel was further developed using UV illumination-digital imaging, and Syngene inGenius documentation system and GeneTools analysis software was used for calculating the expression levels per sample; one-way analysis of variance, followed by Dunnett’s multiple comparison tests, by fixing the significance level at p < 0.05, p < 0.01 and p < 0.001 was used (Nagarajappa et al., 2016).

### LC-MS Conditions: Phyto-Profiling

Stock solutions were prepared by adding 8-10 mg of each extract to 1 ml of 50% methanol in water containing 2% formic acid, followed by dissolution in an ultrasonic bath (0.5 Hz, Integral Systems, RSA) for 20 min at room temperature. The extracts were then centrifuged and supernatants were analyzed for phytocomposition. The reference standards quercetin and gallic acid (Vallverdú-Queralt et al., 2015; Ramos et al., 2017) were prepared in cocktail stock solutions with concentration of 200 mg/L of each standard. 2 μl of each extract was injected into the LC-MS prepped with a mobile phase comprising of 0.1% formic acid (solvent A) and acetonitrile containing 0.1% formic acid as solvent B. A flow rate of 0.3 ml min^−1^, was maintained for gradient elution starting with 100% solvent A for 1 min, which was linearly changed to 28% B over 22 min, then changed to 40% B over 50 seconds followed by a 1.5 min wash step with 100% solvent B, and finally re-equilibration (to initial conditions) for 4 min. The column temperature was maintained at 55°C. The PDA wavelength range was set between 230 and 600 nm.

**Figure 4:**
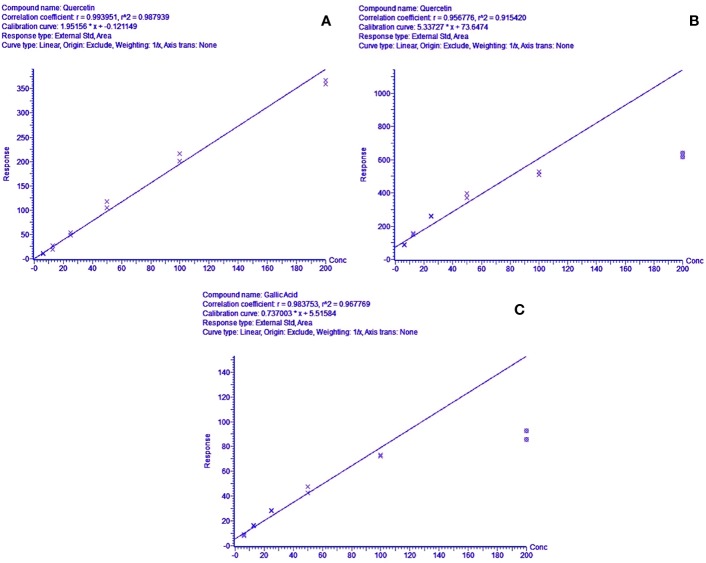
**(A)** Concentration range linearity for quercetin (R^2^ = 0.9879) in positive mode LC-MS scan, **(B, C)**—regression line-fit concentration range linearity for gallic acid & quercetin (R^2^ = 0.9441 & 0.9678) in LC-MS negative mode scans, respectively.

The methods were tested for accuracy and linearity. A linear response was obtained for quercetin for the positive mode run, in the range of 200.000–6.250 mg/l (R^2^ = 0.9900) (**Figure 4**). This concentration range was selected to identify and compare the peak-retention factors and *m/z* of the extract with the reference standard, along with reasonable approximations of the relative amounts of the identified peaks using the standard calibration curve of quercetin. The calibration curve showed slight non-linearity at higher concentrations (200 mg/l) for both standards in the negative mode. A quadratic linear curve fitting model was used for quercetin (R^2^ = 0.9879) for relative quantification of the unknown phytocompounds, based on the peak area (response) for the concentration range used (200.000–6.250 mg/L) whereas a linear fit model was used for gallic acid (R^2^ = 0.9680) since the peak area (response) was less for low concentrations.

Tentative identification of the phytocompounds was done based on the following parameters (Stander et al., 2017):

- Accurate masses
- *m/z* transitions (MS/MS fragments)
- UV maxima
- Relative retention times and comparison with literature review on matching compounds

Online mass spectral repositories such as Metlin Scripps (https://metlin.scripps.edu/), MassBank online Spectral Database (https://massbank.eu/MassBank/), NIST standard reference data online webbook library (http://webbook.nist.gov/chemistry/mw-ser.html), and Pubchem chemistry database (https://pubchem.ncbi.nlm.nih.gov).

## Results

### Extraction and Yield of Basil Extracts for Bioassays

Exhaustive extraction of basil herb was done with water, methanol, ethanol and ethyl acetate; per 4g of the dried basil extract, highest solvent yield was observed in the aqueous extract (Basil_Aq_) of basil with 33.57%, followed by the methanol solvent (Basil_MeOH_) at 9.44% and ethyl acetate (Basil_EtOAc_) at 4.93%. Ethanol extract (Basil_EtOH_) yield was the least with 2.94% (**Table 3**).

**Table 3:** Extraction yield of basil extracts.

Yield (^±^ mg, % ^W^/_W_)			
Aqueous extract (Basil_Aq_)	Methanol extract (Basil_MeOH_)	Ethanol extract (Basil_EtOH_)	Ethyl acetate extract (Basil_EtOAc_)
^±^ 1343 mg, 33.57%	^±^ 378 mg, 9.44%	^±^ 118 mg, 2.94%	^±^ 193 mg, 4.83%

^±^mg - approximate, negating the residual amount of extract retained in the glass tubes after scraping.

### Biochemical Phytoprofiling

The biochemical qualitative tests confirmed the presence of phytoconstituents such as alkaloids, glycosides, terpenoids, phenols, coumarins and flavonoids within the extracts; precipitate formation and color intensity formed the basis for the chemical tests (**Table 4**). All four basil extracts showed positive to the detection tests for all compounds, coumarins and phytosteroids present in trace amounts.

**Table 4:** Biochemical qualitative profile of basil extracts.

Sl #	Phyto Constituent	Test	Reference	Extract	Inference
1 a.	Alkaloids	Harborne Test	*Biochemical Phyto-Profiling*—1, a	Basil_Aq_	✓ ++
				Basil_MeOH_	✓ +++
				Basil_EtOH_	✓ ++
				Basil_EtOAc_	✓ ++
1 b.		Wagner’s Test	*Biochemical Phyto-Profiling*—1, b	Basil_Aq_	✓ +++
				Basil_MeOH_	✓ +++
				Basil_EtOH_	✓ +++
				Basil_EtOAc_	✓ +
2	Saponins	Emulsion test	*Biochemical Phyto-Profiling*—2	Basil_Aq_	✓ +++
				Basil_MeOH_	✓ +++
				Basil_EtOH_	✓ +++
				Basil_EtOAc_	✓ ++
3	Phenols	Ferric Chloride Test	*Biochemical Phyto-Profiling*—3	Basil_Aq_	✓ +++
				Basil_MeOH_	✓ +++
				Basil_EtOH_	✓
				Basil_EtOAc_	✓
4	Tannins	Harborne Test	*Biochemical Phyto-Profiling*—4	Basil_Aq_	✓ +++
				Basil_MeOH_	✓ +++
				Basil_EtOH_	✓ +
				Basil_EtOAc_	✓
5	Glycosides	Keller-Kiliani Test	*Biochemical Phyto-Profiling*—5	Basil_Aq_	✓
				Basil_MeOH_	✓ +++
				Basil_EtOH_	✓ +++
				Basil_EtOAc_	✓
6	Terpenoids	Salkowski Test	*Biochemical Phyto-Profiling*—6	Basil_Aq_	✓ +++
				Basil_MeOH_	✓ +++
				Basil_EtOH_	✓ +++
				Basil_EtOAc_	✓ +
7	Flavonoids	Vanillin Test	*Biochemical Phyto-Profiling*—7	Basil_Aq_	—
				Basil_MeOH_	✓ +++
				Basil_EtOH_	✓ +++
				Basil_EtOAc_	✓ +
8	Steroids (phytosterols)	Liebermann-Burchard Test	*Biochemical Phyto-Profiling*—8.	Basil_Aq_	—
				Basil_MeOH_	—
				Basil_EtOH_	✓
				Basil_EtOAc_	✓
9	Coumarins	Sodium Hydroxide Test	*Biochemical Phyto-Profiling*—9	Basil_Aq_	✓
				Basil_MeOH_	—
				Basil_EtOH_	—
				Basil_EtOAc_	✓

**✓** Present, ++ colour intensity of precipitate/reaction, —Absent

### HLM Screening and IC_50_ Assays

**Figure 5:**
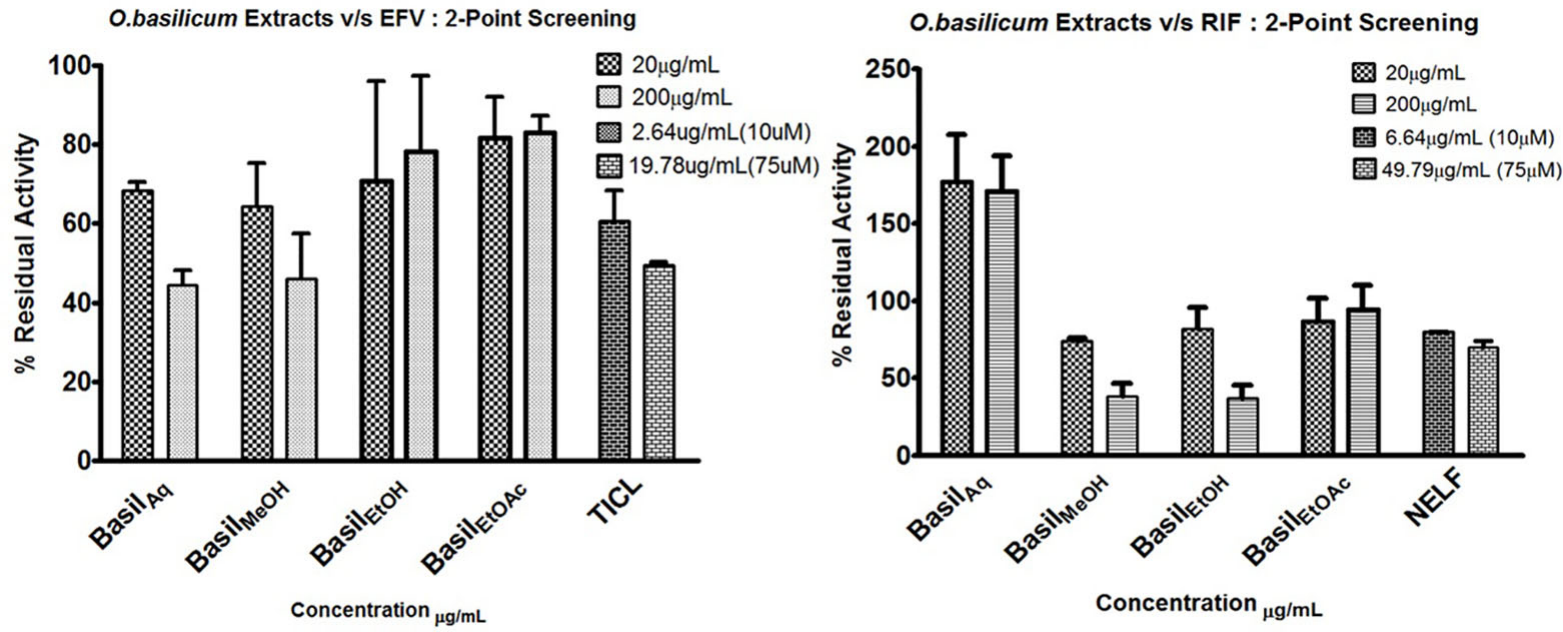
2-Point screening of all basil extracts against CYP2B6 (efavirenz) and rifampicin metabolism; TICL (ticlopidine) and NELF (nelfinavir) are the positive controls.

HLM assays were used to evaluate the inhibitory effect of basil extracts on CYP2B6 and rifampicin metabolism. The aqueous and methanolic extracts reduced CYP2B6 activity by less than 50% at 200 μg/ml concentrations, whereas the positive control ticlopidine reduced the activity to 50% at 19.78 μg/ml. For the two-point screening against rifampicin metabolism, except for the aqueous extract, all extracts inhibited the formation of 25-*O*-desacetyl rifampicin; ethanol extract reduced the activity to less than 40%. The positive control nelfinavir reduced the activity by 30% at 49.79 μg/ml concentrations (**Figure 5**).

**Figure 6:**
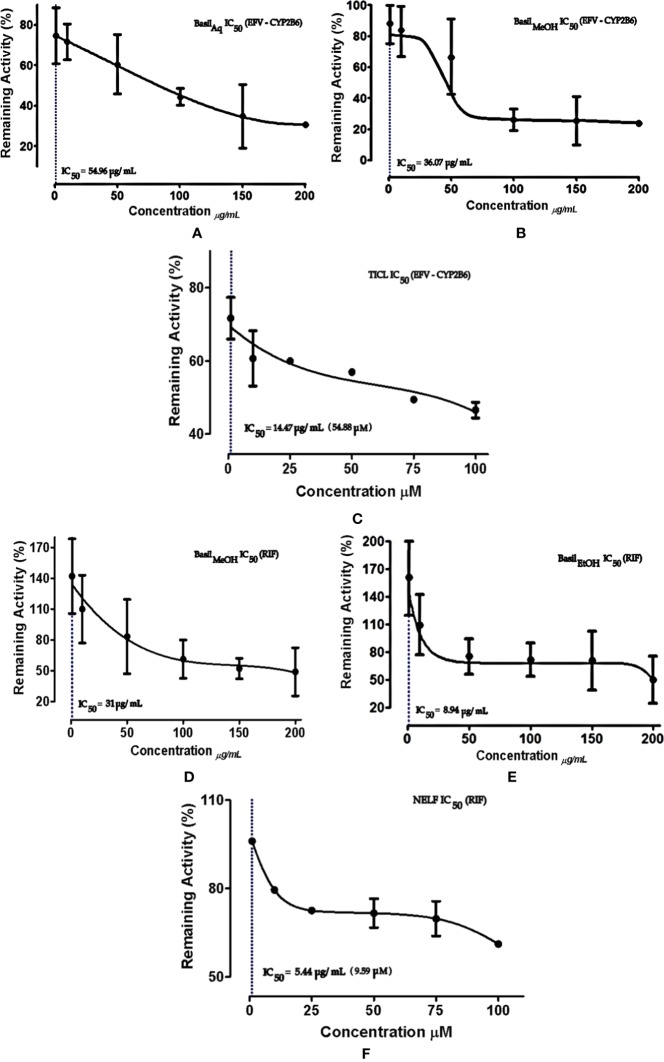
Dose-response curves of basil extracts (with IC_50_s) for **(A)** aqueous extract (54.96 μg/ml), **(B)** methanolic extract (36.07 μg/ml), and positive control **(C)** ticlopidine (14.47 μg/ml, 54.88 μM) against percentage remaining activity of CYP2B6 with efavirenz as the substrate. Figures **(D)** methanolic extract (31 μg/ml), **(E)** ethanolic extract (8.94 μg/ml), and positive control **(F)** nelfinavir (5.44 μg/ml, 9.59 μM) represent the IC_50_ dose-response curves of basil extracts against percentage remaining activity of rifampicin metabolism. The IC_50_ is calculated as log(X) against Y.

For CYP2B6 IC_50_ screening of the extracts, the methanolic extract inhibited the efavirenz metabolism pathway with an IC_50_ value 36.07 μg/ml, while the aqueous extract had an IC_50_ value of 54.96 μg/ml (**Figures 6A–C**). The positive control ticlopidine had an IC_50_ of 14.47 μg/ml (54.88 μM) when tested for inhibition activity, in a concentration range of 0–26.38 μg/ml (0–100 μM).

The ethanolic extract inhibited the rifampicin metabolism pathway, with the lowest IC_50_ value 8.94 μg/ml, while the methanolic extract exhibited an IC_50_ value of 31 μg/ml. The positive control nelfinavir inhibited the formation of 25-*O*-desacetyl rifampicin with an IC_50_ value of 5.44 μg/ml (**Figures 6D–F**).

### TDI IC_50_ Fold Shift Determination

**Figure 7:**
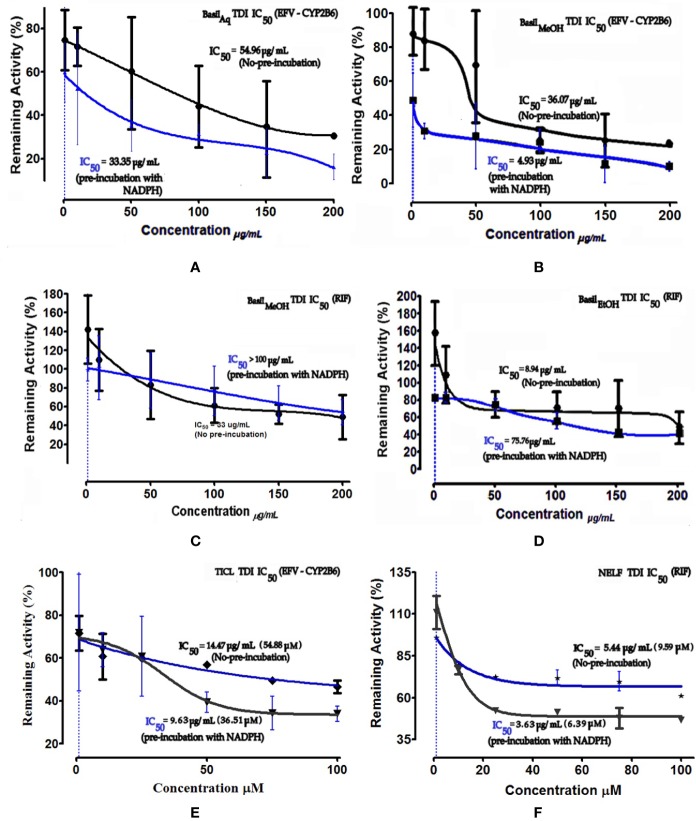
Dose-response curves of basil extracts (with TDI IC_50_s) for **(A)** aqueous extract (33.35 μg/ml), **(B)** methanolic extract (4.93 μg/ml), and positive control **(E)** ticlopidine (9.63 μg/ml, 36.51 μM) against percentage remaining activity of CYP2B6 (efavirenz as substrate) and **(C)** methanolic extract (> 100 μg/ml), **(D)** ethanolic extract (75.76 μg/ml) and, **(F)** positive control nelfinavir (3.63 μg/ml, 6.39 μM) against percentage remaining activity of rifampicin metabolism. The TDI IC_50_ is calculated as log(X) against Y. The plot demonstrates the TDI IC_50_ (μM) calculated using non-linear regression (dose-response inhibition) v/s the actual IC_50_ plot curve-fit.

Time-dependent inhibition (TDI) is the irreversible inhibition of the enzyme activity, where the potency of the inhibitor increases on prolonged exposure to the CYPs during pre-incubation time period. For basil extracts the TDI was assessed by pre-incubation with NADPH for 30 min (Stresser et al., 2014). The aqueous and methanolic extracts exhibited TDI of CYP2B6 activity with IC_50_ values 33.35 and 4.93 μg/ml, respectively. However, none of the extracts demonstrated TDI effects against the rifampicin metabolism pathway; for the ethanolic extract, the IC_50_ shift observed was > 100 μg/ml. Comparatively both positive controls showed clear TDI with IC_50_ shifts to 9.63 μg/ml for ticlopidine against CYP2B6, and 3.63 μg/ml for nelfinavir against rifampicin pathway (**Figure 7**).

**Figure 8:**
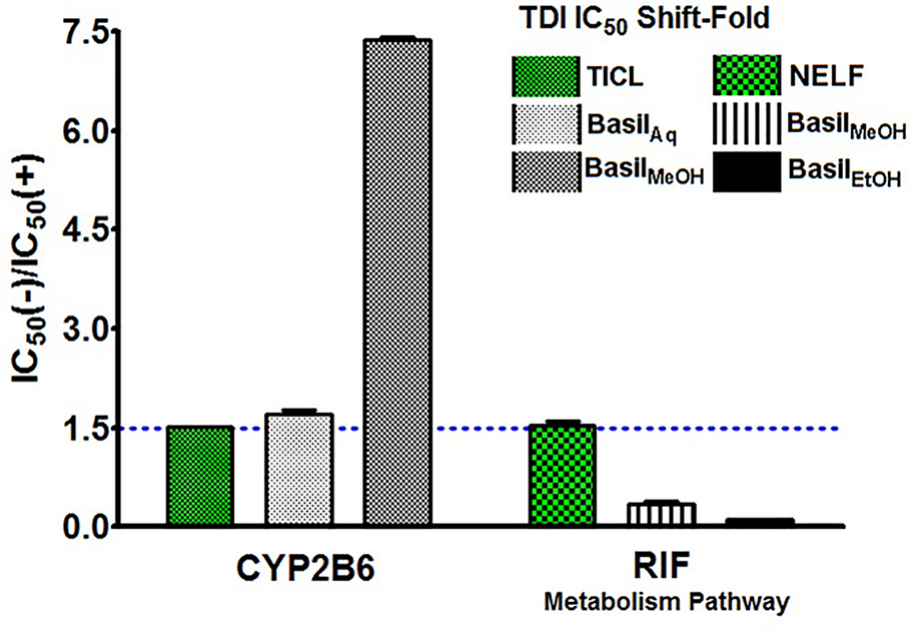
TDI Shift-fold plots. Bars represent the ratio of co-incubation IC_50_(-) values for the extracts to the pre-incubation IC_50_(+) values with NADPH, for basil extracts. Ticlopidine (TICL) and nelfinavir (NELF) were positive controls for CYP2B6 and rifampicin pathway, respectively.

The IC_50_ shift-fold was calculated as the ratio of the co-incubation IC_50_(-) to the pre-incubation IC_50_(+) with NADPH, for each extract and the controls. The aqueous and methanolic extracts showed positive TDI for CYP2B6; the methanolic extract exhibited strong TDI with 7.4-fold increase in the IC_50_. Both positive controls ticlopidine and nelfinavir demonstrated clear TDI with the IC_50_ shift-fold >1.5 (**Figure 8**).

### HEPG2 Induction Assays

For the MTT assays, all basil extracts were screened for *in vitro* cytotoxicity levels against HepG2 cells, by exposing the cells to various concentrations of each extract (1,000.00–31.25 μg/ml). The concentration of the test extract needed to inhibit cell growth by 50%–CC_50_ values were calculated for the four basil extracts as illustrated in **Table 5**. The ethanolic extract had the lowest CC_50_ of 70.58 ± 0.83 μg/ml in HepG2 cells (**Table 5**).

**Table 5:** Concentration of each basil extract that causes 50% cytotoxicity in HepG2 cells (CC_50_).

Sample Name	Concentration (µg/ml)	Cytotoxicity (%)	CC_50_ (µg/ml)^#^
Basil_Aq_	1,000 500 250 125 62.5	66.95 ± 1.35 61.34 ± 1.73 53.85 ± 0.54 42.98 ± 0.94 2.23 ± 0.33	205.73 ± 1.26
Basil_MeOH_	1,000 500 250 125 62.5	72.20 ± 1.17 58.80 ± 0.78 54.50 ± 0.72 37.90 ± 0.52 14.10 ± 1.05	216.30 ± 3.35
Basil_EtOH_	1,000 500 250 125 62.5	90.75 ± 0.15 89.13 ± 0.67 75.38 ± 0.54 65.08 ± 0.33 47.75 ± 0.31	70.58 ± 0.83
Basil_EtOAc_	1,000 500 250 125 62.5	97.43 ± 0.15 61.92 ± 1.67 40.53 ± 2.54 25.77 ± 0.33 7.86 ± 0.31	320.18 ± 2.13

^#^Values are represented as mean ± SD (n = 3). CC_50_ calculated as the mean ± SD of cytotoxicity % values on HepG2 cells at concentration range 1000–31.25 µg/ml for each extract.

**Figure 9:**
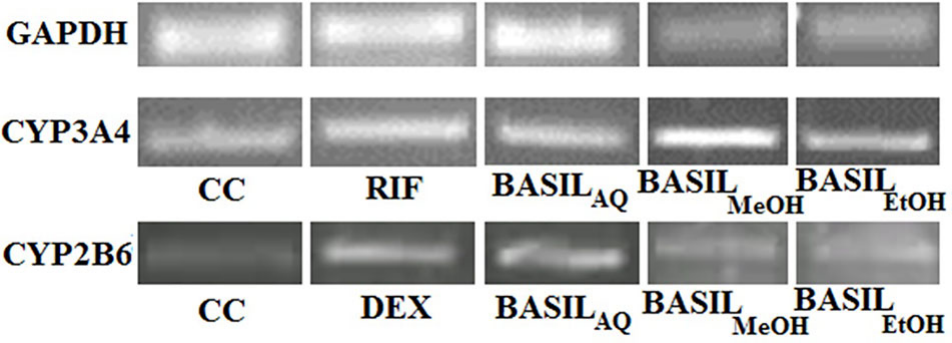
Relative CYP3A4 and CYP2B6 mRNA expressions by CC, rifampicin, dexamethasone and basil extracts on agarose gel; relative expression of GAPDH mRNA on agarose gel for normalization.

Based on the CC_50_ values, the inducing effect of each extract on mRNA expression of the HepG2 cells was determined using RT-PCR and AGE techniques. The expression levels of CYP2B6 and CYP3A4 are depicted as arbitrary units normalized to (GAPDH) mRNA (**Figure 9**).

**Figure 10:**
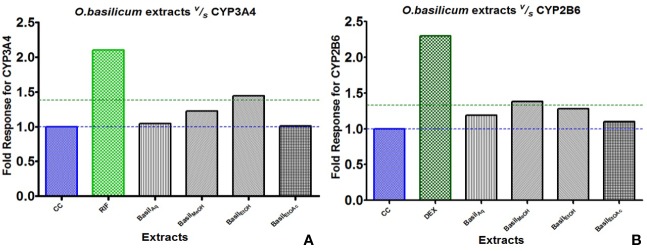
Graphs of the herbal extracts and their fold responses for CYP3A4 and 2B6 mRNA expression, relative to cell control (CC); rifampicin (RIF) and dexamethasone (DEX) as positive controls, **(A, B)** respectively.

For CYP3A4, the positive control rifampicin (50 μM) showed significant fold induction (p < 0.001) compared to the cell control (CC). None of the extracts showed 2-fold induction on CYP3A4 mRNA expression indicating that they were only moderate inducers. All basil extracts induced CYP3A4, with the methanolic extract showing 22%-fold response increase and ethanolic extract at 44%. However, none of the extracts induced both CYPs over 2-fold, indicating that the phytomolecules present in these extracts are not strong inducers of CYP2B6 and 3A4. Based on the results observed in the mRNA expression assays in HepG2 cells, it was concluded that CYP3A4 was more inducible by basil extracts compared to CYP2B6 (**Figure 10A**).

Dexamethasone (10 μM), the positive control against CYP2B6, showed significant fold induction (p < 0.001) when compared to the cell control (CC, no inducer). In comparison, the extracts showed less than 2-fold induction and therefore were only moderate inducers. The methanolic and ethanolic extracts moderately induced CYP2B6 mRNA with 38%- and 28%-fold shifts respectively (**Figure 10B**).

### Fingerprint Analysis of the Phytoconstituents

**Figure 11:**
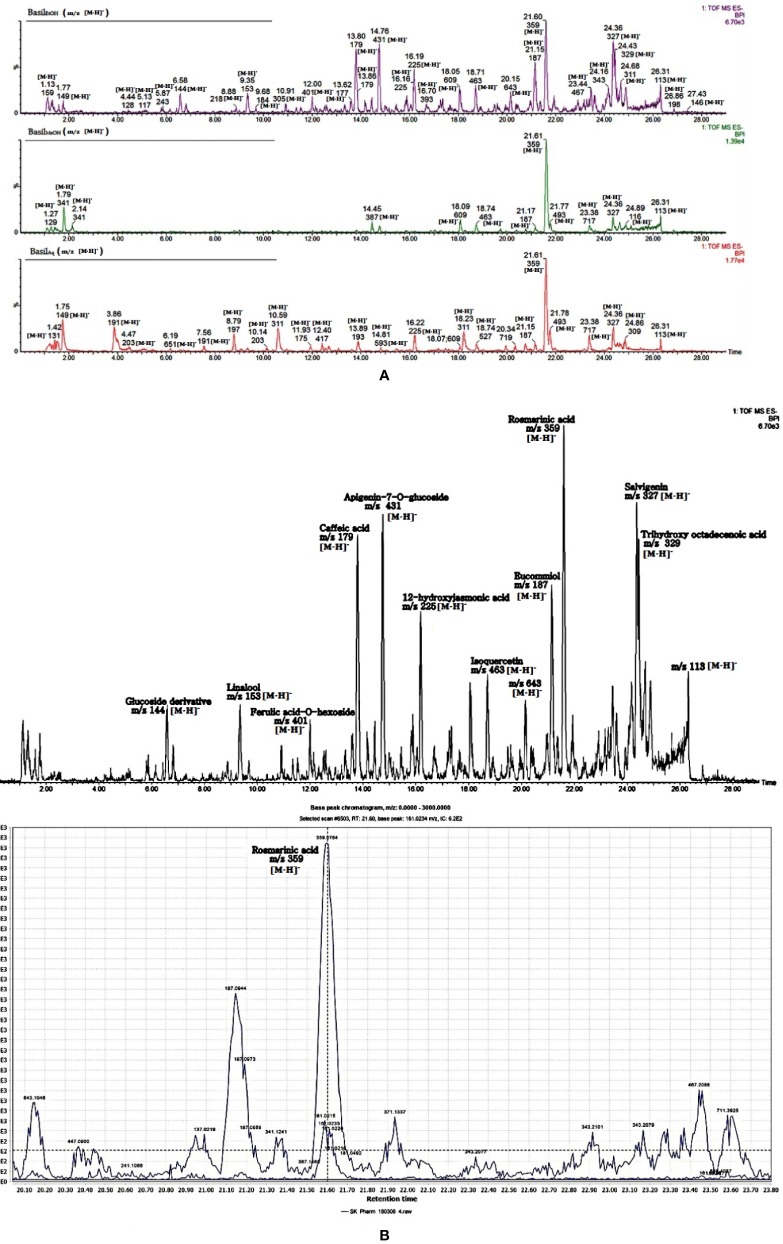
**(A)** LC-MS chromatogram of basil extracts in negative mode scan for time duration of 29 min. **(B)** LC-MS chromatogram of basil (ethanol extract) in negative mode scan for time duration of 29 min, LC-MS spectra focusing on rosmarinic acid [*m/z* 359.0764 (M-H)^-^].

The identified phytocompounds (acidic and non-acidic compounds) were relatively quantified using gallic acid and quercetin as reference standards. Gallic acid calibration was determined in negative scan mode in the MS since most of the acidic compounds were detected in the same scan mode. Each negative scan was performed for 29 min, whereas the positive scan spanned 15 min (**Figures 11A, B**).

In the negative scan mode, the major observations noted for the extracts are as follows (for details, see **Table 6**):

**Table 6:** Compounds detected in *O.basilicum* extracts in negative scan mode in LC-MS/PDA.

SL No.	RT (min)	M-H	[M-H]^–^	MS/MS*^c^*	Tentative ID^#^	Type of Compound
Basil_Aq_						
1	21.65	359.0764	C_18_H_15_O_8_	161, 133, 135, 179, 197	Rosmarinic acid	Phenolic acid
2	1.75	149.0076	C_4_H_5_O_6_	113, 130.997, 141	Tartaric acid	Organic acid
3	3.86	191.0175	C_6_H_7_O_7_	111, 129, 173	Isocitric acid	Citric acid
4	24.36	327.2150	C_18_H_15_O_6_	116.9, 205, 215, 277, 311	Salvigenin (5-Hydroxy-6,7,4′-trimethoxyflavone)	Flavones
5	10.59	311.0392	C_13_H_11_O_9_	135, 149, 179, 311	Caftaric acid (Caffeoyl-tartaric acid)	Non-flavanoid phenolic
8	16.22	225.1110	C_12_H_17_O_4_	112, 135, 161, 192, 203, 216	12-hydroxyjasmonic acid	Carboxylic acid
6	18.23	473.0708	C_22_H_17_O_12_	135, 149, 179, 293, 311	Chicoric acid (dicaffeoyl-tartaric acid)	Hydroxycinnamic acid
7	23.38	717.1448	C_36_H_29_O_16_	243, 343, 519	Unknown	–
Basil_MeOH_						
1	21.61	359.0764	C_18_H_15_O_8_	161, 133, 135, 179, 197	Rosmarinic acid	Phenolic acid
2	1.79	341.1076	C_12_H_21_O_11_	89, 173	Dihexose	Sugar
3	24.36	327.2150	C_18_H_15_O_6_	116.9, 205, 215, 277, 311	Salvigenin (5-Hydroxy-6,7,4′-trimethoxyflavone)	Flavones
4	18.09	609.1499	C_27_H_29_O_16_	151, 255, 271, 300, 301	Rutin (Quercetin-hexoside-rhamnoside)	Flavonoid
5	14.45	387.1648	C_21_H_23_O_7_	59, 119, 207, 300	Medioresinol	Furanoid lignin
6	18.74	463.0882	C_21_H_19_O_12_	89, 151, 255, 271, 300	Isoquercetin (Quercetin-hexoside)	Flavonoid
7	23.38	717.1450	C_36_H_29_O_16_	243, 343, 519	Unknown	–
8	20.99	137.1212	C_9_H_13_O	93, 121	trans-ocimene oxide	Monoterpenes
Basil_EtOH_						
1	21.6	359.0764	C_18_H_15_O_8_	161, 133, 135, 179, 197	Rosmarinic acid	Phenolic acid
2	9.35	153.4201	C_10_H_17_O	79, 93, 109, 127, 137	Linalool (2,6-Dimethyl-2,7-octadien-6-ol; allo-Ocimenol)	Terpene alcohol
3	24.36	327.2150	C_18_H_15_O_6_	116.9, 205, 215, 277, 311	Salvigenin (5-Hydroxy-6,7,4′-trimethoxyflavone)	Flavone
4	14.76	431.1907	C_21_H_19_O_10_	153, 205, 269, 354, 385	Apigenin-7-O-glucoside	Flavonoid glycoside
5	13.82	179.0330	C_9_H_7_O_4_	135	Caffeic acid	Hydroxycinnamic acid
6	24.43	329.2310	C_18_H_33_O_5_	171, 211	Trihydroxy octadecenoic acid	Fatty acid
7	21.15	187.0944	C_9_H_15_O_4_	125, 158, 169	Eucommiol	Cyclopentene dimethanol
8	16.19	225.1113	C_12_H_17_O_4_	112, 135, 161, 192, 203, 216	12-hydroxyjasmonic acid	Carboxylic acid
9	18.71	463.0874	C_21_H_19_O_12_	89, 151, 255, 271, 300	Isoquercetin (Quercetin-hexoside)	Flavonoid

^#^References – Hossain et al., 2010; Mena et al., 2012; Simirgiotis et al., 2015; Chen et al., 2016; Stander et al., 2017; Said et al., 2017; https://www.ncbi.nlm.nih.gov/pccompound; https://massbank.eu/MassBank/; https://metlin.scripps.edu/; https://webbook.nist.gov/chemistry/mw-ser/.

**Figure 12:**
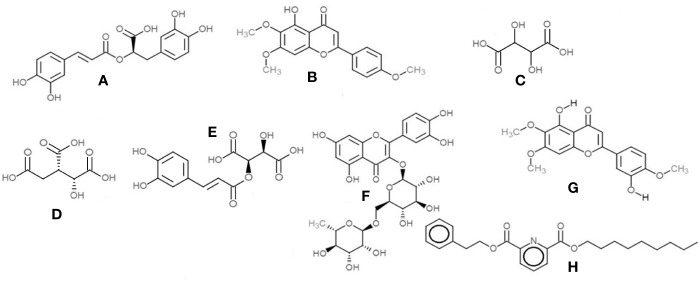
Chemical structures of the main compounds identified from crude herbal extracts of *O.basilicum*: **(A)** Rosmarinic acid; **(B)** Salvigenin; **(C)** Tartaric acid; **(D)** Isocitric acid; **(E)** Caftaric acid; **(F)** Rutin; **(G)** Eupatorin; and **(H)** 2,6-Pyridinedicarboxylic acid, nonyl phenethyl ester.

1. Rosmarinic acid, a polyphenol was the most prominent peak in all extracts of basil (**Figure 12A**). It had a retention time of 21.65 min at *m/z* 359 (M-H)^-^ and detection wavelength of 329nm on the PDA (**Figure 12A**). The product ions were identified at *m/z* 161, 133, 135, 179, and 197 (**Figure 11B**).
2. The flavone salvigenin (5-Hydroxy-6,7,4′-trimethoxyflavone) was prominentlyobserved in the extracts, at retention time 24.36 min and *m/z* 327(M-H)^-^, with product ions at *m/z* 116.9, 205, 215, 277 and 311 (**Figure 12B**).
3. Acidic compounds such as tartaric, isocitric, caftaric and chicoric acids were prominently observed in the aqueous extract at *m/z* 149, 191, 311, and 473, at wavelengths 230 nm for the first two and 328–329 nm for the latter two compounds (**Figures 12C–E**).
4. Rutin, a major flavonoid, was observed only in the methanolic extract at *m/z* 609 (M-H)^-^ with products ions at *m/z* 151, 255, 271, 300 and 301 (**Figure 12F**).
5. Apigenin-7-O-glucoside or apigetrin (also known as cosmosiin), a flavonoid-7-O-glycoside, was detected in the ethanolic extract with a retention time 14.76 min and *m/z* 431 (M-H)^-^.
6. Other significant compounds detected in the extracts were eucommiol [*m/z* 187 (M-H)^-^], 12-hydroxyjasmonic acid [*m/z* 225 (M-H)^-^], trans-ocimene oxide [*m/z* 137 (M-H)^-^], and medioresinol [*m/z* 387 (M-H)^-^].

In the positive mode MS scans (**Figure 11B**), the major phytocompounds detected (**Table 7**) in the extracts included:

1. Salvigenin (5-Hydroxy-6,7,4′-trimethoxyflavone)—the most prominent peak in the methanol and ethanolic extracts, with a retention time of 7.32 min at *m/z* 329 (M+H)^+^ and PDA detection wavelength 230 nm (**Figure 12B**).
2. 2,6-Pyridinedicarboxylic acid, nonyl phenethyl ester [at *m/z* 398 (M+H)^+^] was detected in all the extracts with product ions *m/z* 149, 240, and 266 (**Figure 12H**).
3. The flavone eupatorin [3′,5-dihydroxy-4′,6,7-trimethoxyflavone, *m/z* 345 (M+H)^+^] (**Figure 12G**) and the aromatic lactone furan-2(3*H*)-one complex [*m/z* 311 (M+H)^+^] were the other major compounds identified, at retention times 6.42 and 6.91 min, respectively.

**Table 7:** Secondary metabolites/compounds detected in *O.basilicum* extracts in positive scan mode in LC-MS/PDA.

SL No.	RT (min)	M+H	[M+H]^+^	MS/MS*^c^*	Tentative ID^#^	Type of compound
Basil_Aq_						
1	10.63	398.2331	C_24_H_32_NO_4_	149, 240, 266	2,6-Pyridinedicarboxylic acid, nonyl phenethyl ester	Carboxylic acid
2	7.74	304.3008	C_21_H_38_N	91, 212	Pyridine/Amine complex	Secondary Metabolites
3	12.28	411.2662	C_30_H_35_O	175, 283, 355	Unknown	–
4	6.91	311.2210	C_18_H_31_O_4_	96, 149, 219, 275	Furan-2(3*H*)-one complex	Aromatic lactone
5	9.06	399.2498	C_25_H_35_O_4_, C_18_H_39_O_9_	221, 324	Unknown	–
Basil_MeOH_						
1	7.32	329.1014	C_18_H_17_O_6_	133, 268	Salvigenin (5-Hydroxy-6,7,4′-trimethoxyflavone)	Flavones
2	10.63	398.2324	C_24_H_32_NO_4_	149, 240, 266	2,6-Pyridinedicarboxylic acid, nonyl phenethyl ester	Carboxylic acid
3	7.74	304.2997	C_21_H_38_N	91, 212	Pyridine/Amine complex	Secondary Metabolites
4	6.42	345.0979	C_18_H_17_O_7_	182, 240, 312, 315	Eupatorin (3′,5-Dihydroxy-4′,6,7-trimethoxyflavone)	Flavones
Basil_EtOH_						
1	7.32	329.1023	C_18_H_17_O_6_	133, 268	Salvigenin (5-Hydroxy-6,7,4′-trimethoxyflavone)	Flavones
2	6.42	345.0959	C_18_H_17_O_7_	182, 240, 312, 315	Eupatorin (3′,5-Dihydroxy-4′,6,7-trimethoxyflavone)	Flavones
3	7.16	445.2117	C_21_H_33_O_10_, C_32_H_29_O_2_	105, 194 224, 385, 407	Unknown	–
4	10.63	398.2336	C_24_H_32_NO_4_	149, 240, 266	2,6-Pyridinedicarboxylic acid, nonyl phenethyl ester	Carboxylic acid

^#^References – Ye et al., 2005; Hossain et al., 2010; Čejchanová, 2011; Wang et al., 2012; https://www.ncbi.nlm.nih.gov/pccompound; https://massbank.eu/MassBank/; https://metlin.scripps.edu/; https://webbook.nist.gov/chemistry/mw-ser/.

The identified compounds were quantified (in mg/L equivalent units of gallic acid/quercetin) relative to the linear calibration curve of the reference standards (**Table 8**).

**Table 8:** Concentration of each compounds identified within the basil extracts (mg/L equivalents).

# SL No	Compound Name	Sample name	Area	RT(min)	Conc. units * (mg/L equivalents of gallic acid/quercetin)
Compound (1)	Rosmarinic acid				**
1		Basil_Aq_	1699.1760	21.61	2298.037
2		Basil_MeOH_	1423.3300	21.61	1923.756
3		Basil_EtOH_	585.0240	21.61	786.304
Compound (2)	Tartaric acid				
1		Basil_Aq_	593.9001	1.76	798.347
2		Basil_MeOH_	33.9320	1.76	38.556
3		Basil_EtOH_	45.3010	1.76	53.982
Compound (3)	Isocitric acid				
1		Basil_Aq_	742.4790	3.88	999.946
Compound (4)	Caftaric acid				
1		Basil_Aq_	407.1970	10.60	545.019
Compound (5)	Chicoric acid				
1		Basil_Aq_	371.2370	18.23	496.228
2		Basil_MeOH_	22.3180	18.26	22.798
Compound (6)	Salvigenin (5-Hydroxy-6,7,4′-trimethoxyflavone)				***
1		Basil_Aq_	430.9610	24.36	73.634
2		Basil_MeOH_	213.4770	24.36	24.150
3		Basil_EtOH_	670.6350	24.36	128.167
Compound (7)	Rutin (Quercetin-Hexoside-rhamnoside)				
1		Basil_Aq_	80.0950	18.07	0.010
2		Basil_MeOH_	144.1280	18.09	8.371
3		Basil_EtOH_	146.4550	18.07	8.900
Compound (8)	Isoquercetin (Quercetin-Hexoside)				
1		Basil_MeOH_	108.5580	18.73	0.277
2		Basil_EtOH_	156.3420	18.72	11.150
Compound (9)	Linalool (2,6-Dimethyl-2,7-octadien-6-ol)				
1		Basil_EtOH_	133.6801	9.35	5.993
Compound (10)	12-hydroxyjasmonic acid				
1		Basil_Aq_	262.7230	16.21	348.991
2		Basil_EtOH_	255.5850	16.19	339.305
Compound (11)	Caffeic acid				
1		Basil_MeOH_	29.9890	13.83	33.206
2		Basil_EtOH_	433.6770	13.80	580.949
Compound (12)	(E)-Fukugetin				
1		Basil_EtOH_	45.8240	26.32	0.005
Compound (13)	Medioresinol				
1		Basil_MeOH_	114.7220	14.45	6.020
2		Basil_EtOH_	69.9301	14.44	0.005
Compound (14)	Apigenin-7-O-glucoside				
1		Basil_MeOH_	75.4170	14.77	0.010
2		Basil_EtOH_	404.7730	14.75	67.675
Compound (15)	Trihydroxy octadecenoic acid				
1		Basil_Aq_	127.4110	24.44	165.393
2		Basil_MeOH_	100.3910	24.44	128.731
3		Basil_EtOH_	396.4770	24.44	530.474
Compound (16)	Eucommiol				
1		Basil_Aq_	138.4490	21.16	7.078
2		Basil_EtOH_	354.5340	21.15	56.244
Compound (17)	Dihexose				
1		Basil_MeOH_	279.5990	1.8	39.194
Compound (18)	Eupatorin (3′,5-Dihydroxy-4′,6,7-trimethoxyflavone)				
1		Basil_Aq_	44.5180	6.42	22.874
2		Basil_MeOH_	217.4830	6.42	111.503
3		Basil_EtOH_	1305.0280	6.42	668.772
Compound (19)	Salvigenin (5-Hydroxy-6,7,4′-trimethoxyflavone)				
1		Basil_Aq_	75.5380	7.33	38.769
2		Basil_MeOH_	655.0720	7.32	335.728
3		Basil_EtOH_	3619.8580	7.32	1854.916

*The relatively quantified concentrations are reasonable approximations of the relative amounts of the identified compounds present in each (Bhandari and Rajbhandari, 2015; Punyasiri et al., 2015) **All the acid compounds are relatively quantified equivalent to the linear curve of gallic acid, ***All the non- acid compounds are relatively quantified equivalent to the linear curve of quercetin.

Salvigenin concentration was highest in the ethanolic extract (1854.916 mg/L), followed by the fatty acid trihydroxy octadecenoic acid (530.474 mg/L), the flavone eupatorin (668.772 mg/L) and caffeic acid (580.949 mg/L). Rosmarinic acid was predominantly present in the aqueous extract (2298.037 mg/L), along with isocitric (999.946 mg/L), tartaric acid (798.347 mg/L), chicoric (496.228 mg/L), and caftaric acids (545.019 mg/L).

### Estimated Hepatic Blood Concentration of the Extracts

The putative GIT concentration for the aqueous, methanolic and ethanolic extracts was 2,400 μg/ml (refer section *IC_50_ and TDI Assays* for formula). For a single recommended dose of 600 mg of basil extract, the available hepatic blood concentration [I] was estimated at 805.68 μg/ml for the aqueous extract against CYP2B6, 226.56 μg/ml for the methanolic extract against CYP2B6 and rifampicin metabolism, and 70.56 μg/ml for the ethanolic extract against rifampicin pathway (**Table 9**). The inhibitory potency [I]/K_i_ was >1.0 for all the extracts exhibiting their likely potential of causing HDI. The predicted *in vivo* inhibition percentiles were at 96.70% for the aqueous extract, 94.04% for the ethanolic extract and 92.62–93.60% for the methanolic extract. However other factors such as AUC ratio of the drugs, first order absorption rate K_a_, fraction of systemic clearance of the substrate F_m_, elimination rate, CL_int_ intrinsic clearance, and the fraction absorbed after oral administration F_a_ have not been considered in this prediction model (F.D.A., 2017).

**Table 9:** Predicted values of inhibition percentage of each extract and estimated hepatic concentration.

CYP/RIF	Extract	% Yield	Herbal dosage (mg)	Putative GIT Conc. (μg/ml)	Estimated Hep. Blood Conc. [I] (μg/ml)	IC_50_ (μg/ml)	K_i_ = IC_50_/2	Inhibitory Potency [I]/Ki	Risk of HDI *	Predicted % Inhibition ([I]/([I]+K_i_)) *100
2B6	Basil_Aq_	33.57	600	2400	805.68	54.96	27.48	29.32	Likely	96.70
	Basil_MeOH_	9.44	600	2400	226.56	36.07	18.04	12.56	Likely	92.62
RIF	Basil_MeOH_	9.44	600	2400	226.56	31.00	15.50	14.62	Likely	93.60
	Basil_EtOH_	2.94	600	2400	70.56	8.94	4.47	15.79	Likely	94.04

HDI, herb-drug interaction; K_i_, inhibition constant; RIF, rifampicin; * When these herbal extracts are co-administered with other drugs, the likelihood of a clinically relevant interaction with CYP2B6 and rifampicin metabolism, was based on the assumption that the % yield serves as the bioavailable fraction, which was used in estimating the bioavailable hepatic blood concentration and also if the fact that there was complete absorption. AUC ratio of the drugs was not calculated based on the [I] value and other kinetic parameters such as first order absorption rate K_a_, fraction of systemic clearance of the substrate F_m_, elimination rate, CL_int_ intrinsic clearance etc. Refer section Hepatic Blood Concentrations—Prediction on Inhibition Percentage for formulas.

## Discussion

Traditional health practitioners often prepare herbal formulations using tea infusions and overnight incubations with alcoholic beverages such as brandy (Thring and Weitz, 2006). Hence aqueous and ethanolic extractions of basil were selected for this study. Higher pharmacological activity is generally observed in methanolic, ethanolic, and ethyl acetate extractions (Dhanani et al., 2017). Qualitative analysis is a precursor to analytical fingerprinting of herbal constituents. As per this study, high intensity of flavonoids, phenols, alkaloids, terpenoids, and glycosides were observed in the extracts, some of these compounds being potentially responsible for the aromatic nature of basil oil (Loughrin and Kasperbauer, 2003) and its potential to cause significant interaction with the CYPs. Previous studies have shown the presence of triterpenoid saponins (Habib et al., 2016), alkaloids, anthraquinones, saponins, and flavonoids in the methanolic extract as well as glycosides, phenols, phlobatatannins, tannins, and terpenoids in the aqueous extract (Fakhroo and Sreerama, 2016). In this study more compounds such as phytosteroids and coumarins were also detected, especially in the ethyl acetate extract.

In the HLM screening, except for the aqueous extract (affected only RIF metabolism), all extracts of basil inhibited CYP2B6-mediated metabolism of efavirenz and formation of rifampicin metabolite. There was an increase in rifampicin metabolite formation against the aqueous at extract both concentrations (170% at 200 μg/ml), which could be due to the mechanism of enzyme activation due to the presence of multiple binding sites at the active site of the enzyme (Atkins et al., 2001; Tracy, 2006); such activations being concentration-dependant. The methanolic extract was strong inhibitor of both pathways, whereas the ethanolic extract was a potent inhibitor of rifampicin pathway with an IC_50_ value of 8.94 μg/ml. Nelfinavir was used as a positive control for this pathway on the assumption that strong CYP inhibitors could also inhibit β-esterases (Polsky-Fisher et al., 2006) with an IC_50_ value of 5.44 μg/ml (9.59 μM). The ethanolic extract showed strong inhibition activity in a concentration range from 0-100 μg/ml. At concentrations >100 μg/ml, the IC_50_ curve did not show a drop in the percentage of remaining activity, unlike the 2-point screening (percentage activity drop from 81.7% at 20 μg/ml to 36.97% 200 μg/ml) indicating the latter to be a relative measurement of the inhibitory effect of an extract at two different concentrations, which would not always correlate with the IC_50_ value obtained. For the positive control ticlopidine the IC_50_ value obtained was within the range reported in earlier studies (12.4–55 μM) (Hagihara et al., 2008; Choi et al., 2011). For nelfinavir the IC_50_ was slightly higher than a value of 2.7 μM reported in a previous HLM study done using different assay conditions and bilirubin as the substrate (Zhang et al., 2005). The aqueous and methanolic extracts had strong TDI effect on CYP2B6, the latter with shift-fold >7; this effect may be attributed to the formation of reactive secondary metabolites on preincubation with NADPH. The methanolic extract showed weaker TDI on esterase pathway at higher concentration, which could possibly be attributed to the formation of secondary metabolites at higher concentrations, having the potential to interfere with the binding of the active principle in the extract with the enzyme, reducing the TDI effect (Fowler and Zhang, 2008).

The standard deviation (standard error of the mean) for the data points for few extracts showed high variance in the assays, which could be attributed to external factors that might have interfered with the assays, or the loss of metabolite due to degradation, during the prolonged HPLC runs. All the assays performed were *in vitro*; however in an *in vivo* scenario there are other factors to consider such as concentration differential between tissues, presence of natural barriers such as varying capillary bed permeability, the epithelial membrane barrier, sub-epithelial blood flow, GIT transit time, disease state and dosage form, and intestinal pH (Gavhane and Yadav, 2012; Fasinu et al., 2014).

CYP3A4 is induced more efficiently compared to the other isoenzymes, and is an important criterion for selection in induction screening studies (Denison and Whitlock, 1995; Dogra et al., 1998); CYP2B6 has gained recent importance in clinically significant risk assessment induction *in vitro* studies on cryopreserved human hepatocytes, along with CYP3A4 (Fahmi et al., 2016). In this study, all basil extracts moderately induced both the CYPs, more effectively activating the mRNA expression in CYP2B6 in HepG2 cells. *O.basilicum* was previously reported in a study on CYP isoenzymes, as an inhibitor of CYP3A4 (Nguyen et al., 2014). In this study, the basil extracts inhibited CYP3A4 in liver microsomes, and moderately induced mRNA expression in CYP3A4, especially the ethanolic extract. This could be due to the synergistic effects of various phytoconstituents in the extract, or some other potential unidentified inducer molecule within the extract, causing CYP induction.

LC-MS (positive and negative modes) was used to identify the flavonoids, polyphenols, and carboxylic acids in the extracts; these were relatively quantified (reasonable approximations of the relative amounts of the identified compounds present in each extract) using calibration curves set up for quercetin and gallic acid (Bhandari and Rajbhandari, 2015; Punyasiri et al., 2015). Rosmarinic acid was a major polyphenol identified; a major component in the aromatic basil essential oil (Kiferle et al., 2011; Güez et al., 2017) and a potential inducer of CYPs such as CYP1A, 2B, and 3A (Cho and Yoon, 2015). Other major compounds included salvigenin, eupatorin, isocitric, tartaric, chicoric and caftaric acids, the flavonoid rutin, and apigenin-7-O-glucoside, which could have caused the observed inhibition of CYP2B6 and β-esterase activity. Salvigenin was previously reported as a moderate inhibitor of CYP3A enzymes (Quintieri et al., 2008). Caftaric and chicoric acids in *Echinacea*, rutin and apigenin derivatives have been reported to interfere with CYP3A4 activity (Bossaer and Odle, 2012; Cichello et al., 2016; Karakurt, 2016; Tang et al., 2017). Eupatorin inhibits CYP1A2 (Pan et al., 2014) and the *in vitro* proliferation of MDA-MB-468 human breast cancer cells that express CYP1A1 (Androutsopoulos et al., 2008). High concentrations of eupatorin, salvigenin and caffeic acids were observed in the ethanolic extract, potentially attributing to its strong inhibition of the rifampicin pathway, compared to the aqueous and methanolic extracts. 12-hydroxyjasmonic acid and fukugetin were the other major identified compounds, which could have added to the inducing capabilities of basil extracts on CYP3A4 mRNA expression along with rosmarinic acid (Son et al., 1998; Cho and Yoon, 2015). The calibration curve set up for gallic acid and quercetin was in the range of 6.25-200 mg/L; however some of the identified compounds within the extracts have values above of the range of the calibration curves. The concentrations calculated for these compounds are reasonable approximations of the relative amounts, in comparison to the standard calibrators, and are not absolute quantifications (Al Feteisi et al., 2015). Various classes of compounds detected are likely to ionize in the MS to vastly different degrees, when compared to the standard calibrators; fold-variations can thus occur in peak areas of these compounds.

As per the FDA guidelines of using model-based predictions (F.D.A., 2017) to determine drug interactions, basic to highly dynamic mechanism-based models including physiologically-based pharmacokinetic (PBPK) models have been used to predict DDI in various clinical studies. In this study a basic model was deployed, where GIT concentrations and hepatic blood concentrations were estimated based on single recommended dosages of herbal extracts on the assumption of equal distribution across the entire GIT system. However, factors such as intestinal fluid composition, dosage form, GIT transit time, capillary bed permeability, intestinal pH, tissue distribution and membrane barriers can affect the distribution of the extracts within the human body (El-Kattan and Varma, 2012; Gavhane and Yadav, 2012). The AUCR of the target drug, along with many other factors and parameters such as first order absorption rate K_a_, fraction of systemic clearance of the substrate F_m_, the fraction absorbed after oral administration F_a_, the fraction available after intestinal metabolism F_g_, the elimination rate and the intrinsic clearance CL_int_ were not considered as part of this prediction model. In this study, the predicted percentile of inhibition for the aqueous, methanolic and ethanolic extracts were > 90% against CYP2B6 and rifampicin pathway, however more mechanistic-dynamic models have to applied considering all the above parameters to predict the probability of *in vivo* HDI of these extracts in the gut assuming that the entire soluble extract interacts with the intestinal CYP enzymes, and competitive inhibition characteristics (F.D.A., 2017; Kumar et al., 2018).

## Conclusions

The biochemical phytoprofiling of basil extracts showed the presence of bioactive compounds that had the potential to interfere with CYP activity, including phenols, flavonoids, alkaloids, phenols, glycosides, coumarins, phytosterols, tannins and saponins. As per the data reported in this study, hot tea decoction extracts as prepared by the traditional health practitioners, had less inhibitory effect on CYP2B6 and β–esterases when compared to the methanolic and ethanolic extracts. The aqueous and methanolic extracts showed strong reversible and TDI of CYP2B6 while the methanolic and ethanolic extracts inhibited rifampicin metabolism, which may be attributed to the fact that the dried leaves, inflorescence and seeds of this herb had more phytoconstituents such as flavonoids and other secondary metabolites in the composition of the extracts (Fathiazad et al., 2012; Bhuvaneshwari et al., 2016), that had strong potential to modulate CYP activity (Manikandan et al., 2007) or cause toxicity (W.H.O. Monographs, 2005; Rasekh et al., 2012). Previous studies had only reported the inhibitory effect of the ethanolic extract on CYP3A4.

Previous *in vitro* studies have shown that NADPH is a not a prerequisite in the deacetylation process of rifampicin, and the increased formation of 25-*O*-desacetyl rifampicin on addition of NADPH was ascribed to the possible metabolism of rifampicin by NADPH-dependent CYP enzymes (Benedetti and Dostert, 1994; Jamis-Dow et al., 1997). Both nelfinavir and basil caused strong TDI of rifampicin pathway when pre-incubated with NADPH, which suggested that the involvement of other enzymes such as CYPs in the biotransformation of rifampicin, since exogenous supply of NADPH was not mandatory in its deacetylation. In the induction assays in HepG2 cells, the methanolic extract induced CYP2B6 mRNA expression, while the ethanolic extract induced CYP3A4, which could be attributed to the various phytoconstituents in basil such as rutin, rosmarinic acid and salvigenin (Debersac et al., 2001; Křížková et al., 2009), and the two different cell systems (HLM and HepG2). Polyphenolic compounds, flavonoids, terpenes, and carboxylic acids were the most observed in basil extracts.

Bioassay-guided fractionation, isolation and identification of the active phytoconstituents in the active extracts and their effect on CYP2B6, 3A4 ad rifampicin metabolism would be a critical step forward. As per the FDA recommendations, analysis of the effects of basil extracts on CYPs—1A2, 2A6, 2D6, 2C9, 2C8, 2C19, and 2E1 is essential. Clinical testing of basil extracts with new *in vitro* models such as “Whole Cell” approach deploying certified human hepatocytes in sandwich-culture with the drug clearance pathways of metabolism and transport, and key regulatory pathways (CAR/PXR) (Jackson et al., 2017) would also be beneficial in establishing critical data on the effects of basil on cytochrome P450 for further *in vivo* and clinical trial studies.

In conclusion, this study shows that *O.basilicum* (basil) may cause HDI in patients treated with other medications metabolized by CYP2B6 (such as artemisinin, bupropion, cyclophosphamide, efavirenz, ketamine, and methadone) (Zanger and Klein, 2013), or with rifampicin. Caution must therefore be exercised in patients concurrently taking such medications. This finding is clinically significant, considering the fact that it is commonly used as herbal tea, as well as a major ingredient in many food items, herbal formulations and essential oils.

## Data Availability Statement

All datasets generated for this study are included in the article/**Supplementary Material**.

## Author Contributions

Research conceptualization: BR, PB and SK. Methodology, investigation, and data interpretation: SK. Writing—original draft preparation: SK. Writing—review-editing and supervision: BR and PB. Infrastructure and lab facilities: PB. Funding acquisition (grant holder): BR.

## Funding

This research was funded by the South African National Research Foundation (Indigenous Knowledge Systems NRF-IKS Grant No: 82641).

## Conflict of Interest

PB was employed by the company Synexa Life Sciences Prv. Ltd., Cape Town, RSA.

The remaining authors declare that the research was conducted in the absence of any commercial or financial relationships that could be construed as a potential conflict of interest.

## References

[B1] Akgül A. (1989). Volatile oil composition of sweet basil (*Ocimum basilicum* L.) cultivating in Turkey (Short communication). Nahrung 33, 87–88. 10.1002/food.19890330129

[B2] Al Feteisi H. Achour B. Barber J. Rostami-Hodjegan A. (2015). Choice of LC-MS methods for the absolute quantification of drug-metabolizing enzymes and transporters in human tissue: a comparative cost analysis. AAPS J. 17, 438–446. 10.1208/s12248-014-9712-6 25663651 PMC4365102

[B3] Androutsopoulos V. Arroo R. R. Hall J. F. Surichan S. Potter G. A. (2008). Antiproliferative and cytostatic effects of the natural product eupatorin on MDA-MB-468 human breast cancer cells due to CYP1-mediated metabolism. Breast Cancer Res. 10, R39. 10.1186/bcr2090 18454852 PMC2481486

[B4] Atkins W. M. Wang R. W. Lu A. H. (2001). Allosteric behavior in cytochrome P450-dependent *in vitro* drug-drug interactions: a prospective based on conformational dynamics. Chem. Res. Toxicol. 14, 338–347. 10.1021/tx0002132 11304120

[B5] Atwine D. Bonnet M. Taburet A. M. (2018). Pharmacokinetics of efavirenz in patients on antituberculosis treatment in high human immunodeficiency virus and tuberculosis burden countries: A systematic review. Br. J. Clin. Pharmacol. 84, 1641–1658. 10.1111/bcp.13600 29624706 PMC6046471

[B6] Benedetti S. M. Dostert P. (1994). Induction and autoinduction properties of rifamycin derivatives: a review of animal and human studies. Environ. Health Perspect. 102, 101–105. 10.1289/ehp.94102s9101 PMC1566786 7698069

[B7] Bhandari L. Rajbhandari M. (2015). Isolation of quercetin from flower petals, estimation of total phenolic, total flavonoid and antioxidant activity of the different parts of *Rhododendron arboreum* Smith. Sci. World J. 12, 34. 10.3126/sw.v12i12.13569

[B8] Bhatt N. B. Baudin E. Meggi B. Silva C. Furlan V. Grinsztejn B. (2014). Nevirapine or efavirenz for tuberculosis and HIV coinfected patients: exposure and virological failure relationship. J. Antimicrob. Chemother. 70, 225–232. 10.1093/jac/dku348 25239466 PMC4267502

[B9] Bhuvaneshwari K. Gokulanathan A. Jayanthi M. Govindasamy V. Milella L. Lee S. (2016). Can *Ocimum basilicum* L. and *Ocimum tenuiflorum* L. @ in vitro culture be a potential source of secondary metabolites? Food Chem. 194, 55–60. 10.1016/j.foodchem.2015.07.136 26471526

[B10] Bossaer J. B. Odle B. L. (2012). Probable etoposide interaction with *Echinacea* . J. Diet Suppl. 9, 90–95. 10.3109/19390211.2012.682643 22607644

[B11] Bozin B. Mimica-Dukic N. Simin N. Anackov G. (2006). Characterization of the volatile composition of essential oils of some lamiaceae spices and the antimicrobial and antioxidant activities of the entire oils. J. Agric. Food Chem. 54, 1822–1828. 10.1021/jf051922u 16506839

[B12] Čejchanová J. (2011). Inhibition of drug glucuronidation by extracts and constituents of St. John’s wort (*Hypericum perforatum*) and Thyme (*Thymus vulgaris*). *Charles Univ. Prague Fac. Pharm. Hradec Králové Würzburg, Dept. Biochem. Sc., Thesis Ed.*, 1–64, CU Catalogue: 001366053. Available at: http://hdl.handle.net/20.500.11956/33497

[B13] Chen G. Li X. Saleri F. Guo M. (2016). Analysis of flavonoids in *Rhamnus davurica* and its antiproliferative activities. Molecules 21, 1275. 10.3390/molecules21101275 PMC6273673 27669205

[B14] Cheng J. Fock K. M. Chua K. L. (1988). Reversible hepatic and renal damage from rifampin overdose - a case report. Singapore Med. J. 29, 306–308. 3187586

[B15] Chiang L. C. Ng L. T. Cheng P. W. Chiang W. Lin C. C. (2005). Antiviral activities of extracts and selected pure constituents of *Ocimum basilicum* . Clin. Exp. Pharmacol. Physiol. 32, 811–816. 10.1111/j.1440-1681.2005.04270.x 16173941

[B16] Cho H. J. Yoon I. S. (2015). Pharmacokinetic interactions of herbs with cytochrome p450 and P-glycoprotein. Evid. Based Complement. Alternat. Med., 736431. 10.1155/2015/736431 25632290 PMC4302358

[B17] Choi J. S. Yang J. S. Choi D. H. (2011). Effects of ticlopidine on the pharmacokinetics of diltiazem and its main metabolite, desacetyldiltiazem, in rats. Biomol. Ther. 19, 255–260. 10.4062/biomolther.2011.19.2.255

[B18] Cichello S. A. Yao Q. He X. Q. (2016). Proliferative activity of a blend of *Echinacea angustifolia* and *Echinacea purpurea* root extracts in human vein epithelial, HeLa, and QBC-939 cell lines, but not in Beas-2b cell lines. J. Tradit. Complement. Med. 6, 193–197. 10.1016/j.jtcme.2015.01.002 27114944 PMC4833461

[B19] de Almeida I. Alviano D. S. Vieira D. P. Alves P. B. Blank A. F. Lopes A. H. (2007). Antigiardial activity of *Ocimum basilicum* essential oil. Parasitol. Res. 101, 443–452. 10.1007/s00436-007-0502-2 17342533

[B20] Debersac P. Heydel J. M. Amiot M. J. Goudonnet H. Artur Y. Suschetet M. (2001). Induction of cytochrome P450 and/or detoxication enzymes by various extracts of rosemary: description of specific patterns. Food Chem. Toxicol. 39, 907–918. 10.1016/s0278-6915(01)00034-5 11498267

[B21] Denison M. S. Whitlock J. P. (1995). Xenobiotic-inducible transcription of cytochrome P450 genes. J. Biol. Chem. 270, 18175–18178. 10.1074/jbc.270.31.18175 7629130

[B22] Dhanani T. Shah S. Gajbhiye N. A. Kumar S. (2017). Effect of extraction methods on yield, phytochemical constituents and antioxidant activity of *Withania somnifera* . Arab J. Chem. 10, s1193–s1199. 10.1016/j.arabjc.2013.02.015

[B23] Dogra S. C. Whitelaw M. L. May B. K. (1998). Transcriptional activation of cytochrome P450 genes by different classes of chemical inducers. Clin. Exp. Pharmacol. Physiol. 25, 1–9. 10.1111/j.1440-1681.1998.tb02135.x 9493551

[B24] Duke J. A. (2008). Basil as the Holy Hindu Highness. Altern. Complement. Ther. 14, 5–8. 10.1089/act.2008.14101

[B25] Dzoyem J. P. McGaw L. J. Kuete V. Bakowsky U. (2017). “Chapter 9-Anti-inflammatory and Anti-nociceptive Activities of African Medicinal Spices and Vegetables,” in In Medicinal Spices and Vegetables from Africa. Ed. Kuete V. (Cambridge, MA, USA; Academic Press), 239–270.

[B26] El-Kattan A. Varma M. (2012). “Oral Absorption, Intestinal Metabolism and Human Oral Bioavailability,” in Topics on Drug Metabolism. (InTech). 10.5772/31087

[B27] F.D.A., United States Food and Drug Administration. Clinical Pharmacology Guidelines (2017). *In Vitro* Metabolism- and Transporter-Mediated Drug-Drug Interaction Studies, Guidance for Industry, U.S. Department of Health and Human Services, *Food and Drug Administration, Center for Drug Evaluation and Research (CDER), USA* Available online at: http://www.fda.gov/Drugs/GuidanceComplianceRegulatoryInformation/Guidances/default.htm.

[B28] Fahmi O. A. Shebley M. Palamanda J. Sinz M. W. Ramsden D. Einolf H. J. (2016). CYP2B6 Induction and Prediction of Clinical DDIs: Considerations from the IQ consortium induction working group—an industry perspective. Drug Metab. Dispos. 44, 1720–1730. 10.1124/dmd.116.071076 27422672 PMC11024975

[B29] Fakhroo A. Sreerama L. (2016). Qualitative analysis of phytochemical compounds in Ocimum basilicum grown in Qatar. Int. J. Appl. Pharm. Sci. Bio. Res. 1, 11–17.

[B30] Fasinu P. S. Bouic P. J. Rosenkranz B. (2014). The inhibitory activity of the extracts of popular medicinal herbs on CYP1A2, 2C9, 2C19 and 3A4 and the implications for herb-drug interaction. Afr. J. Tradit. Complement. Altern. Med. 11, 54–61. 10.4314/ajtcam.v11i4.9 PMC4202397 25392581

[B31] Fasinu P. S. Manda V. K. Dale O. R. Egiebor N. O. Walker L. A. Khan S. I. (2017). Modulation of cytochrome P450, P-glycoprotein and pregnane X receptor by selected antimalarial herbs—implication for herb-drug interaction. Molecules 22, 2049. 10.3390/molecules22122049 PMC6150001 29168799

[B32] Fathiazad F. Matlobi A. Khorrami A. Hamedeyazdan S. Soraya H. Hammami M. (2012). Phytochemical screening and evaluation of cardioprotective activity of ethanolic extract of Ocimum basilicum L. (basil) against isoproterenol induced myocardial infarction in rats. DARU J. Pharm. Sci. 20, 87. 10.1186/2008-2231-20-87 PMC3556047 23351503

[B33] Flockhart D. A. (2007). Drug Interactions: Cytochrome P450 drug interaction table (USA: Indiana University School of Medicine). Available online: ‘https://drug-interactions.medicine.iu.edu‘ (Accessed on 12 July 2019).

[B34] Fowler S. Zhang H. (2008). *In vitro* evaluation of reversible and irreversible cytochrome P450 Inhibition: current status on methodologies and their utility for predicting drug–drug interactions. AAPS. J. 10, 410–424. 10.1208/s12248-008-9042-7 18686042 PMC2751392

[B35] Freimoser F. M. Jakob C. A. Aebi M. Tuor U. (1999). The MTT [3-(4,5-Dimethylthiazol-2-yl)- 2,5-diphenyltetrazolium bromide] assay is a fast and reliable method for colorimetric determination of fungal cell densities. Appl. Environ. Microbiol. 65, 3727–3729. 10.1128/AEM.65.8.3727-3729.1999 10427074 PMC91559

[B36] Güez C. M. de Souza R. O. Fischer P. Leão M. F. M. Duarte J. A. Boligon A. A. (2017). Evaluation of basil extract (*Ocimum basilicum* L.) on oxidative, anti-genotoxic and anti-inflammatory effects in human leukocytes cell cultures exposed to challenging agents. Braz. J. Pharm. Sci. 53, e15098. 10.1590/s2175-97902017000115098

[B37] Gavhane Y. N. Yadav A. V. (2012). Loss of orally administered drugs in GI tract. Saudi Pharm. J. 20, 331–344. 10.1016/j.jsps.2012.03.005 23960808 PMC3744959

[B38] Gort A. Falguera M. Schoenenberger J. A. (1997). Rifampicin toxicity in HIV-infected patients: A study of its incidence and the risk factors. An. Med. Interna. 14, 559–564. 9445581

[B39] Habib S. Shaheen B. S. Sabira B. Aslam H. B. (2016). Triterpenoid saponins from the methanol extract of *Ocimum basilicum* aerial parts. J. Chem. Soc Pakistan 38, 1014–1017.

[B40] Habtewold A. Makonnen E. Amogne W. Yimer G. Aderaye G. Bertilsson L. (2015). Is there a need to increase the dose of efavirenz during concomitant rifampicin-based antituberculosis therapy in sub-Saharan Africa? The HIV-TB pharmagene study. Pharmacog. 16, 1047–1064. 10.2217/pgs 25831219

[B41] Hagihara K. Nishiya Y. Kurihara A. Kazui M. Farid N. A. Ikeda T. (2008). Comparison of human cytochrome P450 inhibition by the thienopyridines prasugrel, clopidogrel, and ticlopidine. Drug Metab. Pharmacokinet. 23, 412–420. 10.2133/dmpk.23.412 19122335

[B42] Harborne J. B. (1973). Phytochemical Methods - A Guide to Modern Techniques of Plant Analysis (London: Chapman and Hall Ltd), 1–279.

[B43] Hedrich W. D. Hassan H. E. Wang H. (2016). Insights into CYP2B6-mediated drug-drug interactions. Acta Pharm. Sin. B. 6, 413–425. 10.1016/j.apsb.2016.07.016 27709010 PMC5045548

[B44] Hossain M. B. Rai D. K. Brunton N. P. Martin-Diana A. B. Barry-Ryan C. (2010). Characterization of phenolic composition in Lamiaceae spices by LC-ESI-MS/MS. J. Agric. Food Chem. 58, 10576–10581. 10.1021/jf102042g 20825192

[B45] Iqbal E. Salim A. K. Lim L. B. L. (2015). Phytochemical screening, total phenolics and antioxidant activities of bark and leaf extracts of *Goniothalamus velutinus* (Airy Shaw) from Brunei Darussalam. J. King Saud Univ. Sci. 27, 224–232. 10.1016/j.jksus.2015.02.003

[B46] Jackson J. P. Freeman K. M. Friley W. W. Herman A. G. Black C. B. Brouwer K. R. (2017). Prediction of clinically relevant herb-drug clearance interactions using sandwich-cultured human hepatocytes. Schisandra spp. Case Study. Drug Metab. Dispos. 45, 1019–1026. 10.1124/dmd.117.075408 28698304

[B47] Jadhav B. K. Khandelwal K. R. Ketkar A. R. Pisal S. S. (2004). Formulation and evaluation of mucoadhesive tablets containing eugenol for the treatment of periodontal diseases. Drug Dev. Ind. Pharm. 30, 195–203. 10.1081/ddc-120028715 15089054

[B48] Jamis-Dow C. A. Katki A. G. Collins J. M. Klecker R. W. (1997). Rifampin and rifabutin and their metabolism by human liver esterases. Xenobiotica 27, 1015–1024. 10.1080/004982597239994 9364739

[B49] Jeurissen S. M. Bogaards J. J. Awad H. M. Boersma M. G. Brand W. Fiamegos Y. C. (2004). Human cytochrome p450 enzyme specificity for bioactivation of safrole to the proximate carcinogen 1′-hydroxysafrole. Chem. Res. Toxicol. 17, 1245–1250. 10.1021/tx040001v 15377158

[B50] Jeurissen S. M. Claassen F. W. Havlik J. Bouwmans E. E. Cnubben N. H. Sudhölter E. J. (2007). Development of an on-line high performance liquid chromatography detection system for human cytochrome P450 1A2 inhibitors in extracts of natural products. J. Chromatogr. A. 1141, 81–89. 10.1016/j.chroma.2006.12.007 17184784

[B51] Jeurissen S. M. Punt A. Boersma M. G. Bogaards J. J. Fiamegos Y. C. Schilter B. (2007). Human cytochrome P450 enzyme specificity for the bioactivation of estragole and related alkenylbenzenes. Chem. Res. Toxicol. 20, 798–806. 10.1021/tx700012d 17407329

[B52] Křížková J. Burdová K. Stiborová M. Křen V. Hodek P. (2009). The effects of selected flavonoids on cytochromes P450 in rat liver and small intestine. Interdis. Tox. 2, 201–204. 10.2478/v10102-009-0018-y PMC2984105 21217855

[B53] Karakurt S. (2016). Modulatory effects of rutin on the expression of cytochrome P450s and antioxidant enzymes in human hepatoma cells. Acta Pharm. 66, 491–502. 10.1515/acph-2016-0046 27749250

[B54] Kiferle C. Lucchesini M. Mensuali-Sodi A. Maggini R. Raffaelli A. Pardossi A. (2011). Rosmarinic acid content in basil plants grown in vitro and in hydroponics. Cent. Eur. J. Biol. 6, 946. 10.2478/s11535-011-0057-1

[B55] Kumar S. Bouic P. J. Rosenkranz B. (2017). Simultaneous HPLC determination of efavirenz, 8-hydroxy efavirenz, neostigmine and comparison of their separation using a C18 and biphenyl column through pharmacological evaluation. Indian J. Pharm. Sci. 79, 353–360. 10.4172/pharmaceutical-sciences.1000237

[B56] Kumar S. Sephuhle N. Bouic P. J. Rosenkranz B. (2018). HPLC/LC-MS guided phytochemical and *in vitro* screening of *Astragalus membranaceus* (Fabaceae), and prediction of possible interactions with CYP2B6. J. Herb. Med. 14, 35–47. 10.1016/j.hermed.2018.10.008

[B57] Kumar S. Bouic P. J. Rosenkranz B. (2019). A validated stable HPLC method for the simultaneous determination of rifampicin and 25-O-desacetyl rifampicin – evaluation of in vitro metabolism. Acta Chromatogr. 31, 92–98. 10.1556/1326.2018.00361

[B58] Kwara A. Ramachandran G. Swaminathan S. (2010). Dose adjustment of the non-nucleoside reverse transcriptase inhibitors during concurrent rifampicin-containing tuberculosis therapy: one size does not fit all. Expert Opin. Drug Metab. Toxicol. 6, 55–68. 10.1517/17425250903393752 19968575 PMC2939445

[B59] Loughrin J. H. Kasperbauer M. J. (2003). Aroma content of fresh basil (*Ocimum basilicum* L.) leaves is affected by light reflected from colored mulches. J. Agric. Food Chem. 51, 2272–2276. 10.1021/jf021076c 12670169

[B60] Manikandan P. Murugan R. S. Abbas H. Abraham S. K. Nagini S. (2007). *Ocimum sanctum* Linn. (Holy Basil) ethanolic leaf extract protects against 7,12-dimethylbenz(a)anthracene-induced genotoxicity, oxidative stress, and imbalance in xenobiotic-metabolizing enzymes. J. Med. Food. 10, 495–502. 10.1089/jmf.2006.125 17887944

[B61] Manosroi J. Dhumtanom P. Manosroi A. (2006). Anti-proliferative activity of essential oil extracted from Thai medicinal plants on KB and P388 cell lines. Cancer Lett. 235, 114–120. 10.1016/j.canlet.2005.04.021 15979235

[B62] Marks D. J. Dheda K. Dawson R. Ainslie G. Miller R. F. (2009). Adverse events to antituberculosis therapy: influence of HIV and antiretroviral drugs. Int. J. STD. AIDS. 20, 339–345. 10.1258/ijsa.2008.008361 19386972

[B63] Mena P. Calani L. Dall’Asta C. Galaverna G. García-Viguera C. Bruni R. (2012). Rapid and comprehensive evaluation of (Poly) phenolic compounds in pomegranate (*Punica granatum* L.) juice by UHPLC-MSn. Molecules 17, 14821–14840. 10.3390/molecules171214821 23519255 PMC6268091

[B64] Mudie D. M. Murray K. Hoad C. L. Pritchard S. E. Garnett M. C. Amidon G. L. (2014). Quantification of gastrointestinal liquid volumes and distribution following a 240 mL dose of water in the fasted state. Mol. Pharmaceutics 11, 3039–3047. 10.1021/mp500210c 25115349

[B65] Muráriková A. Ťažký A. Neugebauerová J. Planková A. Jampílek J. Mučaji P. (2017). Characterization of essential oil composition in different basil species and pot cultures by a GC-MS method. Molecules 22, 1221. 10.3390/molecules22071221 PMC6152153 28726757

[B66] Nagarajappa S. H. Pandit S. Divanji M. Mariyanna B. Kumar P. Godavarthi A. (2016). Effect of *Coleus forskohlii* and its major constituents on cytochrome P450 induction. J. Tradit. Complement. Med. 6, 130–133. 10.1016/j.jtcme.2014.11.027 26870691 PMC4737967

[B67] Ngaimisi E. Habtewold A. Minzi O. Makonnen E. Mugusi S. Amogne W. (2013). Importance of ethnicity, CYP2B6 and ABCB1 genotype for efavirenz pharmacokinetics and treatment outcomes: A parallel-group prospective cohort study in two Sub-Saharan Africa populations. PloS One 8, e67946. 10.1371/journal.pone.0067946 23861838 PMC3702506

[B68] Nguyen S. Huang H. Foster B. C. Tam T. W. Xing T. Smith M. L. (2014). Antimicrobial and P450 inhibitory properties of common functional foods. J. Pharm. Pharm. Sci. 17, 254–265. 10.18433/J3P599 24934554

[B69] Nomeir A. A. Palamanda J. R. Favreau L. (2004). “Identification of CYP mechanism-based inhibitors,” in Optimization in Drug Discovery. Methods in Pharmacology and Toxicology. Eds. Yan Z. Caldwell G. W. (USA: Humana Press), 245–262.

[B70] Nurzynska-Wierdak R. Bogucka-Kocka A. Kowalski R. Borowski B. (2012). Changes in the chemical composition of the essential oil of sweet basil (*Ocimum basilicum* L.) depending on the plant growth stage. Chemija 23, 216–222.

[B71] Pan Y. Tiong K. H. Abd-Rashid B. A. Ismail Z. Ismail R. Mak J. W. (2014). *In vitro* effect of important herbal active constituents on human cytochrome P450 1A2 (CYP1A2) activity. Phytomedicine 21, 1645–1650. 10.1016/j.phymed.2014.08.003 25442272

[B72] Park D. Jeon J. H. Shin S. Joo S. S. Kang D. H. Moon S. H. (2009). Green tea extract increases cyclophosphamide induced teratogenesis by modulating the expression of cytochrome P-450 mRNA. Reprod. Toxicol. 27, 79–84. 10.1016/j.reprotox.2008.11.058 19103281

[B73] Plomp T. A. Battista H. J. Unterdorfer H. van Ditmarsch W. C. Maes R. A. (1981). A case of fatal poisoning by rifampicin. Arch. Toxicol. 48, 245–252. 10.1007/bf00319652 7316759

[B74] Polsky-Fisher S. L. Cao H. Lu P. Gibson C. R. (2006). Effect of cytochromes P450 chemical inhibitors and monoclonal antibodies on human liver microsomal esterase activity. Drug Metab. Dispos. 34, 1361–1366. 10.1124/dmd.106.009704 16720683

[B75] Prueksaritanont T. Chu X. Gibson C. Cui D. Yee K. L. Ballard J. (2013). Drug-drug interaction studies: regulatory guidance and an industry perspective. AAPS J. 15, 629–645. 10.1208/s12248-013-9470-x 23543602 PMC3691435

[B76] Punyasiri P. A. N. Jeganathan B. Kottawa-Arachchi D. J. Ranatunga M. A. B. Abeysinghe I. S. B. Gunasekare M. T. K. (2015). New Sample Preparation Method for quantification of phenolic compounds of tea (*Camellia sinensis* L. Kuntze): A polyphenol rich plant. J. Anal. Methods Chem., 964341. 10.1155/2015/964341 26543665 PMC4620427

[B77] Quintieri L. Palatini P. Nassi A. Ruzza P. Floreani M. (2008). Flavonoids diosmetin and luteolin inhibit midazolam metabolism by human liver microsomes and recombinant CYP 3A4 and CYP3A5 enzymes. Biochem. Pharmacol. 75, 1426–1437. 10.1016/j.bcp.2007.11.012 18191104

[B78] Raaman N. (2006). Phytochemical Technique Vol. 19 (New Delhi: New Indian Publishing Agencies).

[B79] Rai R. (2016). Herbal remedies in cure of tuberculosis prevalent among ethnic communities in Central India. Trop. Plant Res. 3, 344–353.

[B80] Ramos R. T. M. Bezerra I. C. F. Ferreira M. R. A. Soares L. A. L. (2017). Spectrophotometric Quantification of flavonoids in herbal material, crude extract, and fractions from leaves of *Eugenia uniflora* Linn. Pharmacognosy Res. 9, 253–260. 10.4103/pr.pr_143_16 28827966 PMC5541481

[B81] Rasekh H. R. Hosseinzadeh L. Mehri S. Kamli-Nejad M. Aslani M. Tanbakoosazan F. (2012). Safety assessment of *Ocimum basilicum* hydroalcoholic extract in wistar rats: acute and subchronic toxicity studies. Iran J. Basic. Med. Sci. 15, 645–653. 10.22038/ijbms.2012.4833 23493182 PMC3586872

[B82] Said R. B. Hamed A. I. Mahalel U. A. Al-Ayed A. S. Kowalczyk M. Moldoch J. (2017). Tentative characterization of polyphenolic compounds in the male flowers of *Phoenix dactylifera* by liquid chromatography coupled with mass spectrometry and DFT. Int. J. Mol. Sci. 18, 512. 10.3390/ijms18030512 PMC5372528 28257091

[B83] Shang P. Xia Y. Liu F. Wang X. Yuan Y. Hu D. (2011). Incidence, clinical features and impact on anti-tuberculosis treatment of anti-tuberculosis drug induced liver injury (ATLI) in China. PloS One 6, e21836. 10.1371/journal.pone.0021836 21750735 PMC3130045

[B84] Shengule S. Kumbhare K. Patil D. Mishra S. Apte K. Patwardhan B. (2018). Herb-drug interaction of *Nisha Amalaki* and Curcuminoids with metformin in normal and diabetic condition: A disease system approach. Biomed. Pharmacother. 101, 591–598. 10.1016/j.biopha.2018.02.032 29518605

[B85] Siddiqui B. S. Bhatti H. A. Begum S. Perwaiz S. (2012). Evaluation of the antimycobacterium activity of the constituents from *Ocimum basilicum* against *Mycobacterium tuberculosis* . J. Ethnopharmacol. 144, 220–222. 10.1016/j.jep.2012.08.003 22982011

[B86] Simirgiotis M. J. Benites J. Areche C. Sepúlveda B. (2015). Antioxidant capacities and analysis of phenolic compounds in three endemic *Nolana* species by HPLC-PDA-ESI-MS. Molecules 20, 11490–11507. 10.3390/molecules200611490 26111178 PMC6272610

[B87] Son J. S. Chang Y. J. Choi Y. D. Kim S. U. (1998). Role of jasmonic acid in biotransformation of (–)-isopiperitenone in suspension cell culture of *Mentha piperita* . Mol. Cells 8, 366–369. 9666476

[B88] Sonar V. P. Corona A. Distinto S. Maccioni E. Meleddu R. Fois B. (2017). Natural product-inspired esters and amides of ferulic and caffeic acid as dual inhibitors of HIV-1 reverse transcriptase. Eur. J. Med. Chem. 130, 248–260. 10.1016/j.ejmech.2017.02.054 28254698

[B89] Sridhar A. Sandeep Y. Krishnakishore C. Sriramnaveen P. Manjusha Y. Sivakumar V. (2012). Fatal poisoning by isoniazid and rifampicin. Indian. J. Nephrol. 22, 385–387. 10.4103/0971-4065.103930 23326053 PMC3544064

[B90] Stander M. A. Van Wyk B. E. Taylor M. J. C. Long H. S. (2017). Analysis of phenolic compounds in rooibos tea (*Aspalathus linearis*) with a comparison of flavonoid-based compounds in natural populations of plants from different regions. J. Agric. Food Chem. 65, 10270–10281. 10.1021/acs.jafc 29063755

[B91] Stresser D. M. Mao J. Kenny J. R. Jones B. C. Grime K. (2014). Exploring concepts of *in vitro* time-dependent CYP inhibition assays. Expert Opin. Drug Metab. Toxicol. 10, 157–174. 10.1517/17425255.2014.856882 24256452

[B92] Swart M. Ren Y. Smith P. Dandara C. (2012). ABCB1 4036A>G and 1236C>T polymorphisms affect plasma efavirenz levels in South African HIV/AIDS Patients. Front. Genet. 3, 236. 10.3389/fgene.2012.00236 23133441 PMC3488761

[B93] Tang D. Chen K. Huang L. Li J. (2017). Pharmacokinetic properties and drug interactions of apigenin, a natural flavone. Expert Opin. Drug Metab. Toxicol. 13, 323–330. 10.1080/17425255.2017.1251903 27766890

[B94] Thomford N. E. Dzobo K. Chopera D. Wonkam A. Maroyi A. Blackhurst D. (2016). *In Vitro* reversible and time-dependent CYP450 inhibition profiles of medicinal herbal plant extracts *Newbouldia laevis* and *Cassia abbreviata*: implications for Herb-Drug Interactions. Molecules 21, 891. 10.3390/molecules21070891 PMC6274561 27399660

[B95] Thring T. S. A. Weitz F. M. (2006). Medicinal plant use in the Bredasdorp/ Elim region of the Southern Overberg in the Western Cape Province of South Africa. J. Ethnopharmacol. 103, 261–275. 10.1016/j.jep.2005.08.013 16169694

[B96] Tracy T. S. (2006). Atypical cytochrome p450 kinetics: implications for drug discovery. Drugs R. D. 7, 349–363. 10.2165/00126839-200607060-00004 17073518

[B97] Tukappa N. K. A. Londonkar R. L. Nayaka H. B. Kumar C. B. S. (2015). Cytotoxicity and hepatoprotective attributes of methanolic extract of *Rumex vesicarius* L. Biol. Res. 48, 19. 10.1186/s40659-015-0009-8 25857314 PMC4384386

[B98] Vallverdú-Queralt A. Regueiro J. Alvarenga J. F. R. Martinez-Huelamo M. Leal L. N. Lamuela-Raventos R. M. (2015). Characterization of the phenolic and antioxidant profiles of selected culinary herbs and spices: caraway, turmeric, dill, marjoram and nutmeg. Food Sci. Technol. 35, 189–195. 10.1590/1678-457X.6580

[B99] Varghese A. Pandita N. Gaud R. S. (2014). *In vitro* and *in vivo* evaluation of CYP1A interaction potential of *Terminalia arjuna* bark. Indian J. Pharm. Sci. 76, 138–147. 24843187 PMC4023283

[B100] W.H.O. Monographs (2005). WHO Monographs on Selected Medicinal Plants Vol. 4 (Salerno-Paestum, Italy: World Health Organization).

[B101] Wang S. Liu L. Wang L. Hu Y. Zhang W. Liu R. (2012). Structural characterization and identification of major constituents in *Jitai* tablets by high-performance liquid chromatography/diode-array detection coupled with electrospray ionization tandem mass spectrometry. Molecules 17, 10470–10493. 10.3390/molecules170910470 22945027 PMC6268525

[B102] Ward B. A. Gorski J. C. Jones D. R. Hall S. D. Flockhart D. A. Desta Z. (2003). The cytochrome P450 2B6 (CYP2B6) is the main catalyst of efavirenz primary and secondary metabolism: implication for HIV/AIDS therapy and utility of efavirenz as a substrate marker of CYP2B6 catalytic activity. J. Pharmacol. Exp. Ther. 306, 287–300. 10.1124/jpet.103.049601 12676886

[B103] Weiss J. (2019). Herb–drug interaction potential of anti-borreliae effective extracts from *Uncaria tomentosa* (Samento) and *Otoba parvifolia* (Banderol) assessed *in vitro* . Molecules 24, 137. 10.3390/molecules24010137 PMC6337116 30602711

[B104] Ye M. Guo D. Ye G. Huang C. (2005). Analysis of homoisoflavonoids in *Ophiopogon japonicus* by HPLC-DAD-ESI-MSn1. J. Am. Soc Mass Spectrom. 16, 234–243. 10.1016/j.jasms.2004.11.007 15694773

[B105] Yee D. Valiquette C. Pelletier M. Parisien I. Rocher I. Menzies D. (2003). Incidence of serious side effects from first-line antituberculosis drugs among patients treated for active tuberculosis. Am. J. Respir. Crit. Care Med. 167, 1472–1477. 10.1164/rccm.200206-626OC 12569078

[B106] Zanger U. M. Klein K. (2013). Pharmacogenetics of cytochrome P450 2B6 (CYP2B6): advances on polymorphisms, mechanisms, and clinical relevance. Front. Genet. 4, 24. 10.3389/fgene.2013.00024 23467454 PMC3588594

[B107] Zhang D. Chando T. J. Everett D. W. Patten C. J. Dehal S. S. Humphreys W. G. (2005). *In vitro* inhibition of UDP glucuronosyltransferases by atazanavir and other HIV protease inhibitors and the relationship of this property to *in vivo* bilirubin glucuronidation. Drug Metab. Dispos. 33, 1729–1739. 10.1124/dmd.105.005447 16118329

