# Supplementary figures and images for: In Vitro Assessment of the Interaction Potential of Ocimum basilicum (L.) Extracts on CYP2B6, 3A4, and Rifampicin Metabolism
Source: Front Pharmacol. 2020 Apr 30;11:517. doi: 10.3389/fphar.2020.00517 (PMC7204527; doi:10.3389/fphar.2020.00517)

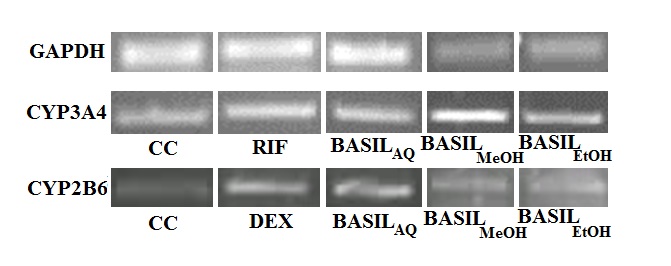

Supplement: Supplementary file 2 [file DataSheet_2.zip › GEL Basil Extracts 1 - 03.10.2019.jpg]

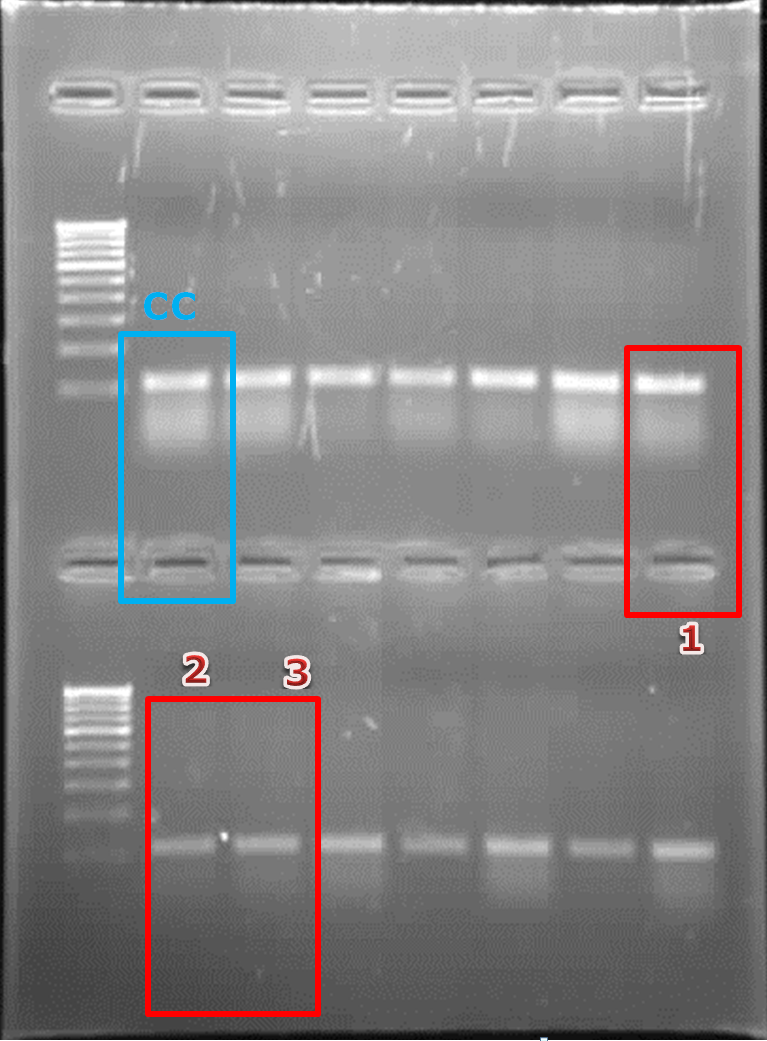

Supplement: Supplementary file 2 [file DataSheet_2.zip › GEL Image 2.jpg]

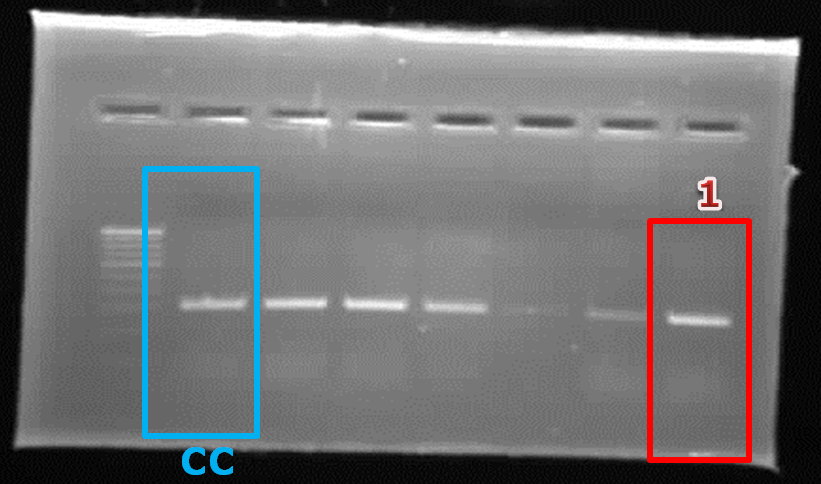

Supplement: Supplementary file 2 [file DataSheet_2.zip › GEL Image 3.jpg]

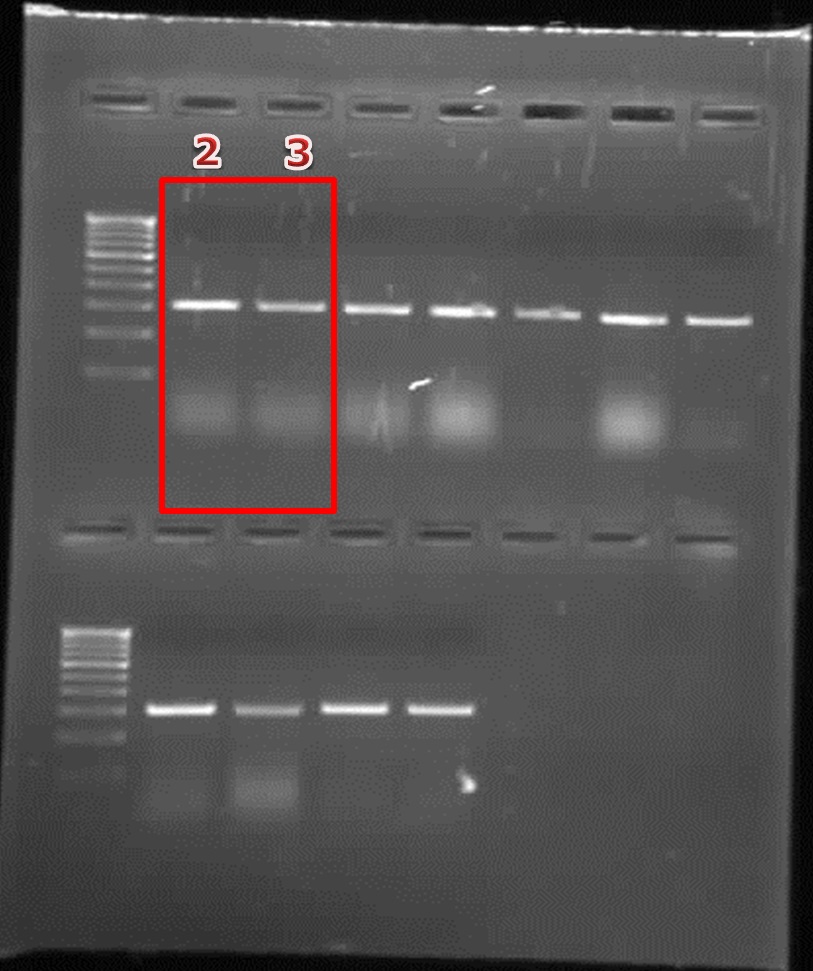

Supplement: Supplementary file 2 [file DataSheet_2.zip › GEL Image 4.jpg]

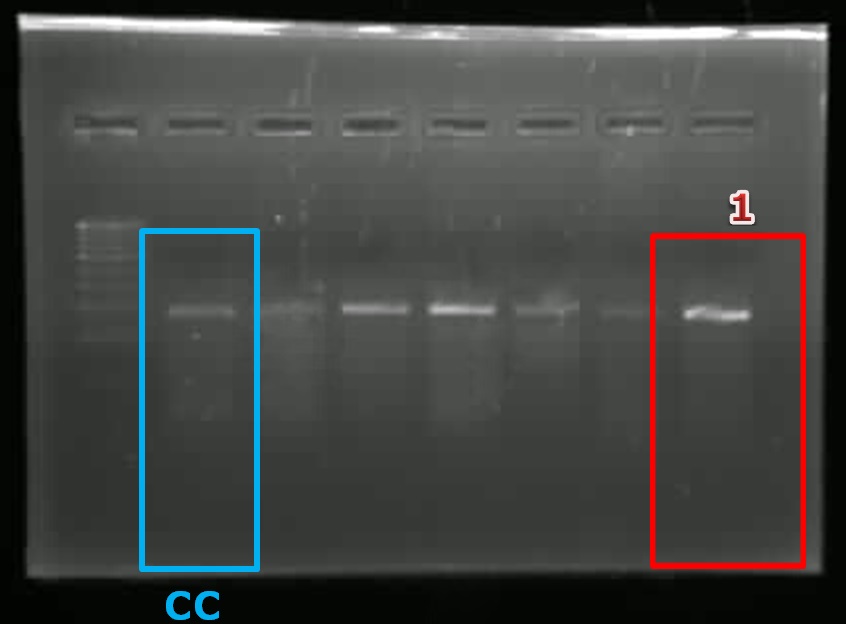

Supplement: Supplementary file 2 [file DataSheet_2.zip › GEL Image 5 (1).jpg]

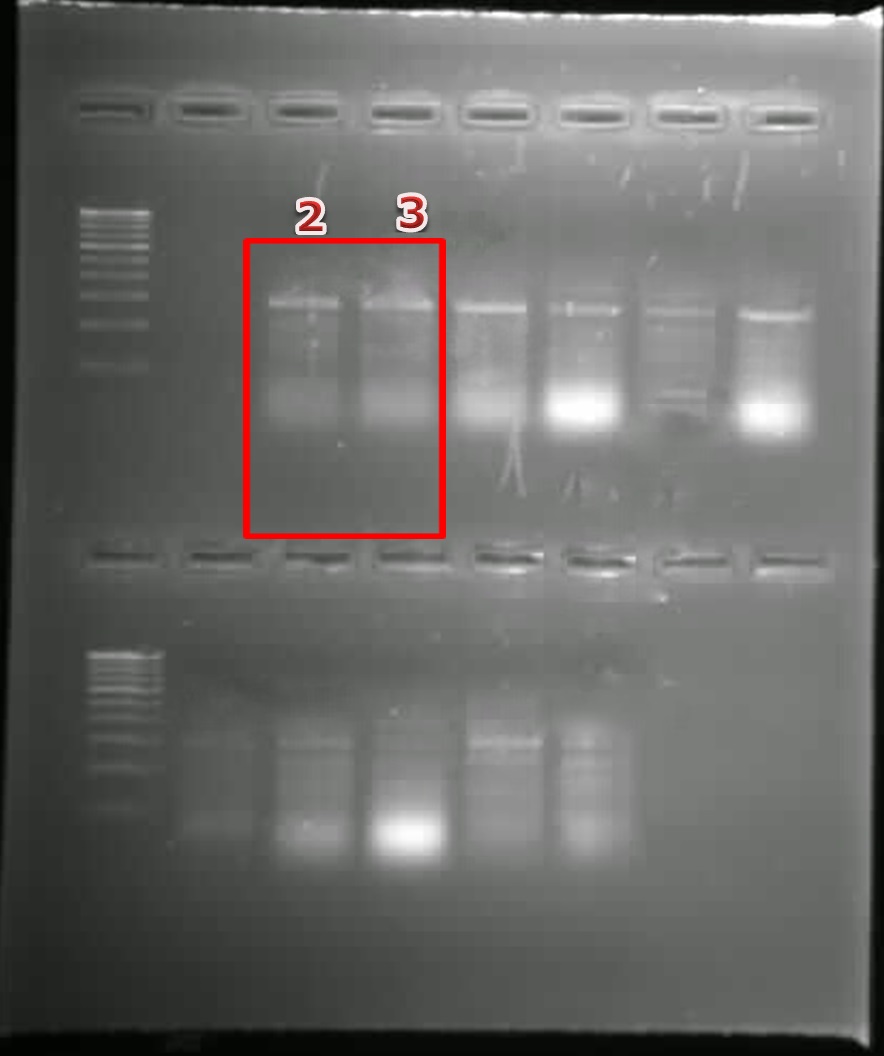

Supplement: Supplementary file 2 [file DataSheet_2.zip › GEL Image 6.jpg]
