# Supplementary material for: In Vitro Assessment of the Interaction Potential of Ocimum basilicum (L.) Extracts on CYP2B6, 3A4, and Rifampicin Metabolism
Source: Front Pharmacol. 2020 Apr 30;11:517. doi: 10.3389/fphar.2020.00517 (PMC7204527; doi:10.3389/fphar.2020.00517)

Supplementary Material

In vitro assessment of the interaction potential of *Ocimum basilicum* (L.) extracts on CYP2B6, 3A4 and rifampicin metabolism

Saneesh Kumar [1]*, Patrick J. Bouic [2, 3], Bernd Rosenkranz [1]

[1] Division of Clinical Pharmacology, Faculty of Medicine and Health Sciences, University of Stellenbosch, Cape Town 7505, South Africa; saneesh.7.kumar@gmail.com, rosenkranz@sun.ac.za

[2] Division of Medical Microbiology, Faculty of Medicine and Health Sciences, University of Stellenbosch, Cape Town 7505, South Africa

[3] Synexa Life Sciences, Omuramba Business Park, Montague Gardens, Cape Town 7441, South Africa; pbouic@synexagroup.com

1 Supplementary Figures

1.1 Specifications table

• Subject area	Natural Herbal Medicine, Phytochemistry, Pharmacology, Ethnopharmacognosy
• More specific subject area	Herb-Drug Interaction
• Type of data	Figures/ Chromatograms
• How data was acquired	HPLC, LC-MS/MS
• Data format	Analyzed.

2 Value of the data

• This dataset will be of value to the scientific community wanting to analyse various herb extracts from different solvent fractions, using reference standards – deploying LC-MS/MS analyses.

• The data may stimulate further research on prediction models of phytochemical fingerprinting versus cytochrome P450 interaction.

• The data also comprises of preliminary *in vitro* screening of the basil extracts against human liver microsomes with efavirenz as the substrate for CYP2B6, and rifampicin as substrate for β–esterases.

The data below describes the LC-MS/MS phytoprofiling of basil extracts selected in this study. The parameters below are used in making the phytochemical compound predictions:

- Accurate masses
- *m/z* transitions (MS/MS fragments)
- UV maxima
- Relative retention times and comparison with literature review on matching compounds
- Online mass spectral repositories such as Metlin Scripps (https://metlin.scripps.edu/), MassBank online Spectral Database (https://massbank.eu/MassBank/), NIST standard reference data online webbook library (http://webbook.nist.gov/chemistry/mw-ser.html), and Pubchem chemistry database (https://pubchem.ncbi.nlm.nih.gov).

3 Supplementary Figures - Information

- Pages 1-4: LC-MS chromatograms of standard calibrators (reference standards) quercetin and gallic acid – phytochemical fingerprinting.
- Pages 5-28: LC-MS/ PDA chromatograms of basil aqueous (Aq), methanol (MeOH) and ethanolic (EtOH) extracts in negative and positive mode.
- Pages 29-78: HPLC chromatograms of basil aqueous (Aq), methanol (MeOH), ethanolic (EtOH) and ethyl acetate (EtOAc) extract-supernatants post microsome incubation assays.
- Pages 79-177: HPLC chromatograms of basil aqueous (Aq), methanol (MeOH), ethanolic (EtOH) and ethyl acetate (EtOAc) extract-supernatants post microsome incubation assays for determination of IC_{50}s and TDI IC_{50}s.
- Pages 178-226: HPLC chromatograms of control nelfinavir (rifampicin) extract-supernatants post microsome incubation assays for determination of IC_{50}s and TDI IC_{50}s.
- Pages 227-320: HPLC chromatograms of control ticlopidine (efavirenz) extract-supernatants post microsome incubation assays for determination of IC_{50}s and TDI IC_{50}s.
- Pages 321-355: HPLC chromatograms of control nelfinavir (rifampicin) extract-supernatants post microsome incubation assays for screening of inhibition activity.
- Pages 356-590: HPLC chromatograms of basil aqueous (Aq), methanol (MeOH), ethanolic (EtOH) and ethyl acetate (EtOAc) extract-supernatants post microsome incubation assays – rifampicin as substrate, 25-O-desacetyl rifampicin (25ODESRIF) as metabolite.
- Pages 591-723: HPLC chromatograms of basil aqueous (Aq), methanol (MeOH), ethanolic (EtOH) and ethyl acetate (EtOAc) extract-supernatants post microsome incubation assays – efavirenz as substrate, 8-hyrdroxy efavirenz (E8H) as metabolite.
- Pages 724-948: HPLC chromatograms of basil aqueous (Aq), methanol (MeOH), ethanolic (EtOH) and ethyl acetate (EtOAc) extract-supernatants post microsome incubation assays – rifampicin as substrate, 25-O-desacetyl rifampicin (25ODESRIF) as metabolite, for two-point screening and other analyses/ repeats.

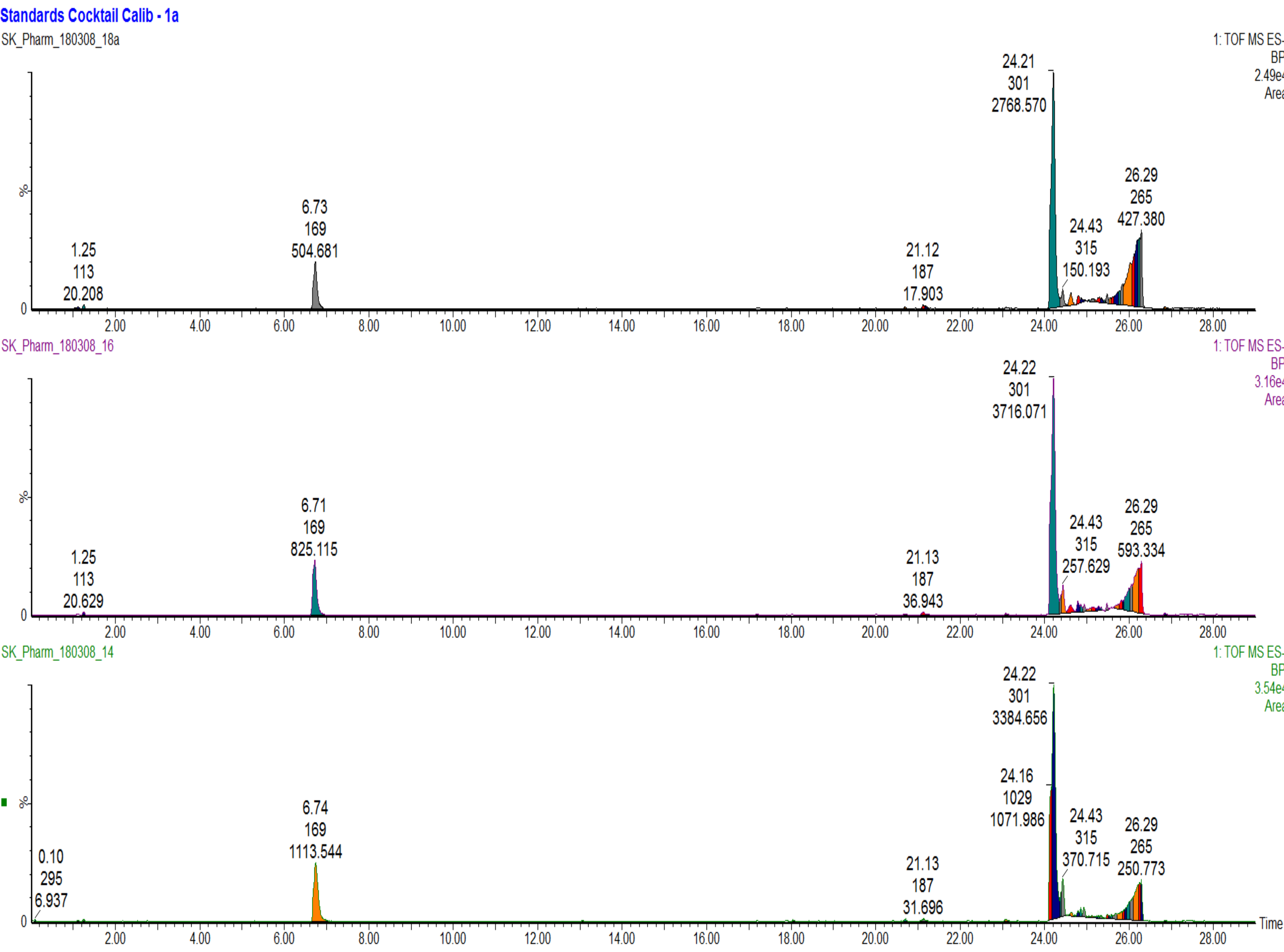
Standards Cocktail Calib - 1a
SK_Pharm_180308_18a
1: TOF MS ES-
BP
2.49e4
Area
24.21
301
2768.570
26.29
265
427.380
24.43
315
150.193
6.73
169
504.681
1.25
113
20.208
21.12
187
17.903
%
SK_Pharm_180308_16
1: TOF MS ES-
BP
3.16e4
Area
24.22
301
3716.071
6.71
169
825.115
24.43
315
257.629
26.29
265
593.334
1.25
113
20.629
21.13
187
36.943
SK_Pharm_180308_14
1: TOF MS ES-
BP
3.54e4
Area
24.22
301
3384.656
24.16
1029
1071.986
24.43
315
370.715
26.29
265
250.773
6.74
169
1113.544
0.10
295
6.937
21.13
187
31.696
Time
2.00
4.00
6.00
8.00
10.00
12.00
14.00
16.00
18.00
20.00
22.00
24.00
26.00
28.00

Standards Cocktail Calib - 6a

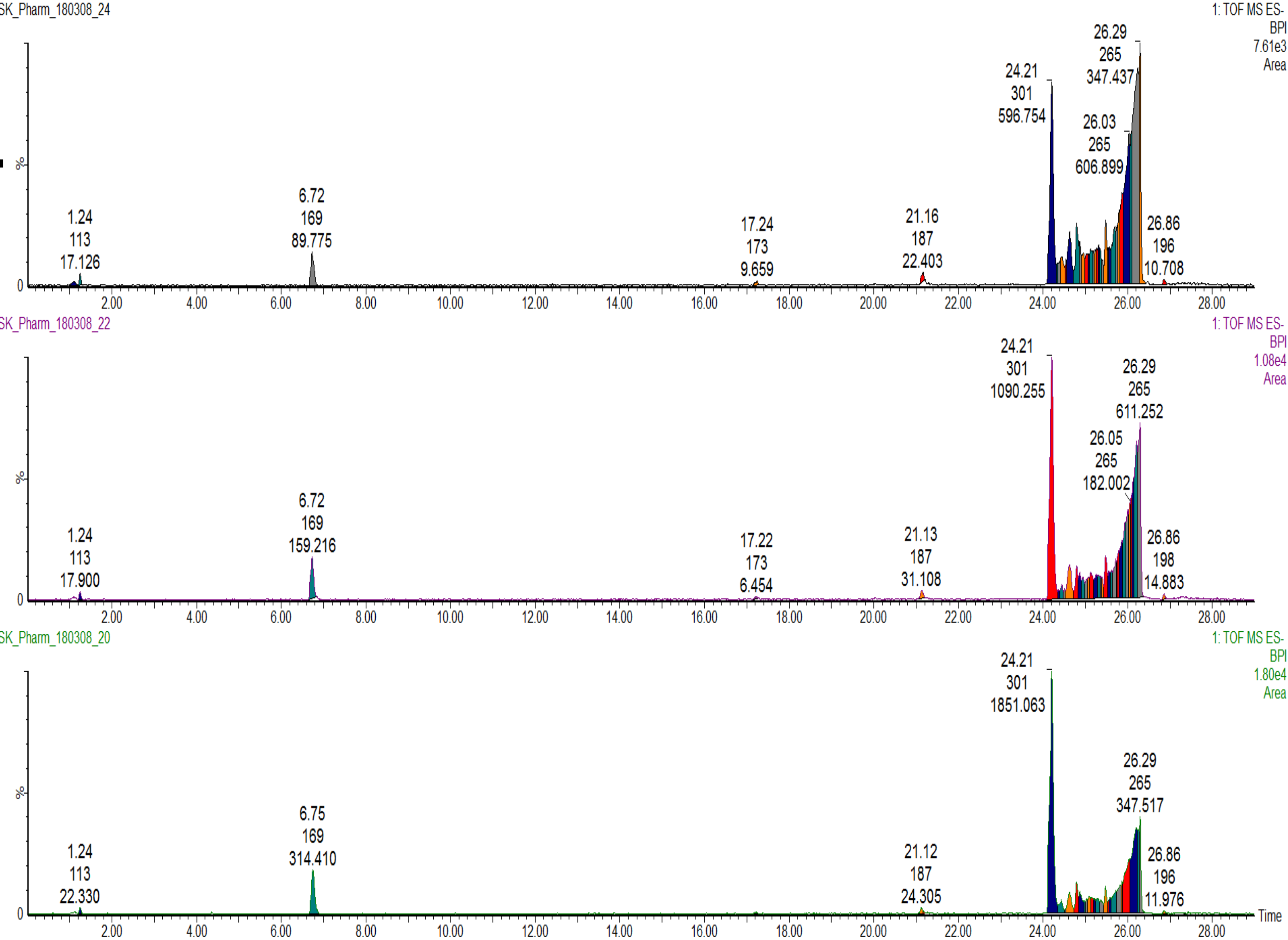

Standards Cocktail Calib - 3b

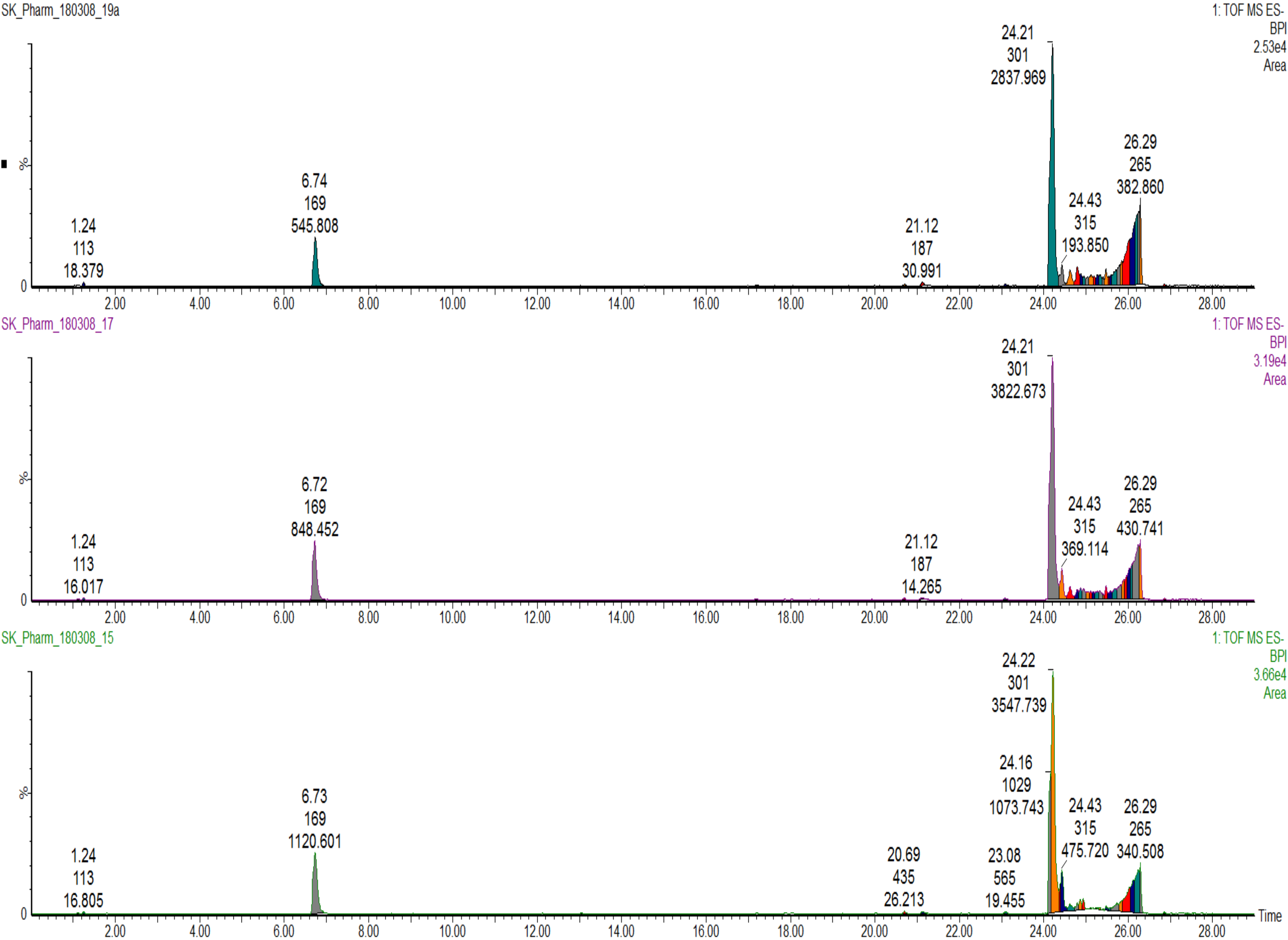

Standards Cocktail Calib - 4b

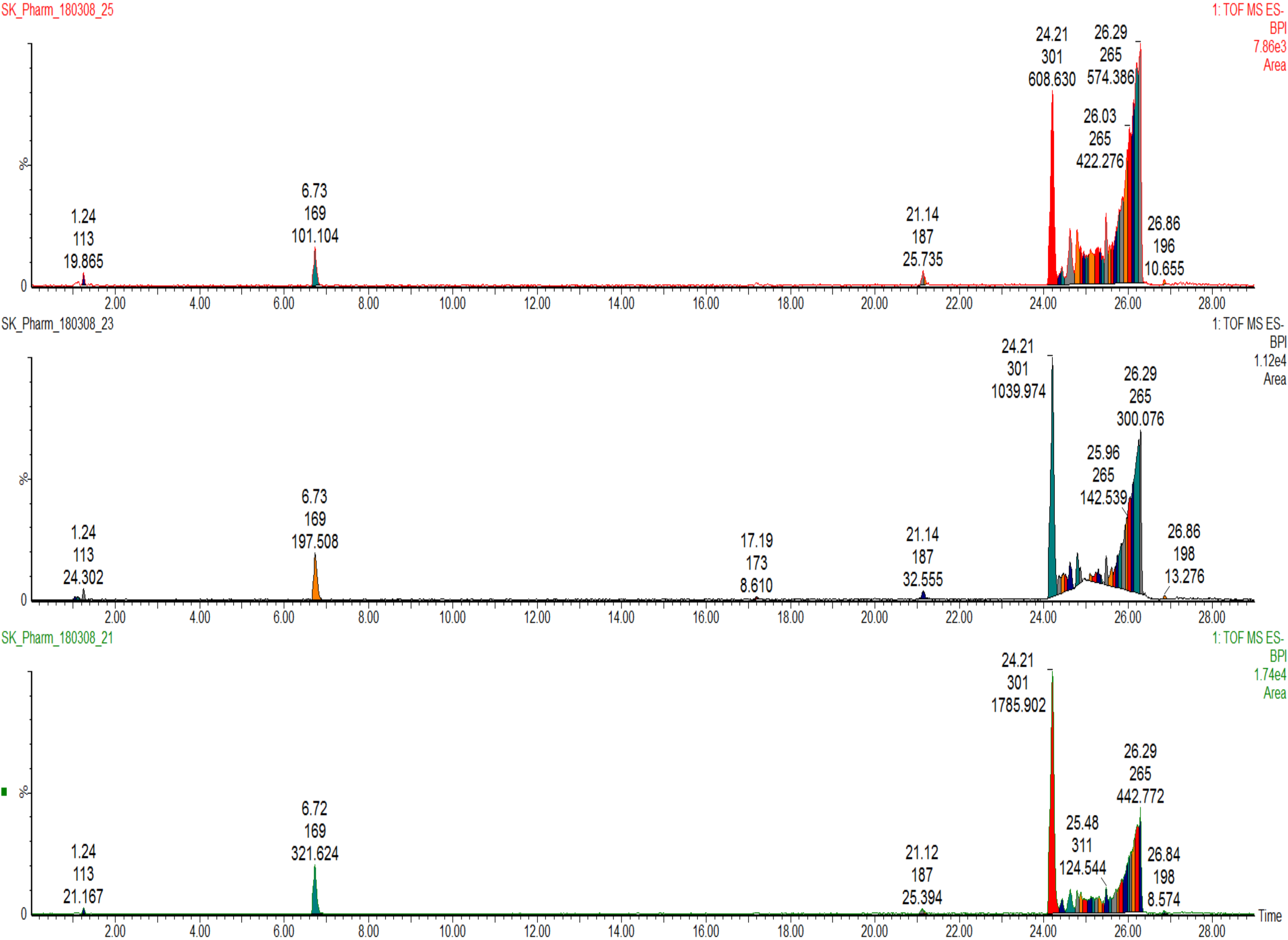

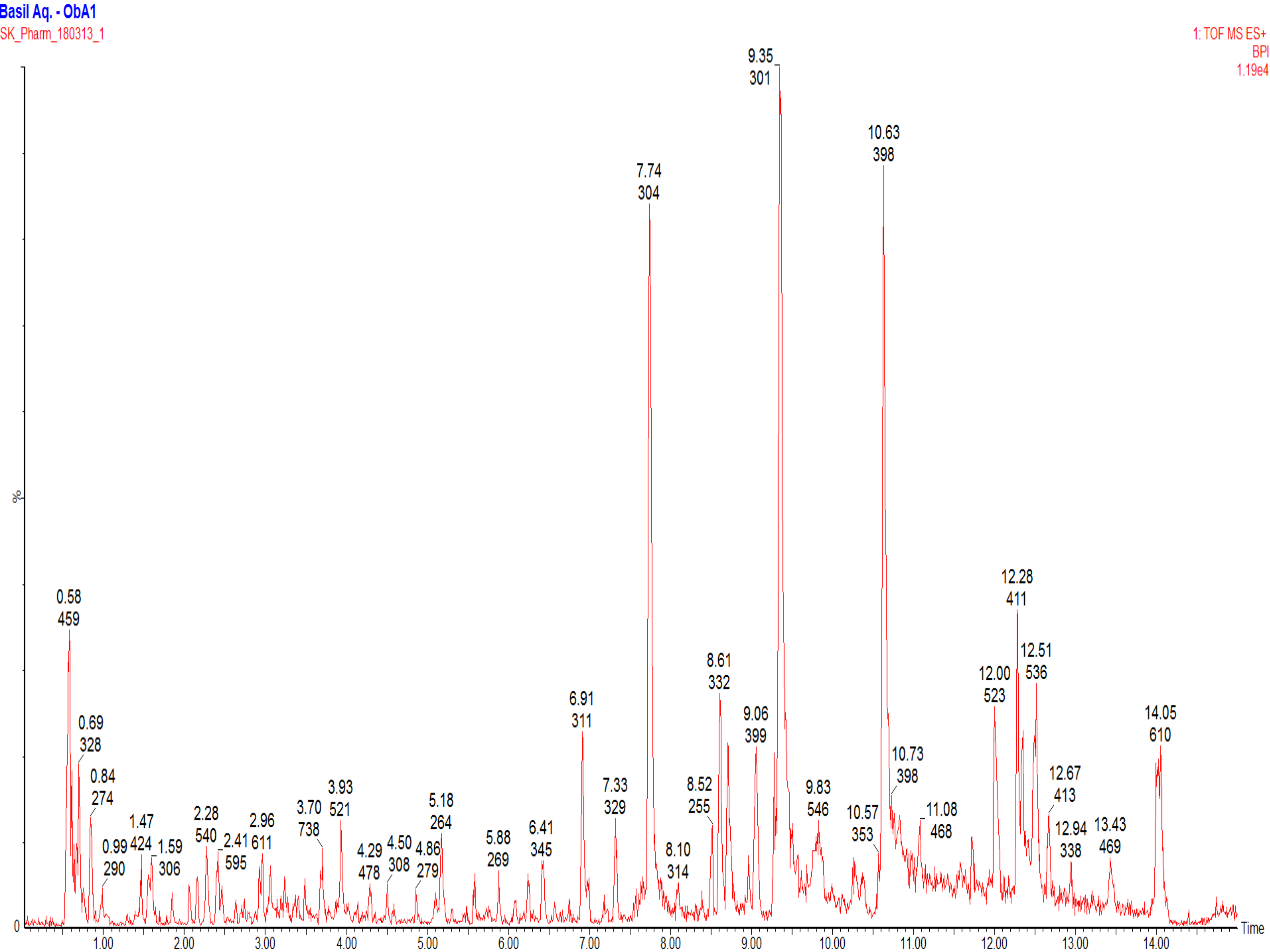
Basil Aq. - ObA1
SK_Pharm_180313_1
1: TOF MS ES+
BPI
1.19e4
9.35
301
10.63
398
7.74
304
12.28
411
0.58
459
12.51
536
12.00
523
8.61
332
6.91
311
9.06
399
14.05
610
0.69
328
10.73
398
7.33
329
8.52
255
9.83
546
12.67
413
0.84
274
3.93
521
5.18
264
3.70
738
6.41
345
10.57
353
11.08
468
12.94
338
13.43
469
1.47
424
2.28
540
2.96
611
0.99
290
1.59
306
2.41
595
4.29
478
4.50
308
4.86
279
5.88
269
8.10
314
%
0
1.00
2.00
3.00
4.00
5.00
6.00
7.00
8.00
9.00
10.00
11.00
12.00
13.00
14.00
Time

Basil Aq. - ObA1

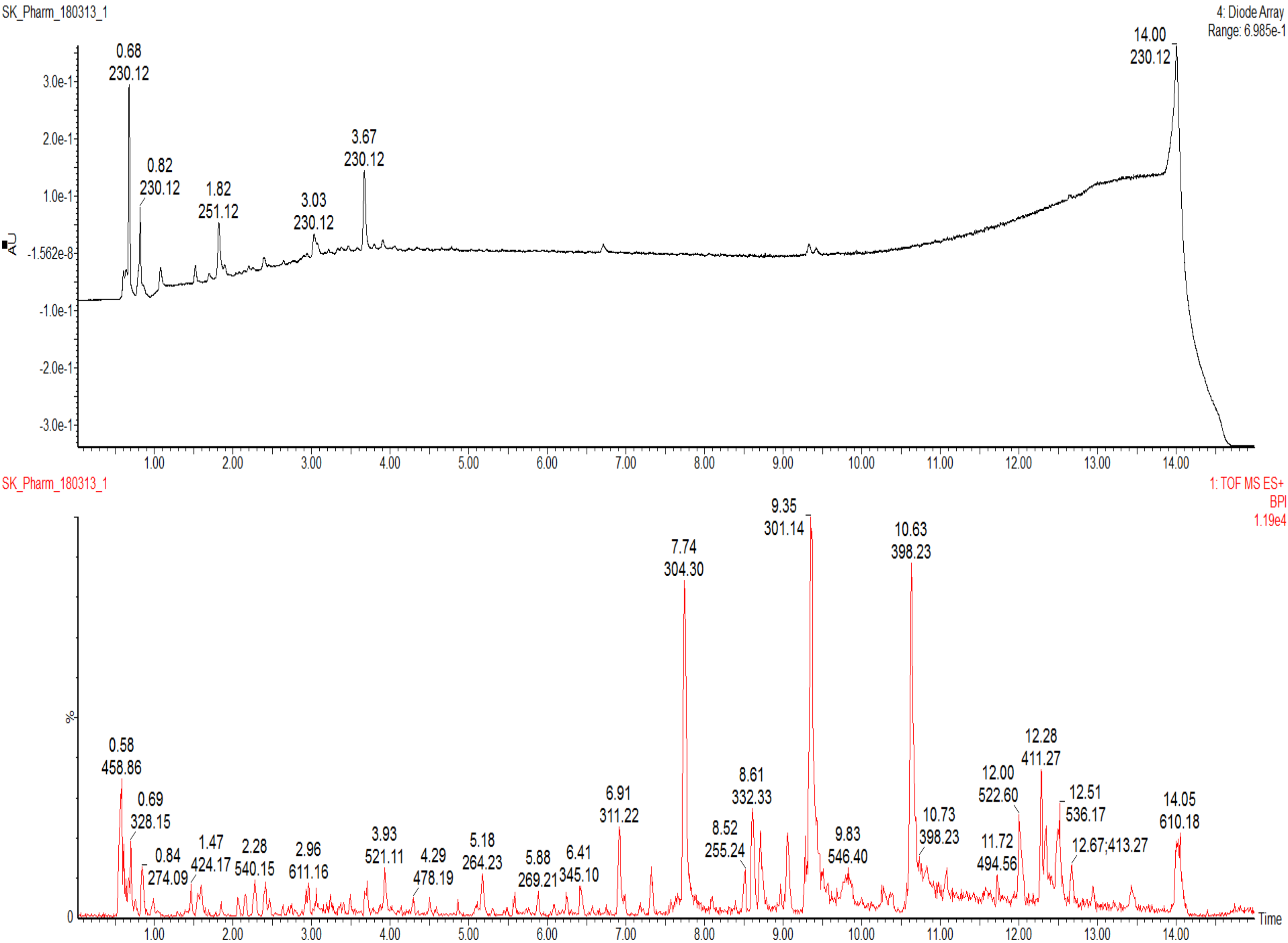

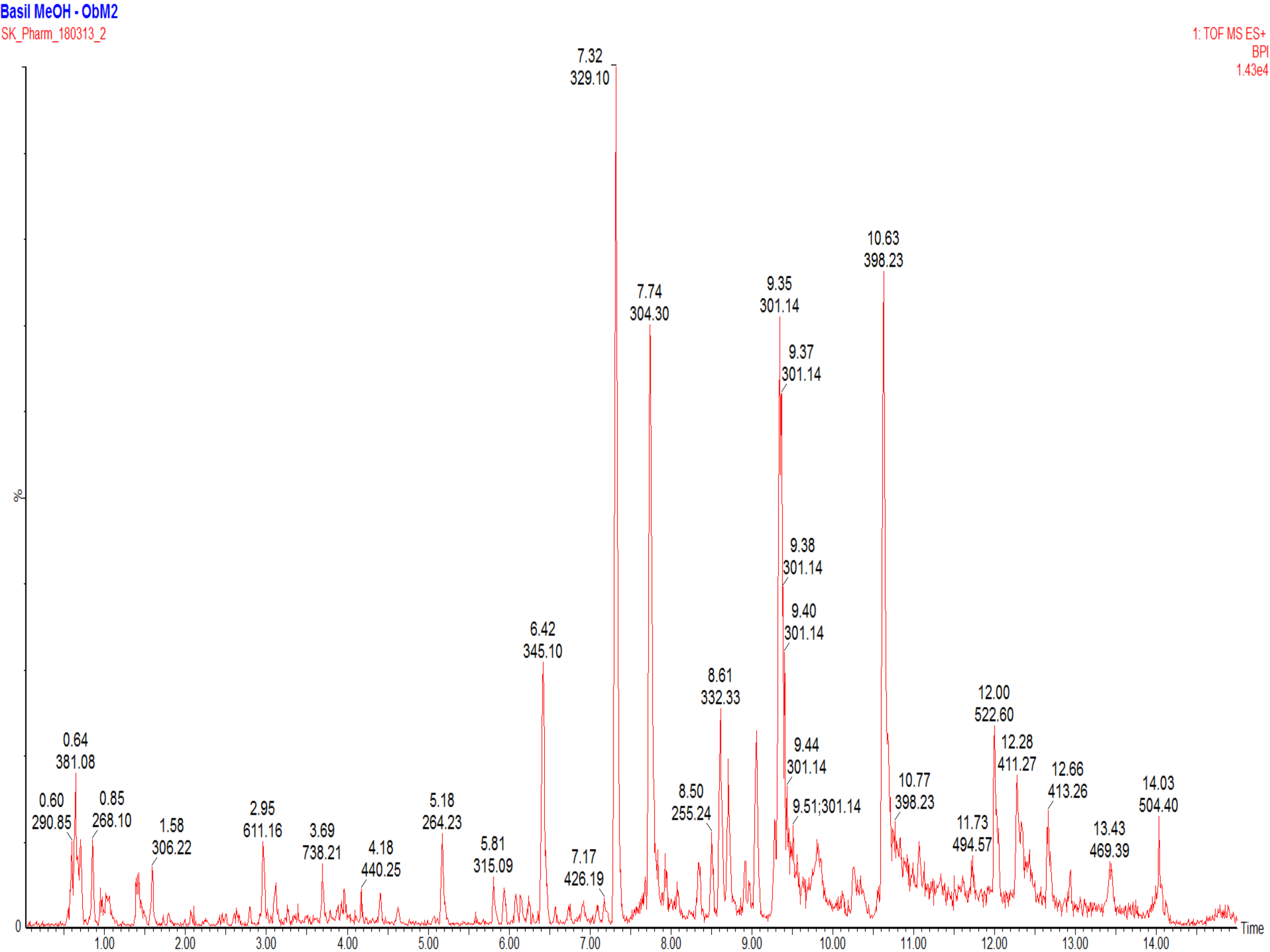
Basil MeOH - ObM2
SK_Pharm_180313_2
1: TOF MS ES+
BPI
1.43e4
%
7.32
329.10
7.74
304.30
9.35
301.14
9.37
301.14
9.38
301.14
9.40
301.14
9.44
301.14
9.51;301.14
10.63
398.23
10.77
398.23
6.42
345.10
8.61
332.33
8.50
255.24
12.00
522.60
12.28
411.27
12.66
413.26
11.73
494.57
13.43
469.39
14.03
504.40
0.64
381.08
0.60
290.85
0.85
268.10
1.58
306.22
2.95
611.16
3.69
738.21
4.18
440.25
5.18
264.23
5.81
315.09
7.17
426.19
0
1.00
2.00
3.00
4.00
5.00
6.00
7.00
8.00
9.00
10.00
11.00
12.00
13.00
14.00
Time

Basil MeOH - ObM2

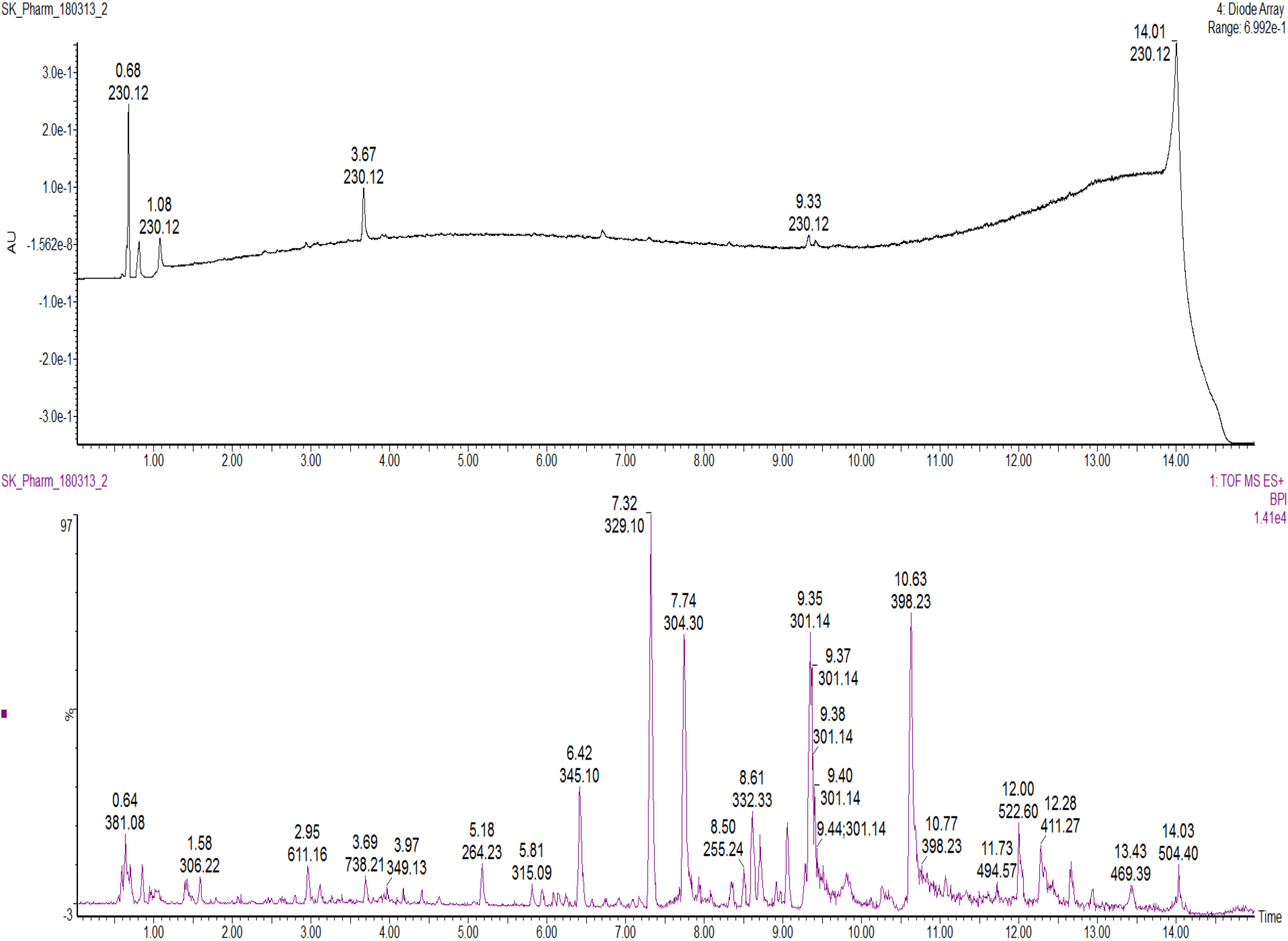

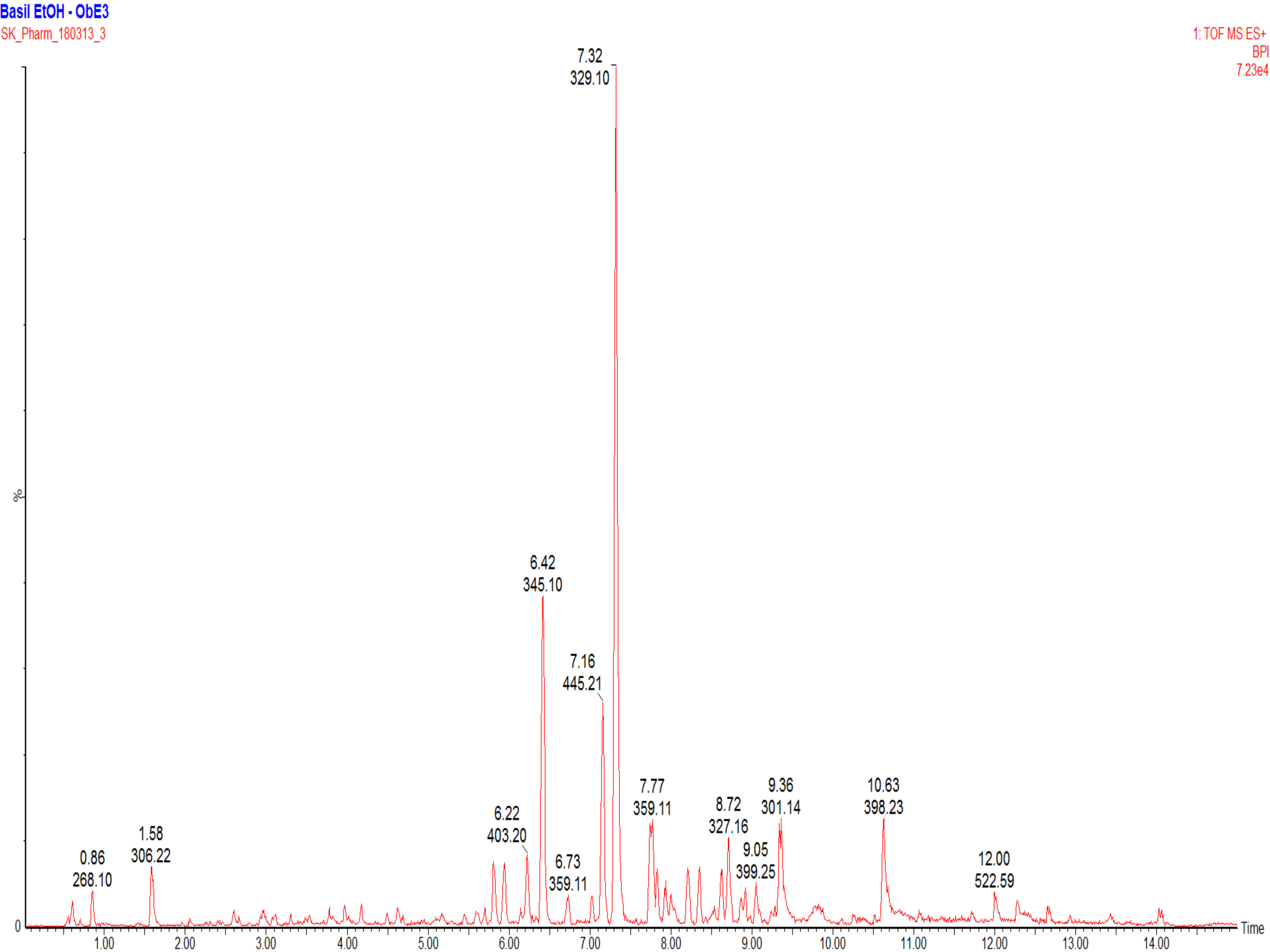

Basil EtOH - ObE3
SK_Pharm_180313_3
1: TOF MS ES+
BPI
7.23e4
%
0
7.32
329.10
6.42
345.10
7.16
445.21
6.22
403.20
6.73
359.11
7.77
359.11
8.72
327.16
9.36
301.14
9.05
399.25
10.63
398.23
12.00
522.59
0.86
268.10
1.58
306.22
1.00
2.00
3.00
4.00
5.00
6.00
7.00
8.00
9.00
10.00
11.00
12.00
13.00
14.00
Time

Basil EtOH - ObE3

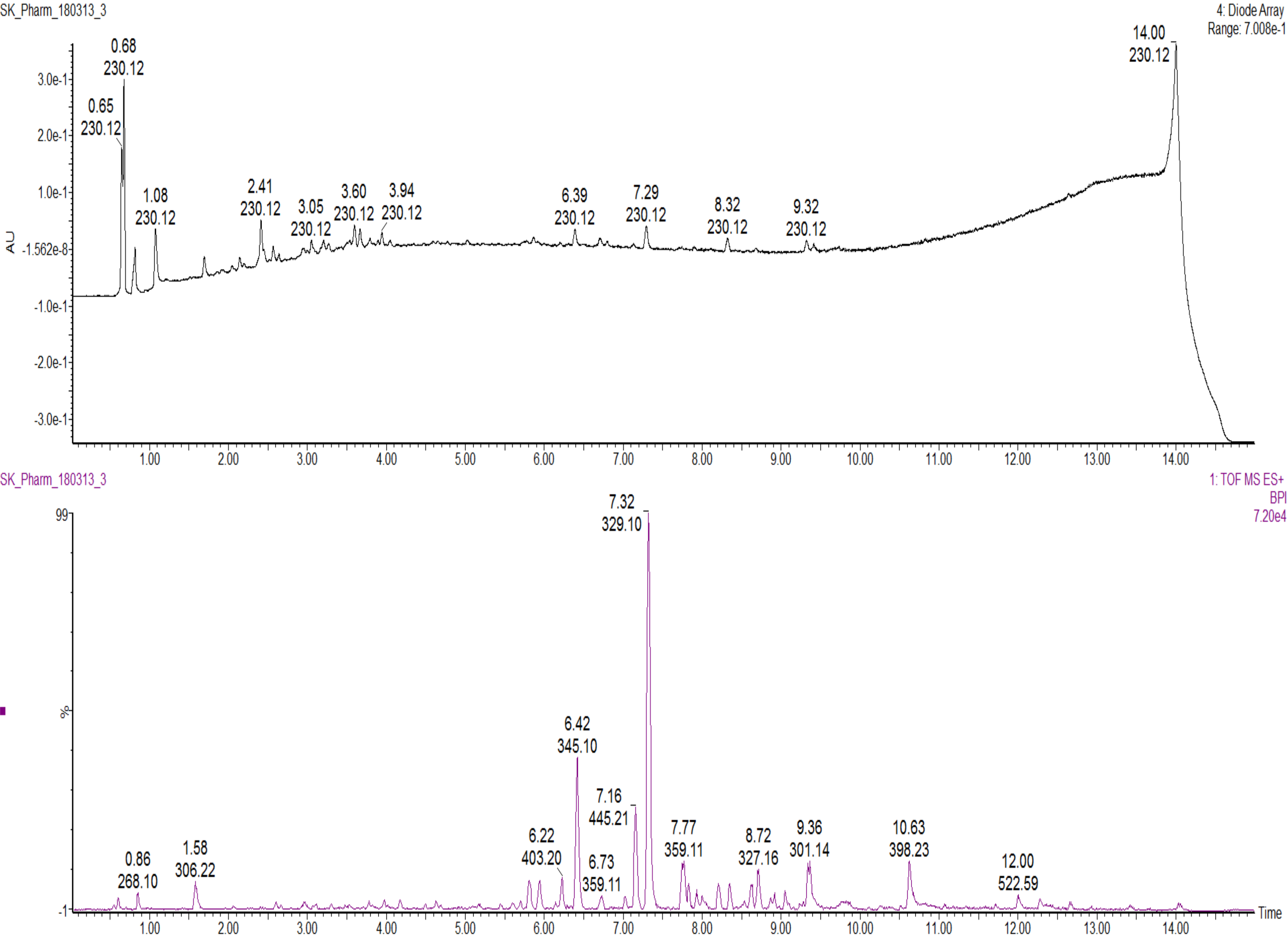

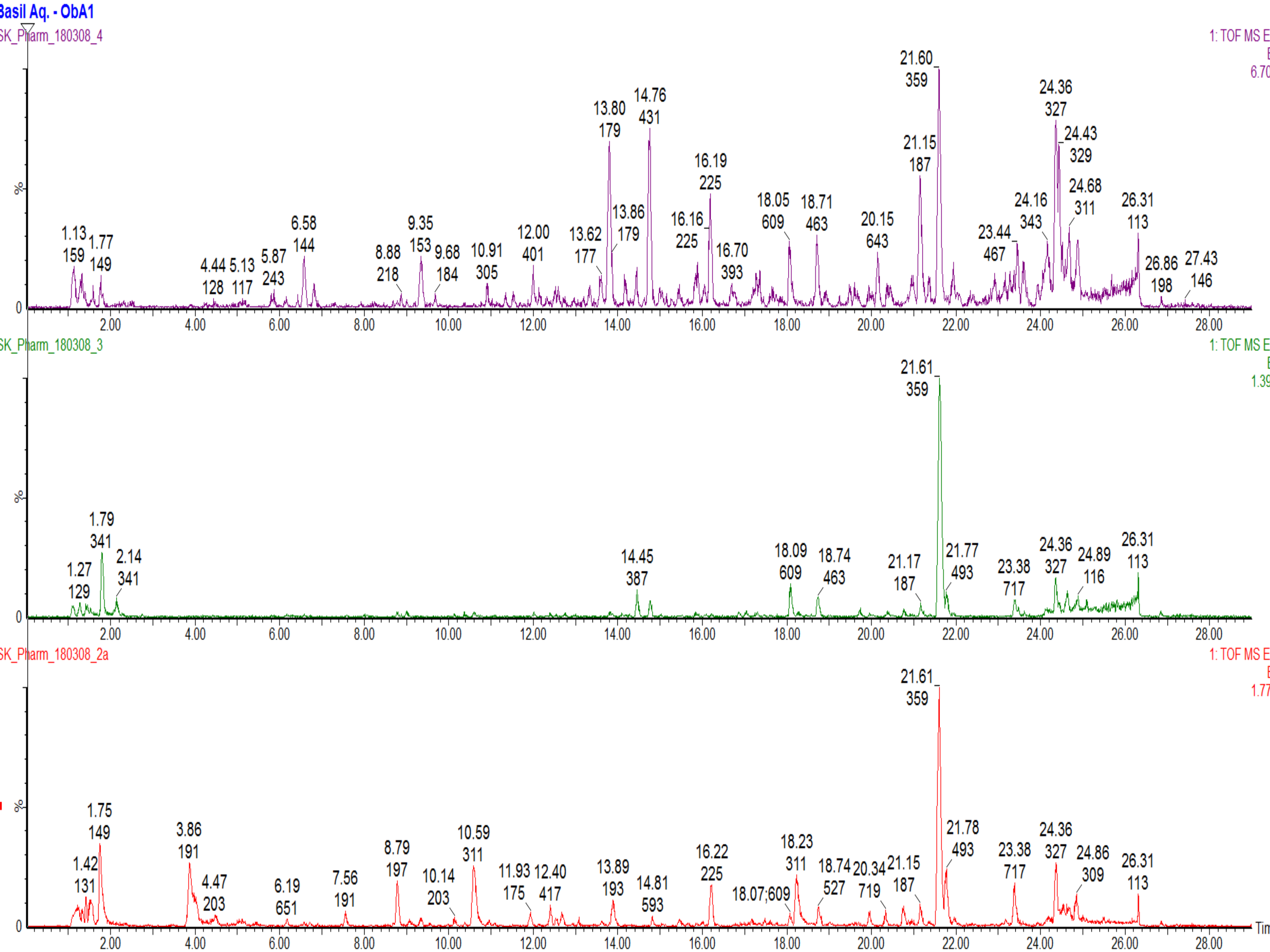
Basil Aq. - ObA1
SK_Pharm_180308_4
1: TOF MS E
6.70
1.13 159
1.77 149
4.44 128
5.13 117
5.87 243
6.58 144
8.88 218
9.35 153
9.68 184
10.91 305
12.00 401
13.62 177
13.80 179
13.86 179
14.76 431
16.16 225
16.19 225
16.70 393
18.05 609
18.71 463
20.15 643
21.15 187
21.60 359
23.44 467
24.16 343
24.36 327
24.43 329
24.68 311
26.31 113
26.86 198
27.43 146
SK_Pharm_180308_3
1: TOF MS E
1.39
1.27 129
1.79 341
2.14 341
14.45 387
18.09 609
18.74 463
21.17 187
21.61 359
21.77 493
23.38 717
24.36 327
24.89 116
26.31 113
SK_Pharm_180308_2a
1: TOF MS E
1.77
1.42 131
1.75 149
3.86 191
4.47 203
6.19 651
7.56 191
8.79 197
10.14 203
10.59 311
11.93 175
12.40 417
13.89 193
14.81 593
16.22 225
18.07;609
18.23 311
18.74 527
20.34 719
21.15 187
21.61 359
21.78 493
23.38 717
24.36 327
24.86 309
26.31 113
%
0
2.00
4.00
6.00
8.00
10.00
12.00
14.00
16.00
18.00
20.00
22.00
24.00
26.00
28.00
Tim

Basil Aq. - ObA1

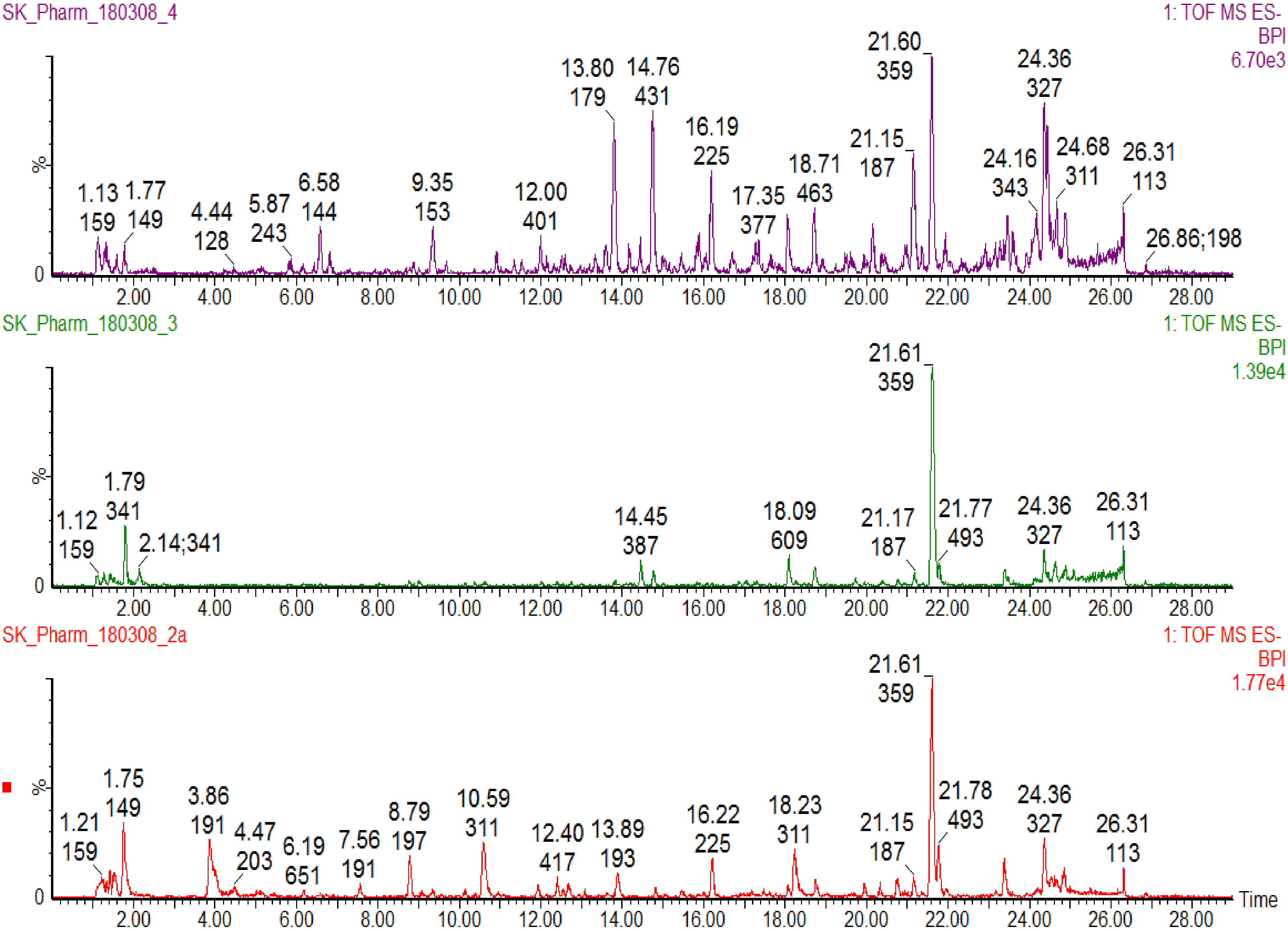

Basil Aq. LC-MS

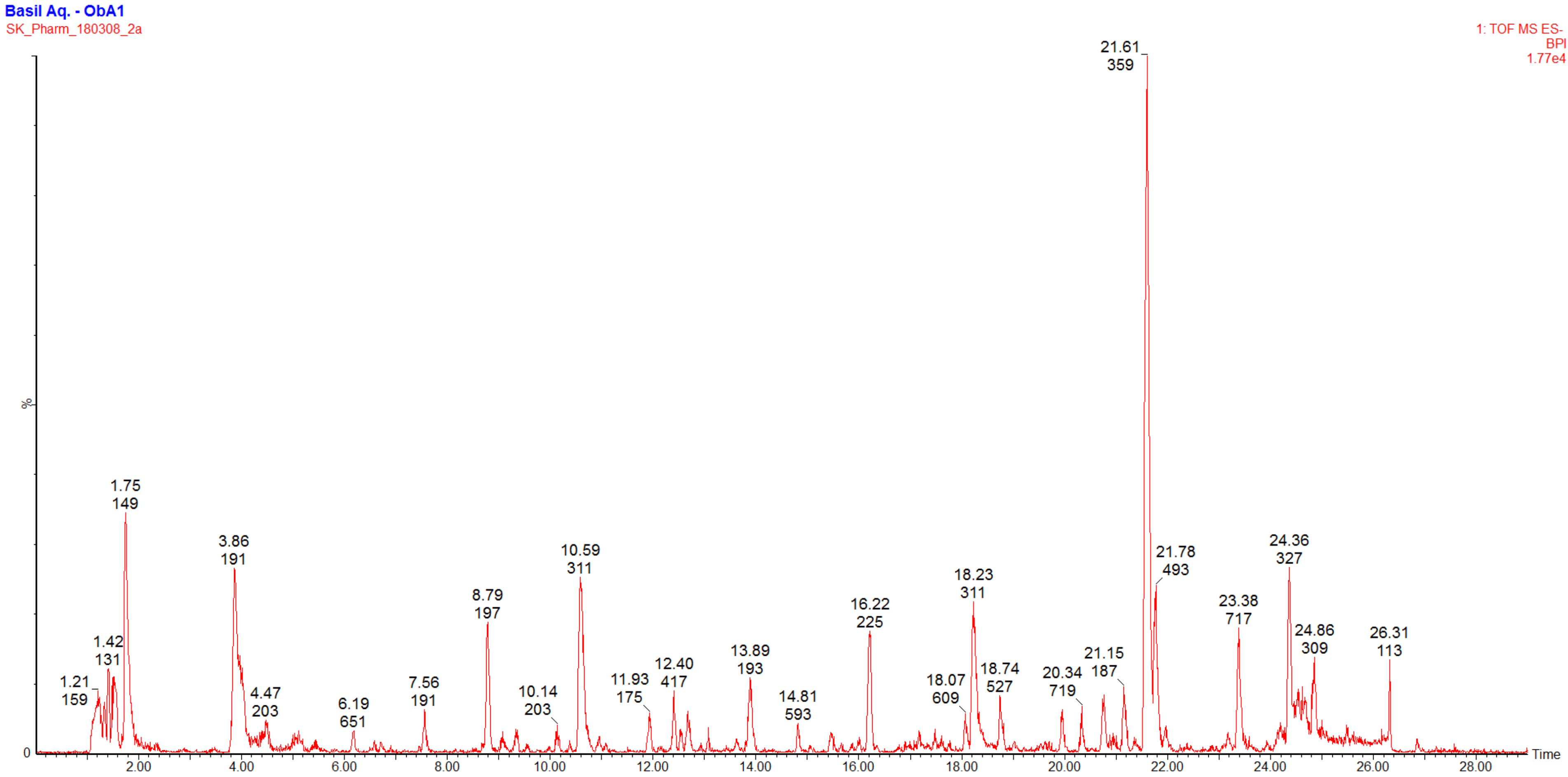

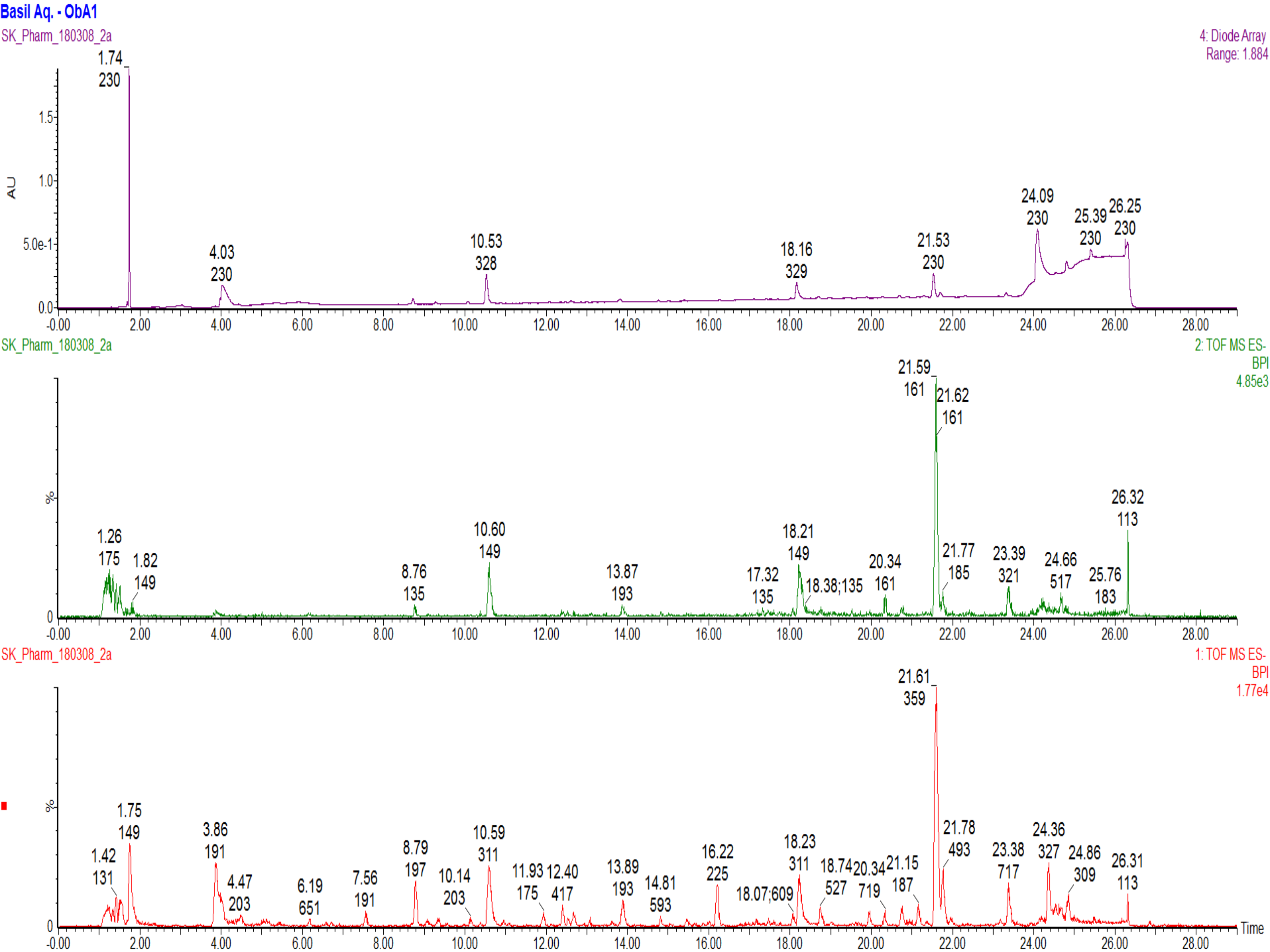
Basil Aq. - ObA1
SK_Pharm_180308_2a
4: Diode Array
Range: 1.884
AU
1.74 230
4.03 230
10.53 328
18.16 329
21.53 230
24.09 230
25.39 230
26.25 230
SK_Pharm_180308_2a
2: TOF MS ES-
BPI
4.85e3
%
1.26 175
1.82 149
8.76 135
10.60 149
13.87 193
17.32 135
18.21 149
18.38;135
20.34 161
21.59 161
21.62 161
21.77 185
23.39 321
24.66 517
25.76 183
26.32 113
SK_Pharm_180308_2a
1: TOF MS ES-
BPI
1.77e4
%
1.42 131
1.75 149
3.86 191
4.47 203
6.19 651
7.56 191
8.79 197
10.14 203
10.59 311
11.93 175
12.40 417
13.89 193
14.81 593
16.22 225
18.07;609
18.23 311
18.74 527
20.34 719
21.15 187
21.61 359
21.78 493
23.38 717
24.36 327
24.86 309
26.31 113
Time

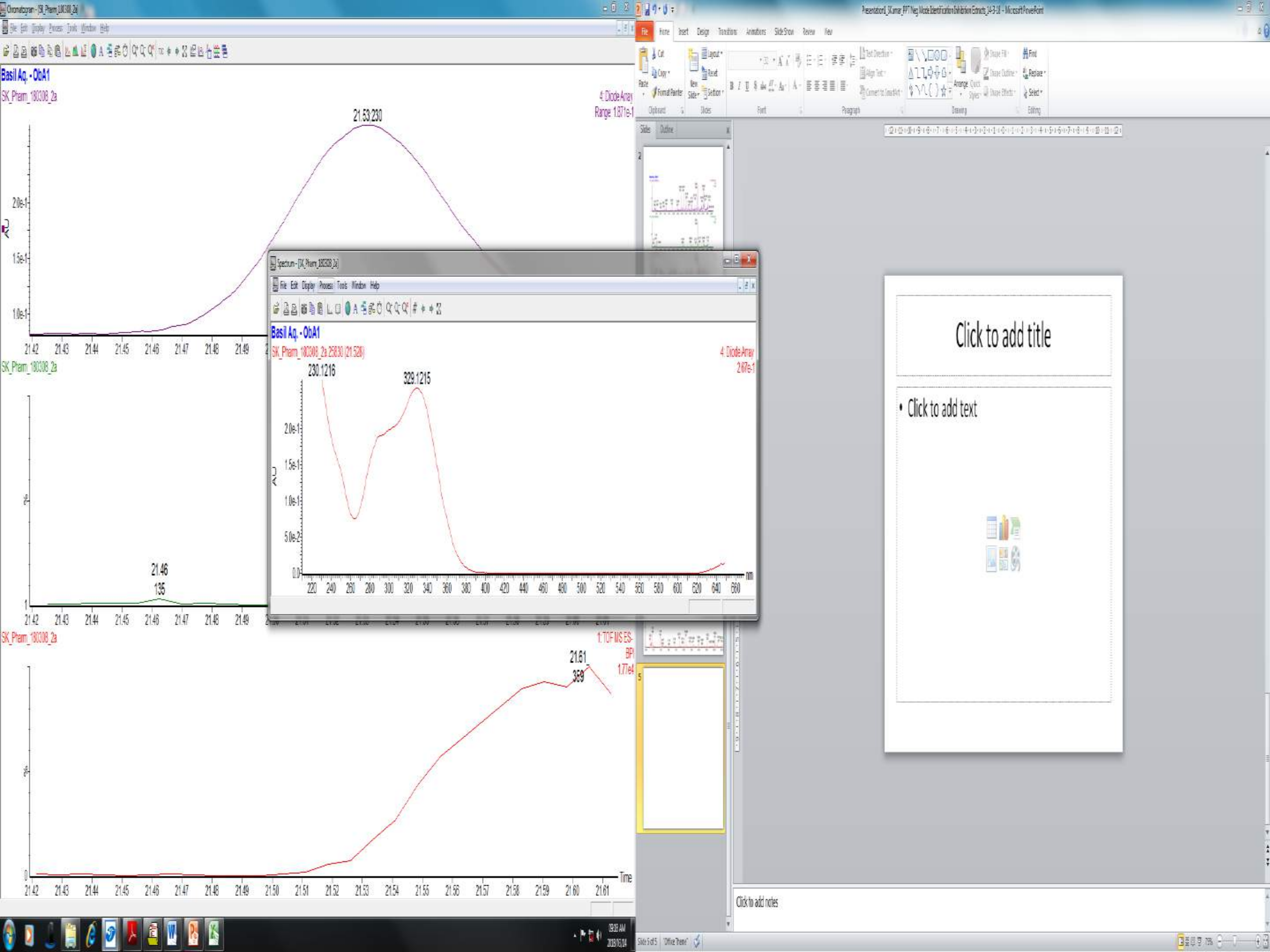
Basil Aq. - ObA1
SK_Pharm_180308_2a
4: Diode Array
21.53;230
21.46
135
1: TOF MS ES-
BPI
1.77e4
21.61
359
Time
230.1216
329.1215
nm
Click to add title
Click to add text
Click to add notes

Basil MeOH - ObM2

SK_Pharm_180308_3

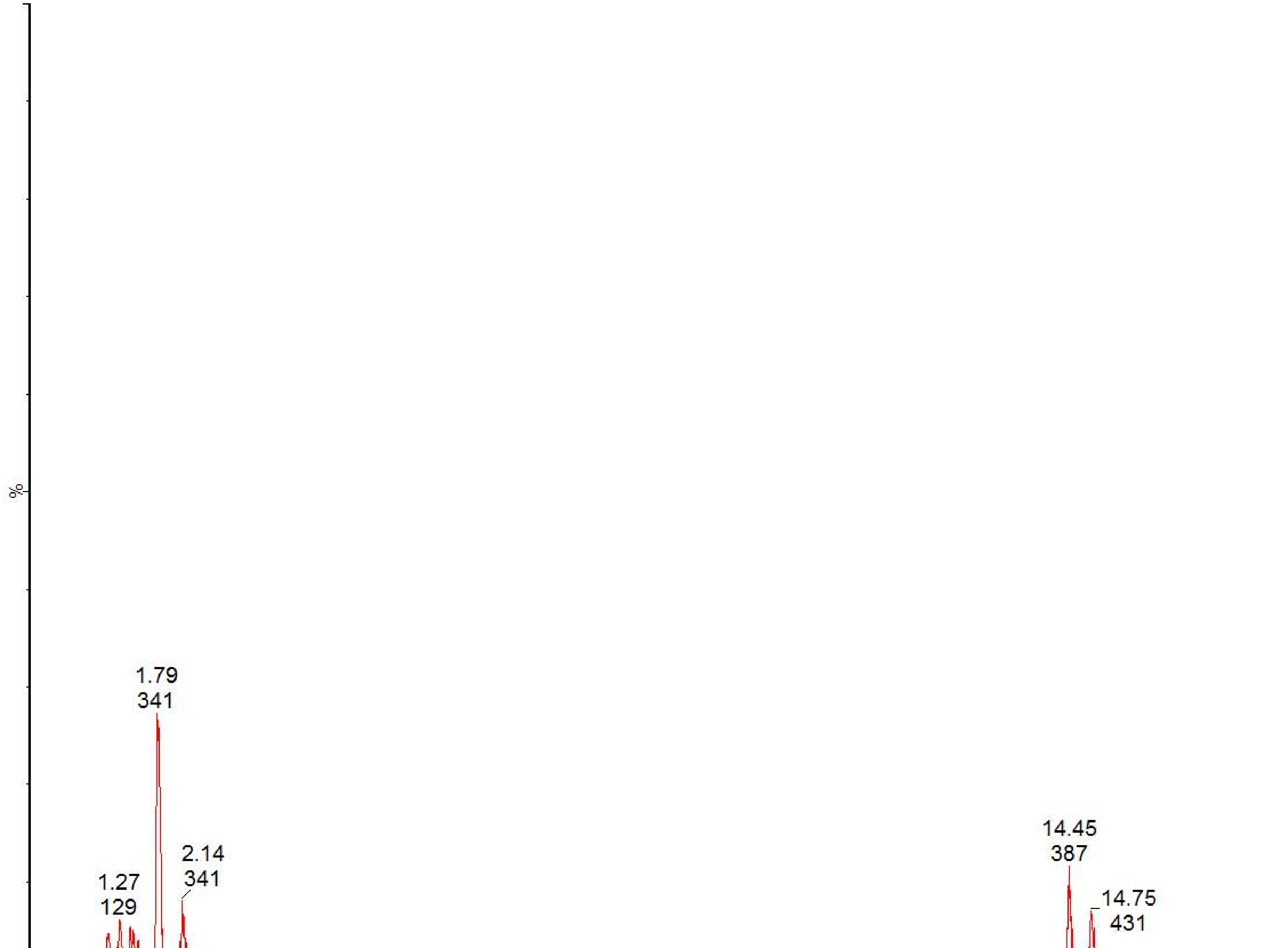

Basil EtOH. LC-MS

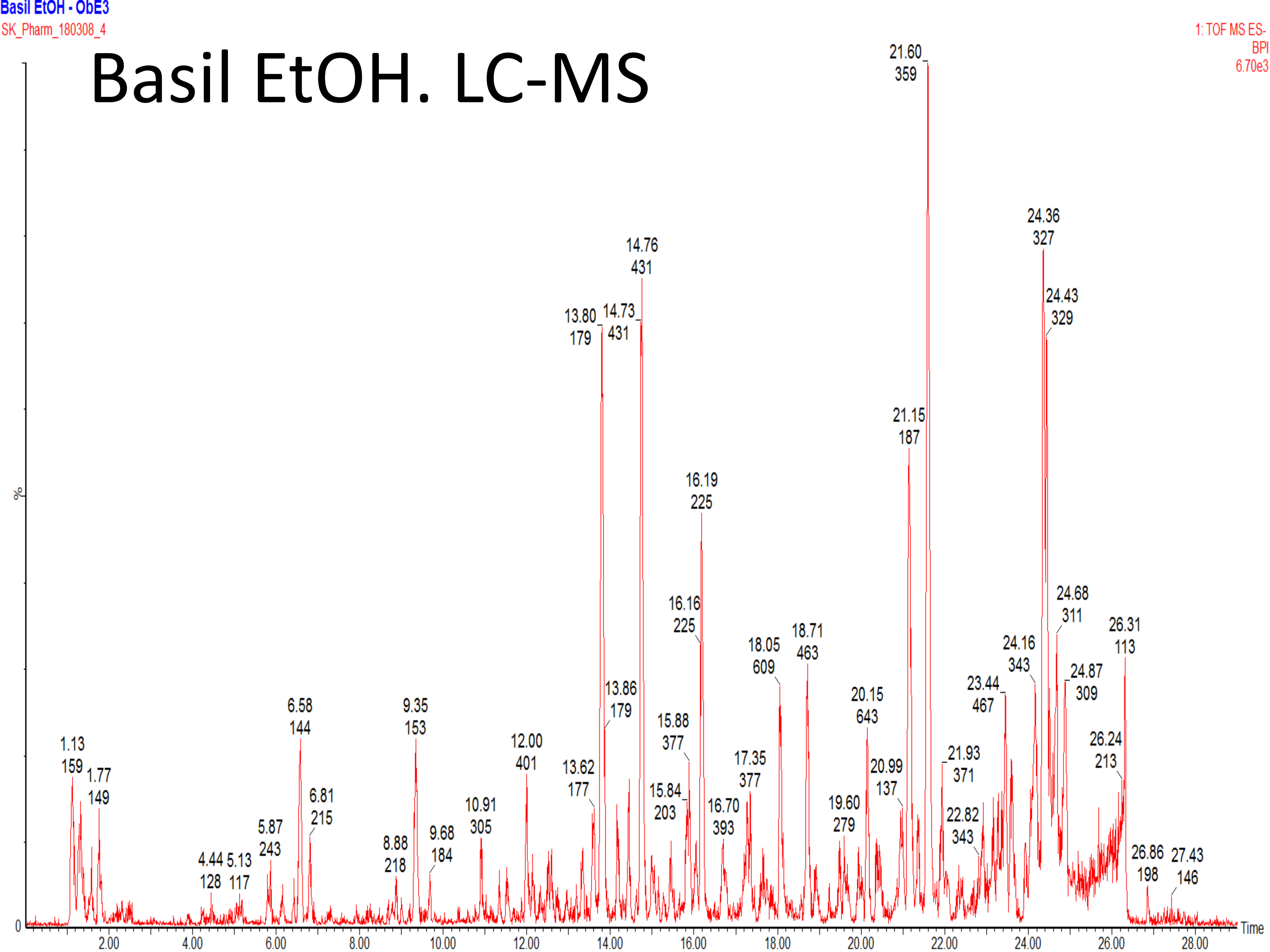

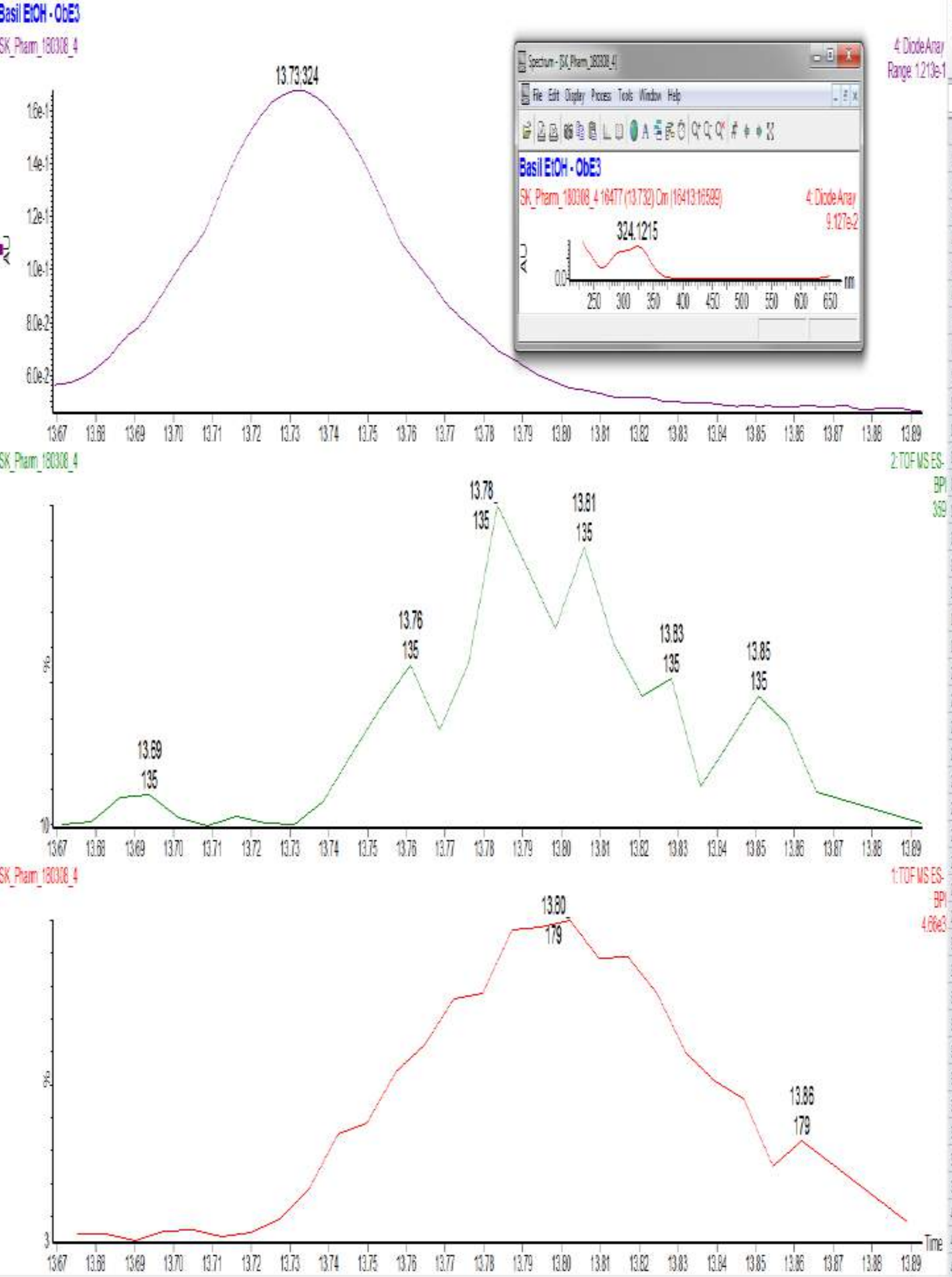

N-Ve Mode

Sample Name	#Rank (Major to minor peaks)	RT (min)	M-H	M-H	UV	Fragment ions (neg)	Tentative ID
ObA1	1	21.65	359.0786	C22H15O3S	285sh, 329	161,133,135,179,197,359	
	OR			C18H15O8			
	2	1.75	149.0076	C4H5O6	230	113, 130.997, 141, 149	
	3	3.86	191.0175	C6H7O7	230	111, 129, 173, 191	
	5	10.59	311.0392	C13H11O9	270sh, 328	131,149, 178, 311	
	OR			C17H11O4S			
	8	16.22	225.111	C12H17O4	230	112, 135, 161, 195,216, 225	
	OR			C9H21O4S			
	6	18.23	311.0392	C13H11O9	270sh, 329	149,179,293,311	
	4	24.36	327.215	C18H31O5	230	116.9, 205, 215, 277, 311, 327	
	7	23.38	717.1448	C36H29O16	267sh, 187	243,343,519,717	
	OR			C40H29O11S			
ObM2	1	21.61	359.0761	C22H15O3S	285sh, 329	161,133,135,179,197,359	
	2	1.79	341.1076	C12H21O11	230	89,173, 341	
	OR			C16H21O6S			
	3	24.36	327.2147	C18H31O5	230	116.9, 205, 215, 277, 311, 327	
	4	18.09	609.1499	C27H29O16	230	151,271,300,515,609	
	OR			C24H33O16S			
	5	14.45	387.1648	C18H27O9	230	59,119,207,300,387	
	OR			C22H27O4S			
	6	18.74	463.0882	C21H19O12	230	89,151,255,271,300	
	7	23.38	717.145	C36H29O16	267sh, 187	243,343,519,717	
ObE3	1	21.6	359.0758	C22H15O3S	285sh, 329	161,133,135,179,197,359	
				C18H15O8			
	2	24.36	327.2155	C18H31O5	230	116.9, 205, 215, 277, 311, 327	
	3	14.76	431.1907	C24H31O5S	230	153,205,354,385,431	
	OR			C20H31O10			
	4	14.73	179.033	C9H7O4	262sh, 324	107,135,179	

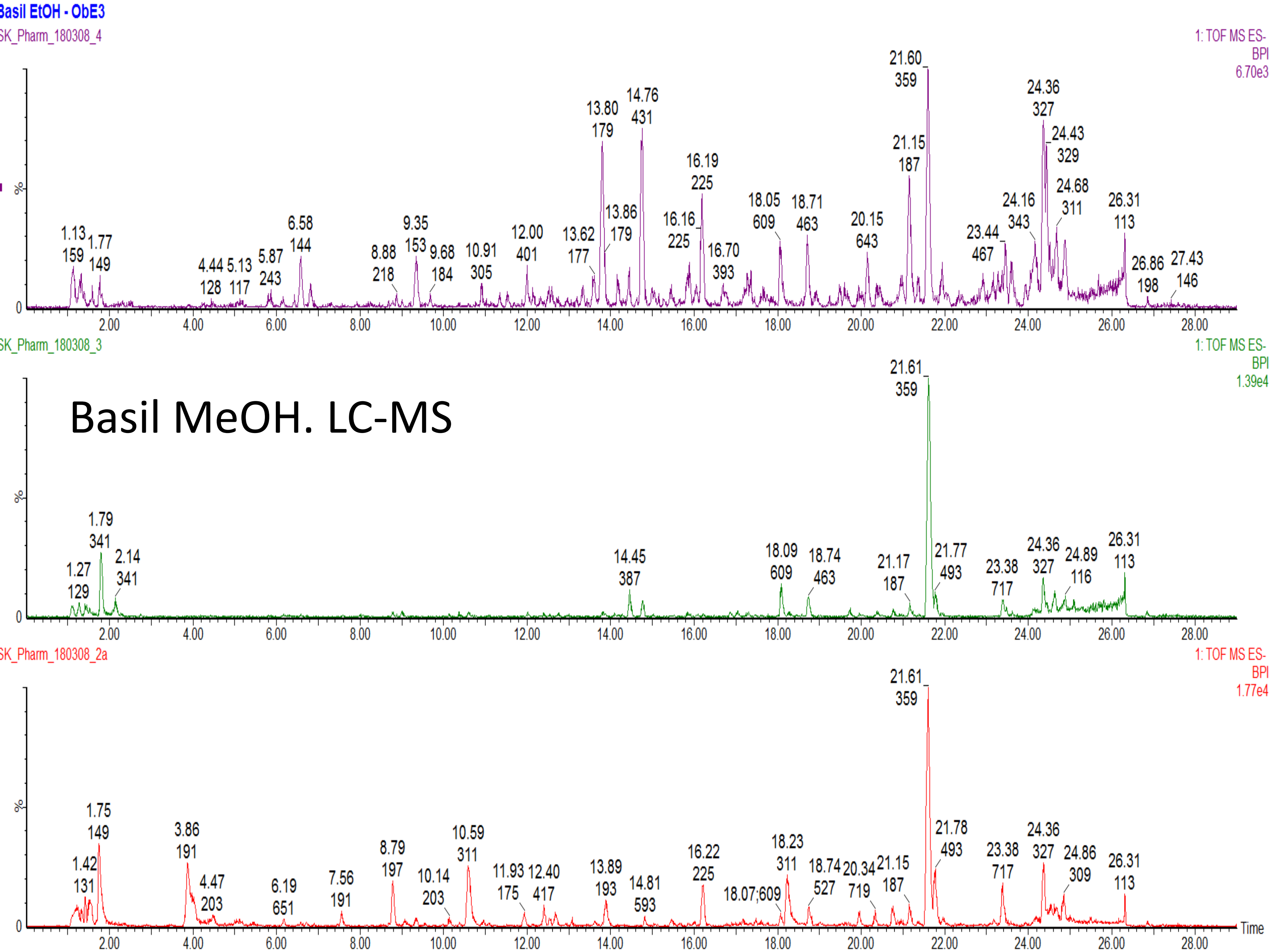

Basil EtOH - ObE3
SK_Pharm_180308_4
1: TOF MS ES-
BPI
6.70e3
1.13 159
1.77 149
4.44 128
5.13 117
5.87 243
6.58 144
8.88 218
9.35 153
9.68 184
10.91 305
12.00 401
13.62 177
13.80 179
13.86 179
14.76 431
16.16 225
16.19 225
16.70 393
18.05 609
18.71 463
20.15 643
21.15 187
21.60 359
23.44 467
24.16 343
24.36 327
24.43 329
24.68 311
26.31 113
26.86 198
27.43 146
SK_Pharm_180308_3
1: TOF MS ES-
BPI
1.39e4
Basil MeOH. LC-MS
1.27 129
1.79 341
2.14 341
14.45 387
18.09 609
18.74 463
21.17 187
21.61 359
21.77 493
23.38 717
24.36 327
24.89 116
26.31 113
SK_Pharm_180308_2a
1: TOF MS ES-
BPI
1.77e4
1.42 131
1.75 149
3.86 191
4.47 203
6.19 651
7.56 191
8.79 197
10.14 203
10.59 311
11.93 175
12.40 417
13.89 193
14.81 593
16.22 225
18.07;609
18.23 311
18.74 527
20.34 719
21.15 187
21.61 359
21.78 493
23.38 717
24.36 327
24.86 309
26.31 113
%
0
2.00
4.00
6.00
8.00
10.00
12.00
14.00
16.00
18.00
20.00
22.00
24.00
26.00
28.00
Time

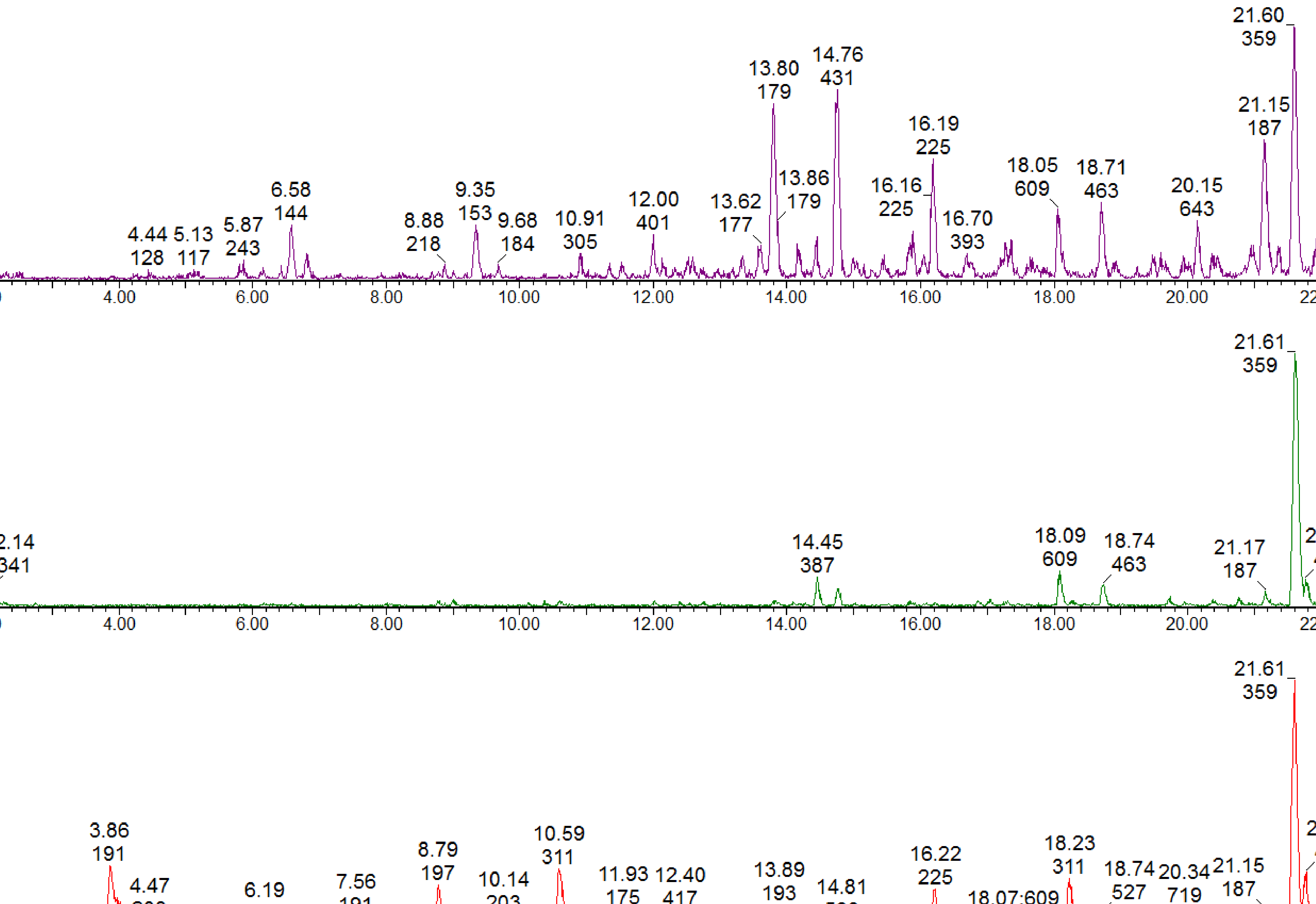
21.60 359
21.15 187
14.76 431
13.80 179
13.86 179
16.19 225
16.16 225
18.05 609
18.71 463
20.15 643
16.70 393
13.62 177
12.00 401
10.91 305
9.68 184
9.35 153
8.88 218
6.58 144
5.87 243
5.13 117
4.44 128
4.00
6.00
8.00
10.00
12.00
14.00
16.00
18.00
20.00
21.61 359
14.45 387
18.09 609
18.74 463
21.17 187
4.00
6.00
8.00
10.00
12.00
14.00
16.00
18.00
20.00
21.61 359
3.86 191
4.47
6.19
7.56
8.79 197
10.14
10.59 311
11.93
12.40
13.89
14.81
16.22 225
18.23 311
18.74
20.34
21.15

Basil Aq. - ObA1

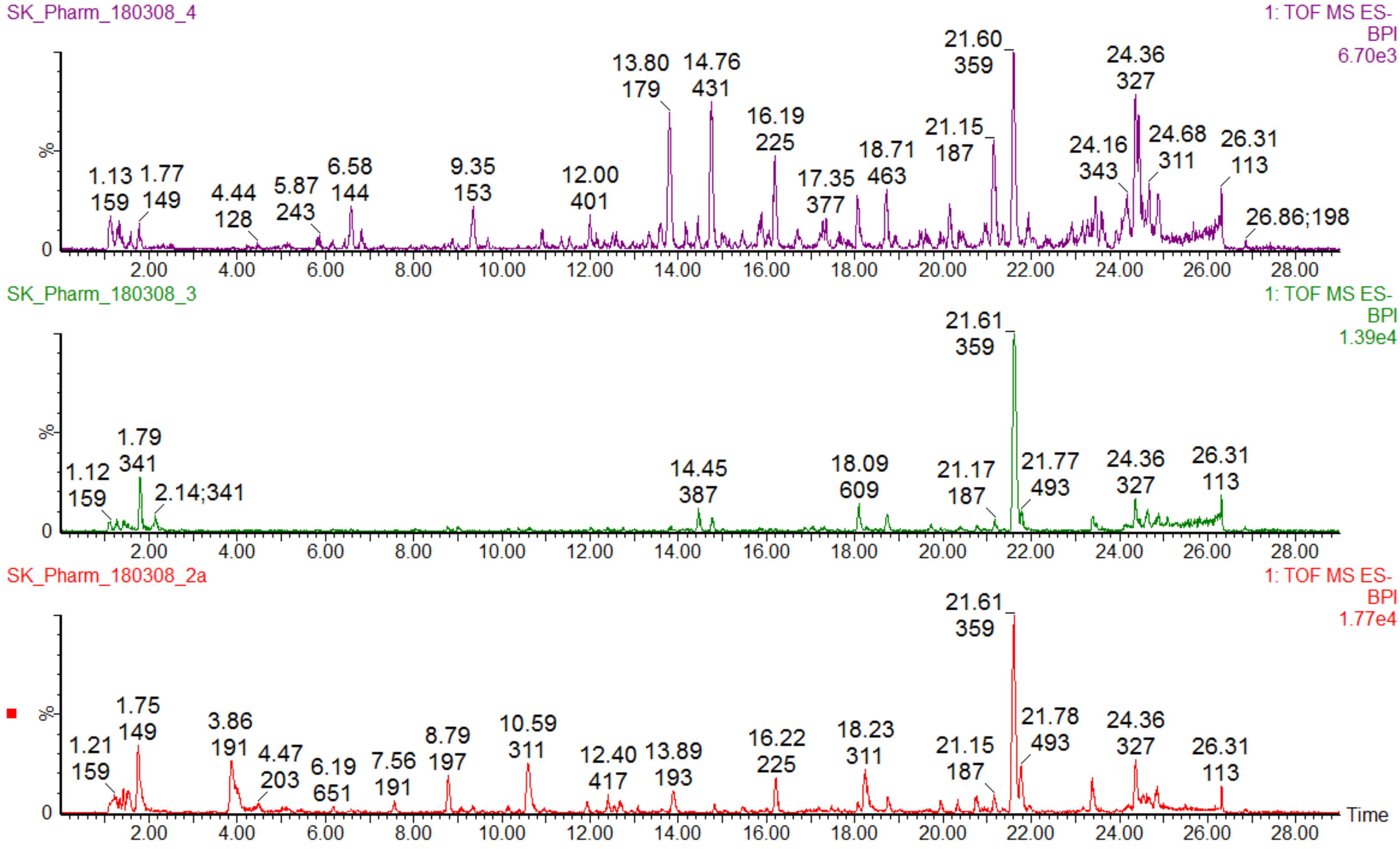

ObA1

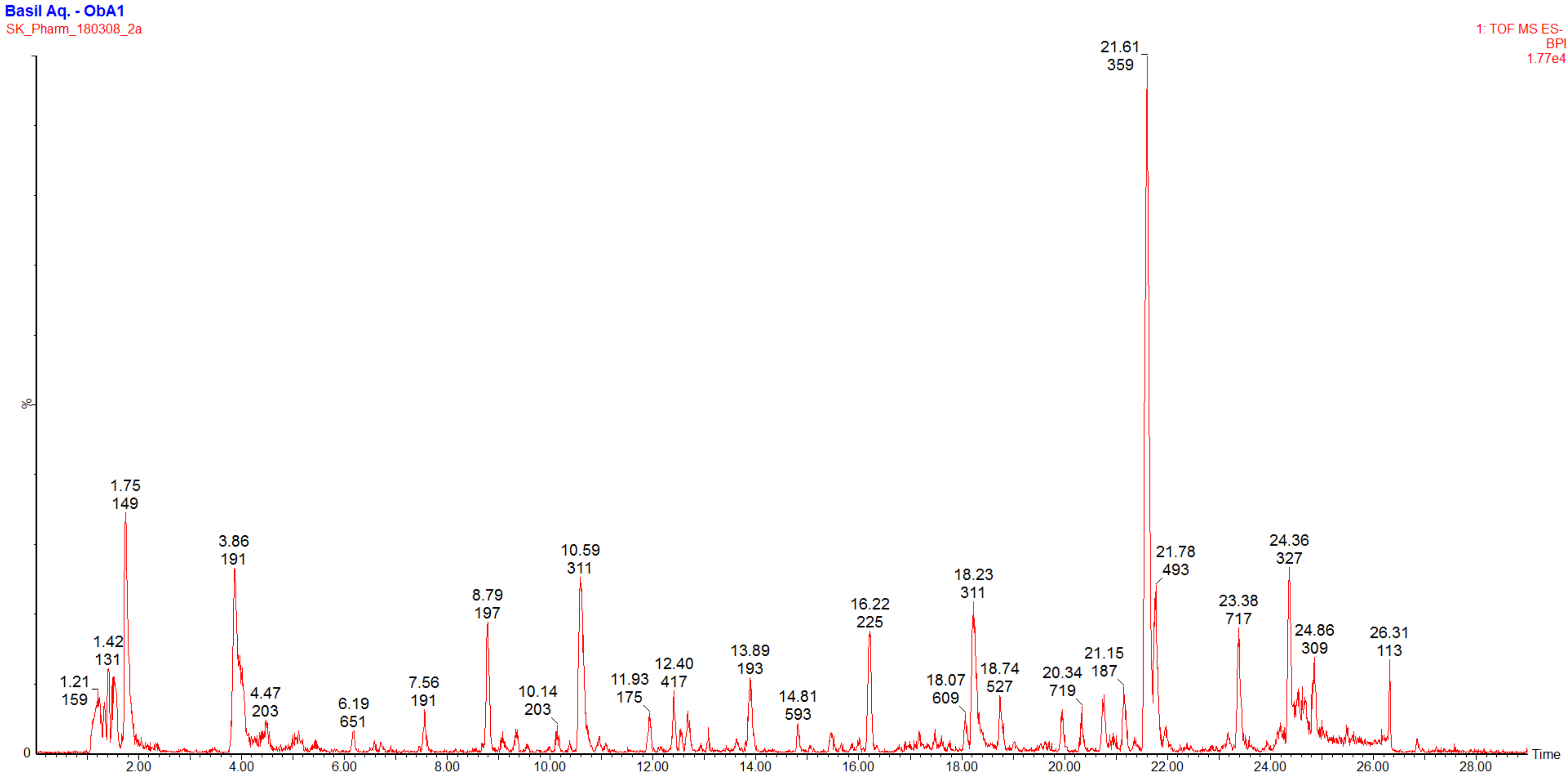
Basil Aq. - ObA1
SK_Pharm_180308_2a
1: TOF MS ES-
BPI
1.77e4
%
0
Time
21.61
359
1.75
149
3.86
191
10.59
311
21.78
493
24.36
327
18.23
311
23.38
717
8.79
197
16.22
225
24.86
309
26.31
113
1.42
131
13.89
193
21.15
187
12.40
417
18.74
527
1.21
159
11.93
175
18.07
609
20.34
719
4.47
203
7.56
191
10.14
203
14.81
593
6.19
651
2.00
4.00
6.00
8.00
10.00
12.00
14.00
16.00
18.00
20.00
22.00
24.00
26.00
28.00

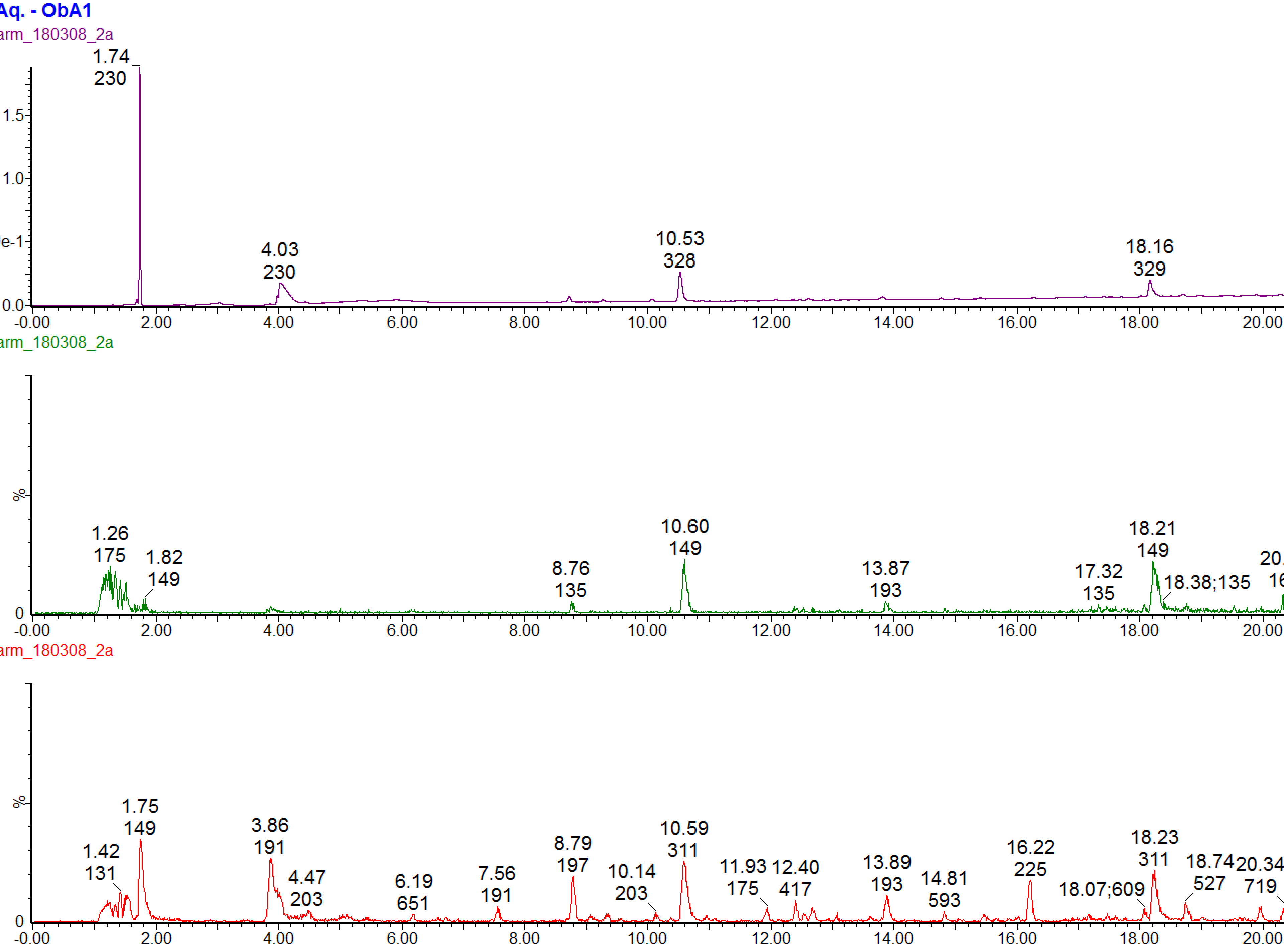

Aq. - ObA1
arm_180308_2a
1.74
230
4.03
230
10.53
328
18.16
329
1.5
1.0
e-1
0.0
-0.00
2.00
4.00
6.00
8.00
10.00
12.00
14.00
16.00
18.00
20.00
arm_180308_2a
%
1.26
175
1.82
149
8.76
135
10.60
149
13.87
193
17.32
135
18.21
149
18.38;135
0
arm_180308_2a
%
1.42
131
1.75
149
3.86
191
4.47
203
6.19
651
7.56
191
8.79
197
10.14
203
10.59
311
11.93
175
12.40
417
13.89
193
14.81
593
16.22
225
18.07;609
18.23
311
18.74
527
20.34
719
0

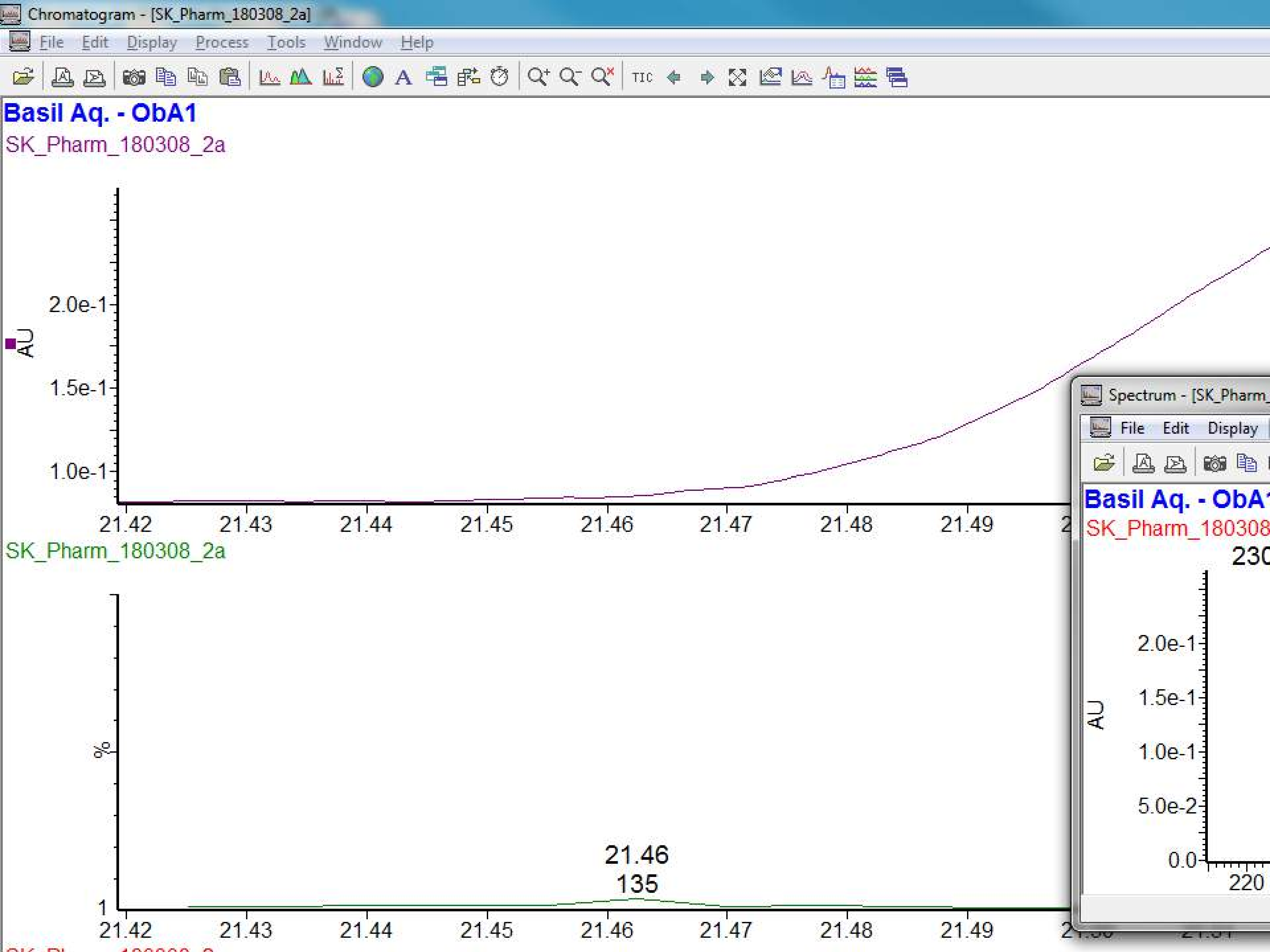
Chromatogram - [SK_Pharm_180308_2a]
File Edit Display Process Tools Window Help
TIC
Basil Aq. - ObA1
SK_Pharm_180308_2a
AU
2.0e-1
1.5e-1
1.0e-1
21.42 21.43 21.44 21.45 21.46 21.47 21.48 21.49
SK_Pharm_180308_2a
%
21.46
135
1
21.42 21.43 21.44 21.45 21.46 21.47 21.48 21.49
Spectrum - [SK_Pharm_
File Edit Display
Basil Aq. - ObA1
SK_Pharm_180308
AU
2.0e-1
1.5e-1
1.0e-1
5.0e-2
0.0
220

Basil MeOH - ObM2

SK_Pharm_180308_3

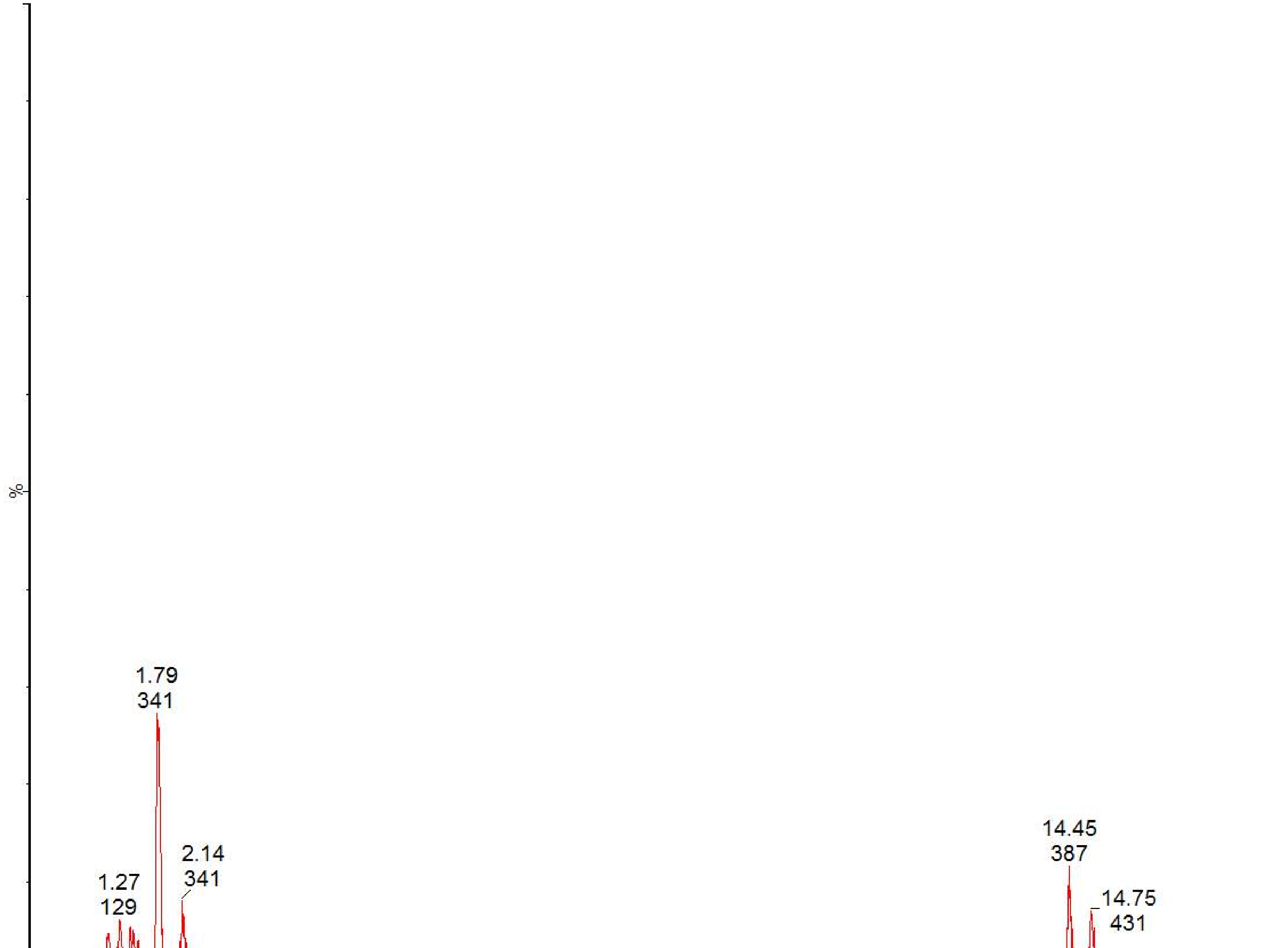

Basil EtOH - ObE3

SK_Pharm_180308_4

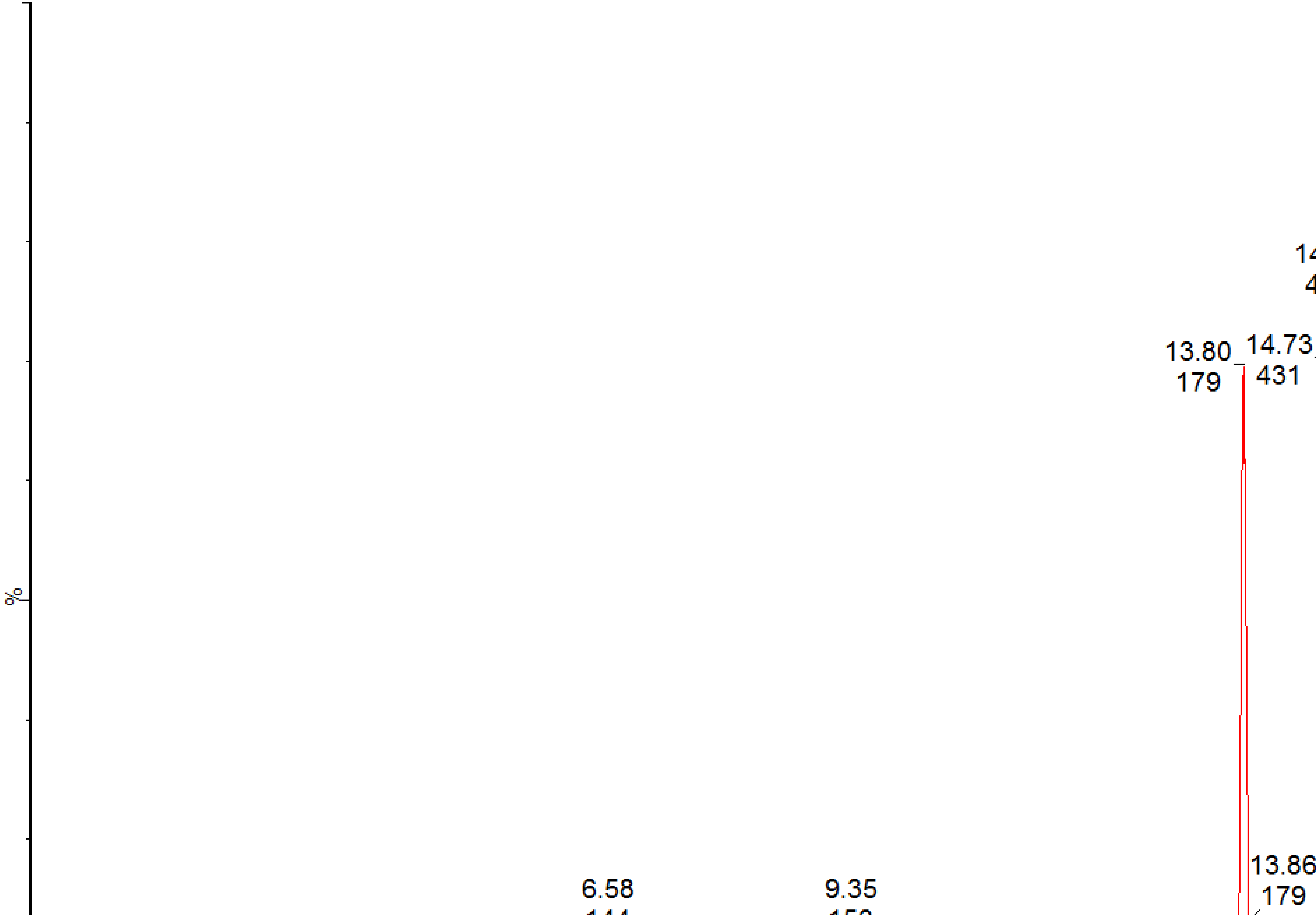

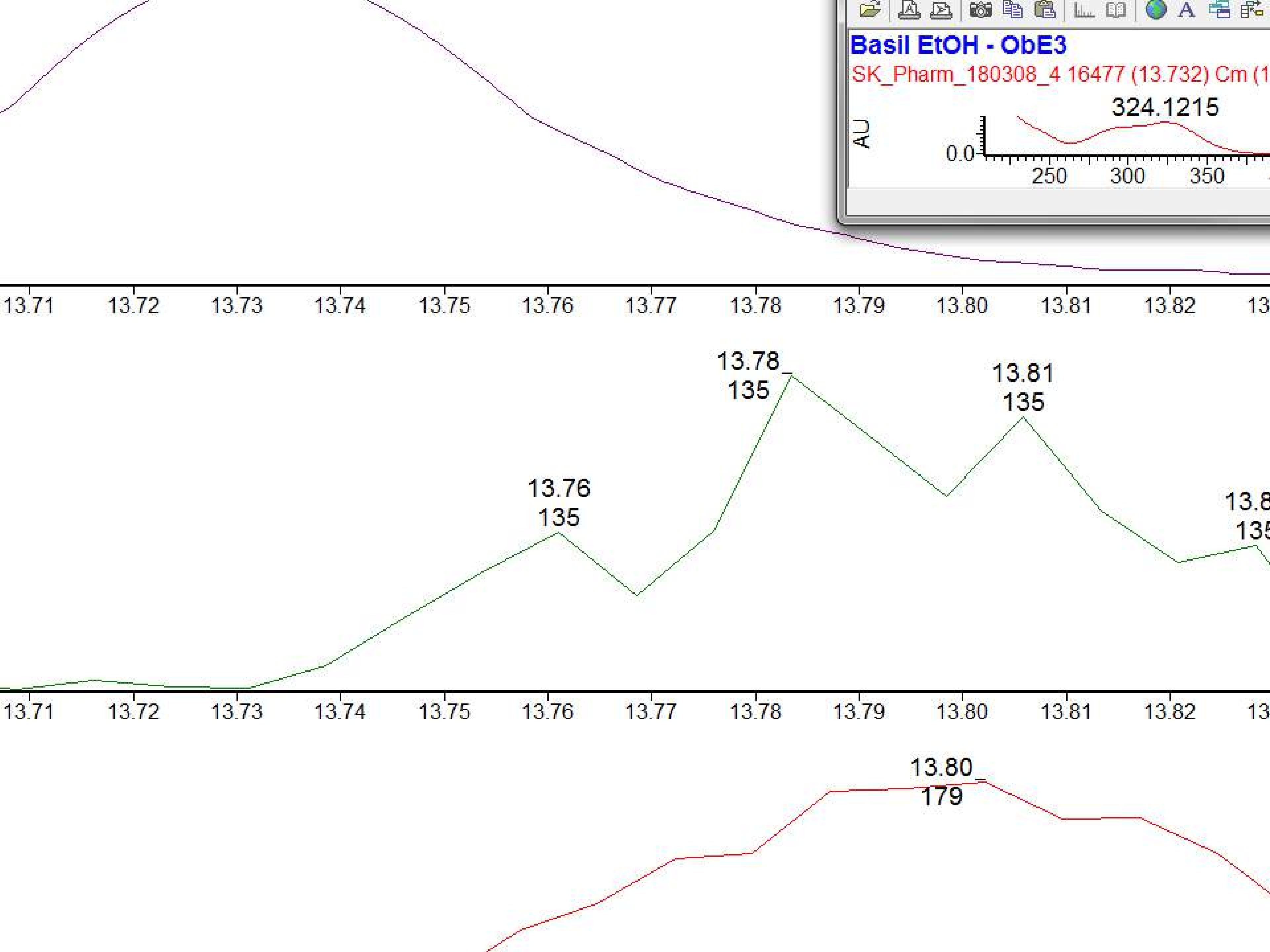

Basil EtOH - ObE3
SK_Pharm_180308_4 16477 (13.732) Cm (1
324.1215
AU
0.0
250
300
350
13.71
13.72
13.73
13.74
13.75
13.76
13.77
13.78
13.79
13.80
13.81
13.82
13.78_
135
13.81
135
13.76
135
13.8
135
13.80_
179

Basil EtOH - ObE3

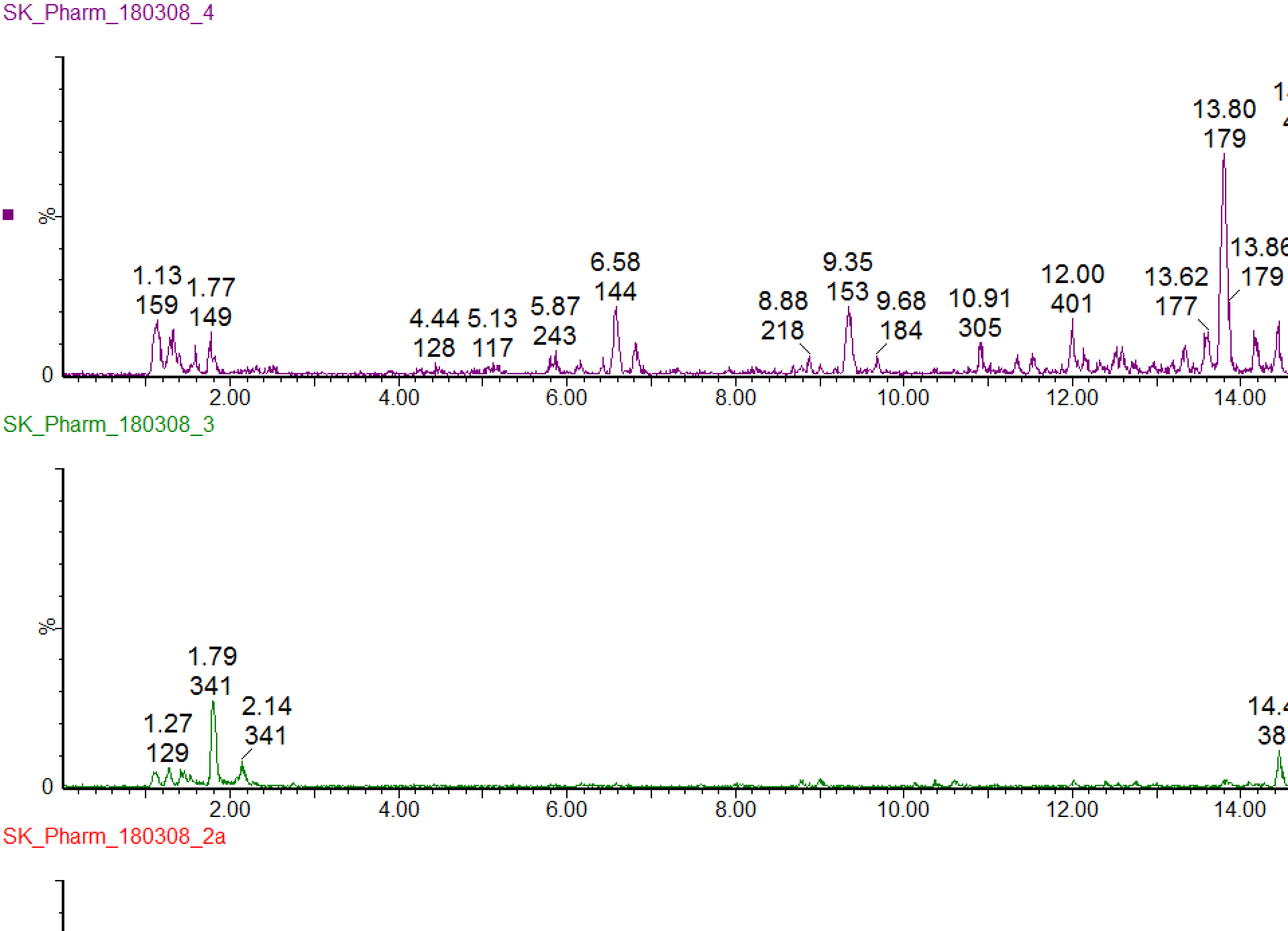

Reported by User: Saneesh Kumar (Saneesh)   Project Name: HDI_Stellenbosch_2016

SAMPLE INFORMATION

Sample Name:	RIF Control Incubate 30min	Acquired By:	Saneesh
Sample Type:	Unknown	Date Acquired:	2016/05/31 10:51:37 AM
Vial:	24	Acq. Method Set:	HDI_RIF_25ODESRIF_NEO
Injection #:	1	Date Processed:	2016/05/31 05:05:27 PM
Injection Volume:	15.00 ul	Processing Method:	HDI_RIF_ W2996 254.0nm-1.2
Run Time:	11.5 Minutes	Channel Name:	W2996 254.0nm-1.2
Sample Set Name:	RIF_Basil Incubats 31_5_16	Proc. Chnl. Descr.:	W2996 PDA 254.0 nm at 1.2

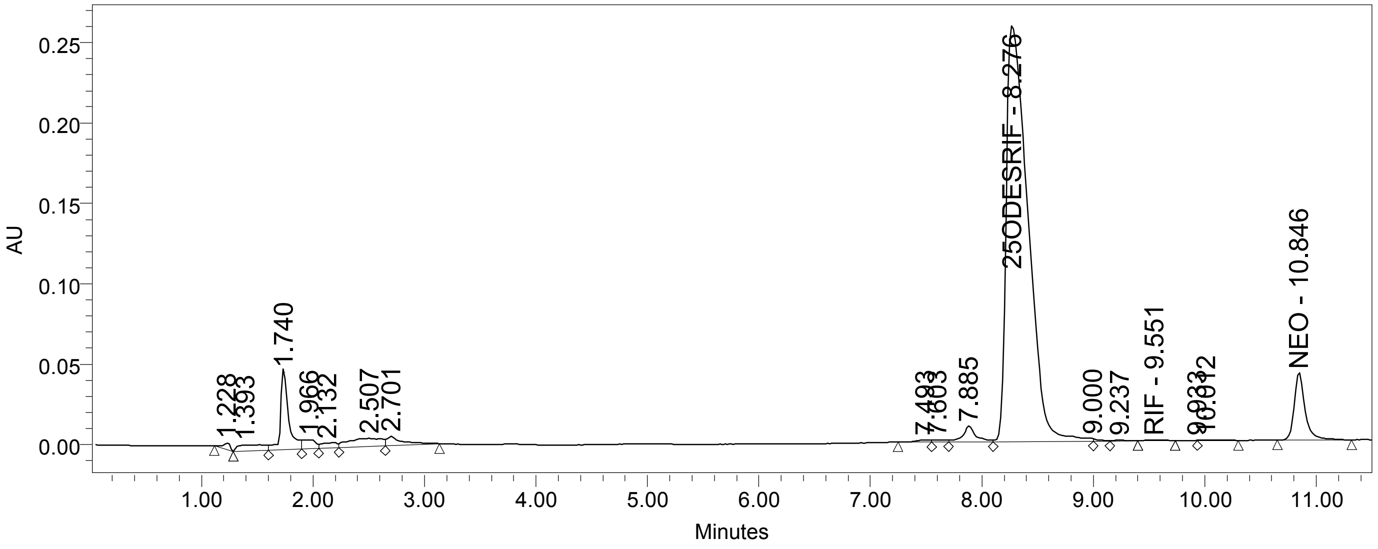

	Peak Name	RT	Area	% Area	Height
1		1.228	19179	0.43	4243
2		1.393	65674	1.46	3880
3		1.740	254086	5.65	48947
4		1.966	44383	0.99	5662
5		2.132	32687	0.73	3241
6		2.507	106919	2.38	4967
7		2.701	56890	1.27	5543
8		7.493	11087	0.25	1340
9		7.603	10825	0.24	1332
10		7.885	79205	1.76	9582
11	25ODESRIF	8.276	3507537	78.06	260931
12		9.000	5595	0.12	1497
13		9.237	5057	0.11	740
14	RIF	9.551	7261	0.16	702

Reported by User: Saneesh Kumar (Saneesh) Project Name: HDI_Stellenbosch_2016

	Peak Name	RT	Area	% Area	Height
15		9.933	1107	0.02	191
16		10.012	5353	0.12	418
17	NEO	10.846	280310	6.24	41721

S A M P L E   I N F O R M A T I O N

Sample Name:	RIF Inc Basil_H2O 10miG/mL 1	Acquired By:	Saneesh
Sample Type:	Unknown	Date Acquired:	2016/05/31 11:07:33 AM
Vial:	2	Acq. Method Set:	HDI_RIF_25ODESRIF_NEO
Injection #:	1	Date Processed:	2016/05/31 05:05:27 PM
Injection Volume:	15.00 ul	Processing Method:	HDI_RIF_ W2996 254.0nm-1.2
Run Time:	11.5 Minutes	Channel Name:	W2996 254.0nm-1.2
Sample Set Name:	RIF_Basil Incubats 31_5_16	Proc. Chnl. Descr.:	W2996 PDA 254.0 nm at 1.2

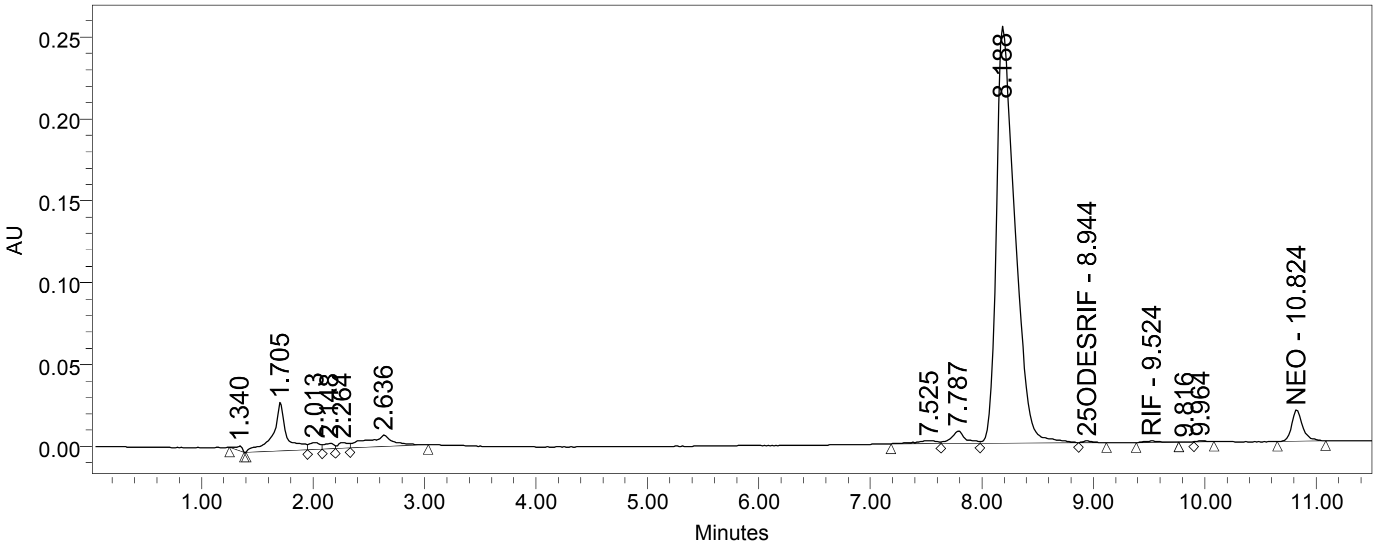

	Peak Name	RT	Area	% Area	Height
1		1.340	12257	0.34	3164
2		1.705	249232	6.89	28734
3		2.013	27875	0.77	4098
4		2.148	19854	0.55	3179
5		2.264	21964	0.61	3420
6		2.636	118894	3.29	6840
7		7.525	23039	0.64	1616
8		7.787	64846	1.79	7525
9		8.188	2930547	81.03	255634
10	25ODESRIF	8.944	6812	0.19	1127
11	RIF	9.524	8297	0.23	816
12		9.816	739	0.02	143

Reported by User: Saneesh Kumar (Saneesh) Project Name: HDI_Stellenbosch_2016

	Peak Name	RT	Area	% Area	Height
13		9.964	2802	0.08	482
14	NEO	10.824	129569	3.58	18963

Reported by User: Saneesh Kumar (Saneesh)   Project Name: HDI_Stellenbosch_2016

SAMPLE INFORMATION

Sample Name:	RIF Inc Basil_H2O 10miG/mL 2	Acquired By:	Saneesh
Sample Type:	Unknown	Date Acquired:	2016/05/31 11:22:49 AM
Vial:	3	Acq. Method Set:	HDI_RIF_25ODESRIF_NEO
Injection #:	1	Date Processed:	2016/05/31 05:05:27 PM
Injection Volume:	15.00 ul	Processing Method:	HDI_RIF_ W2996 254.0nm-1.2
Run Time:	11.5 Minutes	Channel Name:	W2996 254.0nm-1.2
Sample Set Name:	RIF_Basil Incubats 31_5_16	Proc. Chnl. Descr.:	W2996 PDA 254.0 nm at 1.2

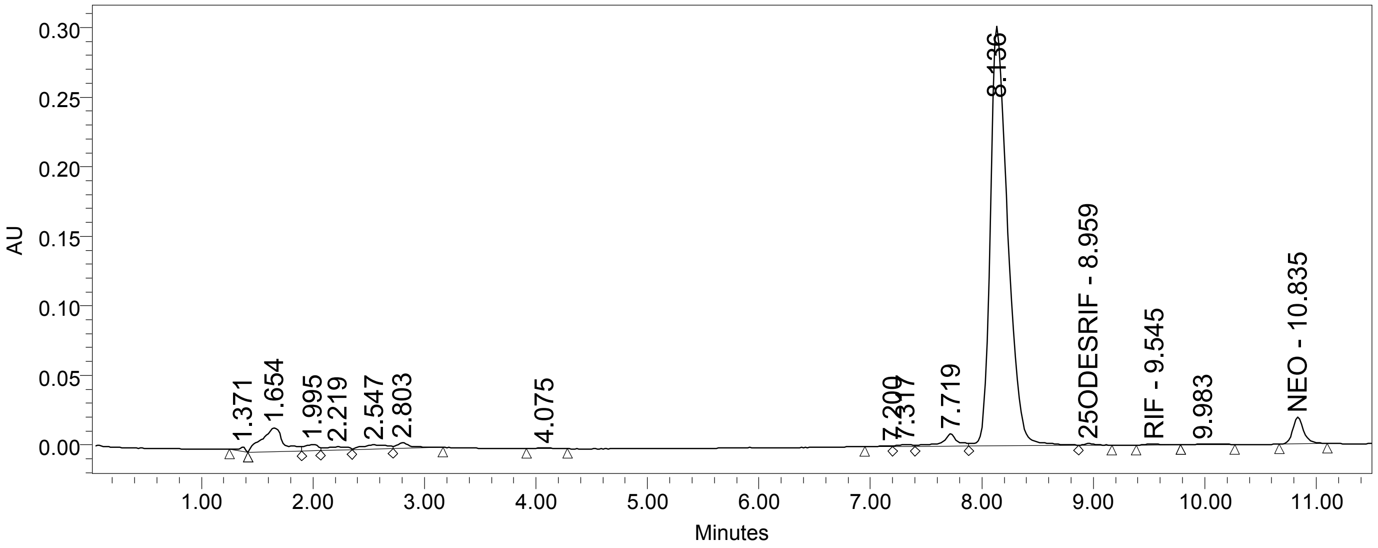

	Peak Name	RT	Area	% Area	Height
1		1.371	13017	0.33	2987
2		1.654	231661	5.94	17021
3		1.995	36360	0.93	4304
4		2.219	33139	0.85	2330
5		2.547	50530	1.30	3008
6		2.803	37604	0.96	4142
7		4.075	8475	0.22	923
8		7.200	3655	0.09	544
9		7.317	11863	0.30	1298
10		7.719	84324	2.16	8712
11		8.136	3231903	82.93	301604
12	25ODESRIF	8.959	8142	0.21	1338

	Peak Name	RT	Area	% Area	Height
13	RIF	9.545	8650	0.22	833
14		9.983	7406	0.19	542
15	NEO	10.835	130611	3.35	19151

Reported by User: Saneesh Kumar (Saneesh) Project Name: HDI_Stellenbosch_2016

SAMPLE INFORMATION

Sample Name:	RIF Inc Basil_MeOH 10miG/mL 1	Acquired By:	Saneesh
Sample Type:	Unknown	Date Acquired:	2016/05/31 11:38:05 AM
Vial:	4	Acq. Method Set:	HDI_RIF_25ODESRIF_NEO
Injection #:	1	Date Processed:	2016/05/31 05:05:27 PM
Injection Volume:	15.00 ul	Processing Method:	HDI_RIF_ W2996 254.0nm-1.2
Run Time:	11.5 Minutes	Channel Name:	W2996 254.0nm-1.2
Sample Set Name:	RIF_Basil Incubats 31_5_16	Proc. Chnl. Descr.:	W2996 PDA 254.0 nm at 1.2

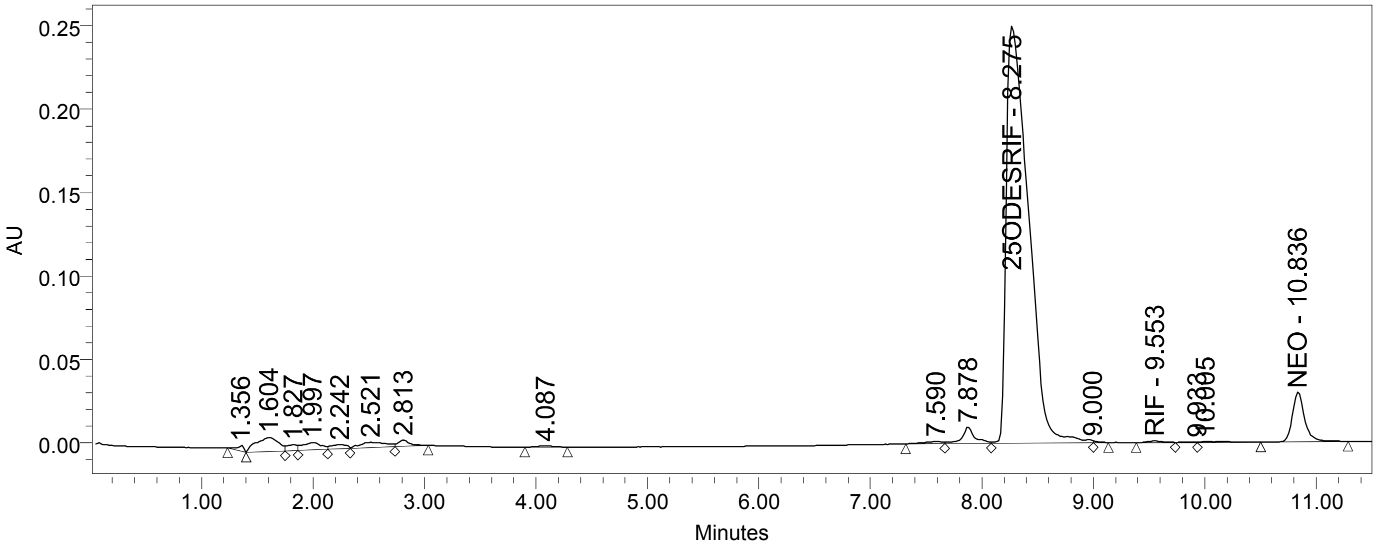

	Peak Name	RT	Area	% Area	Height
1		1.356	14950	0.36	3345
2		1.604	116907	2.85	8296
3		1.827	22628	0.55	3361
4		1.997	51104	1.24	4347
5		2.242	24360	0.59	2390
6		2.521	52044	1.27	3165
7		2.813	26566	0.65	3752
8		4.087	9526	0.23	943
9		7.590	13426	0.33	1136
10		7.878	75222	1.83	9783
11	25ODESRIF	8.275	3455930	84.12	251347
12		9.000	2113	0.05	854

Reported by User: Saneesh Kumar (Saneesh) Project Name: HDI_Stellenbosch_2016

	Peak Name	RT	Area	% Area	Height
13	RIF	9.553	8963	0.22	899
14		9.933	1816	0.04	266
15		10.005	8167	0.20	474
16	NEO	10.836	224659	5.47	29717

SAMPLE INFORMATION

Sample Name:	RIF Inc Basil_MeOH 10miG/mL 2	Acquired By:	Saneesh
Sample Type:	Unknown	Date Acquired:	2016/05/31 11:53:12 AM
Vial:	5	Acq. Method Set:	HDI_RIF_25ODESRIF_NEO
Injection #:	1	Date Processed:	2016/05/31 05:05:27 PM
Injection Volume:	15.00 ul	Processing Method:	HDI_RIF_ W2996 254.0nm-1.2
Run Time:	11.5 Minutes	Channel Name:	W2996 254.0nm-1.2
Sample Set Name:	RIF_Basil Incubats 31_5_16	Proc. Chnl. Descr.:	W2996 PDA 254.0 nm at 1.2

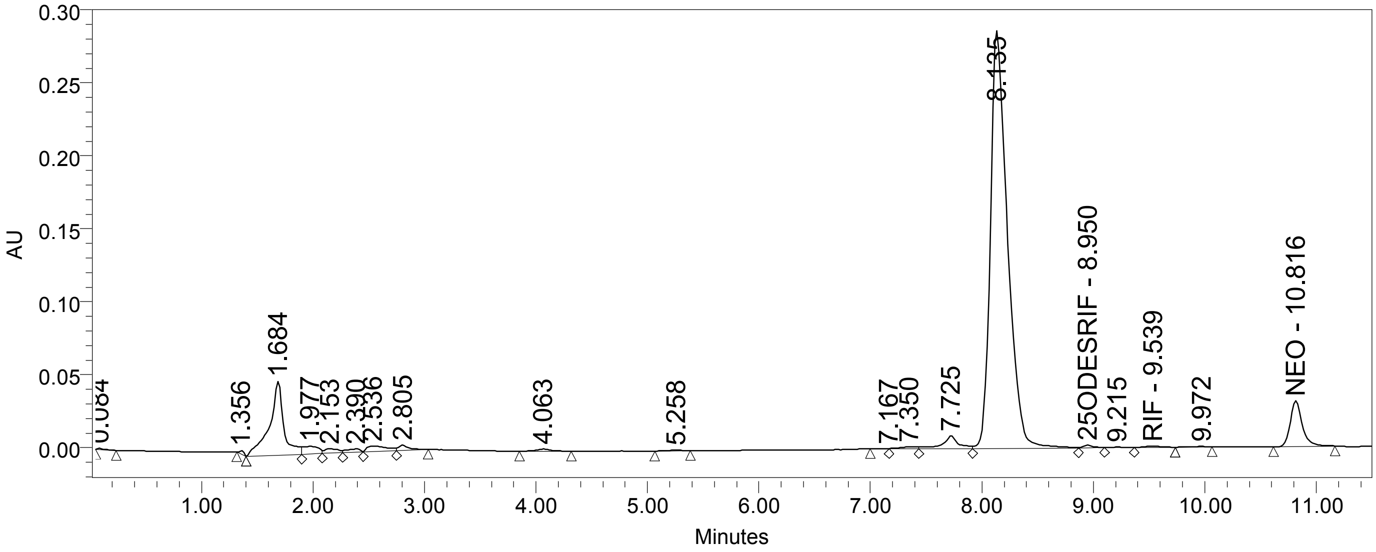

	Peak Name	RT	Area	% Area	Height
1		0.084	3262	0.08	879
2		1.356	7431	0.18	2495
3		1.684	455603	11.31	50251
4		1.977	49751	1.23	5197
5		2.153	26266	0.65	3043
6		2.390	19960	0.50	2459
7		2.536	47659	1.18	3827
8		2.805	23450	0.58	3635
9		4.063	16334	0.41	1603
10		5.258	5550	0.14	817
11		7.167	2024	0.05	379
12		7.350	15217	0.38	1357

Reported by User: Saneesh Kumar (Saneesh) Project Name: HDI_Stellenbosch_2016

	Peak Name	RT	Area	% Area	Height
13		7.725	86989	2.16	8586
14		8.135	3006597	74.60	286113
15	25ODESRIF	8.950	10676	0.26	1716
16		9.215	2918	0.07	347
17	RIF	9.539	10867	0.27	1054
18		9.972	4246	0.11	430
19	NEO	10.816	235272	5.84	31265

Reported by User: Saneesh Kumar (Saneesh) Project Name: HDI_Stellenbosch_2016

SAMPLE INFORMATION

Sample Name:	RIF Inc Basil_EOH 10miG/mL 1	Acquired By:	Saneesh
Sample Type:	Unknown	Date Acquired:	2016/05/31 12:08:19 PM
Vial:	6	Acq. Method Set:	HDI_RIF_25ODESRIF_NEO
Injection #:	1	Date Processed:	2016/05/31 05:05:27 PM
Injection Volume:	15.00 ul	Processing Method:	HDI_RIF_ W2996 254.0nm-1.2
Run Time:	11.5 Minutes	Channel Name:	W2996 254.0nm-1.2
Sample Set Name:	RIF_Basil Incubats 31_5_16	Proc. Chnl. Descr.:	W2996 PDA 254.0 nm at 1.2

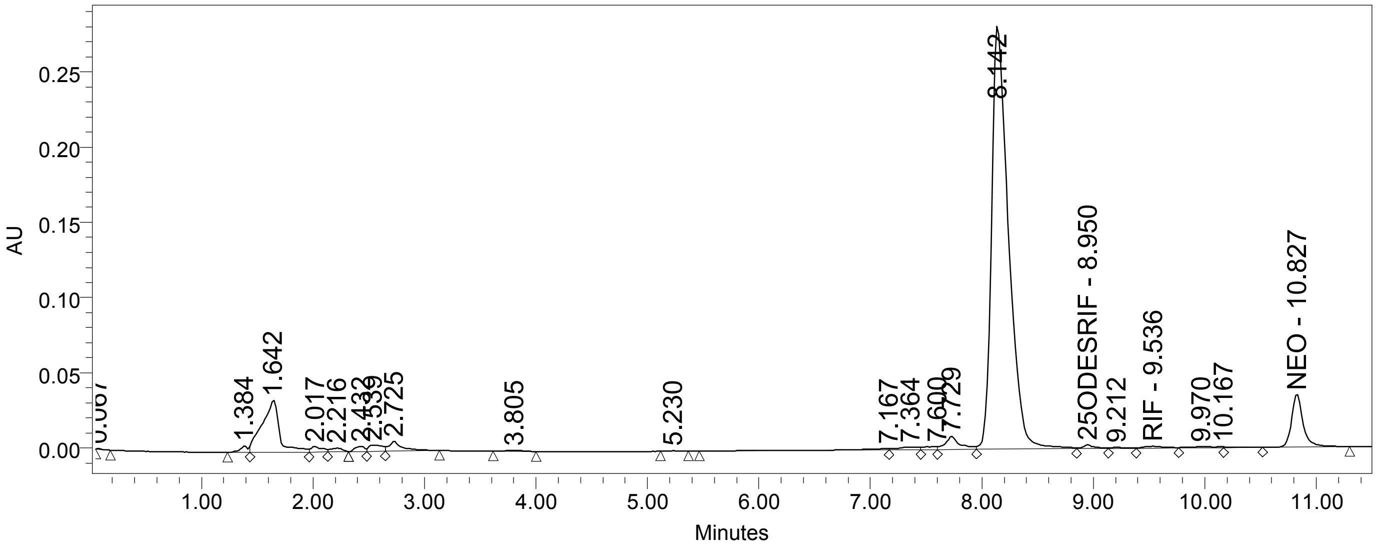

	Peak Name	RT	Area	% Area	Height
1		0.067	1786	0.05	667
2		1.384	20736	0.52	4094
3		1.642	373350	9.41	34445
4		2.017	25492	0.64	3621
5		2.216	19351	0.49	2501
6		2.432	21865	0.55	3468
7		2.539	37019	0.93	4339
8		2.725	57682	1.45	6501
9		3.805	7982	0.20	658
10		5.230	1780	0.04	309
11		7.167	22754	0.57	844
12		7.364	23737	0.60	1751

Reported by User: Saneesh Kumar (Saneesh) Project Name: HDI_Stellenbosch_2016

	Peak Name	RT	Area	% Area	Height
13		7.600	16043	0.40	1875
14		7.729	80184	2.02	8565
15		8.142	2942266	74.20	283359
16	25ODESRIF	8.950	18171	0.46	2350
17		9.212	7097	0.18	656
18	RIF	9.536	16317	0.41	1305
19		9.970	13674	0.34	975
20		10.167	3802	0.10	493
21	NEO	10.827	254419	6.42	34977

Multi Sample Summary

Reported by User: Saneesh Kumar (Saneesh)   Project Name: HDI_Stellenbosch_2016

SAMPLE INFORMATION

Sample Name:	RIF Inc Basil_EOH 10miG/mL 2	Acquired By:	Saneesh
Sample Type:	Unknown	Date Acquired:	2016/05/31 12:23:26 PM
Vial:	7	Acq. Method Set:	HDI_RIF_25ODESRIF_NEO
Injection #:	1	Date Processed:	2016/05/31 05:05:27 PM
Injection Volume:	15.00 ul	Processing Method:	HDI_RIF_ W2996 254.0nm-1.2
Run Time:	11.5 Minutes	Channel Name:	W2996 254.0nm-1.2
Sample Set Name:	RIF_Basil Incubats 31_5_16	Proc. Chnl. Descr.:	W2996 PDA 254.0 nm at 1.2

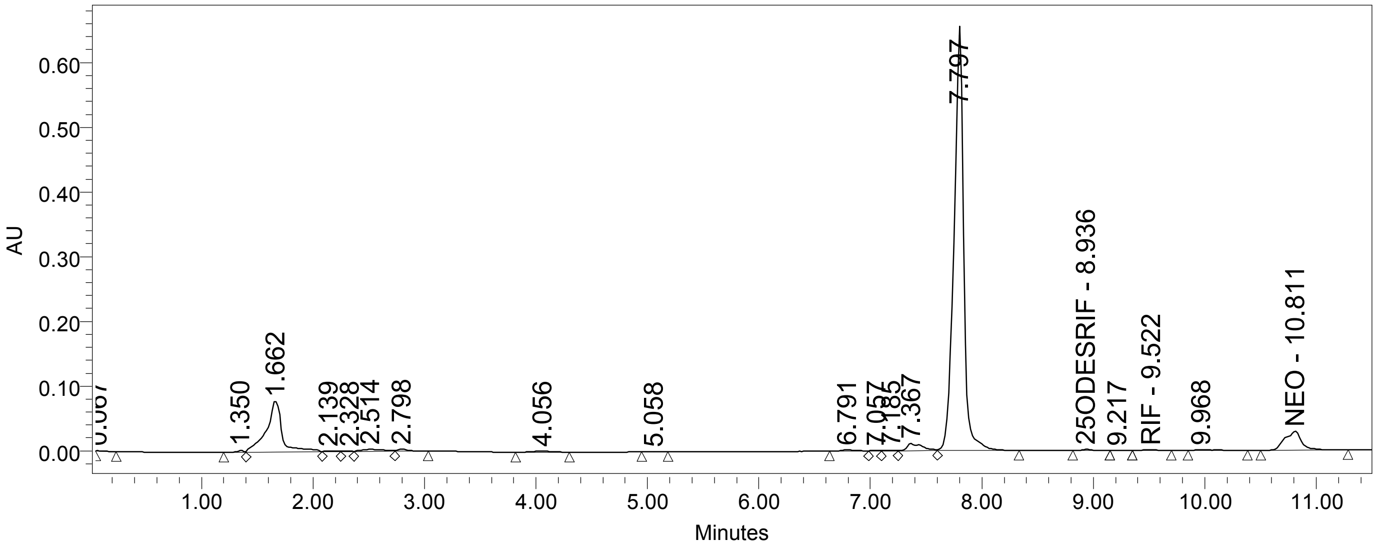

	Peak Name	RT	Area	% Area	Height
1		0.067	1859	0.04	583
2		1.350	11142	0.21	2752
3		1.662	775454	14.71	78113
4		2.139	9088	0.17	1171
5		2.328	5199	0.10	1039
6		2.514	48205	0.91	3213
7		2.798	21616	0.41	3481
8		4.056	16175	0.31	1604
9		5.058	2450	0.05	409
10		6.791	17000	0.32	2456
11		7.057	4692	0.09	861
12		7.185	7673	0.15	1002

Reported by User: Saneesh Kumar (Saneesh) Project Name: HDI_Stellenbosch_2016

	Peak Name	RT	Area	% Area	Height
13		7.367	112143	2.13	11277
14		7.797	3905003	74.10	644878
15	25ODESRIF	8.936	9646	0.18	1776
16		9.217	1898	0.04	257
17	RIF	9.522	7865	0.15	868
18		9.968	7328	0.14	479
19	NEO	10.811	305399	5.80	29554

Multi Sample Summary

Reported by User: Saneesh Kumar (Saneesh)   Project Name: HDI_Stellenbosch_2016

SAMPLE INFORMATION

Sample Name:	RIF Inc Basil_EtoAc 10miG/mL 1	Acquired By:	Saneesh
Sample Type:	Unknown	Date Acquired:	2016/05/31 12:38:42 PM
Vial:	8	Acq. Method Set:	HDI_RIF_25ODESRIF_NEO
Injection #:	1	Date Processed:	2016/05/31 05:05:27 PM
Injection Volume:	15.00 ul	Processing Method:	HDI_RIF_ W2996 254.0nm-1.2
Run Time:	11.5 Minutes	Channel Name:	W2996 254.0nm-1.2
Sample Set Name:	RIF_Basil Incubats 31_5_16	Proc. Chnl. Descr.:	W2996 PDA 254.0 nm at 1.2

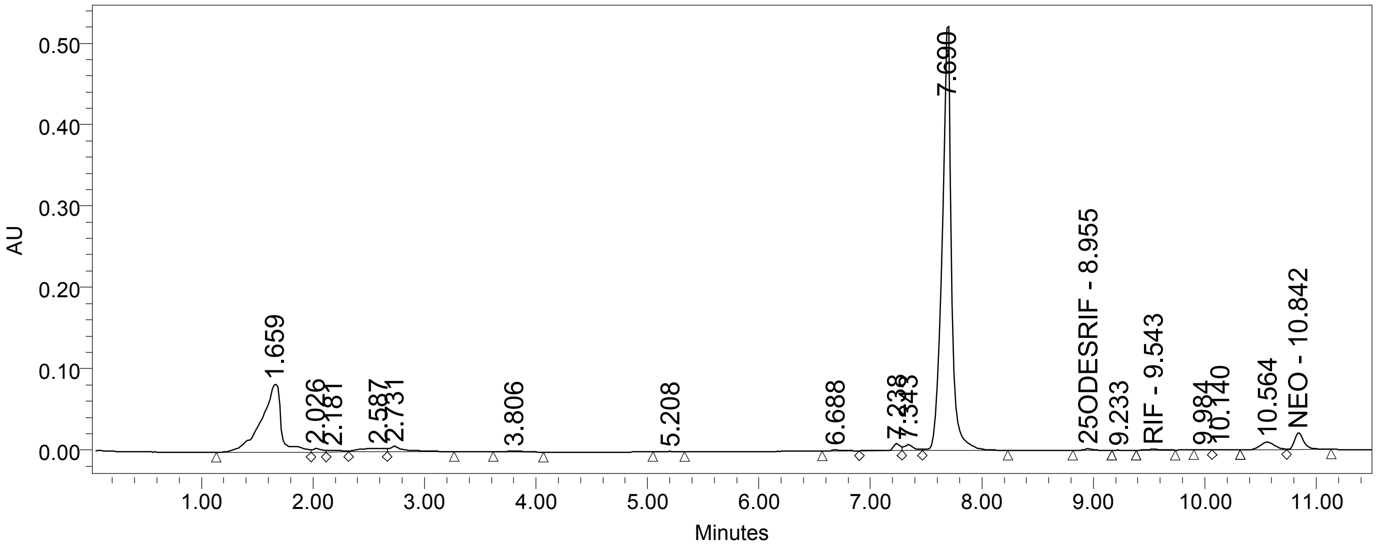

	Peak Name	RT	Area	% Area	Height
1		1.659	1042961	22.38	85747
2		2.026	24607	0.53	3894
3		2.181	22476	0.48	2372
4		2.587	73051	1.57	4483
5		2.731	64325	1.38	6740
6		3.806	11486	0.25	940
7		5.208	3859	0.08	567
8		6.688	11097	0.24	1854
9		7.238	46183	0.99	8550
10		7.343	46359	0.99	7279
11		7.690	3083400	66.16	518315
12	25ODESRIF	8.955	9653	0.21	1812

	Peak Name	RT	Area	% Area	Height
13		9.233	1724	0.04	323
14	RIF	9.543	10086	0.22	1014
15		9.984	2230	0.05	392
16		10.140	1704	0.04	279
17		10.564	85755	1.84	9502
18	NEO	10.842	119242	2.56	20709

Reported by User: Saneesh Kumar (Saneesh) Project Name: HDI_Stellenbosch_2016

SAMPLE INFORMATION

Sample Name:	RIF Inc Basil_EtOaC 10miG/mL 2	Acquired By:	Saneesh
Sample Type:	Unknown	Date Acquired:	2016/05/31 12:53:49 PM
Vial:	9	Acq. Method Set:	HDI_RIF_25ODESRIF_NEO
Injection #:	1	Date Processed:	2016/05/31 05:05:27 PM
Injection Volume:	15.00 ul	Processing Method:	HDI_RIF_ W2996 254.0nm-1.2
Run Time:	11.5 Minutes	Channel Name:	W2996 254.0nm-1.2
Sample Set Name:	RIF_Basil Incubats 31_5_16	Proc. Chnl. Descr.:	W2996 PDA 254.0 nm at 1.2

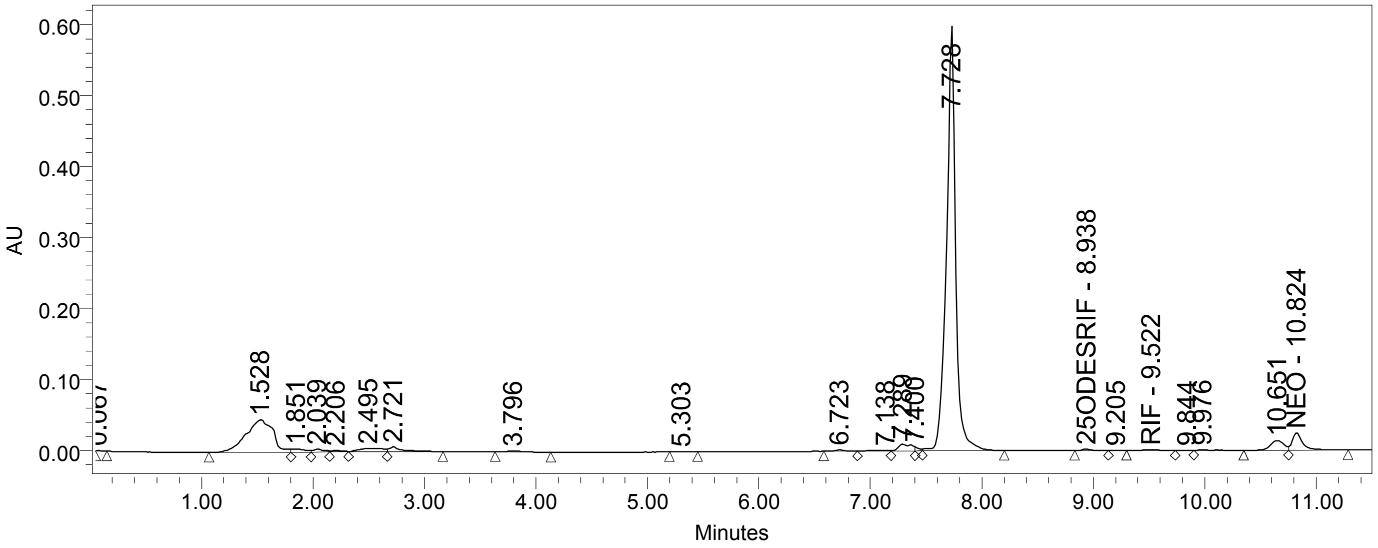

	Peak Name	RT	Area	% Area	Height
1		0.067	1552	0.03	603
2		1.528	759329	15.58	45419
3		1.851	36756	0.75	4290
4		2.039	25250	0.52	4015
5		2.206	13183	0.27	1890
6		2.495	72303	1.48	4342
7		2.721	57085	1.17	6667
8		3.796	12482	0.26	1011
9		5.303	3312	0.07	601
10		6.723	11351	0.23	1801
11		7.138	10584	0.22	852
12		7.289	77773	1.60	9726

Reported by User: Saneesh Kumar (Saneesh) Project Name: HDI_Stellenbosch_2016

	Peak Name	RT	Area	% Area	Height
13		7.400	13673	0.28	5758
14		7.728	3473850	71.29	582280
15	25ODESRIF	8.938	10608	0.22	1996
16		9.205	1790	0.04	307
17	RIF	9.522	11741	0.24	1027
18		9.844	3233	0.07	419
19		9.976	9098	0.19	558
20		10.651	112555	2.31	13695
21	NEO	10.824	155491	3.19	24389

Reported by User: Saneesh Kumar (Saneesh)   Project Name: HDI_Stellenbosch_2016

SAMPLE INFORMATION

Sample Name:	RIF Inc Basil_H2O 200miG/mL 1	Acquired By:	Saneesh
Sample Type:	Unknown	Date Acquired:	2016/05/31 01:09:06 PM
Vial:	10	Acq. Method Set:	HDI_RIF_25ODESRIF_NEO
Injection #:	1	Date Processed:	2016/05/31 05:05:27 PM
Injection Volume:	15.00 ul	Processing Method:	HDI_RIF_ W2996 254.0nm-1.2
Run Time:	11.5 Minutes	Channel Name:	W2996 254.0nm-1.2
Sample Set Name:	RIF_Basil Incubats 31_5_16	Proc. Chnl. Descr.:	W2996 PDA 254.0 nm at 1.2

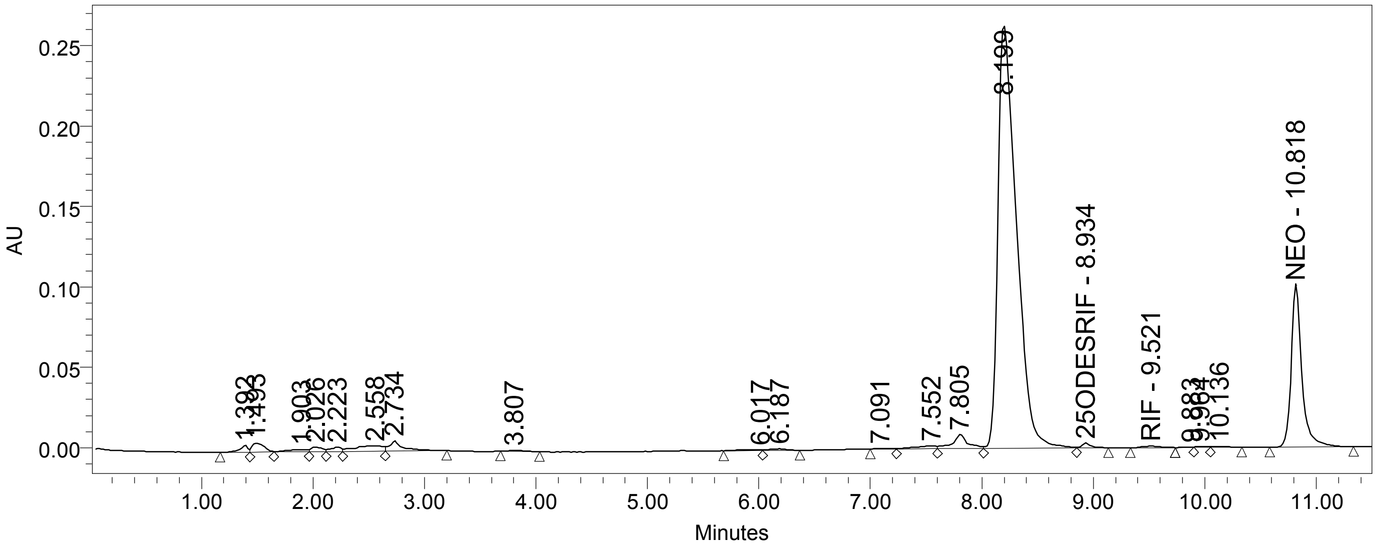

	Peak Name	RT	Area	% Area	Height
1		1.392	20643	0.50	3849
2		1.493	40548	0.98	5433
3		1.903	17641	0.43	1305
4		2.026	19042	0.46	2794
5		2.223	18444	0.45	2892
6		2.558	60622	1.47	3400
7		2.734	55825	1.36	6063
8		3.807	6847	0.17	623
9		6.017	9106	0.22	594
10		6.187	10900	0.26	948
11		7.091	3051	0.07	297
12		7.552	23352	0.57	1717

Reported by User: Saneesh Kumar (Saneesh) Project Name: HDI_Stellenbosch_2016

	Peak Name	RT	Area	% Area	Height
13		7.805	81216	1.97	8632
14		8.199	3042695	73.91	262870
15	25ODESRIF	8.934	15183	0.37	2856
16	RIF	9.521	9989	0.24	1010
17		9.883	1490	0.04	236
18		9.964	3883	0.09	543
19		10.136	5505	0.13	661
20	NEO	10.818	671015	16.30	100301

Empower software

Multi Sample Summary

Reported by User: Saneesh Kumar (Saneesh)   Project Name: HDI_Stellenbosch_2016

SAMPLE INFORMATION

Sample Name:	RIF Inc Basil_H2O 200miG/mL 2	Acquired By:	Saneesh
Sample Type:	Unknown	Date Acquired:	2016/05/31 01:24:33 PM
Vial:	11	Acq. Method Set:	HDI_RIF_25ODESRIF_NEO
Injection #:	1	Date Processed:	2016/05/31 05:05:27 PM
Injection Volume:	15.00 ul	Processing Method:	HDI_RIF_ W2996 254.0nm-1.2
Run Time:	11.5 Minutes	Channel Name:	W2996 254.0nm-1.2
Sample Set Name:	RIF_Basil Incubats 31_5_16	Proc. Chnl. Descr.:	W2996 PDA 254.0 nm at 1.2

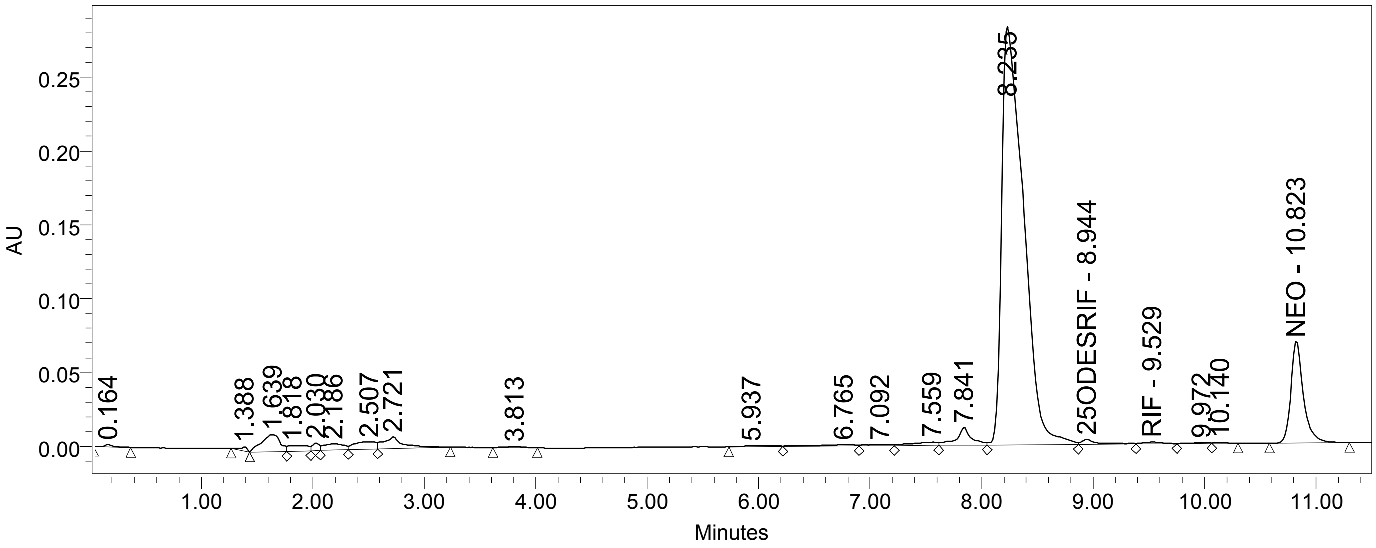

	Peak Name	RT	Area	% Area	Height
1		0.164	9386	0.18	1427
2		1.388	13148	0.26	2926
3		1.639	133259	2.60	11430
4		1.818	45286	0.89	3842
5		2.030	20912	0.41	4931
6		2.186	53919	1.05	4346
7		2.507	70139	1.37	4957
8		2.721	99185	1.94	7652
9		3.813	8878	0.17	763
10		5.937	10801	0.21	581
11		6.765	21178	0.41	852
12		7.092	14154	0.28	891

Reported by User: Saneesh Kumar (Saneesh) Project Name: HDI_Stellenbosch_2016

	Peak Name	RT	Area	% Area	Height
13		7.559	35400	0.69	2166
14		7.841	111518	2.18	11624
15		8.235	3876353	75.78	283744
16	25ODESRIF	8.944	30428	0.59	3195
17	RIF	9.529	13219	0.26	1168
18		9.972	7079	0.14	761
19		10.140	3751	0.07	525
20	NEO	10.823	537532	10.51	69110

Reported by User: Saneesh Kumar (Saneesh)   Project Name: HDI_Stellenbosch_2016

SAMPLE INFORMATION

Sample Name:	RIF Inc Basil_MeOH 200miG/mL 1	Acquired By:	Saneesh
Sample Type:	Unknown	Date Acquired:	2016/05/31 01:39:47 PM
Vial:	12	Acq. Method Set:	HDI_RIF_25ODESRIF_NEO
Injection #:	1	Date Processed:	2016/05/31 05:05:27 PM
Injection Volume:	15.00 ul	Processing Method:	HDI_RIF_ W2996 254.0nm-1.2
Run Time:	11.5 Minutes	Channel Name:	W2996 254.0nm-1.2
Sample Set Name:	RIF_Basil Incubats 31_5_16	Proc. Chnl. Descr.:	W2996 PDA 254.0 nm at 1.2

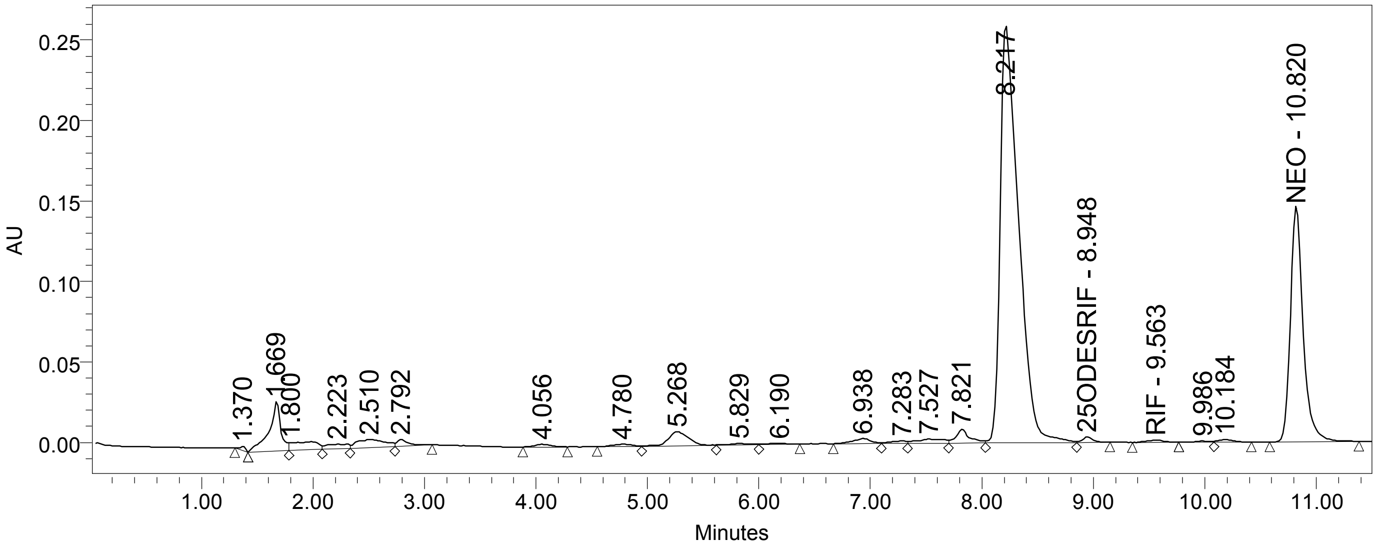

	Peak Name	RT	Area	% Area	Height
1		1.370	9419	0.19	2599
2		1.669	229647	4.58	29498
3		1.800	82844	1.65	5119
4		2.223	39165	0.78	2817
5		2.510	87404	1.74	4913
6		2.792	30425	0.61	4287
7		4.056	17397	0.35	1710
8		4.780	17351	0.35	1471
9		5.268	106451	2.12	8657
10		5.829	10922	0.22	794
11		6.190	7604	0.15	657
12		6.938	35074	0.70	3149

Reported by User: Saneesh Kumar (Saneesh) Project Name: HDI_Stellenbosch_2016

	Peak Name	RT	Area	% Area	Height
13		7.283	15090	0.30	1498
14		7.527	44088	0.88	2475
15		7.821	72253	1.44	8484
16		8.217	2992299	59.63	259380
17	25ODESRIF	8.948	21265	0.42	3357
18	RIF	9.563	14736	0.29	1468
19		9.986	6526	0.13	657
20		10.184	14172	0.28	1706
21	NEO	10.820	1164392	23.20	145812

SAMPLE INFORMATION

Sample Name:	RIF Inc Basil_MeOH 200miG/mL 2	Acquired By:	Saneesh
Sample Type:	Unknown	Date Acquired:	2016/05/31 01:55:03 PM
Vial:	13	Acq. Method Set:	HDI_RIF_25ODESRIF_NEO
Injection #:	1	Date Processed:	2016/05/31 05:05:27 PM
Injection Volume:	15.00 ul	Processing Method:	HDI_RIF_ W2996 254.0nm-1.2
Run Time:	11.5 Minutes	Channel Name:	W2996 254.0nm-1.2
Sample Set Name:	RIF_Basil Incubats 31_5_16	Proc. Chnl. Descr.:	W2996 PDA 254.0 nm at 1.2

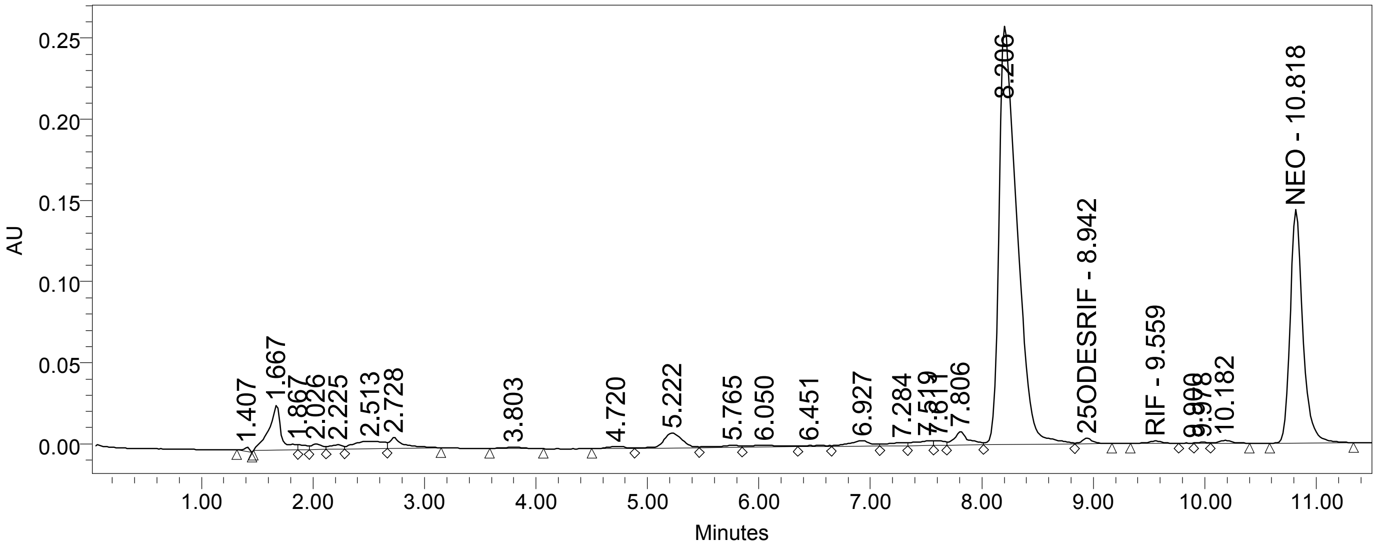

	Peak Name	RT	Area	% Area	Height
1		1.407	8832	0.18	2413
2		1.667	216532	4.42	26949
3		1.867	15567	0.32	3049
4		2.026	22710	0.46	3377
5		2.225	21840	0.45	2819
6		2.513	85928	1.75	4540
7		2.728	57952	1.18	6630
8		3.803	12707	0.26	947
9		4.720	14397	0.29	1206
10		5.222	107351	2.19	9135
11		5.765	17834	0.36	1279
12		6.050	23884	0.49	1185

Reported by User: Saneesh Kumar (Saneesh) Project Name: HDI_Stellenbosch_2016

	Peak Name	RT	Area	% Area	Height
13		6.451	9918	0.20	625
14		6.927	44506	0.91	3458
15		7.284	22411	0.46	1888
16		7.519	32212	0.66	2834
17		7.611	17963	0.37	2819
18		7.806	75090	1.53	8201
19		8.206	2894216	59.01	259541
20	25ODESRIF	8.942	23582	0.48	3635
21	RIF	9.559	14800	0.30	1523
22		9.900	1234	0.03	261
23		9.978	5123	0.10	727
24		10.182	16103	0.33	1836
25	NEO	10.818	1141680	23.28	143123

SAMPLE INFORMATION

Sample Name:	RIF Inc Basil_EOH 200miG/mL 1	Acquired By:	Saneesh
Sample Type:	Unknown	Date Acquired:	2016/05/31 02:10:18 PM
Vial:	14	Acq. Method Set:	HDI_RIF_25ODESRIF_NEO
Injection #:	1	Date Processed:	2016/05/31 05:05:27 PM
Injection Volume:	15.00 ul	Processing Method:	HDI_RIF_ W2996 254.0nm-1.2
Run Time:	11.5 Minutes	Channel Name:	W2996 254.0nm-1.2
Sample Set Name:	RIF_Basil Incubats 31_5_16	Proc. Chnl. Descr.:	W2996 PDA 254.0 nm at 1.2

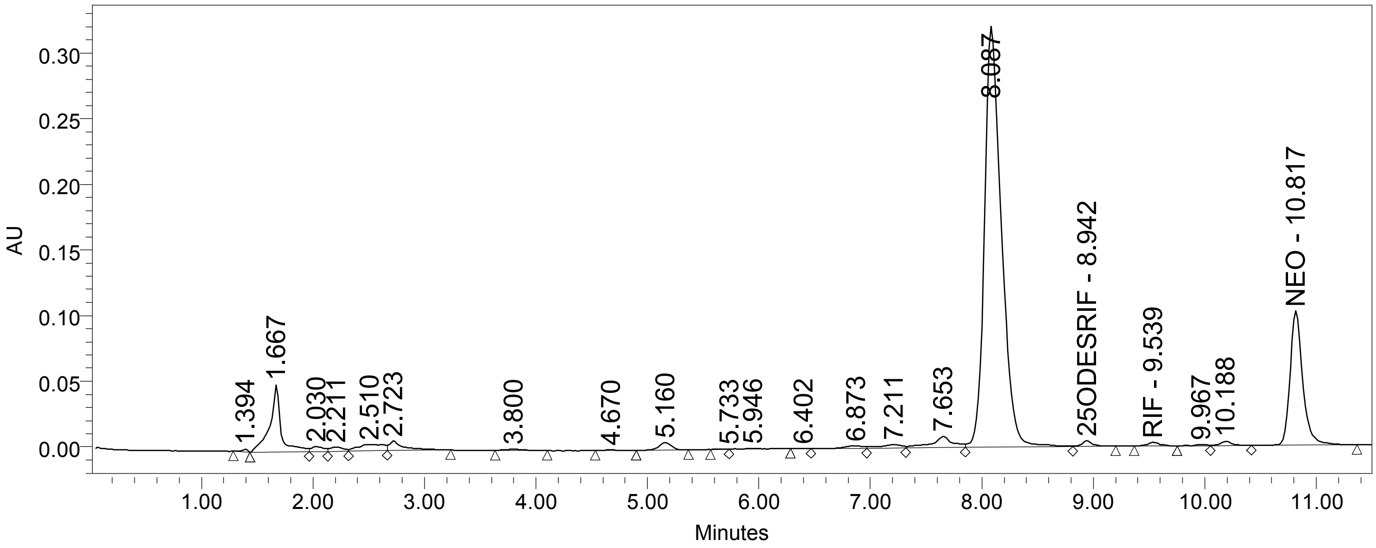

	Peak Name	RT	Area	% Area	Height
1		1.394	8003	0.16	2205
2		1.667	354932	7.04	48035
3		2.030	29799	0.59	3687
4		2.211	26768	0.53	3224
5		2.510	82047	1.63	4943
6		2.723	68298	1.36	7140
7		3.800	10867	0.22	872
8		4.670	3488	0.07	418
9		5.160	43622	0.87	5729
10		5.733	1338	0.03	215
11		5.946	6400	0.13	344
12		6.402	1336	0.03	242

	Peak Name	RT	Area	% Area	Height
13		6.873	22851	0.45	2034
14		7.211	34256	0.68	2529
15		7.653	108757	2.16	8556
16		8.087	3340026	66.29	320050
17	25ODESRIF	8.942	25804	0.51	4163
18	RIF	9.539	22996	0.46	2852
19		9.967	7247	0.14	820
20		10.188	25251	0.50	3259
21	NEO	10.817	814449	16.16	101763

SAMPLE INFORMATION

Sample Name:	RIF Inc Baisl_EOH 200miG/mL 2	Acquired By:	Saneesh
Sample Type:	Unknown	Date Acquired:	2016/05/31 02:25:35 PM
Vial:	15	Acq. Method Set:	HDI_RIF_25ODESRIF_NEO
Injection #:	1	Date Processed:	2016/05/31 05:05:27 PM
Injection Volume:	15.00 ul	Processing Method:	HDI_RIF_ W2996 254.0nm-1.2
Run Time:	11.5 Minutes	Channel Name:	W2996 254.0nm-1.2
Sample Set Name:	RIF_Basil Incubats 31_5_16	Proc. Chnl. Descr.:	W2996 PDA 254.0 nm at 1.2

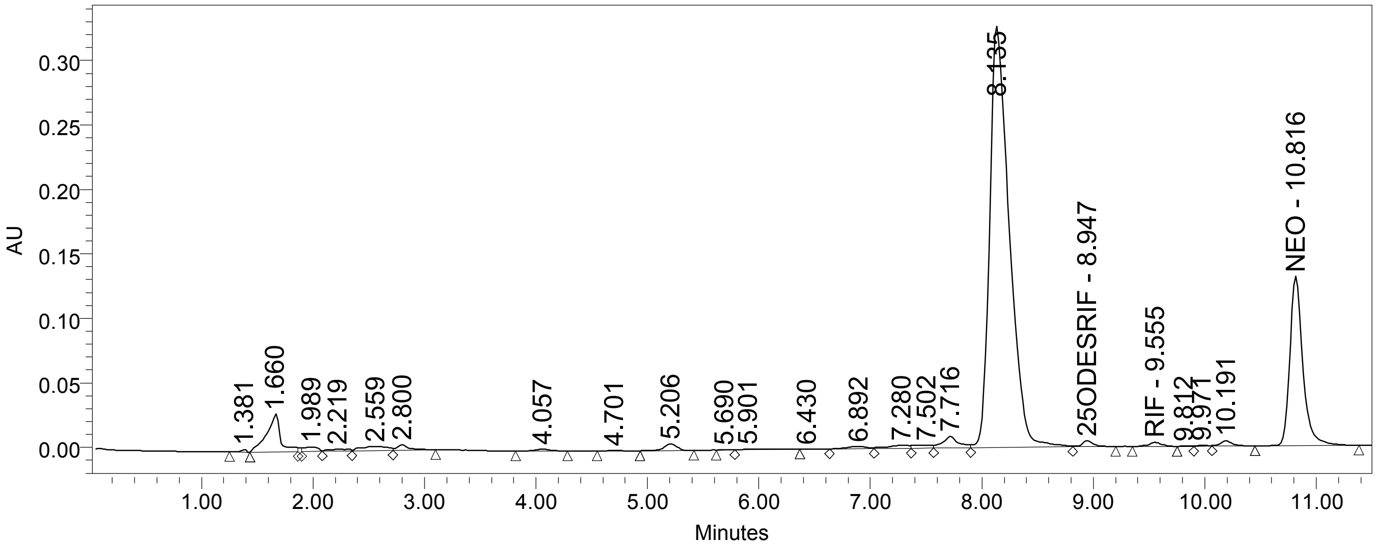

	Peak Name	RT	Area	% Area	Height
1		1.381	7440	0.13	2054
2		1.660	254803	4.44	29123
3		1.989	29945	0.52	3406
4		2.219	18983	0.33	1526
5		2.559	52106	0.91	3024
6		2.800	29661	0.52	4039
7		4.057	15285	0.27	1505
8		4.701	4039	0.07	364
9		5.206	41319	0.72	5244
10		5.690	1613	0.03	245
11		5.901	6294	0.11	395
12		6.430	1723	0.03	207

Reported by User: Saneesh Kumar (Saneesh) Project Name: HDI_Stellenbosch_2016

	Peak Name	RT	Area	% Area	Height
13		6.892	23584	0.41	1886
14		7.280	32806	0.57	2276
15		7.502	26879	0.47	2405
16		7.716	88885	1.55	8932
17		8.135	3943926	68.70	326637
18	25ODESRIF	8.947	30429	0.53	4670
19	RIF	9.555	25310	0.44	3202
20		9.812	1358	0.02	239
21		9.971	4738	0.08	660
22		10.191	30235	0.53	4084
23	NEO	10.816	1069567	18.63	130858

S A M P L E   I N F O R M A T I O N

Sample Name:	RIF Inc Elecam_EtoAc 200miG/mL 1	Acquired By:	Saneesh
Sample Type:	Unknown	Date Acquired:	2016/05/31 02:40:50 PM
Vial:	16	Acq. Method Set:	HDI_RIF_25ODESRIF_NEO
Injection #:	1	Date Processed:	2016/05/31 05:05:27 PM
Injection Volume:	15.00 ul	Processing Method:	HDI_RIF_ W2996 254.0nm-1.2
Run Time:	11.5 Minutes	Channel Name:	W2996 254.0nm-1.2
Sample Set Name:	RIF_Basil Incubats 31_5_16	Proc. Chnl. Descr.:	W2996 PDA 254.0 nm at 1.2

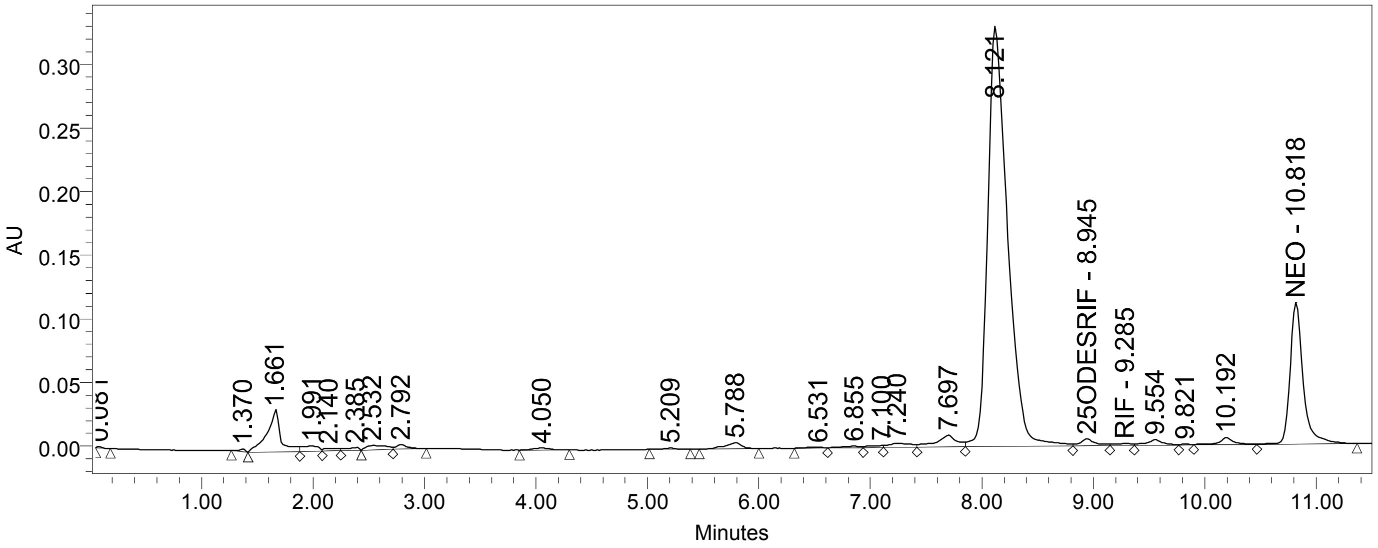

	Peak Name	RT	Area	% Area	Height
1		0.081	2784	0.05	938
2		1.370	8715	0.15	2142
3		1.661	280070	4.80	32172
4		1.991	40899	0.70	4042
5		2.140	18011	0.31	2083
6		2.385	17990	0.31	2252
7		2.532	44491	0.76	3480
8		2.792	26066	0.45	3756
9		4.050	17062	0.29	1674
10		5.209	6786	0.12	1020
11		5.788	45568	0.78	4585
12		6.531	7052	0.12	720

	Peak Name	RT	Area	% Area	Height
13		6.855	15455	0.26	1327
14		7.100	14977	0.26	1550
15		7.240	45283	0.78	3293
16		7.697	119276	2.04	9263
17		8.121	4056573	69.49	330528
18	25ODESRIF	8.945	49956	0.86	5413
19	RIF	9.285	17372	0.30	1697
20		9.554	45324	0.78	4466
21		9.821	4387	0.08	615
22		10.192	51943	0.89	5535
23	NEO	10.818	901924	15.45	111004

Reported by User: Saneesh Kumar (Saneesh) Project Name: HDI_Stellenbosch_2016

SAMPLE INFORMATION

Sample Name:	RIF Inc Elecam_EtoAc 200miG/mL 2	Acquired By:	Saneesh
Sample Type:	Unknown	Date Acquired:	2016/05/31 02:56:05 PM
Vial:	17	Acq. Method Set:	HDI_RIF_25ODESRIF_NEO
Injection #:	1	Date Processed:	2016/05/31 05:05:27 PM
Injection Volume:	15.00 ul	Processing Method:	HDI_RIF_ W2996 254.0nm-1.2
Run Time:	11.5 Minutes	Channel Name:	W2996 254.0nm-1.2
Sample Set Name:	RIF_Basil Incubats 31_5_16	Proc. Chnl. Descr.:	W2996 PDA 254.0 nm at 1.2

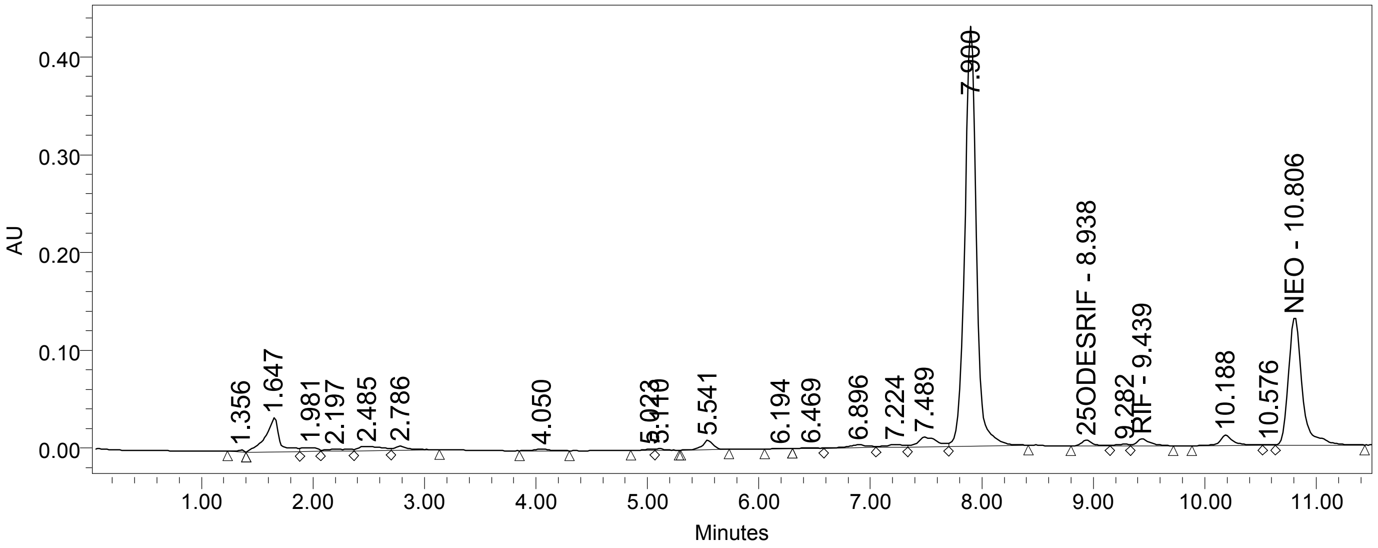

	Peak Name	RT	Area	% Area	Height
1		1.356	8741	0.17	2091
2		1.647	310145	5.94	34663
3		1.981	37818	0.72	3970
4		2.197	30218	0.58	1929
5		2.485	63403	1.21	4184
6		2.786	37430	0.72	4183
7		4.050	17242	0.33	1658
8		5.023	7247	0.14	1146
9		5.110	7417	0.14	1520
10		5.541	61177	1.17	9523
11		6.194	1985	0.04	242
12		6.469	8488	0.16	892

Reported by User: Saneesh Kumar (Saneesh) Project Name: HDI_Stellenbosch_2016

	Peak Name	RT	Area	% Area	Height
13		6.896	40357	0.77	3171
14		7.224	32675	0.63	2898
15		7.489	118933	2.28	9806
16		7.900	3091032	59.22	427237
17	25ODESRIF	8.938	38898	0.75	5961
18		9.282	12905	0.25	1967
19	RIF	9.439	58480	1.12	7071
20		10.188	90384	1.73	10770
21		10.576	4240	0.08	722
22	NEO	10.806	1139939	21.84	130545

Reported by User: Saneesh Kumar (Saneesh) Project Name: HDI_Stellenbosch_2016

SAMPLE INFORMATION

Sample Name:	RIF Inc Basil_H2O 0.1mg/mL	Acquired By:	Saneesh
Sample Type:	Unknown	Date Acquired:	2016/05/31 03:11:12 PM
Vial:	18	Acq. Method Set:	HDI_RIF_25ODESRIF_NEO
Injection #:	1	Date Processed:	2016/05/31 05:05:27 PM
Injection Volume:	15.00 ul	Processing Method:	HDI_RIF_ W2996 254.0nm-1.2
Run Time:	11.5 Minutes	Channel Name:	W2996 254.0nm-1.2
Sample Set Name:	RIF_Basil Incubats 31_5_16	Proc. Chnl. Descr.:	W2996 PDA 254.0 nm at 1.2

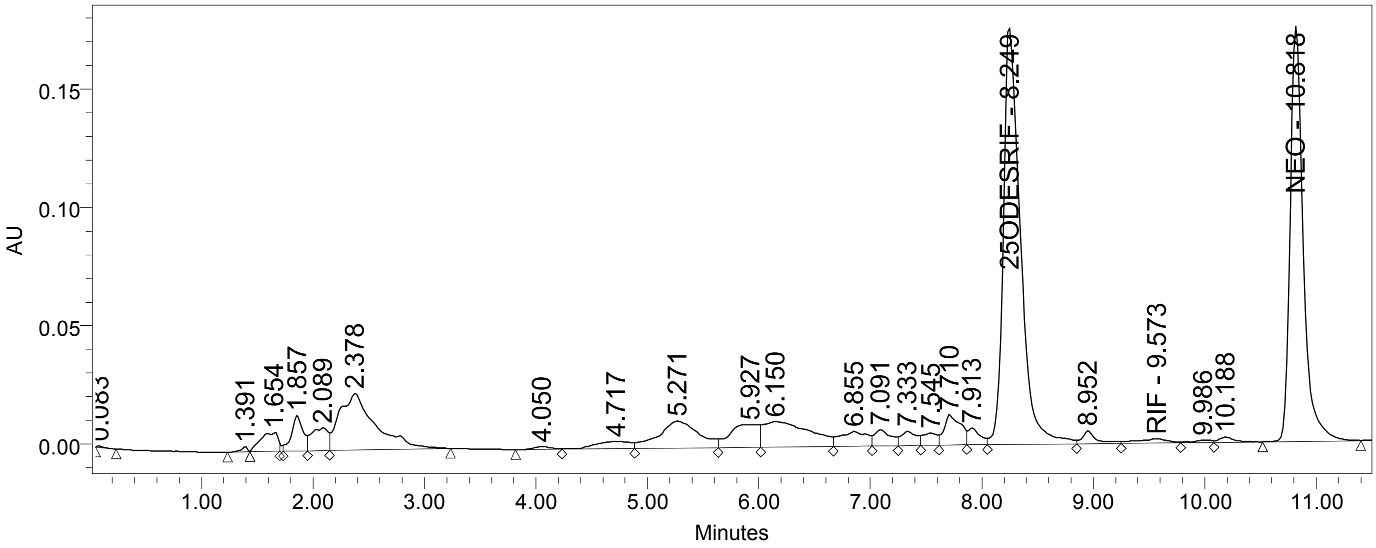

	Peak Name	RT	Area	% Area	Height
1		0.083	3037	0.05	820
2		1.391	8240	0.15	1968
3		1.654	86374	1.55	8046
4		1.857	107548	1.93	14998
5		2.089	99681	1.78	9495
6		2.378	533477	9.55	23956
7		4.050	13828	0.25	1286
8		4.717	70043	1.25	3093
9		5.271	284130	5.09	11408
10		5.927	176114	3.15	9656
11		6.150	309050	5.53	10683
12		6.855	104706	1.87	6273

Reported by User: Saneesh Kumar (Saneesh) Project Name: HDI_Stellenbosch_2016

	Peak Name	RT	Area	% Area	Height
13		7.091	69564	1.25	6767
14		7.333	57689	1.03	6076
15		7.545	46958	0.84	5049
16		7.710	134192	2.40	12918
17		7.913	53846	0.96	7169
18	25ODESRIF	8.249	1882222	33.70	176229
19		8.952	47076	0.84	5448
20	RIF	9.573	33463	0.60	1745
21		9.986	11867	0.21	1070
22		10.188	19668	0.35	2135
23	NEO	10.818	1432970	25.65	174864

SAMPLE INFORMATION

Sample Name:	RIF Inc Basil_MeOH 0.1Mg/mL	Acquired By:	Saneesh
Sample Type:	Unknown	Date Acquired:	2016/05/31 03:26:21 PM
Vial:	19	Acq. Method Set:	HDI_RIF_25ODESRIF_NEO
Injection #:	1	Date Processed:	2016/05/31 05:05:27 PM
Injection Volume:	15.00 ul	Processing Method:	HDI_RIF_ W2996 254.0nm-1.2
Run Time:	11.5 Minutes	Channel Name:	W2996 254.0nm-1.2
Sample Set Name:	RIF_Basil Incubats 31_5_16	Proc. Chnl. Descr.:	W2996 PDA 254.0 nm at 1.2

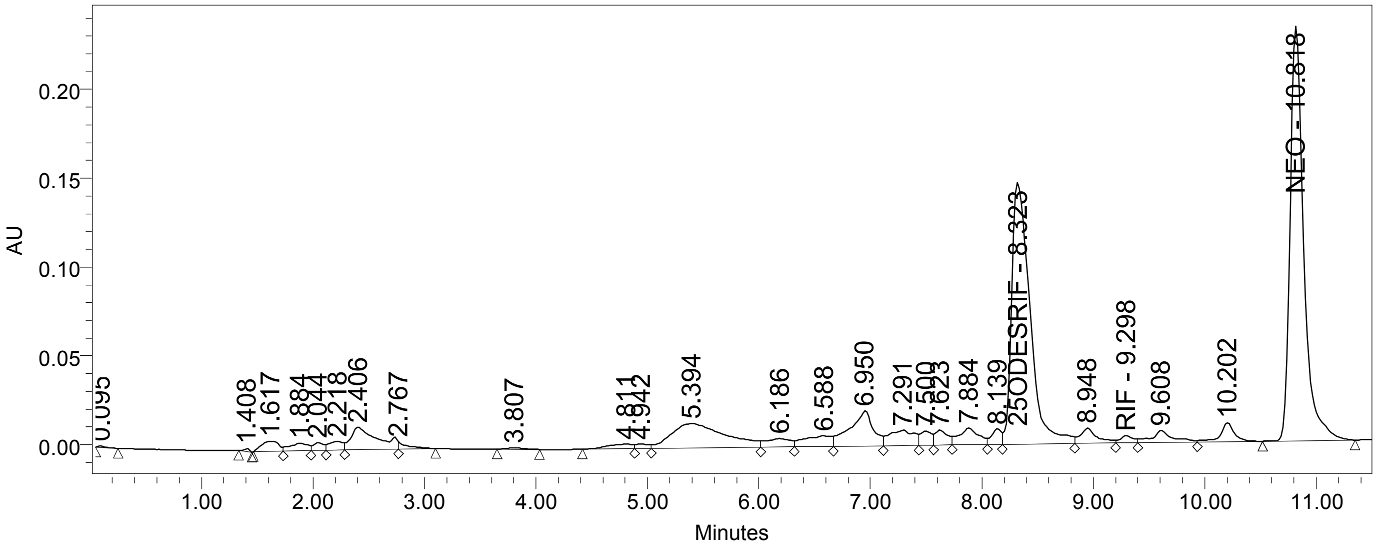

	Peak Name	RT	Area	% Area	Height
1		0.095	4443	0.08	972
2		1.408	5893	0.10	1744
3		1.617	61197	1.06	5603
4		1.884	48322	0.83	4293
5		2.044	28440	0.49	4284
6		2.218	43358	0.75	4950
7		2.406	210093	3.62	12782
8		2.767	22288	0.38	4122
9		3.807	8375	0.14	775
10		4.811	40605	0.70	2563
11		4.942	20905	0.36	2591
12		5.394	458283	7.90	13638

	Peak Name	RT	Area	% Area	Height
13		6.186	71669	1.24	4766
14		6.588	103953	1.79	5867
15		6.950	286846	4.95	19690
16		7.291	132950	2.29	8607
17		7.500	56489	0.97	7821
18		7.623	65737	1.13	8336
19		7.884	115702	1.99	9343
20		8.139	51344	0.89	8748
21	25ODESRIF	8.323	1680384	28.97	148240
22		8.948	90224	1.56	8469
23	RIF	9.298	32814	0.57	4128
24		9.608	87962	1.52	6774
25		10.202	92638	1.60	10738
26	NEO	10.818	1979586	34.13	232746

Multi Sample Summary

Reported by User: Saneesh Kumar (Saneesh)

Project Name: HDI_Stellenbosch_2016

SAMPLE INFORMATION

Sample Name:	RIF Inc Basil_EOH 0.1mg/mL	Acquired By:	Saneesh
Sample Type:	Unknown	Date Acquired:	2016/05/31 03:41:35 PM
Vial:	20	Acq. Method Set:	HDI_RIF_25ODESRIF_NEO
Injection #:	1	Date Processed:	2016/05/31 05:05:27 PM
Injection Volume:	15.00 ul	Processing Method:	HDI_RIF_ W2996 254.0nm-1.2
Run Time:	11.5 Minutes	Channel Name:	W2996 254.0nm-1.2
Sample Set Name:	RIF_Basil Incubats 31_5_16	Proc. Chnl. Descr.:	W2996 PDA 254.0 nm at 1.2

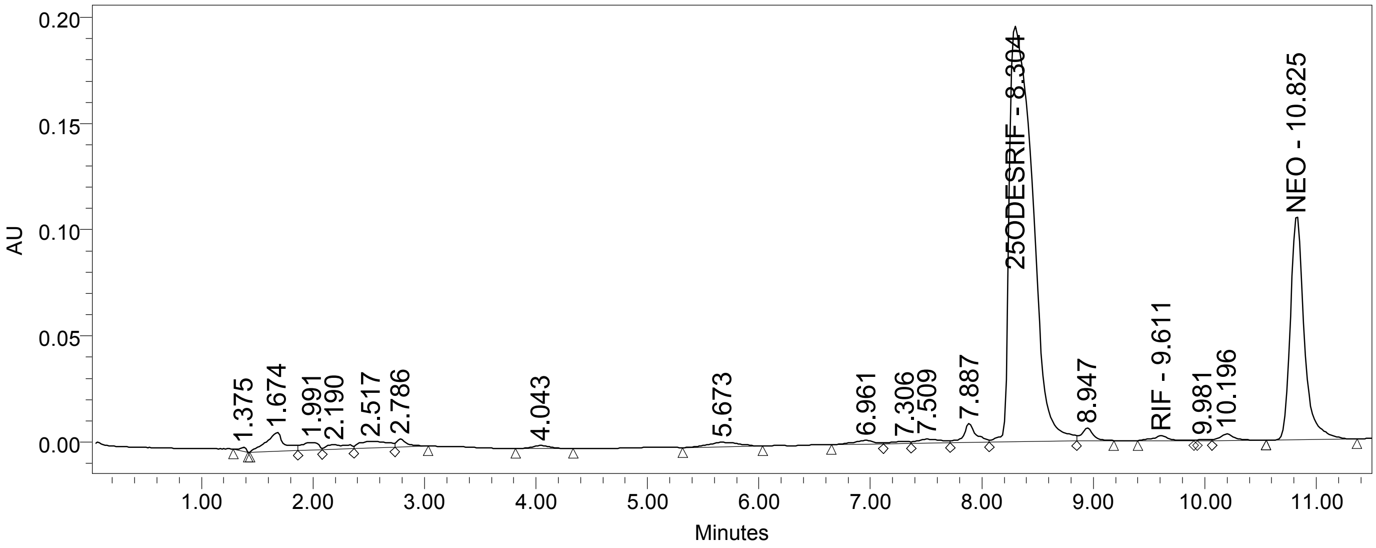

	Peak Name	RT	Area	% Area	Height
1		1.375	6912	0.16	1855
2		1.674	104993	2.45	8903
3		1.991	36969	0.86	3647
4		2.190	28551	0.67	2080
5		2.517	55311	1.29	3106
6		2.786	23481	0.55	3617
7		4.043	15208	0.35	1399
8		5.673	40284	0.94	2143
9		6.961	22642	0.53	1815
10		7.306	12635	0.29	1091
11		7.509	29412	0.69	1943
12		7.887	71231	1.66	8825

	Peak Name	RT	Area	% Area	Height
13	25ODESRIF	8.304	2824719	65.93	196182
14		8.947	45259	1.06	6102
15	RIF	9.611	22735	0.53	2287
16		9.981	3306	0.08	486
17		10.196	26156	0.61	3051
18	NEO	10.825	914470	21.34	105510

SAMPLE INFORMATION

Sample Name:	RIF Inc Basil_EtoAc 0.1mg/mL	Acquired By:	Saneesh
Sample Type:	Unknown	Date Acquired:	2016/05/31 03:56:42 PM
Vial:	21	Acq. Method Set:	HDI_RIF_25ODESRIF_NEO
Injection #:	1	Date Processed:	2016/05/31 05:05:27 PM
Injection Volume:	15.00 ul	Processing Method:	HDI_RIF_ W2996 254.0nm-1.2
Run Time:	11.5 Minutes	Channel Name:	W2996 254.0nm-1.2
Sample Set Name:	RIF_Basil Incubats 31_5_16	Proc. Chnl. Descr.:	W2996 PDA 254.0 nm at 1.2

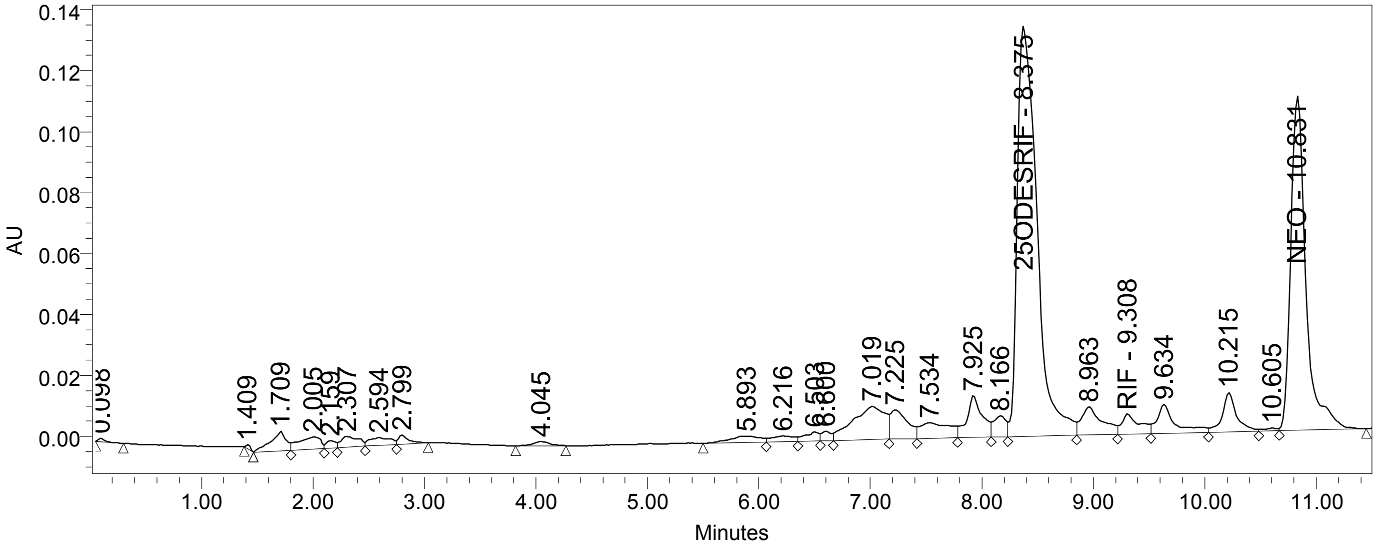

	Peak Name	RT	Area	% Area	Height
1		0.098	4354	0.11	1054
2		1.409	3619	0.09	1067
3		1.709	62653	1.54	6223
4		2.005	53969	1.33	3841
5		2.159	13962	0.34	2446
6		2.307	38170	0.94	3533
7		2.594	33649	0.83	2468
8		2.799	17821	0.44	2923
9		4.045	14342	0.35	1425
10		5.893	39167	0.96	2064
11		6.216	26311	0.65	1862
12		6.503	25931	0.64	2961

Reported by User: Saneesh Kumar (Saneesh) Project Name: HDI_Stellenbosch_2016

	Peak Name	RT	Area	% Area	Height
13		6.600	18373	0.45	3030
14		7.019	219153	5.40	10889
15		7.225	104006	2.56	9626
16		7.534	95253	2.35	5182
17		7.925	130967	3.23	13694
18		8.166	52514	1.29	6845
19	25ODESRIF	8.375	1656063	40.80	135476
20		8.963	118127	2.91	9266
21	RIF	9.308	71875	1.77	6494
22		9.634	105041	2.59	9478
23		10.215	113648	2.80	12842
24		10.605	8011	0.20	925
25	NEO	10.831	1032072	25.43	109460

Reported by User: Saneesh Kumar (Saneesh)   Project Name: HDI_Stellenbosch_2016

S A M P L E   I N F O R M A T I O N

Sample Name:	RIF Inc Basil_H2O 1mg/mL	Acquired By:	Saneesh
Sample Type:	Unknown	Date Acquired:	2016/05/31 04:11:48 PM
Vial:	22	Acq. Method Set:	HDI_RIF_25ODESRIF_NEO
Injection #:	1	Date Processed:	2016/05/31 05:05:27 PM
Injection Volume:	15.00 ul	Processing Method:	HDI_RIF_ W2996 254.0nm-1.2
Run Time:	11.5 Minutes	Channel Name:	W2996 254.0nm-1.2
Sample Set Name:	RIF_Basil Incubats 31_5_16	Proc. Chnl. Descr.:	W2996 PDA 254.0 nm at 1.2

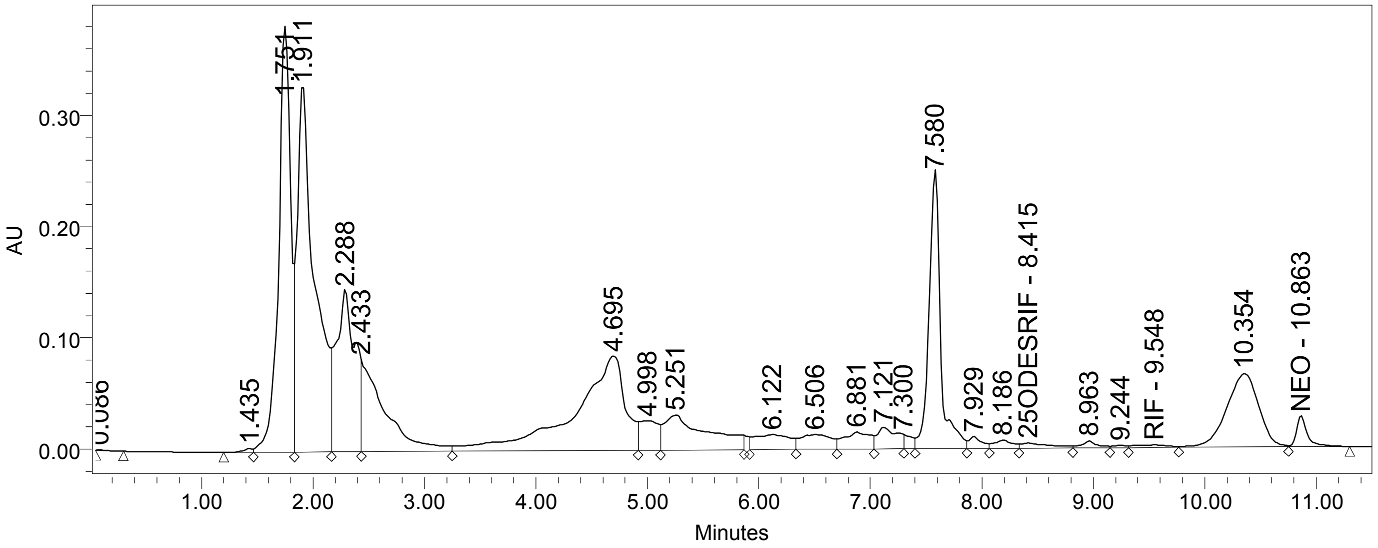

	Peak Name	RT	Area	% Area	Height
1		0.086	4125	0.02	995
2		1.435	18110	0.10	3259
3		1.751	2911367	16.14	382852
4		1.911	3604518	19.99	329460
5		2.288	1712896	9.50	144566
6		2.433	1202597	6.67	82792
7		4.695	2670419	14.81	84864
8		4.998	304445	1.69	26507
9		5.251	858328	4.76	31391
10		6.122	294343	1.63	13442
11		6.506	251726	1.40	13218
12		6.881	244222	1.35	15221

Reported by User: Saneesh Kumar (Saneesh) Project Name: HDI_Stellenbosch_2016

	Peak Name	RT	Area	% Area	Height
13		7.121	240001	1.33	19355
14		7.300	66696	0.37	12673
15		7.580	1785437	9.90	250467
16		7.929	84142	0.47	10535
17		8.186	83358	0.46	7267
18	25ODESRIF	8.415	82537	0.46	4503
19		8.963	56643	0.31	5944
20		9.244	16604	0.09	1945
21	RIF	9.548	45725	0.25	2227
22		10.354	1314400	7.29	65828
23	NEO	10.863	182436	1.01	27401

SAMPLE INFORMATION

Sample Name:	RIF Inc Basil_MeOH 1mg/mL	Acquired By:	Saneesh
Sample Type:	Unknown	Date Acquired:	2016/05/31 04:26:54 PM
Vial:	23	Acq. Method Set:	HDI_RIF_25ODESRIF_NEO
Injection #:	1	Date Processed:	2016/05/31 05:05:27 PM
Injection Volume:	15.00 ul	Processing Method:	HDI_RIF_ W2996 254.0nm-1.2
Run Time:	11.5 Minutes	Channel Name:	W2996 254.0nm-1.2
Sample Set Name:	RIF_Basil Incubats 31_5_16	Proc. Chnl. Descr.:	W2996 PDA 254.0 nm at 1.2

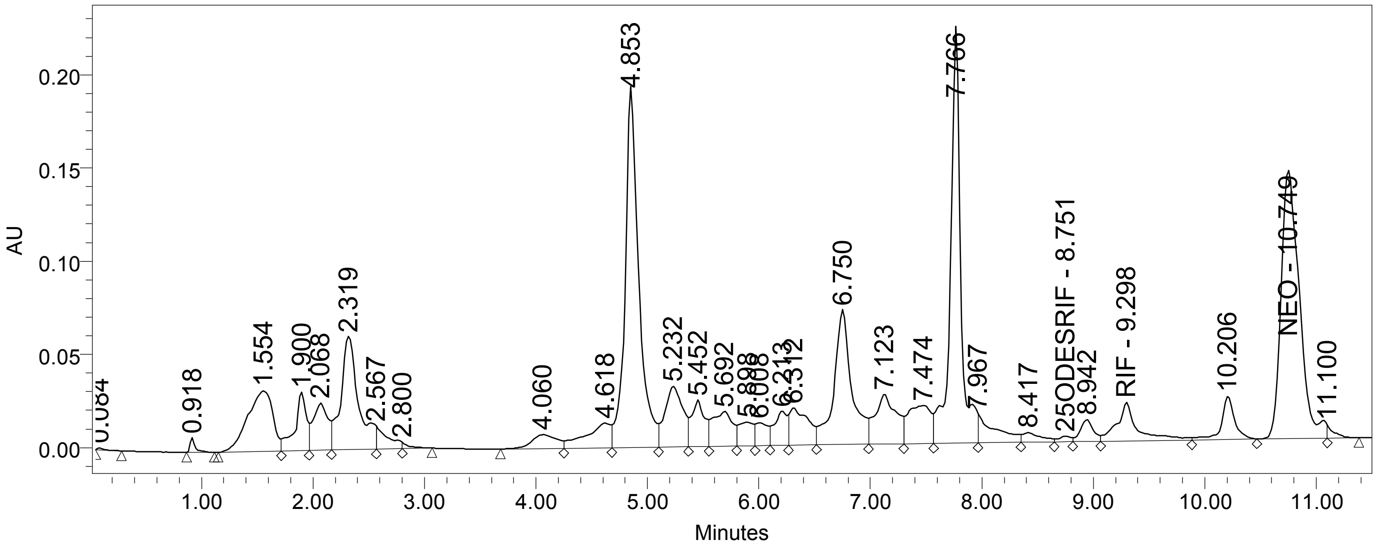

	Peak Name	RT	Area	% Area	Height
1		0.084	3378	0.03	944
2		0.918	24176	0.23	6908
3		1.554	508616	4.88	32312
4		1.900	221493	2.12	30917
5		2.068	233905	2.24	25169
6		2.319	664671	6.38	61008
7		2.567	91647	0.88	12364
8		2.800	13802	0.13	3455
9		4.060	116100	1.11	7446
10		4.618	207505	1.99	13197
11		4.853	1630964	15.65	192497
12		5.232	361414	3.47	32340

	Peak Name	RT	Area	% Area	Height
13		5.452	203516	1.95	24580
14		5.692	229140	2.20	18484
15		5.898	119637	1.15	12586
16		6.008	92226	0.88	12361
17		6.213	147444	1.41	18204
18		6.312	231019	2.22	19863
19		6.750	793972	7.62	71806
20		7.123	352261	3.38	26559
21		7.474	293094	2.81	20424
22		7.766	1413967	13.57	218811
23		7.967	162645	1.56	15654
24		8.417	60190	0.58	5212
25	25ODESRIF	8.751	23112	0.22	2948
26		8.942	95145	0.91	11539
27	RIF	9.298	265230	2.54	20449
28		10.206	189512	1.82	23166
29	NEO	10.749	1644644	15.78	143955
30		11.100	29077	0.28	7179

SAMPLE INFORMATION

Sample Name:	RIF Inc Basil_EOH 1mg/mL	Acquired By:	Saneesh
Sample Type:	Unknown	Date Acquired:	2016/05/31 04:42:00 PM
Vial:	24	Acq. Method Set:	HDI_RIF_25ODESRIF_NEO
Injection #:	1	Date Processed:	2016/05/31 05:05:27 PM
Injection Volume:	15.00 ul	Processing Method:	HDI_RIF_ W2996 254.0nm-1.2
Run Time:	11.5 Minutes	Channel Name:	W2996 254.0nm-1.2
Sample Set Name:	RIF_Basil Incubats 31_5_16	Proc. Chnl. Descr.:	W2996 PDA 254.0 nm at 1.2

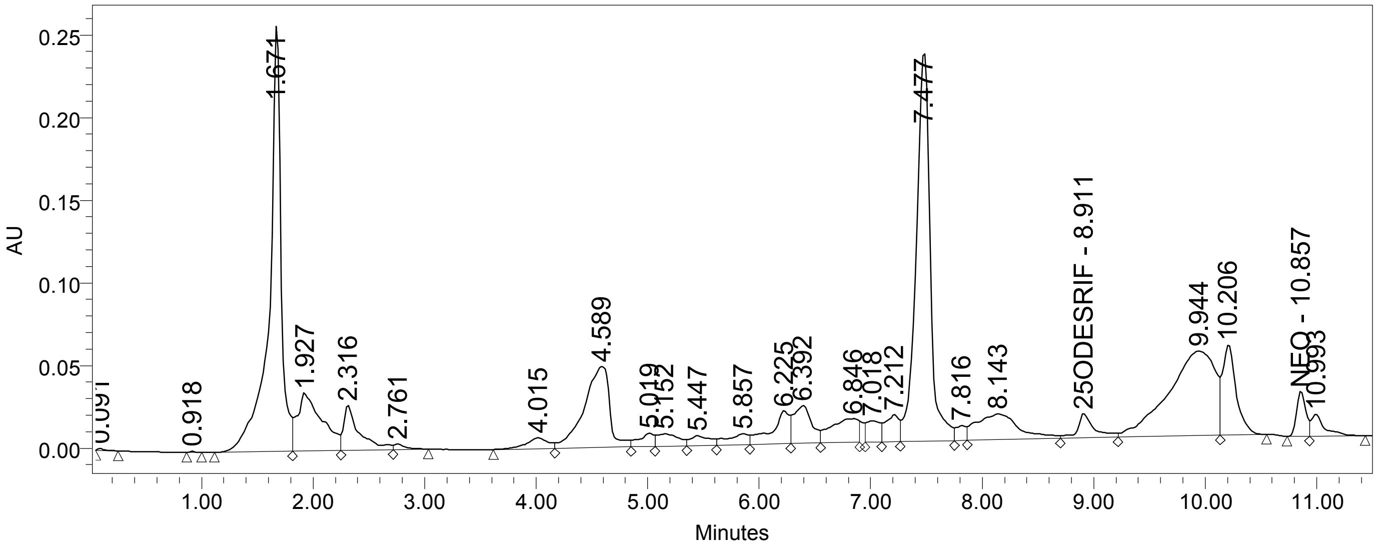

	Peak Name	RT	Area	% Area	Height
1		0.091	4102	0.04	952
2		0.918	1716	0.02	581
3		1.671	1901687	19.35	254630
4		1.927	538438	5.48	35166
5		2.316	263335	2.68	26928
6		2.761	23951	0.24	3412
7		4.015	88968	0.91	6563
8		4.589	782575	7.96	49032
9		5.019	74535	0.76	7913
10		5.152	101344	1.03	7341
11		5.447	72762	0.74	6029
12		5.857	87640	0.89	6533

Reported by User: Saneesh Kumar (Saneesh)   Project Name: HDI_Stellenbosch_2016

	Peak Name	RT	Area	% Area	Height
13		6.225	225171	2.29	20008
14		6.392	257572	2.62	23024
15		6.846	246795	2.51	14352
16		7.018	109691	1.12	12756
17		7.212	137936	1.40	16164
18		7.477	2079550	21.16	235602
19		7.816	57660	0.59	8792
20		8.143	405801	4.13	15498
21	25ODESRIF	8.911	153159	1.56	14601
22	RIF	9.400			
23		9.944	1487614	15.13	50977
24		10.206	463652	4.72	54345
25	NEO	10.857	150986	1.54	27081
26		10.993	112906	1.15	13139

SAMPLE INFORMATION

Sample Name:	RIF Inc Basil_EToAc 1mg/mL	Acquired By:	Saneesh
Sample Type:	Unknown	Date Acquired:	2016/05/31 04:57:07 PM
Vial:	2	Acq. Method Set:	HDI_RIF_25ODESRIF_NEO
Injection #:	1	Date Processed:	2016/05/31 05:09:19 PM
Injection Volume:	15.00 ul	Processing Method:	HDI_RIF_ W2996 254.0nm-1.2
Run Time:	11.5 Minutes	Channel Name:	W2996 254.0nm-1.2
Sample Set Name:	RIF_Basil Incubats 31_5_16	Proc. Chnl. Descr.:	W2996 PDA 254.0 nm at 1.2

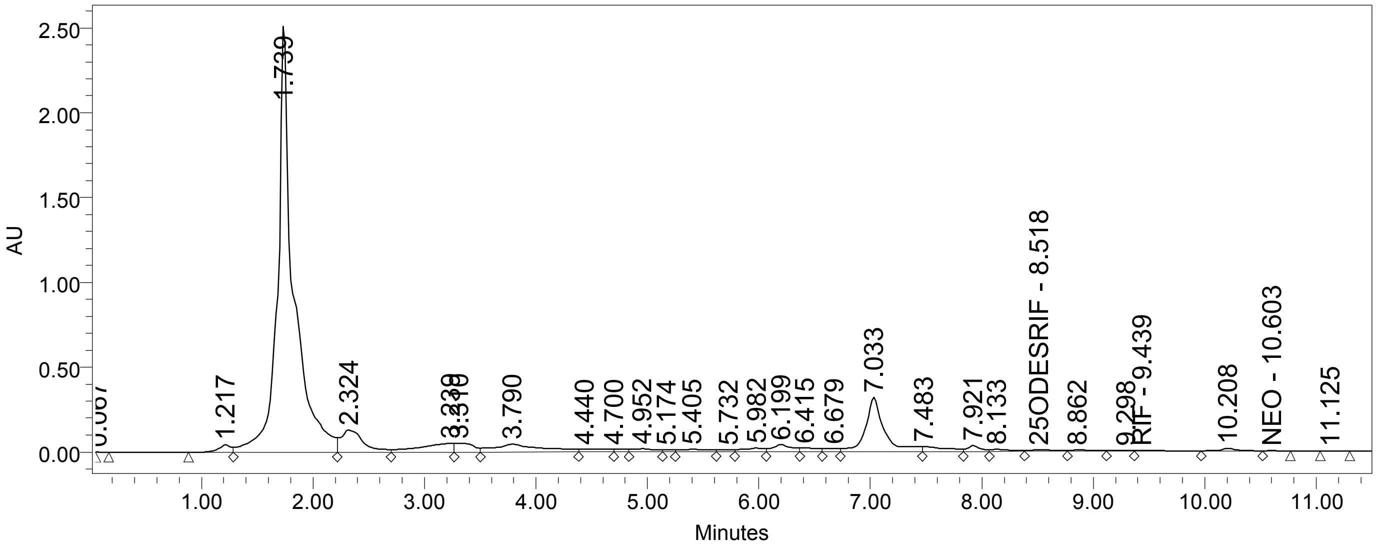

	Peak Name	RT	Area	% Area	Height
1		0.067	1981	0.01	764
2		1.217	359925	0.94	46002
3		1.739	25265653	66.18	2480162
4		2.324	1841245	4.82	132061
5		3.239	1078058	2.82	50780
6		3.310	602592	1.58	50880
7		3.790	1403567	3.68	46564
8		4.440	306454	0.80	17634
9		4.700	116758	0.31	15106
10		4.952	264737	0.69	17947
11		5.174	91372	0.24	13565
12		5.405	278266	0.73	14806

Reported by User: Saneesh Kumar (Saneesh) Project Name: HDI_Stellenbosch_2016

	Peak Name	RT	Area	% Area	Height
13		5.732	120086	0.31	12569
14		5.982	285076	0.75	21370
15		6.199	506411	1.33	42574
16		6.415	237368	0.62	22733
17		6.679	171208	0.45	17730
18		7.033	3710553	9.72	317595
19		7.483	430763	1.13	27170
20		7.921	272555	0.71	34343
21		8.133	166533	0.44	11998
22	25ODESRIF	8.518	165871	0.43	8783
23		8.862	128211	0.34	9409
24		9.298	75118	0.20	6702
25	RIF	9.439	98204	0.26	6051
26		10.208	181199	0.47	16848
27	NEO	10.603	14505	0.04	2119
28		11.125	4447	0.01	585

Multi Sample Summary

Reported by User: Saneesh (Saneesh)   Project Name: HDI_Stellenbosch_2016

SAMPLE INFORMATION

Sample Name:	RIF Control 1	Acquired By:	Saneesh
Sample Type:	Unknown	Date Acquired:	2016/09/10 12:47:10 AM
Vial:	24	Acq. Method Set:	HDI_RIF_25ODESRIF_NEO
Injection #:	1	Date Processed:	2016/09/10 01:28:32 PM
Injection Volume:	15.00 ul	Processing Method:	HDI_RIF_ W2996 254.0nm-1.2
Run Time:	11.5 Minutes	Channel Name:	W2996 254.0nm-1.2
Sample Set Name:	RIF_Basil AqMeOH1 IC50 10_9_16	Proc. Chnl. Descr.:	W2996 PDA 254.0 nm at 1.2

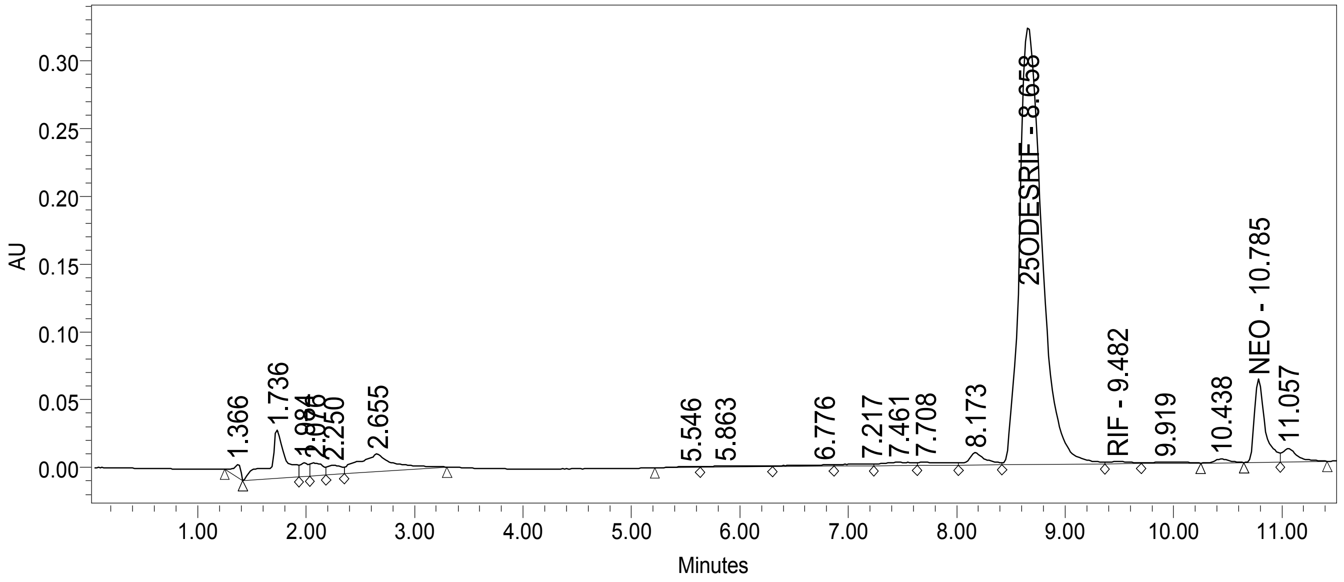

	Peak Name	RT	Area	% Area	Height
1		1.366	43239	0.67	9137
2		1.736	359290	5.58	35405
3		1.984	55110	0.86	10052
4		2.076	73168	1.14	9376
5		2.250	60099	0.93	6647
6		2.655	301732	4.69	12886
7		5.546	11393	0.18	741
8		5.863	30365	0.47	878
9		6.776	28978	0.45	1005
10		7.217	30031	0.47	1605
11		7.461	51263	0.80	2499
12		7.708	46577	0.72	2427
13		8.173	97710	1.52	9140
14	25ODESRIF	8.658	4665646	72.50	323632

Reported by User: Saneesh (Saneesh) Project Name: HDI_Stellenbosch_2016

	Peak Name	RT	Area	% Area	Height
15	RIF	9.482	24616	0.38	1661
16		9.919	21745	0.34	1025
17		10.438	24274	0.38	2757
18	NEO	10.785	415385	6.45	60790
19		11.057	94551	1.47	9800

Reported by User: Saneesh (Saneesh) Project Name: HDI_Stellenbosch_2016

SAMPLE INFORMATION

Sample Name:	RIF Inc Basil_Aq 1miG/mL 1	Acquired By:	Saneesh
Sample Type:	Unknown	Date Acquired:	2016/09/10 01:01:51 AM
Vial:	2	Acq. Method Set:	HDI_RIF_25ODESRIF_NEO
Injection #:	1	Date Processed:	2016/09/10 01:28:31 PM
Injection Volume:	15.00 ul	Processing Method:	HDI_RIF_ W2996 254.0nm-1.2
Run Time:	11.5 Minutes	Channel Name:	W2996 254.0nm-1.2
Sample Set Name:	RIF_Basil AqMeOH1 IC50 10_9_16	Proc. Chnl. Descr.:	W2996 PDA 254.0 nm at 1.2

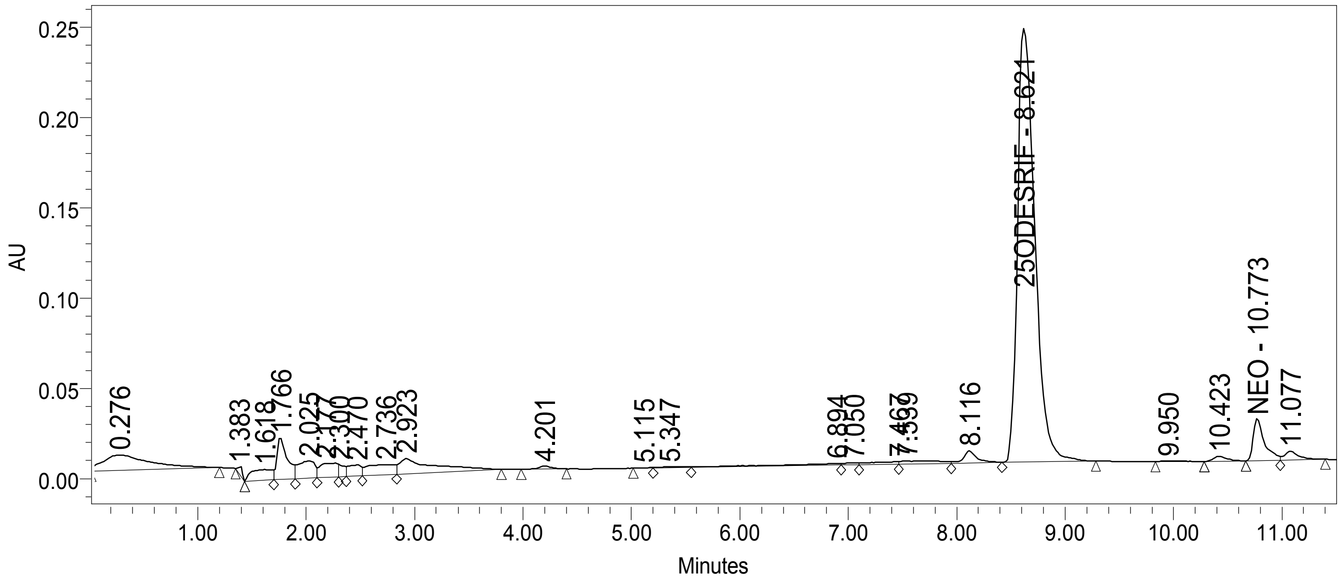

	Peak Name	RT	Area	% Area	Height
1		0.276	262171	6.32	8500
2		1.383	11857	0.29	3633
3		1.618	80918	1.95	5966
4		1.766	154049	3.72	22791
5		2.025	101990	2.46	9512
6		2.177	87327	2.11	7906
7		2.300	23974	0.58	6888
8		2.470	51201	1.23	6105
9		2.736	100961	2.43	5623
10		2.923	185504	4.47	8457
11		4.201	14099	0.34	1635
12		5.115	1312	0.03	178

Reported by User: Saneesh (Saneesh) Project Name: HDI_Stellenbosch_2016

	Peak Name	RT	Area	% Area	Height
13		5.347	6143	0.15	375
14		6.894	33868	0.82	884
15		7.050	9874	0.24	1033
16		7.467	24615	0.59	1334
17		7.539	38061	0.92	1574
18		8.116	57359	1.38	6616
19	25ODESRIF	8.621	2659214	64.13	241006
20	RIF	9.400			
21		9.950	5376	0.13	362
22		10.423	26124	0.63	2760
23	NEO	10.773	165125	3.98	23155
24		11.077	45294	1.09	4819

Reported by User: Saneesh (Saneesh) Project Name: HDI_Stellenbosch_2016

SAMPLE INFORMATION

Sample Name:	RIF Inc Basil_Aq 1miG/mL 2	Acquired By:	Saneesh
Sample Type:	Unknown	Date Acquired:	2016/09/10 01:16:36 AM
Vial:	3	Acq. Method Set:	HDI_RIF_25ODESRIF_NEO
Injection #:	1	Date Processed:	2016/09/10 01:28:31 PM
Injection Volume:	15.00 ul	Processing Method:	HDI_RIF_ W2996 254.0nm-1.2
Run Time:	11.5 Minutes	Channel Name:	W2996 254.0nm-1.2
Sample Set Name:	RIF_Basil AqMeOH1 IC50 10_9_16	Proc. Chnl. Descr.:	W2996 PDA 254.0 nm at 1.2

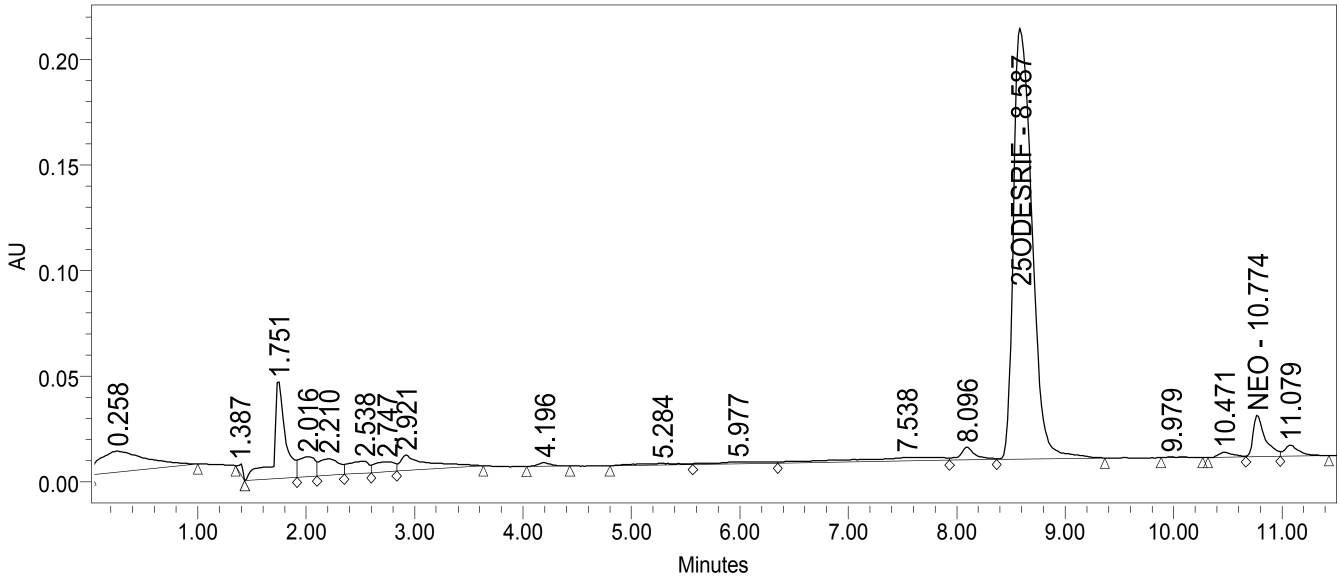

	Peak Name	RT	Area	% Area	Height
1		0.258	293782	7.36	9858
2		1.387	13137	0.33	4142
3		1.751	347945	8.72	46219
4		2.016	93825	2.35	9256
5		2.210	99185	2.48	7756
6		2.538	75093	1.88	5444
7		2.747	59220	1.48	4596
8		2.921	126881	3.18	7198
9		4.196	15180	0.38	1777
10		5.284	20960	0.53	676
11		5.977	28853	0.72	850
12		7.538	103159	2.58	1770

	Peak Name	RT	Area	% Area	Height
13		8.096	59108	1.48	6140
14	25ODESRIF	8.587	2426657	60.79	204552
15	RIF	9.400			
16		9.979	4047	0.10	303
17		10.471	24045	0.60	2423
18	NEO	10.774	150536	3.77	19746
19		11.079	50297	1.26	5134

S A M P L E   I N F O R M A T I O N

Sample Name:	RIF Inc Basil_Aq 10miG/mL	Acquired By:	Saneesh
Sample Type:	Unknown	Date Acquired:	2016/09/10 01:31:24 AM
Vial:	4	Acq. Method Set:	HDI_RIF_25ODESRIF_NEO
Injection #:	1	Date Processed:	2016/09/10 01:28:32 PM
Injection Volume:	15.00 ul	Processing Method:	HDI_RIF_ W2996 254.0nm-1.2
Run Time:	11.5 Minutes	Channel Name:	W2996 254.0nm-1.2
Sample Set Name:	RIF_Basil AqMeOH1 IC50 10_9_16	Proc. Chnl. Descr.:	W2996 PDA 254.0 nm at 1.2

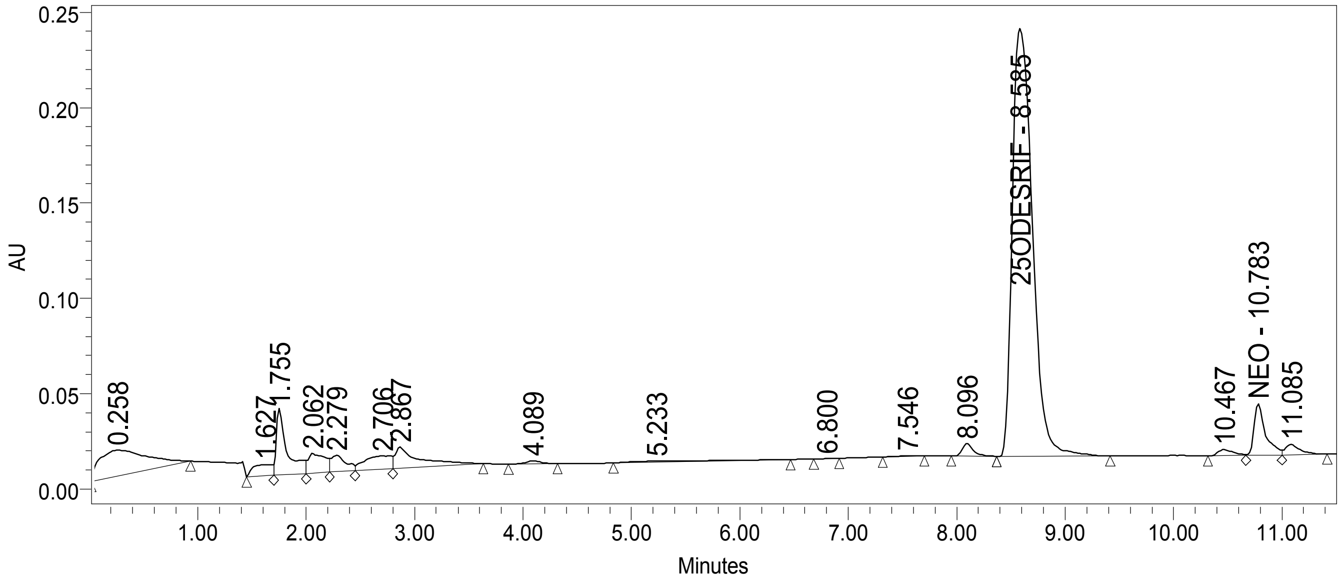

	Peak Name	RT	Area	% Area	Height
1		0.258	409208	9.18	13555
2		1.627	73161	1.64	5894
3		1.755	236281	5.30	35030
4		2.062	110549	2.48	10214
5		2.279	82482	1.85	8720
6		2.706	121324	2.72	6905
7		2.867	171062	3.84	10948
8		4.089	15440	0.35	1581
9		5.233	21617	0.49	519
10		6.800	787	0.02	122
11		7.546	4703	0.11	415
12		8.096	47768	1.07	6591

Reported by User: Saneesh (Saneesh) Project Name: HDI_Stellenbosch_2016

	Peak Name	RT	Area	% Area	Height
13	25ODESRIF	8.585	2882213	64.69	224652
14	RIF	9.400			
15		10.467	32036	0.72	3246
16	NEO	10.783	194273	4.36	26779
17		11.085	52279	1.17	5471

SAMPLE INFORMATION

Sample Name:	RIF Inc Basil_Aq 50miG/mL 1	Acquired By:	Saneesh
Sample Type:	Unknown	Date Acquired:	2016/09/10 01:46:10 AM
Vial:	5	Acq. Method Set:	HDI_RIF_25ODESRIF_NEO
Injection #:	1	Date Processed:	2016/09/10 01:28:31 PM
Injection Volume:	15.00 ul	Processing Method:	HDI_RIF_ W2996 254.0nm-1.2
Run Time:	11.5 Minutes	Channel Name:	W2996 254.0nm-1.2
Sample Set Name:	RIF_Basil AqMeOH1 IC50 10_9_16	Proc. Chnl. Descr.:	W2996 PDA 254.0 nm at 1.2

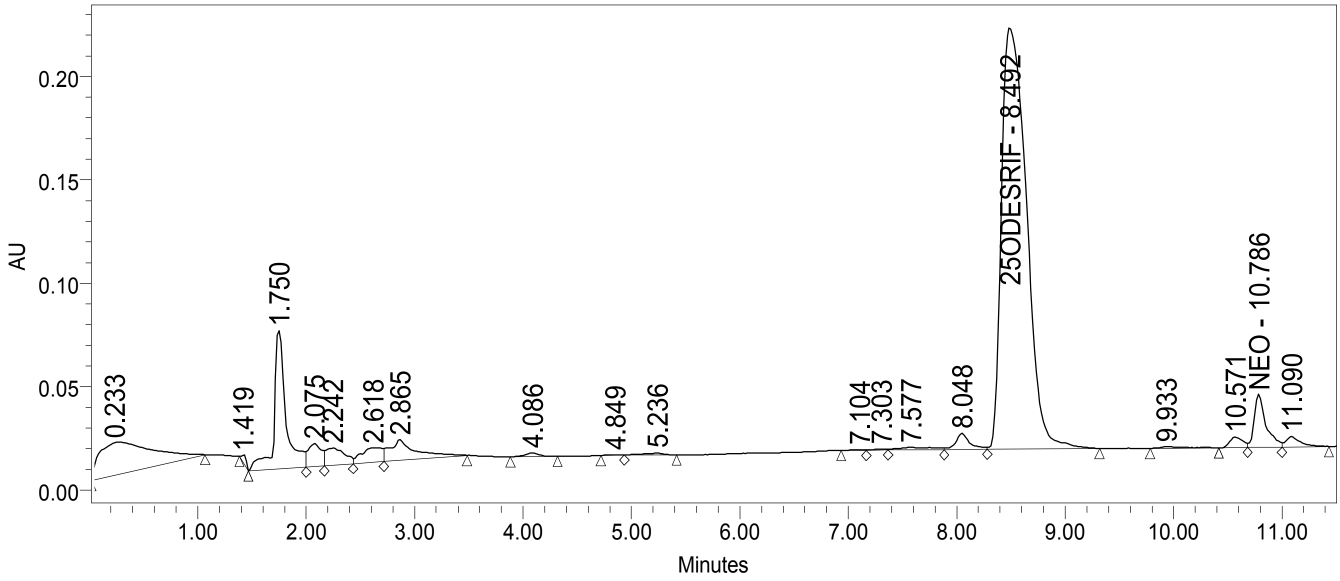

	Peak Name	RT	Area	% Area	Height
1		0.233	518642	10.05	15565
2		1.419	12694	0.25	3952
3		1.750	506066	9.80	67990
4		2.075	90687	1.76	10989
5		2.242	97943	1.90	8213
6		2.618	93311	1.81	6822
7		2.865	166622	3.23	9839
8		4.086	16477	0.32	1851
9		4.849	2923	0.06	334
10		5.236	14321	0.28	986
11		7.104	3202	0.06	342
12		7.303	6329	0.12	653

	Peak Name	RT	Area	% Area	Height
13		7.577	29355	0.57	1231
14		8.048	69693	1.35	7738
15	25ODESRIF	8.492	3243376	62.82	204441
16	RIF	9.400			
17		9.933	11644	0.23	657
18		10.571	43578	0.84	5128
19	NEO	10.786	189497	3.67	25477
20		11.090	46760	0.91	4904

Reported by User: Saneesh (Saneesh)

Project Name: HDI_Stellenbosch_2016

SAMPLE INFORMATION

Sample Name:	RIF Inc Basil_Aq 50miG/mL 2	Acquired By:	Saneesh
Sample Type:	Unknown	Date Acquired:	2016/09/10 02:00:49 AM
Vial:	6	Acq. Method Set:	HDI_RIF_25ODESRIF_NEO
Injection #:	1	Date Processed:	2016/09/10 01:28:31 PM
Injection Volume:	15.00 ul	Processing Method:	HDI_RIF_ W2996 254.0nm-1.2
Run Time:	11.5 Minutes	Channel Name:	W2996 254.0nm-1.2
Sample Set Name:	RIF_Basil AqMeOH1 IC50 10_9_16	Proc. Chnl. Descr.:	W2996 PDA 254.0 nm at 1.2

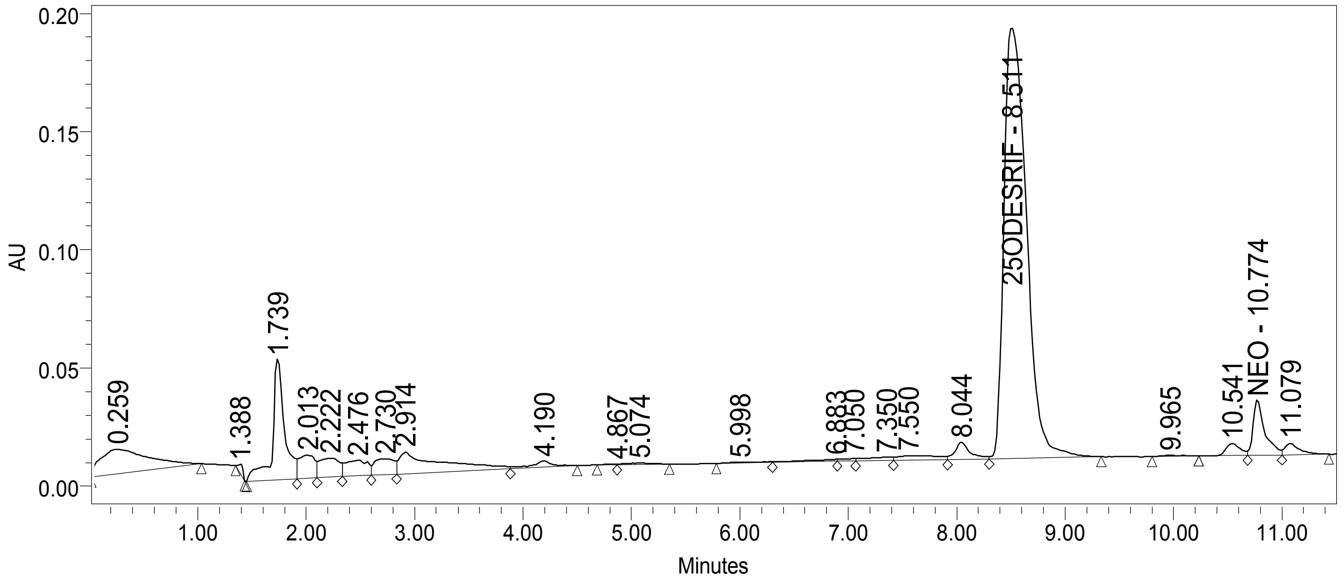

	Peak Name	RT	Area	% Area	Height
1		0.259	320806	7.20	10454
2		1.388	12837	0.29	4057
3		1.739	395175	8.87	52325
4		2.013	98623	2.21	9831
5		2.222	103015	2.31	8076
6		2.476	93906	2.11	6674
7		2.730	85543	1.92	6711
8		2.914	238524	5.35	9064
9		4.190	33112	0.74	2514
10		4.867	2004	0.04	255
11		5.074	11235	0.25	730
12		5.998	8020	0.18	339

Reported by User: Saneesh (Saneesh)   Project Name: HDI_Stellenbosch_2016

	Peak Name	RT	Area	% Area	Height
13		6.883	18300	0.41	894
14		7.050	9736	0.22	1106
15		7.350	25250	0.57	1375
16		7.550	46809	1.05	1833
17		8.044	67134	1.51	7018
18	25ODESRIF	8.511	2614200	58.65	182984
19	RIF	9.400			
20		9.965	7260	0.16	507
21		10.541	48419	1.09	5140
22	NEO	10.774	172341	3.87	23232
23		11.079	45116	1.01	4806

SAMPLE INFORMATION

Sample Name:	RIF Inc Basil_Aq 100miG/mL 1	Acquired By:	Saneesh
Sample Type:	Unknown	Date Acquired:	2016/09/10 02:15:36 AM
Vial:	7	Acq. Method Set:	HDI_RIF_25ODESRIF_NEO
Injection #:	1	Date Processed:	2016/09/10 01:28:32 PM
Injection Volume:	15.00 ul	Processing Method:	HDI_RIF_ W2996 254.0nm-1.2
Run Time:	11.5 Minutes	Channel Name:	W2996 254.0nm-1.2
Sample Set Name:	RIF_Basil AqMeOH1 IC50 10_9_16	Proc. Chnl. Descr.:	W2996 PDA 254.0 nm at 1.2

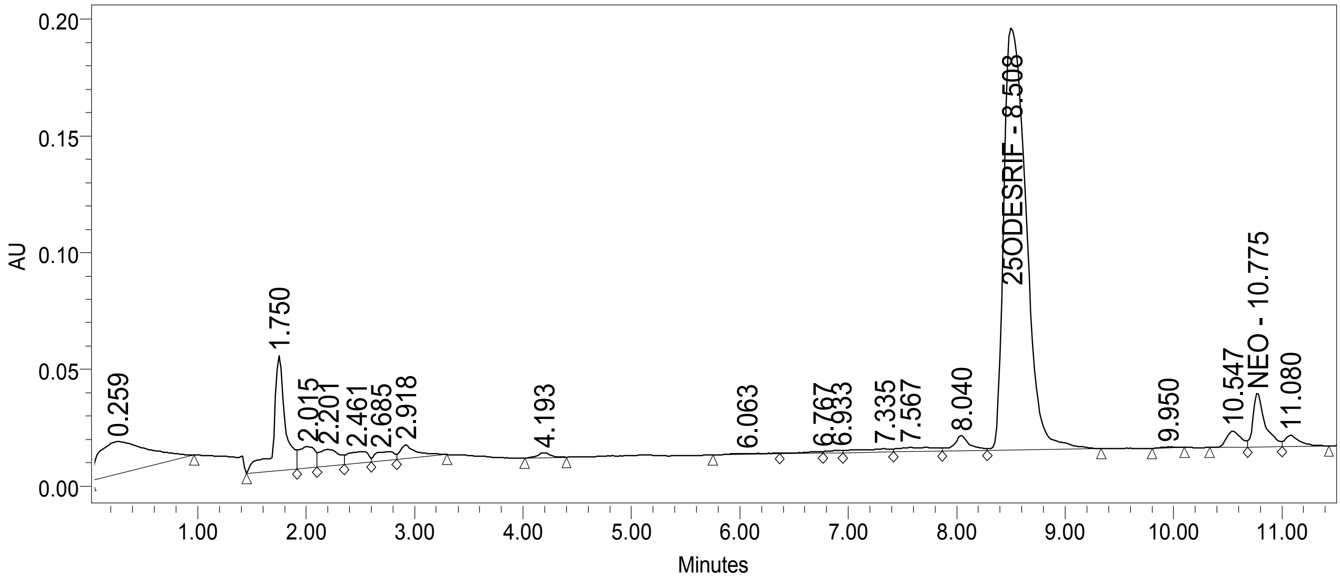

	Peak Name	RT	Area	% Area	Height
1		0.259	429951	10.23	13822
2		1.750	373538	8.88	48392
3		2.015	91517	2.18	9025
4		2.201	88919	2.11	7114
5		2.461	63777	1.52	4719
6		2.685	44122	1.05	3730
7		2.918	61562	1.46	5755
8		4.193	17122	0.41	2085
9		6.063	11314	0.27	450
10		6.767	10787	0.26	684
11		6.933	9762	0.23	979
12		7.335	33366	0.79	1410

Reported by User: Saneesh (Saneesh) Project Name: HDI_Stellenbosch_2016

	Peak Name	RT	Area	% Area	Height
13		7.567	42391	1.01	1791
14		8.040	65347	1.55	6450
15	25ODESRIF	8.508	2570697	61.14	181360
16	RIF	9.400			
17		9.950	3144	0.07	366
18		10.547	65117	1.55	6888
19	NEO	10.775	176246	4.19	23015
20		11.080	45876	1.09	4797

Reported by User: Saneesh (Saneesh)   Project Name: HDI_Stellenbosch_2016

SAMPLE INFORMATION

Sample Name:	RIF Inc Basil_Aq 100miG/mL 2	Acquired By:	Saneesh
Sample Type:	Unknown	Date Acquired:	2016/09/10 02:30:24 AM
Vial:	8	Acq. Method Set:	HDI_RIF_25ODESRIF_NEO
Injection #:	1	Date Processed:	2016/09/10 01:28:32 PM
Injection Volume:	15.00 ul	Processing Method:	HDI_RIF_ W2996 254.0nm-1.2
Run Time:	11.5 Minutes	Channel Name:	W2996 254.0nm-1.2
Sample Set Name:	RIF_Basil AqMeOH1 IC50 10_9_16	Proc. Chnl. Descr.:	W2996 PDA 254.0 nm at 1.2

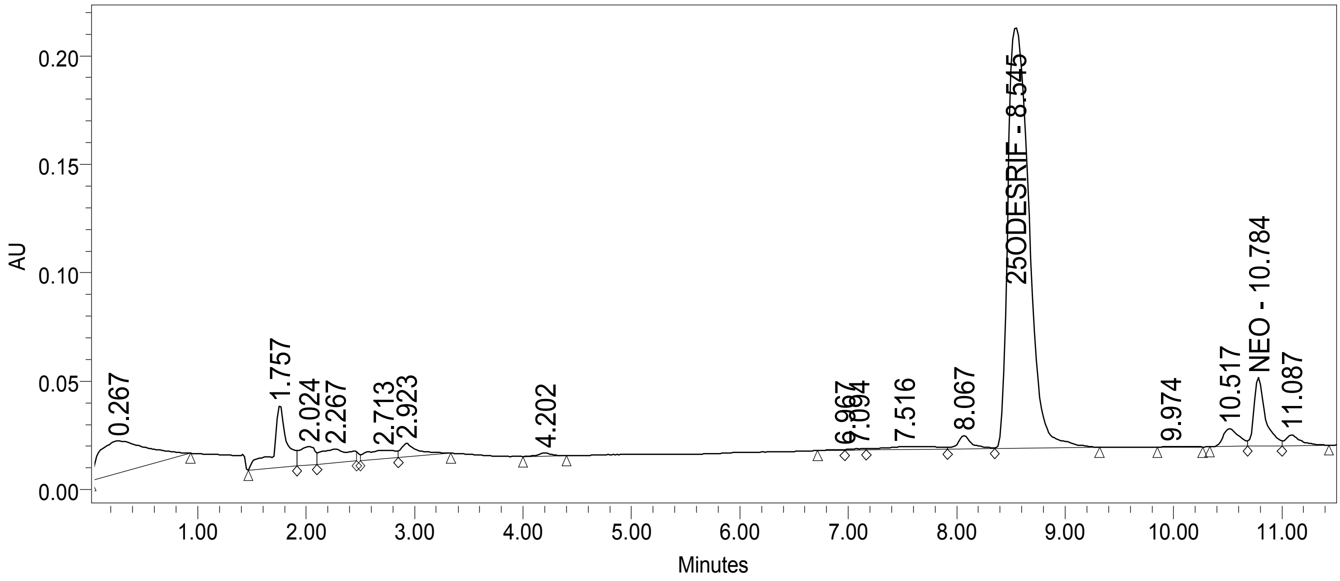

	Peak Name	RT	Area	% Area	Height
1		0.267	453039	10.90	14746
2		1.757	250167	6.02	28735
3		2.024	84234	2.03	8494
4		2.267	118080	2.84	6472
5		2.713	74249	1.79	3884
6		2.923	66444	1.60	6095
7		4.202	12155	0.29	1428
8		6.967	3987	0.10	443
9		7.094	7446	0.18	706
10		7.516	49616	1.19	1416
11		8.067	54644	1.32	6093
12	25ODESRIF	8.545	2614751	62.93	194076

Reported by User: Saneesh (Saneesh) Project Name: HDI_Stellenbosch_2016

	Peak Name	RT	Area	% Area	Height
13	RIF	9.400			
14		9.974	4718	0.11	321
15		10.517	85372	2.05	8296
16	NEO	10.784	228681	5.50	31520
17		11.087	47332	1.14	4970

Multi Sample Summary

Reported by User: Saneesh (Saneesh)   Project Name: HDI_Stellenbosch_2016

SAMPLE INFORMATION

Sample Name:	RIF Inc Basil_Aq 150miG/mL 1	Acquired By:	Saneesh
Sample Type:	Unknown	Date Acquired:	2016/09/10 02:45:13 AM
Vial:	9	Acq. Method Set:	HDI_RIF_25ODESRIF_NEO
Injection #:	1	Date Processed:	2016/09/10 01:28:32 PM
Injection Volume:	15.00 ul	Processing Method:	HDI_RIF_ W2996 254.0nm-1.2
Run Time:	11.5 Minutes	Channel Name:	W2996 254.0nm-1.2
Sample Set Name:	RIF_Basil AqMeOH1 IC50 10_9_16	Proc. Chnl. Descr.:	W2996 PDA 254.0 nm at 1.2

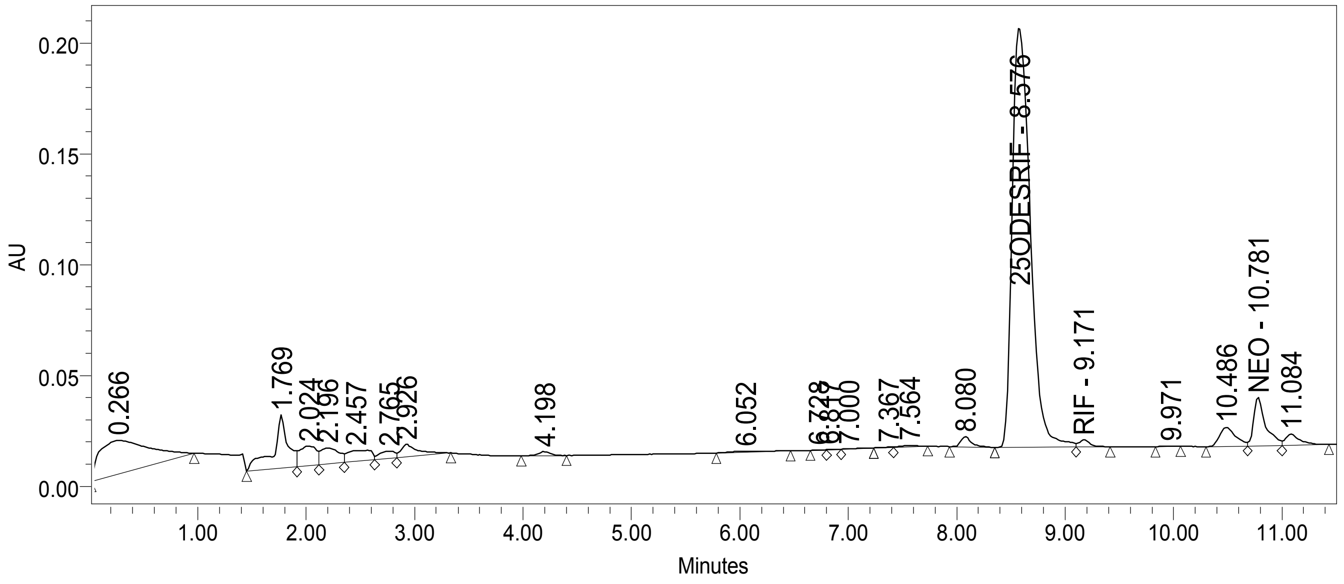

	Peak Name	RT	Area	% Area	Height
1		0.266	475048	13.06	15129
2		1.769	226922	6.24	23077
3		2.024	93880	2.58	8725
4		2.196	83685	2.30	7186
5		2.457	73756	2.03	4852
6		2.765	32503	0.89	3247
7		2.926	63391	1.74	5667
8		4.198	15390	0.42	1843
9		6.052	9885	0.27	452
10		6.728	484	0.01	62
11		6.817	1082	0.03	182
12		7.000	1861	0.05	158

Reported by User: Saneesh (Saneesh)   Project Name: HDI_Stellenbosch_2016

	Peak Name	RT	Area	% Area	Height
13		7.367	1546	0.04	184
14		7.564	6360	0.17	613
15		8.080	32196	0.89	4522
16	25ODESRIF	8.576	2193290	60.30	190928
17	RIF	9.171	23313	0.64	3202
18		9.971	2166	0.06	273
19		10.486	89533	2.46	8564
20	NEO	10.781	164553	4.52	21650
21		11.084	46569	1.28	4992

Reported by User: Saneesh (Saneesh) Project Name: HDI_Stellenbosch_2016

SAMPLE INFORMATION

Sample Name:	RIF Inc Basil_Aq 150mig/mL 2	Acquired By:	Saneesh
Sample Type:	Unknown	Date Acquired:	2016/09/10 02:59:59 AM
Vial:	10	Acq. Method Set:	HDI_RIF_25ODESRIF_NEO
Injection #:	1	Date Processed:	2016/09/10 01:28:32 PM
Injection Volume:	15.00 ul	Processing Method:	HDI_RIF_ W2996 254.0nm-1.2
Run Time:	11.5 Minutes	Channel Name:	W2996 254.0nm-1.2
Sample Set Name:	RIF_Basil AqMeOH1 IC50 10_9_16	Proc. Chnl. Descr.:	W2996 PDA 254.0 nm at 1.2

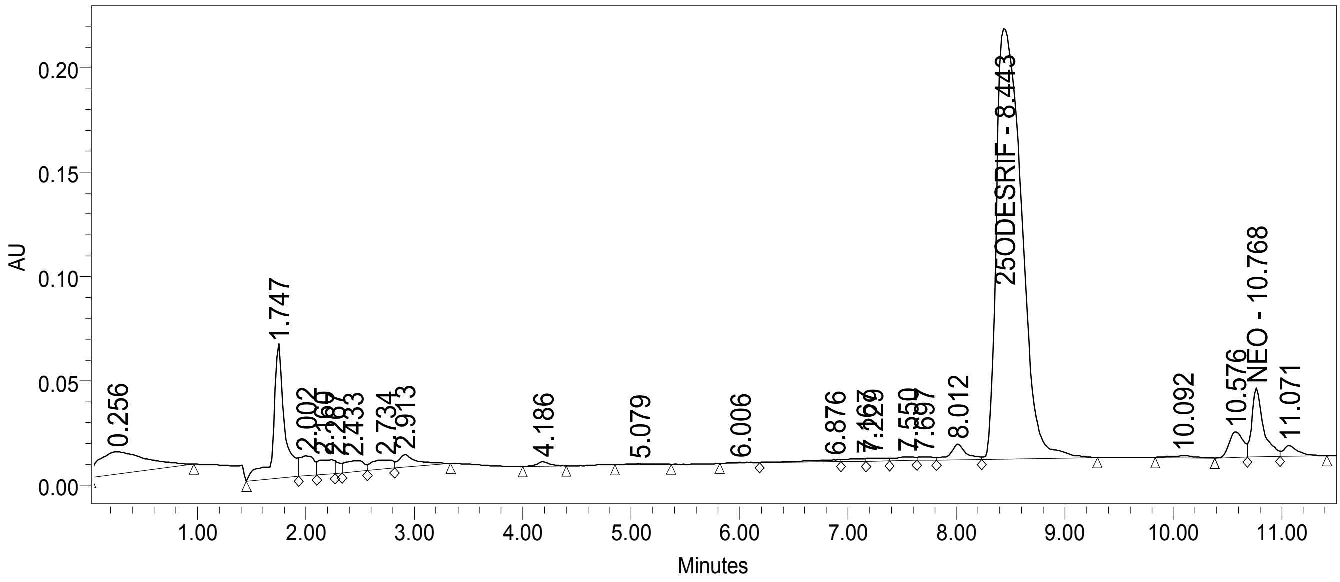

	Peak Name	RT	Area	% Area	Height
1		0.256	316722	6.23	10555
2		1.747	463012	9.10	62230
3		2.002	86127	1.69	9451
4		2.160	66432	1.31	6928
5		2.267	20077	0.39	6047
6		2.433	64546	1.27	5108
7		2.734	58772	1.16	4297
8		2.913	71140	1.40	6095
9		4.186	18015	0.35	2180
10		5.079	7189	0.14	447
11		6.006	5813	0.11	368
12		6.876	24788	0.49	967

	Peak Name	RT	Area	% Area	Height
13		7.167	16765	0.33	1351
14		7.229	17316	0.34	1442
15		7.550	23628	0.46	1877
16		7.697	17537	0.34	1788
17		8.012	77610	1.53	7664
18	25ODESRIF	8.443	3312537	65.13	207325
19	RIF	9.400			
20		10.092	13219	0.26	864
21		10.576	111613	2.19	12302
22	NEO	10.768	242490	4.77	32804
23		11.071	50313	0.99	5160

SAMPLE INFORMATION

Sample Name:	RIF Inc Basil_Aq 200mig/mL	Acquired By:	Saneesh
Sample Type:	Unknown	Date Acquired:	2016/09/10 03:14:37 AM
Vial:	11	Acq. Method Set:	HDI_RIF_25ODESRIF_NEO
Injection #:	1	Date Processed:	2016/09/10 01:28:31 PM
Injection Volume:	15.00 ul	Processing Method:	HDI_RIF_ W2996 254.0nm-1.2
Run Time:	11.5 Minutes	Channel Name:	W2996 254.0nm-1.2
Sample Set Name:	RIF_Basil AqMeOH1 IC50 10_9_16	Proc. Chnl. Descr.:	W2996 PDA 254.0 nm at 1.2

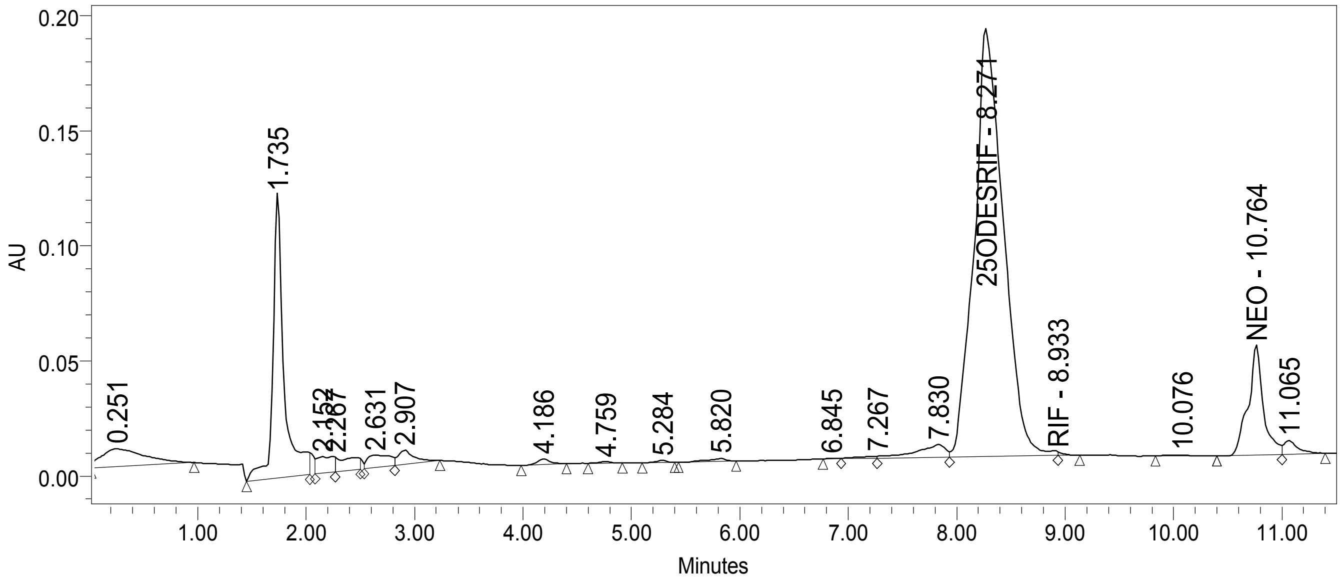

	Peak Name	RT	Area	% Area	Height
1		0.251	218942	3.95	7906
2		1.735	860262	15.53	121459
3		2.152	73514	1.33	7028
4		2.267	71652	1.29	6295
5		2.631	76611	1.38	5373
6		2.907	64515	1.16	6185
7		4.186	19320	0.35	2390
8		4.759	5208	0.09	642
9		5.284	5853	0.11	898
10		5.820	15311	0.28	1297
11		6.845	1994	0.04	279
12		7.267	9931	0.18	741

Reported by User: Saneesh (Saneesh) Project Name: HDI_Stellenbosch_2016

	Peak Name	RT	Area	% Area	Height
13		7.830	102093	1.84	5480
14	25ODESRIF	8.271	3489711	62.99	186351
15	RIF	8.933	4550	0.08	1442
16		10.076	8071	0.15	457
17	NEO	10.764	458876	8.28	47459
18		11.065	53763	0.97	5870

SAMPLE INFORMATION

Sample Name:	RIF Inc Basil_MeOH 1miG/mL 1	Acquired By:	Saneesh
Sample Type:	Unknown	Date Acquired:	2016/09/10 03:38:43 AM
Vial:	12	Acq. Method Set:	HDI_RIF_25ODESRIF_NEO
Injection #:	1	Date Processed:	2016/09/10 01:28:31 PM
Injection Volume:	15.00 ul	Processing Method:	HDI_RIF_ W2996 254.0nm-1.2
Run Time:	11.5 Minutes	Channel Name:	W2996 254.0nm-1.2
Sample Set Name:	RIF_Basil AqMeOH1 IC50 10_9_16	Proc. Chnl. Descr.:	W2996 PDA 254.0 nm at 1.2

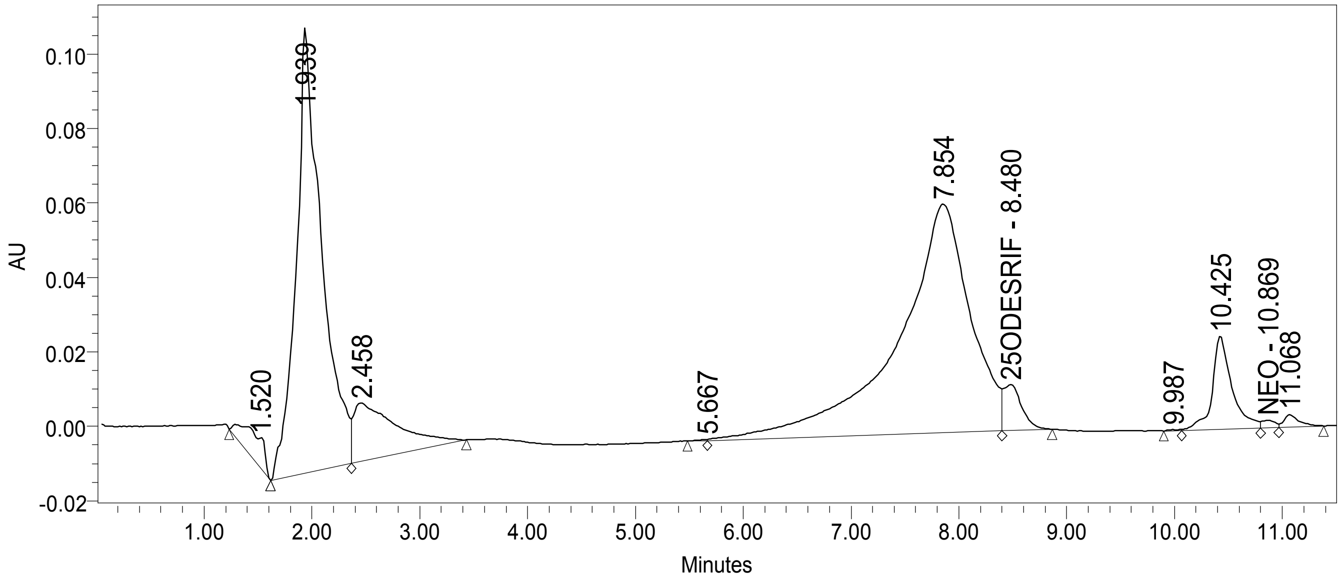

	Peak Name	RT	Area	% Area	Height
1		1.520	116121	1.97	8251
2		1.939	1972614	33.50	120088
3		2.458	431226	7.32	15736
4		5.667	2155	0.04	338
5		7.854	2850426	48.41	61535
6	25ODESRIF	8.480	145762	2.48	12286
7	RIF	9.400			
8		9.987	2506	0.04	308
9		10.425	317493	5.39	25164
10	NEO	10.869	17114	0.29	2076
11		11.068	33264	0.56	3404

SAMPLE INFORMATION

Sample Name:	RIF Inc Basil_MeOH 1miG/mL 2	Acquired By:	Saneesh
Sample Type:	Unknown	Date Acquired:	2016/09/10 03:53:40 AM
Vial:	13	Acq. Method Set:	HDI_RIF_25ODESRIF_NEO
Injection #:	1	Date Processed:	2016/09/10 01:28:31 PM
Injection Volume:	15.00 ul	Processing Method:	HDI_RIF_ W2996 254.0nm-1.2
Run Time:	11.5 Minutes	Channel Name:	W2996 254.0nm-1.2
Sample Set Name:	RIF_Basil AqMeOH1 IC50 10_9_16	Proc. Chnl. Descr.:	W2996 PDA 254.0 nm at 1.2

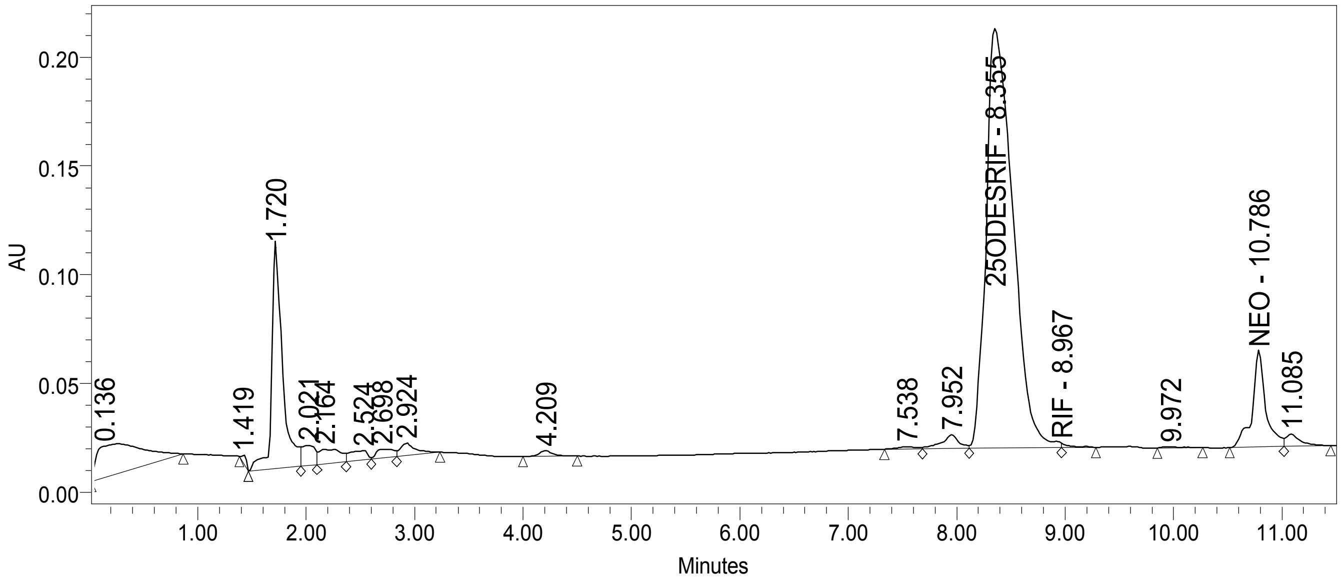

	Peak Name	RT	Area	% Area	Height
1		0.136	409725	7.72	14303
2		1.419	12471	0.23	3861
3		1.720	720390	13.57	102588
4		2.021	76687	1.44	9175
5		2.164	89915	1.69	6715
6		2.524	50519	0.95	4347
7		2.698	43928	0.83	4170
8		2.924	55883	1.05	5625
9		4.209	23977	0.45	2567
10		7.538	12357	0.23	1032
11		7.952	71008	1.34	6293
12	25ODESRIF	8.355	3294149	62.05	193316

Reported by User: Saneesh (Saneesh) Project Name: HDI_Stellenbosch_2016

	Peak Name	RT	Area	% Area	Height
13	RIF	8.967	10953	0.21	2046
14		9.972	7344	0.14	547
15	NEO	10.786	377154	7.10	44030
16		11.085	52432	0.99	5609

Reported by User: Saneesh (Saneesh) Project Name: HDI_Stellenbosch_2016

SAMPLE INFORMATION

Sample Name:	RIF Inc Basil_MeOH 10miG/mL	Acquired By:	Saneesh
Sample Type:	Unknown	Date Acquired:	2016/09/10 04:08:17 AM
Vial:	14	Acq. Method Set:	HDI_RIF_25ODESRIF_NEO
Injection #:	1	Date Processed:	2016/09/10 01:28:31 PM
Injection Volume:	15.00 ul	Processing Method:	HDI_RIF_ W2996 254.0nm-1.2
Run Time:	11.5 Minutes	Channel Name:	W2996 254.0nm-1.2
Sample Set Name:	RIF_Basil AqMeOH1 IC50 10_9_16	Proc. Chnl. Descr.:	W2996 PDA 254.0 nm at 1.2

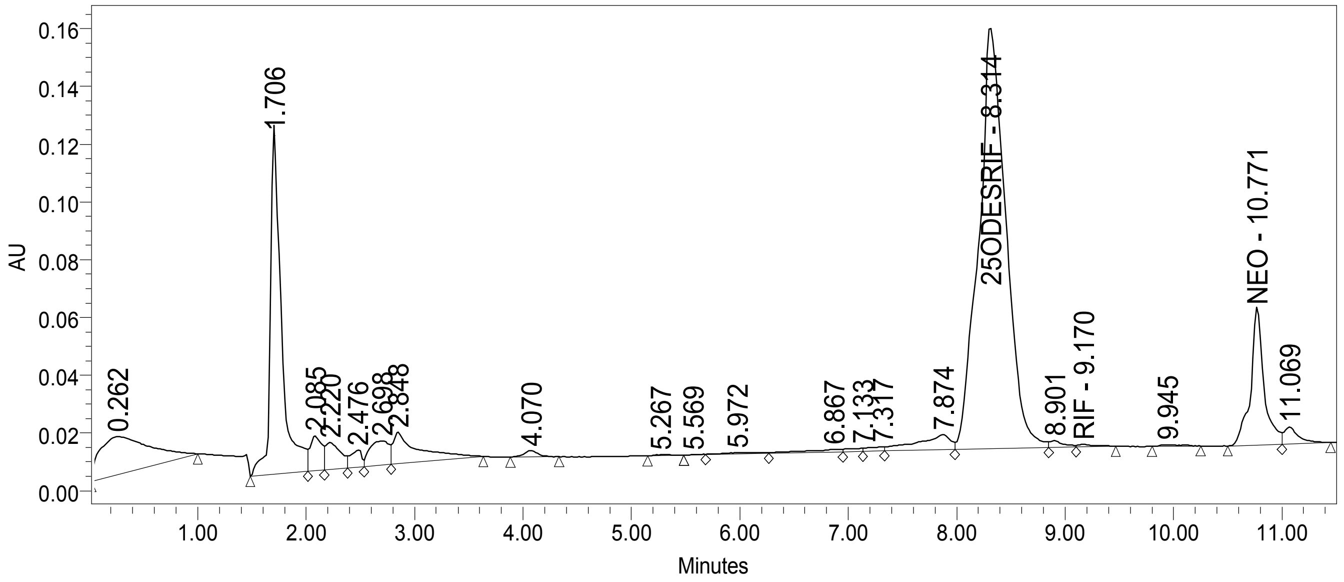

	Peak Name	RT	Area	% Area	Height
1		0.262	412467	8.23	13009
2		1.706	858861	17.14	119758
3		2.085	89247	1.78	12017
4		2.220	91173	1.82	9190
5		2.476	41365	0.83	6097
6		2.698	103660	2.07	8304
7		2.848	177631	3.55	10786
8		4.070	20616	0.41	2255
9		5.267	3117	0.06	302
10		5.569	1421	0.03	145
11		5.972	11338	0.23	434
12		6.867	21907	0.44	874

Reported by User: Saneesh (Saneesh)   Project Name: HDI_Stellenbosch_2016

	Peak Name	RT	Area	% Area	Height
13		7.133	10929	0.22	1080
14		7.317	15166	0.30	1376
15		7.874	112342	2.24	5243
16	25ODESRIF	8.314	2543090	50.77	145681
17		8.901	19643	0.39	2256
18	RIF	9.170	8625	0.17	827
19		9.945	8436	0.17	569
20	NEO	10.771	402134	8.03	47278
21		11.069	56248	1.12	5856

Reported by User: Saneesh (Saneesh) Project Name: HDI_Stellenbosch_2016

SAMPLE INFORMATION

Sample Name:	RIF Inc Basil_MeOh 50miG/mL 1	Acquired By:	Saneesh
Sample Type:	Unknown	Date Acquired:	2016/09/10 04:22:56 AM
Vial:	15	Acq. Method Set:	HDI_RIF_25ODESRIF_NEO
Injection #:	1	Date Processed:	2016/09/10 01:28:31 PM
Injection Volume:	15.00 ul	Processing Method:	HDI_RIF_ W2996 254.0nm-1.2
Run Time:	11.5 Minutes	Channel Name:	W2996 254.0nm-1.2
Sample Set Name:	RIF_Basil AqMeOH1 IC50 10_9_16	Proc. Chnl. Descr.:	W2996 PDA 254.0 nm at 1.2

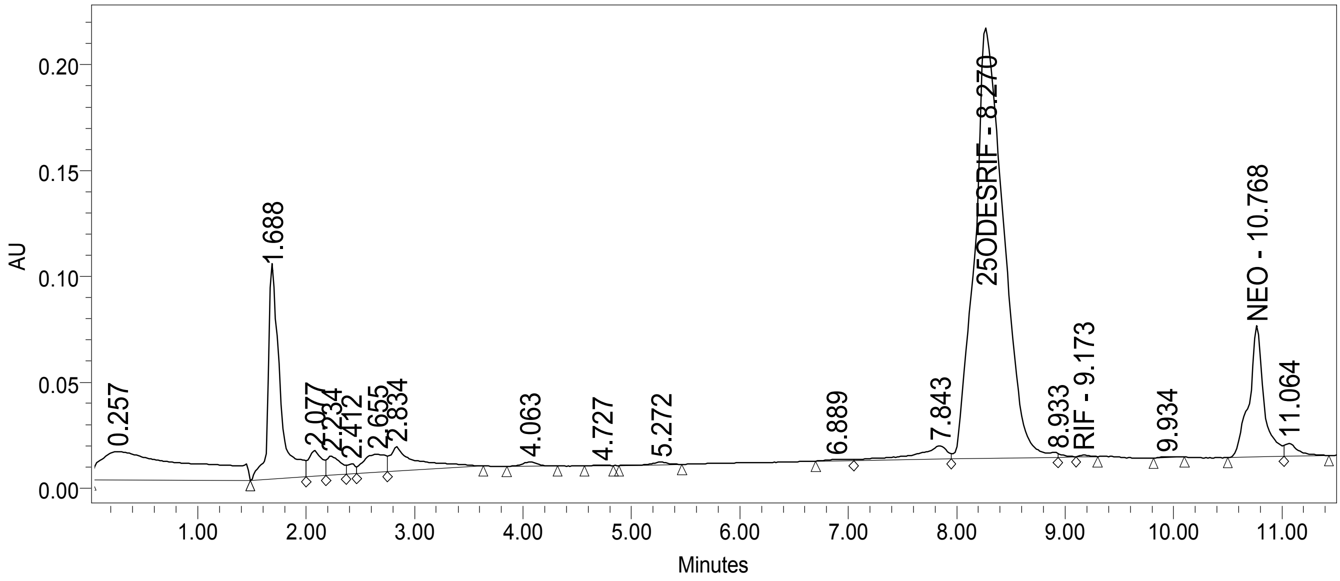

	Peak Name	RT	Area	% Area	Height
1		0.257	784355	11.78	13377
2		1.688	766815	11.52	101682
3		2.077	104060	1.56	12029
4		2.234	77261	1.16	8914
5		2.412	25415	0.38	4868
6		2.655	121563	1.83	8576
7		2.834	194007	2.91	11400
8		4.063	20010	0.30	2145
9		4.727	2807	0.04	301
10		5.272	13491	0.20	1317
11		6.889	8588	0.13	579
12		7.843	123080	1.85	6287

	Peak Name	RT	Area	% Area	Height
13	25ODESRIF	8.270	3760645	56.48	203694
14		8.933	6905	0.10	1889
15	RIF	9.173	4967	0.07	834
16		9.934	3284	0.05	385
17	NEO	10.768	585588	8.80	61573
18		11.064	54962	0.83	6186

SAMPLE INFORMATION

Sample Name:	RIF Inc Basil_MeOH 50miG/mL 2	Acquired By:	Saneesh
Sample Type:	Unknown	Date Acquired:	2016/09/10 04:37:34 AM
Vial:	16	Acq. Method Set:	HDI_RIF_25ODESRIF_NEO
Injection #:	1	Date Processed:	2016/09/10 01:28:31 PM
Injection Volume:	15.00 ul	Processing Method:	HDI_RIF_ W2996 254.0nm-1.2
Run Time:	11.5 Minutes	Channel Name:	W2996 254.0nm-1.2
Sample Set Name:	RIF_Basil AqMeOH1 IC50 10_9_16	Proc. Chnl. Descr.:	W2996 PDA 254.0 nm at 1.2

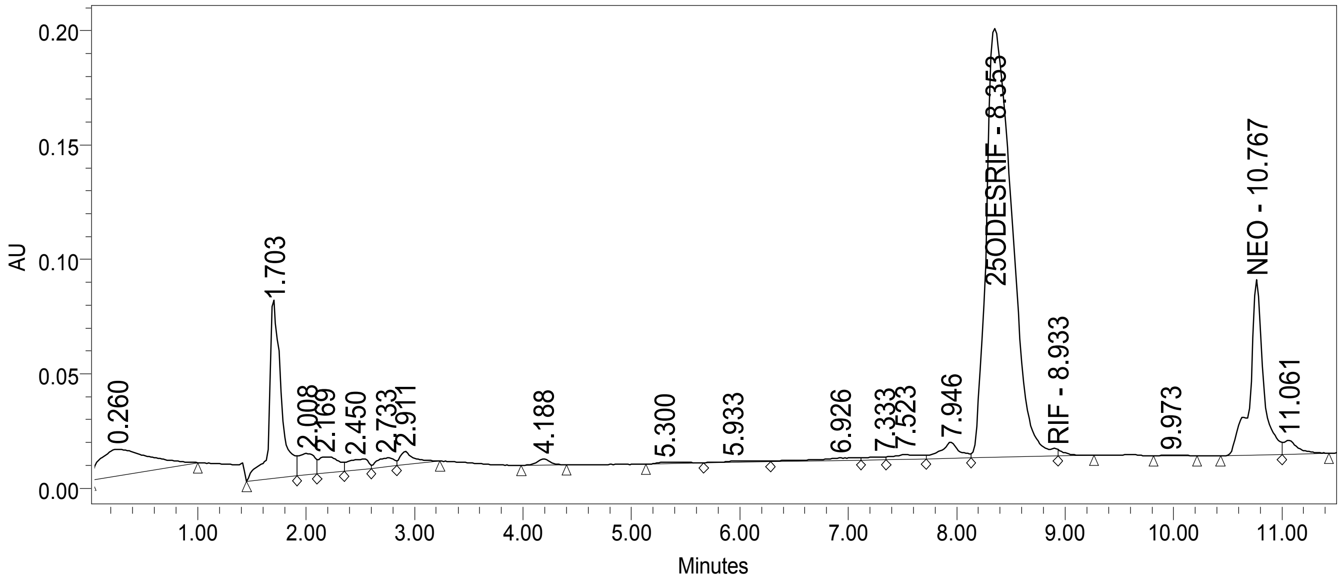

	Peak Name	RT	Area	% Area	Height
1		0.260	366980	6.86	11703
2		1.703	605308	11.31	78050
3		2.008	95225	1.78	9341
4		2.169	88812	1.66	7069
5		2.450	59740	1.12	4421
6		2.733	43298	0.81	3816
7		2.911	54078	1.01	5625
8		4.188	23033	0.43	2751
9		5.300	13461	0.25	664
10		5.933	14646	0.27	592
11		6.926	32607	0.61	1060
12		7.333	15108	0.28	1169

Reported by User: Saneesh (Saneesh) Project Name: HDI_Stellenbosch_2016

	Peak Name	RT	Area	% Area	Height
13		7.523	35386	0.66	2026
14		7.946	84880	1.59	6959
15	25ODESRIF	8.353	3078632	57.54	187779
16	RIF	8.933	14670	0.27	2812
17		9.973	5803	0.11	439
18	NEO	10.767	660466	12.34	75884
19		11.061	58685	1.10	6192

Reported by User: Saneesh (Saneesh)   Project Name: HDI_Stellenbosch_2016

SAMPLE INFORMATION

Sample Name:	RIF Inc Basil_MeOh 100miG/mL 1	Acquired By:	Saneesh
Sample Type:	Unknown	Date Acquired:	2016/09/10 04:52:11 AM
Vial:	17	Acq. Method Set:	HDI_RIF_25ODESRIF_NEO
Injection #:	1	Date Processed:	2016/09/10 01:28:31 PM
Injection Volume:	15.00 ul	Processing Method:	HDI_RIF_ W2996 254.0nm-1.2
Run Time:	11.5 Minutes	Channel Name:	W2996 254.0nm-1.2
Sample Set Name:	RIF_Basil AqMeOH1 IC50 10_9_16	Proc. Chnl. Descr.:	W2996 PDA 254.0 nm at 1.2

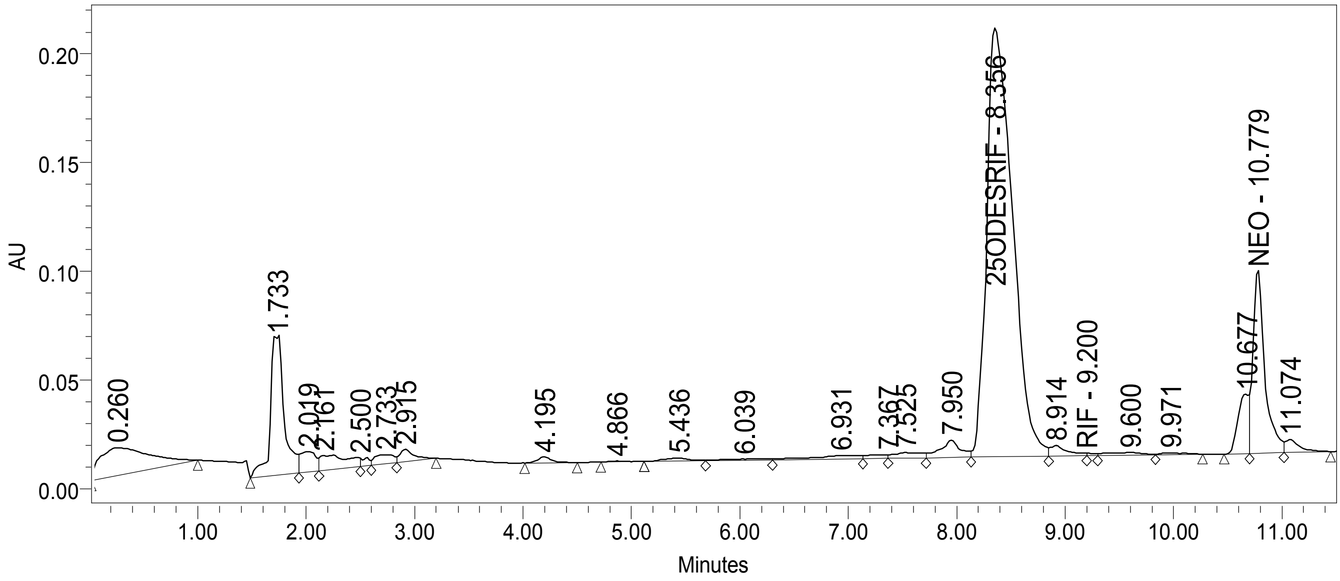

	Peak Name	RT	Area	% Area	Height
1		0.260	402550	6.91	12694
2		1.733	572396	9.83	65907
3		2.019	92370	1.59	9260
4		2.161	119455	2.05	6740
5		2.500	18431	0.32	3888
6		2.733	50906	0.87	4048
7		2.915	51554	0.89	5516
8		4.195	23749	0.41	2701
9		4.866	4003	0.07	377
10		5.436	23791	0.41	1491
11		6.039	19016	0.33	615
12		6.931	48476	0.83	1612

Reported by User: Saneesh (Saneesh) Project Name: HDI_Stellenbosch_2016

	Peak Name	RT	Area	% Area	Height
13		7.367	21182	0.36	1670
14		7.525	44610	0.77	2528
15		7.950	102167	1.75	7885
16	25ODESRIF	8.356	3257596	55.92	197840
17		8.914	53557	0.92	4716
18	RIF	9.200	5834	0.10	1119
19		9.600	27717	0.48	1169
20		9.971	10400	0.18	680
21		10.677	187985	3.23	27185
22	NEO	10.779	631801	10.85	83882
23		11.074	55479	0.95	5907

Empower software

Multi Sample Summary

Reported by User: Saneesh (Saneesh)   Project Name: HDI_Stellenbosch_2016

SAMPLE INFORMATION

Sample Name:	RIF Inc Basil_MeOh 100miG/mL 1	Acquired By:	Saneesh
Sample Type:	Unknown	Date Acquired:	2016/09/10 05:06:57 AM
Vial:	18	Acq. Method Set:	HDI_RIF_25ODESRIF_NEO
Injection #:	1	Date Processed:	2016/09/10 01:28:31 PM
Injection Volume:	15.00 ul	Processing Method:	HDI_RIF_ W2996 254.0nm-1.2
Run Time:	11.5 Minutes	Channel Name:	W2996 254.0nm-1.2
Sample Set Name:	RIF_Basil AqMeOH1 IC50 10_9_16	Proc. Chnl. Descr.:	W2996 PDA 254.0 nm at 1.2

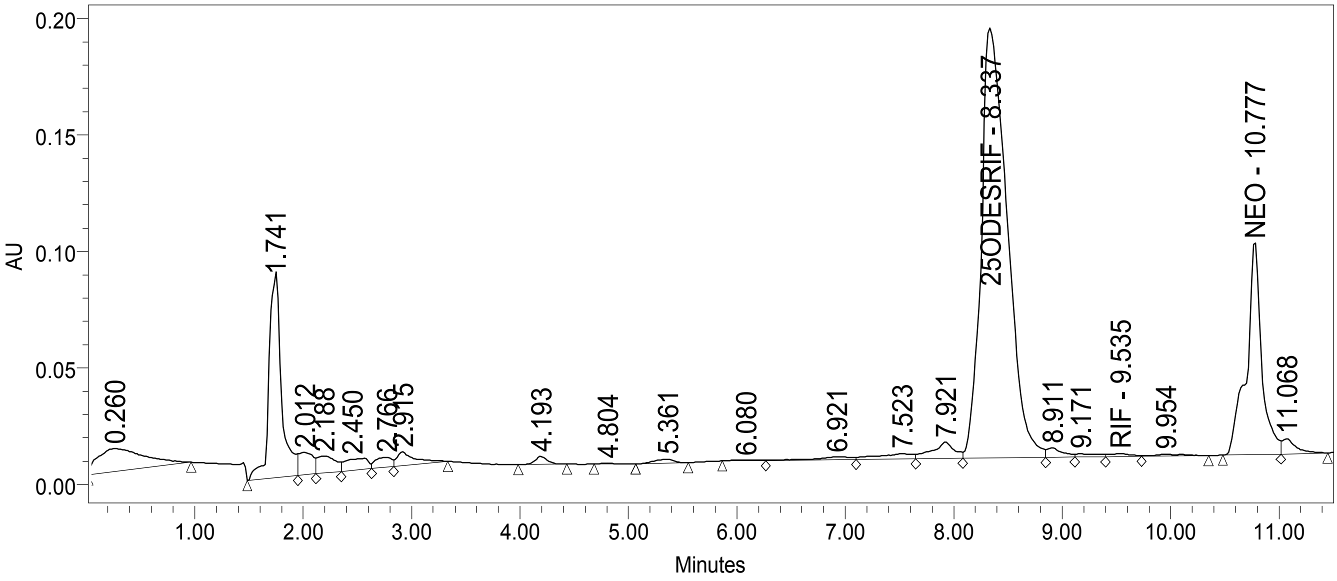

	Peak Name	RT	Area	% Area	Height
1		0.260	292440	5.13	9867
2		1.741	699927	12.29	88483
3		2.012	88382	1.55	9594
4		2.188	84796	1.49	7230
5		2.450	72040	1.26	4721
6		2.766	44095	0.77	4211
7		2.915	67146	1.18	5856
8		4.193	28167	0.49	3434
9		4.804	3256	0.06	398
10		5.361	20553	0.36	1629
11		6.080	4831	0.08	294
12		6.921	28795	0.51	1284

Reported by User: Saneesh (Saneesh) Project Name: HDI_Stellenbosch_2016

	Peak Name	RT	Area	% Area	Height
13		7.523	54011	0.95	2329
14		7.921	100098	1.76	7051
15	25ODESRIF	8.337	3075573	54.00	184811
16		8.911	40891	0.72	4102
17		9.171	20101	0.35	1453
18	RIF	9.535	17975	0.32	1354
19		9.954	14935	0.26	762
20	NEO	10.777	878084	15.42	91014
21		11.068	59834	1.05	6423

SAMPLE INFORMATION

Sample Name:	RIF Inc Basil_MeOH 150miG/mL 1	Acquired By:	Saneesh
Sample Type:	Unknown	Date Acquired:	2016/09/10 05:30:46 AM
Vial:	13	Acq. Method Set:	HDI_RIF_25ODESRIF_NEO
Injection #:	1	Date Processed:	2016/09/10 01:28:42 PM
Injection Volume:	15.00 ul	Processing Method:	HDI_RIF_ W2996 254.0nm-1.2
Run Time:	11.5 Minutes	Channel Name:	W2996 254.0nm-1.2
Sample Set Name:	RIF_Basil MeOHEOH2 IC50 10_916	Proc. Chnl. Descr.:	W2996 PDA 254.0 nm at 1.2

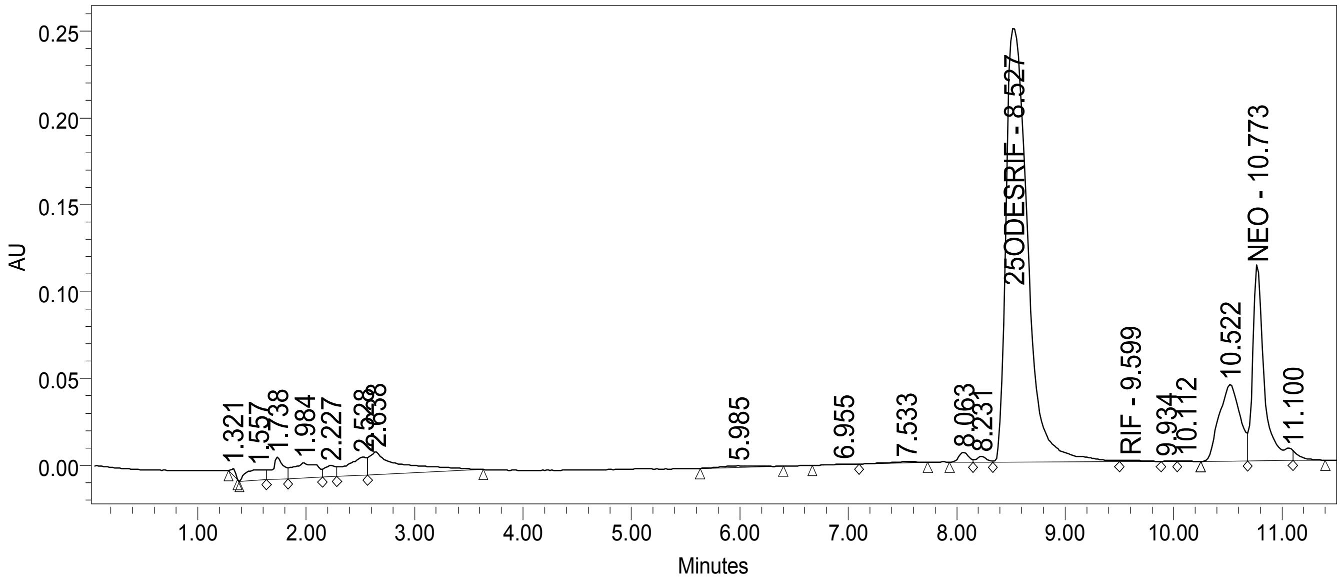

	Peak Name	RT	Area	% Area	Height
1		1.321	11272	0.19	3646
2		1.557	73412	1.27	5904
3		1.738	95247	1.65	12745
4		1.984	134815	2.33	8706
5		2.227	46207	0.80	6333
6		2.528	132235	2.29	10419
7		2.638	275238	4.76	12943
8		5.985	21672	0.37	966
9		6.955	4867	0.08	440
10		7.533	12071	0.21	613
11		8.063	34822	0.60	5613
12		8.231	21430	0.37	3245

	Peak Name	RT	Area	% Area	Height
13	25ODESRIF	8.527	3464400	59.87	251269
14	RIF	9.599	12275	0.21	883
15		9.934	2011	0.03	272
16		10.112	3092	0.05	417
17		10.522	593254	10.25	44230
18	NEO	10.773	823396	14.23	112745
19		11.100	25209	0.44	5811

SAMPLE INFORMATION

Sample Name:	RIF Inc Basil_MeOH 150miG/mL 2	Acquired By:	Saneesh
Sample Type:	Unknown	Date Acquired:	2016/09/10 05:45:32 AM
Vial:	14	Acq. Method Set:	HDI_RIF_25ODESRIF_NEO
Injection #:	1	Date Processed:	2016/09/10 01:28:42 PM
Injection Volume:	15.00 ul	Processing Method:	HDI_RIF_ W2996 254.0nm-1.2
Run Time:	11.5 Minutes	Channel Name:	W2996 254.0nm-1.2
Sample Set Name:	RIF_Basil MeOHEOH2 IC50 10_916	Proc. Chnl. Descr.:	W2996 PDA 254.0 nm at 1.2

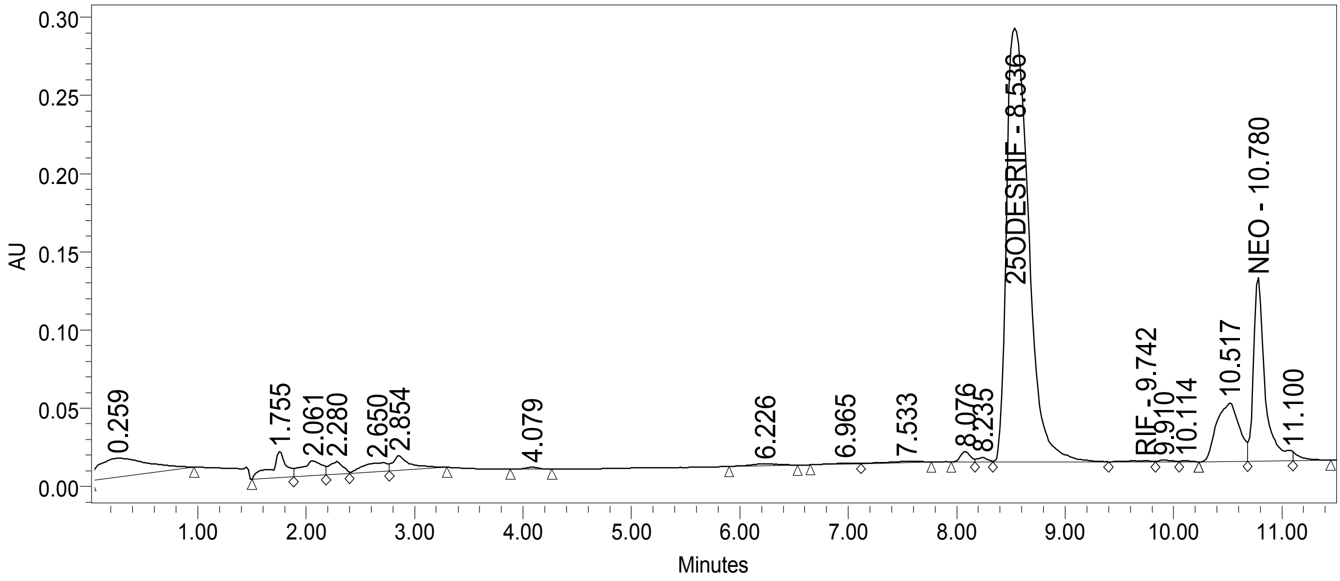

	Peak Name	RT	Area	% Area	Height
1		0.259	371135	5.80	11952
2		1.755	163369	2.55	16970
3		2.061	125157	1.96	9657
4		2.280	71018	1.11	8121
5		2.650	91923	1.44	5526
6		2.854	101932	1.59	9439
7		4.079	11503	0.18	1247
8		6.226	21487	0.34	1137
9		6.965	9136	0.14	703
10		7.533	14847	0.23	716
11		8.076	40090	0.63	6395
12		8.235	18580	0.29	2784

Reported by User: Saneesh (Saneesh) Project Name: HDI_Stellenbosch_2016

	Peak Name	RT	Area	% Area	Height
13	25ODESRIF	8.536	3911345	61.14	277553
14	RIF	9.742	8103	0.13	494
15		9.910	8322	0.13	1016
16		10.114	3354	0.05	496
17		10.517	546619	8.54	37338
18	NEO	10.780	852126	13.32	116553
19		11.100	27243	0.43	5636

Reported by User: Saneesh (Saneesh)

Project Name: HDI_Stellenbosch_2016

SAMPLE INFORMATION

Sample Name:	RIF Inc Basil_MeOH 200miG/mL	Acquired By:	Saneesh
Sample Type:	Unknown	Date Acquired:	2016/09/10 06:00:11 AM
Vial:	15	Acq. Method Set:	HDI_RIF_25ODESRIF_NEO
Injection #:	1	Date Processed:	2016/09/10 01:28:42 PM
Injection Volume:	15.00 ul	Processing Method:	HDI_RIF_ W2996 254.0nm-1.2
Run Time:	11.5 Minutes	Channel Name:	W2996 254.0nm-1.2
Sample Set Name:	RIF_Basil MeOHEOH2 IC50 10_916	Proc. Chnl. Descr.:	W2996 PDA 254.0 nm at 1.2

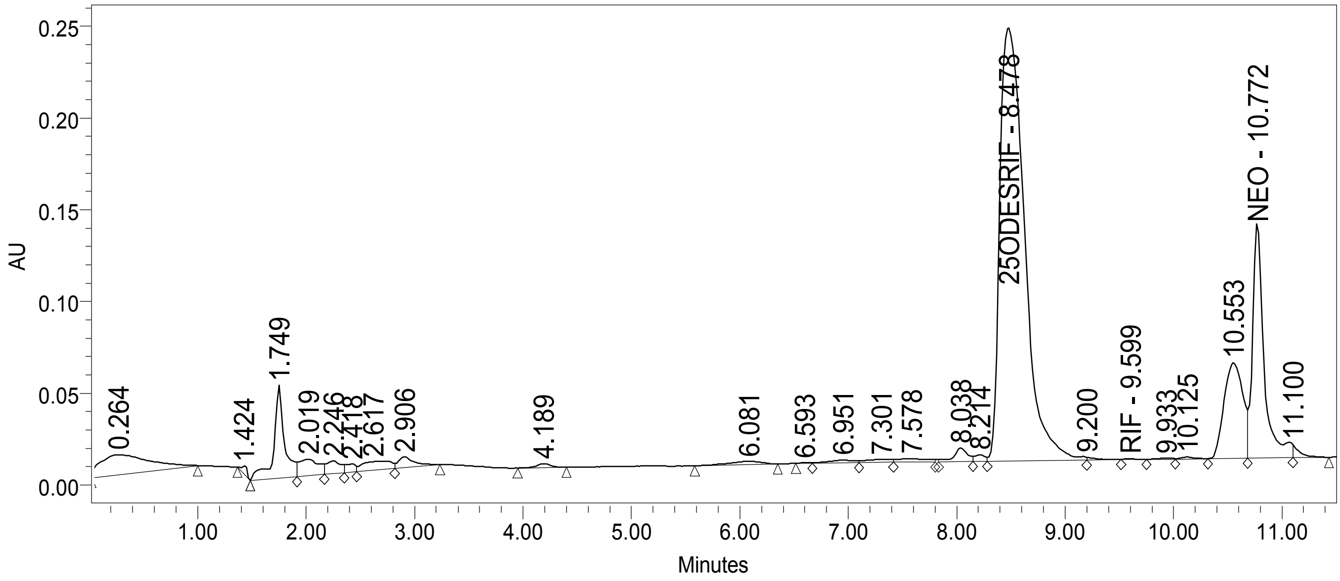

	Peak Name	RT	Area	% Area	Height
1		0.264	336575	5.10	10851
2		1.424	16542	0.25	4115
3		1.749	340719	5.16	47700
4		2.019	110856	1.68	8787
5		2.246	61990	0.94	6752
6		2.418	27732	0.42	4318
7		2.617	85087	1.29	4607
8		2.906	60530	0.92	5911
9		4.189	17839	0.27	2114
10		6.081	37459	0.57	1733
11		6.593	1532	0.02	244
12		6.951	23281	0.35	1500

Reported by User: Saneesh (Saneesh) Project Name: HDI_Stellenbosch_2016

	Peak Name	RT	Area	% Area	Height
13		7.301	24959	0.38	1509
14		7.578	36787	0.56	1937
15		8.038	62976	0.95	7382
16		8.214	23319	0.35	3501
17	25ODESRIF	8.478	3715617	56.25	236193
18		9.200	10716	0.16	1522
19	RIF	9.599	3928	0.06	530
20		9.933	5567	0.08	524
21		10.125	8917	0.13	1104
22		10.553	632708	9.58	52193
23	NEO	10.772	927751	14.05	127411
24		11.100	32064	0.49	7155

SAMPLE INFORMATION

Sample Name:	RIF Inc Basil_EtOH 1miG/mL 1	Acquired By:	Saneesh
Sample Type:	Unknown	Date Acquired:	2016/09/10 06:14:49 AM
Vial:	16	Acq. Method Set:	HDI_RIF_25ODESRIF_NEO
Injection #:	1	Date Processed:	2016/09/10 01:28:42 PM
Injection Volume:	15.00 ul	Processing Method:	HDI_RIF_ W2996 254.0nm-1.2
Run Time:	11.5 Minutes	Channel Name:	W2996 254.0nm-1.2
Sample Set Name:	RIF_Basil MeOHEOH2 IC50 10_916	Proc. Chnl. Descr.:	W2996 PDA 254.0 nm at 1.2

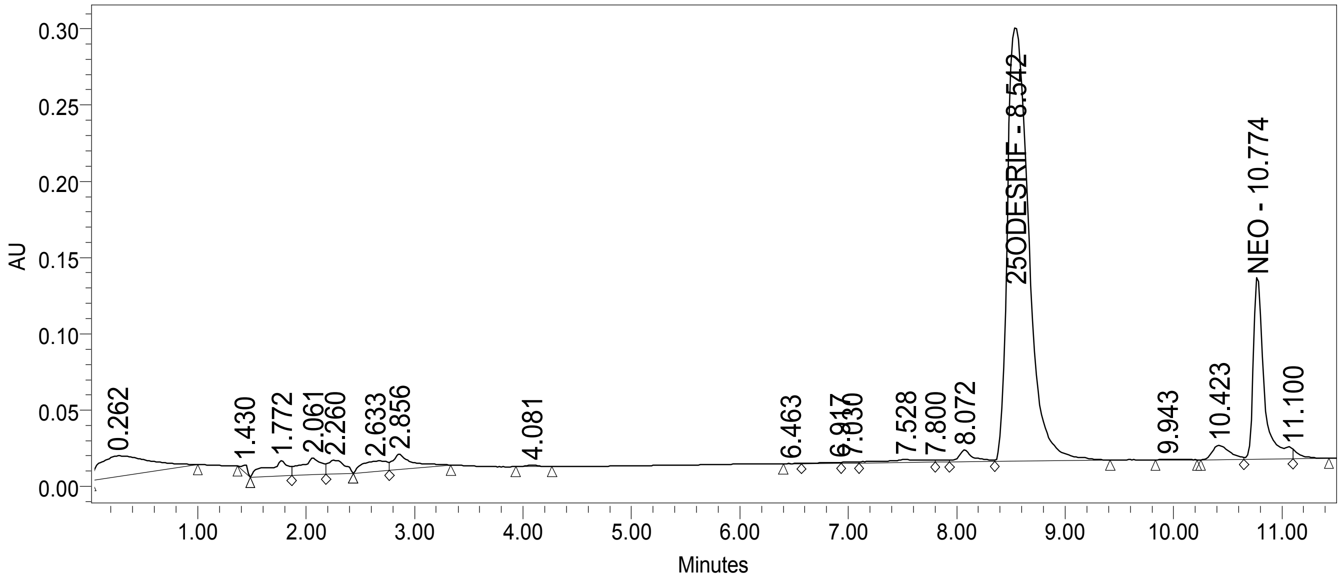

	Peak Name	RT	Area	% Area	Height
1		0.262	430446	7.12	13441
2		1.430	18184	0.30	4644
3		1.772	140855	2.33	10107
4		2.061	147881	2.45	11011
5		2.260	95681	1.58	8952
6		2.633	110173	1.82	6663
7		2.856	124120	2.05	10102
8		4.081	8764	0.15	1032
9		6.463	1257	0.02	158
10		6.917	8036	0.13	545
11		7.030	7063	0.12	765
12		7.528	50226	0.83	1725

Reported by User: Saneesh (Saneesh) Project Name: HDI_Stellenbosch_2016

	Peak Name	RT	Area	% Area	Height
13		7.800	9098	0.15	1255
14		8.072	69502	1.15	7563
15	25ODESRIF	8.542	3860864	63.90	285255
16	RIF	9.400			
17		9.943	6739	0.11	450
18		10.423	106915	1.77	9244
19	NEO	10.774	817314	13.53	119544
20		11.100	28651	0.47	6327

Reported by User: Saneesh (Saneesh) Project Name: HDI_Stellenbosch_2016

SAMPLE INFORMATION

Sample Name:	RIF Inc Basil_EtOH 1miG/mL 2	Acquired By:	Saneesh
Sample Type:	Unknown	Date Acquired:	2016/09/10 06:29:25 AM
Vial:	17	Acq. Method Set:	HDI_RIF_25ODESRIF_NEO
Injection #:	1	Date Processed:	2016/09/10 01:28:42 PM
Injection Volume:	15.00 ul	Processing Method:	HDI_RIF_ W2996 254.0nm-1.2
Run Time:	11.5 Minutes	Channel Name:	W2996 254.0nm-1.2
Sample Set Name:	RIF_Basil MeOHEOH2 IC50 10_916	Proc. Chnl. Descr.:	W2996 PDA 254.0 nm at 1.2

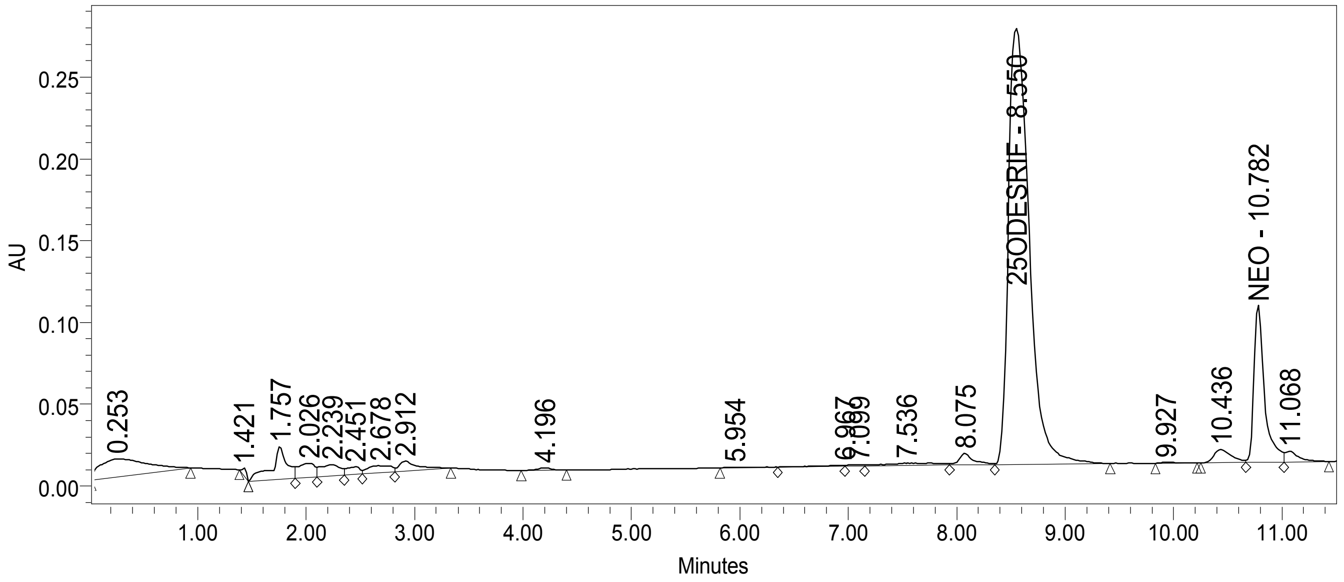

	Peak Name	RT	Area	% Area	Height
1		0.253	332944	6.19	11208
2		1.421	13870	0.26	4432
3		1.757	197520	3.67	19952
4		2.026	91076	1.69	8481
5		2.239	86503	1.61	6755
6		2.451	39110	0.73	4525
7		2.678	63634	1.18	4160
8		2.912	72363	1.35	6085
9		4.196	11778	0.22	1431
10		5.954	5685	0.11	279
11		6.967	11612	0.22	637
12		7.099	8647	0.16	809

Reported by User: Saneesh (Saneesh) Project Name: HDI_Stellenbosch_2016

	Peak Name	RT	Area	% Area	Height
13		7.536	55827	1.04	1552
14		8.075	65876	1.22	7091
15	25ODESRIF	8.550	3526979	65.56	266476
16	RIF	9.400			
17		9.927	6604	0.12	587
18		10.436	91929	1.71	8045
19	NEO	10.782	640509	11.91	95368
20		11.068	57388	1.07	6417

SAMPLE INFORMATION

Sample Name:	RIF Inc Basil_EtOH 10miG/mL 1	Acquired By:	Saneesh
Sample Type:	Unknown	Date Acquired:	2016/09/10 06:44:04 AM
Vial:	18	Acq. Method Set:	HDI_RIF_25ODESRIF_NEO
Injection #:	1	Date Processed:	2016/09/10 01:28:42 PM
Injection Volume:	15.00 ul	Processing Method:	HDI_RIF_ W2996 254.0nm-1.2
Run Time:	11.5 Minutes	Channel Name:	W2996 254.0nm-1.2
Sample Set Name:	RIF_Basil MeOHEOH2 IC50 10_916	Proc. Chnl. Descr.:	W2996 PDA 254.0 nm at 1.2

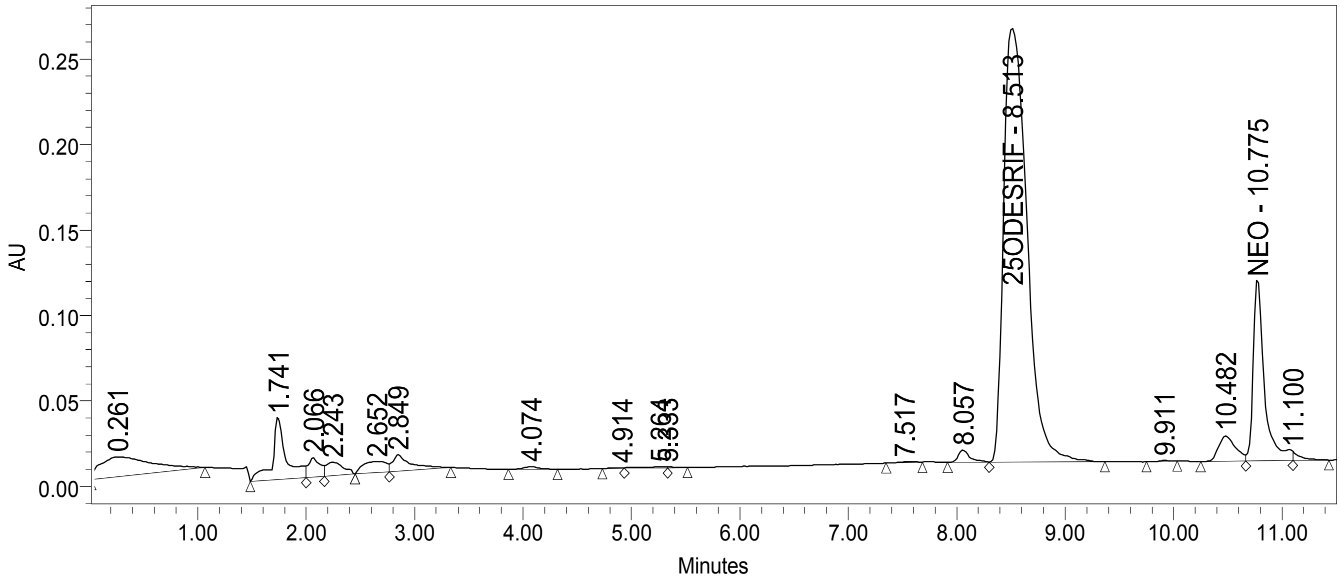

	Peak Name	RT	Area	% Area	Height
1		0.261	375910	6.39	11577
2		1.741	316838	5.38	36618
3		2.066	82640	1.40	11177
4		2.243	85238	1.45	7813
5		2.652	97579	1.66	6292
6		2.849	110256	1.87	9496
7		4.074	14957	0.25	1577
8		4.914	2358	0.04	281
9		5.264	10007	0.17	545
10		5.333	2063	0.04	379
11		7.517	4741	0.08	479
12		8.057	51615	0.88	7071

Reported by User: Saneesh (Saneesh) Project Name: HDI_Stellenbosch_2016

	Peak Name	RT	Area	% Area	Height
13	25ODESRIF	8.513	3787613	64.34	253875
14	RIF	9.400			
15		9.911	4056	0.07	598
16		10.482	164174	2.79	14848
17	NEO	10.775	751918	12.77	106559
18		11.100	25260	0.43	5246

SAMPLE INFORMATION

Sample Name:	RIF Inc Basil_EtOH 50miG/mL 1	Acquired By:	Saneesh
Sample Type:	Unknown	Date Acquired:	2016/09/10 06:58:41 AM
Vial:	19	Acq. Method Set:	HDI_RIF_25ODESRIF_NEO
Injection #:	1	Date Processed:	2016/09/10 01:28:42 PM
Injection Volume:	15.00 ul	Processing Method:	HDI_RIF_ W2996 254.0nm-1.2
Run Time:	11.5 Minutes	Channel Name:	W2996 254.0nm-1.2
Sample Set Name:	RIF_Basil MeOHEOH2 IC50 10_916	Proc. Chnl. Descr.:	W2996 PDA 254.0 nm at 1.2

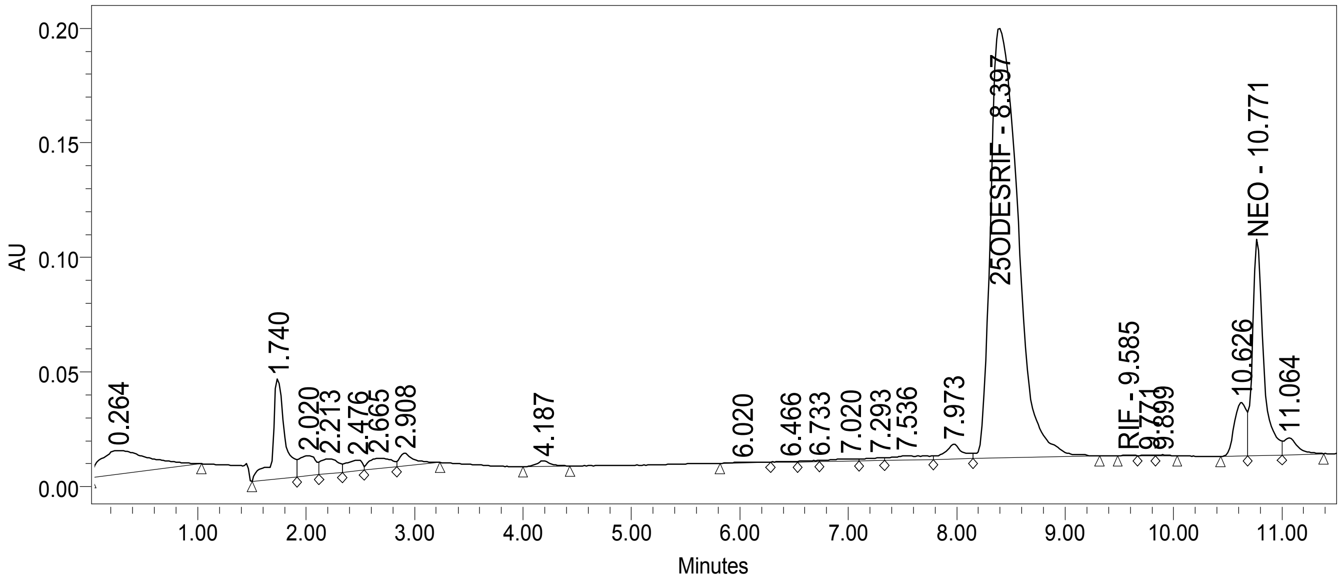

	Peak Name	RT	Area	% Area	Height
1		0.264	327512	6.10	10480
2		1.740	343777	6.40	44556
3		2.020	93353	1.74	8668
4		2.213	72521	1.35	6434
5		2.476	47913	0.89	4745
6		2.665	65000	1.21	4460
7		2.908	52361	0.98	5499
8		4.187	19862	0.37	2331
9		6.020	6914	0.13	337
10		6.466	3527	0.07	294
11		6.733	4209	0.08	431
12		7.020	16367	0.30	879

	Peak Name	RT	Area	% Area	Height
13		7.293	13406	0.25	1077
14		7.536	38890	0.72	1863
15		7.973	72218	1.35	6397
16	25ODESRIF	8.397	3272276	60.96	187607
17	RIF	9.585	2302	0.04	329
18		9.771	2750	0.05	379
19		9.899	3464	0.06	465
20		10.626	179374	3.34	23513
21	NEO	10.771	662609	12.34	93908
22		11.064	66932	1.25	7336

SAMPLE INFORMATION

Sample Name:	RIF Inc Basil_EtOH 50miG/mL 2	Acquired By:	Saneesh
Sample Type:	Unknown	Date Acquired:	2016/09/10 07:13:20 AM
Vial:	20	Acq. Method Set:	HDI_RIF_25ODESRIF_NEO
Injection #:	1	Date Processed:	2016/09/10 01:28:42 PM
Injection Volume:	15.00 ul	Processing Method:	HDI_RIF_ W2996 254.0nm-1.2
Run Time:	11.5 Minutes	Channel Name:	W2996 254.0nm-1.2
Sample Set Name:	RIF_Basil MeOHEOH2 IC50 10_916	Proc. Chnl. Descr.:	W2996 PDA 254.0 nm at 1.2

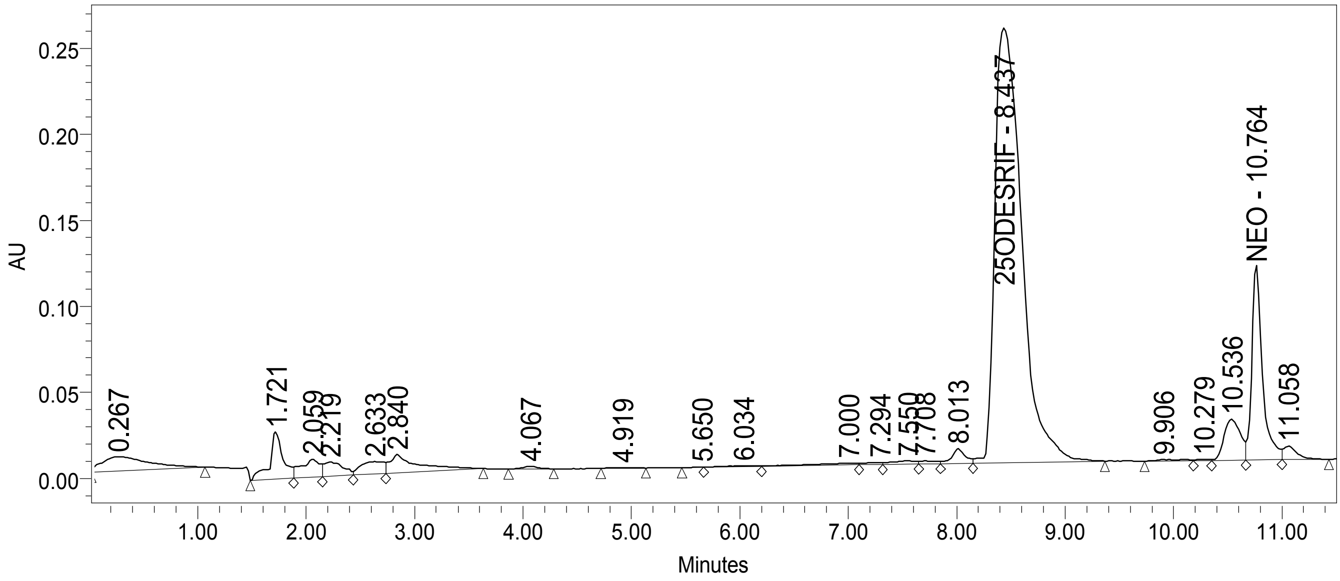

	Peak Name	RT	Area	% Area	Height
1		0.267	249358	3.76	8244
2		1.721	234666	3.54	27926
3		2.059	125172	1.89	10561
4		2.219	101132	1.52	8233
5		2.633	107536	1.62	7170
6		2.840	193931	2.92	10834
7		4.067	14703	0.22	1536
8		4.919	5685	0.09	403
9		5.650	1403	0.02	232
10		6.034	13895	0.21	640
11		7.000	35115	0.53	1082
12		7.294	14731	0.22	1205

Reported by User: Saneesh (Saneesh) Project Name: HDI_Stellenbosch_2016

	Peak Name	RT	Area	% Area	Height
13		7.550	30809	0.46	1928
14		7.708	17682	0.27	1646
15		8.013	72749	1.10	8466
16	25ODESRIF	8.437	4317609	65.08	252712
17	RIF	9.400			
18		9.906	13140	0.20	752
19		10.279	5084	0.08	562
20		10.536	253074	3.81	23777
21	NEO	10.764	756719	11.41	112139
22		11.058	70282	1.06	7808

SAMPLE INFORMATION

Sample Name:	RIF Inc Basil_EtOH 100mig/mL 1	Acquired By:	Saneesh
Sample Type:	Unknown	Date Acquired:	2016/09/10 07:28:27 AM
Vial:	21	Acq. Method Set:	HDI_RIF_25ODESRIF_NEO
Injection #:	1	Date Processed:	2016/09/10 01:28:42 PM
Injection Volume:	15.00 ul	Processing Method:	HDI_RIF_ W2996 254.0nm-1.2
Run Time:	11.5 Minutes	Channel Name:	W2996 254.0nm-1.2
Sample Set Name:	RIF_Basil MeOHEOH2 IC50 10_916	Proc. Chnl. Descr.:	W2996 PDA 254.0 nm at 1.2

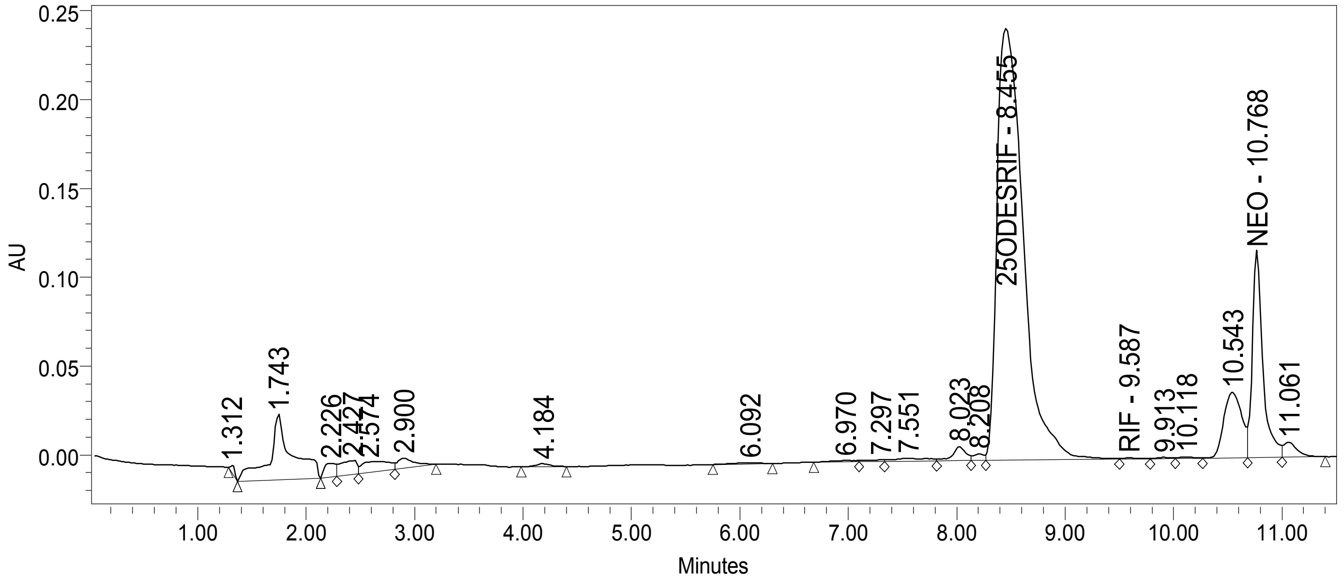

	Peak Name	RT	Area	% Area	Height
1		1.312	12769	0.20	3742
2		1.743	544703	8.47	36110
3		2.226	57067	0.89	7761
4		2.427	84614	1.32	7778
5		2.574	99696	1.55	5832
6		2.900	59083	0.92	5627
7		4.184	15484	0.24	1750
8		6.092	10702	0.17	622
9		6.970	13318	0.21	796
10		7.297	12233	0.19	1018
11		7.551	39940	0.62	1750
12		8.023	68250	1.06	7973

Reported by User: Saneesh (Saneesh) Project Name: HDI_Stellenbosch_2016

	Peak Name	RT	Area	% Area	Height
13		8.208	26958	0.42	3894
14	25ODESRIF	8.455	4079405	63.47	243388
15	RIF	9.587	5871	0.09	756
16		9.913	6595	0.10	647
17		10.118	7757	0.12	830
18		10.543	432431	6.73	37009
19	NEO	10.768	774431	12.05	115527
20		11.061	76347	1.19	8412

SAMPLE INFORMATION

Sample Name:	RIF Inc Basil_EtOH 100mig/mL 2	Acquired By:	Saneesh
Sample Type:	Unknown	Date Acquired:	2016/09/10 07:43:23 AM
Vial:	22	Acq. Method Set:	HDI_RIF_25ODESRIF_NEO
Injection #:	1	Date Processed:	2016/09/10 01:28:42 PM
Injection Volume:	15.00 ul	Processing Method:	HDI_RIF_ W2996 254.0nm-1.2
Run Time:	11.5 Minutes	Channel Name:	W2996 254.0nm-1.2
Sample Set Name:	RIF_Basil MeOHEOH2 IC50 10_916	Proc. Chnl. Descr.:	W2996 PDA 254.0 nm at 1.2

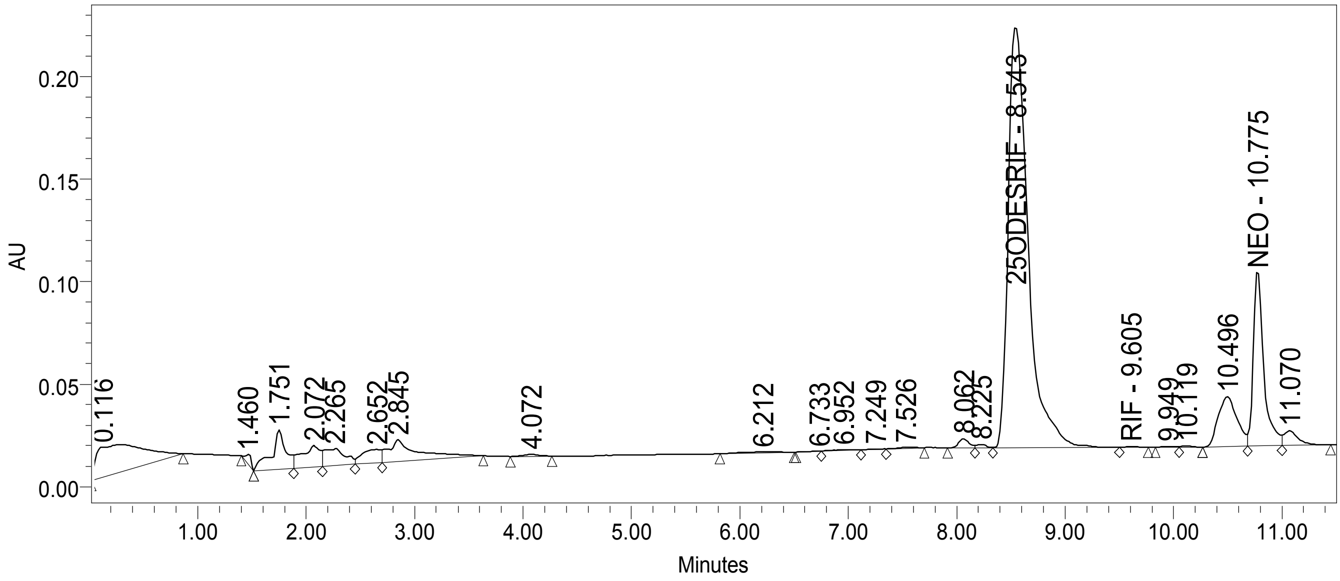

	Peak Name	RT	Area	% Area	Height
1		0.116	417798	8.81	14696
2		1.460	17395	0.37	4355
3		1.751	175317	3.70	19167
4		2.072	123854	2.61	10432
5		2.265	108347	2.28	8233
6		2.652	80827	1.70	6486
7		2.845	198747	4.19	10575
8		4.072	10243	0.22	1139
9		6.212	10674	0.22	461
10		6.733	538	0.01	103
11		6.952	5468	0.12	376
12		7.249	2055	0.04	184

	Peak Name	RT	Area	% Area	Height
13		7.526	5609	0.12	498
14		8.062	28644	0.60	4398
15		8.225	9698	0.20	1561
16	25ODESRIF	8.543	2609659	55.01	206728
17	RIF	9.605	2837	0.06	460
18		9.949	2800	0.06	332
19		10.119	3207	0.07	502
20		10.496	299046	6.30	24322
21	NEO	10.775	563586	11.88	85468
22		11.070	67758	1.43	7203

SAMPLE INFORMATION

Sample Name:	RIF Inc Basil_EtOH 150miG/mL 1	Acquired By:	Saneesh
Sample Type:	Unknown	Date Acquired:	2016/09/10 08:07:00 AM
Vial:	23	Acq. Method Set:	HDI_RIF_25ODESRIF_NEO
Injection #:	1	Date Processed:	2016/09/10 01:28:42 PM
Injection Volume:	15.00 ul	Processing Method:	HDI_RIF_ W2996 254.0nm-1.2
Run Time:	11.5 Minutes	Channel Name:	W2996 254.0nm-1.2
Sample Set Name:	RIF_Basil MeOHEOH2 IC50 10_916	Proc. Chnl. Descr.:	W2996 PDA 254.0 nm at 1.2

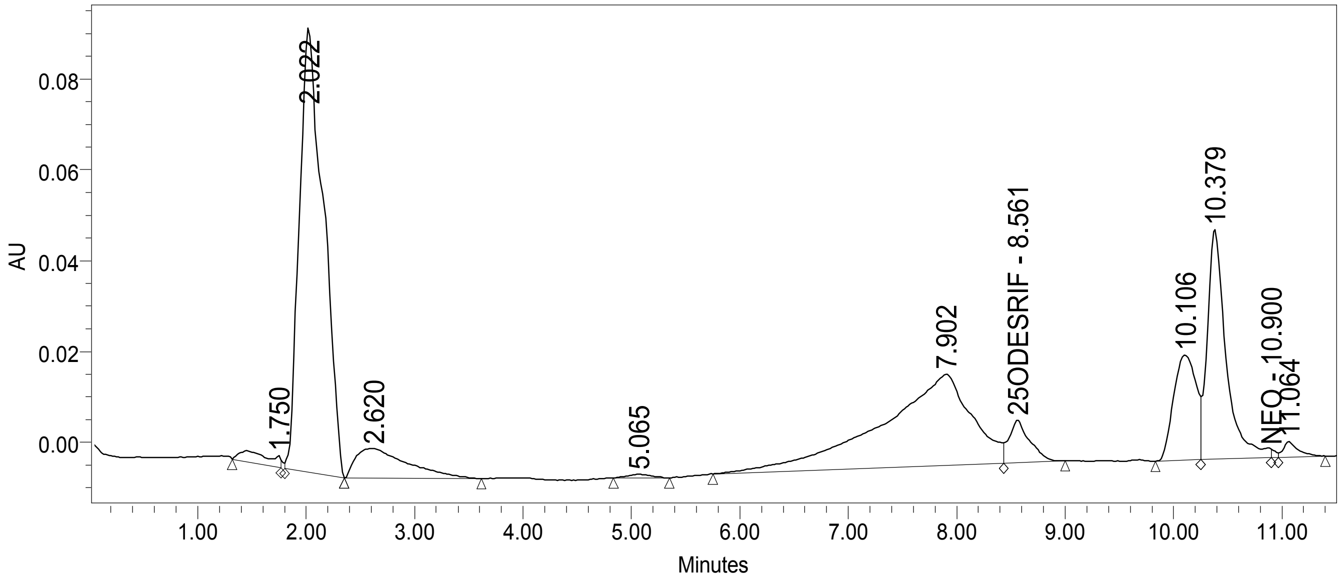

	Peak Name	RT	Area	% Area	Height
1		1.750	50370	1.24	2557
2		2.022	1517537	37.35	97555
3		2.620	215337	5.30	6491
4		5.065	12060	0.30	777
5		7.902	1156832	28.47	20035
6	25ODESRIF	8.561	122982	3.03	9352
7	RIF	9.400			
8		10.106	333590	8.21	23183
9		10.379	615613	15.15	50622
10	NEO	10.900	5657	0.14	1841
11		11.064	33273	0.82	3351

SAMPLE INFORMATION

Sample Name:	RIF Inc Basil_EtOH 150miG/mL 2	Acquired By:	Saneesh
Sample Type:	Unknown	Date Acquired:	2016/09/10 08:21:46 AM
Vial:	24	Acq. Method Set:	HDI_RIF_25ODESRIF_NEO
Injection #:	1	Date Processed:	2016/09/10 01:28:42 PM
Injection Volume:	15.00 ul	Processing Method:	HDI_RIF_ W2996 254.0nm-1.2
Run Time:	11.5 Minutes	Channel Name:	W2996 254.0nm-1.2
Sample Set Name:	RIF_Basil MeOHEOH2 IC50 10_916	Proc. Chnl. Descr.:	W2996 PDA 254.0 nm at 1.2

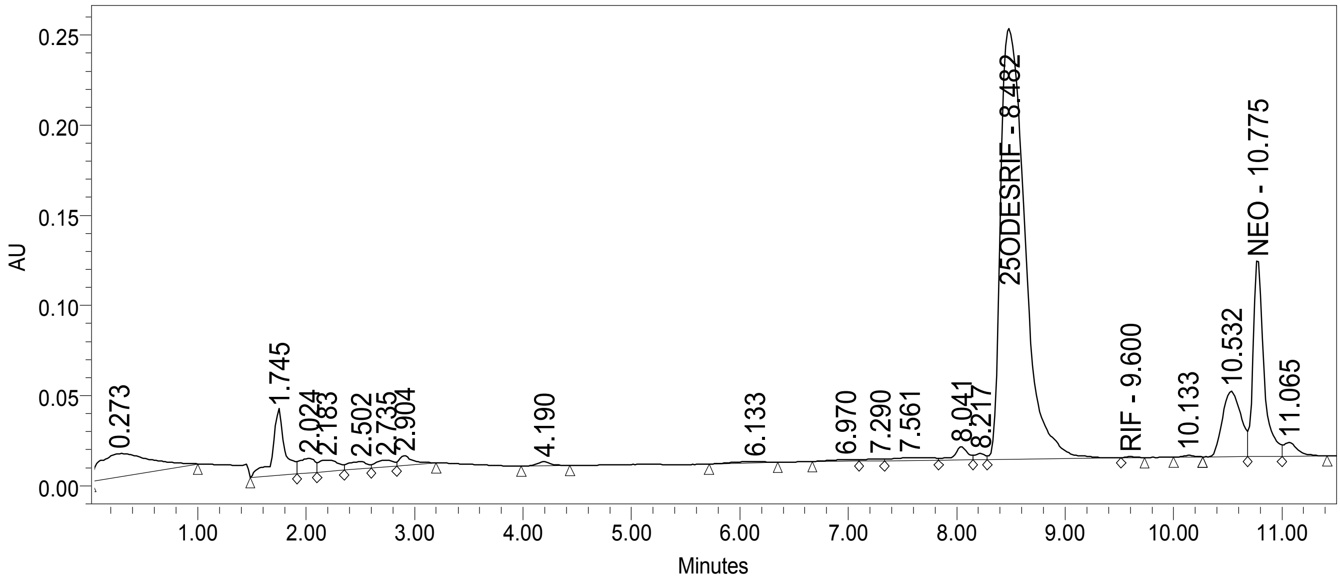

	Peak Name	RT	Area	% Area	Height
1		0.273	411809	6.69	12788
2		1.745	291206	4.73	35576
3		2.024	80770	1.31	8046
4		2.183	77743	1.26	6233
5		2.502	50933	0.83	4010
6		2.735	40994	0.67	3530
7		2.904	47191	0.77	5364
8		4.190	20400	0.33	2326
9		6.133	18530	0.30	858
10		6.970	15330	0.25	1002
11		7.290	12760	0.21	1030
12		7.561	39290	0.64	1624

Reported by User: Saneesh (Saneesh) Project Name: HDI_Stellenbosch_2016

	Peak Name	RT	Area	% Area	Height
13		8.041	60363	0.98	7195
14		8.217	22625	0.37	3406
15	25ODESRIF	8.482	3704084	60.19	238977
16	RIF	9.600	4767	0.08	759
17		10.133	5986	0.10	975
18		10.532	434882	7.07	36235
19	NEO	10.775	744594	12.10	109338
20		11.065	69948	1.14	7581

SAMPLE INFORMATION

Sample Name:	RIF Inc Basil_EtOH 200miG/mL	Acquired By:	Saneesh
Sample Type:	Unknown	Date Acquired:	2016/09/10 08:36:25 AM
Vial:	2	Acq. Method Set:	HDI_RIF_25ODESRIF_NEO
Injection #:	1	Date Processed:	2016/09/10 01:28:42 PM
Injection Volume:	15.00 ul	Processing Method:	HDI_RIF_ W2996 254.0nm-1.2
Run Time:	11.5 Minutes	Channel Name:	W2996 254.0nm-1.2
Sample Set Name:	RIF_Basil MeOHEOH2 IC50 10_916	Proc. Chnl. Descr.:	W2996 PDA 254.0 nm at 1.2

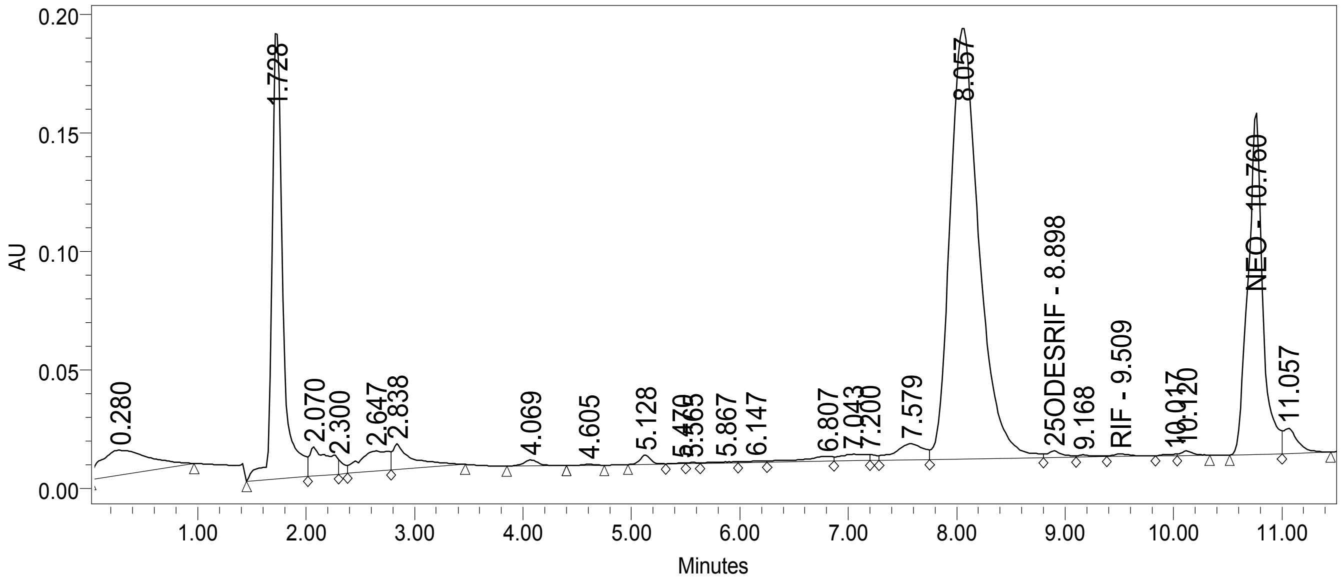

	Peak Name	RT	Area	% Area	Height
1		0.280	321994	4.41	10468
2		1.728	1317484	18.04	192079
3		2.070	151746	2.08	12232
4		2.300	21325	0.29	6136
5		2.647	161109	2.21	8478
6		2.838	138785	1.90	10757
7		4.069	25161	0.34	2576
8		4.605	4136	0.06	599
9		5.128	25474	0.35	3816
10		5.470	1763	0.02	223
11		5.565	2525	0.03	430
12		5.867	7030	0.10	443

Reported by User: Saneesh (Saneesh) Project Name: HDI_Stellenbosch_2016

	Peak Name	RT	Area	% Area	Height
13		6.147	8362	0.11	667
14		6.807	40885	0.56	2047
15		7.043	49380	0.68	2809
16		7.200	11396	0.16	2568
17		7.579	125470	1.72	6819
18		8.057	3378860	46.27	182018
19	25ODESRIF	8.898	28034	0.38	2795
20		9.168	11201	0.15	984
21	RIF	9.509	15148	0.21	1191
22		10.017	5966	0.08	660
23		10.120	14100	0.19	1875
24	NEO	10.760	1333381	18.26	144050
25		11.057	101335	1.39	10521

Reported by User: Saneesh (Saneesh)   Project Name: HDI_Stellenbosch_2016

SAMPLE INFORMATION

Sample Name:	RIF Inc Basil_MeOH 1miG/mL 1 Rep	Acquired By:	Saneesh
Sample Type:	Unknown	Date Acquired:	2016/09/10 08:53:27 AM
Vial:	7	Acq. Method Set:	HDI_RIF_25ODESRIF_NEO
Injection #:	1	Date Processed:	2016/09/10 01:28:49 PM
Injection Volume:	15.00 ul	Processing Method:	HDI_RIF_ W2996 254.0nm-1.2
Run Time:	11.5 Minutes	Channel Name:	W2996 254.0nm-1.2
Sample Set Name:	RIF_Basil MeOHEOH3 IC50 10_916	Proc. Chnl. Descr.:	W2996 PDA 254.0 nm at 1.2

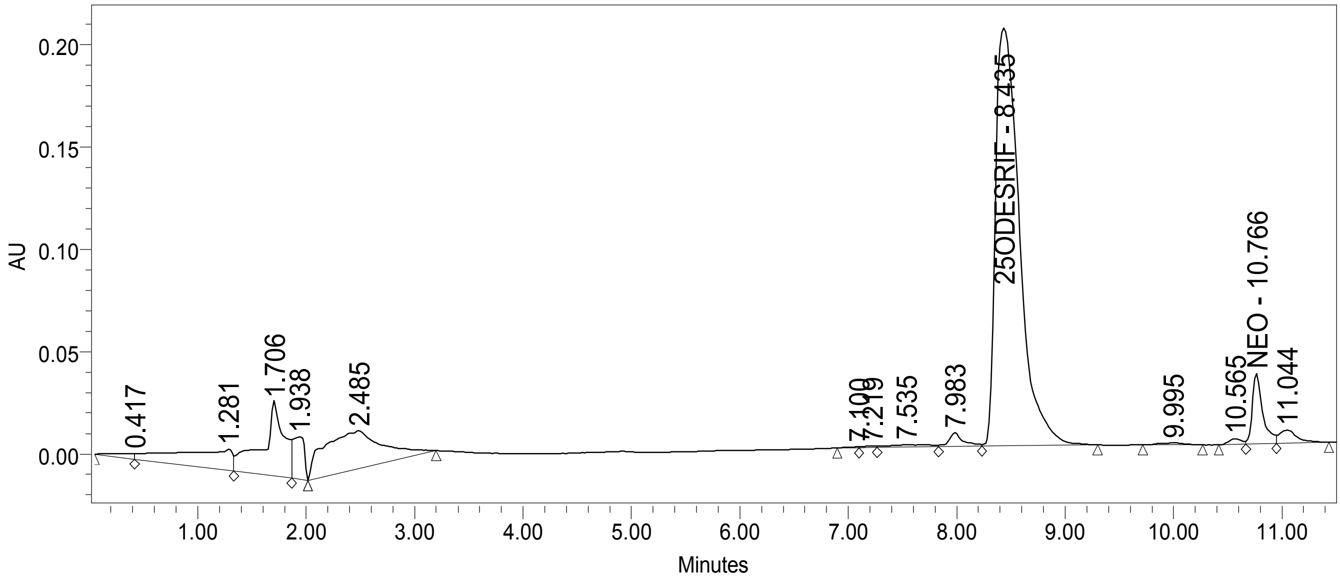

	Peak Name	RT	Area	% Area	Height
1		0.417	33120	0.62	2722
2		1.281	338582	6.35	10419
3		1.706	519360	9.74	36704
4		1.938	153226	2.87	20981
5		2.485	718276	13.47	18510
6		7.100	3402	0.06	476
7		7.219	5928	0.11	658
8		7.535	32098	0.60	1292
9		7.983	61516	1.15	6666
10	25ODESRIF	8.435	3126919	58.63	204255
11	RIF	9.400			
12		9.995	16585	0.31	1008

Reported by User: Saneesh (Saneesh) Project Name: HDI_Stellenbosch_2016

	Peak Name	RT	Area	% Area	Height
13		10.565	22980	0.43	2714
14	NEO	10.766	226916	4.25	34036
15		11.044	74798	1.40	6466

Reported by User: Saneesh (Saneesh) Project Name: HDI_Stellenbosch_2016

SAMPLE INFORMATION

Sample Name:	RIF Inc Basil_EtOH 150miG/mL 1 R	Acquired By:	Saneesh
Sample Type:	Unknown	Date Acquired:	2016/09/10 09:08:23 AM
Vial:	8	Acq. Method Set:	HDI_RIF_25ODESRIF_NEO
Injection #:	1	Date Processed:	2016/09/10 01:28:49 PM
Injection Volume:	15.00 ul	Processing Method:	HDI_RIF_ W2996 254.0nm-1.2
Run Time:	11.5 Minutes	Channel Name:	W2996 254.0nm-1.2
Sample Set Name:	RIF_Basil MeOHEOH3 IC50 10_916	Proc. Chnl. Descr.:	W2996 PDA 254.0 nm at 1.2

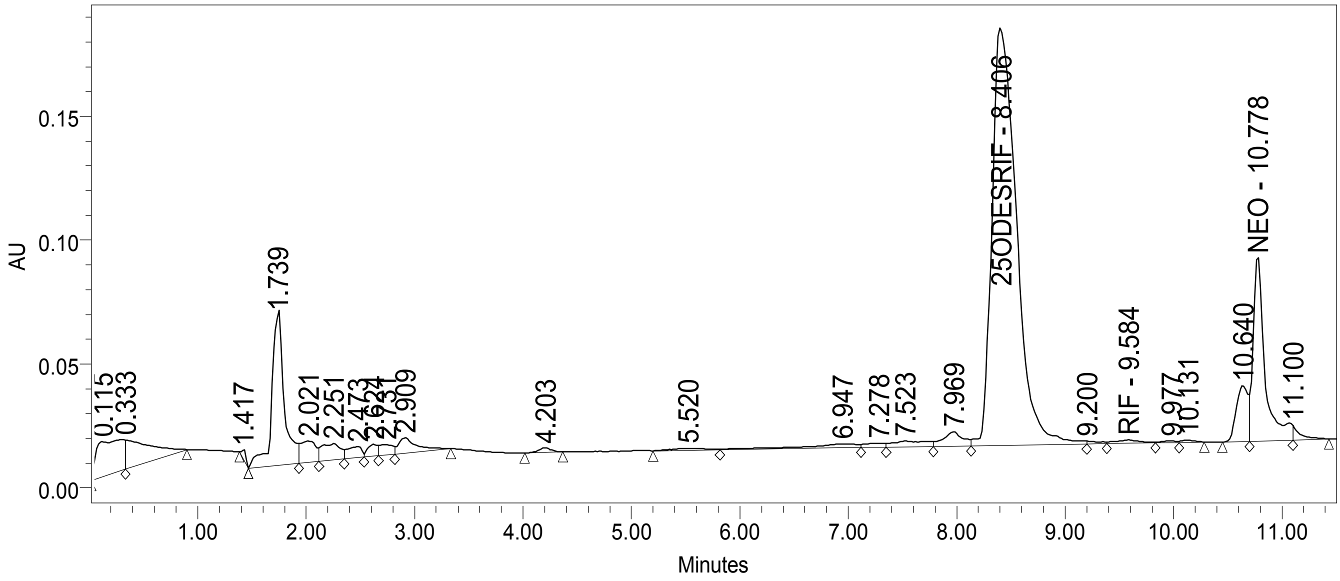

	Peak Name	RT	Area	% Area	Height
1		0.115	226246	4.58	14348
2		0.333	183933	3.72	11862
3		1.417	11152	0.23	3496
4		1.739	497770	10.07	61921
5		2.021	84897	1.72	8563
6		2.251	80857	1.64	6612
7		2.473	42480	0.86	4722
8		2.624	30480	0.62	4773
9		2.731	35162	0.71	4175
10		2.909	74382	1.50	6229
11		4.203	14393	0.29	1819
12		5.520	18275	0.37	818

Reported by User: Saneesh (Saneesh) Project Name: HDI_Stellenbosch_2016

	Peak Name	RT	Area	% Area	Height
13		6.947	57892	1.17	1569
14		7.278	21166	0.43	1633
15		7.523	50069	1.01	2309
16		7.969	73757	1.49	5831
17	25ODESRIF	8.406	2643248	53.47	168821
18		9.200	8864	0.18	1113
19	RIF	9.584	21334	0.43	1406
20		9.977	6559	0.13	643
21		10.131	7397	0.15	1004
22		10.640	169299	3.42	22592
23	NEO	10.778	556244	11.25	74190
24		11.100	27827	0.56	5891

Multi Sample Summary

Reported by User: Saneesh (Saneesh) Project Name: HDI_Stellenbosch_2016

SAMPLE INFORMATION

Sample Name:	RIF Control Incubate 1	Acquired By:	Saneesh
Sample Type:	Unknown	Date Acquired:	2016/12/12 08:15:08 AM
Vial:	23	Acq. Method Set:	HDI_RIF_25ODESRIF_NEO
Injection #:	1	Date Processed:	2016/12/12 04:14:58 PM
Injection Volume:	15.00 ul	Processing Method:	HDI_RIF_ W2996 254.0nm-1.2
Run Time:	11.5 Minutes	Channel Name:	W2996 254.0nm-1.2
Sample Set Name:	RIF_Basil 2P Scrng 12_12_16	Proc. Chnl. Descr.:	W2996 PDA 254.0 nm at 1.2

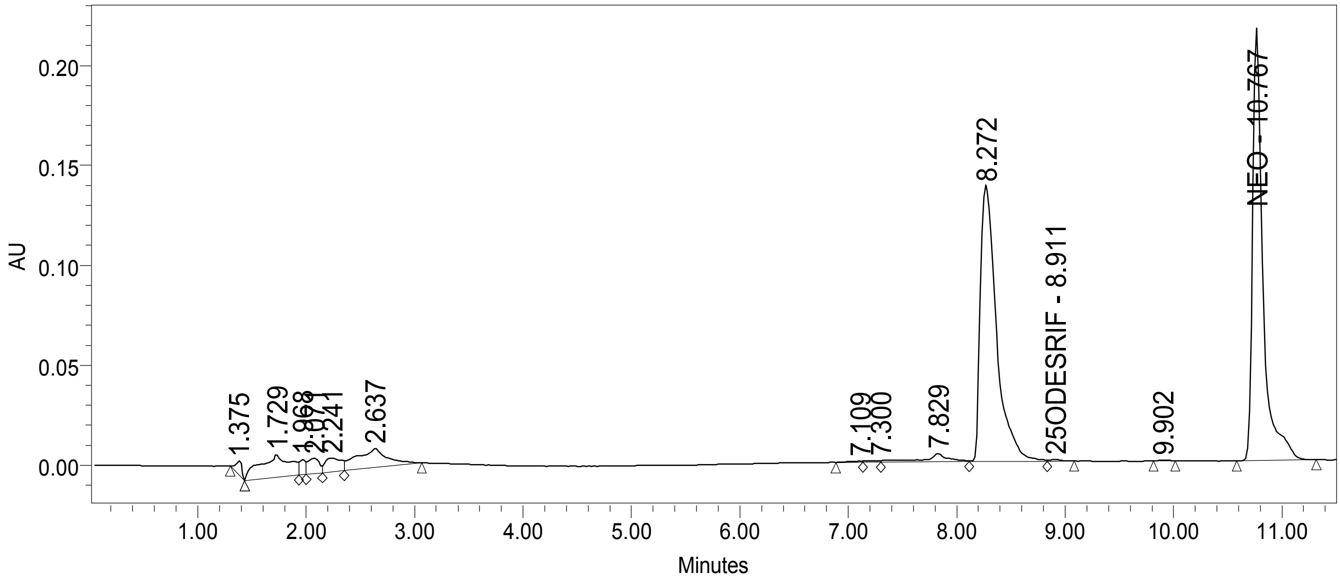

	Peak Name	RT	Area	% Area	Height
1		1.375	26610	0.75	6912
2		1.729	218151	6.17	11002
3		1.968	28211	0.80	7474
4		2.071	56745	1.60	7581
5		2.241	69123	1.95	6725
6		2.637	189514	5.36	9301
7		7.109	5529	0.16	586
8		7.300	6805	0.19	767
9		7.829	65361	1.85	4170
10		8.272	1439844	40.71	138957
11	25ODESRIF	8.911	6353	0.18	883
12	RIF	9.400			
13		9.902	2081	0.06	382
14	NEO	10.767	1422488	40.22	214141

SAMPLE INFORMATION

Sample Name:	RIF Control Incubate 2	Acquired By:	Saneesh
Sample Type:	Unknown	Date Acquired:	2016/12/12 08:30:05 AM
Vial:	24	Acq. Method Set:	HDI_RIF_25ODESRIF_NEO
Injection #:	1	Date Processed:	2016/12/12 04:14:58 PM
Injection Volume:	15.00 ul	Processing Method:	HDI_RIF_ W2996 254.0nm-1.2
Run Time:	11.5 Minutes	Channel Name:	W2996 254.0nm-1.2
Sample Set Name:	RIF_Basil 2P Scrng 12_12_16	Proc. Chnl. Descr.:	W2996 PDA 254.0 nm at 1.2

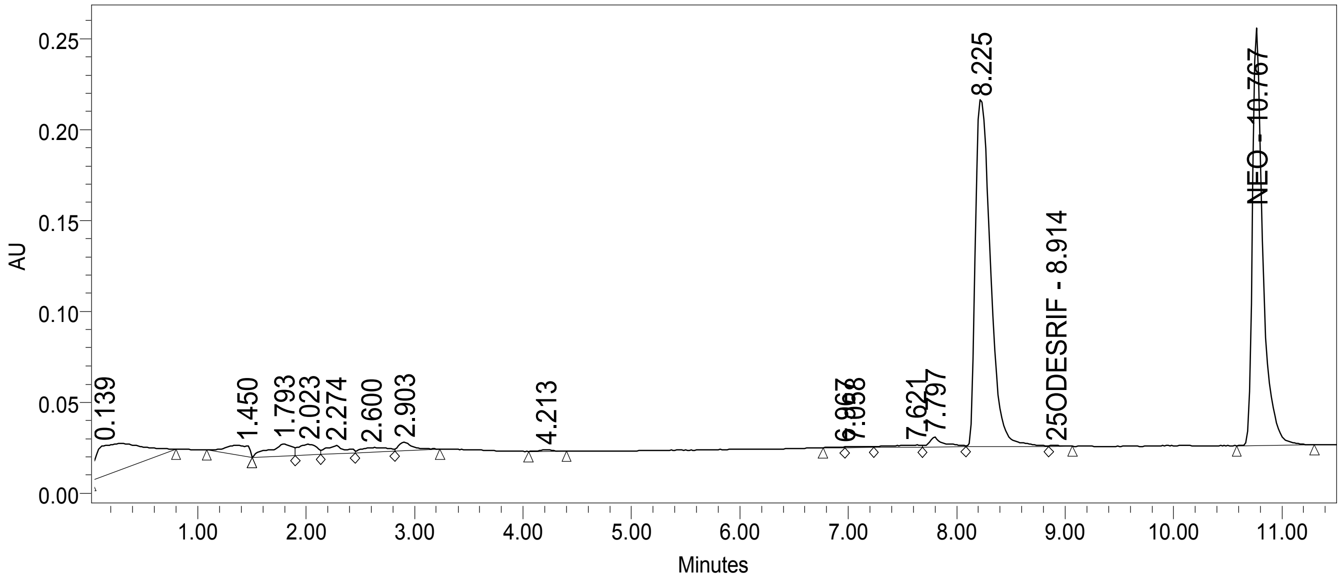

	Peak Name	RT	Area	% Area	Height
1		0.139	442502	10.25	16476
2		1.450	79632	1.84	6218
3		1.793	100286	2.32	6668
4		2.023	67746	1.57	5832
5		2.274	54869	1.27	4427
6		2.600	41434	0.96	2392
7		2.903	40718	0.94	4529
8		4.213	7347	0.17	973
9		6.967	2997	0.07	395
10		7.058	8630	0.20	593
11		7.621	24435	0.57	1282
12		7.797	51195	1.19	5407

Reported by User: Saneesh (Saneesh) Project Name: HDI_Stellenbosch_2016

	Peak Name	RT	Area	% Area	Height
13		8.225	1878913	43.52	192675
14	25ODESRIF	8.914	3579	0.08	519
15	RIF	9.400			
16	NEO	10.767	1512726	35.04	227485

SAMPLE INFORMATION

Sample Name:	RIF Inc Basil_H2O 10miG/mL 1	Acquired By:	Saneesh
Sample Type:	Unknown	Date Acquired:	2016/12/12 08:45:01 AM
Vial:	2	Acq. Method Set:	HDI_RIF_25ODESRIF_NEO
Injection #:	1	Date Processed:	2016/12/12 04:14:58 PM
Injection Volume:	15.00 ul	Processing Method:	HDI_RIF_ W2996 254.0nm-1.2
Run Time:	11.5 Minutes	Channel Name:	W2996 254.0nm-1.2
Sample Set Name:	RIF_Basil 2P Scrng 12_12_16	Proc. Chnl. Descr.:	W2996 PDA 254.0 nm at 1.2

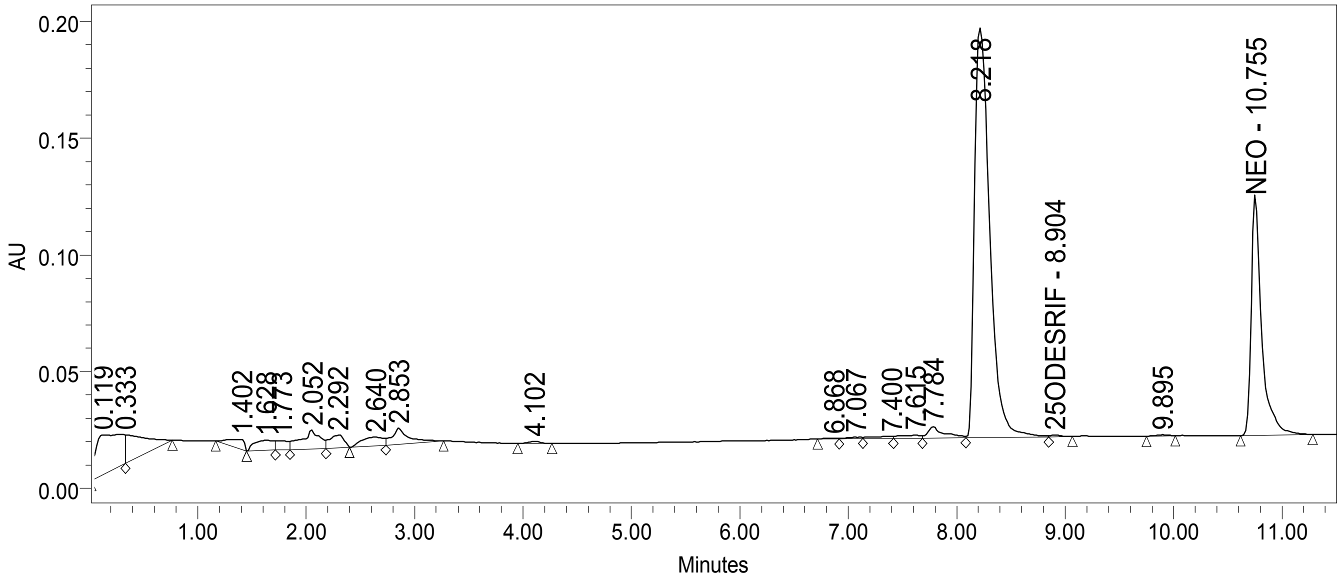

	Peak Name	RT	Area	% Area	Height
1		0.119	265228	7.98	17377
2		0.333	151398	4.56	12240
3		1.402	41306	1.24	4713
4		1.628	54872	1.65	4356
5		1.773	30230	0.91	3933
6		2.052	97125	2.92	7956
7		2.292	50864	1.53	5572
8		2.640	52729	1.59	3684
9		2.853	72513	2.18	6758
10		4.102	6549	0.20	908
11		6.868	2415	0.07	268
12		7.067	5983	0.18	578

Reported by User: Saneesh (Saneesh) Project Name: HDI_Stellenbosch_2016

	Peak Name	RT	Area	% Area	Height
13		7.400	11207	0.34	822
14		7.615	17550	0.53	1314
15		7.784	48399	1.46	4849
16		8.218	1723333	51.87	175617
17	25ODESRIF	8.904	5262	0.16	891
18	RIF	9.400			
19		9.895	3918	0.12	561
20	NEO	10.755	681615	20.52	102160

SAMPLE INFORMATION

Sample Name:	RIF Inc Basil_H2O 10miG/mL 2	Acquired By:	Saneesh
Sample Type:	Unknown	Date Acquired:	2016/12/12 08:59:48 AM
Vial:	3	Acq. Method Set:	HDI_RIF_25ODESRIF_NEO
Injection #:	1	Date Processed:	2016/12/12 04:14:58 PM
Injection Volume:	15.00 ul	Processing Method:	HDI_RIF_ W2996 254.0nm-1.2
Run Time:	11.5 Minutes	Channel Name:	W2996 254.0nm-1.2
Sample Set Name:	RIF_Basil 2P Scrng 12_12_16	Proc. Chnl. Descr.:	W2996 PDA 254.0 nm at 1.2

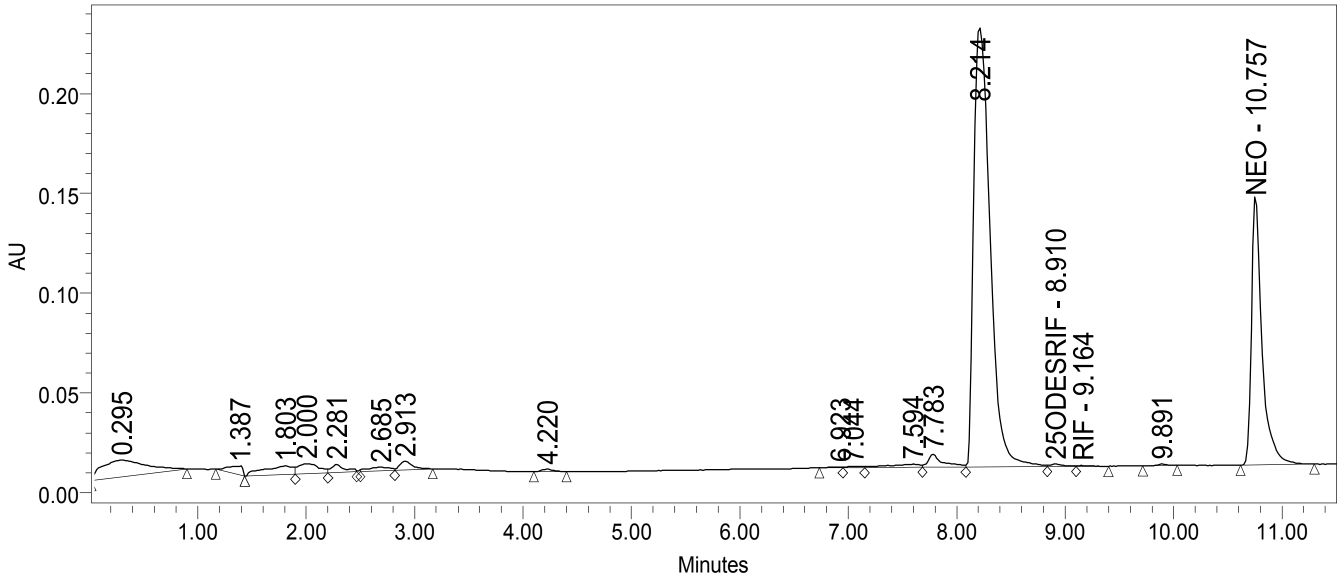

	Peak Name	RT	Area	% Area	Height
1		0.295	234386	6.03	8233
2		1.387	38753	1.00	4711
3		1.803	82567	2.13	4237
4		2.000	65625	1.69	4933
5		2.281	33660	0.87	3953
6		2.685	25467	0.66	1757
7		2.913	33949	0.87	4210
8		4.220	7947	0.20	1149
9		6.923	3528	0.09	390
10		7.044	6751	0.17	652
11		7.594	33294	0.86	1519
12		7.783	62052	1.60	6594

	Peak Name	RT	Area	% Area	Height
13		8.214	2333948	60.08	220196
14	25ODESRIF	8.910	11210	0.29	1331
15	RIF	9.164	3687	0.09	442
16		9.891	4293	0.11	714
17	NEO	10.757	903344	23.26	134647

SAMPLE INFORMATION

Sample Name:	RIF Inc Basil_H2O 200miG/mL 1	Acquired By:	Saneesh
Sample Type:	Unknown	Date Acquired:	2016/12/12 09:14:34 AM
Vial:	4	Acq. Method Set:	HDI_RIF_25ODESRIF_NEO
Injection #:	1	Date Processed:	2016/12/12 04:14:58 PM
Injection Volume:	15.00 ul	Processing Method:	HDI_RIF_ W2996 254.0nm-1.2
Run Time:	11.5 Minutes	Channel Name:	W2996 254.0nm-1.2
Sample Set Name:	RIF_Basil 2P Scrng 12_12_16	Proc. Chnl. Descr.:	W2996 PDA 254.0 nm at 1.2

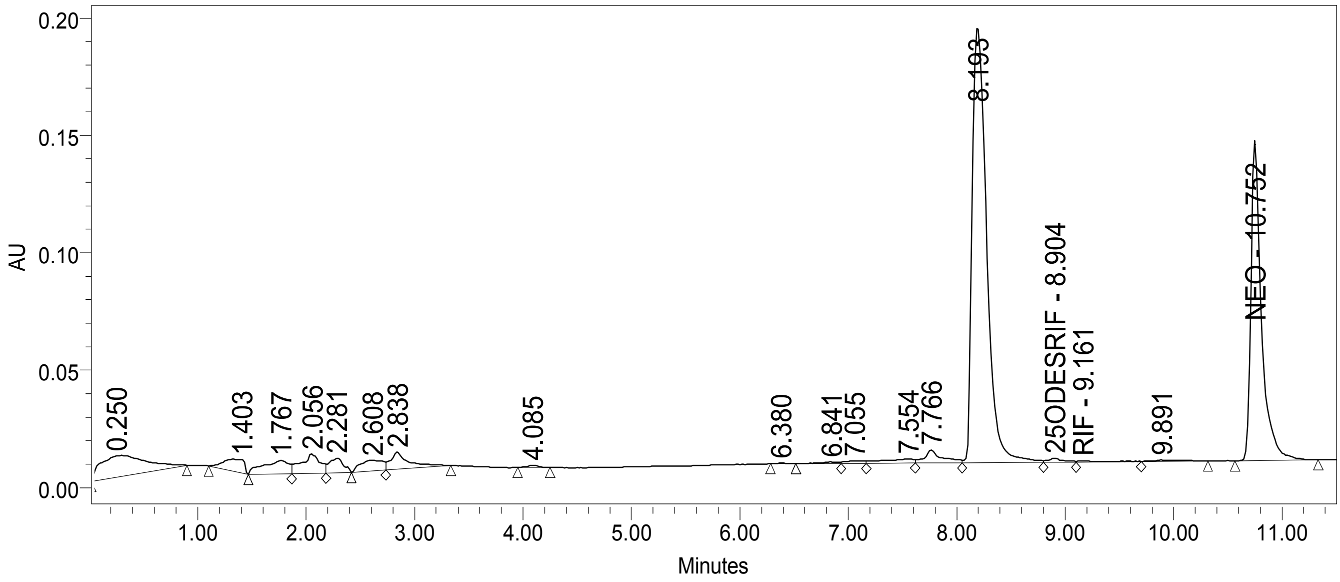

	Peak Name	RT	Area	% Area	Height
1		0.250	258708	7.32	8983
2		1.403	70363	1.99	6191
3		1.767	95187	2.69	5680
4		2.056	99817	2.82	8264
5		2.281	57003	1.61	6229
6		2.608	66438	1.88	4504
7		2.838	87430	2.47	7382
8		4.085	6753	0.19	929
9		6.380	1976	0.06	273
10		6.841	7942	0.22	649
11		7.055	11815	0.33	988
12		7.554	32924	0.93	1631

Reported by User: Saneesh (Saneesh)   Project Name: HDI_Stellenbosch_2016

	Peak Name	RT	Area	% Area	Height
13		7.766	60560	1.71	5463
14		8.193	1734932	49.06	187706
15	25ODESRIF	8.904	13969	0.40	1656
16	RIF	9.161	6163	0.17	458
17		9.891	10323	0.29	626
18	NEO	10.752	913795	25.84	135206

SAMPLE INFORMATION

Sample Name:	RIF Inc Basil_H2O 200miG/mL 2	Acquired By:	Saneesh
Sample Type:	Unknown	Date Acquired:	2016/12/12 09:29:19 AM
Vial:	5	Acq. Method Set:	HDI_RIF_25ODESRIF_NEO
Injection #:	1	Date Processed:	2016/12/12 04:14:58 PM
Injection Volume:	15.00 ul	Processing Method:	HDI_RIF_ W2996 254.0nm-1.2
Run Time:	11.5 Minutes	Channel Name:	W2996 254.0nm-1.2
Sample Set Name:	RIF_Basil 2P Scrng 12_12_16	Proc. Chnl. Descr.:	W2996 PDA 254.0 nm at 1.2

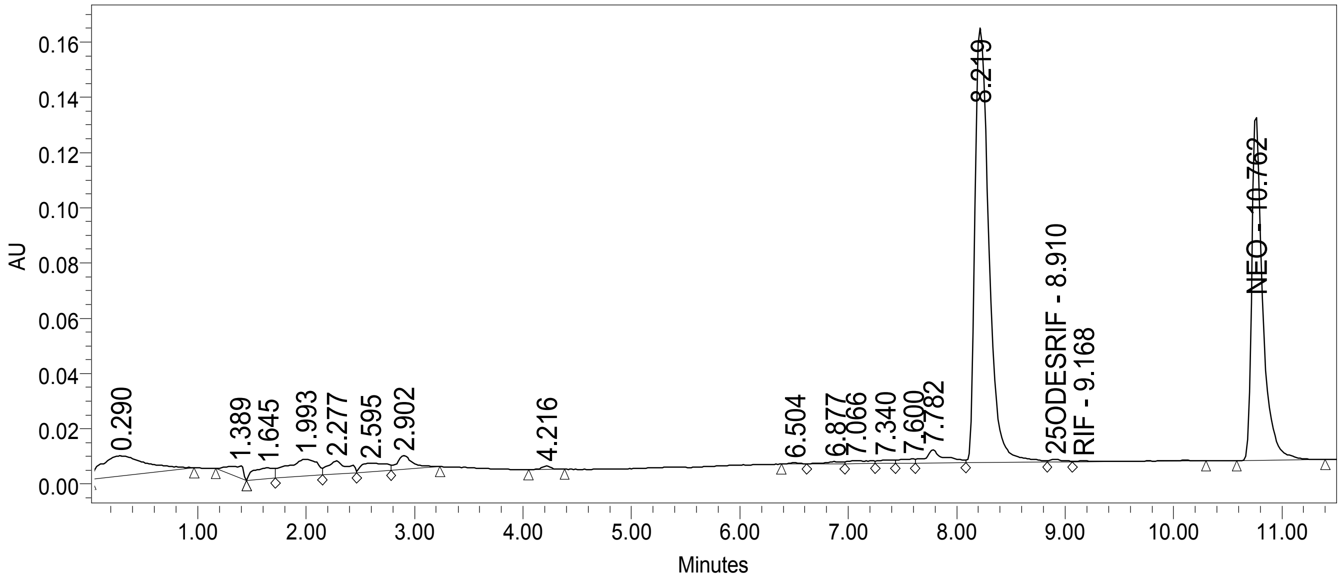

	Peak Name	RT	Area	% Area	Height
1		0.290	211633	7.00	7316
2		1.389	38835	1.28	4653
3		1.645	53519	1.77	3945
4		1.993	120513	3.98	6105
5		2.277	58438	1.93	4674
6		2.595	47810	1.58	2982
7		2.902	49997	1.65	4845
8		4.216	8604	0.28	1239
9		6.504	3560	0.12	476
10		6.877	9851	0.33	763
11		7.066	15448	0.51	995
12		7.340	11980	0.40	1161

Reported by User: Saneesh (Saneesh) Project Name: HDI_Stellenbosch_2016

	Peak Name	RT	Area	% Area	Height
13		7.600	15506	0.51	1532
14		7.782	60258	1.99	4833
15		8.219	1425526	47.13	157526
16	25ODESRIF	8.910	8922	0.30	1062
17	RIF	9.168	13633	0.45	424
18	NEO	10.762	870393	28.78	124077

Reported by User: Saneesh (Saneesh)   Project Name: HDI_Stellenbosch_2016

SAMPLE INFORMATION

Sample Name:	RIF Inc Basil_MeOH 10miG/mL 1	Acquired By:	Saneesh
Sample Type:	Unknown	Date Acquired:	2016/12/12 09:44:07 AM
Vial:	6	Acq. Method Set:	HDI_RIF_25ODESRIF_NEO
Injection #:	1	Date Processed:	2016/12/12 04:14:58 PM
Injection Volume:	15.00 ul	Processing Method:	HDI_RIF_ W2996 254.0nm-1.2
Run Time:	11.5 Minutes	Channel Name:	W2996 254.0nm-1.2
Sample Set Name:	RIF_Basil 2P Scrng 12_12_16	Proc. Chnl. Descr.:	W2996 PDA 254.0 nm at 1.2

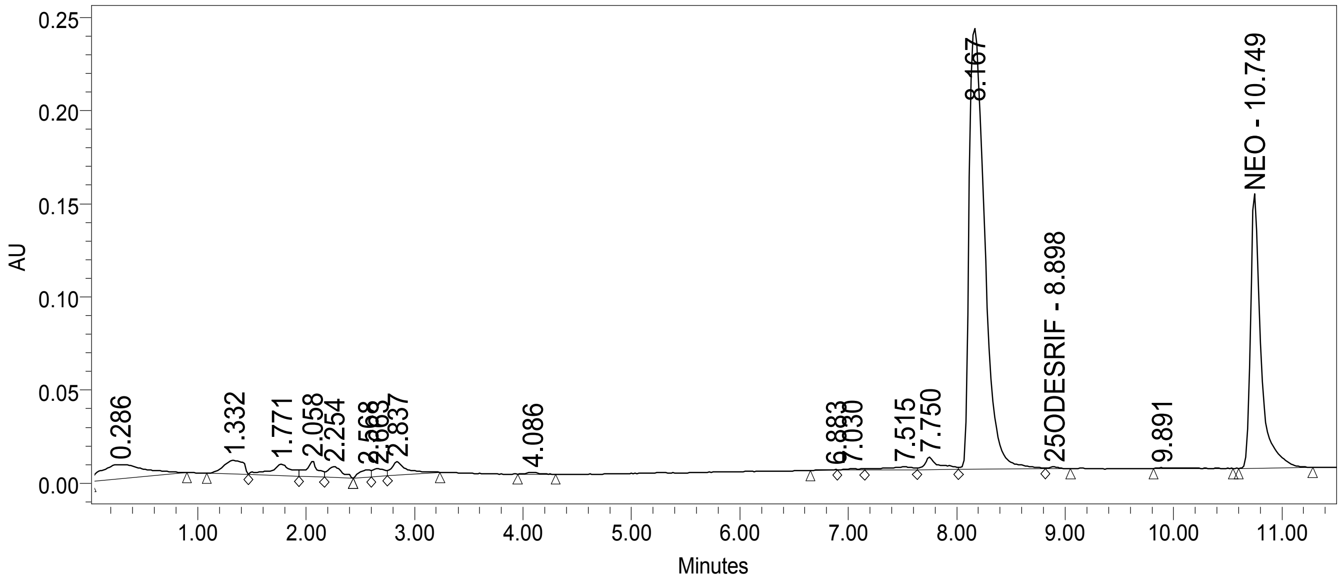

	Peak Name	RT	Area	% Area	Height
1		0.286	209676	5.09	7558
2		1.332	92592	2.25	7191
3		1.771	84990	2.06	6137
4		2.058	64444	1.56	7945
5		2.254	54384	1.32	5888
6		2.568	28763	0.70	3939
7		2.663	33387	0.81	4196
8		2.837	70833	1.72	7290
9		4.086	7428	0.18	980
10		6.883	3758	0.09	471
11		7.030	9422	0.23	762
12		7.515	31001	0.75	1549

Reported by User: Saneesh (Saneesh) Project Name: HDI_Stellenbosch_2016

	Peak Name	RT	Area	% Area	Height
13		7.750	61565	1.49	6571
14		8.167	2376724	57.68	236830
15	25ODESRIF	8.898	6046	0.15	932
16	RIF	9.400			
17		9.891	5293	0.13	395
18	NEO	10.749	980555	23.79	145900

Reported by User: Saneesh (Saneesh) Project Name: HDI_Stellenbosch_2016

SAMPLE INFORMATION

Sample Name:	RIF Inc Basil_MeOH 10miG/mL 2	Acquired By:	Saneesh
Sample Type:	Unknown	Date Acquired:	2016/12/12 09:58:52 AM
Vial:	7	Acq. Method Set:	HDI_RIF_25ODESRIF_NEO
Injection #:	1	Date Processed:	2016/12/12 04:14:58 PM
Injection Volume:	15.00 ul	Processing Method:	HDI_RIF_ W2996 254.0nm-1.2
Run Time:	11.5 Minutes	Channel Name:	W2996 254.0nm-1.2
Sample Set Name:	RIF_Basil 2P Scrng 12_12_16	Proc. Chnl. Descr.:	W2996 PDA 254.0 nm at 1.2

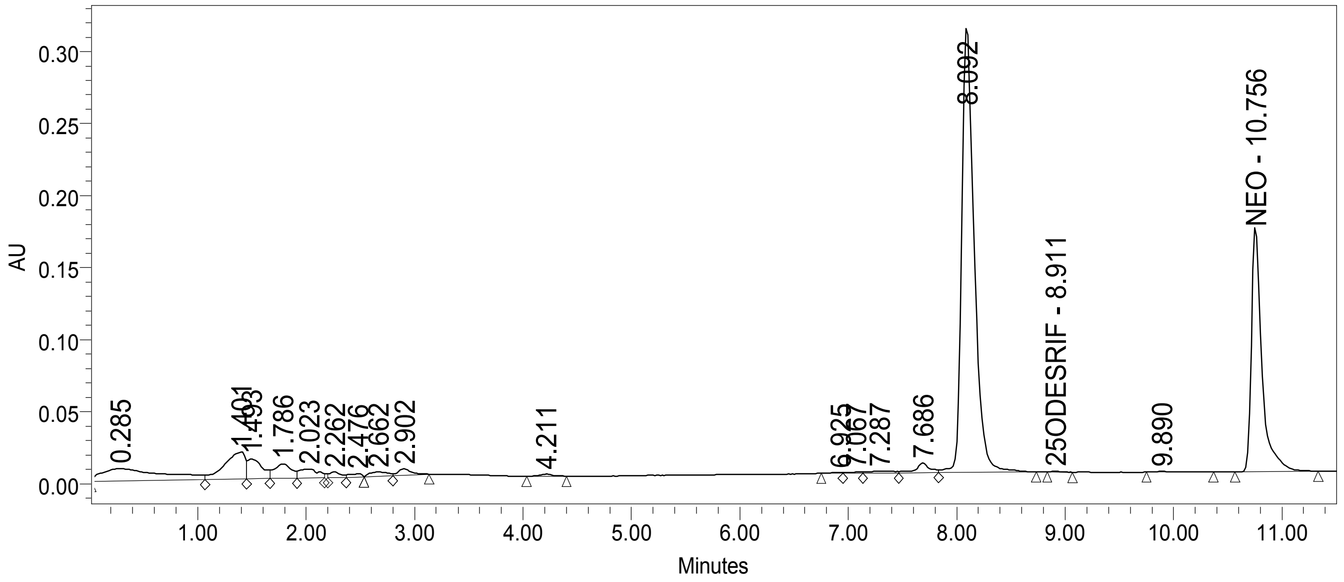

	Peak Name	RT	Area	% Area	Height
1		0.285	327635	6.92	8432
2		1.401	246729	5.21	18975
3		1.493	131262	2.77	13685
4		1.786	110223	2.33	10033
5		2.023	74012	1.56	6080
6		2.262	27915	0.59	3994
7		2.476	19100	0.40	2532
8		2.662	36472	0.77	3011
9		2.902	38345	0.81	4495
10		4.211	11112	0.23	1508
11		6.925	3203	0.07	368
12		7.067	6530	0.14	723

Reported by User: Saneesh (Saneesh) Project Name: HDI_Stellenbosch_2016

	Peak Name	RT	Area	% Area	Height
13		7.287	19040	0.40	1153
14		7.686	58177	1.23	6636
15		8.092	2481030	52.40	311657
16	25ODESRIF	8.911	3507	0.07	705
17	RIF	9.400			
18		9.890	6580	0.14	468
19	NEO	10.756	1134367	23.96	169481

Reported by User: Saneesh (Saneesh)　　Project Name: HDI_Stellenbosch_2016

SAMPLE INFORMATION

Sample Name:	RIF Inc Basil_MeOH 200miG/mL 1	Acquired By:	Saneesh
Sample Type:	Unknown	Date Acquired:	2016/12/12 10:13:31 AM
Vial:	8	Acq. Method Set:	HDI_RIF_25ODESRIF_NEO
Injection #:	1	Date Processed:	2016/12/12 04:14:58 PM
Injection Volume:	15.00 ul	Processing Method:	HDI_RIF_ W2996 254.0nm-1.2
Run Time:	11.5 Minutes	Channel Name:	W2996 254.0nm-1.2
Sample Set Name:	RIF_Basil 2P Scrng 12_12_16	Proc. Chnl. Descr.:	W2996 PDA 254.0 nm at 1.2

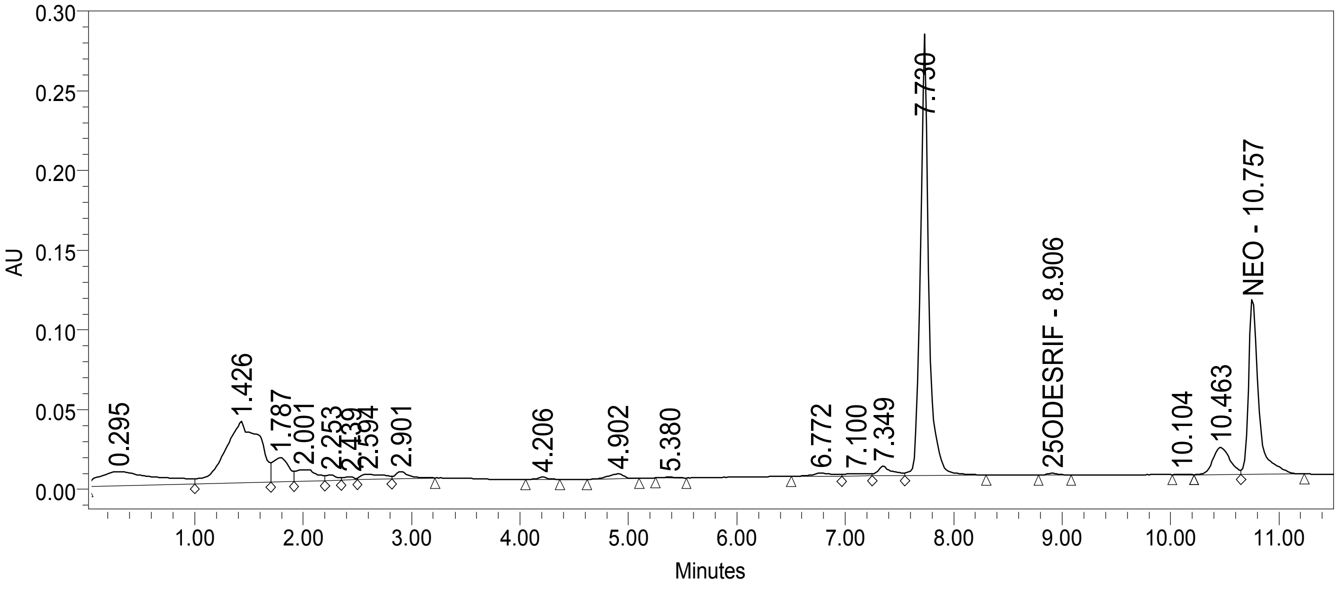

	Peak Name	RT	Area	% Area	Height
1		0.295	326236	7.96	8768
2		1.426	840714	20.52	37881
3		1.787	154214	3.76	14996
4		2.001	94992	2.32	7162
5		2.253	23936	0.58	3551
6		2.439	14002	0.34	2059
7		2.594	49636	1.21	3289
8		2.901	37529	0.92	4418
9		4.206	9892	0.24	1431
10		4.902	29956	0.73	2993
11		5.380	3793	0.09	662
12		6.772	28754	0.70	2211

Reported by User: Saneesh (Saneesh)   Project Name: HDI_Stellenbosch_2016

	Peak Name	RT	Area	% Area	Height
13		7.100	23172	0.57	1589
14		7.349	54781	1.34	5986
15		7.730	1503260	36.69	268178
16	25ODESRIF	8.906	8440	0.21	1267
17	RIF	9.400			
18		10.104	1649	0.04	259
19		10.463	185513	4.53	16964
20	NEO	10.757	707279	17.26	109835

Reported by User: Saneesh (Saneesh)

Project Name: HDI_Stellenbosch_2016

SAMPLE INFORMATION

Sample Name:	RIF Inc Basil_MeOH 200miG/mL 2	Acquired By:	Saneesh
Sample Type:	Unknown	Date Acquired:	2016/12/12 10:28:16 AM
Vial:	9	Acq. Method Set:	HDI_RIF_25ODESRIF_NEO
Injection #:	1	Date Processed:	2016/12/12 04:14:58 PM
Injection Volume:	15.00 ul	Processing Method:	HDI_RIF_ W2996 254.0nm-1.2
Run Time:	11.5 Minutes	Channel Name:	W2996 254.0nm-1.2
Sample Set Name:	RIF_Basil 2P Scrng 12_12_16	Proc. Chnl. Descr.:	W2996 PDA 254.0 nm at 1.2

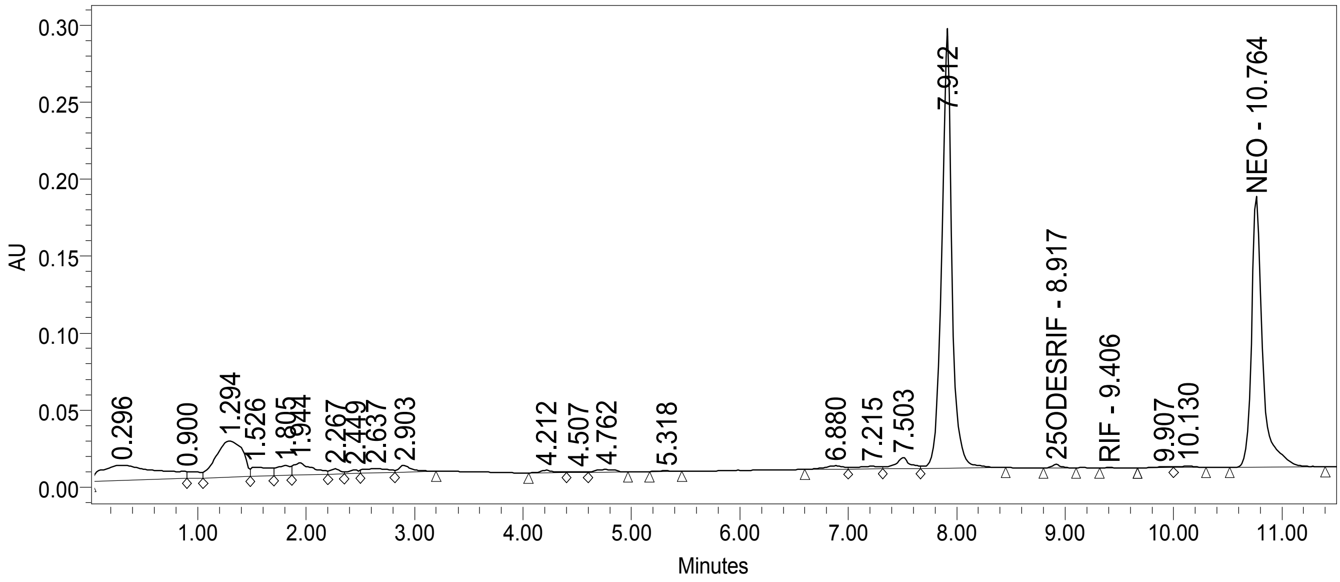

	Peak Name	RT	Area	% Area	Height
1		0.296	352730	8.00	9694
2		0.900	35697	0.81	4440
3		1.294	402774	9.13	23662
4		1.526	71636	1.62	6117
5		1.805	58361	1.32	6412
6		1.944	97660	2.21	7928
7		2.267	22087	0.50	3565
8		2.449	16041	0.36	2396
9		2.637	44681	1.01	2767
10		2.903	35557	0.81	4280
11		4.212	14219	0.32	1649
12		4.507	5287	0.12	588

	Peak Name	RT	Area	% Area	Height
13		4.762	24040	0.55	1862
14		5.318	3408	0.08	532
15		6.880	25223	0.57	2097
16		7.215	24005	0.54	1664
17		7.503	63751	1.45	6971
18		7.912	1853195	42.01	283621
19	25ODESRIF	8.917	11866	0.27	2262
20	RIF	9.406	2974	0.07	357
21		9.907	5054	0.11	587
22		10.130	7955	0.18	927
23	NEO	10.764	1232700	27.95	174650

Reported by User: Saneesh (Saneesh) | Project Name: HDI_Stellenbosch_2016

SAMPLE INFORMATION

Sample Name:	RIF Inc Basil_EtOH 10miG/mL 1	Acquired By:	Saneesh
Sample Type:	Unknown	Date Acquired:	2016/12/12 10:43:02 AM
Vial:	10	Acq. Method Set:	HDI_RIF_25ODESRIF_NEO
Injection #:	1	Date Processed:	2016/12/12 04:14:58 PM
Injection Volume:	15.00 ul	Processing Method:	HDI_RIF_ W2996 254.0nm-1.2
Run Time:	11.5 Minutes	Channel Name:	W2996 254.0nm-1.2
Sample Set Name:	RIF_Basil 2P Scrng 12_12_16	Proc. Chnl. Descr.:	W2996 PDA 254.0 nm at 1.2

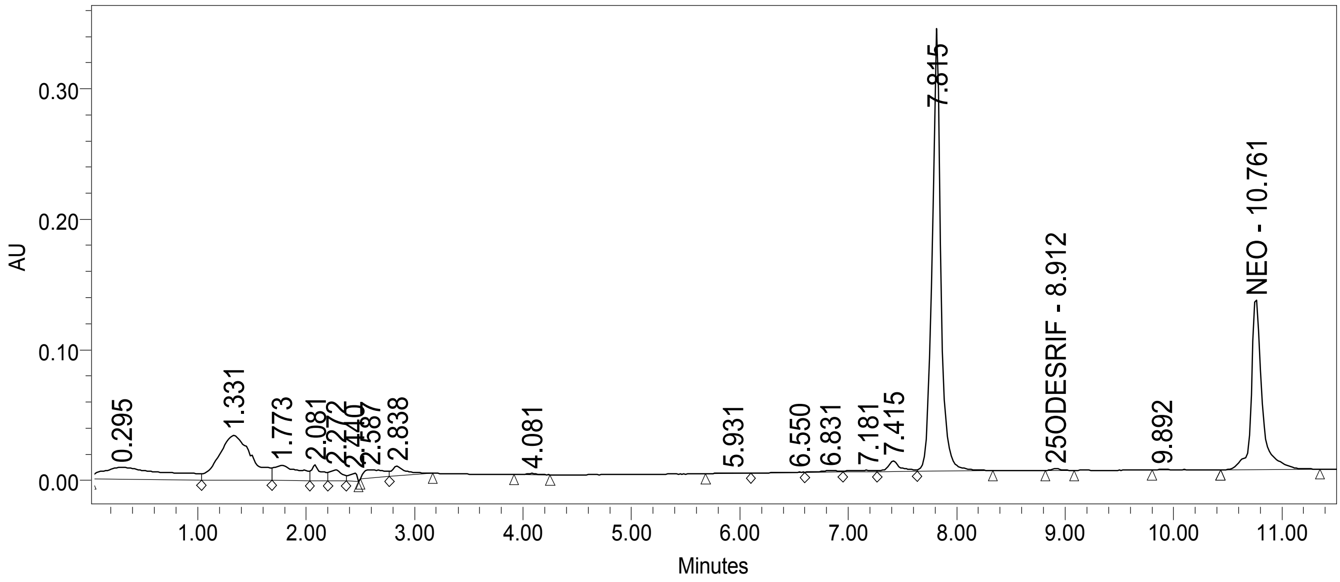

	Peak Name	RT	Area	% Area	Height
1		0.295	387573	8.47	9165
2		1.331	731898	16.00	34440
3		1.773	194243	4.25	11509
4		2.081	83716	1.83	11934
5		2.272	68194	1.49	8709
6		2.440	33098	0.72	6531
7		2.587	80226	1.75	6247
8		2.838	68961	1.51	7673
9		4.081	8564	0.19	1122
10		5.931	3687	0.08	214
11		6.550	5052	0.11	354
12		6.831	16433	0.36	1293

Reported by User: Saneesh (Saneesh)   Project Name: HDI_Stellenbosch_2016

	Peak Name	RT	Area	% Area	Height
13		7.181	19599	0.43	1148
14		7.415	65490	1.43	8108
15		7.815	1895843	41.44	331050
16	25ODESRIF	8.912	8498	0.19	1678
17	RIF	9.400			
18		9.892	9107	0.20	710
19	NEO	10.761	895253	19.57	129892

SAMPLE INFORMATION

Sample Name:	RIF Inc Basil_EtOH 10miG/mL 2	Acquired By:	Saneesh
Sample Type:	Unknown	Date Acquired:	2016/12/12 10:57:51 AM
Vial:	11	Acq. Method Set:	HDI_RIF_25ODESRIF_NEO
Injection #:	1	Date Processed:	2016/12/12 04:14:58 PM
Injection Volume:	15.00 ul	Processing Method:	HDI_RIF_ W2996 254.0nm-1.2
Run Time:	11.5 Minutes	Channel Name:	W2996 254.0nm-1.2
Sample Set Name:	RIF_Basil 2P Scrng 12_12_16	Proc. Chnl. Descr.:	W2996 PDA 254.0 nm at 1.2

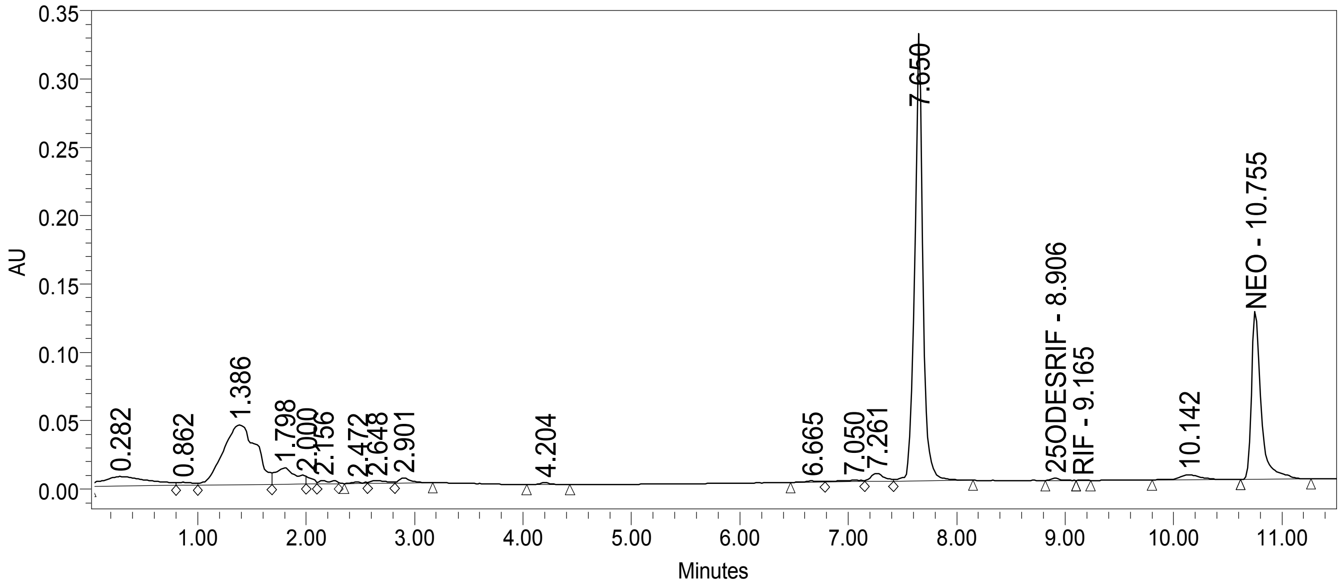

	Peak Name	RT	Area	% Area	Height
1		0.282	198961	4.92	6867
2		0.862	22286	0.55	2113
3		1.386	929487	22.98	43564
4		1.798	161545	3.99	11998
5		2.000	22584	0.56	5492
6		2.156	21055	0.52	2461
7		2.472	7604	0.19	1058
8		2.648	19219	0.48	1857
9		2.901	27910	0.69	3792
10		4.204	13064	0.32	1708
11		6.665	8517	0.21	926
12		7.050	17792	0.44	1039

	Peak Name	RT	Area	% Area	Height
13		7.261	51557	1.27	5795
14		7.650	1727804	42.71	317156
15	25ODESRIF	8.906	8915	0.22	1708
16	RIF	9.165	1203	0.03	272
17		10.142	51933	1.28	3628
18	NEO	10.755	753563	18.63	121977

Reported by User: Saneesh (Saneesh)   Project Name: HDI_Stellenbosch_2016

SAMPLE INFORMATION

Sample Name:	RIF Inc Basil_EOH 200miG/mL 1	Acquired By:	Saneesh
Sample Type:	Unknown	Date Acquired:	2016/12/12 11:12:37 AM
Vial:	12	Acq. Method Set:	HDI_RIF_25ODESRIF_NEO
Injection #:	1	Date Processed:	2016/12/12 04:14:58 PM
Injection Volume:	15.00 ul	Processing Method:	HDI_RIF_ W2996 254.0nm-1.2
Run Time:	11.5 Minutes	Channel Name:	W2996 254.0nm-1.2
Sample Set Name:	RIF_Basil 2P Scrng 12_12_16	Proc. Chnl. Descr.:	W2996 PDA 254.0 nm at 1.2

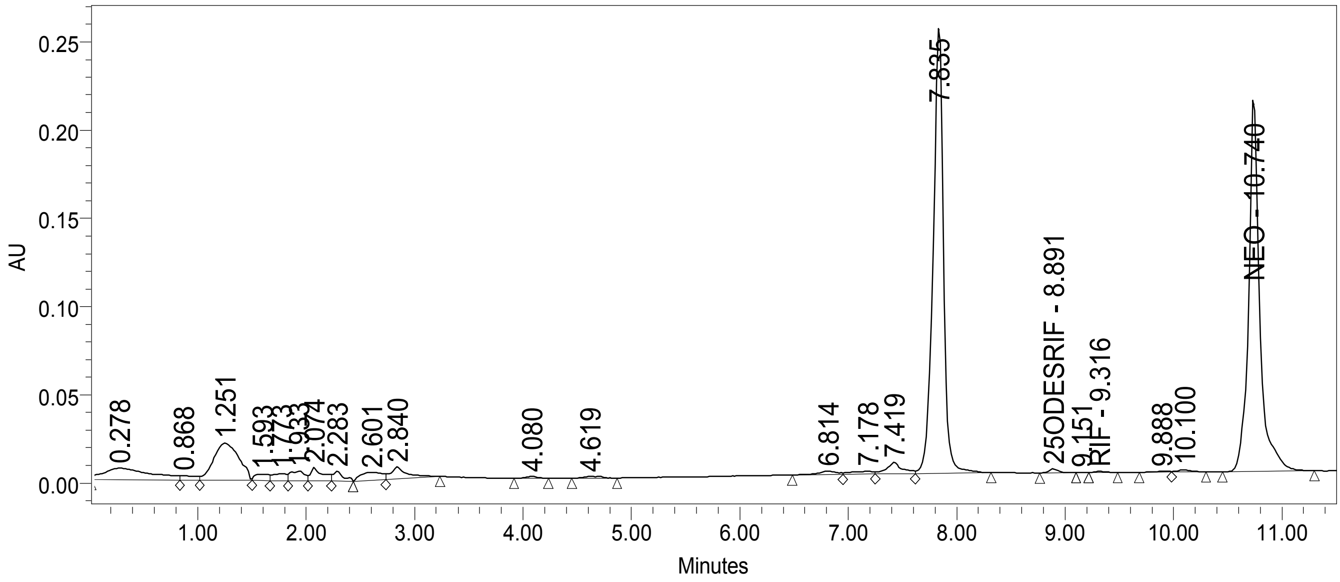

	Peak Name	RT	Area	% Area	Height
1		0.278	199038	4.69	6536
2		0.868	24814	0.58	2566
3		1.251	342923	8.07	20977
4		1.593	31997	0.75	3633
5		1.773	36895	0.87	4106
6		1.933	49529	1.17	5350
7		2.074	60244	1.42	7364
8		2.283	35833	0.84	5414
9		2.601	61709	1.45	4340
10		2.840	69630	1.64	6674
11		4.080	8119	0.19	1094
12		4.619	11338	0.27	995

Reported by User: Saneesh (Saneesh) Project Name: HDI_Stellenbosch_2016

	Peak Name	RT	Area	% Area	Height
13		6.814	23971	0.56	1959
14		7.178	23250	0.55	1708
15		7.419	62800	1.48	6367
16		7.835	1664189	39.18	251638
17	25ODESRIF	8.891	12964	0.31	2253
18		9.151	877	0.02	211
19	RIF	9.316	4969	0.12	739
20		9.888	4798	0.11	517
21		10.100	9845	0.23	1298
22	NEO	10.740	1508186	35.50	209845

SAMPLE INFORMATION

Sample Name:	RIF Inc Basill_EOH 200miG/mL 2	Acquired By:	Saneesh
Sample Type:	Unknown	Date Acquired:	2016/12/12 11:27:22 AM
Vial:	13	Acq. Method Set:	HDI_RIF_25ODESRIF_NEO
Injection #:	1	Date Processed:	2016/12/12 04:14:59 PM
Injection Volume:	15.00 ul	Processing Method:	HDI_RIF_ W2996 254.0nm-1.2
Run Time:	11.5 Minutes	Channel Name:	W2996 254.0nm-1.2
Sample Set Name:	RIF_Basil 2P Scrng 12_12_16	Proc. Chnl. Descr.:	W2996 PDA 254.0 nm at 1.2

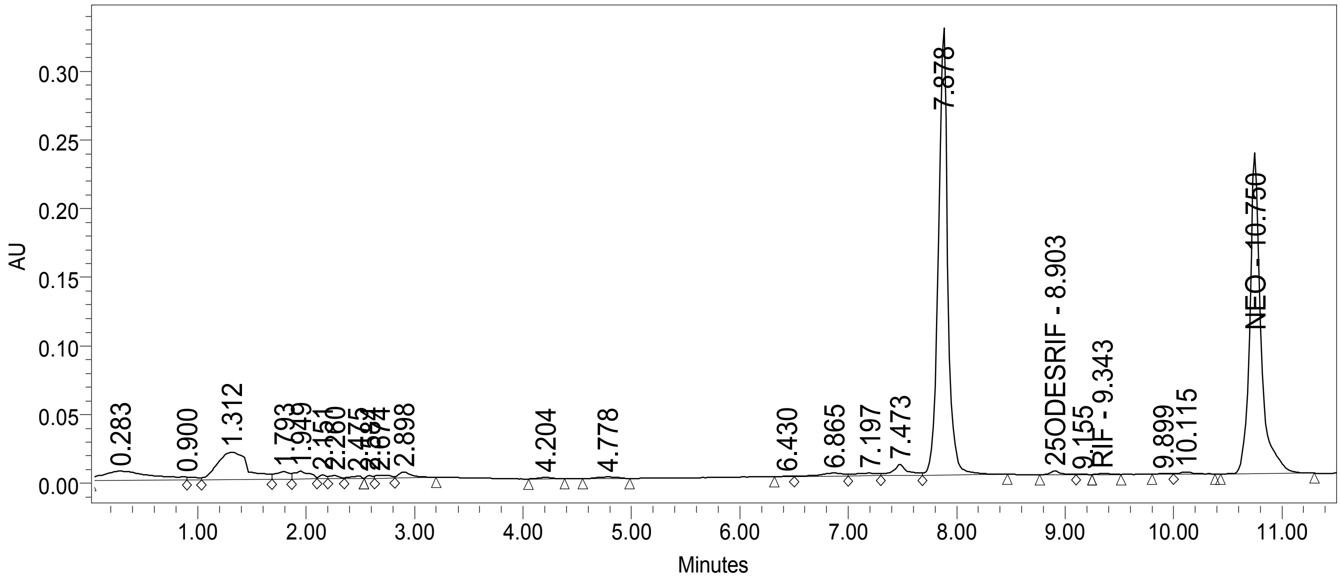

	Peak Name	RT	Area	% Area	Height
1		0.283	203280	4.39	6551
2		0.900	12814	0.28	1909
3		1.312	389432	8.42	19826
4		1.793	50167	1.08	5385
5		1.949	58564	1.27	5595
6		2.151	13070	0.28	2866
7		2.260	16395	0.35	2634
8		2.475	14450	0.31	2085
9		2.584	9369	0.20	2144
10		2.674	20577	0.44	2241
11		2.898	35629	0.77	4207
12		4.204	10319	0.22	1399

Reported by User: Saneesh (Saneesh) Project Name: HDI_Stellenbosch_2016

	Peak Name	RT	Area	% Area	Height
13		4.778	16519	0.36	1271
14		6.430	1652	0.04	232
15		6.865	32526	0.70	2332
16		7.197	27865	0.60	1977
17		7.473	75990	1.64	8110
18		7.878	1948593	42.11	321818
19	25ODESRIF	8.903	17536	0.38	2868
20		9.155	1809	0.04	371
21	RIF	9.343	6247	0.14	863
22		9.899	3239	0.07	401
23		10.115	14231	0.31	1655
24	NEO	10.750	1647019	35.59	231008

Reported by User: Saneesh (Saneesh) Project Name: HDI_Stellenbosch_2016

SAMPLE INFORMATION

Sample Name:	RIF Inc Basil_EtOAc 10miG/mL 1	Acquired By:	Saneesh
Sample Type:	Unknown	Date Acquired:	2016/12/12 11:42:09 AM
Vial:	14	Acq. Method Set:	HDI_RIF_25ODESRIF_NEO
Injection #:	1	Date Processed:	2016/12/12 04:14:59 PM
Injection Volume:	15.00 ul	Processing Method:	HDI_RIF_ W2996 254.0nm-1.2
Run Time:	11.5 Minutes	Channel Name:	W2996 254.0nm-1.2
Sample Set Name:	RIF_Basil 2P Scrng 12_12_16	Proc. Chnl. Descr.:	W2996 PDA 254.0 nm at 1.2

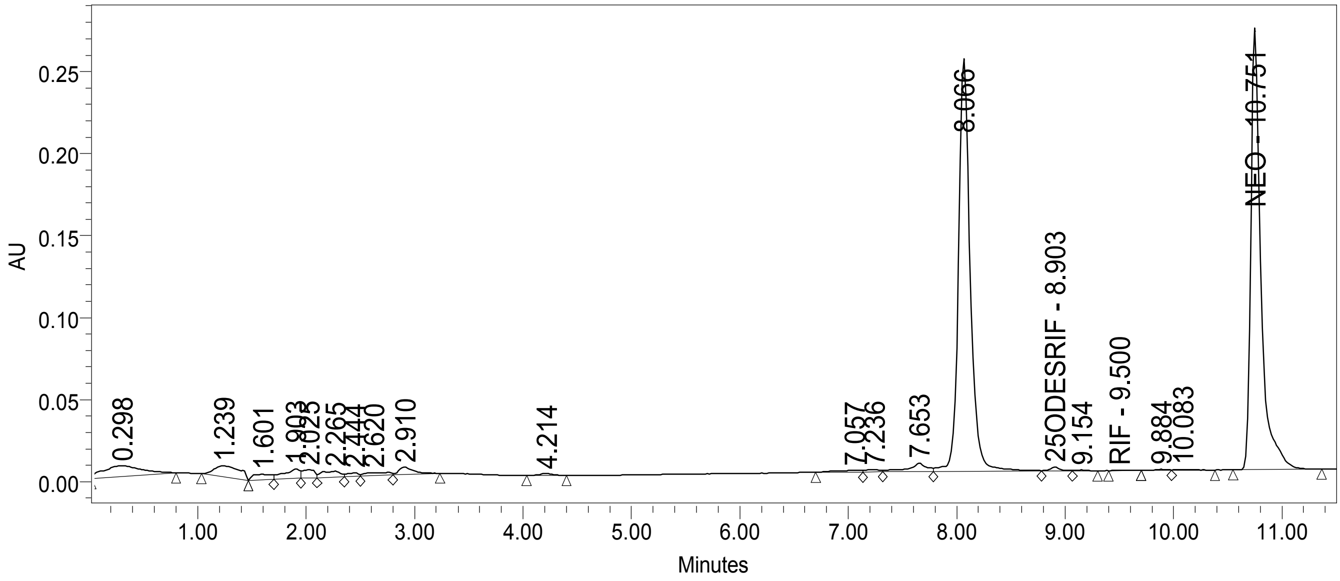

	Peak Name	RT	Area	% Area	Height
1		0.298	166684	3.87	6577
2		1.239	113039	2.63	6630
3		1.601	39293	0.91	3221
4		1.903	59351	1.38	5749
5		2.025	39725	0.92	4909
6		2.265	44226	1.03	3643
7		2.444	15625	0.36	2299
8		2.620	29228	0.68	1811
9		2.910	42478	0.99	4437
10		4.214	8890	0.21	1093
11		7.057	14542	0.34	969
12		7.236	12442	0.29	1311

Reported by User: Saneesh (Saneesh) Project Name: HDI_Stellenbosch_2016

	Peak Name	RT	Area	% Area	Height
13		7.653	60496	1.41	5140
14		8.066	1888984	43.90	250203
15	25ODESRIF	8.903	15098	0.35	2432
16		9.154	3247	0.08	498
17	RIF	9.500	2519	0.06	279
18		9.884	5353	0.12	663
19		10.083	5445	0.13	449
20	NEO	10.751	1735944	40.35	266442

SAMPLE INFORMATION

Sample Name:	RIF Inc Basil_EtOAc 10miG/mL 2	Acquired By:	Saneesh
Sample Type:	Unknown	Date Acquired:	2016/12/12 11:56:47 AM
Vial:	15	Acq. Method Set:	HDI_RIF_25ODESRIF_NEO
Injection #:	1	Date Processed:	2016/12/12 04:14:59 PM
Injection Volume:	15.00 ul	Processing Method:	HDI_RIF_ W2996 254.0nm-1.2
Run Time:	11.5 Minutes	Channel Name:	W2996 254.0nm-1.2
Sample Set Name:	RIF_Basil 2P Scrng 12_12_16	Proc. Chnl. Descr.:	W2996 PDA 254.0 nm at 1.2

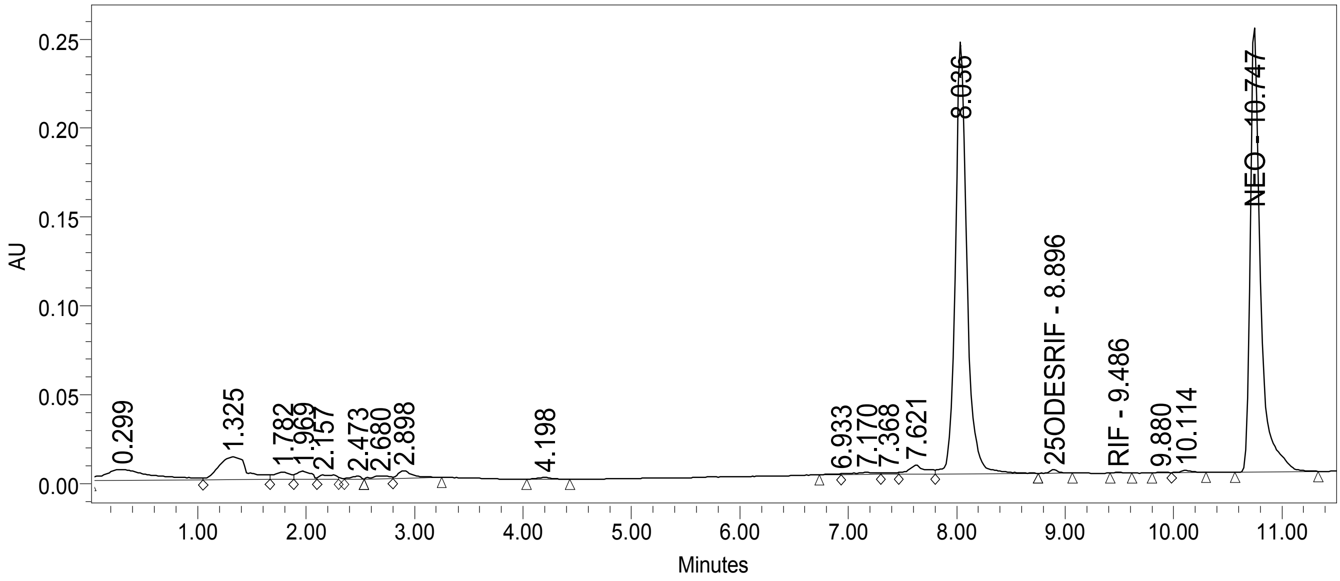

	Peak Name	RT	Area	% Area	Height
1		0.299	182991	4.40	5989
2		1.325	237276	5.70	12856
3		1.782	43514	1.05	4222
4		1.969	43620	1.05	4593
5		2.157	25104	0.60	2547
6		2.473	11459	0.28	1853
7		2.680	18686	0.45	1552
8		2.898	40719	0.98	4254
9		4.198	10049	0.24	1163
10		6.933	3656	0.09	506
11		7.170	19904	0.48	1222
12		7.368	9492	0.23	1014

	Peak Name	RT	Area	% Area	Height
13		7.621	55024	1.32	5149
14		8.036	1793199	43.08	241775
15	25ODESRIF	8.896	10029	0.24	1813
16	RIF	9.486	2567	0.06	417
17		9.880	1874	0.05	299
18		10.114	8833	0.21	1136
19	NEO	10.747	1644078	39.50	248727

SAMPLE INFORMATION

Sample Name:	RIF Inc Basil_EtOAc 200miG/mL 1	Acquired By:	Saneesh
Sample Type:	Unknown	Date Acquired:	2016/12/12 12:11:32 PM
Vial:	16	Acq. Method Set:	HDI_RIF_25ODESRIF_NEO
Injection #:	1	Date Processed:	2016/12/12 04:14:59 PM
Injection Volume:	15.00 ul	Processing Method:	HDI_RIF_ W2996 254.0nm-1.2
Run Time:	11.5 Minutes	Channel Name:	W2996 254.0nm-1.2
Sample Set Name:	RIF_Basil 2P Scrng 12_12_16	Proc. Chnl. Descr.:	W2996 PDA 254.0 nm at 1.2

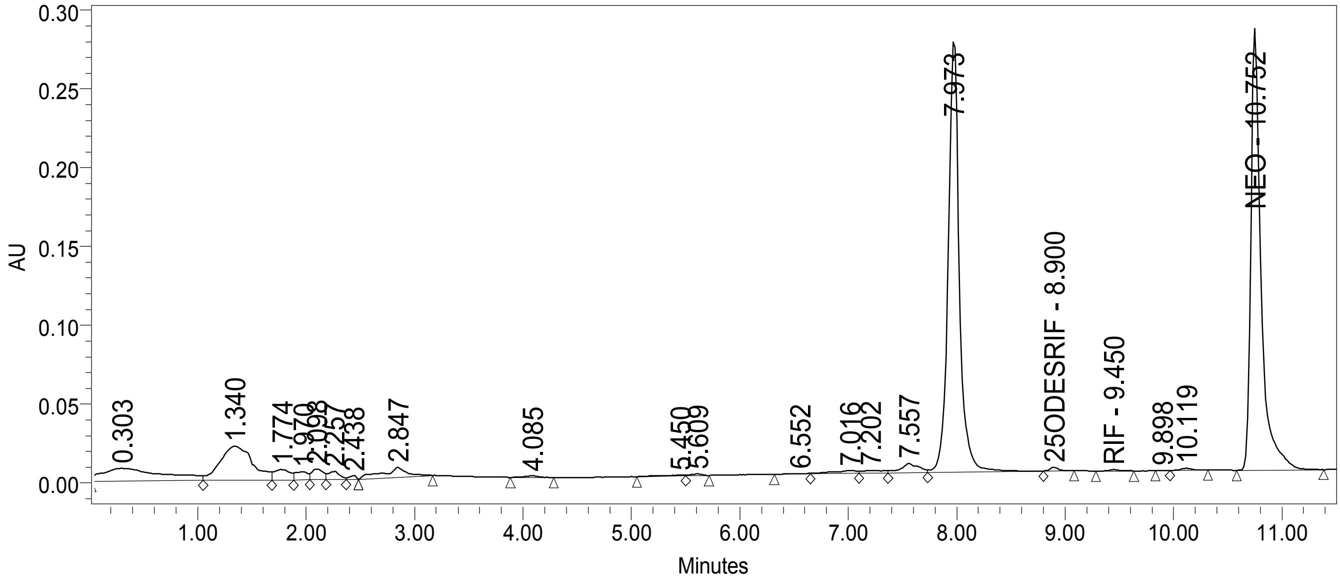

	Peak Name	RT	Area	% Area	Height
1		0.303	311552	6.17	8073
2		1.340	449765	8.91	21565
3		1.774	68795	1.36	6724
4		1.970	42799	0.85	5175
5		2.098	50576	1.00	6838
6		2.257	40264	0.80	5365
7		2.438	11476	0.23	2504
8		2.847	104976	2.08	6511
9		4.085	9161	0.18	1013
10		5.450	9459	0.19	479
11		5.609	7648	0.15	1167
12		6.552	5465	0.11	382

	Peak Name	RT	Area	% Area	Height
13		7.016	29529	0.59	1818
14		7.202	23338	0.46	1742
15		7.557	68670	1.36	5873
16		7.973	1956345	38.76	273667
17	25ODESRIF	8.900	13852	0.27	2438
18	RIF	9.450	7557	0.15	1046
19		9.898	937	0.02	185
20		10.119	10711	0.21	1382
21	NEO	10.752	1824018	36.14	277422

SAMPLE INFORMATION

Sample Name:	RIF Inc Basil_EtOAc 200miG/mL 2	Acquired By:	Saneesh
Sample Type:	Unknown	Date Acquired:	2016/12/12 12:26:19 PM
Vial:	17	Acq. Method Set:	HDI_RIF_25ODESRIF_NEO
Injection #:	1	Date Processed:	2016/12/12 04:14:59 PM
Injection Volume:	15.00 ul	Processing Method:	HDI_RIF_ W2996 254.0nm-1.2
Run Time:	11.5 Minutes	Channel Name:	W2996 254.0nm-1.2
Sample Set Name:	RIF_Basil 2P Scrng 12_12_16	Proc. Chnl. Descr.:	W2996 PDA 254.0 nm at 1.2

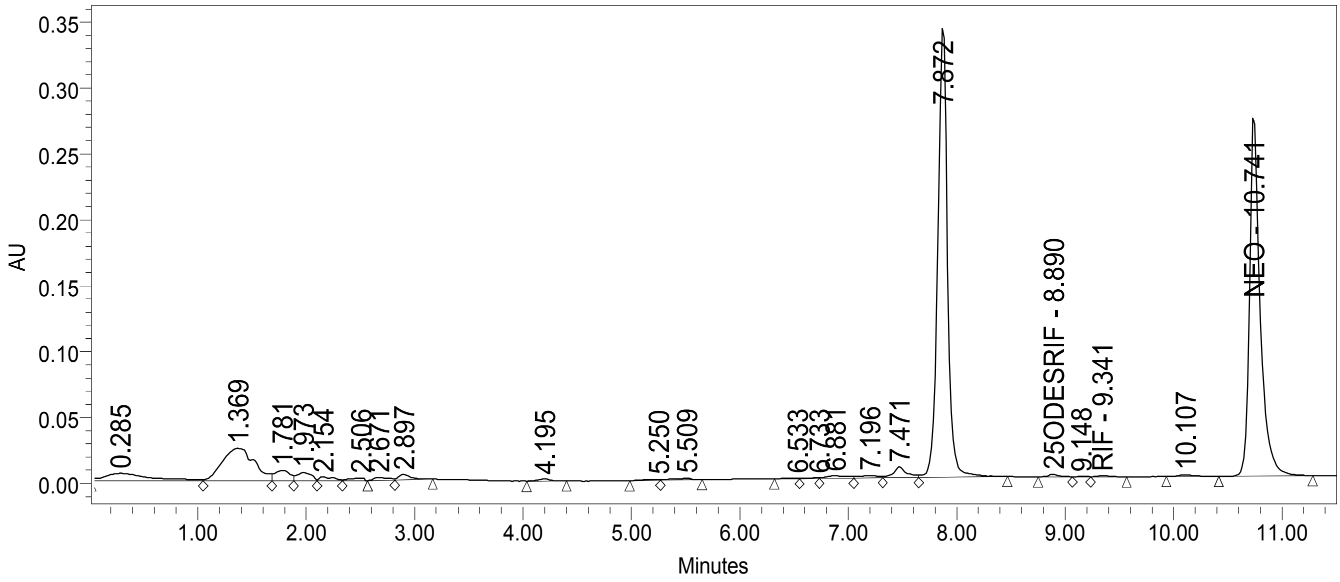

	Peak Name	RT	Area	% Area	Height
1		0.285	174069	3.42	5805
2		1.369	511268	10.04	24792
3		1.781	77148	1.52	8012
4		1.973	60223	1.18	6469
5		2.154	28866	0.57	3136
6		2.506	20403	0.40	2404
7		2.671	23214	0.46	2361
8		2.897	34217	0.67	4109
9		4.195	12007	0.24	1519
10		5.250	6601	0.13	661
11		5.509	16595	0.33	1477
12		6.533	3636	0.07	373

Reported by User: Saneesh (Saneesh) Project Name: HDI_Stellenbosch_2016

	Peak Name	RT	Area	% Area	Height
13		6.733	4369	0.09	512
14		6.881	25055	0.49	1808
15		7.196	22529	0.44	1719
16		7.471	68567	1.35	8207
17		7.872	2110699	41.46	339410
18	25ODESRIF	8.890	12821	0.25	1963
19		9.148	1802	0.04	310
20	RIF	9.341	6590	0.13	799
21		10.107	11333	0.22	1210
22	NEO	10.741	1858399	36.51	273174

Reported by User: Saneesh (Saneesh) Project Name: HDI_Stellenbosch_2016

SAMPLE INFORMATION

Sample Name:	RIF NELF Control Incubate K1	Acquired By:	Saneesh
Sample Type:	Unknown	Date Acquired:	2016/12/22 02:48:55 PM
Vial:	2	Acq. Method Set:	HDI_RIF_25ODESRIF_NEO
Injection #:	1	Date Processed:	2016/12/23 09:43:41 AM
Injection Volume:	15.00 ul	Processing Method:	HDI_RIF_ W2996 254.0nm-1.2
Run Time:	12.5 Minutes	Channel Name:	W2996 254.0nm-1.2
Sample Set Name:	RIF NELF Inhibitr_Asy 22_12_16	Proc. Chnl. Descr.:	W2996 PDA 254.0 nm at 1.2

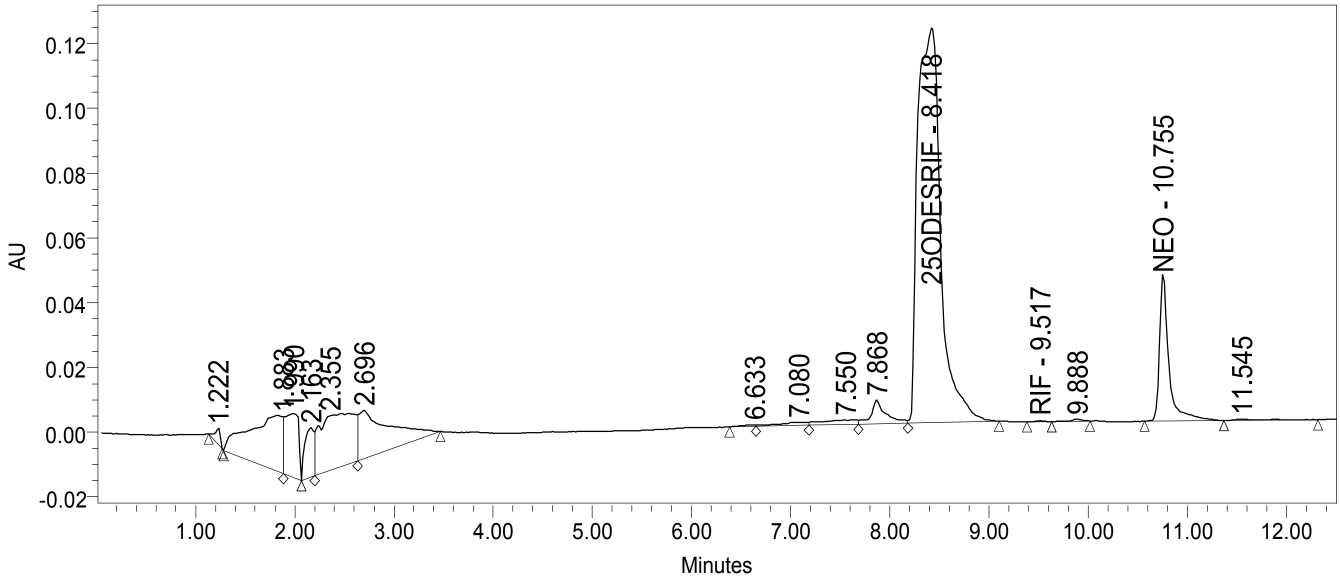

	Peak Name	RT	Area	% Area	Height
1		1.222	18959	0.50	4901
2		1.883	381267	10.02	17466
3		1.990	188436	4.95	19727
4		2.163	97069	2.55	15057
5		2.355	403082	10.60	16937
6		2.696	319701	8.40	14845
7		6.633	4822	0.13	510
8		7.080	20330	0.53	908
9		7.550	34652	0.91	1341
10		7.868	74742	1.96	7172
11	25ODESRIF	8.418	1946957	51.18	121736
12	RIF	9.517	2293	0.06	357
13		9.888	5262	0.14	632
14	NEO	10.755	299227	7.87	44977

	Peak Name	RT	Area	% Area	Height
15		11.545	7468	0.20	340

SAMPLE INFORMATION

Sample Name:	RIF NELF Inc 1miM 1	Acquired By:	Saneesh
Sample Type:	Unknown	Date Acquired:	2016/12/22 03:04:50 PM
Vial:	3	Acq. Method Set:	HDI_RIF_25ODESRIF_NEO
Injection #:	1	Date Processed:	2016/12/23 09:43:41 AM
Injection Volume:	15.00 ul	Processing Method:	HDI_RIF_ W2996 254.0nm-1.2
Run Time:	12.5 Minutes	Channel Name:	W2996 254.0nm-1.2
Sample Set Name:	RIF NELF Inhibitr_Asy 22_12_16	Proc. Chnl. Descr.:	W2996 PDA 254.0 nm at 1.2

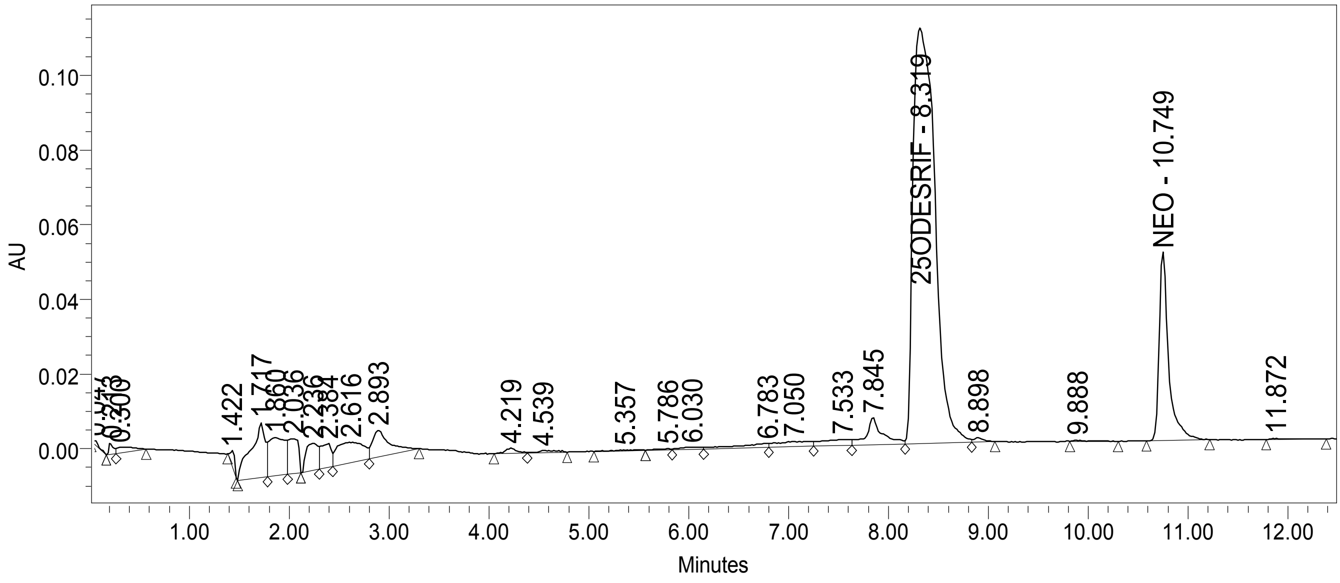

	Peak Name	RT	Area	% Area	Height
1		0.047	5065	0.17	1062
2		0.213	9980	0.34	2609
3		0.300	15914	0.54	1449
4		1.422	13770	0.47	4428
5		1.717	150165	5.14	14319
6		1.860	116780	4.00	10227
7		2.036	66838	2.29	9383
8		2.236	60012	2.05	7258
9		2.384	46432	1.59	6426
10		2.616	104620	3.58	5472
11		2.893	84974	2.91	7128
12		4.219	11063	0.38	1368

	Peak Name	RT	Area	% Area	Height
13		4.539	7456	0.26	522
14		5.357	4913	0.17	198
15		5.786	4393	0.15	355
16		6.030	9354	0.32	549
17		6.783	25554	0.87	1102
18		7.050	32927	1.13	1326
19		7.533	33388	1.14	1646
20		7.845	80184	2.74	7088
21	25ODESRIF	8.319	1687933	57.78	111300
22		8.898	8020	0.27	1180
23	RIF	9.400			
24		9.888	3256	0.11	273
25	NEO	10.749	334243	11.44	50017
26		11.872	3950	0.14	278

Reported by User: Saneesh (Saneesh)   Project Name: HDI_Stellenbosch_2016

SAMPLE INFORMATION

Sample Name:	RIF NELF Inc 10miM 1	Acquired By:	Saneesh
Sample Type:	Unknown	Date Acquired:	2016/12/22 03:20:35 PM
Vial:	4	Acq. Method Set:	HDI_RIF_25ODESRIF_NEO
Injection #:	1	Date Processed:	2016/12/23 09:43:41 AM
Injection Volume:	15.00 ul	Processing Method:	HDI_RIF_ W2996 254.0nm-1.2
Run Time:	12.5 Minutes	Channel Name:	W2996 254.0nm-1.2
Sample Set Name:	RIF NELF Inhibitr_Asy 22_12_16	Proc. Chnl. Descr.:	W2996 PDA 254.0 nm at 1.2

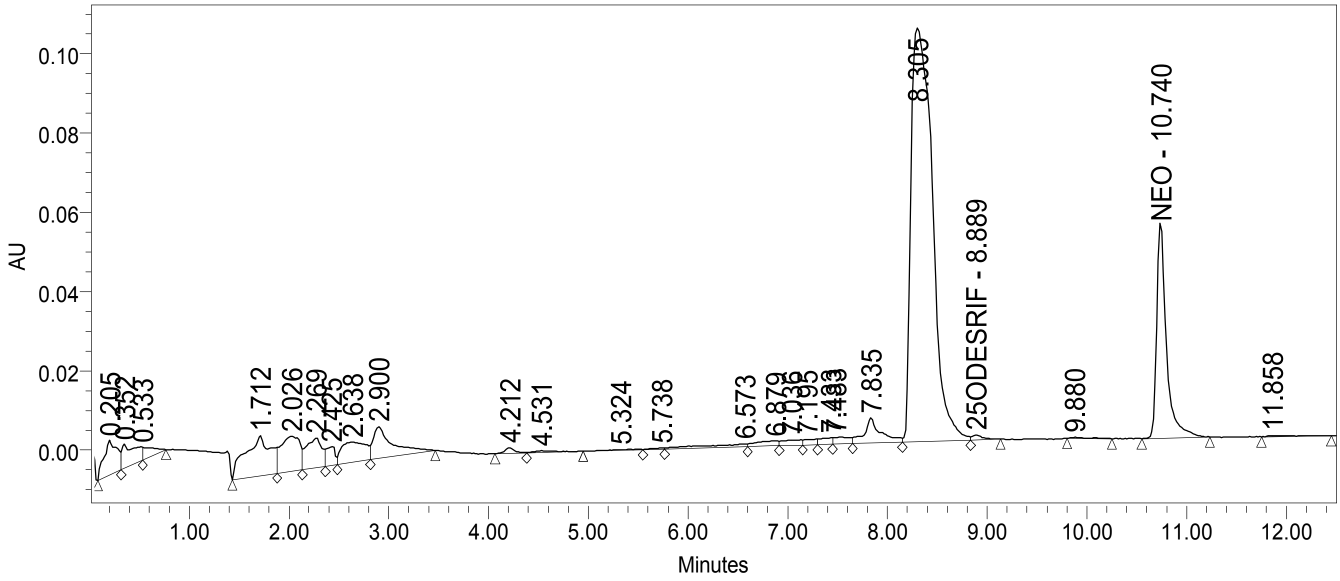

	Peak Name	RT	Area	% Area	Height
1		0.205	78855	2.72	8555
2		0.352	55942	1.93	5896
3		0.533	22384	0.77	3185
4		1.712	179075	6.19	9971
5		2.026	115031	3.97	8755
6		2.269	83667	2.89	7433
7		2.425	27968	0.97	5001
8		2.638	87741	3.03	5124
9		2.900	108113	3.73	8011
10		4.212	9439	0.33	1411
11		4.531	5723	0.20	416
12		5.324	4058	0.14	197

Reported by User: Saneesh (Saneesh) Project Name: HDI_Stellenbosch_2016

	Peak Name	RT	Area	% Area	Height
13		5.738	2691	0.09	277
14		6.573	24712	0.85	679
15		6.879	19670	0.68	1207
16		7.036	17941	0.62	1372
17		7.195	11978	0.41	1363
18		7.433	13500	0.47	1606
19		7.483	19297	0.67	1667
20		7.835	72485	2.50	6329
21		8.305	1552283	53.62	104532
22	25ODESRIF	8.889	9566	0.33	1292
23	RIF	9.400			
24		9.880	3103	0.11	286
25	NEO	10.740	365105	12.61	54257
26		11.858	4734	0.16	313

Reported by User: Saneesh (Saneesh) Project Name: HDI_Stellenbosch_2016

SAMPLE INFORMATION

Sample Name:	RIF NELF Inc 25miM 1	Acquired By:	Saneesh
Sample Type:	Unknown	Date Acquired:	2016/12/22 03:36:18 PM
Vial:	5	Acq. Method Set:	HDI_RIF_25ODESRIF_NEO
Injection #:	1	Date Processed:	2016/12/23 09:43:41 AM
Injection Volume:	15.00 ul	Processing Method:	HDI_RIF_ W2996 254.0nm-1.2
Run Time:	12.5 Minutes	Channel Name:	W2996 254.0nm-1.2
Sample Set Name:	RIF NELF Inhibitr_Asy 22_12_16	Proc. Chnl. Descr.:	W2996 PDA 254.0 nm at 1.2

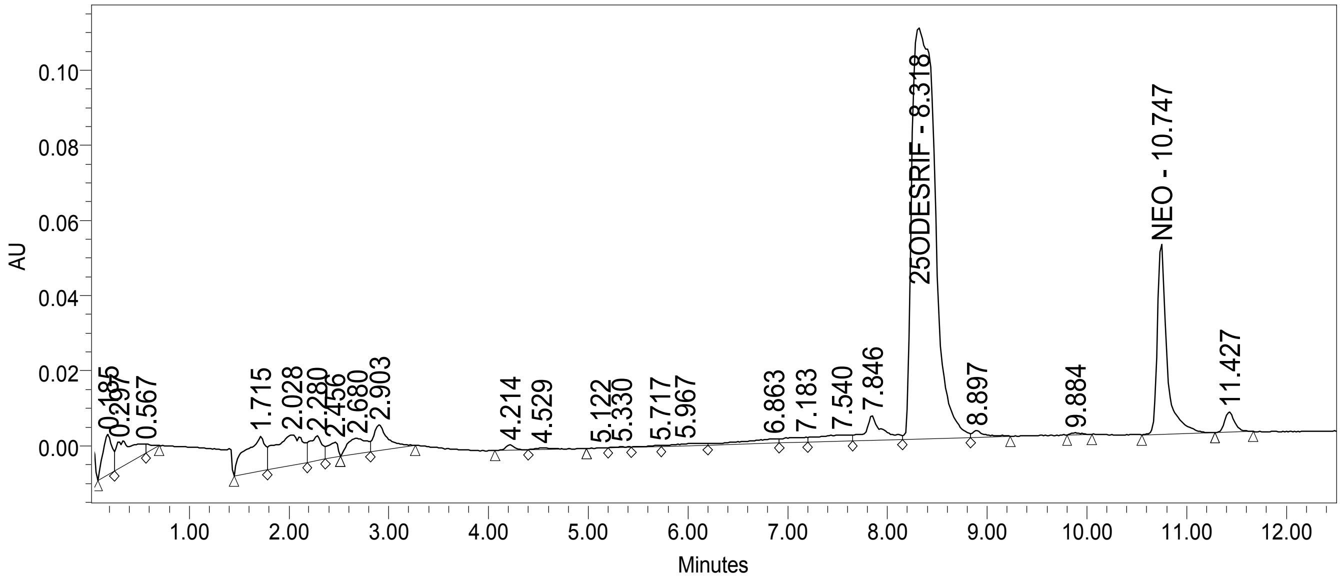

	Peak Name	RT	Area	% Area	Height
1		0.185	68610	2.26	10615
2		0.297	87812	2.90	6863
3		0.567	9202	0.30	2358
4		1.715	130041	4.29	9136
5		2.028	160953	5.31	8147
6		2.280	57138	1.89	6528
7		2.456	28685	0.95	4165
8		2.680	54893	1.81	4064
9		2.903	69265	2.29	6890
10		4.214	9814	0.32	1421
11		4.529	6354	0.21	443
12		5.122	1050	0.03	121

Reported by User: Saneesh (Saneesh) Project Name: HDI_Stellenbosch_2016

	Peak Name	RT	Area	% Area	Height
13		5.330	1988	0.07	196
14		5.717	2463	0.08	263
15		5.967	11839	0.39	541
16		6.863	31209	1.03	1182
17		7.183	21339	0.70	1349
18		7.540	40432	1.33	1678
19		7.846	76726	2.53	6515
20	25ODESRIF	8.318	1763720	58.22	109353
21		8.897	15691	0.52	1773
22	RIF	9.400			
23		9.884	3279	0.11	501
24	NEO	10.747	338779	11.18	50205
25		11.427	37959	1.25	5400

Reported by User: Saneesh (Saneesh)   Project Name: HDI_Stellenbosch_2016

SAMPLE INFORMATION

Sample Name:	RIF NELF Inc 25miM 2	Acquired By:	Saneesh
Sample Type:	Unknown	Date Acquired:	2016/12/22 03:52:03 PM
Vial:	6	Acq. Method Set:	HDI_RIF_25ODESRIF_NEO
Injection #:	1	Date Processed:	2016/12/23 09:43:41 AM
Injection Volume:	15.00 ul	Processing Method:	HDI_RIF_ W2996 254.0nm-1.2
Run Time:	12.5 Minutes	Channel Name:	W2996 254.0nm-1.2
Sample Set Name:	RIF NELF Inhibitr_Asy 22_12_16	Proc. Chnl. Descr.:	W2996 PDA 254.0 nm at 1.2

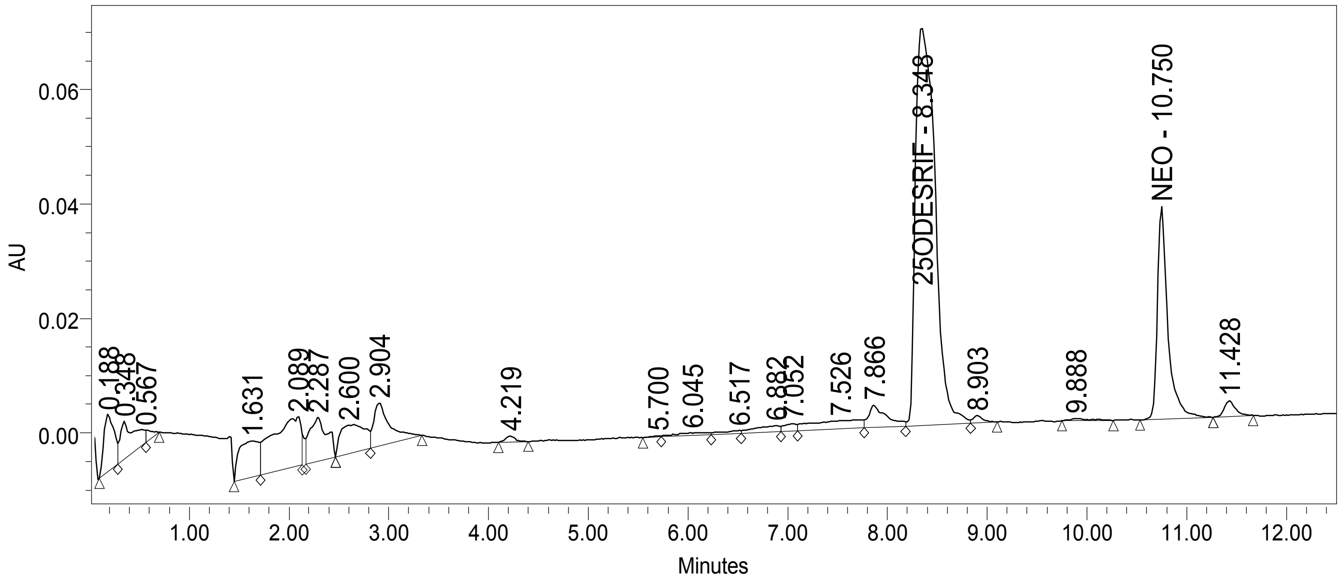

	Peak Name	RT	Area	% Area	Height
1		0.188	71434	3.42	9888
2		0.348	67548	3.24	6421
3		0.567	8288	0.40	2122
4		1.631	88126	4.22	6260
5		2.089	176876	8.47	8614
6		2.287	93330	4.47	7566
7		2.600	85480	4.09	4960
8		2.904	86179	4.13	7604
9		4.219	7241	0.35	1093
10		5.700	1660	0.08	249
11		6.045	11290	0.54	468
12		6.517	7623	0.37	544

Reported by User: Saneesh (Saneesh) Project Name: HDI_Stellenbosch_2016

	Peak Name	RT	Area	% Area	Height
13		6.882	20395	0.98	1108
14		7.052	11653	0.56	1250
15		7.526	53926	2.58	1475
16		7.866	48499	2.32	3902
17	25ODESRIF	8.348	945819	45.31	69401
18		8.903	8955	0.43	1300
19	RIF	9.400			
20		9.888	4614	0.22	329
21	NEO	10.750	267787	12.83	36886
22		11.428	20860	1.00	2704

Reported by User: Saneesh (Saneesh) Project Name: HDI_Stellenbosch_2016

SAMPLE INFORMATION

Sample Name:	RIF NELF Inc 50miM 1	Acquired By:	Saneesh
Sample Type:	Unknown	Date Acquired:	2016/12/22 04:08:45 PM
Vial:	7	Acq. Method Set:	HDI_RIF_25ODESRIF_NEO
Injection #:	1	Date Processed:	2016/12/23 09:43:41 AM
Injection Volume:	15.00 ul	Processing Method:	HDI_RIF_ W2996 254.0nm-1.2
Run Time:	12.5 Minutes	Channel Name:	W2996 254.0nm-1.2
Sample Set Name:	RIF NELF Inhibitr_Asy 22_12_16	Proc. Chnl. Descr.:	W2996 PDA 254.0 nm at 1.2

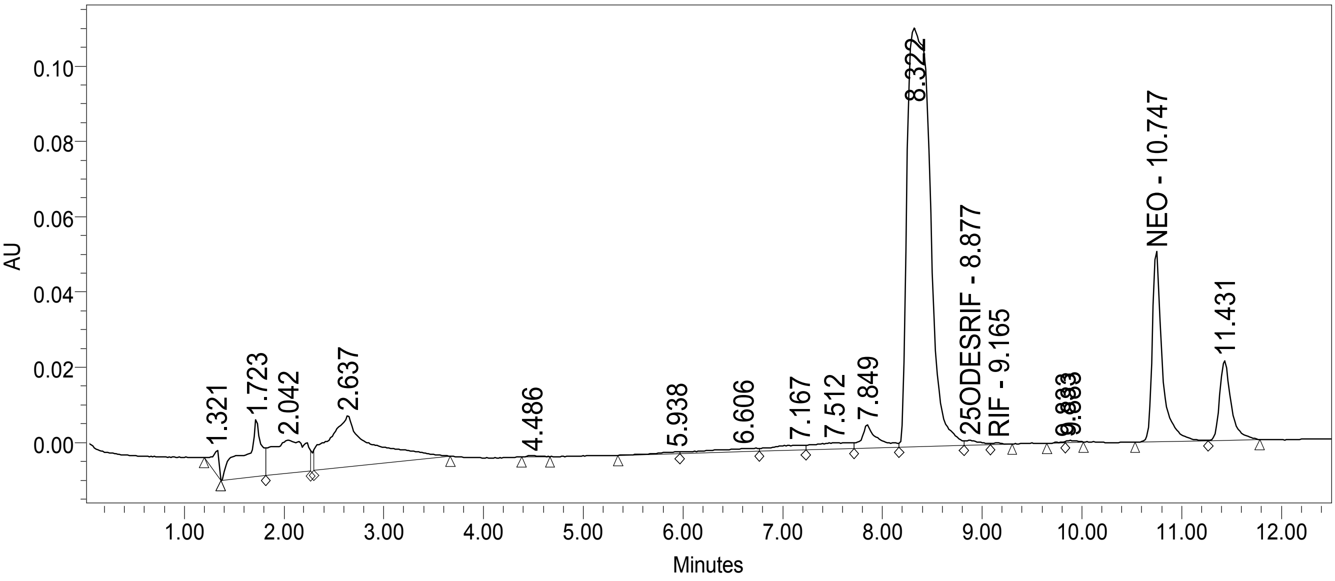

	Peak Name	RT	Area	% Area	Height
1		1.321	30317	0.91	6426
2		1.723	179653	5.40	14501
3		2.042	206890	6.22	8798
4		2.637	414605	12.46	13504
5		4.486	2987	0.09	320
6		5.938	9204	0.28	495
7		6.606	31218	0.94	823
8		7.167	31202	0.94	1330
9		7.512	43680	1.31	1646
10		7.849	67830	2.04	6119
11		8.322	1765237	53.05	111349
12	25ODESRIF	8.877	12613	0.38	1299

	Peak Name	RT	Area	% Area	Height
13	RIF	9.165	2719	0.08	430
14		9.833	1312	0.04	243
15		9.883	3650	0.11	586
16	NEO	10.747	352953	10.61	50293
17		11.431	171340	5.15	21171

Reported by User: Saneesh (Saneesh)   Project Name: HDI_Stellenbosch_2016

SAMPLE INFORMATION

Sample Name:	RIF NELF Inc 75miM 1	Acquired By:	Saneesh
Sample Type:	Unknown	Date Acquired:	2016/12/22 04:24:28 PM
Vial:	8	Acq. Method Set:	HDI_RIF_25ODESRIF_NEO
Injection #:	1	Date Processed:	2016/12/23 09:43:41 AM
Injection Volume:	15.00 ul	Processing Method:	HDI_RIF_ W2996 254.0nm-1.2
Run Time:	12.5 Minutes	Channel Name:	W2996 254.0nm-1.2
Sample Set Name:	RIF NELF Inhibitr_Asy 22_12_16	Proc. Chnl. Descr.:	W2996 PDA 254.0 nm at 1.2

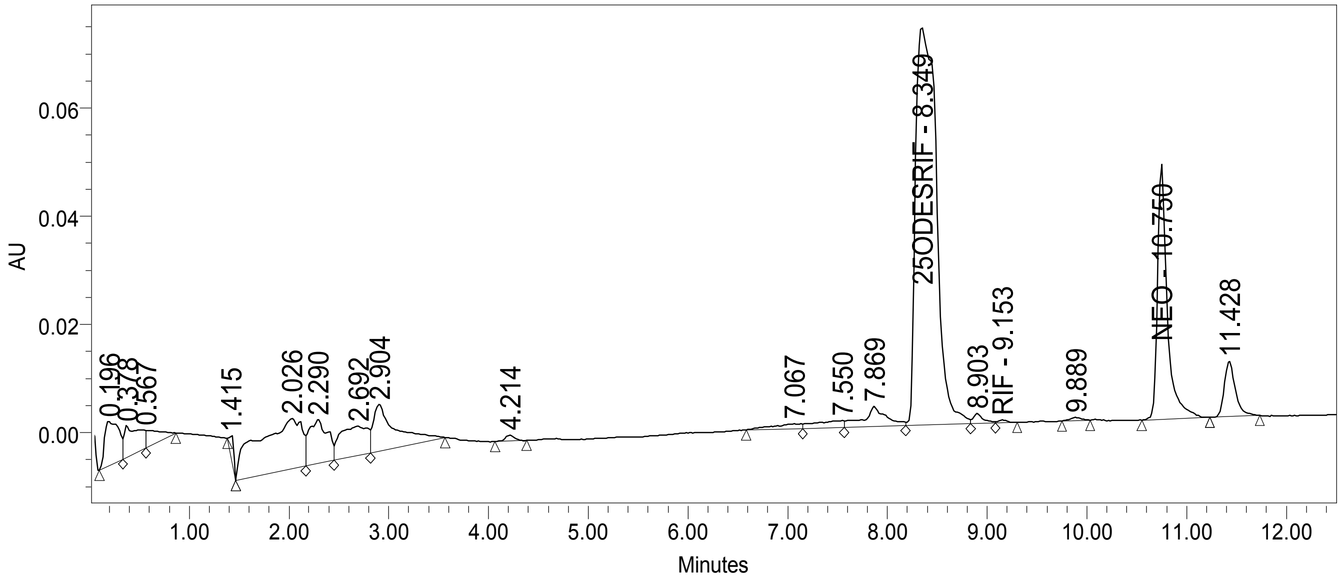

	Peak Name	RT	Area	% Area	Height
1		0.196	80048	3.32	8333
2		0.378	59455	2.46	5558
3		0.567	28554	1.18	3212
4		1.415	12664	0.52	3753
5		2.026	291730	12.09	9303
6		2.290	105487	4.37	8055
7		2.692	106101	4.40	5381
8		2.904	134789	5.59	8643
9		4.214	7210	0.30	1133
10		7.067	18784	0.78	879
11		7.550	25665	1.06	1260
12		7.869	58898	2.44	3660

	Peak Name	RT	Area	% Area	Height
13	25ODESRIF	8.349	1064138	44.10	73475
14		8.903	13446	0.56	1886
15	RIF	9.153	3572	0.15	578
16		9.889	3756	0.16	515
17	NEO	10.750	319004	13.22	46665
18		11.428	79859	3.31	10216

Reported by User: Saneesh (Saneesh) Project Name: HDI_Stellenbosch_2016

SAMPLE INFORMATION

Sample Name:	RIF NELF Inc 75miM 2	Acquired By:	Saneesh
Sample Type:	Unknown	Date Acquired:	2016/12/22 04:40:13 PM
Vial:	9	Acq. Method Set:	HDI_RIF_25ODESRIF_NEO
Injection #:	1	Date Processed:	2016/12/23 09:43:41 AM
Injection Volume:	15.00 ul	Processing Method:	HDI_RIF_ W2996 254.0nm-1.2
Run Time:	12.5 Minutes	Channel Name:	W2996 254.0nm-1.2
Sample Set Name:	RIF NELF Inhibitr_Asy 22_12_16	Proc. Chnl. Descr.:	W2996 PDA 254.0 nm at 1.2

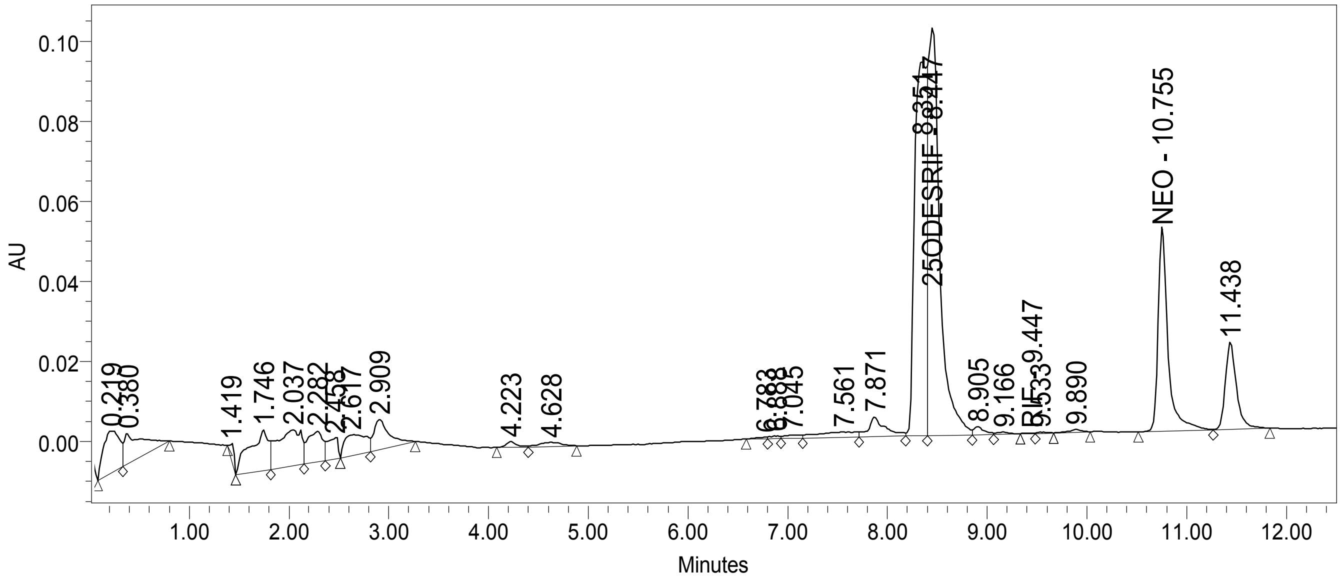

	Peak Name	RT	Area	% Area	Height
1		0.219	117287	3.79	10543
2		0.380	100313	3.24	7374
3		1.419	13292	0.43	4103
4		1.746	136505	4.41	9955
5		2.037	153644	4.97	9003
6		2.282	83623	2.70	7711
7		2.458	39487	1.28	5766
8		2.617	77091	2.49	5256
9		2.909	81259	2.63	7534
10		4.223	10360	0.33	1422
11		4.628	17858	0.58	1066
12		6.783	2889	0.09	429

Reported by User: Saneesh (Saneesh) Project Name: HDI_Stellenbosch_2016

	Peak Name	RT	Area	% Area	Height
13		6.888	4191	0.14	611
14		7.045	9398	0.30	812
15		7.561	36524	1.18	1275
16		7.871	56967	1.84	4942
17		8.351	763555	24.68	93296
18	25ODESRIF	8.447	843399	27.26	101899
19		8.905	14738	0.48	2023
20		9.166	5059	0.16	551
21	RIF	9.447	1353	0.04	196
22		9.533	1918	0.06	294
23		9.890	6086	0.20	719
24	NEO	10.755	347886	11.24	50906
25		11.438	169533	5.48	21717

Reported by User: Saneesh (Saneesh) Project Name: HDI_Stellenbosch_2016

SAMPLE INFORMATION

Sample Name:	RIF NELF Inc 100miM 1	Acquired By:	Saneesh
Sample Type:	Unknown	Date Acquired:	2016/12/22 04:55:57 PM
Vial:	10	Acq. Method Set:	HDI_RIF_25ODESRIF_NEO
Injection #:	1	Date Processed:	2016/12/23 09:43:41 AM
Injection Volume:	15.00 ul	Processing Method:	HDI_RIF_ W2996 254.0nm-1.2
Run Time:	12.5 Minutes	Channel Name:	W2996 254.0nm-1.2
Sample Set Name:	RIF NELF Inhibitr_Asy 22_12_16	Proc. Chnl. Descr.:	W2996 PDA 254.0 nm at 1.2

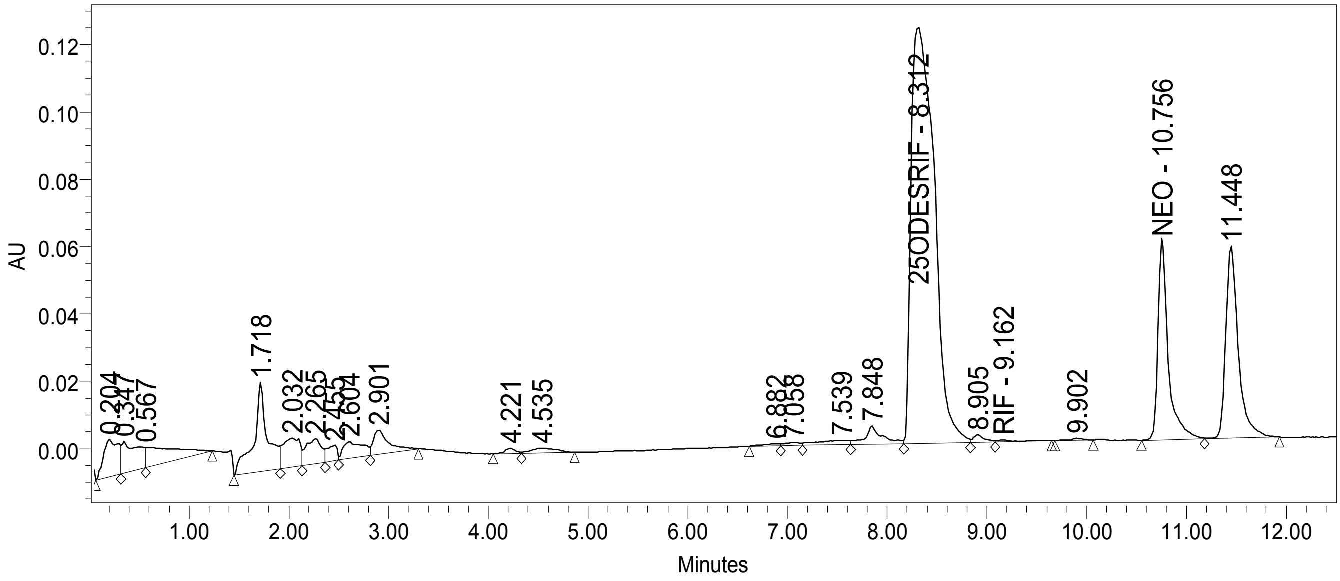

	Peak Name	RT	Area	% Area	Height
1		0.204	111615	2.77	11014
2		0.347	107203	2.66	9135
3		0.567	120461	2.99	6004
4		1.718	252279	6.26	26097
5		2.032	98880	2.45	8489
6		2.265	80707	2.00	7355
7		2.455	31501	0.78	4806
8		2.604	68391	1.70	5003
9		2.901	77833	1.93	7273
10		4.221	11337	0.28	1556
11		4.535	23828	0.59	1422
12		6.882	6664	0.17	573

	Peak Name	RT	Area	% Area	Height
13		7.058	8929	0.22	784
14		7.539	28485	0.71	1249
15		7.848	64322	1.60	5314
16	25ODESRIF	8.312	1987826	49.34	123478
17		8.905	17131	0.43	2175
18	RIF	9.162	6741	0.17	570
19		9.902	5673	0.14	567
20	NEO	10.756	421000	10.45	59755
21		11.448	497674	12.35	56832

Reported by User: Saneesh (Saneesh)   Project Name: HDI_Stellenbosch_2016

SAMPLE INFORMATION

Sample Name:	RIF NELF Inc 150miM 1	Acquired By:	Saneesh
Sample Type:	Unknown	Date Acquired:	2016/12/22 05:11:40 PM
Vial:	11	Acq. Method Set:	HDI_RIF_25ODESRIF_NEO
Injection #:	1	Date Processed:	2016/12/23 09:43:41 AM
Injection Volume:	15.00 ul	Processing Method:	HDI_RIF_ W2996 254.0nm-1.2
Run Time:	12.5 Minutes	Channel Name:	W2996 254.0nm-1.2
Sample Set Name:	RIF NELF Inhibitr_Asy 22_12_16	Proc. Chnl. Descr.:	W2996 PDA 254.0 nm at 1.2

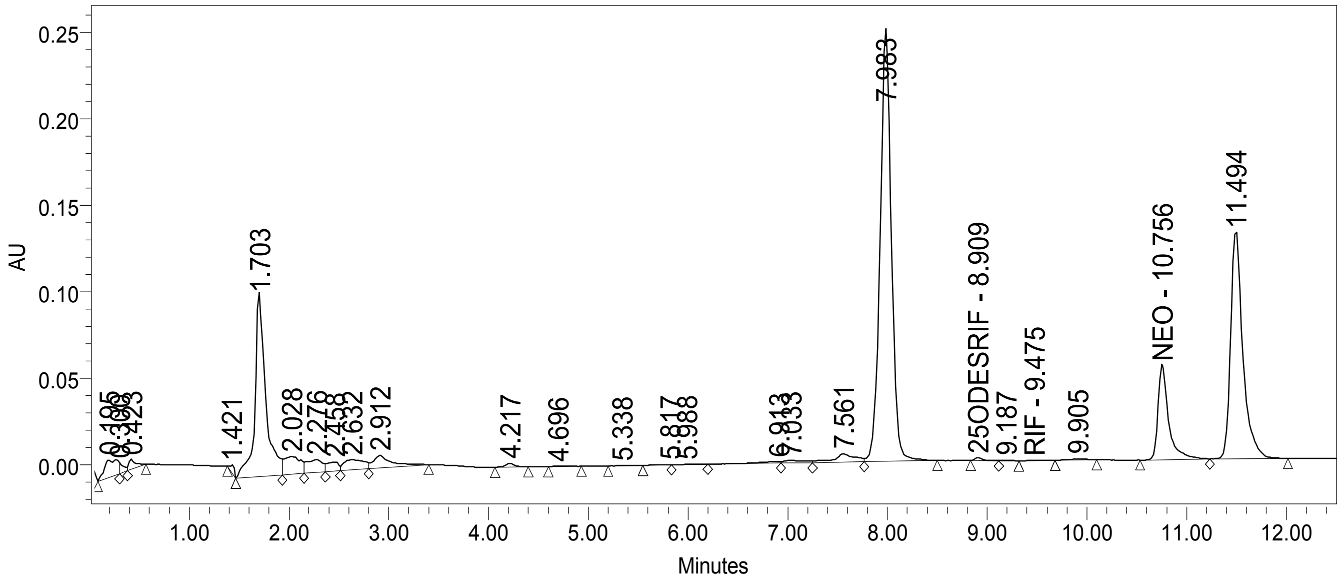

	Peak Name	RT	Area	% Area	Height
1		0.195	86632	1.72	10170
2		0.300	17325	0.34	6091
3		0.423	26507	0.53	5450
4		1.421	13654	0.27	4321
5		1.703	802378	15.97	105677
6		2.028	117216	2.33	10146
7		2.276	83259	1.66	7514
8		2.458	42599	0.85	5494
9		2.632	82109	1.63	5880
10		2.912	92609	1.84	7259
11		4.217	12849	0.26	2047
12		4.696	1729	0.03	222

	Peak Name	RT	Area	% Area	Height
13		5.338	1245	0.02	84
14		5.817	1580	0.03	163
15		5.988	4764	0.09	288
16		6.913	19263	0.38	921
17		7.033	21797	0.43	1414
18		7.561	71195	1.42	4853
19		7.983	1972802	39.27	249458
20	25ODESRIF	8.909	10558	0.21	1783
21		9.187	2172	0.04	370
22	RIF	9.475	2648	0.05	242
23		9.905	9117	0.18	713
24	NEO	10.756	401777	8.00	55146
25		11.494	1126435	22.42	131469

SAMPLE INFORMATION

Sample Name:	RIF NELF Inc 200miM 1	Acquired By:	Saneesh
Sample Type:	Unknown	Date Acquired:	2016/12/22 05:27:25 PM
Vial:	12	Acq. Method Set:	HDI_RIF_25ODESRIF_NEO
Injection #:	1	Date Processed:	2016/12/23 09:43:41 AM
Injection Volume:	15.00 ul	Processing Method:	HDI_RIF_ W2996 254.0nm-1.2
Run Time:	12.5 Minutes	Channel Name:	W2996 254.0nm-1.2
Sample Set Name:	RIF NELF Inhibitr_Asy 22_12_16	Proc. Chnl. Descr.:	W2996 PDA 254.0 nm at 1.2

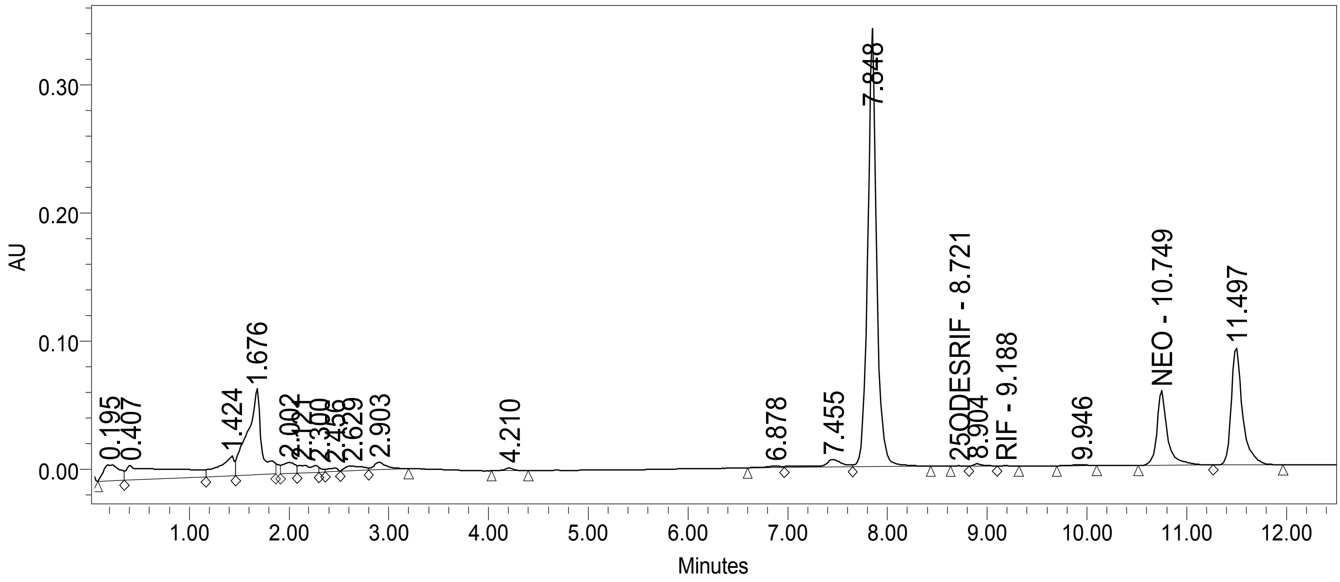

	Peak Name	RT	Area	% Area	Height
1		0.195	139623	2.79	12401
2		0.407	359865	7.20	11057
3		1.424	163284	3.27	14366
4		1.676	653010	13.06	66485
5		2.002	75161	1.50	8543
6		2.121	65194	1.30	5769
7		2.300	10967	0.22	3946
8		2.456	19602	0.39	2929
9		2.629	45945	0.92	3677
10		2.903	53035	1.06	5803
11		4.210	15538	0.31	2323
12		6.878	13091	0.26	1236

Reported by User: Saneesh (Saneesh) Project Name: HDI_Stellenbosch_2016

	Peak Name	RT	Area	% Area	Height
13		7.455	80576	1.61	5836
14		7.848	2130596	42.62	335985
15	25ODESRIF	8.721	1876	0.04	315
16		8.904	11798	0.24	1992
17	RIF	9.188	2370	0.05	381
18		9.946	8171	0.16	566
19	NEO	10.749	426752	8.54	57678
20		11.497	722483	14.45	91163

SAMPLE INFORMATION

Sample Name:	RIF NELF Control Incubate K1	Acquired By:	Saneesh
Sample Type:	Unknown	Date Acquired:	2016/12/22 05:59:24 PM
Vial:	2	Acq. Method Set:	HDI_RIF_25ODESRIF_NEO
Injection #:	1	Date Processed:	2016/12/23 09:45:08 AM
Injection Volume:	15.00 ul	Processing Method:	HDI_RIF_ W2996 254.0nm-1.2
Run Time:	12.5 Minutes	Channel Name:	W2996 254.0nm-1.2
Sample Set Name:	RIF NELF Inhibtr_Asy2 22_12_16	Proc. Chnl. Descr.:	W2996 PDA 254.0 nm at 1.2

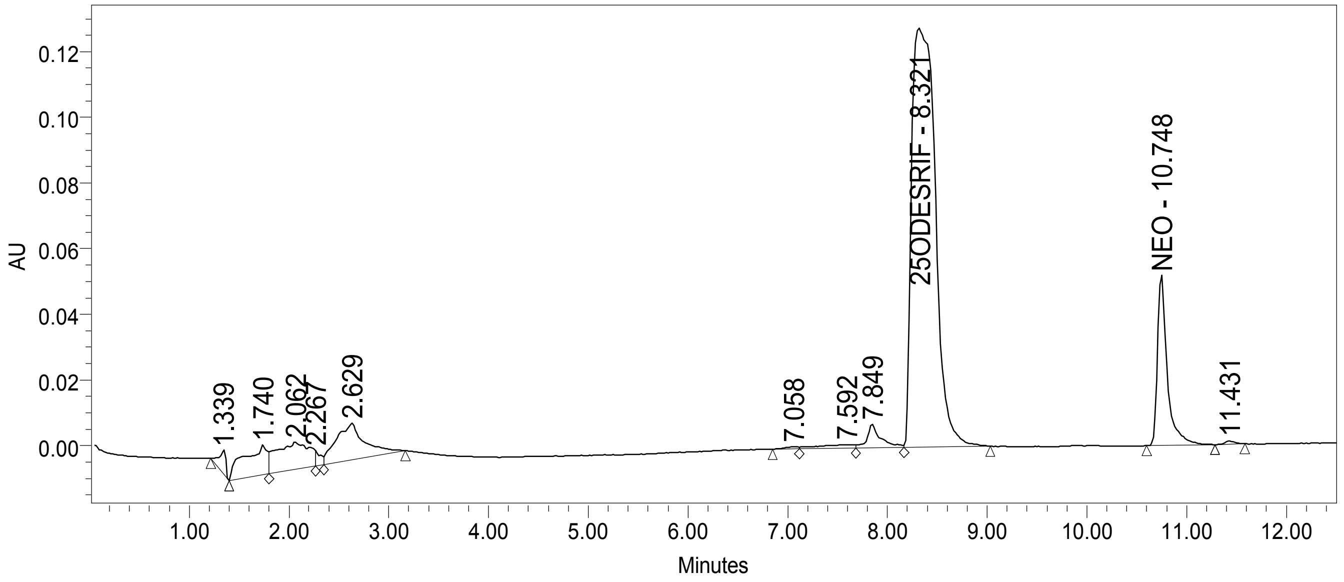

	Peak Name	RT	Area	% Area	Height
1		1.339	33014	1.04	7210
2		1.740	149843	4.71	8744
3		2.062	190444	5.99	8155
4		2.267	15881	0.50	4730
5		2.629	225907	7.10	11121
6		7.058	6297	0.20	617
7		7.592	28633	0.90	1094
8		7.849	69415	2.18	7133
9	25ODESRIF	8.321	2102653	66.13	127641
10	RIF	9.400			
11	NEO	10.748	351000	11.04	51362
12		11.431	6537	0.21	929

Reported by User: Saneesh (Saneesh)

Project Name: HDI_Stellenbosch_2016

SAMPLE INFORMATION

Sample Name:	RIF NELF Control Incubate K2	Acquired By:	Saneesh
Sample Type:	Unknown	Date Acquired:	2016/12/22 06:15:08 PM
Vial:	3	Acq. Method Set:	HDI_RIF_25ODESRIF_NEO
Injection #:	1	Date Processed:	2016/12/23 09:45:08 AM
Injection Volume:	15.00 ul	Processing Method:	HDI_RIF_ W2996 254.0nm-1.2
Run Time:	12.5 Minutes	Channel Name:	W2996 254.0nm-1.2
Sample Set Name:	RIF NELF Inhibtr_Asy2 22_12_16	Proc. Chnl. Descr.:	W2996 PDA 254.0 nm at 1.2

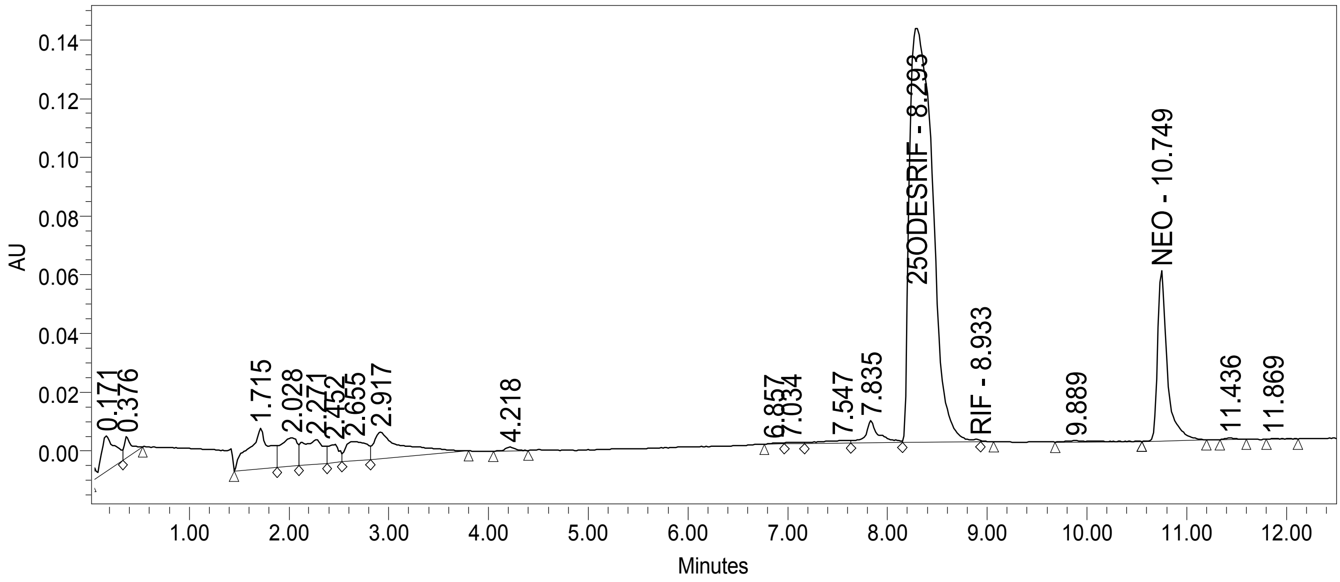

	Peak Name	RT	Area	% Area	Height
1		0.171	116373	3.15	11964
2		0.376	35570	0.96	6639
3		1.715	199512	5.41	13666
4		2.028	111881	3.03	9691
5		2.271	121325	3.29	8284
6		2.452	46498	1.26	6226
7		2.655	95681	2.59	6570
8		2.917	186060	5.04	9026
9		4.218	10095	0.27	1339
10		6.857	2254	0.06	266
11		7.034	5078	0.14	474
12		7.547	19867	0.54	963

Reported by User: Saneesh (Saneesh)   Project Name: HDI_Stellenbosch_2016

	Peak Name	RT	Area	% Area	Height
13		7.835	71634	1.94	7440
14	25ODESRIF	8.293	2265734	61.39	141668
15	RIF	8.933	1486	0.04	561
16		9.889	9011	0.24	463
17	NEO	10.749	386733	10.48	57480
18		11.436	4222	0.11	582
19		11.869	1858	0.05	176

Multi Sample Summary

Reported by User: Saneesh (Saneesh)   Project Name: HDI_Stellenbosch_2016

SAMPLE INFORMATION

Sample Name:	RIF NELF Inc 1miM 1	Acquired By:	Saneesh
Sample Type:	Unknown	Date Acquired:	2016/12/22 06:30:52 PM
Vial:	4	Acq. Method Set:	HDI_RIF_25ODESRIF_NEO
Injection #:	1	Date Processed:	2016/12/23 09:45:08 AM
Injection Volume:	15.00 ul	Processing Method:	HDI_RIF_ W2996 254.0nm-1.2
Run Time:	12.5 Minutes	Channel Name:	W2996 254.0nm-1.2
Sample Set Name:	RIF NELF Inhibtr_Asy2 22_12_16	Proc. Chnl. Descr.:	W2996 PDA 254.0 nm at 1.2

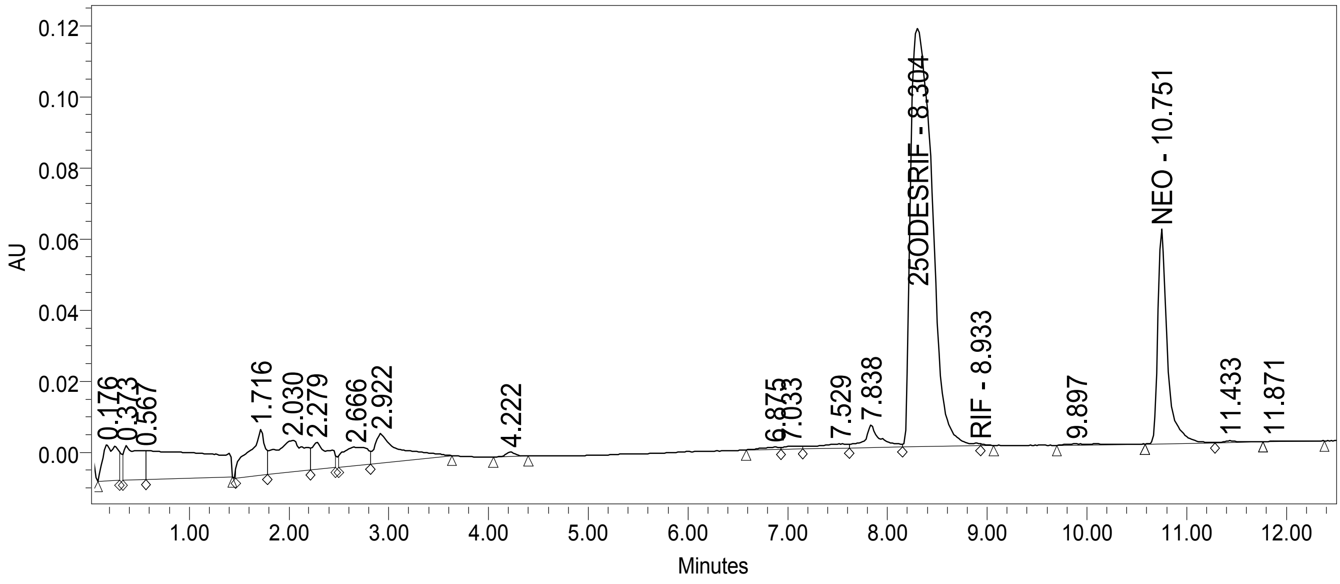

	Peak Name	RT	Area	% Area	Height
1		0.176	99593	2.82	10163
2		0.373	117131	3.32	9574
3		0.567	368456	10.45	8122
4		1.716	146839	4.16	12686
5		2.030	189535	5.37	8913
6		2.279	86671	2.46	7637
7		2.666	87625	2.48	5152
8		2.922	143407	4.07	8127
9		4.222	9252	0.26	1291
10		6.875	6991	0.20	581
11		7.033	8579	0.24	718
12		7.529	25743	0.73	1173

Reported by User: Saneesh (Saneesh) Project Name: HDI_Stellenbosch_2016

	Peak Name	RT	Area	% Area	Height
13		7.838	68134	1.93	6204
14	25ODESRIF	8.304	1746654	49.52	117723
15	RIF	8.933	1435	0.04	475
16		9.897	7481	0.21	358
17	NEO	10.751	402158	11.40	59798
18		11.433	6515	0.18	558
19		11.871	4673	0.13	302

SAMPLE INFORMATION

Sample Name:	RIF NELF Inc 1miM 2	Acquired By:	Saneesh
Sample Type:	Unknown	Date Acquired:	2016/12/22 06:46:35 PM
Vial:	5	Acq. Method Set:	HDI_RIF_25ODESRIF_NEO
Injection #:	1	Date Processed:	2016/12/23 09:45:08 AM
Injection Volume:	15.00 ul	Processing Method:	HDI_RIF_ W2996 254.0nm-1.2
Run Time:	12.5 Minutes	Channel Name:	W2996 254.0nm-1.2
Sample Set Name:	RIF NELF Inhibtr_Asy2 22_12_16	Proc. Chnl. Descr.:	W2996 PDA 254.0 nm at 1.2

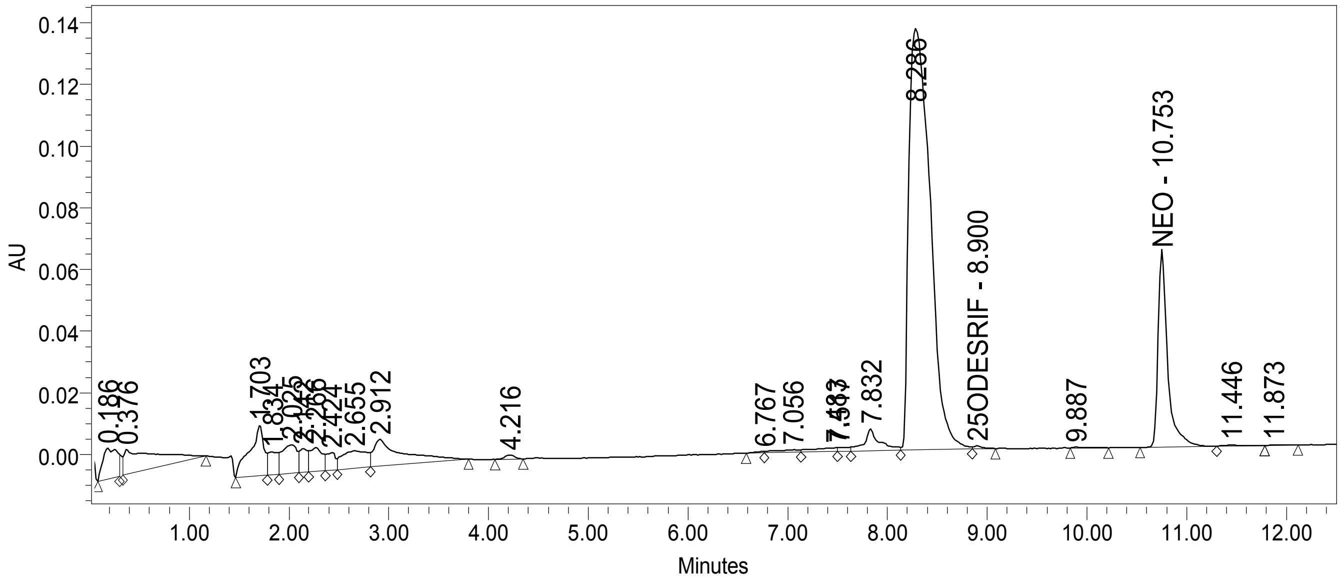

	Peak Name	RT	Area	% Area	Height
1		0.186	96478	2.67	9904
2		0.376	184939	5.12	7899
3		1.703	165965	4.59	16229
4		1.834	50488	1.40	7339
5		2.025	98514	2.73	9247
6		2.142	43698	1.21	7653
7		2.266	66589	1.84	7756
8		2.424	35415	0.98	5844
9		2.655	99551	2.75	5632
10		2.912	180308	4.99	8714
11		4.216	9210	0.25	1344
12		6.767	2447	0.07	433

Reported by User: Saneesh (Saneesh) Project Name: HDI_Stellenbosch_2016

	Peak Name	RT	Area	% Area	Height
13		7.056	14206	0.39	778
14		7.483	20637	0.57	1199
15		7.517	9899	0.27	1294
16		7.832	76543	2.12	7087
17		8.286	2019965	55.88	136635
18	25ODESRIF	8.900	6663	0.18	1019
19	RIF	9.400			
20		9.887	2901	0.08	247
21	NEO	10.753	423397	11.71	63495
22		11.446	4653	0.13	392
23		11.873	2346	0.06	211

SAMPLE INFORMATION

Sample Name:	RIF NELF Inc 10miM 1	Acquired By:	Saneesh
Sample Type:	Unknown	Date Acquired:	2016/12/22 07:02:19 PM
Vial:	6	Acq. Method Set:	HDI_RIF_25ODESRIF_NEO
Injection #:	1	Date Processed:	2016/12/23 09:45:08 AM
Injection Volume:	15.00 ul	Processing Method:	HDI_RIF_ W2996 254.0nm-1.2
Run Time:	12.5 Minutes	Channel Name:	W2996 254.0nm-1.2
Sample Set Name:	RIF NELF Inhibtr_Asy2 22_12_16	Proc. Chnl. Descr.:	W2996 PDA 254.0 nm at 1.2

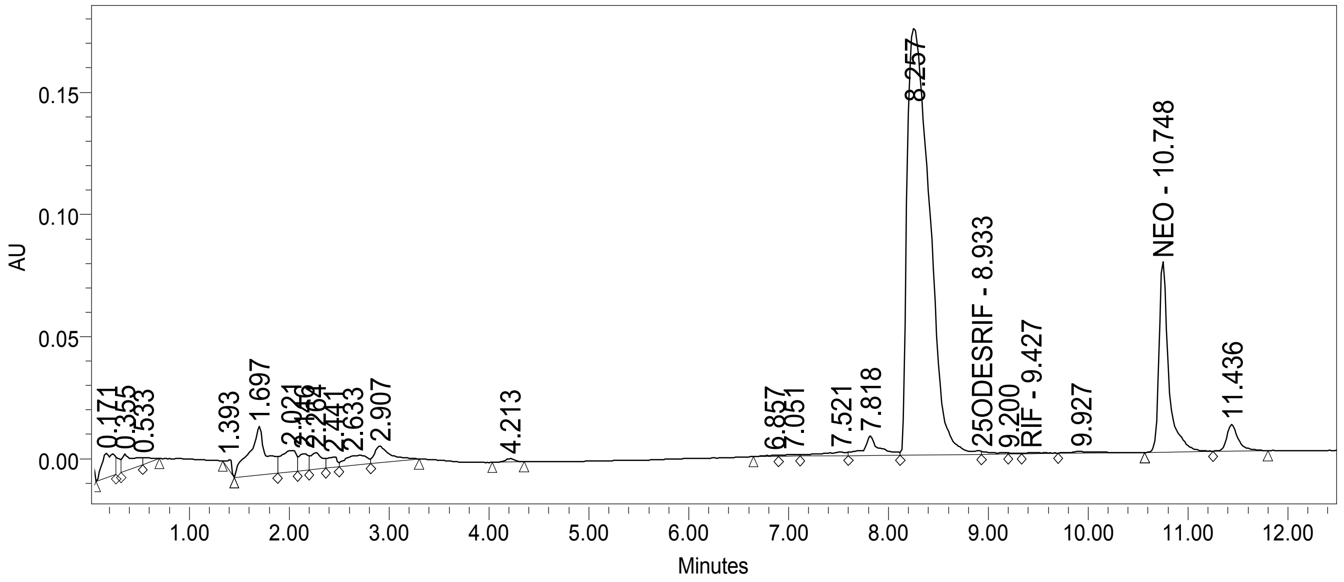

	Peak Name	RT	Area	% Area	Height
1		0.171	81630	1.92	9797
2		0.355	57912	1.37	6610
3		0.533	13806	0.33	2730
4		1.393	16019	0.38	3964
5		1.697	240945	5.68	19775
6		2.021	93283	2.20	8765
7		2.146	44625	1.05	6876
8		2.264	54943	1.30	6826
9		2.441	29954	0.71	4509
10		2.633	60620	1.43	3927
11		2.907	74062	1.75	6722
12		4.213	10316	0.24	1483

Reported by User: Saneesh (Saneesh) Project Name: HDI_Stellenbosch_2016

	Peak Name	RT	Area	% Area	Height
13		6.857	4436	0.10	470
14		7.051	7969	0.19	716
15		7.521	29277	0.69	1425
16		7.818	84694	2.00	7813
17		8.257	2685939	63.34	175137
18	25ODESRIF	8.933	10110	0.24	1111
19		9.200	2357	0.06	425
20	RIF	9.427	6265	0.15	405
21		9.927	14518	0.34	664
22	NEO	10.748	527813	12.45	77317
23		11.436	89132	2.10	10951

SAMPLE INFORMATION

Sample Name:	RIF NELF Inc 10miM 2	Acquired By:	Saneesh
Sample Type:	Unknown	Date Acquired:	2016/12/22 07:18:03 PM
Vial:	7	Acq. Method Set:	HDI_RIF_25ODESRIF_NEO
Injection #:	1	Date Processed:	2016/12/23 09:45:08 AM
Injection Volume:	15.00 ul	Processing Method:	HDI_RIF_ W2996 254.0nm-1.2
Run Time:	12.5 Minutes	Channel Name:	W2996 254.0nm-1.2
Sample Set Name:	RIF NELF Inhibtr_Asy2 22_12_16	Proc. Chnl. Descr.:	W2996 PDA 254.0 nm at 1.2

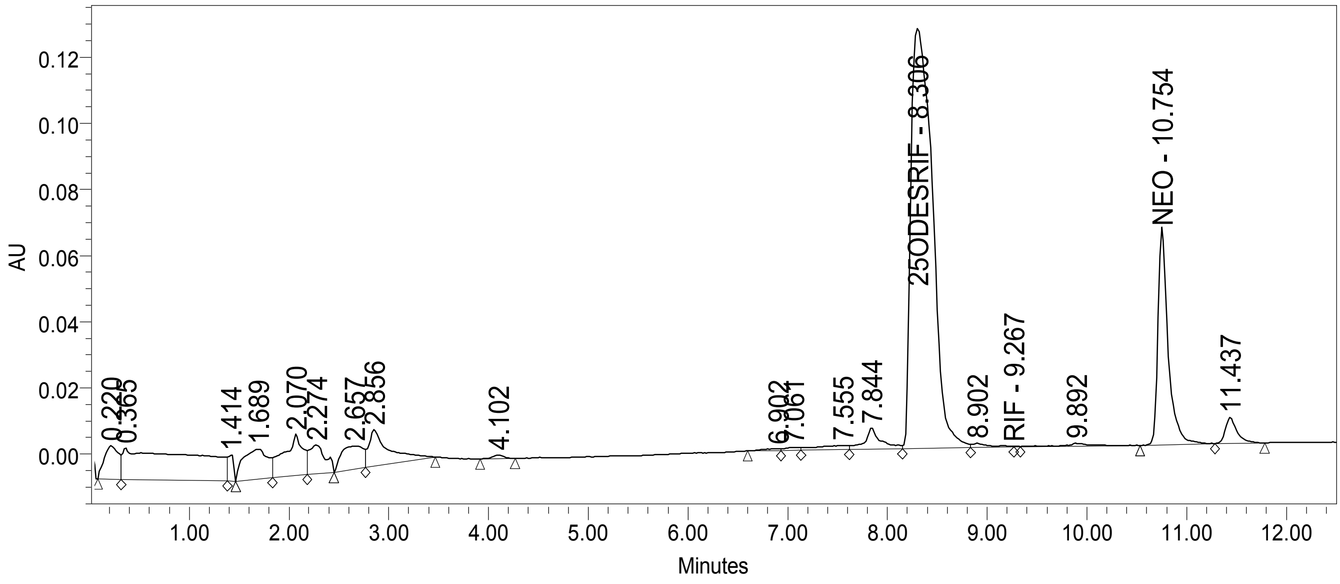

	Peak Name	RT	Area	% Area	Height
1		0.220	101787	2.61	10183
2		0.365	500159	12.81	9354
3		1.414	29706	0.76	8018
4		1.689	155351	3.98	9030
5		2.070	169277	4.33	12203
6		2.274	98981	2.53	8716
7		2.657	106702	2.73	6981
8		2.856	155281	3.98	11037
9		4.102	9004	0.23	1162
10		6.902	6989	0.18	503
11		7.061	8715	0.22	809
12		7.555	29254	0.75	1237

	Peak Name	RT	Area	% Area	Height
13		7.844	73677	1.89	6319
14	25ODESRIF	8.306	1894633	48.51	127201
15		8.902	17073	0.44	1281
16	RIF	9.267	855	0.02	261
17		9.892	20454	0.52	907
18	NEO	10.754	462388	11.84	65529
19		11.437	65645	1.68	7912

SAMPLE INFORMATION

Sample Name:	RIF NELF Inc 50miM 1	Acquired By:	Saneesh
Sample Type:	Unknown	Date Acquired:	2016/12/22 07:34:45 PM
Vial:	8	Acq. Method Set:	HDI_RIF_25ODESRIF_NEO
Injection #:	1	Date Processed:	2016/12/23 09:45:08 AM
Injection Volume:	15.00 ul	Processing Method:	HDI_RIF_ W2996 254.0nm-1.2
Run Time:	12.5 Minutes	Channel Name:	W2996 254.0nm-1.2
Sample Set Name:	RIF NELF Inhibtr_Asy2 22_12_16	Proc. Chnl. Descr.:	W2996 PDA 254.0 nm at 1.2

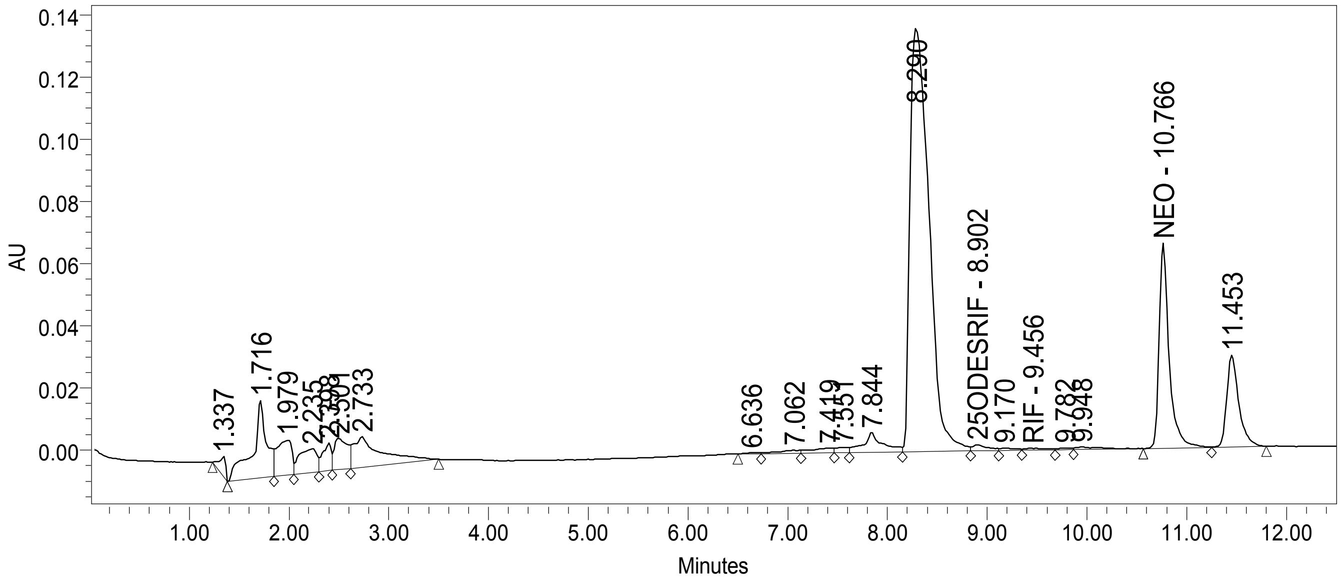

	Peak Name	RT	Area	% Area	Height
1		1.337	27172	0.77	6239
2		1.716	248415	7.04	24616
3		1.979	115684	3.28	11162
4		2.235	94900	2.69	7722
5		2.398	52923	1.50	8738
6		2.501	93342	2.65	10046
7		2.733	191484	5.43	9700
8		6.636	3257	0.09	315
9		7.062	15077	0.43	984
10		7.419	23778	0.67	1432
11		7.551	13987	0.40	1607
12		7.844	85441	2.42	6205

Reported by User: Saneesh (Saneesh) Project Name: HDI_Stellenbosch_2016

	Peak Name	RT	Area	% Area	Height
13		8.290	1800537	51.05	136508
14	25ODESRIF	8.902	20645	0.59	1969
15		9.170	8287	0.23	794
16	RIF	9.456	9098	0.26	531
17		9.782	5129	0.15	554
18		9.948	14189	0.40	755
19	NEO	10.766	455457	12.91	65471
20		11.453	248174	7.04	29537

SAMPLE INFORMATION

Sample Name:	RIF NELF Inc 50miM 2	Acquired By:	Saneesh
Sample Type:	Unknown	Date Acquired:	2016/12/22 07:50:30 PM
Vial:	9	Acq. Method Set:	HDI_RIF_25ODESRIF_NEO
Injection #:	1	Date Processed:	2016/12/23 09:45:08 AM
Injection Volume:	15.00 ul	Processing Method:	HDI_RIF_ W2996 254.0nm-1.2
Run Time:	12.5 Minutes	Channel Name:	W2996 254.0nm-1.2
Sample Set Name:	RIF NELF Inhibtr_Asy2 22_12_16	Proc. Chnl. Descr.:	W2996 PDA 254.0 nm at 1.2

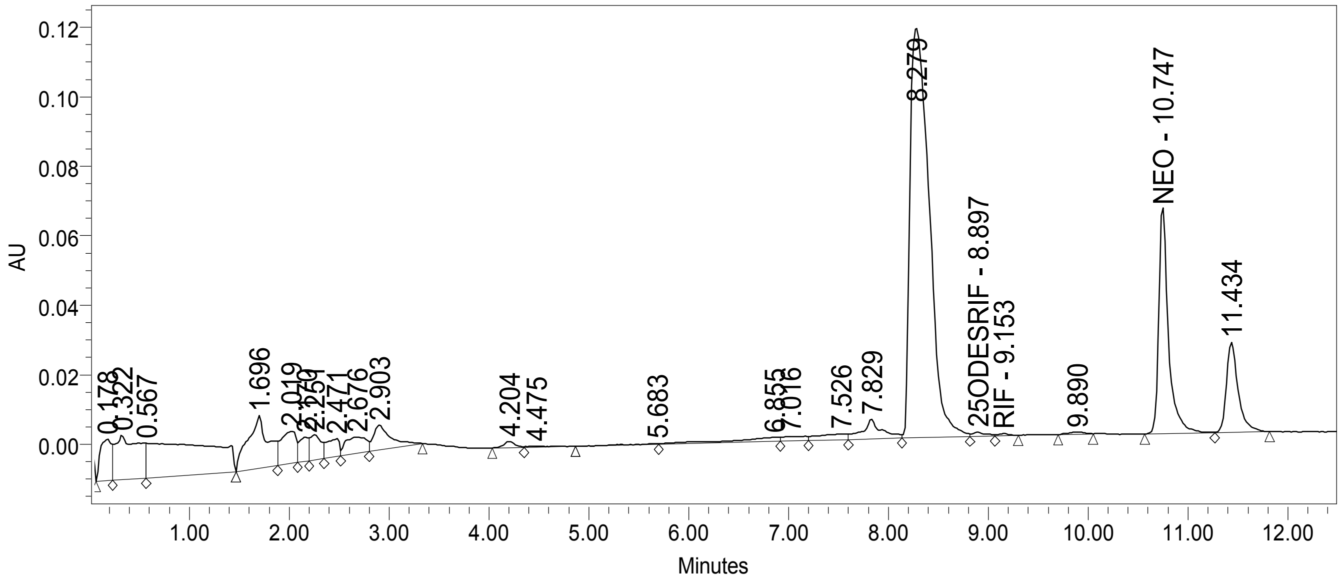

	Peak Name	RT	Area	% Area	Height
1		0.178	92551	2.42	11824
2		0.322	209629	5.49	12418
3		0.567	452659	11.85	9961
4		1.696	213388	5.59	14982
5		2.019	98425	2.58	9283
6		2.170	45766	1.20	6882
7		2.251	55360	1.45	7251
8		2.471	46332	1.21	5487
9		2.676	68414	1.79	4717
10		2.903	88299	2.31	7158
11		4.204	14482	0.38	1834
12		4.475	5898	0.15	432

Reported by User: Saneesh (Saneesh) Project Name: HDI_Stellenbosch_2016

	Peak Name	RT	Area	% Area	Height
13		5.683	8002	0.21	300
14		6.855	43721	1.14	1185
15		7.016	21471	0.56	1353
16		7.526	35249	0.92	1685
17		7.829	75002	1.96	5571
18		8.279	1572628	41.17	117967
19	25ODESRIF	8.897	10358	0.27	1199
20	RIF	9.153	3678	0.10	546
21		9.890	6551	0.17	577
22	NEO	10.747	440547	11.53	64696
23		11.434	211303	5.53	25830

SAMPLE INFORMATION

Sample Name:	RIF NELF Inc 100miM 1	Acquired By:	Saneesh
Sample Type:	Unknown	Date Acquired:	2016/12/22 08:06:14 PM
Vial:	10	Acq. Method Set:	HDI_RIF_25ODESRIF_NEO
Injection #:	1	Date Processed:	2016/12/23 09:45:08 AM
Injection Volume:	15.00 ul	Processing Method:	HDI_RIF_ W2996 254.0nm-1.2
Run Time:	12.5 Minutes	Channel Name:	W2996 254.0nm-1.2
Sample Set Name:	RIF NELF Inhibtr_Asy2 22_12_16	Proc. Chnl. Descr.:	W2996 PDA 254.0 nm at 1.2

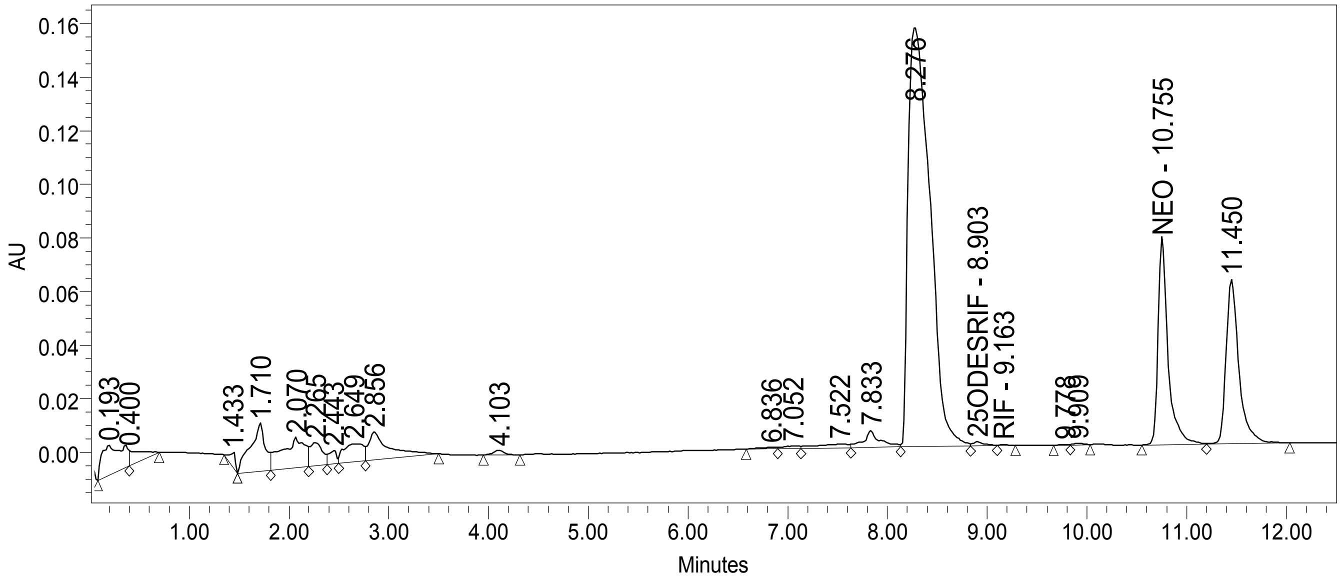

	Peak Name	RT	Area	% Area	Height
1		0.193	156036	3.44	10853
2		0.400	48714	1.07	5634
3		1.433	22514	0.50	5248
4		1.710	186380	4.11	17586
5		2.070	180857	3.99	11011
6		2.265	74100	1.63	8538
7		2.443	27328	0.60	5223
8		2.649	93696	2.07	6805
9		2.856	150491	3.32	10496
10		4.103	12522	0.28	1598
11		6.836	5976	0.13	495
12		7.052	9354	0.21	804

Reported by User: Saneesh (Saneesh) Project Name: HDI_Stellenbosch_2016

	Peak Name	RT	Area	% Area	Height
13		7.522	30939	0.68	1422
14		7.833	70791	1.56	6256
15		8.276	2363780	52.13	157122
16	25ODESRIF	8.903	12962	0.29	1548
17	RIF	9.163	2619	0.06	455
18		9.778	1691	0.04	260
19		9.909	4209	0.09	522
20	NEO	10.755	531741	11.73	77247
21		11.450	547495	12.07	61095

Reported by User: Saneesh (Saneesh) | Project Name: HDI_Stellenbosch_2016

SAMPLE INFORMATION

Sample Name:	RIF NELF Inc 100miM 2	Acquired By:	Saneesh
Sample Type:	Unknown	Date Acquired:	2016/12/22 08:21:58 PM
Vial:	11	Acq. Method Set:	HDI_RIF_25ODESRIF_NEO
Injection #:	1	Date Processed:	2016/12/23 09:45:08 AM
Injection Volume:	15.00 ul	Processing Method:	HDI_RIF_ W2996 254.0nm-1.2
Run Time:	12.5 Minutes	Channel Name:	W2996 254.0nm-1.2
Sample Set Name:	RIF NELF Inhibtr_Asy2 22_12_16	Proc. Chnl. Descr.:	W2996 PDA 254.0 nm at 1.2

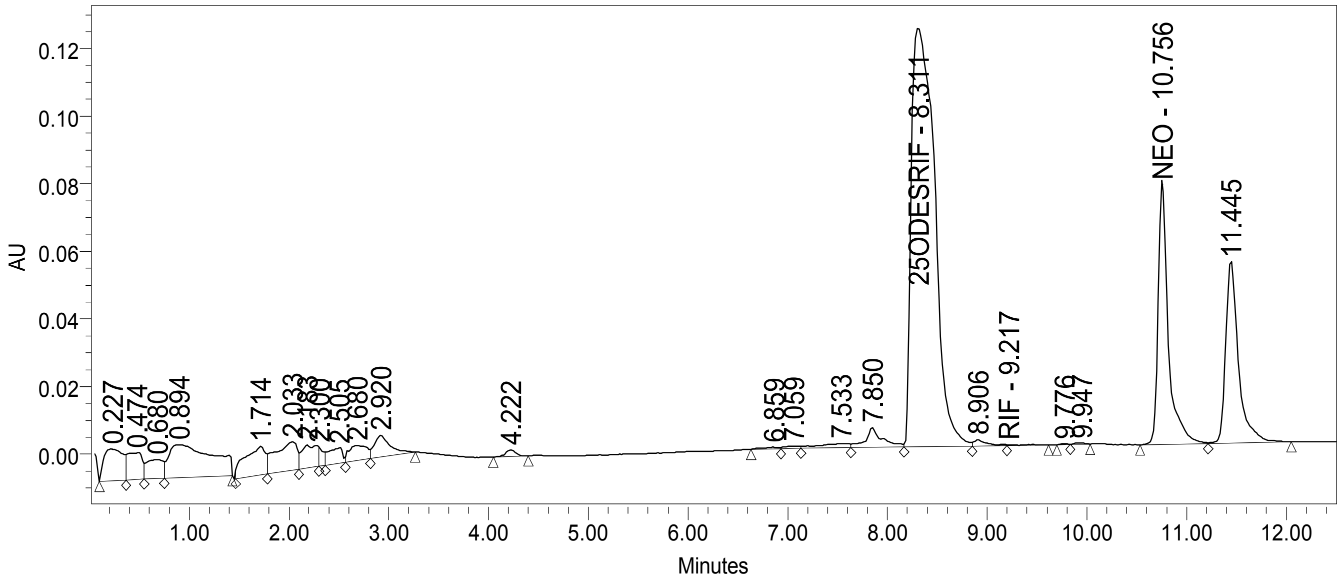

	Peak Name	RT	Area	% Area	Height
1		0.227	124390	2.92	9377
2		0.474	81529	1.91	7991
3		0.680	64195	1.51	5615
4		0.894	297279	6.98	9778
5		1.714	122640	2.88	8442
6		2.033	129868	3.05	8336
7		2.183	71621	1.68	6761
8		2.300	17950	0.42	5654
9		2.505	45860	1.08	4825
10		2.680	56208	1.32	4559
11		2.920	71842	1.69	6382
12		4.222	13476	0.32	1928

Reported by User: Saneesh (Saneesh)   Project Name: HDI_Stellenbosch_2016

	Peak Name	RT	Area	% Area	Height
13		6.859	5766	0.14	487
14		7.059	7946	0.19	781
15		7.533	28945	0.68	1300
16		7.850	68718	1.61	5903
17	25ODESRIF	8.311	2000379	46.98	124402
18		8.906	16863	0.40	1829
19	RIF	9.217	2720	0.06	270
20		9.776	1320	0.03	253
21		9.947	3224	0.08	379
22	NEO	10.756	544102	12.78	77875
23		11.445	481404	11.31	53763

SAMPLE INFORMATION

Sample Name:	RIF NELF Inc 150miM 1	Acquired By:	Saneesh
Sample Type:	Unknown	Date Acquired:	2016/12/22 08:37:41 PM
Vial:	12	Acq. Method Set:	HDI_RIF_25ODESRIF_NEO
Injection #:	1	Date Processed:	2016/12/23 09:45:08 AM
Injection Volume:	15.00 ul	Processing Method:	HDI_RIF_ W2996 254.0nm-1.2
Run Time:	12.5 Minutes	Channel Name:	W2996 254.0nm-1.2
Sample Set Name:	RIF NELF Inhibtr_Asy2 22_12_16	Proc. Chnl. Descr.:	W2996 PDA 254.0 nm at 1.2

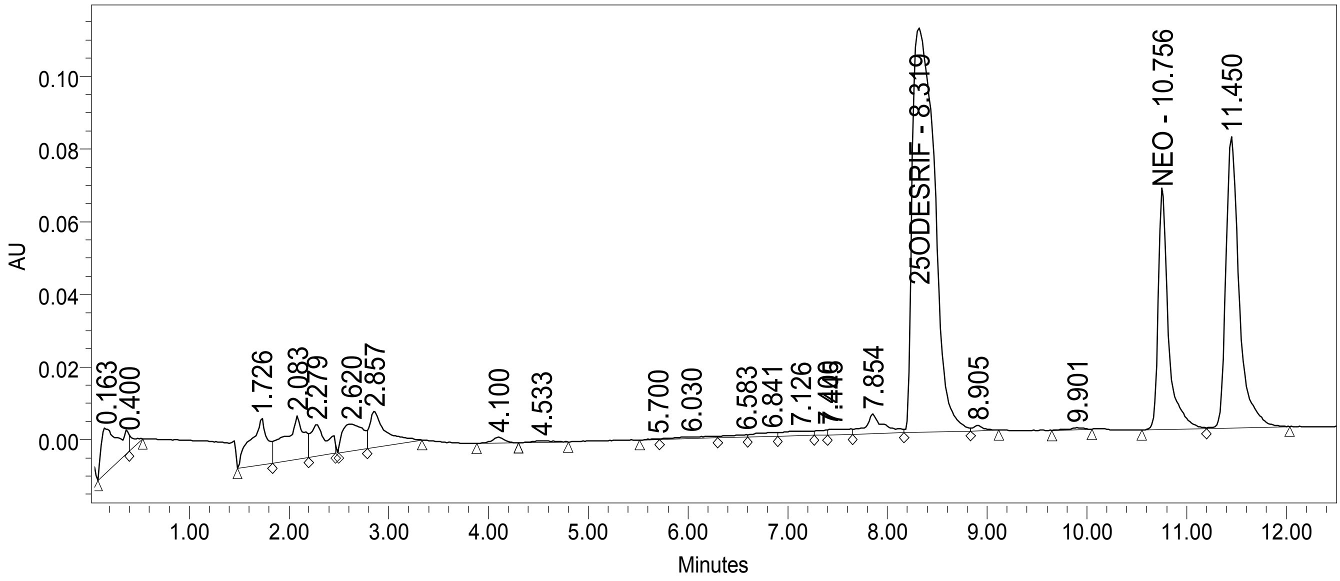

	Peak Name	RT	Area	% Area	Height
1		0.163	145755	3.73	12609
2		0.400	14405	0.37	4336
3		1.726	150354	3.85	12375
4		2.083	162627	4.17	11508
5		2.279	93307	2.39	8649
6		2.620	104126	2.67	7372
7		2.857	111049	2.84	9942
8		4.100	14463	0.37	1613
9		4.533	6731	0.17	464
10		5.700	1630	0.04	221
11		6.030	13315	0.34	465
12		6.583	9353	0.24	689

Reported by User: Saneesh (Saneesh) Project Name: HDI_Stellenbosch_2016

	Peak Name	RT	Area	% Area	Height
13		6.841	16474	0.42	1027
14		7.126	25490	0.65	1236
15		7.400	10038	0.26	1364
16		7.445	21606	0.55	1505
17		7.854	66554	1.70	5360
18	25ODESRIF	8.319	1710703	43.82	111308
19		8.905	10205	0.26	1446
20	RIF	9.400			
21		9.901	4824	0.12	482
22	NEO	10.756	465646	11.93	66403
23		11.450	745173	19.09	79976

Multi Sample Summary

Reported by User: Saneesh (Saneesh)   Project Name: HDI_Stellenbosch_2016

SAMPLE INFORMATION

Sample Name:	RIF NELF Inc 150miM 2	Acquired By:	Saneesh
Sample Type:	Unknown	Date Acquired:	2016/12/22 08:53:27 PM
Vial:	13	Acq. Method Set:	HDI_RIF_25ODESRIF_NEO
Injection #:	1	Date Processed:	2016/12/23 09:45:08 AM
Injection Volume:	15.00 ul	Processing Method:	HDI_RIF_ W2996 254.0nm-1.2
Run Time:	12.5 Minutes	Channel Name:	W2996 254.0nm-1.2
Sample Set Name:	RIF NELF Inhibtr_Asy2 22_12_16	Proc. Chnl. Descr.:	W2996 PDA 254.0 nm at 1.2

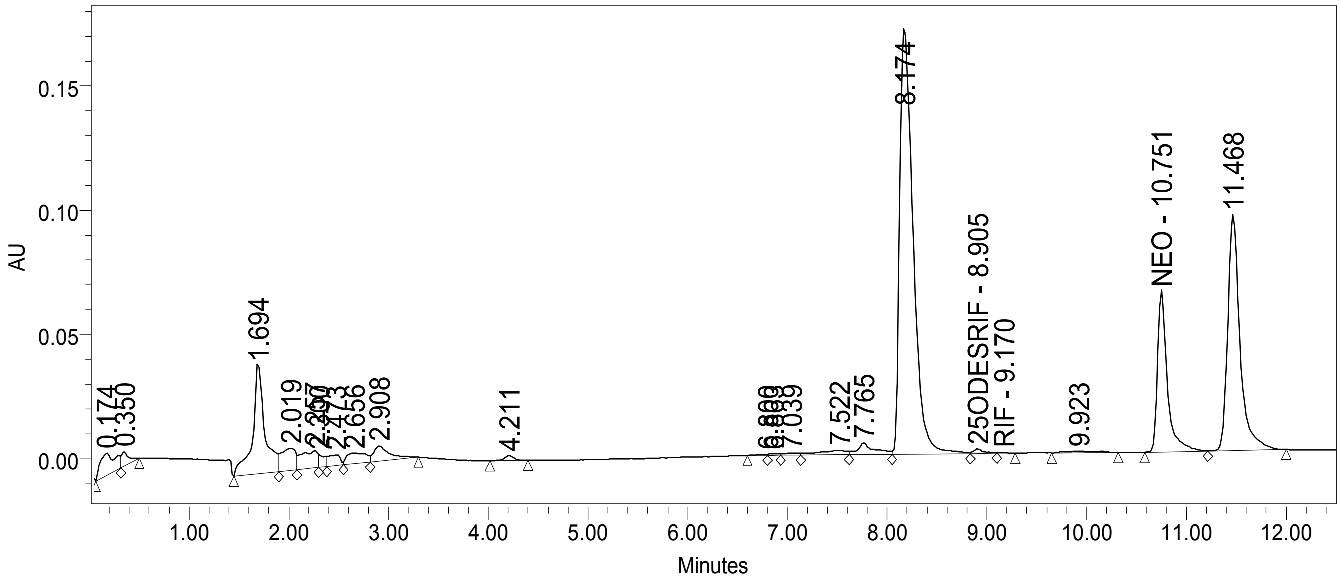

	Peak Name	RT	Area	% Area	Height
1		0.174	90427	2.35	8716
2		0.350	28985	0.75	5551
3		1.694	376620	9.78	44262
4		2.019	87582	2.27	8752
5		2.257	80636	2.09	6763
6		2.300	21509	0.56	5244
7		2.473	36196	0.94	4477
8		2.656	58102	1.51	4323
9		2.908	69710	1.81	6262
10		4.211	14364	0.37	1932
11		6.800	3138	0.08	438
12		6.863	4116	0.11	537

	Peak Name	RT	Area	% Area	Height
13		7.039	8244	0.21	778
14		7.522	33304	0.86	1721
15		7.765	53988	1.40	4565
16		8.174	1602609	41.60	172638
17	25ODESRIF	8.905	12560	0.33	1804
18	RIF	9.170	2238	0.06	372
19		9.923	14755	0.38	647
20	NEO	10.751	441361	11.46	64574
21		11.468	812378	21.09	94778

SAMPLE INFORMATION

Sample Name:	RIF NELF Inc 200miM 1	Acquired By:	Saneesh
Sample Type:	Unknown	Date Acquired:	2016/12/22 09:09:10 PM
Vial:	14	Acq. Method Set:	HDI_RIF_25ODESRIF_NEO
Injection #:	1	Date Processed:	2016/12/23 09:45:08 AM
Injection Volume:	15.00 ul	Processing Method:	HDI_RIF_ W2996 254.0nm-1.2
Run Time:	12.5 Minutes	Channel Name:	W2996 254.0nm-1.2
Sample Set Name:	RIF NELF Inhibtr_Asy2 22_12_16	Proc. Chnl. Descr.:	W2996 PDA 254.0 nm at 1.2

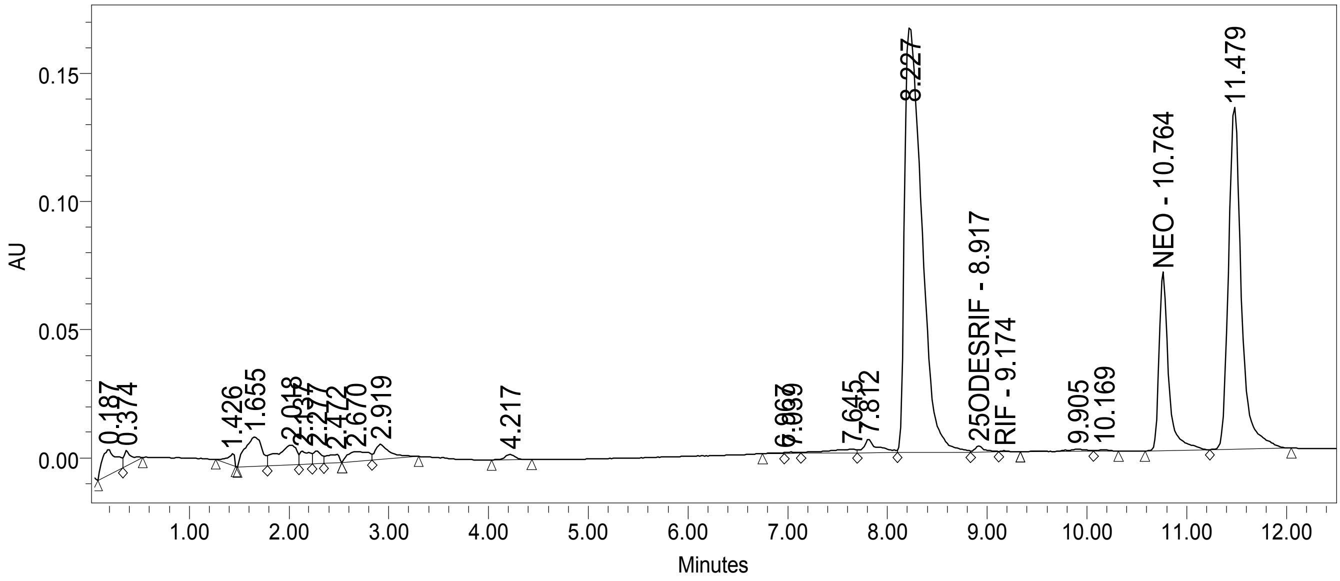

	Peak Name	RT	Area	% Area	Height
1		0.187	96922	2.21	9790
2		0.374	31170	0.71	5489
3		1.426	23074	0.53	4695
4		1.655	134788	3.07	11408
5		2.018	106347	2.42	7725
6		2.137	36131	0.82	5128
7		2.277	28551	0.65	4934
8		2.472	30012	0.68	3604
9		2.670	55134	1.26	3822
10		2.919	61173	1.39	5860
11		4.217	15789	0.36	2117
12		6.967	1942	0.04	258

Reported by User: Saneesh (Saneesh) Project Name: HDI_Stellenbosch_2016

	Peak Name	RT	Area	% Area	Height
13		7.039	3935	0.09	446
14		7.645	29229	0.67	1458
15		7.812	51641	1.18	5209
16		8.227	1980397	45.12	167745
17	25ODESRIF	8.917	15823	0.36	2341
18	RIF	9.174	2583	0.06	415
19		9.905	14755	0.34	988
20		10.169	4872	0.11	579
21	NEO	10.764	461760	10.52	69238
22		11.479	1203277	27.41	133321

Reported by User: Saneesh (Saneesh) Project Name: HDI_Stellenbosch_2016

SAMPLE INFORMATION

Sample Name:	RIF NELF Inc 200miM 2	Acquired By:	Saneesh
Sample Type:	Unknown	Date Acquired:	2016/12/22 09:24:55 PM
Vial:	15	Acq. Method Set:	HDI_RIF_25ODESRIF_NEO
Injection #:	1	Date Processed:	2016/12/23 09:45:08 AM
Injection Volume:	15.00 ul	Processing Method:	HDI_RIF_ W2996 254.0nm-1.2
Run Time:	12.5 Minutes	Channel Name:	W2996 254.0nm-1.2
Sample Set Name:	RIF NELF Inhibtr_Asy2 22_12_16	Proc. Chnl. Descr.:	W2996 PDA 254.0 nm at 1.2

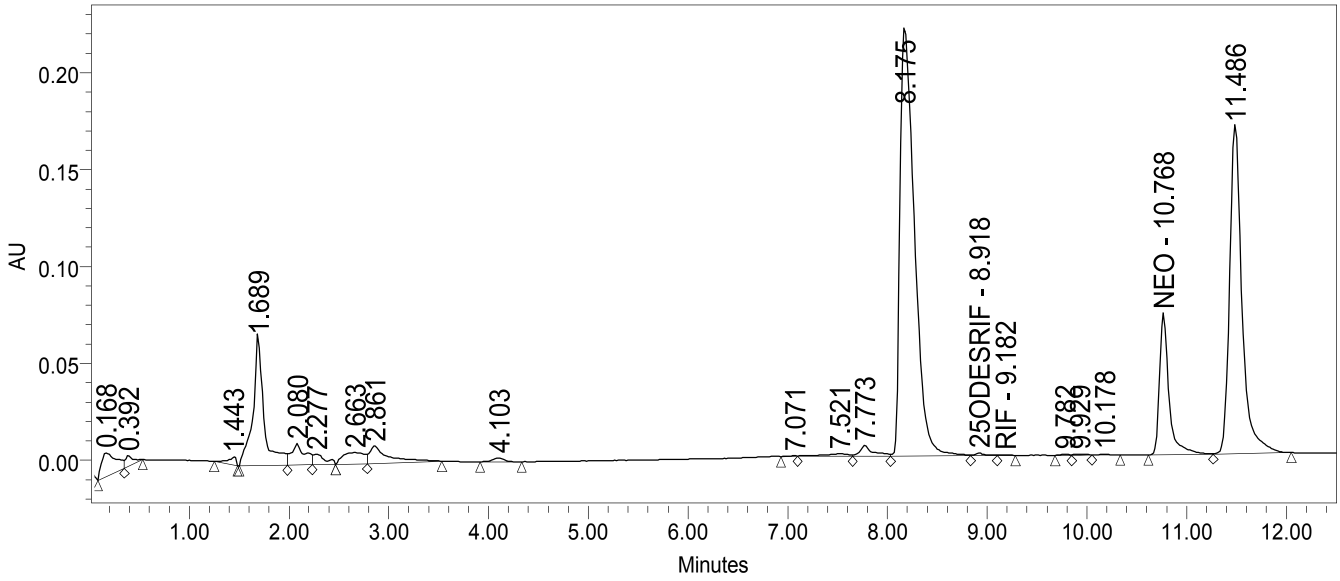

	Peak Name	RT	Area	% Area	Height
1		0.168	122066	2.28	12249
2		0.392	28962	0.54	5225
3		1.443	24855	0.46	4600
4		1.689	521669	9.73	65523
5		2.080	104962	1.96	10700
6		2.277	46975	0.88	5365
7		2.663	89519	1.67	5812
8		2.861	117679	2.20	8882
9		4.103	16234	0.30	2034
10		7.071	2465	0.05	341
11		7.521	26008	0.49	1350
12		7.773	52509	0.98	5415

	Peak Name	RT	Area	% Area	Height
13		8.175	2236045	41.71	223616
14	25ODESRIF	8.918	11435	0.21	1499
15	RIF	9.182	3860	0.07	551
16		9.782	3964	0.07	633
17		9.929	6855	0.13	729
18		10.178	5596	0.10	602
19	NEO	10.768	477024	8.90	72519
20		11.486	1462413	27.28	169333

Multi Sample Summary

Reported by User: Saneesh (Saneesh)   Project Name: HDI_Stellenbosch_2016

SAMPLE INFORMATION

Sample Name:	EFV TICL Control K1 17.12.16	Acquired By:	Saneesh
Sample Type:	Unknown	Date Acquired:	2016/12/22 11:31:44 PM
Vial:	23	Acq. Method Set:	HDI_EFV_E8H_NEO
Injection #:	1	Date Processed:	2016/12/23 09:46:08 AM
Injection Volume:	15.00 ul	Processing Method:	HDI_EFV_ W2996 245.0nm-1.2
Run Time:	11.0 Minutes	Channel Name:	W2996 245.0nm-1.2
Sample Set Name:	EFV TICL Inhibtr_Asy1 22_12_16	Proc. Chnl. Descr.:	W2996 PDA 245.0 nm at 1.2

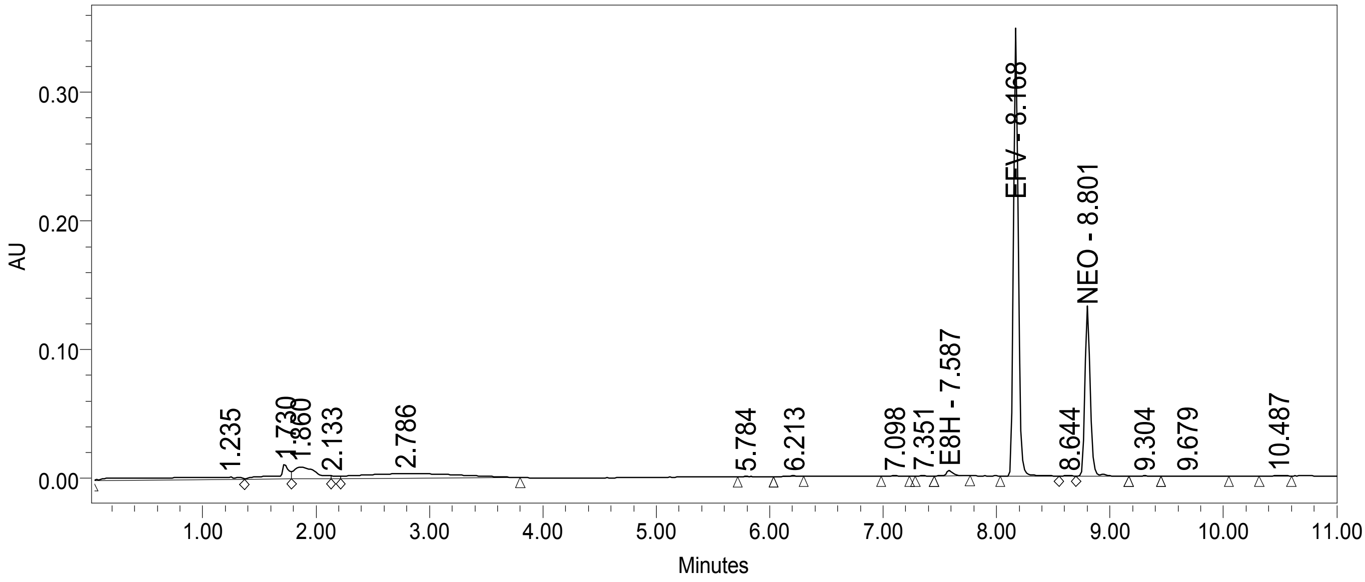

	Peak Name	RT	Area	% Area	Height
1		1.235	118665	5.84	1863
2		1.730	78714	3.87	10153
3		1.860	124141	6.11	9399
4		2.133	10606	0.52	2367
5		2.786	220969	10.87	3836
6		5.784	2068	0.10	240
7		6.213	2663	0.13	544
8		7.098	1848	0.09	447
9		7.351	2294	0.11	564
10	E8H	7.587	18642	0.92	4160
11	EFV	8.168	1018350	50.09	312636
12		8.644	2343	0.12	440
13	NEO	8.801	422795	20.80	122227
14		9.304	2200	0.11	410

	Peak Name	RT	Area	% Area	Height
15		9.679	3339	0.16	301
16		10.487	3420	0.17	426

Reported by User: Saneesh (Saneesh) Project Name: HDI_Stellenbosch_2016

SAMPLE INFORMATION

Sample Name:	EFV TICL Control K2 17.12.16	Acquired By:	Saneesh
Sample Type:	Unknown	Date Acquired:	2016/12/22 11:46:01 PM
Vial:	24	Acq. Method Set:	HDI_EFV_E8H_NEO
Injection #:	1	Date Processed:	2016/12/23 09:46:08 AM
Injection Volume:	15.00 ul	Processing Method:	HDI_EFV_ W2996 245.0nm-1.2
Run Time:	11.0 Minutes	Channel Name:	W2996 245.0nm-1.2
Sample Set Name:	EFV TICL Inhibtr_Asy1 22_12_16	Proc. Chnl. Descr.:	W2996 PDA 245.0 nm at 1.2

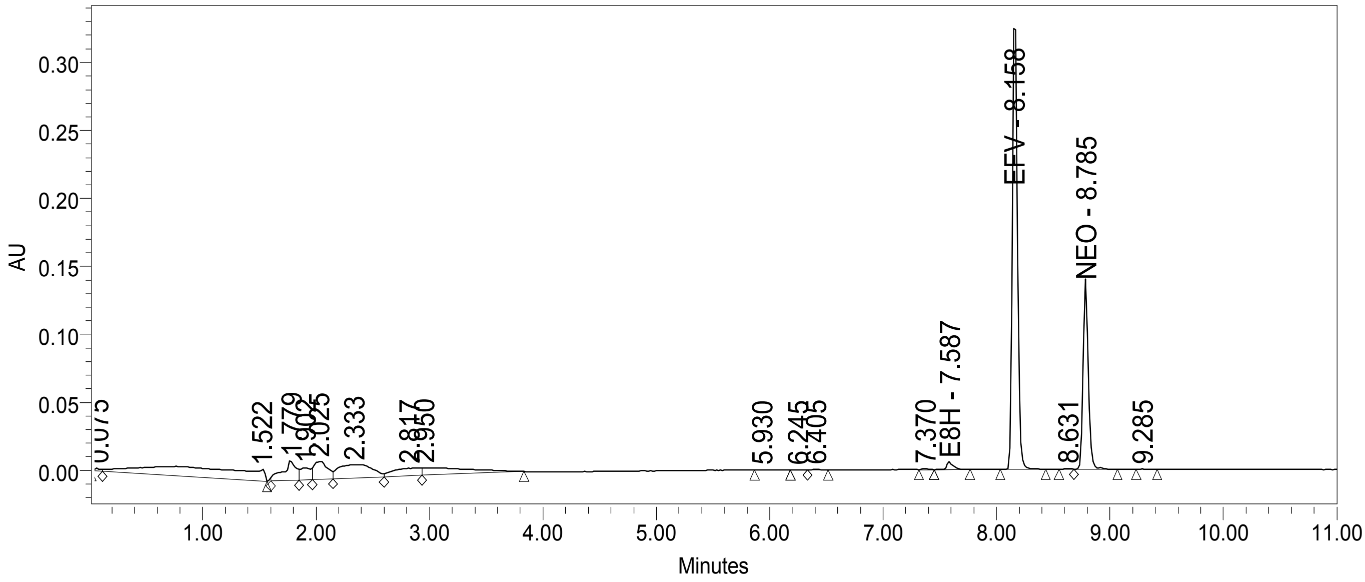

	Peak Name	RT	Area	% Area	Height
1		0.075	7334	0.27	1957
2		1.522	485779	17.79	8588
3		1.779	115776	4.24	13280
4		1.902	57515	2.11	8746
5		2.025	108779	3.98	13001
6		2.333	202027	7.40	9609
7		2.817	95539	3.50	5576
8		2.950	154543	5.66	5448
9		5.930	2242	0.08	403
10		6.245	969	0.04	140
11		6.405	2315	0.08	497
12		7.370	2790	0.10	803

Reported by User: Saneesh (Saneesh) Project Name: HDI_Stellenbosch_2016

	Peak Name	RT	Area	% Area	Height
13	E8H	7.587	21040	0.77	5082
14	EFV	8.158	1035574	37.92	306132
15		8.631	2193	0.08	567
16	NEO	8.785	435179	15.93	128211
17		9.285	1529	0.06	358

SAMPLE INFORMATION

Sample Name:	EFV TICL Inc 1miM 1	Acquired By:	Saneesh
Sample Type:	Unknown	Date Acquired:	2016/12/23 12:00:19 AM
Vial:	2	Acq. Method Set:	HDI_EFV_E8H_NEO
Injection #:	1	Date Processed:	2016/12/23 09:46:08 AM
Injection Volume:	15.00 ul	Processing Method:	HDI_EFV_ W2996 245.0nm-1.2
Run Time:	11.0 Minutes	Channel Name:	W2996 245.0nm-1.2
Sample Set Name:	EFV TICL Inhibtr_Asy1 22_12_16	Proc. Chnl. Descr.:	W2996 PDA 245.0 nm at 1.2

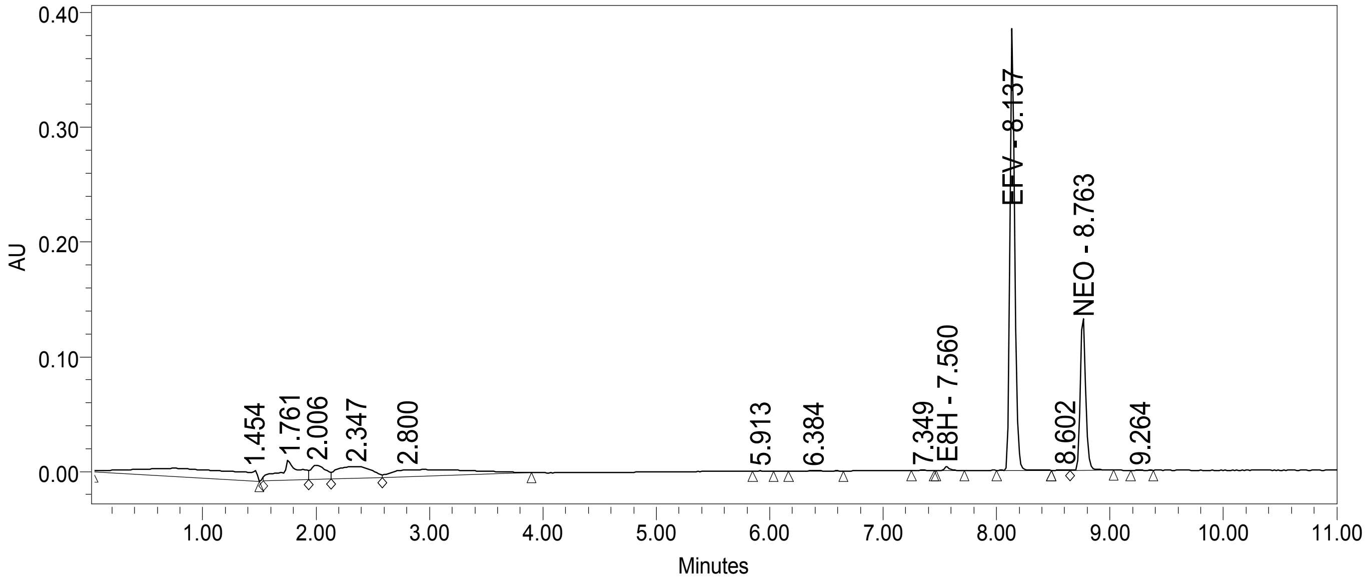

	Peak Name	RT	Area	% Area	Height
1		1.454	483417	16.90	8923
2		1.761	202625	7.08	15986
3		2.006	114009	3.98	12451
4		2.347	214316	7.49	10028
5		2.800	274498	9.59	5976
6		5.913	1580	0.06	373
7		6.384	4138	0.14	431
8		7.349	2426	0.08	624
9	E8H	7.560	12688	0.44	3126
10	EFV	8.137	1112411	38.88	344324
11		8.602	1737	0.06	470
12	NEO	8.763	436151	15.24	125489

	Peak Name	RT	Area	% Area	Height
13		9.264	1282	0.04	360

Reported by User: Saneesh (Saneesh)   Project Name: HDI_Stellenbosch_2016

SAMPLE INFORMATION

Sample Name:	EFV TICL Inc 1miM 2	Acquired By:	Saneesh
Sample Type:	Unknown	Date Acquired:	2016/12/23 12:14:37 AM
Vial:	3	Acq. Method Set:	HDI_EFV_E8H_NEO
Injection #:	1	Date Processed:	2016/12/23 09:46:08 AM
Injection Volume:	15.00 ul	Processing Method:	HDI_EFV_ W2996 245.0nm-1.2
Run Time:	11.0 Minutes	Channel Name:	W2996 245.0nm-1.2
Sample Set Name:	EFV TICL Inhibtr_Asy1 22_12_16	Proc. Chnl. Descr.:	W2996 PDA 245.0 nm at 1.2

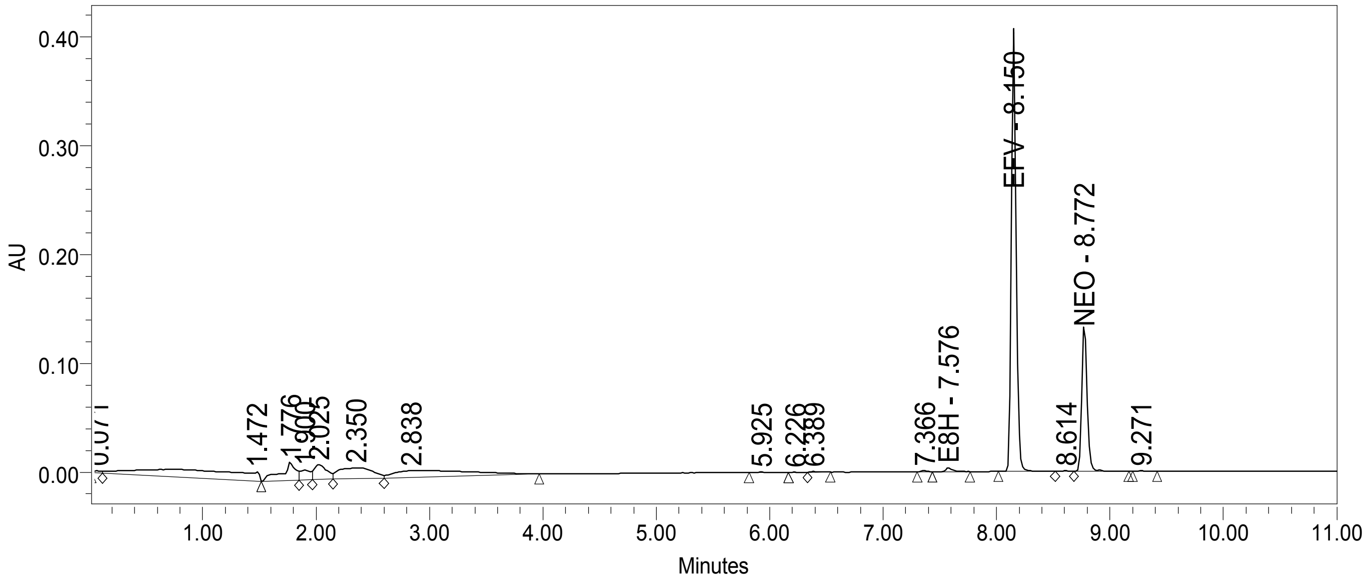

	Peak Name	RT	Area	% Area	Height
1		0.071	7795	0.27	1891
2		1.472	469530	16.15	8663
3		1.776	145108	4.99	15548
4		1.900	58804	2.02	9017
5		2.025	108875	3.75	13577
6		2.350	207088	7.12	9818
7		2.838	273376	9.40	5804
8		5.925	1728	0.06	295
9		6.226	776	0.03	150
10		6.389	2821	0.10	654
11		7.366	3610	0.12	1054
12	E8H	7.576	16379	0.56	3563

Reported by User: Saneesh (Saneesh) Project Name: HDI_Stellenbosch_2016

	Peak Name	RT	Area	% Area	Height
13	EFV	8.150	1168341	40.19	365007
14		8.614	2837	0.10	672
15	NEO	8.772	437763	15.06	124828
16		9.271	2303	0.08	502

SAMPLE INFORMATION

Sample Name:	EFV TICL Inc 10miM 1	Acquired By:	Saneesh
Sample Type:	Unknown	Date Acquired:	2016/12/23 12:28:55 AM
Vial:	4	Acq. Method Set:	HDI_EFV_E8H_NEO
Injection #:	1	Date Processed:	2016/12/23 09:46:08 AM
Injection Volume:	15.00 ul	Processing Method:	HDI_EFV_ W2996 245.0nm-1.2
Run Time:	11.0 Minutes	Channel Name:	W2996 245.0nm-1.2
Sample Set Name:	EFV TICL Inhibtr_Asy1 22_12_16	Proc. Chnl. Descr.:	W2996 PDA 245.0 nm at 1.2

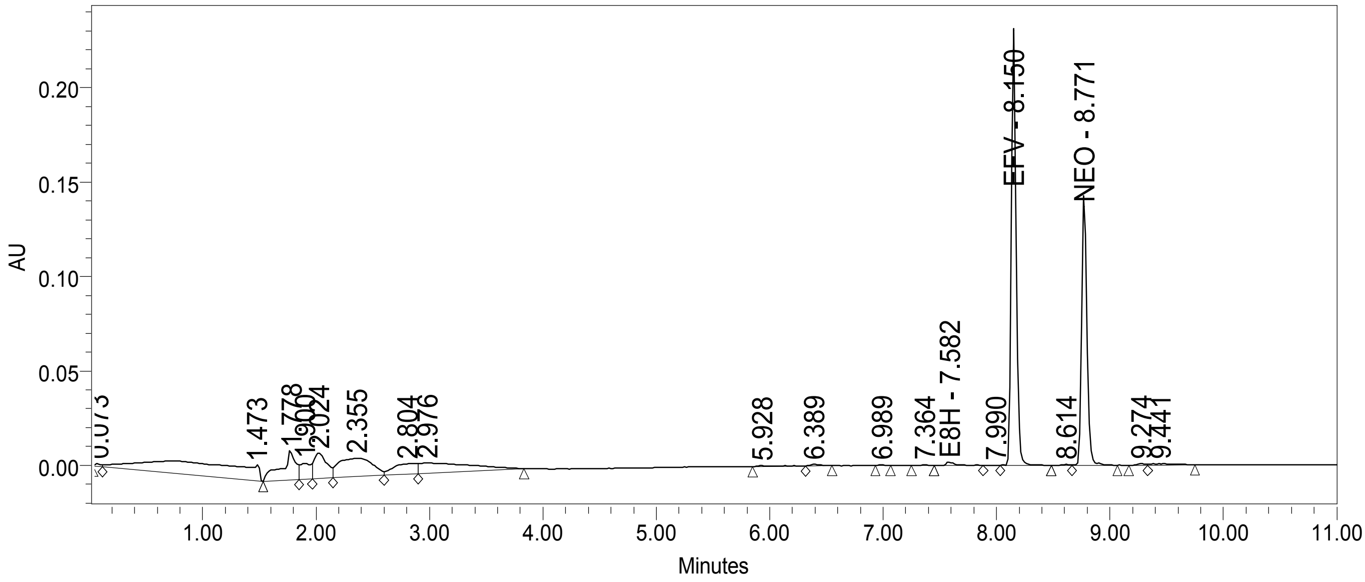

	Peak Name	RT	Area	% Area	Height
1		0.073	5366	0.23	1493
2		1.473	445608	18.78	8518
3		1.778	136017	5.73	14079
4		1.900	55344	2.33	8402
5		2.024	105586	4.45	13367
6		2.355	202642	8.54	9676
7		2.804	81521	3.44	5514
8		2.976	164376	6.93	5379
9		5.928	4953	0.21	428
10		6.389	4906	0.21	924
11		6.989	1846	0.08	551
12		7.364	2193	0.09	572

Reported by User: Saneesh (Saneesh) Project Name: HDI_Stellenbosch_2016

	Peak Name	RT	Area	% Area	Height
13	E8H	7.582	11728	0.49	1785
14		7.990	1290	0.05	173
15	EFV	8.150	673986	28.41	208523
16		8.614	2386	0.10	560
17	NEO	8.771	456612	19.24	132376
18		9.274	4324	0.18	839
19		9.441	12085	0.51	709

SAMPLE INFORMATION

Sample Name:	EFV TICL Inc 10miM 2	Acquired By:	Saneesh
Sample Type:	Unknown	Date Acquired:	2016/12/23 12:43:25 AM
Vial:	5	Acq. Method Set:	HDI_EFV_E8H_NEO
Injection #:	1	Date Processed:	2016/12/23 09:46:09 AM
Injection Volume:	15.00 ul	Processing Method:	HDI_EFV_ W2996 245.0nm-1.2
Run Time:	11.0 Minutes	Channel Name:	W2996 245.0nm-1.2
Sample Set Name:	EFV TICL Inhibtr_Asy1 22_12_16	Proc. Chnl. Descr.:	W2996 PDA 245.0 nm at 1.2

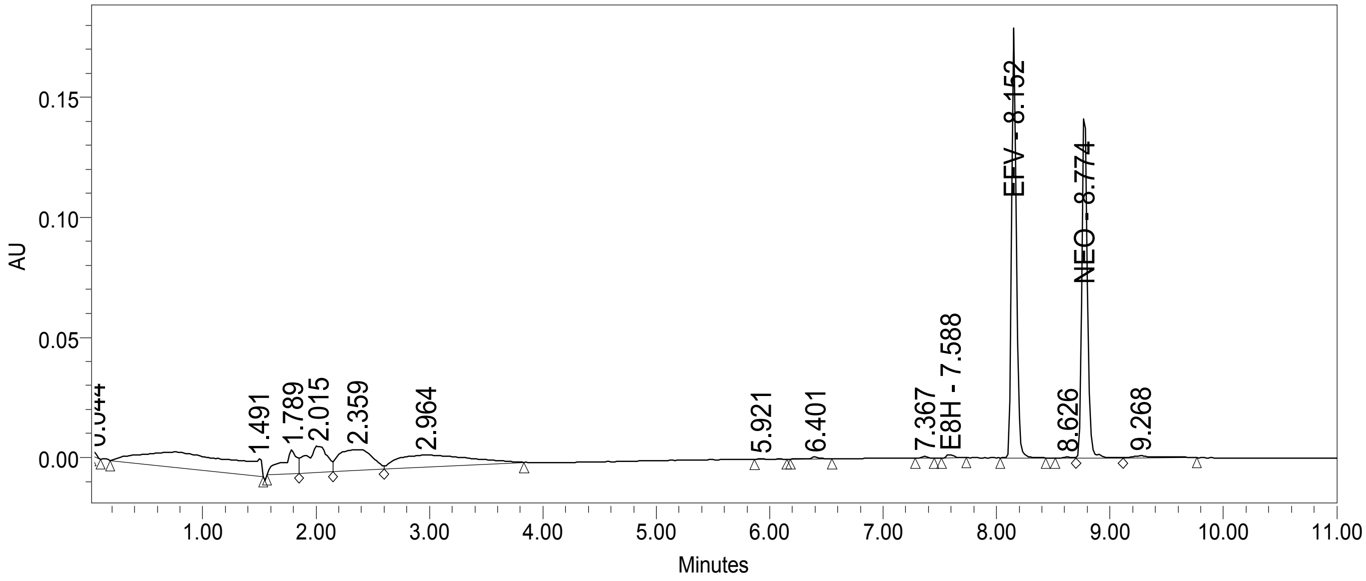

	Peak Name	RT	Area	% Area	Height
1		0.044	4976	0.24	1848
2		1.491	404503	19.70	7806
3		1.789	89163	4.34	9694
4		2.015	137977	6.72	11101
5		2.359	175922	8.57	8646
6		2.964	219174	10.67	4958
7		5.921	1734	0.08	303
8		6.401	4883	0.24	813
9		7.367	2817	0.14	828
10	E8H	7.588	6619	0.32	1336
11	EFV	8.152	513495	25.01	160029
12		8.626	1961	0.10	474

Reported by User: Saneesh (Saneesh) Project Name: HDI_Stellenbosch_2016

	Peak Name	RT	Area	% Area	Height
13	NEO	8.774	475745	23.17	134614
14		9.268	14391	0.70	910

SAMPLE INFORMATION

Sample Name:	EFV TICL Inc 50miM 1	Acquired By:	Saneesh
Sample Type:	Unknown	Date Acquired:	2016/12/23 12:57:42 AM
Vial:	6	Acq. Method Set:	HDI_EFV_E8H_NEO
Injection #:	1	Date Processed:	2016/12/23 09:46:09 AM
Injection Volume:	15.00 ul	Processing Method:	HDI_EFV_ W2996 245.0nm-1.2
Run Time:	11.0 Minutes	Channel Name:	W2996 245.0nm-1.2
Sample Set Name:	EFV TICL Inhibtr_Asy1 22_12_16	Proc. Chnl. Descr.:	W2996 PDA 245.0 nm at 1.2

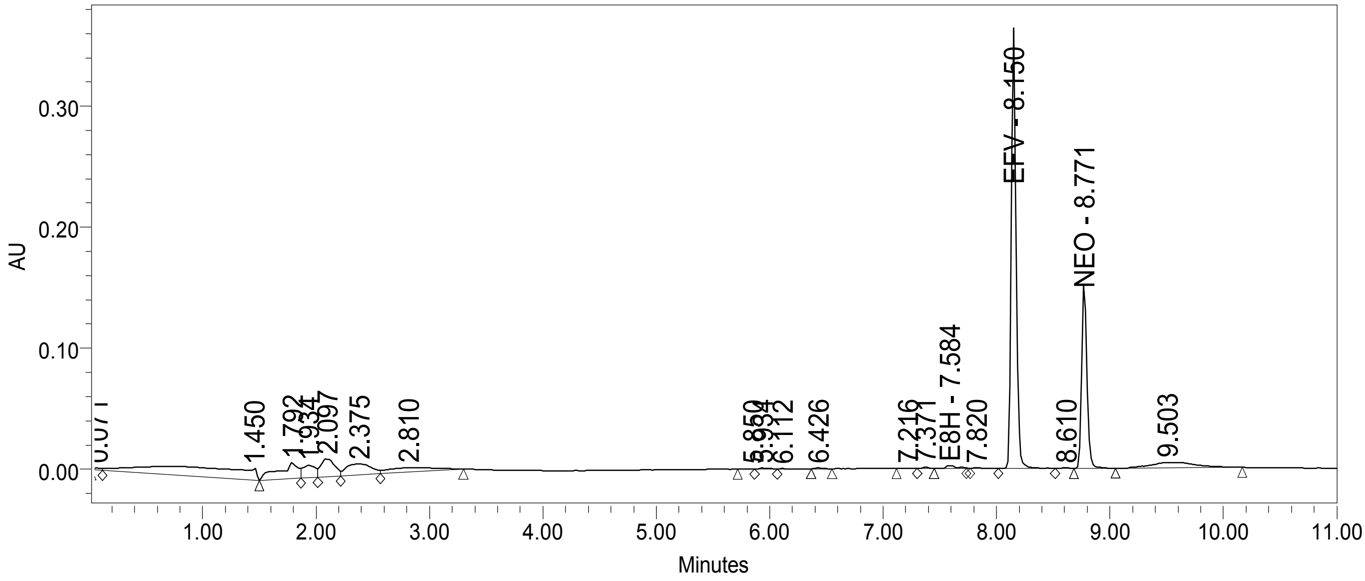

	Peak Name	RT	Area	% Area	Height
1		0.071	7384	0.26	1738
2		1.450	498654	17.70	9199
3		1.792	159003	5.64	12518
4		1.934	78479	2.79	10009
5		2.097	125163	4.44	14774
6		2.375	139430	4.95	8762
7		2.810	105171	3.73	3595
8		5.850	1533	0.05	315
9		5.934	5650	0.20	905
10		6.112	2728	0.10	362
11		6.426	3796	0.13	754
12		7.216	1593	0.06	324

Reported by User: Saneesh (Saneesh)   Project Name: HDI_Stellenbosch_2016

	Peak Name	RT	Area	% Area	Height
13		7.371	3683	0.13	1049
14	E8H	7.584	14727	0.52	2303
15		7.820	4126	0.15	487
16	EFV	8.150	1056106	37.49	327628
17		8.610	1882	0.07	355
18	NEO	8.771	484577	17.20	140616
19		9.503	123193	4.37	4523

Reported by User: Saneesh (Saneesh)
Project Name: HDI_Stellenbosch_2016

SAMPLE INFORMATION

Sample Name:	EFV TICL Inc 50miM 2	Acquired By:	Saneesh
Sample Type:	Unknown	Date Acquired:	2016/12/23 01:12:00 AM
Vial:	7	Acq. Method Set:	HDI_EFV_E8H_NEO
Injection #:	1	Date Processed:	2016/12/23 09:46:09 AM
Injection Volume:	15.00 ul	Processing Method:	HDI_EFV_ W2996 245.0nm-1.2
Run Time:	11.0 Minutes	Channel Name:	W2996 245.0nm-1.2
Sample Set Name:	EFV TICL Inhibtr_Asy1 22_12_16	Proc. Chnl. Descr.:	W2996 PDA 245.0 nm at 1.2

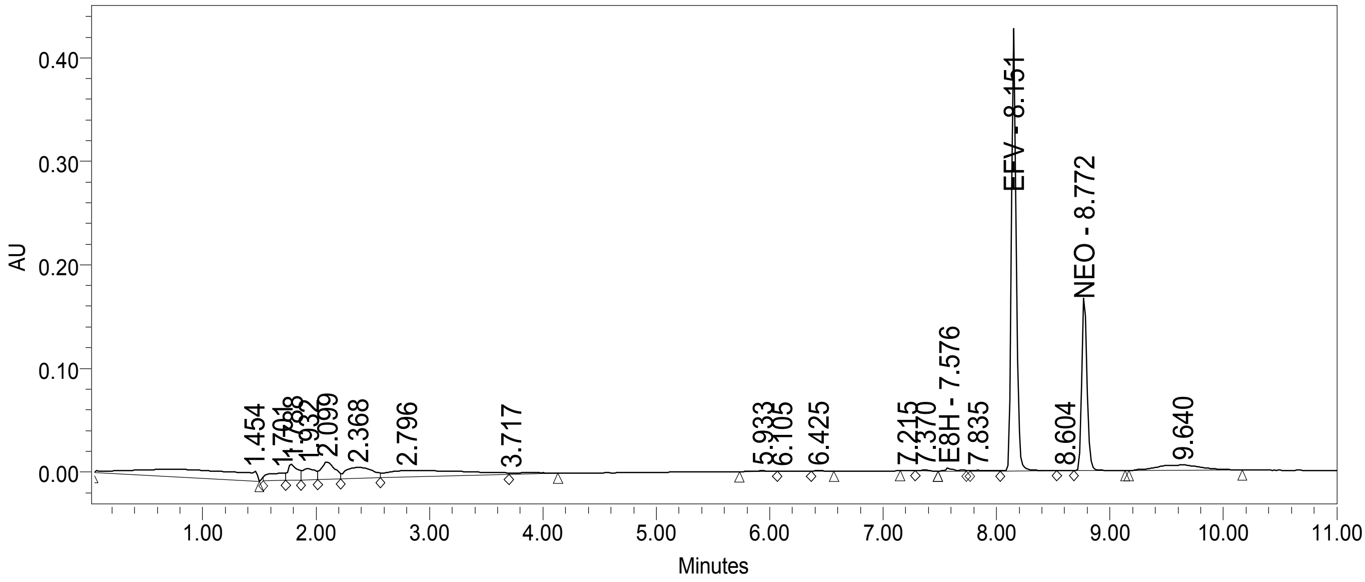

	Peak Name	RT	Area	% Area	Height
1		1.454	519080	15.36	9484
2		1.701	79889	2.36	7187
3		1.788	91180	2.70	15815
4		1.932	87538	2.59	10981
5		2.099	140332	4.15	16722
6		2.368	176764	5.23	10554
7		2.796	297272	8.80	6272
8		3.717	21320	0.63	1324
9		5.933	8889	0.26	1058
10		6.105	5230	0.15	516
11		6.425	4008	0.12	778
12		7.215	1195	0.04	357

	Peak Name	RT	Area	% Area	Height
13		7.370	3385	0.10	939
14	E8H	7.576	14952	0.44	2479
15		7.835	5058	0.15	591
16	EFV	8.151	1231341	36.43	382050
17		8.604	1926	0.06	371
18	NEO	8.772	541529	16.02	156128
19		9.640	148909	4.41	5300

SAMPLE INFORMATION

Sample Name:	EFV TICL Inc 100miM 1	Acquired By:	Saneesh
Sample Type:	Unknown	Date Acquired:	2016/12/23 01:26:28 AM
Vial:	8	Acq. Method Set:	HDI_EFV_E8H_NEO
Injection #:	1	Date Processed:	2016/12/23 09:46:09 AM
Injection Volume:	15.00 ul	Processing Method:	HDI_EFV_ W2996 245.0nm-1.2
Run Time:	11.0 Minutes	Channel Name:	W2996 245.0nm-1.2
Sample Set Name:	EFV TICL Inhibtr_Asy1 22_12_16	Proc. Chnl. Descr.:	W2996 PDA 245.0 nm at 1.2

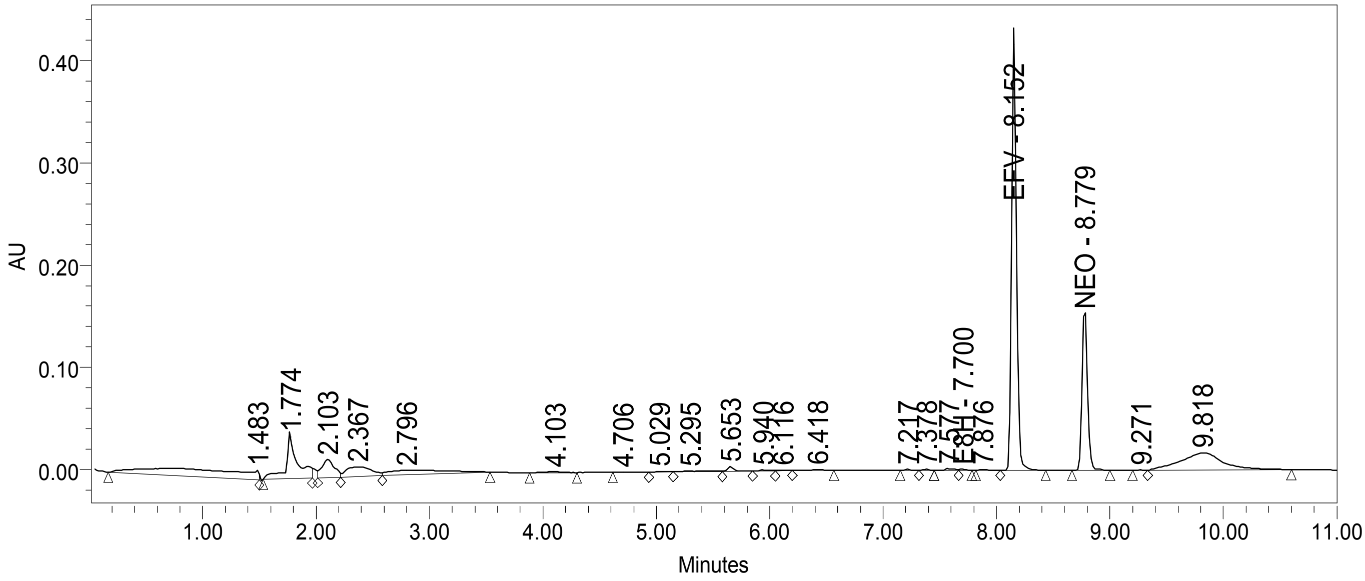

	Peak Name	RT	Area	% Area	Height
1		1.483	484469	13.71	9230
2		1.774	306693	8.68	39642
3		2.103	133590	3.78	17432
4		2.367	147040	4.16	9137
5		2.796	151799	4.30	4378
6		4.103	10916	0.31	834
7		4.706	1929	0.05	221
8		5.029	1985	0.06	274
9		5.295	7783	0.22	605
10		5.653	16376	0.46	4116
11		5.940	5710	0.16	876
12		6.116	4562	0.13	636

Reported by User: Saneesh (Saneesh) Project Name: HDI_Stellenbosch_2016

	Peak Name	RT	Area	% Area	Height
13		6.418	8344	0.24	843
14		7.217	3666	0.10	1040
15		7.378	3857	0.11	1101
16		7.577	11202	0.32	1998
17	E8H	7.700	4496	0.13	1317
18		7.876	4853	0.14	874
19	EFV	8.152	1253834	35.48	386037
20	NEO	8.779	521439	14.76	148667
21		9.271	1726	0.05	501
22		9.818	447293	12.66	17013

SAMPLE INFORMATION

Sample Name:	EFV TICL Inc 100miM 2	Acquired By:	Saneesh
Sample Type:	Unknown	Date Acquired:	2016/12/23 01:40:46 AM
Vial:	9	Acq. Method Set:	HDI_EFV_E8H_NEO
Injection #:	1	Date Processed:	2016/12/23 09:46:10 AM
Injection Volume:	15.00 ul	Processing Method:	HDI_EFV_ W2996 245.0nm-1.2
Run Time:	11.0 Minutes	Channel Name:	W2996 245.0nm-1.2
Sample Set Name:	EFV TICL Inhibtr_Asy1 22_12_16	Proc. Chnl. Descr.:	W2996 PDA 245.0 nm at 1.2

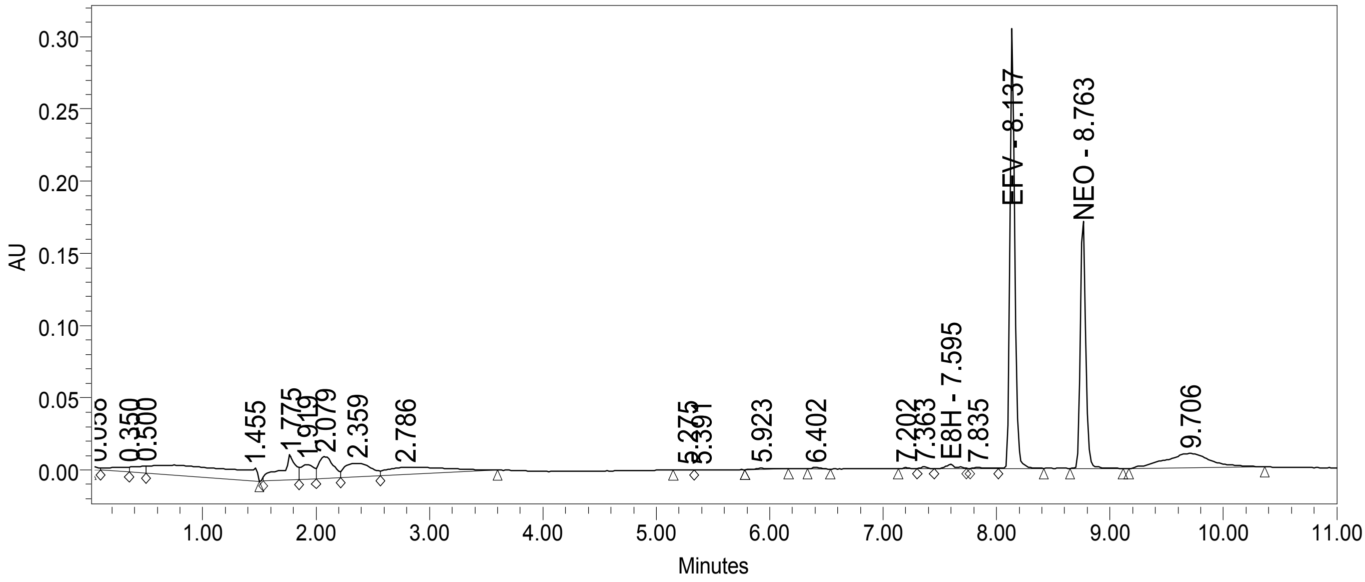

	Peak Name	RT	Area	% Area	Height
1		0.058	7160	0.24	1925
2		0.350	38035	1.28	3465
3		0.500	38267	1.29	4885
4		1.455	401307	13.54	8872
5		1.775	152185	5.13	15844
6		1.919	79524	2.68	10089
7		2.079	133510	4.50	14924
8		2.359	151194	5.10	9315
9		2.786	184834	6.24	4996
10		5.275	2133	0.07	347
11		5.391	3262	0.11	259
12		5.923	8043	0.27	895

Reported by User: Saneesh (Saneesh)   Project Name: HDI_Stellenbosch_2016

	Peak Name	RT	Area	% Area	Height
13		6.402	4943	0.17	1065
14		7.202	2543	0.09	690
15		7.363	4185	0.14	1135
16	E8H	7.595	17014	0.57	2704
17		7.835	4264	0.14	651
18	EFV	8.137	888340	29.97	272572
19	NEO	8.763	560890	18.92	162122
20		9.706	282681	9.54	9878

Multi Sample Summary

Reported by User: Saneesh (Saneesh) Project Name: HDI_Stellenbosch_2016

S A M P L E   I N F O R M A T I O N

Sample Name:	EFV TICL Inc 150miM 1	Acquired By:	Saneesh
Sample Type:	Unknown	Date Acquired:	2016/12/23 01:55:04 AM
Vial:	10	Acq. Method Set:	HDI_EFV_E8H_NEO
Injection #:	1	Date Processed:	2016/12/23 09:46:10 AM
Injection Volume:	15.00 ul	Processing Method:	HDI_EFV_ W2996 245.0nm-1.2
Run Time:	11.0 Minutes	Channel Name:	W2996 245.0nm-1.2
Sample Set Name:	EFV TICL Inhibtr_Asy1 22_12_16	Proc. Chnl. Descr.:	W2996 PDA 245.0 nm at 1.2

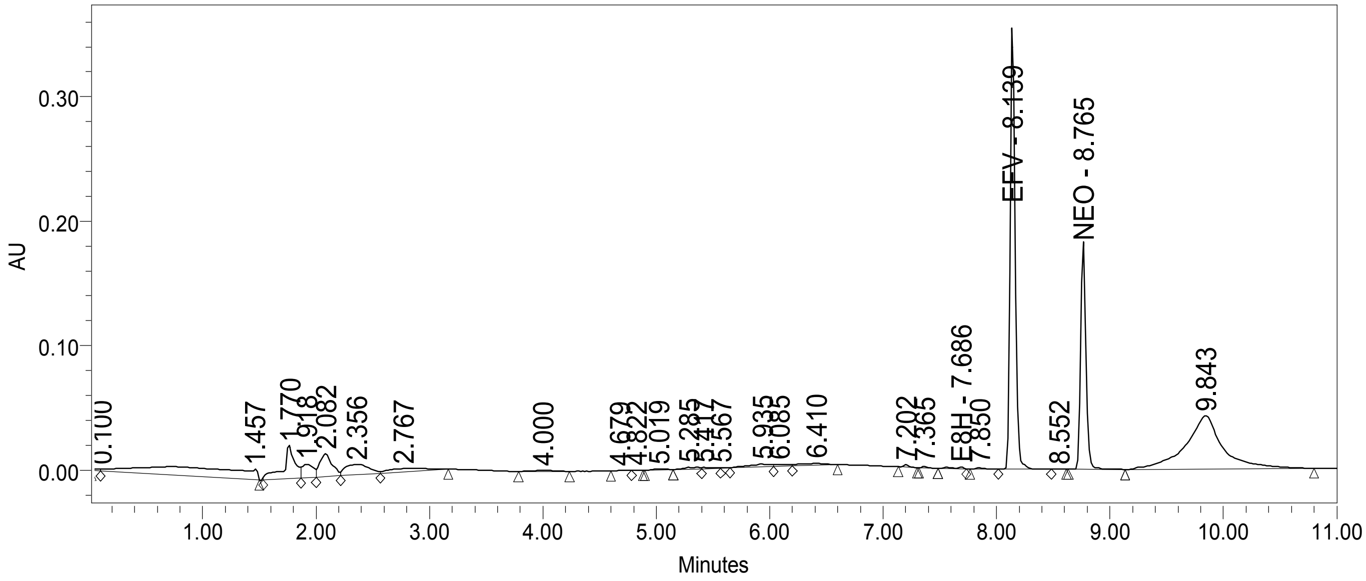

	Peak Name	RT	Area	% Area	Height
1		0.100	4954	0.13	1408
2		1.457	460681	12.07	8526
3		1.770	194343	5.09	26202
4		1.918	71889	1.88	10590
5		2.082	137913	3.61	18137
6		2.356	119702	3.14	7967
7		2.767	63842	1.67	2771
8		4.000	12901	0.34	945
9		4.679	1790	0.05	273
10		4.822	1206	0.03	336
11		5.019	5562	0.15	728
12		5.285	12587	0.33	1487

	Peak Name	RT	Area	% Area	Height
13		5.417	6998	0.18	1034
14		5.567	1717	0.05	442
15		5.935	29696	0.78	2249
16		6.085	12803	0.34	1470
17		6.410	21524	0.56	1739
18		7.202	5463	0.14	1643
19		7.365	4009	0.11	1221
20	E8H	7.686	9972	0.26	1388
21		7.850	4958	0.13	960
22	EFV	8.139	1073180	28.13	322815
23		8.552	776	0.02	166
24	NEO	8.765	580918	15.23	170181
25		9.843	976160	25.58	42723

Reported by User: Saneesh (Saneesh) Project Name: HDI_Stellenbosch_2016

SAMPLE INFORMATION

Sample Name:	EFV TICL Inc 150miM 2	Acquired By:	Saneesh
Sample Type:	Unknown	Date Acquired:	2016/12/23 02:09:22 AM
Vial:	11	Acq. Method Set:	HDI_EFV_E8H_NEO
Injection #:	1	Date Processed:	2016/12/23 09:46:10 AM
Injection Volume:	15.00 ul	Processing Method:	HDI_EFV_ W2996 245.0nm-1.2
Run Time:	11.0 Minutes	Channel Name:	W2996 245.0nm-1.2
Sample Set Name:	EFV TICL Inhibtr_Asy1 22_12_16	Proc. Chnl. Descr.:	W2996 PDA 245.0 nm at 1.2

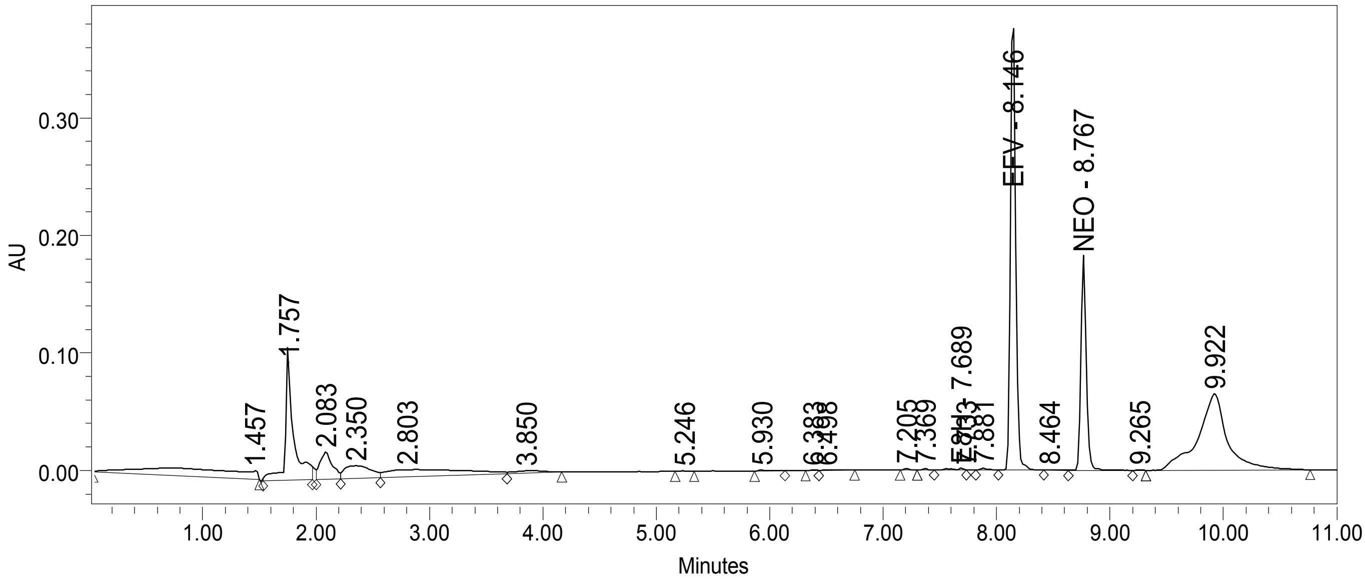

	Peak Name	RT	Area	% Area	Height
1		1.457	460280	9.74	8101
2		1.757	549592	11.63	93994
3		2.083	177672	3.76	22992
4		2.350	173237	3.67	10562
5		2.803	282413	5.98	6083
6		3.850	33825	0.72	1842
7		5.246	2097	0.04	383
8		5.930	4071	0.09	761
9		6.383	1644	0.03	443
10		6.498	3268	0.07	295
11		7.205	4476	0.09	1221
12		7.369	4908	0.10	1364

Reported by User: Saneesh (Saneesh) Project Name: HDI_Stellenbosch_2016

	Peak Name	RT	Area	% Area	Height
13	E8H	7.689	14584	0.31	1600
14		7.733	1657	0.04	524
15		7.881	10203	0.22	1678
16	EFV	8.146	1233434	26.11	359979
17		8.464	3758	0.08	422
18	NEO	8.767	587129	12.43	169008
19		9.265	2159	0.05	644
20		9.922	1173656	24.84	64937

SAMPLE INFORMATION

Sample Name:	EFV TICL Inc 200miM 1	Acquired By:	Saneesh
Sample Type:	Unknown	Date Acquired:	2016/12/23 02:23:41 AM
Vial:	12	Acq. Method Set:	HDI_EFV_E8H_NEO
Injection #:	1	Date Processed:	2016/12/23 09:46:10 AM
Injection Volume:	15.00 ul	Processing Method:	HDI_EFV_ W2996 245.0nm-1.2
Run Time:	11.0 Minutes	Channel Name:	W2996 245.0nm-1.2
Sample Set Name:	EFV TICL Inhibtr_Asy1 22_12_16	Proc. Chnl. Descr.:	W2996 PDA 245.0 nm at 1.2

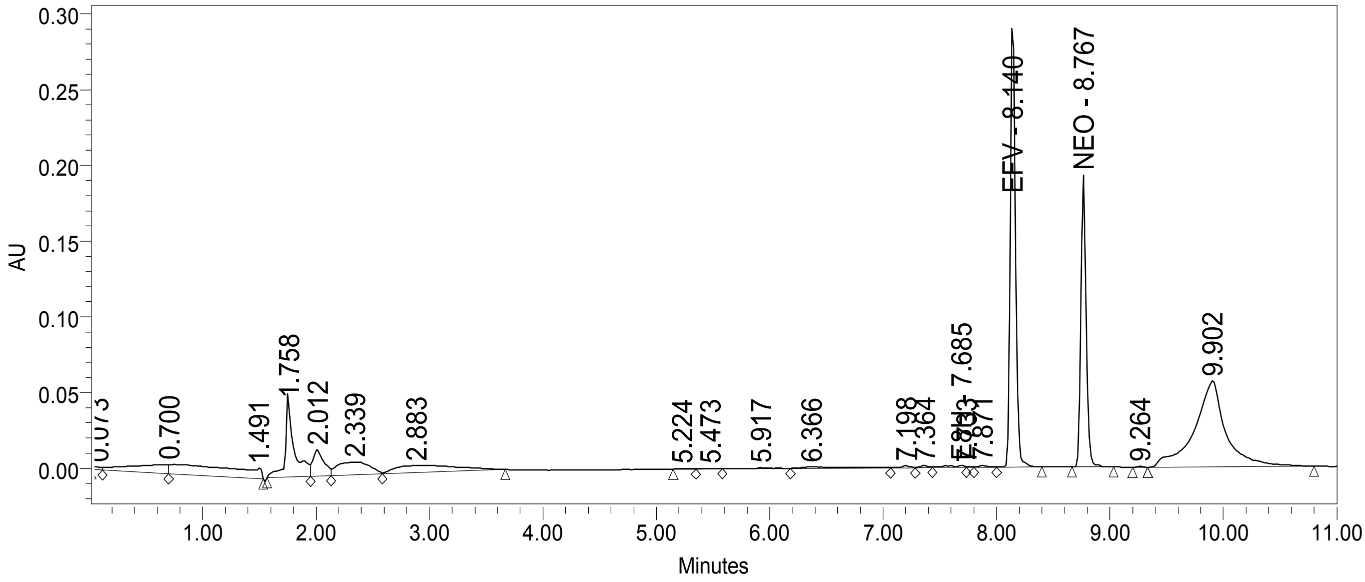

	Peak Name	RT	Area	% Area	Height
1		0.073	7491	0.19	1812
2		0.700	130917	3.37	5935
3		1.491	298413	7.68	7811
4		1.758	283781	7.30	45804
5		2.012	117664	3.03	17083
6		2.339	164881	4.24	8252
7		2.883	173975	4.48	4509
8		5.224	2194	0.06	291
9		5.473	1992	0.05	293
10		5.917	6281	0.16	658
11		6.366	28311	0.73	1011
12		7.198	7879	0.20	1317

Reported by User: Saneesh (Saneesh) Project Name: HDI_Stellenbosch_2016

	Peak Name	RT	Area	% Area	Height
13		7.364	6773	0.17	1422
14	E8H	7.685	14000	0.36	1228
15		7.733	1260	0.03	460
16		7.871	7095	0.18	1228
17	EFV	8.140	929555	23.92	271204
18	NEO	8.767	608722	15.67	178075
19		9.264	2035	0.05	569
20		9.902	1092100	28.11	56561

SAMPLE INFORMATION

Sample Name:	EFV TICL Inc 200miM 2	Acquired By:	Saneesh
Sample Type:	Unknown	Date Acquired:	2016/12/23 02:37:59 AM
Vial:	13	Acq. Method Set:	HDI_EFV_E8H_NEO
Injection #:	1	Date Processed:	2016/12/23 09:46:10 AM
Injection Volume:	15.00 ul	Processing Method:	HDI_EFV_ W2996 245.0nm-1.2
Run Time:	11.0 Minutes	Channel Name:	W2996 245.0nm-1.2
Sample Set Name:	EFV TICL Inhibtr_Asy1 22_12_16	Proc. Chnl. Descr.:	W2996 PDA 245.0 nm at 1.2

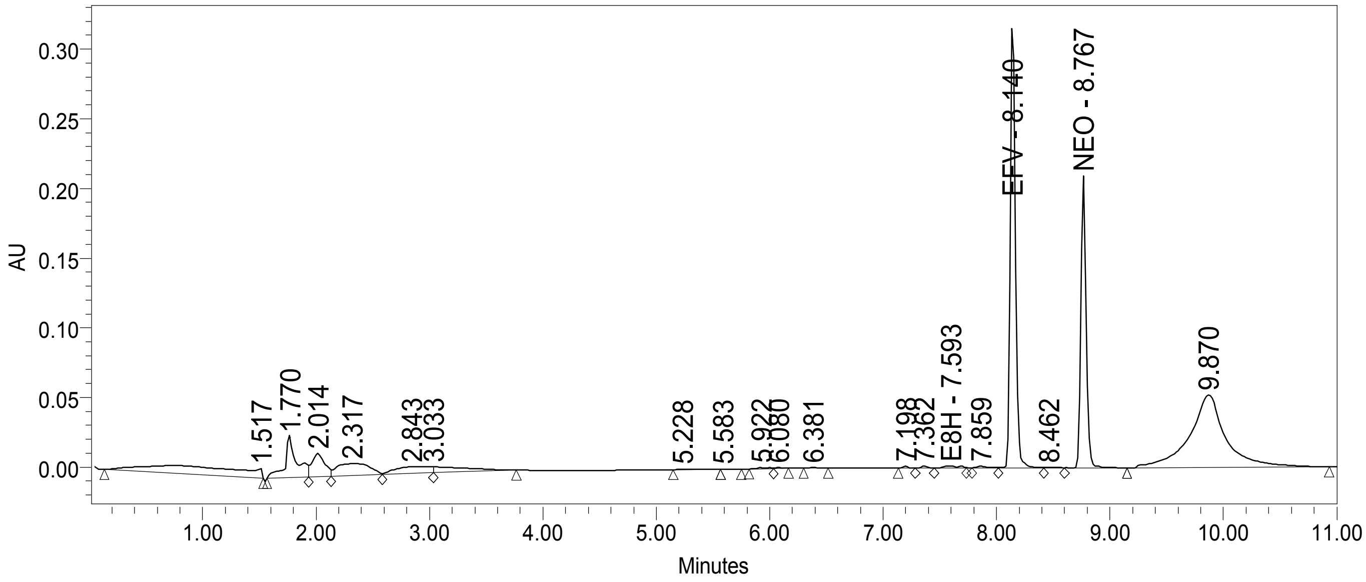

	Peak Name	RT	Area	% Area	Height
1		1.517	354872	9.01	6535
2		1.770	209384	5.31	29007
3		2.014	130248	3.31	16658
4		2.317	170338	4.32	8448
5		2.843	101746	2.58	4644
6		3.033	88262	2.24	4108
7		5.228	3480	0.09	287
8		5.583	807	0.02	115
9		5.922	5957	0.15	892
10		6.080	2543	0.06	534
11		6.381	2414	0.06	381
12		7.198	3807	0.10	1114

Reported by User: Saneesh (Saneesh) Project Name: HDI_Stellenbosch_2016

	Peak Name	RT	Area	% Area	Height
13		7.362	5136	0.13	1348
14	E8H	7.593	14214	0.36	1459
15		7.859	9057	0.23	1219
16	EFV	8.140	1004266	25.49	293405
17		8.462	3905	0.10	447
18	NEO	8.767	664778	16.87	193454
19		9.870	1164511	29.56	52080

Multi Sample Summary

Reported by User: Saneesh (Saneesh)

Project Name: HDI_Stellenbosch_2016

SAMPLE INFORMATION

Sample Name:	EFV TICL Control K1 19.12.16	Acquired By:	Saneesh
Sample Type:	Unknown	Date Acquired:	2016/12/23 03:19:15 AM
Vial:	24	Acq. Method Set:	HDI_EFV_E8H_NEO
Injection #:	1	Date Processed:	2016/12/23 09:47:11 AM
Injection Volume:	15.00 ul	Processing Method:	HDI_EFV_ W2996 245.0nm-1.2
Run Time:	11.0 Minutes	Channel Name:	W2996 245.0nm-1.2
Sample Set Name:	EFV TICL Inhibtr_Asy2 22_12_16	Proc. Chnl. Descr.:	W2996 PDA 245.0 nm at 1.2

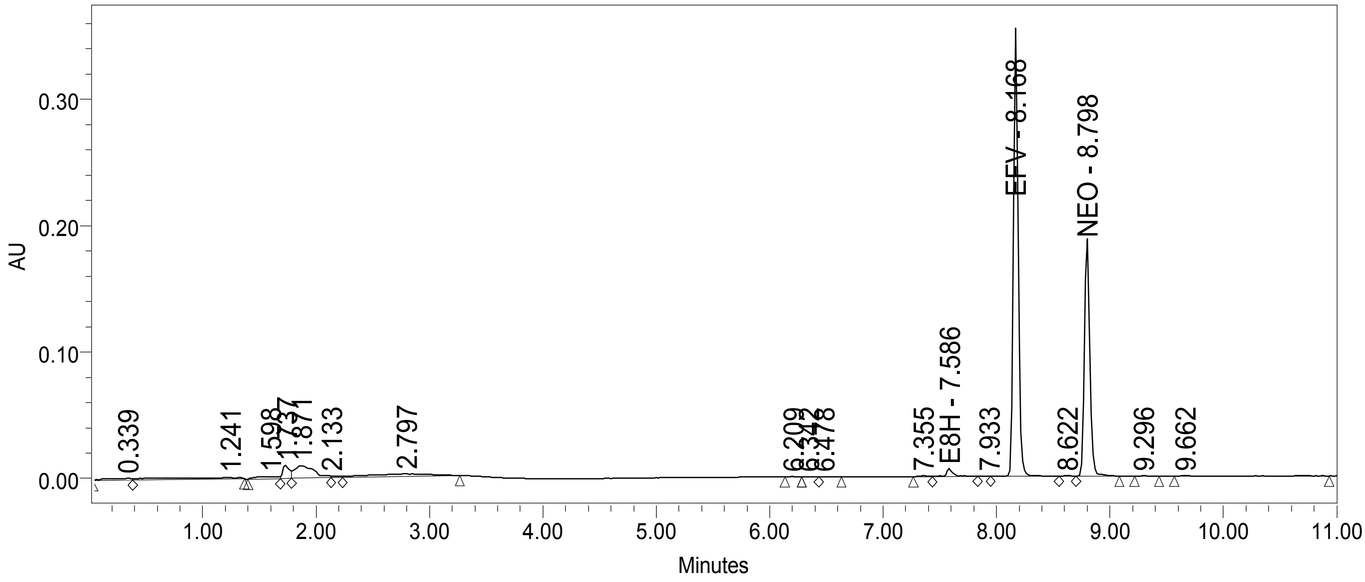

	Peak Name	RT	Area	% Area	Height
1		0.339	21215	1.03	1249
2		1.241	68059	3.30	1385
3		1.598	25404	1.23	1737
4		1.737	41789	2.03	10439
5		1.871	118708	5.76	9908
6		2.133	6260	0.30	1354
7		2.797	75522	3.66	1861
8		6.209	1586	0.08	379
9		6.342	612	0.03	137
10		6.478	1759	0.09	242
11		7.355	3420	0.17	832
12	E8H	7.586	27400	1.33	5688
13		7.933	1711	0.08	298
14	EFV	8.168	1040569	50.49	318853

Reported by User: Saneesh (Saneesh) Project Name: HDI_Stellenbosch_2016

	Peak Name	RT	Area	% Area	Height
15		8.622	2348	0.11	531
16	NEO	8.798	611222	29.66	176233
17		9.296	1529	0.07	371
18		9.662	11770	0.57	492

SAMPLE INFORMATION

Sample Name:	EFV TICL Inc 1miM 1	Acquired By:	Saneesh
Sample Type:	Unknown	Date Acquired:	2016/12/23 03:33:34 AM
Vial:	2	Acq. Method Set:	HDI_EFV_E8H_NEO
Injection #:	1	Date Processed:	2016/12/23 09:47:11 AM
Injection Volume:	15.00 ul	Processing Method:	HDI_EFV_ W2996 245.0nm-1.2
Run Time:	11.0 Minutes	Channel Name:	W2996 245.0nm-1.2
Sample Set Name:	EFV TICL Inhibtr_Asy2 22_12_16	Proc. Chnl. Descr.:	W2996 PDA 245.0 nm at 1.2

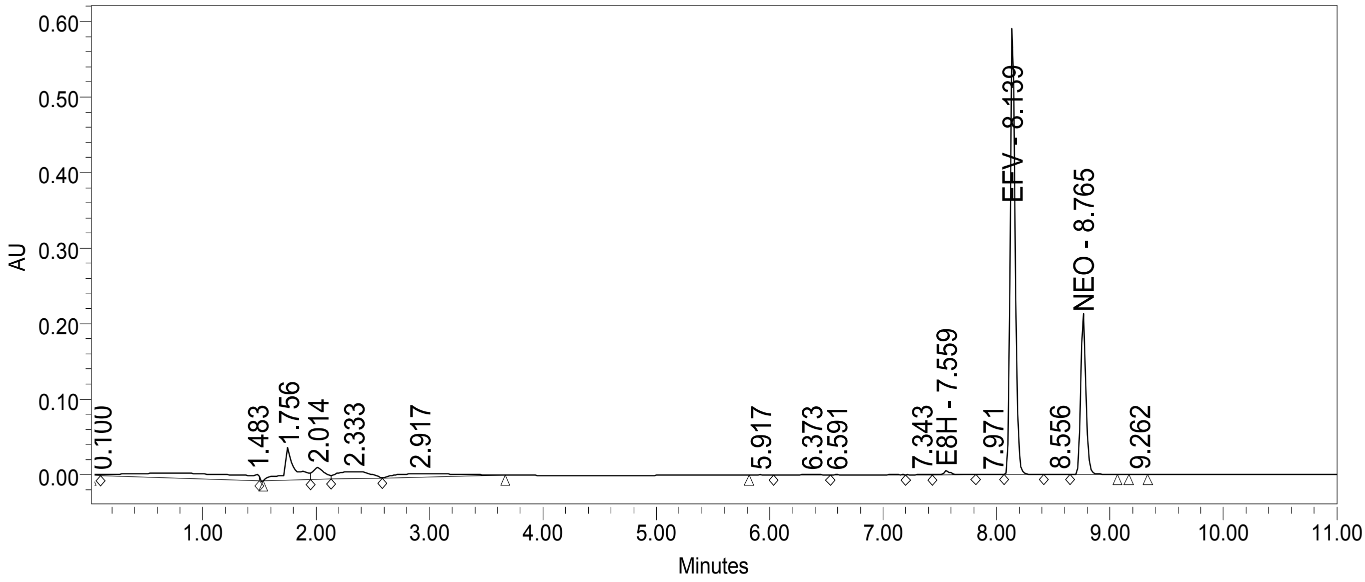

	Peak Name	RT	Area	% Area	Height
1		0.100	4163	0.11	1115
2		1.483	480656	12.72	8932
3		1.756	290618	7.69	39119
4		2.014	117674	3.11	15835
5		2.333	190967	5.05	9418
6		2.917	185593	4.91	4772
7		5.917	2477	0.07	507
8		6.373	9732	0.26	785
9		6.591	6903	0.18	308
10		7.343	4491	0.12	966
11	E8H	7.559	25953	0.69	5064
12		7.971	3579	0.09	425

	Peak Name	RT	Area	% Area	Height
13	EFV	8.139	1778646	47.06	538115
14		8.556	4200	0.11	533
15	NEO	8.765	672229	17.79	197482
16		9.262	1634	0.04	387

Reported by User: Saneesh (Saneesh)   Project Name: HDI_Stellenbosch_2016

SAMPLE INFORMATION

Sample Name:	EFV TICL Inc 1miM 2	Acquired By:	Saneesh
Sample Type:	Unknown	Date Acquired:	2016/12/23 03:47:52 AM
Vial:	3	Acq. Method Set:	HDI_EFV_E8H_NEO
Injection #:	1	Date Processed:	2016/12/23 09:47:11 AM
Injection Volume:	15.00 ul	Processing Method:	HDI_EFV_ W2996 245.0nm-1.2
Run Time:	11.0 Minutes	Channel Name:	W2996 245.0nm-1.2
Sample Set Name:	EFV TICL Inhibtr_Asy2 22_12_16	Proc. Chnl. Descr.:	W2996 PDA 245.0 nm at 1.2

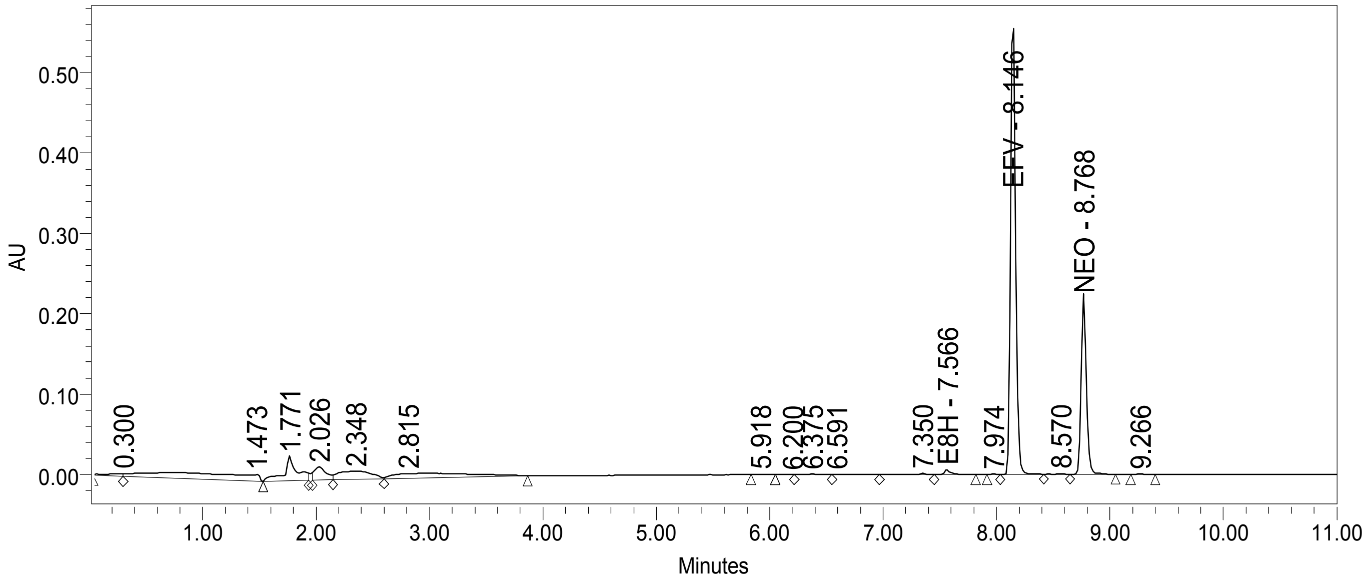

	Peak Name	RT	Area	% Area	Height
1		0.300	30216	0.80	2985
2		1.473	441609	11.64	8670
3		1.771	235671	6.21	28510
4		2.026	119727	3.16	16123
5		2.348	205635	5.42	9888
6		2.815	266779	7.03	5843
7		5.918	2155	0.06	513
8		6.200	1305	0.03	223
9		6.375	7932	0.21	925
10		6.591	3358	0.09	273
11		7.350	7969	0.21	1220
12	E8H	7.566	22332	0.59	5292

Reported by User: Saneesh (Saneesh) Project Name: HDI_Stellenbosch_2016

	Peak Name	RT	Area	% Area	Height
13		7.974	1061	0.03	304
14	EFV	8.146	1743683	45.98	527570
15		8.570	4256	0.11	663
16	NEO	8.768	696807	18.37	205731
17		9.266	1983	0.05	515

Reported by User: Saneesh (Saneesh) Project Name: HDI_Stellenbosch_2016

SAMPLE INFORMATION

Sample Name:	EFV TICL Inc 10miM 1	Acquired By:	Saneesh
Sample Type:	Unknown	Date Acquired:	2016/12/23 04:02:10 AM
Vial:	4	Acq. Method Set:	HDI_EFV_E8H_NEO
Injection #:	1	Date Processed:	2016/12/23 09:47:11 AM
Injection Volume:	15.00 ul	Processing Method:	HDI_EFV_ W2996 245.0nm-1.2
Run Time:	11.0 Minutes	Channel Name:	W2996 245.0nm-1.2
Sample Set Name:	EFV TICL Inhibtr_Asy2 22_12_16	Proc. Chnl. Descr.:	W2996 PDA 245.0 nm at 1.2

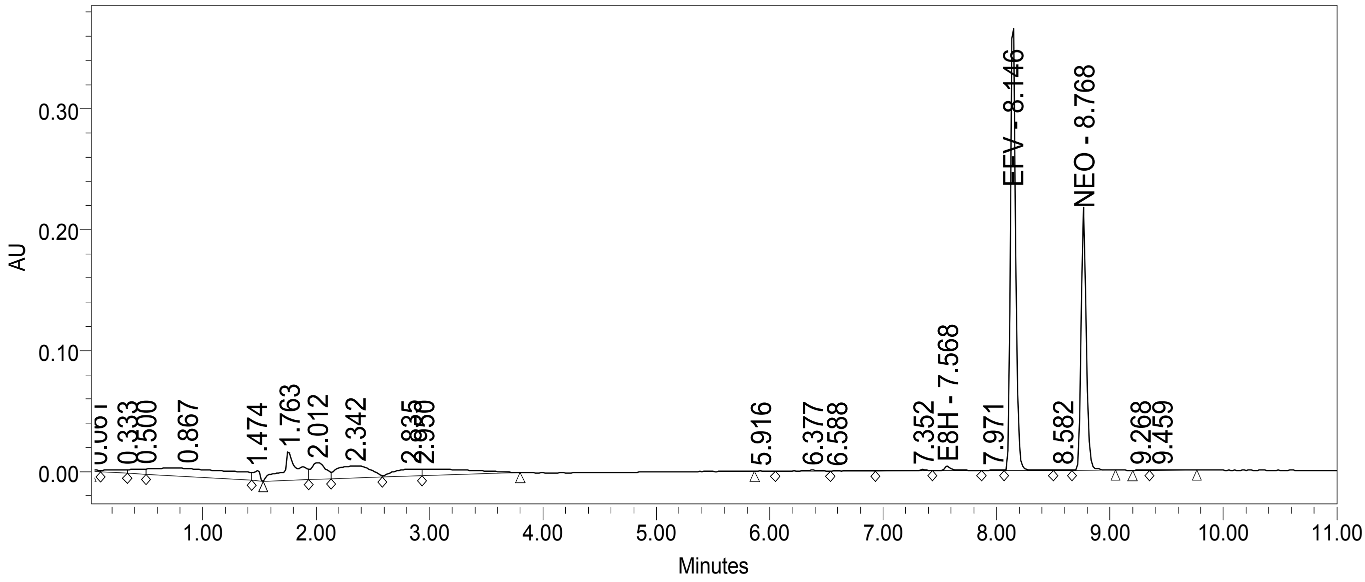

	Peak Name	RT	Area	% Area	Height
1		0.061	3966	0.13	1075
2		0.333	28475	0.90	2993
3		0.500	37441	1.19	4441
4		0.867	357667	11.35	6856
5		1.474	33928	1.08	8830
6		1.763	221398	7.02	21528
7		2.012	123050	3.90	13979
8		2.342	205780	6.53	9846
9		2.835	97059	3.08	5723
10		2.950	154835	4.91	5474
11		5.916	1083	0.03	291
12		6.377	7696	0.24	855

Reported by User: Saneesh (Saneesh) Project Name: HDI_Stellenbosch_2016

	Peak Name	RT	Area	% Area	Height
13		6.588	3142	0.10	230
14		7.352	9128	0.29	1071
15	E8H	7.568	20274	0.64	3514
16		7.971	3112	0.10	405
17	EFV	8.146	1161305	36.84	349358
18		8.582	2572	0.08	511
19	NEO	8.768	673568	21.37	199357
20		9.268	1528	0.05	392
21		9.459	5525	0.18	400

Reported by User: Saneesh (Saneesh)   Project Name: HDI_Stellenbosch_2016

SAMPLE INFORMATION

Sample Name:	EFV TICL Inc 10miM 2	Acquired By:	Saneesh
Sample Type:	Unknown	Date Acquired:	2016/12/23 04:16:39 AM
Vial:	5	Acq. Method Set:	HDI_EFV_E8H_NEO
Injection #:	1	Date Processed:	2016/12/23 09:47:11 AM
Injection Volume:	15.00 ul	Processing Method:	HDI_EFV_ W2996 245.0nm-1.2
Run Time:	11.0 Minutes	Channel Name:	W2996 245.0nm-1.2
Sample Set Name:	EFV TICL Inhibtr_Asy2 22_12_16	Proc. Chnl. Descr.:	W2996 PDA 245.0 nm at 1.2

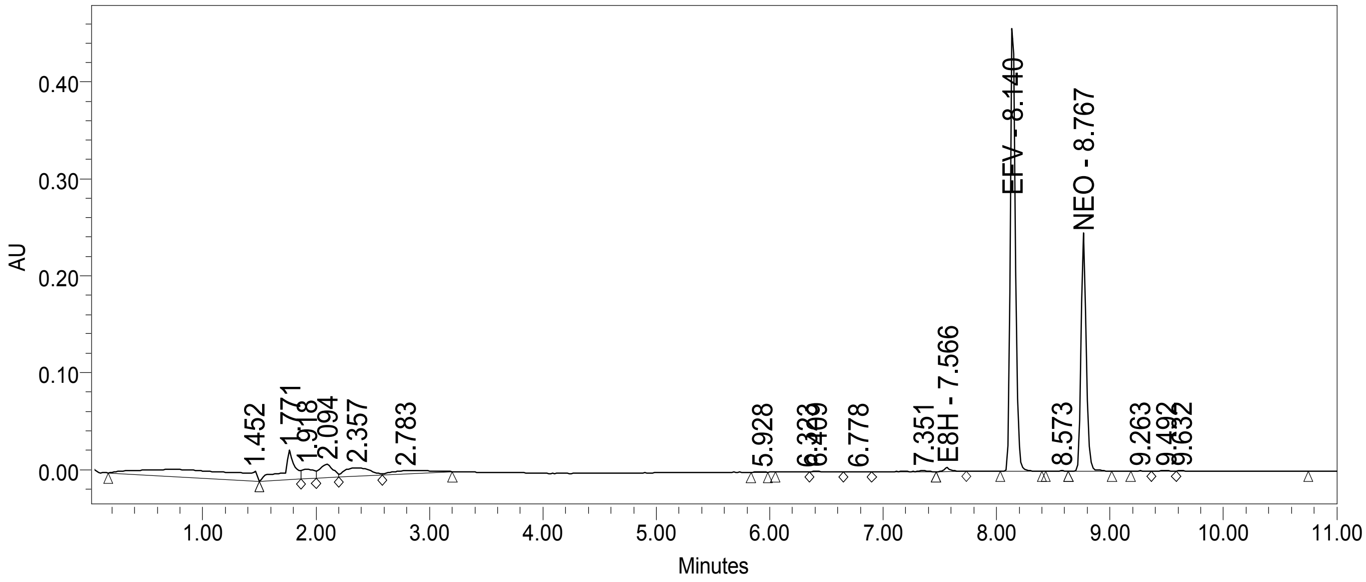

	Peak Name	RT	Area	% Area	Height
1		1.452	490156	14.57	9575
2		1.771	213668	6.35	28139
3		1.918	72930	2.17	10125
4		2.094	117656	3.50	14116
5		2.357	140137	4.17	8758
6		2.783	79445	2.36	3341
7		5.928	1365	0.04	389
8		6.323	2202	0.07	274
9		6.409	4876	0.14	660
10		6.778	1199	0.04	108
11		7.351	7946	0.24	1099
12	E8H	7.566	18247	0.54	3940

Reported by User: Saneesh (Saneesh) Project Name: HDI_Stellenbosch_2016

	Peak Name	RT	Area	% Area	Height
13	EFV	8.140	1427249	42.42	424783
14		8.573	2216	0.07	473
15	NEO	8.767	766505	22.78	226142
16		9.263	2055	0.06	496
17		9.492	4905	0.15	533
18		9.632	11661	0.35	552

S A M P L E   I N F O R M A T I O N

Sample Name:	EFV TICL Inc 25miM 1	Acquired By:	Saneesh
Sample Type:	Unknown	Date Acquired:	2016/12/23 04:31:07 AM
Vial:	6	Acq. Method Set:	HDI_EFV_E8H_NEO
Injection #:	1	Date Processed:	2016/12/23 09:47:11 AM
Injection Volume:	15.00 ul	Processing Method:	HDI_EFV_ W2996 245.0nm-1.2
Run Time:	11.0 Minutes	Channel Name:	W2996 245.0nm-1.2
Sample Set Name:	EFV TICL Inhibtr_Asy2 22_12_16	Proc. Chnl. Descr.:	W2996 PDA 245.0 nm at 1.2

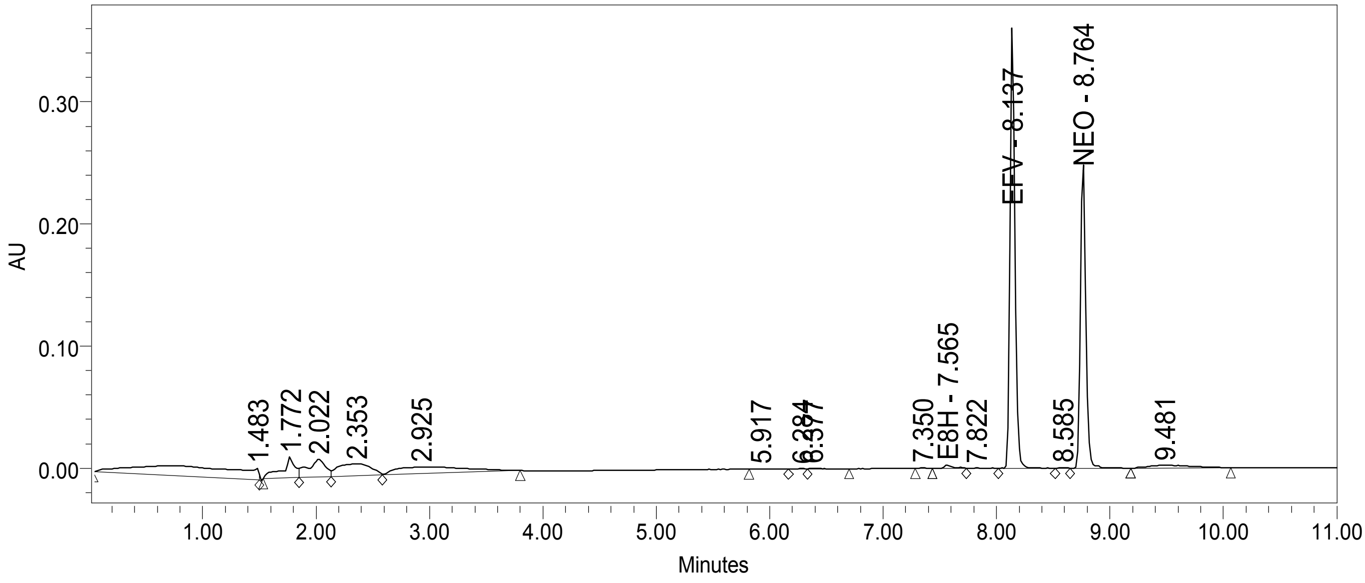

	Peak Name	RT	Area	% Area	Height
1		1.483	569615	17.36	9719
2		1.772	133984	4.08	15720
3		2.022	157713	4.81	14366
4		2.353	196119	5.98	9486
5		2.925	235059	7.16	5374
6		5.917	4545	0.14	461
7		6.284	2945	0.09	490
8		6.377	4987	0.15	634
9		7.350	3054	0.09	870
10	E8H	7.565	16146	0.49	2789
11		7.822	4595	0.14	386
12	EFV	8.137	1079441	32.89	326460

Reported by User: Saneesh (Saneesh) Project Name: HDI_Stellenbosch_2016

	Peak Name	RT	Area	% Area	Height
13		8.585	2876	0.09	573
14	NEO	8.764	812790	24.77	233982
15		9.481	58054	1.77	2104

SAMPLE INFORMATION

Sample Name:	EFV TICL Inc 25miM 2	Acquired By:	Saneesh
Sample Type:	Unknown	Date Acquired:	2016/12/23 04:45:25 AM
Vial:	7	Acq. Method Set:	HDI_EFV_E8H_NEO
Injection #:	1	Date Processed:	2016/12/23 09:47:11 AM
Injection Volume:	15.00 ul	Processing Method:	HDI_EFV_ W2996 245.0nm-1.2
Run Time:	11.0 Minutes	Channel Name:	W2996 245.0nm-1.2
Sample Set Name:	EFV TICL Inhibtr_Asy2 22_12_16	Proc. Chnl. Descr.:	W2996 PDA 245.0 nm at 1.2

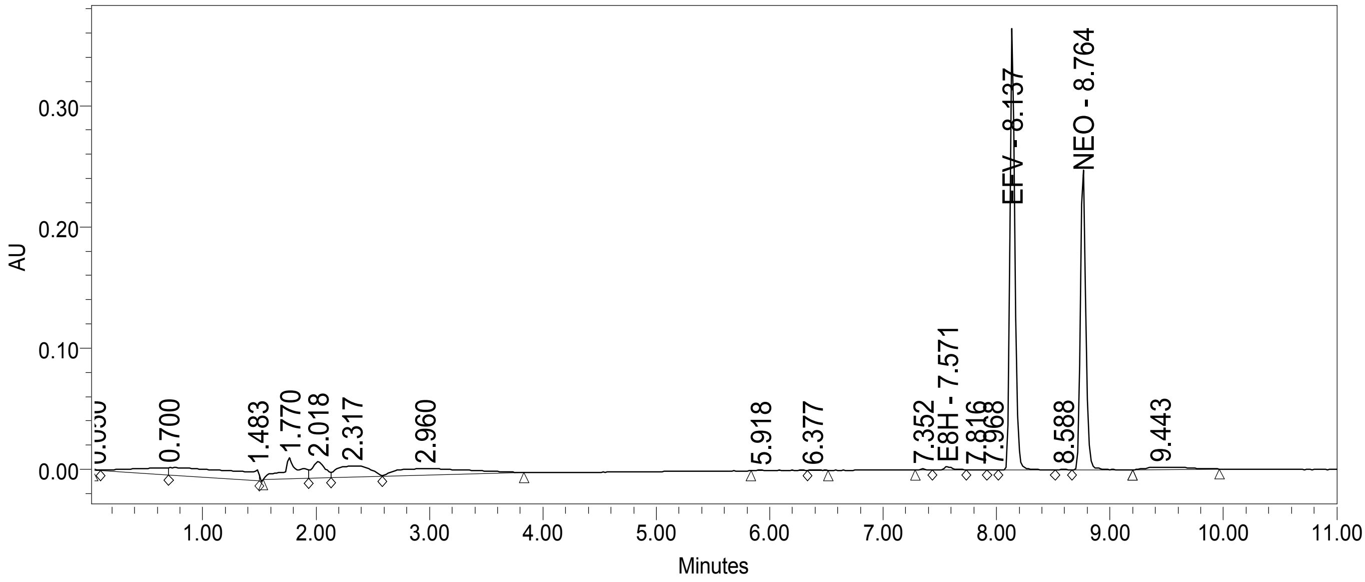

	Peak Name	RT	Area	% Area	Height
1		0.050	2473	0.08	989
2		0.700	126329	3.99	6198
3		1.483	330840	10.45	9175
4		1.770	174120	5.50	16777
5		2.018	109989	3.47	13512
6		2.317	190184	6.01	9296
7		2.960	241246	7.62	5403
8		5.918	7614	0.24	526
9		6.377	3372	0.11	638
10		7.352	3876	0.12	1084
11	E8H	7.571	17842	0.56	2901
12		7.816	2691	0.08	355

Reported by User: Saneesh (Saneesh) Project Name: HDI_Stellenbosch_2016

	Peak Name	RT	Area	% Area	Height
13		7.968	1590	0.05	331
14	EFV	8.137	1086903	34.32	329027
15		8.588	3290	0.10	621
16	NEO	8.764	813769	25.70	233588
17		9.443	50534	1.60	1942

Reported by User: Saneesh (Saneesh) Project Name: HDI_Stellenbosch_2016

SAMPLE INFORMATION

Sample Name:	EFV TICL Inc 50miM 1	Acquired By:	Saneesh
Sample Type:	Unknown	Date Acquired:	2016/12/23 04:59:43 AM
Vial:	8	Acq. Method Set:	HDI_EFV_E8H_NEO
Injection #:	1	Date Processed:	2016/12/23 09:47:11 AM
Injection Volume:	15.00 ul	Processing Method:	HDI_EFV_ W2996 245.0nm-1.2
Run Time:	11.0 Minutes	Channel Name:	W2996 245.0nm-1.2
Sample Set Name:	EFV TICL Inhibtr_Asy2 22_12_16	Proc. Chnl. Descr.:	W2996 PDA 245.0 nm at 1.2

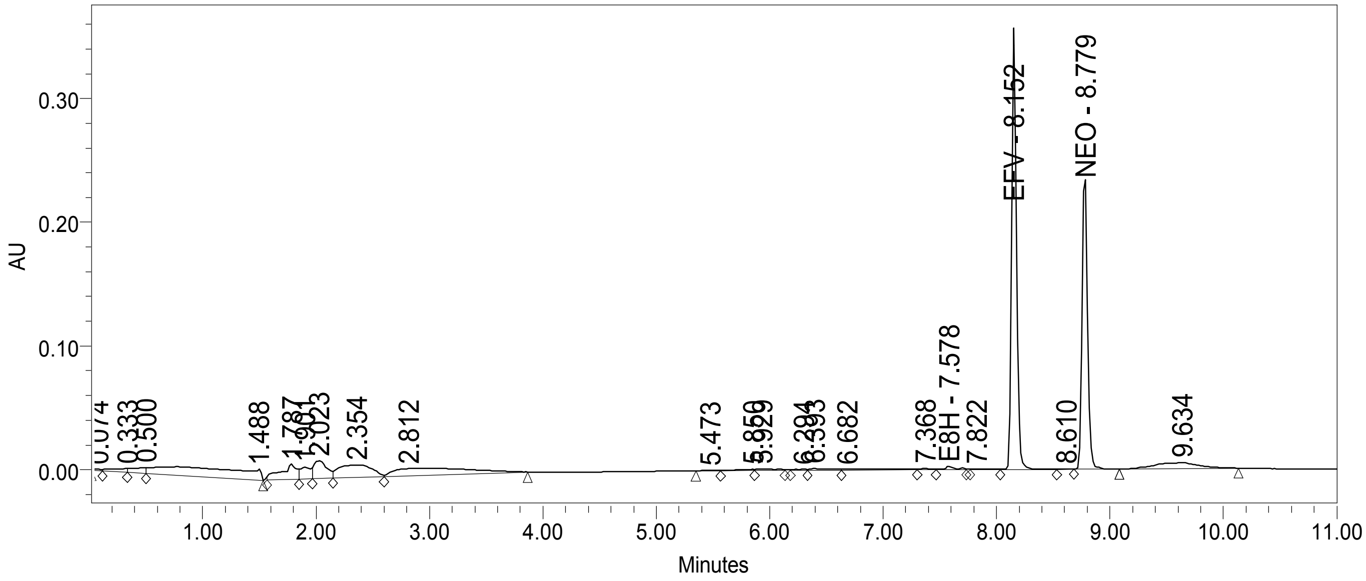

	Peak Name	RT	Area	% Area	Height
1		0.074	4466	0.13	1131
2		0.333	27640	0.83	3027
3		0.500	38059	1.14	4494
4		1.488	401984	12.09	8724
5		1.787	121717	3.66	12139
6		1.901	61440	1.85	9610
7		2.023	115058	3.46	14365
8		2.354	204563	6.15	9902
9		2.812	266754	8.02	5807
10		5.473	2265	0.07	329
11		5.850	10104	0.30	930
12		5.929	15583	0.47	1286

	Peak Name	RT	Area	% Area	Height
13		6.294	7009	0.21	863
14		6.393	18409	0.55	1790
15		6.682	28292	0.85	827
16		7.368	7841	0.24	1416
17	E8H	7.578	19736	0.59	2579
18		7.822	8423	0.25	847
19	EFV	8.152	1043264	31.38	319452
20		8.610	2475	0.07	436
21	NEO	8.779	785873	23.63	224569
22		9.634	134162	4.03	4906

SAMPLE INFORMATION

Sample Name:	EFV TICL Inc 50miM 2	Acquired By:	Saneesh
Sample Type:	Unknown	Date Acquired:	2016/12/23 05:14:01 AM
Vial:	9	Acq. Method Set:	HDI_EFV_E8H_NEO
Injection #:	1	Date Processed:	2016/12/23 09:47:11 AM
Injection Volume:	15.00 ul	Processing Method:	HDI_EFV_ W2996 245.0nm-1.2
Run Time:	11.0 Minutes	Channel Name:	W2996 245.0nm-1.2
Sample Set Name:	EFV TICL Inhibtr_Asy2 22_12_16	Proc. Chnl. Descr.:	W2996 PDA 245.0 nm at 1.2

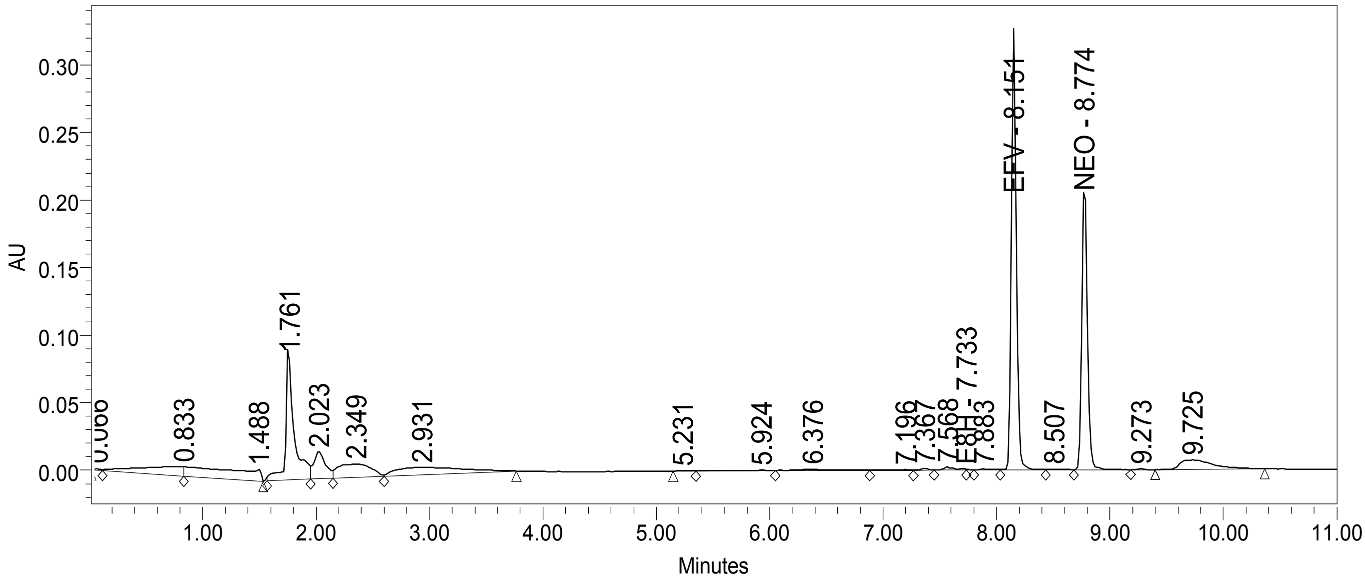

	Peak Name	RT	Area	% Area	Height
1		0.066	5503	0.16	1267
2		0.833	178822	5.16	6789
3		1.488	278778	8.05	8667
4		1.761	491589	14.19	85292
5		2.023	148351	4.28	19922
6		2.349	200912	5.80	9843
7		2.931	227757	6.58	5549
8		5.231	2007	0.06	261
9		5.924	10114	0.29	603
10		6.376	28728	0.83	1041
11		7.196	11598	0.33	646
12		7.367	7417	0.21	1311

	Peak Name	RT	Area	% Area	Height
13		7.568	19590	0.57	2349
14	E8H	7.733	1785	0.05	703
15		7.883	8527	0.25	925
16	EFV	8.151	982176	28.35	295386
17		8.507	6220	0.18	635
18	NEO	8.774	710960	20.52	196858
19		9.273	2706	0.08	591
20		9.725	140413	4.05	6995

SAMPLE INFORMATION

Sample Name:	EFV TICL Inc 75miM 1	Acquired By:	Saneesh
Sample Type:	Unknown	Date Acquired:	2016/12/23 05:28:19 AM
Vial:	10	Acq. Method Set:	HDI_EFV_E8H_NEO
Injection #:	1	Date Processed:	2016/12/23 09:47:11 AM
Injection Volume:	15.00 ul	Processing Method:	HDI_EFV_ W2996 245.0nm-1.2
Run Time:	11.0 Minutes	Channel Name:	W2996 245.0nm-1.2
Sample Set Name:	EFV TICL Inhibtr_Asy2 22_12_16	Proc. Chnl. Descr.:	W2996 PDA 245.0 nm at 1.2

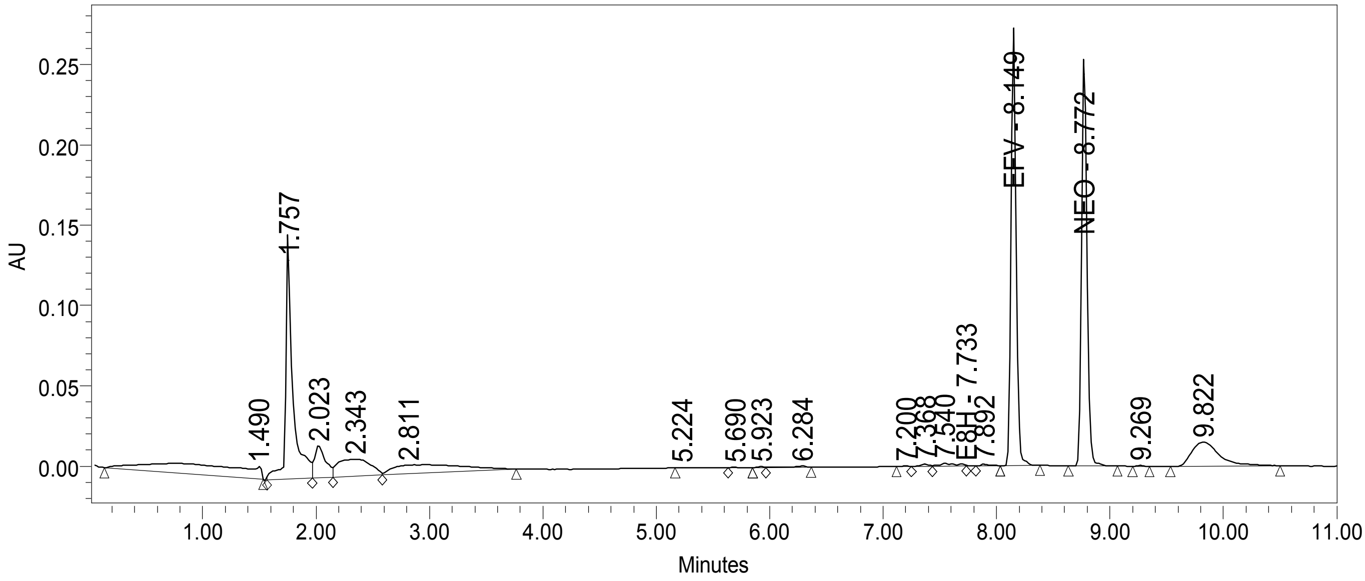

	Peak Name	RT	Area	% Area	Height
1		1.490	405523	11.28	8097
2		1.757	644312	17.92	127974
3		2.023	137055	3.81	19747
4		2.343	212261	5.90	10552
5		2.811	228964	6.37	5356
6		5.224	3701	0.10	263
7		5.690	1482	0.04	249
8		5.923	2004	0.06	586
9		6.284	6162	0.17	705
10		7.200	1120	0.03	278
11		7.368	5716	0.16	1154
12		7.540	18426	0.51	1949

Reported by User: Saneesh (Saneesh) Project Name: HDI_Stellenbosch_2016

	Peak Name	RT	Area	% Area	Height
13	E8H	7.733	1019	0.03	485
14		7.892	5095	0.14	1001
15	EFV	8.149	836379	23.26	249572
16	NEO	8.772	833818	23.19	237156
17		9.269	1948	0.05	543
18		9.822	250968	6.98	14971

SAMPLE INFORMATION

Sample Name:	EFV TICL Inc 75miM 2	Acquired By:	Saneesh
Sample Type:	Unknown	Date Acquired:	2016/12/23 05:42:37 AM
Vial:	11	Acq. Method Set:	HDI_EFV_E8H_NEO
Injection #:	1	Date Processed:	2016/12/23 09:47:11 AM
Injection Volume:	15.00 ul	Processing Method:	HDI_EFV_ W2996 245.0nm-1.2
Run Time:	11.0 Minutes	Channel Name:	W2996 245.0nm-1.2
Sample Set Name:	EFV TICL Inhibtr_Asy2 22_12_16	Proc. Chnl. Descr.:	W2996 PDA 245.0 nm at 1.2

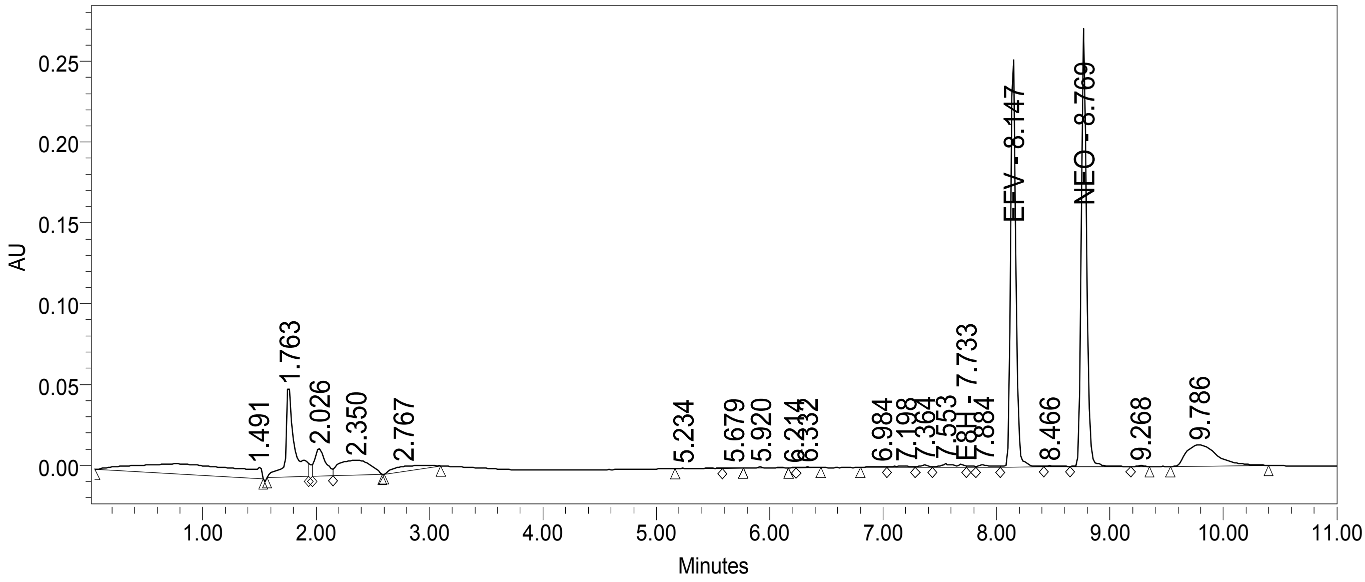

	Peak Name	RT	Area	% Area	Height
1		1.491	435798	14.16	7620
2		1.763	296257	9.63	49258
3		2.026	113478	3.69	16760
4		2.350	177980	5.78	9082
5		2.767	59095	1.92	3090
6		5.234	2818	0.09	212
7		5.679	1069	0.03	178
8		5.920	3135	0.10	561
9		6.214	291	0.01	106
10		6.332	2237	0.07	395
11		6.984	5054	0.16	505
12		7.198	8342	0.27	731

Reported by User: Saneesh (Saneesh) Project Name: HDI_Stellenbosch_2016

	Peak Name	RT	Area	% Area	Height
13		7.364	6858	0.22	1340
14		7.553	20433	0.66	2080
15	E8H	7.733	2401	0.08	712
16		7.884	10010	0.33	1319
17	EFV	8.147	807617	26.24	237351
18		8.466	5979	0.19	726
19	NEO	8.769	866313	28.15	249792
20		9.268	2862	0.09	648
21		9.786	249412	8.10	13130

SAMPLE INFORMATION

Sample Name:	EFV TICL Inc 100miM 1	Acquired By:	Saneesh
Sample Type:	Unknown	Date Acquired:	2016/12/23 05:57:06 AM
Vial:	12	Acq. Method Set:	HDI_EFV_E8H_NEO
Injection #:	1	Date Processed:	2016/12/23 09:47:11 AM
Injection Volume:	15.00 ul	Processing Method:	HDI_EFV_ W2996 245.0nm-1.2
Run Time:	11.0 Minutes	Channel Name:	W2996 245.0nm-1.2
Sample Set Name:	EFV TICL Inhibtr_Asy2 22_12_16	Proc. Chnl. Descr.:	W2996 PDA 245.0 nm at 1.2

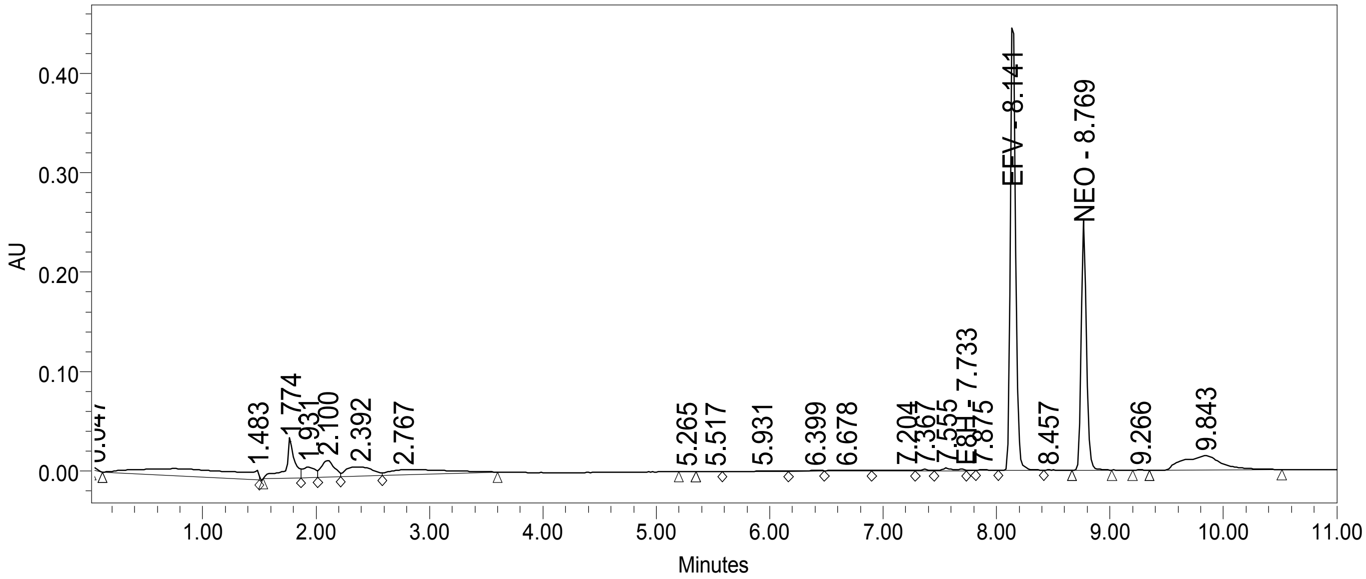

	Peak Name	RT	Area	% Area	Height
1		0.047	8003	0.20	2558
2		1.483	486805	12.42	9600
3		1.774	211858	5.41	34135
4		1.931	79841	2.04	10450
5		2.100	131603	3.36	16532
6		2.392	148941	3.80	9165
7		2.767	173909	4.44	4902
8		5.265	1296	0.03	261
9		5.517	2054	0.05	194
10		5.931	7431	0.19	632
11		6.399	7608	0.19	817
12		6.678	15106	0.39	679

Reported by User: Saneesh (Saneesh) Project Name: HDI_Stellenbosch_2016

	Peak Name	RT	Area	% Area	Height
13		7.204	12607	0.32	816
14		7.367	7066	0.18	1332
15		7.555	20722	0.53	2665
16	E8H	7.733	1923	0.05	600
17		7.875	7706	0.20	1113
18	EFV	8.141	1452511	37.06	420948
19		8.457	2891	0.07	369
20	NEO	8.769	791927	20.21	231462
21		9.266	1778	0.05	517
22		9.843	345700	8.82	14232

Reported by User: Saneesh (Saneesh)   Project Name: HDI_Stellenbosch_2016

SAMPLE INFORMATION

Sample Name:	EFV TICL Inc 100miM 2	Acquired By:	Saneesh
Sample Type:	Unknown	Date Acquired:	2016/12/23 06:11:24 AM
Vial:	13	Acq. Method Set:	HDI_EFV_E8H_NEO
Injection #:	1	Date Processed:	2016/12/23 09:47:11 AM
Injection Volume:	15.00 ul	Processing Method:	HDI_EFV_ W2996 245.0nm-1.2
Run Time:	11.0 Minutes	Channel Name:	W2996 245.0nm-1.2
Sample Set Name:	EFV TICL Inhibtr_Asy2 22_12_16	Proc. Chnl. Descr.:	W2996 PDA 245.0 nm at 1.2

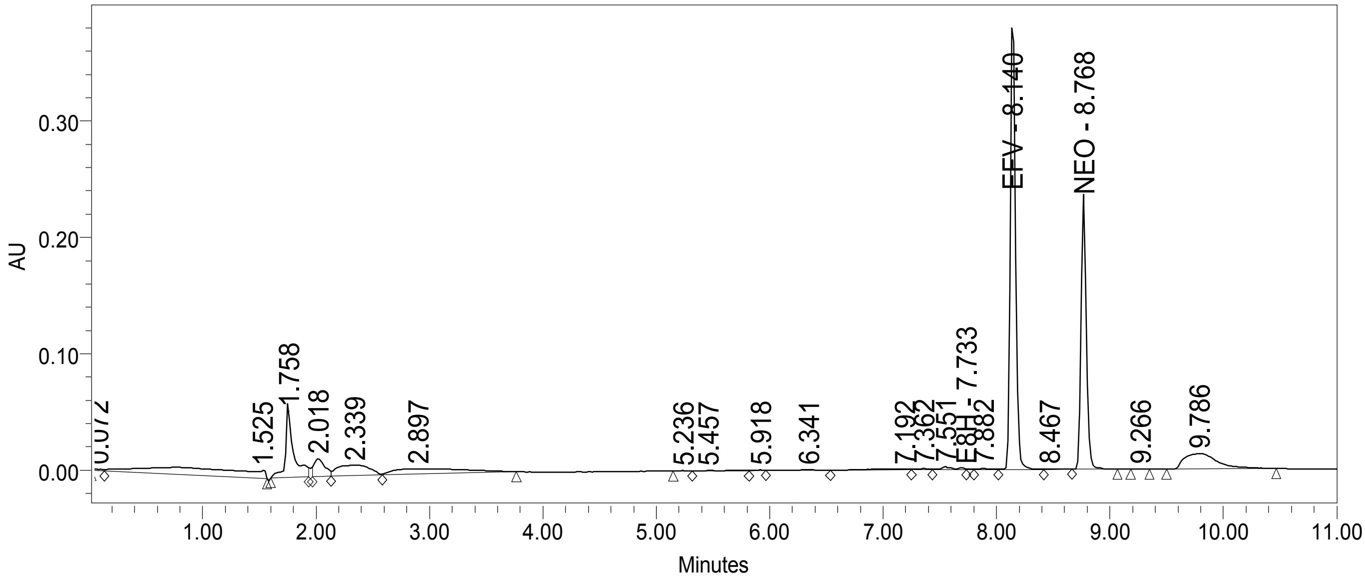

	Peak Name	RT	Area	% Area	Height
1		0.072	7316	0.21	1440
2		1.525	414383	11.74	7483
3		1.758	291264	8.25	52994
4		2.018	102613	2.91	15370
5		2.339	179312	5.08	8922
6		2.897	198063	5.61	4613
7		5.236	1457	0.04	241
8		5.457	3560	0.10	236
9		5.918	2235	0.06	554
10		6.341	9618	0.27	726
11		7.192	22852	0.65	847
12		7.362	10002	0.28	1560

Reported by User: Saneesh (Saneesh) Project Name: HDI_Stellenbosch_2016

	Peak Name	RT	Area	% Area	Height
13		7.551	24426	0.69	2595
14	E8H	7.733	2386	0.07	717
15		7.882	10120	0.29	1245
16	EFV	8.140	1228302	34.81	356813
17		8.467	3826	0.11	481
18	NEO	8.768	745596	21.13	217446
19		9.266	1874	0.05	488
20		9.786	269873	7.65	13380

Reported by User: Saneesh (Saneesh)   Project Name: HDI_Stellenbosch_2016

SAMPLE INFORMATION

Sample Name:	EFV TICL Inc 150miM 1	Acquired By:	Saneesh
Sample Type:	Unknown	Date Acquired:	2016/12/23 06:25:42 AM
Vial:	14	Acq. Method Set:	HDI_EFV_E8H_NEO
Injection #:	1	Date Processed:	2016/12/23 09:47:11 AM
Injection Volume:	15.00 ul	Processing Method:	HDI_EFV_ W2996 245.0nm-1.2
Run Time:	11.0 Minutes	Channel Name:	W2996 245.0nm-1.2
Sample Set Name:	EFV TICL Inhibtr_Asy2 22_12_16	Proc. Chnl. Descr.:	W2996 PDA 245.0 nm at 1.2

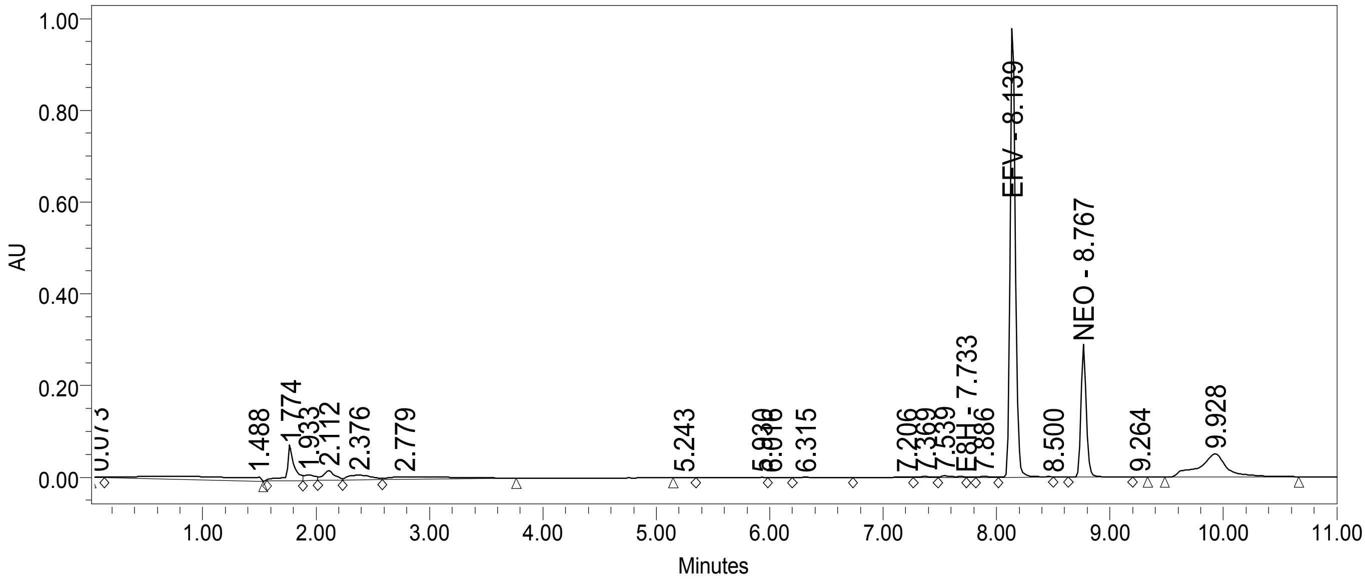

	Peak Name	RT	Area	% Area	Height
1		0.073	7882	0.12	1680
2		1.488	460616	7.12	8604
3		1.774	343160	5.30	65606
4		1.933	84181	1.30	11836
5		2.112	153571	2.37	20507
6		2.376	158393	2.45	10208
7		2.779	229589	3.55	5758
8		5.243	2409	0.04	412
9		5.930	7459	0.12	760
10		6.016	4051	0.06	415
11		6.315	9184	0.14	758
12		7.206	11981	0.19	1114

Reported by User: Saneesh (Saneesh) Project Name: HDI_Stellenbosch_2016

	Peak Name	RT	Area	% Area	Height
13		7.369	16330	0.25	2182
14		7.539	29534	0.46	3748
15	E8H	7.733	3618	0.06	987
16		7.886	14903	0.23	2116
17	EFV	8.139	3126390	48.29	907871
18		8.500	4136	0.06	699
19	NEO	8.767	929579	14.36	266919
20		9.264	2548	0.04	713
21		9.928	874143	13.50	50364

Reported by User: Saneesh (Saneesh) Project Name: HDI_Stellenbosch_2016

SAMPLE INFORMATION

Sample Name:	EFV TICL Inc 150miM 2	Acquired By:	Saneesh
Sample Type:	Unknown	Date Acquired:	2016/12/23 06:40:00 AM
Vial:	15	Acq. Method Set:	HDI_EFV_E8H_NEO
Injection #:	1	Date Processed:	2016/12/23 09:47:11 AM
Injection Volume:	15.00 ul	Processing Method:	HDI_EFV_ W2996 245.0nm-1.2
Run Time:	11.0 Minutes	Channel Name:	W2996 245.0nm-1.2
Sample Set Name:	EFV TICL Inhibtr_Asy2 22_12_16	Proc. Chnl. Descr.:	W2996 PDA 245.0 nm at 1.2

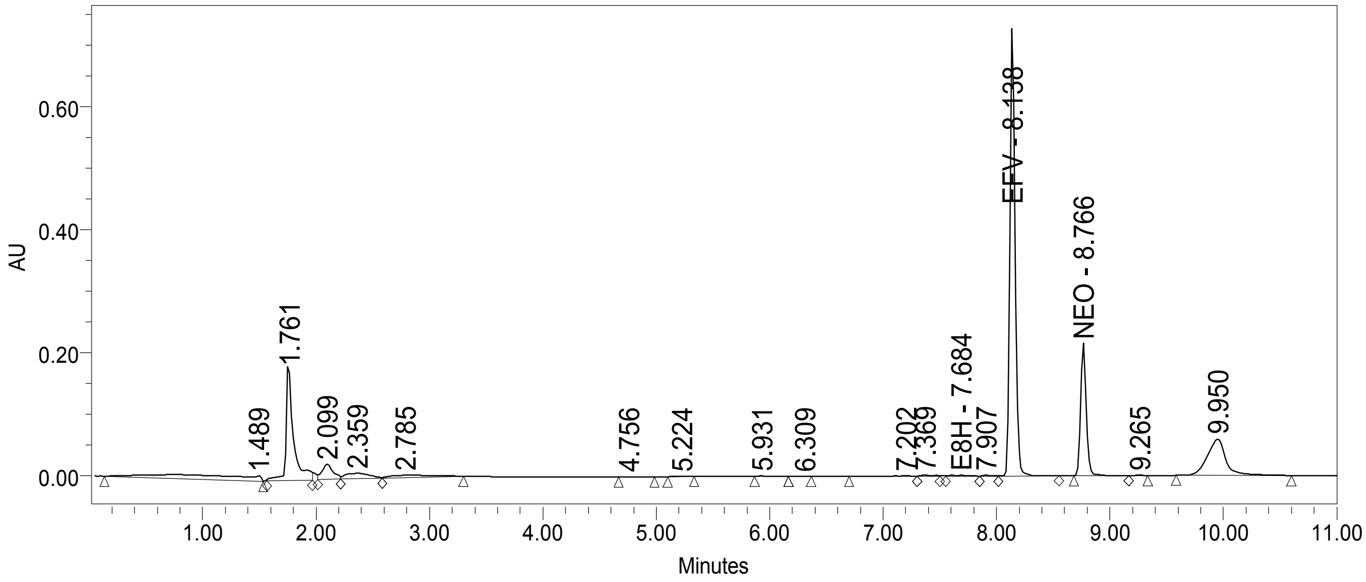

	Peak Name	RT	Area	% Area	Height
1		1.489	412422	7.63	8378
2		1.761	835906	15.46	164775
3		2.099	170979	3.16	24709
4		2.359	137247	2.54	8973
5		2.785	98238	1.82	3720
6		4.756	2640	0.05	279
7		5.224	2840	0.05	476
8		5.931	5524	0.10	829
9		6.309	1209	0.02	208
10		7.202	9187	0.17	691
11		7.369	11565	0.21	1694
12	E8H	7.684	12108	0.22	1518

Reported by User: Saneesh (Saneesh) Project Name: HDI_Stellenbosch_2016

	Peak Name	RT	Area	% Area	Height
13		7.907	7387	0.14	1487
14	EFV	8.138	2286492	42.28	667258
15	NEO	8.766	725744	13.42	202046
16		9.265	2467	0.05	611
17		9.950	686342	12.69	59258

SAMPLE INFORMATION

Sample Name:	EFV TICL Inc 200miM 1	Acquired By:	Saneesh
Sample Type:	Unknown	Date Acquired:	2016/12/23 06:54:18 AM
Vial:	16	Acq. Method Set:	HDI_EFV_E8H_NEO
Injection #:	1	Date Processed:	2016/12/23 09:47:11 AM
Injection Volume:	15.00 ul	Processing Method:	HDI_EFV_ W2996 245.0nm-1.2
Run Time:	11.0 Minutes	Channel Name:	W2996 245.0nm-1.2
Sample Set Name:	EFV TICL Inhibtr_Asy2 22_12_16	Proc. Chnl. Descr.:	W2996 PDA 245.0 nm at 1.2

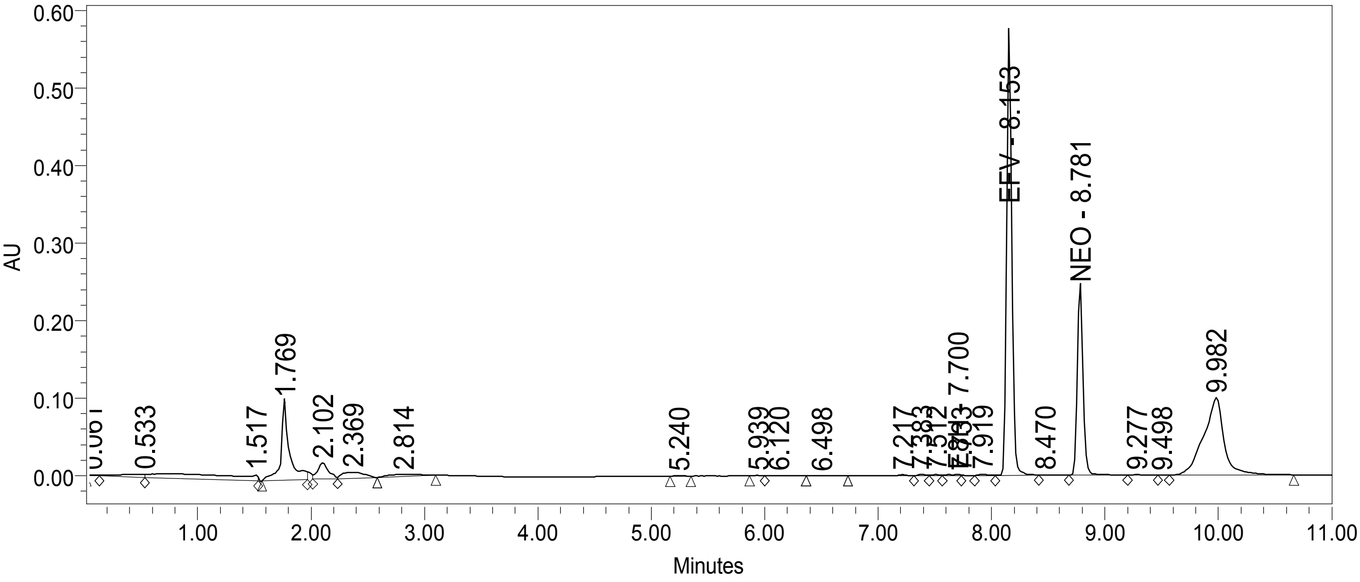

	Peak Name	RT	Area	% Area	Height
1		0.061	5114	0.10	817
2		0.533	59573	1.14	4062
3		1.517	338365	6.50	7993
4		1.769	520847	10.01	93077
5		2.102	138781	2.67	20389
6		2.369	102864	1.98	7541
7		2.814	45793	0.88	2319
8		5.240	2943	0.06	593
9		5.939	2506	0.05	704
10		6.120	5766	0.11	557
11		6.498	2088	0.04	224
12		7.217	8448	0.16	1192

	Peak Name	RT	Area	% Area	Height
13		7.383	6951	0.13	1608
14		7.512	5380	0.10	1123
15	E8H	7.700	10132	0.19	1659
16		7.733	1375	0.03	683
17		7.919	8800	0.17	1854
18	EFV	8.153	1816722	34.90	527154
19		8.470	8131	0.16	724
20	NEO	8.781	857726	16.48	233243
21		9.277	5923	0.11	766
22		9.498	770	0.01	155
23		9.982	1250647	24.02	99533

SAMPLE INFORMATION

Sample Name:	EFV TICL Inc 200miM 2	Acquired By:	Saneesh
Sample Type:	Unknown	Date Acquired:	2016/12/23 07:08:36 AM
Vial:	17	Acq. Method Set:	HDI_EFV_E8H_NEO
Injection #:	1	Date Processed:	2016/12/23 09:47:11 AM
Injection Volume:	15.00 ul	Processing Method:	HDI_EFV_ W2996 245.0nm-1.2
Run Time:	11.0 Minutes	Channel Name:	W2996 245.0nm-1.2
Sample Set Name:	EFV TICL Inhibtr_Asy2 22_12_16	Proc. Chnl. Descr.:	W2996 PDA 245.0 nm at 1.2

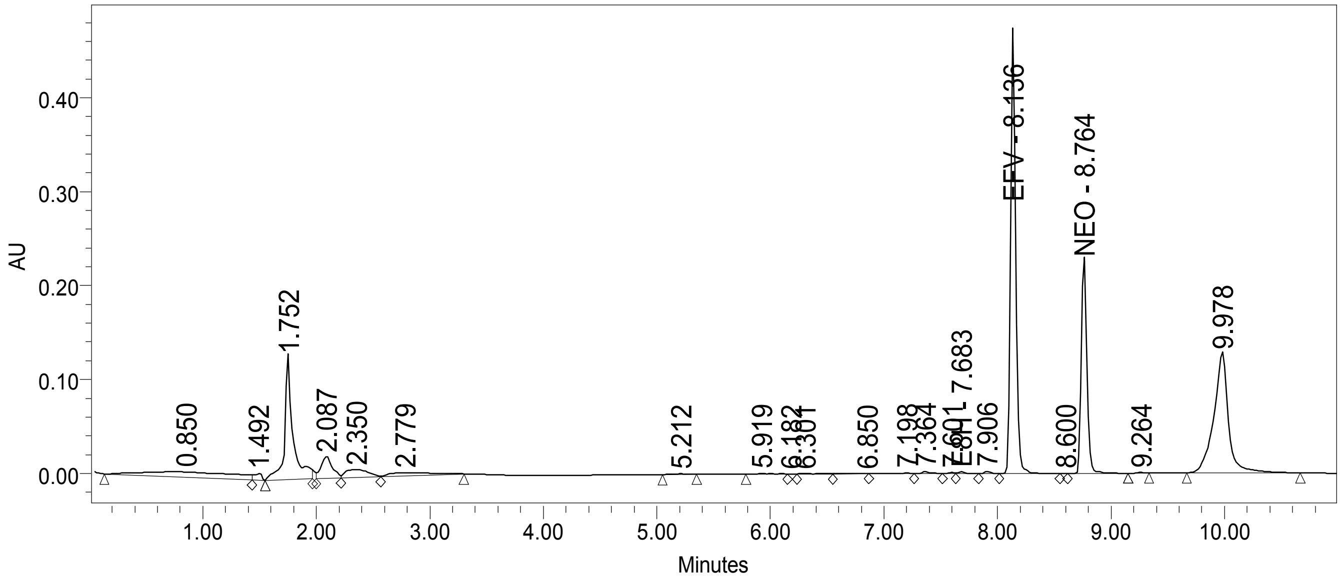

	Peak Name	RT	Area	% Area	Height
1		0.850	334411	6.95	5732
2		1.492	34683	0.72	7606
3		1.752	655383	13.62	120554
4		2.087	158326	3.29	23014
5		2.350	113805	2.37	8061
6		2.779	86264	1.79	3364
7		5.212	3784	0.08	652
8		5.919	4645	0.10	799
9		6.182	248	0.01	76
10		6.301	2322	0.05	278
11		6.850	2178	0.05	201
12		7.198	9517	0.20	1136

	Peak Name	RT	Area	% Area	Height
13		7.364	13929	0.29	2350
14		7.601	6007	0.12	1366
15	E8H	7.683	8966	0.19	1877
16		7.906	9391	0.20	2056
17	EFV	8.136	1448360	30.11	429690
18		8.600	298	0.01	126
19	NEO	8.764	784505	16.31	217751
20		9.264	2735	0.06	772
21		9.978	1130503	23.50	127947

Reported by User: Saneesh (Saneesh) Project Name: HDI_Stellenbosch_2016

SAMPLE INFORMATION

Sample Name:	EFV TICL TDI Inc 1miM 1	Acquired By:	Saneesh
Sample Type:	Unknown	Date Acquired:	2017/01/06 05:50:59 PM
Vial:	2	Acq. Method Set:	HDI_EFV_E8H_NEO
Injection #:	1	Date Processed:	2017/01/07 11:10:32 AM
Injection Volume:	15.00 ul	Processing Method:	HDI_EFV_ W2996 245.0nm-1.2
Run Time:	11.0 Minutes	Channel Name:	W2996 245.0nm-1.2
Sample Set Name:	EFV TICL Inhib IC50_TD1 6_1_17	Proc. Chnl. Descr.:	W2996 PDA 245.0 nm at 1.2

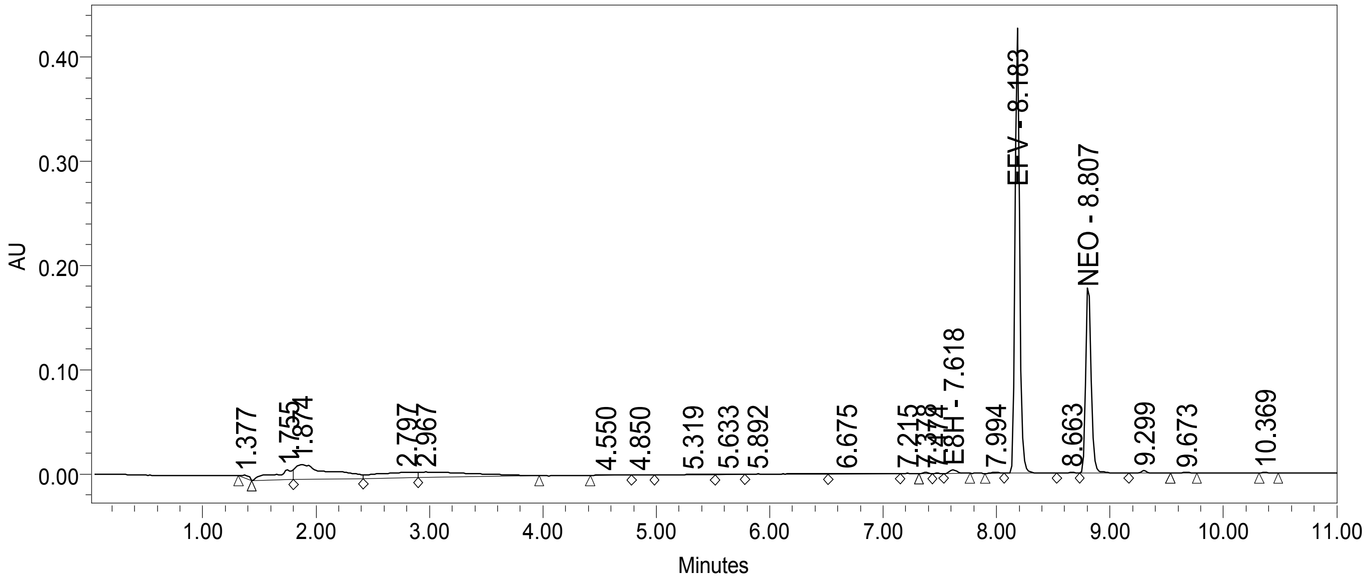

	Peak Name	RT	Area	% Area	Height
1		1.377	12399	0.46	2685
2		1.755	114958	4.30	9454
3		1.874	307659	11.51	14347
4		2.797	135317	5.06	5324
5		2.967	178497	6.68	5346
6		4.550	5197	0.19	386
7		4.850	2013	0.08	225
8		5.319	8905	0.33	418
9		5.633	6972	0.26	575
10		5.892	12992	0.49	450
11		6.675	8349	0.31	436
12		7.215	1747	0.07	385
13		7.378	3248	0.12	963
14		7.474	777	0.03	218

Reported by User: Saneesh (Saneesh) Project Name: HDI_Stellenbosch_2016

	Peak Name	RT	Area	% Area	Height
15	E8H	7.618	14656	0.55	3198
16		7.994	4170	0.16	771
17	EFV	8.183	1239459	46.38	385978
18		8.663	3730	0.14	792
19	NEO	8.807	597776	22.37	168797
20		9.299	8856	0.33	2232
21		9.673	2945	0.11	558
22		10.369	1533	0.06	374

Multi Sample Summary

Reported by User: Saneesh (Saneesh)   Project Name: HDI_Stellenbosch_2016

SAMPLE INFORMATION

Sample Name:	EFV TICLTDI Inc 1miM 2	Acquired By:	Saneesh
Sample Type:	Unknown	Date Acquired:	2017/01/06 06:05:28 PM
Vial:	3	Acq. Method Set:	HDI_EFV_E8H_NEO
Injection #:	1	Date Processed:	2017/01/07 11:10:32 AM
Injection Volume:	15.00 ul	Processing Method:	HDI_EFV_ W2996 245.0nm-1.2
Run Time:	11.0 Minutes	Channel Name:	W2996 245.0nm-1.2
Sample Set Name:	EFV TICL Inhib IC50_TD1 6_1_17	Proc. Chnl. Descr.:	W2996 PDA 245.0 nm at 1.2

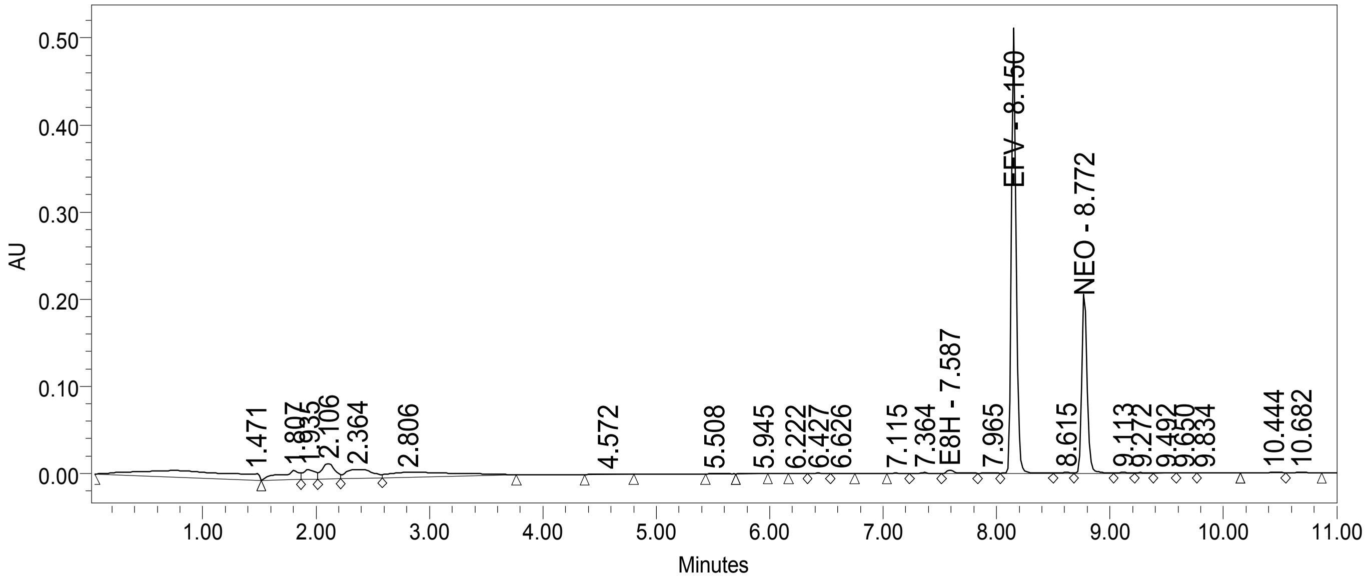

	Peak Name	RT	Area	% Area	Height
1		1.471	479385	13.92	7824
2		1.807	124495	3.62	10254
3		1.935	82275	2.39	10988
4		2.106	140924	4.09	17529
5		2.364	164412	4.77	10057
6		2.806	224424	6.52	5409
7		4.572	7959	0.23	474
8		5.508	2307	0.07	399
9		5.945	1301	0.04	196
10		6.222	895	0.03	175
11		6.427	5089	0.15	869
12		6.626	2600	0.08	360

	Peak Name	RT	Area	% Area	Height
13		7.115	2748	0.08	510
14		7.364	5577	0.16	1172
15	E8H	7.587	23054	0.67	3408
16		7.965	5285	0.15	641
17	EFV	8.150	1460803	42.42	456900
18		8.615	7679	0.22	1205
19	NEO	8.772	671590	19.50	192335
20		9.113	5972	0.17	1132
21		9.272	3969	0.12	822
22		9.492	2824	0.08	344
23		9.650	2886	0.08	510
24		9.834	2433	0.07	282
25		10.444	7290	0.21	720
26		10.682	5332	0.15	549

SAMPLE INFORMATION

Sample Name:	EFV TICL TDI Inc 10miM 1	Acquired By:	Saneesh
Sample Type:	Unknown	Date Acquired:	2017/01/06 06:19:55 PM
Vial:	4	Acq. Method Set:	HDI_EFV_E8H_NEO
Injection #:	1	Date Processed:	2017/01/07 11:10:32 AM
Injection Volume:	15.00 ul	Processing Method:	HDI_EFV_ W2996 245.0nm-1.2
Run Time:	11.0 Minutes	Channel Name:	W2996 245.0nm-1.2
Sample Set Name:	EFV TICL Inhib IC50_TD1 6_1_17	Proc. Chnl. Descr.:	W2996 PDA 245.0 nm at 1.2

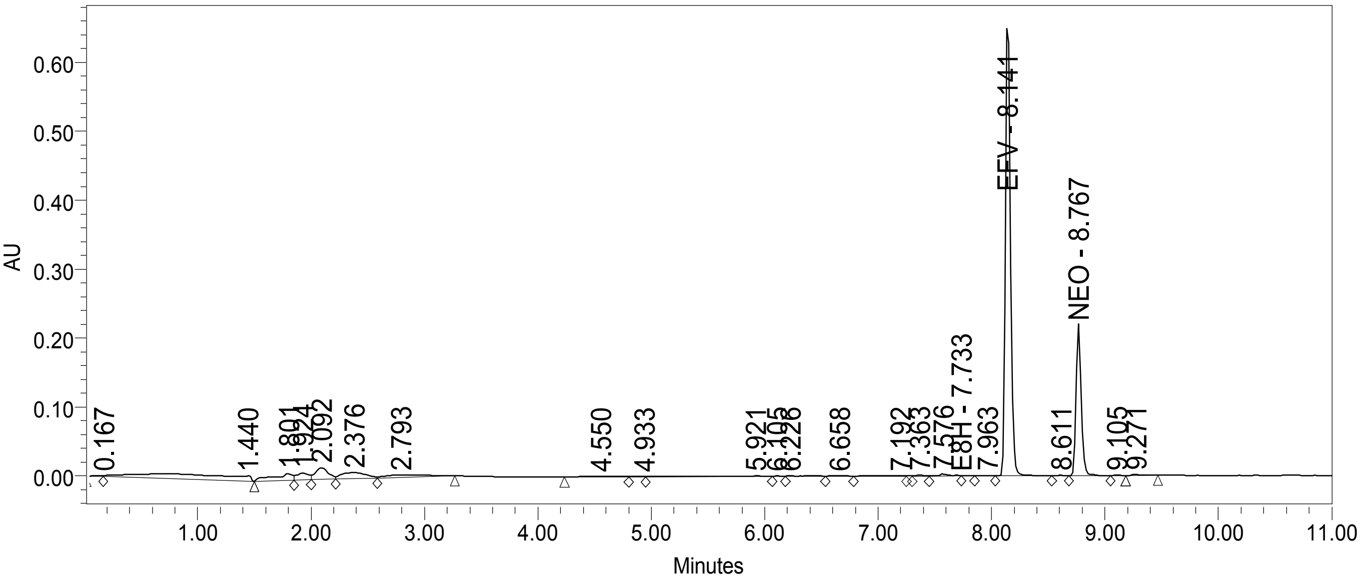

	Peak Name	RT	Area	% Area	Height
1		0.167	7215	0.19	1354
2		1.440	438583	11.43	7829
3		1.801	119535	3.11	9690
4		1.924	72735	1.89	9842
5		2.092	134458	3.50	16970
6		2.376	131980	3.44	8546
7		2.793	82147	2.14	3214
8		4.550	17528	0.46	807
9		4.933	3029	0.08	397
10		5.921	35312	0.92	739
11		6.105	3779	0.10	627
12		6.226	11423	0.30	634

Reported by User: Saneesh (Saneesh) Project Name: HDI_Stellenbosch_2016

	Peak Name	RT	Area	% Area	Height
13		6.658	6896	0.18	578
14		7.192	10180	0.27	461
15		7.363	6536	0.17	1500
16		7.576	18594	0.48	2934
17	E8H	7.733	2843	0.07	518
18		7.963	4242	0.11	540
19	EFV	8.141	2033163	52.97	604337
20		8.611	3639	0.09	745
21	NEO	8.767	684410	17.83	202471
22		9.105	2678	0.07	758
23		9.271	7583	0.20	1343

Reported by User: Saneesh (Saneesh) Project Name: HDI_Stellenbosch_2016

SAMPLE INFORMATION

Sample Name:	EFV TICL TDI Inc 10miM 2	Acquired By:	Saneesh
Sample Type:	Unknown	Date Acquired:	2017/01/06 06:34:24 PM
Vial:	5	Acq. Method Set:	HDI_EFV_E8H_NEO
Injection #:	1	Date Processed:	2017/01/07 11:10:32 AM
Injection Volume:	15.00 ul	Processing Method:	HDI_EFV_ W2996 245.0nm-1.2
Run Time:	11.0 Minutes	Channel Name:	W2996 245.0nm-1.2
Sample Set Name:	EFV TICL Inhib IC50_TD1 6_1_17	Proc. Chnl. Descr.:	W2996 PDA 245.0 nm at 1.2

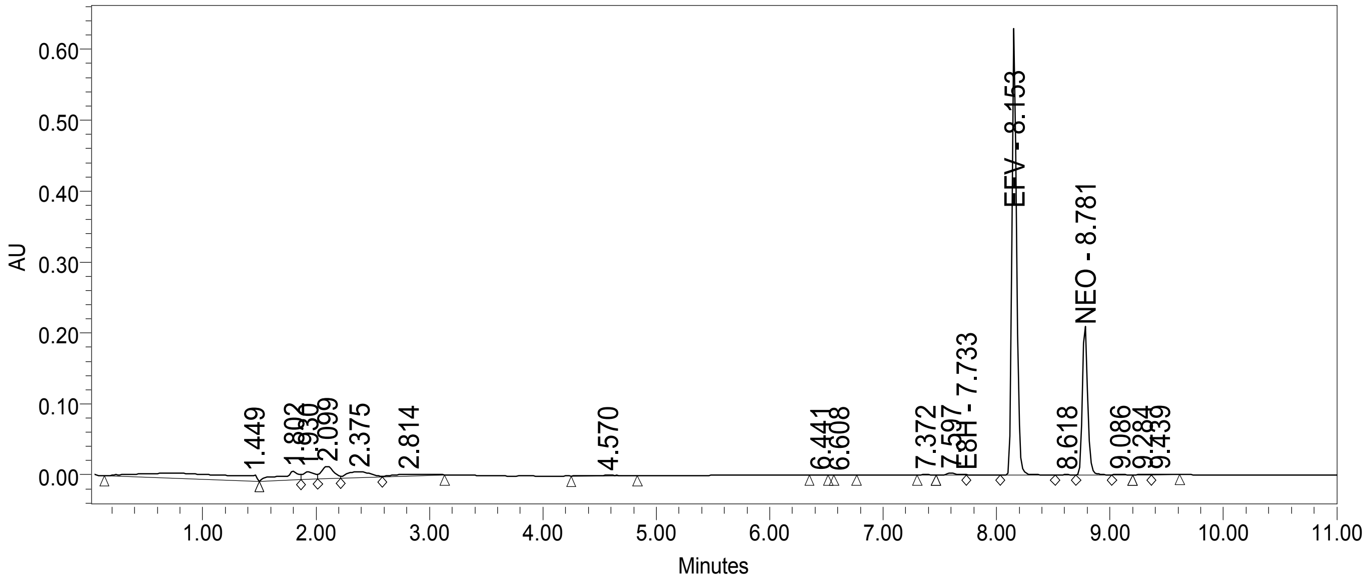

	Peak Name	RT	Area	% Area	Height
1		1.449	456083	12.93	7837
2		1.802	139922	3.97	11946
3		1.930	77298	2.19	10494
4		2.099	133432	3.78	17654
5		2.375	125049	3.54	8322
6		2.814	51727	1.47	2396
7		4.570	16008	0.45	788
8		6.441	2882	0.08	587
9		6.608	1584	0.04	285
10		7.372	5011	0.14	1399
11		7.597	17861	0.51	2902
12	E8H	7.733	5955	0.17	554

Reported by User: Saneesh (Saneesh) Project Name: HDI_Stellenbosch_2016

	Peak Name	RT	Area	% Area	Height
13	EFV	8.153	1792762	50.82	557455
14		8.618	3845	0.11	742
15	NEO	8.781	680037	19.28	197704
16		9.086	6242	0.18	1227
17		9.284	6176	0.18	1155
18		9.439	5794	0.16	697

SAMPLE INFORMATION

Sample Name:	EFV TICL TDI Inc 25miM 1	Acquired By:	Saneesh
Sample Type:	Unknown	Date Acquired:	2017/01/06 06:48:42 PM
Vial:	6	Acq. Method Set:	HDI_EFV_E8H_NEO
Injection #:	1	Date Processed:	2017/01/07 11:10:32 AM
Injection Volume:	15.00 ul	Processing Method:	HDI_EFV_ W2996 245.0nm-1.2
Run Time:	11.0 Minutes	Channel Name:	W2996 245.0nm-1.2
Sample Set Name:	EFV TICL Inhib IC50_TD1 6_1_17	Proc. Chnl. Descr.:	W2996 PDA 245.0 nm at 1.2

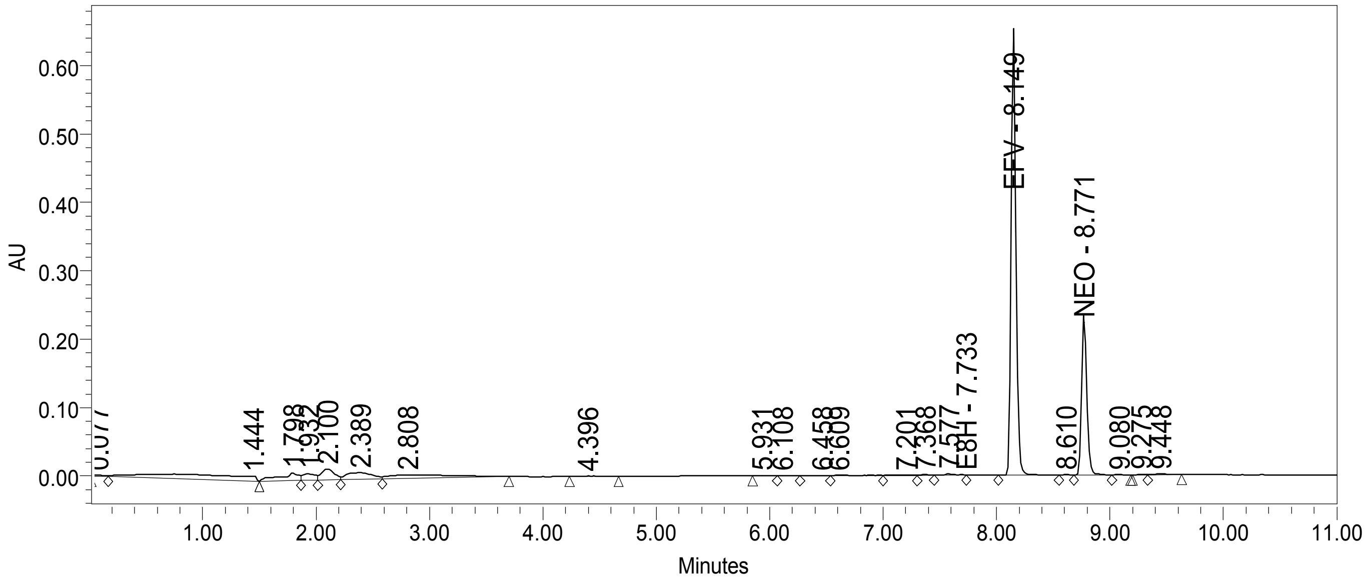

	Peak Name	RT	Area	% Area	Height
1		0.077	11016	0.29	1577
2		1.444	427643	11.10	7103
3		1.798	134243	3.48	10844
4		1.932	73495	1.91	9543
5		2.100	129020	3.35	16239
6		2.389	155480	4.04	9607
7		2.808	194458	5.05	4993
8		4.396	10486	0.27	614
9		5.931	1135	0.03	210
10		6.108	1207	0.03	202
11		6.458	1547	0.04	165
12		6.609	3506	0.09	255

Reported by User: Saneesh (Saneesh) Project Name: HDI_Stellenbosch_2016

	Peak Name	RT	Area	% Area	Height
13		7.201	3578	0.09	431
14		7.368	5311	0.14	1370
15		7.577	16456	0.43	2481
16	E8H	7.733	6975	0.18	536
17	EFV	8.149	1910243	49.58	592633
18		8.610	4076	0.11	805
19	NEO	8.771	741310	19.24	215440
20		9.080	4077	0.11	872
21		9.275	5545	0.14	1312
22		9.448	12247	0.32	1213

SAMPLE INFORMATION

Sample Name:	EFV TICL TDI Inc 25miM 2	Acquired By:	Saneesh
Sample Type:	Unknown	Date Acquired:	2017/01/06 07:03:00 PM
Vial:	7	Acq. Method Set:	HDI_EFV_E8H_NEO
Injection #:	1	Date Processed:	2017/01/07 11:10:32 AM
Injection Volume:	15.00 ul	Processing Method:	HDI_EFV_ W2996 245.0nm-1.2
Run Time:	11.0 Minutes	Channel Name:	W2996 245.0nm-1.2
Sample Set Name:	EFV TICL Inhib IC50_TD1 6_1_17	Proc. Chnl. Descr.:	W2996 PDA 245.0 nm at 1.2

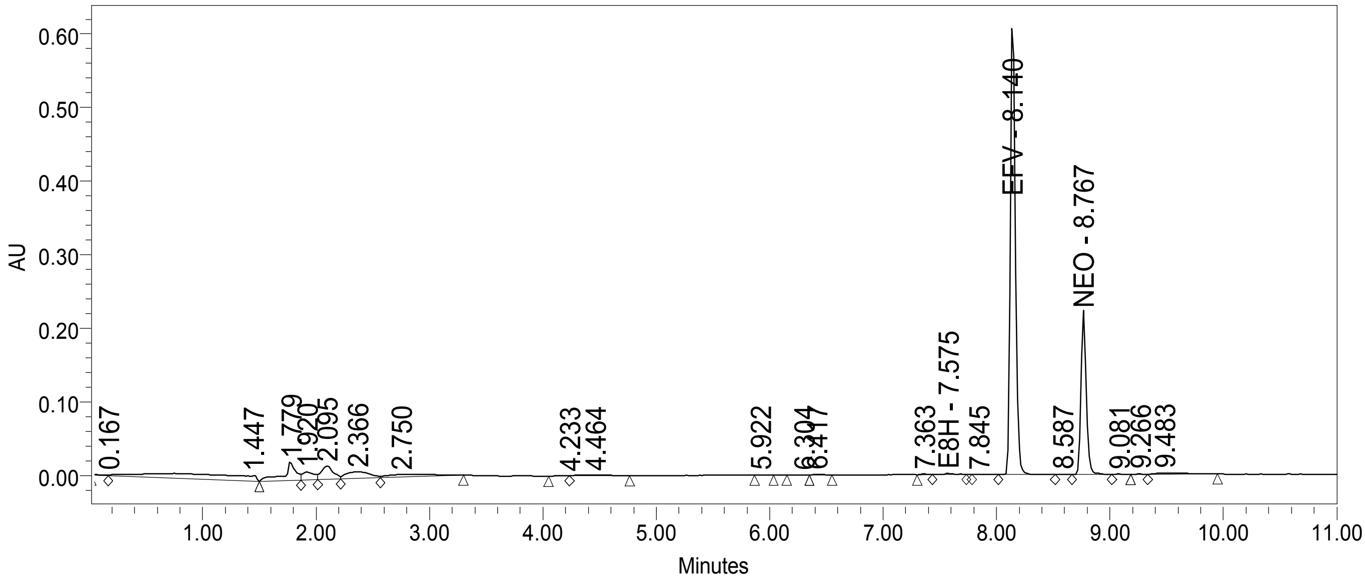

	Peak Name	RT	Area	% Area	Height
1		0.167	6344	0.17	1005
2		1.447	414737	11.11	7304
3		1.779	178060	4.77	22192
4		1.920	77056	2.06	10354
5		2.095	131492	3.52	18000
6		2.366	129179	3.46	8596
7		2.750	92106	2.47	3410
8		4.233	2199	0.06	387
9		4.464	22979	0.62	1071
10		5.922	1112	0.03	226
11		6.304	883	0.02	156
12		6.417	3958	0.11	818

Reported by User: Saneesh (Saneesh)   Project Name: HDI_Stellenbosch_2016

	Peak Name	RT	Area	% Area	Height
13		7.363	4444	0.12	1216
14	E8H	7.575	15306	0.41	2190
15		7.845	4738	0.13	374
16	EFV	8.140	1900986	50.91	562717
17		8.587	3997	0.11	701
18	NEO	8.767	702521	18.81	205124
19		9.081	4063	0.11	871
20		9.266	3202	0.09	881
21		9.483	34741	0.93	1910

Reported by User: Saneesh (Saneesh) Project Name: HDI_Stellenbosch_2016

SAMPLE INFORMATION

Sample Name:	EFV TICL TDI Inc 50miM 1	Acquired By:	Saneesh
Sample Type:	Unknown	Date Acquired:	2017/01/06 07:17:28 PM
Vial:	8	Acq. Method Set:	HDI_EFV_E8H_NEO
Injection #:	1	Date Processed:	2017/01/07 11:10:32 AM
Injection Volume:	15.00 ul	Processing Method:	HDI_EFV_ W2996 245.0nm-1.2
Run Time:	11.0 Minutes	Channel Name:	W2996 245.0nm-1.2
Sample Set Name:	EFV TICL Inhib IC50_TD1 6_1_17	Proc. Chnl. Descr.:	W2996 PDA 245.0 nm at 1.2

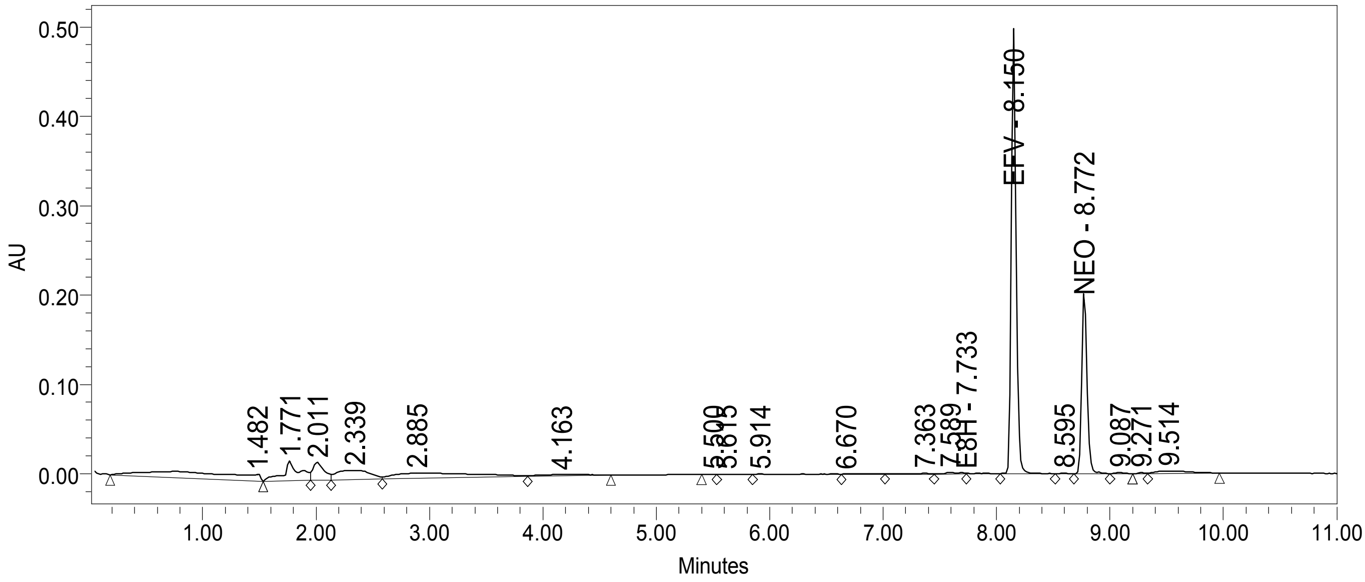

	Peak Name	RT	Area	% Area	Height
1		1.482	430451	11.90	7140
2		1.771	216289	5.98	21483
3		2.011	141889	3.92	20028
4		2.339	217369	6.01	10380
5		2.885	310290	8.58	6017
6		4.163	52039	1.44	1734
7		5.500	2118	0.06	373
8		5.615	9604	0.27	579
9		5.914	18365	0.51	621
10		6.670	8153	0.23	438
11		7.363	13316	0.37	1222
12		7.589	18249	0.50	2012

Reported by User: Saneesh (Saneesh) Project Name: HDI_Stellenbosch_2016

	Peak Name	RT	Area	% Area	Height
13	E8H	7.733	10751	0.30	801
14	EFV	8.150	1448369	40.04	448291
15		8.595	3718	0.10	638
16	NEO	8.772	654579	18.10	188164
17		9.087	6359	0.18	1232
18		9.271	3548	0.10	1023
19		9.514	51442	1.42	2518

SAMPLE INFORMATION

Sample Name:	EFV TICL TDI Inc 50miM 2	Acquired By:	Saneesh
Sample Type:	Unknown	Date Acquired:	2017/01/06 07:31:46 PM
Vial:	9	Acq. Method Set:	HDI_EFV_E8H_NEO
Injection #:	1	Date Processed:	2017/01/07 11:10:32 AM
Injection Volume:	15.00 ul	Processing Method:	HDI_EFV_ W2996 245.0nm-1.2
Run Time:	11.0 Minutes	Channel Name:	W2996 245.0nm-1.2
Sample Set Name:	EFV TICL Inhib IC50_TD1 6_1_17	Proc. Chnl. Descr.:	W2996 PDA 245.0 nm at 1.2

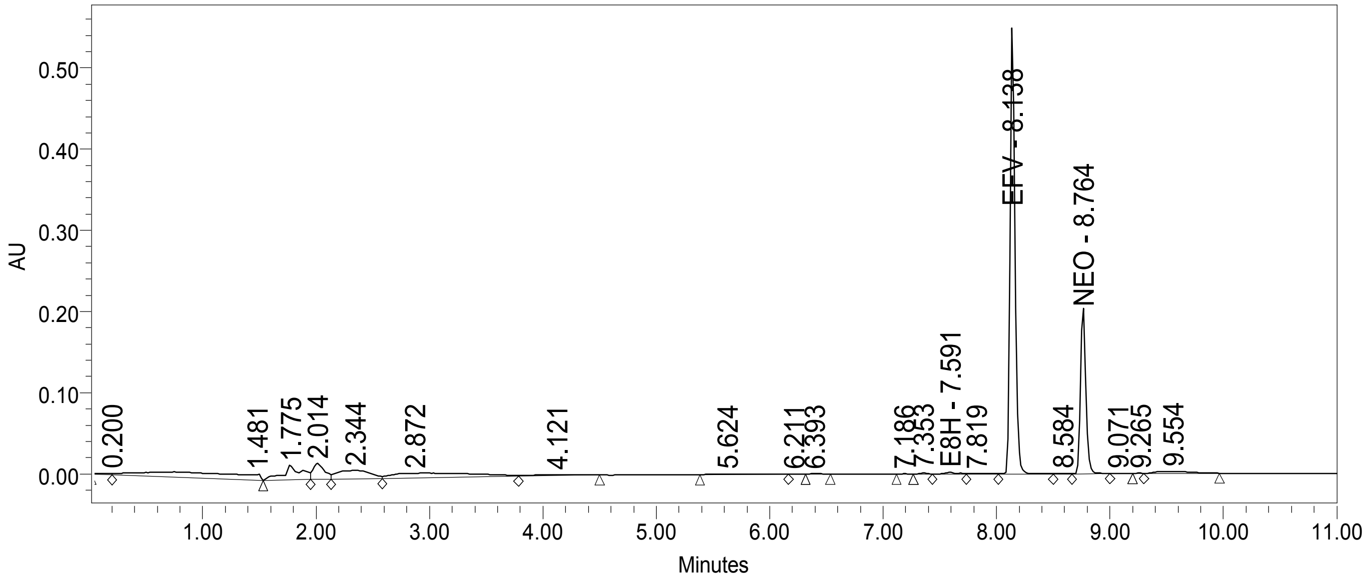

	Peak Name	RT	Area	% Area	Height
1		0.200	13567	0.36	2031
2		1.481	431543	11.47	7070
3		1.775	200321	5.32	17297
4		2.014	139723	3.71	19658
5		2.344	213176	5.67	10260
6		2.872	295357	7.85	5943
7		4.121	46506	1.24	1508
8		5.624	14384	0.38	553
9		6.211	969	0.03	160
10		6.393	3123	0.08	543
11		7.186	863	0.02	236
12		7.353	5559	0.15	1596

Reported by User: Saneesh (Saneesh) Project Name: HDI_Stellenbosch_2016

	Peak Name	RT	Area	% Area	Height
13	E8H	7.591	16302	0.43	2225
14		7.819	5722	0.15	413
15	EFV	8.138	1638110	43.54	495812
16		8.584	3855	0.10	684
17	NEO	8.764	664394	17.66	191685
18		9.071	4095	0.11	840
19		9.265	3453	0.09	996
20		9.554	61406	1.63	2643

Reported by User: Saneesh (Saneesh) Project Name: HDI_Stellenbosch_2016

SAMPLE INFORMATION

Sample Name:	EFV TICL TDI Inc 75miM 1	Acquired By:	Saneesh
Sample Type:	Unknown	Date Acquired:	2017/01/06 07:46:03 PM
Vial:	10	Acq. Method Set:	HDI_EFV_E8H_NEO
Injection #:	1	Date Processed:	2017/01/07 11:10:32 AM
Injection Volume:	15.00 ul	Processing Method:	HDI_EFV_ W2996 245.0nm-1.2
Run Time:	11.0 Minutes	Channel Name:	W2996 245.0nm-1.2
Sample Set Name:	EFV TICL Inhib IC50_TD1 6_1_17	Proc. Chnl. Descr.:	W2996 PDA 245.0 nm at 1.2

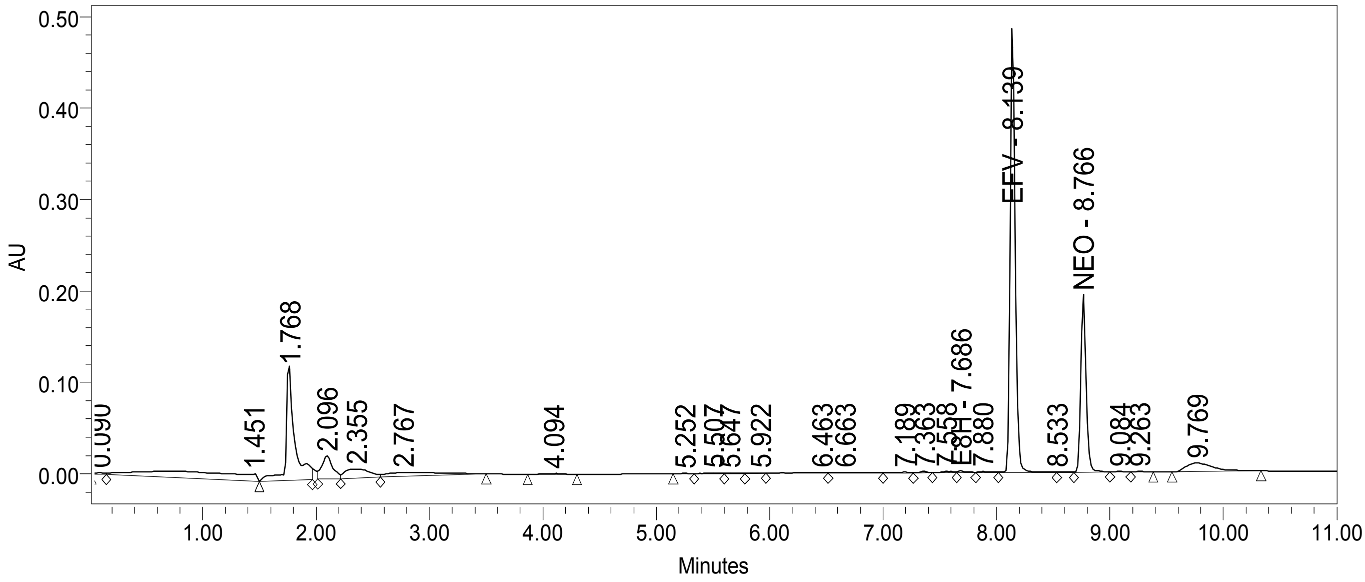

	Peak Name	RT	Area	% Area	Height
1		0.090	11865	0.30	1776
2		1.451	468879	11.73	8022
3		1.768	647625	16.20	121491
4		2.096	171569	4.29	25016
5		2.355	152339	3.81	9904
6		2.767	152567	3.82	4573
7		4.094	12500	0.31	976
8		5.252	2162	0.05	396
9		5.507	3756	0.09	369
10		5.647	3405	0.09	365
11		5.922	3708	0.09	515
12		6.463	17384	0.43	652

Reported by User: Saneesh (Saneesh)   Project Name: HDI_Stellenbosch_2016

	Peak Name	RT	Area	% Area	Height
13		6.663	16766	0.42	745
14		7.189	8799	0.22	857
15		7.363	7988	0.20	1490
16		7.558	11533	0.29	1547
17	E8H	7.686	9872	0.25	1903
18		7.880	9408	0.24	1147
19	EFV	8.139	1494010	37.37	442717
20		8.533	3244	0.08	525
21	NEO	8.766	620223	15.51	180044
22		9.084	4563	0.11	887
23		9.263	3687	0.09	936
24		9.769	159840	4.00	9414

SAMPLE INFORMATION

Sample Name:	EFV TICL TDI Inc 75miM 2	Acquired By:	Saneesh
Sample Type:	Unknown	Date Acquired:	2017/01/06 08:00:21 PM
Vial:	11	Acq. Method Set:	HDI_EFV_E8H_NEO
Injection #:	1	Date Processed:	2017/01/07 11:10:32 AM
Injection Volume:	15.00 ul	Processing Method:	HDI_EFV_ W2996 245.0nm-1.2
Run Time:	11.0 Minutes	Channel Name:	W2996 245.0nm-1.2
Sample Set Name:	EFV TICL Inhib IC50_TD1 6_1_17	Proc. Chnl. Descr.:	W2996 PDA 245.0 nm at 1.2

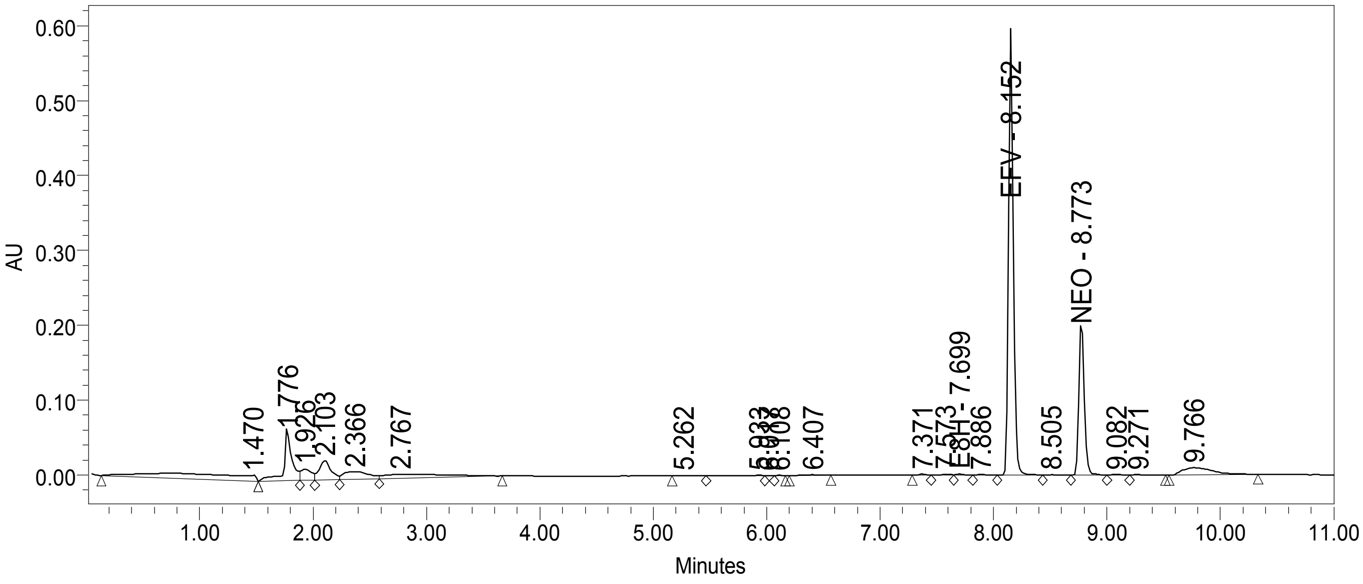

	Peak Name	RT	Area	% Area	Height
1		1.470	424050	10.54	7771
2		1.776	326330	8.11	58712
3		1.926	97785	2.43	14805
4		2.103	184713	4.59	25333
5		2.366	156491	3.89	9921
6		2.767	192036	4.78	5120
7		5.262	2709	0.07	356
8		5.933	5100	0.13	410
9		6.017	667	0.02	191
10		6.108	1030	0.03	281
11		6.407	5367	0.13	727
12		7.371	5927	0.15	1674

	Peak Name	RT	Area	% Area	Height
13		7.573	7317	0.18	1302
14	E8H	7.699	7839	0.19	1794
15		7.886	6814	0.17	1016
16	EFV	8.152	1729362	43.00	532197
17		8.505	4659	0.12	566
18	NEO	8.773	672453	16.72	189512
19		9.082	5126	0.13	966
20		9.271	4362	0.11	995
21		9.766	181340	4.51	9687

Reported by User: Saneesh (Saneesh) Project Name: HDI_Stellenbosch_2016

SAMPLE INFORMATION

Sample Name:	EFV TICL TDI Inc 100miM 1	Acquired By:	Saneesh
Sample Type:	Unknown	Date Acquired:	2017/01/06 08:14:59 PM
Vial:	12	Acq. Method Set:	HDI_EFV_E8H_NEO
Injection #:	1	Date Processed:	2017/01/07 11:10:32 AM
Injection Volume:	15.00 ul	Processing Method:	HDI_EFV_ W2996 245.0nm-1.2
Run Time:	11.0 Minutes	Channel Name:	W2996 245.0nm-1.2
Sample Set Name:	EFV TICL Inhib IC50_TD1 6_1_17	Proc. Chnl. Descr.:	W2996 PDA 245.0 nm at 1.2

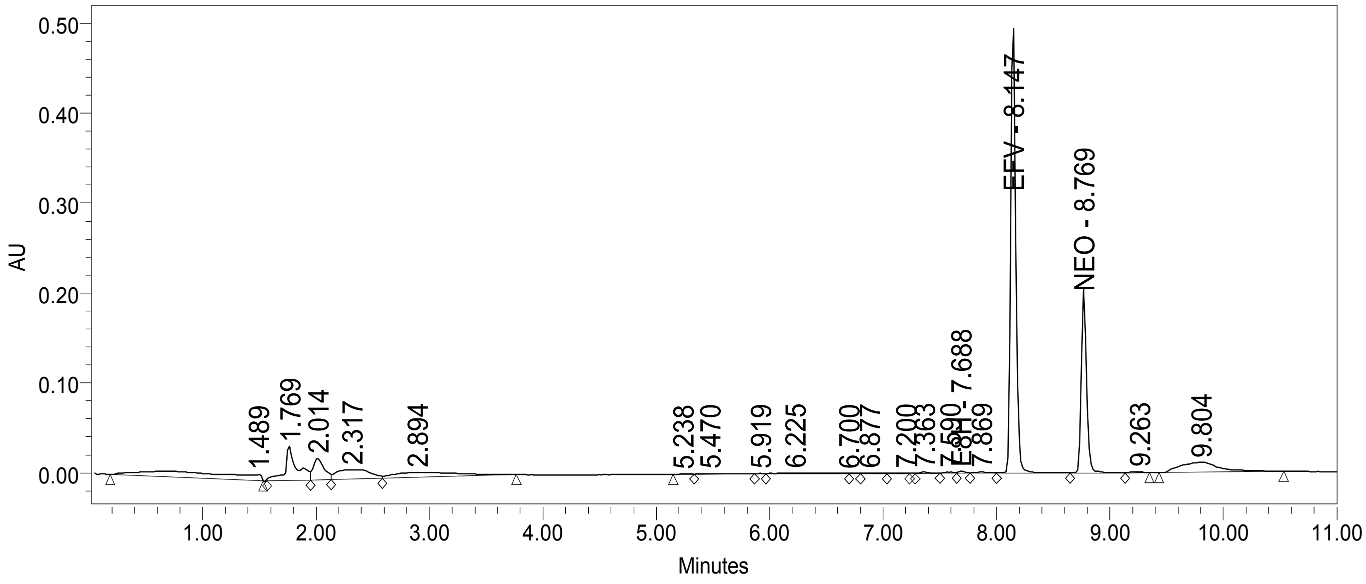

	Peak Name	RT	Area	% Area	Height
1		1.489	419180	10.84	7657
2		1.769	277764	7.18	37423
3		2.014	161200	4.17	23978
4		2.317	212043	5.48	10210
5		2.894	217825	5.63	5339
6		5.238	2354	0.06	314
7		5.470	10515	0.27	445
8		5.919	2880	0.07	619
9		6.225	30507	0.79	863
10		6.700	2705	0.07	509
11		6.877	6220	0.16	530
12		7.200	6164	0.16	630

	Peak Name	RT	Area	% Area	Height
13		7.363	10989	0.28	1732
14		7.590	9480	0.25	1411
15	E8H	7.688	9296	0.24	2266
16		7.869	12724	0.33	1491
17	EFV	8.147	1555742	40.23	464396
18	NEO	8.769	645517	16.69	187217
19		9.263	7800	0.20	1226
20		9.804	266518	6.89	11079

SAMPLE INFORMATION

Sample Name:	EFV TICL TDI Inc 100miM 2	Acquired By:	Saneesh
Sample Type:	Unknown	Date Acquired:	2017/01/06 08:29:17 PM
Vial:	13	Acq. Method Set:	HDI_EFV_E8H_NEO
Injection #:	1	Date Processed:	2017/01/07 11:10:33 AM
Injection Volume:	15.00 ul	Processing Method:	HDI_EFV_ W2996 245.0nm-1.2
Run Time:	11.0 Minutes	Channel Name:	W2996 245.0nm-1.2
Sample Set Name:	EFV TICL Inhib IC50_TD1 6_1_17	Proc. Chnl. Descr.:	W2996 PDA 245.0 nm at 1.2

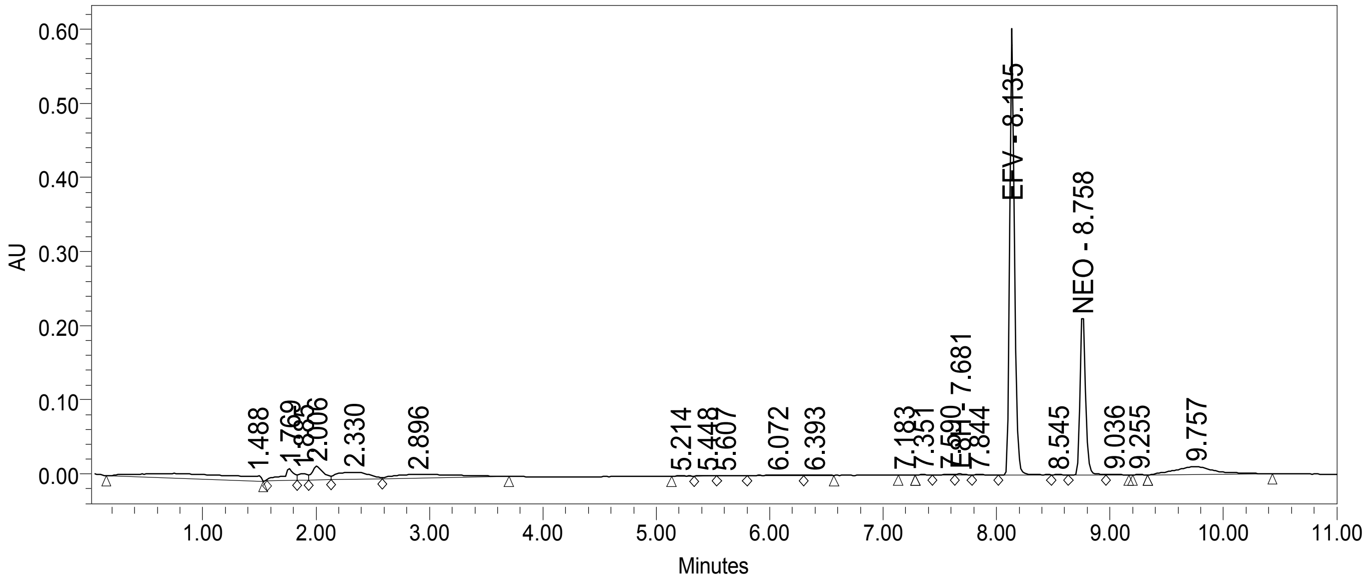

	Peak Name	RT	Area	% Area	Height
1		1.488	409279	10.56	7505
2		1.769	119139	3.07	15988
3		1.885	50221	1.30	9266
4		2.006	134158	3.46	18742
5		2.330	191827	4.95	9529
6		2.896	190511	4.91	4735
7		5.214	1642	0.04	245
8		5.448	1761	0.05	232
9		5.607	1549	0.04	183
10		6.072	12614	0.33	733
11		6.393	4148	0.11	519
12		7.183	1199	0.03	374

Reported by User: Saneesh (Saneesh) Project Name: HDI_Stellenbosch_2016

	Peak Name	RT	Area	% Area	Height
13		7.351	5631	0.15	1559
14		7.590	9948	0.26	1630
15	E8H	7.681	10284	0.27	2331
16		7.844	8239	0.21	1082
17	EFV	8.135	1727581	44.56	535207
18		8.545	3294	0.08	534
19	NEO	8.758	720996	18.60	202404
20		9.036	6735	0.17	1135
21		9.255	3489	0.09	1059
22		9.757	263062	6.78	10582

SAMPLE INFORMATION

Sample Name:	EFV TICL TDI Inc 150miM 1	Acquired By:	Saneesh
Sample Type:	Unknown	Date Acquired:	2017/01/06 08:44:08 PM
Vial:	14	Acq. Method Set:	HDI_EFV_E8H_NEO
Injection #:	1	Date Processed:	2017/01/07 11:10:33 AM
Injection Volume:	15.00 ul	Processing Method:	HDI_EFV_ W2996 245.0nm-1.2
Run Time:	11.0 Minutes	Channel Name:	W2996 245.0nm-1.2
Sample Set Name:	EFV TICL Inhib IC50_TD1 6_1_17	Proc. Chnl. Descr.:	W2996 PDA 245.0 nm at 1.2

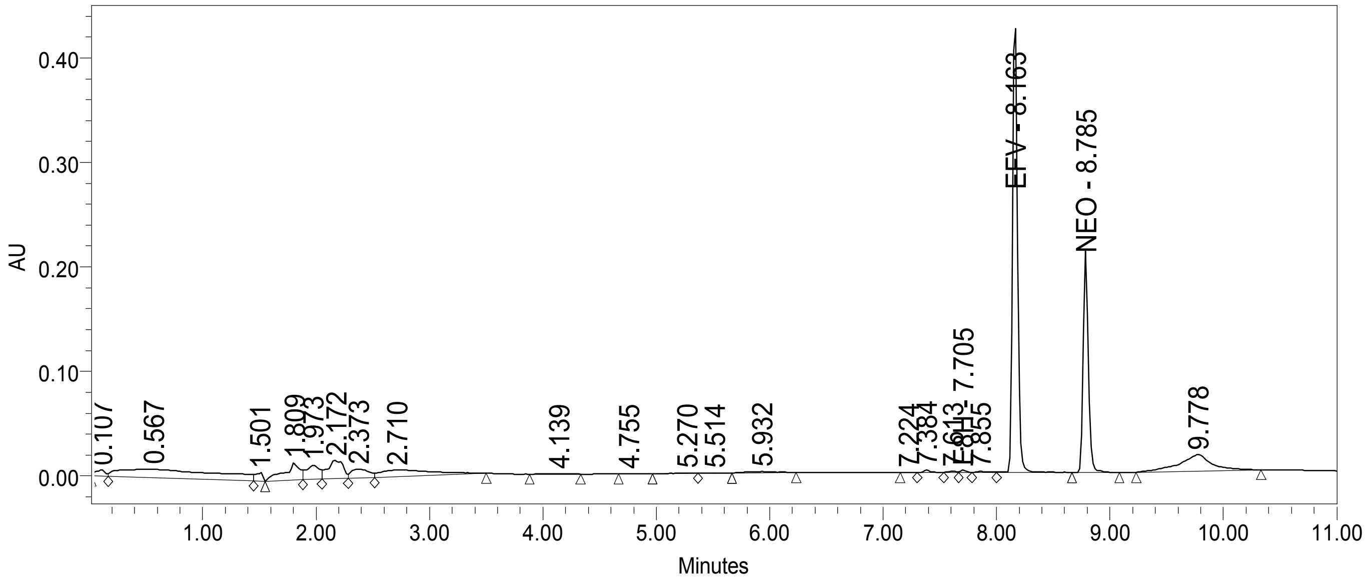

	Peak Name	RT	Area	% Area	Height
1		0.107	34372	0.94	5966
2		0.567	512269	13.97	8144
3		1.501	35344	0.96	7756
4		1.809	159330	4.35	15680
5		1.973	110190	3.01	13557
6		2.172	165987	4.53	17647
7		2.373	92339	2.52	8332
8		2.710	196297	5.35	5983
9		4.139	9056	0.25	638
10		4.755	1814	0.05	184
11		5.270	2997	0.08	284
12		5.514	2157	0.06	250

Reported by User: Saneesh (Saneesh) Project Name: HDI_Stellenbosch_2016

	Peak Name	RT	Area	% Area	Height
13		5.932	19826	0.54	1156
14		7.224	1066	0.03	230
15		7.384	9750	0.27	2381
16		7.613	5979	0.16	1197
17	E8H	7.705	7170	0.20	2035
18		7.855	6311	0.17	937
19	EFV	8.163	1329030	36.25	402889
20	NEO	8.785	653000	17.81	193761
21		9.778	311815	8.51	15681

SAMPLE INFORMATION

Sample Name:	EFV TICL TDI Inc 150miM 2	Acquired By:	Saneesh
Sample Type:	Unknown	Date Acquired:	2017/01/06 08:58:25 PM
Vial:	15	Acq. Method Set:	HDI_EFV_E8H_NEO
Injection #:	1	Date Processed:	2017/01/07 11:10:33 AM
Injection Volume:	15.00 ul	Processing Method:	HDI_EFV_ W2996 245.0nm-1.2
Run Time:	11.0 Minutes	Channel Name:	W2996 245.0nm-1.2
Sample Set Name:	EFV TICL Inhib IC50_TD1 6_1_17	Proc. Chnl. Descr.:	W2996 PDA 245.0 nm at 1.2

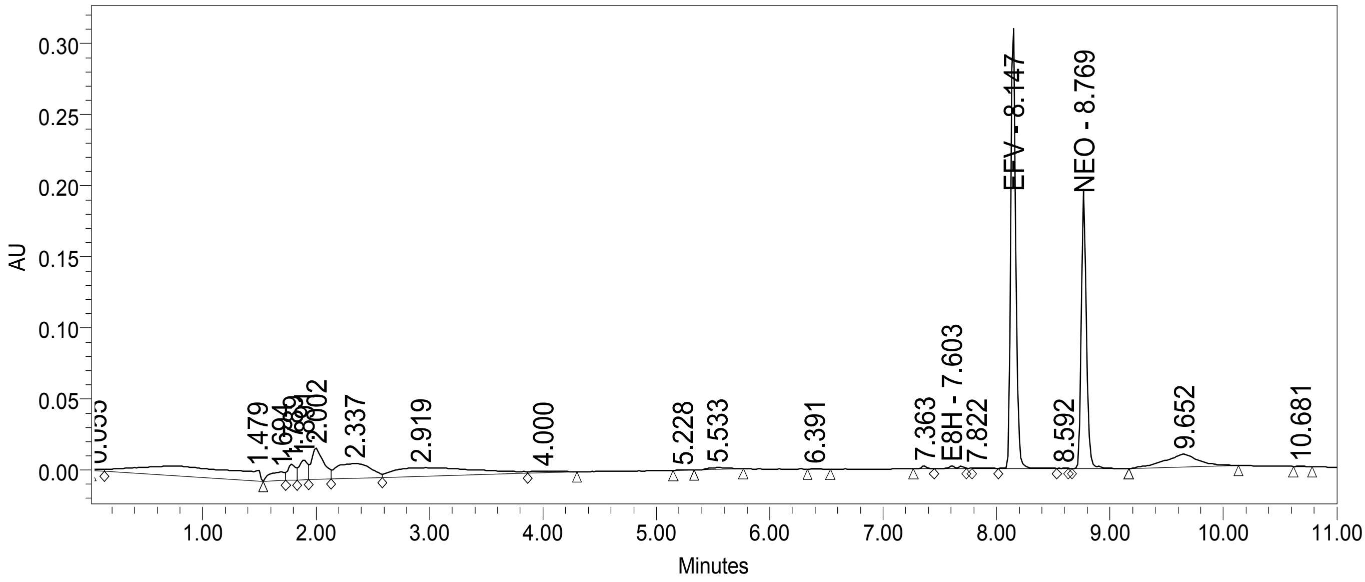

	Peak Name	RT	Area	% Area	Height
1		0.055	7941	0.25	1258
2		1.479	449211	14.28	7201
3		1.694	58772	1.87	6018
4		1.789	54594	1.74	11304
5		1.891	70573	2.24	13953
6		2.002	168657	5.36	22113
7		2.337	220434	7.01	10467
8		2.919	303253	9.64	6015
9		4.000	18530	0.59	1122
10		5.228	1402	0.04	177
11		5.533	18163	0.58	1379
12		6.391	1981	0.06	392

Reported by User: Saneesh (Saneesh) Project Name: HDI_Stellenbosch_2016

	Peak Name	RT	Area	% Area	Height
13		7.363	6826	0.22	1926
14	E8H	7.603	13525	0.43	1759
15		7.822	5403	0.17	632
16	EFV	8.147	948445	30.15	290067
17		8.592	1652	0.05	348
18	NEO	8.769	601861	19.14	178945
19		9.652	192442	6.12	9085
20		10.681	1630	0.05	339

Reported by User: Saneesh (Saneesh)   Project Name: HDI_Stellenbosch_2016

SAMPLE INFORMATION

Sample Name:	EFV TICL TDI Inc 200miM 1	Acquired By:	Saneesh
Sample Type:	Unknown	Date Acquired:	2017/01/06 09:12:43 PM
Vial:	16	Acq. Method Set:	HDI_EFV_E8H_NEO
Injection #:	1	Date Processed:	2017/01/07 11:10:33 AM
Injection Volume:	15.00 ul	Processing Method:	HDI_EFV_ W2996 245.0nm-1.2
Run Time:	11.0 Minutes	Channel Name:	W2996 245.0nm-1.2
Sample Set Name:	EFV TICL Inhib IC50_TD1 6_1_17	Proc. Chnl. Descr.:	W2996 PDA 245.0 nm at 1.2

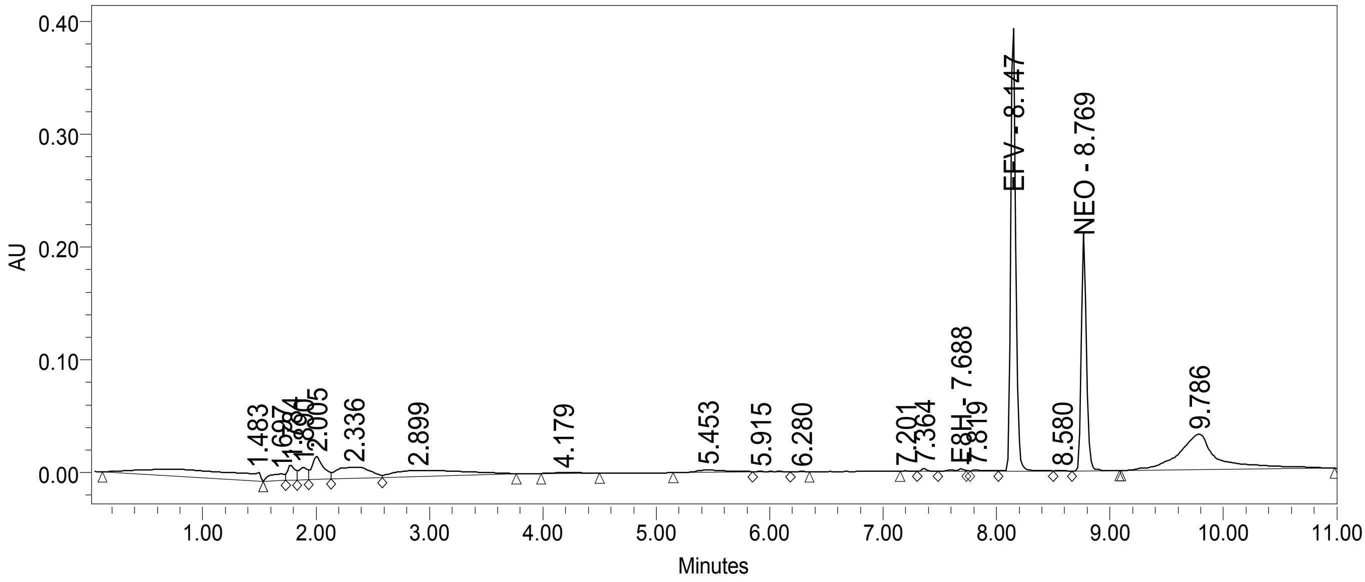

	Peak Name	RT	Area	% Area	Height
1		1.483	405684	10.41	7432
2		1.697	57437	1.47	5953
3		1.784	62000	1.59	13328
4		1.890	58272	1.50	10939
5		2.005	150351	3.86	20422
6		2.336	206484	5.30	10004
7		2.899	229005	5.88	5220
8		4.179	11792	0.30	640
9		5.453	43082	1.11	2278
10		5.915	7692	0.20	696
11		6.280	1281	0.03	222
12		7.201	1680	0.04	432

Reported by User: Saneesh (Saneesh)   Project Name: HDI_Stellenbosch_2016

	Peak Name	RT	Area	% Area	Height
13		7.364	8743	0.22	2422
14	E8H	7.688	13522	0.35	2170
15		7.819	7198	0.18	1006
16	EFV	8.147	1198937	30.78	367104
17		8.580	1293	0.03	198
18	NEO	8.769	648415	16.65	193288
19		9.786	782329	20.08	31649

SAMPLE INFORMATION

Sample Name:	EFV TICL TDI Inc 200miM 2	Acquired By:	Saneesh
Sample Type:	Unknown	Date Acquired:	2017/01/06 09:27:03 PM
Vial:	17	Acq. Method Set:	HDI_EFV_E8H_NEO
Injection #:	1	Date Processed:	2017/01/07 11:10:33 AM
Injection Volume:	15.00 ul	Processing Method:	HDI_EFV_ W2996 245.0nm-1.2
Run Time:	11.0 Minutes	Channel Name:	W2996 245.0nm-1.2
Sample Set Name:	EFV TICL Inhib IC50_TD1 6_1_17	Proc. Chnl. Descr.:	W2996 PDA 245.0 nm at 1.2

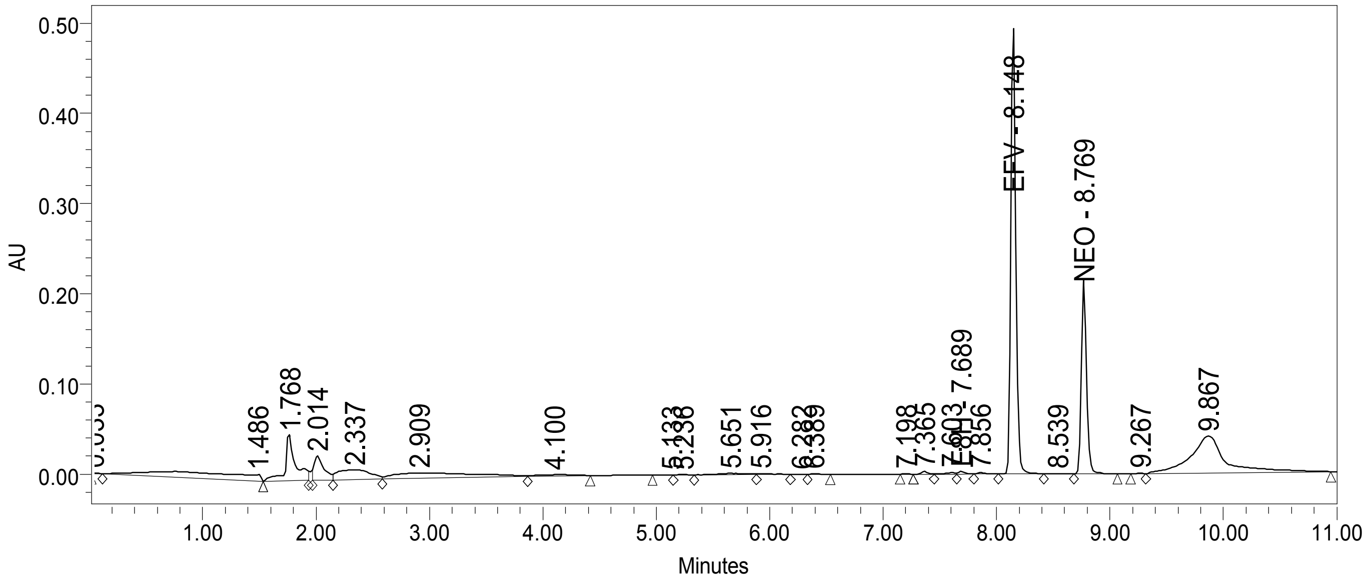

	Peak Name	RT	Area	% Area	Height
1		0.033	3337	0.07	915
2		1.486	413778	8.94	7523
3		1.768	319380	6.90	50365
4		2.014	166059	3.59	26510
5		2.337	215868	4.67	10631
6		2.909	310596	6.71	6049
7		4.100	36314	0.78	1607
8		5.133	1440	0.03	212
9		5.236	3416	0.07	455
10		5.651	33616	0.73	1692
11		5.916	13719	0.30	1311
12		6.282	3069	0.07	414

Reported by User: Saneesh (Saneesh) Project Name: HDI_Stellenbosch_2016

	Peak Name	RT	Area	% Area	Height
13		6.389	4082	0.09	653
14		7.198	1586	0.03	452
15		7.365	10267	0.22	2889
16		7.603	9700	0.21	1667
17	E8H	7.689	11535	0.25	2796
18		7.856	9140	0.20	1450
19	EFV	8.148	1500693	32.43	459074
20		8.539	3603	0.08	340
21	NEO	8.769	668506	14.45	197226
22		9.267	2936	0.06	902
23		9.867	884574	19.12	40994

Multi Sample Summary

Reported by User: Saneesh (Saneesh) Project Name: HDI_Stellenbosch_2016

SAMPLE INFORMATION

Sample Name:	RIF NELF TDI Control Incubate K1	Acquired By:	Saneesh
Sample Type:	Unknown	Date Acquired:	2017/01/11 10:08:25 AM
Vial:	2	Acq. Method Set:	HDI_RIF_25ODESRIF_NEO
Injection #:	1	Date Processed:	2017/01/11 03:04:45 PM
Injection Volume:	15.00 ul	Processing Method:	HDI_RIF_ W2996 254.0nm-1.2
Run Time:	12.5 Minutes	Channel Name:	W2996 254.0nm-1.2
Sample Set Name:	RIF NELF Inhb_TDI IC50 11_1_17	Proc. Chnl. Descr.:	W2996 PDA 254.0 nm at 1.2

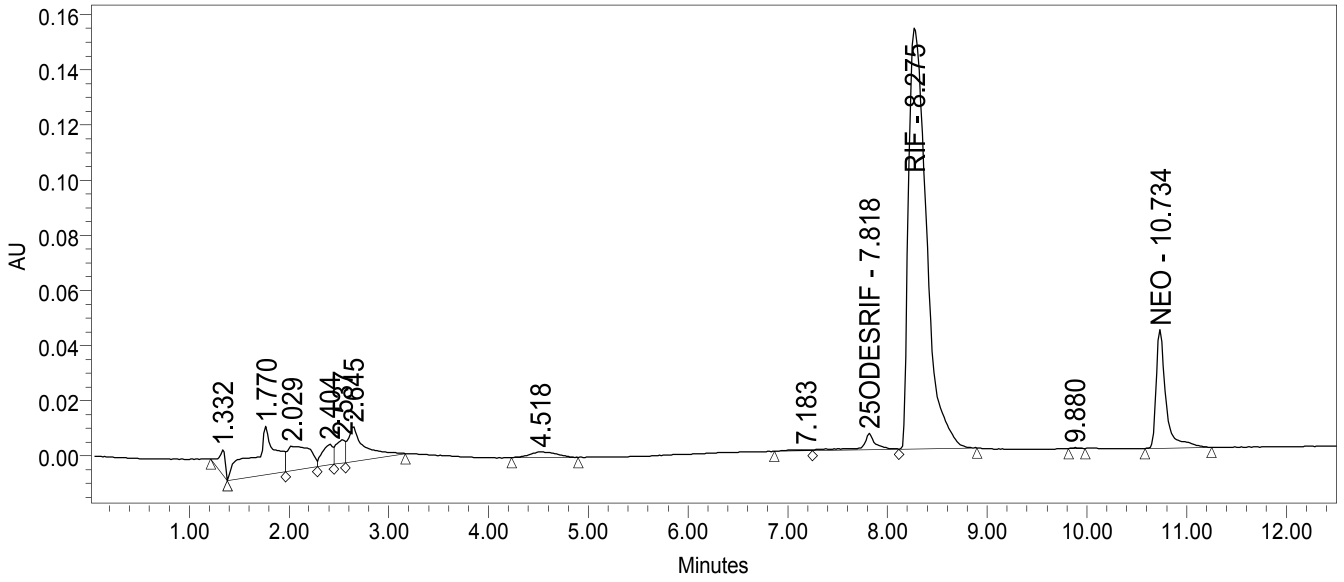

	Peak Name	RT	Area	% Area	Height
1		1.332	39767	1.32	8464
2		1.770	274916	9.16	17059
3		2.029	136616	4.55	8891
4		2.404	56836	1.89	7437
5		2.537	53484	1.78	8353
6		2.645	144268	4.81	12320
7		4.518	37446	1.25	1957
8		7.183	7907	0.26	461
9	25ODESRIF	7.818	56872	1.89	5909
10	RIF	8.275	1902726	63.37	153500
11		9.880	1648	0.05	322
12	NEO	10.734	289898	9.66	42593

SAMPLE INFORMATION

Sample Name:	RIF NELF TDI Control Incubate K2	Acquired By:	Saneesh
Sample Type:	Unknown	Date Acquired:	2017/01/11 10:24:08 AM
Vial:	3	Acq. Method Set:	HDI_RIF_25ODESRIF_NEO
Injection #:	1	Date Processed:	2017/01/11 03:04:46 PM
Injection Volume:	15.00 ul	Processing Method:	HDI_RIF_ W2996 254.0nm-1.2
Run Time:	12.5 Minutes	Channel Name:	W2996 254.0nm-1.2
Sample Set Name:	RIF NELF Inhb_TDI IC50 11_1_17	Proc. Chnl. Descr.:	W2996 PDA 254.0 nm at 1.2

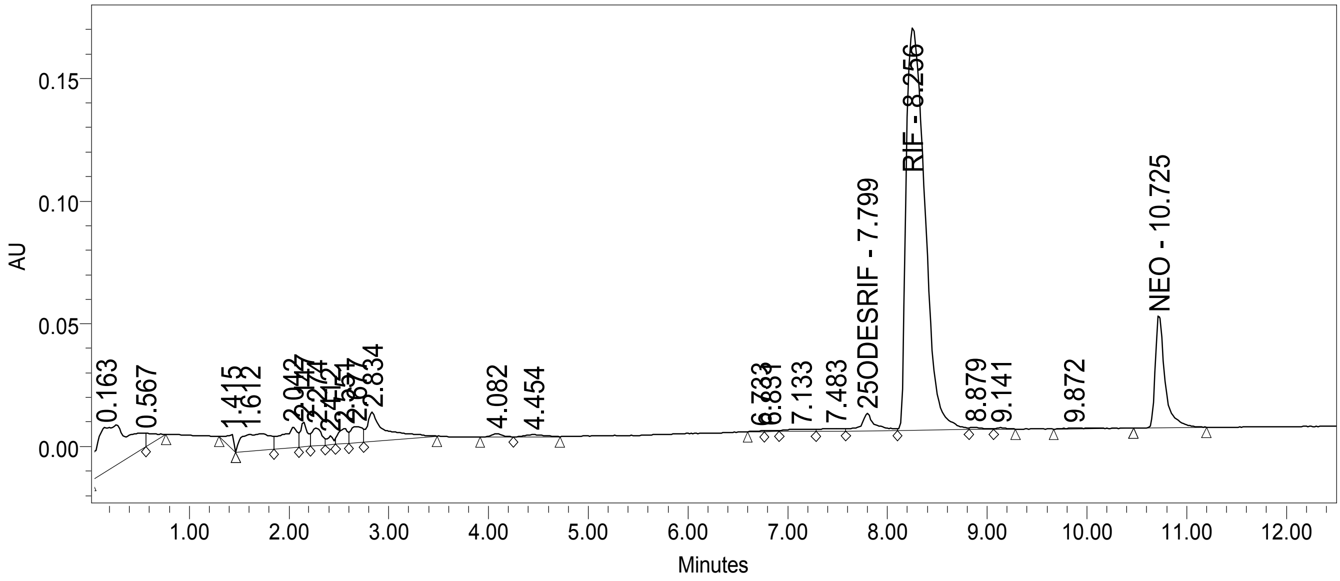

	Peak Name	RT	Area	% Area	Height
1		0.163	388626	10.72	17934
2		0.567	31621	0.87	5381
3		1.415	27651	0.76	5370
4		1.612	135335	3.73	6711
5		2.042	91831	2.53	8147
6		2.147	49655	1.37	9595
7		2.274	49430	1.36	7281
8		2.412	15461	0.43	3328
9		2.551	40054	1.10	6102
10		2.677	53753	1.48	6577
11		2.834	153376	4.23	11824
12		4.082	10614	0.29	1374

Reported by User: Saneesh (Saneesh) Project Name: HDI_Stellenbosch_2016

	Peak Name	RT	Area	% Area	Height
13		4.454	12891	0.36	953
14		6.733	2789	0.08	419
15		6.831	4484	0.12	522
16		7.133	16594	0.46	881
17		7.483	15803	0.44	969
18	25ODESRIF	7.799	66677	1.84	7005
19	RIF	8.256	2127801	58.68	164382
20		8.879	7787	0.21	889
21		9.141	3550	0.10	551
22		9.872	7830	0.22	424
23	NEO	10.725	312591	8.62	45946

SAMPLE INFORMATION

Sample Name:	RIF NELF TDI Inc 1miM 1	Acquired By:	Saneesh
Sample Type:	Unknown	Date Acquired:	2017/01/11 10:44:42 AM
Vial:	4	Acq. Method Set:	HDI_RIF_25ODESRIF_NEO
Injection #:	1	Date Processed:	2017/01/11 03:05:04 PM
Injection Volume:	15.00 ul	Processing Method:	HDI_RIF_ W2996 254.0nm-1.2
Run Time:	12.5 Minutes	Channel Name:	W2996 254.0nm-1.2
Sample Set Name:	RIF NELF Inhb_TDI IC50 11_1_17	Proc. Chnl. Descr.:	W2996 PDA 254.0 nm at 1.2

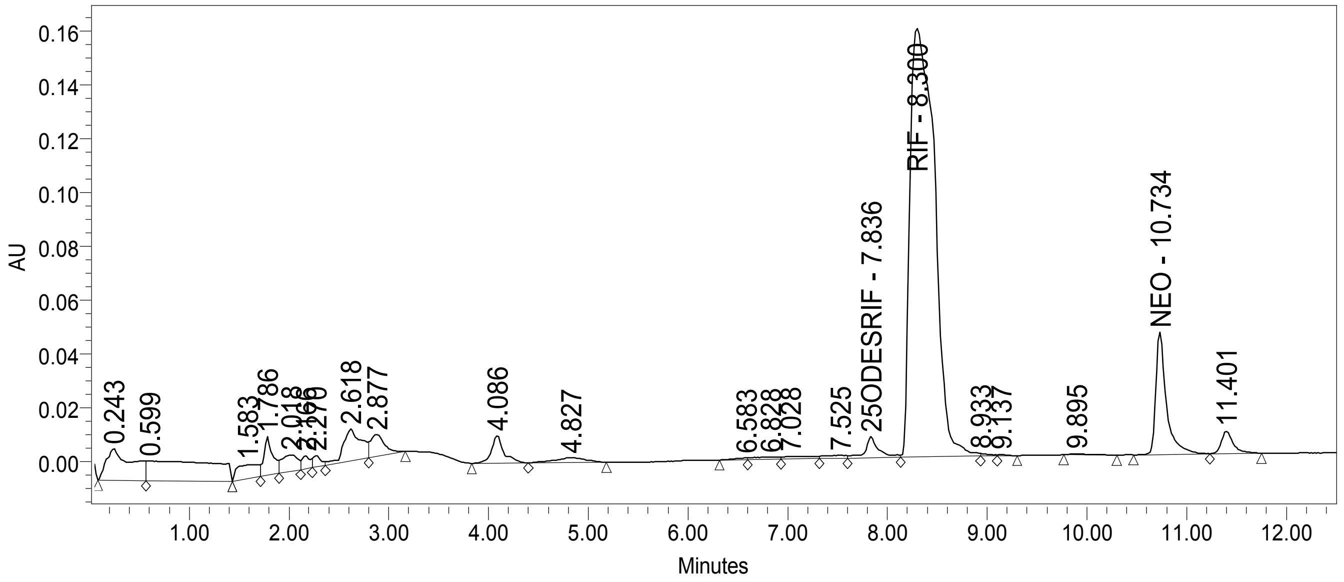

	Peak Name	RT	Area	% Area	Height
1		0.243	209666	4.69	11631
2		0.599	350634	7.85	7251
3		1.583	76800	1.72	5142
4		1.786	86476	1.94	13795
5		2.018	67903	1.52	6078
6		2.166	26901	0.60	4662
7		2.270	22741	0.51	4137
8		2.618	148509	3.32	11738
9		2.877	81696	1.83	8249
10		4.086	86568	1.94	10146
11		4.827	39184	0.88	1815
12		6.583	6423	0.14	671

Reported by User: Saneesh (Saneesh) Project Name: HDI_Stellenbosch_2016

	Peak Name	RT	Area	% Area	Height
13		6.828	15003	0.34	782
14		7.028	20587	0.46	1017
15		7.525	17684	0.40	1123
16	25ODESRIF	7.836	74144	1.66	7777
17	RIF	8.300	2712678	60.73	159215
18		8.933	6283	0.14	961
19		9.137	3231	0.07	545
20		9.895	6323	0.14	412
21	NEO	10.734	334287	7.48	45139
22		11.401	72720	1.63	8391

Reported by User: Saneesh (Saneesh) Project Name: HDI_Stellenbosch_2016

SAMPLE INFORMATION

Sample Name:	RIF NELF TDI Inc 1miM 2	Acquired By:	Saneesh
Sample Type:	Unknown	Date Acquired:	2017/01/11 11:00:25 AM
Vial:	5	Acq. Method Set:	HDI_RIF_25ODESRIF_NEO
Injection #:	1	Date Processed:	2017/01/11 03:05:04 PM
Injection Volume:	15.00 ul	Processing Method:	HDI_RIF_ W2996 254.0nm-1.2
Run Time:	12.5 Minutes	Channel Name:	W2996 254.0nm-1.2
Sample Set Name:	RIF NELF Inhb_TDI IC50 11_1_17	Proc. Chnl. Descr.:	W2996 PDA 254.0 nm at 1.2

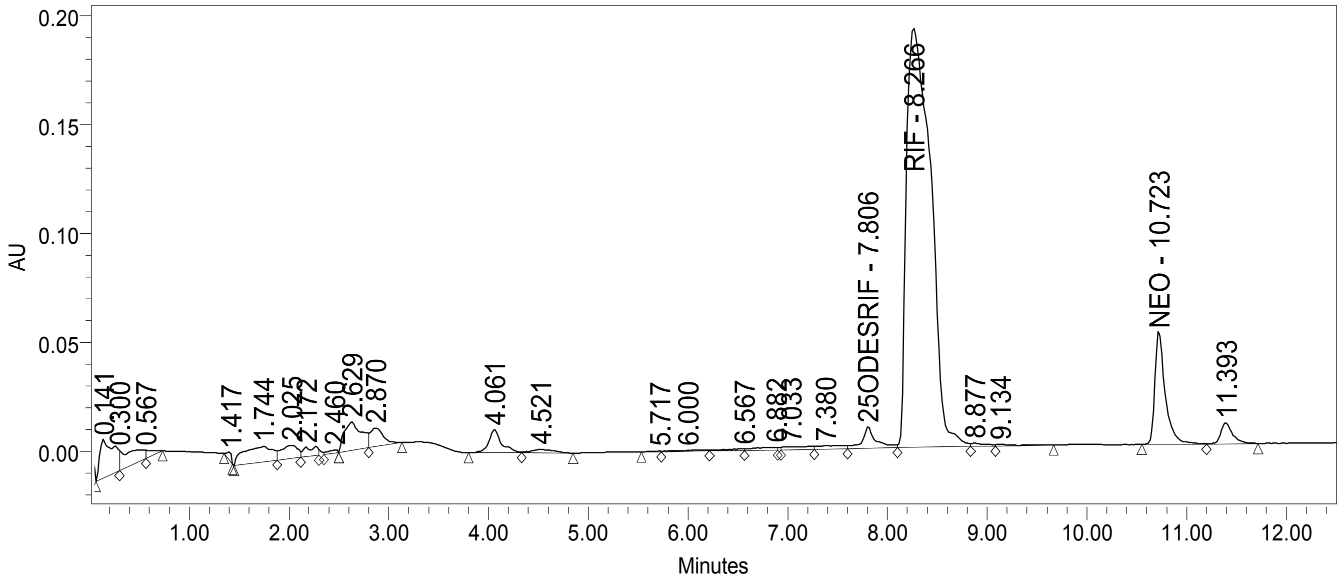

	Peak Name	RT	Area	% Area	Height
1		0.141	167203	3.46	17580
2		0.300	96384	2.00	9286
3		0.567	19058	0.39	3814
4		1.417	10606	0.22	3735
5		1.744	147050	3.04	7071
6		2.025	68931	1.43	5984
7		2.172	36761	0.76	4197
8		2.460	10278	0.21	1625
9		2.629	157959	3.27	12991
10		2.870	78406	1.62	8580
11		4.061	96146	1.99	10744
12		4.521	25833	0.53	1610

Reported by User: Saneesh (Saneesh) Project Name: HDI_Stellenbosch_2016

	Peak Name	RT	Area	% Area	Height
13		5.717	2191	0.05	311
14		6.000	10947	0.23	496
15		6.567	11103	0.23	695
16		6.882	19712	0.41	1145
17		7.033	25124	0.52	1326
18		7.380	28328	0.59	1550
19	25ODESRIF	7.806	94317	1.95	9759
20	RIF	8.266	3242173	67.11	192441
21		8.877	15977	0.33	1539
22		9.134	11568	0.24	822
23	NEO	10.723	375097	7.76	51784
24		11.393	79943	1.65	9437

SAMPLE INFORMATION

Sample Name:	RIF NELF TDI Inc 10miM 1	Acquired By:	Saneesh
Sample Type:	Unknown	Date Acquired:	2017/01/11 11:16:08 AM
Vial:	6	Acq. Method Set:	HDI_RIF_25ODESRIF_NEO
Injection #:	1	Date Processed:	2017/01/11 03:05:04 PM
Injection Volume:	15.00 ul	Processing Method:	HDI_RIF_ W2996 254.0nm-1.2
Run Time:	12.5 Minutes	Channel Name:	W2996 254.0nm-1.2
Sample Set Name:	RIF NELF Inhb_TDI IC50 11_1_17	Proc. Chnl. Descr.:	W2996 PDA 254.0 nm at 1.2

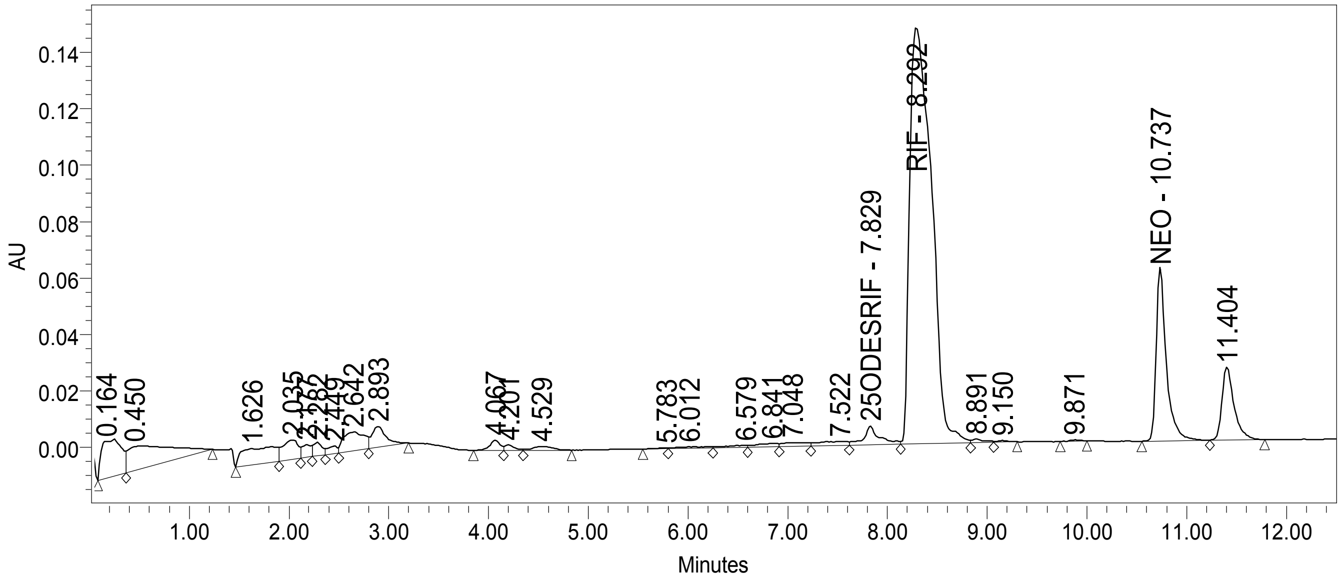

	Peak Name	RT	Area	% Area	Height
1		0.164	181002	4.52	13303
2		0.450	245828	6.14	8425
3		1.626	135710	3.39	5856
4		2.035	74586	1.86	6834
5		2.177	28951	0.72	4438
6		2.282	29211	0.73	4719
7		2.449	17944	0.45	2515
8		2.642	97046	2.42	6609
9		2.893	73703	1.84	7438
10		4.067	26287	0.66	3691
11		4.201	13572	0.34	1941
12		4.529	19814	0.49	1356

Reported by User: Saneesh (Saneesh) Project Name: HDI_Stellenbosch_2016

	Peak Name	RT	Area	% Area	Height
13		5.783	2639	0.07	268
14		6.012	10269	0.26	459
15		6.579	9787	0.24	619
16		6.841	17807	0.44	1117
17		7.048	22303	0.56	1279
18		7.522	28251	0.71	1358
19	25ODESRIF	7.829	72024	1.80	6641
20	RIF	8.292	2228960	55.65	148086
21		8.891	11028	0.28	1266
22		9.150	4021	0.10	604
23		9.871	4223	0.11	501
24	NEO	10.737	431378	10.77	61170
25		11.404	218991	5.47	25847

SAMPLE INFORMATION

Sample Name:	RIF NELF TDI Inc 10miM 2	Acquired By:	Saneesh
Sample Type:	Unknown	Date Acquired:	2017/01/11 11:31:53 AM
Vial:	7	Acq. Method Set:	HDI_RIF_25ODESRIF_NEO
Injection #:	1	Date Processed:	2017/01/11 03:05:04 PM
Injection Volume:	15.00 ul	Processing Method:	HDI_RIF_ W2996 254.0nm-1.2
Run Time:	12.5 Minutes	Channel Name:	W2996 254.0nm-1.2
Sample Set Name:	RIF NELF Inhb_TDI IC50 11_1_17	Proc. Chnl. Descr.:	W2996 PDA 254.0 nm at 1.2

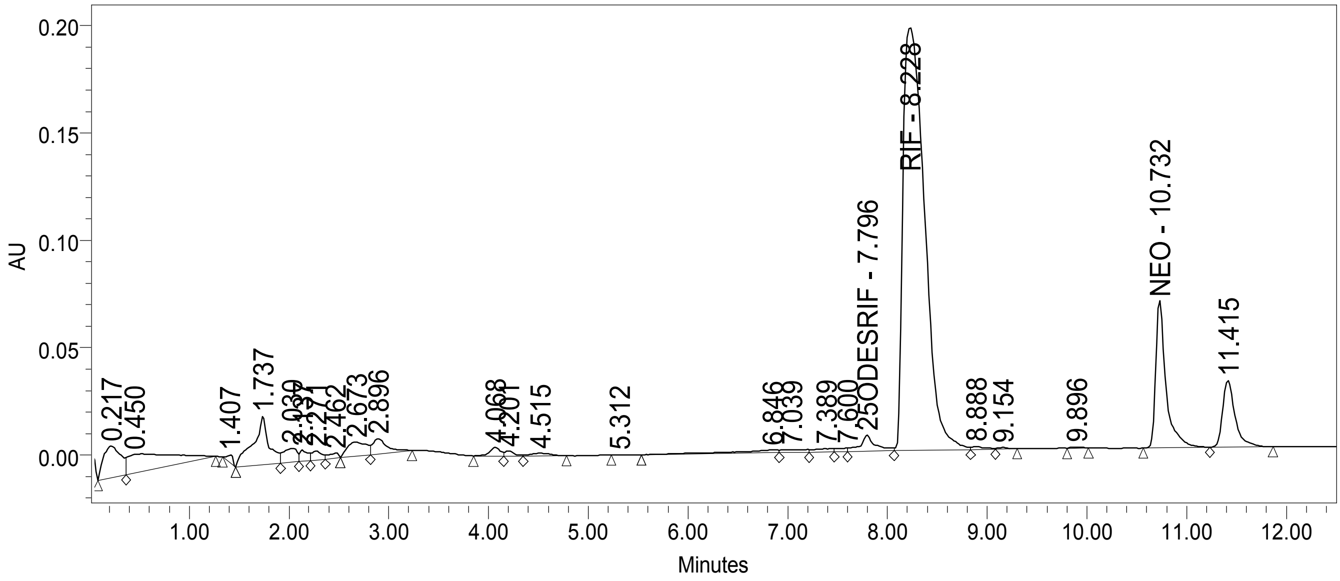

	Peak Name	RT	Area	% Area	Height
1		0.217	177302	3.56	14596
2		0.450	259784	5.22	8590
3		1.407	14754	0.30	3969
4		1.737	253397	5.09	22055
5		2.030	61955	1.24	6485
6		2.137	28900	0.58	5052
7		2.271	28922	0.58	4172
8		2.462	17261	0.35	2503
9		2.673	86772	1.74	6503
10		2.896	69360	1.39	7058
11		4.068	28933	0.58	3998
12		4.201	15451	0.31	2293

Reported by User: Saneesh (Saneesh) Project Name: HDI_Stellenbosch_2016

	Peak Name	RT	Area	% Area	Height
13		4.515	18479	0.37	1287
14		5.312	2141	0.04	188
15		6.846	44042	0.88	1188
16		7.039	22252	0.45	1301
17		7.389	19765	0.40	1437
18		7.600	12286	0.25	1565
19	25ODESRIF	7.796	78242	1.57	7391
20	RIF	8.228	2957665	59.38	196967
21		8.888	12214	0.25	1319
22		9.154	4587	0.09	659
23		9.896	3896	0.08	474
24	NEO	10.732	490267	9.84	68042
25		11.415	272466	5.47	30971

SAMPLE INFORMATION

Sample Name:	RIF NELF TDI Inc 25miM 1	Acquired By:	Saneesh
Sample Type:	Unknown	Date Acquired:	2017/01/11 11:48:34 AM
Vial:	8	Acq. Method Set:	HDI_RIF_25ODESRIF_NEO
Injection #:	1	Date Processed:	2017/01/11 03:05:04 PM
Injection Volume:	15.00 ul	Processing Method:	HDI_RIF_ W2996 254.0nm-1.2
Run Time:	12.5 Minutes	Channel Name:	W2996 254.0nm-1.2
Sample Set Name:	RIF NELF Inhb_TDI IC50 11_1_17	Proc. Chnl. Descr.:	W2996 PDA 254.0 nm at 1.2

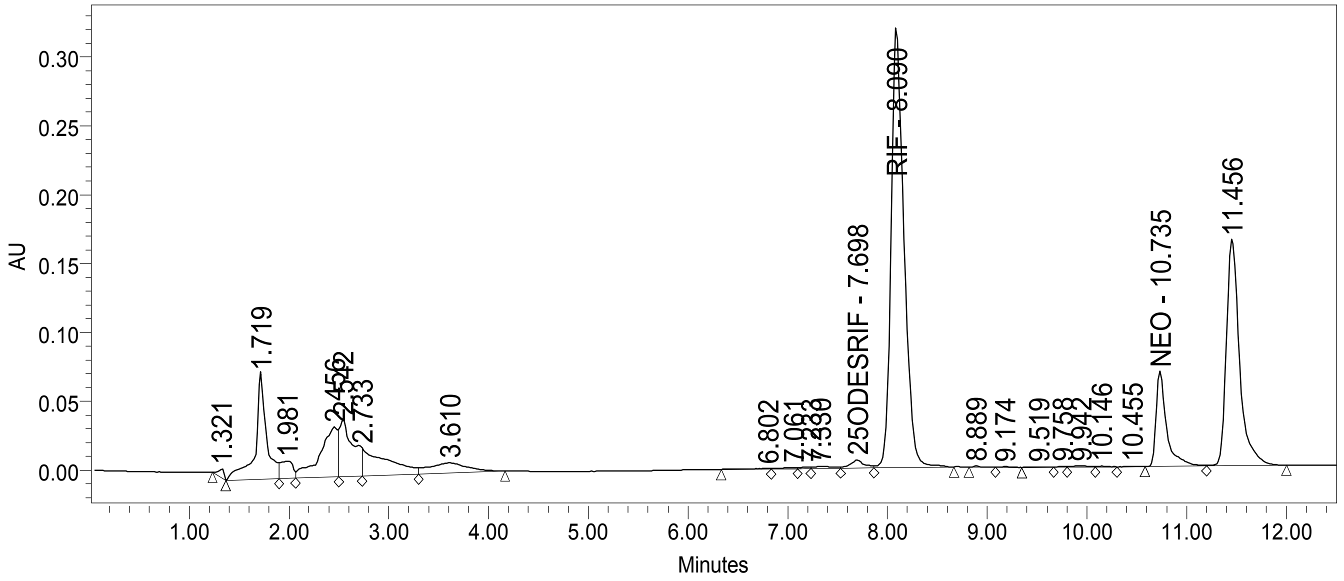

	Peak Name	RT	Area	% Area	Height
1		1.321	24547	0.34	6227
2		1.719	601398	8.45	75683
3		1.981	107777	1.51	12233
4		2.456	453664	6.37	36115
5		2.542	391935	5.50	40976
6		2.733	347150	4.88	20124
7		3.610	199121	2.80	7355
8		6.802	8559	0.12	344
9		7.061	8917	0.13	759
10		7.233	6929	0.10	944
11		7.330	19337	0.27	1274
12	25ODESRIF	7.698	54632	0.77	5799

	Peak Name	RT	Area	% Area	Height
13	RIF	8.090	2854909	40.09	320466
14		8.889	5017	0.07	864
15		9.174	3202	0.04	519
16		9.519	3662	0.05	327
17		9.758	3323	0.05	658
18		9.942	10375	0.15	847
19		10.146	4172	0.06	584
20		10.455	2406	0.03	331
21	NEO	10.735	501898	7.05	68798
22		11.456	1507832	21.18	164691

Reported by User: Saneesh (Saneesh)   Project Name: HDI_Stellenbosch_2016

SAMPLE INFORMATION

Sample Name:	RIF NELF TDI Inc 25miM 2	Acquired By:	Saneesh
Sample Type:	Unknown	Date Acquired:	2017/01/11 12:04:19 PM
Vial:	9	Acq. Method Set:	HDI_RIF_25ODESRIF_NEO
Injection #:	1	Date Processed:	2017/01/11 03:05:04 PM
Injection Volume:	15.00 ul	Processing Method:	HDI_RIF_ W2996 254.0nm-1.2
Run Time:	12.5 Minutes	Channel Name:	W2996 254.0nm-1.2
Sample Set Name:	RIF NELF Inhb_TDI IC50 11_1_17	Proc. Chnl. Descr.:	W2996 PDA 254.0 nm at 1.2

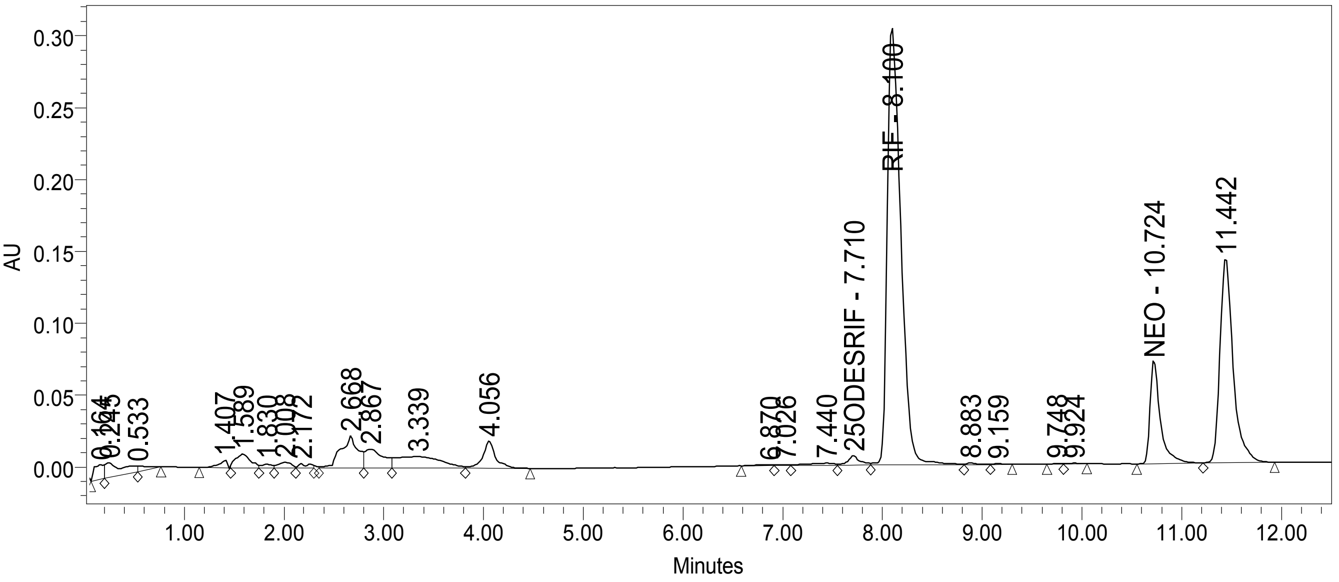

	Peak Name	RT	Area	% Area	Height
1		0.164	67764	1.11	9774
2		0.245	123283	2.02	10274
3		0.533	25843	0.42	3765
4		1.407	30657	0.50	5086
5		1.589	97765	1.61	9415
6		1.830	17175	0.28	2580
7		2.008	34195	0.56	3780
8		2.172	21926	0.36	2927
9		2.668	264132	4.34	21701
10		2.867	169083	2.78	13111
11		3.339	252413	4.15	7922
12		4.056	170806	2.81	18758

Reported by User: Saneesh (Saneesh) Project Name: HDI_Stellenbosch_2016

	Peak Name	RT	Area	% Area	Height
13		6.870	7176	0.12	551
14		7.026	6641	0.11	754
15		7.440	31343	0.51	1624
16	25ODESRIF	7.710	56466	0.93	6474
17	RIF	8.100	2876259	47.24	304174
18		8.883	9080	0.15	1149
19		9.159	3762	0.06	592
20		9.748	2408	0.04	398
21		9.924	3303	0.05	449
22	NEO	10.724	513369	8.43	71500
23		11.442	1304338	21.42	142394

SAMPLE INFORMATION

Sample Name:	RIF NELF TDI Inc 50miM 1	Acquired By:	Saneesh
Sample Type:	Unknown	Date Acquired:	2017/01/11 12:20:02 PM
Vial:	10	Acq. Method Set:	HDI_RIF_25ODESRIF_NEO
Injection #:	1	Date Processed:	2017/01/11 03:05:04 PM
Injection Volume:	15.00 ul	Processing Method:	HDI_RIF_ W2996 254.0nm-1.2
Run Time:	12.5 Minutes	Channel Name:	W2996 254.0nm-1.2
Sample Set Name:	RIF NELF Inhb_TDI IC50 11_1_17	Proc. Chnl. Descr.:	W2996 PDA 254.0 nm at 1.2

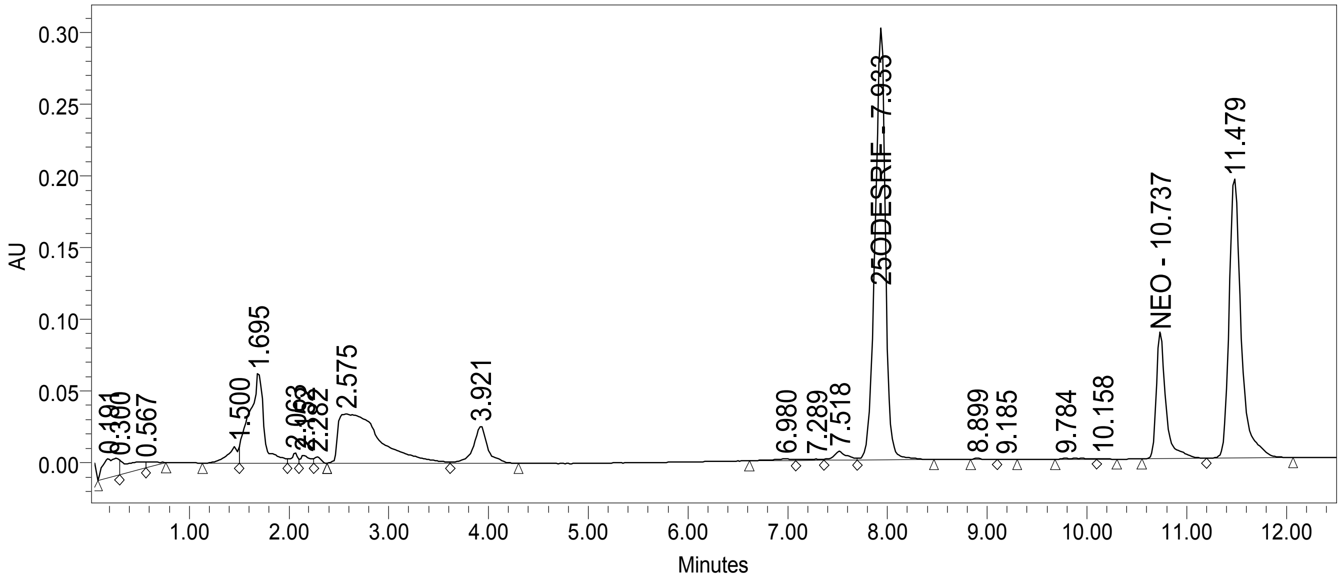

	Peak Name	RT	Area	% Area	Height
1		0.191	132305	1.92	12999
2		0.300	95787	1.39	9955
3		0.567	23651	0.34	3953
4		1.500	86951	1.26	11600
5		1.695	658648	9.57	61018
6		2.063	31252	0.45	6830
7		2.152	35501	0.52	5401
8		2.282	19055	0.28	4395
9		2.575	1023518	14.87	34413
10		3.921	262708	3.82	25713
11		6.980	17818	0.26	1324
12		7.289	14596	0.21	937

Reported by User: Saneesh (Saneesh)   Project Name: HDI_Stellenbosch_2016

	Peak Name	RT	Area	% Area	Height
13		7.518	57377	0.83	6098
14	25ODESRIF	7.933	2161199	31.40	299828
15	RIF	8.100			
16		8.899	6176	0.09	1106
17		9.185	2378	0.03	386
18		9.784	13162	0.19	793
19		10.158	3226	0.05	515
20	NEO	10.737	587778	8.54	87569
21		11.479	1650068	23.97	194599

SAMPLE INFORMATION

Sample Name:	RIF NELF TDI Inc 50miM 2	Acquired By:	Saneesh
Sample Type:	Unknown	Date Acquired:	2017/01/11 12:35:55 PM
Vial:	11	Acq. Method Set:	HDI_RIF_25ODESRIF_NEO
Injection #:	1	Date Processed:	2017/01/11 03:05:04 PM
Injection Volume:	15.00 ul	Processing Method:	HDI_RIF_ W2996 254.0nm-1.2
Run Time:	12.5 Minutes	Channel Name:	W2996 254.0nm-1.2
Sample Set Name:	RIF NELF Inhb_TDI IC50 11_1_17	Proc. Chnl. Descr.:	W2996 PDA 254.0 nm at 1.2

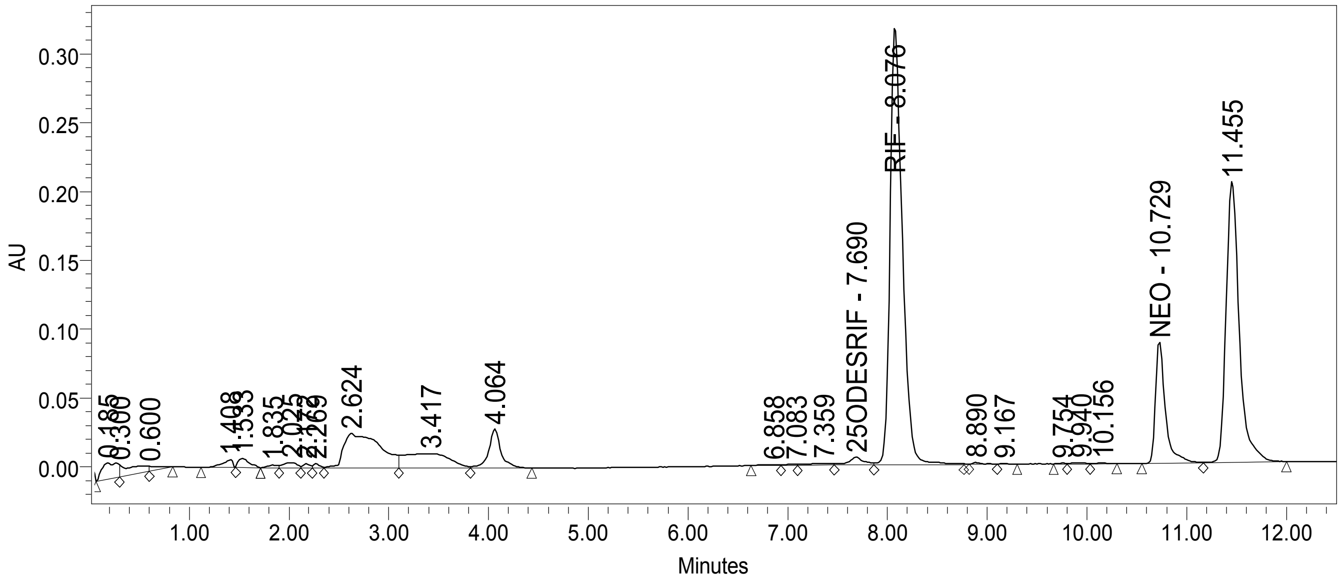

	Peak Name	RT	Area	% Area	Height
1		0.185	121350	1.75	11557
2		0.300	93770	1.35	8489
3		0.600	23663	0.34	3465
4		1.408	41684	0.60	6149
5		1.533	52254	0.75	6597
6		1.835	12358	0.18	1891
7		2.025	35585	0.51	3761
8		2.172	13916	0.20	2953
9		2.269	12615	0.18	2957
10		2.624	632592	9.12	25064
11		3.417	335553	4.84	10464
12		4.064	243660	3.51	28516

Reported by User: Saneesh (Saneesh) Project Name: HDI_Stellenbosch_2016

	Peak Name	RT	Area	% Area	Height
13		6.858	7052	0.10	572
14		7.083	7050	0.10	821
15		7.359	23818	0.34	1276
16	25ODESRIF	7.690	61501	0.89	5790
17	RIF	8.076	2716736	39.15	321448
18		8.890	9706	0.14	1271
19		9.167	2914	0.04	500
20		9.754	3275	0.05	641
21		9.940	8335	0.12	820
22		10.156	5487	0.08	681
23	NEO	10.729	607964	8.76	87820
24		11.455	1866266	26.89	204289

Reported by User: Saneesh (Saneesh)   Project Name: HDI_Stellenbosch_2016

SAMPLE INFORMATION

Sample Name:	RIF NELF TDI Inc 75miM 1	Acquired By:	Saneesh
Sample Type:	Unknown	Date Acquired:	2017/01/11 12:51:40 PM
Vial:	12	Acq. Method Set:	HDI_RIF_25ODESRIF_NEO
Injection #:	1	Date Processed:	2017/01/11 03:05:04 PM
Injection Volume:	15.00 ul	Processing Method:	HDI_RIF_ W2996 254.0nm-1.2
Run Time:	12.5 Minutes	Channel Name:	W2996 254.0nm-1.2
Sample Set Name:	RIF NELF Inhb_TDI IC50 11_1_17	Proc. Chnl. Descr.:	W2996 PDA 254.0 nm at 1.2

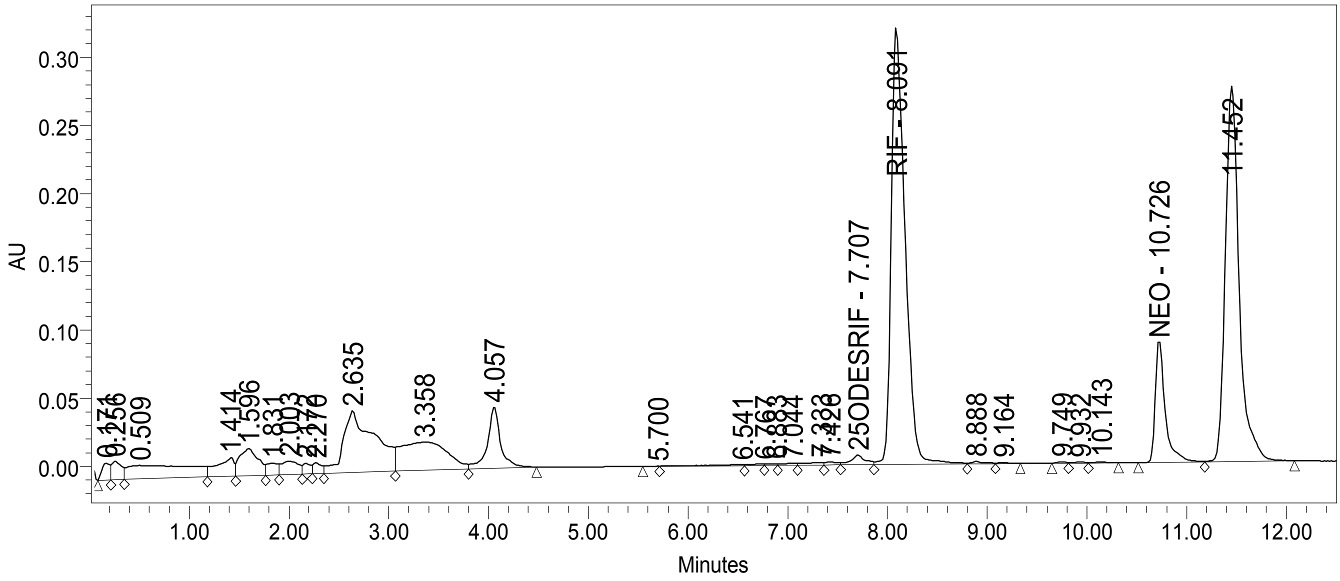

	Peak Name	RT	Area	% Area	Height
1		0.171	71244	0.72	12548
2		0.256	90920	0.92	13433
3		0.509	438772	4.42	9854
4		1.414	163898	1.65	13741
5		1.596	258781	2.61	19712
6		1.831	65748	0.66	8785
7		2.003	117516	1.18	9755
8		2.172	42444	0.43	7927
9		2.270	46546	0.47	7770
10		2.635	989704	9.97	44902
11		3.358	674430	6.80	20646
12		4.057	410518	4.14	45008

Reported by User: Saneesh (Saneesh) Project Name: HDI_Stellenbosch_2016

	Peak Name	RT	Area	% Area	Height
13		5.700	1632	0.02	225
14		6.541	21597	0.22	690
15		6.767	10680	0.11	1072
16		6.883	9159	0.09	1209
17		7.044	15550	0.16	1414
18		7.333	26198	0.26	1832
19		7.426	19013	0.19	2103
20	25ODESRIF	7.707	69789	0.70	6914
21	RIF	8.091	3075585	30.99	322478
22		8.888	13735	0.14	1592
23		9.164	5324	0.05	708
24		9.749	5110	0.05	887
25		9.932	6193	0.06	712
26		10.143	6827	0.07	818
27	NEO	10.726	614051	6.19	89204
28		11.452	2653114	26.73	275234

SAMPLE INFORMATION

Sample Name:	RIF NELF TDI Inc 75miM 2	Acquired By:	Saneesh
Sample Type:	Unknown	Date Acquired:	2017/01/11 01:07:23 PM
Vial:	13	Acq. Method Set:	HDI_RIF_25ODESRIF_NEO
Injection #:	1	Date Processed:	2017/01/11 03:05:04 PM
Injection Volume:	15.00 ul	Processing Method:	HDI_RIF_ W2996 254.0nm-1.2
Run Time:	12.5 Minutes	Channel Name:	W2996 254.0nm-1.2
Sample Set Name:	RIF NELF Inhb_TDI IC50 11_1_17	Proc. Chnl. Descr.:	W2996 PDA 254.0 nm at 1.2

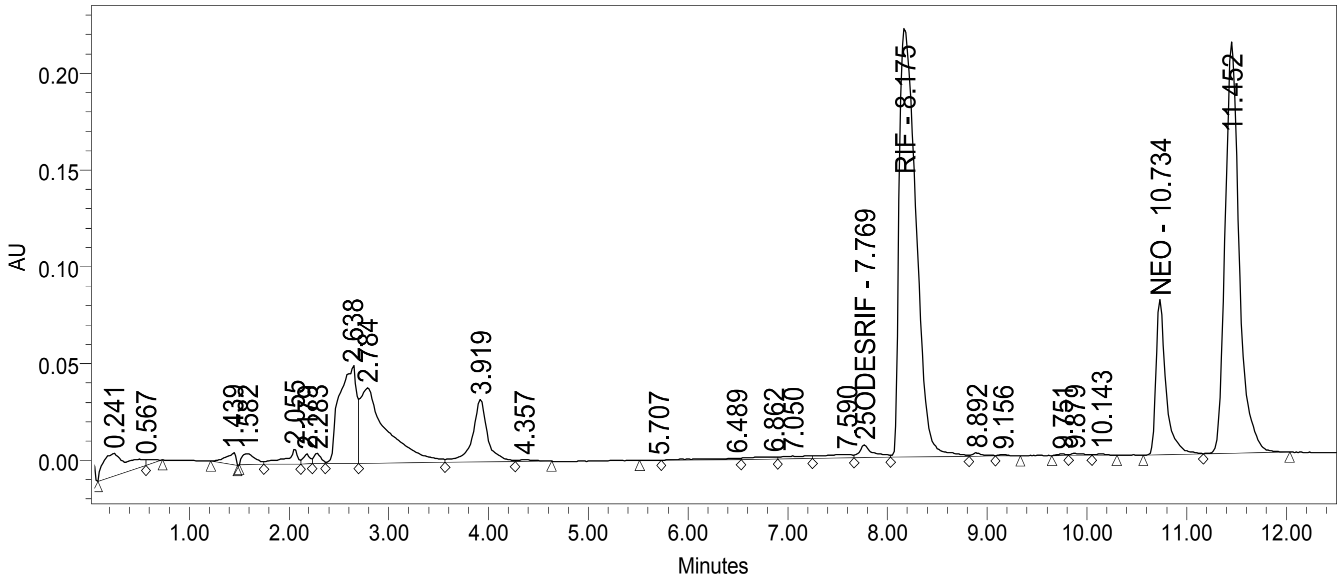

	Peak Name	RT	Area	% Area	Height
1		0.241	199181	2.63	11494
2		0.567	15165	0.20	3078
3		1.439	43231	0.57	6353
4		1.582	51526	0.68	5526
5		2.055	64780	0.86	7269
6		2.179	24396	0.32	4899
7		2.283	26295	0.35	5280
8		2.638	612283	8.09	49678
9		2.784	729400	9.64	38882
10		3.919	340312	4.50	32141
11		4.357	12501	0.17	979
12		5.707	2102	0.03	227

Reported by User: Saneesh (Saneesh) Project Name: HDI_Stellenbosch_2016

	Peak Name	RT	Area	% Area	Height
13		6.489	20476	0.27	681
14		6.862	20128	0.27	1059
15		7.050	25061	0.33	1322
16		7.590	40179	0.53	1966
17	25ODESRIF	7.769	63009	0.83	6666
18	RIF	8.175	2594840	34.30	223232
19		8.892	14409	0.19	1764
20		9.156	5276	0.07	760
21		9.751	4401	0.06	724
22		9.879	8667	0.11	947
23		10.143	5391	0.07	683
24	NEO	10.734	552733	7.31	79456
25		11.452	2089625	27.62	212080

SAMPLE INFORMATION

Sample Name:	RIF NELF TDI Inc 100miM 1	Acquired By:	Saneesh
Sample Type:	Unknown	Date Acquired:	2017/01/11 01:23:08 PM
Vial:	14	Acq. Method Set:	HDI_RIF_25ODESRIF_NEO
Injection #:	1	Date Processed:	2017/01/11 03:05:04 PM
Injection Volume:	15.00 ul	Processing Method:	HDI_RIF_ W2996 254.0nm-1.2
Run Time:	12.5 Minutes	Channel Name:	W2996 254.0nm-1.2
Sample Set Name:	RIF NELF Inhb_TDI IC50 11_1_17	Proc. Chnl. Descr.:	W2996 PDA 254.0 nm at 1.2

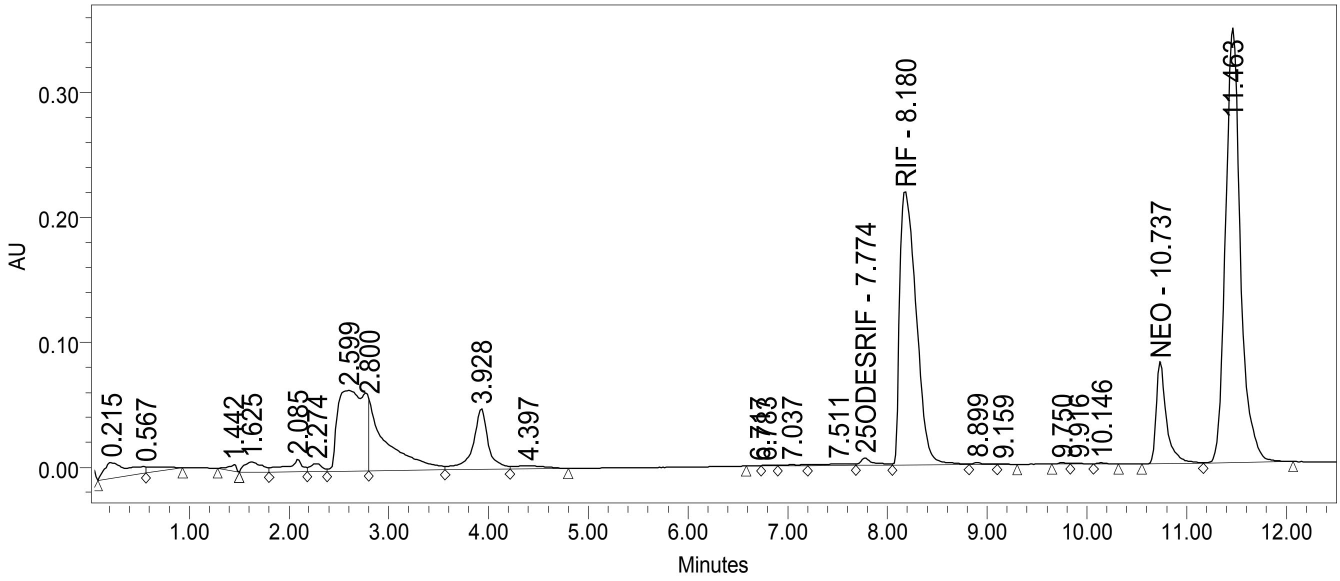

	Peak Name	RT	Area	% Area	Height
1		0.215	215214	2.16	12677
2		0.567	54626	0.55	4988
3		1.442	30102	0.30	5597
4		1.625	102181	1.03	8179
5		2.085	111092	1.11	9545
6		2.274	51130	0.51	6319
7		2.599	1240348	12.45	64442
8		2.800	723054	7.26	57767
9		3.928	518978	5.21	48672
10		4.397	53248	0.53	2482
11		6.717	1639	0.02	327
12		6.783	4543	0.05	538

	Peak Name	RT	Area	% Area	Height
13		7.037	11772	0.12	777
14		7.511	31929	0.32	1504
15	25ODESRIF	7.774	53522	0.54	6048
16	RIF	8.180	2522904	25.32	219141
17		8.899	13366	0.13	1772
18		9.159	3886	0.04	656
19		9.750	7741	0.08	1214
20		9.916	7726	0.08	769
21		10.146	7145	0.07	1022
22	NEO	10.737	576868	5.79	81352
23		11.463	3621261	36.34	348511

SAMPLE INFORMATION

Sample Name:	RIF NELF TDI Inc 100miM 2	Acquired By:	Saneesh
Sample Type:	Unknown	Date Acquired:	2017/01/11 01:38:50 PM
Vial:	15	Acq. Method Set:	HDI_RIF_25ODESRIF_NEO
Injection #:	1	Date Processed:	2017/01/11 03:05:04 PM
Injection Volume:	15.00 ul	Processing Method:	HDI_RIF_ W2996 254.0nm-1.2
Run Time:	12.5 Minutes	Channel Name:	W2996 254.0nm-1.2
Sample Set Name:	RIF NELF Inhb_TDI IC50 11_1_17	Proc. Chnl. Descr.:	W2996 PDA 254.0 nm at 1.2

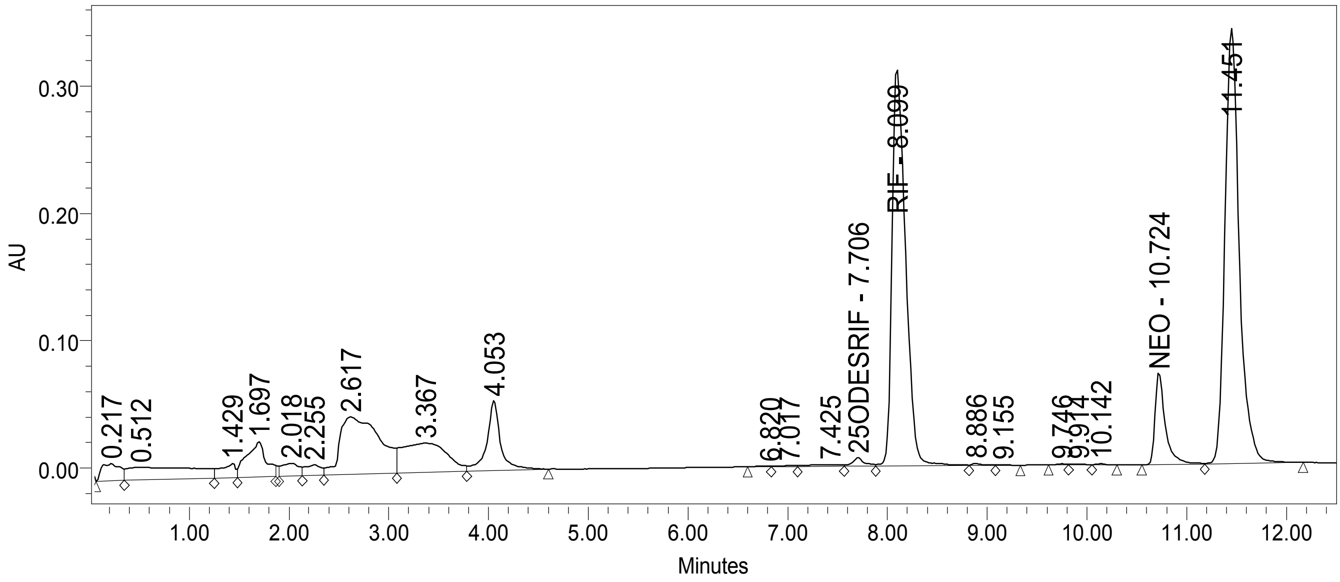

	Peak Name	RT	Area	% Area	Height
1		0.217	173390	1.60	13264
2		0.512	472856	4.35	9876
3		1.429	115238	1.06	10936
4		1.697	372504	3.43	27296
5		2.018	120349	1.11	9898
6		2.255	90642	0.83	8116
7		2.617	1249806	11.50	45178
8		3.367	716760	6.60	22843
9		4.053	521489	4.80	54750
10		6.820	5079	0.05	516
11		7.017	10635	0.10	805
12		7.425	33408	0.31	1578

Reported by User: Saneesh (Saneesh) Project Name: HDI_Stellenbosch_2016

	Peak Name	RT	Area	% Area	Height
13	25ODESRIF	7.706	57114	0.53	6610
14	RIF	8.099	2951020	27.16	311875
15		8.886	14079	0.13	1910
16		9.155	5113	0.05	664
17		9.746	6672	0.06	1051
18		9.914	9140	0.08	860
19		10.142	6759	0.06	939
20	NEO	10.724	513010	4.72	72197
21		11.451	3418344	31.47	341812

SAMPLE INFORMATION

Sample Name:	RIF NELF TDI Inc 150miM 1	Acquired By:	Saneesh
Sample Type:	Unknown	Date Acquired:	2017/01/11 01:55:32 PM
Vial:	16	Acq. Method Set:	HDI_RIF_25ODESRIF_NEO
Injection #:	1	Date Processed:	2017/01/11 03:05:04 PM
Injection Volume:	15.00 ul	Processing Method:	HDI_RIF_ W2996 254.0nm-1.2
Run Time:	12.5 Minutes	Channel Name:	W2996 254.0nm-1.2
Sample Set Name:	RIF NELF Inhb_TDI IC50 11_1_17	Proc. Chnl. Descr.:	W2996 PDA 254.0 nm at 1.2

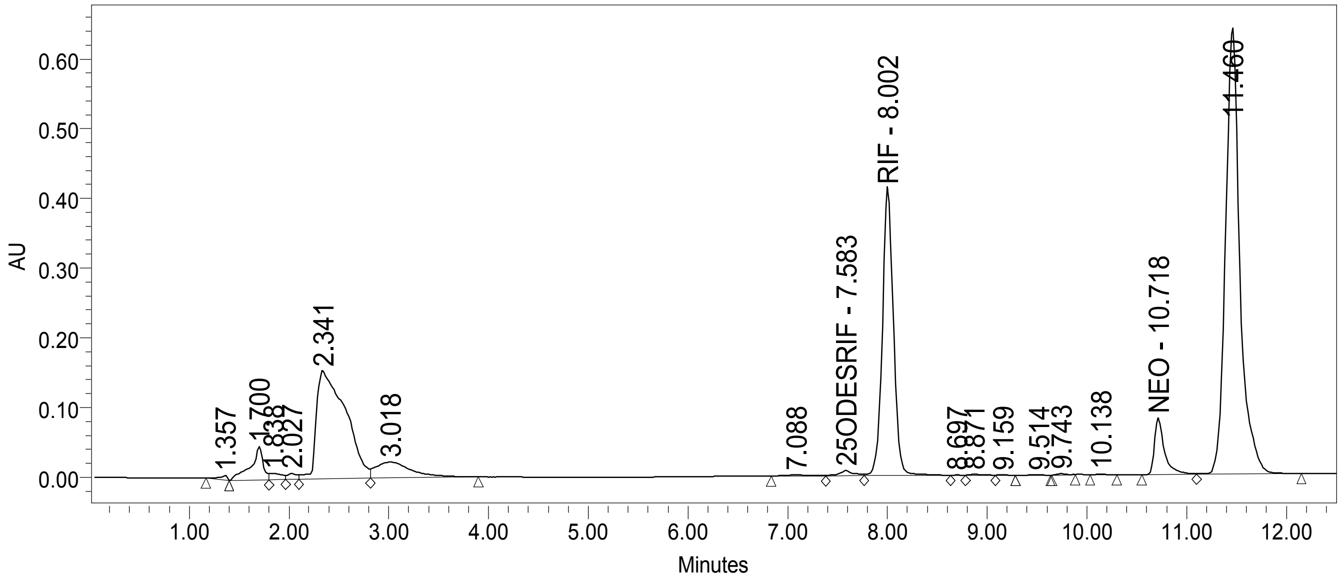

	Peak Name	RT	Area	% Area	Height
1		1.357	35862	0.24	6626
2		1.700	414831	2.83	47240
3		1.838	76723	0.52	8699
4		2.027	57453	0.39	8583
5		2.341	3154816	21.50	155449
6		3.018	577287	3.94	23138
7		7.088	27631	0.19	1302
8	25ODESRIF	7.583	72860	0.50	7456
9	RIF	8.002	3217375	21.93	412701
10		8.697	3883	0.03	584
11		8.871	14443	0.10	1919
12		9.159	4024	0.03	559

Reported by User: Saneesh (Saneesh) Project Name: HDI_Stellenbosch_2016

	Peak Name	RT	Area	% Area	Height
13		9.514	3001	0.02	321
14		9.743	8657	0.06	1585
15		10.138	7267	0.05	1246
16	NEO	10.718	575815	3.93	81098
17		11.460	6418394	43.75	642095

Reported by User: Saneesh (Saneesh) Project Name: HDI_Stellenbosch_2016

SAMPLE INFORMATION

Sample Name:	RIF NELF TDI Inc 150miM 2	Acquired By:	Saneesh
Sample Type:	Unknown	Date Acquired:	2017/01/11 02:11:25 PM
Vial:	17	Acq. Method Set:	HDI_RIF_25ODESRIF_NEO
Injection #:	1	Date Processed:	2017/01/11 03:05:04 PM
Injection Volume:	15.00 ul	Processing Method:	HDI_RIF_ W2996 254.0nm-1.2
Run Time:	12.5 Minutes	Channel Name:	W2996 254.0nm-1.2
Sample Set Name:	RIF NELF Inhb_TDI IC50 11_1_17	Proc. Chnl. Descr.:	W2996 PDA 254.0 nm at 1.2

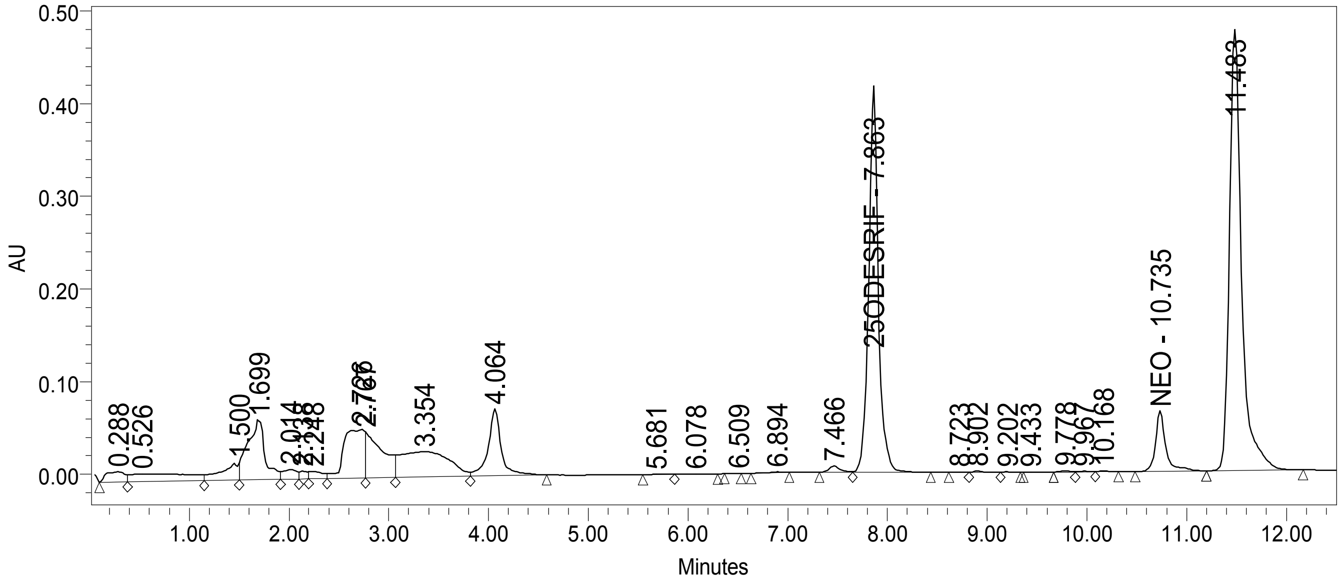

	Peak Name	RT	Area	% Area	Height
1		0.288	141919	1.18	10773
2		0.526	336308	2.79	8074
3		1.500	219295	1.82	18618
4		1.699	790204	6.56	63703
5		2.014	100878	0.84	10342
6		2.138	46877	0.39	8404
7		2.248	73884	0.61	7950
8		2.726	748300	6.22	52227
9		2.767	590846	4.91	49430
10		3.354	930817	7.73	27136
11		4.064	657783	5.46	71863
12		5.681	2123	0.02	153

	Peak Name	RT	Area	% Area	Height
13		6.078	3015	0.03	165
14		6.509	910	0.01	97
15		6.894	6837	0.06	908
16		7.466	54338	0.45	6843
17	25ODESRIF	7.863	2799980	23.26	413647
18	RIF	8.100			
19		8.723	1534	0.01	259
20		8.902	8463	0.07	1261
21		9.202	2333	0.02	394
22		9.433	1951	0.02	186
23		9.778	9052	0.08	1372
24		9.967	6637	0.06	711
25		10.168	6727	0.06	976
26	NEO	10.735	462675	3.84	64550
27		11.483	4035796	33.52	474353

SAMPLE INFORMATION

Sample Name:	RIF NELF TDI Inc 200miM 1	Acquired By:	Saneesh
Sample Type:	Unknown	Date Acquired:	2017/01/11 02:27:08 PM
Vial:	18	Acq. Method Set:	HDI_RIF_25ODESRIF_NEO
Injection #:	1	Date Processed:	2017/01/11 03:05:04 PM
Injection Volume:	15.00 ul	Processing Method:	HDI_RIF_ W2996 254.0nm-1.2
Run Time:	12.5 Minutes	Channel Name:	W2996 254.0nm-1.2
Sample Set Name:	RIF NELF Inhb_TDI IC50 11_1_17	Proc. Chnl. Descr.:	W2996 PDA 254.0 nm at 1.2

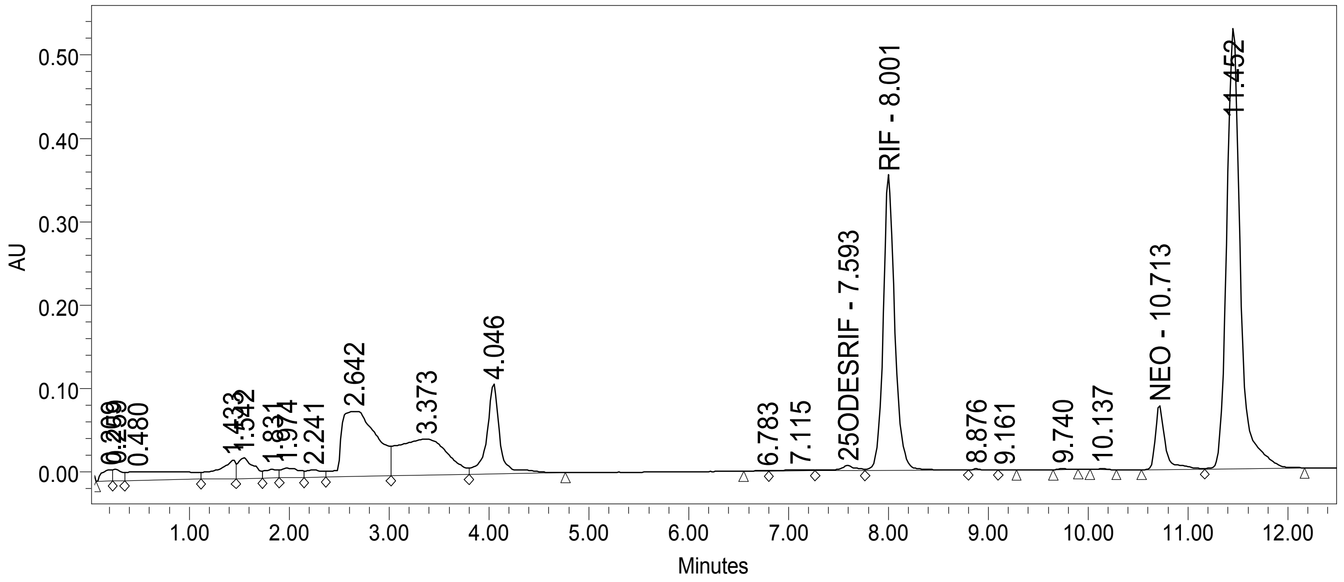

	Peak Name	RT	Area	% Area	Height
1		0.209	95014	0.66	13191
2		0.259	83878	0.58	13854
3		0.480	440247	3.05	10446
4		1.433	272708	1.89	21882
5		1.542	275161	1.90	24734
6		1.831	93315	0.65	10188
7		1.974	146719	1.02	11311
8		2.241	99004	0.69	8750
9		2.642	1907303	13.20	78006
10		3.373	1489674	10.31	42937
11		4.046	971102	6.72	107599
12		6.783	4176	0.03	527

Reported by User: Saneesh (Saneesh) Project Name: HDI_Stellenbosch_2016

	Peak Name	RT	Area	% Area	Height
13		7.115	27723	0.19	1599
14	25ODESRIF	7.593	72309	0.50	6261
15	RIF	8.001	2872826	19.89	353098
16		8.876	13695	0.09	1988
17		9.161	3048	0.02	505
18		9.740	7165	0.05	1290
19		10.137	6335	0.04	1069
20	NEO	10.713	524519	3.63	75875
21		11.452	5040894	34.89	526915

Reported by User: Saneesh (Saneesh)

Project Name: HDI_Stellenbosch_2016

SAMPLE INFORMATION

Sample Name:	RIF NELF TDI Inc 200miM 2	Acquired By:	Saneesh
Sample Type:	Unknown	Date Acquired:	2017/01/11 02:42:53 PM
Vial:	19	Acq. Method Set:	HDI_RIF_25ODESRIF_NEO
Injection #:	1	Date Processed:	2017/01/11 03:05:04 PM
Injection Volume:	15.00 ul	Processing Method:	HDI_RIF_ W2996 254.0nm-1.2
Run Time:	12.5 Minutes	Channel Name:	W2996 254.0nm-1.2
Sample Set Name:	RIF NELF Inhb_TDI IC50 11_1_17	Proc. Chnl. Descr.:	W2996 PDA 254.0 nm at 1.2

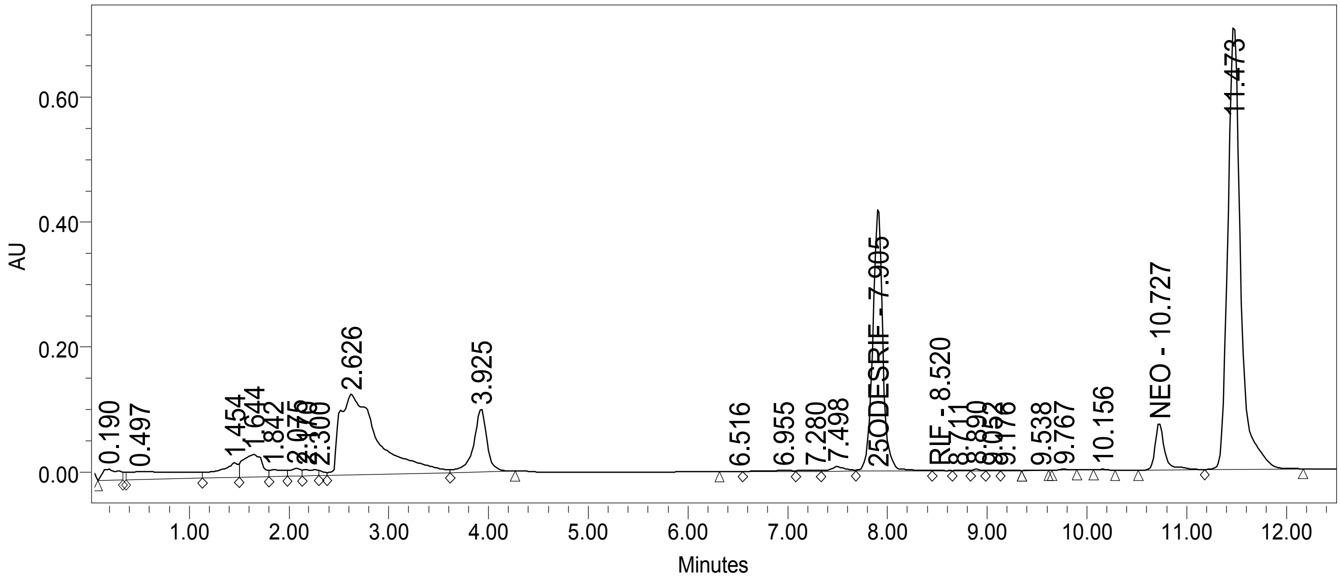

	Peak Name	RT	Area	% Area	Height
1		0.190	195931	1.21	17264
2		0.497	501986	3.10	12134
3		1.454	309170	1.91	23008
4		1.644	481008	2.97	35846
5		1.842	109098	0.67	10373
6		2.075	92336	0.57	11976
7		2.170	89067	0.55	9682
8		2.300	28275	0.17	7688
9		2.626	3487562	21.51	128690
10		3.925	937527	5.78	100286
11		6.516	3328	0.02	334
12		6.955	32384	0.20	1904

Reported by User: Saneesh (Saneesh) Project Name: HDI_Stellenbosch_2016

	Peak Name	RT	Area	% Area	Height
13		7.280	19551	0.12	1366
14		7.498	77422	0.48	7789
15	25ODESRIF	7.905	2904426	17.91	419062
16	RIF	8.520	6572	0.04	692
17		8.711	5962	0.04	660
18		8.890	12965	0.08	2492
19		9.052	5079	0.03	691
20		9.176	4487	0.03	708
21		9.538	3391	0.02	326
22		9.767	8588	0.05	1605
23		10.156	6742	0.04	1226
24	NEO	10.727	505422	3.12	74143
25		11.473	6385603	39.38	709839

Reported by User: Saneesh (Saneesh)   Project Name: HDI_Stellenbosch_2016

SAMPLE INFORMATION

Sample Name:	RIF Inc Basil_EtOH BER1 0min 1	Acquired By:	Saneesh
Sample Type:	Unknown	Date Acquired:	2017/02/02 07:15:22 AM
Vial:	2	Acq. Method Set:	HDI_RIF_25ODESRIF_NEO
Injection #:	1	Date Processed:	2017/02/02 10:22:06 AM
Injection Volume:	15.00 ul	Processing Method:	HDI_RIF_ W2996 254.0nm-1.2
Run Time:	11.5 Minutes	Channel Name:	W2996 254.0nm-1.2
Sample Set Name:	RIF_Basil EtOH AssaySam 2_1_17	Proc. Chnl. Descr.:	W2996 PDA 254.0 nm at 1.2

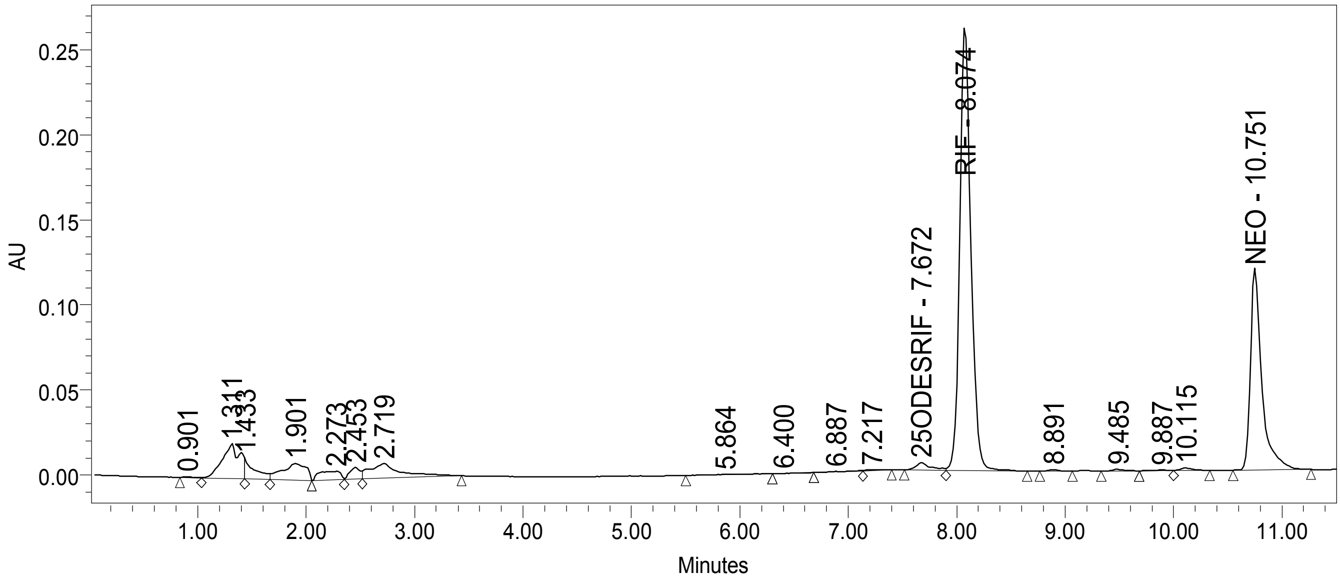

	Peak Name	RT	Area	% Area	Height
1		0.901	3005	0.08	430
2		1.311	233507	6.59	20444
3		1.433	76350	2.15	12320
4		1.901	148697	4.19	9695
5		2.273	69276	1.95	4999
6		2.453	45557	1.29	6795
7		2.719	176062	4.97	8631
8		5.864	5000	0.14	172
9		6.400	1624	0.05	143
10		6.887	3976	0.11	257
11		7.217	4855	0.14	469
12	25ODESRIF	7.672	41847	1.18	4336
13	RIF	8.074	1847445	52.12	261073
14		8.891	5648	0.16	902

Reported by User: Saneesh (Saneesh) Project Name: HDI_Stellenbosch_2016

	Peak Name	RT	Area	% Area	Height
15		9.485	6992	0.20	906
16		9.887	5740	0.16	530
17		10.115	11439	0.32	1518
18	NEO	10.751	857687	24.20	117890

SAMPLE INFORMATION

Sample Name:	RIF Inc Basil_EtOH BER2 0min 2	Acquired By:	Saneesh
Sample Type:	Unknown	Date Acquired:	2017/02/02 07:30:07 AM
Vial:	3	Acq. Method Set:	HDI_RIF_25ODESRIF_NEO
Injection #:	1	Date Processed:	2017/02/02 10:22:06 AM
Injection Volume:	15.00 ul	Processing Method:	HDI_RIF_ W2996 254.0nm-1.2
Run Time:	11.5 Minutes	Channel Name:	W2996 254.0nm-1.2
Sample Set Name:	RIF_Basil EtOH AssaySam 2_1_17	Proc. Chnl. Descr.:	W2996 PDA 254.0 nm at 1.2

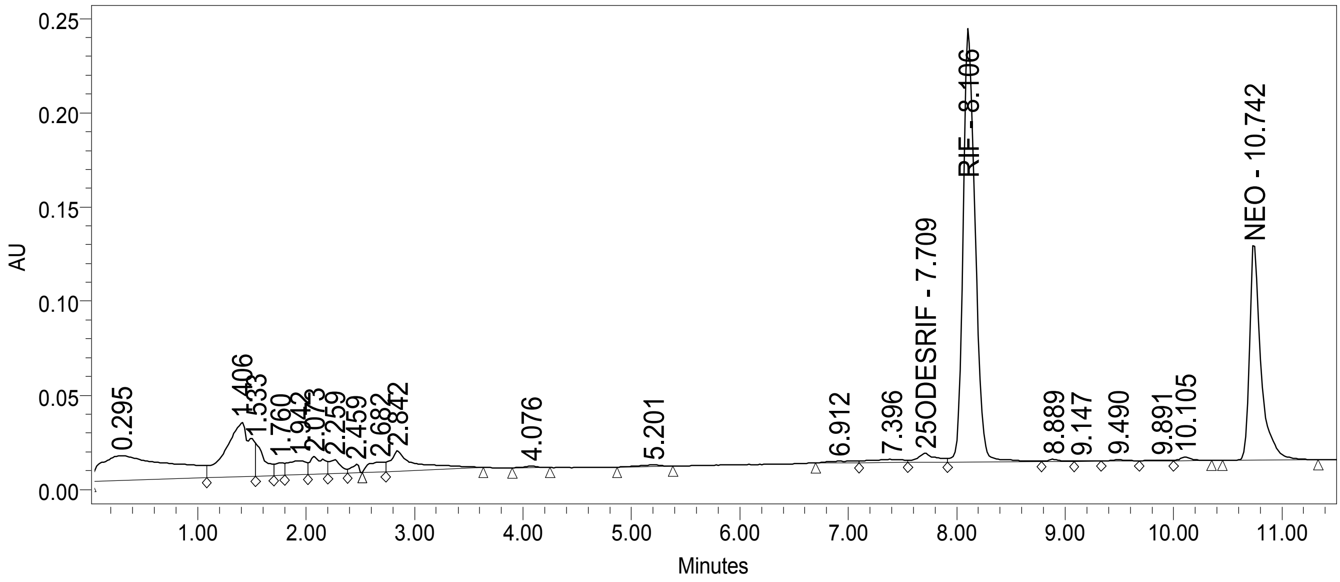

	Peak Name	RT	Area	% Area	Height
1		0.295	585500	13.14	13006
2		1.406	454231	10.19	29078
3		1.533	99719	2.24	17577
4		1.760	38664	0.87	6730
5		1.942	88653	1.99	7340
6		2.073	83329	1.87	9450
7		2.259	56116	1.26	7353
8		2.459	22796	0.51	4788
9		2.682	55575	1.25	5224
10		2.842	170830	3.83	10731
11		4.076	6928	0.16	909
12		5.201	12376	0.28	861

Reported by User: Saneesh (Saneesh)   Project Name: HDI_Stellenbosch_2016

	Peak Name	RT	Area	% Area	Height
13		6.912	16888	0.38	899
14		7.396	38225	0.86	1756
15	25ODESRIF	7.709	59066	1.33	4893
16	RIF	8.106	1790013	40.16	231231
17		8.889	9280	0.21	1214
18		9.147	3884	0.09	346
19		9.490	8476	0.19	968
20		9.891	5982	0.13	481
21		10.105	14929	0.33	1841
22	NEO	10.742	835904	18.75	115179

SAMPLE INFORMATION

Sample Name:	RIF Inc Basil_EtOH BER3 0min3	Acquired By:	Saneesh
Sample Type:	Unknown	Date Acquired:	2017/02/02 07:44:52 AM
Vial:	4	Acq. Method Set:	HDI_RIF_25ODESRIF_NEO
Injection #:	1	Date Processed:	2017/02/02 10:22:06 AM
Injection Volume:	15.00 ul	Processing Method:	HDI_RIF_ W2996 254.0nm-1.2
Run Time:	11.5 Minutes	Channel Name:	W2996 254.0nm-1.2
Sample Set Name:	RIF_Basil EtOH AssaySam 2_1_17	Proc. Chnl. Descr.:	W2996 PDA 254.0 nm at 1.2

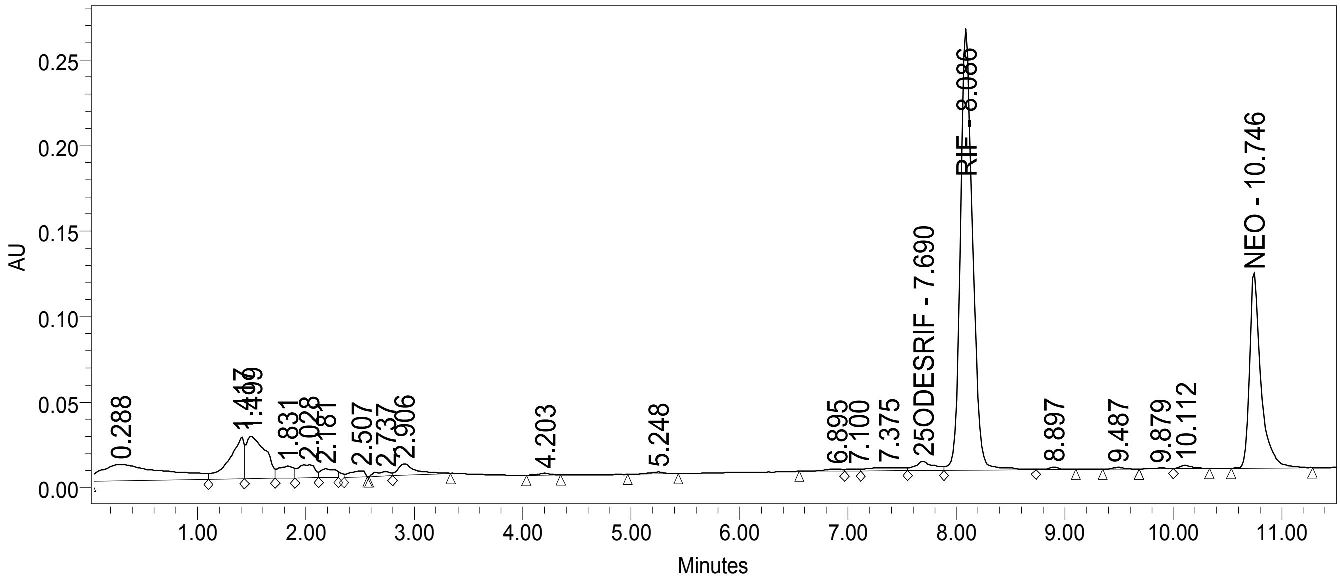

	Peak Name	RT	Area	% Area	Height
1		0.288	387148	9.05	9461
2		1.417	225000	5.26	24379
3		1.499	305756	7.15	24819
4		1.831	68647	1.60	6975
5		2.028	83219	1.95	7528
6		2.181	49744	1.16	5183
7		2.507	36382	0.85	3822
8		2.737	28116	0.66	2665
9		2.906	77149	1.80	6779
10		4.203	8468	0.20	1142
11		5.248	12904	0.30	1257
12		6.895	14244	0.33	1234

Reported by User: Saneesh (Saneesh) Project Name: HDI_Stellenbosch_2016

	Peak Name	RT	Area	% Area	Height
13		7.100	9364	0.22	1101
14		7.375	39779	0.93	1676
15	25ODESRIF	7.690	60495	1.41	5332
16	RIF	8.086	2011166	47.01	257228
17		8.897	9539	0.22	1374
18		9.487	7391	0.17	950
19		9.879	5955	0.14	512
20		10.112	14636	0.34	1855
21	NEO	10.746	822953	19.24	113634

SAMPLE INFORMATION

Sample Name:	RIF Inc Basil_EtOH BER4 2min 1	Acquired By:	Saneesh
Sample Type:	Unknown	Date Acquired:	2017/02/02 07:59:37 AM
Vial:	5	Acq. Method Set:	HDI_RIF_25ODESRIF_NEO
Injection #:	1	Date Processed:	2017/02/02 10:22:06 AM
Injection Volume:	15.00 ul	Processing Method:	HDI_RIF_ W2996 254.0nm-1.2
Run Time:	11.5 Minutes	Channel Name:	W2996 254.0nm-1.2
Sample Set Name:	RIF_Basil EtOH AssaySam 2_1_17	Proc. Chnl. Descr.:	W2996 PDA 254.0 nm at 1.2

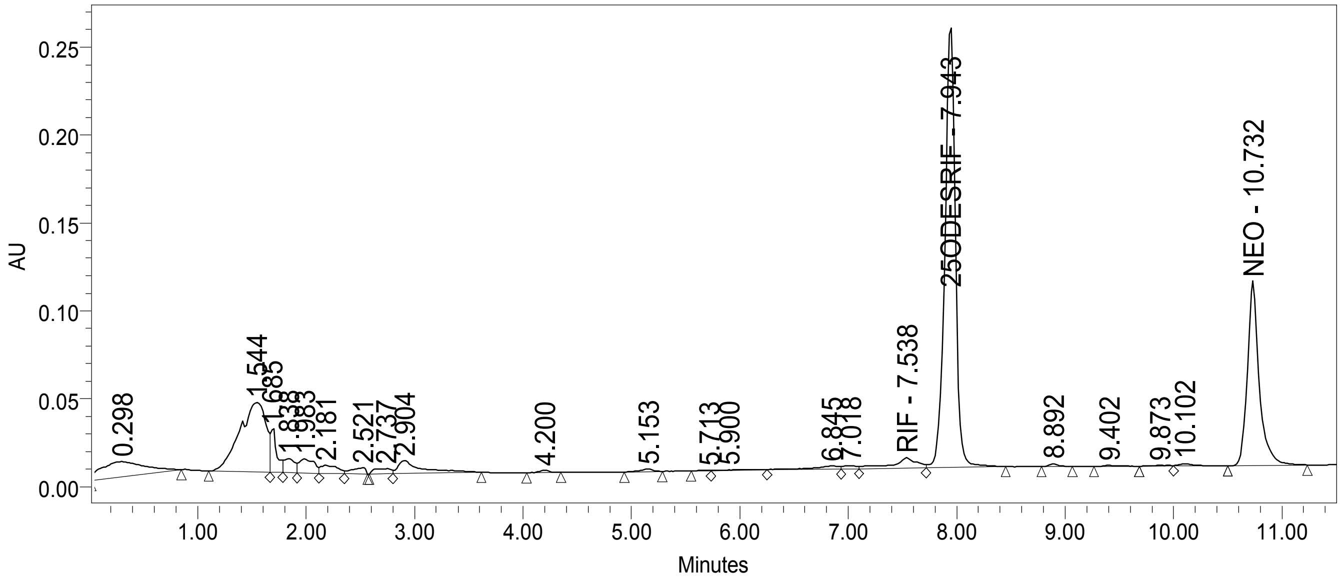

	Peak Name	RT	Area	% Area	Height
1		0.298	241057	5.96	8580
2		1.544	666781	16.50	39447
3		1.685	100390	2.48	24384
4		1.838	55234	1.37	7799
5		1.983	76359	1.89	7859
6		2.181	49352	1.22	4583
7		2.521	32842	0.81	3884
8		2.737	31691	0.78	3017
9		2.904	108471	2.68	7238
10		4.200	9528	0.24	1326
11		5.153	11684	0.29	1435
12		5.713	1637	0.04	214

Reported by User: Saneesh (Saneesh) Project Name: HDI_Stellenbosch_2016

	Peak Name	RT	Area	% Area	Height
13		5.900	8278	0.20	365
14		6.845	30457	0.75	1698
15		7.018	15460	0.38	1767
16	RIF	7.538	90391	2.24	5820
17	25ODESRIF	7.943	1685619	41.70	251416
18		8.892	9621	0.24	1640
19		9.402	6144	0.15	647
20		9.873	6224	0.15	524
21		10.102	12039	0.30	1383
22	NEO	10.732	793004	19.62	104410

SAMPLE INFORMATION

Sample Name:	RIF Inc Basil_EtOH BER5 2min 2	Acquired By:	Saneesh
Sample Type:	Unknown	Date Acquired:	2017/02/02 08:14:20 AM
Vial:	6	Acq. Method Set:	HDI_RIF_25ODESRIF_NEO
Injection #:	1	Date Processed:	2017/02/02 10:22:06 AM
Injection Volume:	15.00 ul	Processing Method:	HDI_RIF_ W2996 254.0nm-1.2
Run Time:	11.5 Minutes	Channel Name:	W2996 254.0nm-1.2
Sample Set Name:	RIF_Basil EtOH AssaySam 2_1_17	Proc. Chnl. Descr.:	W2996 PDA 254.0 nm at 1.2

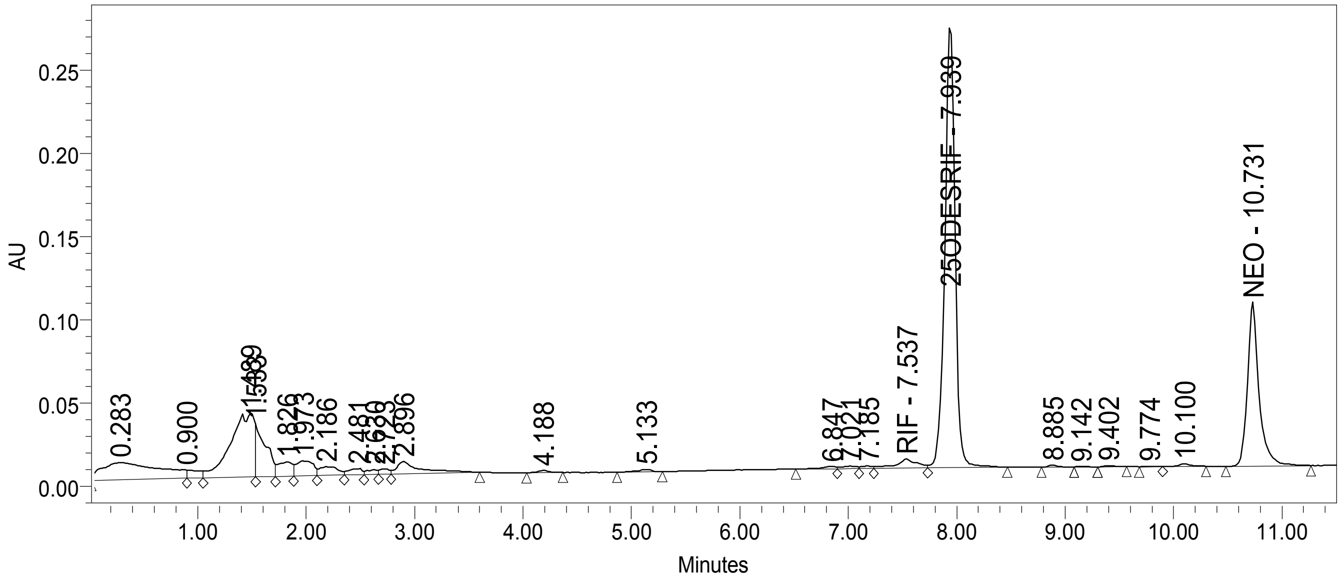

	Peak Name	RT	Area	% Area	Height
1		0.283	380925	8.95	10285
2		0.900	40190	0.94	4930
3		1.489	584310	13.74	38199
4		1.533	206292	4.85	31830
5		1.826	77584	1.82	8521
6		1.973	99579	2.34	8962
7		2.186	62153	1.46	5226
8		2.481	32256	0.76	3692
9		2.630	20062	0.47	2893
10		2.723	19254	0.45	3115
11		2.896	111982	2.63	7260
12		4.188	9787	0.23	1259

Reported by User: Saneesh (Saneesh)   Project Name: HDI_Stellenbosch_2016

	Peak Name	RT	Area	% Area	Height
13		5.133	13044	0.31	1368
14		6.847	14261	0.34	1408
15		7.021	14967	0.35	1497
16		7.185	9951	0.23	1420
17	RIF	7.537	72087	1.69	5465
18	25ODESRIF	7.939	1732993	40.74	264443
19		8.885	7444	0.17	1175
20		9.142	2312	0.05	323
21		9.402	4381	0.10	612
22		9.774	2199	0.05	305
23		10.100	12036	0.28	1418
24	NEO	10.731	724113	17.02	97987

Reported by User: Saneesh (Saneesh) Project Name: HDI_Stellenbosch_2016

SAMPLE INFORMATION

Sample Name:	RIF Inc Basil_EtOH BER6 2min 3	Acquired By:	Saneesh
Sample Type:	Unknown	Date Acquired:	2017/02/02 08:29:05 AM
Vial:	7	Acq. Method Set:	HDI_RIF_25ODESRIF_NEO
Injection #:	1	Date Processed:	2017/02/02 10:22:06 AM
Injection Volume:	15.00 ul	Processing Method:	HDI_RIF_ W2996 254.0nm-1.2
Run Time:	11.5 Minutes	Channel Name:	W2996 254.0nm-1.2
Sample Set Name:	RIF_Basil EtOH AssaySam 2_1_17	Proc. Chnl. Descr.:	W2996 PDA 254.0 nm at 1.2

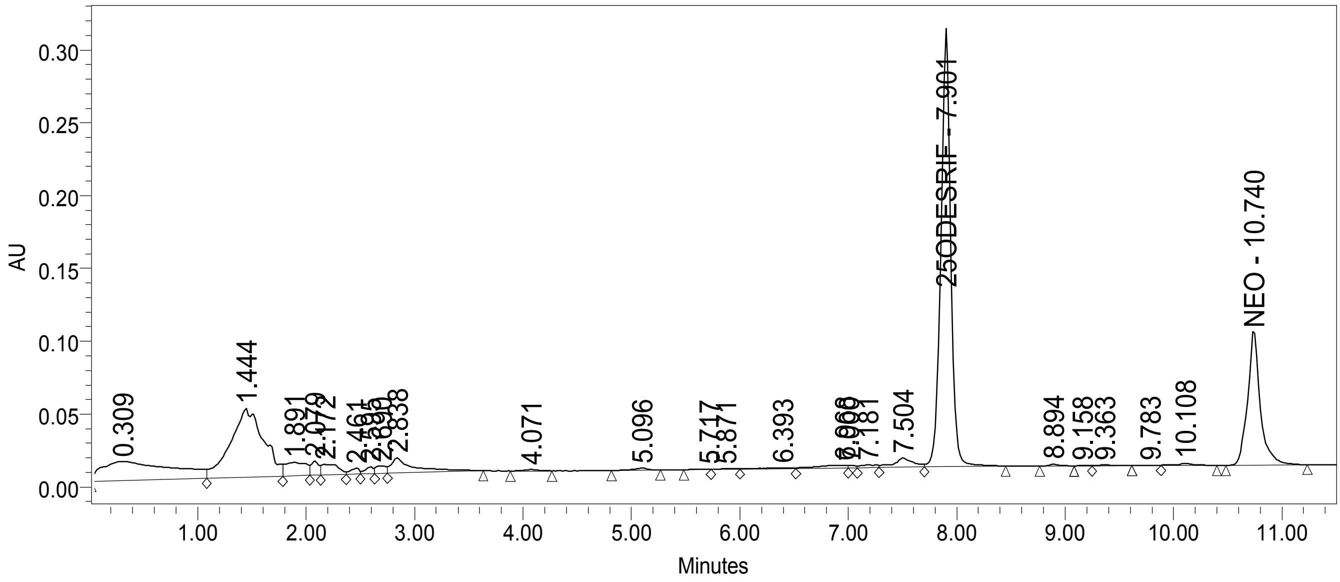

	Peak Name	RT	Area	% Area	Height
1		0.309	572878	11.70	12892
2		1.444	983440	20.09	46016
3		1.891	126400	2.58	9230
4		2.079	48479	0.99	9575
5		2.172	77666	1.59	7292
6		2.461	23235	0.47	4311
7		2.595	25867	0.53	4362
8		2.690	33211	0.68	5184
9		2.838	152021	3.11	10605
10		4.071	9363	0.19	1121
11		5.096	13917	0.28	1456
12		5.717	2738	0.06	342

	Peak Name	RT	Area	% Area	Height
13		5.871	4534	0.09	340
14		6.393	11290	0.23	487
15		6.968	34226	0.70	1754
16		7.000	6896	0.14	1591
17		7.181	18270	0.37	1682
18		7.504	77249	1.58	6448
19	25ODESRIF	7.901	1892136	38.66	298261
20	RIF	8.100			
21		8.894	8414	0.17	1376
22		9.158	2813	0.06	479
23		9.363	7522	0.15	611
24		9.783	3132	0.06	337
25		10.108	16432	0.34	1630
26	NEO	10.740	742596	15.17	91567

SAMPLE INFORMATION

Sample Name:	RIF Inc Basil_EtOH BER7 5min 1	Acquired By:	Saneesh
Sample Type:	Unknown	Date Acquired:	2017/02/02 08:43:50 AM
Vial:	8	Acq. Method Set:	HDI_RIF_25ODESRIF_NEO
Injection #:	1	Date Processed:	2017/02/02 10:22:06 AM
Injection Volume:	15.00 ul	Processing Method:	HDI_RIF_ W2996 254.0nm-1.2
Run Time:	11.5 Minutes	Channel Name:	W2996 254.0nm-1.2
Sample Set Name:	RIF_Basil EtOH AssaySam 2_1_17	Proc. Chnl. Descr.:	W2996 PDA 254.0 nm at 1.2

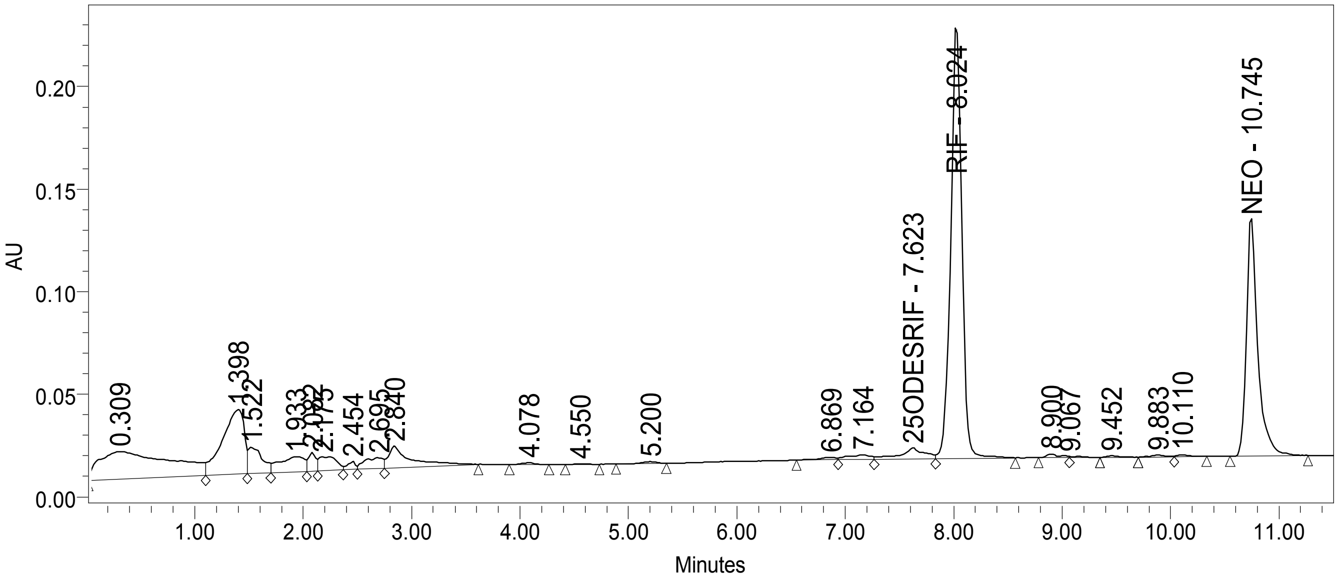

	Peak Name	RT	Area	% Area	Height
1		0.309	605289	14.48	13357
2		1.398	439203	10.50	31610
3		1.522	118187	2.83	12808
4		1.933	121246	2.90	7412
5		2.082	44359	1.06	8909
6		2.175	75859	1.81	6827
7		2.454	21308	0.51	3797
8		2.695	64146	1.53	5275
9		2.840	157396	3.76	10773
10		4.078	9052	0.22	1137
11		4.550	4384	0.10	547
12		5.200	10492	0.25	966

Reported by User: Saneesh (Saneesh) Project Name: HDI_Stellenbosch_2016

	Peak Name	RT	Area	% Area	Height
13		6.869	14083	0.34	1211
14		7.164	34134	0.82	2418
15	25ODESRIF	7.623	84104	2.01	5448
16	RIF	8.024	1495252	35.76	210716
17		8.900	14451	0.35	1773
18		9.067	4962	0.12	590
19		9.452	6997	0.17	806
20		9.883	9890	0.24	998
21		10.110	7741	0.19	1071
22	NEO	10.745	839053	20.07	115738

Reported by User: Saneesh (Saneesh)   Project Name: HDI_Stellenbosch_2016

SAMPLE INFORMATION

Sample Name:	RIF Inc Basil_EtOH BER8 5min 2	Acquired By:	Saneesh
Sample Type:	Unknown	Date Acquired:	2017/02/02 08:58:35 AM
Vial:	9	Acq. Method Set:	HDI_RIF_25ODESRIF_NEO
Injection #:	1	Date Processed:	2017/02/02 10:22:06 AM
Injection Volume:	15.00 ul	Processing Method:	HDI_RIF_ W2996 254.0nm-1.2
Run Time:	11.5 Minutes	Channel Name:	W2996 254.0nm-1.2
Sample Set Name:	RIF_Basil EtOH AssaySam 2_1_17	Proc. Chnl. Descr.:	W2996 PDA 254.0 nm at 1.2

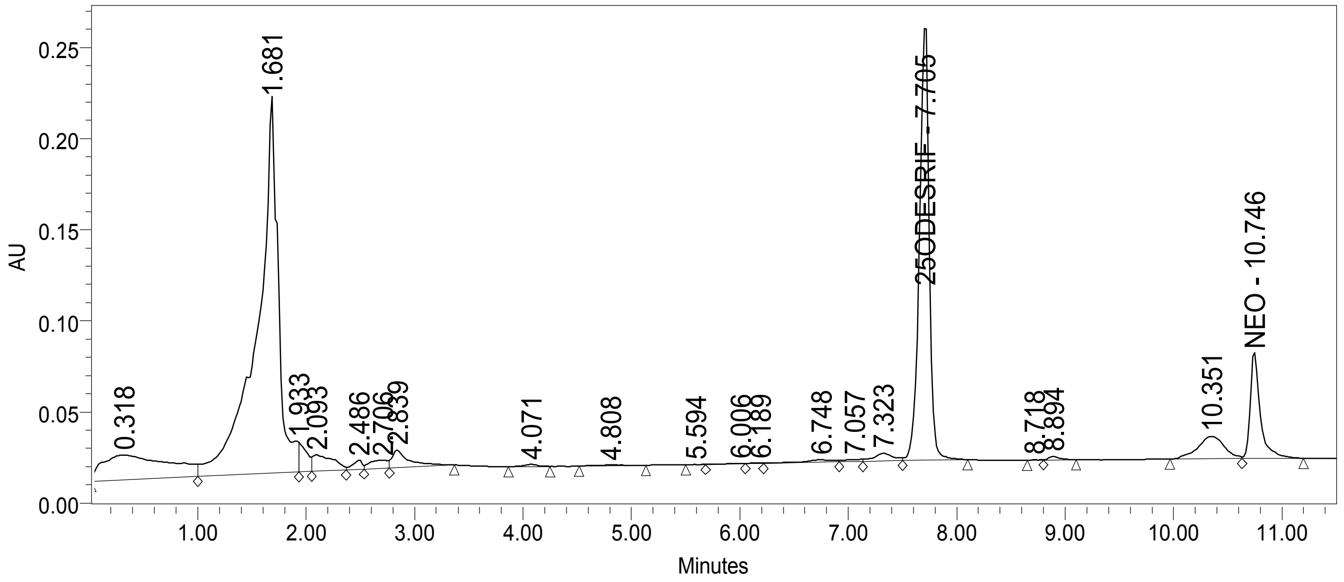

	Peak Name	RT	Area	% Area	Height
1		0.318	571992	9.60	13571
2		1.681	2769588	46.49	198373
3		1.933	81732	1.37	16261
4		2.093	117004	1.96	8874
5		2.486	29447	0.49	4702
6		2.706	52061	0.87	4310
7		2.839	105352	1.77	9766
8		4.071	9865	0.17	1225
9		4.808	8592	0.14	399
10		5.594	1057	0.02	131
11		6.006	3781	0.06	235
12		6.189	2594	0.04	296

	Peak Name	RT	Area	% Area	Height
13		6.748	26094	0.44	1501
14		7.057	14384	0.24	1256
15		7.323	54020	0.91	4385
16	25ODESRIF	7.705	1511397	25.37	238413
17	RIF	8.100			
18		8.718	1534	0.03	264
19		8.894	13735	0.23	1942
20		10.351	208528	3.50	12260
21	NEO	10.746	374281	6.28	57728

SAMPLE INFORMATION

Sample Name:	RIF Inc Basil_EtOH BER9 5min 3	Acquired By:	Saneesh
Sample Type:	Unknown	Date Acquired:	2017/02/02 09:13:20 AM
Vial:	10	Acq. Method Set:	HDI_RIF_25ODESRIF_NEO
Injection #:	1	Date Processed:	2017/02/02 10:22:06 AM
Injection Volume:	15.00 ul	Processing Method:	HDI_RIF_ W2996 254.0nm-1.2
Run Time:	11.5 Minutes	Channel Name:	W2996 254.0nm-1.2
Sample Set Name:	RIF_Basil EtOH AssaySam 2_1_17	Proc. Chnl. Descr.:	W2996 PDA 254.0 nm at 1.2

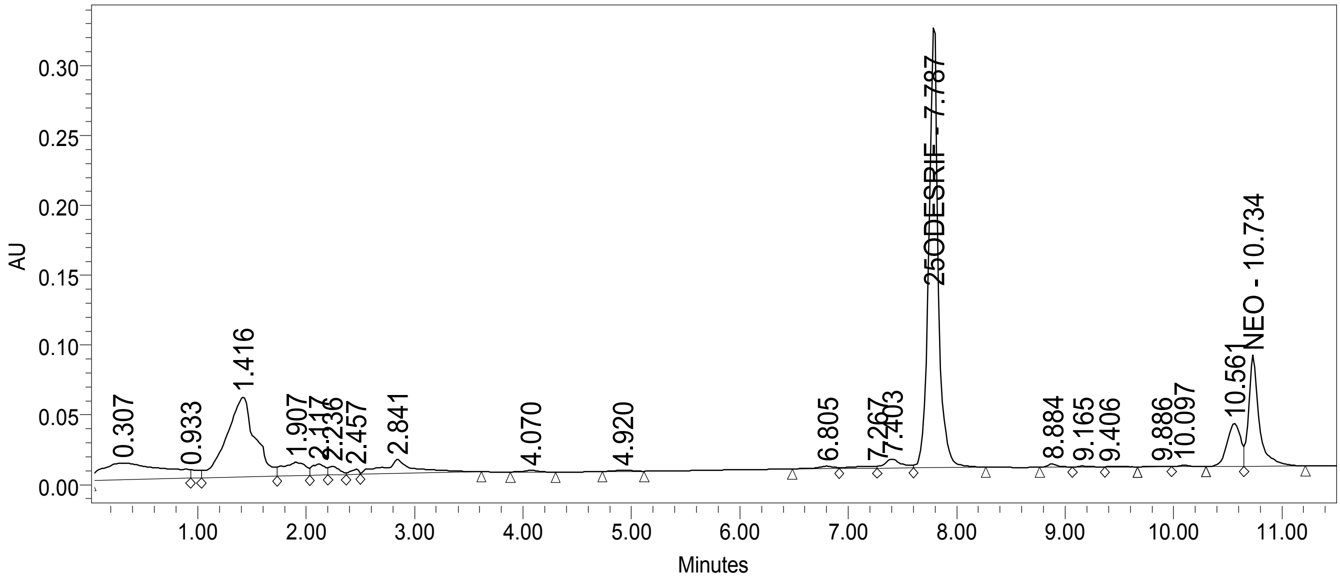

	Peak Name	RT	Area	% Area	Height
1		0.307	467263	9.51	11910
2		0.933	33412	0.68	5829
3		1.416	1046369	21.29	57173
4		1.907	140864	2.87	9776
5		2.117	67725	1.38	7966
6		2.236	43025	0.88	6233
7		2.457	20569	0.42	3895
8		2.841	202366	4.12	10153
9		4.070	10264	0.21	1237
10		4.920	9618	0.20	853
11		6.805	18631	0.38	1776
12		7.267	23458	0.48	1395

Reported by User: Saneesh (Saneesh) Project Name: HDI_Stellenbosch_2016

	Peak Name	RT	Area	% Area	Height
13		7.403	67188	1.37	6483
14	25ODESRIF	7.787	1932575	39.33	313920
15	RIF	8.100			
16		8.884	14212	0.29	2324
17		9.165	5987	0.12	701
18		9.406	3104	0.06	257
19		9.886	4984	0.10	380
20		10.097	7705	0.16	1067
21		10.561	267177	5.44	30692
22	NEO	10.734	527768	10.74	78725

SAMPLE INFORMATION

Sample Name:	RIF Inc Basil_EtOH BER10 10min 1	Acquired By:	Saneesh
Sample Type:	Unknown	Date Acquired:	2017/02/02 09:28:05 AM
Vial:	11	Acq. Method Set:	HDI_RIF_25ODESRIF_NEO
Injection #:	1	Date Processed:	2017/02/02 10:22:06 AM
Injection Volume:	15.00 ul	Processing Method:	HDI_RIF_ W2996 254.0nm-1.2
Run Time:	11.5 Minutes	Channel Name:	W2996 254.0nm-1.2
Sample Set Name:	RIF_Basil EtOH AssaySam 2_1_17	Proc. Chnl. Descr.:	W2996 PDA 254.0 nm at 1.2

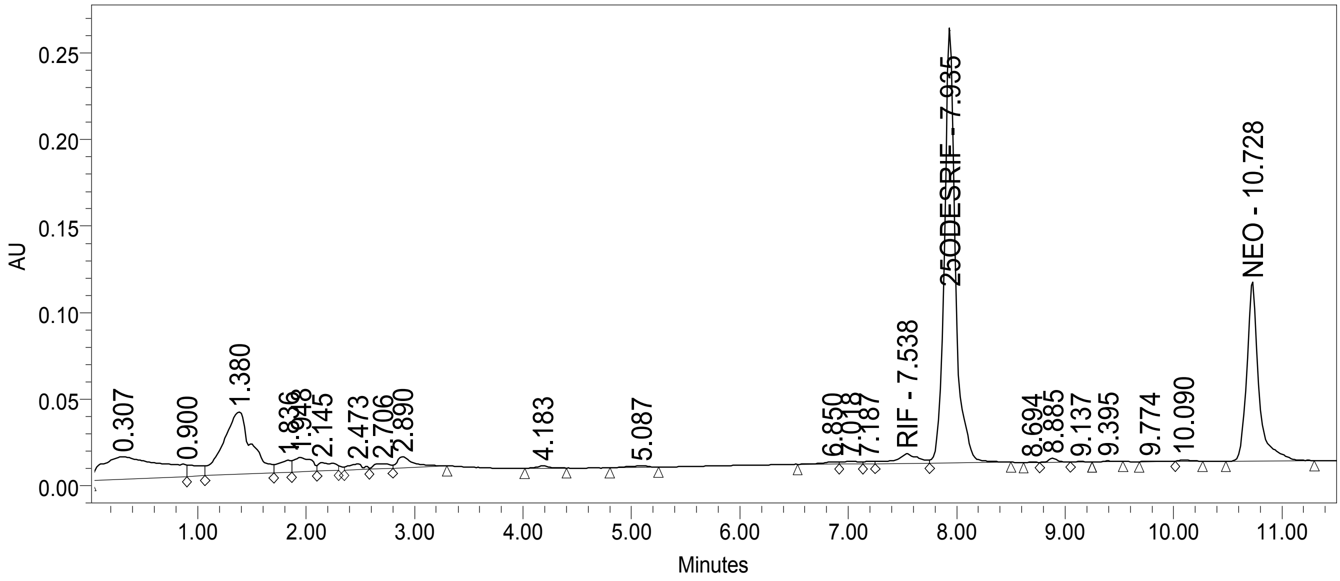

	Peak Name	RT	Area	% Area	Height
1		0.307	488110	11.46	12715
2		0.900	59894	1.41	6615
3		1.380	647781	15.21	36031
4		1.836	60281	1.42	6986
5		1.948	95294	2.24	8323
6		2.145	50407	1.18	5085
7		2.473	30702	0.72	3478
8		2.706	29498	0.69	2904
9		2.890	66297	1.56	6444
10		4.183	10264	0.24	1344
11		5.087	10334	0.24	832
12		6.850	15131	0.36	1363

Reported by User: Saneesh (Saneesh) Project Name: HDI_Stellenbosch_2016

	Peak Name	RT	Area	% Area	Height
13		7.018	16753	0.39	1605
14		7.187	8939	0.21	1412
15	RIF	7.538	78057	1.83	5609
16	25ODESRIF	7.935	1763849	41.41	249362
17		8.694	908	0.02	207
18		8.885	12420	0.29	2225
19		9.137	2770	0.07	472
20		9.395	3075	0.07	495
21		9.774	4699	0.11	336
22		10.090	5937	0.14	845
23	NEO	10.728	797698	18.73	103511

SAMPLE INFORMATION

Sample Name:	RIF Inc Basil_EtOH BER11 10min 2	Acquired By:	Saneesh
Sample Type:	Unknown	Date Acquired:	2017/02/02 09:42:49 AM
Vial:	12	Acq. Method Set:	HDI_RIF_25ODESRIF_NEO
Injection #:	1	Date Processed:	2017/02/02 10:22:06 AM
Injection Volume:	15.00 ul	Processing Method:	HDI_RIF_ W2996 254.0nm-1.2
Run Time:	11.5 Minutes	Channel Name:	W2996 254.0nm-1.2
Sample Set Name:	RIF_Basil EtOH AssaySam 2_1_17	Proc. Chnl. Descr.:	W2996 PDA 254.0 nm at 1.2

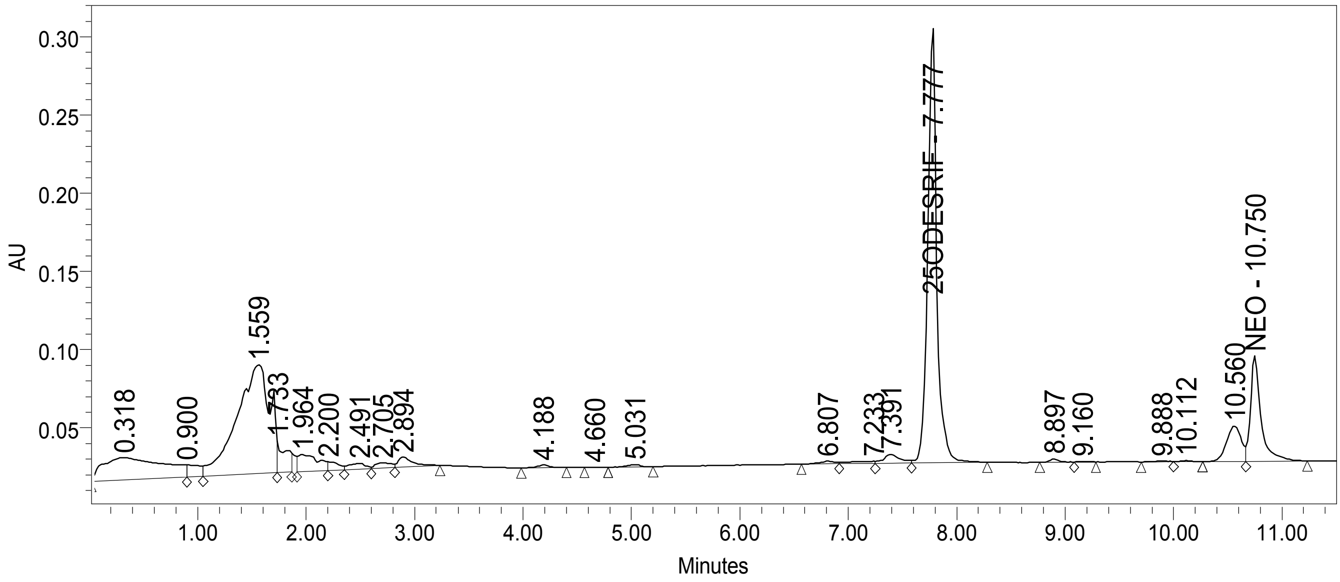

	Peak Name	RT	Area	% Area	Height
1		0.318	546993	11.10	14039
2		0.900	62580	1.27	7646
3		1.559	1469486	29.82	69829
4		1.733	110210	2.24	20144
5		1.964	141520	2.87	10808
6		2.200	36297	0.74	5399
7		2.491	38631	0.78	3731
8		2.705	33425	0.68	3284
9		2.894	57464	1.17	6258
10		4.188	13152	0.27	1642
11		4.660	1313	0.03	243
12		5.031	12799	0.26	1504

	Peak Name	RT	Area	% Area	Height
13		6.807	15597	0.32	1596
14		7.233	22162	0.45	1323
15		7.391	59352	1.20	5613
16	25ODESRIF	7.777	1615957	32.80	272821
17	RIF	8.100			
18		8.897	13112	0.27	2167
19		9.160	3125	0.06	460
20		9.888	5695	0.12	549
21		10.112	4928	0.10	699
22		10.560	217222	4.41	22694
23	NEO	10.750	446203	9.06	66516

SAMPLE INFORMATION

Sample Name:	RIF Inc Basil_EtOH BER12 10min 3	Acquired By:	Saneesh
Sample Type:	Unknown	Date Acquired:	2017/02/02 09:57:34 AM
Vial:	13	Acq. Method Set:	HDI_RIF_25ODESRIF_NEO
Injection #:	1	Date Processed:	2017/02/02 10:22:06 AM
Injection Volume:	15.00 ul	Processing Method:	HDI_RIF_ W2996 254.0nm-1.2
Run Time:	11.5 Minutes	Channel Name:	W2996 254.0nm-1.2
Sample Set Name:	RIF_Basil EtOH AssaySam 2_1_17	Proc. Chnl. Descr.:	W2996 PDA 254.0 nm at 1.2

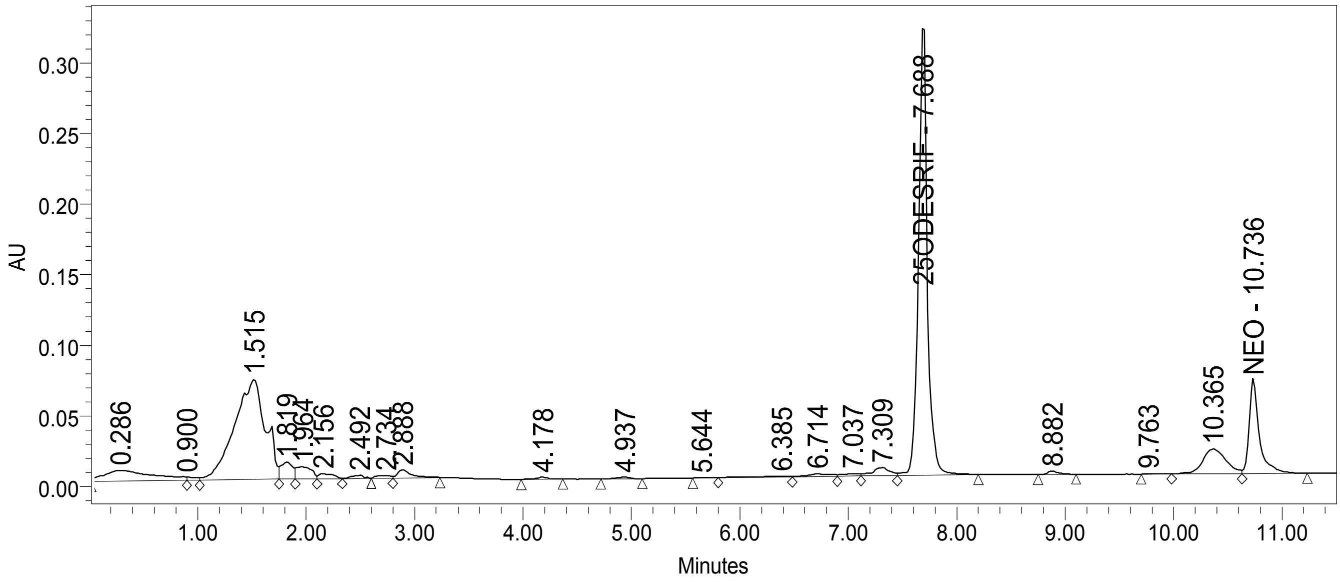

	Peak Name	RT	Area	% Area	Height
1		0.286	231725	5.04	7380
2		0.900	13209	0.29	2253
3		1.515	1402271	30.48	70945
4		1.819	91449	1.99	12010
5		1.964	84831	1.84	8584
6		2.156	34036	0.74	3810
7		2.492	18137	0.39	2289
8		2.734	17979	0.39	1851
9		2.888	49141	1.07	5632
10		4.178	11393	0.25	1534
11		4.937	11013	0.24	1178
12		5.644	1787	0.04	154

Reported by User: Saneesh (Saneesh)   Project Name: HDI_Stellenbosch_2016

	Peak Name	RT	Area	% Area	Height
13		6.385	11984	0.26	503
14		6.714	25753	0.56	1968
15		7.037	17914	0.39	1590
16		7.309	63457	1.38	5756
17	25ODESRIF	7.688	1805759	39.25	314139
18	RIF	8.100			
19		8.882	15085	0.33	2224
20		9.763	1517	0.03	190
21		10.365	260788	5.67	17588
22	NEO	10.736	431101	9.37	66564

Reported by User: Saneesh (Saneesh) Project Name: HDI_Stellenbosch_2016

SAMPLE INFORMATION

Sample Name:	RIF Inc Basil_EtOH BER14 15min 2	Acquired By:	Saneesh
Sample Type:	Unknown	Date Acquired:	2017/02/02 10:12:19 AM
Vial:	14	Acq. Method Set:	HDI_RIF_25ODESRIF_NEO
Injection #:	1	Date Processed:	2017/02/02 10:50:52 AM
Injection Volume:	15.00 ul	Processing Method:	HDI_RIF_ W2996 254.0nm-1.2
Run Time:	11.5 Minutes	Channel Name:	W2996 254.0nm-1.2
Sample Set Name:	RIF_Basil EtOH AssaySam 2_1_17	Proc. Chnl. Descr.:	W2996 PDA 254.0 nm at 1.2

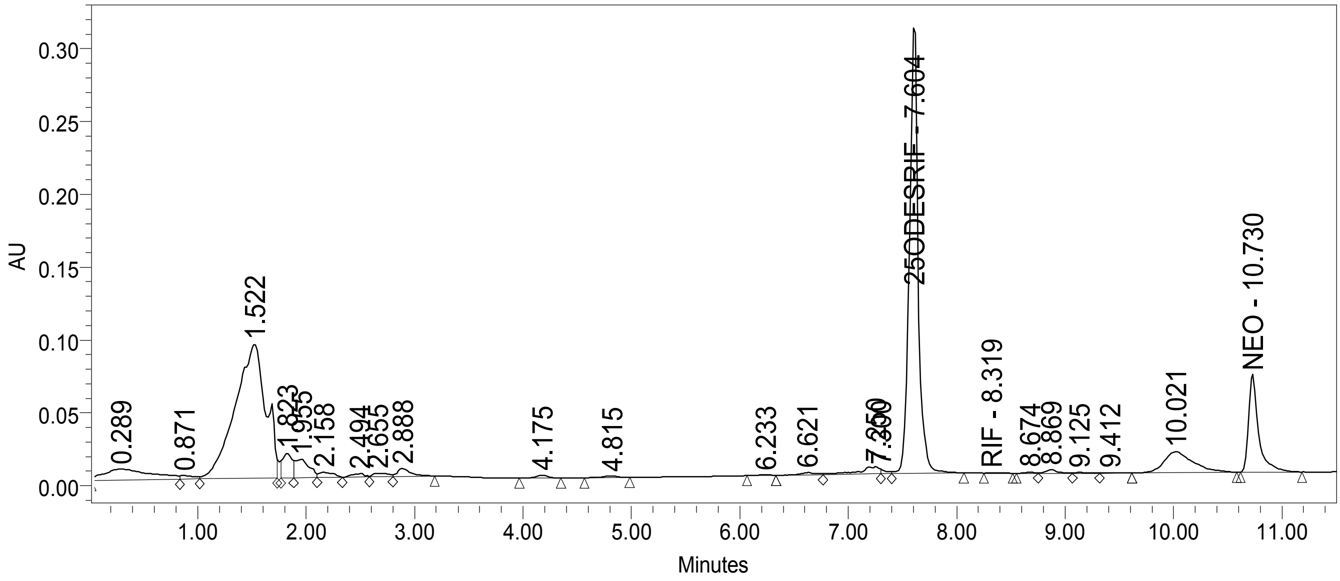

	Peak Name	RT	Area	% Area	Height
1		0.289	221407	4.47	7448
2		0.871	22612	0.46	2509
3		1.522	1802806	36.37	92546
4		1.823	101142	2.04	16787
5		1.955	117723	2.38	12499
6		2.158	32165	0.65	3532
7		2.494	17053	0.34	2212
8		2.655	22949	0.46	2445
9		2.888	44292	0.89	5417
10		4.175	15014	0.30	1995
11		4.815	13507	0.27	1159
12		6.233	3024	0.06	337

	Peak Name	RT	Area	% Area	Height
13		6.621	14573	0.29	1664
14		7.250	65673	1.32	4886
15		7.300	11657	0.24	3229
16	25ODESRIF	7.604	1712885	34.56	303143
17	RIF	8.319	2852	0.06	303
18		8.674	4424	0.09	734
19		8.869	17445	0.35	2326
20		9.125	4248	0.09	527
21		9.412	4120	0.08	307
22		10.021	274040	5.53	14481
23	NEO	10.730	431142	8.70	66795

Reported by User: Saneesh (Saneesh) Project Name: HDI_Stellenbosch_2016

SAMPLE INFORMATION

Sample Name:	RIF Inc Basil_EtOH BER17 20min 2	Acquired By:	Saneesh
Sample Type:	Unknown	Date Acquired:	2017/02/02 10:27:04 AM
Vial:	15	Acq. Method Set:	HDI_RIF_25ODESRIF_NEO
Injection #:	1	Date Processed:	2017/02/02 10:50:52 AM
Injection Volume:	15.00 ul	Processing Method:	HDI_RIF_ W2996 254.0nm-1.2
Run Time:	11.5 Minutes	Channel Name:	W2996 254.0nm-1.2
Sample Set Name:	RIF_Basil EtOH AssaySam 2_1_17	Proc. Chnl. Descr.:	W2996 PDA 254.0 nm at 1.2

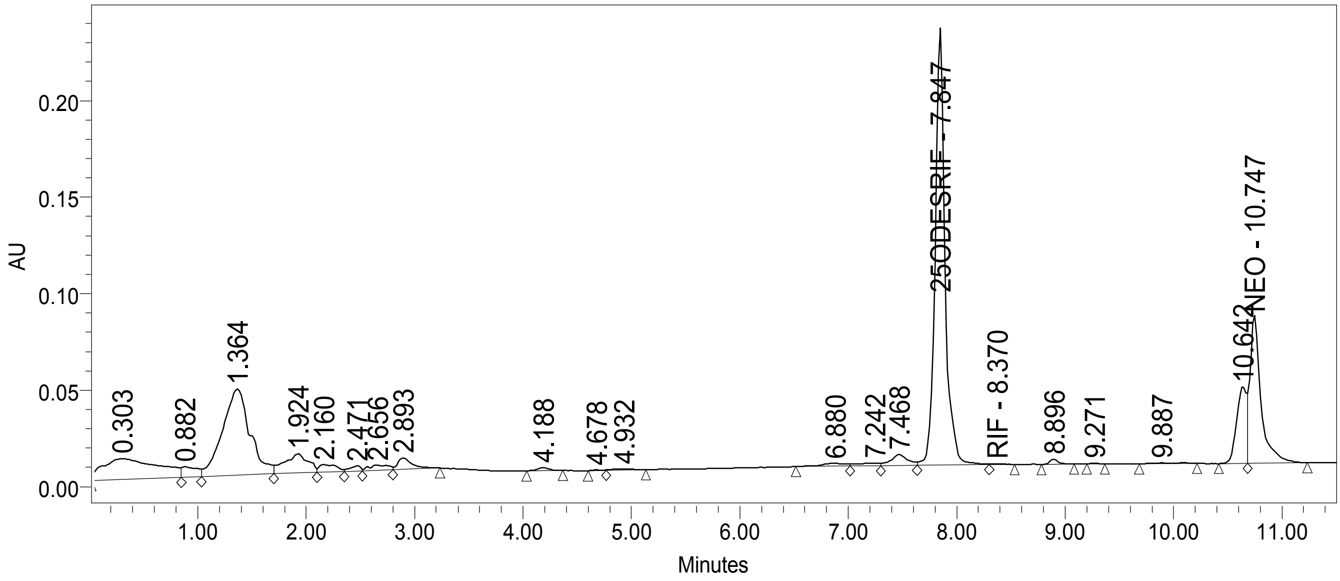

	Peak Name	RT	Area	% Area	Height
1		0.303	371364	9.58	10547
2		0.882	50812	1.31	5327
3		1.364	785610	20.26	44588
4		1.924	151202	3.90	10043
5		2.160	41688	1.08	3853
6		2.471	17446	0.45	2597
7		2.656	32580	0.84	2490
8		2.893	52704	1.36	5917
9		4.188	11743	0.30	1654
10		4.678	2321	0.06	319
11		4.932	9360	0.24	773
12		6.880	24129	0.62	1516

Reported by User: Saneesh (Saneesh) Project Name: HDI_Stellenbosch_2016

	Peak Name	RT	Area	% Area	Height
13		7.242	21563	0.56	1392
14		7.468	62749	1.62	5813
15	25ODESRIF	7.847	1420838	36.65	223266
16	RIF	8.370	3736	0.10	466
17		8.896	14266	0.37	2573
18		9.271	1930	0.05	345
19		9.887	9605	0.25	488
20		10.642	255749	6.60	39785
21	NEO	10.747	535838	13.82	75835

SAMPLE INFORMATION

Sample Name:	RIF Inc Basil_EtOH BER13 15min 1	Acquired By:	Saneesh
Sample Type:	Unknown	Date Acquired:	2017/02/06 09:10:24 AM
Vial:	2	Acq. Method Set:	HDI_RIF_25ODESRIF_NEO
Injection #:	1	Date Processed:	2017/02/08 06:42:20 AM
Injection Volume:	15.00 ul	Processing Method:	HDI_RIF_ W2996 254.0nm-1.2
Run Time:	11.5 Minutes	Channel Name:	W2996 254.0nm-1.2
Sample Set Name:	RIF_Basil EtOH AssaySM2 6_1_17	Proc. Chnl. Descr.:	W2996 PDA 254.0 nm at 1.2

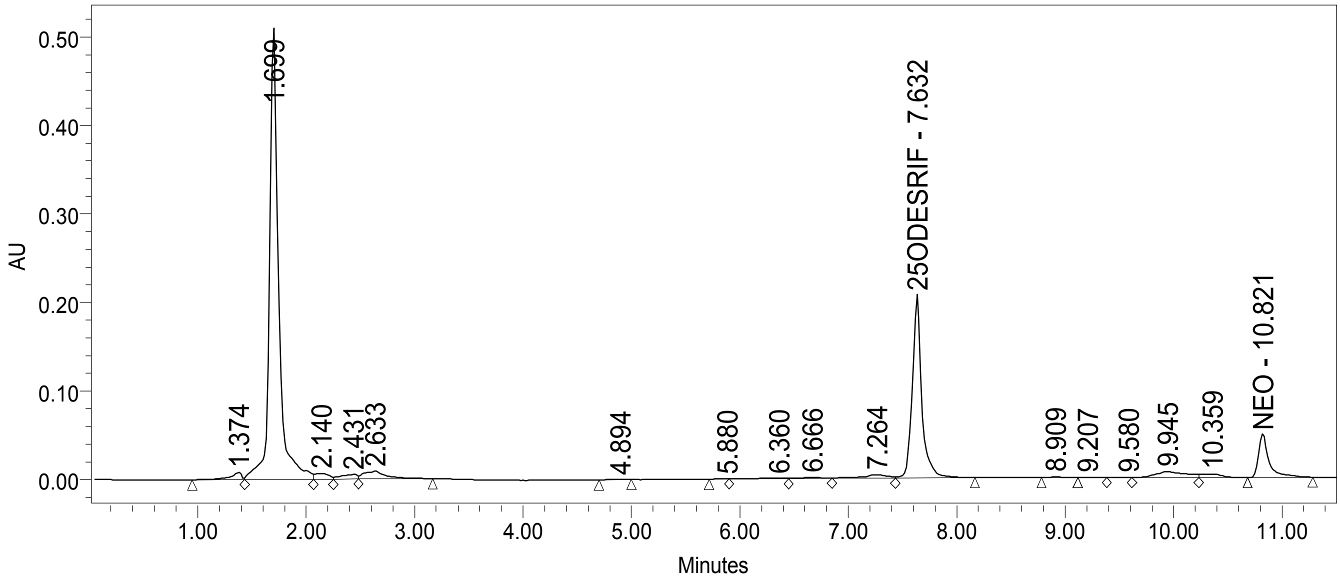

	Peak Name	RT	Area	% Area	Height
1		1.374	65608	1.26	8472
2		1.699	3006252	57.91	500455
3		2.140	58017	1.12	6453
4		2.431	54932	1.06	5329
5		2.633	128564	2.48	8778
6		4.894	5038	0.10	467
7		5.880	1991	0.04	244
8		6.360	15453	0.30	665
9		6.666	21868	0.42	1453
10		7.264	65841	1.27	3919
11	25ODESRIF	7.632	1253540	24.15	203317
12	RIF	8.100			
13		8.909	5051	0.10	777
14		9.207	1758	0.03	250

Reported by User: Saneesh (Saneesh) Project Name: HDI_Stellenbosch_2016

	Peak Name	RT	Area	% Area	Height
15		9.580	3187	0.06	296
16		9.945	132225	2.55	6826
17		10.359	48344	0.93	3684
18	NEO	10.821	323349	6.23	48692

SAMPLE INFORMATION

Sample Name:	RIF Inc Basil_EtOH BER15 15min 3	Acquired By:	Saneesh
Sample Type:	Unknown	Date Acquired:	2017/02/06 09:25:19 AM
Vial:	3	Acq. Method Set:	HDI_RIF_25ODESRIF_NEO
Injection #:	1	Date Processed:	2017/02/08 06:42:20 AM
Injection Volume:	15.00 ul	Processing Method:	HDI_RIF_ W2996 254.0nm-1.2
Run Time:	11.5 Minutes	Channel Name:	W2996 254.0nm-1.2
Sample Set Name:	RIF_Basil EtOH AssaySM2 6_1_17	Proc. Chnl. Descr.:	W2996 PDA 254.0 nm at 1.2

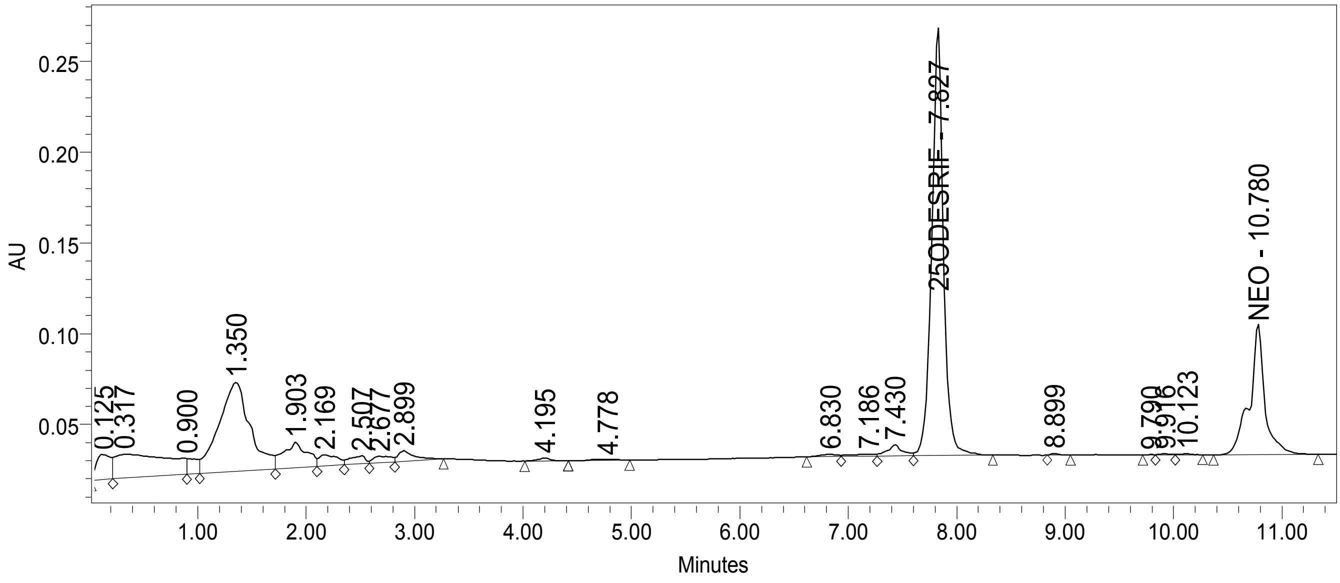

	Peak Name	RT	Area	% Area	Height
1		0.125	129004	2.78	13878
2		0.317	452019	9.76	13221
3		0.900	57939	1.25	8686
4		1.350	986299	21.29	49001
5		1.903	212113	4.58	14023
6		2.169	71590	1.55	6218
7		2.507	45058	0.97	4385
8		2.677	39036	0.84	3382
9		2.899	59167	1.28	5572
10		4.195	13696	0.30	1663
11		4.778	9993	0.22	602
12		6.830	14128	0.30	1410

	Peak Name	RT	Area	% Area	Height
13		7.186	19637	0.42	1237
14		7.430	58660	1.27	6157
15	25ODESRIF	7.827	1755094	37.88	234990
16	RIF	8.100			
17		8.899	5494	0.12	1014
18		9.790	2126	0.05	429
19		9.916	5527	0.12	720
20		10.123	4545	0.10	573
21	NEO	10.780	691818	14.93	71251

SAMPLE INFORMATION

Sample Name:	RIF Inc Basil_EtOH BER16 20min 1	Acquired By:	Saneesh
Sample Type:	Unknown	Date Acquired:	2017/02/06 09:40:02 AM
Vial:	4	Acq. Method Set:	HDI_RIF_25ODESRIF_NEO
Injection #:	1	Date Processed:	2017/02/08 06:42:20 AM
Injection Volume:	15.00 ul	Processing Method:	HDI_RIF_ W2996 254.0nm-1.2
Run Time:	11.5 Minutes	Channel Name:	W2996 254.0nm-1.2
Sample Set Name:	RIF_Basil EtOH AssaySM2 6_1_17	Proc. Chnl. Descr.:	W2996 PDA 254.0 nm at 1.2

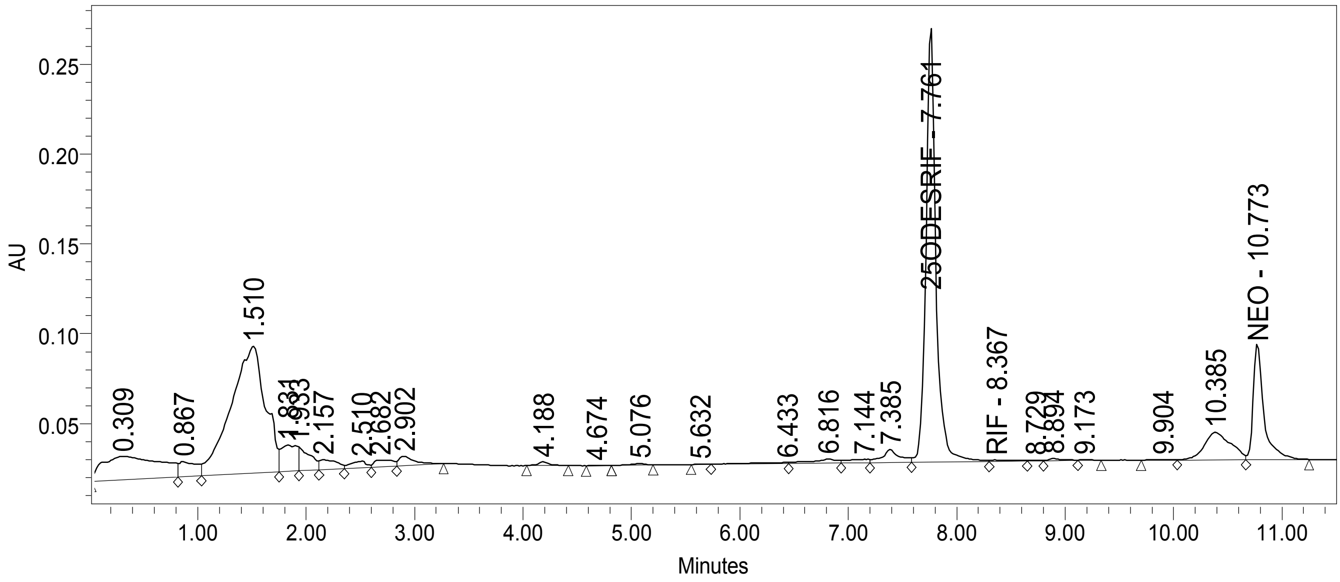

	Peak Name	RT	Area	% Area	Height
1		0.309	473389	9.39	12847
2		0.867	94575	1.88	8370
3		1.510	1564499	31.03	70926
4		1.831	151302	3.00	14613
5		1.933	95311	1.89	13146
6		2.157	59112	1.17	5633
7		2.510	37590	0.75	3866
8		2.682	43004	0.85	3813
9		2.902	51217	1.02	5142
10		4.188	15890	0.32	2034
11		4.674	1608	0.03	260
12		5.076	8435	0.17	926

Reported by User: Saneesh (Saneesh) Project Name: HDI_Stellenbosch_2016

	Peak Name	RT	Area	% Area	Height
13		5.632	1257	0.02	194
14		6.433	15233	0.30	592
15		6.816	36063	0.72	2341
16		7.144	27210	0.54	1893
17		7.385	79837	1.58	7135
18	25ODESRIF	7.761	1559913	30.94	238767
19	RIF	8.367	10725	0.21	791
20		8.729	3346	0.07	446
21		8.894	11998	0.24	1230
22		9.173	4246	0.08	606
23		9.904	4325	0.09	364
24		10.385	260200	5.16	15163
25	NEO	10.773	431408	8.56	63868

SAMPLE INFORMATION

Sample Name:	RIF Inc Basil_EtOH BER18 20min 3	Acquired By:	Saneesh
Sample Type:	Unknown	Date Acquired:	2017/02/06 09:54:47 AM
Vial:	5	Acq. Method Set:	HDI_RIF_25ODESRIF_NEO
Injection #:	1	Date Processed:	2017/02/08 06:42:20 AM
Injection Volume:	15.00 ul	Processing Method:	HDI_RIF_ W2996 254.0nm-1.2
Run Time:	11.5 Minutes	Channel Name:	W2996 254.0nm-1.2
Sample Set Name:	RIF_Basil EtOH AssaySM2 6_1_17	Proc. Chnl. Descr.:	W2996 PDA 254.0 nm at 1.2

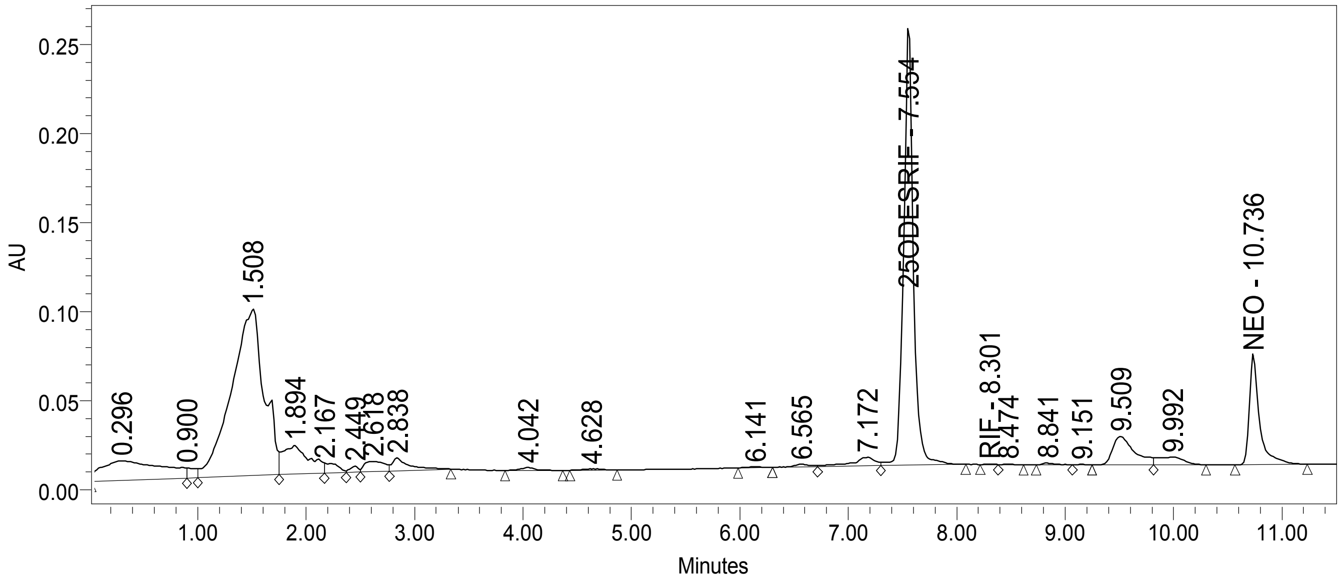

	Peak Name	RT	Area	% Area	Height
1		0.296	417188	7.93	10880
2		0.900	31473	0.60	5582
3		1.508	1949743	37.08	94395
4		1.894	277515	5.28	16149
5		2.167	47451	0.90	5871
6		2.449	18123	0.34	3467
7		2.618	74882	1.42	5717
8		2.838	77778	1.48	7176
9		4.042	15807	0.30	1685
10		4.628	10249	0.19	761
11		6.141	3965	0.08	487
12		6.565	13923	0.26	1510

	Peak Name	RT	Area	% Area	Height
13		7.172	72998	1.39	4820
14	25ODESRIF	7.554	1534507	29.18	243867
15	RIF	8.301	2294	0.04	401
16		8.474	2684	0.05	455
17		8.841	7936	0.15	981
18		9.151	1821	0.03	352
19		9.509	222293	4.23	16154
20		9.992	74914	1.42	4267
21	NEO	10.736	400450	7.62	61425

SAMPLE INFORMATION

Sample Name:	RIF Inc Basil_EtOH BER19 30min 1	Acquired By:	Saneesh
Sample Type:	Unknown	Date Acquired:	2017/02/06 10:09:32 AM
Vial:	6	Acq. Method Set:	HDI_RIF_25ODESRIF_NEO
Injection #:	1	Date Processed:	2017/02/08 06:42:20 AM
Injection Volume:	15.00 ul	Processing Method:	HDI_RIF_ W2996 254.0nm-1.2
Run Time:	11.5 Minutes	Channel Name:	W2996 254.0nm-1.2
Sample Set Name:	RIF_Basil EtOH AssaySM2 6_1_17	Proc. Chnl. Descr.:	W2996 PDA 254.0 nm at 1.2

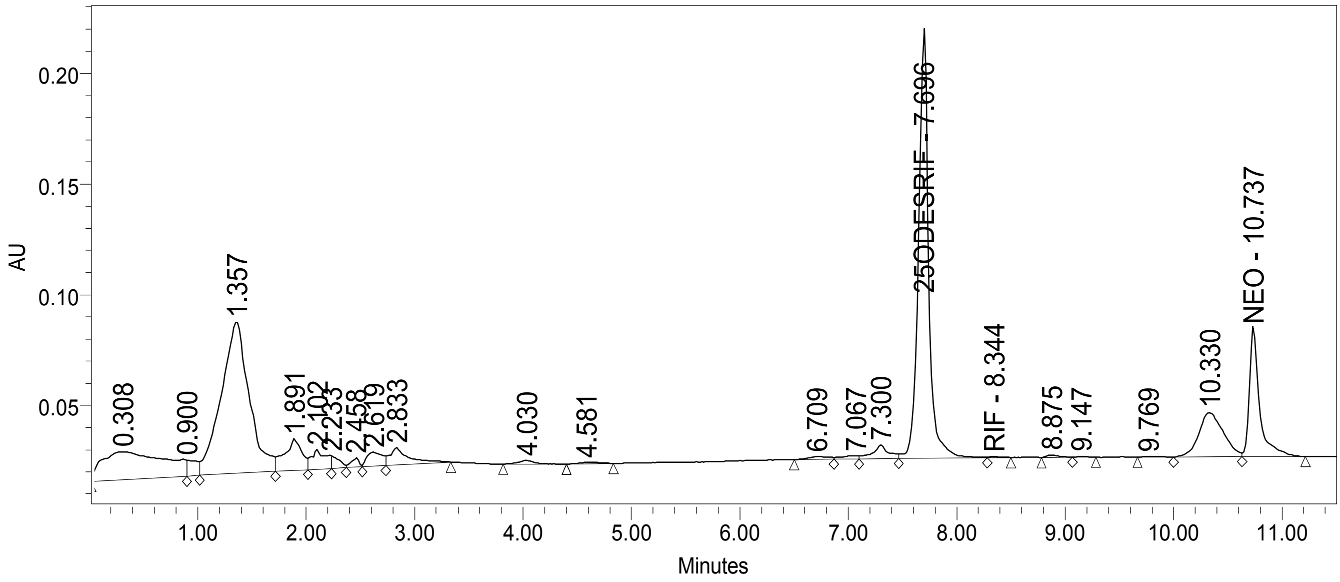

	Peak Name	RT	Area	% Area	Height
1		0.308	497270	11.45	12518
2		0.900	46589	1.07	7157
3		1.357	1236483	28.47	68517
4		1.891	158862	3.66	13990
5		2.102	80940	1.86	8461
6		2.233	27476	0.63	5511
7		2.458	21132	0.49	4154
8		2.619	62600	1.44	6294
9		2.833	93785	2.16	7643
10		4.030	14827	0.34	1482
11		4.581	9539	0.22	619
12		6.709	17271	0.40	1266

Reported by User: Saneesh (Saneesh) Project Name: HDI_Stellenbosch_2016

	Peak Name	RT	Area	% Area	Height
13		7.067	16321	0.38	1426
14		7.300	64648	1.49	6079
15	25ODESRIF	7.696	1281451	29.50	192778
16	RIF	8.344	3060	0.07	464
17		8.875	8775	0.20	1124
18		9.147	2874	0.07	437
19		9.769	4177	0.10	349
20		10.330	314556	7.24	19929
21	NEO	10.737	381190	8.78	58319

SAMPLE INFORMATION

Sample Name:	RIF Inc Basil_EtOH BER20 30min 2	Acquired By:	Saneesh
Sample Type:	Unknown	Date Acquired:	2017/02/06 10:24:17 AM
Vial:	7	Acq. Method Set:	HDI_RIF_25ODESRIF_NEO
Injection #:	1	Date Processed:	2017/02/08 06:42:20 AM
Injection Volume:	15.00 ul	Processing Method:	HDI_RIF_ W2996 254.0nm-1.2
Run Time:	11.5 Minutes	Channel Name:	W2996 254.0nm-1.2
Sample Set Name:	RIF_Basil EtOH AssaySM2 6_1_17	Proc. Chnl. Descr.:	W2996 PDA 254.0 nm at 1.2

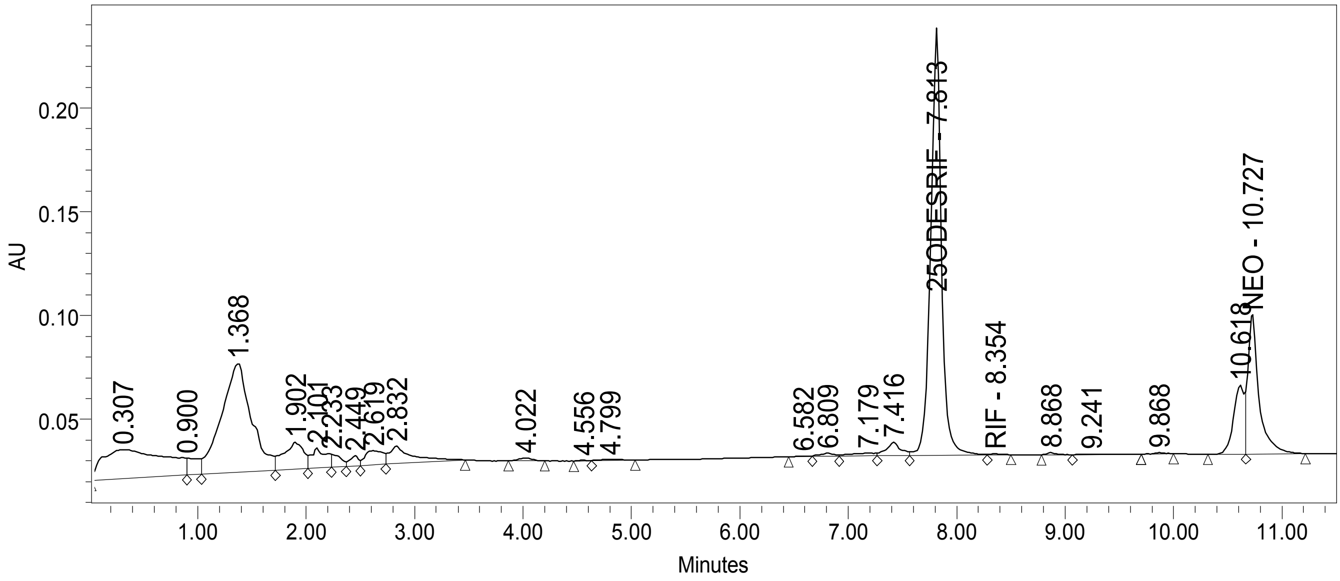

	Peak Name	RT	Area	% Area	Height
1		0.307	558950	12.59	13950
2		0.900	62007	1.40	8095
3		1.368	1010799	22.78	52375
4		1.902	166808	3.76	12860
5		2.101	91784	2.07	9385
6		2.233	35523	0.80	6301
7		2.449	28096	0.63	4861
8		2.619	78766	1.77	6850
9		2.832	127044	2.86	8506
10		4.022	12953	0.29	1488
11		4.556	1917	0.04	306
12		4.799	8054	0.18	540

Reported by User: Saneesh (Saneesh) Project Name: HDI_Stellenbosch_2016

	Peak Name	RT	Area	% Area	Height
13		6.582	2056	0.05	248
14		6.809	15074	0.34	1561
15		7.179	24011	0.54	1404
16		7.416	59657	1.34	6428
17	25ODESRIF	7.813	1422741	32.06	204490
18	RIF	8.354	3529	0.08	499
19		8.868	7229	0.16	1130
20		9.241	6751	0.15	333
21		9.868	4835	0.11	662
22		10.618	237560	5.35	32896
23	NEO	10.727	471882	10.63	67200

SAMPLE INFORMATION

Sample Name:	RIF Inc Basil_EtOH BER21 30min 3	Acquired By:	Saneesh
Sample Type:	Unknown	Date Acquired:	2017/02/06 10:39:02 AM
Vial:	8	Acq. Method Set:	HDI_RIF_25ODESRIF_NEO
Injection #:	1	Date Processed:	2017/02/08 06:42:20 AM
Injection Volume:	15.00 ul	Processing Method:	HDI_RIF_ W2996 254.0nm-1.2
Run Time:	11.5 Minutes	Channel Name:	W2996 254.0nm-1.2
Sample Set Name:	RIF_Basil EtOH AssaySM2 6_1_17	Proc. Chnl. Descr.:	W2996 PDA 254.0 nm at 1.2

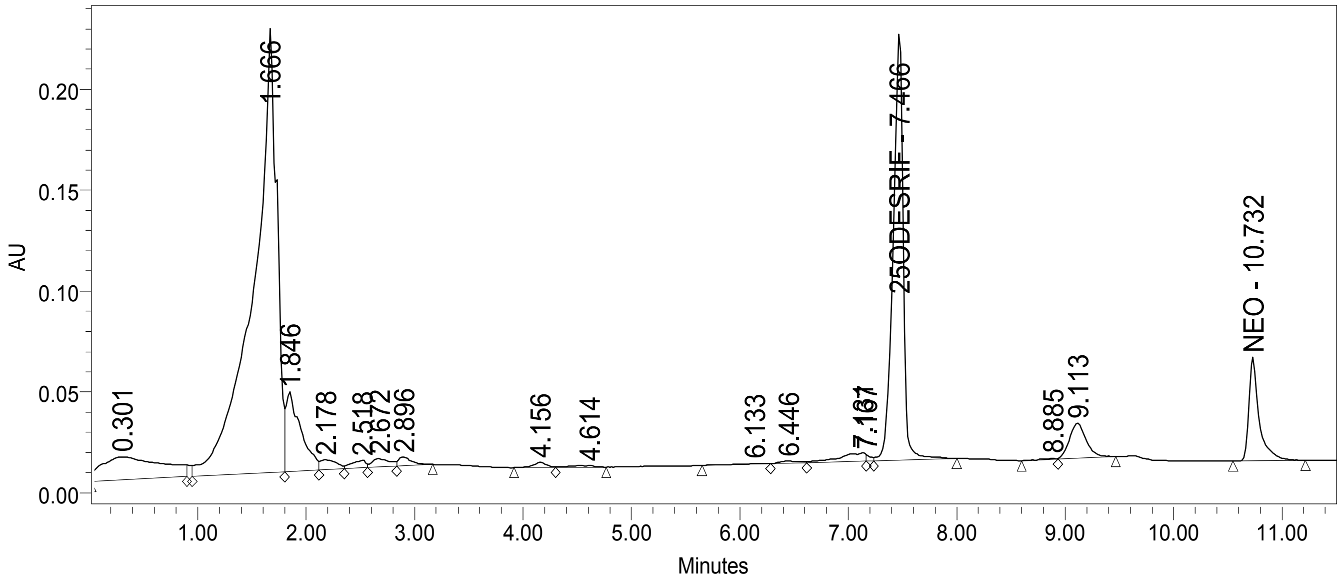

	Peak Name	RT	Area	% Area	Height
1		0.301	431933	6.87	11289
2		1.666	3271231	52.06	213430
3		1.846	383879	6.11	38734
4		2.178	51021	0.81	4903
5		2.518	34608	0.55	3728
6		2.672	46910	0.75	4152
7		2.896	36737	0.58	4348
8		4.156	21367	0.34	2579
9		4.614	13396	0.21	783
10		6.133	6255	0.10	265
11		6.446	12188	0.19	1039
12		7.131	68069	1.08	4145

Reported by User: Saneesh (Saneesh) Project Name: HDI_Stellenbosch_2016

	Peak Name	RT	Area	% Area	Height
13		7.167	8581	0.14	3226
14	25ODESRIF	7.466	1368801	21.78	211764
15	RIF	8.100			
16		8.885	5239	0.08	523
17		9.113	185804	2.96	17263
18	NEO	10.732	337744	5.37	50944

SAMPLE INFORMATION

Sample Name:	RIF Inc Basil_EtOH BER22 45min 1	Acquired By:	Saneesh
Sample Type:	Unknown	Date Acquired:	2017/02/06 10:53:46 AM
Vial:	9	Acq. Method Set:	HDI_RIF_25ODESRIF_NEO
Injection #:	1	Date Processed:	2017/02/08 06:42:20 AM
Injection Volume:	15.00 ul	Processing Method:	HDI_RIF_ W2996 254.0nm-1.2
Run Time:	11.5 Minutes	Channel Name:	W2996 254.0nm-1.2
Sample Set Name:	RIF_Basil EtOH AssaySM2 6_1_17	Proc. Chnl. Descr.:	W2996 PDA 254.0 nm at 1.2

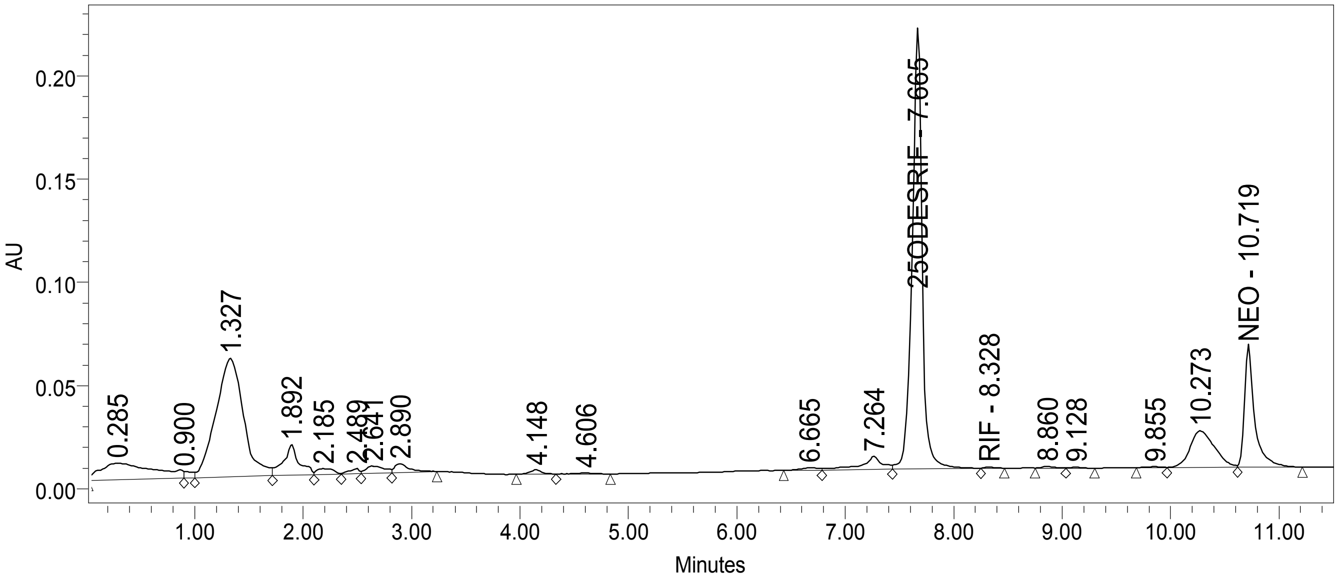

	Peak Name	RT	Area	% Area	Height
1		0.285	270619	7.18	7868
2		0.900	16914	0.45	3184
3		1.327	1016281	26.96	57406
4		1.892	149455	3.97	14753
5		2.185	29022	0.77	2820
6		2.489	14909	0.40	2255
7		2.641	40287	1.07	3427
8		2.890	39211	1.04	4256
9		4.148	17128	0.45	2170
10		4.606	8201	0.22	763
11		6.665	14625	0.39	1252
12		7.264	87849	2.33	6399

Reported by User: Saneesh (Saneesh) Project Name: HDI_Stellenbosch_2016

	Peak Name	RT	Area	% Area	Height
13	25ODESRIF	7.665	1382224	36.67	213044
14	RIF	8.328	3669	0.10	558
15		8.860	6704	0.18	887
16		9.128	3533	0.09	521
17		9.855	4882	0.13	642
18		10.273	280099	7.43	17916
19	NEO	10.719	383631	10.18	58849

SAMPLE INFORMATION

Sample Name:	RIF Inc Basil_EtOH BER23 45min 2	Acquired By:	Saneesh
Sample Type:	Unknown	Date Acquired:	2017/02/06 11:08:31 AM
Vial:	10	Acq. Method Set:	HDI_RIF_25ODESRIF_NEO
Injection #:	1	Date Processed:	2017/02/08 06:42:20 AM
Injection Volume:	15.00 ul	Processing Method:	HDI_RIF_ W2996 254.0nm-1.2
Run Time:	11.5 Minutes	Channel Name:	W2996 254.0nm-1.2
Sample Set Name:	RIF_Basil EtOH AssaySM2 6_1_17	Proc. Chnl. Descr.:	W2996 PDA 254.0 nm at 1.2

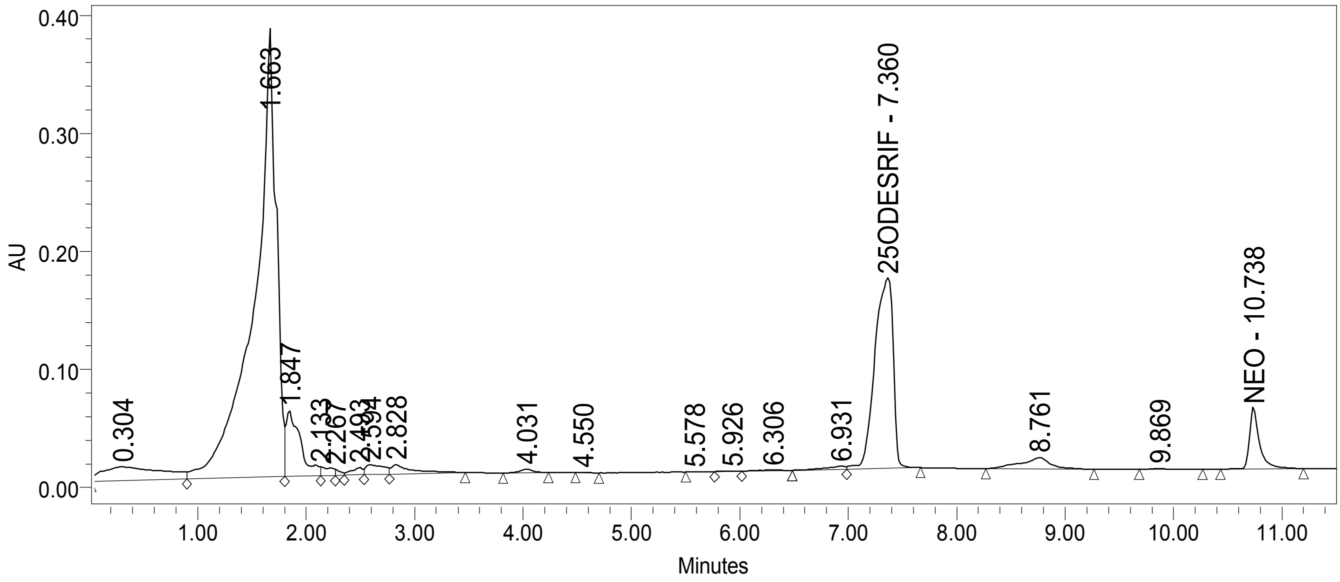

	Peak Name	RT	Area	% Area	Height
1		0.304	439855	4.86	11444
2		1.663	5275370	58.34	366089
3		1.847	528217	5.84	54429
4		2.133	49998	0.55	7410
5		2.267	17206	0.19	5065
6		2.493	45294	0.50	5976
7		2.594	95546	1.06	8271
8		2.828	105677	1.17	8056
9		4.031	24488	0.27	3020
10		4.550	2443	0.03	510
11		5.578	1439	0.02	152
12		5.926	1674	0.02	117

	Peak Name	RT	Area	% Area	Height
13		6.306	8947	0.10	564
14		6.931	35295	0.39	2650
15	25ODESRIF	7.360	1883061	20.83	162698
16	RIF	8.100			
17		8.761	187934	2.08	9740
18		9.869	8934	0.10	599
19	NEO	10.738	330500	3.66	51658

SAMPLE INFORMATION

Sample Name:	RIF Inc Basil_EtOH BER24 45min 3	Acquired By:	Saneesh
Sample Type:	Unknown	Date Acquired:	2017/02/06 11:23:26 AM
Vial:	11	Acq. Method Set:	HDI_RIF_25ODESRIF_NEO
Injection #:	1	Date Processed:	2017/02/08 06:42:20 AM
Injection Volume:	15.00 ul	Processing Method:	HDI_RIF_ W2996 254.0nm-1.2
Run Time:	11.5 Minutes	Channel Name:	W2996 254.0nm-1.2
Sample Set Name:	RIF_Basil EtOH AssaySM2 6_1_17	Proc. Chnl. Descr.:	W2996 PDA 254.0 nm at 1.2

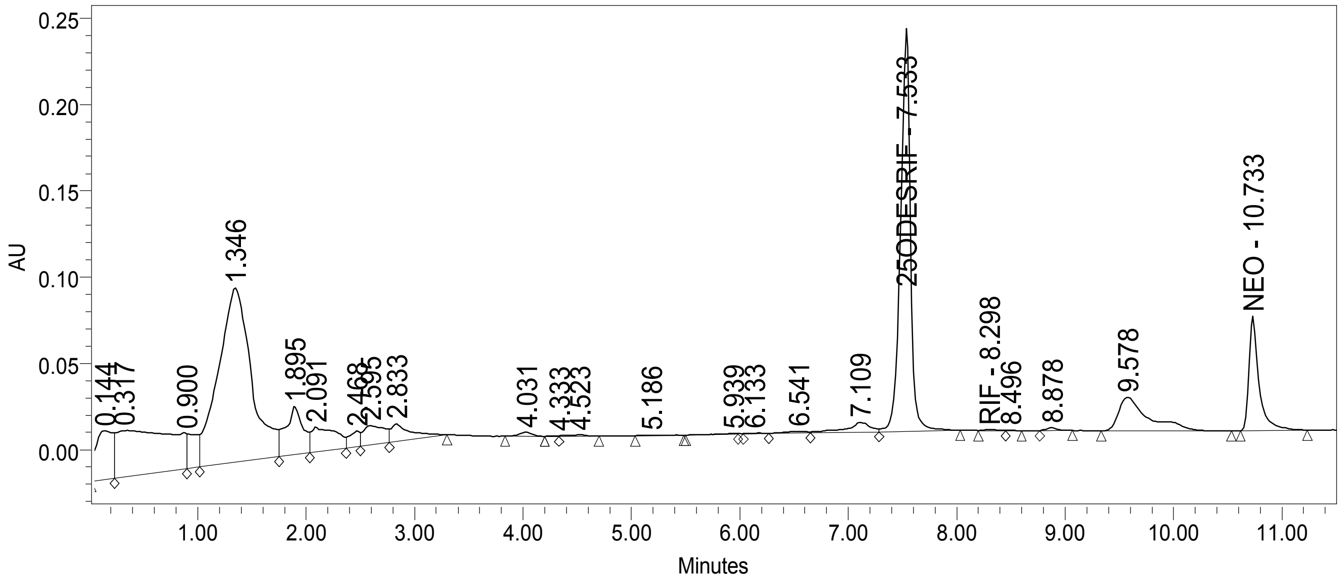

	Peak Name	RT	Area	% Area	Height
1		0.144	311764	4.54	28191
2		0.317	943548	13.73	26799
3		0.900	134990	1.96	20209
4		1.346	2155679	31.37	100924
5		1.895	310772	4.52	27695
6		2.091	219481	3.19	13954
7		2.468	61044	0.89	8870
8		2.595	148752	2.16	11002
9		2.833	127942	1.86	10100
10		4.031	18571	0.27	2232
11		4.333	1370	0.02	277
12		4.523	9589	0.14	873

Reported by User: Saneesh (Saneesh) Project Name: HDI_Stellenbosch_2016

	Peak Name	RT	Area	% Area	Height
13		5.186	4062	0.06	273
14		5.939	7010	0.10	423
15		6.133	4884	0.07	454
16		6.541	17191	0.25	1149
17		7.109	88581	1.29	5795
18	25ODESRIF	7.533	1496301	21.78	233906
19	RIF	8.298	5042	0.07	628
20		8.496	2004	0.03	409
21		8.878	10437	0.15	1582
22		9.578	369662	5.38	19476
23	NEO	10.733	422404	6.15	65419

Reported by User: Saneesh (Saneesh) Project Name: HDI_Stellenbosch_2016

SAMPLE INFORMATION

Sample Name:	RIF Inc Basil_EtOH BER23 45mn2 R	Acquired By:	Saneesh
Sample Type:	Unknown	Date Acquired:	2017/02/06 11:50:10 AM
Vial:	12	Acq. Method Set:	HDI_RIF_25ODESRIF_NEO
Injection #:	1	Date Processed:	2017/02/08 06:42:36 AM
Injection Volume:	15.00 ul	Processing Method:	HDI_RIF_ W2996 254.0nm-1.2
Run Time:	11.5 Minutes	Channel Name:	W2996 254.0nm-1.2
Sample Set Name:	RIF_Basil EtOH AssaySM3 6_1_17	Proc. Chnl. Descr.:	W2996 PDA 254.0 nm at 1.2

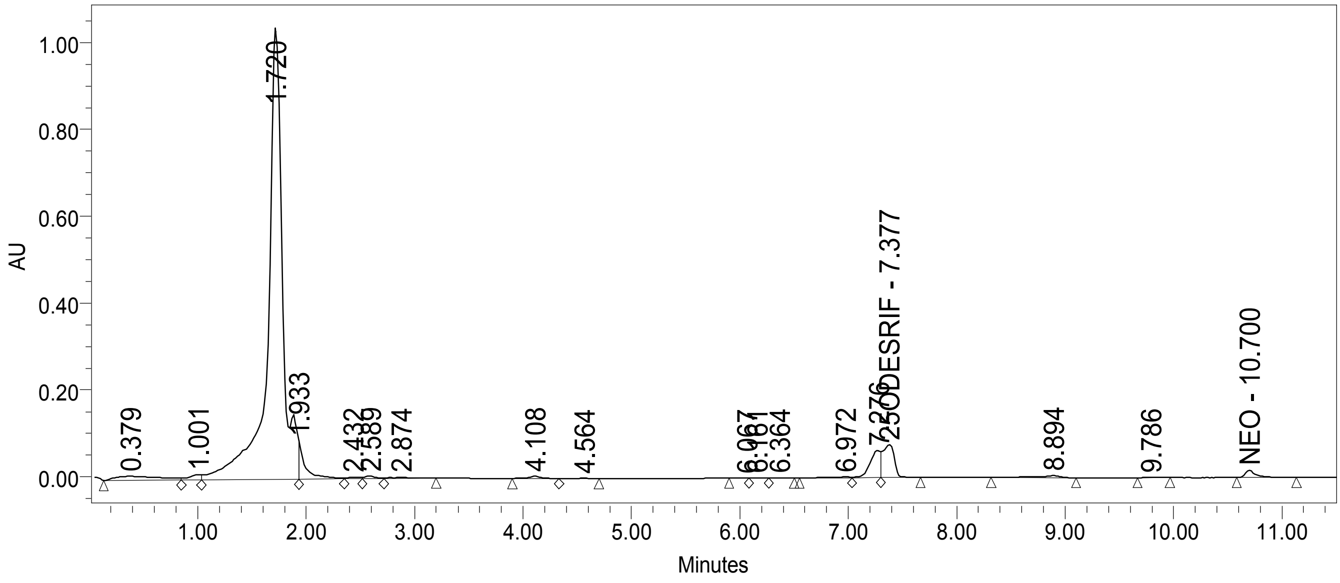

	Peak Name	RT	Area	% Area	Height
1		0.379	251402	2.11	8765
2		1.001	84296	0.71	11207
3		1.720	9899092	82.96	1046052
4		1.933	347359	2.91	88022
5		2.432	32843	0.28	4085
6		2.589	43336	0.36	5925
7		2.874	40165	0.34	3354
8		4.108	39242	0.33	5053
9		4.564	10088	0.08	1151
10		6.067	1942	0.02	250
11		6.161	3907	0.03	482
12		6.364	4880	0.04	625

Reported by User: Saneesh (Saneesh) Project Name: HDI_Stellenbosch_2016

	Peak Name	RT	Area	% Area	Height
13		6.972	27540	0.23	1882
14		7.276	408013	3.42	62186
15	25ODESRIF	7.377	548712	4.60	76276
16	RIF	8.100			
17		8.894	73190	0.61	4550
18		9.786	3061	0.03	300
19	NEO	10.700	112651	0.94	16720

SAMPLE INFORMATION

Sample Name:	RIF Inc Basil_EtOH BER23 45mn2 R	Acquired By:	Saneesh
Sample Type:	Unknown	Date Acquired:	2017/02/06 12:14:22 PM
Vial:	13	Acq. Method Set:	HDI_RIF_25ODESRIF_NEO
Injection #:	1	Date Processed:	2017/02/08 06:42:42 AM
Injection Volume:	15.00 ul	Processing Method:	HDI_RIF_ W2996 254.0nm-1.2
Run Time:	11.5 Minutes	Channel Name:	W2996 254.0nm-1.2
Sample Set Name:	RIF_Basil EtOH AssaySM4 6_1_17	Proc. Chnl. Descr.:	W2996 PDA 254.0 nm at 1.2

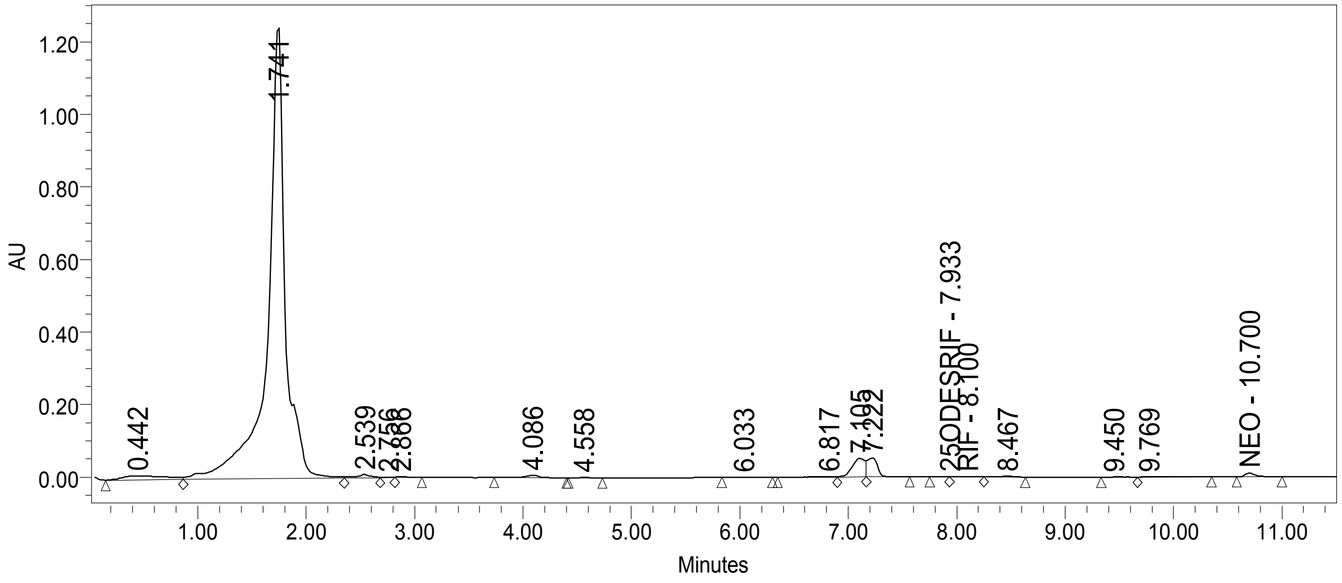

	Peak Name	RT	Area	% Area	Height
1		0.442	325752	2.11	11329
2		1.741	13989643	90.72	1257816
3		2.539	90370	0.59	9094
4		2.756	10820	0.07	1653
5		2.866	16163	0.10	2426
6		4.086	67832	0.44	6809
7		4.558	8656	0.06	1549
8		6.033	7520	0.05	490
9		6.817	20117	0.13	1427
10		7.105	425398	2.76	51361
11		7.222	335785	2.18	52716
12	25ODESRIF	7.933	4049	0.03	569

Reported by User: Saneesh (Saneesh)   Project Name: HDI_Stellenbosch_2016

	Peak Name	RT	Area	% Area	Height
13	RIF	8.100	16603	0.11	1019
14		8.467	24031	0.16	2105
15		9.450	3767	0.02	296
16		9.769	6838	0.04	367
17	NEO	10.700	67257	0.44	10801

Reported by User: Saneesh (Saneesh) Project Name: HDI_Stellenbosch_2016

SAMPLE INFORMATION

Sample Name:	RIF Inc Basil_EtOH C1 0min 1	Acquired By:	Saneesh
Sample Type:	Unknown	Date Acquired:	2017/02/02 12:45:58 AM
Vial:	2	Acq. Method Set:	HDI_RIF_25ODESRIF_NEO
Injection #:	1	Date Processed:	2017/02/02 10:20:53 AM
Injection Volume:	15.00 ul	Processing Method:	HDI_RIF_ W2996 254.0nm-1.2
Run Time:	11.5 Minutes	Channel Name:	W2996 254.0nm-1.2
Sample Set Name:	RIF_Basil EtOH Controls 2_1_17	Proc. Chnl. Descr.:	W2996 PDA 254.0 nm at 1.2

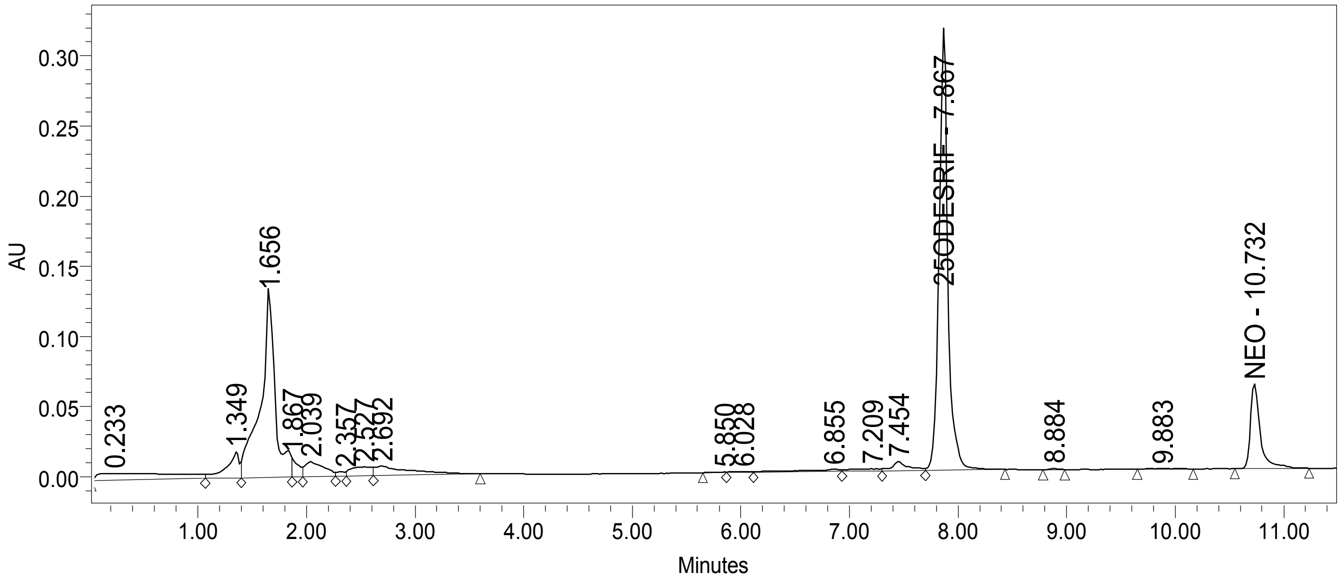

	Peak Name	RT	Area	% Area	Height
1		0.233	236824	5.36	4739
2		1.349	152128	3.44	18280
3		1.656	1254472	28.40	130770
4		1.867	54649	1.24	13964
5		2.039	130275	2.95	10556
6		2.357	17861	0.40	2945
7		2.527	85658	1.94	6371
8		2.692	153600	3.48	6759
9		5.850	3554	0.08	408
10		6.028	7109	0.16	520
11		6.855	38650	0.88	1514
12		7.209	29559	0.67	1464
13		7.454	67859	1.54	6604
14	25ODESRIF	7.867	1793453	40.60	309330

	Peak Name	RT	Area	% Area	Height
15	RIF	8.100			
16		8.884	4173	0.09	851
17		9.883	6905	0.16	419
18	NEO	10.732	380361	8.61	59940

SAMPLE INFORMATION

Sample Name:	RIF Inc Basil_EtOH C2 0min 2	Acquired By:	Saneesh
Sample Type:	Unknown	Date Acquired:	2017/02/02 01:00:52 AM
Vial:	3	Acq. Method Set:	HDI_RIF_25ODESRIF_NEO
Injection #:	1	Date Processed:	2017/02/02 10:20:53 AM
Injection Volume:	15.00 ul	Processing Method:	HDI_RIF_ W2996 254.0nm-1.2
Run Time:	11.5 Minutes	Channel Name:	W2996 254.0nm-1.2
Sample Set Name:	RIF_Basil EtOH Controls 2_1_17	Proc. Chnl. Descr.:	W2996 PDA 254.0 nm at 1.2

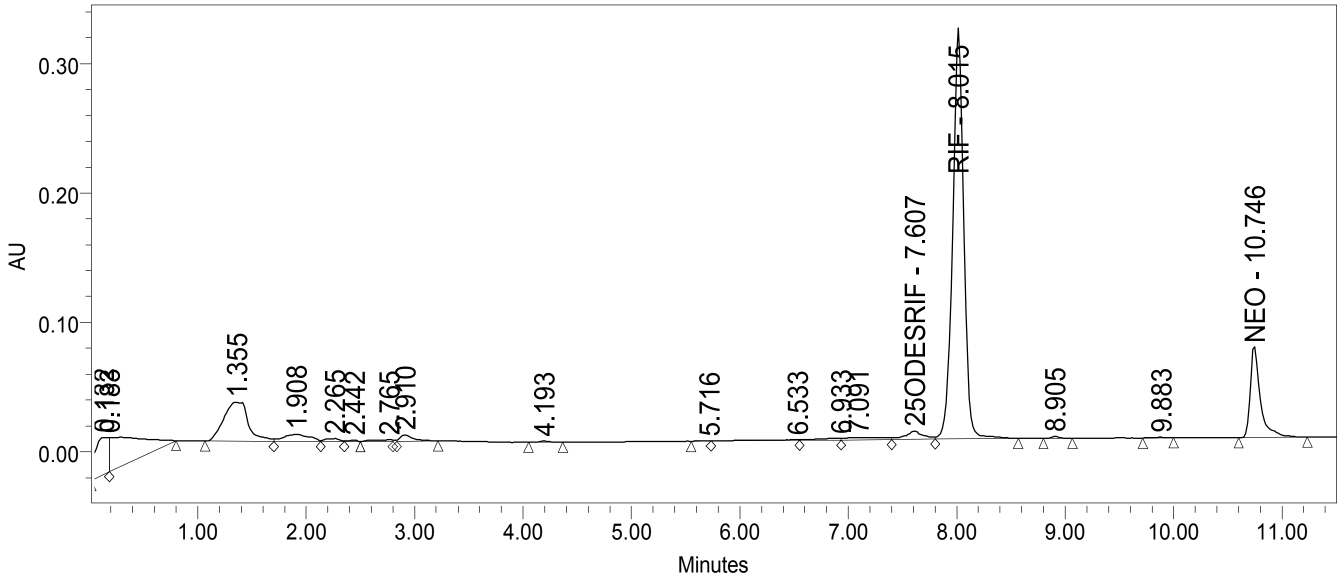

	Peak Name	RT	Area	% Area	Height
1		0.132	243338	5.70	28805
2		0.183	494454	11.58	26281
3		1.355	477256	11.18	30072
4		1.908	92860	2.17	5533
5		2.265	22532	0.53	2542
6		2.442	7942	0.19	1510
7		2.765	19998	0.47	1358
8		2.910	40969	0.96	4930
9		4.193	6805	0.16	961
10		5.716	1963	0.05	248
11		6.533	18620	0.44	519
12		6.933	20152	0.47	1277

	Peak Name	RT	Area	% Area	Height
13		7.091	40976	0.96	1669
14	25ODESRIF	7.607	72154	1.69	6449
15	RIF	8.015	2249378	52.67	316051
16		8.905	6717	0.16	1333
17		9.883	4125	0.10	446
18	NEO	10.746	450395	10.55	69715

SAMPLE INFORMATION

Sample Name:	RIF Inc Basil_EtOH C3 0min3	Acquired By:	Saneesh
Sample Type:	Unknown	Date Acquired:	2017/02/02 01:15:37 AM
Vial:	4	Acq. Method Set:	HDI_RIF_25ODESRIF_NEO
Injection #:	1	Date Processed:	2017/02/02 10:20:53 AM
Injection Volume:	15.00 ul	Processing Method:	HDI_RIF_ W2996 254.0nm-1.2
Run Time:	11.5 Minutes	Channel Name:	W2996 254.0nm-1.2
Sample Set Name:	RIF_Basil EtOH Controls 2_1_17	Proc. Chnl. Descr.:	W2996 PDA 254.0 nm at 1.2

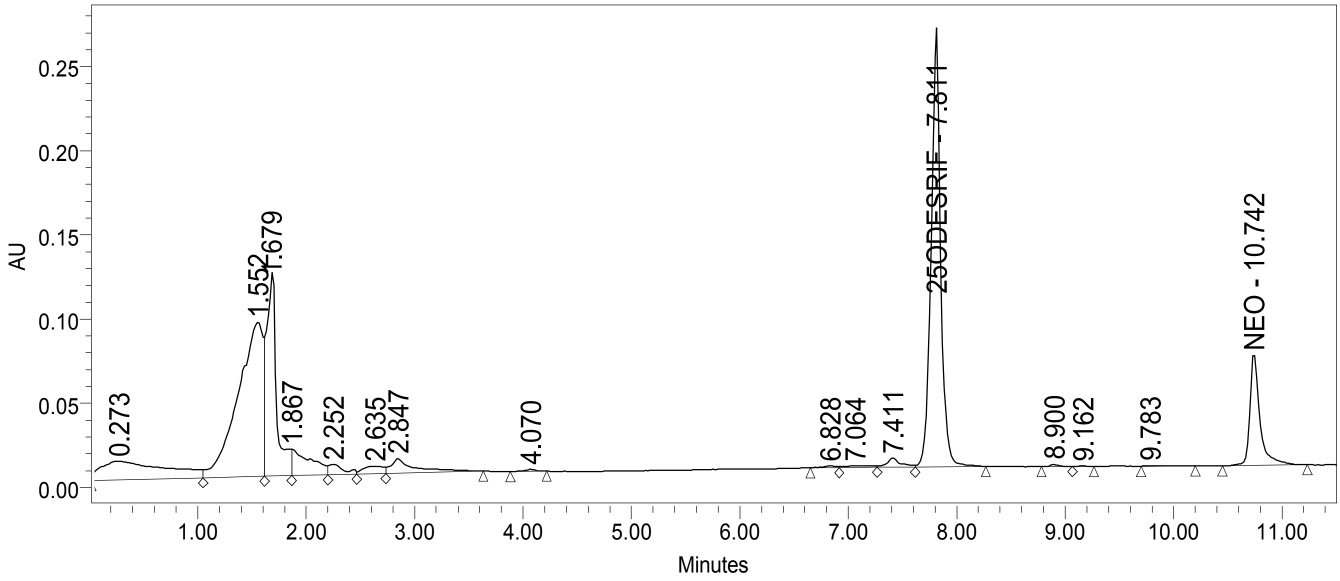

	Peak Name	RT	Area	% Area	Height
1		0.273	450882	8.61	10895
2		1.552	1476392	28.18	91482
3		1.679	774043	14.78	121125
4		1.867	191319	3.65	15555
5		2.252	57351	1.09	5932
6		2.635	57765	1.10	4407
7		2.847	132981	2.54	8636
8		4.070	8069	0.15	1154
9		6.828	6653	0.13	868
10		7.064	14873	0.28	831
11		7.411	44465	0.85	5166
12	25ODESRIF	7.811	1583222	30.22	257769

Reported by User: Saneesh (Saneesh) Project Name: HDI_Stellenbosch_2016

	Peak Name	RT	Area	% Area	Height
13	RIF	8.100			
14		8.900	6782	0.13	1202
15		9.162	2056	0.04	343
16		9.783	5678	0.11	292
17	NEO	10.742	426208	8.14	66008

SAMPLE INFORMATION

Sample Name:	RIF Inc Basil_EtOH C4 2min 1	Acquired By:	Saneesh
Sample Type:	Unknown	Date Acquired:	2017/02/02 01:30:20 AM
Vial:	5	Acq. Method Set:	HDI_RIF_25ODESRIF_NEO
Injection #:	1	Date Processed:	2017/02/02 10:20:53 AM
Injection Volume:	15.00 ul	Processing Method:	HDI_RIF_ W2996 254.0nm-1.2
Run Time:	11.5 Minutes	Channel Name:	W2996 254.0nm-1.2
Sample Set Name:	RIF_Basil EtOH Controls 2_1_17	Proc. Chnl. Descr.:	W2996 PDA 254.0 nm at 1.2

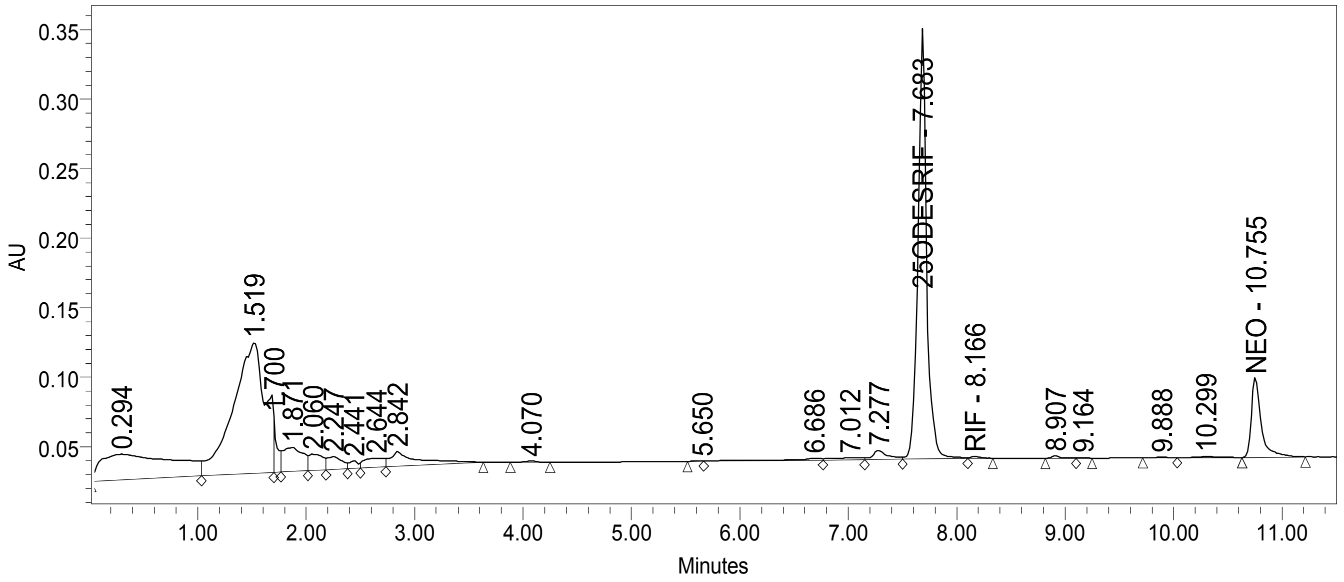

	Peak Name	RT	Area	% Area	Height
1		0.294	853272	14.62	18348
2		1.519	1926652	33.01	93976
3		1.700	84549	1.45	40289
4		1.871	224538	3.85	17425
5		2.060	105537	1.81	11673
6		2.247	87327	1.50	9137
7		2.441	31449	0.54	5379
8		2.644	85551	1.47	6679
9		2.842	189828	3.25	10637
10		4.070	7736	0.13	1010
11		5.650	1164	0.02	188
12		6.686	30004	0.51	1315

	Peak Name	RT	Area	% Area	Height
13		7.012	26196	0.45	1344
14		7.277	59505	1.02	6361
15	25ODESRIF	7.683	1726461	29.58	299551
16	RIF	8.166	7525	0.13	1273
17		8.907	8703	0.15	1530
18		9.164	2183	0.04	458
19		9.888	5619	0.10	578
20		10.299	11533	0.20	773
21	NEO	10.755	361136	6.19	57094

SAMPLE INFORMATION

Sample Name:	RIF Inc Basil_EtOH C5 2min 2	Acquired By:	Saneesh
Sample Type:	Unknown	Date Acquired:	2017/02/02 01:44:56 AM
Vial:	6	Acq. Method Set:	HDI_RIF_25ODESRIF_NEO
Injection #:	1	Date Processed:	2017/02/02 10:20:53 AM
Injection Volume:	15.00 ul	Processing Method:	HDI_RIF_ W2996 254.0nm-1.2
Run Time:	11.5 Minutes	Channel Name:	W2996 254.0nm-1.2
Sample Set Name:	RIF_Basil EtOH Controls 2_1_17	Proc. Chnl. Descr.:	W2996 PDA 254.0 nm at 1.2

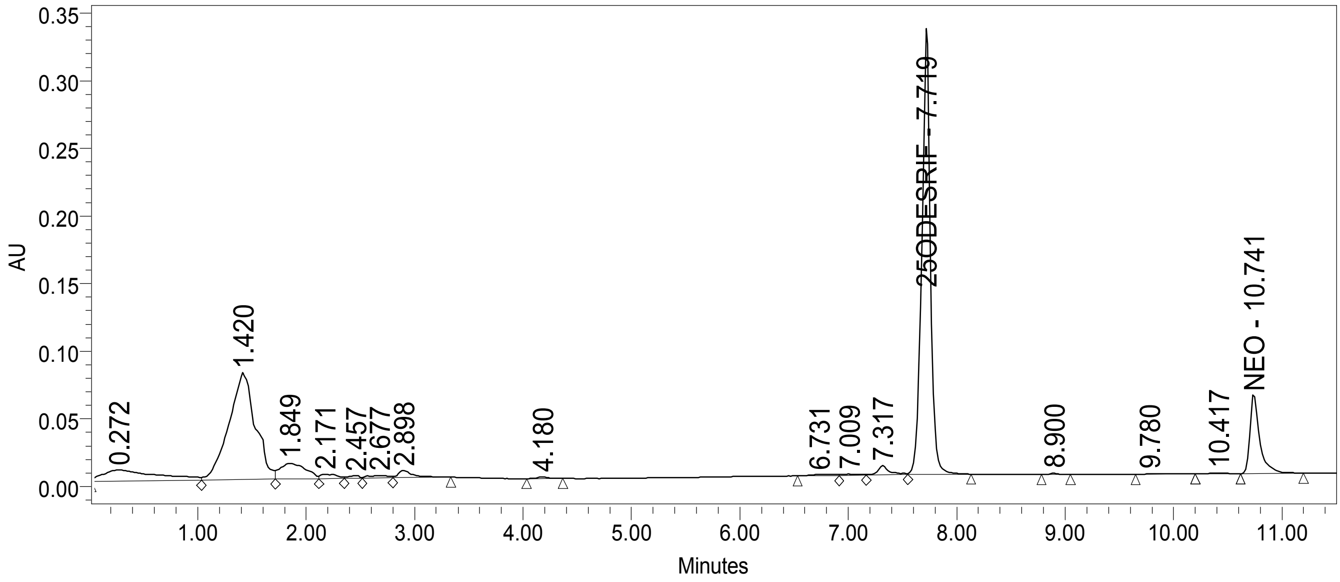

	Peak Name	RT	Area	% Area	Height
1		0.272	277479	6.54	8054
2		1.420	1341782	31.64	78342
3		1.849	191887	4.52	11571
4		2.171	32315	0.76	3229
5		2.457	13480	0.32	1976
6		2.677	24231	0.57	1756
7		2.898	51002	1.20	5299
8		4.180	8981	0.21	1250
9		6.731	12516	0.30	887
10		7.009	12865	0.30	1037
11		7.317	53799	1.27	6705
12	25ODESRIF	7.719	1837118	43.32	326168

Reported by User: Saneesh (Saneesh)   Project Name: HDI_Stellenbosch_2016

	Peak Name	RT	Area	% Area	Height
13	RIF	8.100			
14		8.900	5601	0.13	1040
15		9.780	4694	0.11	283
16		10.417	8410	0.20	711
17	NEO	10.741	365131	8.61	58116

Reported by User: Saneesh (Saneesh)

Project Name: HDI_Stellenbosch_2016

SAMPLE INFORMATION

Sample Name:	RIF Inc Basil_EtOH C6 2min 3	Acquired By:	Saneesh
Sample Type:	Unknown	Date Acquired:	2017/02/02 01:59:41 AM
Vial:	7	Acq. Method Set:	HDI_RIF_25ODESRIF_NEO
Injection #:	1	Date Processed:	2017/02/02 10:20:53 AM
Injection Volume:	15.00 ul	Processing Method:	HDI_RIF_ W2996 254.0nm-1.2
Run Time:	11.5 Minutes	Channel Name:	W2996 254.0nm-1.2
Sample Set Name:	RIF_Basil EtOH Controls 2_1_17	Proc. Chnl. Descr.:	W2996 PDA 254.0 nm at 1.2

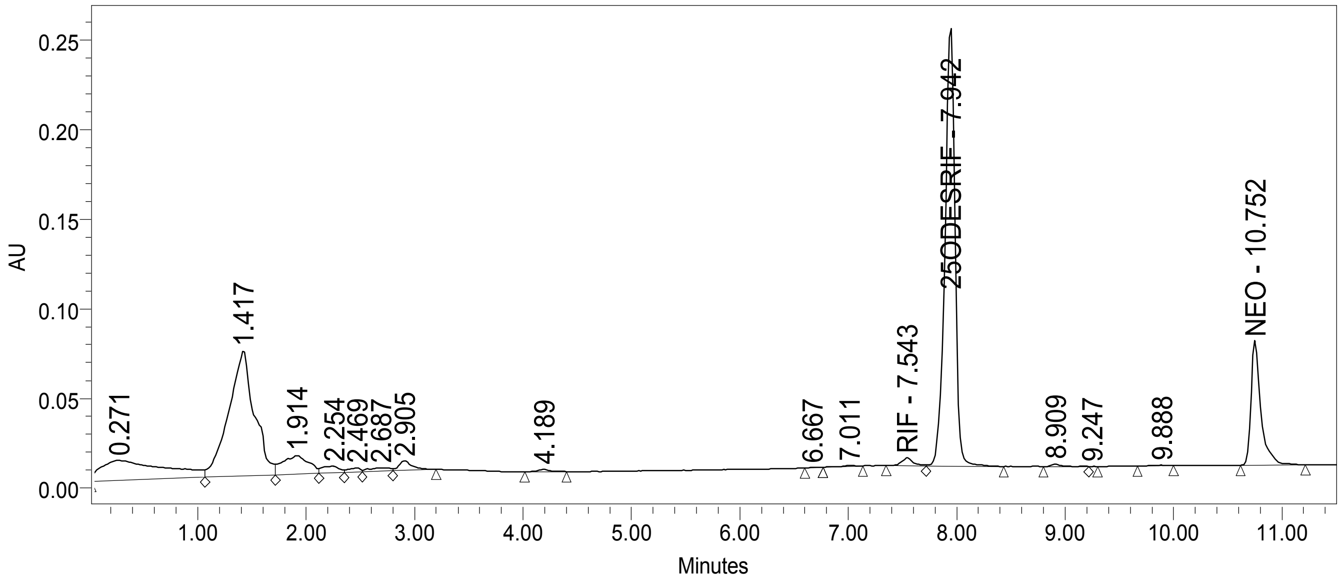

	Peak Name	RT	Area	% Area	Height
1		0.271	446721	11.21	11091
2		1.417	1153601	28.96	69565
3		1.914	174252	4.37	10235
4		2.254	41005	1.03	3546
5		2.469	18227	0.46	2391
6		2.687	25918	0.65	1885
7		2.905	45403	1.14	5192
8		4.189	9523	0.24	1370
9		6.667	828	0.02	136
10		7.011	5207	0.13	532
11	RIF	7.543	33889	0.85	4540
12	25ODESRIF	7.942	1577562	39.60	245774

	Peak Name	RT	Area	% Area	Height
13		8.909	8960	0.22	1500
14		9.247	209	0.01	64
15		9.888	4304	0.11	413
16	NEO	10.752	437737	10.99	68995

Reported by User: Saneesh (Saneesh)   Project Name: HDI_Stellenbosch_2016

SAMPLE INFORMATION

Sample Name:	RIF Inc Basil_EtOH C7 5min 1	Acquired By:	Saneesh
Sample Type:	Unknown	Date Acquired:	2017/02/02 02:14:26 AM
Vial:	8	Acq. Method Set:	HDI_RIF_25ODESRIF_NEO
Injection #:	1	Date Processed:	2017/02/02 10:20:53 AM
Injection Volume:	15.00 ul	Processing Method:	HDI_RIF_ W2996 254.0nm-1.2
Run Time:	11.5 Minutes	Channel Name:	W2996 254.0nm-1.2
Sample Set Name:	RIF_Basil EtOH Controls 2_1_17	Proc. Chnl. Descr.:	W2996 PDA 254.0 nm at 1.2

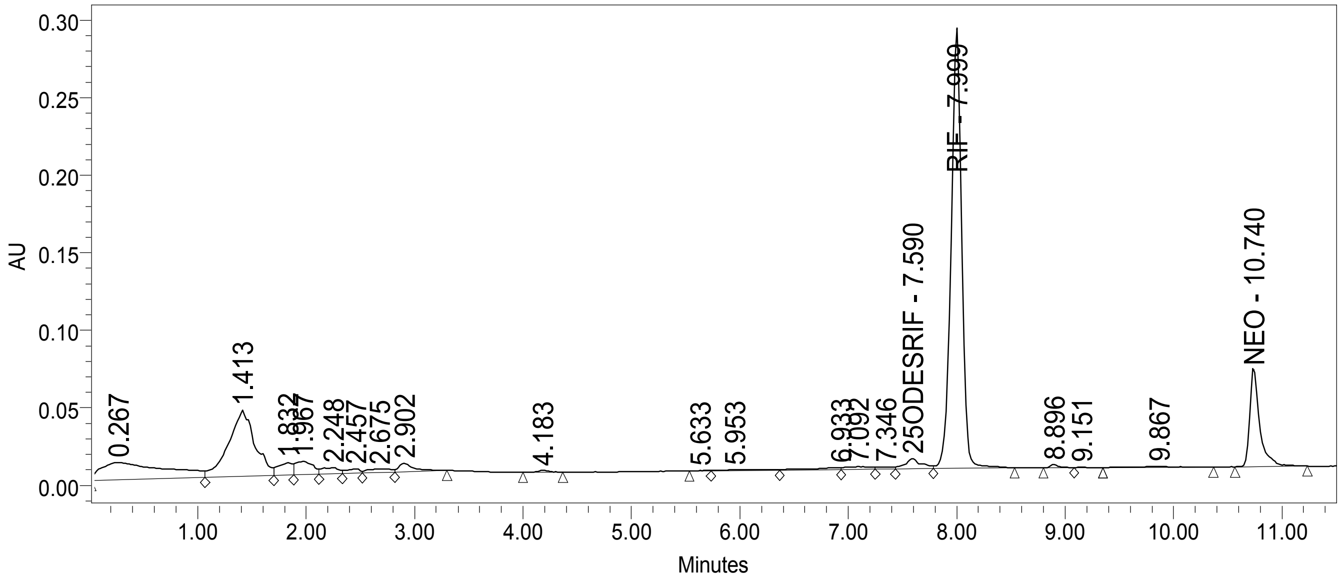

	Peak Name	RT	Area	% Area	Height
1		0.267	443342	11.15	10958
2		1.413	710940	17.88	41832
3		1.832	73452	1.85	7926
4		1.967	93939	2.36	8301
5		2.248	42763	1.08	3982
6		2.457	22303	0.56	2718
7		2.675	32088	0.81	2105
8		2.902	51477	1.29	5507
9		4.183	8872	0.22	1230
10		5.633	2299	0.06	269
11		5.953	14628	0.37	531
12		6.933	23672	0.60	1165

Reported by User: Saneesh (Saneesh) Project Name: HDI_Stellenbosch_2016

	Peak Name	RT	Area	% Area	Height
13		7.092	27646	0.70	1628
14		7.346	14500	0.36	1381
15	25ODESRIF	7.590	66018	1.66	6536
16	RIF	7.999	1920619	48.29	282013
17		8.896	9453	0.24	1794
18		9.151	1890	0.05	309
19		9.867	12723	0.32	596
20	NEO	10.740	404319	10.17	63290

SAMPLE INFORMATION

Sample Name:	RIF Inc Basil_EtOH C8 5min 2	Acquired By:	Saneesh
Sample Type:	Unknown	Date Acquired:	2017/02/02 02:29:10 AM
Vial:	9	Acq. Method Set:	HDI_RIF_25ODESRIF_NEO
Injection #:	1	Date Processed:	2017/02/02 10:20:53 AM
Injection Volume:	15.00 ul	Processing Method:	HDI_RIF_ W2996 254.0nm-1.2
Run Time:	11.5 Minutes	Channel Name:	W2996 254.0nm-1.2
Sample Set Name:	RIF_Basil EtOH Controls 2_1_17	Proc. Chnl. Descr.:	W2996 PDA 254.0 nm at 1.2

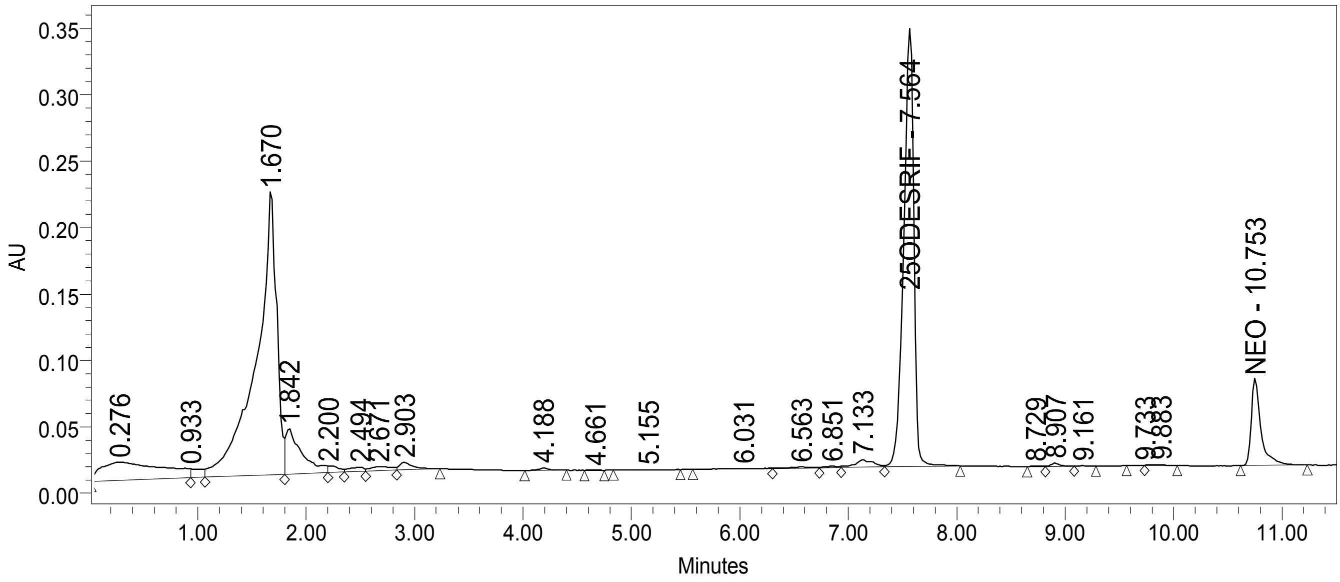

	Peak Name	RT	Area	% Area	Height
1		0.276	530542	8.12	13546
2		0.933	50358	0.77	6622
3		1.670	2750464	42.10	207956
4		1.842	360526	5.52	33568
5		2.200	33541	0.51	4960
6		2.494	33326	0.51	3400
7		2.671	43011	0.66	3094
8		2.903	49664	0.76	5683
9		4.188	12616	0.19	1832
10		4.661	1203	0.02	245
11		5.155	3067	0.05	132
12		6.031	6733	0.10	221

Reported by User: Saneesh (Saneesh) Project Name: HDI_Stellenbosch_2016

	Peak Name	RT	Area	% Area	Height
13		6.563	11059	0.17	787
14		6.851	8324	0.13	894
15		7.133	65139	1.00	5650
16	25ODESRIF	7.564	2141904	32.78	331766
17	RIF	8.100			
18		8.729	1481	0.02	238
19		8.907	13416	0.21	2521
20		9.161	1957	0.03	369
21		9.733	2806	0.04	403
22		9.883	7562	0.12	853
23	NEO	10.753	404587	6.19	64905

Reported by User: Saneesh (Saneesh) Project Name: HDI_Stellenbosch_2016

SAMPLE INFORMATION

Sample Name:	RIF Inc Basil_EtOH C9 5min 3	Acquired By:	Saneesh
Sample Type:	Unknown	Date Acquired:	2017/02/02 02:43:56 AM
Vial:	10	Acq. Method Set:	HDI_RIF_25ODESRIF_NEO
Injection #:	1	Date Processed:	2017/02/02 10:20:53 AM
Injection Volume:	15.00 ul	Processing Method:	HDI_RIF_ W2996 254.0nm-1.2
Run Time:	11.5 Minutes	Channel Name:	W2996 254.0nm-1.2
Sample Set Name:	RIF_Basil EtOH Controls 2_1_17	Proc. Chnl. Descr.:	W2996 PDA 254.0 nm at 1.2

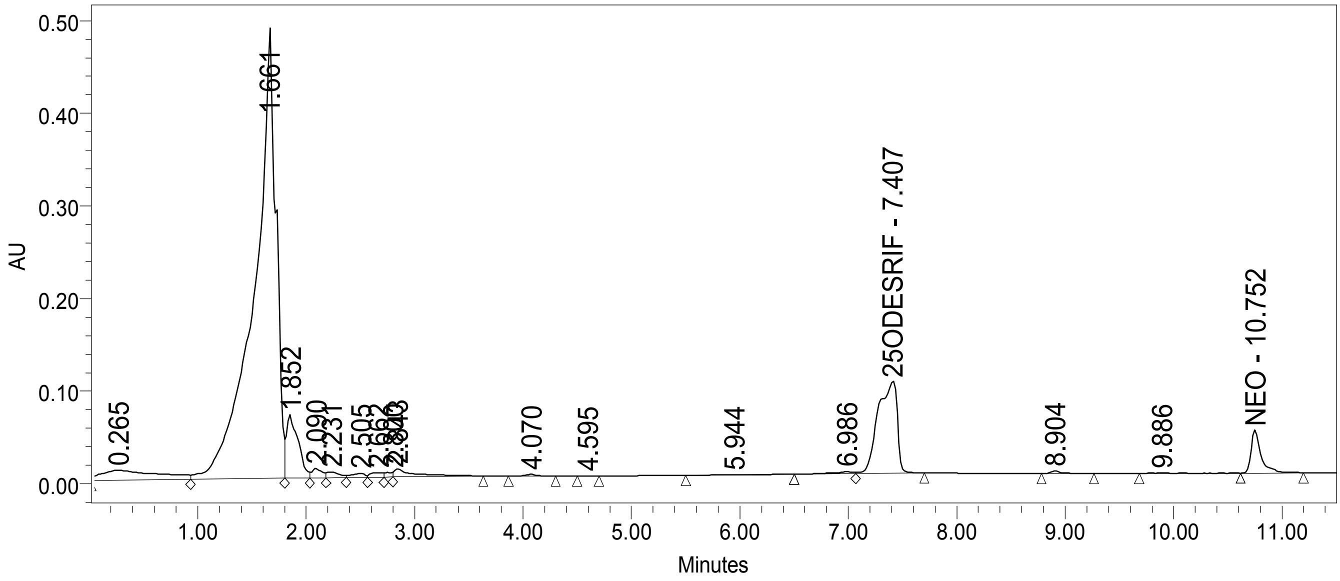

	Peak Name	RT	Area	% Area	Height
1		0.265	376546	3.96	10305
2		1.661	6698319	70.46	467927
3		1.852	520778	5.48	66211
4		2.090	67182	0.71	9998
5		2.231	47882	0.50	6174
6		2.505	36929	0.39	4420
7		2.662	36822	0.39	4584
8		2.800	23540	0.25	5040
9		2.843	115984	1.22	8777
10		4.070	14700	0.15	1902
11		4.595	1750	0.02	325
12		5.944	10001	0.11	249

	Peak Name	RT	Area	% Area	Height
13		6.986	30016	0.32	2279
14	25ODESRIF	7.407	1209050	12.72	101139
15	RIF	8.100			
16		8.904	18915	0.20	2659
17		9.886	8482	0.09	398
18	NEO	10.752	289407	3.04	45575

SAMPLE INFORMATION

Sample Name:	RIF Inc Basil_EtOH C10 10min 1	Acquired By:	Saneesh
Sample Type:	Unknown	Date Acquired:	2017/02/02 02:58:40 AM
Vial:	11	Acq. Method Set:	HDI_RIF_25ODESRIF_NEO
Injection #:	1	Date Processed:	2017/02/02 10:20:53 AM
Injection Volume:	15.00 ul	Processing Method:	HDI_RIF_ W2996 254.0nm-1.2
Run Time:	11.5 Minutes	Channel Name:	W2996 254.0nm-1.2
Sample Set Name:	RIF_Basil EtOH Controls 2_1_17	Proc. Chnl. Descr.:	W2996 PDA 254.0 nm at 1.2

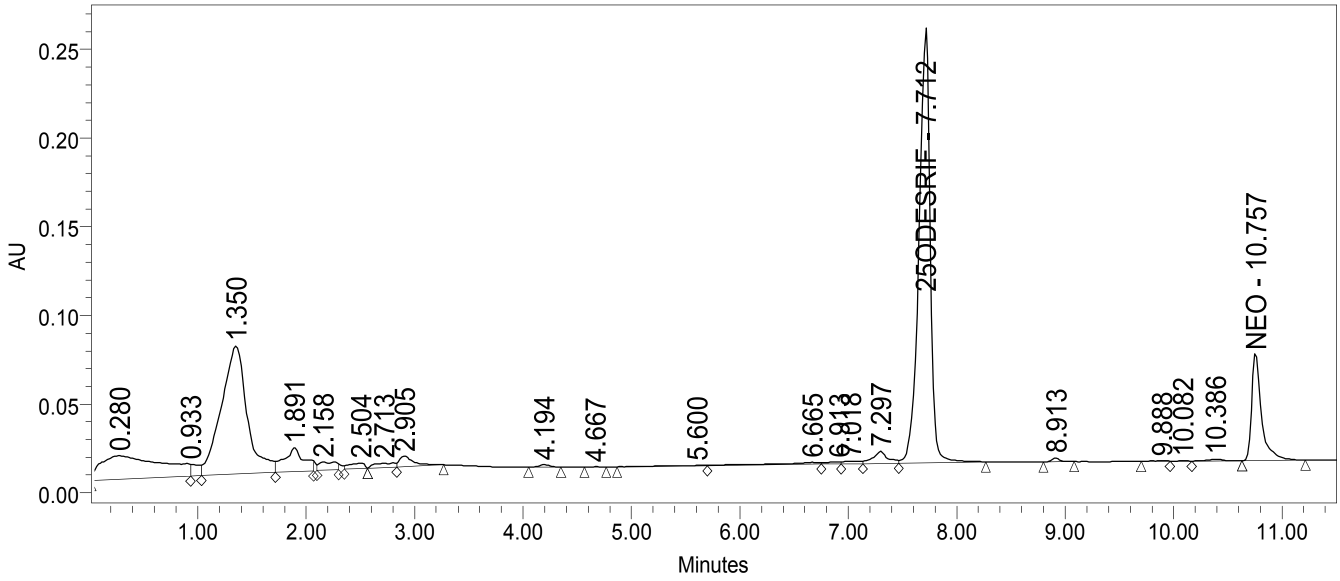

	Peak Name	RT	Area	% Area	Height
1		0.280	512232	11.62	13042
2		0.933	36584	0.83	6555
3		1.350	1201328	27.25	72160
4		1.891	168185	3.81	13484
5		2.158	49520	1.12	4779
6		2.504	32447	0.74	3315
7		2.713	32794	0.74	2480
8		2.905	54103	1.23	5932
9		4.194	9486	0.22	1395
10		4.667	1029	0.02	217
11		5.600	5791	0.13	225
12		6.665	28275	0.64	1125

	Peak Name	RT	Area	% Area	Height
13		6.913	10254	0.23	1041
14		7.018	14916	0.34	1326
15		7.297	62449	1.42	6605
16	25ODESRIF	7.712	1782346	40.42	247089
17	RIF	8.100			
18		8.913	10678	0.24	2149
19		9.888	4301	0.10	414
20		10.082	1839	0.04	193
21		10.386	10645	0.24	946
22	NEO	10.757	379928	8.62	60420

Reported by User: Saneesh (Saneesh)   Project Name: HDI_Stellenbosch_2016

SAMPLE INFORMATION

Sample Name:	RIF Inc Basil_EtOH C11 10min 2	Acquired By:	Saneesh
Sample Type:	Unknown	Date Acquired:	2017/02/02 03:13:26 AM
Vial:	12	Acq. Method Set:	HDI_RIF_25ODESRIF_NEO
Injection #:	1	Date Processed:	2017/02/02 10:20:53 AM
Injection Volume:	15.00 ul	Processing Method:	HDI_RIF_ W2996 254.0nm-1.2
Run Time:	11.5 Minutes	Channel Name:	W2996 254.0nm-1.2
Sample Set Name:	RIF_Basil EtOH Controls 2_1_17	Proc. Chnl. Descr.:	W2996 PDA 254.0 nm at 1.2

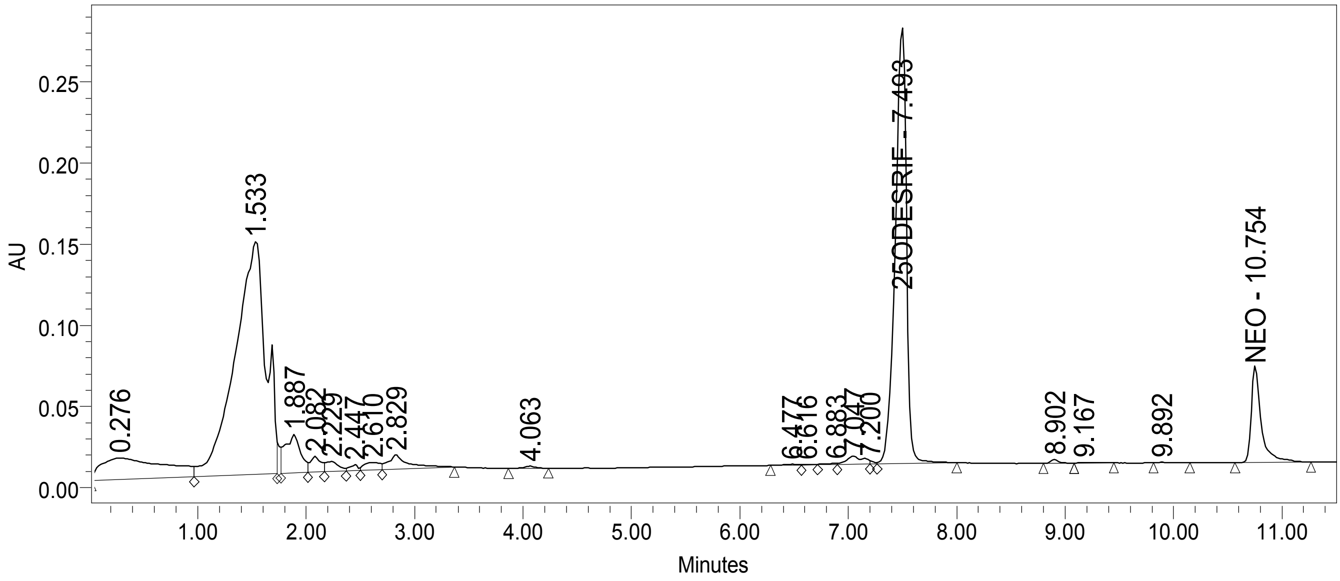

	Peak Name	RT	Area	% Area	Height
1		0.276	524640	8.36	13081
2		1.533	2813995	44.83	143556
3		1.887	237022	3.78	23340
4		2.082	64853	1.03	9466
5		2.229	50938	0.81	6222
6		2.447	19345	0.31	3509
7		2.610	47291	0.75	4496
8		2.829	109113	1.74	8652
9		4.063	10065	0.16	1322
10		6.477	4497	0.07	551
11		6.616	2653	0.04	326
12		6.883	6587	0.10	704

Reported by User: Saneesh (Saneesh)   Project Name: HDI_Stellenbosch_2016

	Peak Name	RT	Area	% Area	Height
13		7.047	54059	0.86	4941
14		7.200	4873	0.08	2010
15	25ODESRIF	7.493	1933837	30.81	272072
16	RIF	8.100			
17		8.902	11153	0.18	2213
18		9.167	5475	0.09	417
19		9.892	2367	0.04	362
20	NEO	10.754	374879	5.97	58808

Reported by User: Saneesh (Saneesh) Project Name: HDI_Stellenbosch_2016

SAMPLE INFORMATION

Sample Name:	RIF Inc Basil_EtOH C12 10min 3	Acquired By:	Saneesh
Sample Type:	Unknown	Date Acquired:	2017/02/02 03:28:10 AM
Vial:	13	Acq. Method Set:	HDI_RIF_25ODESRIF_NEO
Injection #:	1	Date Processed:	2017/02/02 10:20:53 AM
Injection Volume:	15.00 ul	Processing Method:	HDI_RIF_ W2996 254.0nm-1.2
Run Time:	11.5 Minutes	Channel Name:	W2996 254.0nm-1.2
Sample Set Name:	RIF_Basil EtOH Controls 2_1_17	Proc. Chnl. Descr.:	W2996 PDA 254.0 nm at 1.2

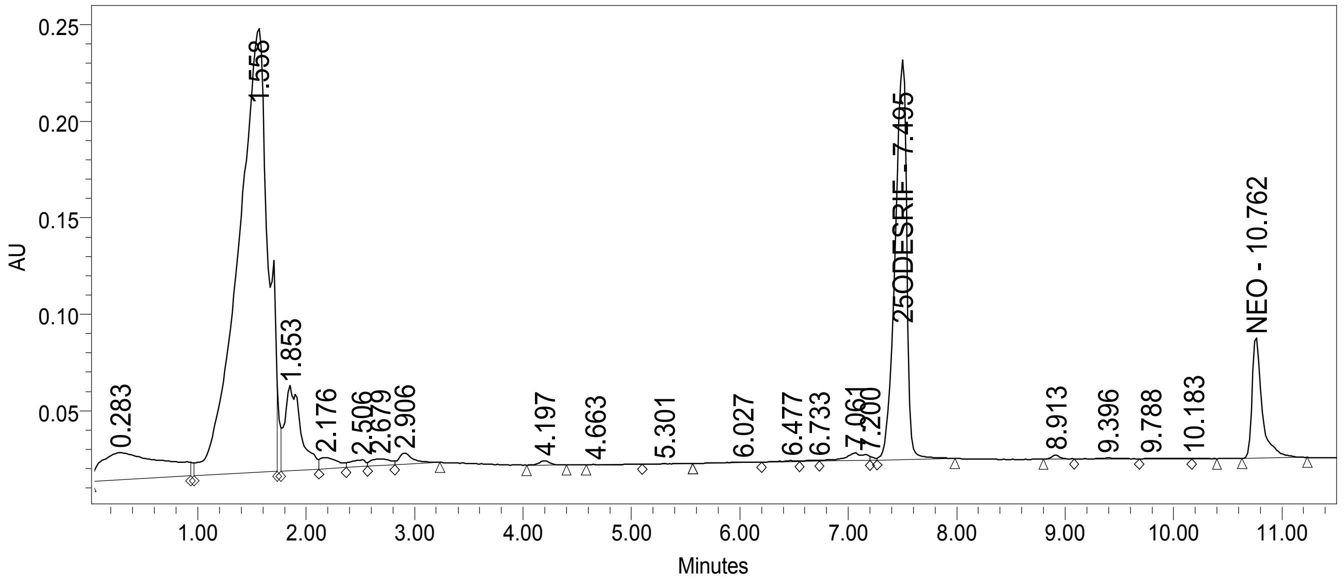

	Peak Name	RT	Area	% Area	Height
1		0.283	546782	7.18	13895
2		1.558	4339800	56.98	231350
3		1.853	460893	6.05	42810
4		2.176	64157	0.84	5725
5		2.506	35606	0.47	3681
6		2.679	43700	0.57	3528
7		2.906	53125	0.70	5786
8		4.197	16433	0.22	2232
9		4.663	3640	0.05	328
10		5.301	3072	0.04	206
11		6.027	7794	0.10	254
12		6.477	6076	0.08	511

	Peak Name	RT	Area	% Area	Height
13		6.733	3825	0.05	413
14		7.061	51330	0.67	3885
15		7.200	5065	0.07	1967
16	25ODESRIF	7.495	1549023	20.34	208666
17	RIF	8.100			
18		8.913	11432	0.15	2099
19		9.396	8506	0.11	534
20		9.788	5054	0.07	353
21		10.183	949	0.01	128
22	NEO	10.762	400343	5.26	61984

SAMPLE INFORMATION

Sample Name:	RIF Inc Basil_EtOH C13 15min 1	Acquired By:	Saneesh
Sample Type:	Unknown	Date Acquired:	2017/02/02 03:42:56 AM
Vial:	14	Acq. Method Set:	HDI_RIF_25ODESRIF_NEO
Injection #:	1	Date Processed:	2017/02/02 10:20:53 AM
Injection Volume:	15.00 ul	Processing Method:	HDI_RIF_ W2996 254.0nm-1.2
Run Time:	11.5 Minutes	Channel Name:	W2996 254.0nm-1.2
Sample Set Name:	RIF_Basil EtOH Controls 2_1_17	Proc. Chnl. Descr.:	W2996 PDA 254.0 nm at 1.2

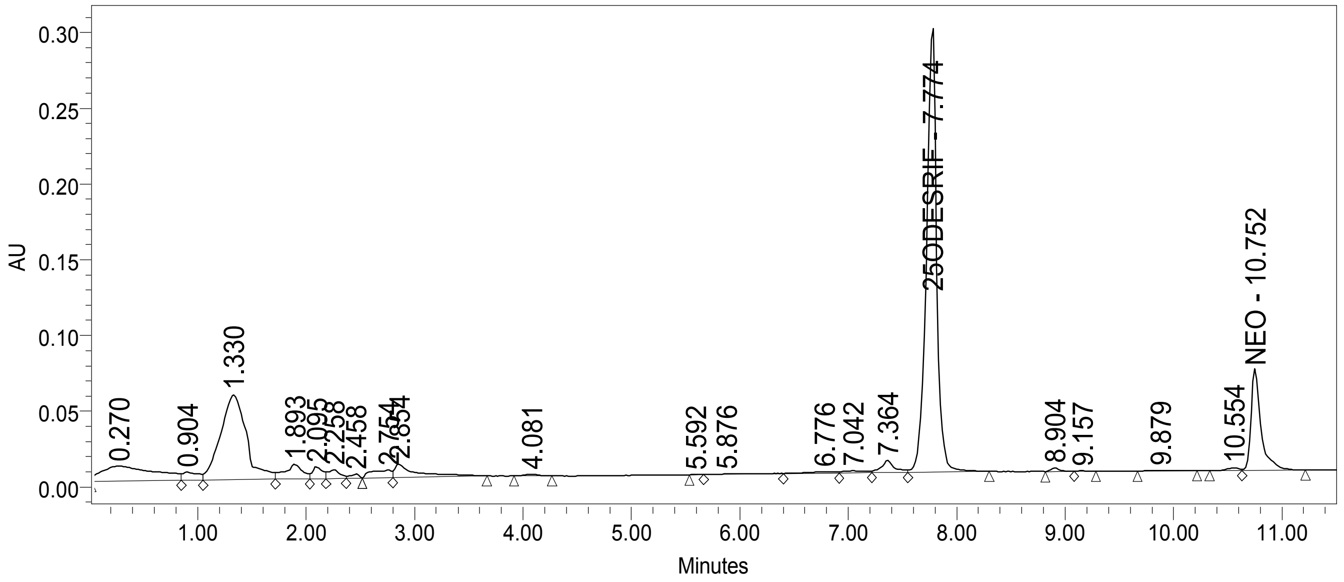

	Peak Name	RT	Area	% Area	Height
1		0.270	330579	7.77	9678
2		0.904	53919	1.27	5377
3		1.330	923448	21.70	56003
4		1.893	109144	2.56	9580
5		2.095	51203	1.20	7847
6		2.258	43045	1.01	5807
7		2.458	15826	0.37	2863
8		2.754	67019	1.57	4938
9		2.854	122898	2.89	9028
10		4.081	9261	0.22	1175
11		5.592	1205	0.03	196
12		5.876	12771	0.30	357

Reported by User: Saneesh (Saneesh) Project Name: HDI_Stellenbosch_2016

	Peak Name	RT	Area	% Area	Height
13		6.776	22803	0.54	1105
14		7.042	21672	0.51	1384
15		7.364	67468	1.59	7966
16	25ODESRIF	7.774	1937976	45.53	293943
17	RIF	8.100			
18		8.904	13379	0.31	2418
19		9.157	2652	0.06	443
20		9.879	6935	0.16	429
21		10.554	15670	0.37	1938
22	NEO	10.752	427170	10.04	66293

SAMPLE INFORMATION

Sample Name:	RIF Inc Basil_EtOH C14 15min 2	Acquired By:	Saneesh
Sample Type:	Unknown	Date Acquired:	2017/02/02 03:57:40 AM
Vial:	15	Acq. Method Set:	HDI_RIF_25ODESRIF_NEO
Injection #:	1	Date Processed:	2017/02/02 10:20:53 AM
Injection Volume:	15.00 ul	Processing Method:	HDI_RIF_ W2996 254.0nm-1.2
Run Time:	11.5 Minutes	Channel Name:	W2996 254.0nm-1.2
Sample Set Name:	RIF_Basil EtOH Controls 2_1_17	Proc. Chnl. Descr.:	W2996 PDA 254.0 nm at 1.2

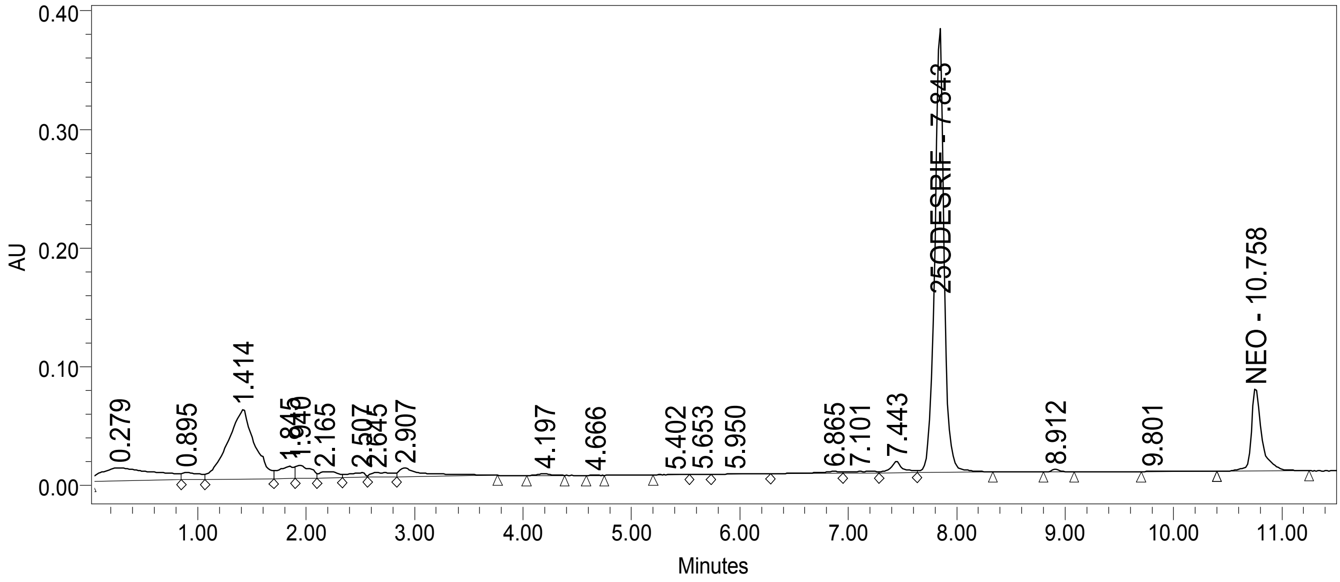

	Peak Name	RT	Area	% Area	Height
1		0.279	384275	7.95	10845
2		0.895	69360	1.44	6277
3		1.414	1018505	21.08	58580
4		1.845	102823	2.13	9916
5		1.940	101129	2.09	10868
6		2.165	60754	1.26	5160
7		2.507	43496	0.90	4115
8		2.645	52797	1.09	3810
9		2.907	124924	2.59	7271
10		4.197	12045	0.25	1640
11		4.666	1102	0.02	237
12		5.402	1815	0.04	133

Reported by User: Saneesh (Saneesh) Project Name: HDI_Stellenbosch_2016

	Peak Name	RT	Area	% Area	Height
13		5.653	1370	0.03	162
14		5.950	9159	0.19	363
15		6.865	26449	0.55	1581
16		7.101	24265	0.50	1328
17		7.443	74288	1.54	9447
18	25ODESRIF	7.843	2235092	46.27	370630
19	RIF	8.100			
20		8.912	11471	0.24	2161
21		9.801	7653	0.16	362
22	NEO	10.758	468257	9.69	69719

SAMPLE INFORMATION

Sample Name:	RIF Inc Basil_EtOH C15 15min 3	Acquired By:	Saneesh
Sample Type:	Unknown	Date Acquired:	2017/02/02 04:12:26 AM
Vial:	16	Acq. Method Set:	HDI_RIF_25ODESRIF_NEO
Injection #:	1	Date Processed:	2017/02/02 10:20:53 AM
Injection Volume:	15.00 ul	Processing Method:	HDI_RIF_ W2996 254.0nm-1.2
Run Time:	11.5 Minutes	Channel Name:	W2996 254.0nm-1.2
Sample Set Name:	RIF_Basil EtOH Controls 2_1_17	Proc. Chnl. Descr.:	W2996 PDA 254.0 nm at 1.2

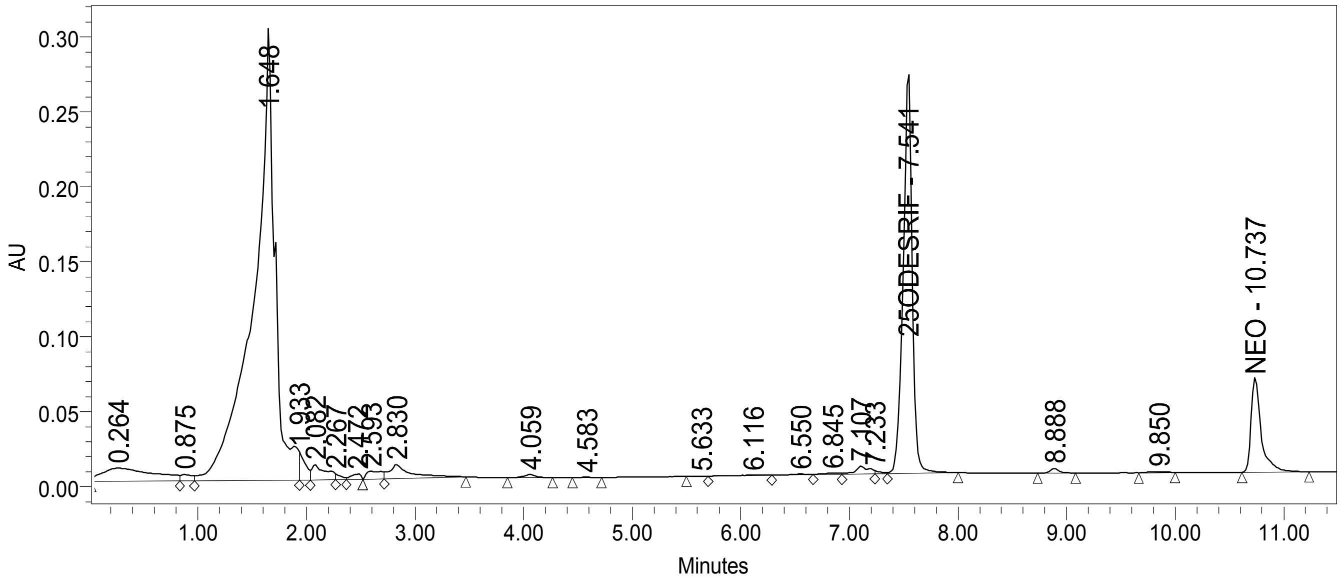

	Peak Name	RT	Area	% Area	Height
1		0.264	279810	4.08	8606
2		0.875	29945	0.44	4278
3		1.648	4099194	59.71	292150
4		1.933	71043	1.03	18776
5		2.082	93470	1.36	9982
6		2.267	13760	0.20	4073
7		2.472	24012	0.35	4179
8		2.593	54379	0.79	5505
9		2.830	129938	1.89	9320
10		4.059	16588	0.24	1993
11		4.583	2352	0.03	393
12		5.633	1345	0.02	148

Reported by User: Saneesh (Saneesh) Project Name: HDI_Stellenbosch_2016

	Peak Name	RT	Area	% Area	Height
13		6.116	6270	0.09	255
14		6.550	7763	0.11	747
15		6.845	10071	0.15	792
16		7.107	46077	0.67	5081
17		7.233	8306	0.12	2298
18	25ODESRIF	7.541	1538893	22.42	265595
19	RIF	8.100			
20		8.888	18025	0.26	3068
21		9.850	6619	0.10	560
22	NEO	10.737	407092	5.93	62568

SAMPLE INFORMATION

Sample Name:	RIF Inc Basil_EtOH C16 20min 1	Acquired By:	Saneesh
Sample Type:	Unknown	Date Acquired:	2017/02/02 04:27:11 AM
Vial:	17	Acq. Method Set:	HDI_RIF_25ODESRIF_NEO
Injection #:	1	Date Processed:	2017/02/02 10:20:53 AM
Injection Volume:	15.00 ul	Processing Method:	HDI_RIF_ W2996 254.0nm-1.2
Run Time:	11.5 Minutes	Channel Name:	W2996 254.0nm-1.2
Sample Set Name:	RIF_Basil EtOH Controls 2_1_17	Proc. Chnl. Descr.:	W2996 PDA 254.0 nm at 1.2

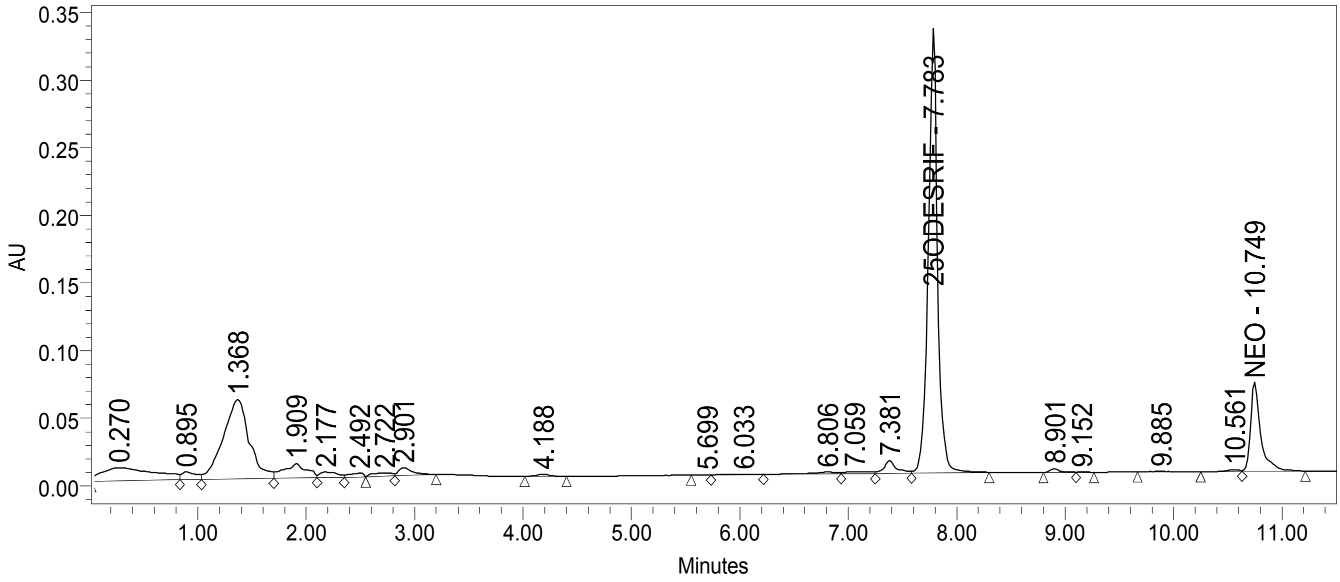

	Peak Name	RT	Area	% Area	Height
1		0.270	324601	7.64	9675
2		0.895	55148	1.30	5775
3		1.368	983983	23.17	58584
4		1.909	157325	3.71	10675
5		2.177	45287	1.07	4083
6		2.492	26602	0.63	3104
7		2.722	32899	0.77	2429
8		2.901	51982	1.22	5717
9		4.188	12793	0.30	1670
10		5.699	1675	0.04	214
11		6.033	8408	0.20	368
12		6.806	27950	0.66	1435

Reported by User: Saneesh (Saneesh) Project Name: HDI_Stellenbosch_2016

	Peak Name	RT	Area	% Area	Height
13		7.059	23331	0.55	1393
14		7.381	72522	1.71	9220
15	25ODESRIF	7.783	1960486	46.17	323555
16	RIF	8.100			
17		8.901	15371	0.36	2791
18		9.152	1805	0.04	336
19		9.885	8083	0.19	538
20		10.561	11330	0.27	1372
21	NEO	10.749	424444	10.00	65159

SAMPLE INFORMATION

Sample Name:	RIF Inc Basil_EtOH C17 20min 2	Acquired By:	Saneesh
Sample Type:	Unknown	Date Acquired:	2017/02/02 04:41:47 AM
Vial:	18	Acq. Method Set:	HDI_RIF_25ODESRIF_NEO
Injection #:	1	Date Processed:	2017/02/02 10:20:53 AM
Injection Volume:	15.00 ul	Processing Method:	HDI_RIF_ W2996 254.0nm-1.2
Run Time:	11.5 Minutes	Channel Name:	W2996 254.0nm-1.2
Sample Set Name:	RIF_Basil EtOH Controls 2_1_17	Proc. Chnl. Descr.:	W2996 PDA 254.0 nm at 1.2

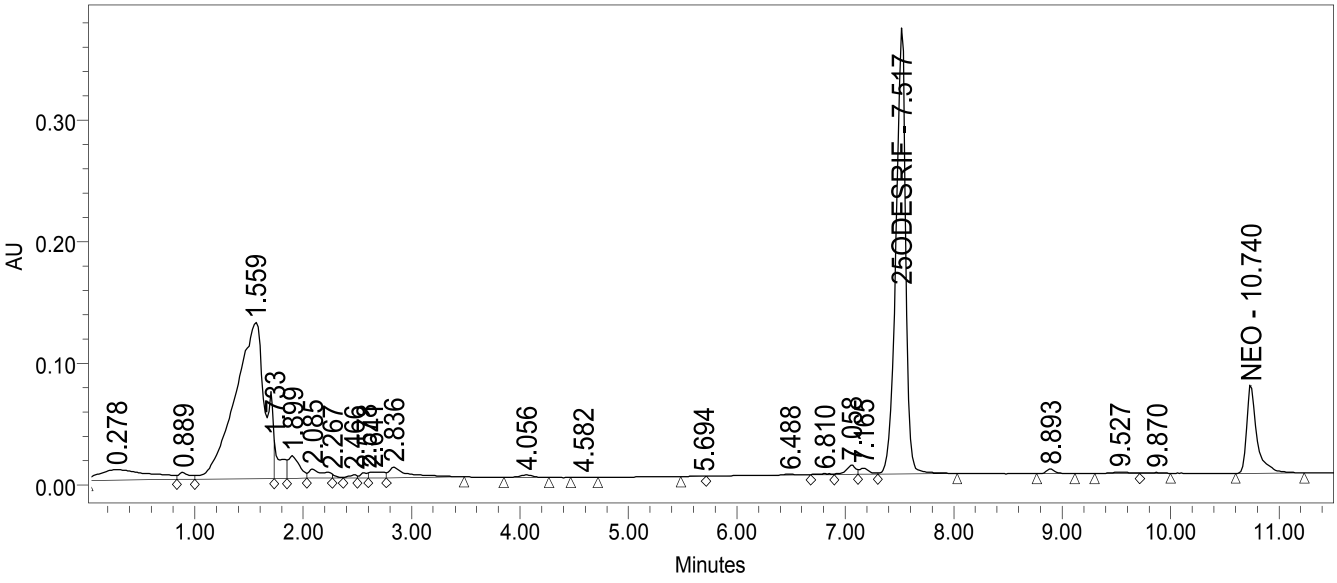

	Peak Name	RT	Area	% Area	Height
1		0.278	272761	4.36	8389
2		0.889	40495	0.65	5798
3		1.559	2468155	39.42	129405
4		1.733	119808	1.91	31535
5		1.899	136207	2.18	18501
6		2.085	74045	1.18	7638
7		2.267	8368	0.13	3322
8		2.466	12672	0.20	2687
9		2.578	18831	0.30	4012
10		2.641	43424	0.69	4520
11		2.836	102906	1.64	8733
12		4.056	17235	0.28	2095

Reported by User: Saneesh (Saneesh)   Project Name: HDI_Stellenbosch_2016

	Peak Name	RT	Area	% Area	Height
13		4.582	2098	0.03	321
14		5.694	1547	0.02	158
15		6.488	17362	0.28	915
16		6.810	8782	0.14	886
17		7.058	47096	0.75	7495
18		7.165	33248	0.53	5202
19	25ODESRIF	7.517	2325174	37.14	364638
20	RIF	8.100			
21		8.893	20174	0.32	3511
22		9.527	14031	0.22	1024
23		9.870	6266	0.10	652
24	NEO	10.740	470661	7.52	72460

SAMPLE INFORMATION

Sample Name:	RIF Inc Basil_EtOH C18 20min 3	Acquired By:	Saneesh
Sample Type:	Unknown	Date Acquired:	2017/02/02 04:56:32 AM
Vial:	19	Acq. Method Set:	HDI_RIF_25ODESRIF_NEO
Injection #:	1	Date Processed:	2017/02/02 10:20:53 AM
Injection Volume:	15.00 ul	Processing Method:	HDI_RIF_ W2996 254.0nm-1.2
Run Time:	11.5 Minutes	Channel Name:	W2996 254.0nm-1.2
Sample Set Name:	RIF_Basil EtOH Controls 2_1_17	Proc. Chnl. Descr.:	W2996 PDA 254.0 nm at 1.2

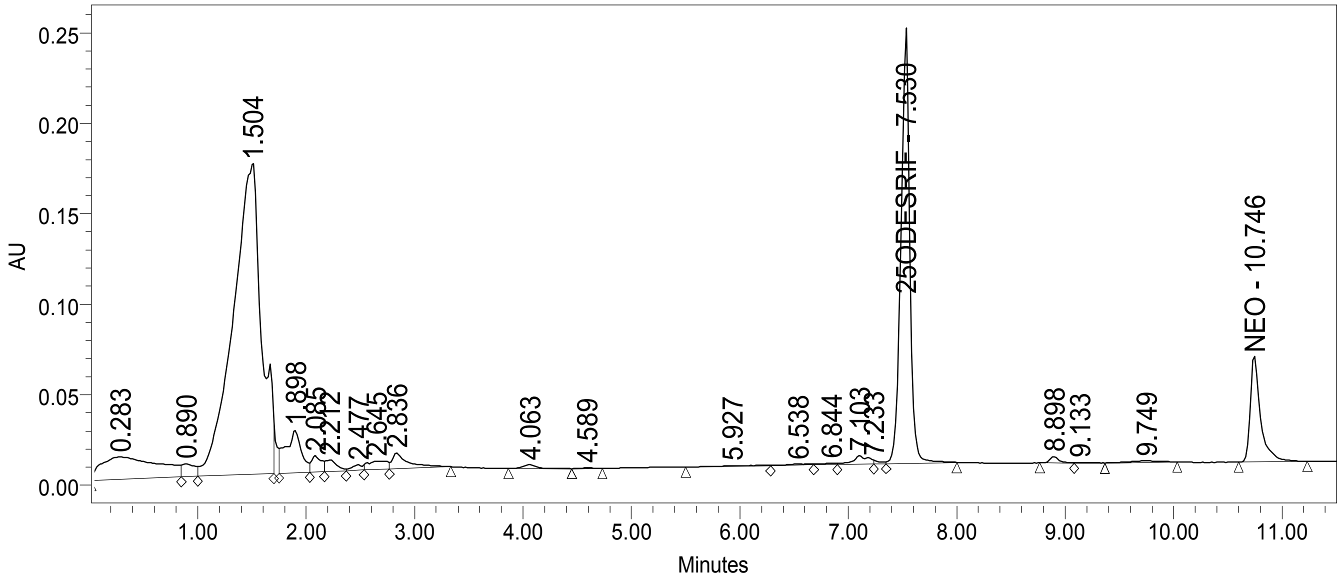

	Peak Name	RT	Area	% Area	Height
1		0.283	446970	7.46	12296
2		0.890	56808	0.95	7094
3		1.504	3082402	51.43	174418
4		1.898	237654	3.97	23460
5		2.085	55516	0.93	8835
6		2.212	46522	0.78	6279
7		2.477	20399	0.34	2961
8		2.645	57849	0.97	4665
9		2.836	90284	1.51	8831
10		4.063	19015	0.32	2083
11		4.589	3335	0.06	452
12		5.927	8553	0.14	342

Reported by User: Saneesh (Saneesh) Project Name: HDI_Stellenbosch_2016

	Peak Name	RT	Area	% Area	Height
13		6.538	8040	0.13	567
14		6.844	7667	0.13	786
15		7.103	45563	0.76	4632
16		7.233	7988	0.13	2082
17	25ODESRIF	7.530	1375410	22.95	237920
18	RIF	8.100			
19		8.898	19654	0.33	3411
20		9.133	2677	0.04	341
21		9.749	17340	0.29	868
22	NEO	10.746	384039	6.41	58053

SAMPLE INFORMATION

Sample Name:	RIF Inc Basil_EtOH C19 30min 1	Acquired By:	Saneesh
Sample Type:	Unknown	Date Acquired:	2017/02/02 05:11:25 AM
Vial:	20	Acq. Method Set:	HDI_RIF_25ODESRIF_NEO
Injection #:	1	Date Processed:	2017/02/02 10:20:53 AM
Injection Volume:	15.00 ul	Processing Method:	HDI_RIF_ W2996 254.0nm-1.2
Run Time:	11.5 Minutes	Channel Name:	W2996 254.0nm-1.2
Sample Set Name:	RIF_Basil EtOH Controls 2_1_17	Proc. Chnl. Descr.:	W2996 PDA 254.0 nm at 1.2

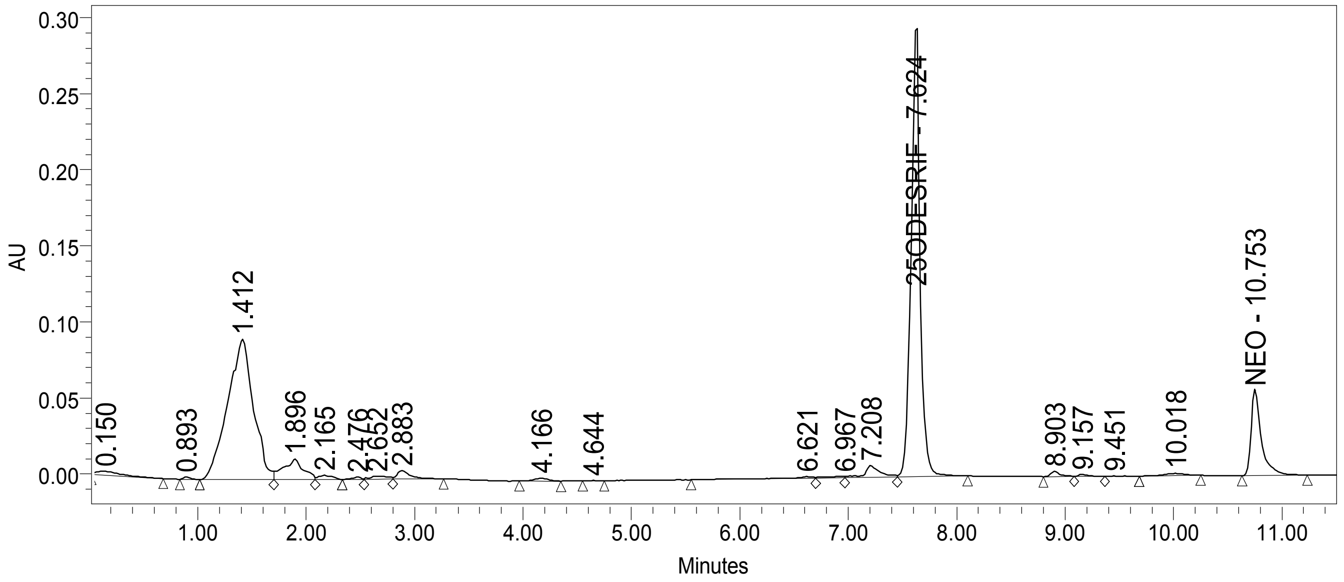

	Peak Name	RT	Area	% Area	Height
1		0.150	38612	0.94	2509
2		0.893	7298	0.18	1695
3		1.412	1534147	37.40	92500
4		1.896	170460	4.16	13521
5		2.165	24646	0.60	2677
6		2.476	9086	0.22	1638
7		2.652	23217	0.57	1861
8		2.883	48009	1.17	5428
9		4.166	16263	0.40	1977
10		4.644	1630	0.04	273
11		6.621	19292	0.47	1046
12		6.967	11605	0.28	917

	Peak Name	RT	Area	% Area	Height
13		7.208	75372	1.84	7687
14	25ODESRIF	7.624	1696927	41.36	292634
15	RIF	8.100			
16		8.903	21042	0.51	3382
17		9.157	9381	0.23	1416
18		9.451	3087	0.08	262
19		10.018	22516	0.55	1551
20	NEO	10.753	369913	9.02	56343

Reported by User: Saneesh (Saneesh)

Project Name: HDI_Stellenbosch_2016

SAMPLE INFORMATION

Sample Name:	RIF Inc Basil_EtOH C20 30min 2	Acquired By:	Saneesh
Sample Type:	Unknown	Date Acquired:	2017/02/02 05:26:10 AM
Vial:	21	Acq. Method Set:	HDI_RIF_25ODESRIF_NEO
Injection #:	1	Date Processed:	2017/02/02 10:20:53 AM
Injection Volume:	15.00 ul	Processing Method:	HDI_RIF_ W2996 254.0nm-1.2
Run Time:	11.5 Minutes	Channel Name:	W2996 254.0nm-1.2
Sample Set Name:	RIF_Basil EtOH Controls 2_1_17	Proc. Chnl. Descr.:	W2996 PDA 254.0 nm at 1.2

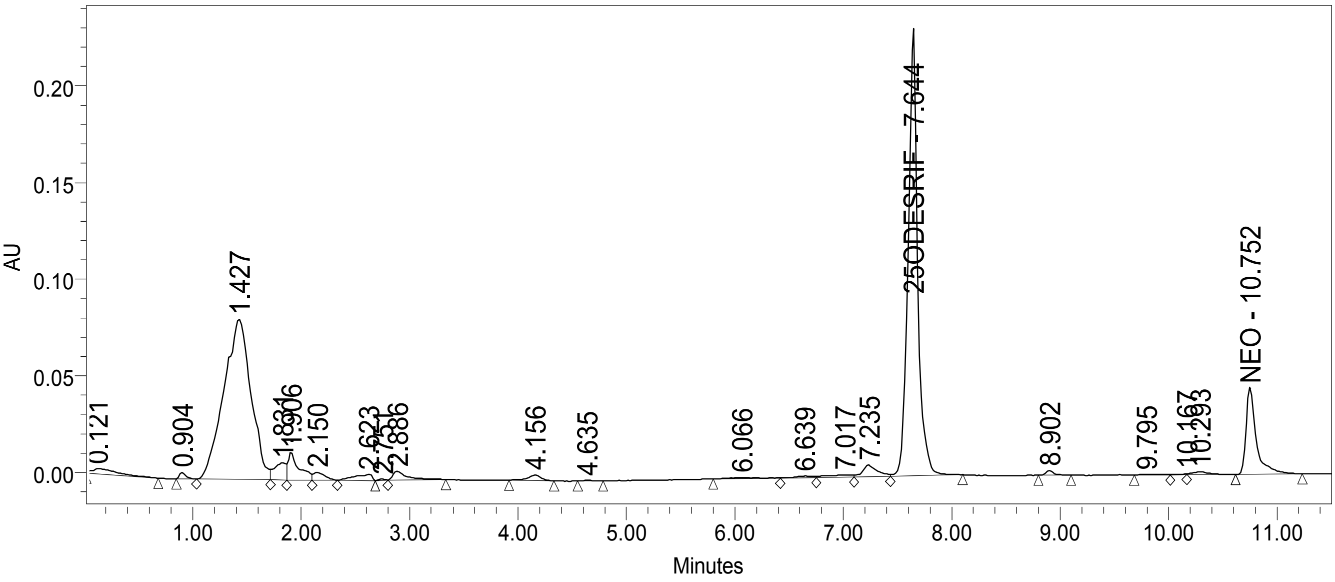

	Peak Name	RT	Area	% Area	Height
1		0.121	44671	1.22	2680
2		0.904	14628	0.40	3449
3		1.427	1453171	39.70	82906
4		1.831	67324	1.84	8792
5		1.906	101639	2.78	13614
6		2.150	30108	0.82	4008
7		2.623	37312	1.02	3393
8		2.751	2659	0.07	666
9		2.886	43214	1.18	4492
10		4.156	24510	0.67	2940
11		4.635	2187	0.06	387
12		6.066	9595	0.26	447

	Peak Name	RT	Area	% Area	Height
13		6.639	7698	0.21	743
14		7.017	16320	0.45	973
15		7.235	54256	1.48	5911
16	25ODESRIF	7.644	1425590	38.95	229524
17	RIF	8.100			
18		8.902	12457	0.34	2372
19		9.795	3713	0.10	274
20		10.167	2943	0.08	532
21		10.293	16355	0.45	1344
22	NEO	10.752	289850	7.92	44600

SAMPLE INFORMATION

Sample Name:	RIF Inc Basil_EtOH C21 30min 3	Acquired By:	Saneesh
Sample Type:	Unknown	Date Acquired:	2017/02/02 05:41:05 AM
Vial:	22	Acq. Method Set:	HDI_RIF_25ODESRIF_NEO
Injection #:	1	Date Processed:	2017/02/02 10:20:53 AM
Injection Volume:	15.00 ul	Processing Method:	HDI_RIF_ W2996 254.0nm-1.2
Run Time:	11.5 Minutes	Channel Name:	W2996 254.0nm-1.2
Sample Set Name:	RIF_Basil EtOH Controls 2_1_17	Proc. Chnl. Descr.:	W2996 PDA 254.0 nm at 1.2

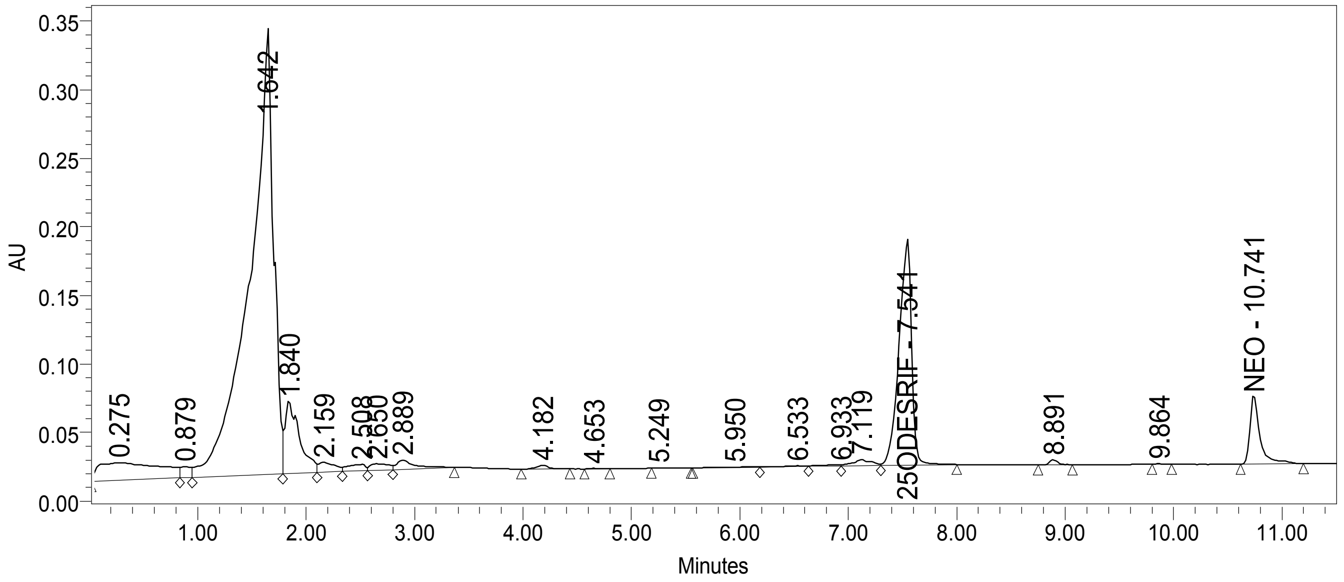

	Peak Name	RT	Area	% Area	Height
1		0.275	496023	6.21	12675
2		0.879	53831	0.67	7986
3		1.642	4903387	61.36	320073
4		1.840	504163	6.31	51877
5		2.159	76397	0.96	7168
6		2.508	54972	0.69	4849
7		2.650	55889	0.70	4657
8		2.889	76889	0.96	6626
9		4.182	20121	0.25	2642
10		4.653	2823	0.04	507
11		5.249	2109	0.03	193
12		5.950	5464	0.07	235

Reported by User: Saneesh (Saneesh) Project Name: HDI_Stellenbosch_2016

	Peak Name	RT	Area	% Area	Height
13		6.533	7226	0.09	540
14		6.933	11017	0.14	834
15		7.119	52634	0.66	4440
16	25ODESRIF	7.541	1327053	16.61	166949
17	RIF	8.100			
18		8.891	18286	0.23	3556
19		9.864	1706	0.02	310
20	NEO	10.741	321554	4.02	49556

Reported by User: Saneesh (Saneesh)   Project Name: HDI_Stellenbosch_2016

SAMPLE INFORMATION

Sample Name:	RIF Inc Basil_EtOH C22 45min 1	Acquired By:	Saneesh
Sample Type:	Unknown	Date Acquired:	2017/02/02 06:01:28 AM
Vial:	23	Acq. Method Set:	HDI_RIF_25ODESRIF_NEO
Injection #:	1	Date Processed:	2017/02/02 10:20:53 AM
Injection Volume:	15.00 ul	Processing Method:	HDI_RIF_ W2996 254.0nm-1.2
Run Time:	11.5 Minutes	Channel Name:	W2996 254.0nm-1.2
Sample Set Name:	RIF_Basil EtOH Controls 2_1_17	Proc. Chnl. Descr.:	W2996 PDA 254.0 nm at 1.2

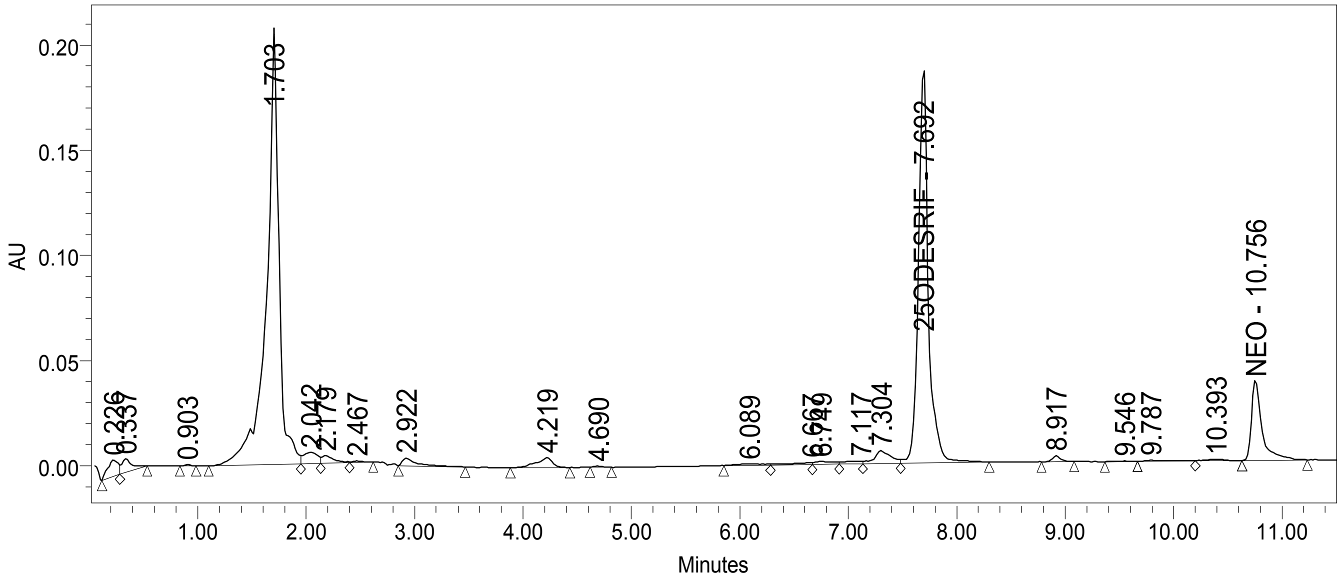

	Peak Name	RT	Area	% Area	Height
1		0.226	52947	1.51	7773
2		0.337	42611	1.21	6509
3		0.903	2268	0.06	620
4		1.703	1655165	47.07	200751
5		2.042	48201	1.37	5206
6		2.179	28539	0.81	3506
7		2.467	4613	0.13	710
8		2.922	31557	0.90	3641
9		4.219	49342	1.40	4650
10		4.690	2070	0.06	436
11		6.089	9746	0.28	577
12		6.667	12207	0.35	881

	Peak Name	RT	Area	% Area	Height
13		6.749	16239	0.46	1339
14		7.117	14574	0.41	1229
15		7.304	62785	1.79	6022
16	25ODESRIF	7.692	1196498	34.03	186344
17	RIF	8.100			
18		8.917	13386	0.38	2643
19		9.546	2860	0.08	247
20		9.787	7978	0.23	500
21		10.393	8428	0.24	665
22	NEO	10.756	254200	7.23	37919

SAMPLE INFORMATION

Sample Name:	RIF Inc Basil_EtOH C23 45min 2	Acquired By:	Saneesh
Sample Type:	Unknown	Date Acquired:	2017/02/02 06:16:13 AM
Vial:	24	Acq. Method Set:	HDI_RIF_25ODESRIF_NEO
Injection #:	1	Date Processed:	2017/02/02 10:20:53 AM
Injection Volume:	15.00 ul	Processing Method:	HDI_RIF_ W2996 254.0nm-1.2
Run Time:	11.5 Minutes	Channel Name:	W2996 254.0nm-1.2
Sample Set Name:	RIF_Basil EtOH Controls 2_1_17	Proc. Chnl. Descr.:	W2996 PDA 254.0 nm at 1.2

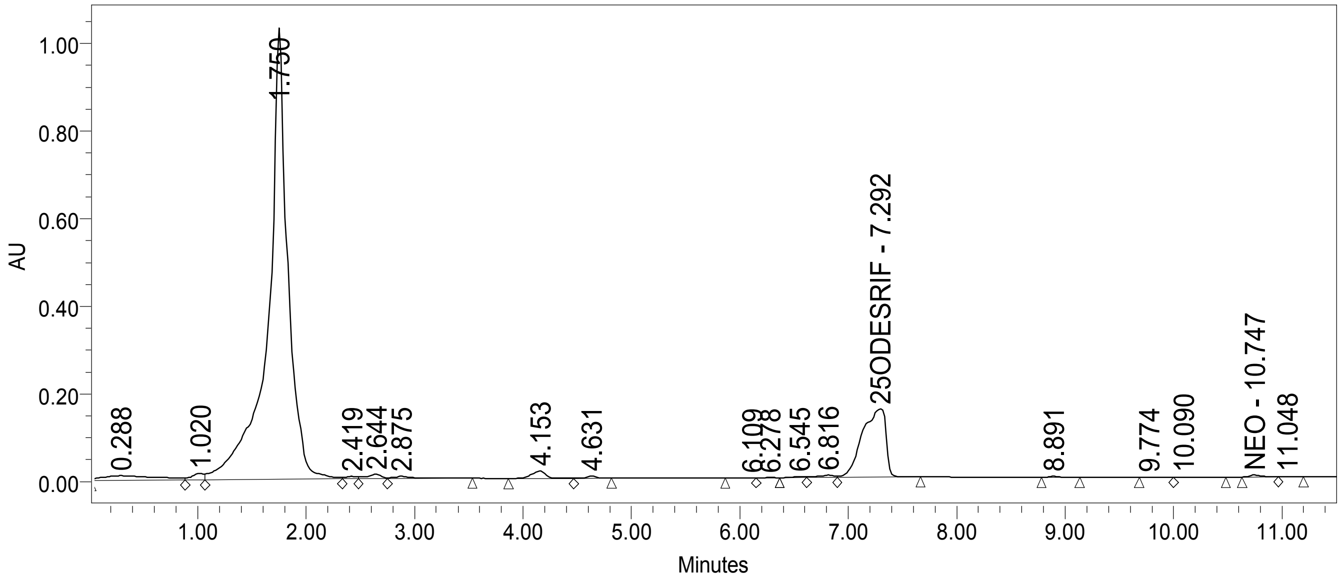

	Peak Name	RT	Area	% Area	Height
1		0.288	353026	2.14	10036
2		1.020	109242	0.66	14006
3		1.750	13103757	79.50	1024230
4		2.419	44416	0.27	5892
5		2.644	101507	0.62	10600
6		2.875	86574	0.53	5710
7		4.153	158422	0.96	17528
8		4.631	35327	0.21	5589
9		6.109	2651	0.02	250
10		6.278	3565	0.02	459
11		6.545	17627	0.11	2029
12		6.816	53512	0.32	5291

Reported by User: Saneesh (Saneesh) Project Name: HDI_Stellenbosch_2016

	Peak Name	RT	Area	% Area	Height
13	25ODESRIF	7.292	2355019	14.29	156894
14	RIF	8.100			
15		8.891	13125	0.08	2235
16		9.774	4058	0.02	346
17		10.090	3333	0.02	233
18	NEO	10.747	33093	0.20	4527
19		11.048	4879	0.03	629

Reported by User: Saneesh (Saneesh)

Project Name: HDI_Stellenbosch_2016

SAMPLE INFORMATION

Sample Name:	RIF Inc Basil_EtOH C24 45min 3	Acquired By:	Saneesh
Sample Type:	Unknown	Date Acquired:	2017/02/02 06:30:47 AM
Vial:	2	Acq. Method Set:	HDI_RIF_25ODESRIF_NEO
Injection #:	1	Date Processed:	2017/02/02 10:20:53 AM
Injection Volume:	15.00 ul	Processing Method:	HDI_RIF_ W2996 254.0nm-1.2
Run Time:	11.5 Minutes	Channel Name:	W2996 254.0nm-1.2
Sample Set Name:	RIF_Basil EtOH Controls 2_1_17	Proc. Chnl. Descr.:	W2996 PDA 254.0 nm at 1.2

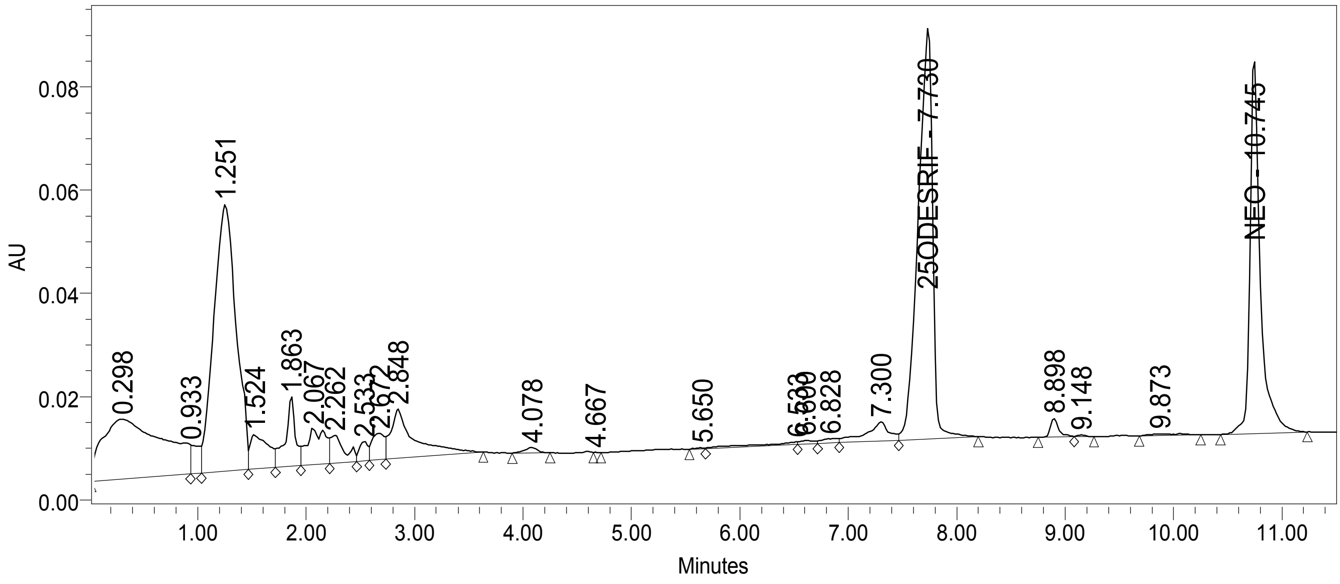

	Peak Name	RT	Area	% Area	Height
1		0.298	449460	14.67	11533
2		0.933	31782	1.04	5606
3		1.251	739570	24.15	51568
4		1.524	76509	2.50	6697
5		1.863	85416	2.79	13014
6		2.067	83867	2.74	6946
7		2.262	47434	1.55	5419
8		2.533	20765	0.68	3680
9		2.672	40514	1.32	5072
10		2.848	146117	4.77	9497
11		4.078	8891	0.29	1073
12		4.667	58	0.00	32

Reported by User: Saneesh (Saneesh) Project Name: HDI_Stellenbosch_2016

	Peak Name	RT	Area	% Area	Height
13		5.650	1168	0.04	161
14		6.533	15293	0.50	544
15		6.600	7048	0.23	765
16		6.828	9231	0.30	862
17		7.300	54874	1.79	3658
18	25ODESRIF	7.730	739534	24.14	79976
19	RIF	8.100			
20		8.898	19703	0.64	3610
21		9.148	2337	0.08	408
22		9.873	6719	0.22	319
23	NEO	10.745	476731	15.56	71716

SAMPLE INFORMATION

Sample Name:	RIF Inc Basil_EtOH C23 45min2 Rp	Acquired By:	Saneesh
Sample Type:	Unknown	Date Acquired:	2017/02/02 06:46:50 AM
Vial:	2	Acq. Method Set:	HDI_RIF_25ODESRIF_NEO
Injection #:	1	Date Processed:	2017/02/02 10:20:54 AM
Injection Volume:	15.00 ul	Processing Method:	HDI_RIF_ W2996 254.0nm-1.2
Run Time:	11.5 Minutes	Channel Name:	W2996 254.0nm-1.2
Sample Set Name:	RIF_Basil EtOH Controls 2_1_17	Proc. Chnl. Descr.:	W2996 PDA 254.0 nm at 1.2

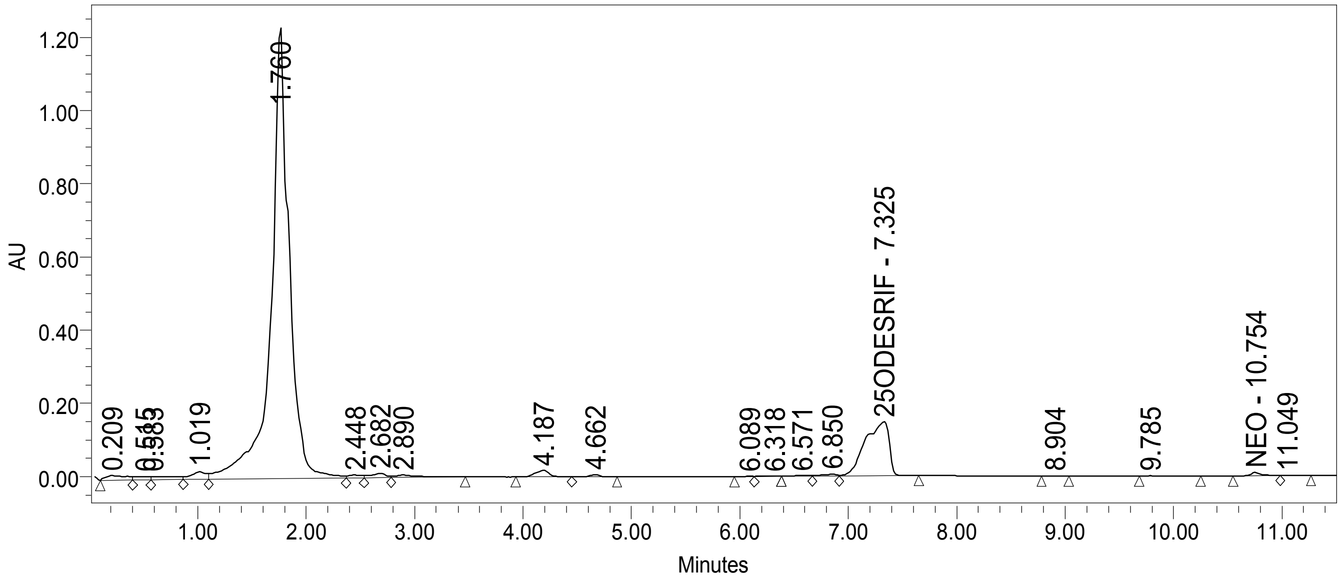

	Peak Name	RT	Area	% Area	Height
1		0.209	174100	0.98	13057
2		0.515	88293	0.50	8927
3		0.583	146828	0.83	8745
4		1.019	203148	1.14	20970
5		1.760	14273914	80.35	1220158
6		2.448	65271	0.37	7858
7		2.682	109523	0.62	11329
8		2.890	96133	0.54	6403
9		4.187	159552	0.90	18082
10		4.662	37388	0.21	5995
11		6.089	2167	0.01	292
12		6.318	4343	0.02	459

Reported by User: Saneesh (Saneesh)   Project Name: HDI_Stellenbosch_2016

	Peak Name	RT	Area	% Area	Height
13		6.571	18925	0.11	1943
14		6.850	48980	0.28	5321
15	25ODESRIF	7.325	2260854	12.73	148263
16	RIF	8.100			
17		8.904	3094	0.02	545
18		9.785	5307	0.03	360
19	NEO	10.754	60770	0.34	9083
20		11.049	6309	0.04	777

Multi Sample Summary

Reported by User: Saneesh (Saneesh) Project Name: HDI_Stellenbosch_2016

SAMPLE INFORMATION

Sample Name:	RIF Inc Basil_MeOH BMR1 0min 1	Acquired By:	Saneesh
Sample Type:	Unknown	Date Acquired:	2017/02/07 03:51:51 PM
Vial:	2	Acq. Method Set:	HDI_RIF_25ODESRIF_NEO
Injection #:	1	Date Processed:	2017/02/08 06:52:59 AM
Injection Volume:	15.00 ul	Processing Method:	HDI_RIF_ W2996 254.0nm-1.2
Run Time:	11.5 Minutes	Channel Name:	W2996 254.0nm-1.2
Sample Set Name:	RIF_Basil MeOH AssaySam 7_1_17	Proc. Chnl. Descr.:	W2996 PDA 254.0 nm at 1.2

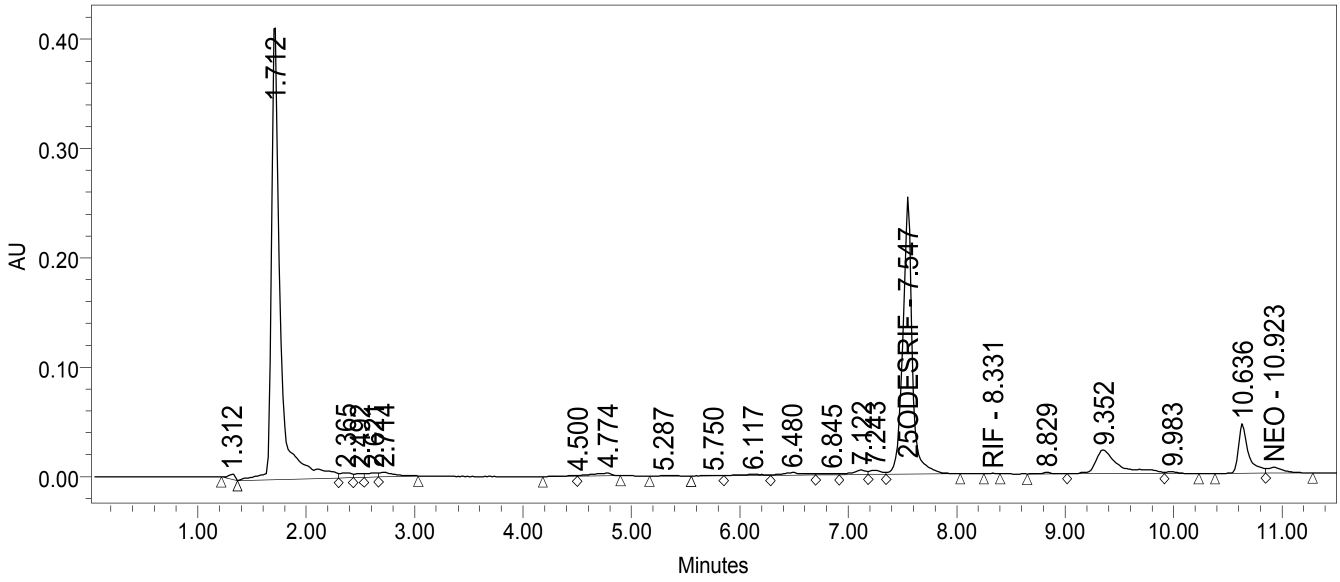

	Peak Name	RT	Area	% Area	Height
1		1.312	20048	0.41	4637
2		1.712	2381144	48.38	408605
3		2.365	32835	0.67	4572
4		2.492	20188	0.41	3736
5		2.621	28744	0.58	3751
6		2.714	38217	0.78	4275
7		4.500	6243	0.13	654
8		4.774	34059	0.69	2875
9		5.287	4541	0.09	313
10		5.750	2880	0.06	266
11		6.117	15255	0.31	853
12		6.480	33031	0.67	2395
13		6.845	13243	0.27	1239
14		7.122	35673	0.72	3985

Reported by User: Saneesh (Saneesh) Project Name: HDI_Stellenbosch_2016

	Peak Name	RT	Area	% Area	Height
15		7.243	28330	0.58	3797
16	25ODESRIF	7.547	1522783	30.94	251221
17	RIF	8.331	1851	0.04	441
18		8.829	11463	0.23	1552
19		9.352	329624	6.70	21756
20		9.983	14268	0.29	1894
21		10.636	292789	5.95	44989
22	NEO	10.923	54434	1.11	5453

SAMPLE INFORMATION

Sample Name:	RIF Inc Basil_MeOH BMR2 0min 2	Acquired By:	Saneesh
Sample Type:	Unknown	Date Acquired:	2017/02/07 04:06:47 PM
Vial:	3	Acq. Method Set:	HDI_RIF_25ODESRIF_NEO
Injection #:	1	Date Processed:	2017/02/08 06:52:59 AM
Injection Volume:	15.00 ul	Processing Method:	HDI_RIF_ W2996 254.0nm-1.2
Run Time:	11.5 Minutes	Channel Name:	W2996 254.0nm-1.2
Sample Set Name:	RIF_Basil MeOH AssaySam 7_1_17	Proc. Chnl. Descr.:	W2996 PDA 254.0 nm at 1.2

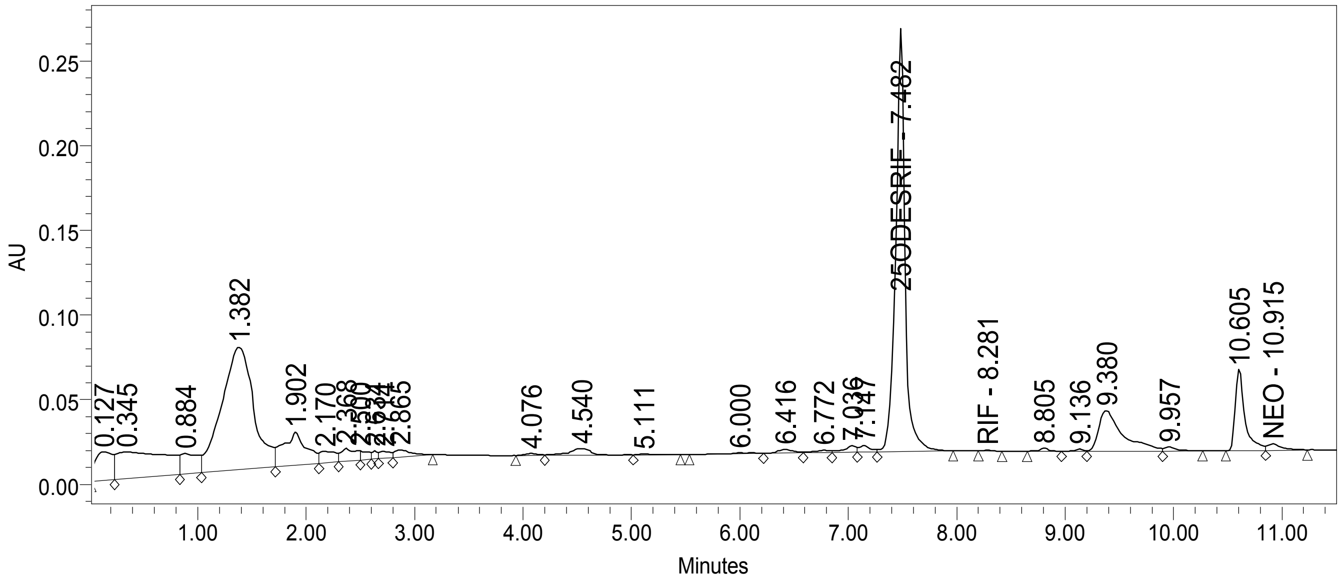

	Peak Name	RT	Area	% Area	Height
1		0.127	183774	3.50	16815
2		0.345	489282	9.31	15566
3		0.884	132735	2.53	11974
4		1.382	1486559	28.29	72178
5		1.902	289247	5.50	19451
6		2.170	67246	1.28	6777
7		2.368	71270	1.36	7495
8		2.500	27109	0.52	5319
9		2.634	16771	0.32	4480
10		2.714	28567	0.54	3880
11		2.865	42517	0.81	4166
12		4.076	9191	0.17	1158

Reported by User: Saneesh (Saneesh) Project Name: HDI_Stellenbosch_2016

	Peak Name	RT	Area	% Area	Height
13		4.540	55789	1.06	3961
14		5.111	6143	0.12	489
15		6.000	10609	0.20	633
16		6.416	23251	0.44	2393
17		6.772	14478	0.28	1476
18		7.036	29043	0.55	3737
19		7.147	27451	0.52	3804
20	25ODESRIF	7.482	1492716	28.41	245518
21	RIF	8.281	3324	0.06	638
22		8.805	13514	0.26	2022
23		9.136	7553	0.14	1352
24		9.380	370366	7.05	24052
25		9.957	20005	0.38	2349
26		10.605	302382	5.75	47723
27	NEO	10.915	33998	0.65	3803

SAMPLE INFORMATION

Sample Name:	RIF Inc Basil_MeOH BMR3 0min3	Acquired By:	Saneesh
Sample Type:	Unknown	Date Acquired:	2017/02/07 04:21:31 PM
Vial:	4	Acq. Method Set:	HDI_RIF_25ODESRIF_NEO
Injection #:	1	Date Processed:	2017/02/08 06:52:59 AM
Injection Volume:	15.00 ul	Processing Method:	HDI_RIF_ W2996 254.0nm-1.2
Run Time:	11.5 Minutes	Channel Name:	W2996 254.0nm-1.2
Sample Set Name:	RIF_Basil MeOH AssaySam 7_1_17	Proc. Chnl. Descr.:	W2996 PDA 254.0 nm at 1.2

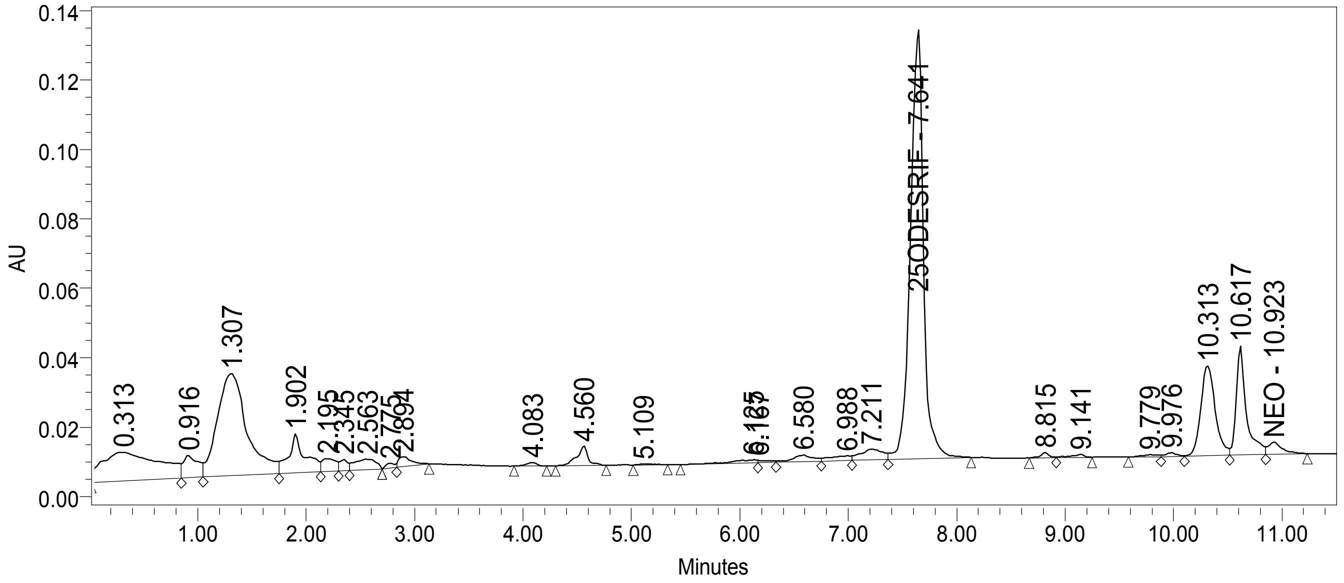

	Peak Name	RT	Area	% Area	Height
1		0.313	295484	10.34	8172
2		0.916	60545	2.12	6390
3		1.307	561247	19.64	29451
4		1.902	118179	4.13	11026
5		2.195	32339	1.13	3677
6		2.345	15968	0.56	3148
7		2.563	41886	1.47	3005
8		2.775	7522	0.26	1345
9		2.894	23013	0.81	2867
10		4.083	7989	0.28	1030
11		4.560	41259	1.44	5544
12		5.109	3347	0.12	306

	Peak Name	RT	Area	% Area	Height
13		6.125	13732	0.48	843
14		6.167	4855	0.17	645
15		6.580	24414	0.85	1869
16		6.988	18418	0.64	1336
17		7.211	44828	1.57	3100
18	25ODESRIF	7.641	1036920	36.28	125629
19	RIF	8.100			
20		8.815	8135	0.28	1433
21		9.141	8208	0.29	832
22		9.779	6982	0.24	655
23		9.976	7698	0.27	1026
24		10.313	239886	8.39	25950
25		10.617	203320	7.11	30882
26	NEO	10.923	31954	1.12	3500

SAMPLE INFORMATION

Sample Name:	RIF Inc Basil_MeOH BMR4 2min 1	Acquired By:	Saneesh
Sample Type:	Unknown	Date Acquired:	2017/02/07 04:36:16 PM
Vial:	5	Acq. Method Set:	HDI_RIF_25ODESRIF_NEO
Injection #:	1	Date Processed:	2017/02/08 06:52:59 AM
Injection Volume:	15.00 ul	Processing Method:	HDI_RIF_ W2996 254.0nm-1.2
Run Time:	11.5 Minutes	Channel Name:	W2996 254.0nm-1.2
Sample Set Name:	RIF_Basil MeOH AssaySam 7_1_17	Proc. Chnl. Descr.:	W2996 PDA 254.0 nm at 1.2

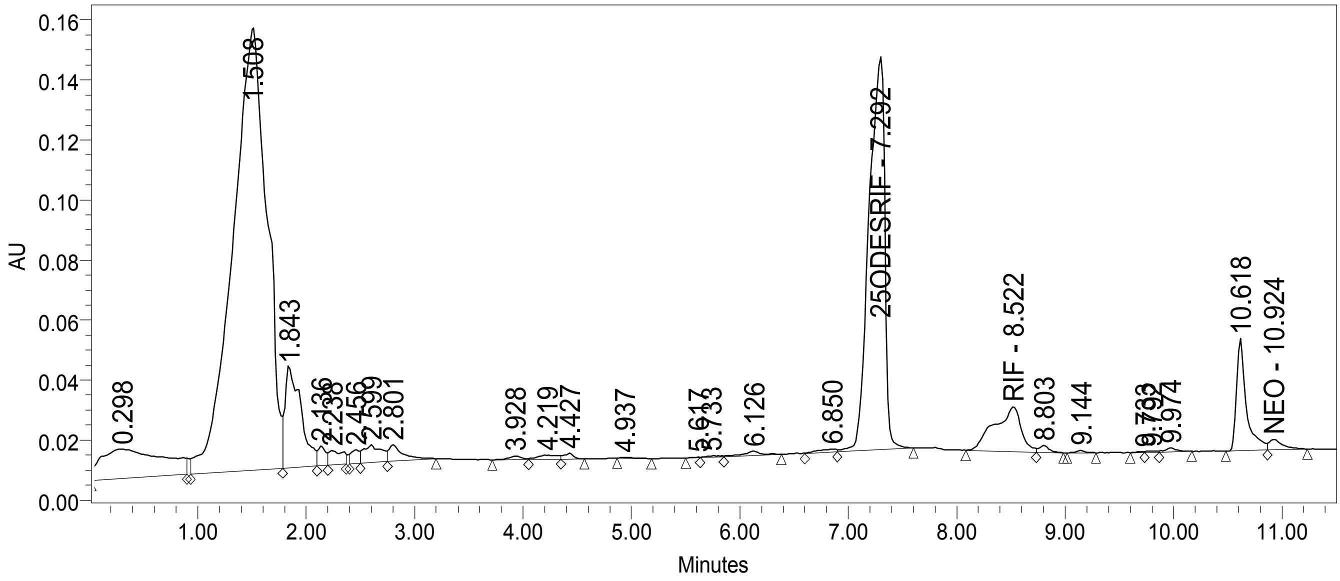

	Peak Name	RT	Area	% Area	Height
1		0.298	382737	6.27	9740
2		1.508	3208891	52.55	147693
3		1.843	349505	5.72	33549
4		2.136	32419	0.53	6376
5		2.238	41847	0.69	4815
6		2.456	24732	0.40	4570
7		2.599	67918	1.11	5704
8		2.801	49227	0.81	5428
9		3.928	9818	0.16	1159
10		4.219	17421	0.29	1315
11		4.427	10956	0.18	1853
12		4.937	2906	0.05	290

Reported by User: Saneesh (Saneesh) Project Name: HDI_Stellenbosch_2016

	Peak Name	RT	Area	% Area	Height
13		5.617	798	0.01	158
14		5.733	3500	0.06	374
15		6.126	16504	0.27	1405
16		6.850	11537	0.19	994
17	25ODESRIF	7.292	1321915	21.65	132818
18	RIF	8.522	252658	4.14	14915
19		8.803	14304	0.23	2201
20		9.144	4276	0.07	805
21		9.733	1486	0.02	261
22		9.792	3084	0.05	428
23		9.974	10096	0.17	1352
24		10.618	237314	3.89	36965
25	NEO	10.924	30867	0.51	3552

SAMPLE INFORMATION

Sample Name:	RIF Inc Basil_MeOH BMR5 2min 2	Acquired By:	Saneesh
Sample Type:	Unknown	Date Acquired:	2017/02/07 04:51:01 PM
Vial:	6	Acq. Method Set:	HDI_RIF_25ODESRIF_NEO
Injection #:	1	Date Processed:	2017/02/08 06:52:59 AM
Injection Volume:	15.00 ul	Processing Method:	HDI_RIF_ W2996 254.0nm-1.2
Run Time:	11.5 Minutes	Channel Name:	W2996 254.0nm-1.2
Sample Set Name:	RIF_Basil MeOH AssaySam 7_1_17	Proc. Chnl. Descr.:	W2996 PDA 254.0 nm at 1.2

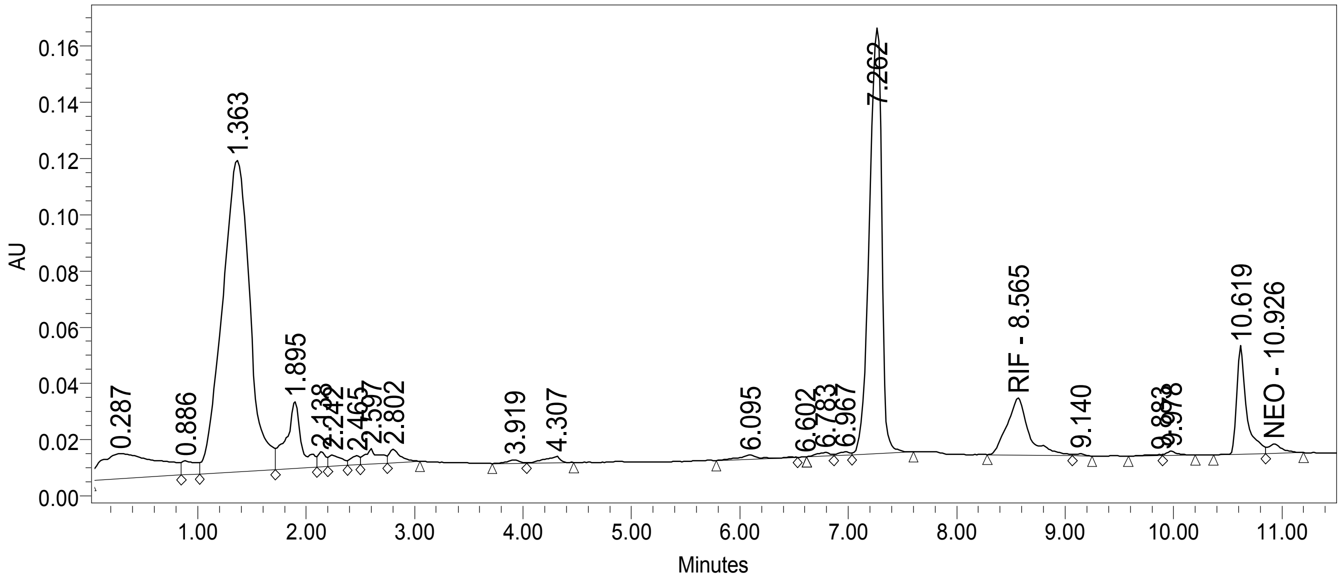

	Peak Name	RT	Area	% Area	Height
1		0.287	324937	7.10	8862
2		0.886	44467	0.97	4895
3		1.363	1962404	42.88	110786
4		1.895	236305	5.16	23994
5		2.138	26328	0.58	5393
6		2.242	32828	0.72	3963
7		2.465	19082	0.42	3266
8		2.597	51670	1.13	5042
9		2.802	34740	0.76	4754
10		3.919	9322	0.20	1149
11		4.307	21397	0.47	2224
12		6.095	18252	0.40	1495

Reported by User: Saneesh (Saneesh) Project Name: HDI_Stellenbosch_2016

	Peak Name	RT	Area	% Area	Height
13		6.602	110	0.00	26
14		6.783	11351	0.25	1261
15		6.967	8870	0.19	1200
16		7.262	1194565	26.10	152727
17	25ODESRIF	7.800			
18	RIF	8.565	282169	6.17	20270
19		9.140	4884	0.11	886
20		9.883	4718	0.10	395
21		9.978	10548	0.23	1480
22		10.619	247142	5.40	38231
23	NEO	10.926	30486	0.67	3352

Multi Sample Summary

Reported by User: Saneesh (Saneesh)   Project Name: HDI_Stellenbosch_2016

SAMPLE INFORMATION

Sample Name:	RIF Inc Basil_MeOH BMR6 2min 3	Acquired By:	Saneesh
Sample Type:	Unknown	Date Acquired:	2017/02/07 05:05:46 PM
Vial:	7	Acq. Method Set:	HDI_RIF_25ODESRIF_NEO
Injection #:	1	Date Processed:	2017/02/08 06:52:59 AM
Injection Volume:	15.00 ul	Processing Method:	HDI_RIF_ W2996 254.0nm-1.2
Run Time:	11.5 Minutes	Channel Name:	W2996 254.0nm-1.2
Sample Set Name:	RIF_Basil MeOH AssaySam 7_1_17	Proc. Chnl. Descr.:	W2996 PDA 254.0 nm at 1.2

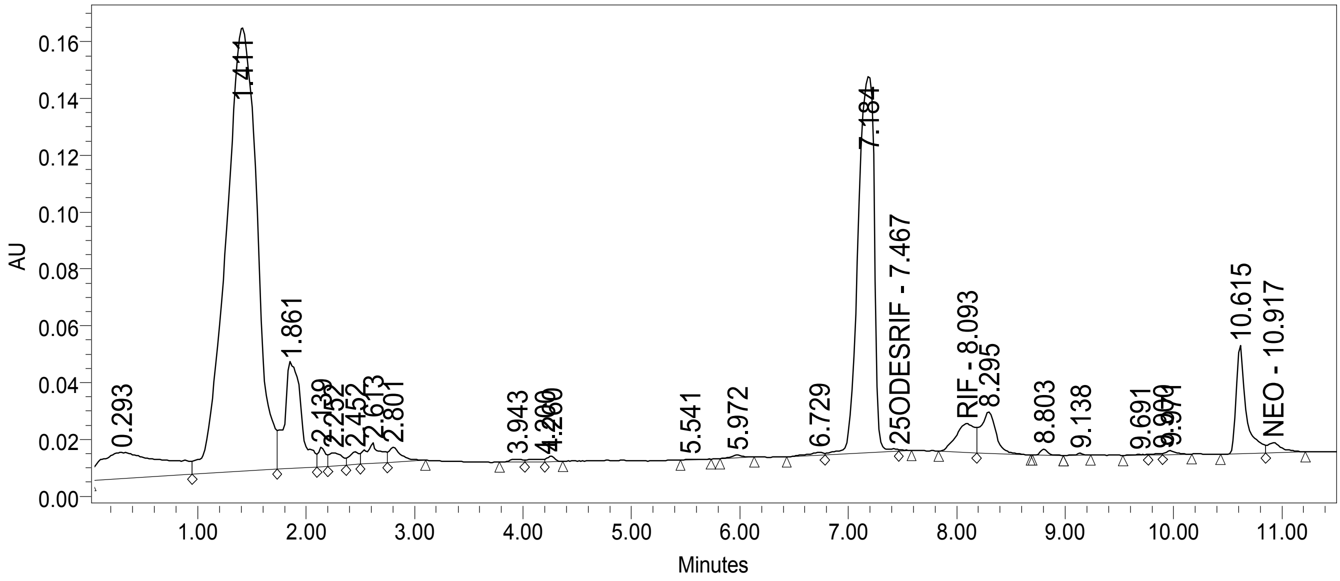

	Peak Name	RT	Area	% Area	Height
1		0.293	369540	6.06	9140
2		1.411	3107137	50.94	156320
3		1.861	410113	6.72	38279
4		2.139	31944	0.52	6474
5		2.252	38419	0.63	4572
6		2.452	29278	0.48	4382
7		2.613	70061	1.15	6681
8		2.801	42281	0.69	5283
9		3.943	7851	0.13	1026
10		4.200	8967	0.15	939
11		4.260	9353	0.15	1863
12		5.541	1377	0.02	138

	Peak Name	RT	Area	% Area	Height
13		5.972	6082	0.10	890
14		6.729	11241	0.18	1103
15		7.184	1380593	22.63	132463
16	25ODESRIF	7.467	1739	0.03	584
17	RIF	8.093	122499	2.01	10122
18		8.295	144809	2.37	14589
19		8.803	11258	0.18	2204
20		9.138	3696	0.06	768
21		9.691	1692	0.03	209
22		9.900	2265	0.04	392
23		9.971	8323	0.14	1287
24		10.615	245959	4.03	37665
25	NEO	10.917	33427	0.55	3602

Reported by User: Saneesh (Saneesh) Project Name: HDI_Stellenbosch_2016

SAMPLE INFORMATION

Sample Name:	RIF Inc Basil_MeOH BMR7 5min 1	Acquired By:	Saneesh
Sample Type:	Unknown	Date Acquired:	2017/02/07 05:23:14 PM
Vial:	8	Acq. Method Set:	HDI_RIF_25ODESRIF_NEO
Injection #:	1	Date Processed:	2017/02/08 06:52:59 AM
Injection Volume:	15.00 ul	Processing Method:	HDI_RIF_ W2996 254.0nm-1.2
Run Time:	11.5 Minutes	Channel Name:	W2996 254.0nm-1.2
Sample Set Name:	RIF_Basil MeOH AssaySam 7_1_17	Proc. Chnl. Descr.:	W2996 PDA 254.0 nm at 1.2

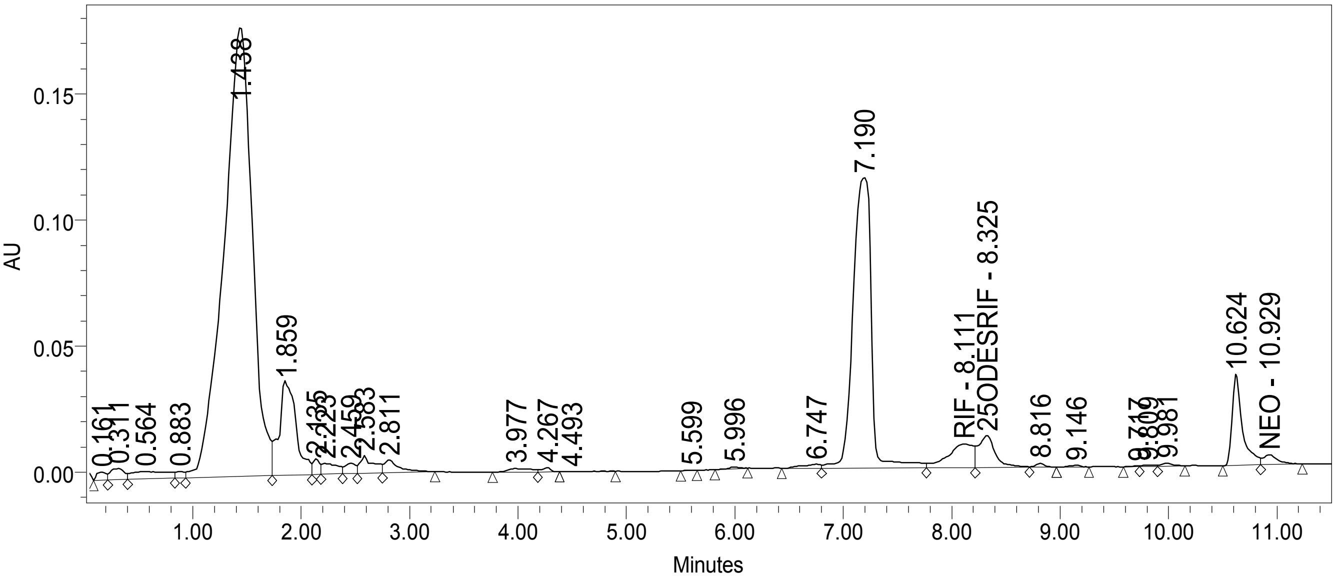

	Peak Name	RT	Area	% Area	Height
1		0.161	19158	0.32	3095
2		0.311	39327	0.66	4508
3		0.564	68979	1.15	2902
4		0.883	15002	0.25	2651
5		1.438	3239807	53.97	178117
6		1.859	409653	6.82	37988
7		2.135	25660	0.43	6045
8		2.223	44034	0.73	4314
9		2.459	28441	0.47	4129
10		2.583	60304	1.00	6588
11		2.811	47578	0.79	4954
12		3.977	20958	0.35	1468

Reported by User: Saneesh (Saneesh) Project Name: HDI_Stellenbosch_2016

	Peak Name	RT	Area	% Area	Height
13		4.267	9314	0.16	1640
14		4.493	2653	0.04	168
15		5.599	1232	0.02	187
16		5.996	5458	0.09	785
17		6.747	17796	0.30	1523
18		7.190	1357496	22.62	115766
19	RIF	8.111	151689	2.53	9299
20	25ODESRIF	8.325	133528	2.22	12484
21		8.816	8345	0.14	1503
22		9.146	4807	0.08	682
23		9.717	1452	0.02	266
24		9.809	3939	0.07	470
25		9.981	7917	0.13	1183
26		10.624	240244	4.00	36300
27	NEO	10.929	37675	0.63	4010

SAMPLE INFORMATION

Sample Name:	RIF Inc Basil_MeOH BMR8 5min 2	Acquired By:	Saneesh
Sample Type:	Unknown	Date Acquired:	2017/02/07 05:37:50 PM
Vial:	9	Acq. Method Set:	HDI_RIF_25ODESRIF_NEO
Injection #:	1	Date Processed:	2017/02/08 06:52:59 AM
Injection Volume:	15.00 ul	Processing Method:	HDI_RIF_ W2996 254.0nm-1.2
Run Time:	11.5 Minutes	Channel Name:	W2996 254.0nm-1.2
Sample Set Name:	RIF_Basil MeOH AssaySam 7_1_17	Proc. Chnl. Descr.:	W2996 PDA 254.0 nm at 1.2

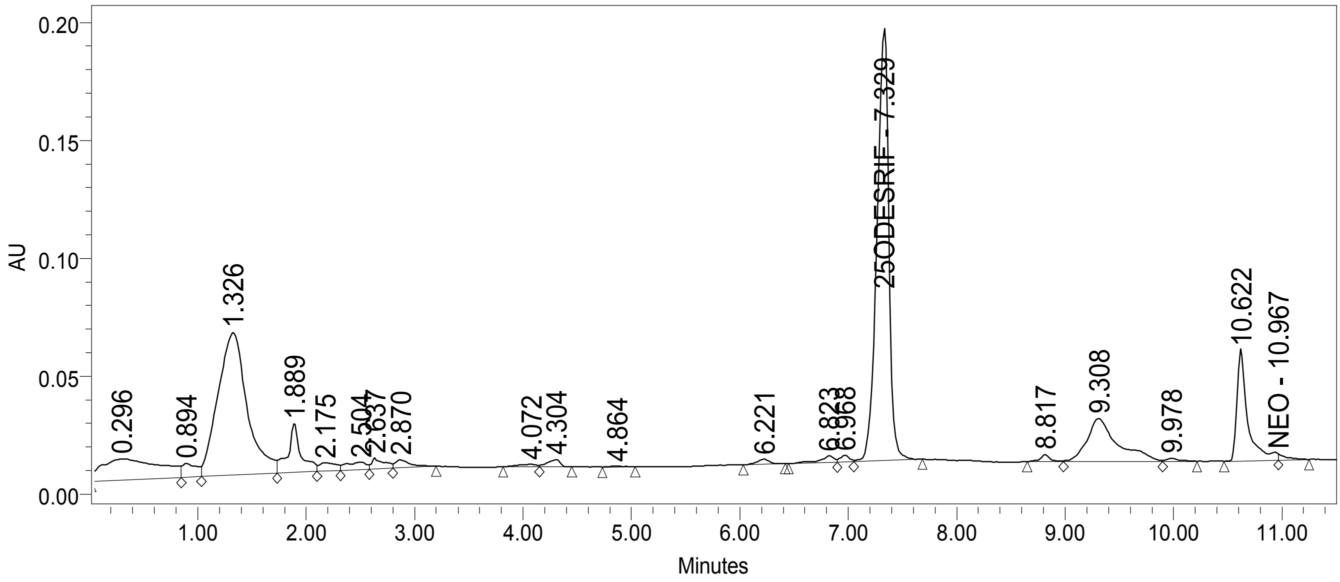

	Peak Name	RT	Area	% Area	Height
1		0.296	326405	8.02	8888
2		0.894	53447	1.31	5798
3		1.326	1135555	27.90	60550
4		1.889	174858	4.30	21216
5		2.175	38588	0.95	3570
6		2.504	42541	1.05	3155
7		2.637	37299	0.92	4586
8		2.870	33916	0.83	3375
9		4.072	13784	0.34	1151
10		4.304	25588	0.63	3202
11		4.864	3830	0.09	417
12		6.221	16233	0.40	2129

Reported by User: Saneesh (Saneesh) Project Name: HDI_Stellenbosch_2016

	Peak Name	RT	Area	% Area	Height
13		6.823	23639	0.58	2608
14		6.968	15259	0.37	2674
15	25ODESRIF	7.329	1394696	34.27	185708
16	RIF	8.100			
17		8.817	20106	0.49	3090
18		9.308	360210	8.85	18312
19		9.978	11366	0.28	1396
20		10.622	329316	8.09	47166
21	NEO	10.967	13396	0.33	2703

SAMPLE INFORMATION

Sample Name:	RIF Inc Basil_MeOH BMR9 5min 3	Acquired By:	Saneesh
Sample Type:	Unknown	Date Acquired:	2017/02/07 05:52:36 PM
Vial:	10	Acq. Method Set:	HDI_RIF_25ODESRIF_NEO
Injection #:	1	Date Processed:	2017/02/08 06:52:59 AM
Injection Volume:	15.00 ul	Processing Method:	HDI_RIF_ W2996 254.0nm-1.2
Run Time:	11.5 Minutes	Channel Name:	W2996 254.0nm-1.2
Sample Set Name:	RIF_Basil MeOH AssaySam 7_1_17	Proc. Chnl. Descr.:	W2996 PDA 254.0 nm at 1.2

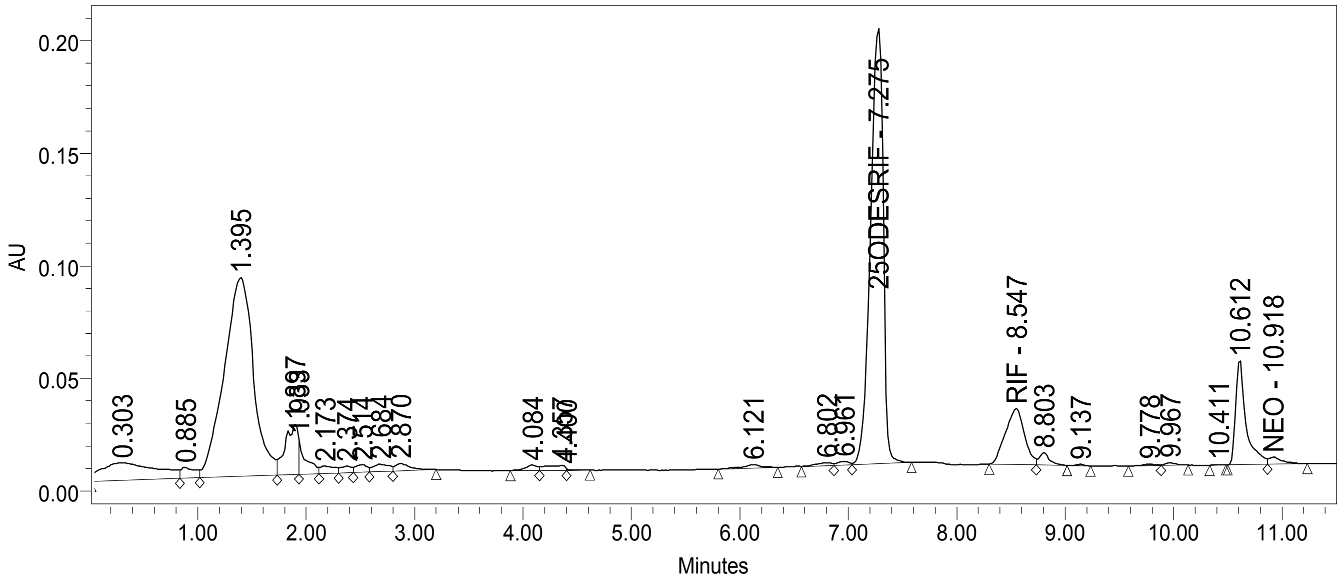

	Peak Name	RT	Area	% Area	Height
1		0.303	267901	5.67	7670
2		0.885	42252	0.89	4742
3		1.395	1661559	35.15	88408
4		1.897	181363	3.84	22338
5		1.933	66282	1.40	15598
6		2.173	30119	0.64	3113
7		2.374	19630	0.42	2723
8		2.514	24132	0.51	3188
9		2.684	32832	0.69	3203
10		2.870	30891	0.65	3208
11		4.084	19777	0.42	2454
12		4.357	28732	0.61	2305

	Peak Name	RT	Area	% Area	Height
13		4.400	2040	0.04	941
14		6.121	16819	0.36	1489
15		6.802	12600	0.27	1246
16		6.961	11603	0.25	1547
17	25ODESRIF	7.275	1579301	33.41	195532
18	RIF	8.547	302835	6.41	24913
19		8.803	37930	0.80	5568
20		9.137	3376	0.07	687
21		9.778	7357	0.16	760
22		9.967	8397	0.18	1239
23		10.411	946	0.02	195
24		10.612	311235	6.58	46017
25	NEO	10.918	27025	0.57	3077

SAMPLE INFORMATION

Sample Name:	RIF Inc Basil_MeOH BMR10 10min 1	Acquired By:	Saneesh
Sample Type:	Unknown	Date Acquired:	2017/02/07 06:07:20 PM
Vial:	11	Acq. Method Set:	HDI_RIF_25ODESRIF_NEO
Injection #:	1	Date Processed:	2017/02/08 06:52:59 AM
Injection Volume:	15.00 ul	Processing Method:	HDI_RIF_ W2996 254.0nm-1.2
Run Time:	11.5 Minutes	Channel Name:	W2996 254.0nm-1.2
Sample Set Name:	RIF_Basil MeOH AssaySam 7_1_17	Proc. Chnl. Descr.:	W2996 PDA 254.0 nm at 1.2

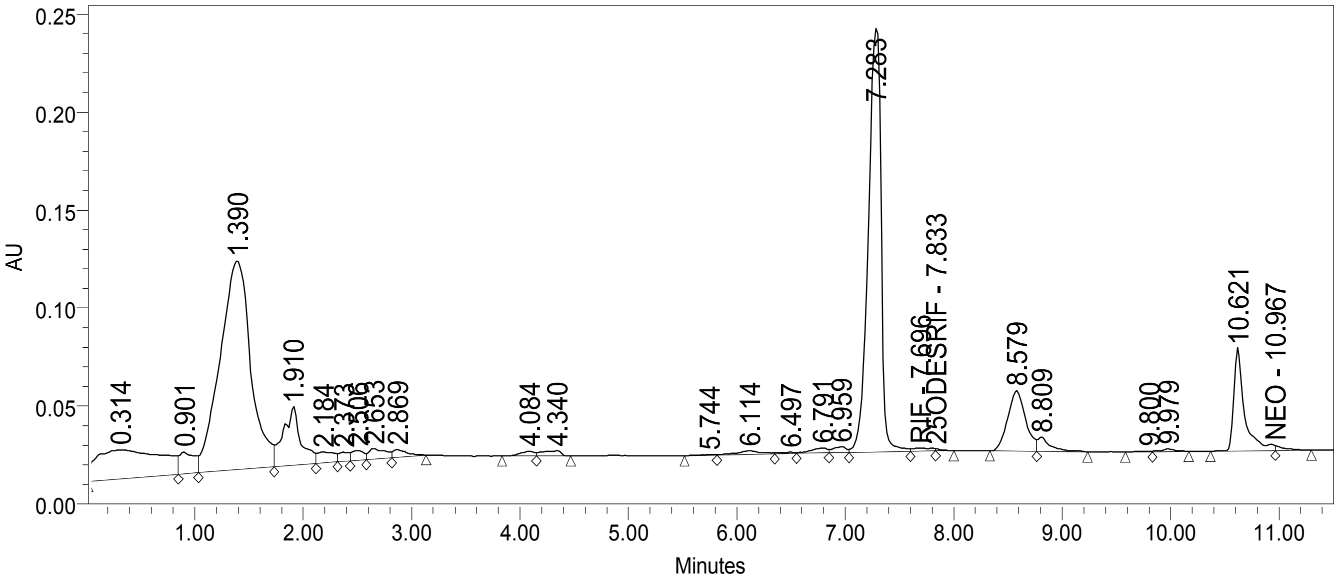

	Peak Name	RT	Area	% Area	Height
1		0.314	599407	9.81	14690
2		0.901	106060	1.74	11082
3		1.390	2102447	34.40	106454
4		1.910	336955	5.51	29657
5		2.184	59667	0.98	5462
6		2.373	30230	0.49	4594
7		2.506	38463	0.63	4774
8		2.653	54910	0.90	5253
9		2.869	32886	0.54	3758
10		4.084	21083	0.34	2340
11		4.340	30581	0.50	2642
12		5.744	3216	0.05	264

	Peak Name	RT	Area	% Area	Height
13		6.114	28353	0.46	1893
14		6.497	7317	0.12	748
15		6.791	27912	0.46	2591
16		6.959	25408	0.42	2927
17		7.283	1773066	29.01	217463
18	RIF	7.696	19467	0.32	1450
19	25ODESRIF	7.833	4123	0.07	1135
20		8.579	354809	5.81	30910
21		8.809	63110	1.03	7333
22		9.800	3053	0.05	337
23		9.979	11314	0.19	1355
24		10.621	364308	5.96	52272
25	NEO	10.967	13353	0.22	2600

SAMPLE INFORMATION

Sample Name:	RIF Inc Basil_MeOH BMR11 10min 2	Acquired By:	Saneesh
Sample Type:	Unknown	Date Acquired:	2017/02/07 06:22:05 PM
Vial:	12	Acq. Method Set:	HDI_RIF_25ODESRIF_NEO
Injection #:	1	Date Processed:	2017/02/08 06:52:59 AM
Injection Volume:	15.00 ul	Processing Method:	HDI_RIF_ W2996 254.0nm-1.2
Run Time:	11.5 Minutes	Channel Name:	W2996 254.0nm-1.2
Sample Set Name:	RIF_Basil MeOH AssaySam 7_1_17	Proc. Chnl. Descr.:	W2996 PDA 254.0 nm at 1.2

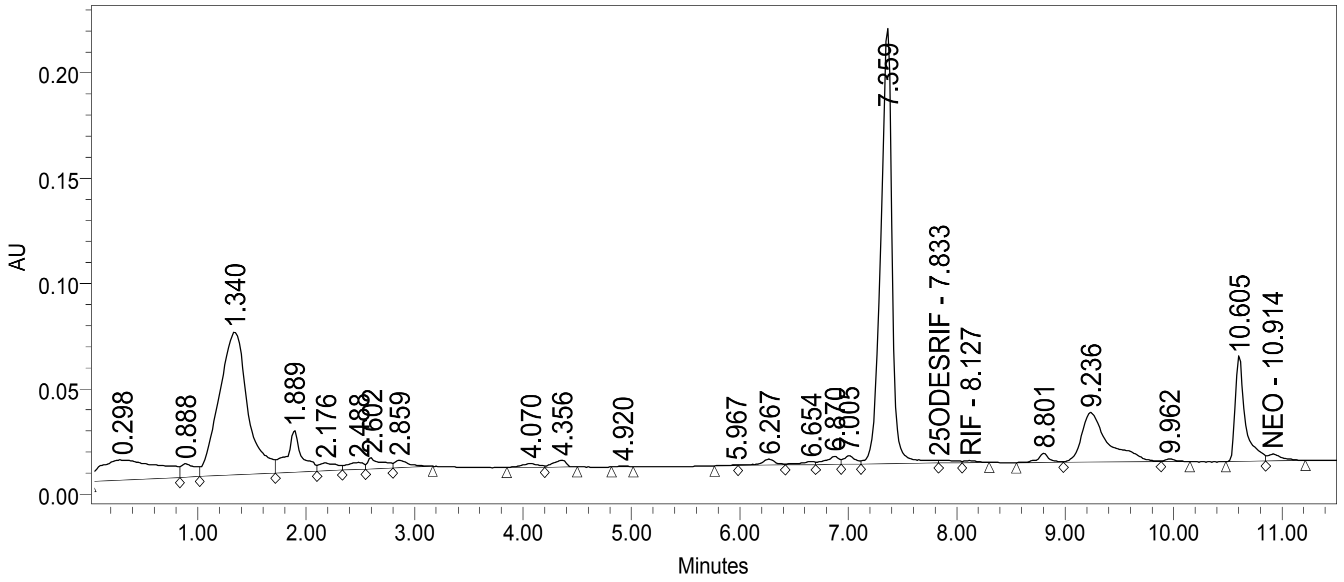

	Peak Name	RT	Area	% Area	Height
1		0.298	347833	7.76	9480
2		0.888	58483	1.30	6282
3		1.340	1254711	27.98	67732
4		1.889	196861	4.39	19859
5		2.176	43173	0.96	3738
6		2.488	36927	0.82	3298
7		2.602	48754	1.09	5211
8		2.859	32615	0.73	3462
9		4.070	19624	0.44	1887
10		4.356	26223	0.58	3269
11		4.920	3315	0.07	519
12		5.967	2062	0.05	258

Reported by User: Saneesh (Saneesh)   Project Name: HDI_Stellenbosch_2016

	Peak Name	RT	Area	% Area	Height
13		6.267	26233	0.58	2799
14		6.654	14681	0.33	1444
15		6.870	31725	0.71	3759
16		7.005	29449	0.66	4045
17		7.359	1491369	33.25	210790
18	25ODESRIF	7.833	11700	0.26	1228
19	RIF	8.127	6620	0.15	813
20		8.801	31292	0.70	4390
21		9.236	404245	9.01	23725
22		9.962	9659	0.22	1381
23		10.605	325831	7.27	49845
24	NEO	10.914	31454	0.70	3315

SAMPLE INFORMATION

Sample Name:	RIF Inc Basil_MeOH BMR12 10min 3	Acquired By:	Saneesh
Sample Type:	Unknown	Date Acquired:	2017/02/07 06:36:52 PM
Vial:	13	Acq. Method Set:	HDI_RIF_25ODESRIF_NEO
Injection #:	1	Date Processed:	2017/02/08 06:52:59 AM
Injection Volume:	15.00 ul	Processing Method:	HDI_RIF_ W2996 254.0nm-1.2
Run Time:	11.5 Minutes	Channel Name:	W2996 254.0nm-1.2
Sample Set Name:	RIF_Basil MeOH AssaySam 7_1_17	Proc. Chnl. Descr.:	W2996 PDA 254.0 nm at 1.2

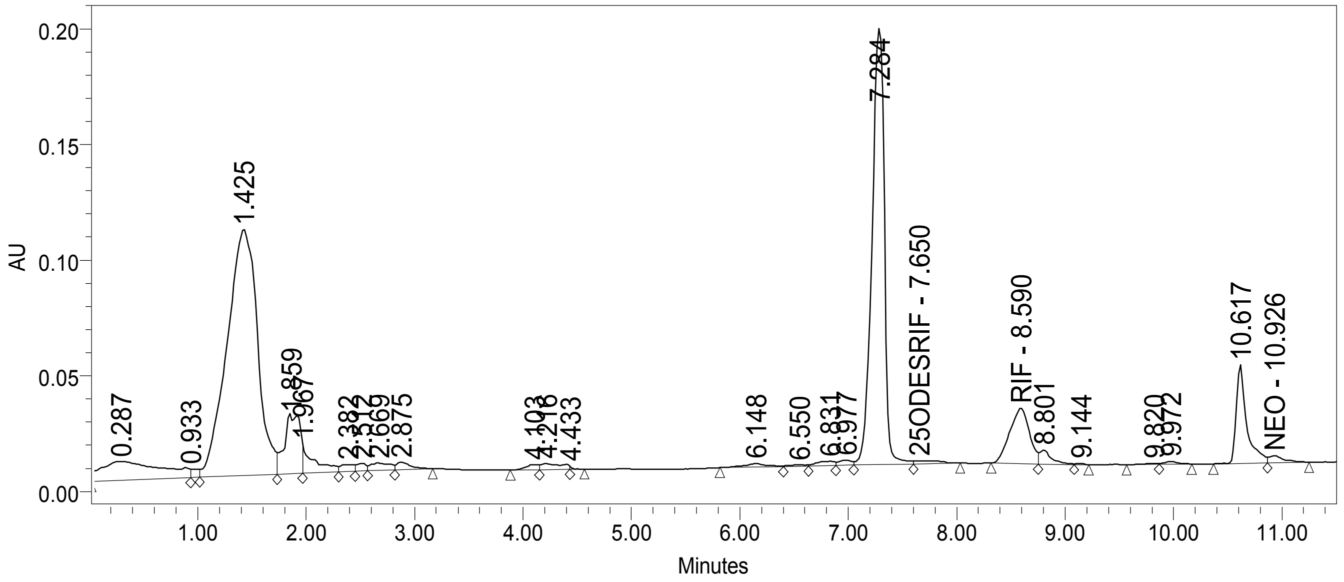

	Peak Name	RT	Area	% Area	Height
1		0.287	304837	5.88	8001
2		0.933	17295	0.33	3632
3		1.425	2083832	40.20	106371
4		1.859	256035	4.94	25738
5		1.967	93479	1.80	12683
6		2.382	25864	0.50	3081
7		2.512	21147	0.41	3348
8		2.669	41220	0.80	3279
9		2.875	29308	0.57	3275
10		4.103	18644	0.36	2265
11		4.216	36203	0.70	2616
12		4.433	1820	0.04	1394

Reported by User: Saneesh (Saneesh)   Project Name: HDI_Stellenbosch_2016

	Peak Name	RT	Area	% Area	Height
13		6.148	20587	0.40	1638
14		6.550	5592	0.11	519
15		6.831	20890	0.40	1865
16		6.977	18411	0.36	2275
17		7.284	1490513	28.75	188994
18	25ODESRIF	7.650	20789	0.40	1290
19	RIF	8.590	298660	5.76	23884
20		8.801	47157	0.91	6046
21		9.144	2566	0.05	558
22		9.820	4889	0.09	493
23		9.972	9649	0.19	1186
24		10.617	285616	5.51	41999
25	NEO	10.926	28839	0.56	3138

SAMPLE INFORMATION

Sample Name:	RIF Inc Basil_MeOH BMR13 15min 1	Acquired By:	Saneesh
Sample Type:	Unknown	Date Acquired:	2017/02/07 06:51:37 PM
Vial:	14	Acq. Method Set:	HDI_RIF_25ODESRIF_NEO
Injection #:	1	Date Processed:	2017/02/08 06:52:59 AM
Injection Volume:	15.00 ul	Processing Method:	HDI_RIF_ W2996 254.0nm-1.2
Run Time:	11.5 Minutes	Channel Name:	W2996 254.0nm-1.2
Sample Set Name:	RIF_Basil MeOH AssaySam 7_1_17	Proc. Chnl. Descr.:	W2996 PDA 254.0 nm at 1.2

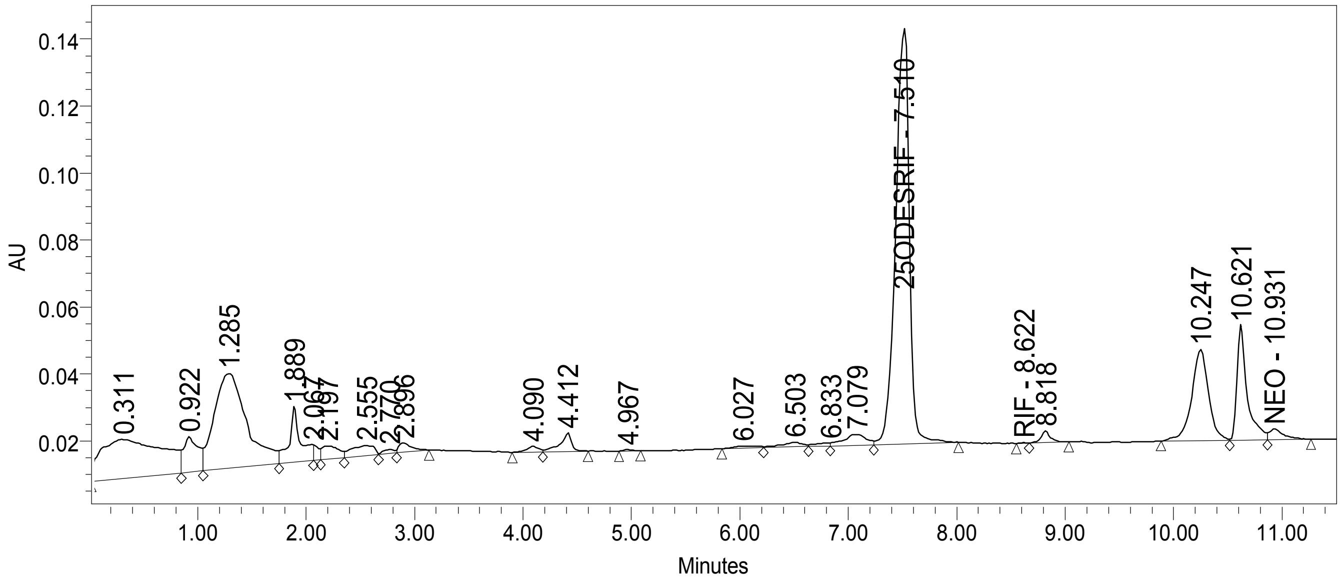

	Peak Name	RT	Area	% Area	Height
1		0.311	449809	14.04	11569
2		0.922	98028	3.06	10485
3		1.285	591299	18.45	28317
4		1.889	124599	3.89	16240
5		2.067	15603	0.49	4640
6		2.197	41829	1.31	4001
7		2.555	46409	1.45	3056
8		2.770	9099	0.28	1246
9		2.896	21554	0.67	2752
10		4.090	13006	0.41	1657
11		4.412	41209	1.29	5462
12		4.967	2835	0.09	485

Reported by User: Saneesh (Saneesh) Project Name: HDI_Stellenbosch_2016

	Peak Name	RT	Area	% Area	Height
13		6.027	10205	0.32	705
14		6.503	18811	0.59	1356
15		6.833	8577	0.27	825
16		7.079	46749	1.46	3204
17	25ODESRIF	7.510	1097569	34.26	125012
18	RIF	8.622	722	0.02	186
19		8.818	21861	0.68	3475
20		10.247	291374	9.09	27089
21		10.621	224204	7.00	34212
22	NEO	10.931	28712	0.90	3180

Multi Sample Summary

Reported by User: Saneesh (Saneesh) Project Name: HDI_Stellenbosch_2016

SAMPLE INFORMATION

Sample Name:	RIF Inc Basil_MeOH BMR14 15min 2	Acquired By:	Saneesh
Sample Type:	Unknown	Date Acquired:	2017/02/07 07:06:22 PM
Vial:	15	Acq. Method Set:	HDI_RIF_25ODESRIF_NEO
Injection #:	1	Date Processed:	2017/02/08 06:52:59 AM
Injection Volume:	15.00 ul	Processing Method:	HDI_RIF_ W2996 254.0nm-1.2
Run Time:	11.5 Minutes	Channel Name:	W2996 254.0nm-1.2
Sample Set Name:	RIF_Basil MeOH AssaySam 7_1_17	Proc. Chnl. Descr.:	W2996 PDA 254.0 nm at 1.2

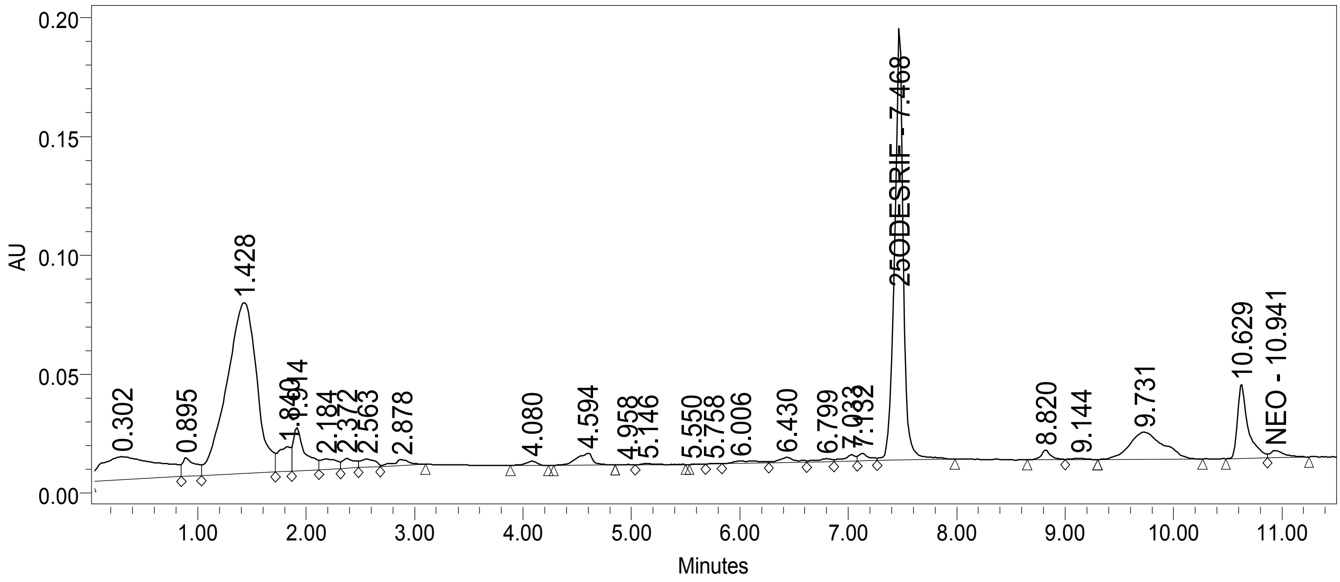

	Peak Name	RT	Area	% Area	Height
1		0.302	354322	9.18	9600
2		0.895	63714	1.65	7571
3		1.428	1383245	35.85	71952
4		1.840	83849	2.17	10090
5		1.914	138152	3.58	17939
6		2.184	45799	1.19	4287
7		2.372	32861	0.85	4203
8		2.563	31986	0.83	3398
9		2.878	25738	0.67	2487
10		4.080	13306	0.34	1781
11		4.594	53754	1.39	5124
12		4.958	1531	0.04	253

Reported by User: Saneesh (Saneesh) Project Name: HDI_Stellenbosch_2016

	Peak Name	RT	Area	% Area	Height
13		5.146	5878	0.15	506
14		5.550	505	0.01	117
15		5.758	984	0.03	161
16		6.006	16134	0.42	1023
17		6.430	21244	0.55	2137
18		6.799	12511	0.32	1240
19		7.033	20729	0.54	2724
20		7.132	21178	0.55	3092
21	25ODESRIF	7.468	1014069	26.28	179363
22	RIF	8.100			
23		8.820	24468	0.63	4021
24		9.144	4445	0.12	536
25		9.731	246608	6.39	11345
26		10.629	213081	5.52	30922
27	NEO	10.941	28291	0.73	3107

Reported by User: Saneesh (Saneesh) Project Name: HDI_Stellenbosch_2016

SAMPLE INFORMATION

Sample Name:	RIF Inc Basil_MeOH BMR15 15min 3	Acquired By:	Saneesh
Sample Type:	Unknown	Date Acquired:	2017/02/07 07:21:07 PM
Vial:	16	Acq. Method Set:	HDI_RIF_25ODESRIF_NEO
Injection #:	1	Date Processed:	2017/02/08 06:52:59 AM
Injection Volume:	15.00 ul	Processing Method:	HDI_RIF_ W2996 254.0nm-1.2
Run Time:	11.5 Minutes	Channel Name:	W2996 254.0nm-1.2
Sample Set Name:	RIF_Basil MeOH AssaySam 7_1_17	Proc. Chnl. Descr.:	W2996 PDA 254.0 nm at 1.2

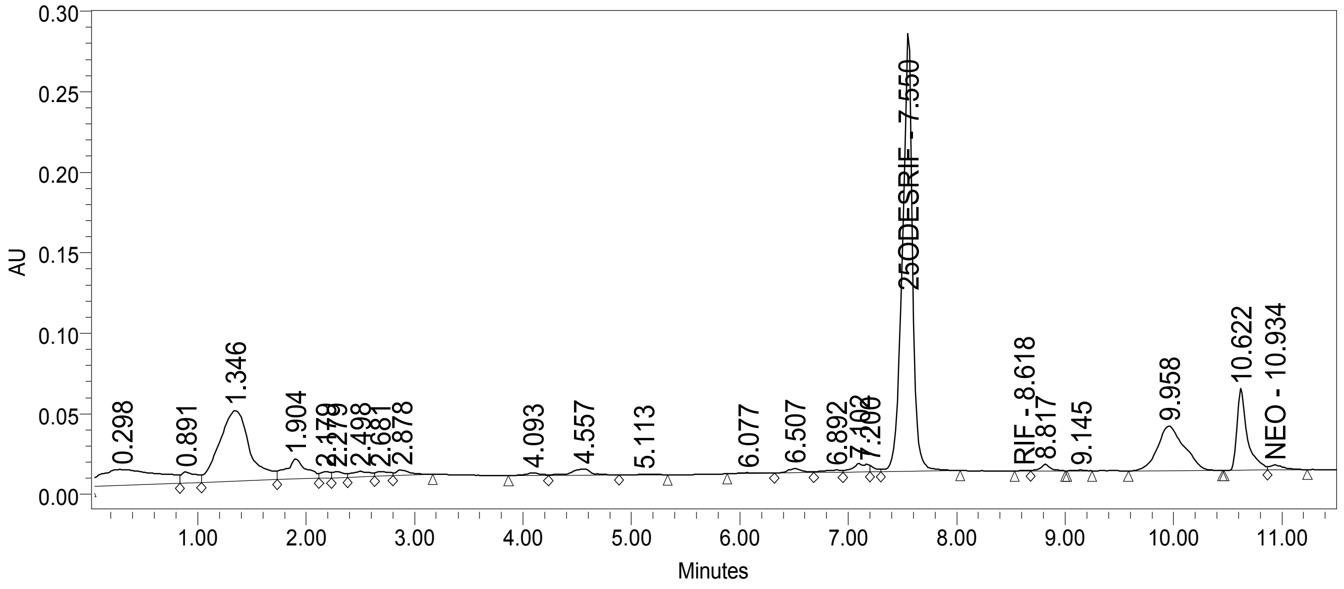

	Peak Name	RT	Area	% Area	Height
1		0.298	358578	8.09	9766
2		0.891	66939	1.51	6738
3		1.346	852916	19.25	43809
4		1.904	161946	3.65	12521
5		2.179	25063	0.57	3900
6		2.279	27866	0.63	3712
7		2.498	40097	0.90	3393
8		2.681	23399	0.53	2731
9		2.878	31480	0.71	3292
10		4.093	17302	0.39	1726
11		4.557	54483	1.23	4006
12		5.113	6006	0.14	484

	Peak Name	RT	Area	% Area	Height
13		6.077	7019	0.16	510
14		6.507	25634	0.58	2759
15		6.892	16742	0.38	1376
16		7.102	50292	1.13	5139
17		7.200	13593	0.31	3856
18	25ODESRIF	7.550	1775477	40.06	271755
19	RIF	8.618	2727	0.06	484
20		8.817	27170	0.61	4092
21		9.145	3360	0.08	548
22		9.958	478350	10.79	27857
23		10.622	337370	7.61	50775
24	NEO	10.934	27860	0.63	3130

SAMPLE INFORMATION

Sample Name:	RIF Inc Basil_MeOH BMR16 20min 1	Acquired By:	Saneesh
Sample Type:	Unknown	Date Acquired:	2017/02/07 07:35:50 PM
Vial:	17	Acq. Method Set:	HDI_RIF_25ODESRIF_NEO
Injection #:	1	Date Processed:	2017/02/08 06:52:59 AM
Injection Volume:	15.00 ul	Processing Method:	HDI_RIF_ W2996 254.0nm-1.2
Run Time:	11.5 Minutes	Channel Name:	W2996 254.0nm-1.2
Sample Set Name:	RIF_Basil MeOH AssaySam 7_1_17	Proc. Chnl. Descr.:	W2996 PDA 254.0 nm at 1.2

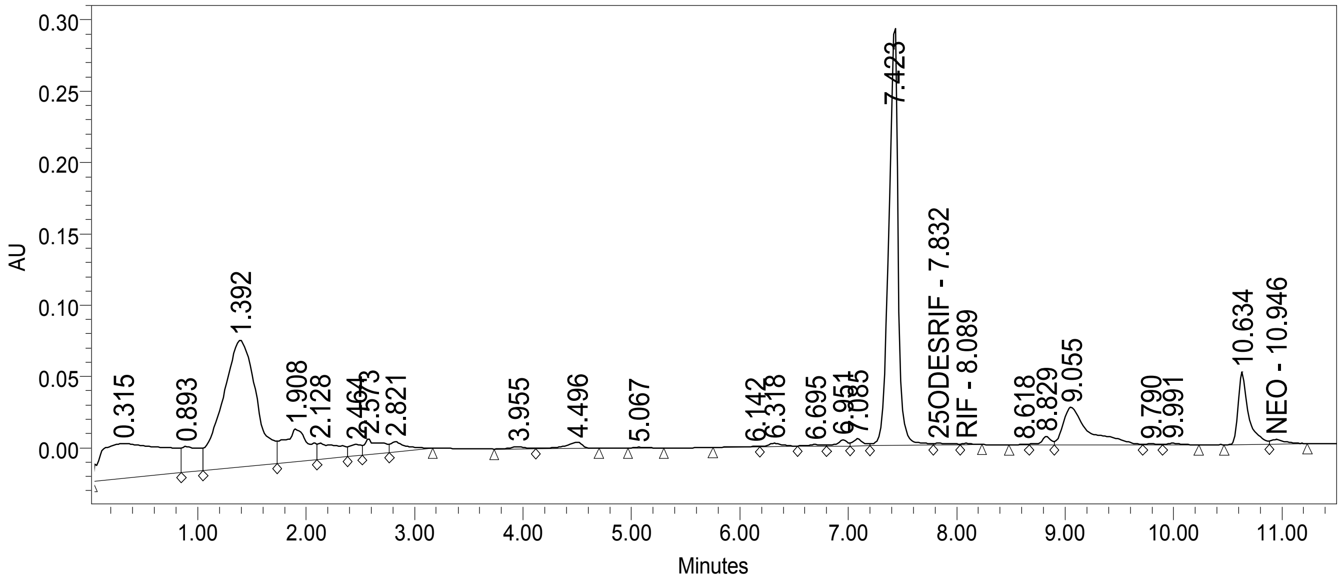

	Peak Name	RT	Area	% Area	Height
1		0.315	997642	14.97	24294
2		0.893	200204	3.00	17817
3		1.392	1941043	29.12	88873
4		1.908	355591	5.33	22945
5		2.128	151050	2.27	10984
6		2.464	58598	0.88	7788
7		2.573	113811	1.71	10549
8		2.821	71360	1.07	7046
9		3.955	16050	0.24	1598
10		4.496	49180	0.74	4380
11		5.067	5807	0.09	619
12		6.142	7230	0.11	426

	Peak Name	RT	Area	% Area	Height
13		6.318	25444	0.38	2468
14		6.695	14854	0.22	1323
15		6.951	28917	0.43	4414
16		7.085	31635	0.47	5070
17		7.423	1723367	25.86	291357
18	25ODESRIF	7.832	12522	0.19	1169
19	RIF	8.089	4999	0.07	886
20		8.618	4436	0.07	765
21		8.829	38529	0.58	6116
22		9.055	431936	6.48	26411
23		9.790	6042	0.09	745
24		9.991	9295	0.14	1112
25		10.634	337525	5.06	50237
26	NEO	10.946	28415	0.43	3165

SAMPLE INFORMATION

Sample Name:	RIF Inc Basil_MeOH BMR17 20min 2	Acquired By:	Saneesh
Sample Type:	Unknown	Date Acquired:	2017/02/07 07:50:36 PM
Vial:	18	Acq. Method Set:	HDI_RIF_25ODESRIF_NEO
Injection #:	1	Date Processed:	2017/02/08 06:52:59 AM
Injection Volume:	15.00 ul	Processing Method:	HDI_RIF_ W2996 254.0nm-1.2
Run Time:	11.5 Minutes	Channel Name:	W2996 254.0nm-1.2
Sample Set Name:	RIF_Basil MeOH AssaySam 7_1_17	Proc. Chnl. Descr.:	W2996 PDA 254.0 nm at 1.2

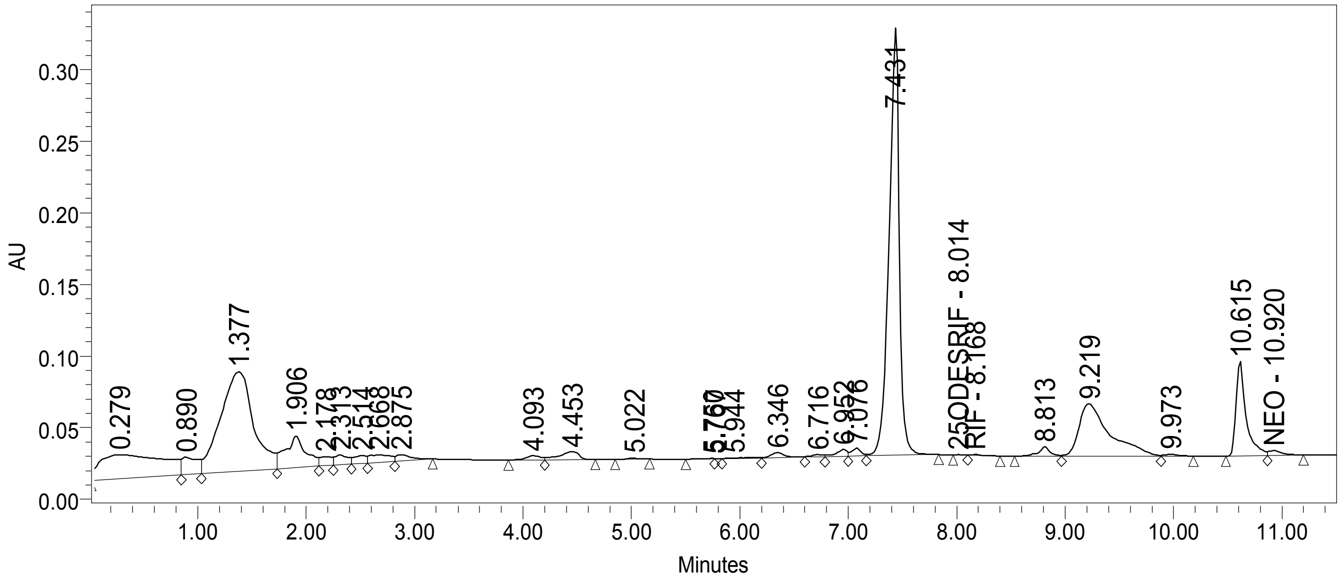

	Peak Name	RT	Area	% Area	Height
1		0.279	662242	10.67	16665
2		0.890	115851	1.87	11889
3		1.377	1464097	23.60	69078
4		1.906	288016	4.64	22166
5		2.178	47546	0.77	6214
6		2.313	56840	0.92	6913
7		2.514	44802	0.72	5428
8		2.668	68150	1.10	5269
9		2.875	40605	0.65	4181
10		4.093	28347	0.46	2988
11		4.453	70710	1.14	5489
12		5.022	6103	0.10	766

Reported by User: Saneesh (Saneesh) Project Name: HDI_Stellenbosch_2016

	Peak Name	RT	Area	% Area	Height
13		5.750	2548	0.04	297
14		5.767	410	0.01	172
15		5.944	4954	0.08	363
16		6.346	29414	0.47	3524
17		6.716	10807	0.17	1532
18		6.952	32879	0.53	4747
19		7.076	33589	0.54	5539
20		7.431	1946217	31.37	297911
21	25ODESRIF	8.014	1651	0.03	351
22	RIF	8.168	4834	0.08	733
23		8.813	52374	0.84	6605
24		9.219	722905	11.65	36605
25		9.973	11650	0.19	1468
26		10.615	427509	6.89	65120
27	NEO	10.920	29000	0.47	3299

SAMPLE INFORMATION

Sample Name:	RIF Inc Basil_MeOH BMR18 20min 3	Acquired By:	Saneesh
Sample Type:	Unknown	Date Acquired:	2017/02/07 08:05:20 PM
Vial:	19	Acq. Method Set:	HDI_RIF_25ODESRIF_NEO
Injection #:	1	Date Processed:	2017/02/08 06:52:59 AM
Injection Volume:	15.00 ul	Processing Method:	HDI_RIF_ W2996 254.0nm-1.2
Run Time:	11.5 Minutes	Channel Name:	W2996 254.0nm-1.2
Sample Set Name:	RIF_Basil MeOH AssaySam 7_1_17	Proc. Chnl. Descr.:	W2996 PDA 254.0 nm at 1.2

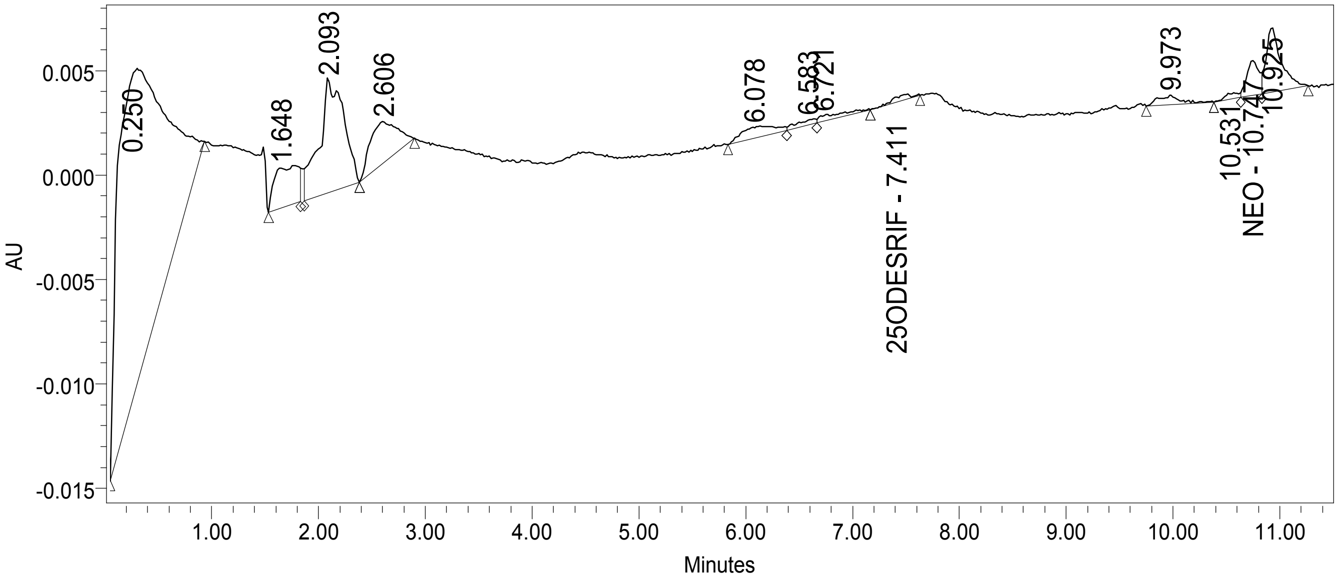

	Peak Name	RT	Area	% Area	Height
1		0.250	465811	67.15	15530
2		1.648	29616	4.27	1953
3		2.093	87072	12.55	5472
4		2.606	35796	5.16	1994
5		6.078	11378	1.64	504
6		6.583	3519	0.51	250
7		6.721	5069	0.73	290
8	25ODESRIF	7.411	3446	0.50	253
9	RIF	8.100			
10		9.973	6522	0.94	426
11		10.531	2399	0.35	268
12	NEO	10.747	13156	1.90	1666

Reported by User: Saneesh (Saneesh) Project Name: HDI_Stellenbosch_2016

	Peak Name	RT	Area	% Area	Height
13		10.925	29943	4.32	3051

Reported by User: Saneesh (Saneesh) Project Name: HDI_Stellenbosch_2016

SAMPLE INFORMATION

Sample Name:	RIF Inc Basil_MeOH BMR19 30min 1	Acquired By:	Saneesh
Sample Type:	Unknown	Date Acquired:	2017/02/07 08:20:13 PM
Vial:	20	Acq. Method Set:	HDI_RIF_25ODESRIF_NEO
Injection #:	1	Date Processed:	2017/02/08 06:52:59 AM
Injection Volume:	15.00 ul	Processing Method:	HDI_RIF_ W2996 254.0nm-1.2
Run Time:	11.5 Minutes	Channel Name:	W2996 254.0nm-1.2
Sample Set Name:	RIF_Basil MeOH AssaySam 7_1_17	Proc. Chnl. Descr.:	W2996 PDA 254.0 nm at 1.2

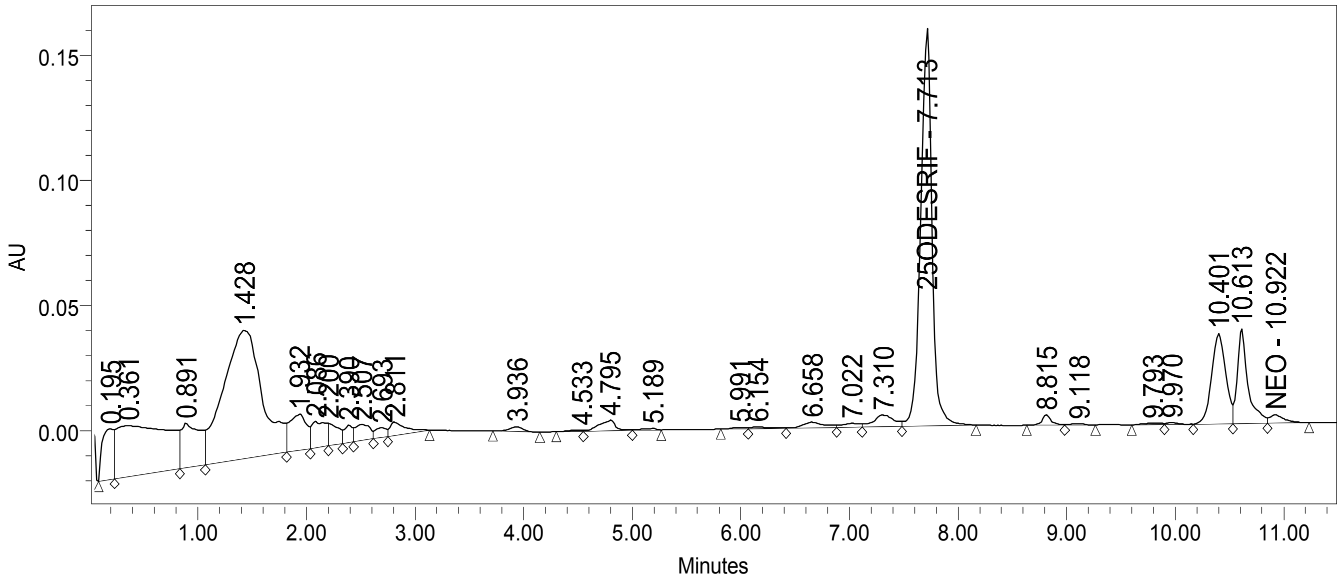

	Peak Name	RT	Area	% Area	Height
1		0.195	151737	3.16	20136
2		0.361	653945	13.61	20407
3		0.891	214850	4.47	17648
4		1.428	1282868	26.70	51273
5		1.932	158768	3.31	14438
6		2.086	95366	1.99	10407
7		2.200	57147	1.19	8885
8		2.390	36521	0.76	6833
9		2.507	60498	1.26	6474
10		2.693	28506	0.59	3920
11		2.811	53059	1.10	5496
12		3.936	17006	0.35	1891

	Peak Name	RT	Area	% Area	Height
13		4.533	4500	0.09	504
14		4.795	45141	0.94	4258
15		5.189	6700	0.14	672
16		5.991	4815	0.10	504
17		6.154	9429	0.20	679
18		6.658	30156	0.63	2247
19		7.022	16750	0.35	1429
20		7.310	60903	1.27	4691
21	25ODESRIF	7.713	1158213	24.11	159337
22	RIF	8.100			
23		8.815	23679	0.49	4141
24		9.118	4638	0.10	479
25		9.793	7528	0.16	624
26		9.970	5101	0.11	734
27		10.401	324839	6.76	35984
28		10.613	259551	5.40	37617
29	NEO	10.922	31656	0.66	3419

SAMPLE INFORMATION

Sample Name:	RIF Inc Basil_MeOH BMR20 30min 2	Acquired By:	Saneesh
Sample Type:	Unknown	Date Acquired:	2017/02/07 08:34:58 PM
Vial:	21	Acq. Method Set:	HDI_RIF_25ODESRIF_NEO
Injection #:	1	Date Processed:	2017/02/08 06:52:59 AM
Injection Volume:	15.00 ul	Processing Method:	HDI_RIF_ W2996 254.0nm-1.2
Run Time:	11.5 Minutes	Channel Name:	W2996 254.0nm-1.2
Sample Set Name:	RIF_Basil MeOH AssaySam 7_1_17	Proc. Chnl. Descr.:	W2996 PDA 254.0 nm at 1.2

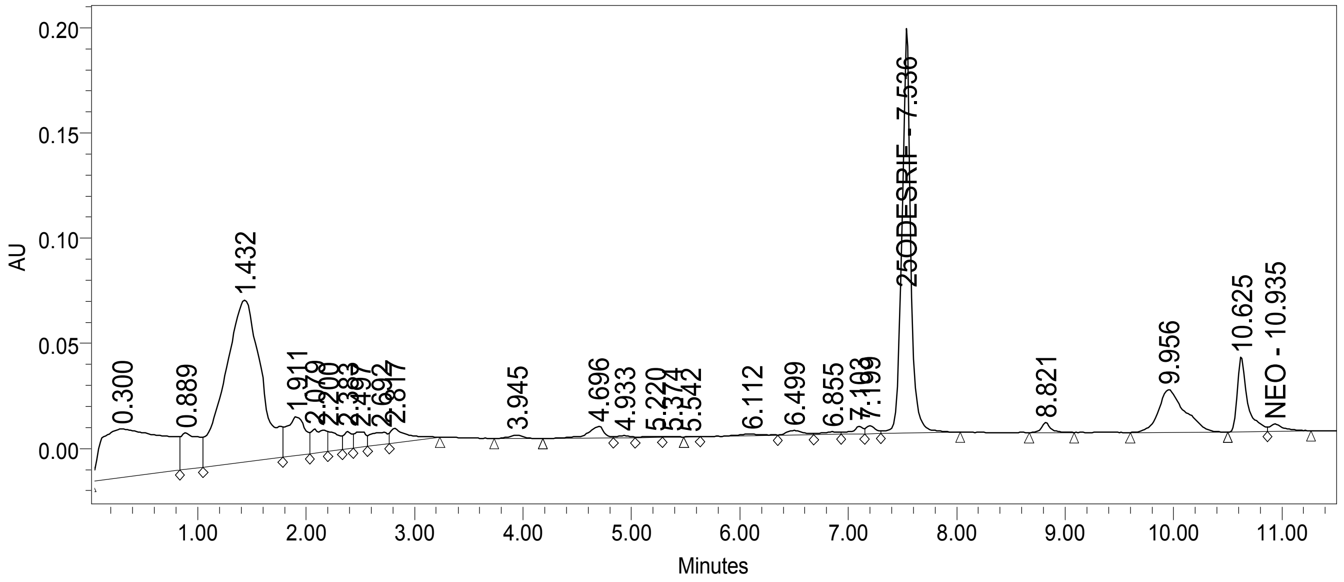

	Peak Name	RT	Area	% Area	Height
1		0.300	916266	16.71	22930
2		0.889	203074	3.70	17349
3		1.432	1789646	32.65	76841
4		1.911	223670	4.08	18316
5		2.079	103644	1.89	11140
6		2.200	63837	1.16	9483
7		2.383	44346	0.81	8287
8		2.497	54744	1.00	7453
9		2.692	66885	1.22	5796
10		2.817	77674	1.42	6954
11		3.945	15781	0.29	1682
12		4.696	59323	1.08	5634

	Peak Name	RT	Area	% Area	Height
13		4.933	8979	0.16	1042
14		5.220	5250	0.10	512
15		5.374	2985	0.05	391
16		5.542	688	0.01	137
17		6.112	15975	0.29	874
18		6.499	24073	0.44	2228
19		6.855	14539	0.27	1299
20		7.103	25725	0.47	3659
21		7.199	24432	0.45	3895
22	25ODESRIF	7.536	1095428	19.98	189914
23	RIF	8.100			
24		8.821	30164	0.55	4759
25		9.956	347435	6.34	20445
26		10.625	236477	4.31	35668
27	NEO	10.935	30881	0.56	3384

SAMPLE INFORMATION

Sample Name:	RIF Inc Basil_MeOH BMR21 30min 3	Acquired By:	Saneesh
Sample Type:	Unknown	Date Acquired:	2017/02/07 08:49:41 PM
Vial:	22	Acq. Method Set:	HDI_RIF_25ODESRIF_NEO
Injection #:	1	Date Processed:	2017/02/08 06:52:59 AM
Injection Volume:	15.00 ul	Processing Method:	HDI_RIF_ W2996 254.0nm-1.2
Run Time:	11.5 Minutes	Channel Name:	W2996 254.0nm-1.2
Sample Set Name:	RIF_Basil MeOH AssaySam 7_1_17	Proc. Chnl. Descr.:	W2996 PDA 254.0 nm at 1.2

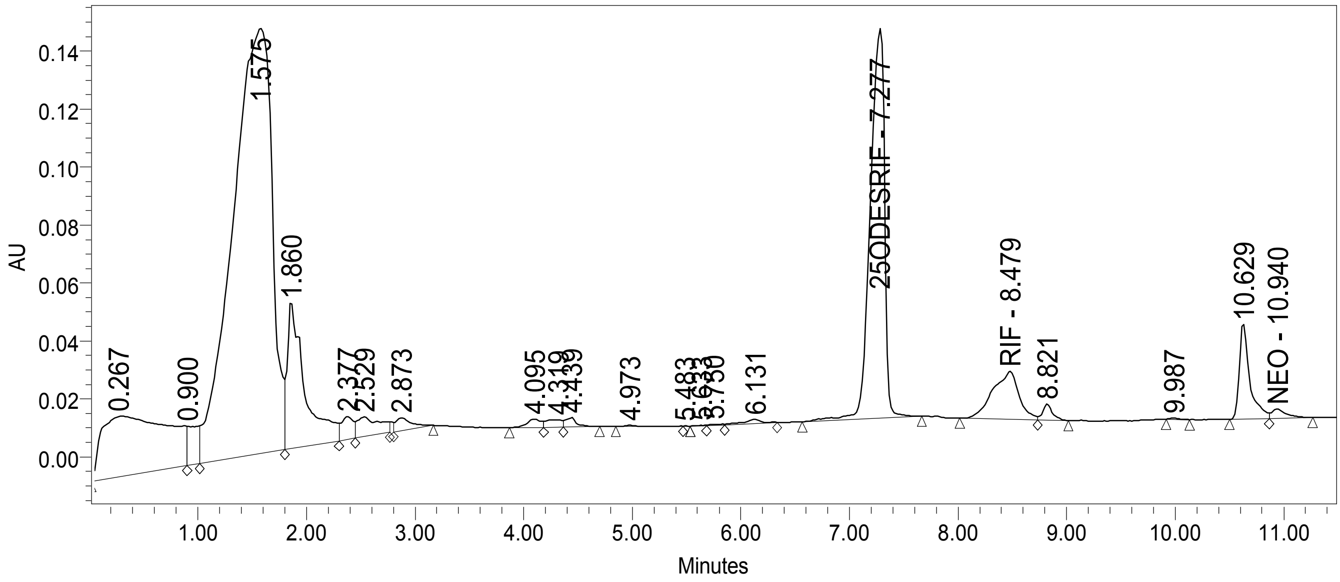

	Peak Name	RT	Area	% Area	Height
1		0.267	870962	11.85	20757
2		0.900	91925	1.25	13570
3		1.575	3712960	50.52	146831
4		1.860	572797	7.79	50093
5		2.377	60510	0.82	7860
6		2.529	95971	1.31	6866
7		2.873	45013	0.61	4374
8		4.095	25955	0.35	2897
9		4.319	25811	0.35	2485
10		4.439	21624	0.29	3208
11		4.973	3094	0.04	381
12		5.483	124	0.00	55

	Peak Name	RT	Area	% Area	Height
13		5.633	760	0.01	127
14		5.750	2299	0.03	301
15		6.131	17405	0.24	1443
16	25ODESRIF	7.277	1234702	16.80	135594
17	RIF	8.479	278425	3.79	16565
18		8.821	34791	0.47	5449
19		9.987	2765	0.04	464
20		10.629	222086	3.02	32494
21	NEO	10.940	29571	0.40	3178

Reported by User: Saneesh (Saneesh)   Project Name: HDI_Stellenbosch_2016

SAMPLE INFORMATION

Sample Name:	RIF Inc Basil_MeOH BMR22 45min 1	Acquired By:	Saneesh
Sample Type:	Unknown	Date Acquired:	2017/02/07 09:04:17 PM
Vial:	23	Acq. Method Set:	HDI_RIF_25ODESRIF_NEO
Injection #:	1	Date Processed:	2017/02/08 06:52:59 AM
Injection Volume:	15.00 ul	Processing Method:	HDI_RIF_ W2996 254.0nm-1.2
Run Time:	11.5 Minutes	Channel Name:	W2996 254.0nm-1.2
Sample Set Name:	RIF_Basil MeOH AssaySam 7_1_17	Proc. Chnl. Descr.:	W2996 PDA 254.0 nm at 1.2

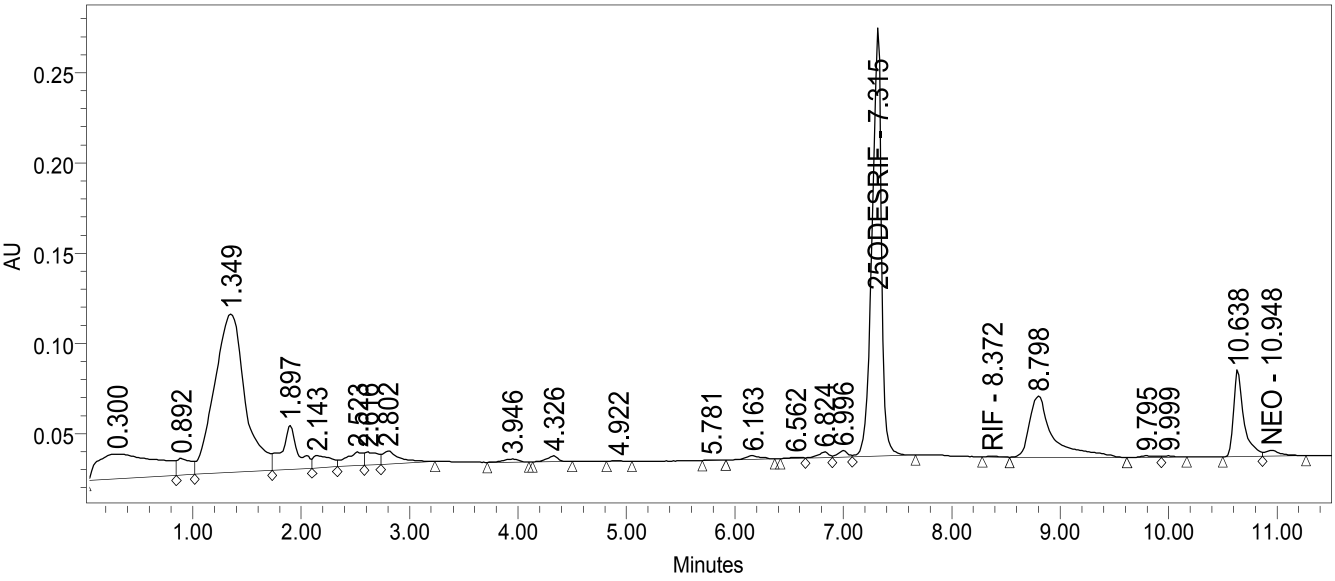

	Peak Name	RT	Area	% Area	Height
1		0.300	510298	9.67	13692
2		0.892	81578	1.55	9265
3		1.349	1721170	32.61	87916
4		1.897	250116	4.74	24417
5		2.143	75898	1.44	6855
6		2.523	83100	1.57	7439
7		2.616	58266	1.10	7190
8		2.802	78233	1.48	7103
9		3.946	16225	0.31	1766
10		4.326	23039	0.44	3198
11		4.922	2604	0.05	453
12		5.781	1357	0.03	207

	Peak Name	RT	Area	% Area	Height
13		6.163	20117	0.38	2057
14		6.562	4174	0.08	570
15		6.824	20150	0.38	3028
16		6.996	21648	0.41	3549
17	25ODESRIF	7.315	1432722	27.15	235848
18	RIF	8.372	4215	0.08	632
19		8.798	510926	9.68	33944
20		9.795	8248	0.16	918
21		9.999	5206	0.10	692
22		10.638	315964	5.99	47921
23	NEO	10.948	32058	0.61	3263

Reported by User: Saneesh (Saneesh)   Project Name: HDI_Stellenbosch_2016

SAMPLE INFORMATION

Sample Name:	RIF Inc Basil_MeOH BMR23 45min 2	Acquired By:	Saneesh
Sample Type:	Unknown	Date Acquired:	2017/02/07 09:18:52 PM
Vial:	24	Acq. Method Set:	HDI_RIF_25ODESRIF_NEO
Injection #:	1	Date Processed:	2017/02/08 06:52:59 AM
Injection Volume:	15.00 ul	Processing Method:	HDI_RIF_ W2996 254.0nm-1.2
Run Time:	11.5 Minutes	Channel Name:	W2996 254.0nm-1.2
Sample Set Name:	RIF_Basil MeOH AssaySam 7_1_17	Proc. Chnl. Descr.:	W2996 PDA 254.0 nm at 1.2

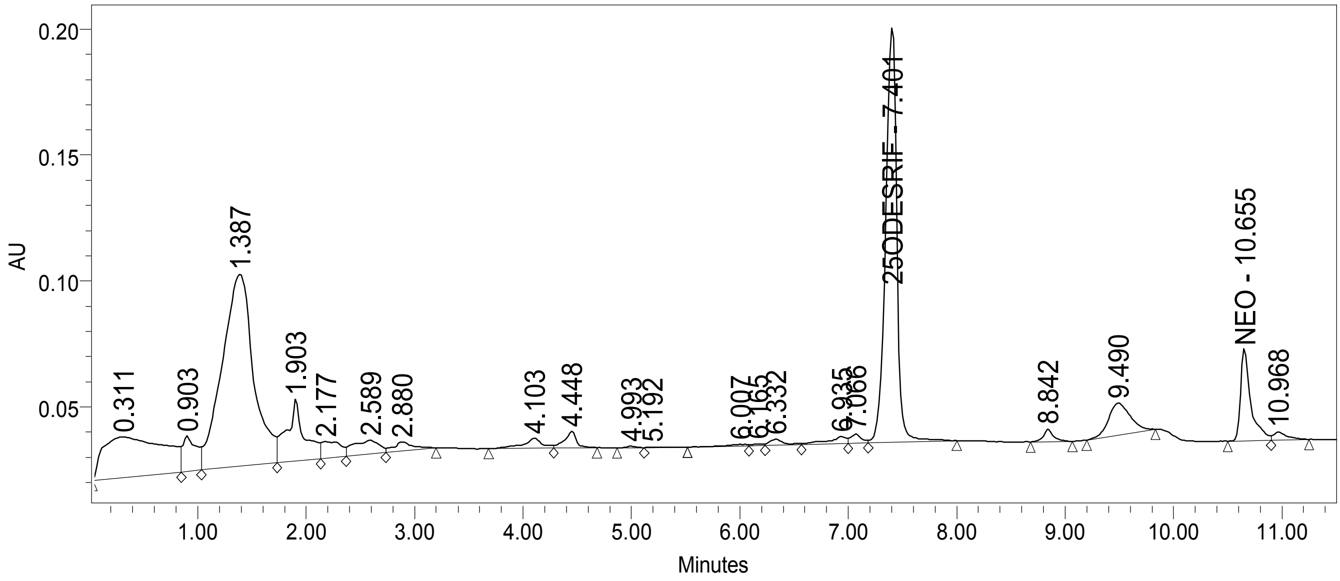

	Peak Name	RT	Area	% Area	Height
1		0.311	615569	13.36	16026
2		0.903	119978	2.60	13812
3		1.387	1543042	33.48	76243
4		1.903	275823	5.98	24016
5		2.177	78317	1.70	6536
6		2.589	93427	2.03	5438
7		2.880	45003	0.98	3656
8		4.103	49915	1.08	3933
9		4.448	50030	1.09	6616
10		4.993	4678	0.10	768
11		5.192	2506	0.05	237
12		6.007	8513	0.18	669

	Peak Name	RT	Area	% Area	Height
13		6.165	5008	0.11	620
14		6.332	21085	0.46	2422
15		6.935	32934	0.71	2740
16		7.066	24553	0.53	3579
17	25ODESRIF	7.401	1162388	25.22	165554
18	RIF	8.100			
19		8.842	32431	0.70	4999
20		9.490	176127	3.82	12674
21	NEO	10.655	239259	5.19	36249
22		10.968	28005	0.61	3151

SAMPLE INFORMATION

Sample Name:	RIF Inc Basil_MeOH BMR24 45min 3	Acquired By:	Saneesh
Sample Type:	Unknown	Date Acquired:	2017/02/07 09:33:28 PM
Vial:	2	Acq. Method Set:	HDI_RIF_25ODESRIF_NEO
Injection #:	1	Date Processed:	2017/02/08 06:53:01 AM
Injection Volume:	15.00 ul	Processing Method:	HDI_RIF_ W2996 254.0nm-1.2
Run Time:	11.5 Minutes	Channel Name:	W2996 254.0nm-1.2
Sample Set Name:	RIF_Basil MeOH AssaySam 7_1_17	Proc. Chnl. Descr.:	W2996 PDA 254.0 nm at 1.2

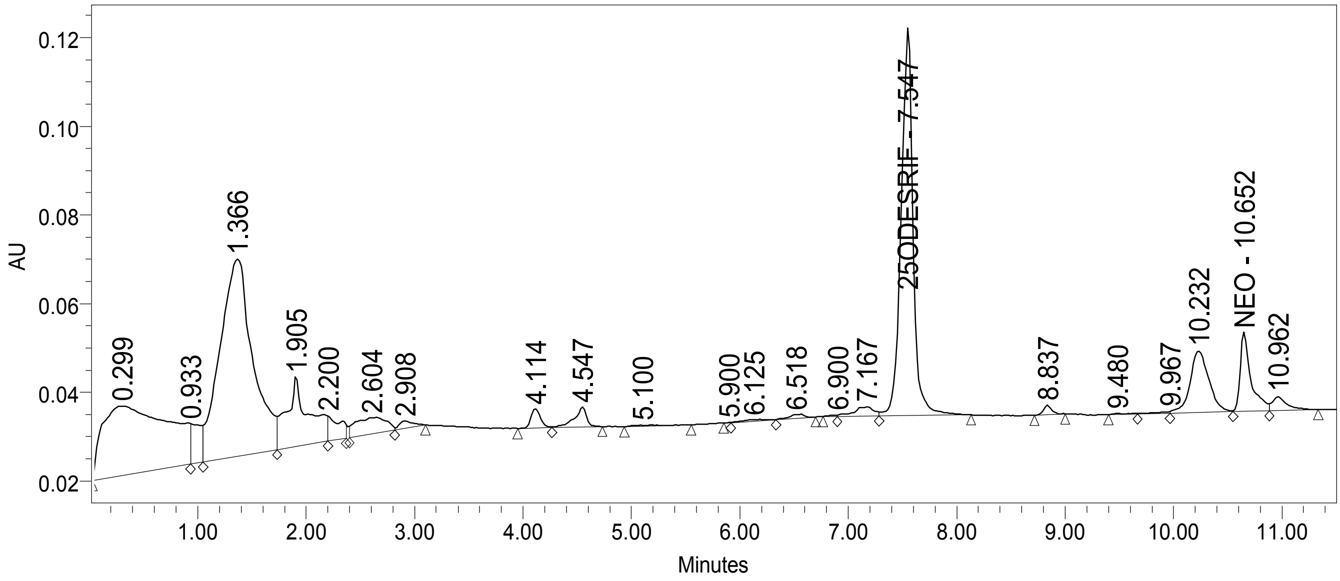

	Peak Name	RT	Area	% Area	Height
1		0.299	646045	20.76	15646
2		0.933	60143	1.93	9062
3		1.366	943037	30.30	44429
4		1.905	222713	7.16	15214
5		2.200	40700	1.31	5681
6		2.604	72542	2.33	3581
7		2.908	14055	0.45	1657
8		4.114	27066	0.87	4362
9		4.547	35915	1.15	4514
10		5.100	4176	0.13	233
11		5.900	170	0.01	60
12		6.125	4974	0.16	351

	Peak Name	RT	Area	% Area	Height
13		6.518	7980	0.26	866
14		6.900	1825	0.06	341
15		7.167	27149	0.87	2085
16	25ODESRIF	7.547	668917	21.49	87629
17	RIF	8.100			
18		8.837	11517	0.37	2139
19		9.480	1494	0.05	199
20		9.967	3219	0.10	442
21		10.232	165822	5.33	13850
22	NEO	10.652	122924	3.95	17652
23		10.962	30078	0.97	3172

SAMPLE INFORMATION

Sample Name:	RIF Inc Basil_MeOH BMR6 2min 3 R	Acquired By:	Saneesh
Sample Type:	Unknown	Date Acquired:	2017/02/07 09:54:24 PM
Vial:	7	Acq. Method Set:	HDI_RIF_25ODESRIF_NEO
Injection #:	1	Date Processed:	2017/02/08 06:53:01 AM
Injection Volume:	15.00 ul	Processing Method:	HDI_RIF_ W2996 254.0nm-1.2
Run Time:	11.5 Minutes	Channel Name:	W2996 254.0nm-1.2
Sample Set Name:	RIF_Basil MeOH AssaySam 7_1_17	Proc. Chnl. Descr.:	W2996 PDA 254.0 nm at 1.2

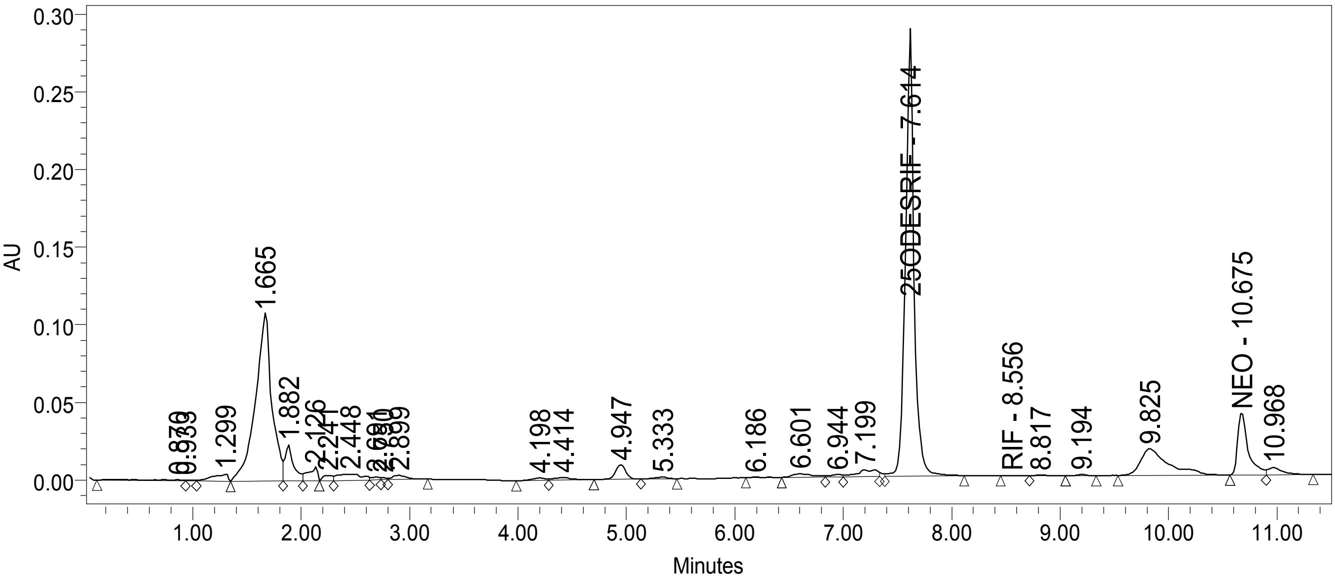

	Peak Name	RT	Area	% Area	Height
1		0.870	21583	0.55	613
2		0.933	2844	0.07	501
3		1.299	45039	1.15	4440
4		1.665	1120426	28.64	107508
5		1.882	129734	3.32	22318
6		2.126	51147	1.31	8419
7		2.241	20576	0.53	3405
8		2.448	62961	1.61	3850
9		2.691	9918	0.25	1805
10		2.750	5391	0.14	1553
11		2.899	25944	0.66	2737
12		4.198	14811	0.38	1700

	Peak Name	RT	Area	% Area	Height
13		4.414	21194	0.54	1699
14		4.947	64715	1.65	9460
15		5.333	11314	0.29	1308
16		6.186	3225	0.08	391
17		6.601	28809	0.74	2406
18		6.944	11076	0.28	1447
19		7.199	54556	1.39	4450
20	25ODESRIF	7.614	1570116	40.14	276230
21	RIF	8.556	2570	0.07	306
22		8.817	5033	0.13	550
23		9.194	4644	0.12	831
24		9.825	310951	7.95	17266
25	NEO	10.675	268745	6.87	40119
26		10.968	44451	1.14	4655

Reported by User: Saneesh (Saneesh) Project Name: HDI_Stellenbosch_2016

SAMPLE INFORMATION

Sample Name:	RIF Inc Basil_MeOH BMR18 20min3R	Acquired By:	Saneesh
Sample Type:	Unknown	Date Acquired:	2017/02/07 10:09:08 PM
Vial:	19	Acq. Method Set:	HDI_RIF_25ODESRIF_NEO
Injection #:	1	Date Processed:	2017/02/08 06:53:01 AM
Injection Volume:	15.00 ul	Processing Method:	HDI_RIF_ W2996 254.0nm-1.2
Run Time:	11.5 Minutes	Channel Name:	W2996 254.0nm-1.2
Sample Set Name:	RIF_Basil MeOH AssaySam 7_1_17	Proc. Chnl. Descr.:	W2996 PDA 254.0 nm at 1.2

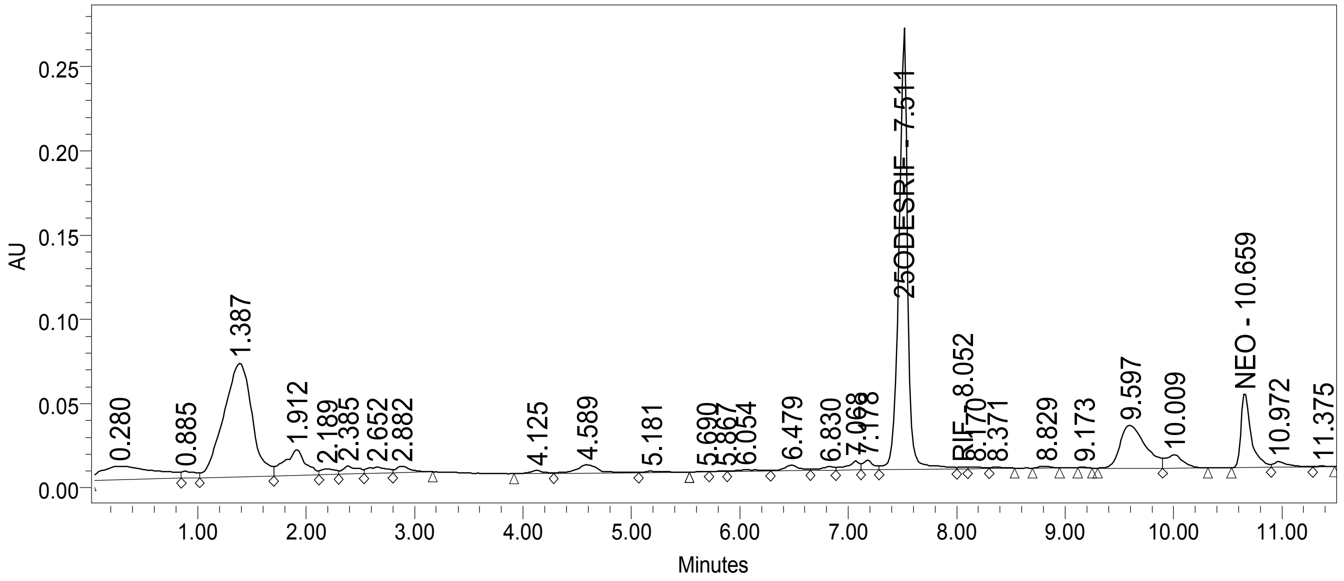

	Peak Name	RT	Area	% Area	Height
1		0.280	273732	6.13	7951
2		0.885	34227	0.77	4104
3		1.387	1228499	27.53	67379
4		1.912	203325	4.56	15021
5		2.189	29821	0.67	3272
6		2.385	45505	1.02	4632
7		2.652	45992	1.03	3642
8		2.882	34200	0.77	3684
9		4.125	16369	0.37	1812
10		4.589	69814	1.56	4811
11		5.181	8820	0.20	764
12		5.690	998	0.02	141

	Peak Name	RT	Area	% Area	Height
13		5.867	2552	0.06	337
14		6.054	14591	0.33	936
15		6.479	34861	0.78	3435
16		6.830	21438	0.48	2208
17		7.068	43747	0.98	5421
18		7.178	38885	0.87	5686
19	25ODESRIF	7.511	1464746	32.83	254836
20	RIF	8.052	6028	0.14	1053
21		8.170	8992	0.20	1020
22		8.371	6543	0.15	941
23		8.829	8321	0.19	1046
24		9.173	2057	0.05	491
25		9.597	409990	9.19	25423
26		10.009	86289	1.93	7786
27	NEO	10.659	287039	6.43	44104
28		10.972	31881	0.71	3325
29		11.375	2563	0.06	319

Multi Sample Summary

Reported by User: Saneesh (Saneesh) Project Name: HDI_Stellenbosch_2016

SAMPLE INFORMATION

Sample Name:	RIF Inc Basil_MeOH BMRc1 0min 1	Acquired By:	Saneesh
Sample Type:	Unknown	Date Acquired:	2017/02/07 10:38:08 PM
Vial:	2	Acq. Method Set:	HDI_RIF_25ODESRIF_NEO
Injection #:	1	Date Processed:	2017/02/08 06:54:17 AM
Injection Volume:	15.00 ul	Processing Method:	HDI_RIF_ W2996 254.0nm-1.2
Run Time:	11.5 Minutes	Channel Name:	W2996 254.0nm-1.2
Sample Set Name:	RIF_Basil MeOH Controls 7_1_17	Proc. Chnl. Descr.:	W2996 PDA 254.0 nm at 1.2

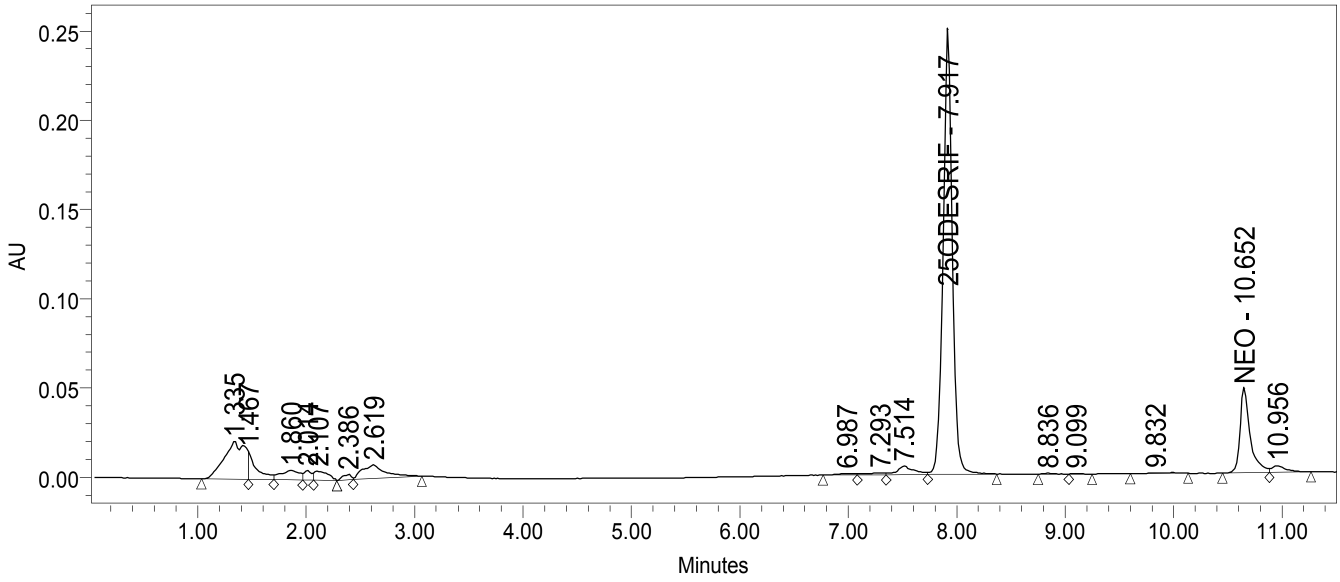

	Peak Name	RT	Area	% Area	Height
1		1.335	285368	11.25	20981
2		1.467	78892	3.11	15616
3		1.860	64771	2.55	5266
4		2.014	26442	1.04	5245
5		2.107	44490	1.75	5043
6		2.386	16270	0.64	2907
7		2.619	113933	4.49	7630
8		6.987	8040	0.32	765
9		7.293	13244	0.52	1037
10		7.514	52229	2.06	4796
11	25ODESRIF	7.917	1476867	58.22	246815
12	RIF	8.100			
13		8.836	3763	0.15	644
14		9.099	2117	0.08	358

	Peak Name	RT	Area	% Area	Height
15		9.832	9123	0.36	538
16	NEO	10.652	307606	12.13	47262
17		10.956	33375	1.32	3682

SAMPLE INFORMATION

Sample Name:	RIF Inc Basil_MeOH BMRc2 0min 2	Acquired By:	Saneesh
Sample Type:	Unknown	Date Acquired:	2017/02/07 10:53:02 PM
Vial:	3	Acq. Method Set:	HDI_RIF_25ODESRIF_NEO
Injection #:	1	Date Processed:	2017/02/08 06:54:17 AM
Injection Volume:	15.00 ul	Processing Method:	HDI_RIF_ W2996 254.0nm-1.2
Run Time:	11.5 Minutes	Channel Name:	W2996 254.0nm-1.2
Sample Set Name:	RIF_Basil MeOH Controls 7_1_17	Proc. Chnl. Descr.:	W2996 PDA 254.0 nm at 1.2

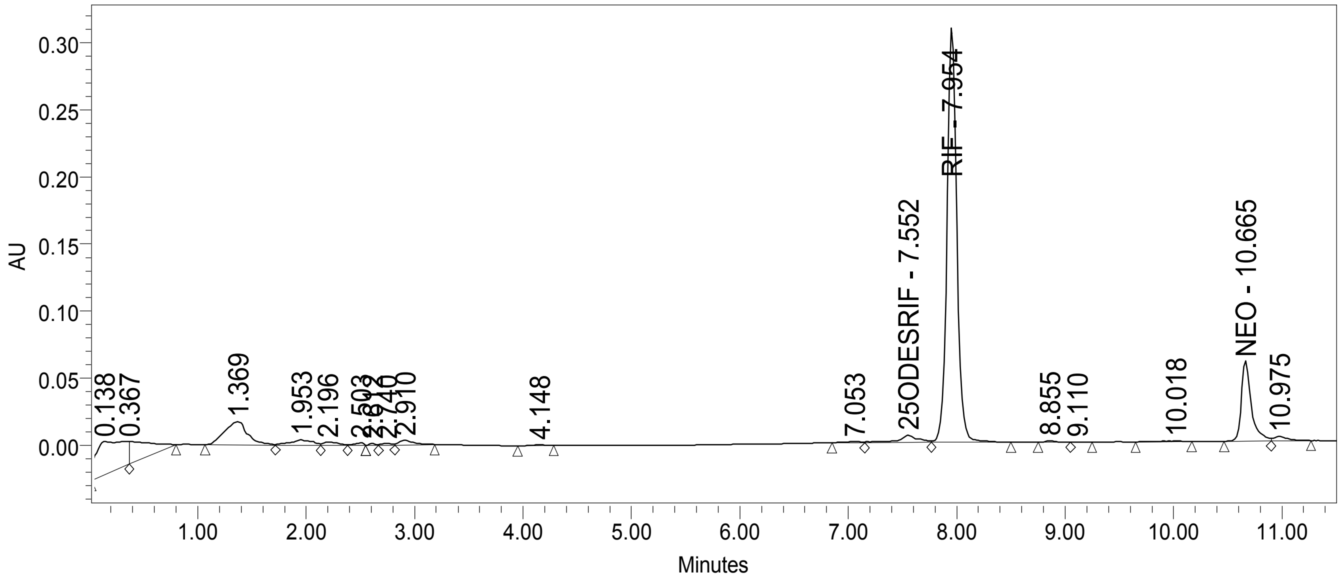

	Peak Name	RT	Area	% Area	Height
1		0.138	419350	12.09	24775
2		0.367	214506	6.19	16944
3		1.369	257754	7.43	17036
4		1.953	56473	1.63	3960
5		2.196	23913	0.69	2413
6		2.503	12017	0.35	2062
7		2.612	6561	0.19	1514
8		2.740	10536	0.30	1422
9		2.910	32653	0.94	3456
10		4.148	6973	0.20	865
11		7.053	7321	0.21	683
12	25ODESRIF	7.552	67512	1.95	5579

	Peak Name	RT	Area	% Area	Height
13	RIF	7.954	1926930	55.57	307161
14		8.855	6684	0.19	1157
15		9.110	1669	0.05	286
16		10.018	8190	0.24	447
17	NEO	10.665	378867	10.93	58955
18		10.975	29518	0.85	3258

SAMPLE INFORMATION

Sample Name:	RIF Inc Basil_MeOH BMRc3 0min3	Acquired By:	Saneesh
Sample Type:	Unknown	Date Acquired:	2017/02/07 11:07:47 PM
Vial:	4	Acq. Method Set:	HDI_RIF_25ODESRIF_NEO
Injection #:	1	Date Processed:	2017/02/08 06:54:17 AM
Injection Volume:	15.00 ul	Processing Method:	HDI_RIF_ W2996 254.0nm-1.2
Run Time:	11.5 Minutes	Channel Name:	W2996 254.0nm-1.2
Sample Set Name:	RIF_Basil MeOH Controls 7_1_17	Proc. Chnl. Descr.:	W2996 PDA 254.0 nm at 1.2

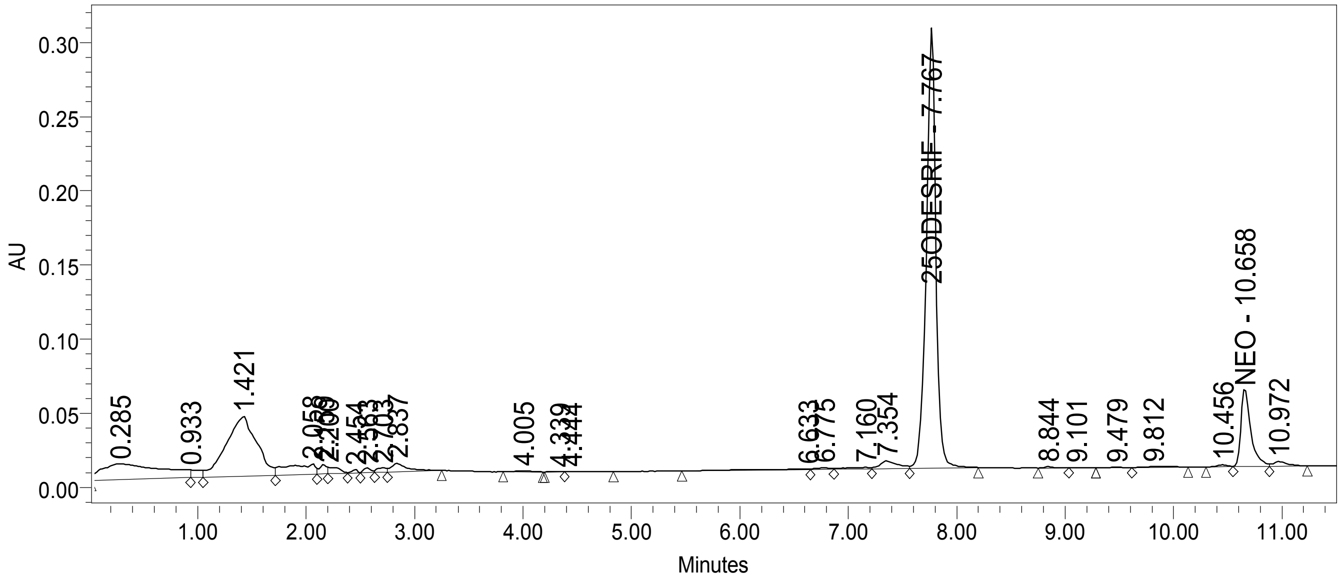

	Peak Name	RT	Area	% Area	Height
1		0.285	403328	10.54	10423
2		0.933	33532	0.88	5055
3		1.421	783810	20.48	40513
4		2.058	129093	3.37	6528
5		2.159	28235	0.74	5903
6		2.200	31258	0.82	4297
7		2.454	8816	0.23	2065
8		2.563	15371	0.40	3068
9		2.703	17979	0.47	2925
10		2.837	59309	1.55	5824
11		4.005	7896	0.21	789
12		4.339	1142	0.03	139

	Peak Name	RT	Area	% Area	Height
13		4.444	3295	0.09	190
14		6.633	14318	0.37	479
15		6.775	10400	0.27	1057
16		7.160	17915	0.47	1004
17		7.354	57931	1.51	5141
18	25ODESRIF	7.767	1816008	47.45	293048
19	RIF	8.100			
20		8.844	5597	0.15	903
21		9.101	2042	0.05	294
22		9.479	2882	0.08	281
23		9.812	9818	0.26	582
24		10.456	9015	0.24	1259
25	NEO	10.658	330065	8.62	52135
26		10.972	28259	0.74	3096

Reported by User: Saneesh (Saneesh)   Project Name: HDI_Stellenbosch_2016

SAMPLE INFORMATION

Sample Name:	RIF Inc Basil_MeOH BMRc4 2min 1	Acquired By:	Saneesh
Sample Type:	Unknown	Date Acquired:	2017/02/07 11:22:32 PM
Vial:	5	Acq. Method Set:	HDI_RIF_25ODESRIF_NEO
Injection #:	1	Date Processed:	2017/02/08 06:54:17 AM
Injection Volume:	15.00 ul	Processing Method:	HDI_RIF_ W2996 254.0nm-1.2
Run Time:	11.5 Minutes	Channel Name:	W2996 254.0nm-1.2
Sample Set Name:	RIF_Basil MeOH Controls 7_1_17	Proc. Chnl. Descr.:	W2996 PDA 254.0 nm at 1.2

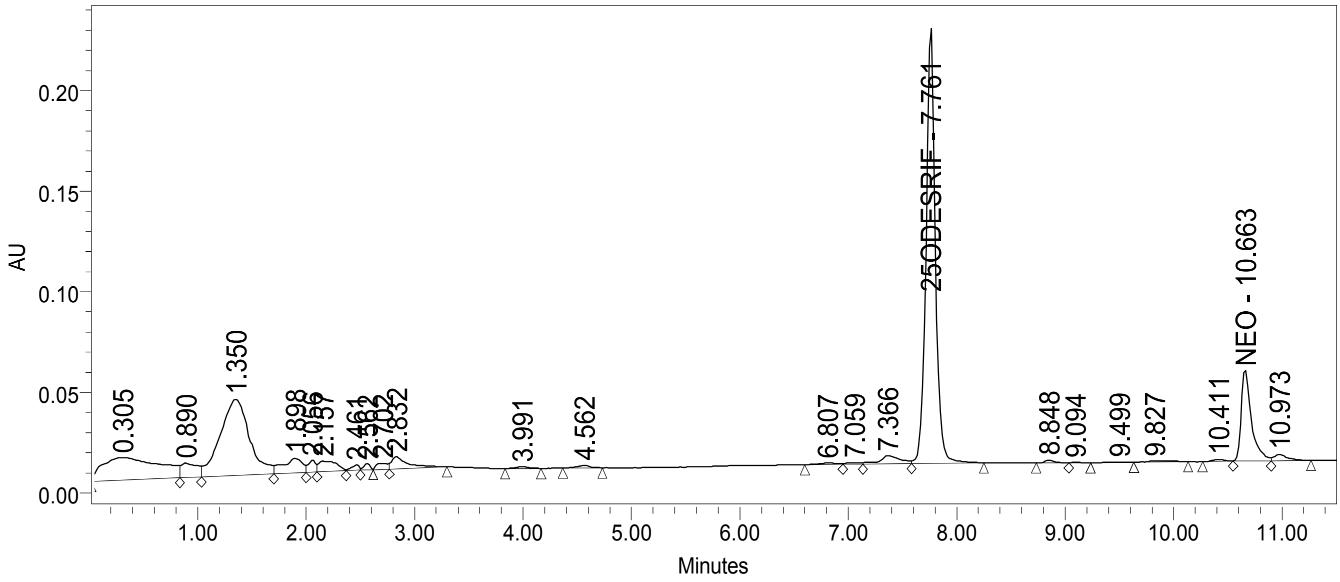

	Peak Name	RT	Area	% Area	Height
1		0.305	399677	12.51	11042
2		0.890	73286	2.29	7128
3		1.350	707093	22.14	37809
4		1.898	91608	2.87	7345
5		2.056	28108	0.88	5776
6		2.157	60738	1.90	5386
7		2.461	13362	0.42	2767
8		2.562	12655	0.40	3153
9		2.702	21952	0.69	2977
10		2.832	60370	1.89	6024
11		3.991	6751	0.21	824
12		4.562	9656	0.30	1286

Reported by User: Saneesh (Saneesh) Project Name: HDI_Stellenbosch_2016

	Peak Name	RT	Area	% Area	Height
13		6.807	9208	0.29	796
14		7.059	6973	0.22	719
15		7.366	51856	1.62	3984
16	25ODESRIF	7.761	1290064	40.39	212678
17	RIF	8.100			
18		8.848	8683	0.27	1438
19		9.094	1755	0.05	306
20		9.499	2868	0.09	276
21		9.827	8347	0.26	481
22		10.411	8212	0.26	1053
23	NEO	10.663	294124	9.21	44883
24		10.973	26639	0.83	3068

SAMPLE INFORMATION

Sample Name:	RIF Inc Basil_MeOH BMRc5 2min 2	Acquired By:	Saneesh
Sample Type:	Unknown	Date Acquired:	2017/02/07 11:37:26 PM
Vial:	6	Acq. Method Set:	HDI_RIF_25ODESRIF_NEO
Injection #:	1	Date Processed:	2017/02/08 06:54:17 AM
Injection Volume:	15.00 ul	Processing Method:	HDI_RIF_ W2996 254.0nm-1.2
Run Time:	11.5 Minutes	Channel Name:	W2996 254.0nm-1.2
Sample Set Name:	RIF_Basil MeOH Controls 7_1_17	Proc. Chnl. Descr.:	W2996 PDA 254.0 nm at 1.2

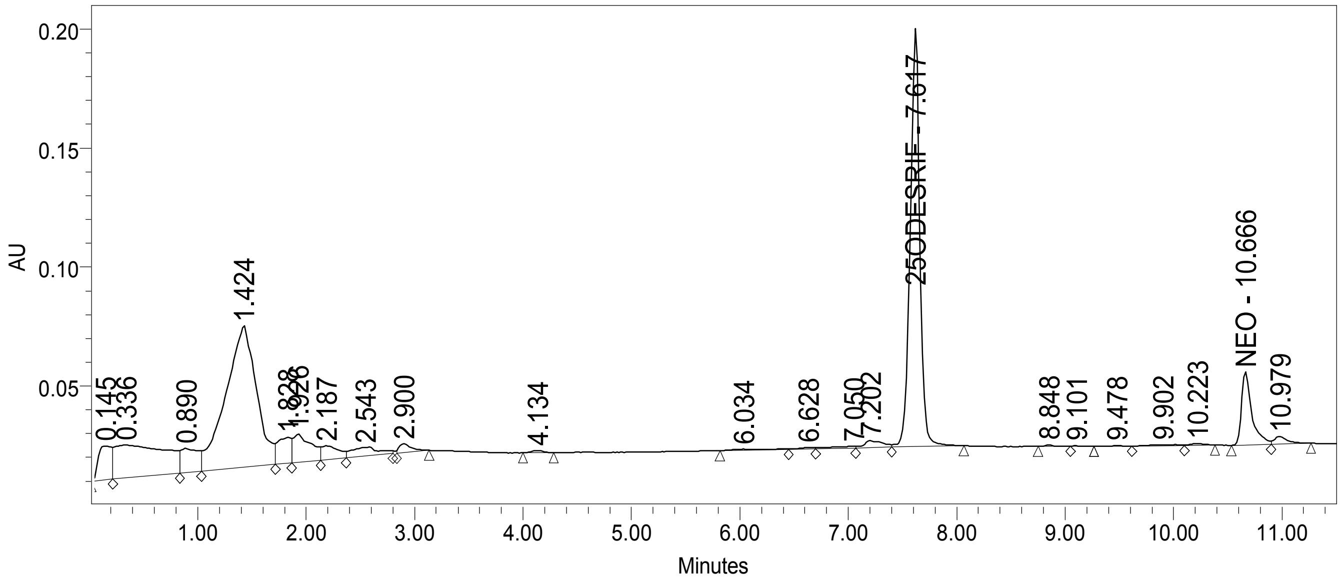

	Peak Name	RT	Area	% Area	Height
1		0.145	121865	3.36	14436
2		0.336	439896	12.11	13846
3		0.890	112035	3.08	10150
4		1.424	1177112	32.41	59192
5		1.828	91666	2.52	10773
6		1.926	136453	3.76	11710
7		2.187	62230	1.71	5832
8		2.543	65830	1.81	3554
9		2.900	29407	0.81	3665
10		4.134	7598	0.21	1045
11		6.034	8398	0.23	422
12		6.628	5259	0.14	609

Reported by User: Saneesh (Saneesh) Project Name: HDI_Stellenbosch_2016

	Peak Name	RT	Area	% Area	Height
13		7.050	11595	0.32	664
14		7.202	34803	0.96	2929
15	25ODESRIF	7.617	1075071	29.60	173879
16	RIF	8.100			
17		8.848	4821	0.13	737
18		9.101	2585	0.07	392
19		9.478	3010	0.08	322
20		9.902	7726	0.21	418
21		10.223	5522	0.15	573
22	NEO	10.666	201466	5.55	30341
23		10.979	27817	0.77	3145

S A M P L E I N F O R M A T I O N

Sample Name:	RIF Inc Basil_MeOH BMRc6 2min 3	Acquired By:	Saneesh
Sample Type:	Unknown	Date Acquired:	2017/02/07 11:52:11 PM
Vial:	7	Acq. Method Set:	HDI_RIF_25ODESRIF_NEO
Injection #:	1	Date Processed:	2017/02/08 06:54:17 AM
Injection Volume:	15.00 ul	Processing Method:	HDI_RIF_ W2996 254.0nm-1.2
Run Time:	11.5 Minutes	Channel Name:	W2996 254.0nm-1.2
Sample Set Name:	RIF_Basil MeOH Controls 7_1_17	Proc. Chnl. Descr.:	W2996 PDA 254.0 nm at 1.2

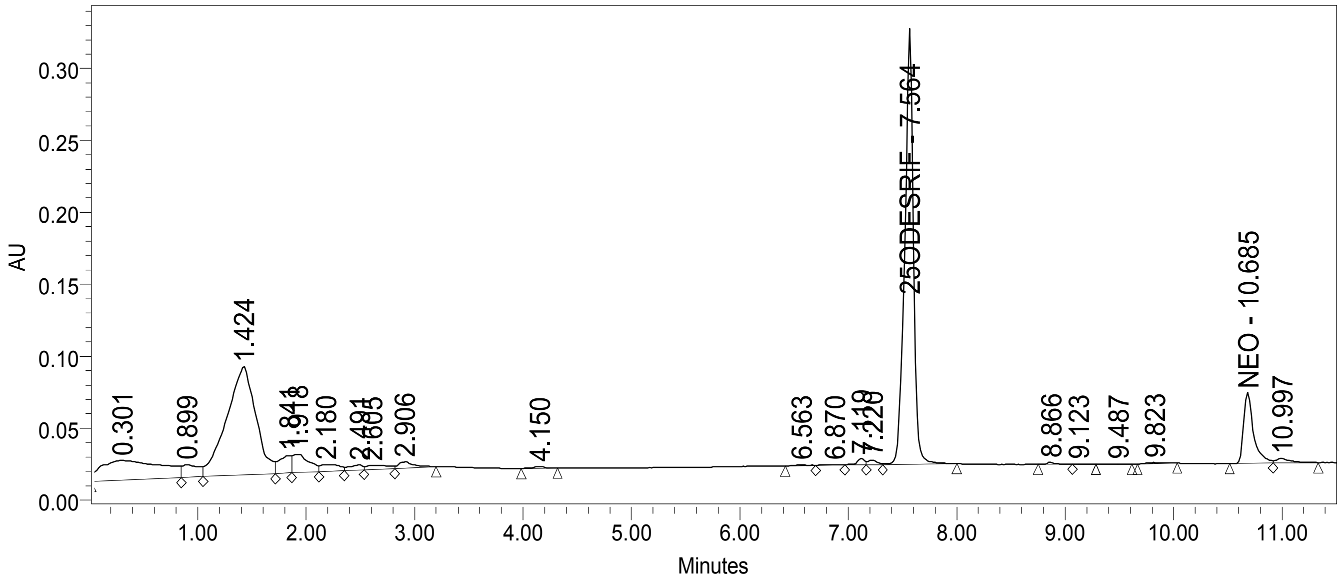

	Peak Name	RT	Area	% Area	Height
1		0.301	512532	11.37	13463
2		0.899	91031	2.02	8626
3		1.424	1404966	31.16	75400
4		1.841	93944	2.08	11949
5		1.918	133789	2.97	12563
6		2.180	56108	1.24	4781
7		2.491	30000	0.67	3359
8		2.605	37307	0.83	2677
9		2.906	41076	0.91	4259
10		4.150	8432	0.19	1136
11		6.563	5636	0.12	759
12		6.870	6201	0.14	532

Reported by User: Saneesh (Saneesh) Project Name: HDI_Stellenbosch_2016

	Peak Name	RT	Area	% Area	Height
13		7.119	23247	0.52	4428
14		7.220	19697	0.44	3300
15	25ODESRIF	7.564	1683424	37.33	296346
16	RIF	8.100			
17		8.866	8151	0.18	1366
18		9.123	1651	0.04	252
19		9.487	2608	0.06	246
20		9.823	8140	0.18	728
21	NEO	10.685	310924	6.90	48771
22		10.997	30235	0.67	3188

SAMPLE INFORMATION

Sample Name:	RIF Inc Basil_MeOH BMRc7 5min 1	Acquired By:	Saneesh
Sample Type:	Unknown	Date Acquired:	2017/02/08 12:06:55 AM
Vial:	8	Acq. Method Set:	HDI_RIF_25ODESRIF_NEO
Injection #:	1	Date Processed:	2017/02/08 06:54:17 AM
Injection Volume:	15.00 ul	Processing Method:	HDI_RIF_ W2996 254.0nm-1.2
Run Time:	11.5 Minutes	Channel Name:	W2996 254.0nm-1.2
Sample Set Name:	RIF_Basil MeOH Controls 7_1_17	Proc. Chnl. Descr.:	W2996 PDA 254.0 nm at 1.2

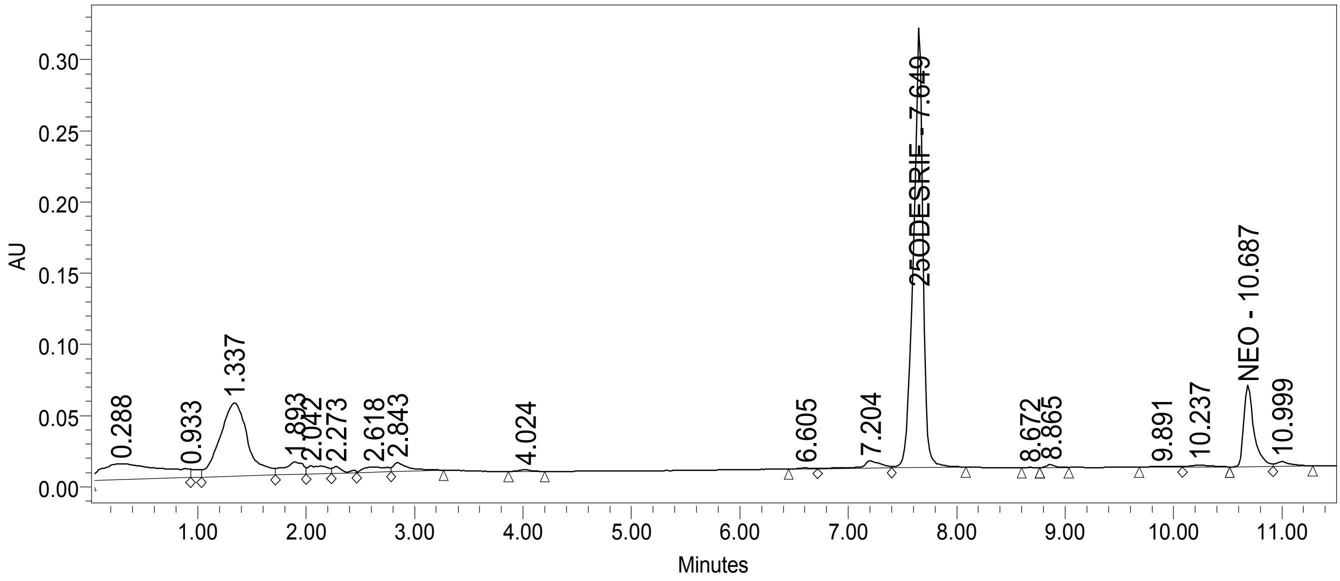

	Peak Name	RT	Area	% Area	Height
1		0.288	435359	10.58	11019
2		0.933	32046	0.78	5734
3		1.337	921050	22.38	51563
4		1.893	109596	2.66	9046
5		2.042	68254	1.66	5538
6		2.273	32401	0.79	4688
7		2.618	55679	1.35	3575
8		2.843	60529	1.47	6357
9		4.024	9770	0.24	1132
10		6.605	6801	0.17	816
11		7.204	63186	1.54	5204
12	25ODESRIF	7.649	1884668	45.80	304639

	Peak Name	RT	Area	% Area	Height
13	RIF	8.100			
14		8.672	1295	0.03	244
15		8.865	10802	0.26	1967
16		9.891	8938	0.22	502
17		10.237	17004	0.41	1326
18	NEO	10.687	368129	8.95	56537
19		10.999	29179	0.71	3183

Reported by User: Saneesh (Saneesh) Project Name: HDI_Stellenbosch_2016

SAMPLE INFORMATION

Sample Name:	RIF Inc Basil_MeOH BMRc8 5min 2	Acquired By:	Saneesh
Sample Type:	Unknown	Date Acquired:	2017/02/08 12:21:40 AM
Vial:	9	Acq. Method Set:	HDI_RIF_25ODESRIF_NEO
Injection #:	1	Date Processed:	2017/02/08 06:54:17 AM
Injection Volume:	15.00 ul	Processing Method:	HDI_RIF_ W2996 254.0nm-1.2
Run Time:	11.5 Minutes	Channel Name:	W2996 254.0nm-1.2
Sample Set Name:	RIF_Basil MeOH Controls 7_1_17	Proc. Chnl. Descr.:	W2996 PDA 254.0 nm at 1.2

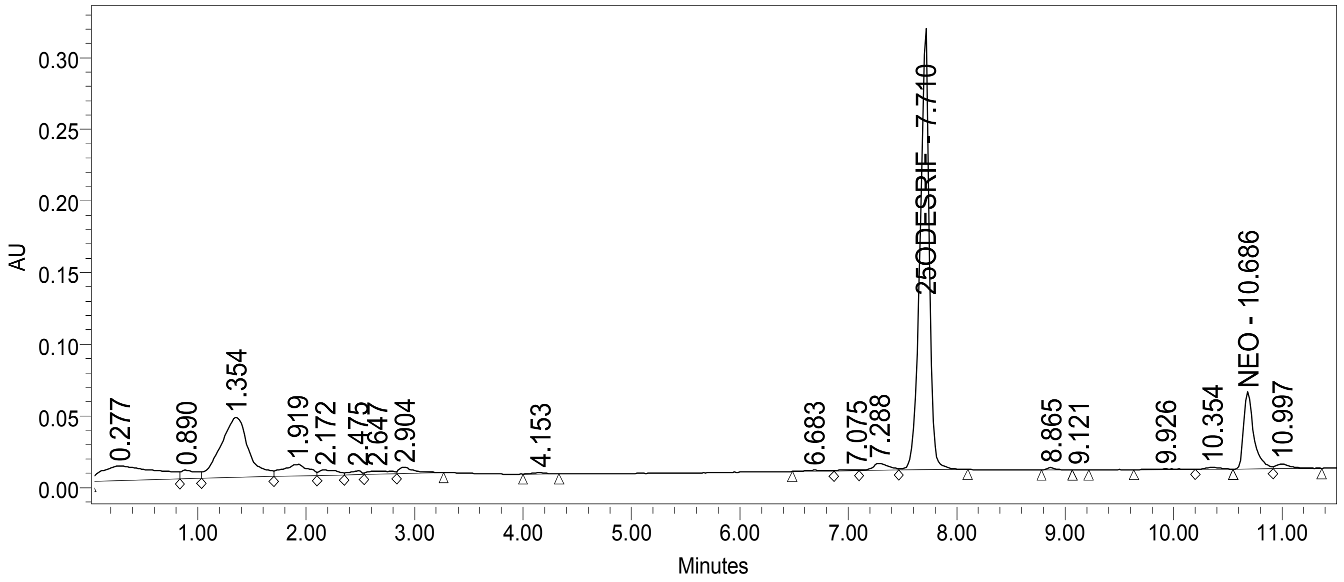

	Peak Name	RT	Area	% Area	Height
1		0.277	350139	9.29	9996
2		0.890	60954	1.62	5969
3		1.354	748335	19.86	41983
4		1.919	133764	3.55	8149
5		2.172	42980	1.14	3660
6		2.475	19433	0.52	2562
7		2.647	29508	0.78	2041
8		2.904	39875	1.06	4217
9		4.153	7312	0.19	992
10		6.683	8950	0.24	707
11		7.075	7869	0.21	667
12		7.288	52137	1.38	5023

Reported by User: Saneesh (Saneesh)   Project Name: HDI_Stellenbosch_2016

	Peak Name	RT	Area	% Area	Height
13	25ODESRIF	7.710	1857151	49.29	304612
14	RIF	8.100			
15		8.865	9602	0.25	1707
16		9.121	1080	0.03	220
17		9.926	9316	0.25	470
18		10.354	12851	0.34	1281
19	NEO	10.686	345520	9.17	53333
20		10.997	30917	0.82	3211

Multi Sample Summary

Reported by User: Saneesh (Saneesh)

Project Name: HDI_Stellenbosch_2016

SAMPLE INFORMATION

Sample Name:	RIF Inc Basil_MeOH BMRc9 5min 3	Acquired By:	Saneesh
Sample Type:	Unknown	Date Acquired:	2017/02/08 12:36:23 AM
Vial:	10	Acq. Method Set:	HDI_RIF_25ODESRIF_NEO
Injection #:	1	Date Processed:	2017/02/08 06:54:17 AM
Injection Volume:	15.00 ul	Processing Method:	HDI_RIF_ W2996 254.0nm-1.2
Run Time:	11.5 Minutes	Channel Name:	W2996 254.0nm-1.2
Sample Set Name:	RIF_Basil MeOH Controls 7_1_17	Proc. Chnl. Descr.:	W2996 PDA 254.0 nm at 1.2

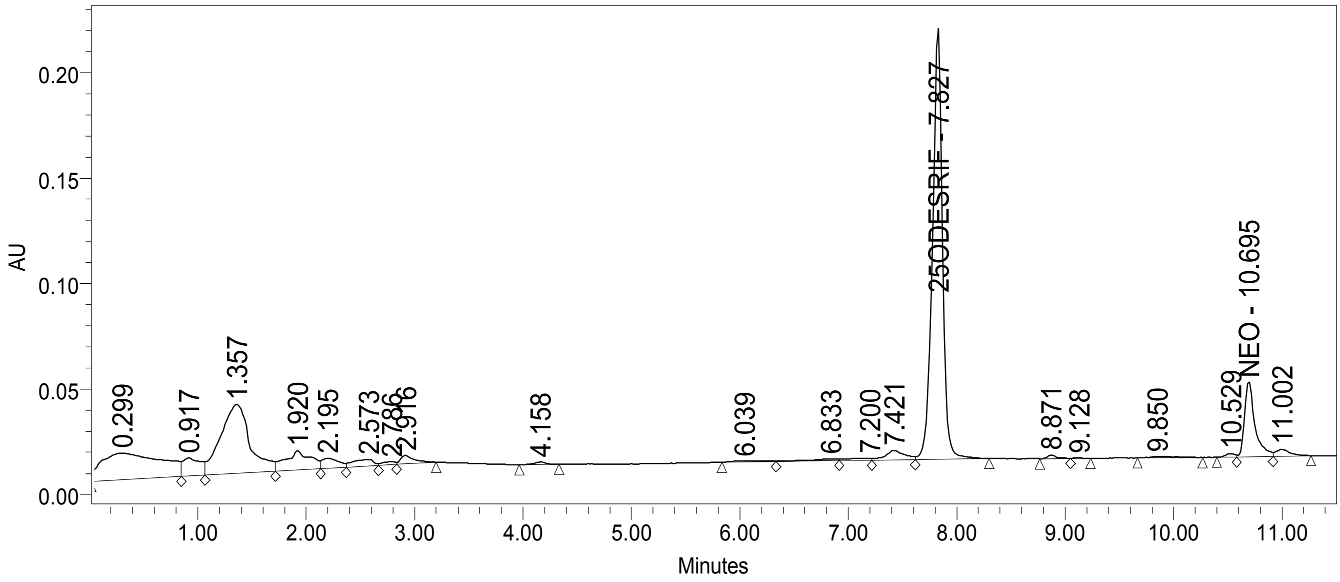

	Peak Name	RT	Area	% Area	Height
1		0.299	471760	14.89	12497
2		0.917	94360	2.98	8436
3		1.357	611268	19.30	32463
4		1.920	143618	4.53	9052
5		2.195	48739	1.54	4689
6		2.573	42722	1.35	3189
7		2.786	11562	0.37	1344
8		2.916	34385	1.09	3920
9		4.158	8418	0.27	1156
10		6.039	8268	0.26	476
11		6.833	15764	0.50	893
12		7.200	15215	0.48	951

	Peak Name	RT	Area	% Area	Height
13		7.421	54327	1.72	4466
14	25ODESRIF	7.827	1307832	41.29	203547
15	RIF	8.100			
16		8.871	10591	0.33	1802
17		9.128	2769	0.09	426
18		9.850	11557	0.36	626
19		10.529	10212	0.32	1670
20	NEO	10.695	234681	7.41	35371
21		11.002	29533	0.93	3231

Reported by User: Saneesh (Saneesh)   Project Name: HDI_Stellenbosch_2016

SAMPLE INFORMATION

Sample Name:	RIF Inc Basil_MeOH BMRc10 10min1	Acquired By:	Saneesh
Sample Type:	Unknown	Date Acquired:	2017/02/08 12:51:08 AM
Vial:	11	Acq. Method Set:	HDI_RIF_25ODESRIF_NEO
Injection #:	1	Date Processed:	2017/02/08 06:54:17 AM
Injection Volume:	15.00 ul	Processing Method:	HDI_RIF_ W2996 254.0nm-1.2
Run Time:	11.5 Minutes	Channel Name:	W2996 254.0nm-1.2
Sample Set Name:	RIF_Basil MeOH Controls 7_1_17	Proc. Chnl. Descr.:	W2996 PDA 254.0 nm at 1.2

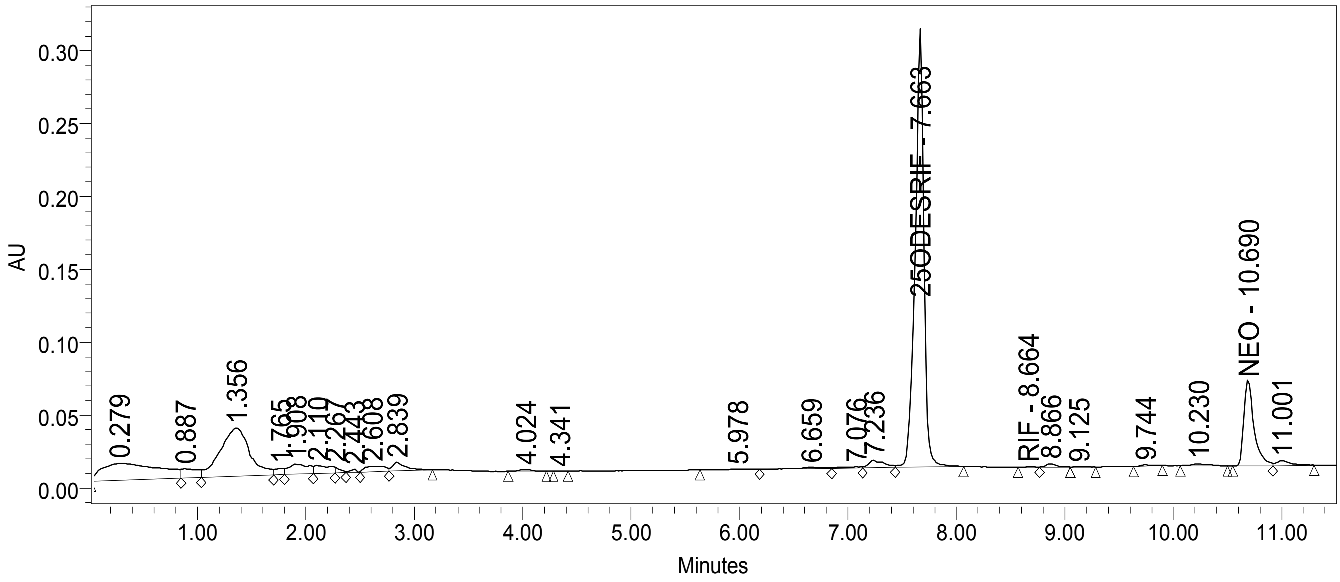

	Peak Name	RT	Area	% Area	Height
1		0.279	417731	11.56	11499
2		0.887	63044	1.74	6349
3		1.356	627259	17.36	32950
4		1.765	24794	0.69	4351
5		1.908	89069	2.47	6927
6		2.110	55670	1.54	5364
7		2.267	12565	0.35	4107
8		2.443	8855	0.25	2078
9		2.608	46256	1.28	3599
10		2.839	49564	1.37	5824
11		4.024	9952	0.28	1158
12		4.341	397	0.01	75

Reported by User: Saneesh (Saneesh) Project Name: HDI_Stellenbosch_2016

	Peak Name	RT	Area	% Area	Height
13		5.978	4373	0.12	244
14		6.659	13505	0.37	862
15		7.076	10547	0.29	735
16		7.236	49239	1.36	5145
17	25ODESRIF	7.663	1680669	46.52	293381
18	RIF	8.664	1214	0.03	206
19		8.866	13311	0.37	2232
20		9.125	1703	0.05	294
21		9.744	6178	0.17	926
22		10.230	15763	0.44	1383
23	NEO	10.690	381017	10.55	58746
24		11.001	30343	0.84	3243

SAMPLE INFORMATION

Sample Name:	RIF Inc Basil_MeOH BMRc11 10min2	Acquired By:	Saneesh
Sample Type:	Unknown	Date Acquired:	2017/02/08 01:05:53 AM
Vial:	12	Acq. Method Set:	HDI_RIF_25ODESRIF_NEO
Injection #:	1	Date Processed:	2017/02/08 06:54:17 AM
Injection Volume:	15.00 ul	Processing Method:	HDI_RIF_ W2996 254.0nm-1.2
Run Time:	11.5 Minutes	Channel Name:	W2996 254.0nm-1.2
Sample Set Name:	RIF_Basil MeOH Controls 7_1_17	Proc. Chnl. Descr.:	W2996 PDA 254.0 nm at 1.2

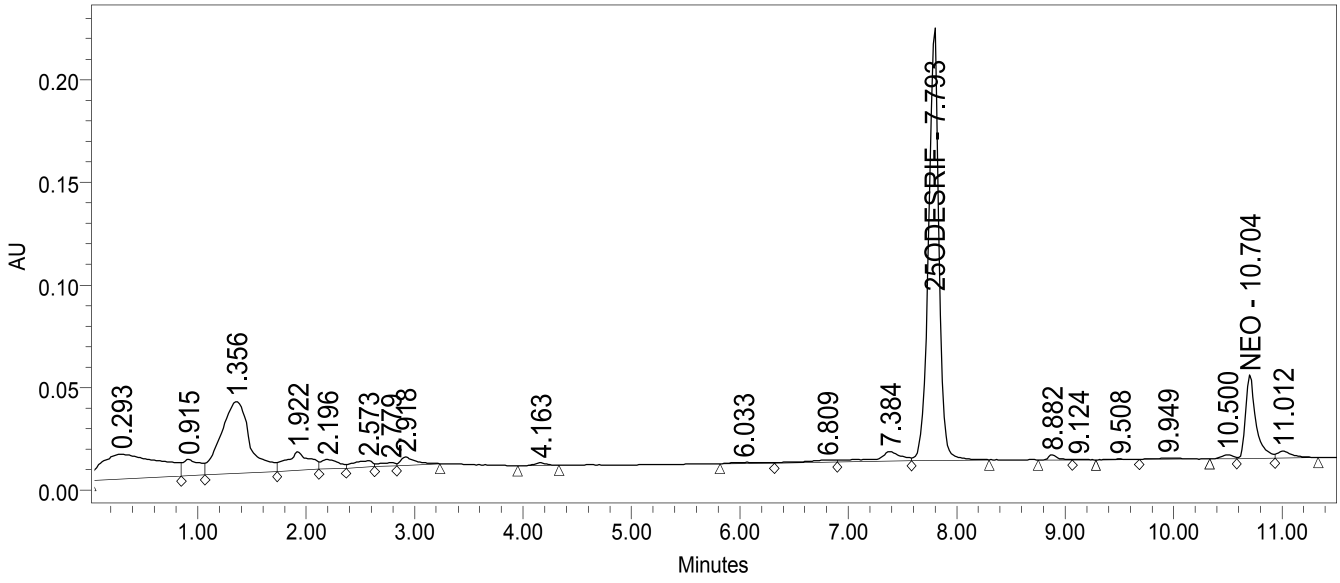

	Peak Name	RT	Area	% Area	Height
1		0.293	443265	13.25	11974
2		0.915	85791	2.56	7790
3		1.356	652277	19.50	34968
4		1.922	135356	4.05	9061
5		2.196	51327	1.53	4535
6		2.573	39426	1.18	3123
7		2.779	16893	0.50	1573
8		2.918	36692	1.10	3933
9		4.163	10673	0.32	1370
10		6.033	10512	0.31	527
11		6.809	19728	0.59	1014
12		7.384	74106	2.22	4745

	Peak Name	RT	Area	% Area	Height
13	25ODESRIF	7.793	1423746	42.56	210630
14	RIF	8.100			
15		8.882	14609	0.44	2407
16		9.124	2890	0.09	427
17		9.508	4939	0.15	418
18		9.949	11886	0.36	553
19		10.500	13156	0.39	1787
20	NEO	10.704	267421	7.99	40518
21		11.012	30651	0.92	3303

SAMPLE INFORMATION

Sample Name:	RIF Inc Basil_MeOH BMRc12 10min3	Acquired By:	Saneesh
Sample Type:	Unknown	Date Acquired:	2017/02/08 01:20:37 AM
Vial:	13	Acq. Method Set:	HDI_RIF_25ODESRIF_NEO
Injection #:	1	Date Processed:	2017/02/08 06:54:17 AM
Injection Volume:	15.00 ul	Processing Method:	HDI_RIF_ W2996 254.0nm-1.2
Run Time:	11.5 Minutes	Channel Name:	W2996 254.0nm-1.2
Sample Set Name:	RIF_Basil MeOH Controls 7_1_17	Proc. Chnl. Descr.:	W2996 PDA 254.0 nm at 1.2

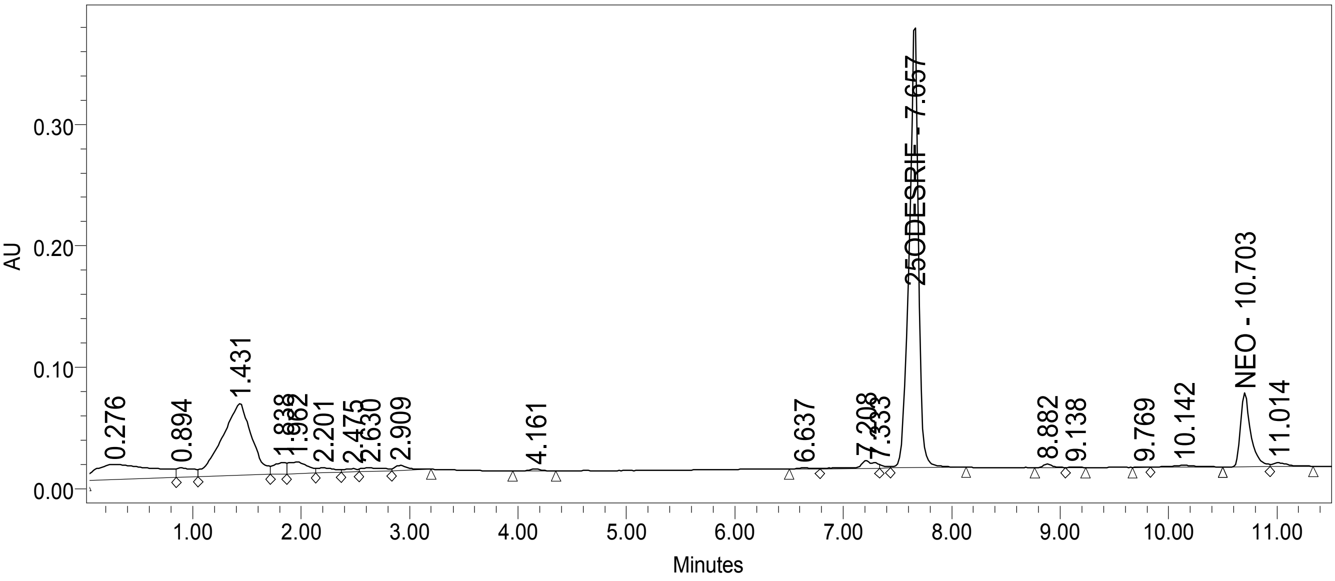

	Peak Name	RT	Area	% Area	Height
1		0.276	460301	9.70	12217
2		0.894	82571	1.74	8056
3		1.431	1120506	23.61	59261
4		1.838	76948	1.62	9487
5		1.962	120639	2.54	9629
6		2.201	46062	0.97	4179
7		2.475	22805	0.48	2790
8		2.630	41764	0.88	3010
9		2.909	36238	0.76	4014
10		4.161	12476	0.26	1637
11		6.637	7779	0.16	977
12		7.208	60911	1.28	6190

	Peak Name	RT	Area	% Area	Height
13		7.333	9648	0.20	2913
14	25ODESRIF	7.657	2167196	45.66	361227
15	RIF	8.100			
16		8.882	16359	0.34	2760
17		9.138	3396	0.07	550
18		9.769	1816	0.04	256
19		10.142	29534	0.62	1499
20	NEO	10.703	398512	8.40	60675
21		11.014	30440	0.64	3162

SAMPLE INFORMATION

Sample Name:	RIF Inc Basil_MeOH BMRc13 15min1	Acquired By:	Saneesh
Sample Type:	Unknown	Date Acquired:	2017/02/08 01:35:22 AM
Vial:	14	Acq. Method Set:	HDI_RIF_25ODESRIF_NEO
Injection #:	1	Date Processed:	2017/02/08 06:54:17 AM
Injection Volume:	15.00 ul	Processing Method:	HDI_RIF_ W2996 254.0nm-1.2
Run Time:	11.5 Minutes	Channel Name:	W2996 254.0nm-1.2
Sample Set Name:	RIF_Basil MeOH Controls 7_1_17	Proc. Chnl. Descr.:	W2996 PDA 254.0 nm at 1.2

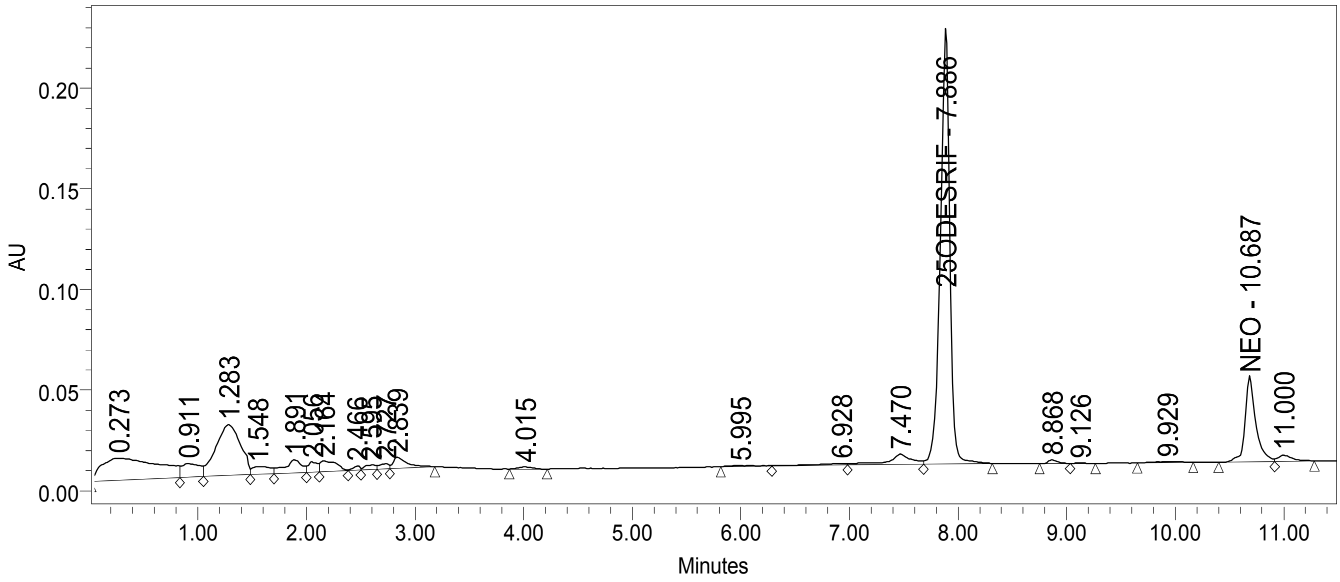

	Peak Name	RT	Area	% Area	Height
1		0.273	390144	13.29	10873
2		0.911	78048	2.66	6779
3		1.283	397471	13.54	25145
4		1.548	43460	1.48	3811
5		1.891	69331	2.36	6435
6		2.056	29850	1.02	5008
7		2.164	53799	1.83	5249
8		2.466	8842	0.30	2126
9		2.595	16219	0.55	2217
10		2.727	15441	0.53	2606
11		2.839	49936	1.70	5670
12		4.015	8525	0.29	965

	Peak Name	RT	Area	% Area	Height
13		5.995	7761	0.26	413
14		6.928	20535	0.70	884
15		7.470	77894	2.65	5150
16	25ODESRIF	7.886	1324201	45.09	215203
17	RIF	8.100			
18		8.868	10358	0.35	1841
19		9.126	1795	0.06	308
20		9.929	9074	0.31	454
21	NEO	10.687	294572	10.03	42421
22		11.000	29331	1.00	3208

SAMPLE INFORMATION

Sample Name:	RIF Inc Basil_MeOH BMRc14 15min2	Acquired By:	Saneesh
Sample Type:	Unknown	Date Acquired:	2017/02/08 01:50:07 AM
Vial:	15	Acq. Method Set:	HDI_RIF_25ODESRIF_NEO
Injection #:	1	Date Processed:	2017/02/08 06:54:17 AM
Injection Volume:	15.00 ul	Processing Method:	HDI_RIF_ W2996 254.0nm-1.2
Run Time:	11.5 Minutes	Channel Name:	W2996 254.0nm-1.2
Sample Set Name:	RIF_Basil MeOH Controls 7_1_17	Proc. Chnl. Descr.:	W2996 PDA 254.0 nm at 1.2

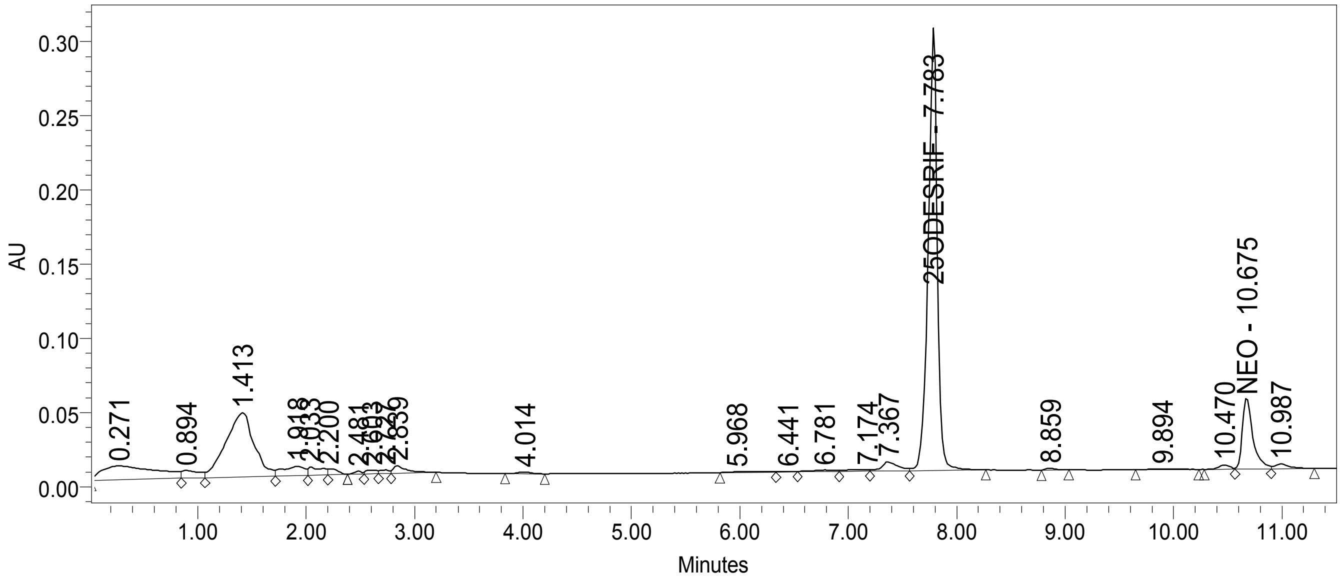

	Peak Name	RT	Area	% Area	Height
1		0.271	323734	8.81	9254
2		0.894	56200	1.53	5074
3		1.413	784849	21.35	43024
4		1.918	91215	2.48	6319
5		2.033	50309	1.37	5552
6		2.200	24921	0.68	3978
7		2.481	11262	0.31	2233
8		2.603	18393	0.50	2598
9		2.727	15159	0.41	2508
10		2.839	44519	1.21	5246
11		4.014	12033	0.33	1349
12		5.968	7328	0.20	381

Reported by User: Saneesh (Saneesh) Project Name: HDI_Stellenbosch_2016

	Peak Name	RT	Area	% Area	Height
13		6.441	2476	0.07	236
14		6.781	14317	0.39	1006
15		7.174	14279	0.39	979
16		7.367	64595	1.76	6216
17	25ODESRIF	7.783	1752370	47.68	293783
18	RIF	8.100			
19		8.859	7620	0.21	1372
20		9.894	9759	0.27	504
21		10.470	20014	0.54	2832
22	NEO	10.675	320456	8.72	47600
23		10.987	29469	0.80	3065

Reported by User: Saneesh (Saneesh) Project Name: HDI_Stellenbosch_2016

SAMPLE INFORMATION

Sample Name:	RIF Inc Basil_MeOH BMRc15 15min3	Acquired By:	Saneesh
Sample Type:	Unknown	Date Acquired:	2017/02/08 02:04:50 AM
Vial:	16	Acq. Method Set:	HDI_RIF_25ODESRIF_NEO
Injection #:	1	Date Processed:	2017/02/08 06:54:17 AM
Injection Volume:	15.00 ul	Processing Method:	HDI_RIF_ W2996 254.0nm-1.2
Run Time:	11.5 Minutes	Channel Name:	W2996 254.0nm-1.2
Sample Set Name:	RIF_Basil MeOH Controls 7_1_17	Proc. Chnl. Descr.:	W2996 PDA 254.0 nm at 1.2

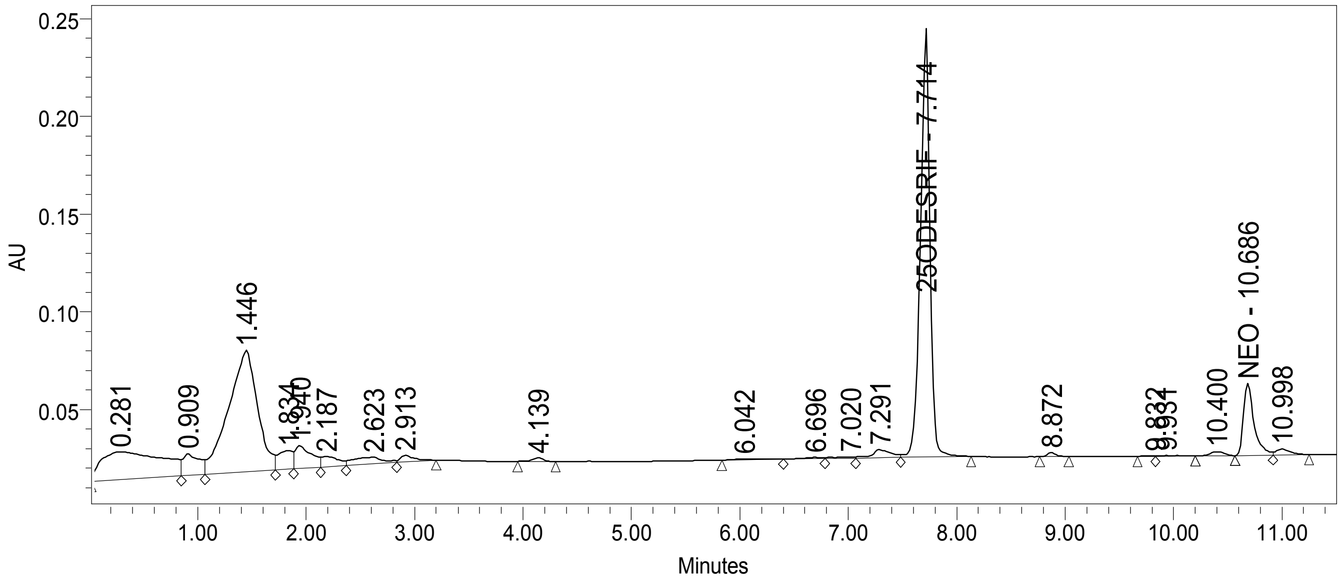

	Peak Name	RT	Area	% Area	Height
1		0.281	541870	13.77	14058
2		0.909	115305	2.93	10968
3		1.446	1158493	29.43	61962
4		1.834	87746	2.23	9383
5		1.940	124064	3.15	11645
6		2.187	56920	1.45	5249
7		2.623	69366	1.76	3543
8		2.913	30685	0.78	3394
9		4.139	14268	0.36	1901
10		6.042	10107	0.26	564
11		6.696	8833	0.22	715
12		7.020	9791	0.25	629

Reported by User: Saneesh (Saneesh) Project Name: HDI_Stellenbosch_2016

	Peak Name	RT	Area	% Area	Height
13		7.291	49555	1.26	4200
14	25ODESRIF	7.714	1346402	34.20	216957
15	RIF	8.100			
16		8.872	11947	0.30	2106
17		9.832	2034	0.05	251
18		9.931	6573	0.17	446
19		10.400	18637	0.47	2023
20	NEO	10.686	247223	6.28	36604
21		10.998	26678	0.68	2987

SAMPLE INFORMATION

Sample Name:	RIF Inc Basil_MeOH BMRc16 20min1	Acquired By:	Saneesh
Sample Type:	Unknown	Date Acquired:	2017/02/08 02:19:25 AM
Vial:	17	Acq. Method Set:	HDI_RIF_25ODESRIF_NEO
Injection #:	1	Date Processed:	2017/02/08 06:54:17 AM
Injection Volume:	15.00 ul	Processing Method:	HDI_RIF_ W2996 254.0nm-1.2
Run Time:	11.5 Minutes	Channel Name:	W2996 254.0nm-1.2
Sample Set Name:	RIF_Basil MeOH Controls 7_1_17	Proc. Chnl. Descr.:	W2996 PDA 254.0 nm at 1.2

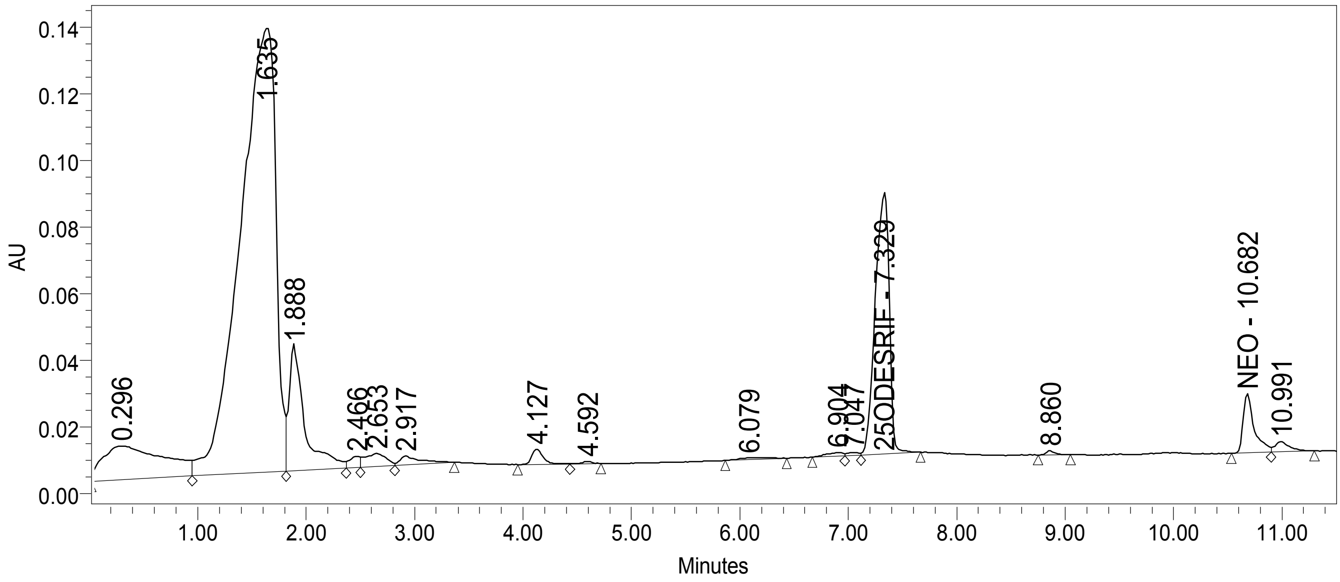

	Peak Name	RT	Area	% Area	Height
1		0.296	392182	8.09	10140
2		1.635	3032671	62.56	133217
3		1.888	377935	7.80	37713
4		2.466	22047	0.45	3278
5		2.653	52547	1.08	3836
6		2.917	33923	0.70	2559
7		4.127	32801	0.68	4532
8		4.592	4381	0.09	693
9		6.079	11248	0.23	596
10		6.904	12475	0.26	1151
11		7.047	6844	0.14	901
12	25ODESRIF	7.329	705956	14.56	78911

Reported by User: Saneesh (Saneesh)　　Project Name: HDI_Stellenbosch_2016

	Peak Name	RT	Area	% Area	Height
13	RIF	8.100			
14		8.860	6969	0.14	1207
15	NEO	10.682	126006	2.60	17575
16		10.991	29945	0.62	3045

Reported by User: Saneesh (Saneesh) Project Name: HDI_Stellenbosch_2016

SAMPLE INFORMATION

Sample Name:	RIF Inc Basil_MeOH BMRc17 20min2	Acquired By:	Saneesh
Sample Type:	Unknown	Date Acquired:	2017/02/08 02:34:10 AM
Vial:	18	Acq. Method Set:	HDI_RIF_25ODESRIF_NEO
Injection #:	1	Date Processed:	2017/02/08 06:54:17 AM
Injection Volume:	15.00 ul	Processing Method:	HDI_RIF_ W2996 254.0nm-1.2
Run Time:	11.5 Minutes	Channel Name:	W2996 254.0nm-1.2
Sample Set Name:	RIF_Basil MeOH Controls 7_1_17	Proc. Chnl. Descr.:	W2996 PDA 254.0 nm at 1.2

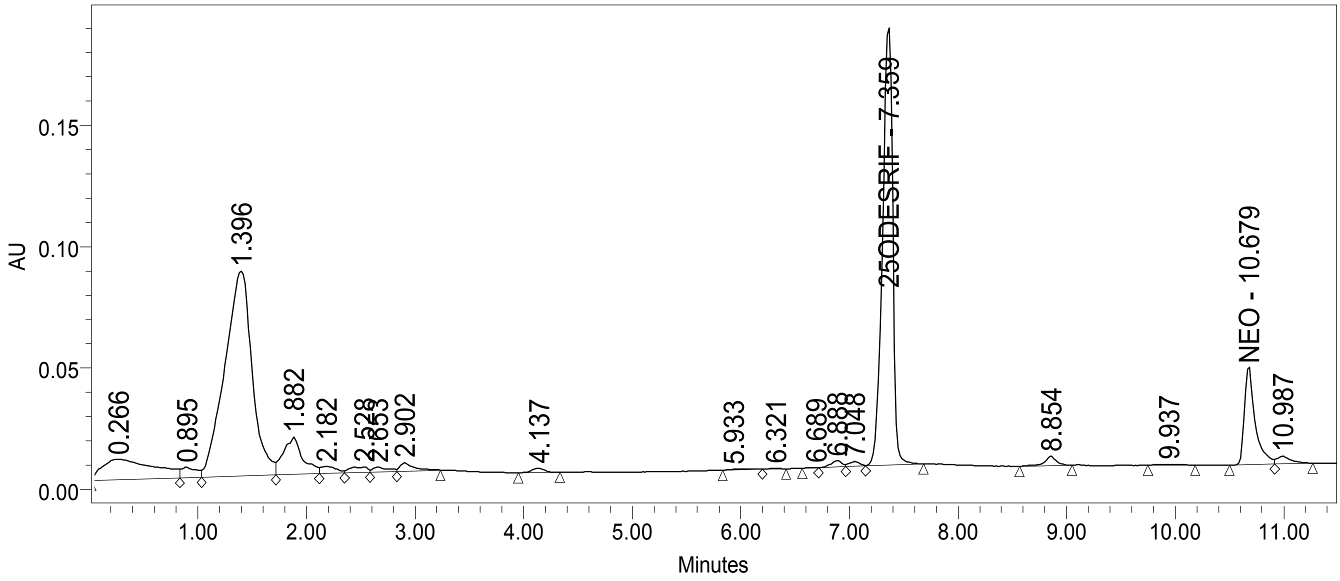

	Peak Name	RT	Area	% Area	Height
1		0.266	271075	7.40	8224
2		0.895	41111	1.12	4196
3		1.396	1470631	40.12	84553
4		1.882	189741	5.18	14987
5		2.182	29242	0.80	2894
6		2.528	25537	0.70	2305
7		2.653	19726	0.54	1973
8		2.902	29463	0.80	3485
9		4.137	13629	0.37	1778
10		5.933	4424	0.12	325
11		6.321	2830	0.08	377
12		6.689	686	0.02	77

Reported by User: Saneesh (Saneesh) Project Name: HDI_Stellenbosch_2016

	Peak Name	RT	Area	% Area	Height
13		6.888	18366	0.50	2544
14		7.048	12988	0.35	1911
15	25ODESRIF	7.359	1194977	32.60	182859
16	RIF	8.100			
17		8.854	29878	0.82	3947
18		9.937	5564	0.15	340
19	NEO	10.679	277694	7.58	39984
20		10.987	27880	0.76	3065

SAMPLE INFORMATION

Sample Name:	RIF Inc Basil_MeOH BMRc18 20min3	Acquired By:	Saneesh
Sample Type:	Unknown	Date Acquired:	2017/02/08 02:48:53 AM
Vial:	19	Acq. Method Set:	HDI_RIF_25ODESRIF_NEO
Injection #:	1	Date Processed:	2017/02/08 06:54:17 AM
Injection Volume:	15.00 ul	Processing Method:	HDI_RIF_ W2996 254.0nm-1.2
Run Time:	11.5 Minutes	Channel Name:	W2996 254.0nm-1.2
Sample Set Name:	RIF_Basil MeOH Controls 7_1_17	Proc. Chnl. Descr.:	W2996 PDA 254.0 nm at 1.2

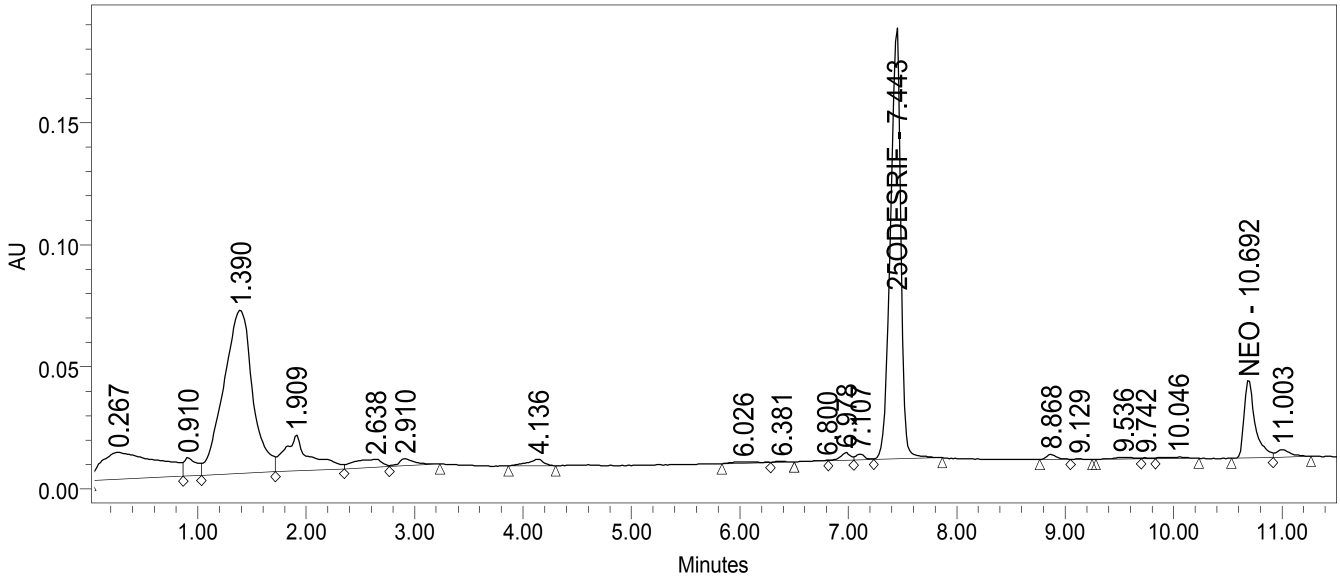

	Peak Name	RT	Area	% Area	Height
1		0.267	395352	11.06	10852
2		0.910	59822	1.67	7345
3		1.390	1247231	34.89	66780
4		1.909	240734	6.73	14494
5		2.638	60457	1.69	3178
6		2.910	31392	0.88	3095
7		4.136	28461	0.80	2661
8		6.026	9103	0.25	619
9		6.381	2805	0.08	411
10		6.800	2476	0.07	249
11		6.978	20803	0.58	3184
12		7.107	14568	0.41	2389

Reported by User: Saneesh (Saneesh) Project Name: HDI_Stellenbosch_2016

	Peak Name	RT	Area	% Area	Height
13	25ODESRIF	7.443	1173301	32.82	178362
14	RIF	8.100			
15		8.868	12987	0.36	2148
16		9.129	1425	0.04	246
17		9.536	9602	0.27	701
18		9.742	3237	0.09	457
19		10.046	9671	0.27	605
20	NEO	10.692	223427	6.25	31793
21		11.003	28376	0.79	2991

Reported by User: Saneesh (Saneesh) Project Name: HDI_Stellenbosch_2016

SAMPLE INFORMATION

Sample Name:	RIF Inc Basil_MeOH BMRc19 30min1	Acquired By:	Saneesh
Sample Type:	Unknown	Date Acquired:	2017/02/08 03:03:30 AM
Vial:	20	Acq. Method Set:	HDI_RIF_25ODESRIF_NEO
Injection #:	1	Date Processed:	2017/02/08 06:54:17 AM
Injection Volume:	15.00 ul	Processing Method:	HDI_RIF_ W2996 254.0nm-1.2
Run Time:	11.5 Minutes	Channel Name:	W2996 254.0nm-1.2
Sample Set Name:	RIF_Basil MeOH Controls 7_1_17	Proc. Chnl. Descr.:	W2996 PDA 254.0 nm at 1.2

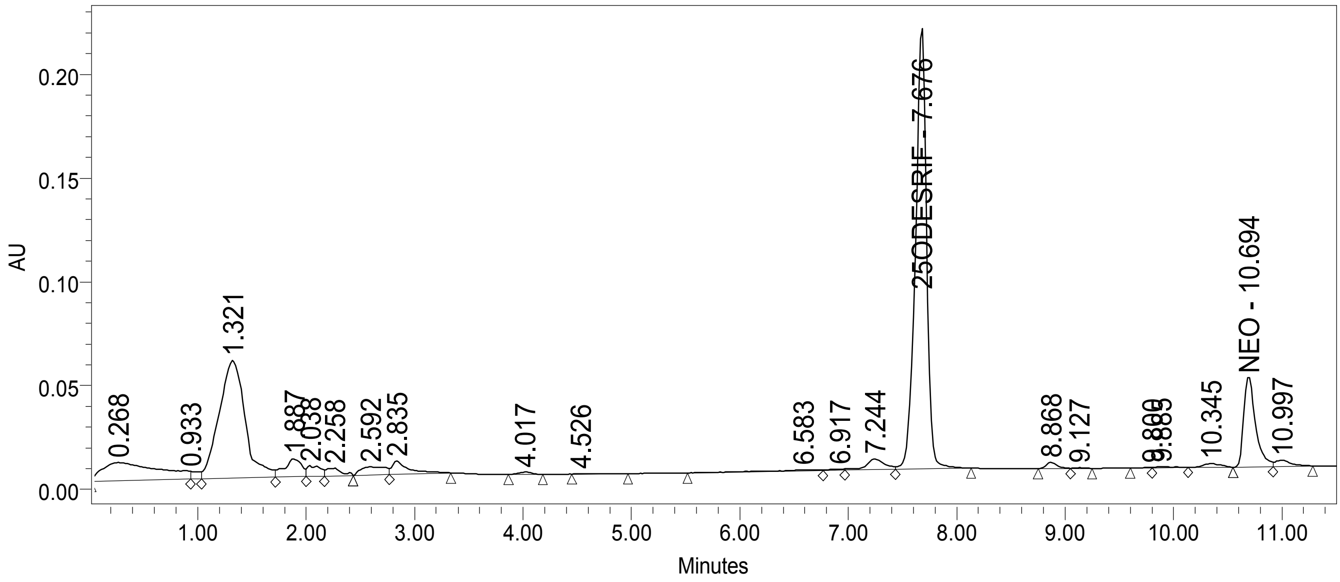

	Peak Name	RT	Area	% Area	Height
1		0.268	316469	8.71	8741
2		0.933	20393	0.56	3643
3		1.321	949652	26.13	56674
4		1.887	96573	2.66	8719
5		2.038	42763	1.18	5022
6		2.258	37722	1.04	3804
7		2.592	62614	1.72	3945
8		2.835	68436	1.88	6473
9		4.017	9458	0.26	1098
10		4.526	2865	0.08	265
11		6.583	18209	0.50	680
12		6.917	7024	0.19	679

Reported by User: Saneesh (Saneesh) Project Name: HDI_Stellenbosch_2016

	Peak Name	RT	Area	% Area	Height
13		7.244	63314	1.74	5359
14	25ODESRIF	7.676	1527522	42.04	212659
15	RIF	8.100			
16		8.868	19708	0.54	2947
17		9.127	2235	0.06	352
18		9.800	1895	0.05	345
19		9.885	7177	0.20	444
20		10.345	21405	0.59	1821
21	NEO	10.694	327482	9.01	43181
22		10.997	30905	0.85	2978

Reported by User: Saneesh (Saneesh)   Project Name: HDI_Stellenbosch_2016

S A M P L E   I N F O R M A T I O N

Sample Name:	RIF Inc Basil_MeOH BMRc20 30min2	Acquired By:	Saneesh
Sample Type:	Unknown	Date Acquired:	2017/02/08 03:18:04 AM
Vial:	21	Acq. Method Set:	HDI_RIF_25ODESRIF_NEO
Injection #:	1	Date Processed:	2017/02/08 06:54:17 AM
Injection Volume:	15.00 ul	Processing Method:	HDI_RIF_ W2996 254.0nm-1.2
Run Time:	11.5 Minutes	Channel Name:	W2996 254.0nm-1.2
Sample Set Name:	RIF_Basil MeOH Controls 7_1_17	Proc. Chnl. Descr.:	W2996 PDA 254.0 nm at 1.2

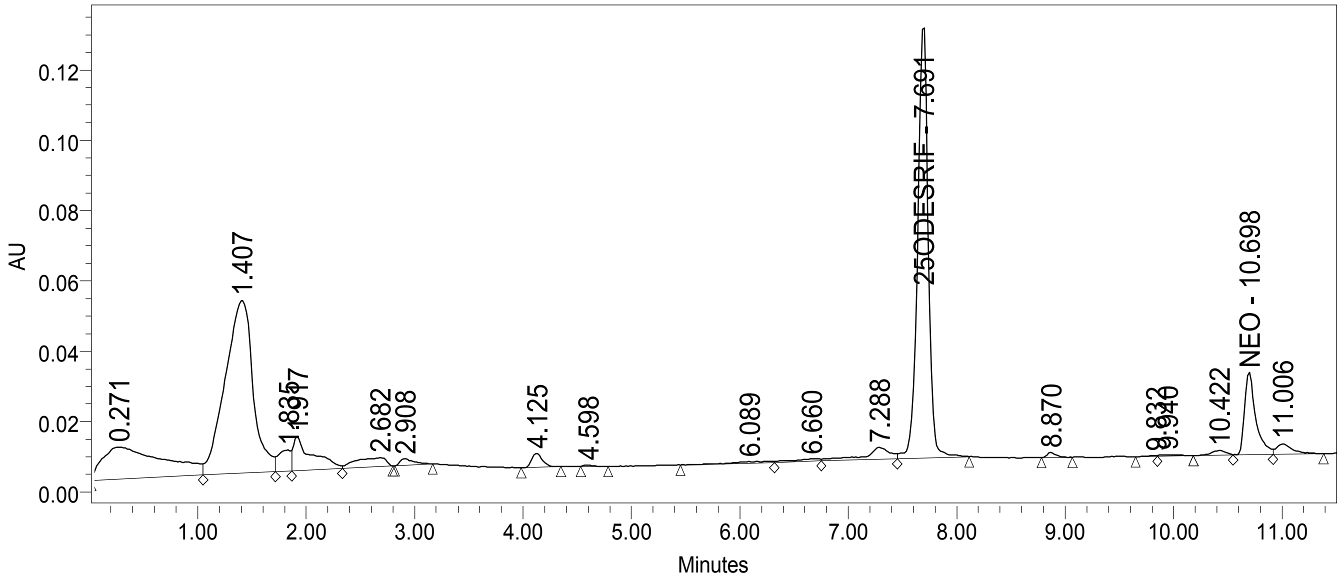

	Peak Name	RT	Area	% Area	Height
1		0.271	352868	13.32	9052
2		1.407	880601	33.23	49190
3		1.835	48696	1.84	5844
4		1.917	118208	4.46	9555
5		2.682	51915	1.96	2559
6		2.908	16474	0.62	1906
7		4.125	27926	1.05	4004
8		4.598	2069	0.08	376
9		6.089	11365	0.43	419
10		6.660	12452	0.47	713
11		7.288	55978	2.11	3447
12	25ODESRIF	7.691	842245	31.78	123490

	Peak Name	RT	Area	% Area	Height
13	RIF	8.100			
14		8.870	8881	0.34	1481
15		9.832	2150	0.08	268
16		9.940	5811	0.22	431
17		10.422	14325	0.54	1398
18	NEO	10.698	167304	6.31	23339
19		11.006	30812	1.16	3008

SAMPLE INFORMATION

Sample Name:	RIF Inc Basil_MeOH BMRc21 30min3	Acquired By:	Saneesh
Sample Type:	Unknown	Date Acquired:	2017/02/08 03:32:39 AM
Vial:	22	Acq. Method Set:	HDI_RIF_25ODESRIF_NEO
Injection #:	1	Date Processed:	2017/02/08 06:54:17 AM
Injection Volume:	15.00 ul	Processing Method:	HDI_RIF_ W2996 254.0nm-1.2
Run Time:	11.5 Minutes	Channel Name:	W2996 254.0nm-1.2
Sample Set Name:	RIF_Basil MeOH Controls 7_1_17	Proc. Chnl. Descr.:	W2996 PDA 254.0 nm at 1.2

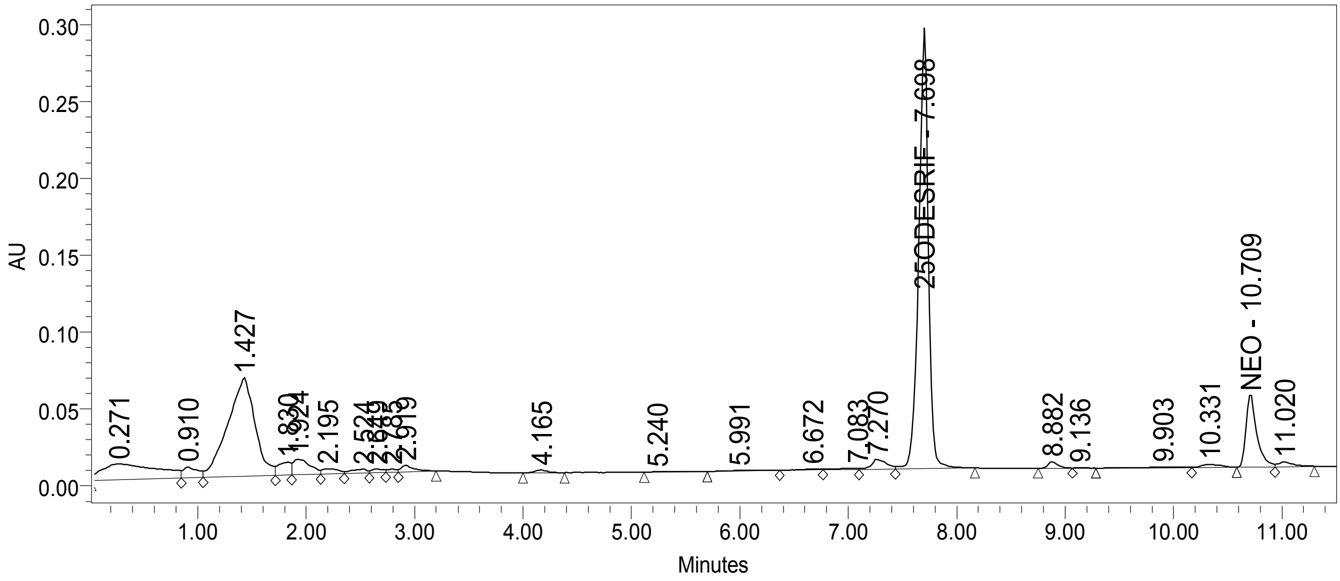

	Peak Name	RT	Area	% Area	Height
1		0.271	354814	8.58	10045
2		0.910	63822	1.54	6837
3		1.427	1117257	27.00	63699
4		1.830	66790	1.61	8178
5		1.924	109420	2.64	9965
6		2.195	33090	0.80	3365
7		2.524	25319	0.61	2550
8		2.649	15813	0.38	2189
9		2.785	11087	0.27	1765
10		2.919	32099	0.78	3947
11		4.165	14312	0.35	1861
12		5.240	3401	0.08	220

Reported by User: Saneesh (Saneesh) Project Name: HDI_Stellenbosch_2016

	Peak Name	RT	Area	% Area	Height
13		5.991	8192	0.20	338
14		6.672	10250	0.25	919
15		7.083	13790	0.33	850
16		7.270	62259	1.50	6381
17	25ODESRIF	7.698	1778351	42.98	281818
18	RIF	8.100			
19		8.882	24989	0.60	4118
20		9.136	2241	0.05	352
21		9.903	13763	0.33	558
22		10.331	24141	0.58	1984
23	NEO	10.709	323078	7.81	47187
24		11.020	29212	0.71	3033

Reported by User: Saneesh (Saneesh) Project Name: HDI_Stellenbosch_2016

SAMPLE INFORMATION

Sample Name:	RIF Inc Basil_MeOH BMRc22 45min1	Acquired By:	Saneesh
Sample Type:	Unknown	Date Acquired:	2017/02/08 03:47:24 AM
Vial:	23	Acq. Method Set:	HDI_RIF_25ODESRIF_NEO
Injection #:	1	Date Processed:	2017/02/08 06:54:17 AM
Injection Volume:	15.00 ul	Processing Method:	HDI_RIF_ W2996 254.0nm-1.2
Run Time:	11.5 Minutes	Channel Name:	W2996 254.0nm-1.2
Sample Set Name:	RIF_Basil MeOH Controls 7_1_17	Proc. Chnl. Descr.:	W2996 PDA 254.0 nm at 1.2

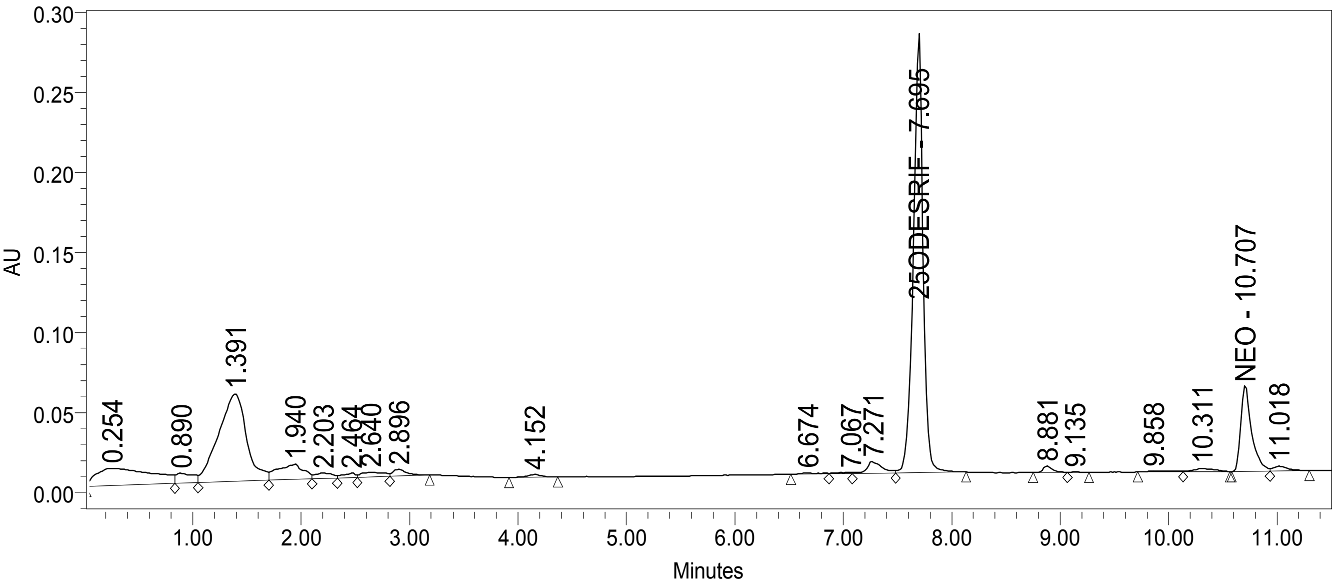

	Peak Name	RT	Area	% Area	Height
1		0.254	376189	9.39	10659
2		0.890	68395	1.71	6256
3		1.391	981324	24.49	54678
4		1.940	161163	4.02	9549
5		2.203	38702	0.97	3376
6		2.464	22916	0.57	2814
7		2.640	43385	1.08	3029
8		2.896	39107	0.98	4288
9		4.152	14738	0.37	1761
10		6.674	7826	0.20	716
11		7.067	6795	0.17	626
12		7.271	70951	1.77	7208

Reported by User: Saneesh (Saneesh) Project Name: HDI_Stellenbosch_2016

	Peak Name	RT	Area	% Area	Height
13	25ODESRIF	7.695	1715971	42.83	269383
14	RIF	8.100			
15		8.881	23239	0.58	3844
16		9.135	2170	0.05	392
17		9.858	7349	0.18	452
18		10.311	26673	0.67	2009
19	NEO	10.707	370739	9.25	53581
20		11.018	29030	0.72	3003

Reported by User: Saneesh (Saneesh)   Project Name: HDI_Stellenbosch_2016

SAMPLE INFORMATION

Sample Name:	RIF Inc Basil_MeOH BMRc23 45min2	Acquired By:	Saneesh
Sample Type:	Unknown	Date Acquired:	2017/02/08 04:01:58 AM
Vial:	24	Acq. Method Set:	HDI_RIF_25ODESRIF_NEO
Injection #:	1	Date Processed:	2017/02/08 06:54:17 AM
Injection Volume:	15.00 ul	Processing Method:	HDI_RIF_ W2996 254.0nm-1.2
Run Time:	11.5 Minutes	Channel Name:	W2996 254.0nm-1.2
Sample Set Name:	RIF_Basil MeOH Controls 7_1_17	Proc. Chnl. Descr.:	W2996 PDA 254.0 nm at 1.2

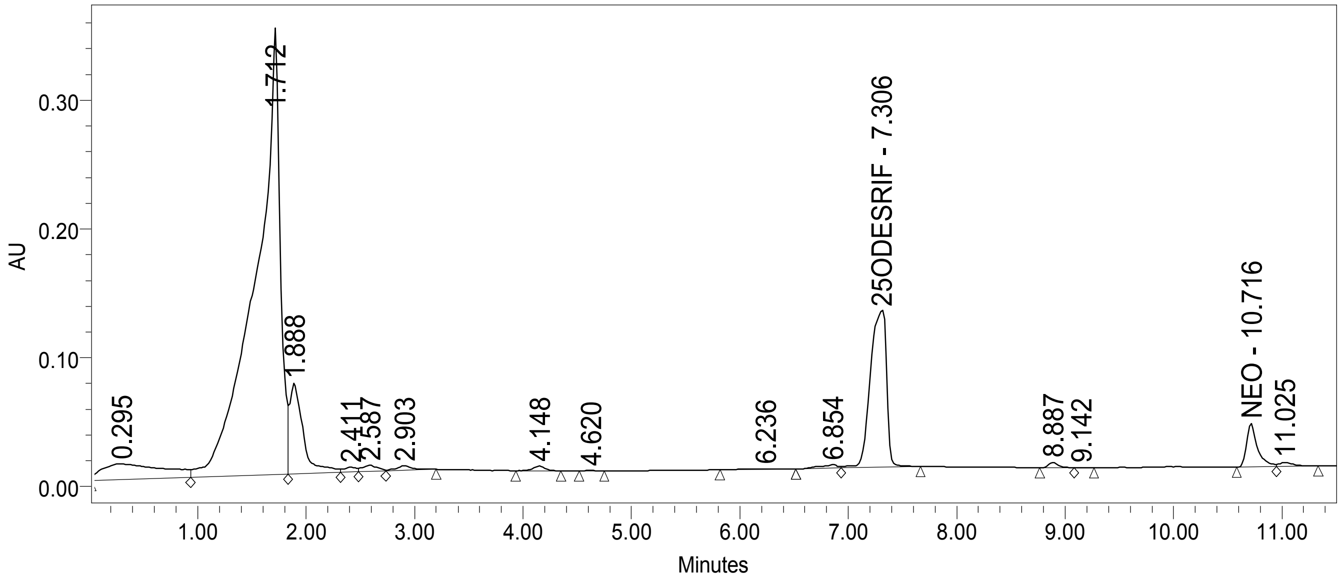

	Peak Name	RT	Area	% Area	Height
1		0.295	467465	5.69	11886
2		1.712	5386209	65.56	343904
3		1.888	621117	7.56	69957
4		2.411	30378	0.37	3712
5		2.587	46438	0.57	4669
6		2.903	37880	0.46	3594
7		4.148	28348	0.35	3753
8		4.620	2786	0.03	476
9		6.236	7647	0.09	271
10		6.854	30528	0.37	2654
11	25ODESRIF	7.306	1267506	15.43	124120
12	RIF	8.100			

Reported by User: Saneesh (Saneesh) Project Name: HDI_Stellenbosch_2016

	Peak Name	RT	Area	% Area	Height
13		8.887	25160	0.31	4057
14		9.142	1686	0.02	281
15	NEO	10.716	233621	2.84	33548
16		11.025	29081	0.35	3123

SAMPLE INFORMATION

Sample Name:	RIF Inc Basil_MeOH BMRc24 45min3	Acquired By:	Saneesh
Sample Type:	Unknown	Date Acquired:	2017/02/08 04:16:33 AM
Vial:	2	Acq. Method Set:	HDI_RIF_25ODESRIF_NEO
Injection #:	1	Date Processed:	2017/02/08 06:54:17 AM
Injection Volume:	15.00 ul	Processing Method:	HDI_RIF_ W2996 254.0nm-1.2
Run Time:	11.5 Minutes	Channel Name:	W2996 254.0nm-1.2
Sample Set Name:	RIF_Basil MeOH Controls 7_1_17	Proc. Chnl. Descr.:	W2996 PDA 254.0 nm at 1.2

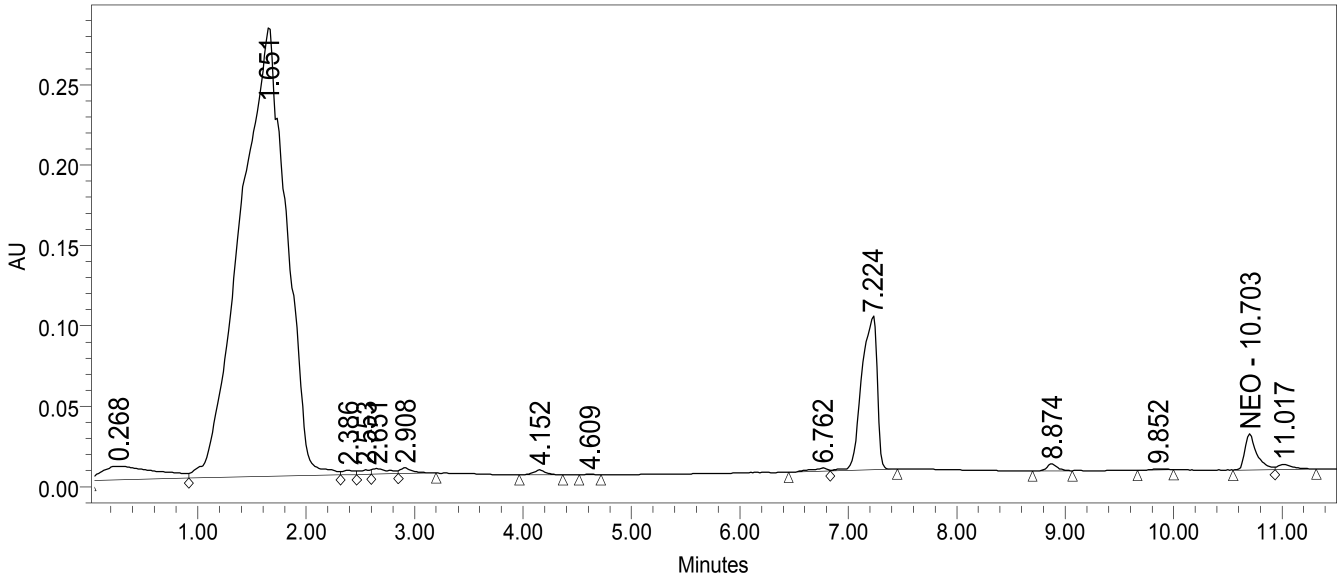

	Peak Name	RT	Area	% Area	Height
1		0.268	295849	3.02	8411
2		1.651	8159023	83.30	279127
3		2.386	21777	0.22	2863
4		2.553	19905	0.20	2836
5		2.651	37129	0.38	3532
6		2.908	28854	0.29	3603
7		4.152	23688	0.24	3091
8		4.609	2183	0.02	391
9		6.762	21299	0.22	2029
10		7.224	955276	9.75	97589
11	25ODESRIF	7.800			
12	RIF	8.100			

Reported by User: Saneesh (Saneesh)   Project Name: HDI_Stellenbosch_2016

	Peak Name	RT	Area	% Area	Height
13		8.874	27231	0.28	4248
14		9.852	6201	0.06	651
15	NEO	10.703	167559	1.71	22253
16		11.017	28718	0.29	2934

SAMPLE INFORMATION

Sample Name:	RIF Inc Basl_MeOH BMRc23	Acquired By:	Saneesh
Sample Type:	Unknown	Date Acquired:	2017/02/08 05:08:15 AM
Vial:	24	Acq. Method Set:	HDI_RIF_25ODESRIF_NEO
Injection #:	1	Date Processed:	2017/02/08 06:54:24 AM
Injection Volume:	15.00 ul	Processing Method:	HDI_RIF_ W2996 254.0nm-1.2
Run Time:	11.5 Minutes	Channel Name:	W2996 254.0nm-1.2
Sample Set Name:	RIF_Basil MeOH ControlR 8_1_17	Proc. Chnl. Descr.:	W2996 PDA 254.0 nm at 1.2

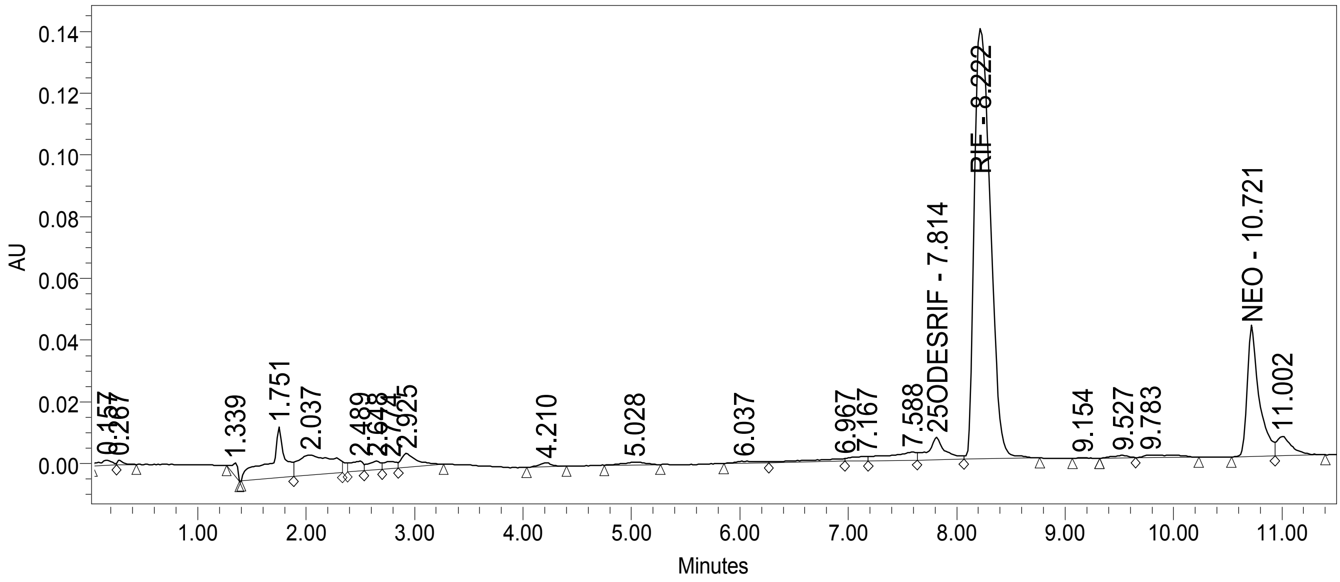

	Peak Name	RT	Area	% Area	Height
1		0.157	14450	0.57	1768
2		0.267	5952	0.24	1528
3		1.339	16166	0.64	4381
4		1.751	161404	6.42	15508
5		2.037	143852	5.72	6560
6		2.489	26680	1.06	3287
7		2.648	23520	0.93	2820
8		2.774	18558	0.74	2216
9		2.925	43801	1.74	4410
10		4.210	10535	0.42	1333
11		5.028	12715	0.51	888
12		6.037	8468	0.34	544

Reported by User: Saneesh (Saneesh) Project Name: HDI_Stellenbosch_2016

	Peak Name	RT	Area	% Area	Height
13		6.967	22081	0.88	876
14		7.167	16557	0.66	1433
15		7.588	50074	1.99	2539
16	25ODESRIF	7.814	77681	3.09	7253
17	RIF	8.222	1448127	57.56	139810
18		9.154	2104	0.08	328
19		9.527	9241	0.37	917
20		9.783	18734	0.74	940
21	NEO	10.721	322317	12.81	42327
22		11.002	62630	2.49	6258

SAMPLE INFORMATION

Sample Name:	RIF Inc Basl_MeOH BMRc24	Acquired By:	Saneesh
Sample Type:	Unknown	Date Acquired:	2017/02/08 05:22:51 AM
Vial:	2	Acq. Method Set:	HDI_RIF_25ODESRIF_NEO
Injection #:	1	Date Processed:	2017/02/08 06:54:24 AM
Injection Volume:	15.00 ul	Processing Method:	HDI_RIF_ W2996 254.0nm-1.2
Run Time:	11.5 Minutes	Channel Name:	W2996 254.0nm-1.2
Sample Set Name:	RIF_Basil MeOH ControlR 8_1_17	Proc. Chnl. Descr.:	W2996 PDA 254.0 nm at 1.2

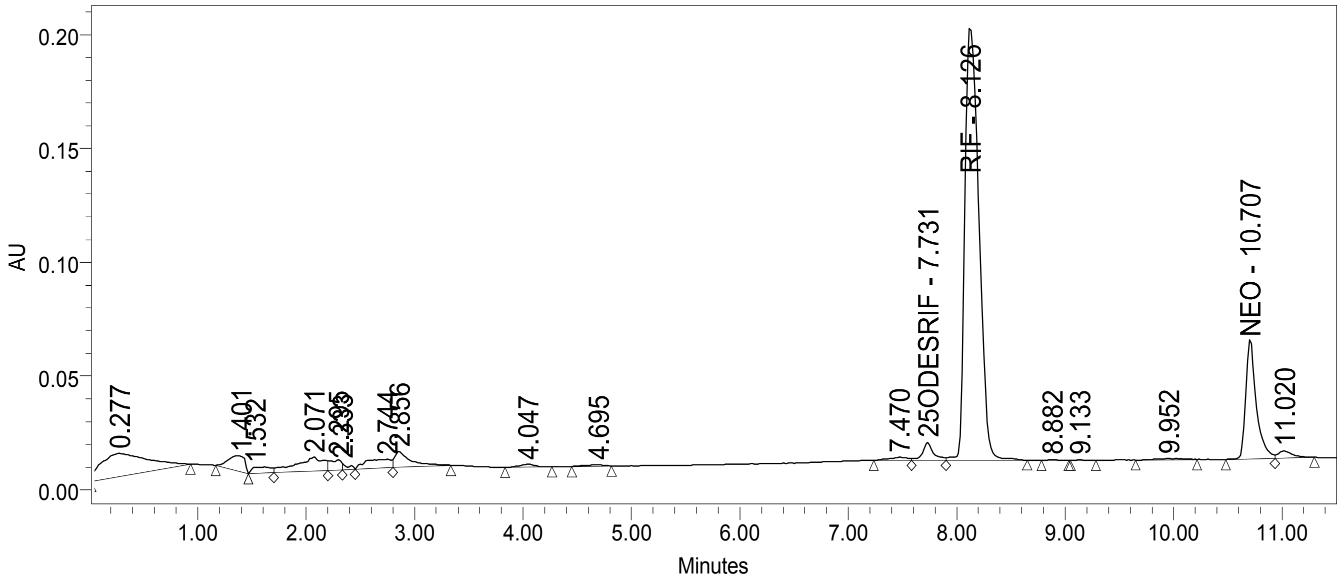

	Peak Name	RT	Area	% Area	Height
1		0.277	296349	10.21	10011
2		1.401	70890	2.44	7260
3		1.532	32070	1.10	2804
4		2.071	110023	3.79	6054
5		2.295	31621	1.09	4441
6		2.333	9428	0.32	2856
7		2.744	66644	2.30	3889
8		2.856	72042	2.48	6953
9		4.047	11591	0.40	1269
10		4.695	6840	0.24	598
11		7.470	16335	0.56	1264
12	25ODESRIF	7.731	54261	1.87	7847

Reported by User: Saneesh (Saneesh) Project Name: HDI_Stellenbosch_2016

	Peak Name	RT	Area	% Area	Height
13	RIF	8.126	1732278	59.66	191949
14		8.882	2480	0.09	422
15		9.133	1646	0.06	328
16		9.952	10094	0.35	510
17	NEO	10.707	349823	12.05	52265
18		11.020	29072	1.00	3117

Multi Sample Summary

Reported by User: Saneesh (Saneesh) Project Name: HDI_Stellenbosch_2016

SAMPLE INFORMATION

Sample Name:	RIF Inc Basil_EtOH BERp1 0min A	Acquired By:	Saneesh
Sample Type:	Unknown	Date Acquired:	2017/06/09 01:46:03 PM
Vial:	2	Acq. Method Set:	HDI_RIF_25ODESRIF_NEO
Injection #:	1	Date Processed:	2017/06/09 04:04:07 PM
Injection Volume:	15.00 ul	Processing Method:	HDI_RIF_ W2996 254.0nm-1.2
Run Time:	11.5 Minutes	Channel Name:	W2996 254.0nm-1.2
Sample Set Name:	RIF_Basil EOH AssySM Rp 9_6_17	Proc. Chnl. Descr.:	W2996 PDA 254.0 nm at 1.2

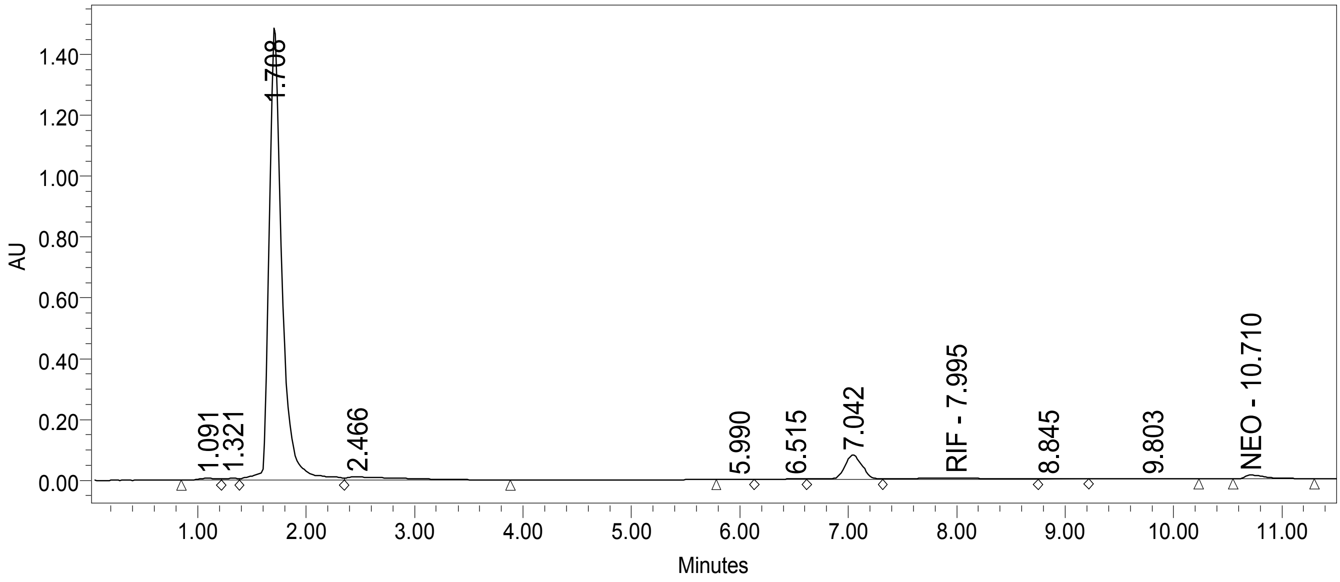

	Peak Name	RT	Area	% Area	Height
1		1.091	71158	0.50	6066
2		1.321	53841	0.38	7644
3		1.708	12226596	86.55	1487441
4		2.466	421321	2.98	11591
5		5.990	7673	0.05	568
6		6.515	27934	0.20	1726
7		7.042	918983	6.51	79644
8	25ODESRIF	7.800			
9	RIF	7.995	195502	1.38	4072
10		8.845	8791	0.06	595
11		9.803	19059	0.13	601
12	NEO	10.710	175451	1.24	13018

SAMPLE INFORMATION

Sample Name:	RIF Inc Basil_EtOH BERp2 2min A	Acquired By:	Saneesh
Sample Type:	Unknown	Date Acquired:	2017/06/09 02:01:00 PM
Vial:	3	Acq. Method Set:	HDI_RIF_25ODESRIF_NEO
Injection #:	1	Date Processed:	2017/06/09 04:04:07 PM
Injection Volume:	15.00 ul	Processing Method:	HDI_RIF_ W2996 254.0nm-1.2
Run Time:	11.5 Minutes	Channel Name:	W2996 254.0nm-1.2
Sample Set Name:	RIF_Basil EOH AssySM Rp 9_6_17	Proc. Chnl. Descr.:	W2996 PDA 254.0 nm at 1.2

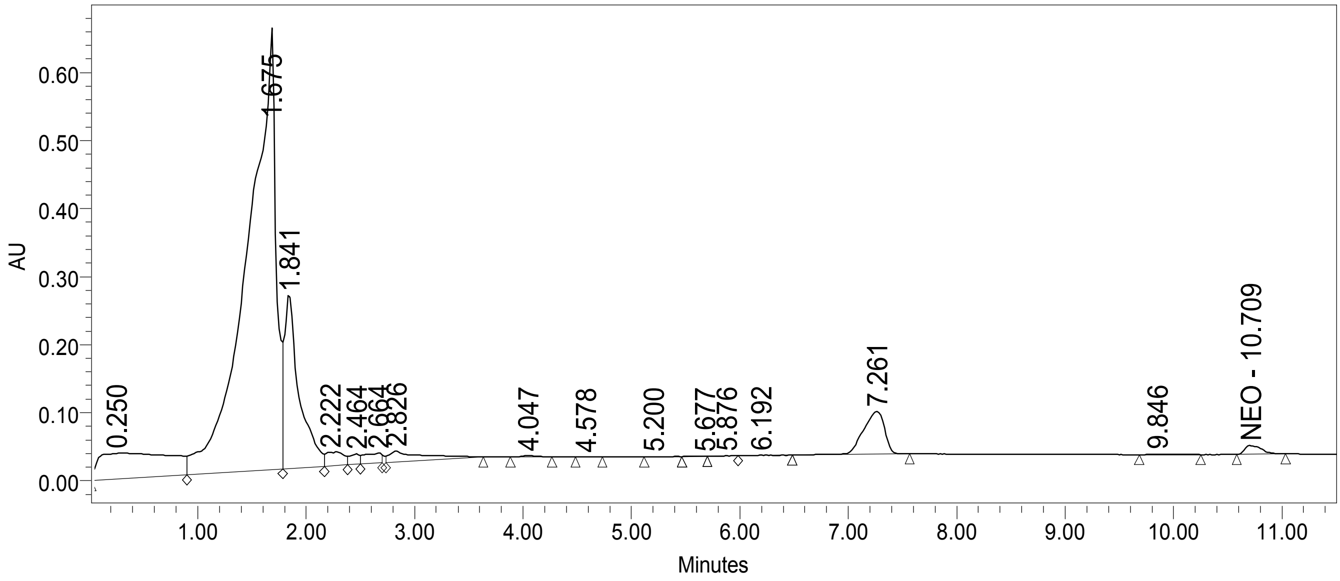

	Peak Name	RT	Area	% Area	Height
1		0.250	1701210	9.58	37332
2		1.675	11727028	66.06	637346
3		1.841	2450960	13.81	249734
4		2.222	232681	1.31	20012
5		2.464	96054	0.54	15076
6		2.664	164163	0.92	14644
7		2.826	340326	1.92	15657
8		4.047	16139	0.09	1867
9		4.578	1806	0.01	242
10		5.200	1868	0.01	166
11		5.677	675	0.00	53
12		5.876	2519	0.01	214

Reported by User: Saneesh (Saneesh) Project Name: HDI_Stellenbosch_2016

	Peak Name	RT	Area	% Area	Height
13		6.192	8213	0.05	543
14		7.261	865890	4.88	63058
15	25ODESRIF	7.800			
16	RIF	8.100			
17		9.846	11406	0.06	485
18	NEO	10.709	130140	0.73	12636

Reported by User: Saneesh (Saneesh)   Project Name: HDI_Stellenbosch_2016

SAMPLE INFORMATION

Sample Name:	RIF Inc Basil_EtOH BERp3 15min A	Acquired By:	Saneesh
Sample Type:	Unknown	Date Acquired:	2017/06/09 02:16:05 PM
Vial:	4	Acq. Method Set:	HDI_RIF_25ODESRIF_NEO
Injection #:	1	Date Processed:	2017/06/09 04:04:07 PM
Injection Volume:	15.00 ul	Processing Method:	HDI_RIF_ W2996 254.0nm-1.2
Run Time:	11.5 Minutes	Channel Name:	W2996 254.0nm-1.2
Sample Set Name:	RIF_Basil EOH AssySM Rp 9_6_17	Proc. Chnl. Descr.:	W2996 PDA 254.0 nm at 1.2

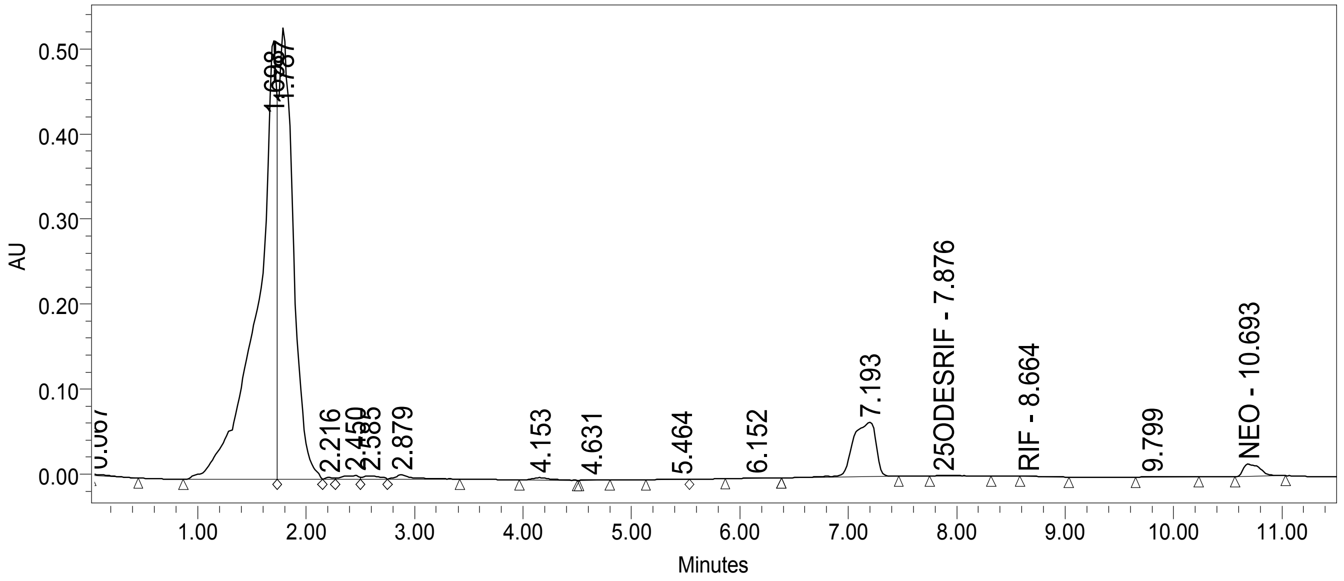

	Peak Name	RT	Area	% Area	Height
1		0.067	16364	0.13	1398
2		1.698	6295394	49.53	514158
3		1.787	5073073	39.92	526057
4		2.216	11863	0.09	2181
5		2.450	47898	0.38	4467
6		2.585	44161	0.35	3991
7		2.879	57527	0.45	4960
8		4.153	33050	0.26	3104
9		4.631	3869	0.03	470
10		5.464	1237	0.01	81
11		6.152	7900	0.06	504
12		7.193	935642	7.36	63763

	Peak Name	RT	Area	% Area	Height
13	25ODESRIF	7.876	11472	0.09	550
14	RIF	8.664	5076	0.04	473
15		9.799	7791	0.06	350
16	NEO	10.693	157268	1.24	14741

SAMPLE INFORMATION

Sample Name:	RIF Inc Basil_EtOH BERp4 20min A	Acquired By:	Saneesh
Sample Type:	Unknown	Date Acquired:	2017/06/09 02:30:49 PM
Vial:	5	Acq. Method Set:	HDI_RIF_25ODESRIF_NEO
Injection #:	1	Date Processed:	2017/06/09 04:04:07 PM
Injection Volume:	15.00 ul	Processing Method:	HDI_RIF_ W2996 254.0nm-1.2
Run Time:	11.5 Minutes	Channel Name:	W2996 254.0nm-1.2
Sample Set Name:	RIF_Basil EOH AssySM Rp 9_6_17	Proc. Chnl. Descr.:	W2996 PDA 254.0 nm at 1.2

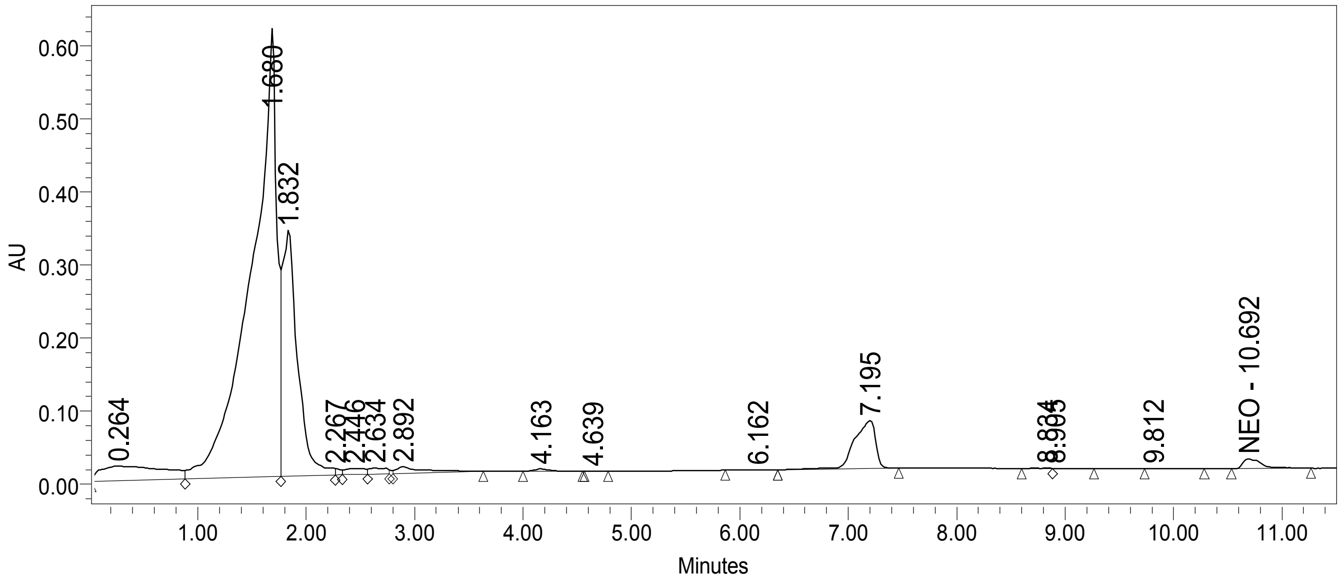

	Peak Name	RT	Area	% Area	Height
1		0.264	795881	5.15	19595
2		1.680	9875027	63.85	607118
3		1.832	3329921	21.53	332647
4		2.267	30795	0.20	8714
5		2.446	105410	0.68	7958
6		2.634	86225	0.56	7922
7		2.892	144769	0.94	8650
8		4.163	32760	0.21	3383
9		4.639	3559	0.02	590
10		6.162	6547	0.04	536
11		7.195	878423	5.68	65507
12	25ODESRIF	7.800			

Reported by User: Saneesh (Saneesh) Project Name: HDI_Stellenbosch_2016

	Peak Name	RT	Area	% Area	Height
13	RIF	8.100			
14		8.834	6225	0.04	551
15		8.905	6233	0.04	458
16		9.812	5267	0.03	269
17	NEO	10.692	158182	1.02	13281

SAMPLE INFORMATION

Sample Name:	RIF Inc Basil_EtOH BERp5 20min B	Acquired By:	Saneesh
Sample Type:	Unknown	Date Acquired:	2017/06/09 02:45:34 PM
Vial:	6	Acq. Method Set:	HDI_RIF_25ODESRIF_NEO
Injection #:	1	Date Processed:	2017/06/09 04:04:07 PM
Injection Volume:	15.00 ul	Processing Method:	HDI_RIF_ W2996 254.0nm-1.2
Run Time:	11.5 Minutes	Channel Name:	W2996 254.0nm-1.2
Sample Set Name:	RIF_Basil EOH AssySM Rp 9_6_17	Proc. Chnl. Descr.:	W2996 PDA 254.0 nm at 1.2

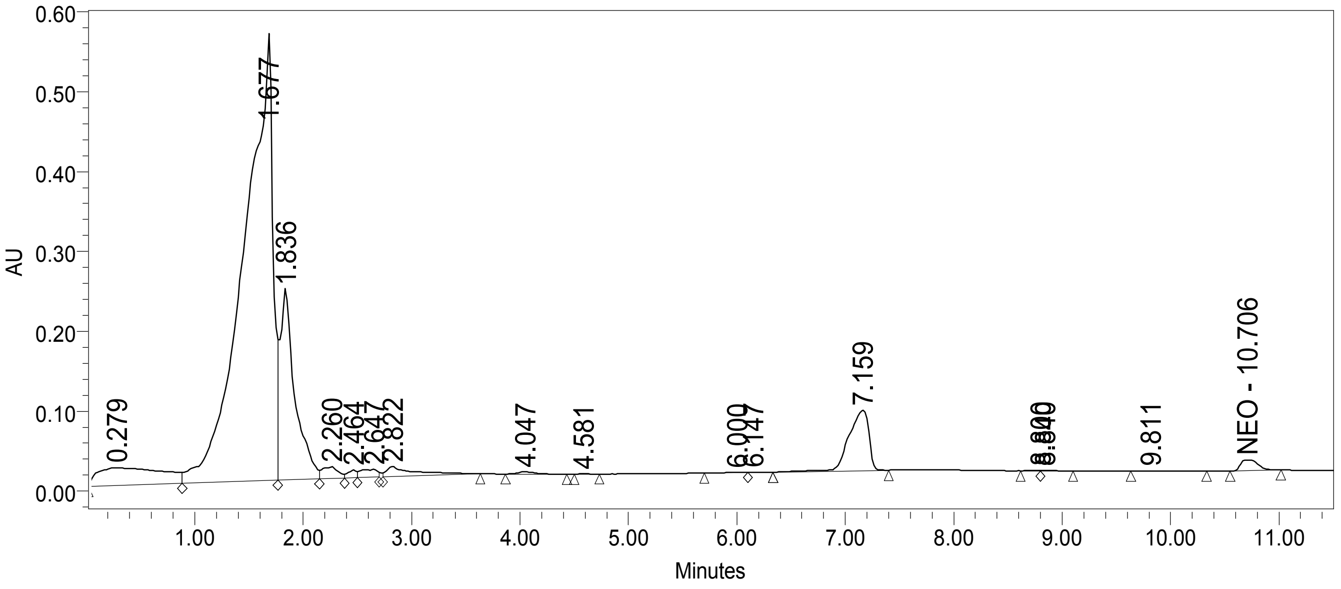

	Peak Name	RT	Area	% Area	Height
1		0.279	905434	5.95	21996
2		1.677	10281527	67.60	553156
3		1.836	2309779	15.19	233095
4		2.260	150556	0.99	14476
5		2.464	53337	0.35	9294
6		2.647	105148	0.69	9626
7		2.822	224670	1.48	12499
8		4.047	32211	0.21	3000
9		4.581	3256	0.02	484
10		6.000	5304	0.03	392
11		6.147	2604	0.02	441
12		7.159	970875	6.38	76369

Reported by User: Saneesh (Saneesh) Project Name: HDI_Stellenbosch_2016

	Peak Name	RT	Area	% Area	Height
13	25ODESRIF	7.800			
14	RIF	8.100			
15		8.800	3445	0.02	473
16		8.840	5604	0.04	651
17		9.811	9121	0.06	344
18	NEO	10.706	146778	0.97	13633

Reported by User: Saneesh (Saneesh)   Project Name: HDI_Stellenbosch_2016

SAMPLE INFORMATION

Sample Name:	RIF Inc Basil_EtOH BERp6 30min A	Acquired By:	Saneesh
Sample Type:	Unknown	Date Acquired:	2017/06/09 03:00:10 PM
Vial:	7	Acq. Method Set:	HDI_RIF_25ODESRIF_NEO
Injection #:	1	Date Processed:	2017/06/09 04:04:07 PM
Injection Volume:	15.00 ul	Processing Method:	HDI_RIF_ W2996 254.0nm-1.2
Run Time:	11.5 Minutes	Channel Name:	W2996 254.0nm-1.2
Sample Set Name:	RIF_Basil EOH AssySM Rp 9_6_17	Proc. Chnl. Descr.:	W2996 PDA 254.0 nm at 1.2

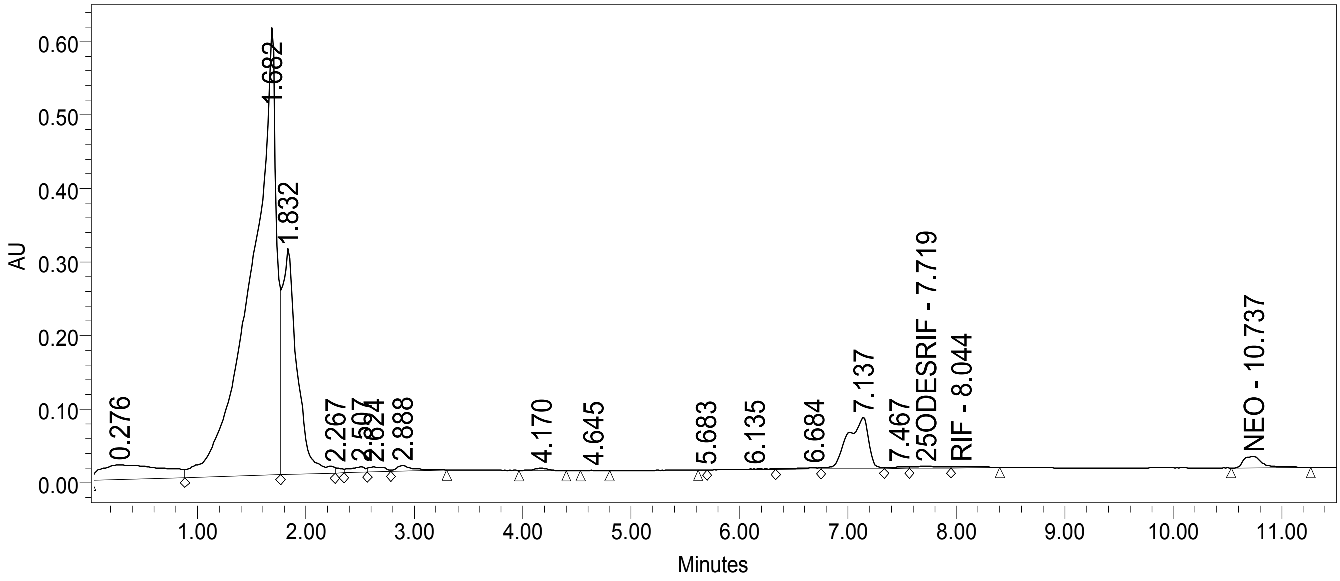

	Peak Name	RT	Area	% Area	Height
1		0.276	776900	5.22	19309
2		1.682	9619259	64.62	602973
3		1.832	2948422	19.81	300816
4		2.267	30852	0.21	7882
5		2.507	77252	0.52	7363
6		2.624	68845	0.46	6693
7		2.888	84323	0.57	7492
8		4.170	31111	0.21	3572
9		4.645	4509	0.03	649
10		5.683	450	0.00	134
11		6.135	14253	0.10	568
12		6.684	32852	0.22	2059

Reported by User: Saneesh (Saneesh) Project Name: HDI_Stellenbosch_2016

	Peak Name	RT	Area	% Area	Height
13		7.137	928153	6.24	68975
14		7.467	20409	0.14	1552
15	25ODESRIF	7.719	40551	0.27	2169
16	RIF	8.044	23513	0.16	1420
17	NEO	10.737	183382	1.23	15291

Multi Sample Summary

Reported by User: Saneesh (Saneesh)   Project Name: HDI_Stellenbosch_2016

SAMPLE INFORMATION

Sample Name:	RIF Inc Basil_EtOH BERp7 30min B	Acquired By:	Saneesh
Sample Type:	Unknown	Date Acquired:	2017/06/09 03:14:46 PM
Vial:	8	Acq. Method Set:	HDI_RIF_25ODESRIF_NEO
Injection #:	1	Date Processed:	2017/06/09 04:04:07 PM
Injection Volume:	15.00 ul	Processing Method:	HDI_RIF_ W2996 254.0nm-1.2
Run Time:	11.5 Minutes	Channel Name:	W2996 254.0nm-1.2
Sample Set Name:	RIF_Basil EOH AssySM Rp 9_6_17	Proc. Chnl. Descr.:	W2996 PDA 254.0 nm at 1.2

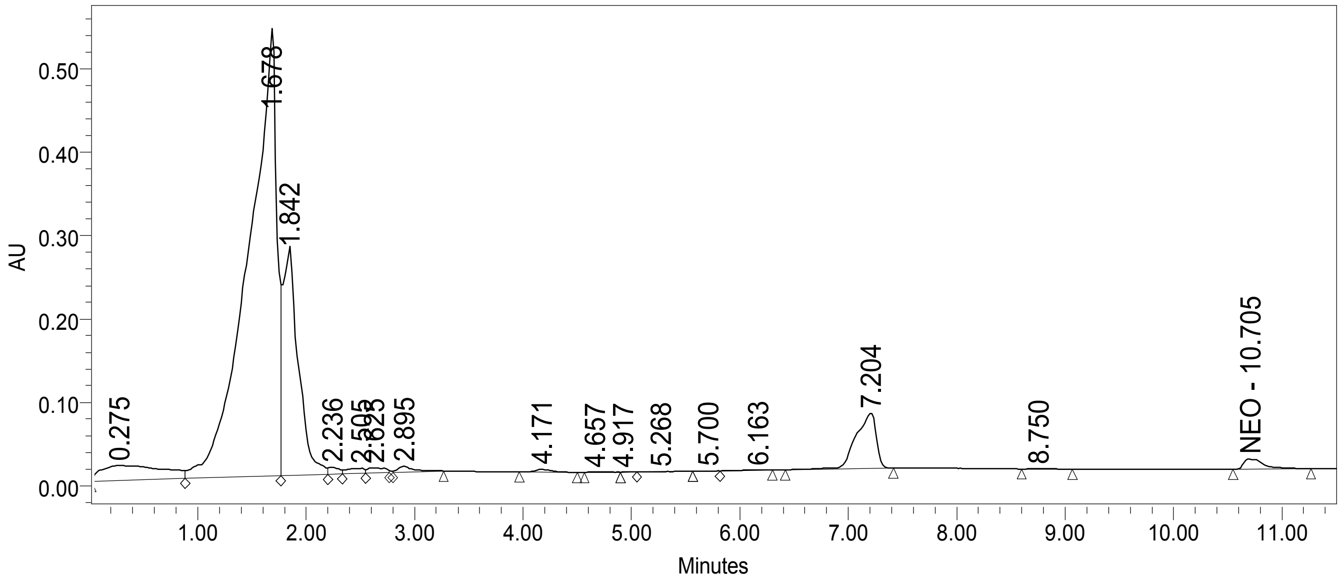

	Peak Name	RT	Area	% Area	Height
1		0.275	703737	4.85	17781
2		1.678	9670056	66.70	538539
3		1.842	2774926	19.14	270648
4		2.236	56973	0.39	8493
5		2.505	71315	0.49	6030
6		2.625	69556	0.48	6278
7		2.895	69839	0.48	6919
8		4.171	34639	0.24	3472
9		4.657	4550	0.03	626
10		4.917	532	0.00	86
11		5.268	2884	0.02	170
12		5.700	1395	0.01	181

	Peak Name	RT	Area	% Area	Height
13		6.163	7247	0.05	493
14		7.204	871439	6.01	65991
15	25ODESRIF	7.800			
16	RIF	8.100			
17		8.750	6132	0.04	384
18	NEO	10.705	153067	1.06	12121

SAMPLE INFORMATION

Sample Name:	RIF Inc Basil_EtOH BERp8 45min A	Acquired By:	Saneesh
Sample Type:	Unknown	Date Acquired:	2017/06/09 03:29:22 PM
Vial:	9	Acq. Method Set:	HDI_RIF_25ODESRIF_NEO
Injection #:	1	Date Processed:	2017/06/09 04:04:07 PM
Injection Volume:	15.00 ul	Processing Method:	HDI_RIF_ W2996 254.0nm-1.2
Run Time:	11.5 Minutes	Channel Name:	W2996 254.0nm-1.2
Sample Set Name:	RIF_Basil EOH AssySM Rp 9_6_17	Proc. Chnl. Descr.:	W2996 PDA 254.0 nm at 1.2

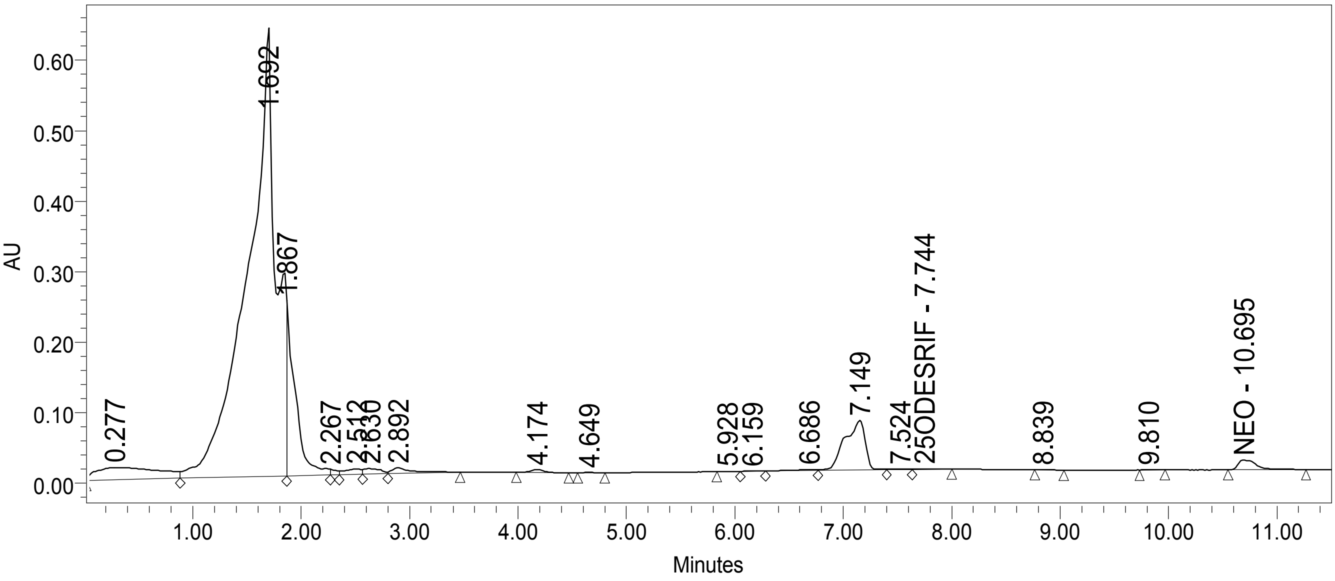

	Peak Name	RT	Area	% Area	Height
1		0.277	687504	4.57	17378
2		1.692	11508205	76.51	628430
3		1.867	1350917	8.98	248287
4		2.267	32343	0.22	7905
5		2.512	83502	0.56	7967
6		2.630	84435	0.56	7958
7		2.892	107979	0.72	8048
8		4.174	37042	0.25	4196
9		4.649	4466	0.03	662
10		5.928	1733	0.01	243
11		6.159	2297	0.02	310
12		6.686	22911	0.15	1605

Reported by User: Saneesh (Saneesh) Project Name: HDI_Stellenbosch_2016

	Peak Name	RT	Area	% Area	Height
13		7.149	923220	6.14	70526
14		7.524	9398	0.06	763
15	25ODESRIF	7.744	9593	0.06	779
16	RIF	8.100			
17		8.839	3307	0.02	391
18		9.810	2375	0.02	330
19	NEO	10.695	170658	1.13	13981

SAMPLE INFORMATION

Sample Name:	RIF Inc Basil_EtOH BERp9 45min B	Acquired By:	Saneesh
Sample Type:	Unknown	Date Acquired:	2017/06/09 03:43:58 PM
Vial:	10	Acq. Method Set:	HDI_RIF_25ODESRIF_NEO
Injection #:	1	Date Processed:	2017/06/09 04:04:07 PM
Injection Volume:	15.00 ul	Processing Method:	HDI_RIF_ W2996 254.0nm-1.2
Run Time:	11.5 Minutes	Channel Name:	W2996 254.0nm-1.2
Sample Set Name:	RIF_Basil EOH AssySM Rp 9_6_17	Proc. Chnl. Descr.:	W2996 PDA 254.0 nm at 1.2

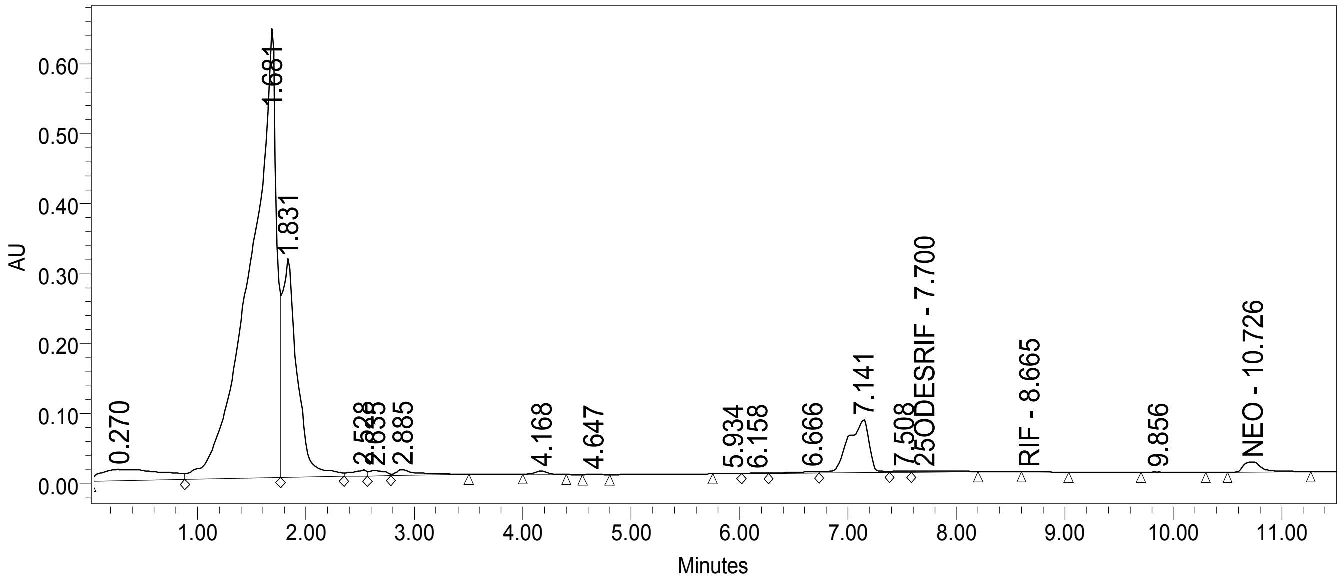

	Peak Name	RT	Area	% Area	Height
1		0.270	628964	3.96	15937
2		1.681	10629825	66.88	639069
3		1.831	3055122	19.22	307853
4		2.528	84702	0.53	8389
5		2.635	78383	0.49	7583
6		2.885	110880	0.70	7903
7		4.168	36564	0.23	4475
8		4.647	5416	0.03	814
9		5.934	2770	0.02	251
10		6.158	4573	0.03	413
11		6.666	29181	0.18	1927
12		7.141	998186	6.28	75947

Reported by User: Saneesh (Saneesh) Project Name: HDI_Stellenbosch_2016

	Peak Name	RT	Area	% Area	Height
13		7.508	14131	0.09	1237
14	25ODESRIF	7.700	28468	0.18	1281
15	RIF	8.665	5538	0.03	326
16		9.856	7953	0.05	417
17	NEO	10.726	174404	1.10	14272

Multi Sample Summary

Reported by User: Saneesh (Saneesh)   Project Name: HDI_Stellenbosch_2016

SAMPLE INFORMATION

Sample Name:	RIF Inc Basil_EtOH C1 Rep 0min A	Acquired By:	Saneesh
Sample Type:	Unknown	Date Acquired:	2017/06/12 08:56:46 AM
Vial:	21	Acq. Method Set:	HDI_RIF_25ODESRIF_NEO
Injection #:	1	Date Processed:	2017/06/13 04:15:46 PM
Injection Volume:	15.00 ul	Processing Method:	HDI_RIF_ W2996 254.0nm-1.2
Run Time:	11.5 Minutes	Channel Name:	W2996 254.0nm-1.2
Sample Set Name:	RIF_Basil EOH Cntrls Rp12_6_17	Proc. Chnl. Descr.:	W2996 PDA 254.0 nm at 1.2

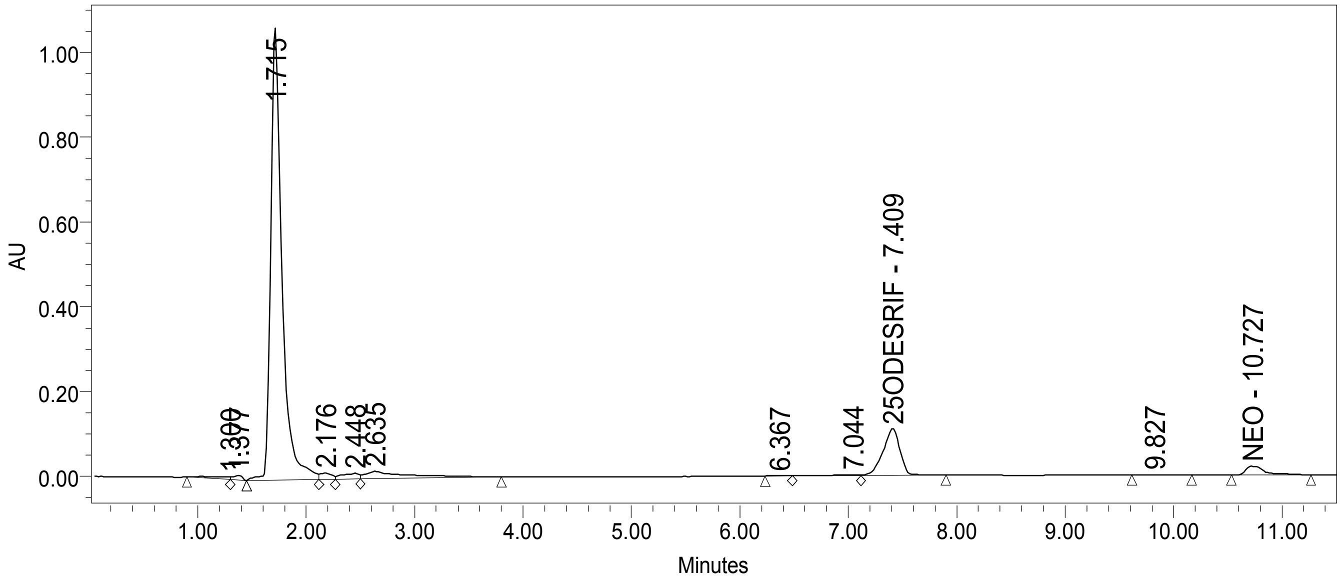

	Peak Name	RT	Area	% Area	Height
1		1.300	84120	0.84	5998
2		1.377	61006	0.61	10167
3		1.715	7714340	76.73	1066022
4		2.176	112309	1.12	15237
5		2.448	145094	1.44	13233
6		2.635	474805	4.72	17403
7		6.367	1918	0.02	219
8		7.044	25422	0.25	1713
9	25ODESRIF	7.409	1171669	11.65	110008
10	RIF	8.100			
11		9.827	11729	0.12	638
12	NEO	10.727	251235	2.50	20889

Reported by User: Saneesh (Saneesh) Project Name: HDI_Stellenbosch_2016

SAMPLE INFORMATION

Sample Name:	RIF Inc Basil_EtOH C2 Rep 0min B	Acquired By:	Saneesh
Sample Type:	Unknown	Date Acquired:	2017/06/12 09:11:32 AM
Vial:	22	Acq. Method Set:	HDI_RIF_25ODESRIF_NEO
Injection #:	1	Date Processed:	2017/06/13 04:15:46 PM
Injection Volume:	15.00 ul	Processing Method:	HDI_RIF_ W2996 254.0nm-1.2
Run Time:	11.5 Minutes	Channel Name:	W2996 254.0nm-1.2
Sample Set Name:	RIF_Basil EOH Cntrls Rp12_6_17	Proc. Chnl. Descr.:	W2996 PDA 254.0 nm at 1.2

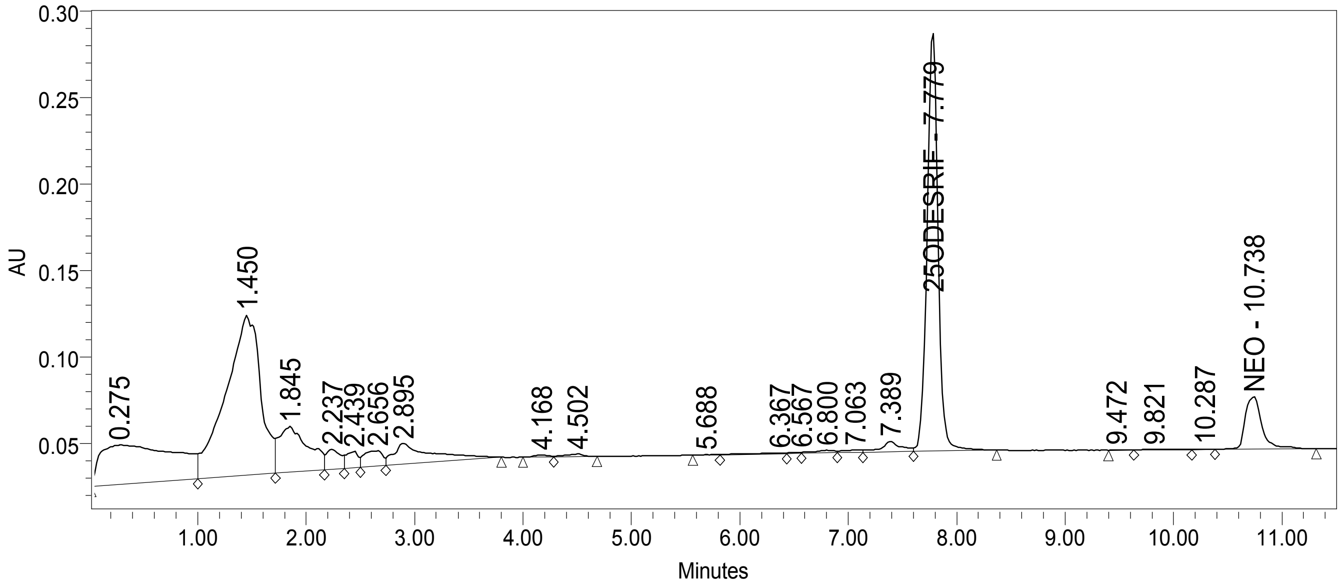

	Peak Name	RT	Area	% Area	Height
1		0.275	1066411	16.66	22634
2		1.450	2068734	32.32	91870
3		1.845	489403	7.64	26208
4		2.237	104821	1.64	11494
5		2.439	71718	1.12	9278
6		2.656	106018	1.66	8869
7		2.895	285996	4.47	12061
8		4.168	11497	0.18	1278
9		4.502	19156	0.30	1636
10		5.688	2362	0.04	210
11		6.367	11644	0.18	434
12		6.567	3754	0.06	557

Reported by User: Saneesh (Saneesh) Project Name: HDI_Stellenbosch_2016

	Peak Name	RT	Area	% Area	Height
13		6.800	20250	0.32	1547
14		7.063	17243	0.27	1385
15		7.389	76363	1.19	6023
16	25ODESRIF	7.779	1709107	26.70	241387
17	RIF	8.100			
18		9.472	2230	0.03	245
19		9.821	8914	0.14	396
20		10.287	2902	0.05	295
21	NEO	10.738	323195	5.05	30525

SAMPLE INFORMATION

Sample Name:	RIF Inc Basil_EtOH C3 Rep 45minA	Acquired By:	Saneesh
Sample Type:	Unknown	Date Acquired:	2017/06/12 09:26:17 AM
Vial:	23	Acq. Method Set:	HDI_RIF_25ODESRIF_NEO
Injection #:	1	Date Processed:	2017/06/13 04:15:46 PM
Injection Volume:	15.00 ul	Processing Method:	HDI_RIF_ W2996 254.0nm-1.2
Run Time:	11.5 Minutes	Channel Name:	W2996 254.0nm-1.2
Sample Set Name:	RIF_Basil EOH Cntrls Rp12_6_17	Proc. Chnl. Descr.:	W2996 PDA 254.0 nm at 1.2

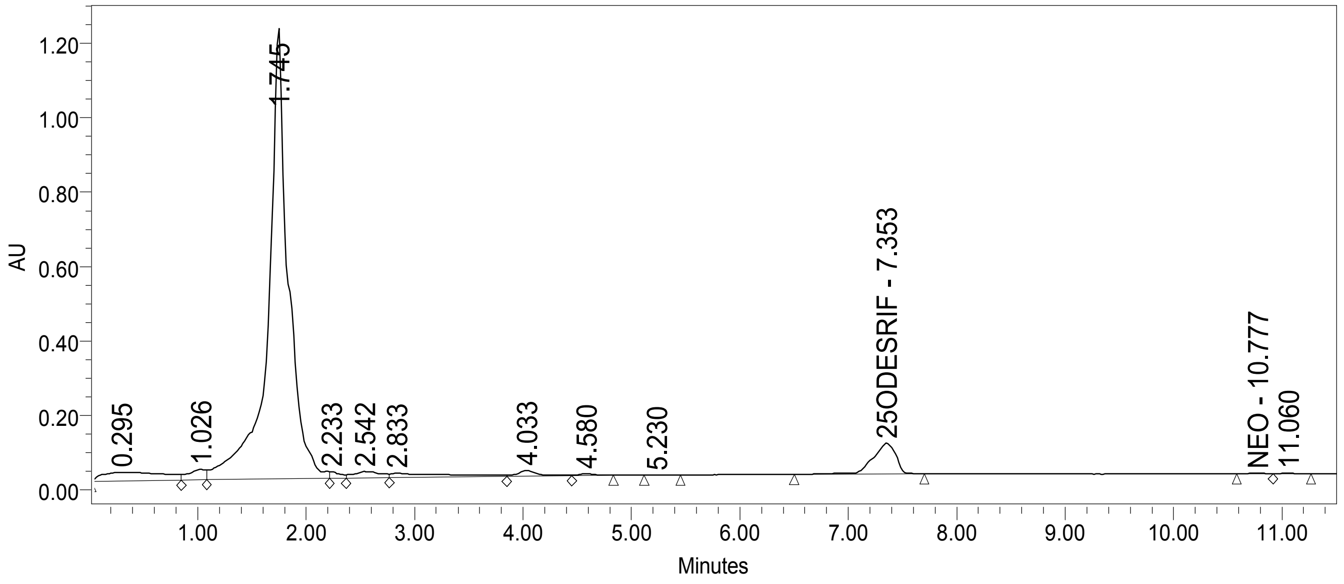

	Peak Name	RT	Area	% Area	Height
1		0.295	920067	4.74	22985
2		1.026	311130	1.60	28217
3		1.745	15815873	81.55	1198756
4		2.233	119991	0.62	18066
5		2.542	317700	1.64	17951
6		2.833	433501	2.24	12675
7		4.033	196760	1.01	15523
8		4.580	48066	0.25	5707
9		5.230	1741	0.01	179
10	25ODESRIF	7.353	1197358	6.17	82229
11	RIF	8.100			
12	NEO	10.777	18255	0.09	1565

Reported by User: Saneesh (Saneesh) Project Name: HDI_Stellenbosch_2016

	Peak Name	RT	Area	% Area	Height
13		11.060	13434	0.07	1047

Reported by User: Saneesh (Saneesh)   Project Name: HDI_Stellenbosch_2016

SAMPLE INFORMATION

Sample Name:	RIF Inc Basil_EtOH C4 Rep 45minB	Acquired By:	Saneesh
Sample Type:	Unknown	Date Acquired:	2017/06/12 09:41:02 AM
Vial:	24	Acq. Method Set:	HDI_RIF_25ODESRIF_NEO
Injection #:	1	Date Processed:	2017/06/13 04:15:46 PM
Injection Volume:	15.00 ul	Processing Method:	HDI_RIF_ W2996 254.0nm-1.2
Run Time:	11.5 Minutes	Channel Name:	W2996 254.0nm-1.2
Sample Set Name:	RIF_Basil EOH Cntrls Rp12_6_17	Proc. Chnl. Descr.:	W2996 PDA 254.0 nm at 1.2

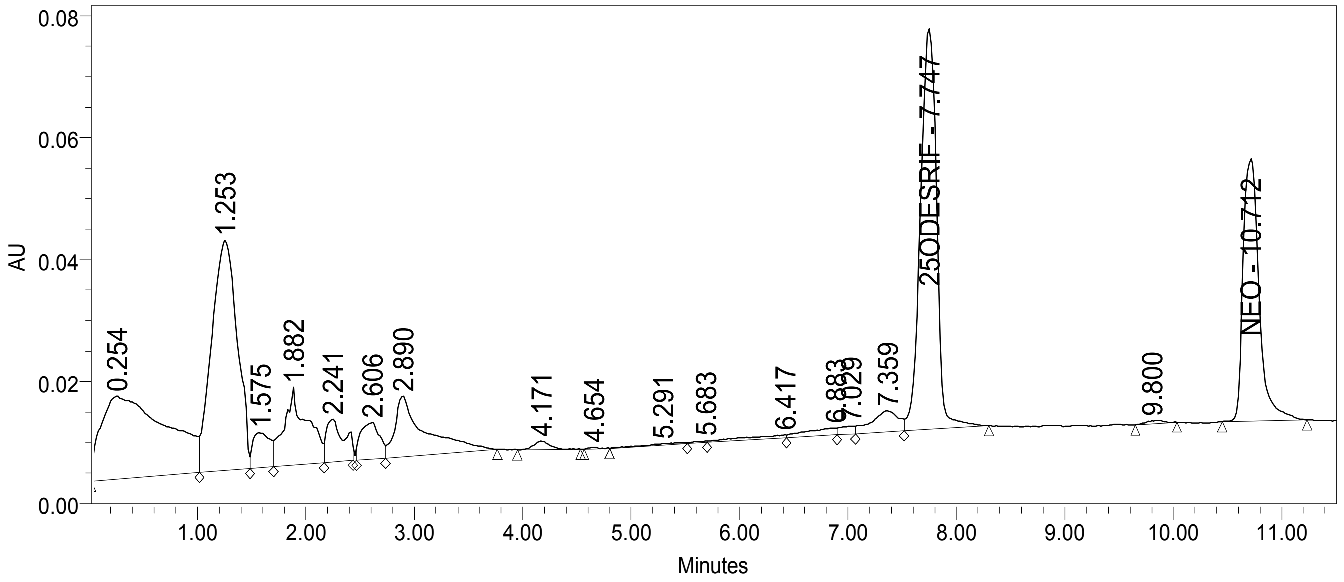

	Peak Name	RT	Area	% Area	Height
1		0.254	562983	18.45	13577
2		1.253	618386	20.27	37716
3		1.575	63720	2.09	5733
4		1.882	189643	6.22	11784
5		2.241	78392	2.57	7064
6		2.606	72370	2.37	6044
7		2.890	191706	6.28	9904
8		4.171	15187	0.50	1421
9		4.654	1758	0.06	272
10		5.291	6568	0.22	277
11		5.683	2513	0.08	324
12		6.417	17938	0.59	527

Reported by User: Saneesh (Saneesh) Project Name: HDI_Stellenbosch_2016

	Peak Name	RT	Area	% Area	Height
13		6.883	24612	0.81	1142
14		7.029	12359	0.41	1292
15		7.359	60169	1.97	3415
16	25ODESRIF	7.747	688352	22.56	65719
17	RIF	8.100			
18		9.800	5403	0.18	498
19	NEO	10.712	438521	14.37	43121

Reported by User: Saneesh (Saneesh)   Project Name: HDI_Stellenbosch_2016

SAMPLE INFORMATION

Sample Name:	RIF Inc Basil_MeOH EMRp1 20min A	Acquired By:	Saneesh
Sample Type:	Unknown	Date Acquired:	2017/06/12 02:15:07 PM
Vial:	11	Acq. Method Set:	HDI_RIF_25ODESRIF_NEO
Injection #:	1	Date Processed:	2017/06/13 04:17:50 PM
Injection Volume:	15.00 ul	Processing Method:	HDI_RIF_ W2996 254.0nm-1.2
Run Time:	11.5 Minutes	Channel Name:	W2996 254.0nm-1.2
Sample Set Name:	RIF_BasL MOH Cntrls Rp 12_6_17	Proc. Chnl. Descr.:	W2996 PDA 254.0 nm at 1.2

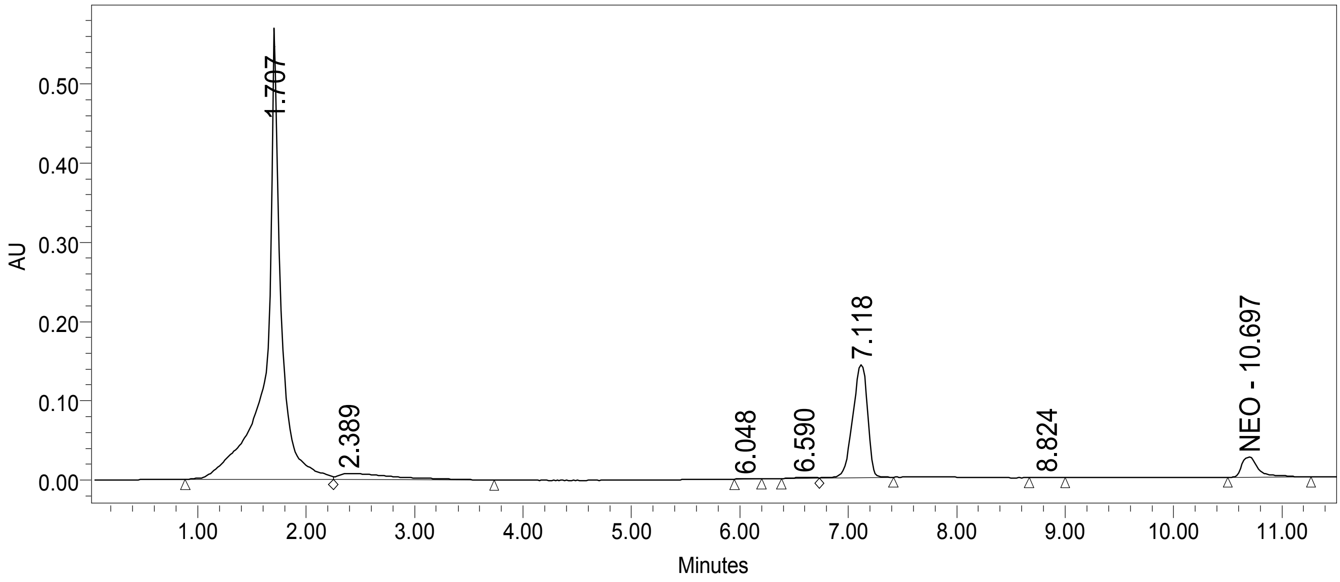

	Peak Name	RT	Area	% Area	Height
1		1.707	5371044	73.23	539133
2		2.389	293154	4.00	8228
3		6.048	1685	0.02	206
4		6.590	19830	0.27	1558
5		7.118	1369710	18.68	142890
6	25ODESRIF	7.800			
7	RIF	8.100			
8		8.824	5755	0.08	651
9	NEO	10.697	273230	3.73	25330

Reported by User: Saneesh (Saneesh)　　Project Name: HDI_Stellenbosch_2016

SAMPLE INFORMATION

Sample Name:	RIF Inc Basil_MeOH EMRp2 20min B	Acquired By:	Saneesh
Sample Type:	Unknown	Date Acquired:	2017/06/12 02:29:51 PM
Vial:	12	Acq. Method Set:	HDI_RIF_25ODESRIF_NEO
Injection #:	1	Date Processed:	2017/06/13 04:17:50 PM
Injection Volume:	15.00 ul	Processing Method:	HDI_RIF_ W2996 254.0nm-1.2
Run Time:	11.5 Minutes	Channel Name:	W2996 254.0nm-1.2
Sample Set Name:	RIF_BasL MOH Cntrls Rp 12_6_17	Proc. Chnl. Descr.:	W2996 PDA 254.0 nm at 1.2

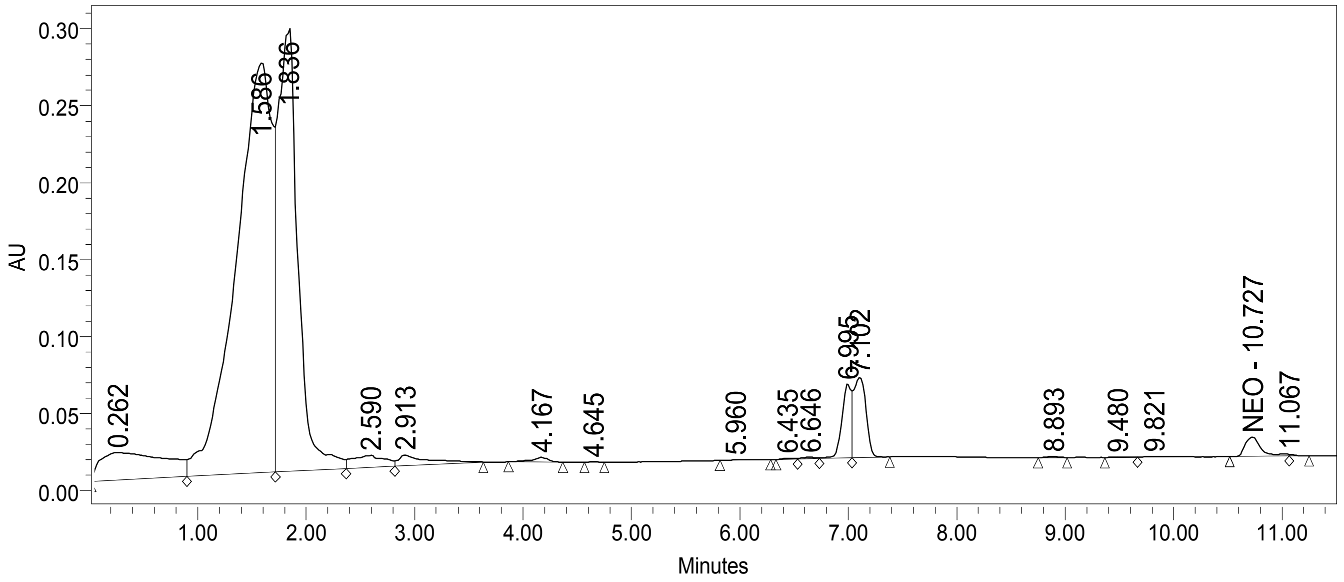

	Peak Name	RT	Area	% Area	Height
1		0.262	734965	6.21	17764
2		1.586	6189263	52.29	265963
3		1.836	3704210	31.29	291921
4		2.590	164585	1.39	7983
5		2.913	130720	1.10	6974
6		4.167	34127	0.29	3123
7		4.645	2334	0.02	403
8		5.960	4662	0.04	338
9		6.435	5302	0.04	691
10		6.646	8888	0.08	1013
11		6.995	285788	2.41	48468
12		7.102	420276	3.55	51920

	Peak Name	RT	Area	% Area	Height
13	25ODESRIF	7.800			
14	RIF	8.100			
15		8.893	5164	0.04	643
16		9.480	1915	0.02	196
17		9.821	11375	0.10	485
18	NEO	10.727	129104	1.09	12634
19		11.067	4674	0.04	1154

Reported by User: Saneesh (Saneesh) Project Name: HDI_Stellenbosch_2016

SAMPLE INFORMATION

Sample Name:	RIF Inc Basil_MeOH EMRp3 20min C	Acquired By:	Saneesh
Sample Type:	Unknown	Date Acquired:	2017/06/12 02:44:36 PM
Vial:	13	Acq. Method Set:	HDI_RIF_25ODESRIF_NEO
Injection #:	1	Date Processed:	2017/06/13 04:17:50 PM
Injection Volume:	15.00 ul	Processing Method:	HDI_RIF_ W2996 254.0nm-1.2
Run Time:	11.5 Minutes	Channel Name:	W2996 254.0nm-1.2
Sample Set Name:	RIF_BasL MOH Cntrls Rp 12_6_17	Proc. Chnl. Descr.:	W2996 PDA 254.0 nm at 1.2

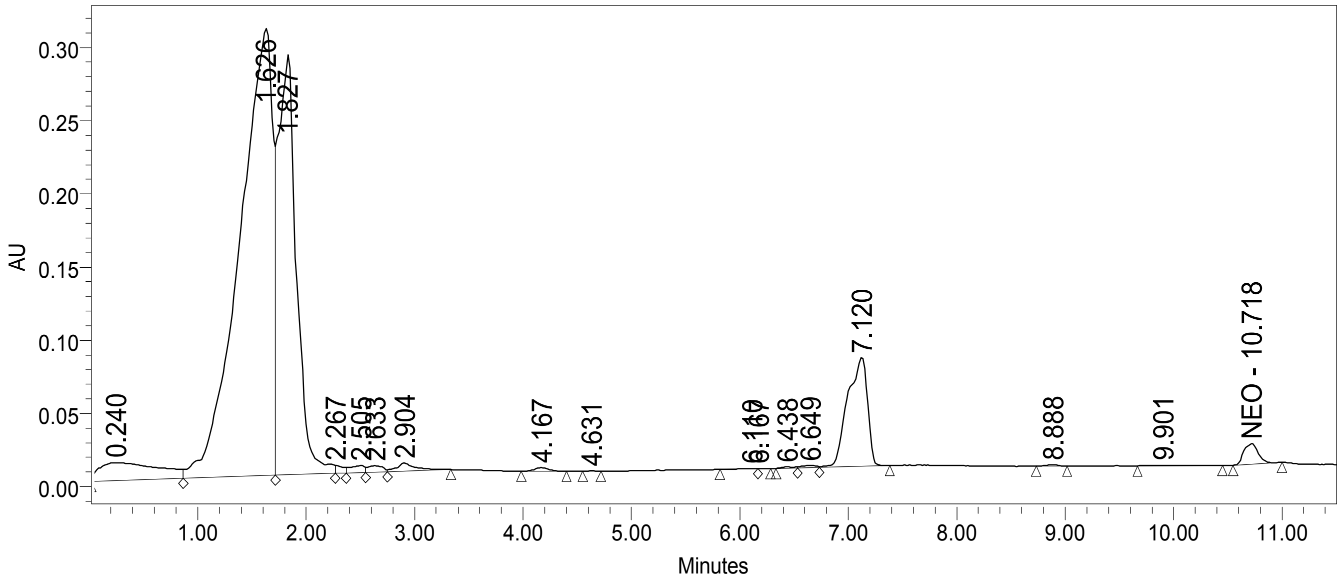

	Peak Name	RT	Area	% Area	Height
1		0.240	457117	3.93	12198
2		1.626	6462777	55.63	307094
3		1.827	3382982	29.12	287787
4		2.267	25922	0.22	5424
5		2.505	44172	0.38	4645
6		2.633	40529	0.35	4217
7		2.904	65375	0.56	5436
8		4.167	22088	0.19	2520
9		4.631	2005	0.02	350
10		6.110	3409	0.03	268
11		6.167	403	0.00	121
12		6.438	6411	0.06	761

Reported by User: Saneesh (Saneesh)   Project Name: HDI_Stellenbosch_2016

	Peak Name	RT	Area	% Area	Height
13		6.649	11967	0.10	1297
14		7.120	948313	8.16	74089
15	25ODESRIF	7.800			
16	RIF	8.100			
17		8.888	7484	0.06	863
18		9.901	8069	0.07	322
19	NEO	10.718	129107	1.11	13975

Reported by User: Saneesh (Saneesh) Project Name: HDI_Stellenbosch_2016

SAMPLE INFORMATION

Sample Name:	EFV Test Control 1	Acquired By:	Saneesh
Sample Type:	Unknown	Date Acquired:	2017/09/05 09:27:08 PM
Vial:	22	Acq. Method Set:	HDI_EFV_E8H_NEO
Injection #:	1	Date Processed:	2017/09/08 05:07:49 PM
Injection Volume:	15.00 ul	Processing Method:	HDI_EFV_ W2996 245.0nm-1.2
Run Time:	10.5 Minutes	Channel Name:	W2996 245.0nm-1.2
Sample Set Name:	EFV_Basil Aq IC50 5_9_17	Proc. Chnl. Descr.:	W2996 PDA 245.0 nm at 1.2

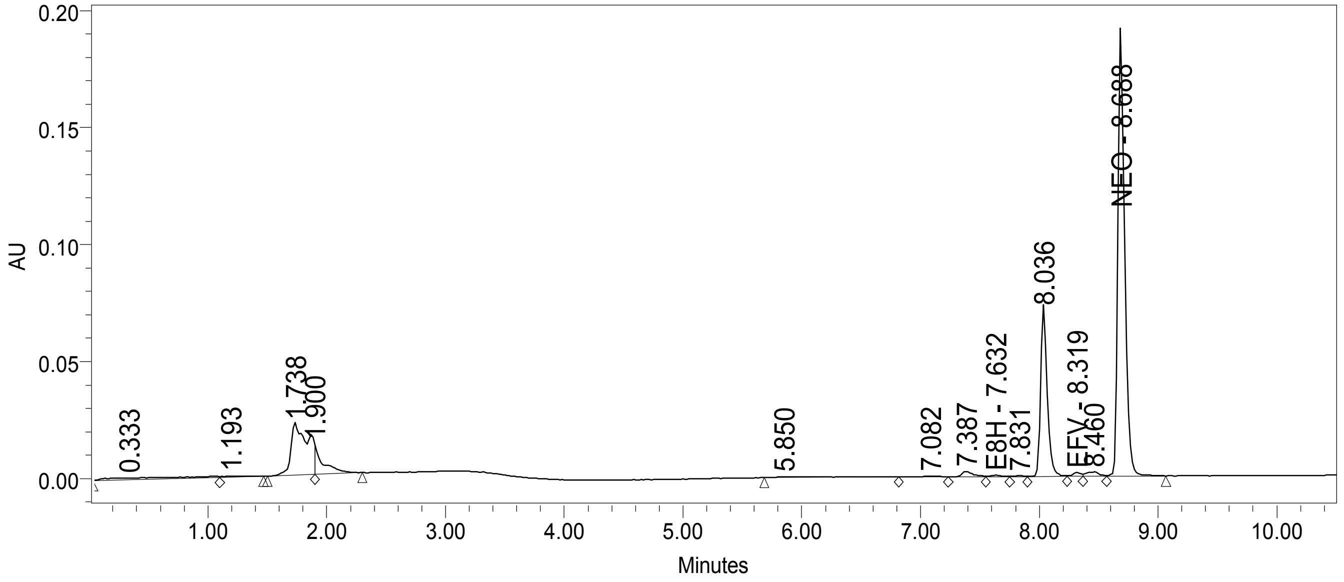

	Peak Name	RT	Area	% Area	Height
1		0.333	35436	2.51	765
2		1.193	5261	0.37	415
3		1.738	223035	15.82	21995
4		1.900	61040	4.33	12802
5		5.850	12406	0.88	315
6		7.082	4664	0.33	424
7		7.387	15142	1.07	2312
8	E8H	7.632	3709	0.26	639
9		7.831	2066	0.15	325
10		8.036	285415	20.24	70103
11	EFV	8.319	8162	0.58	1665
12		8.460	13953	0.99	1897
13	NEO	8.688	739918	52.47	182419

SAMPLE INFORMATION

Sample Name:	EFV Test Control 2	Acquired By:	Saneesh
Sample Type:	Unknown	Date Acquired:	2017/09/05 09:40:53 PM
Vial:	23	Acq. Method Set:	HDI_EFV_E8H_NEO
Injection #:	1	Date Processed:	2017/09/08 05:07:49 PM
Injection Volume:	15.00 ul	Processing Method:	HDI_EFV_ W2996 245.0nm-1.2
Run Time:	10.5 Minutes	Channel Name:	W2996 245.0nm-1.2
Sample Set Name:	EFV_Basil Aq IC50 5_9_17	Proc. Chnl. Descr.:	W2996 PDA 245.0 nm at 1.2

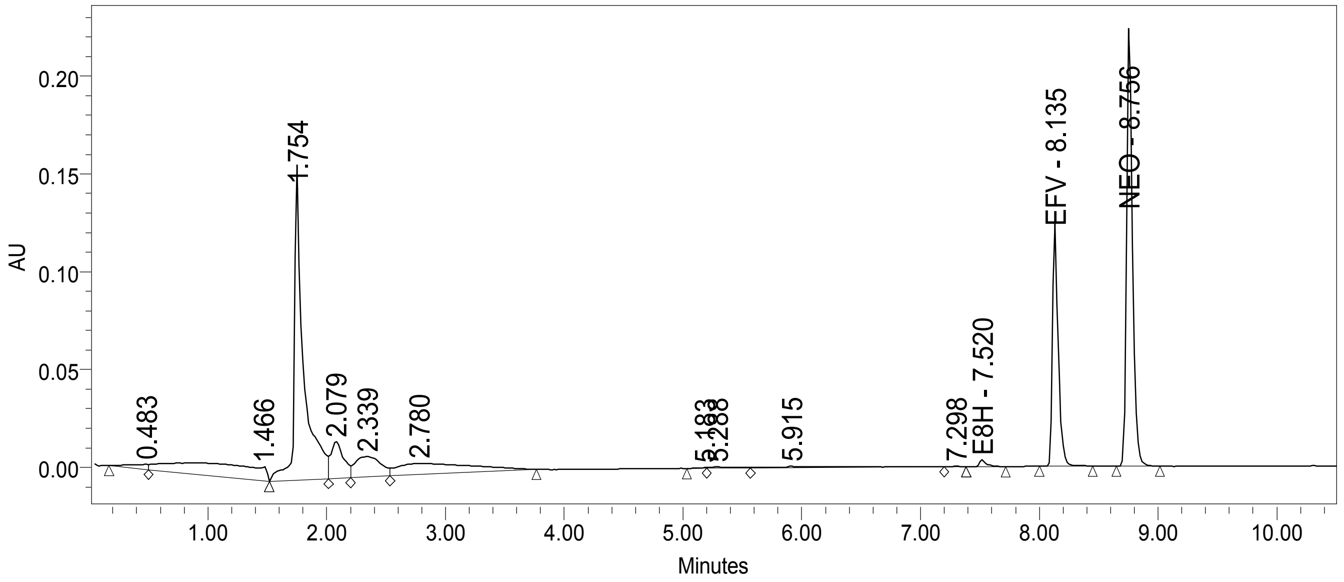

	Peak Name	RT	Area	% Area	Height
1		0.483	28604	0.94	2674
2		1.466	332170	10.92	7209
3		1.754	882352	29.01	148305
4		2.079	138593	4.56	18567
5		2.339	157450	5.18	10289
6		2.780	241417	7.94	5695
7		5.183	1767	0.06	338
8		5.288	8755	0.29	659
9		5.915	28176	0.93	817
10		7.298	1965	0.06	432
11	E8H	7.520	14620	0.48	3358
12	EFV	8.135	413574	13.60	114724

Reported by User: Saneesh (Saneesh) Project Name: HDI_Stellenbosch_2016

	Peak Name	RT	Area	% Area	Height
13	NEO	8.756	792124	26.04	209774

SAMPLE INFORMATION

Sample Name:	EFV Test Control 3	Acquired By:	Saneesh
Sample Type:	Unknown	Date Acquired:	2017/09/05 09:55:33 PM
Vial:	24	Acq. Method Set:	HDI_EFV_E8H_NEO
Injection #:	1	Date Processed:	2017/09/08 05:07:49 PM
Injection Volume:	15.00 ul	Processing Method:	HDI_EFV_ W2996 245.0nm-1.2
Run Time:	10.5 Minutes	Channel Name:	W2996 245.0nm-1.2
Sample Set Name:	EFV_Basil Aq IC50 5_9_17	Proc. Chnl. Descr.:	W2996 PDA 245.0 nm at 1.2

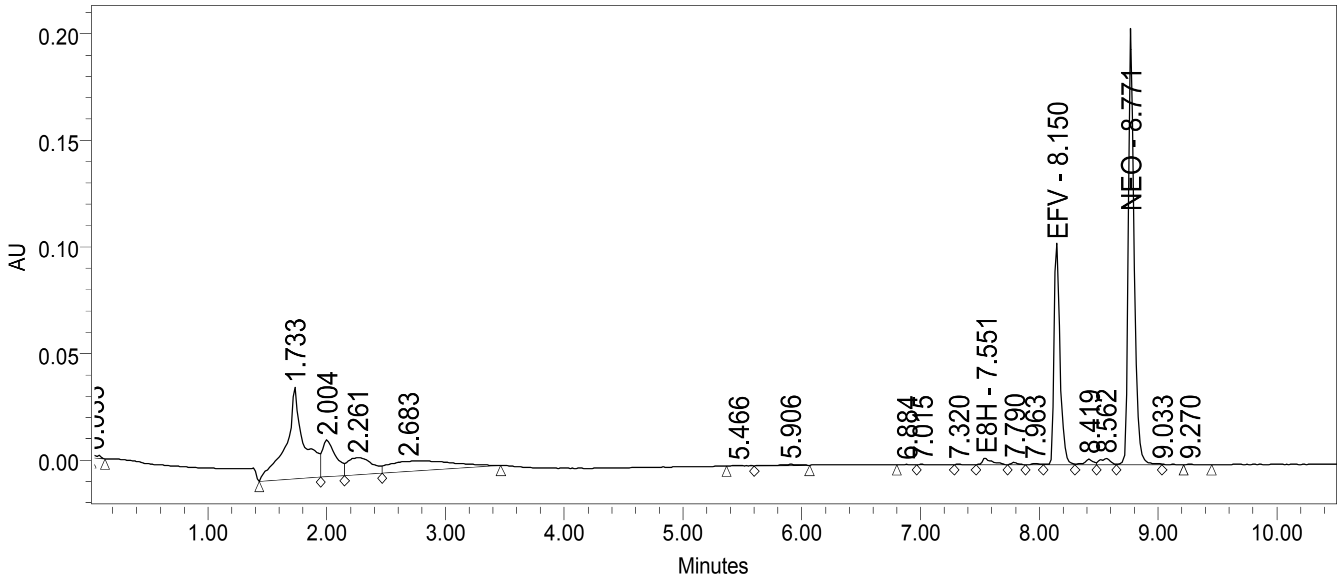

	Peak Name	RT	Area	% Area	Height
1		0.033	6057	0.30	1518
2		1.733	453030	22.16	40758
3		2.004	136651	6.68	17252
4		2.261	116521	5.70	7985
5		2.683	177721	8.69	4786
6		5.466	2804	0.14	427
7		5.906	7210	0.35	733
8		6.884	1004	0.05	255
9		7.015	2602	0.13	326
10		7.320	2151	0.11	485
11	E8H	7.551	19435	0.95	3055
12		7.790	4596	0.22	1050

Reported by User: Saneesh (Saneesh) Project Name: HDI_Stellenbosch_2016

	Peak Name	RT	Area	% Area	Height
13		7.963	3517	0.17	617
14	EFV	8.150	357142	17.47	98622
15		8.419	14051	0.69	2671
16		8.562	17520	0.86	2948
17	NEO	8.771	720282	35.23	190121
18		9.033	1084	0.05	300
19		9.270	1266	0.06	256

SAMPLE INFORMATION

Sample Name:	EFV Inc Basil_Aq1miG/mL 1	Acquired By:	Saneesh
Sample Type:	Unknown	Date Acquired:	2017/09/05 10:09:39 PM
Vial:	2	Acq. Method Set:	HDI_EFV_E8H_NEO
Injection #:	1	Date Processed:	2017/09/08 05:07:49 PM
Injection Volume:	15.00 ul	Processing Method:	HDI_EFV_ W2996 245.0nm-1.2
Run Time:	10.5 Minutes	Channel Name:	W2996 245.0nm-1.2
Sample Set Name:	EFV_Basil Aq IC50 5_9_17	Proc. Chnl. Descr.:	W2996 PDA 245.0 nm at 1.2

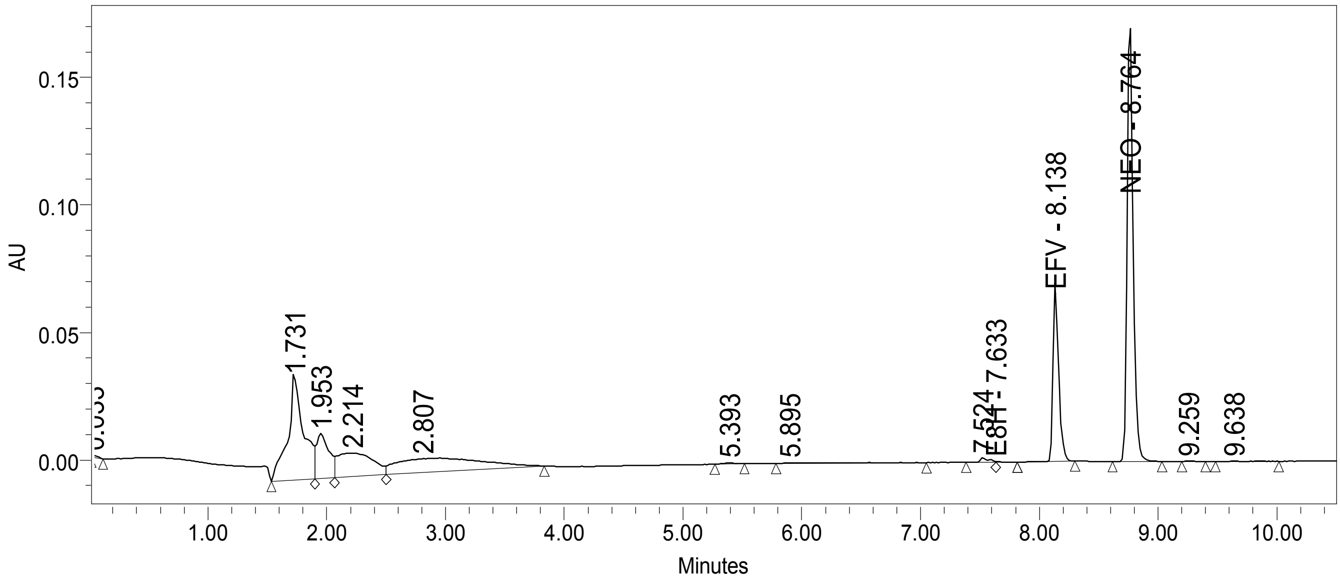

	Peak Name	RT	Area	% Area	Height
1		0.033	2637	0.14	871
2		1.731	378221	20.37	39235
3		1.953	130464	7.03	17395
4		2.214	194095	10.45	9278
5		2.807	275034	14.81	5528
6		5.393	2959	0.16	482
7		5.895	10194	0.55	266
8		7.524	9193	0.50	1630
9	E8H	7.633	1056	0.06	374
10	EFV	8.138	228180	12.29	63717
11	NEO	8.764	621124	33.45	164039
12		9.259	1297	0.07	248

	Peak Name	RT	Area	% Area	Height
13		9.638	2457	0.13	208

Reported by User: Saneesh (Saneesh)   Project Name: HDI_Stellenbosch_2016

SAMPLE INFORMATION

Sample Name:	EFV Inc Basil_Aq 1miG/mL 2	Acquired By:	Saneesh
Sample Type:	Unknown	Date Acquired:	2017/09/05 10:23:58 PM
Vial:	3	Acq. Method Set:	HDI_EFV_E8H_NEO
Injection #:	1	Date Processed:	2017/09/08 05:07:49 PM
Injection Volume:	15.00 ul	Processing Method:	HDI_EFV_ W2996 245.0nm-1.2
Run Time:	10.5 Minutes	Channel Name:	W2996 245.0nm-1.2
Sample Set Name:	EFV_Basil Aq IC50 5_9_17	Proc. Chnl. Descr.:	W2996 PDA 245.0 nm at 1.2

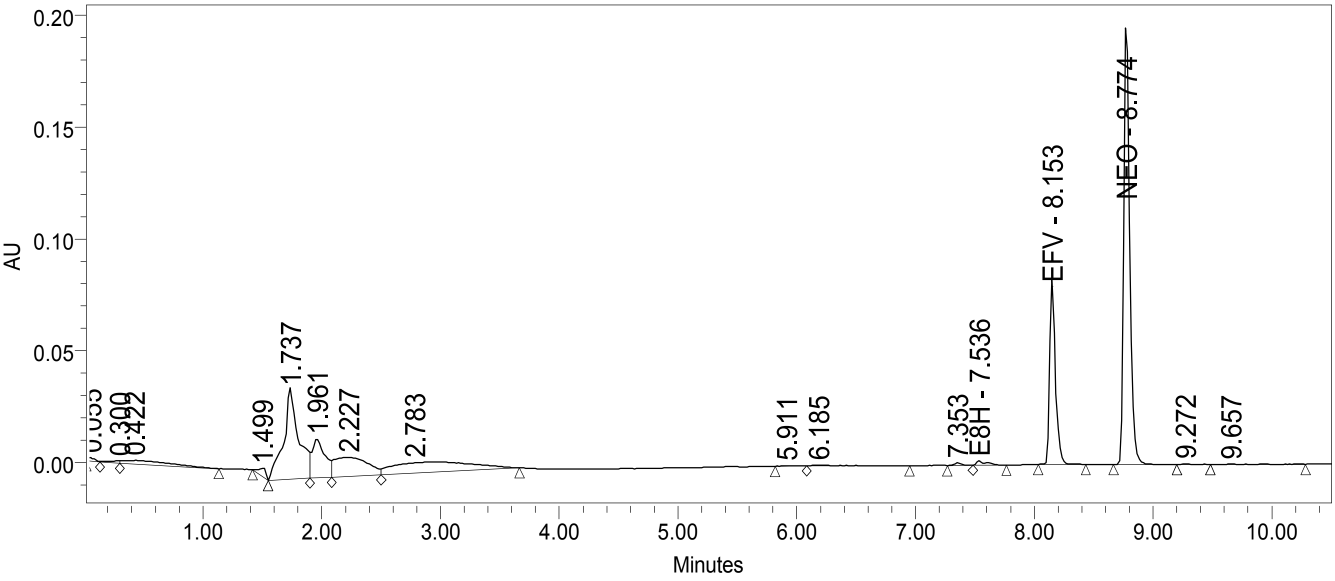

	Peak Name	RT	Area	% Area	Height
1		0.055	6399	0.33	1633
2		0.300	4776	0.24	885
3		0.422	41547	2.12	1483
4		1.499	15519	0.79	3608
5		1.737	357516	18.26	40337
6		1.961	133626	6.83	17121
7		2.227	166337	8.50	8410
8		2.783	213152	10.89	4699
9		5.911	2613	0.13	267
10		6.185	8157	0.42	435
11		7.353	5226	0.27	1150
12	E8H	7.536	11732	0.60	1894

	Peak Name	RT	Area	% Area	Height
13	EFV	8.153	272324	13.91	76339
14	NEO	8.774	711374	36.34	186197
15		9.272	2778	0.14	490
16		9.657	4372	0.22	309

Reported by User: Saneesh (Saneesh) Project Name: HDI_Stellenbosch_2016

SAMPLE INFORMATION

Sample Name:	EFV Inc Basil_Aq 1miG/mL 3	Acquired By:	Saneesh
Sample Type:	Unknown	Date Acquired:	2017/09/05 10:37:54 PM
Vial:	4	Acq. Method Set:	HDI_EFV_E8H_NEO
Injection #:	1	Date Processed:	2017/09/08 05:07:49 PM
Injection Volume:	15.00 ul	Processing Method:	HDI_EFV_ W2996 245.0nm-1.2
Run Time:	10.5 Minutes	Channel Name:	W2996 245.0nm-1.2
Sample Set Name:	EFV_Basil Aq IC50 5_9_17	Proc. Chnl. Descr.:	W2996 PDA 245.0 nm at 1.2

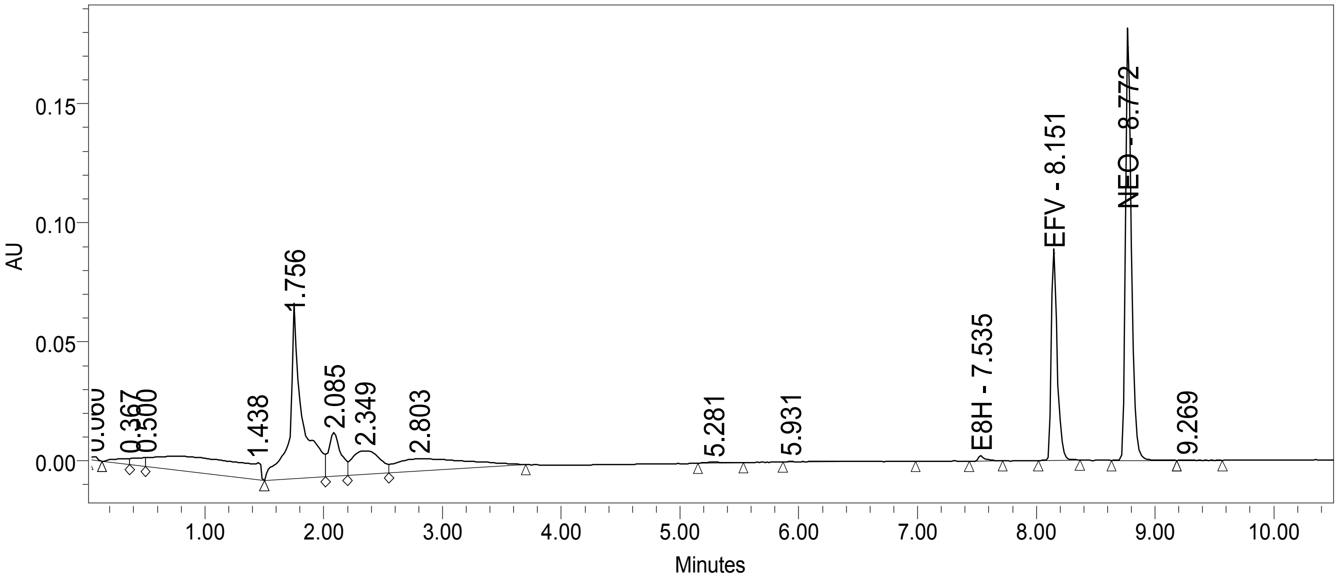

	Peak Name	RT	Area	% Area	Height
1		0.060	6376	0.26	1607
2		0.367	22093	0.91	2655
3		0.500	25751	1.06	3714
4		1.438	345639	14.26	7668
5		1.756	546623	22.54	64773
6		2.085	130521	5.38	18192
7		2.349	159650	6.58	9773
8		2.803	213876	8.82	5238
9		5.281	4127	0.17	484
10		5.931	9711	0.40	381
11	E8H	7.535	9369	0.39	2070
12	EFV	8.151	300758	12.40	82958

Reported by User: Saneesh (Saneesh) Project Name: HDI_Stellenbosch_2016

	Peak Name	RT	Area	% Area	Height
13	NEO	8.772	647866	26.72	170253
14		9.269	2244	0.09	297

SAMPLE INFORMATION

Sample Name:	EFV Inc Basil_Aq 10miG/mL 1	Acquired By:	Saneesh
Sample Type:	Unknown	Date Acquired:	2017/09/05 10:51:49 PM
Vial:	5	Acq. Method Set:	HDI_EFV_E8H_NEO
Injection #:	1	Date Processed:	2017/09/08 05:07:49 PM
Injection Volume:	15.00 ul	Processing Method:	HDI_EFV_ W2996 245.0nm-1.2
Run Time:	10.5 Minutes	Channel Name:	W2996 245.0nm-1.2
Sample Set Name:	EFV_Basil Aq IC50 5_9_17	Proc. Chnl. Descr.:	W2996 PDA 245.0 nm at 1.2

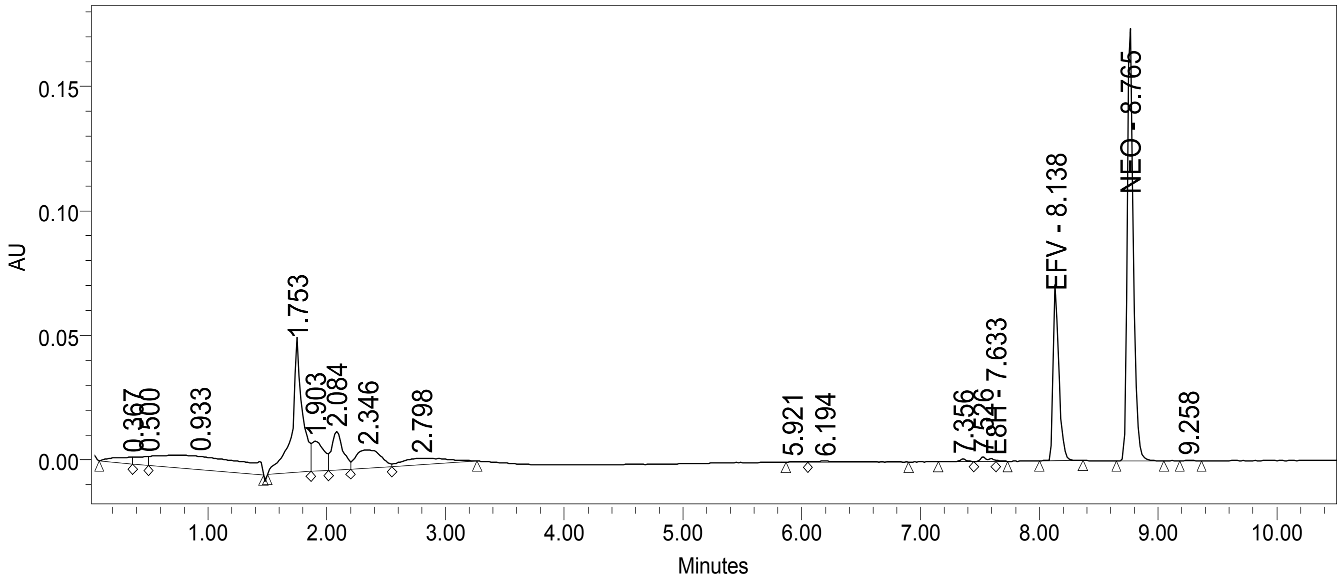

	Peak Name	RT	Area	% Area	Height
1		0.367	29019	1.51	2689
2		0.500	25179	1.31	3527
3		0.933	276478	14.35	5362
4		1.753	333031	17.28	49426
5		1.903	89684	4.65	12021
6		2.084	102470	5.32	15428
7		2.346	105469	5.47	7238
8		2.798	67825	3.52	2608
9		5.921	1294	0.07	237
10		6.194	6889	0.36	409
11		7.356	4549	0.24	1052
12		7.526	9721	0.50	1678

	Peak Name	RT	Area	% Area	Height
13	E8H	7.633	936	0.05	520
14	EFV	8.138	236071	12.25	64777
15	NEO	8.765	636368	33.03	166790
16		9.258	1869	0.10	404

SAMPLE INFORMATION

Sample Name:	EFV Inc Basil_Aq 10miG/mL 2	Acquired By:	Saneesh
Sample Type:	Unknown	Date Acquired:	2017/09/05 11:05:46 PM
Vial:	6	Acq. Method Set:	HDI_EFV_E8H_NEO
Injection #:	1	Date Processed:	2017/09/08 05:07:49 PM
Injection Volume:	15.00 ul	Processing Method:	HDI_EFV_ W2996 245.0nm-1.2
Run Time:	10.5 Minutes	Channel Name:	W2996 245.0nm-1.2
Sample Set Name:	EFV_Basil Aq IC50 5_9_17	Proc. Chnl. Descr.:	W2996 PDA 245.0 nm at 1.2

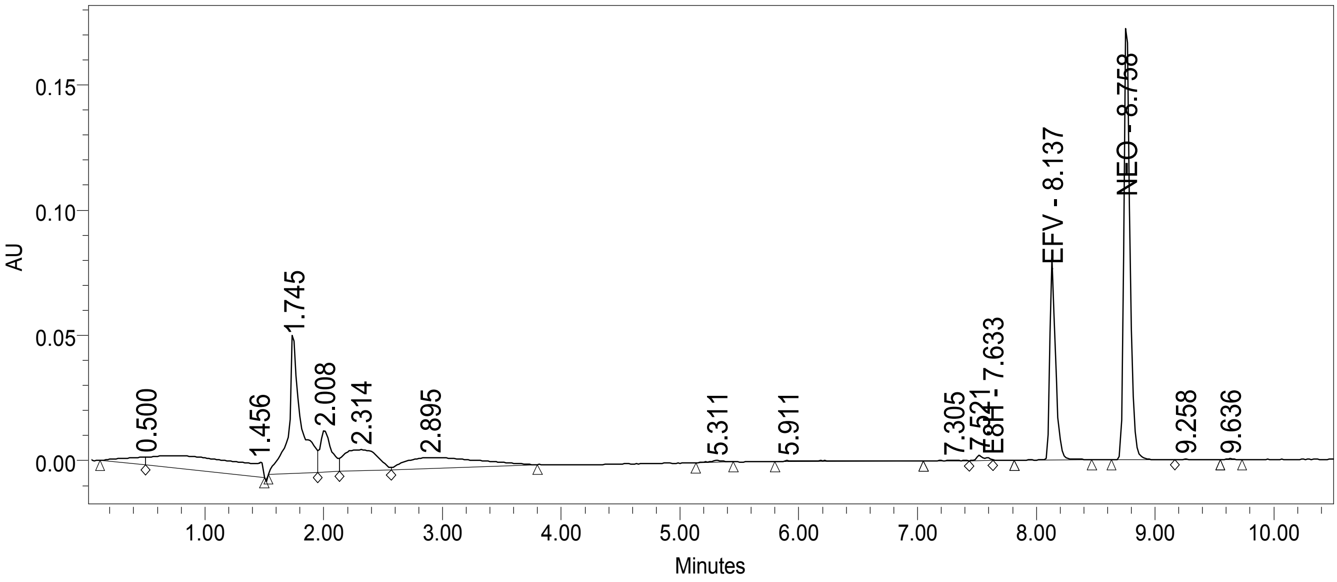

	Peak Name	RT	Area	% Area	Height
1		0.500	38741	1.81	3168
2		1.456	293826	13.76	6397
3		1.745	401005	18.77	50464
4		2.008	112316	5.26	16506
5		2.314	164232	7.69	8458
6		2.895	182471	8.54	4261
7		5.311	4531	0.21	603
8		5.911	11348	0.53	333
9		7.305	1771	0.08	208
10		7.521	10696	0.50	2081
11	E8H	7.633	1083	0.05	367
12	EFV	8.137	270714	12.67	73809

	Peak Name	RT	Area	% Area	Height
13	NEO	8.758	638865	29.91	165177
14		9.258	2049	0.10	298
15		9.636	2269	0.11	453

SAMPLE INFORMATION

Sample Name:	EFV Inc Basil_Aq 10miG/mL 3	Acquired By:	Saneesh
Sample Type:	Unknown	Date Acquired:	2017/09/05 11:19:42 PM
Vial:	7	Acq. Method Set:	HDI_EFV_E8H_NEO
Injection #:	1	Date Processed:	2017/09/08 05:07:49 PM
Injection Volume:	15.00 ul	Processing Method:	HDI_EFV_ W2996 245.0nm-1.2
Run Time:	10.5 Minutes	Channel Name:	W2996 245.0nm-1.2
Sample Set Name:	EFV_Basil Aq IC50 5_9_17	Proc. Chnl. Descr.:	W2996 PDA 245.0 nm at 1.2

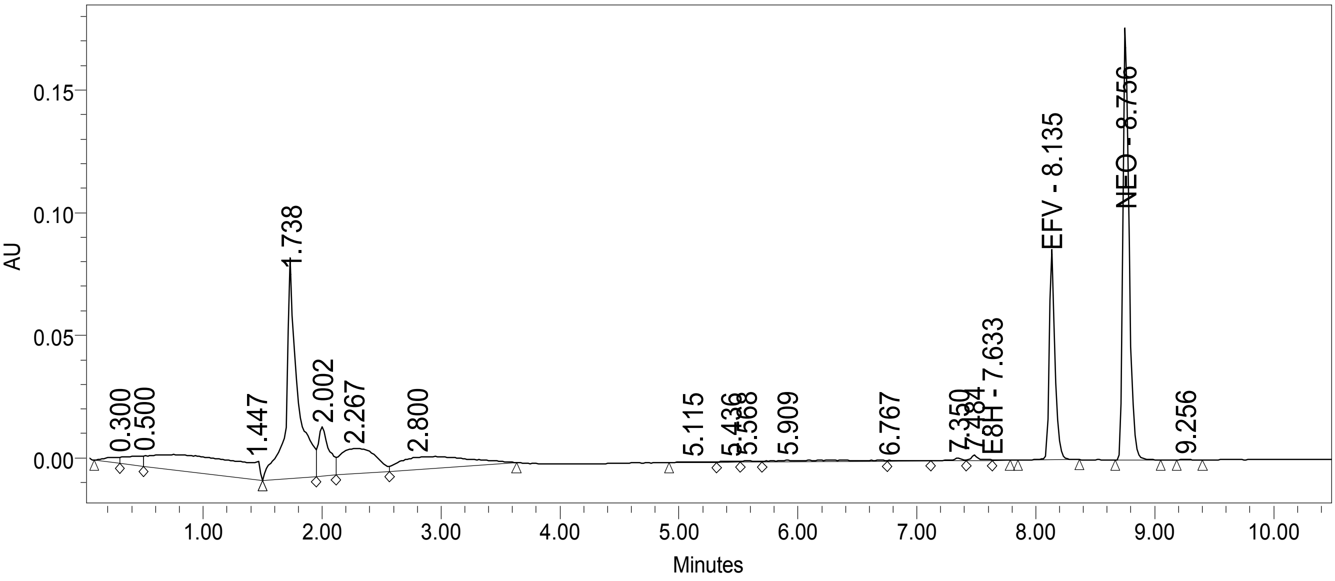

	Peak Name	RT	Area	% Area	Height
1		0.300	19265	0.75	2510
2		0.500	40266	1.57	4175
3		1.447	367764	14.38	7516
4		1.738	603411	23.60	81939
5		2.002	131146	5.13	19899
6		2.267	211475	8.27	10255
7		2.800	195323	7.64	4978
8		5.115	6043	0.24	337
9		5.436	3939	0.15	362
10		5.568	4967	0.19	650
11		5.909	28722	1.12	666
12		6.767	3814	0.15	324

	Peak Name	RT	Area	% Area	Height
13		7.350	5884	0.23	1063
14		7.484	10484	0.41	2105
15	E8H	7.633	915	0.04	225
16	EFV	8.135	292246	11.43	79873
17	NEO	8.756	630124	24.64	165230
18		9.256	1519	0.06	350

SAMPLE INFORMATION

Sample Name:	EFV Inc Basil_Aq 50miG/mL 1	Acquired By:	Saneesh
Sample Type:	Unknown	Date Acquired:	2017/09/05 11:33:35 PM
Vial:	8	Acq. Method Set:	HDI_EFV_E8H_NEO
Injection #:	1	Date Processed:	2017/09/08 05:07:49 PM
Injection Volume:	15.00 ul	Processing Method:	HDI_EFV_ W2996 245.0nm-1.2
Run Time:	10.5 Minutes	Channel Name:	W2996 245.0nm-1.2
Sample Set Name:	EFV_Basil Aq IC50 5_9_17	Proc. Chnl. Descr.:	W2996 PDA 245.0 nm at 1.2

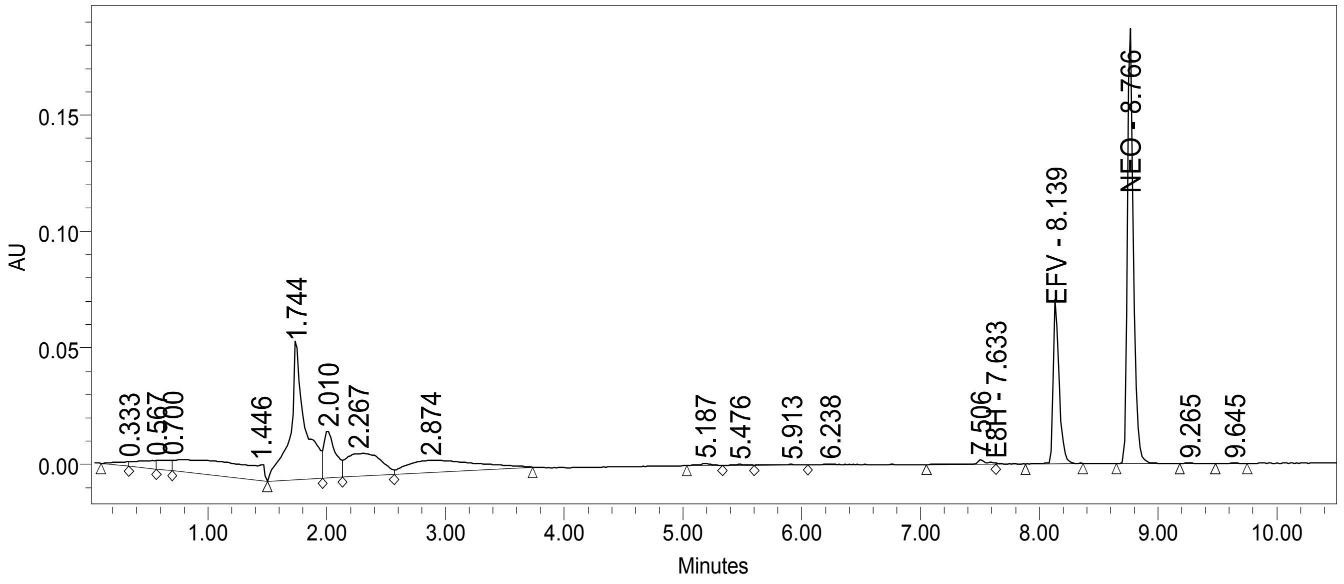

	Peak Name	RT	Area	% Area	Height
1		0.333	13968	0.58	1894
2		0.567	39636	1.66	3798
3		0.700	33966	1.42	4657
4		1.446	275687	11.52	6767
5		1.744	525612	21.97	54794
6		2.010	133362	5.57	19966
7		2.267	197578	8.26	9739
8		2.874	210787	8.81	5125
9		5.187	7573	0.32	1006
10		5.476	3822	0.16	542
11		5.913	6057	0.25	407
12		6.238	10903	0.46	466

Reported by User: Saneesh (Saneesh) Project Name: HDI_Stellenbosch_2016

	Peak Name	RT	Area	% Area	Height
13		7.506	11067	0.46	1716
14	E8H	7.633	1437	0.06	420
15	EFV	8.139	240912	10.07	64535
16	NEO	8.766	676511	28.28	178171
17		9.265	1735	0.07	259
18		9.645	1622	0.07	239

SAMPLE INFORMATION

Sample Name:	EFV Inc Basil_Aq 50miG/mL 2	Acquired By:	Saneesh
Sample Type:	Unknown	Date Acquired:	2017/09/05 11:47:33 PM
Vial:	9	Acq. Method Set:	HDI_EFV_E8H_NEO
Injection #:	1	Date Processed:	2017/09/08 05:07:49 PM
Injection Volume:	15.00 ul	Processing Method:	HDI_EFV_ W2996 245.0nm-1.2
Run Time:	10.5 Minutes	Channel Name:	W2996 245.0nm-1.2
Sample Set Name:	EFV_Basil Aq IC50 5_9_17	Proc. Chnl. Descr.:	W2996 PDA 245.0 nm at 1.2

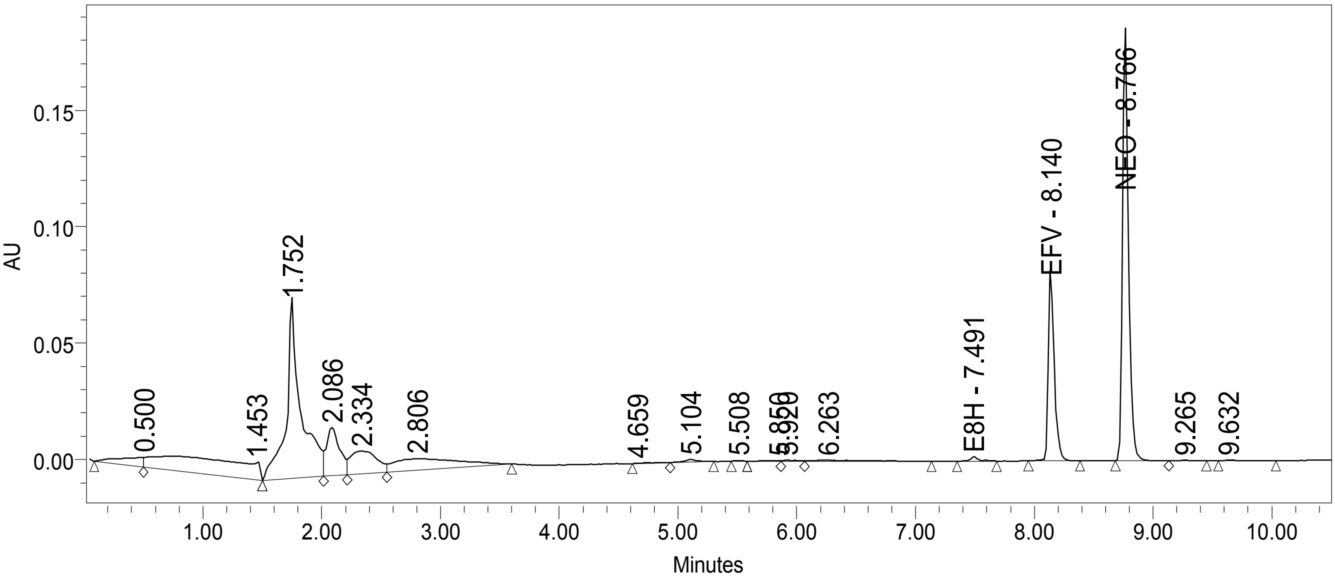

	Peak Name	RT	Area	% Area	Height
1		0.500	55631	2.15	3991
2		1.453	361825	13.99	7911
3		1.752	665224	25.72	73937
4		2.086	163244	6.31	20839
5		2.334	151503	5.86	9768
6		2.806	188874	7.30	4988
7		4.659	3087	0.12	260
8		5.104	9194	0.36	1180
9		5.508	1675	0.06	381
10		5.850	2949	0.11	278
11		5.920	4448	0.17	524
12		6.263	15595	0.60	578

Reported by User: Saneesh (Saneesh) Project Name: HDI_Stellenbosch_2016

	Peak Name	RT	Area	% Area	Height
13	E8H	7.491	9400	0.36	1774
14	EFV	8.140	281875	10.90	75233
15	NEO	8.766	668152	25.83	176085
16		9.265	2031	0.08	332
17		9.632	2196	0.08	203

SAMPLE INFORMATION

Sample Name:	EFV IncBasil_Aq 50mig/mL 3	Acquired By:	Saneesh
Sample Type:	Unknown	Date Acquired:	2017/09/06 12:01:28 AM
Vial:	10	Acq. Method Set:	HDI_EFV_E8H_NEO
Injection #:	1	Date Processed:	2017/09/08 05:07:49 PM
Injection Volume:	15.00 ul	Processing Method:	HDI_EFV_ W2996 245.0nm-1.2
Run Time:	10.5 Minutes	Channel Name:	W2996 245.0nm-1.2
Sample Set Name:	EFV_Basil Aq IC50 5_9_17	Proc. Chnl. Descr.:	W2996 PDA 245.0 nm at 1.2

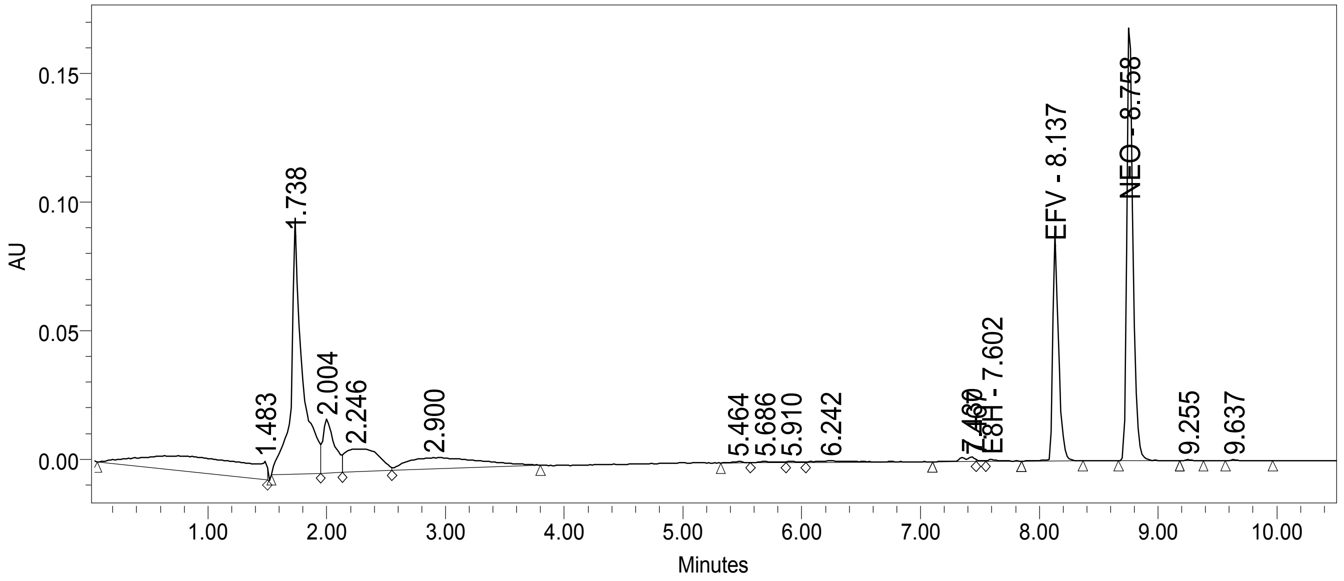

	Peak Name	RT	Area	% Area	Height
1		1.483	394082	15.54	7134
2		1.738	661646	26.09	91431
3		2.004	141998	5.60	20699
4		2.246	171701	6.77	8932
5		2.900	191220	7.54	4415
6		5.464	3808	0.15	567
7		5.686	5340	0.21	472
8		5.910	3496	0.14	494
9		6.242	14296	0.56	574
10		7.430	12770	0.50	1781
11		7.467	1627	0.06	711
12	E8H	7.602	4235	0.17	764

Reported by User: Saneesh (Saneesh) Project Name: HDI_Stellenbosch_2016

	Peak Name	RT	Area	% Area	Height
13	EFV	8.137	296547	11.69	81020
14	NEO	8.758	628966	24.80	160845
15		9.255	1929	0.08	435
16		9.637	2619	0.10	398

Reported by User: Saneesh (Saneesh)   Project Name: HDI_Stellenbosch_2016

SAMPLE INFORMATION

Sample Name:	EFV Inc Basil_Aq 100mig/mL 1	Acquired By:	Saneesh
Sample Type:	Unknown	Date Acquired:	2017/09/06 12:15:23 AM
Vial:	11	Acq. Method Set:	HDI_EFV_E8H_NEO
Injection #:	1	Date Processed:	2017/09/08 05:07:49 PM
Injection Volume:	15.00 ul	Processing Method:	HDI_EFV_ W2996 245.0nm-1.2
Run Time:	10.5 Minutes	Channel Name:	W2996 245.0nm-1.2
Sample Set Name:	EFV_Basil Aq IC50 5_9_17	Proc. Chnl. Descr.:	W2996 PDA 245.0 nm at 1.2

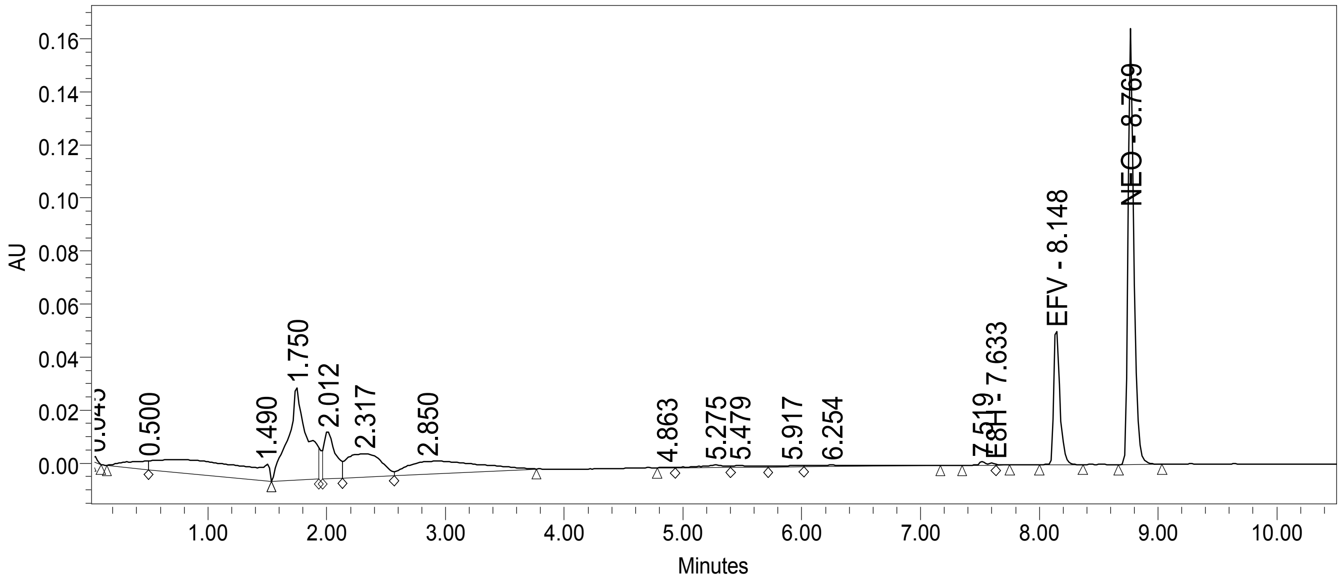

	Peak Name	RT	Area	% Area	Height
1		0.045	4921	0.24	1853
2		0.500	40731	2.02	3229
3		1.490	290239	14.38	6499
4		1.750	384244	19.04	34041
5		2.012	116286	5.76	17518
6		2.317	174463	8.64	8686
7		2.850	198307	9.83	4699
8		4.863	1618	0.08	262
9		5.275	12726	0.63	955
10		5.479	7912	0.39	744
11		5.917	7086	0.35	552
12		6.254	18032	0.89	576

Reported by User: Saneesh (Saneesh) Project Name: HDI_Stellenbosch_2016

	Peak Name	RT	Area	% Area	Height
13		7.519	8294	0.41	1356
14	E8H	7.633	1117	0.06	483
15	EFV	8.148	181734	9.00	48813
16	NEO	8.769	570634	28.27	152886

Multi Sample Summary

Reported by User: Saneesh (Saneesh)   Project Name: HDI_Stellenbosch_2016

SAMPLE INFORMATION

Sample Name:	EFV Inc Basil_Aq 100mig/mL 2	Acquired By:	Saneesh
Sample Type:	Unknown	Date Acquired:	2017/09/06 12:29:18 AM
Vial:	12	Acq. Method Set:	HDI_EFV_E8H_NEO
Injection #:	1	Date Processed:	2017/09/08 05:07:49 PM
Injection Volume:	15.00 ul	Processing Method:	HDI_EFV_ W2996 245.0nm-1.2
Run Time:	10.5 Minutes	Channel Name:	W2996 245.0nm-1.2
Sample Set Name:	EFV_Basil Aq IC50 5_9_17	Proc. Chnl. Descr.:	W2996 PDA 245.0 nm at 1.2

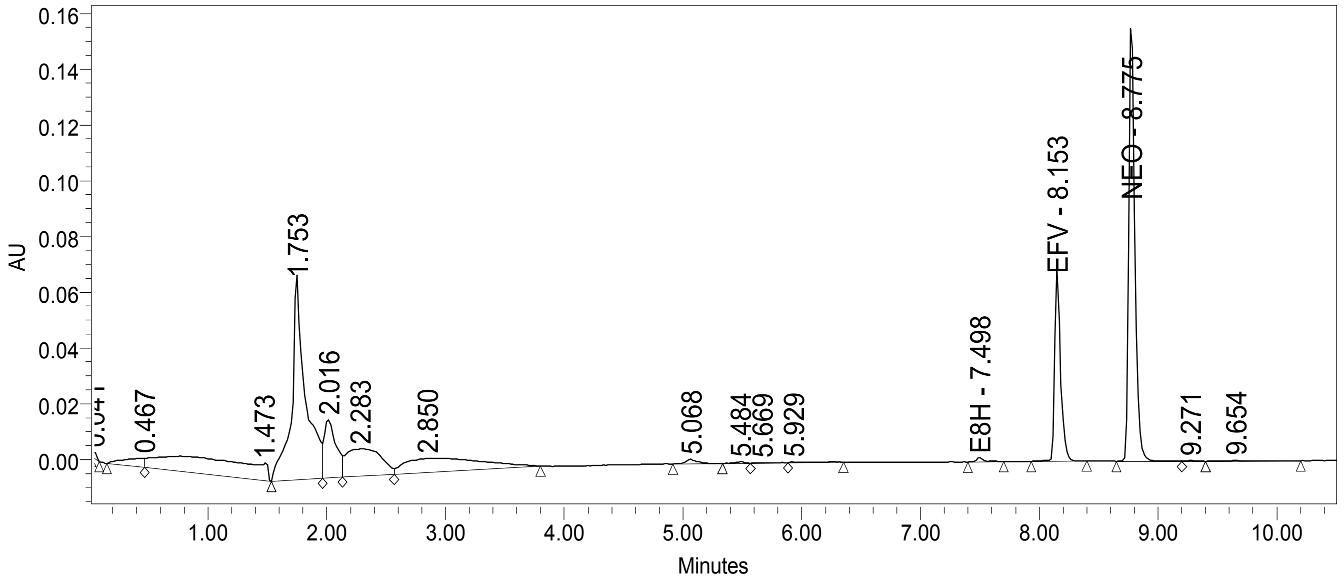

	Peak Name	RT	Area	% Area	Height
1		0.041	4913	0.21	1923
2		0.467	39404	1.66	3369
3		1.473	325435	13.71	6729
4		1.753	597574	25.17	71187
5		2.016	142659	6.01	20884
6		2.283	199553	8.41	9736
7		2.850	219579	9.25	4973
8		5.068	11318	0.48	1602
9		5.484	3447	0.15	622
10		5.669	1746	0.07	195
11		5.929	4128	0.17	318
12	E8H	7.498	7639	0.32	1422

Reported by User: Saneesh (Saneesh) Project Name: HDI_Stellenbosch_2016

	Peak Name	RT	Area	% Area	Height
13	EFV	8.153	232853	9.81	63515
14	NEO	8.775	578003	24.35	148436
15		9.271	1813	0.08	332
16		9.654	4152	0.17	328

Reported by User: Saneesh (Saneesh)   Project Name: HDI_Stellenbosch_2016

SAMPLE INFORMATION

Sample Name:	EFV Inc Basil_Aq 100mig/mL 3	Acquired By:	Saneesh
Sample Type:	Unknown	Date Acquired:	2017/09/06 12:43:15 AM
Vial:	13	Acq. Method Set:	HDI_EFV_E8H_NEO
Injection #:	1	Date Processed:	2017/09/08 05:07:49 PM
Injection Volume:	15.00 ul	Processing Method:	HDI_EFV_ W2996 245.0nm-1.2
Run Time:	10.5 Minutes	Channel Name:	W2996 245.0nm-1.2
Sample Set Name:	EFV_Basil Aq IC50 5_9_17	Proc. Chnl. Descr.:	W2996 PDA 245.0 nm at 1.2

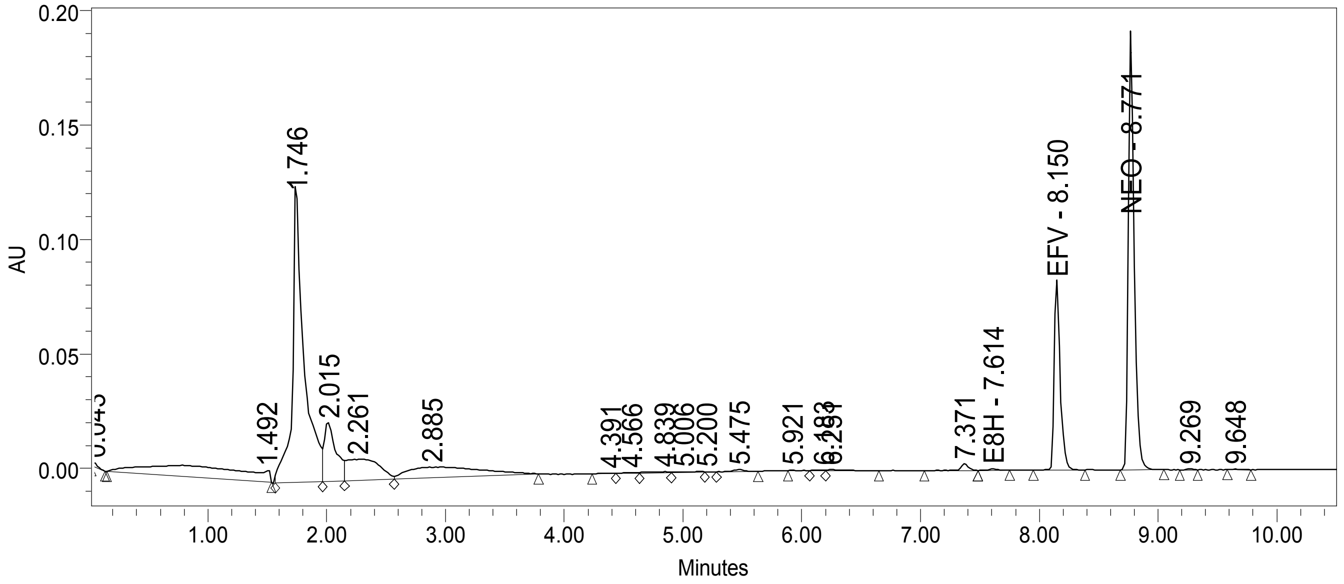

	Peak Name	RT	Area	% Area	Height
1		0.043	4873	0.18	1581
2		1.492	301271	10.92	5377
3		1.746	886397	32.12	120232
4		2.015	176483	6.39	25537
5		2.261	174965	6.34	9083
6		2.885	196505	7.12	4512
7		4.391	2255	0.08	256
8		4.566	3332	0.12	320
9		4.839	7381	0.27	543
10		5.006	5295	0.19	381
11		5.200	1075	0.04	220
12		5.475	6836	0.25	915

Reported by User: Saneesh (Saneesh) Project Name: HDI_Stellenbosch_2016

	Peak Name	RT	Area	% Area	Height
13		5.921	1538	0.06	302
14		6.183	1491	0.05	271
15		6.251	4906	0.18	470
16		7.371	13464	0.49	2898
17	E8H	7.614	3068	0.11	690
18	EFV	8.150	285635	10.35	78118
19	NEO	8.771	679715	24.63	178812
20		9.269	1775	0.06	469
21		9.648	1699	0.06	292

SAMPLE INFORMATION

Sample Name:	EFV Inc Basil_Aq 150mig/mL 1	Acquired By:	Saneesh
Sample Type:	Unknown	Date Acquired:	2017/09/06 12:57:09 AM
Vial:	14	Acq. Method Set:	HDI_EFV_E8H_NEO
Injection #:	1	Date Processed:	2017/09/08 05:07:49 PM
Injection Volume:	15.00 ul	Processing Method:	HDI_EFV_ W2996 245.0nm-1.2
Run Time:	10.5 Minutes	Channel Name:	W2996 245.0nm-1.2
Sample Set Name:	EFV_Basil Aq IC50 5_9_17	Proc. Chnl. Descr.:	W2996 PDA 245.0 nm at 1.2

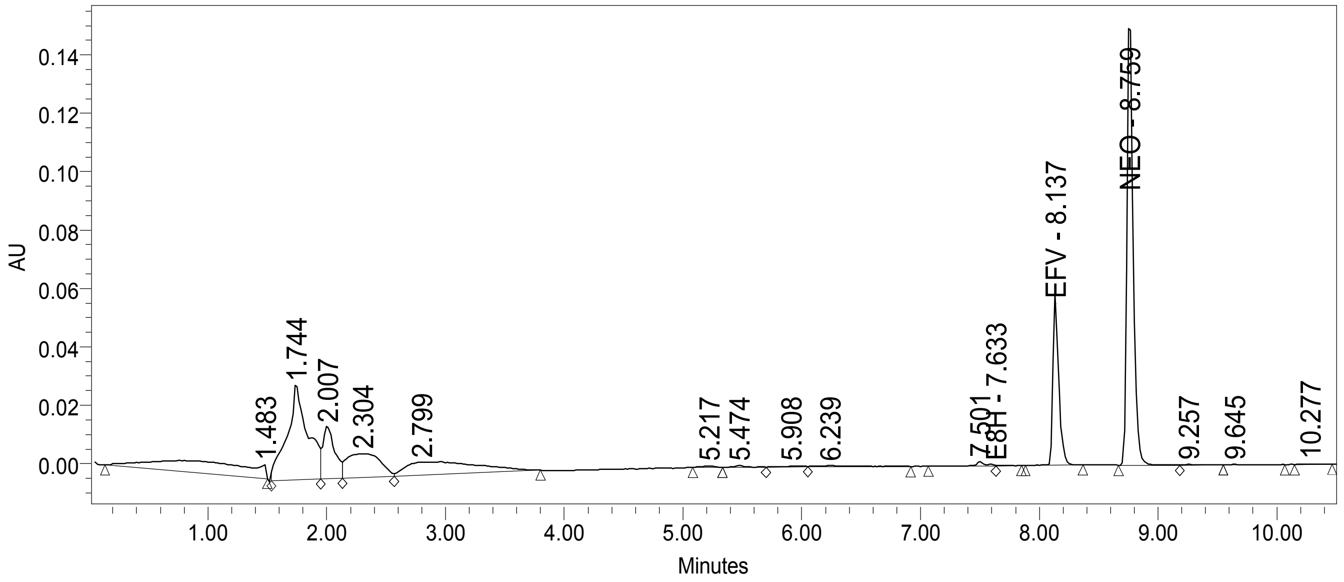

	Peak Name	RT	Area	% Area	Height
1		1.483	239386	12.50	4523
2		1.744	397237	20.75	31278
3		2.007	125475	6.55	17947
4		2.304	159977	8.36	8103
5		2.799	186896	9.76	4366
6		5.217	2987	0.16	432
7		5.474	3537	0.18	561
8		5.908	3912	0.20	369
9		6.239	9565	0.50	466
10		7.501	9281	0.48	1435
11	E8H	7.633	1002	0.05	228
12	EFV	8.137	199292	10.41	53961

	Peak Name	RT	Area	% Area	Height
13	NEO	8.759	569829	29.76	144725
14		9.257	2440	0.13	311
15		9.645	2541	0.13	237
16		10.277	1209	0.06	85

Reported by User: Saneesh (Saneesh) Project Name: HDI_Stellenbosch_2016

SAMPLE INFORMATION

Sample Name:	EFV Inc Basil_Aq 150mig/mL 2	Acquired By:	Saneesh
Sample Type:	Unknown	Date Acquired:	2017/09/06 01:11:06 AM
Vial:	15	Acq. Method Set:	HDI_EFV_E8H_NEO
Injection #:	1	Date Processed:	2017/09/08 05:07:50 PM
Injection Volume:	15.00 ul	Processing Method:	HDI_EFV_ W2996 245.0nm-1.2
Run Time:	10.5 Minutes	Channel Name:	W2996 245.0nm-1.2
Sample Set Name:	EFV_Basil Aq IC50 5_9_17	Proc. Chnl. Descr.:	W2996 PDA 245.0 nm at 1.2

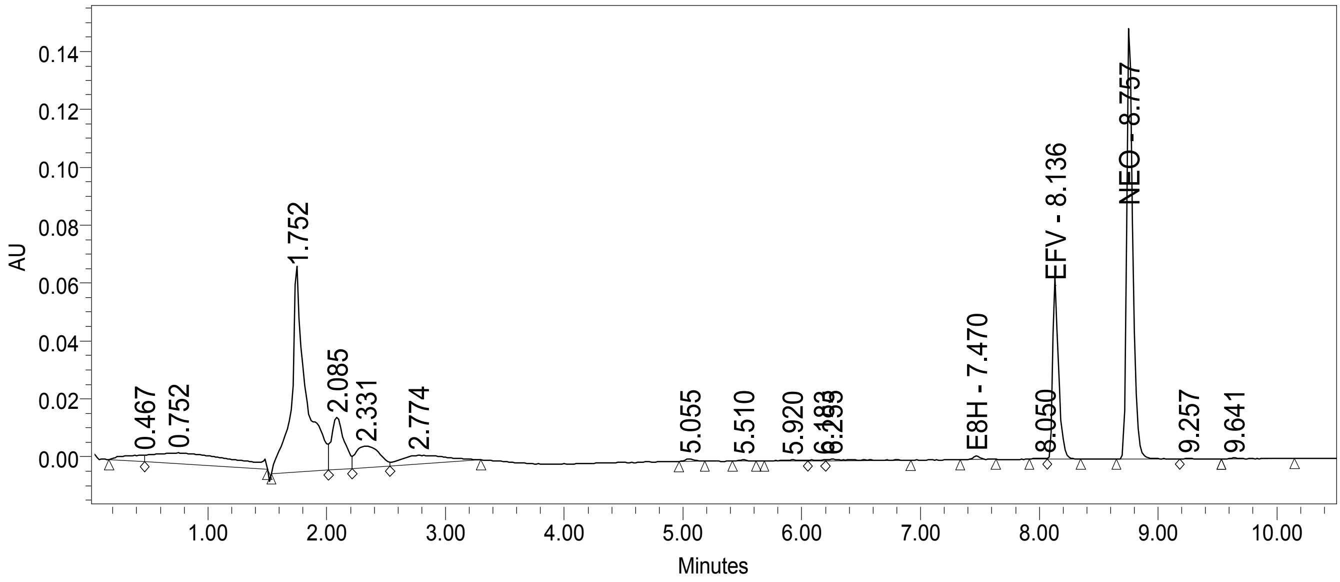

	Peak Name	RT	Area	% Area	Height
1		0.467	26879	1.38	2332
2		0.752	185546	9.53	3734
3		1.752	615536	31.60	68829
4		2.085	136718	7.02	18077
5		2.331	100709	5.17	7352
6		2.774	81531	4.19	3056
7		5.055	4269	0.22	869
8		5.510	2482	0.13	564
9		5.920	3933	0.20	426
10		6.183	2444	0.13	366
11		6.255	8699	0.45	509
12	E8H	7.470	5440	0.28	1230

Reported by User: Saneesh (Saneesh) Project Name: HDI_Stellenbosch_2016

	Peak Name	RT	Area	% Area	Height
13		8.050	1778	0.09	346
14	EFV	8.136	213345	10.95	58115
15	NEO	8.757	552398	28.36	141121
16		9.257	2299	0.12	298
17		9.641	3831	0.20	364

SAMPLE INFORMATION

Sample Name:	EFV Inc Basil_Aq 150mig/mL 3	Acquired By:	Saneesh
Sample Type:	Unknown	Date Acquired:	2017/09/06 01:25:00 AM
Vial:	16	Acq. Method Set:	HDI_EFV_E8H_NEO
Injection #:	1	Date Processed:	2017/09/08 05:07:50 PM
Injection Volume:	15.00 ul	Processing Method:	HDI_EFV_ W2996 245.0nm-1.2
Run Time:	10.5 Minutes	Channel Name:	W2996 245.0nm-1.2
Sample Set Name:	EFV_Basil Aq IC50 5_9_17	Proc. Chnl. Descr.:	W2996 PDA 245.0 nm at 1.2

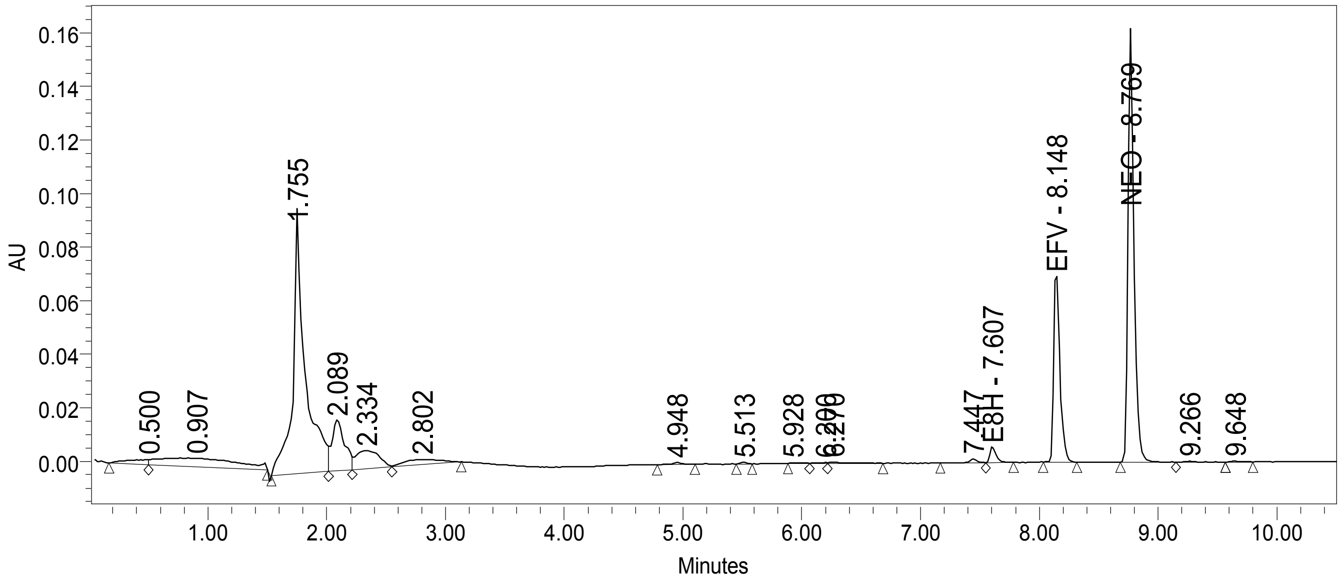

	Peak Name	RT	Area	% Area	Height
1		0.500	24313	1.18	2011
2		0.907	156366	7.62	3206
3		1.755	712747	34.72	89724
4		2.089	142962	6.96	19015
5		2.334	91900	4.48	6668
6		2.802	42520	2.07	1990
7		4.948	4350	0.21	672
8		5.513	2362	0.12	570
9		5.928	1522	0.07	252
10		6.200	1607	0.08	293
11		6.270	5296	0.26	443
12		7.447	6675	0.33	1402

Reported by User: Saneesh (Saneesh) Project Name: HDI_Stellenbosch_2016

	Peak Name	RT	Area	% Area	Height
13	E8H	7.607	20894	1.02	5519
14	EFV	8.148	253776	12.36	67678
15	NEO	8.769	580988	28.30	151654
16		9.266	2631	0.13	328
17		9.648	1891	0.09	314

SAMPLE INFORMATION

Sample Name:	EFV Inc Basil_Aq 200mig/mL 1	Acquired By:	Saneesh
Sample Type:	Unknown	Date Acquired:	2017/09/06 01:38:57 AM
Vial:	17	Acq. Method Set:	HDI_EFV_E8H_NEO
Injection #:	1	Date Processed:	2017/09/08 05:07:50 PM
Injection Volume:	15.00 ul	Processing Method:	HDI_EFV_ W2996 245.0nm-1.2
Run Time:	10.5 Minutes	Channel Name:	W2996 245.0nm-1.2
Sample Set Name:	EFV_Basil Aq IC50 5_9_17	Proc. Chnl. Descr.:	W2996 PDA 245.0 nm at 1.2

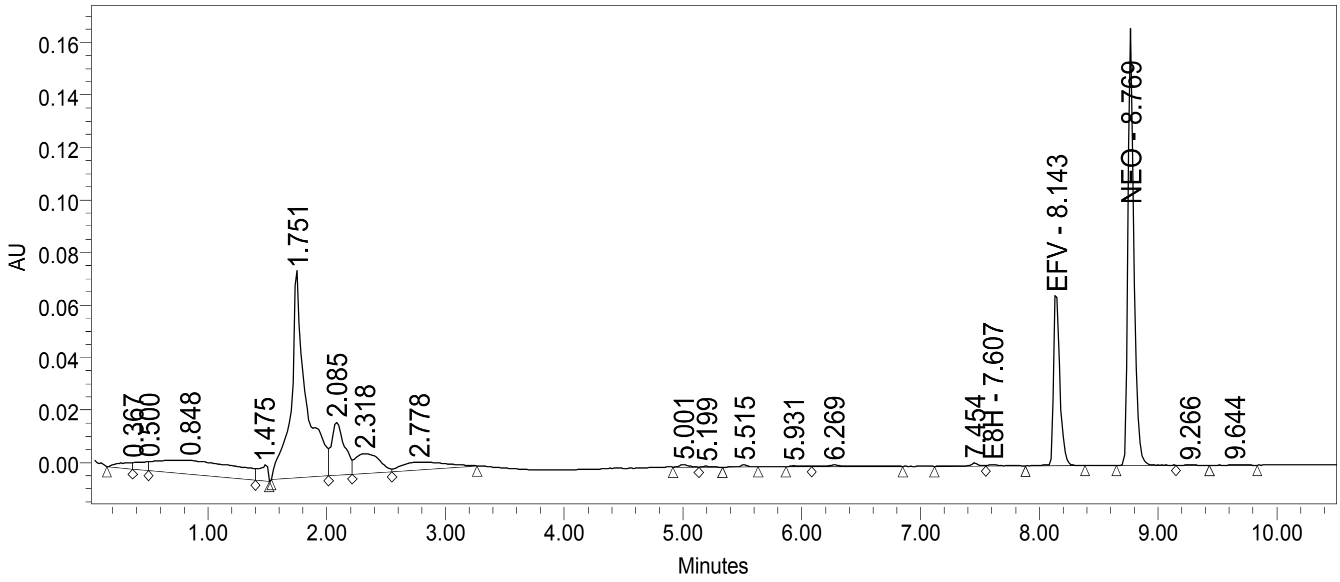

	Peak Name	RT	Area	% Area	Height
1		0.367	19732	0.88	2387
2		0.500	23063	1.03	3326
3		0.848	249695	11.19	5131
4		1.475	32335	1.45	6361
5		1.751	696083	31.19	76553
6		2.085	156255	7.00	20237
7		2.318	105185	4.71	7484
8		2.778	76276	3.42	2923
9		5.001	5382	0.24	1001
10		5.199	2327	0.10	357
11		5.515	4912	0.22	847
12		5.931	1828	0.08	279

Reported by User: Saneesh (Saneesh) Project Name: HDI_Stellenbosch_2016

	Peak Name	RT	Area	% Area	Height
13		6.269	9213	0.41	569
14		7.454	6942	0.31	1281
15	E8H	7.607	3830	0.17	608
16	EFV	8.143	239374	10.73	61599
17	NEO	8.769	595221	26.67	155851
18		9.266	1827	0.08	274
19		9.644	2297	0.10	271

Reported by User: Saneesh (Saneesh)   Project Name: HDI_Stellenbosch_2016

SAMPLE INFORMATION

Sample Name:	EFV Inc Basil_Aq 200mig/mL 2	Acquired By:	Saneesh
Sample Type:	Unknown	Date Acquired:	2017/09/06 01:52:52 AM
Vial:	18	Acq. Method Set:	HDI_EFV_E8H_NEO
Injection #:	1	Date Processed:	2017/09/08 05:07:50 PM
Injection Volume:	15.00 ul	Processing Method:	HDI_EFV_ W2996 245.0nm-1.2
Run Time:	10.5 Minutes	Channel Name:	W2996 245.0nm-1.2
Sample Set Name:	EFV_Basil Aq IC50 5_9_17	Proc. Chnl. Descr.:	W2996 PDA 245.0 nm at 1.2

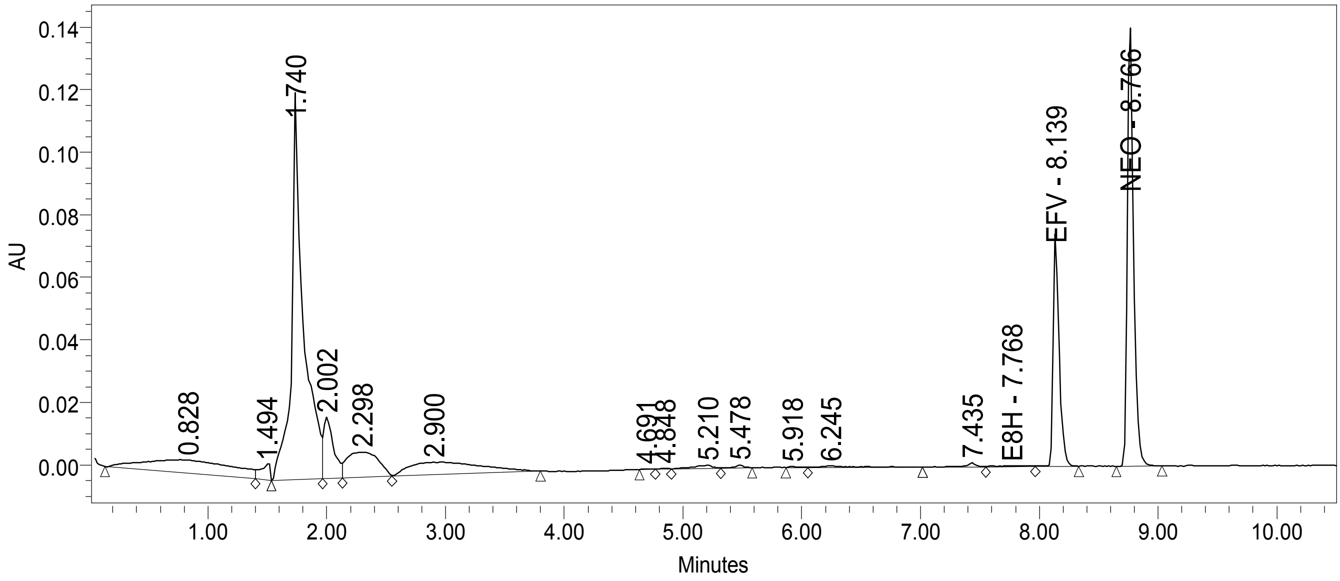

	Peak Name	RT	Area	% Area	Height
1		0.828	229459	9.62	4196
2		1.494	28562	1.20	5401
3		1.740	865552	36.30	112825
4		2.002	113480	4.76	19265
5		2.298	146087	6.13	7957
6		2.900	165849	6.95	3954
7		4.691	1054	0.04	175
8		4.848	1983	0.08	370
9		5.210	13022	0.55	1012
10		5.478	5944	0.25	936
11		5.918	1945	0.08	314
12		6.245	10096	0.42	497

	Peak Name	RT	Area	% Area	Height
13		7.435	9463	0.40	1249
14	E8H	7.768	5883	0.25	313
15	EFV	8.139	258216	10.83	68459
16	NEO	8.766	528019	22.14	134720

SAMPLE INFORMATION

Sample Name:	EFV Inc Basil_Aq 200mig/mL 3	Acquired By:	Saneesh
Sample Type:	Unknown	Date Acquired:	2017/09/06 02:06:38 AM
Vial:	19	Acq. Method Set:	HDI_EFV_E8H_NEO
Injection #:	1	Date Processed:	2017/09/08 05:07:50 PM
Injection Volume:	15.00 ul	Processing Method:	HDI_EFV_ W2996 245.0nm-1.2
Run Time:	10.5 Minutes	Channel Name:	W2996 245.0nm-1.2
Sample Set Name:	EFV_Basil Aq IC50 5_9_17	Proc. Chnl. Descr.:	W2996 PDA 245.0 nm at 1.2

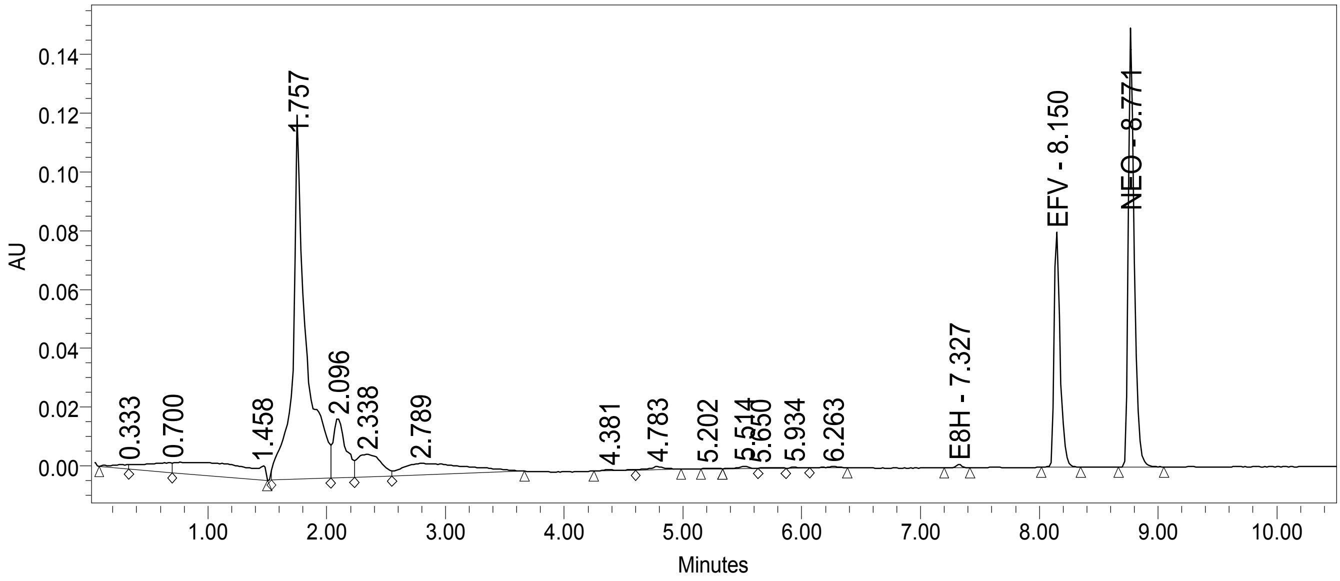

	Peak Name	RT	Area	% Area	Height
1		0.333	13040	0.53	1465
2		0.700	52643	2.13	3292
3		1.458	192769	7.79	5231
4		1.757	962399	38.87	113906
5		2.096	152127	6.14	20146
6		2.338	107714	4.35	7638
7		2.789	148256	5.99	3892
8		4.381	3895	0.16	363
9		4.783	9647	0.39	1079
10		5.202	2028	0.08	312
11		5.514	5211	0.21	793
12		5.650	2245	0.09	222

Reported by User: Saneesh (Saneesh)   Project Name: HDI_Stellenbosch_2016

	Peak Name	RT	Area	% Area	Height
13		5.934	2375	0.10	394
14		6.263	3323	0.13	455
15	E8H	7.327	4301	0.17	1029
16	EFV	8.150	278317	11.24	75852
17	NEO	8.771	535802	21.64	139858

Multi Sample Summary

Reported by User: Saneesh (Saneesh)   Project Name: HDI_Stellenbosch_2016

SAMPLE INFORMATION

Sample Name:	EFV Test Control 1	Acquired By:	Saneesh
Sample Type:	Unknown	Date Acquired:	2017/09/05 04:16:07 PM
Vial:	23	Acq. Method Set:	HDI_EFV_E8H_NEO
Injection #:	1	Date Processed:	2017/09/08 05:06:27 PM
Injection Volume:	15.00 ul	Processing Method:	HDI_EFV_ W2996 245.0nm-1.2
Run Time:	10.5 Minutes	Channel Name:	W2996 245.0nm-1.2
Sample Set Name:	EFV_Basil MeOH IC50 5_9_17	Proc. Chnl. Descr.:	W2996 PDA 245.0 nm at 1.2

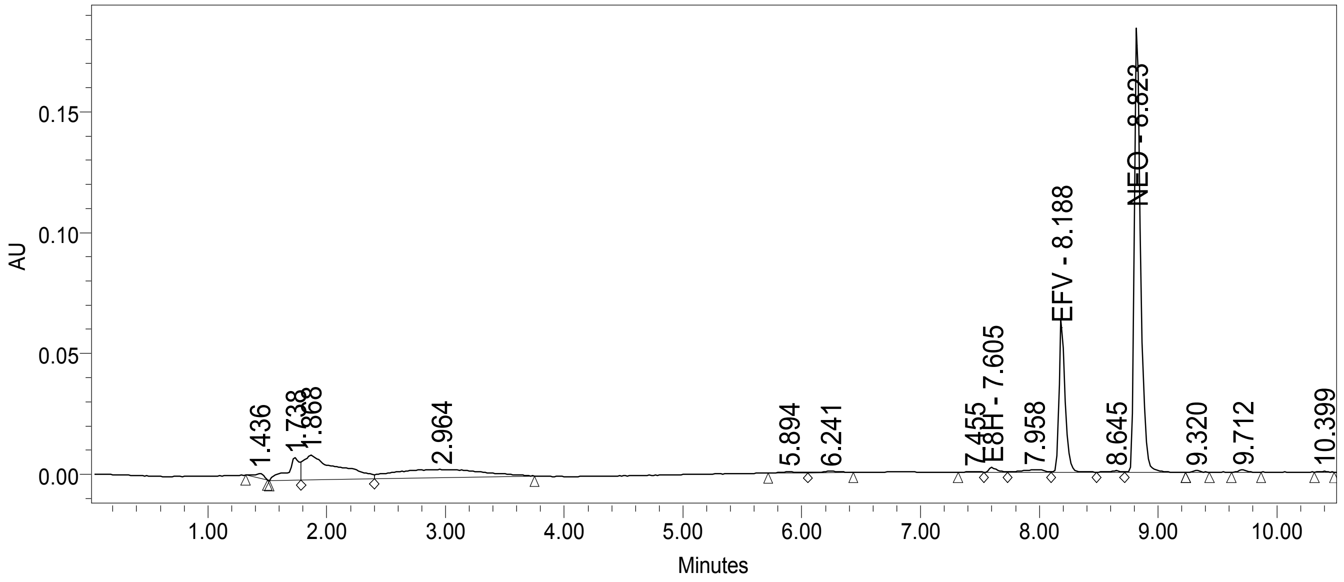

	Peak Name	RT	Area	% Area	Height
1		1.436	11547	0.81	2037
2		1.738	68308	4.79	9228
3		1.868	204579	14.34	10057
4		2.964	178888	12.54	3339
5		5.894	4744	0.33	454
6		6.241	5709	0.40	549
7		7.455	3007	0.21	383
8	E8H	7.605	10352	0.73	2047
9		7.958	15188	1.06	1128
10	EFV	8.188	222199	15.58	58754
11		8.645	4613	0.32	663
12	NEO	8.823	688141	48.24	174725
13		9.320	3015	0.21	694
14		9.712	4912	0.34	978

Reported by User: Saneesh (Saneesh)   Project Name: HDI_Stellenbosch_2016

	Peak Name	RT	Area	% Area	Height
15		10.399	1248	0.09	287

Reported by User: Saneesh (Saneesh)   Project Name: HDI_Stellenbosch_2016

SAMPLE INFORMATION

Sample Name:	EFV Test Control 2	Acquired By:	Saneesh
Sample Type:	Unknown	Date Acquired:	2017/09/05 04:30:25 PM
Vial:	24	Acq. Method Set:	HDI_EFV_E8H_NEO
Injection #:	1	Date Processed:	2017/09/08 05:06:27 PM
Injection Volume:	15.00 ul	Processing Method:	HDI_EFV_ W2996 245.0nm-1.2
Run Time:	10.5 Minutes	Channel Name:	W2996 245.0nm-1.2
Sample Set Name:	EFV_Basil MeOH IC50 5_9_17	Proc. Chnl. Descr.:	W2996 PDA 245.0 nm at 1.2

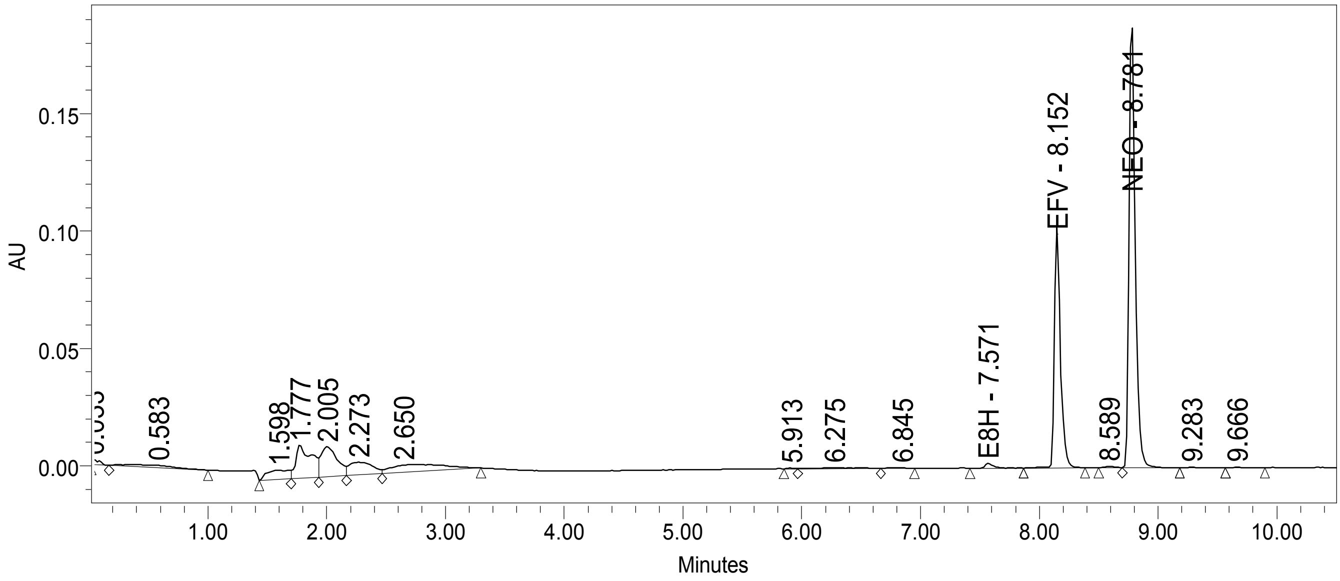

	Peak Name	RT	Area	% Area	Height
1		0.033	9457	0.59	2199
2		0.583	29513	1.84	1007
3		1.598	48995	3.05	3753
4		1.777	128544	8.01	13979
5		2.005	120989	7.54	12697
6		2.273	73346	4.57	5330
7		2.650	95994	5.98	3047
8		5.913	1410	0.09	292
9		6.275	9547	0.60	469
10		6.845	3744	0.23	353
11	E8H	7.571	10036	0.63	2076
12	EFV	8.152	351839	21.93	95568

Reported by User: Saneesh (Saneesh)   Project Name: HDI_Stellenbosch_2016

	Peak Name	RT	Area	% Area	Height
13		8.589	3462	0.22	603
14	NEO	8.781	713493	44.48	181941
15		9.283	1804	0.11	280
16		9.666	1863	0.12	290

Reported by User: Saneesh (Saneesh) Project Name: HDI_Stellenbosch_2016

SAMPLE INFORMATION

Sample Name:	EFV Inc Basil_MeOH 1miG/mL 1	Acquired By:	Saneesh
Sample Type:	Unknown	Date Acquired:	2017/09/05 04:44:44 PM
Vial:	2	Acq. Method Set:	HDI_EFV_E8H_NEO
Injection #:	1	Date Processed:	2017/09/08 05:06:27 PM
Injection Volume:	15.00 ul	Processing Method:	HDI_EFV_ W2996 245.0nm-1.2
Run Time:	10.5 Minutes	Channel Name:	W2996 245.0nm-1.2
Sample Set Name:	EFV_Basil MeOH IC50 5_9_17	Proc. Chnl. Descr.:	W2996 PDA 245.0 nm at 1.2

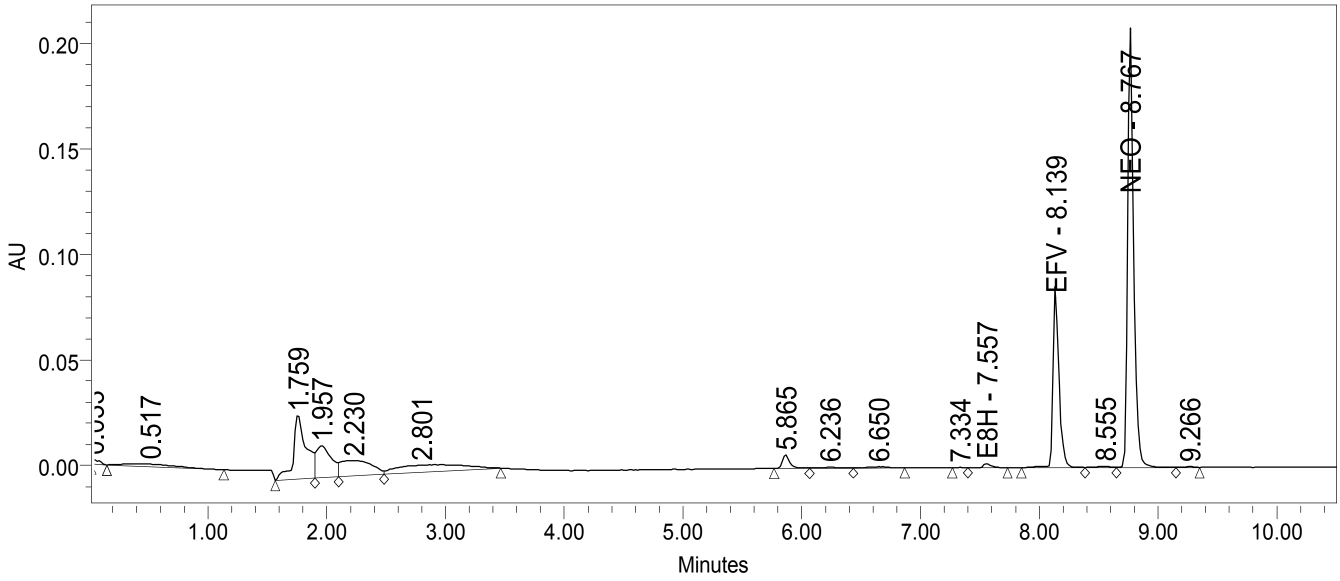

	Peak Name	RT	Area	% Area	Height
1		0.033	9149	0.51	2134
2		0.517	39981	2.23	1307
3		1.759	233276	12.99	30944
4		1.957	136056	7.58	15093
5		2.230	132765	7.39	7226
6		2.801	135842	7.56	3445
7		5.865	29161	1.62	6204
8		6.236	7206	0.40	580
9		6.650	7347	0.41	565
10		7.334	922	0.05	268
11	E8H	7.557	9751	0.54	1914
12	EFV	8.139	297446	16.56	78607

Reported by User: Saneesh (Saneesh)   Project Name: HDI_Stellenbosch_2016

	Peak Name	RT	Area	% Area	Height
13		8.555	5346	0.30	621
14	NEO	8.767	749079	41.71	196401
15		9.266	2574	0.14	436

Reported by User: Saneesh (Saneesh)   Project Name: HDI_Stellenbosch_2016

SAMPLE INFORMATION

Sample Name:	EFV Inc Basil_MeOH 1miG/mL 2	Acquired By:	Saneesh
Sample Type:	Unknown	Date Acquired:	2017/09/05 04:59:00 PM
Vial:	3	Acq. Method Set:	HDI_EFV_E8H_NEO
Injection #:	1	Date Processed:	2017/09/08 05:06:27 PM
Injection Volume:	15.00 ul	Processing Method:	HDI_EFV_ W2996 245.0nm-1.2
Run Time:	10.5 Minutes	Channel Name:	W2996 245.0nm-1.2
Sample Set Name:	EFV_Basil MeOH IC50 5_9_17	Proc. Chnl. Descr.:	W2996 PDA 245.0 nm at 1.2

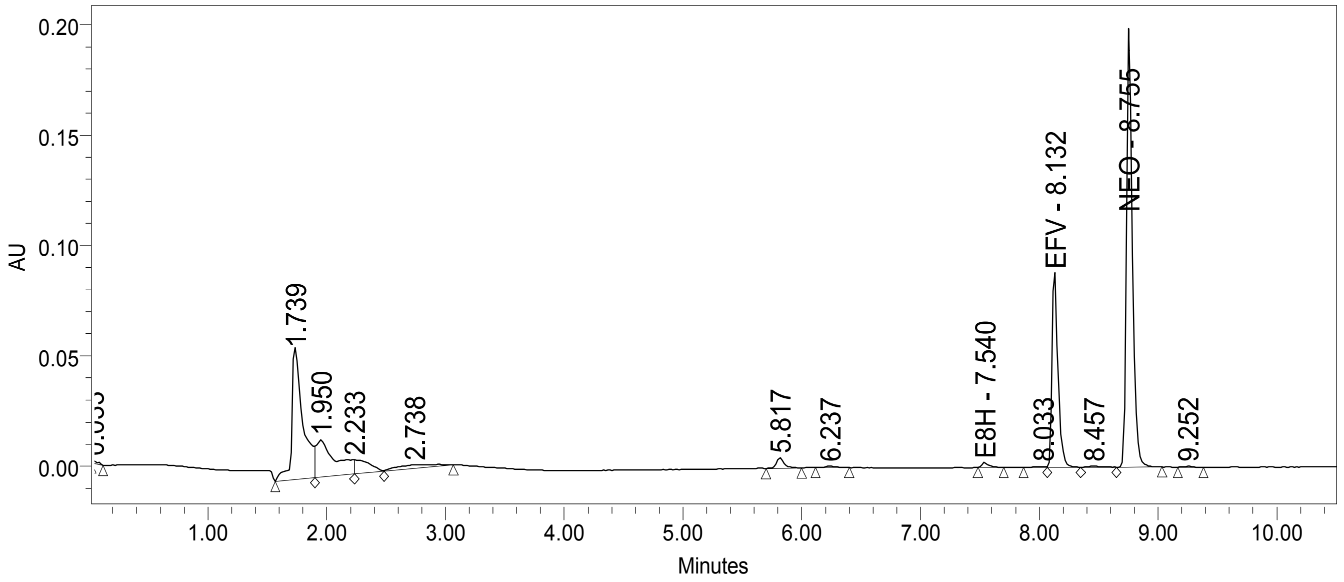

	Peak Name	RT	Area	% Area	Height
1		0.033	3789	0.22	1187
2		1.739	394943	22.69	62476
3		1.950	193055	11.09	16520
4		2.233	55540	3.19	6387
5		2.738	37082	2.13	1511
6		5.817	20361	1.17	4448
7		6.237	4146	0.24	605
8	E8H	7.540	10008	0.57	2190
9		8.033	3200	0.18	432
10	EFV	8.132	310714	17.85	84557
11		8.457	4568	0.26	519
12	NEO	8.755	701519	40.30	185456

	Peak Name	RT	Area	% Area	Height
13		9.252	1977	0.11	440

SAMPLE INFORMATION

Sample Name:	EFV Inc Basil_MeOH 1miG/mL 3	Acquired By:	Saneesh
Sample Type:	Unknown	Date Acquired:	2017/09/05 05:12:57 PM
Vial:	4	Acq. Method Set:	HDI_EFV_E8H_NEO
Injection #:	1	Date Processed:	2017/09/08 05:06:27 PM
Injection Volume:	15.00 ul	Processing Method:	HDI_EFV_ W2996 245.0nm-1.2
Run Time:	10.5 Minutes	Channel Name:	W2996 245.0nm-1.2
Sample Set Name:	EFV_Basil MeOH IC50 5_9_17	Proc. Chnl. Descr.:	W2996 PDA 245.0 nm at 1.2

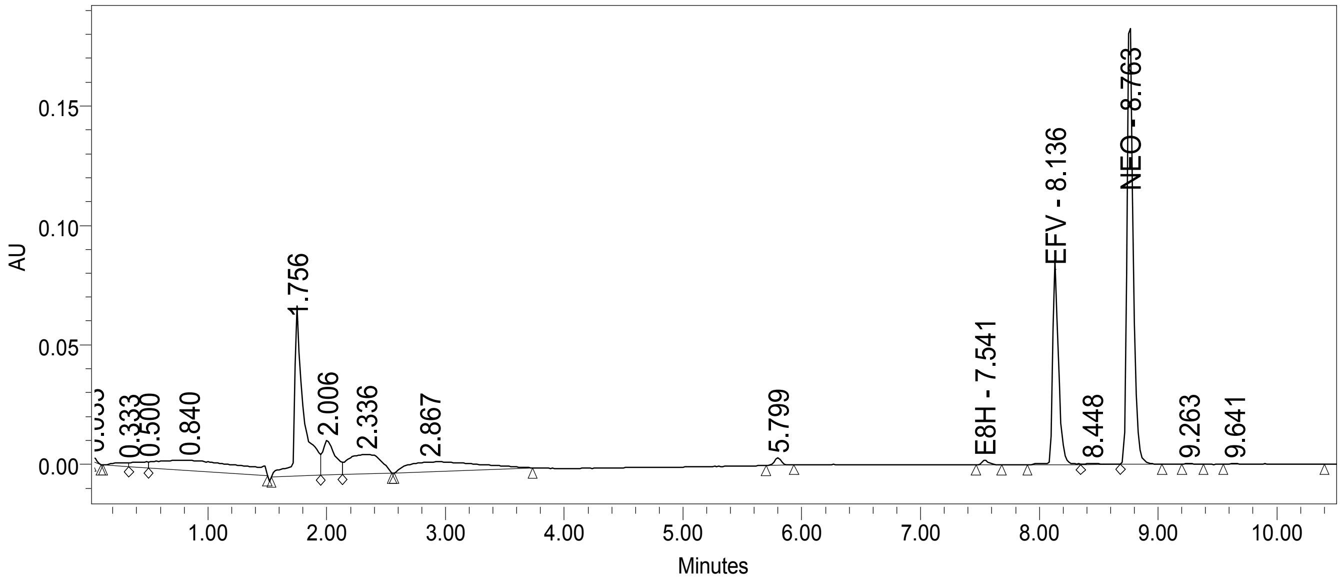

	Peak Name	RT	Area	% Area	Height
1		0.033	4449	0.21	1800
2		0.333	13431	0.65	1660
3		0.500	21496	1.03	2538
4		0.840	220020	10.58	4240
5		1.756	389541	18.73	65539
6		2.006	105780	5.09	14261
7		2.336	155162	7.46	8106
8		2.867	165861	7.97	3985
9		5.799	11431	0.55	2865
10	E8H	7.541	7852	0.38	1817
11	EFV	8.136	287996	13.85	78838
12		8.448	4802	0.23	420

	Peak Name	RT	Area	% Area	Height
13	NEO	8.763	685226	32.95	178328
14		9.263	1578	0.08	372
15		9.641	5255	0.25	368

SAMPLE INFORMATION

Sample Name:	EFV Inc Basil_MeOH 10miG/mL 1	Acquired By:	Saneesh
Sample Type:	Unknown	Date Acquired:	2017/09/05 05:26:51 PM
Vial:	5	Acq. Method Set:	HDI_EFV_E8H_NEO
Injection #:	1	Date Processed:	2017/09/08 05:06:27 PM
Injection Volume:	15.00 ul	Processing Method:	HDI_EFV_ W2996 245.0nm-1.2
Run Time:	10.5 Minutes	Channel Name:	W2996 245.0nm-1.2
Sample Set Name:	EFV_Basil MeOH IC50 5_9_17	Proc. Chnl. Descr.:	W2996 PDA 245.0 nm at 1.2

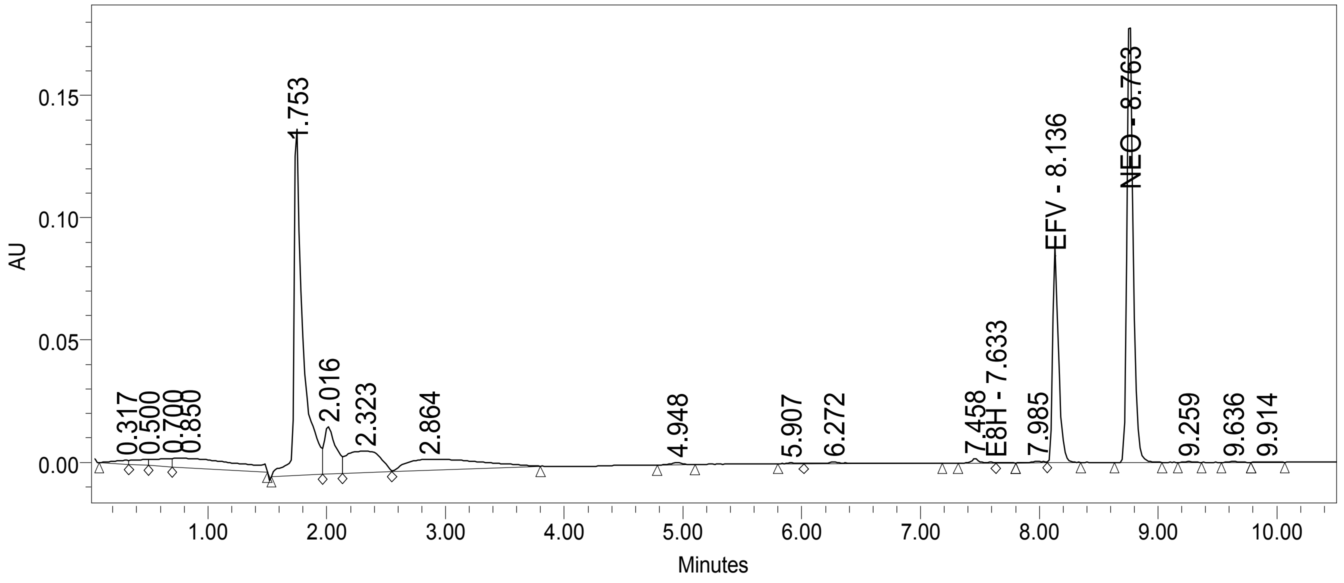

	Peak Name	RT	Area	% Area	Height
1		0.317	14110	0.56	1654
2		0.500	20487	0.81	2399
3		0.700	35038	1.39	3381
4		0.850	155083	6.15	3827
5		1.753	766387	30.41	140044
6		2.016	130770	5.19	19276
7		2.323	175102	6.95	8876
8		2.864	197466	7.83	4464
9		4.948	7080	0.28	971
10		5.907	2016	0.08	343
11		6.272	12736	0.51	722
12		7.458	9809	0.39	1710

	Peak Name	RT	Area	% Area	Height
13	E8H	7.633	812	0.03	160
14		7.985	4564	0.18	589
15	EFV	8.136	299267	11.87	81893
16	NEO	8.763	683076	27.10	174348
17		9.259	2003	0.08	437
18		9.636	2424	0.10	375
19		9.914	2244	0.09	261

SAMPLE INFORMATION

Sample Name:	EFV Inc Basil_MeOH 10miG/mL 2	Acquired By:	Saneesh
Sample Type:	Unknown	Date Acquired:	2017/09/05 05:40:37 PM
Vial:	6	Acq. Method Set:	HDI_EFV_E8H_NEO
Injection #:	1	Date Processed:	2017/09/08 05:06:27 PM
Injection Volume:	15.00 ul	Processing Method:	HDI_EFV_ W2996 245.0nm-1.2
Run Time:	10.5 Minutes	Channel Name:	W2996 245.0nm-1.2
Sample Set Name:	EFV_Basil MeOH IC50 5_9_17	Proc. Chnl. Descr.:	W2996 PDA 245.0 nm at 1.2

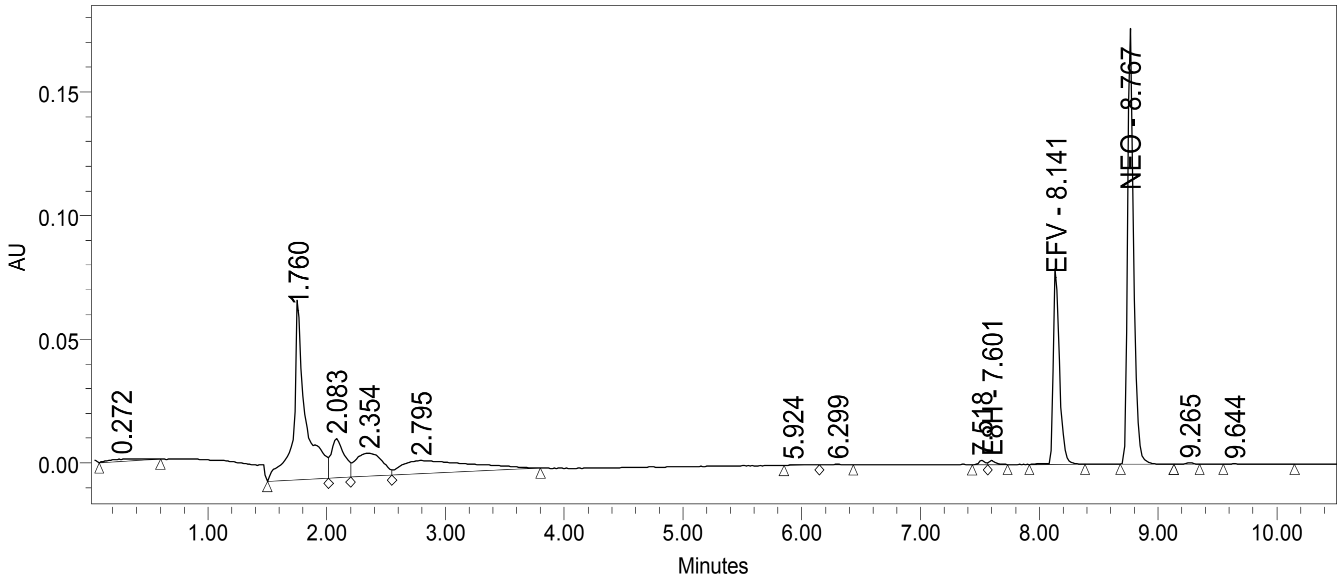

	Peak Name	RT	Area	% Area	Height
1		0.272	16208	0.82	834
2		1.760	519247	26.32	64722
3		2.083	122173	6.19	15743
4		2.354	148092	7.51	9305
5		2.795	219381	11.12	5355
6		5.924	2544	0.13	372
7		6.299	2792	0.14	440
8		7.518	6482	0.33	1701
9	E8H	7.601	5958	0.30	1475
10	EFV	8.141	284106	14.40	73649
11	NEO	8.767	640435	32.46	167071
12		9.265	3013	0.15	636

Reported by User: Saneesh (Saneesh)   Project Name: HDI_Stellenbosch_2016

	Peak Name	RT	Area	% Area	Height
13		9.644	2674	0.14	257

SAMPLE INFORMATION

Sample Name:	EFV Inc Basil_MeOH 10miG/mL 3	Acquired By:	Saneesh
Sample Type:	Unknown	Date Acquired:	2017/09/05 05:54:32 PM
Vial:	7	Acq. Method Set:	HDI_EFV_E8H_NEO
Injection #:	1	Date Processed:	2017/09/08 05:06:27 PM
Injection Volume:	15.00 ul	Processing Method:	HDI_EFV_ W2996 245.0nm-1.2
Run Time:	10.5 Minutes	Channel Name:	W2996 245.0nm-1.2
Sample Set Name:	EFV_Basil MeOH IC50 5_9_17	Proc. Chnl. Descr.:	W2996 PDA 245.0 nm at 1.2

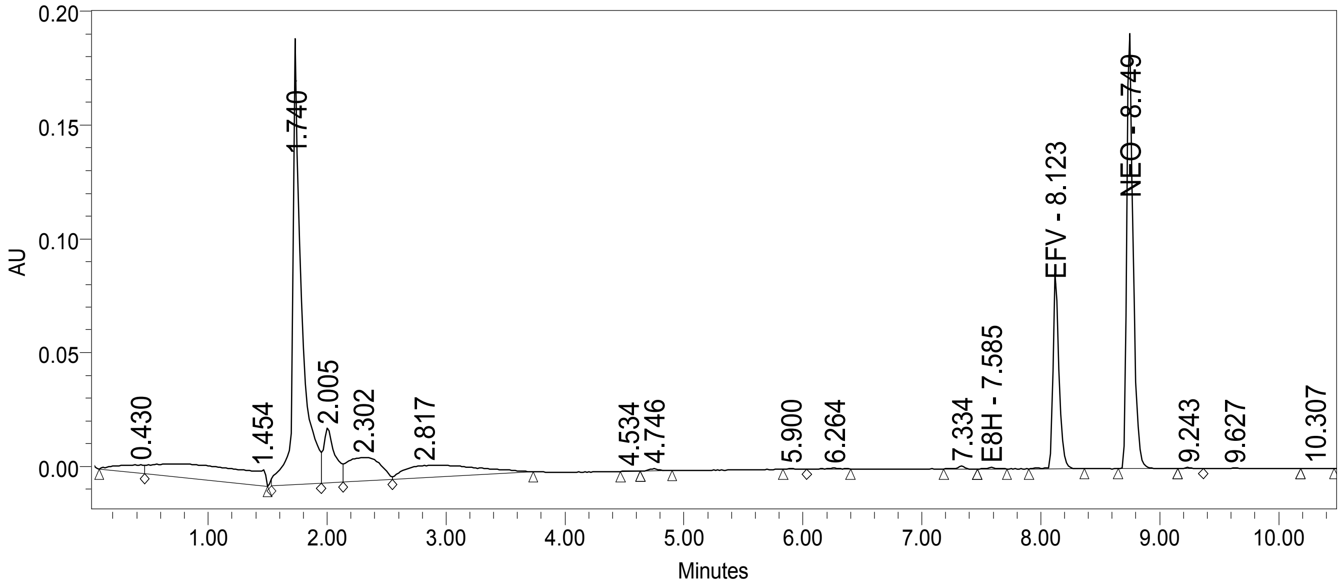

	Peak Name	RT	Area	% Area	Height
1		0.430	55134	1.80	3665
2		1.454	354542	11.61	7303
3		1.740	1009455	33.05	174380
4		2.005	165567	5.42	23770
5		2.302	200606	6.57	10289
6		2.817	224926	7.36	5298
7		4.534	827	0.03	194
8		4.746	6786	0.22	1080
9		5.900	1596	0.05	365
10		6.264	3124	0.10	450
11		7.334	4620	0.15	1138
12	E8H	7.585	2927	0.10	629

Reported by User: Saneesh (Saneesh) Project Name: HDI_Stellenbosch_2016

	Peak Name	RT	Area	% Area	Height
13	EFV	8.123	302554	9.90	79606
14	NEO	8.749	713105	23.35	183177
15		9.243	2368	0.08	490
16		9.627	4965	0.16	350
17		10.307	1468	0.05	206

Reported by User: Saneesh (Saneesh)   Project Name: HDI_Stellenbosch_2016

SAMPLE INFORMATION

Sample Name:	EFV Inc Basil_MeOH 50miG/mL 1	Acquired By:	Saneesh
Sample Type:	Unknown	Date Acquired:	2017/09/05 06:08:29 PM
Vial:	8	Acq. Method Set:	HDI_EFV_E8H_NEO
Injection #:	1	Date Processed:	2017/09/08 05:06:27 PM
Injection Volume:	15.00 ul	Processing Method:	HDI_EFV_ W2996 245.0nm-1.2
Run Time:	10.5 Minutes	Channel Name:	W2996 245.0nm-1.2
Sample Set Name:	EFV_Basil MeOH IC50 5_9_17	Proc. Chnl. Descr.:	W2996 PDA 245.0 nm at 1.2

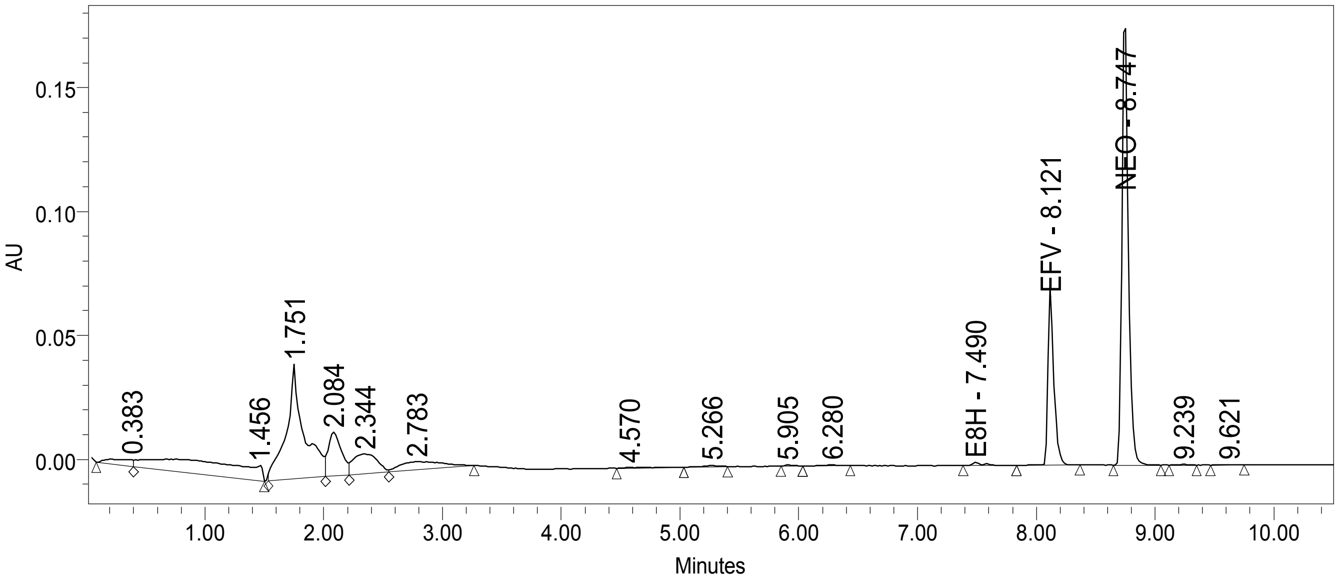

	Peak Name	RT	Area	% Area	Height
1		0.383	33868	1.61	2731
2		1.456	306376	14.55	6573
3		1.751	478404	22.72	42615
4		2.084	138192	6.56	17627
5		2.344	112814	5.36	7970
6		2.783	82802	3.93	3272
7		4.570	4086	0.19	307
8		5.266	4561	0.22	567
9		5.905	1941	0.09	428
10		6.280	3681	0.17	495
11	E8H	7.490	8354	0.40	1291
12	EFV	8.121	249063	11.83	66406

	Peak Name	RT	Area	% Area	Height
13	NEO	8.747	677603	32.19	172653
14		9.239	2052	0.10	388
15		9.621	1538	0.07	237

SAMPLE INFORMATION

Sample Name:	EFV Inc Basil_MeOH 50miG/mL 2	Acquired By:	Saneesh
Sample Type:	Unknown	Date Acquired:	2017/09/05 06:22:25 PM
Vial:	9	Acq. Method Set:	HDI_EFV_E8H_NEO
Injection #:	1	Date Processed:	2017/09/08 05:06:28 PM
Injection Volume:	15.00 ul	Processing Method:	HDI_EFV_ W2996 245.0nm-1.2
Run Time:	10.5 Minutes	Channel Name:	W2996 245.0nm-1.2
Sample Set Name:	EFV_Basil MeOH IC50 5_9_17	Proc. Chnl. Descr.:	W2996 PDA 245.0 nm at 1.2

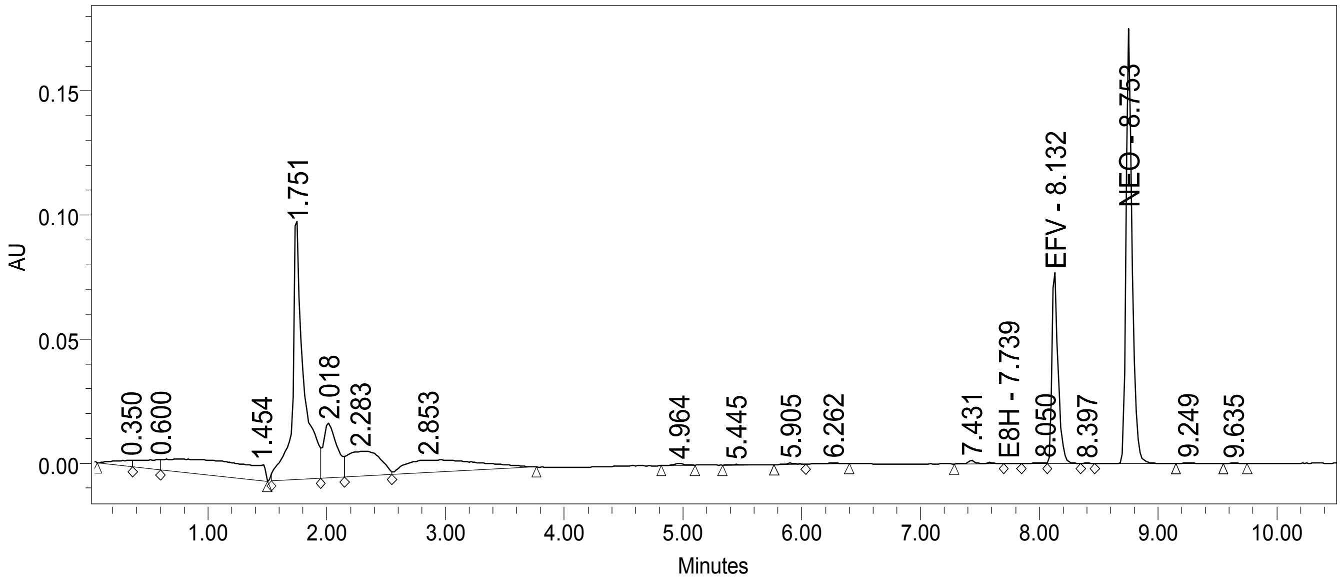

	Peak Name	RT	Area	% Area	Height
1		0.350	25574	0.99	2464
2		0.600	45662	1.76	4016
3		1.454	297736	11.48	6906
4		1.751	692561	26.70	103174
5		2.018	177145	6.83	21822
6		2.283	189715	7.31	9915
7		2.853	220028	8.48	5037
8		4.964	4876	0.19	793
9		5.445	3387	0.13	280
10		5.905	3163	0.12	542
11		6.262	4316	0.17	462
12		7.431	8510	0.33	1193

Reported by User: Saneesh (Saneesh)   Project Name: HDI_Stellenbosch_2016

	Peak Name	RT	Area	% Area	Height
13	E8H	7.739	826	0.03	131
14		8.050	3171	0.12	445
15	EFV	8.132	279093	10.76	74213
16		8.397	1040	0.04	222
17	NEO	8.753	633484	24.42	164352
18		9.249	2248	0.09	318
19		9.635	1322	0.05	245

Reported by User: Saneesh (Saneesh) Project Name: HDI_Stellenbosch_2016

SAMPLE INFORMATION

Sample Name:	EFV IncBasil_MeOH 50mig/mL 3	Acquired By:	Saneesh
Sample Type:	Unknown	Date Acquired:	2017/09/05 06:36:20 PM
Vial:	10	Acq. Method Set:	HDI_EFV_E8H_NEO
Injection #:	1	Date Processed:	2017/09/08 05:06:28 PM
Injection Volume:	15.00 ul	Processing Method:	HDI_EFV_ W2996 245.0nm-1.2
Run Time:	10.5 Minutes	Channel Name:	W2996 245.0nm-1.2
Sample Set Name:	EFV_Basil MeOH IC50 5_9_17	Proc. Chnl. Descr.:	W2996 PDA 245.0 nm at 1.2

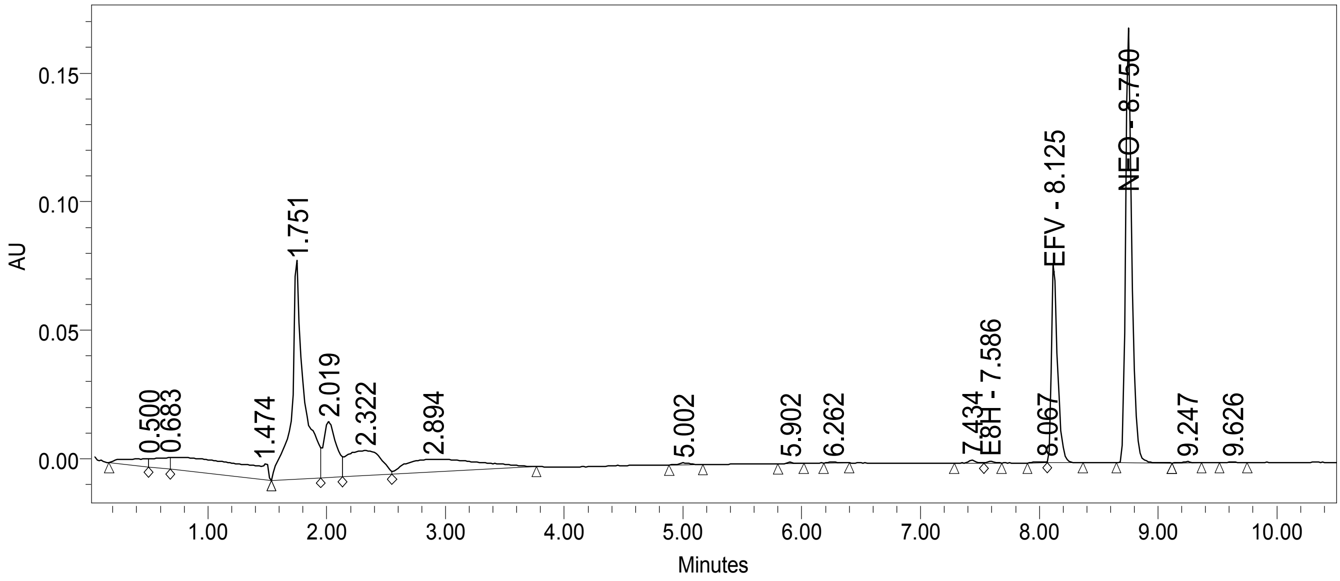

	Peak Name	RT	Area	% Area	Height
1		0.500	39700	1.62	3125
2		0.683	42089	1.72	4445
3		1.474	253816	10.36	6428
4		1.751	621077	25.36	82326
5		2.019	161623	6.60	21728
6		2.322	190007	7.76	9590
7		2.894	213914	8.74	4887
8		5.002	5197	0.21	751
9		5.902	2159	0.09	517
10		6.262	1847	0.08	393
11		7.434	5116	0.21	1157
12	E8H	7.586	2956	0.12	675

	Peak Name	RT	Area	% Area	Height
13		8.067	2687	0.11	400
14	EFV	8.125	277873	11.35	72465
15	NEO	8.750	624567	25.50	160646
16		9.247	2270	0.09	460
17		9.626	1907	0.08	350

Multi Sample Summary

Reported by User: Saneesh (Saneesh) Project Name: HDI_Stellenbosch_2016

SAMPLE INFORMATION

Sample Name:	EFV Inc Basil_MeOH 100mig/mL 1	Acquired By:	Saneesh
Sample Type:	Unknown	Date Acquired:	2017/09/05 06:50:16 PM
Vial:	11	Acq. Method Set:	HDI_EFV_E8H_NEO
Injection #:	1	Date Processed:	2017/09/08 05:06:28 PM
Injection Volume:	15.00 ul	Processing Method:	HDI_EFV_ W2996 245.0nm-1.2
Run Time:	10.5 Minutes	Channel Name:	W2996 245.0nm-1.2
Sample Set Name:	EFV_Basil MeOH IC50 5_9_17	Proc. Chnl. Descr.:	W2996 PDA 245.0 nm at 1.2

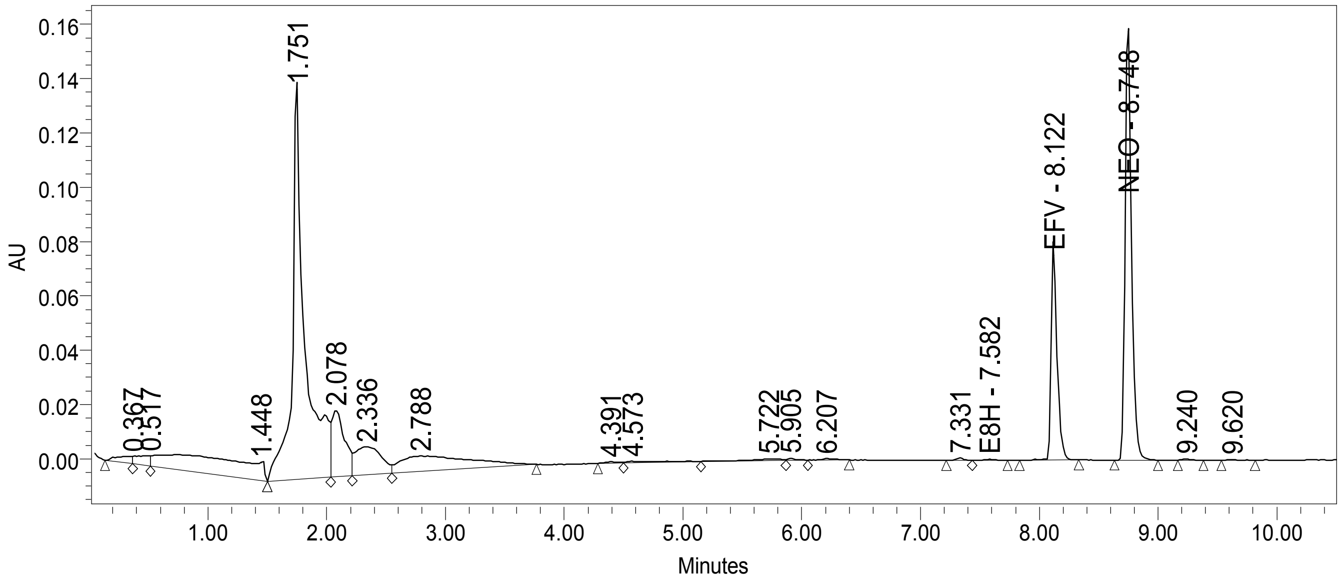

	Peak Name	RT	Area	% Area	Height
1		0.367	22563	0.76	2689
2		0.517	29018	0.98	3686
3		1.448	325461	10.97	6827
4		1.751	1075325	36.25	141014
5		2.078	189032	6.37	24544
6		2.336	158880	5.36	10267
7		2.788	233561	7.87	5684
8		4.391	3465	0.12	449
9		4.573	12535	0.42	524
10		5.722	15046	0.51	737
11		5.905	4227	0.14	817
12		6.207	4728	0.16	563

	Peak Name	RT	Area	% Area	Height
13		7.331	3644	0.12	805
14	E8H	7.582	2954	0.10	445
15	EFV	8.122	276780	9.33	73937
16	NEO	8.748	605022	20.39	154137
17		9.240	2420	0.08	531
18		9.620	1963	0.07	296

SAMPLE INFORMATION

Sample Name:	EFV Inc Basil_MeOH 100mig/mL 2	Acquired By:	Saneesh
Sample Type:	Unknown	Date Acquired:	2017/09/05 07:04:11 PM
Vial:	12	Acq. Method Set:	HDI_EFV_E8H_NEO
Injection #:	1	Date Processed:	2017/09/08 05:06:28 PM
Injection Volume:	15.00 ul	Processing Method:	HDI_EFV_ W2996 245.0nm-1.2
Run Time:	10.5 Minutes	Channel Name:	W2996 245.0nm-1.2
Sample Set Name:	EFV_Basil MeOH IC50 5_9_17	Proc. Chnl. Descr.:	W2996 PDA 245.0 nm at 1.2

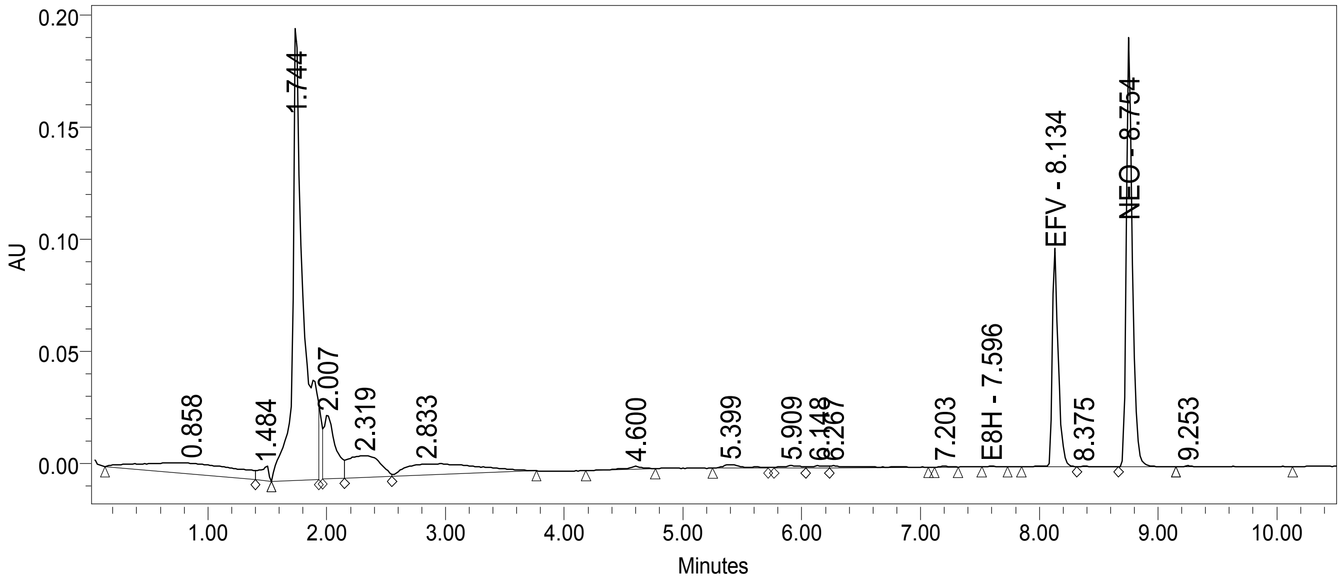

	Peak Name	RT	Area	% Area	Height
1		0.858	279087	8.47	4875
2		1.484	34614	1.05	6013
3		1.744	1295657	39.32	187667
4		2.007	195143	5.92	27621
5		2.319	180608	5.48	9669
6		2.833	210927	6.40	4963
7		4.600	13069	0.40	1255
8		5.399	17920	0.54	1597
9		5.909	9545	0.29	1076
10		6.148	7577	0.23	843
11		6.267	14177	0.43	873
12		7.203	2559	0.08	533

Reported by User: Saneesh (Saneesh) Project Name: HDI_Stellenbosch_2016

	Peak Name	RT	Area	% Area	Height
13	E8H	7.596	1830	0.06	384
14	EFV	8.134	336897	10.22	91333
15		8.375	3131	0.09	356
16	NEO	8.754	688225	20.88	179115
17		9.253	4592	0.14	432

SAMPLE INFORMATION

Sample Name:	EFV Inc Basil_MeOH 100mig/mL 3	Acquired By:	Saneesh
Sample Type:	Unknown	Date Acquired:	2017/09/05 07:18:05 PM
Vial:	13	Acq. Method Set:	HDI_EFV_E8H_NEO
Injection #:	1	Date Processed:	2017/09/08 05:06:28 PM
Injection Volume:	15.00 ul	Processing Method:	HDI_EFV_ W2996 245.0nm-1.2
Run Time:	10.5 Minutes	Channel Name:	W2996 245.0nm-1.2
Sample Set Name:	EFV_Basil MeOH IC50 5_9_17	Proc. Chnl. Descr.:	W2996 PDA 245.0 nm at 1.2

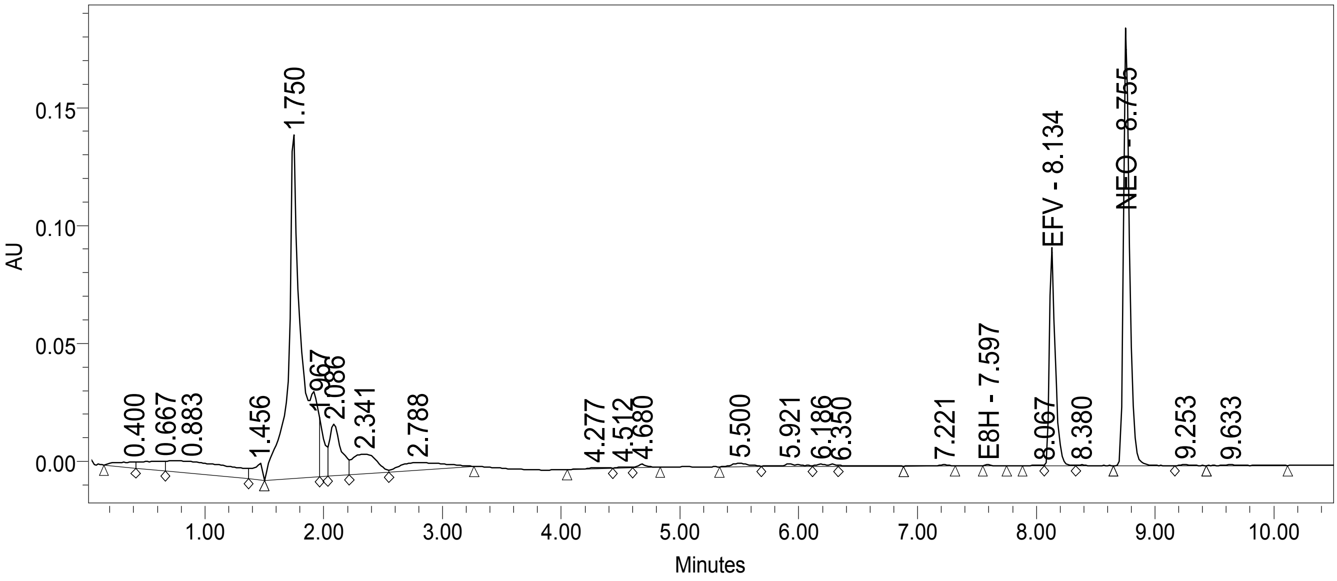

	Peak Name	RT	Area	% Area	Height
1		0.400	26049	0.88	2493
2		0.667	49867	1.69	4123
3		0.883	193676	6.55	4969
4		1.456	39457	1.33	7050
5		1.750	1161575	39.27	141166
6		1.967	67611	2.29	24907
7		2.086	151284	5.11	21885
8		2.341	117468	3.97	8305
9		2.788	81970	2.77	3213
10		4.277	7089	0.24	543
11		4.512	3493	0.12	470
12		4.680	6675	0.23	1299

Reported by User: Saneesh (Saneesh) Project Name: HDI_Stellenbosch_2016

	Peak Name	RT	Area	% Area	Height
13		5.500	15174	0.51	1534
14		5.921	12845	0.43	1178
15		6.186	9130	0.31	963
16		6.350	6849	0.23	432
17		7.221	4140	0.14	565
18	E8H	7.597	1478	0.05	365
19		8.067	1797	0.06	307
20	EFV	8.134	317756	10.74	86614
21		8.380	2869	0.10	374
22	NEO	8.755	672434	22.73	174133
23		9.253	2624	0.09	503
24		9.633	4924	0.17	462

SAMPLE INFORMATION

Sample Name:	EFV Inc Basil_MeOH 150mig/mL 1	Acquired By:	Saneesh
Sample Type:	Unknown	Date Acquired:	2017/09/05 07:32:02 PM
Vial:	14	Acq. Method Set:	HDI_EFV_E8H_NEO
Injection #:	1	Date Processed:	2017/09/08 05:06:28 PM
Injection Volume:	15.00 ul	Processing Method:	HDI_EFV_ W2996 245.0nm-1.2
Run Time:	10.5 Minutes	Channel Name:	W2996 245.0nm-1.2
Sample Set Name:	EFV_Basil MeOH IC50 5_9_17	Proc. Chnl. Descr.:	W2996 PDA 245.0 nm at 1.2

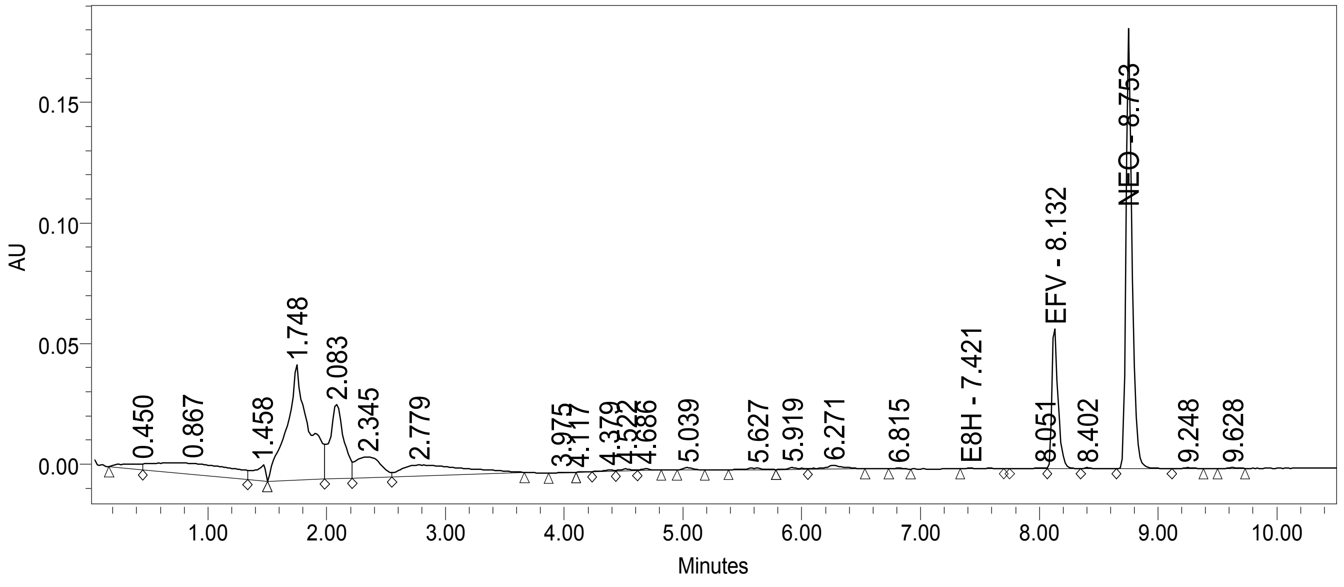

	Peak Name	RT	Area	% Area	Height
1		0.450	29115	1.22	2552
2		0.867	213409	8.94	4667
3		1.458	44609	1.87	6808
4		1.748	615565	25.79	46006
5		2.083	259737	10.88	31075
6		2.345	128601	5.39	8632
7		2.779	164734	6.90	4483
8		3.975	804	0.03	136
9		4.117	581	0.02	122
10		4.379	3740	0.16	573
11		4.522	5709	0.24	823
12		4.686	3609	0.15	605

Reported by User: Saneesh (Saneesh) Project Name: HDI_Stellenbosch_2016

	Peak Name	RT	Area	% Area	Height
13		5.039	4818	0.20	912
14		5.627	6257	0.26	633
15		5.919	5594	0.23	843
16		6.271	15556	0.65	1512
17		6.815	1563	0.07	344
18	E8H	7.421	3617	0.15	386
19		8.051	3299	0.14	372
20	EFV	8.132	211911	8.88	56148
21		8.402	2950	0.12	401
22	NEO	8.753	657204	27.53	171046
23		9.248	2156	0.09	366
24		9.628	1834	0.08	352

SAMPLE INFORMATION

Sample Name:	EFV Inc Basil_MeOH 150mig/mL 2	Acquired By:	Saneesh
Sample Type:	Unknown	Date Acquired:	2017/09/05 07:45:57 PM
Vial:	15	Acq. Method Set:	HDI_EFV_E8H_NEO
Injection #:	1	Date Processed:	2017/09/08 05:06:28 PM
Injection Volume:	15.00 ul	Processing Method:	HDI_EFV_ W2996 245.0nm-1.2
Run Time:	10.5 Minutes	Channel Name:	W2996 245.0nm-1.2
Sample Set Name:	EFV_Basil MeOH IC50 5_9_17	Proc. Chnl. Descr.:	W2996 PDA 245.0 nm at 1.2

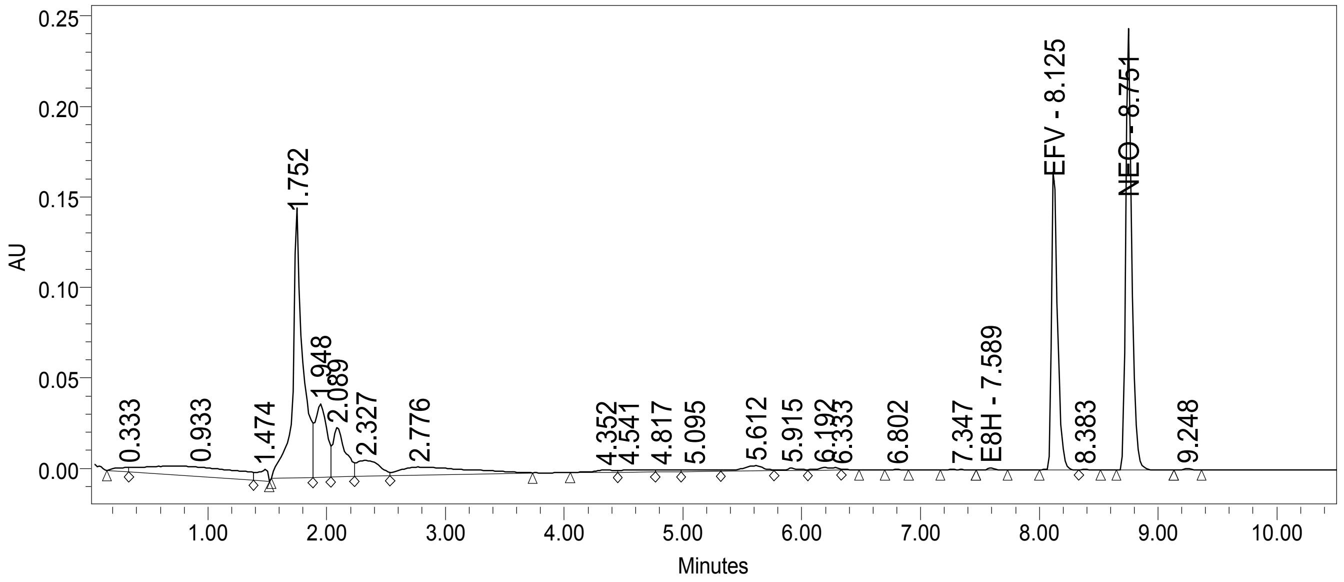

	Peak Name	RT	Area	% Area	Height
1		0.333	18034	0.50	2480
2		0.933	279747	7.73	5257
3		1.474	38263	1.06	6737
4		1.752	885694	24.49	140255
5		1.948	285256	7.89	39928
6		2.089	205431	5.68	27258
7		2.327	117456	3.25	8630
8		2.776	176224	4.87	4452
9		4.352	16370	0.45	1247
10		4.541	22090	0.61	1390
11		4.817	12493	0.35	1125
12		5.095	14976	0.41	967

Reported by User: Saneesh (Saneesh) Project Name: HDI_Stellenbosch_2016

	Peak Name	RT	Area	% Area	Height
13		5.612	36434	1.01	2802
14		5.915	11890	0.33	1424
15		6.192	16197	0.45	1565
16		6.333	1829	0.05	490
17		6.802	1602	0.04	418
18		7.347	3971	0.11	491
19	E8H	7.589	5361	0.15	1225
20	EFV	8.125	582543	16.11	154014
21		8.383	1974	0.05	463
22	NEO	8.751	878588	24.29	230014
23		9.248	4281	0.12	996

Reported by User: Saneesh (Saneesh) Project Name: HDI_Stellenbosch_2016

SAMPLE INFORMATION

Sample Name:	EFV Inc Basil_MeOH 150mig/mL 3	Acquired By:	Saneesh
Sample Type:	Unknown	Date Acquired:	2017/09/05 07:59:53 PM
Vial:	16	Acq. Method Set:	HDI_EFV_E8H_NEO
Injection #:	1	Date Processed:	2017/09/08 05:06:28 PM
Injection Volume:	15.00 ul	Processing Method:	HDI_EFV_ W2996 245.0nm-1.2
Run Time:	10.5 Minutes	Channel Name:	W2996 245.0nm-1.2
Sample Set Name:	EFV_Basil MeOH IC50 5_9_17	Proc. Chnl. Descr.:	W2996 PDA 245.0 nm at 1.2

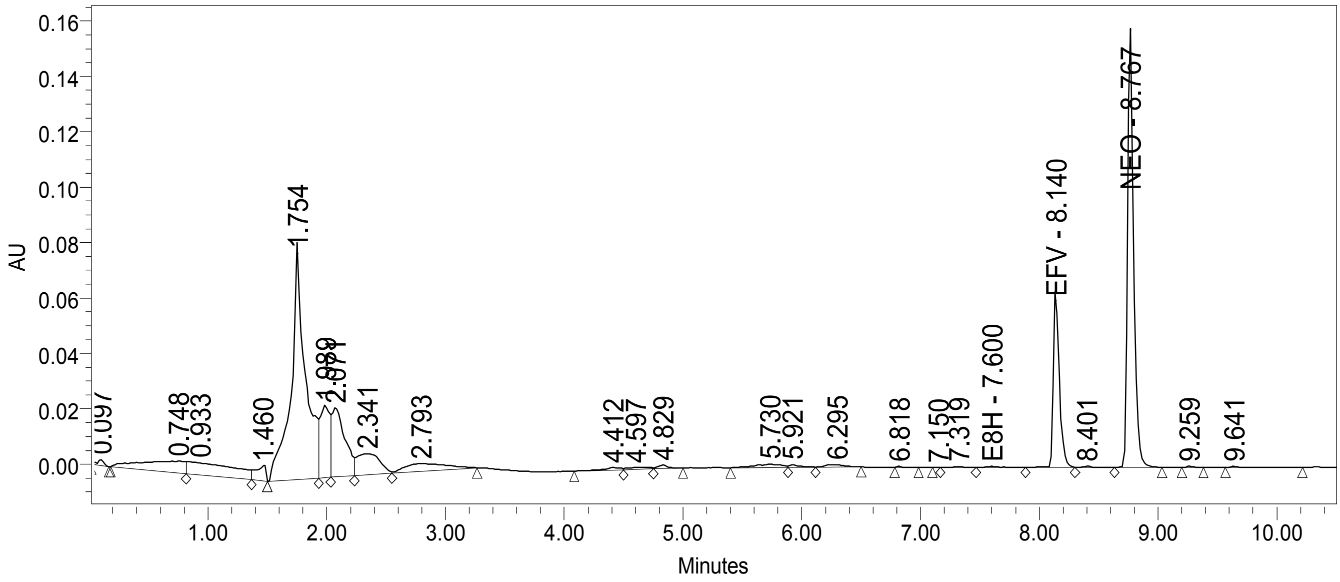

	Peak Name	RT	Area	% Area	Height
1		0.097	8099	0.34	2035
2		0.748	108619	4.55	4304
3		0.933	132547	5.55	4505
4		1.460	32698	1.37	6013
5		1.754	715467	29.98	78487
6		1.989	142833	5.98	25656
7		2.071	198740	8.33	24569
8		2.341	102591	4.30	7569
9		2.793	68865	2.89	2788
10		4.412	10630	0.45	958
11		4.597	8942	0.37	713
12		4.829	7425	0.31	1276

Reported by User: Saneesh (Saneesh) Project Name: HDI_Stellenbosch_2016

	Peak Name	RT	Area	% Area	Height
13		5.730	19246	0.81	1287
14		5.921	5714	0.24	951
15		6.295	10222	0.43	944
16		6.818	1195	0.05	307
17		7.150	278	0.01	109
18		7.319	2727	0.11	358
19	E8H	7.600	4071	0.17	418
20	EFV	8.140	221772	9.29	58518
21		8.401	2476	0.10	410
22	NEO	8.767	575761	24.12	150359
23		9.259	1915	0.08	416
24		9.641	3915	0.16	341

SAMPLE INFORMATION

Sample Name:	EFV Inc Basil_MeOH 200mig/mL 1	Acquired By:	Saneesh
Sample Type:	Unknown	Date Acquired:	2017/09/05 08:13:50 PM
Vial:	17	Acq. Method Set:	HDI_EFV_E8H_NEO
Injection #:	1	Date Processed:	2017/09/08 05:06:28 PM
Injection Volume:	15.00 ul	Processing Method:	HDI_EFV_ W2996 245.0nm-1.2
Run Time:	10.5 Minutes	Channel Name:	W2996 245.0nm-1.2
Sample Set Name:	EFV_Basil MeOH IC50 5_9_17	Proc. Chnl. Descr.:	W2996 PDA 245.0 nm at 1.2

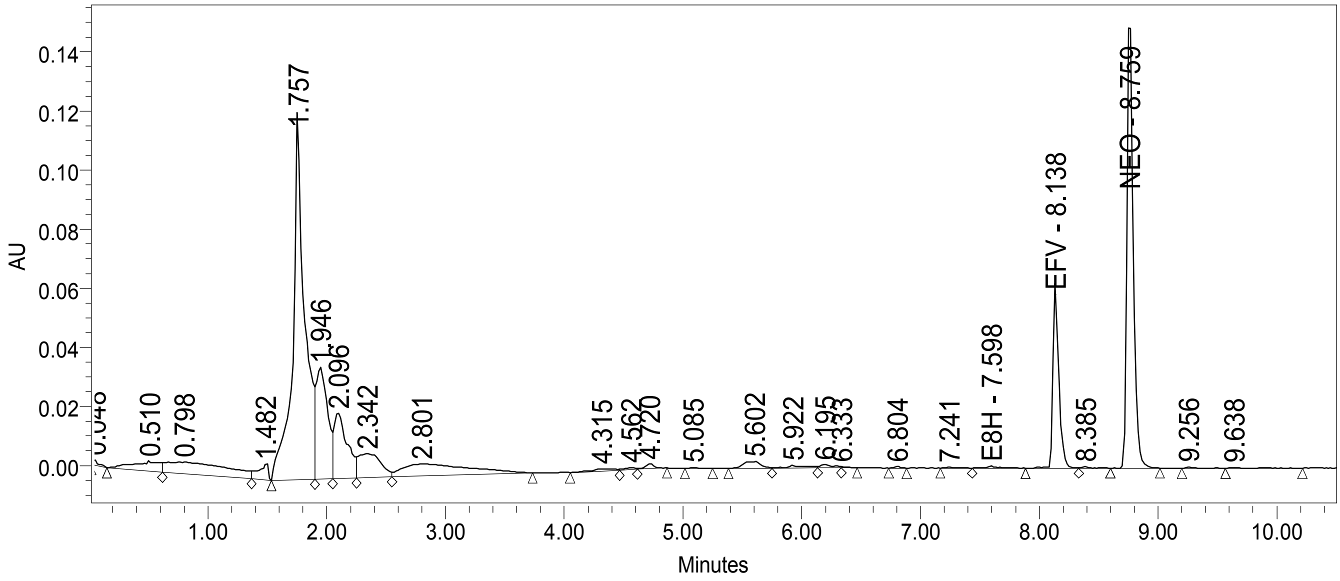

	Peak Name	RT	Area	% Area	Height
1		0.048	6057	0.23	1581
2		0.510	52938	1.98	3068
3		0.798	149705	5.61	3842
4		1.482	30863	1.16	4848
5		1.757	859983	32.21	114024
6		1.946	260295	9.75	37219
7		2.096	173317	6.49	22209
8		2.342	107106	4.01	8054
9		2.801	159389	5.97	4064
10		4.315	10196	0.38	808
11		4.562	4820	0.18	678
12		4.720	8852	0.33	1599

Reported by User: Saneesh (Saneesh) Project Name: HDI_Stellenbosch_2016

	Peak Name	RT	Area	% Area	Height
13		5.085	1375	0.05	238
14		5.602	22525	0.84	2237
15		5.922	9469	0.35	880
16		6.195	8086	0.30	1059
17		6.333	983	0.04	336
18		6.804	2021	0.08	509
19		7.241	1946	0.07	311
20	E8H	7.598	5096	0.19	651
21	EFV	8.138	212552	7.96	57282
22		8.385	2599	0.10	501
23	NEO	8.759	574620	21.52	144651
24		9.256	2057	0.08	373
25		9.638	3281	0.12	183

SAMPLE INFORMATION

Sample Name:	EFV Inc Basil_MeOH 200mig/mL 2	Acquired By:	Saneesh
Sample Type:	Unknown	Date Acquired:	2017/09/05 08:27:44 PM
Vial:	18	Acq. Method Set:	HDI_EFV_E8H_NEO
Injection #:	1	Date Processed:	2017/09/08 05:06:28 PM
Injection Volume:	15.00 ul	Processing Method:	HDI_EFV_ W2996 245.0nm-1.2
Run Time:	10.5 Minutes	Channel Name:	W2996 245.0nm-1.2
Sample Set Name:	EFV_Basil MeOH IC50 5_9_17	Proc. Chnl. Descr.:	W2996 PDA 245.0 nm at 1.2

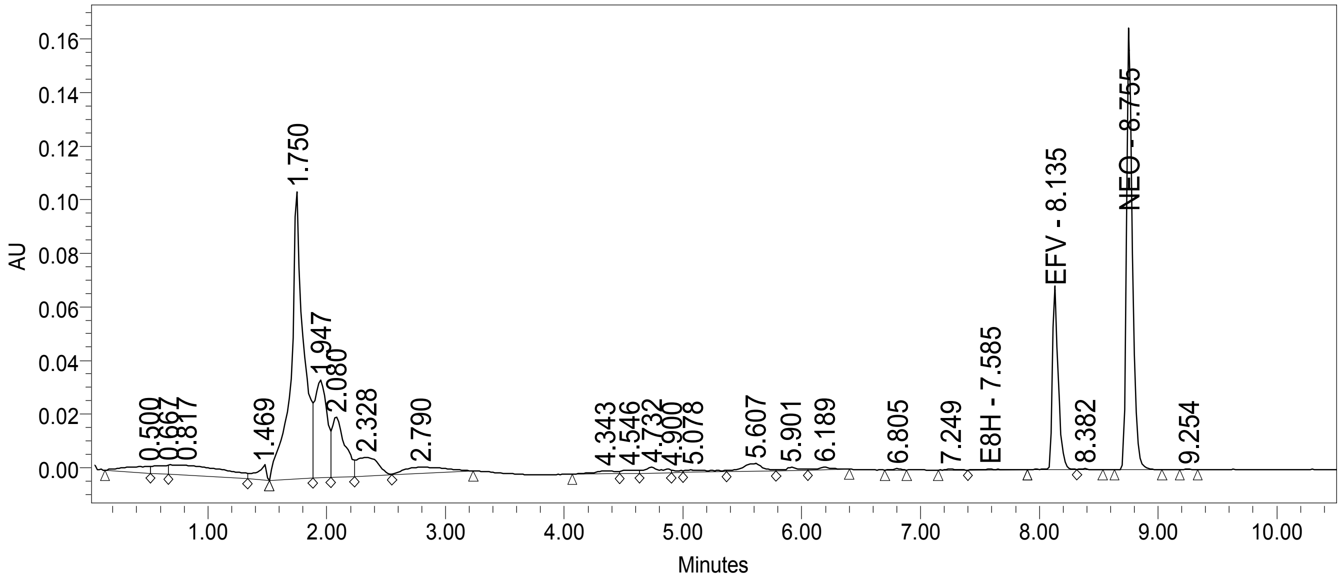

	Peak Name	RT	Area	% Area	Height
1		0.500	36001	1.41	2552
2		0.667	26507	1.04	3321
3		0.817	125417	4.91	3714
4		1.469	31781	1.24	4927
5		1.750	799252	31.27	102837
6		1.947	266069	10.41	36200
7		2.080	174785	6.84	22407
8		2.328	86717	3.39	6829
9		2.790	54585	2.14	2333
10		4.343	15633	0.61	1145
11		4.546	10995	0.43	1220
12		4.732	21490	0.84	2074

Reported by User: Saneesh (Saneesh) Project Name: HDI_Stellenbosch_2016

	Peak Name	RT	Area	% Area	Height
13		4.900	4476	0.18	893
14		5.078	12745	0.50	869
15		5.607	32242	1.26	2854
16		5.901	9557	0.37	1150
17		6.189	9733	0.38	1123
18		6.805	2278	0.09	595
19		7.249	2085	0.08	404
20	E8H	7.585	4634	0.18	465
21	EFV	8.135	235198	9.20	64082
22		8.382	2222	0.09	517
23	NEO	8.755	590313	23.09	154333
24		9.254	1527	0.06	370

Reported by User: Saneesh (Saneesh) Project Name: HDI_Stellenbosch_2016

SAMPLE INFORMATION

Sample Name:	EFV Inc Basil_MeOH 200mig/mL 3	Acquired By:	Saneesh
Sample Type:	Unknown	Date Acquired:	2017/09/05 08:41:41 PM
Vial:	19	Acq. Method Set:	HDI_EFV_E8H_NEO
Injection #:	1	Date Processed:	2017/09/08 05:06:28 PM
Injection Volume:	15.00 ul	Processing Method:	HDI_EFV_ W2996 245.0nm-1.2
Run Time:	10.5 Minutes	Channel Name:	W2996 245.0nm-1.2
Sample Set Name:	EFV_Basil MeOH IC50 5_9_17	Proc. Chnl. Descr.:	W2996 PDA 245.0 nm at 1.2

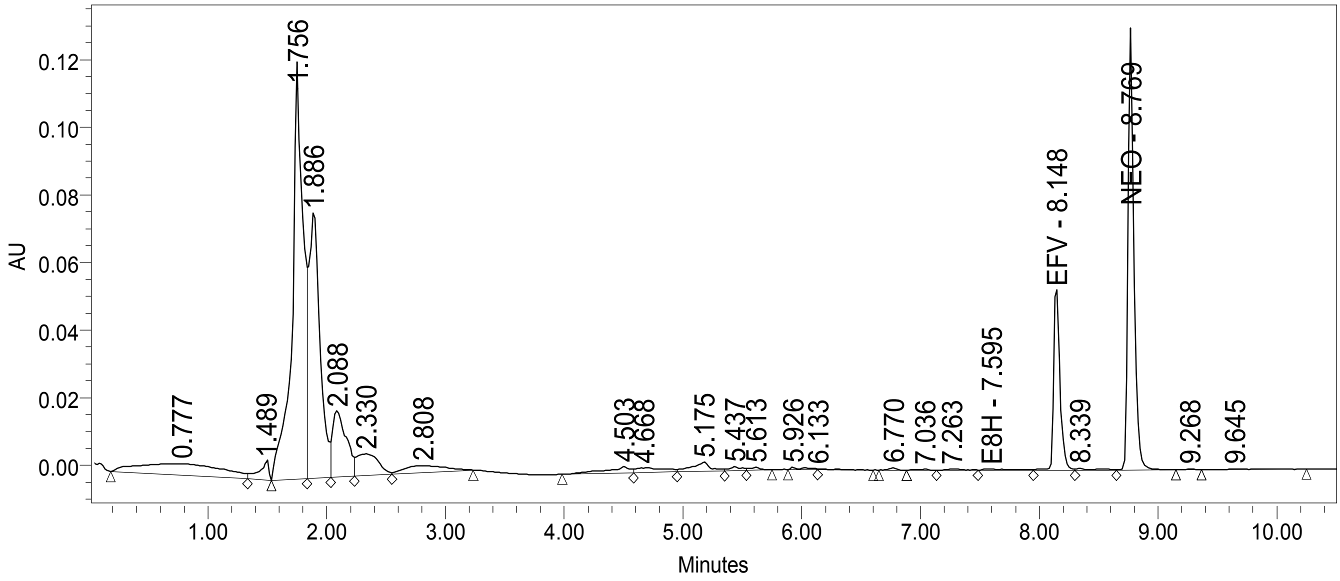

	Peak Name	RT	Area	% Area	Height
1		0.777	171252	6.51	3460
2		1.489	31420	1.19	5161
3		1.756	803394	30.55	117575
4		1.886	537634	20.45	77211
5		2.088	159665	6.07	19692
6		2.330	82824	3.15	6427
7		2.808	52382	1.99	2134
8		4.503	28028	1.07	1902
9		4.668	23647	0.90	1392
10		5.175	29765	1.13	2683
11		5.437	7810	0.30	1096
12		5.613	5051	0.19	787

Reported by User: Saneesh (Saneesh) Project Name: HDI_Stellenbosch_2016

	Peak Name	RT	Area	% Area	Height
13		5.926	6246	0.24	614
14		6.133	2645	0.10	283
15		6.770	3818	0.15	772
16		7.036	2283	0.09	312
17		7.263	3534	0.13	393
18	E8H	7.595	5414	0.21	339
19	EFV	8.148	195744	7.44	51762
20		8.339	5173	0.20	533
21	NEO	8.769	464345	17.66	122201
22		9.268	1794	0.07	340
23		9.645	5542	0.21	267

Reported by User: Saneesh (Saneesh) Project Name: HDI_Stellenbosch_2016

SAMPLE INFORMATION

Sample Name:	EFV Test Control 1 TDI	Acquired By:	Saneesh
Sample Type:	Unknown	Date Acquired:	2017/09/07 10:59:43 AM
Vial:	23	Acq. Method Set:	HDI_EFV_E8H_NEO
Injection #:	1	Date Processed:	2017/09/08 05:12:37 PM
Injection Volume:	15.00 ul	Processing Method:	HDI_EFV_ W2996 245.0nm-1.2
Run Time:	10.5 Minutes	Channel Name:	W2996 245.0nm-1.2
Sample Set Name:	EFV_Basil Aq TDI IC50 7_9_17	Proc. Chnl. Descr.:	W2996 PDA 245.0 nm at 1.2

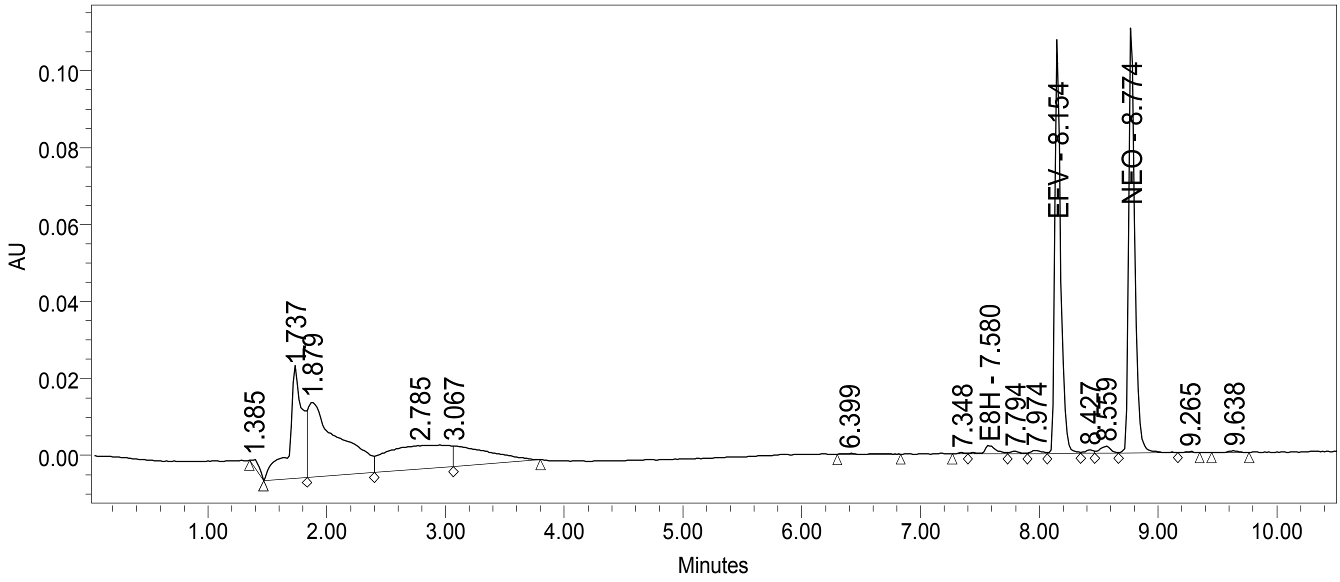

	Peak Name	RT	Area	% Area	Height
1		1.385	10378	0.59	1843
2		1.737	232712	13.32	28486
3		1.879	355399	20.34	19294
4		2.785	221703	12.69	5995
5		3.067	111385	6.37	5282
6		6.399	3620	0.21	244
7		7.348	1491	0.09	371
8	E8H	7.580	14290	0.82	2114
9		7.794	3387	0.19	610
10		7.974	4812	0.28	780
11	EFV	8.154	357843	20.48	98400
12		8.427	4106	0.24	853
13		8.559	10819	0.62	1717
14	NEO	8.774	411137	23.53	104665

Reported by User: Saneesh (Saneesh) Project Name: HDI_Stellenbosch_2016

	Peak Name	RT	Area	% Area	Height
15		9.265	1448	0.08	303
16		9.638	2749	0.16	418

SAMPLE INFORMATION

Sample Name:	EFV Test Control 2 TDI	Acquired By:	Saneesh
Sample Type:	Unknown	Date Acquired:	2017/09/07 11:14:22 AM
Vial:	24	Acq. Method Set:	HDI_EFV_E8H_NEO
Injection #:	1	Date Processed:	2017/09/08 05:12:37 PM
Injection Volume:	15.00 ul	Processing Method:	HDI_EFV_ W2996 245.0nm-1.2
Run Time:	10.5 Minutes	Channel Name:	W2996 245.0nm-1.2
Sample Set Name:	EFV_Basil Aq TDI IC50 7_9_17	Proc. Chnl. Descr.:	W2996 PDA 245.0 nm at 1.2

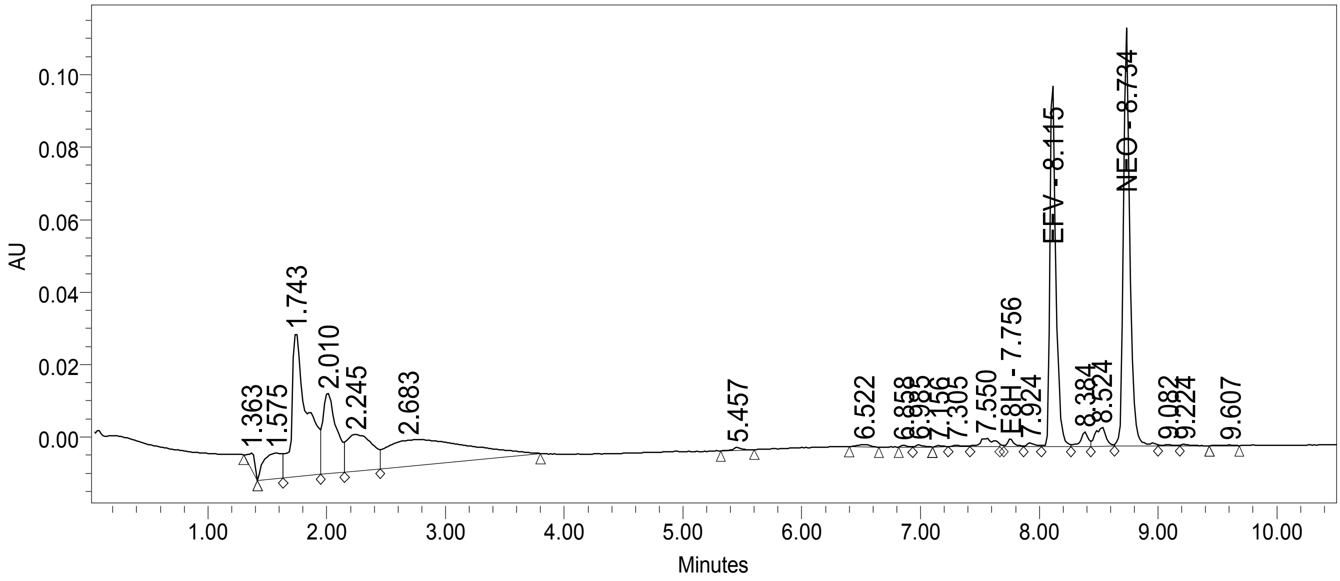

	Peak Name	RT	Area	% Area	Height
1		1.363	18505	0.92	4490
2		1.575	73773	3.67	7012
3		1.743	357848	17.80	39339
4		2.010	178373	8.87	22205
5		2.245	151685	7.54	10100
6		2.683	337505	16.78	7124
7		5.457	3753	0.19	741
8		6.522	5784	0.29	738
9		6.858	1971	0.10	541
10		6.985	2939	0.15	682
11		7.156	1906	0.09	421
12		7.305	2555	0.13	416

	Peak Name	RT	Area	% Area	Height
13		7.550	19665	0.98	2334
14	E8H	7.756	8486	0.42	1989
15		7.924	4950	0.25	988
16	EFV	8.115	353707	17.59	95943
17		8.384	17633	0.88	3844
18		8.524	32299	1.61	5101
19	NEO	8.734	430101	21.39	109142
20		9.082	2860	0.14	426
21		9.224	2696	0.13	497
22		9.607	1889	0.09	252

Reported by User: Saneesh (Saneesh)   Project Name: HDI_Stellenbosch_2016

SAMPLE INFORMATION

Sample Name:	EFV Inc Basil_Aq 1miG/mL TDI1	Acquired By:	Saneesh
Sample Type:	Unknown	Date Acquired:	2017/09/07 11:28:38 AM
Vial:	2	Acq. Method Set:	HDI_EFV_E8H_NEO
Injection #:	1	Date Processed:	2017/09/08 05:12:37 PM
Injection Volume:	15.00 ul	Processing Method:	HDI_EFV_ W2996 245.0nm-1.2
Run Time:	10.5 Minutes	Channel Name:	W2996 245.0nm-1.2
Sample Set Name:	EFV_Basil Aq TDI IC50 7_9_17	Proc. Chnl. Descr.:	W2996 PDA 245.0 nm at 1.2

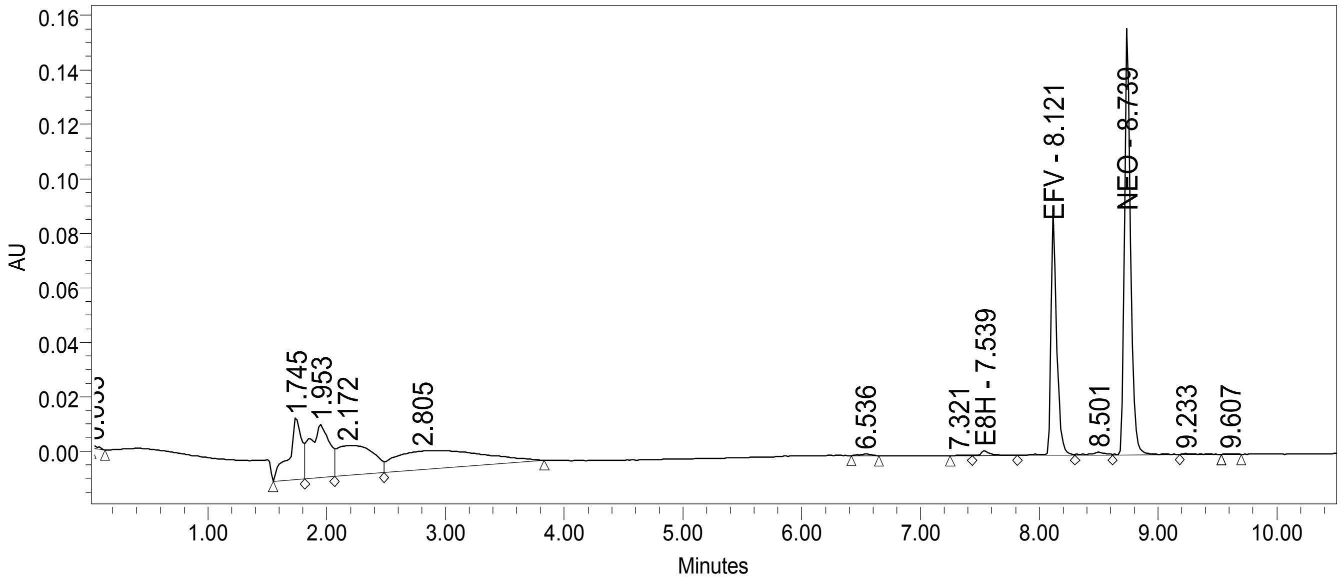

	Peak Name	RT	Area	% Area	Height
1		0.033	3723	0.20	1145
2		1.745	171132	9.25	22265
3		1.953	219806	11.88	19328
4		2.172	229367	12.40	10936
5		2.805	342328	18.51	6782
6		6.536	3730	0.20	545
7		7.321	1270	0.07	242
8	E8H	7.539	9685	0.52	1711
9	EFV	8.121	300980	16.27	82005
10		8.501	8043	0.43	925
11	NEO	8.739	556264	30.08	146290
12		9.233	2280	0.12	334

Reported by User: Saneesh (Saneesh) Project Name: HDI_Stellenbosch_2016

	Peak Name	RT	Area	% Area	Height
13		9.607	903	0.05	190

SAMPLE INFORMATION

Sample Name:	EFV Inc Basil_Aq 1miG/mL TDI2	Acquired By:	Saneesh
Sample Type:	Unknown	Date Acquired:	2017/09/07 11:42:56 AM
Vial:	3	Acq. Method Set:	HDI_EFV_E8H_NEO
Injection #:	1	Date Processed:	2017/09/08 05:12:37 PM
Injection Volume:	15.00 ul	Processing Method:	HDI_EFV_ W2996 245.0nm-1.2
Run Time:	10.5 Minutes	Channel Name:	W2996 245.0nm-1.2
Sample Set Name:	EFV_Basil Aq TDI IC50 7_9_17	Proc. Chnl. Descr.:	W2996 PDA 245.0 nm at 1.2

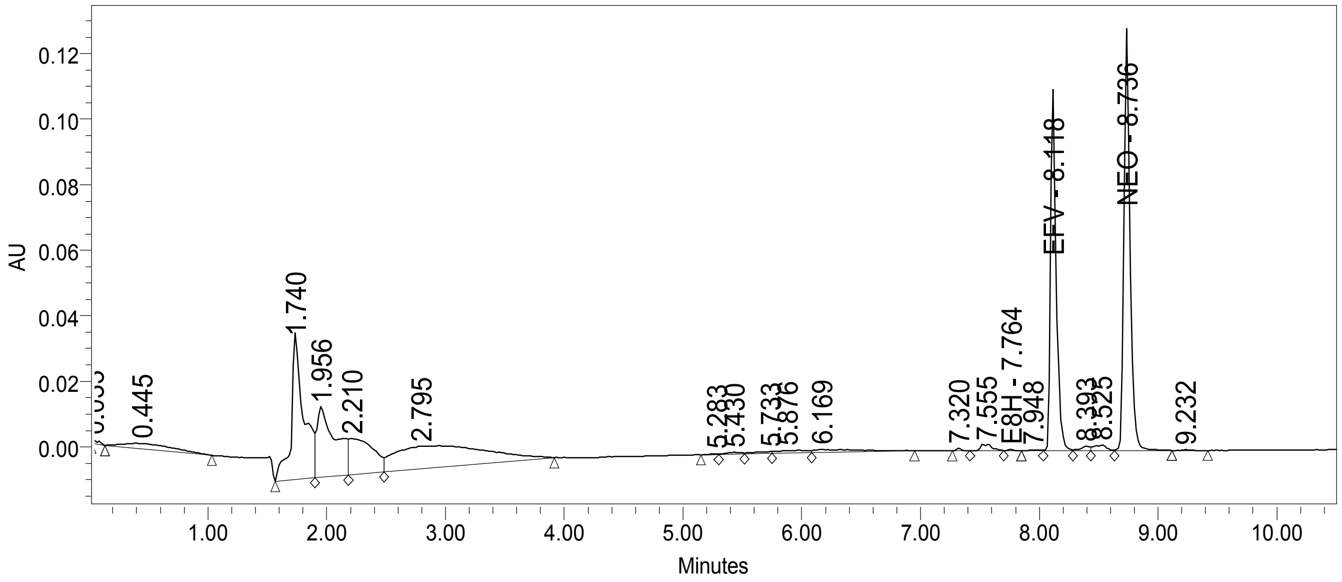

	Peak Name	RT	Area	% Area	Height
1		0.033	4336	0.21	1173
2		0.445	49980	2.43	1635
3		1.740	328211	15.96	44450
4		1.956	238250	11.58	21321
5		2.210	154342	7.50	10995
6		2.795	362381	17.62	6876
7		5.283	1326	0.06	236
8		5.430	5516	0.27	588
9		5.733	7009	0.34	588
10		5.876	14965	0.73	836
11		6.169	27021	1.31	1012
12		7.320	2966	0.14	719

	Peak Name	RT	Area	% Area	Height
13		7.555	14371	0.70	1998
14	E8H	7.764	1934	0.09	510
15		7.948	2161	0.11	290
16	EFV	8.118	367399	17.86	102417
17		8.393	7380	0.36	1391
18		8.525	12445	0.60	1659
19	NEO	8.736	453600	22.05	119920
20		9.232	1434	0.07	264

SAMPLE INFORMATION

Sample Name:	EFV Inc Basil_Aq 10miG/mL TDI1	Acquired By:	Saneesh
Sample Type:	Unknown	Date Acquired:	2017/09/07 11:56:53 AM
Vial:	4	Acq. Method Set:	HDI_EFV_E8H_NEO
Injection #:	1	Date Processed:	2017/09/08 05:12:37 PM
Injection Volume:	15.00 ul	Processing Method:	HDI_EFV_ W2996 245.0nm-1.2
Run Time:	10.5 Minutes	Channel Name:	W2996 245.0nm-1.2
Sample Set Name:	EFV_Basil Aq TDI IC50 7_9_17	Proc. Chnl. Descr.:	W2996 PDA 245.0 nm at 1.2

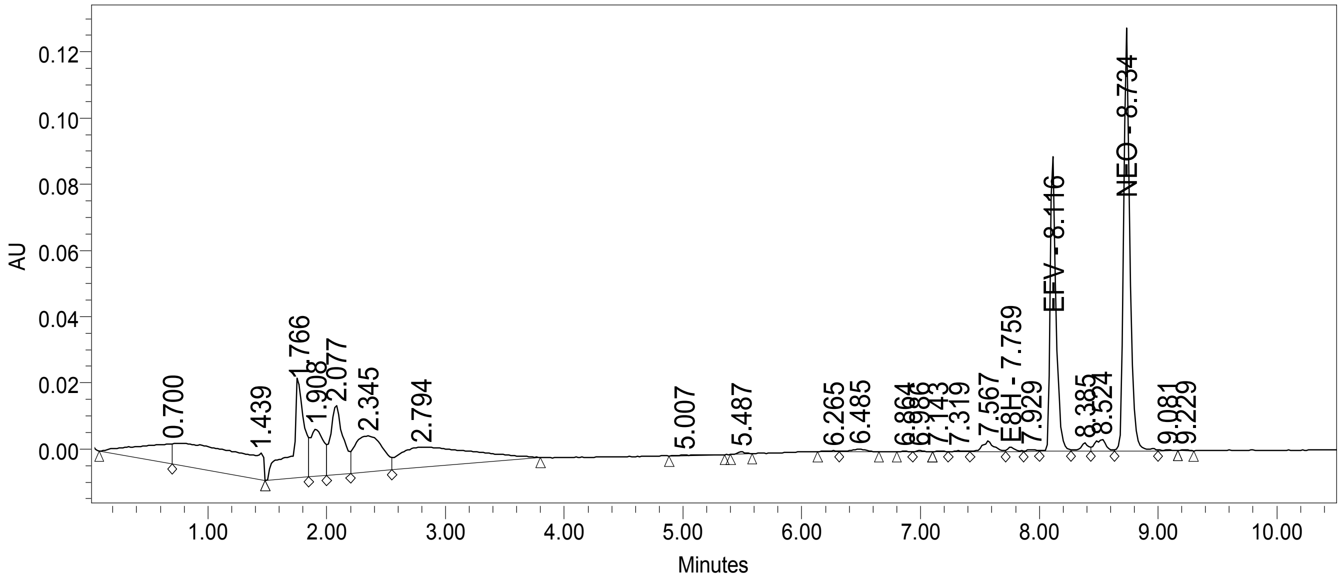

	Peak Name	RT	Area	% Area	Height
1		0.700	121244	5.44	6116
2		1.439	324773	14.58	8585
3		1.766	220182	9.88	27739
4		1.908	109571	4.92	14156
5		2.077	155918	7.00	20687
6		2.345	177487	7.97	10770
7		2.794	259052	11.63	6086
8		5.007	2240	0.10	273
9		5.487	3175	0.14	755
10		6.265	2671	0.12	388
11		6.485	8568	0.38	914
12		6.864	1393	0.06	352

Reported by User: Saneesh (Saneesh) Project Name: HDI_Stellenbosch_2016

	Peak Name	RT	Area	% Area	Height
13		6.986	1888	0.08	438
14		7.143	1323	0.06	267
15		7.319	1937	0.09	276
16		7.567	21622	0.97	3029
17	E8H	7.759	5453	0.24	1255
18		7.929	3146	0.14	587
19	EFV	8.116	308321	13.84	84460
20		8.385	11816	0.53	2446
21		8.524	22371	1.00	3398
22	NEO	8.734	460499	20.67	119842
23		9.081	1646	0.07	305
24		9.229	1508	0.07	346

SAMPLE INFORMATION

Sample Name:	EFV Inc Basil_Aq 10miG/mL TDI2	Acquired By:	Saneesh
Sample Type:	Unknown	Date Acquired:	2017/09/07 12:10:48 PM
Vial:	5	Acq. Method Set:	HDI_EFV_E8H_NEO
Injection #:	1	Date Processed:	2017/09/08 05:12:37 PM
Injection Volume:	15.00 ul	Processing Method:	HDI_EFV_ W2996 245.0nm-1.2
Run Time:	10.5 Minutes	Channel Name:	W2996 245.0nm-1.2
Sample Set Name:	EFV_Basil Aq TDI IC50 7_9_17	Proc. Chnl. Descr.:	W2996 PDA 245.0 nm at 1.2

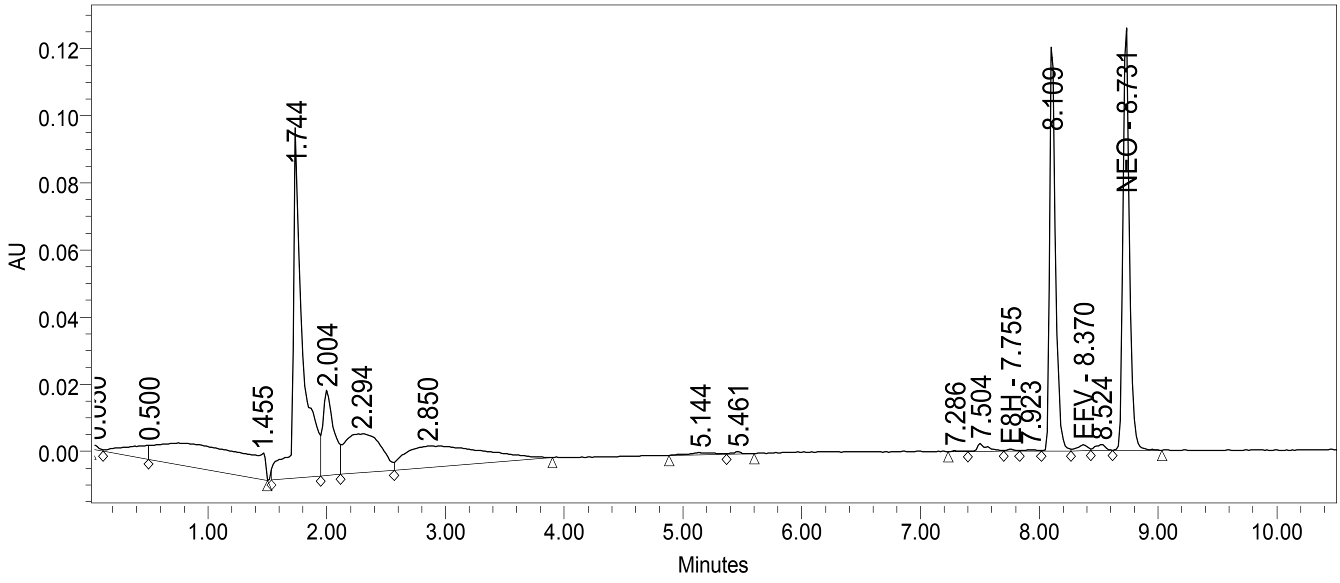

	Peak Name	RT	Area	% Area	Height
1		0.050	4524	0.17	1319
2		0.500	52791	1.97	4033
3		1.455	381607	14.25	8454
4		1.744	600814	22.44	91515
5		2.004	161728	6.04	25155
6		2.294	243124	9.08	11596
7		2.850	287775	10.75	6291
8		5.144	10041	0.38	633
9		5.461	3231	0.12	596
10		7.286	1266	0.05	251
11		7.504	14906	0.56	2284
12	E8H	7.755	2870	0.11	650

Reported by User: Saneesh (Saneesh)   Project Name: HDI_Stellenbosch_2016

	Peak Name	RT	Area	% Area	Height
13		7.923	2946	0.11	453
14		8.109	423070	15.80	112885
15	EFV	8.370	11043	0.41	1804
16		8.524	10818	0.40	1796
17	NEO	8.731	464920	17.36	121309

SAMPLE INFORMATION

Sample Name:	EFV Inc Basil_Aq 50miG/mL TDI1	Acquired By:	Saneesh
Sample Type:	Unknown	Date Acquired:	2017/09/07 12:24:44 PM
Vial:	6	Acq. Method Set:	HDI_EFV_E8H_NEO
Injection #:	1	Date Processed:	2017/09/08 05:12:37 PM
Injection Volume:	15.00 ul	Processing Method:	HDI_EFV_ W2996 245.0nm-1.2
Run Time:	10.5 Minutes	Channel Name:	W2996 245.0nm-1.2
Sample Set Name:	EFV_Basil Aq TDI IC50 7_9_17	Proc. Chnl. Descr.:	W2996 PDA 245.0 nm at 1.2

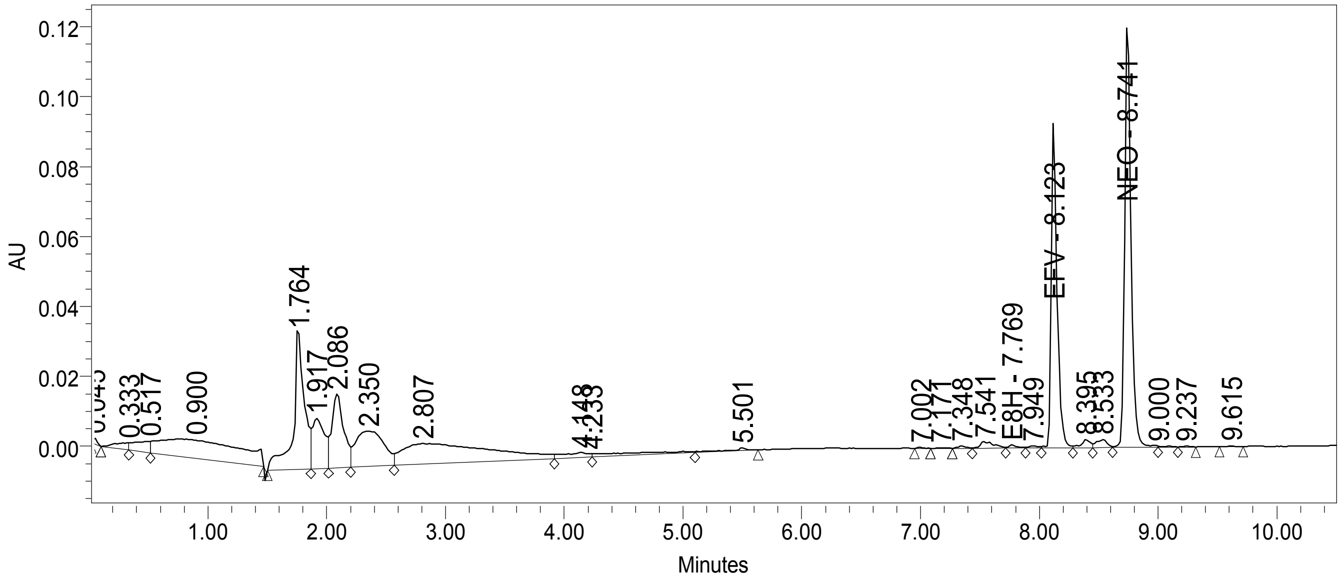

	Peak Name	RT	Area	% Area	Height
1		0.045	3820	0.18	1369
2		0.333	16263	0.75	2059
3		0.517	29328	1.35	3239
4		0.900	258369	11.92	5238
5		1.764	256920	11.85	36537
6		1.917	105683	4.88	14225
7		2.086	145498	6.71	21218
8		2.350	167626	7.73	10021
9		2.807	298515	13.77	5873
10		4.148	22181	1.02	1416
11		4.233	31705	1.46	901
12		5.501	8611	0.40	788

Reported by User: Saneesh (Saneesh)   Project Name: HDI_Stellenbosch_2016

	Peak Name	RT	Area	% Area	Height
13		7.002	1190	0.05	292
14		7.171	1146	0.05	193
15		7.348	3349	0.15	628
16		7.541	14877	0.69	1808
17	E8H	7.769	4232	0.20	892
18		7.949	3202	0.15	533
19	EFV	8.123	314078	14.49	84900
20		8.395	11877	0.55	2201
21		8.533	14318	0.66	2279
22	NEO	8.741	449741	20.75	114050
23		9.000	2031	0.09	290
24		9.237	1286	0.06	273
25		9.615	1559	0.07	308

SAMPLE INFORMATION

Sample Name:	EFV Inc Basil_Aq 50miG/mL TDI2	Acquired By:	Saneesh
Sample Type:	Unknown	Date Acquired:	2017/09/07 12:38:41 PM
Vial:	7	Acq. Method Set:	HDI_EFV_E8H_NEO
Injection #:	1	Date Processed:	2017/09/08 05:12:37 PM
Injection Volume:	15.00 ul	Processing Method:	HDI_EFV_ W2996 245.0nm-1.2
Run Time:	10.5 Minutes	Channel Name:	W2996 245.0nm-1.2
Sample Set Name:	EFV_Basil Aq TDI IC50 7_9_17	Proc. Chnl. Descr.:	W2996 PDA 245.0 nm at 1.2

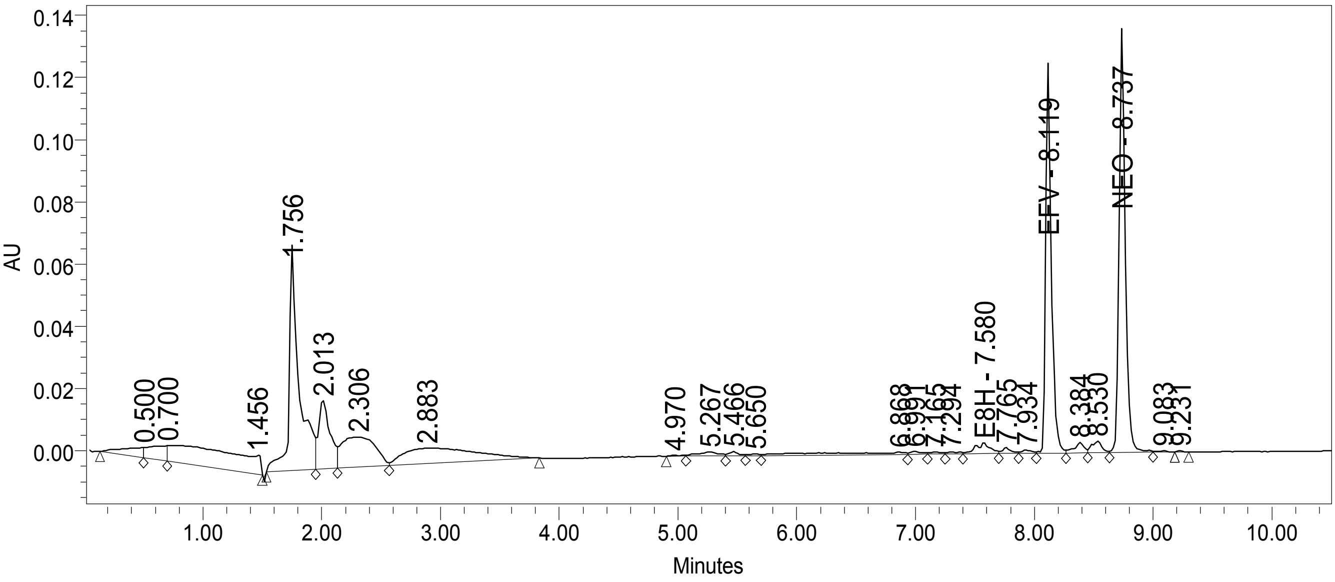

	Peak Name	RT	Area	% Area	Height
1		0.500	40101	1.66	3337
2		0.700	50441	2.09	5018
3		1.456	267626	11.09	6850
4		1.756	429499	17.80	67811
5		2.013	146963	6.09	21838
6		2.306	190020	7.87	9614
7		2.883	212627	8.81	4910
8		4.970	2072	0.09	289
9		5.267	14907	0.62	1209
10		5.466	7043	0.29	1229
11		5.650	2887	0.12	453
12		6.868	38485	1.59	721

Reported by User: Saneesh (Saneesh) Project Name: HDI_Stellenbosch_2016

	Peak Name	RT	Area	% Area	Height
13		6.991	5688	0.24	865
14		7.165	4562	0.19	605
15		7.294	4703	0.19	639
16	E8H	7.580	29583	1.23	3266
17		7.765	9764	0.40	1815
18		7.934	5971	0.25	985
19	EFV	8.119	418183	17.33	115444
20		8.384	19084	0.79	3228
21		8.530	21658	0.90	3620
22	NEO	8.737	487739	20.21	126930
23		9.083	2111	0.09	377
24		9.231	1283	0.05	363

Reported by User: Saneesh (Saneesh)   Project Name: HDI_Stellenbosch_2016

SAMPLE INFORMATION

Sample Name:	EFV Inc Basil_Aq 100mig/mL TDI1	Acquired By:	Saneesh
Sample Type:	Unknown	Date Acquired:	2017/09/07 12:52:35 PM
Vial:	8	Acq. Method Set:	HDI_EFV_E8H_NEO
Injection #:	1	Date Processed:	2017/09/08 05:12:37 PM
Injection Volume:	15.00 ul	Processing Method:	HDI_EFV_ W2996 245.0nm-1.2
Run Time:	10.5 Minutes	Channel Name:	W2996 245.0nm-1.2
Sample Set Name:	EFV_Basil Aq TDI IC50 7_9_17	Proc. Chnl. Descr.:	W2996 PDA 245.0 nm at 1.2

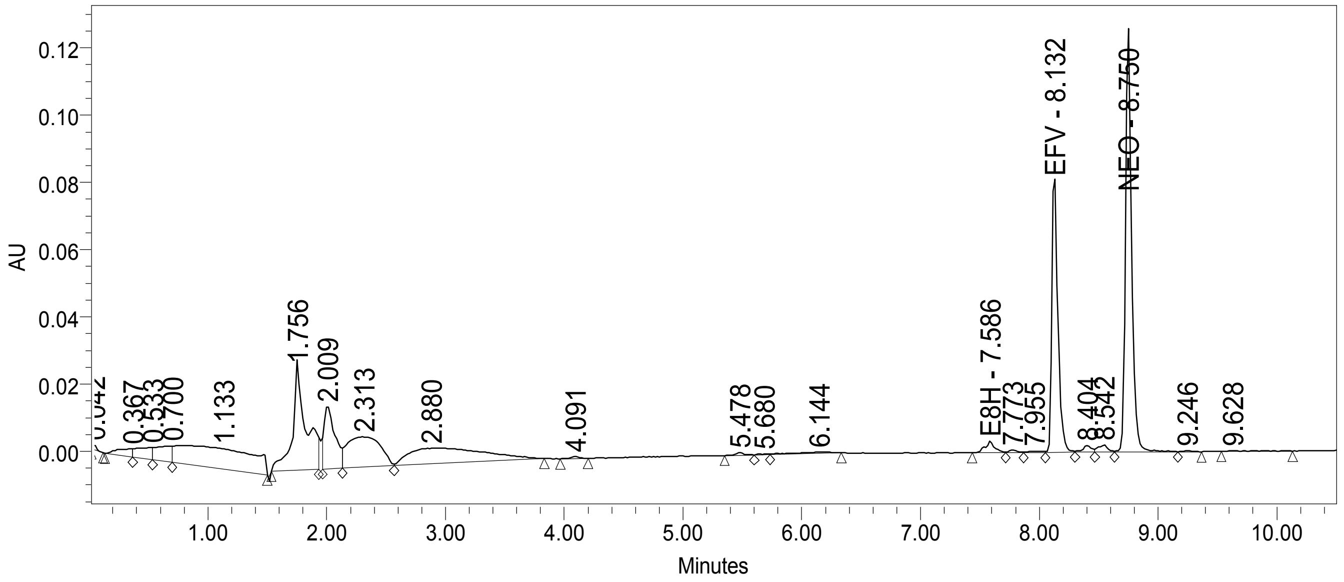

	Peak Name	RT	Area	% Area	Height
1		0.042	2835	0.15	1137
2		0.367	19434	1.01	2383
3		0.533	29945	1.55	3481
4		0.700	41766	2.17	4774
5		1.133	268181	13.92	6004
6		1.756	250802	13.02	29706
7		2.009	117396	6.10	18462
8		2.313	174863	9.08	8935
9		2.880	201146	10.44	4606
10		4.091	2986	0.16	583
11		5.478	3883	0.20	750
12		5.680	1212	0.06	167

Reported by User: Saneesh (Saneesh) Project Name: HDI_Stellenbosch_2016

	Peak Name	RT	Area	% Area	Height
13		6.144	9177	0.48	435
14	E8H	7.586	18939	0.98	3167
15		7.773	3251	0.17	705
16		7.955	3095	0.16	404
17	EFV	8.132	290960	15.11	78741
18		8.404	10570	0.55	1952
19		8.542	11968	0.62	1998
20	NEO	8.750	458336	23.80	119392
21		9.246	1505	0.08	261
22		9.628	3659	0.19	214

SAMPLE INFORMATION

Sample Name:	EFV Inc Basil_Aq 100mig/mL TDI2	Acquired By:	Saneesh
Sample Type:	Unknown	Date Acquired:	2017/09/07 01:06:32 PM
Vial:	9	Acq. Method Set:	HDI_EFV_E8H_NEO
Injection #:	1	Date Processed:	2017/09/08 05:12:38 PM
Injection Volume:	15.00 ul	Processing Method:	HDI_EFV_ W2996 245.0nm-1.2
Run Time:	10.5 Minutes	Channel Name:	W2996 245.0nm-1.2
Sample Set Name:	EFV_Basil Aq TDI IC50 7_9_17	Proc. Chnl. Descr.:	W2996 PDA 245.0 nm at 1.2

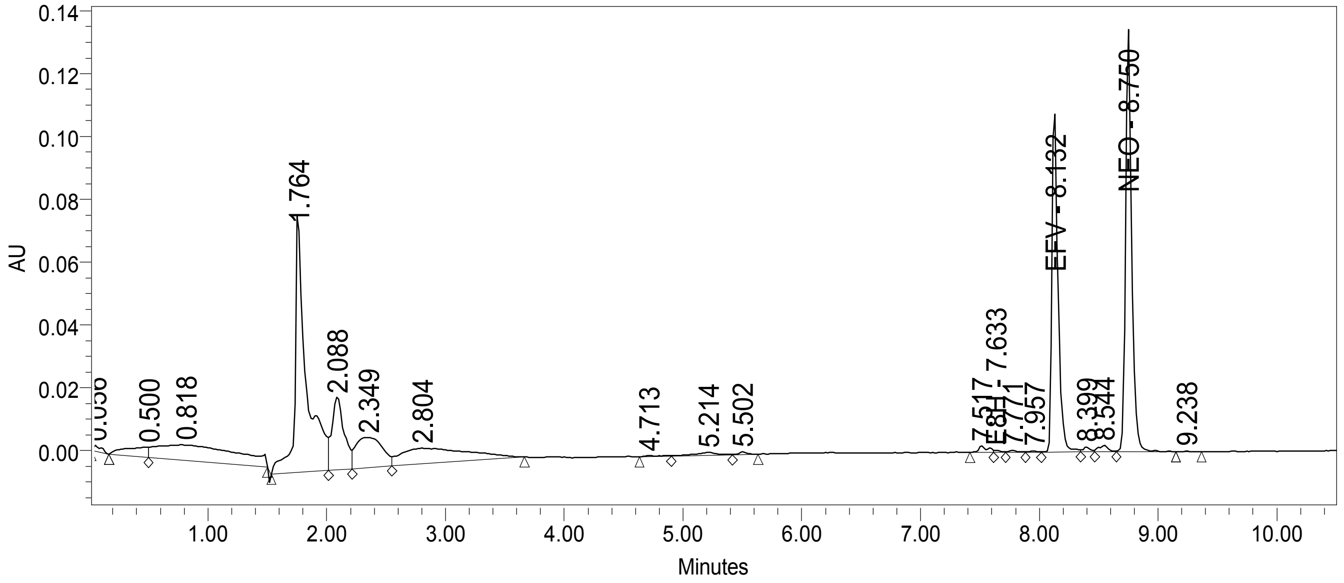

	Peak Name	RT	Area	% Area	Height
1		0.056	9043	0.39	1659
2		0.500	42914	1.87	3227
3		0.818	244782	10.66	4864
4		1.764	553206	24.08	73998
5		2.088	167988	7.31	23226
6		2.349	149100	6.49	9586
7		2.804	197096	8.58	5028
8		4.713	2626	0.11	254
9		5.214	12235	0.53	908
10		5.502	4061	0.18	810
11		7.517	11033	0.48	1946
12	E8H	7.633	2692	0.12	711

	Peak Name	RT	Area	% Area	Height
13		7.771	3267	0.14	679
14		7.957	2197	0.10	378
15	EFV	8.132	389457	16.95	103698
16		8.399	7000	0.30	1522
17		8.544	11542	0.50	1985
18	NEO	8.750	485939	21.15	126899
19		9.238	1042	0.05	282

Reported by User: Saneesh (Saneesh)

Project Name: HDI_Stellenbosch_2016

SAMPLE INFORMATION

Sample Name:	EFV Inc Basil_Aq 150mig/mL TDI1	Acquired By:	Saneesh
Sample Type:	Unknown	Date Acquired:	2017/09/07 01:20:28 PM
Vial:	10	Acq. Method Set:	HDI_EFV_E8H_NEO
Injection #:	1	Date Processed:	2017/09/08 05:12:38 PM
Injection Volume:	15.00 ul	Processing Method:	HDI_EFV_ W2996 245.0nm-1.2
Run Time:	10.5 Minutes	Channel Name:	W2996 245.0nm-1.2
Sample Set Name:	EFV_Basil Aq TDI IC50 7_9_17	Proc. Chnl. Descr.:	W2996 PDA 245.0 nm at 1.2

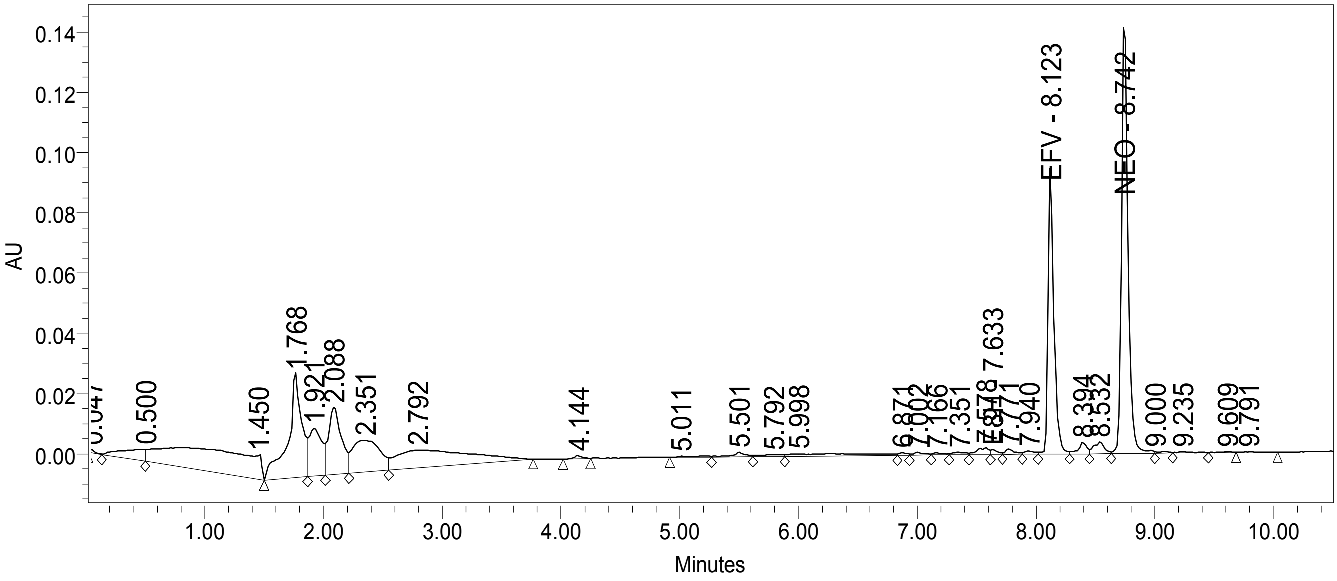

	Peak Name	RT	Area	% Area	Height
1		0.047	3357	0.14	995
2		0.500	51422	2.07	3959
3		1.450	391530	15.75	8354
4		1.768	282073	11.35	33965
5		1.921	121044	4.87	15844
6		2.088	171816	6.91	22565
7		2.351	168911	6.79	10553
8		2.792	250004	10.06	5923
9		4.144	4569	0.18	981
10		5.011	4727	0.19	331
11		5.501	11054	0.44	1382
12		5.792	8187	0.33	579

Reported by User: Saneesh (Saneesh) Project Name: HDI_Stellenbosch_2016

	Peak Name	RT	Area	% Area	Height
13		5.998	36029	1.45	785
14		6.871	3859	0.16	841
15		7.002	6588	0.26	899
16		7.166	4891	0.20	679
17		7.351	6756	0.27	831
18		7.578	16215	0.65	2182
19	E8H	7.633	6557	0.26	1629
20		7.771	9513	0.38	1762
21		7.940	6501	0.26	1038
22	EFV	8.123	324713	13.06	86803
23		8.394	18553	0.75	3541
24		8.532	23429	0.94	3630
25	NEO	8.742	543535	21.86	135547
26		9.000	2992	0.12	535
27		9.235	3009	0.12	417
28		9.609	1764	0.07	296
29		9.791	2648	0.11	210

SAMPLE INFORMATION

Sample Name:	EFV Inc Basil_Aq 150mig/mL TDI2	Acquired By:	Saneesh
Sample Type:	Unknown	Date Acquired:	2017/09/07 01:34:23 PM
Vial:	11	Acq. Method Set:	HDI_EFV_E8H_NEO
Injection #:	1	Date Processed:	2017/09/08 05:12:38 PM
Injection Volume:	15.00 ul	Processing Method:	HDI_EFV_ W2996 245.0nm-1.2
Run Time:	10.5 Minutes	Channel Name:	W2996 245.0nm-1.2
Sample Set Name:	EFV_Basil Aq TDI IC50 7_9_17	Proc. Chnl. Descr.:	W2996 PDA 245.0 nm at 1.2

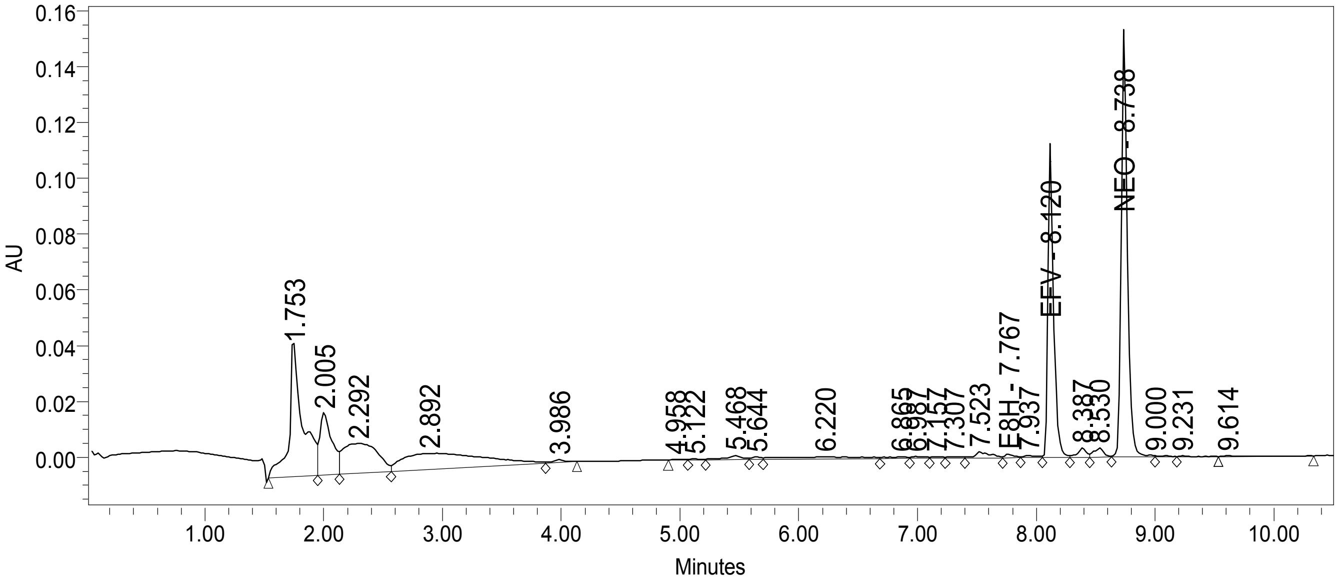

	Peak Name	RT	Area	% Area	Height
1		1.753	383911	18.20	48853
2		2.005	158436	7.51	22352
3		2.292	216048	10.24	10641
4		2.892	273771	12.98	5714
5		3.986	6923	0.33	1031
6		4.958	1695	0.08	293
7		5.122	2350	0.11	326
8		5.468	14007	0.66	1462
9		5.644	4845	0.23	857
10		6.220	35680	1.69	780
11		6.865	7473	0.35	686
12		6.987	5157	0.24	720

Reported by User: Saneesh (Saneesh) Project Name: HDI_Stellenbosch_2016

	Peak Name	RT	Area	% Area	Height
13		7.157	3849	0.18	579
14		7.307	4398	0.21	525
15		7.523	19918	0.94	2153
16	E8H	7.767	7116	0.34	1359
17		7.937	6495	0.31	834
18	EFV	8.120	369980	17.54	102506
19		8.387	16570	0.79	3205
20		8.530	18838	0.89	3094
21	NEO	8.738	543562	25.77	142305
22		9.000	2567	0.12	403
23		9.231	2093	0.10	334
24		9.614	3876	0.18	275

Multi Sample Summary

Reported by User: Saneesh (Saneesh)   Project Name: HDI_Stellenbosch_2016

SAMPLE INFORMATION

Sample Name:	EFV Inc Basil_Aq 200mig/mL TDI1	Acquired By:	Saneesh
Sample Type:	Unknown	Date Acquired:	2017/09/07 01:48:18 PM
Vial:	12	Acq. Method Set:	HDI_EFV_E8H_NEO
Injection #:	1	Date Processed:	2017/09/08 05:12:38 PM
Injection Volume:	15.00 ul	Processing Method:	HDI_EFV_ W2996 245.0nm-1.2
Run Time:	10.5 Minutes	Channel Name:	W2996 245.0nm-1.2
Sample Set Name:	EFV_Basil Aq TDI IC50 7_9_17	Proc. Chnl. Descr.:	W2996 PDA 245.0 nm at 1.2

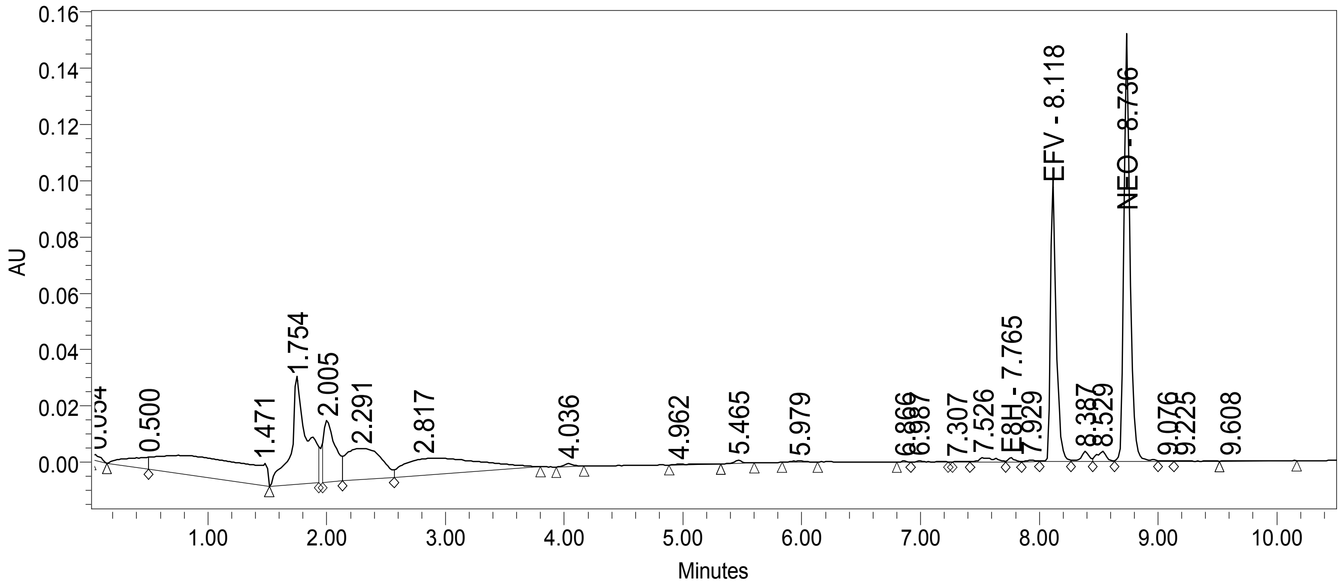

	Peak Name	RT	Area	% Area	Height
1		0.054	9303	0.39	2115
2		0.500	54067	2.25	4213
3		1.471	390432	16.24	8304
4		1.754	350857	14.59	38254
5		2.005	148707	6.19	21788
6		2.291	224865	9.35	10969
7		2.817	254649	10.59	5782
8		4.036	5617	0.23	1091
9		4.962	3064	0.13	242
10		5.465	5725	0.24	1162
11		5.979	3683	0.15	431
12		6.866	1164	0.05	378

Reported by User: Saneesh (Saneesh) Project Name: HDI_Stellenbosch_2016

	Peak Name	RT	Area	% Area	Height
13		6.987	3491	0.15	501
14		7.307	1633	0.07	226
15		7.526	14648	0.61	1515
16	E8H	7.765	5780	0.24	1438
17		7.929	3914	0.16	709
18	EFV	8.118	336803	14.01	93178
19		8.387	17133	0.71	3453
20		8.529	20109	0.84	3422
21	NEO	8.736	540783	22.49	141710
22		9.076	1788	0.07	282
23		9.225	2254	0.09	305
24		9.608	3564	0.15	226

SAMPLE INFORMATION

Sample Name:	EFV Inc Basil_Aq 200mig/mL TDI2	Acquired By:	Saneesh
Sample Type:	Unknown	Date Acquired:	2017/09/07 02:02:14 PM
Vial:	13	Acq. Method Set:	HDI_EFV_E8H_NEO
Injection #:	1	Date Processed:	2017/09/08 05:12:38 PM
Injection Volume:	15.00 ul	Processing Method:	HDI_EFV_ W2996 245.0nm-1.2
Run Time:	10.5 Minutes	Channel Name:	W2996 245.0nm-1.2
Sample Set Name:	EFV_Basil Aq TDI IC50 7_9_17	Proc. Chnl. Descr.:	W2996 PDA 245.0 nm at 1.2

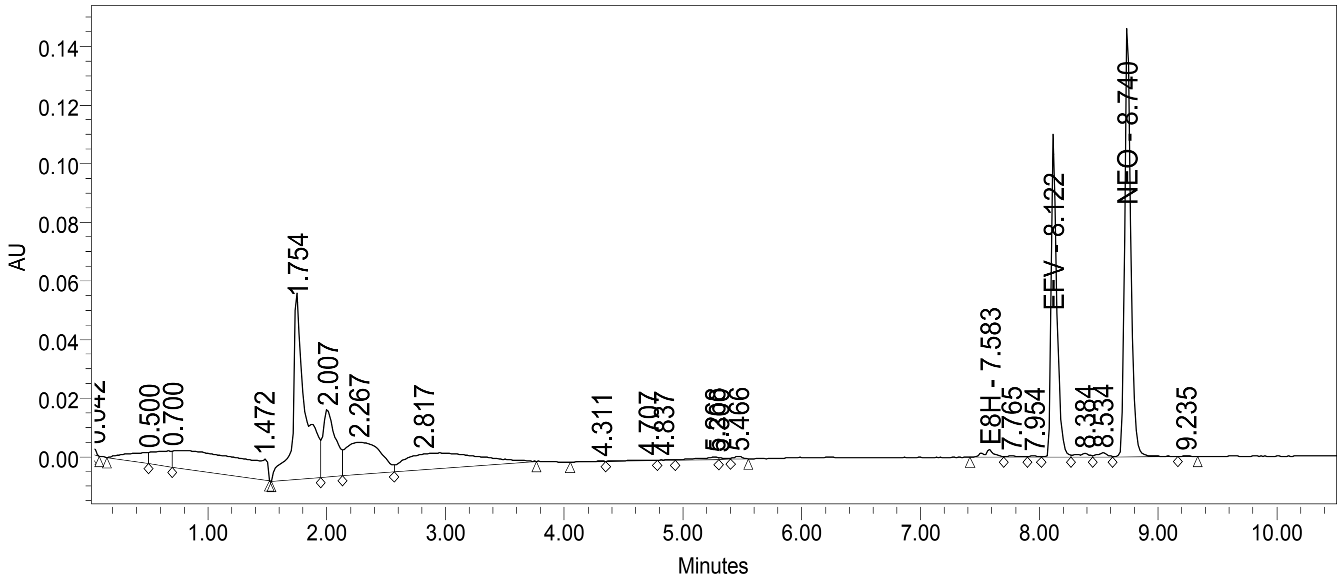

	Peak Name	RT	Area	% Area	Height
1		0.042	4051	0.16	1624
2		0.500	46130	1.87	3754
3		0.700	56258	2.28	5412
4		1.472	305870	12.37	7825
5		1.754	465977	18.85	63455
6		2.007	169916	6.87	22992
7		2.267	220756	8.93	10944
8		2.817	233973	9.47	5433
9		4.311	2404	0.10	215
10		4.707	4622	0.19	252
11		4.837	1729	0.07	223
12		5.268	10396	0.42	854

Reported by User: Saneesh (Saneesh)   Project Name: HDI_Stellenbosch_2016

	Peak Name	RT	Area	% Area	Height
13		5.300	2565	0.10	642
14		5.466	4539	0.18	917
15	E8H	7.583	16096	0.65	2487
16		7.765	3618	0.15	605
17		7.954	2210	0.09	406
18	EFV	8.122	367466	14.87	100557
19		8.384	9384	0.38	1167
20		8.534	8161	0.33	1319
21	NEO	8.740	533757	21.59	138294
22		9.235	2077	0.08	429

Reported by User: Saneesh (Saneesh) Project Name: HDI_Stellenbosch_2016

SAMPLE INFORMATION

Sample Name:	EFV Inc Basil_MeOH 1uG/mL TDI1	Acquired By:	Saneesh
Sample Type:	Unknown	Date Acquired:	2017/09/07 02:35:24 PM
Vial:	2	Acq. Method Set:	HDI_EFV_E8H_NEO
Injection #:	1	Date Processed:	2017/09/08 05:13:38 PM
Injection Volume:	15.00 ul	Processing Method:	HDI_EFV_ W2996 245.0nm-1.2
Run Time:	10.5 Minutes	Channel Name:	W2996 245.0nm-1.2
Sample Set Name:	EFV_Basil MeOH TDI IC50 7_9_17	Proc. Chnl. Descr.:	W2996 PDA 245.0 nm at 1.2

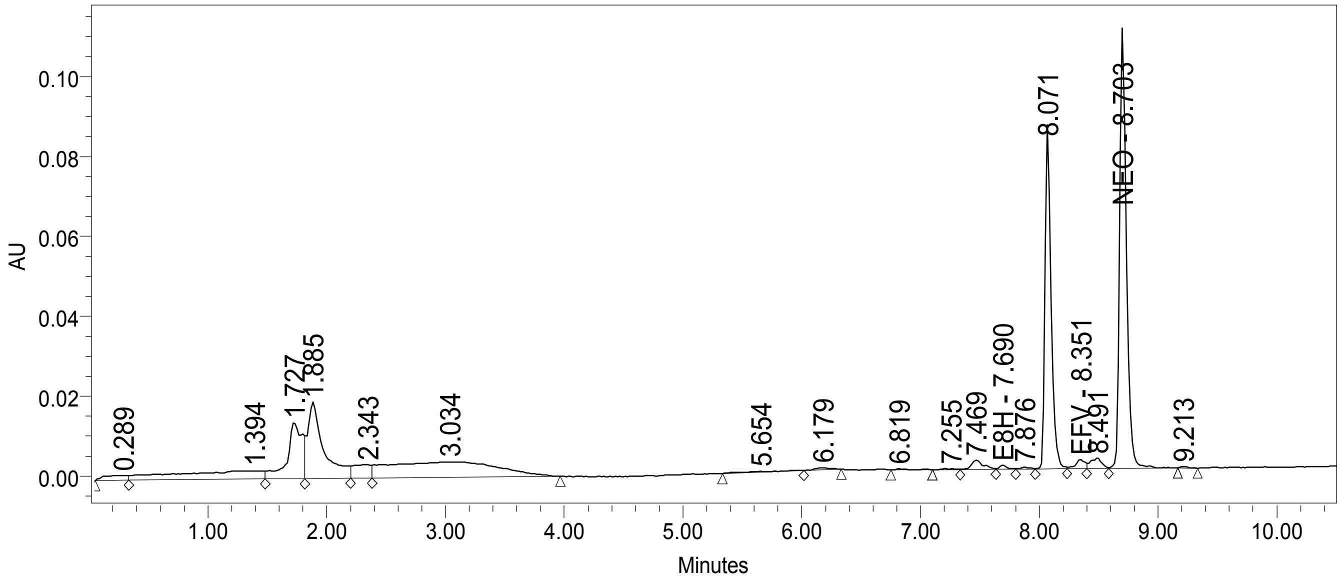

	Peak Name	RT	Area	% Area	Height
1		0.289	16252	1.07	1152
2		1.394	109815	7.23	2019
3		1.727	124575	8.21	14178
4		1.885	178935	11.79	18894
5		2.343	35260	2.32	3273
6		3.034	247763	16.32	3799
7		5.654	4937	0.33	192
8		6.179	4816	0.32	539
9		6.819	2294	0.15	264
10		7.255	2330	0.15	240
11		7.469	17363	1.14	2330
12	E8H	7.690	4959	0.33	991
13		7.876	3470	0.23	503
14		8.071	315292	20.77	81287

Reported by User: Saneesh (Saneesh) Project Name: HDI_Stellenbosch_2016

	Peak Name	RT	Area	% Area	Height
15	EFV	8.351	12467	0.82	2251
16		8.491	17469	1.15	2572
17	NEO	8.703	418295	27.56	104132
18		9.213	1719	0.11	343

SAMPLE INFORMATION

Sample Name:	EFV Inc Basil_MeOH 1uG/mL TDI2	Acquired By:	Saneesh
Sample Type:	Unknown	Date Acquired:	2017/09/07 02:49:42 PM
Vial:	3	Acq. Method Set:	HDI_EFV_E8H_NEO
Injection #:	1	Date Processed:	2017/09/08 05:13:38 PM
Injection Volume:	15.00 ul	Processing Method:	HDI_EFV_ W2996 245.0nm-1.2
Run Time:	10.5 Minutes	Channel Name:	W2996 245.0nm-1.2
Sample Set Name:	EFV_Basil MeOH TDI IC50 7_9_17	Proc. Chnl. Descr.:	W2996 PDA 245.0 nm at 1.2

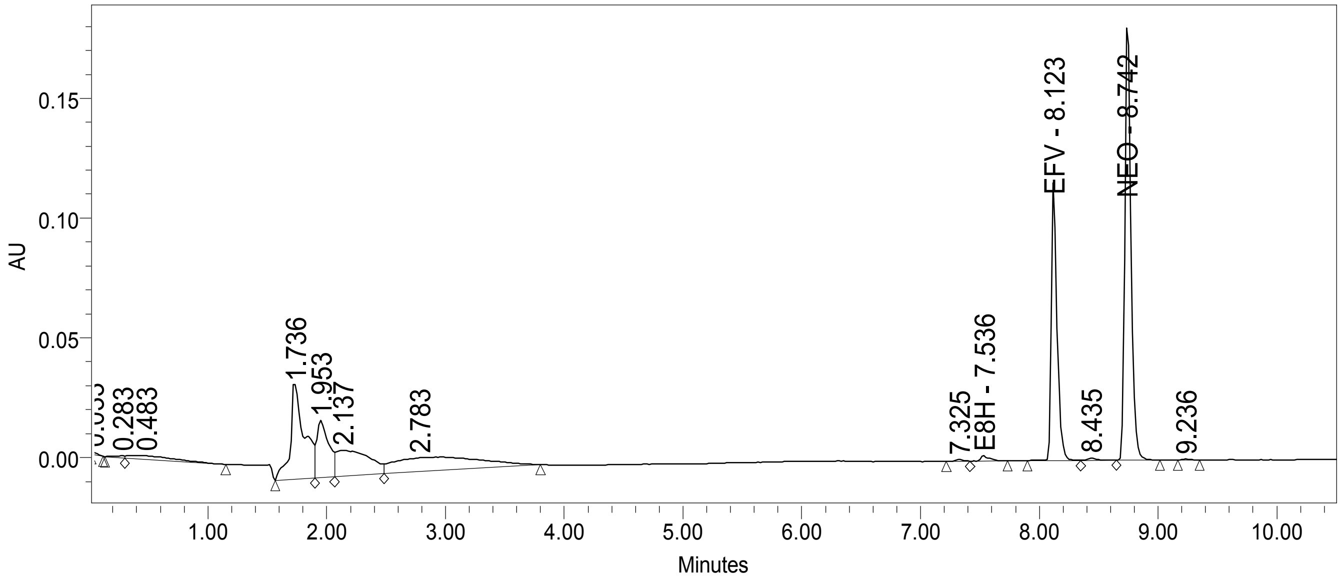

	Peak Name	RT	Area	% Area	Height
1		0.033	2953	0.14	981
2		0.283	4928	0.23	832
3		0.483	40706	1.91	1523
4		1.736	327053	15.37	38997
5		1.953	168851	7.94	23827
6		2.137	210924	9.91	10830
7		2.783	296950	13.96	5928
8		7.325	3381	0.16	720
9	E8H	7.536	11678	0.55	2125
10	EFV	8.123	390296	18.34	106112
11		8.435	6338	0.30	1042
12	NEO	8.742	661568	31.09	172427

	Peak Name	RT	Area	% Area	Height
13		9.236	2070	0.10	490

SAMPLE INFORMATION

Sample Name:	EFV Inc Basil_MeOH 10uG/mL TDI1	Acquired By:	Saneesh
Sample Type:	Unknown	Date Acquired:	2017/09/07 03:03:48 PM
Vial:	4	Acq. Method Set:	HDI_EFV_E8H_NEO
Injection #:	1	Date Processed:	2017/09/08 05:13:38 PM
Injection Volume:	15.00 ul	Processing Method:	HDI_EFV_ W2996 245.0nm-1.2
Run Time:	10.5 Minutes	Channel Name:	W2996 245.0nm-1.2
Sample Set Name:	EFV_Basil MeOH TDI IC50 7_9_17	Proc. Chnl. Descr.:	W2996 PDA 245.0 nm at 1.2

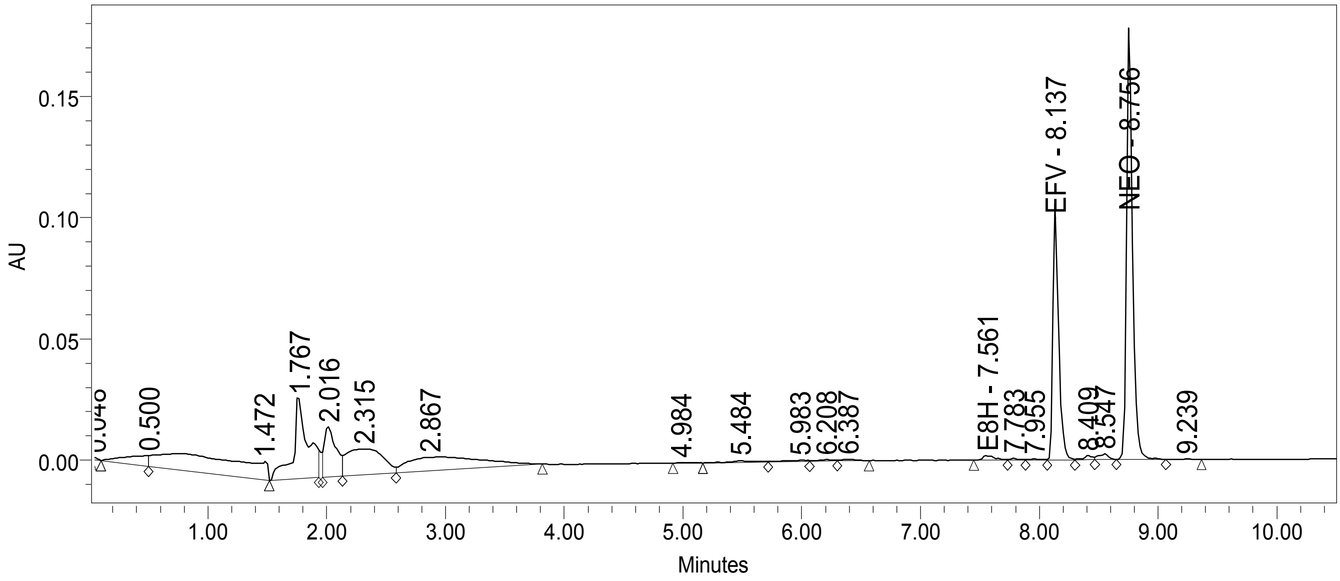

	Peak Name	RT	Area	% Area	Height
1		0.048	2640	0.11	868
2		0.500	59771	2.49	4372
3		1.472	377953	15.75	8118
4		1.767	288391	12.02	31545
5		2.016	139787	5.83	20433
6		2.315	226310	9.43	10599
7		2.867	243440	10.14	5556
8		4.984	1701	0.07	254
9		5.484	8362	0.35	724
10		5.983	7731	0.32	477
11		6.208	5865	0.24	568
12		6.387	6089	0.25	751

Reported by User: Saneesh (Saneesh) Project Name: HDI_Stellenbosch_2016

	Peak Name	RT	Area	% Area	Height
13	E8H	7.561	13256	0.55	1889
14		7.783	3052	0.13	718
15		7.955	3246	0.14	429
16	EFV	8.137	348483	14.52	96500
17		8.409	9058	0.38	1694
18		8.547	14195	0.59	2258
19	NEO	8.756	638258	26.60	166299
20		9.239	2147	0.09	260

SAMPLE INFORMATION

Sample Name:	EFV Inc Basil_MeOH 10uG/mL TDI2	Acquired By:	Saneesh
Sample Type:	Unknown	Date Acquired:	2017/09/07 03:17:44 PM
Vial:	5	Acq. Method Set:	HDI_EFV_E8H_NEO
Injection #:	1	Date Processed:	2017/09/08 05:13:38 PM
Injection Volume:	15.00 ul	Processing Method:	HDI_EFV_ W2996 245.0nm-1.2
Run Time:	10.5 Minutes	Channel Name:	W2996 245.0nm-1.2
Sample Set Name:	EFV_Basil MeOH TDI IC50 7_9_17	Proc. Chnl. Descr.:	W2996 PDA 245.0 nm at 1.2

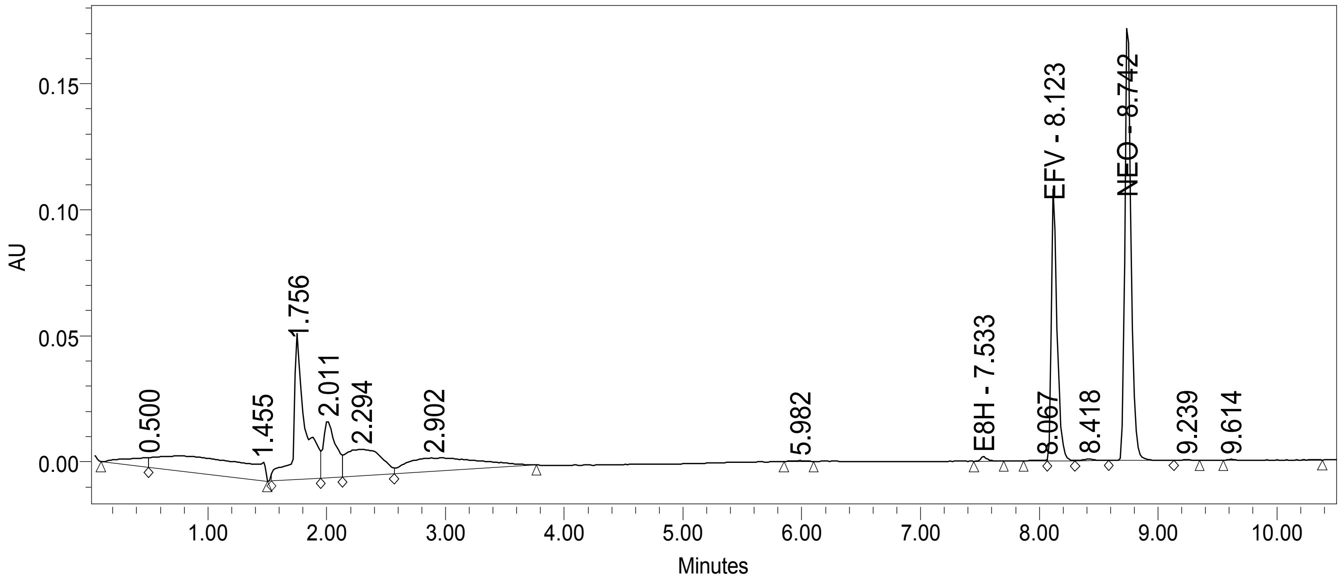

	Peak Name	RT	Area	% Area	Height
1		0.500	51431	2.12	3824
2		1.455	351142	14.45	7662
3		1.756	402305	16.55	55754
4		2.011	162633	6.69	22381
5		2.294	213947	8.80	10352
6		2.902	228006	9.38	5234
7		5.982	2719	0.11	347
8	E8H	7.533	8095	0.33	1838
9		8.067	2195	0.09	403
10	EFV	8.123	362336	14.91	98580
11		8.418	6199	0.26	795
12	NEO	8.742	633703	26.07	164102

Reported by User: Saneesh (Saneesh)   Project Name: HDI_Stellenbosch_2016

	Peak Name	RT	Area	% Area	Height
13		9.239	2033	0.08	406
14		9.614	4014	0.17	394

SAMPLE INFORMATION

Sample Name:	EFV Inc Basil_MeOH 50uG/mL TDI1	Acquired By:	Saneesh
Sample Type:	Unknown	Date Acquired:	2017/09/07 03:31:39 PM
Vial:	6	Acq. Method Set:	HDI_EFV_E8H_NEO
Injection #:	1	Date Processed:	2017/09/08 05:13:38 PM
Injection Volume:	15.00 ul	Processing Method:	HDI_EFV_ W2996 245.0nm-1.2
Run Time:	10.5 Minutes	Channel Name:	W2996 245.0nm-1.2
Sample Set Name:	EFV_Basil MeOH TDI IC50 7_9_17	Proc. Chnl. Descr.:	W2996 PDA 245.0 nm at 1.2

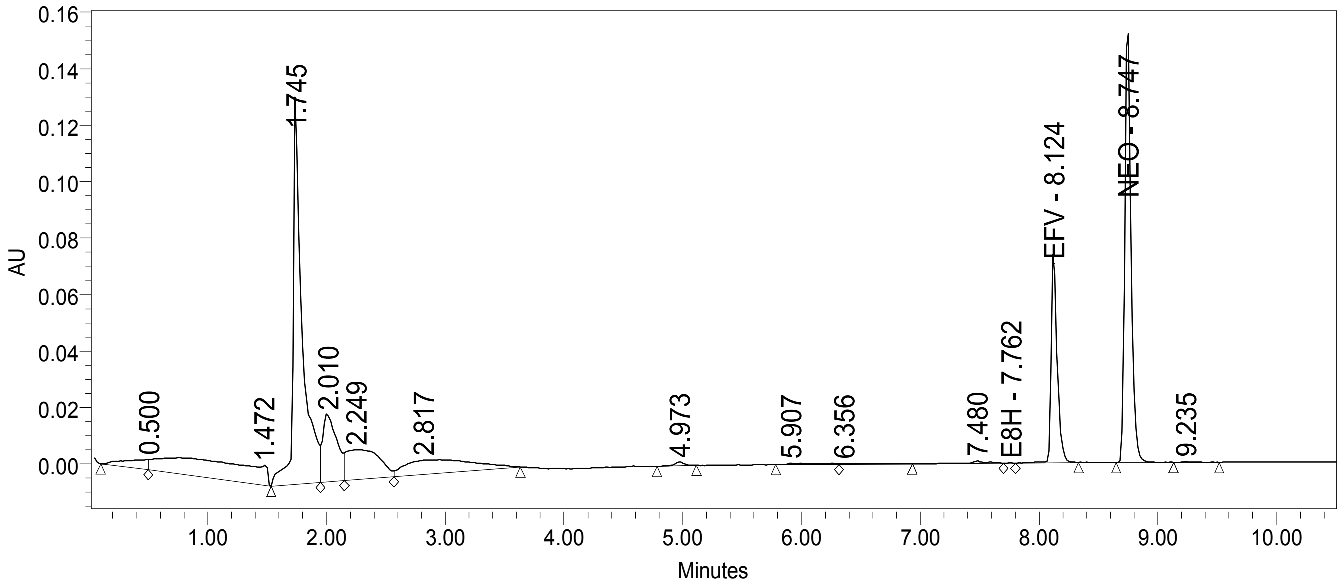

	Peak Name	RT	Area	% Area	Height
1		0.500	47166	1.82	3630
2		1.472	348139	13.40	7616
3		1.745	744394	28.65	121371
4		2.010	199193	7.67	24095
5		2.249	203266	7.82	10495
6		2.817	196975	7.58	4986
7		4.973	8660	0.33	1357
8		5.907	7022	0.27	374
9		6.356	3754	0.14	198
10		7.480	8183	0.31	817
11	E8H	7.762	356	0.01	81
12	EFV	8.124	259640	9.99	68386

Reported by User: Saneesh (Saneesh)   Project Name: HDI_Stellenbosch_2016

	Peak Name	RT	Area	% Area	Height
13	NEO	8.747	569520	21.92	147673
14		9.235	2024	0.08	286

Reported by User: Saneesh (Saneesh)   Project Name: HDI_Stellenbosch_2016

SAMPLE INFORMATION

Sample Name:	EFV Inc Basil_MeOH 50uG/mL TDI2	Acquired By:	Saneesh
Sample Type:	Unknown	Date Acquired:	2017/09/07 03:45:26 PM
Vial:	7	Acq. Method Set:	HDI_EFV_E8H_NEO
Injection #:	1	Date Processed:	2017/09/08 05:13:38 PM
Injection Volume:	15.00 ul	Processing Method:	HDI_EFV_ W2996 245.0nm-1.2
Run Time:	10.5 Minutes	Channel Name:	W2996 245.0nm-1.2
Sample Set Name:	EFV_Basil MeOH TDI IC50 7_9_17	Proc. Chnl. Descr.:	W2996 PDA 245.0 nm at 1.2

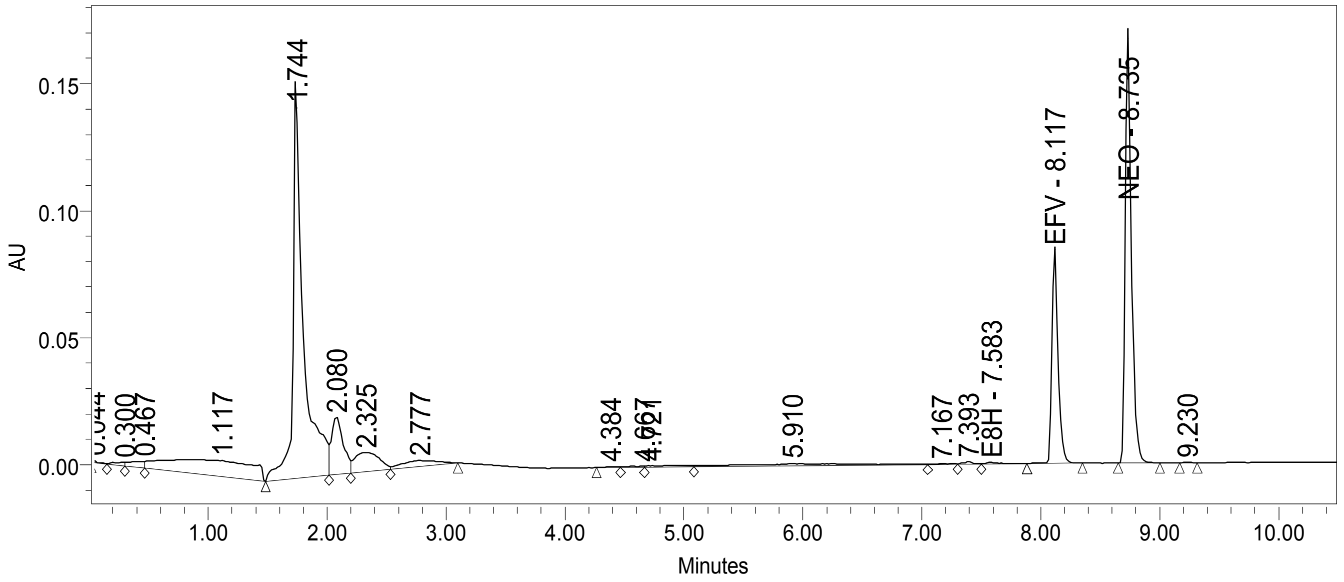

	Peak Name	RT	Area	% Area	Height
1		0.044	2752	0.11	673
2		0.300	8523	0.33	1511
3		0.467	20888	0.81	2697
4		1.117	306862	11.93	6231
5		1.744	925278	35.98	139526
6		2.080	155239	6.04	22579
7		2.325	107657	4.19	7506
8		2.777	49247	1.92	2329
9		4.384	1976	0.08	295
10		4.667	3023	0.12	362
11		4.721	11082	0.43	494
12		5.910	51347	2.00	868

	Peak Name	RT	Area	% Area	Height
13		7.167	2680	0.10	249
14		7.393	5219	0.20	838
15	E8H	7.583	4246	0.17	550
16	EFV	8.117	298305	11.60	80297
17	NEO	8.735	616300	23.97	160976
18		9.230	929	0.04	234

SAMPLE INFORMATION

Sample Name:	EFV Inc Basil_MeOH 100ug/mL TDI1	Acquired By:	Saneesh
Sample Type:	Unknown	Date Acquired:	2017/09/07 03:59:20 PM
Vial:	8	Acq. Method Set:	HDI_EFV_E8H_NEO
Injection #:	1	Date Processed:	2017/09/08 05:13:38 PM
Injection Volume:	15.00 ul	Processing Method:	HDI_EFV_ W2996 245.0nm-1.2
Run Time:	10.5 Minutes	Channel Name:	W2996 245.0nm-1.2
Sample Set Name:	EFV_Basil MeOH TDI IC50 7_9_17	Proc. Chnl. Descr.:	W2996 PDA 245.0 nm at 1.2

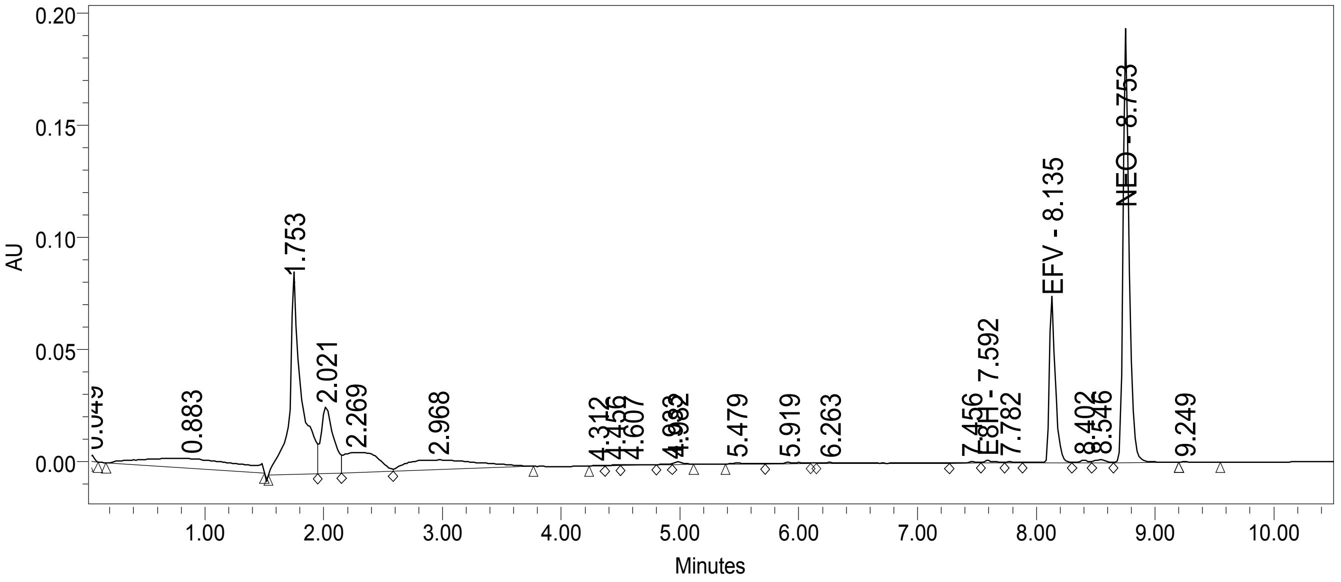

	Peak Name	RT	Area	% Area	Height
1		0.049	6574	0.26	2478
2		0.883	253877	10.11	4371
3		1.753	636943	25.37	84481
4		2.021	216715	8.63	29473
5		2.269	175403	6.99	8846
6		2.968	185172	7.38	4362
7		4.312	966	0.04	147
8		4.456	2172	0.09	425
9		4.607	4422	0.18	364
10		4.933	1472	0.06	357
11		4.982	6212	0.25	1234
12		5.479	4161	0.17	530

Reported by User: Saneesh (Saneesh)   Project Name: HDI_Stellenbosch_2016

	Peak Name	RT	Area	% Area	Height
13		5.919	7560	0.30	662
14		6.263	14467	0.58	511
15		7.456	4703	0.19	665
16	E8H	7.592	7170	0.29	1095
17		7.782	3726	0.15	666
18	EFV	8.135	261567	10.42	69749
19		8.402	6209	0.25	1178
20		8.546	8896	0.35	1463
21	NEO	8.753	699975	27.88	181307
22		9.249	1954	0.08	309

Multi Sample Summary

Reported by User: Saneesh (Saneesh)   Project Name: HDI_Stellenbosch_2016

SAMPLE INFORMATION

Sample Name:	EFV Inc Basil_MeOH 100ug/mL TDI2	Acquired By:	Saneesh
Sample Type:	Unknown	Date Acquired:	2017/09/07 04:13:17 PM
Vial:	9	Acq. Method Set:	HDI_EFV_E8H_NEO
Injection #:	1	Date Processed:	2017/09/08 05:13:38 PM
Injection Volume:	15.00 ul	Processing Method:	HDI_EFV_ W2996 245.0nm-1.2
Run Time:	10.5 Minutes	Channel Name:	W2996 245.0nm-1.2
Sample Set Name:	EFV_Basil MeOH TDI IC50 7_9_17	Proc. Chnl. Descr.:	W2996 PDA 245.0 nm at 1.2

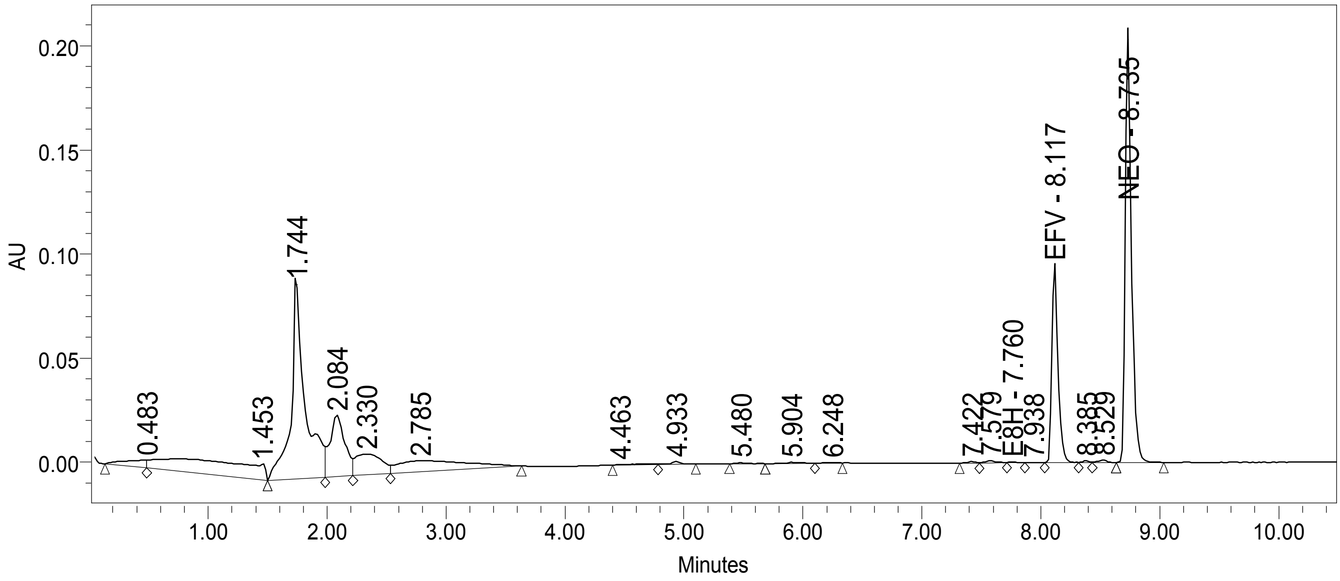

	Peak Name	RT	Area	% Area	Height
1		0.483	46090	1.58	3752
2		1.453	364027	12.47	7864
3		1.744	754348	25.83	88854
4		2.084	256179	8.77	29492
5		2.330	152298	5.22	9967
6		2.785	207525	7.11	5245
7		4.463	4866	0.17	336
8		4.933	6561	0.22	1153
9		5.480	2970	0.10	507
10		5.904	5774	0.20	638
11		6.248	2912	0.10	359
12		7.422	2734	0.09	632

Reported by User: Saneesh (Saneesh) Project Name: HDI_Stellenbosch_2016

	Peak Name	RT	Area	% Area	Height
13		7.579	6957	0.24	1053
14	E8H	7.760	2348	0.08	489
15		7.938	1591	0.05	219
16	EFV	8.117	336064	11.51	90516
17		8.385	3529	0.12	883
18		8.529	6303	0.22	1186
19	NEO	8.735	757303	25.93	196430

SAMPLE INFORMATION

Sample Name:	EFV Inc Basil_MeOH 150ug/mL TDI1	Acquired By:	Saneesh
Sample Type:	Unknown	Date Acquired:	2017/09/07 04:27:13 PM
Vial:	10	Acq. Method Set:	HDI_EFV_E8H_NEO
Injection #:	1	Date Processed:	2017/09/08 05:13:38 PM
Injection Volume:	15.00 ul	Processing Method:	HDI_EFV_ W2996 245.0nm-1.2
Run Time:	10.5 Minutes	Channel Name:	W2996 245.0nm-1.2
Sample Set Name:	EFV_Basil MeOH TDI IC50 7_9_17	Proc. Chnl. Descr.:	W2996 PDA 245.0 nm at 1.2

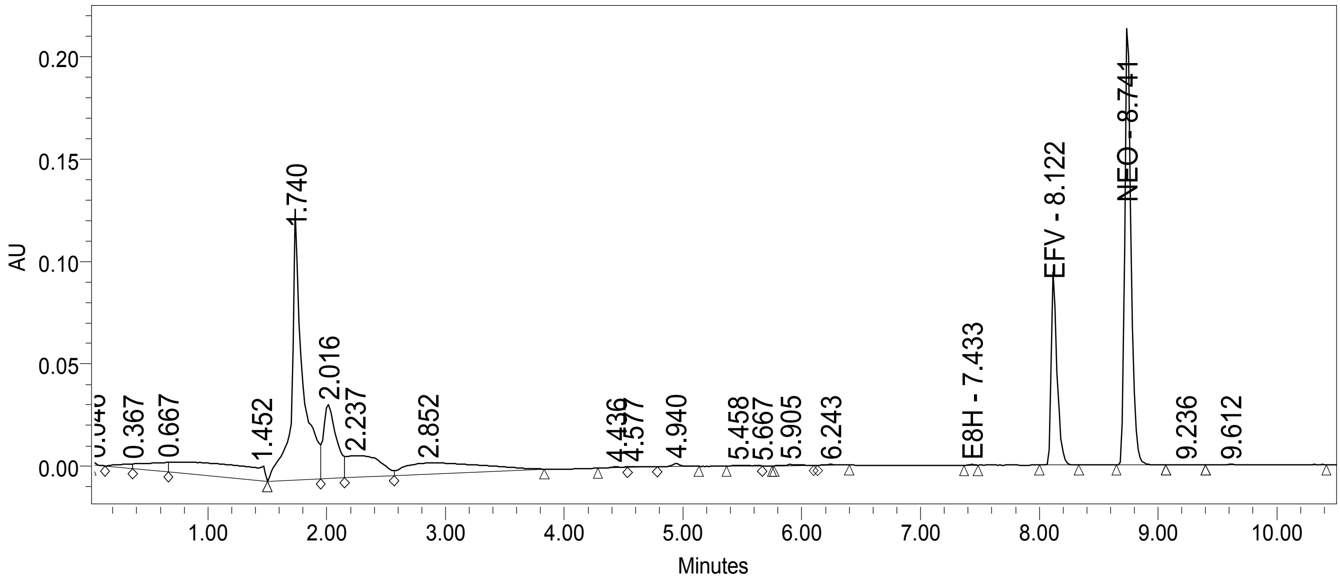

	Peak Name	RT	Area	% Area	Height
1		0.046	2606	0.08	928
2		0.367	18337	0.59	2370
3		0.667	63251	2.03	4629
4		1.452	293066	9.42	7194
5		1.740	853042	27.42	117149
6		2.016	270471	8.69	35753
7		2.237	206481	6.64	10454
8		2.852	241694	7.77	5386
9		4.436	3016	0.10	418
10		4.577	3746	0.12	405
11		4.940	8599	0.28	1552
12		5.458	4415	0.14	450

Reported by User: Saneesh (Saneesh) Project Name: HDI_Stellenbosch_2016

	Peak Name	RT	Area	% Area	Height
13		5.667	182	0.01	97
14		5.905	5502	0.18	733
15		6.243	3442	0.11	549
16	E8H	7.433	1278	0.04	431
17	EFV	8.122	322889	10.38	86744
18	NEO	8.741	801183	25.75	203397
19		9.236	2086	0.07	336
20		9.612	6272	0.20	384

Reported by User: Saneesh (Saneesh) Project Name: HDI_Stellenbosch_2016

SAMPLE INFORMATION

Sample Name:	EFV Inc Basil_MeOH 150ug/mL TDI2	Acquired By:	Saneesh
Sample Type:	Unknown	Date Acquired:	2017/09/07 04:41:08 PM
Vial:	11	Acq. Method Set:	HDI_EFV_E8H_NEO
Injection #:	1	Date Processed:	2017/09/08 05:13:38 PM
Injection Volume:	15.00 ul	Processing Method:	HDI_EFV_ W2996 245.0nm-1.2
Run Time:	10.5 Minutes	Channel Name:	W2996 245.0nm-1.2
Sample Set Name:	EFV_Basil MeOH TDI IC50 7_9_17	Proc. Chnl. Descr.:	W2996 PDA 245.0 nm at 1.2

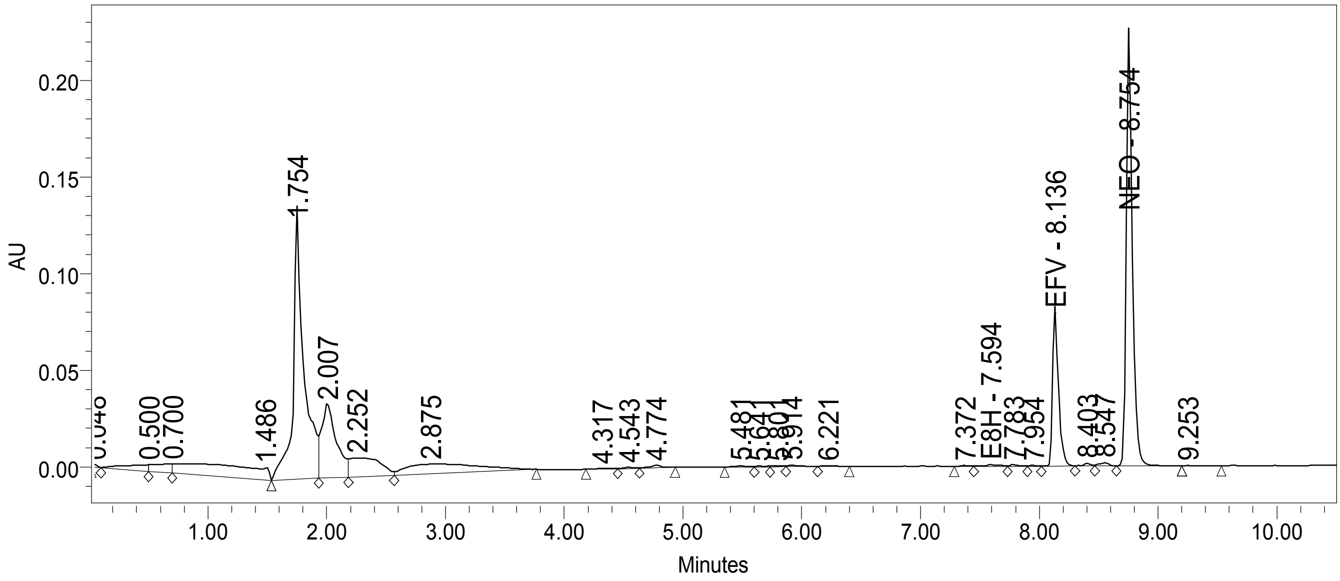

	Peak Name	RT	Area	% Area	Height
1		0.048	3674	0.12	1363
2		0.500	45496	1.44	3312
3		0.700	47991	1.52	4584
4		1.486	278059	8.82	6578
5		1.754	881613	27.97	131770
6		2.007	333884	10.59	37550
7		2.252	173404	5.50	9674
8		2.875	215113	6.82	4920
9		4.317	3774	0.12	321
10		4.543	4079	0.13	564
11		4.774	8432	0.27	1280
12		5.481	4870	0.15	692

Reported by User: Saneesh (Saneesh) Project Name: HDI_Stellenbosch_2016

	Peak Name	RT	Area	% Area	Height
13		5.641	3010	0.10	475
14		5.801	3851	0.12	506
15		5.914	6406	0.20	833
16		6.221	3209	0.10	449
17		7.372	2085	0.07	446
18	E8H	7.594	7350	0.23	905
19		7.783	3706	0.12	752
20		7.954	1966	0.06	412
21	EFV	8.136	281325	8.92	76548
22		8.403	6747	0.21	1449
23		8.547	10253	0.33	1794
24	NEO	8.754	820247	26.02	211787
25		9.253	1565	0.05	237

SAMPLE INFORMATION

Sample Name:	EFV Inc Basil_MeOH 200ug/mL TDI1	Acquired By:	Saneesh
Sample Type:	Unknown	Date Acquired:	2017/09/07 04:55:02 PM
Vial:	12	Acq. Method Set:	HDI_EFV_E8H_NEO
Injection #:	1	Date Processed:	2017/09/08 05:13:38 PM
Injection Volume:	15.00 ul	Processing Method:	HDI_EFV_ W2996 245.0nm-1.2
Run Time:	10.5 Minutes	Channel Name:	W2996 245.0nm-1.2
Sample Set Name:	EFV_Basil MeOH TDI IC50 7_9_17	Proc. Chnl. Descr.:	W2996 PDA 245.0 nm at 1.2

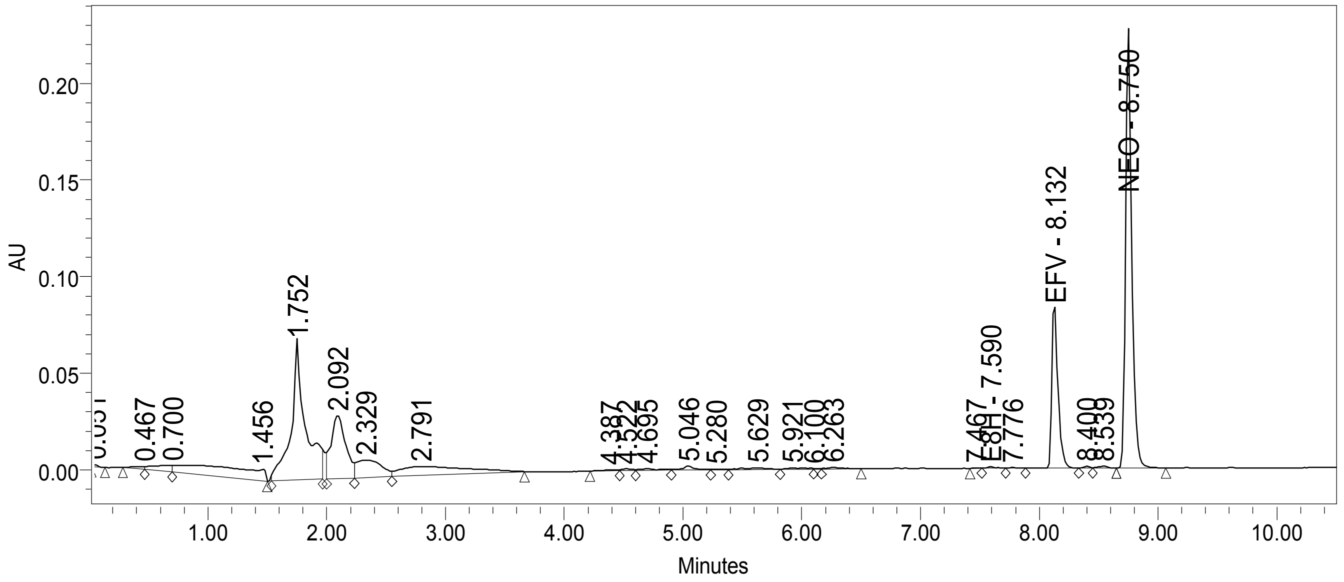

	Peak Name	RT	Area	% Area	Height
1		0.051	3795	0.14	1236
2		0.467	8518	0.32	1521
3		0.700	34842	1.29	3374
4		1.456	225753	8.38	6375
5		1.752	602781	22.36	66944
6		2.092	281548	10.45	32857
7		2.329	131596	4.88	8903
8		2.791	180831	6.71	4611
9		4.387	4647	0.17	542
10		4.522	6400	0.24	1049
11		4.695	11910	0.44	1020
12		5.046	16447	0.61	2062

Reported by User: Saneesh (Saneesh)   Project Name: HDI_Stellenbosch_2016

	Peak Name	RT	Area	% Area	Height
13		5.280	3051	0.11	403
14		5.629	13609	0.50	912
15		5.921	7914	0.29	721
16		6.100	1170	0.04	362
17		6.263	6657	0.25	840
18		7.467	695	0.03	219
19	E8H	7.590	3513	0.13	714
20		7.776	1622	0.06	354
21	EFV	8.132	306566	11.37	80907
22		8.400	3636	0.13	915
23		8.539	5243	0.19	886
24	NEO	8.750	832644	30.89	215627

SAMPLE INFORMATION

Sample Name:	EFV Inc Basil_MeOH 200ug/mL TDI2	Acquired By:	Saneesh
Sample Type:	Unknown	Date Acquired:	2017/09/07 05:08:59 PM
Vial:	13	Acq. Method Set:	HDI_EFV_E8H_NEO
Injection #:	1	Date Processed:	2017/09/08 05:13:38 PM
Injection Volume:	15.00 ul	Processing Method:	HDI_EFV_ W2996 245.0nm-1.2
Run Time:	10.5 Minutes	Channel Name:	W2996 245.0nm-1.2
Sample Set Name:	EFV_Basil MeOH TDI IC50 7_9_17	Proc. Chnl. Descr.:	W2996 PDA 245.0 nm at 1.2

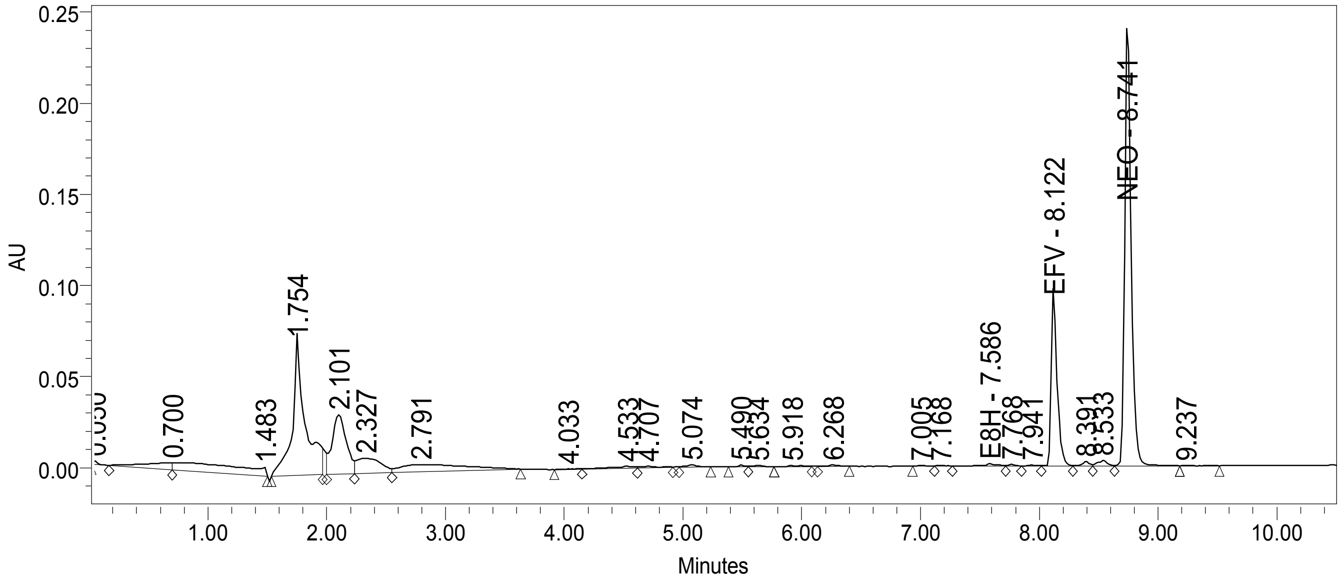

	Peak Name	RT	Area	% Area	Height
1		0.050	4692	0.17	1612
2		0.700	62830	2.29	3775
3		1.483	200522	7.32	4883
4		1.754	567941	20.72	69651
5		2.101	276966	10.11	32819
6		2.327	121229	4.42	8303
7		2.791	154768	5.65	4097
8		4.033	2164	0.08	250
9		4.533	15478	0.56	1156
10		4.707	9924	0.36	939
11		5.074	8883	0.32	1314
12		5.490	3730	0.14	900

Reported by User: Saneesh (Saneesh) Project Name: HDI_Stellenbosch_2016

	Peak Name	RT	Area	% Area	Height
13		5.634	4239	0.15	712
14		5.918	4024	0.15	553
15		6.268	4562	0.17	766
16		7.005	2194	0.08	444
17		7.168	1822	0.07	279
18	E8H	7.586	10926	0.40	1328
19		7.768	3745	0.14	985
20		7.941	2796	0.10	502
21	EFV	8.122	333582	12.17	89250
22		8.391	10512	0.38	2267
23		8.533	16182	0.59	2822
24	NEO	8.741	915080	33.39	229322
25		9.237	1644	0.06	229

Reported by User: Saneesh (Saneesh)   Project Name: HDI_Stellenbosch_2016

SAMPLE INFORMATION

Sample Name:	RIF Basil Test Control Inc K1	Acquired By:	Saneesh
Sample Type:	Unknown	Date Acquired:	2017/09/11 01:55:55 PM
Vial:	22	Acq. Method Set:	HDI_RIF_25ODESRIF_NEO
Injection #:	1	Date Processed:	2017/09/12 11:45:40 AM
Injection Volume:	15.00 ul	Processing Method:	HDI_RIF_ W2996 254.0nm-1.2
Run Time:	11.5 Minutes	Channel Name:	W2996 254.0nm-1.2
Sample Set Name:	RIF_Basil EtOH IC50 11_9_17	Proc. Chnl. Descr.:	W2996 PDA 254.0 nm at 1.2

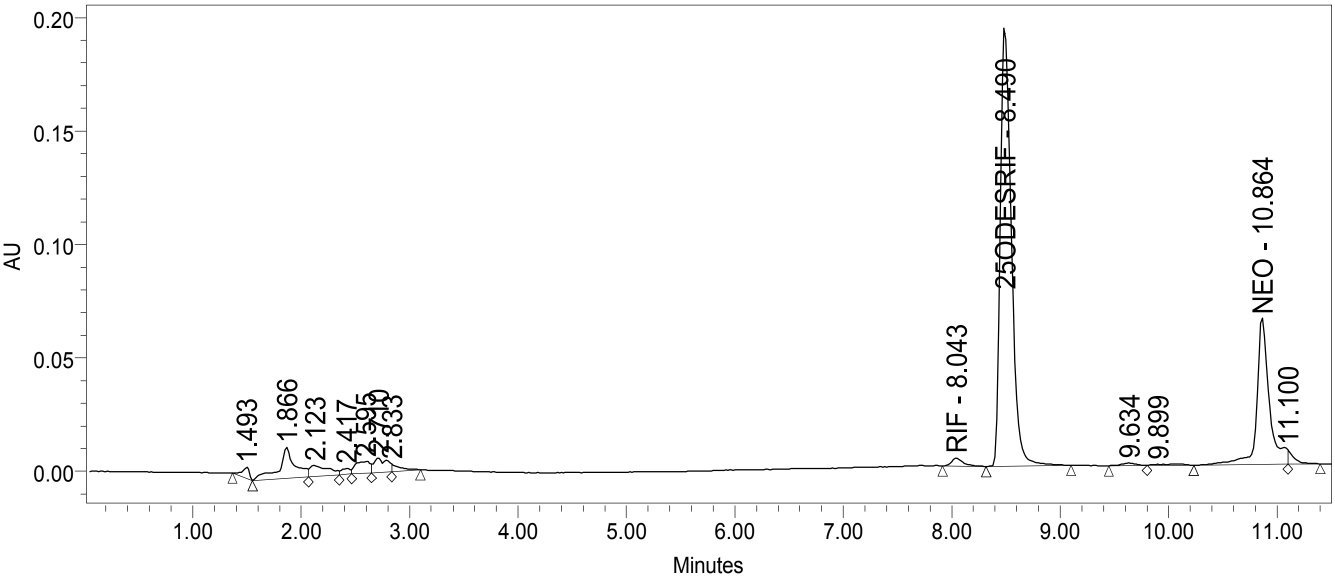

	Peak Name	RT	Area	% Area	Height
1		1.493	22454	0.97	4861
2		1.866	139645	6.01	13281
3		2.123	57457	2.47	4877
4		2.417	15208	0.66	2605
5		2.595	48938	2.11	5162
6		2.710	51891	2.23	6194
7		2.833	17273	0.74	3320
8	RIF	8.043	25656	1.11	3599
9	25ODESRIF	8.490	1380096	59.44	194611
10		9.634	9671	0.42	1165
11		9.899	8721	0.38	448
12	NEO	10.864	519427	22.37	64220
13		11.100	25351	1.09	6355

Reported by User: Saneesh (Saneesh)   Project Name: HDI_Stellenbosch_2016

SAMPLE INFORMATION

Sample Name:	RIF Basil Test Control Inc K2	Acquired By:	Saneesh
Sample Type:	Unknown	Date Acquired:	2017/09/11 02:10:38 PM
Vial:	23	Acq. Method Set:	HDI_RIF_25ODESRIF_NEO
Injection #:	1	Date Processed:	2017/09/12 11:45:40 AM
Injection Volume:	15.00 ul	Processing Method:	HDI_RIF_ W2996 254.0nm-1.2
Run Time:	11.5 Minutes	Channel Name:	W2996 254.0nm-1.2
Sample Set Name:	RIF_Basil EtOH IC50 11_9_17	Proc. Chnl. Descr.:	W2996 PDA 254.0 nm at 1.2

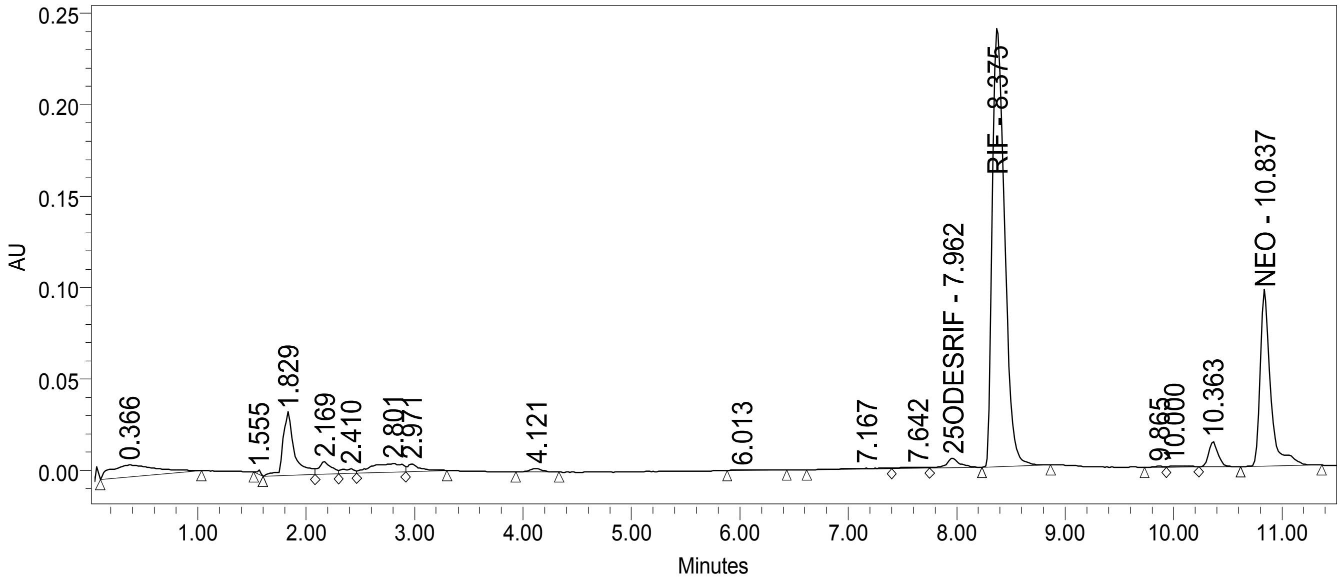

	Peak Name	RT	Area	% Area	Height
1		0.366	197181	5.82	6511
2		1.555	4880	0.14	1693
3		1.829	257605	7.61	33273
4		2.169	55795	1.65	6985
5		2.410	21636	0.64	2603
6		2.801	96078	2.84	4658
7		2.971	33486	0.99	4120
8		4.121	15715	0.46	1996
9		6.013	5574	0.16	249
10		7.167	9173	0.27	337
11		7.642	7719	0.23	427
12	25ODESRIF	7.962	41414	1.22	4969

	Peak Name	RT	Area	% Area	Height
13	RIF	8.375	1897577	56.03	242118
14		9.865	3025	0.09	492
15		10.000	7544	0.22	616
16		10.363	83282	2.46	13591
17	NEO	10.837	649286	19.17	96217

Reported by User: Saneesh (Saneesh) Project Name: HDI_Stellenbosch_2016

SAMPLE INFORMATION

Sample Name:	RIF Basil Test Control Inc K3	Acquired By:	Saneesh
Sample Type:	Unknown	Date Acquired:	2017/09/11 02:25:20 PM
Vial:	24	Acq. Method Set:	HDI_RIF_25ODESRIF_NEO
Injection #:	1	Date Processed:	2017/09/12 11:45:40 AM
Injection Volume:	15.00 ul	Processing Method:	HDI_RIF_ W2996 254.0nm-1.2
Run Time:	11.5 Minutes	Channel Name:	W2996 254.0nm-1.2
Sample Set Name:	RIF_Basil EtOH IC50 11_9_17	Proc. Chnl. Descr.:	W2996 PDA 254.0 nm at 1.2

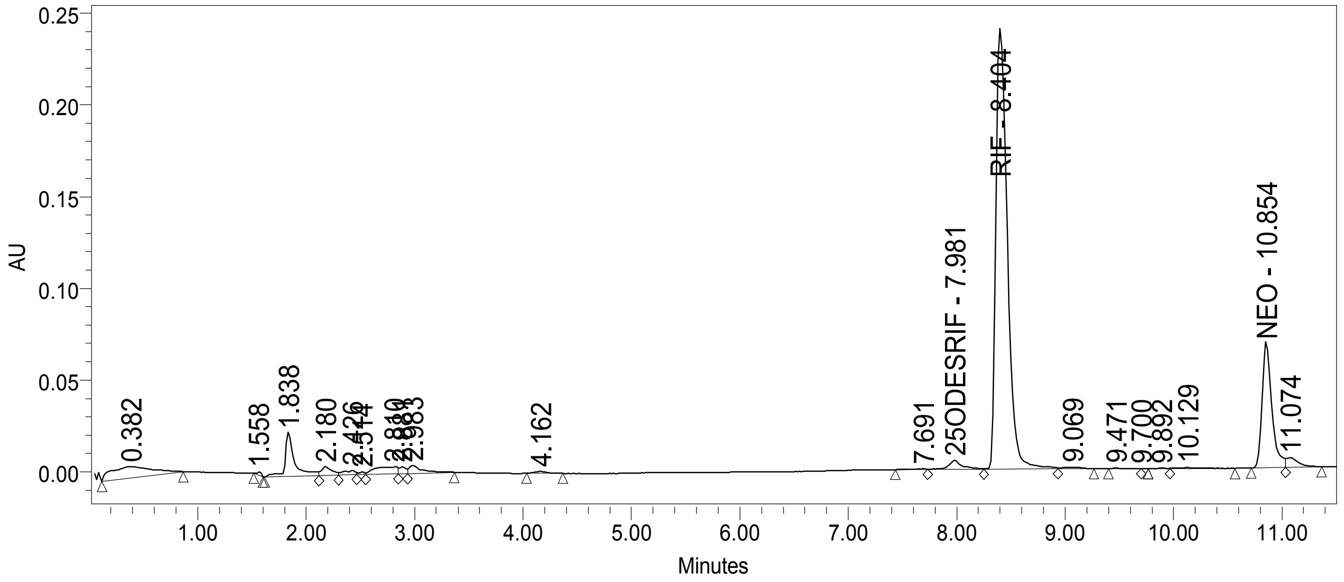

	Peak Name	RT	Area	% Area	Height
1		0.382	160829	5.73	6018
2		1.558	4962	0.18	1718
3		1.838	147919	5.27	23604
4		2.180	33212	1.18	4703
5		2.426	19028	0.68	2371
6		2.514	5831	0.21	1369
7		2.810	57336	2.04	3830
8		2.881	15864	0.57	3643
9		2.983	36278	1.29	4231
10		4.162	7462	0.27	1003
11		7.691	4248	0.15	327
12	25ODESRIF	7.981	42135	1.50	4708

Reported by User: Saneesh (Saneesh) Project Name: HDI_Stellenbosch_2016

	Peak Name	RT	Area	% Area	Height
13	RIF	8.404	1774023	63.24	240598
14		9.069	10406	0.37	832
15		9.471	4346	0.15	466
16		9.700	407	0.01	172
17		9.892	2900	0.10	346
18		10.129	9419	0.34	561
19	NEO	10.854	427879	15.25	68323
20		11.074	40770	1.45	5225

SAMPLE INFORMATION

Sample Name:	RIF Inc Basil_EtOH 1ug/mL 1	Acquired By:	Saneesh
Sample Type:	Unknown	Date Acquired:	2017/09/11 02:40:04 PM
Vial:	2	Acq. Method Set:	HDI_RIF_25ODESRIF_NEO
Injection #:	1	Date Processed:	2017/09/12 11:45:40 AM
Injection Volume:	15.00 ul	Processing Method:	HDI_RIF_ W2996 254.0nm-1.2
Run Time:	11.5 Minutes	Channel Name:	W2996 254.0nm-1.2
Sample Set Name:	RIF_Basil EtOH IC50 11_9_17	Proc. Chnl. Descr.:	W2996 PDA 254.0 nm at 1.2

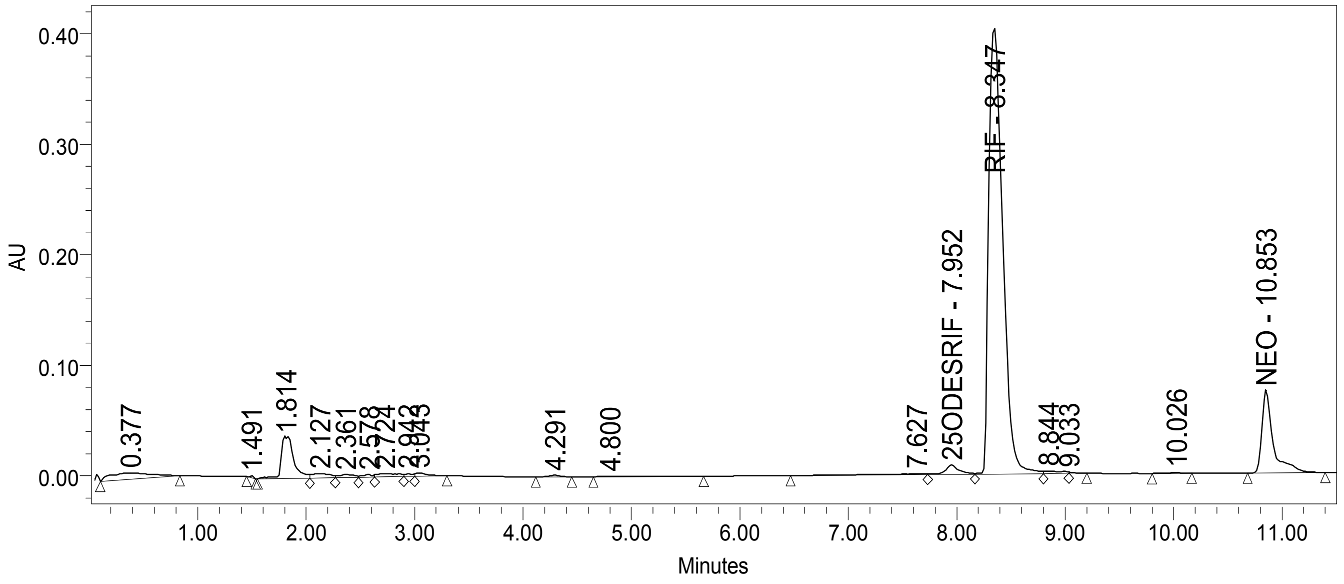

	Peak Name	RT	Area	% Area	Height
1		0.377	147907	3.01	5712
2		1.491	4891	0.10	1687
3		1.814	309872	6.31	39570
4		2.127	45080	0.92	3941
5		2.361	26062	0.53	2695
6		2.578	14632	0.30	2175
7		2.724	39926	0.81	2761
8		2.942	13160	0.27	2463
9		3.043	22792	0.46	3107
10		4.291	10235	0.21	1413
11		4.800	11730	0.24	543
12		7.627	29274	0.60	943

Reported by User: Saneesh (Saneesh) Project Name: HDI_Stellenbosch_2016

	Peak Name	RT	Area	% Area	Height
13	25ODESRIF	7.952	72942	1.49	8330
14	RIF	8.347	3580087	72.91	403512
15		8.844	25163	0.51	2274
16		9.033	3727	0.08	1090
17		10.026	5727	0.12	598
18	NEO	10.853	546757	11.14	75054

SAMPLE INFORMATION

Sample Name:	RIF Inc Basil_EtOH 1ug/mL 2	Acquired By:	Saneesh
Sample Type:	Unknown	Date Acquired:	2017/09/11 02:54:47 PM
Vial:	3	Acq. Method Set:	HDI_RIF_25ODESRIF_NEO
Injection #:	1	Date Processed:	2017/09/12 11:45:40 AM
Injection Volume:	15.00 ul	Processing Method:	HDI_RIF_ W2996 254.0nm-1.2
Run Time:	11.5 Minutes	Channel Name:	W2996 254.0nm-1.2
Sample Set Name:	RIF_Basil EtOH IC50 11_9_17	Proc. Chnl. Descr.:	W2996 PDA 254.0 nm at 1.2

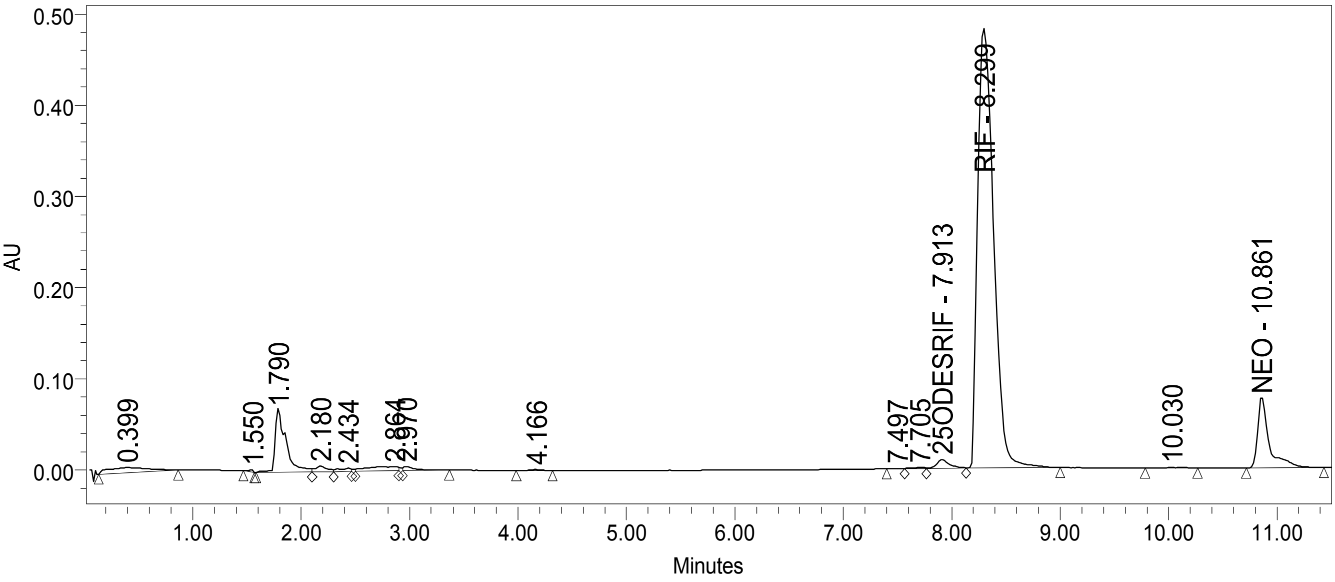

	Peak Name	RT	Area	% Area	Height
1		0.399	144152	2.24	5426
2		1.550	5662	0.09	1934
3		1.790	481485	7.47	69118
4		2.180	46437	0.72	6002
5		2.434	26916	0.42	3305
6		2.864	91223	1.42	4516
7		2.970	35872	0.56	4415
8		4.166	9216	0.14	1335
9		7.497	2809	0.04	386
10		7.705	9827	0.15	1135
11	25ODESRIF	7.913	77588	1.20	9857
12	RIF	8.299	4935745	76.62	481943

Reported by User: Saneesh (Saneesh) Project Name: HDI_Stellenbosch_2016

	Peak Name	RT	Area	% Area	Height
13		10.030	6472	0.10	493
14	NEO	10.861	568109	8.82	76656

Reported by User: Saneesh (Saneesh) Project Name: HDI_Stellenbosch_2016

SAMPLE INFORMATION

Sample Name:	RIF Inc Basil_EtOH 1ug/mL 3	Acquired By:	Saneesh
Sample Type:	Unknown	Date Acquired:	2017/09/11 03:09:29 PM
Vial:	4	Acq. Method Set:	HDI_RIF_25ODESRIF_NEO
Injection #:	1	Date Processed:	2017/09/12 11:45:40 AM
Injection Volume:	15.00 ul	Processing Method:	HDI_RIF_ W2996 254.0nm-1.2
Run Time:	11.5 Minutes	Channel Name:	W2996 254.0nm-1.2
Sample Set Name:	RIF_Basil EtOH IC50 11_9_17	Proc. Chnl. Descr.:	W2996 PDA 254.0 nm at 1.2

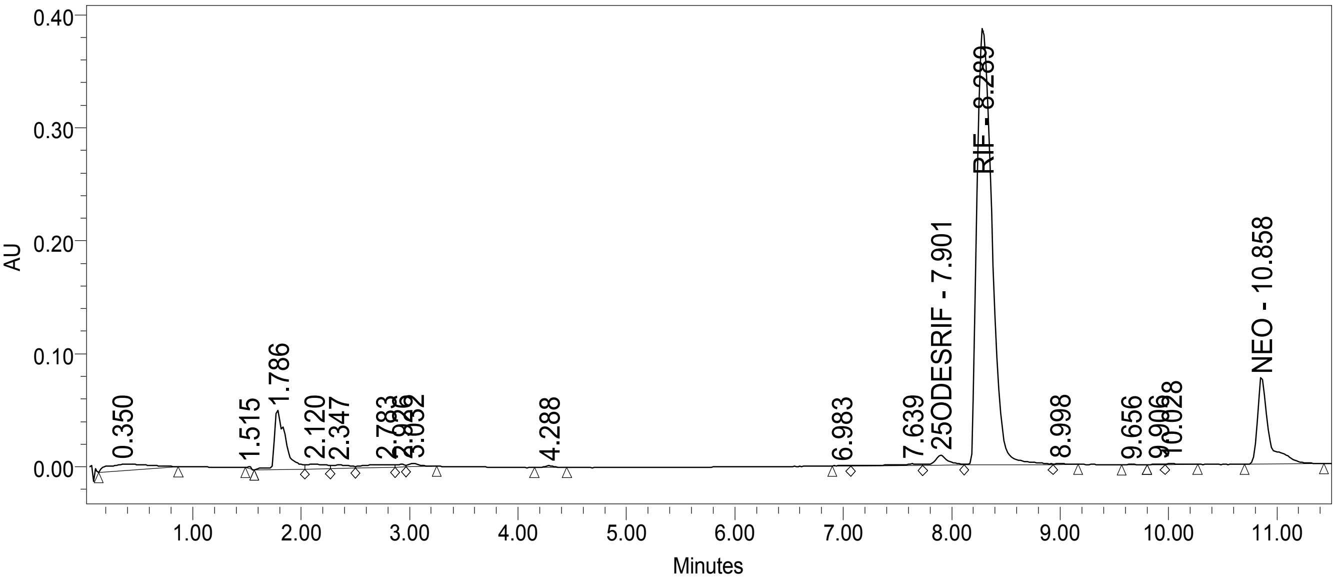

	Peak Name	RT	Area	% Area	Height
1		0.350	148145	2.89	5636
2		1.515	3802	0.07	1269
3		1.786	378846	7.38	52180
4		2.120	52225	1.02	4339
5		2.347	33735	0.66	3069
6		2.783	49205	0.96	2655
7		2.926	13491	0.26	2633
8		3.032	21765	0.42	3042
9		4.288	9672	0.19	1562
10		6.983	1570	0.03	231
11		7.639	23949	0.47	1519
12	25ODESRIF	7.901	75003	1.46	8935

	Peak Name	RT	Area	% Area	Height
13	RIF	8.289	3743404	72.90	387652
14		8.998	5702	0.11	812
15		9.656	1513	0.03	269
16		9.906	1946	0.04	278
17		10.028	6541	0.13	667
18	NEO	10.858	564264	10.99	76691

Reported by User: Saneesh (Saneesh) Project Name: HDI_Stellenbosch_2016

SAMPLE INFORMATION

Sample Name:	RIF Inc Basil_EtOH 10ug/mL 1	Acquired By:	Saneesh
Sample Type:	Unknown	Date Acquired:	2017/09/11 03:24:14 PM
Vial:	5	Acq. Method Set:	HDI_RIF_25ODESRIF_NEO
Injection #:	1	Date Processed:	2017/09/12 11:45:40 AM
Injection Volume:	15.00 ul	Processing Method:	HDI_RIF_ W2996 254.0nm-1.2
Run Time:	11.5 Minutes	Channel Name:	W2996 254.0nm-1.2
Sample Set Name:	RIF_Basil EtOH IC50 11_9_17	Proc. Chnl. Descr.:	W2996 PDA 254.0 nm at 1.2

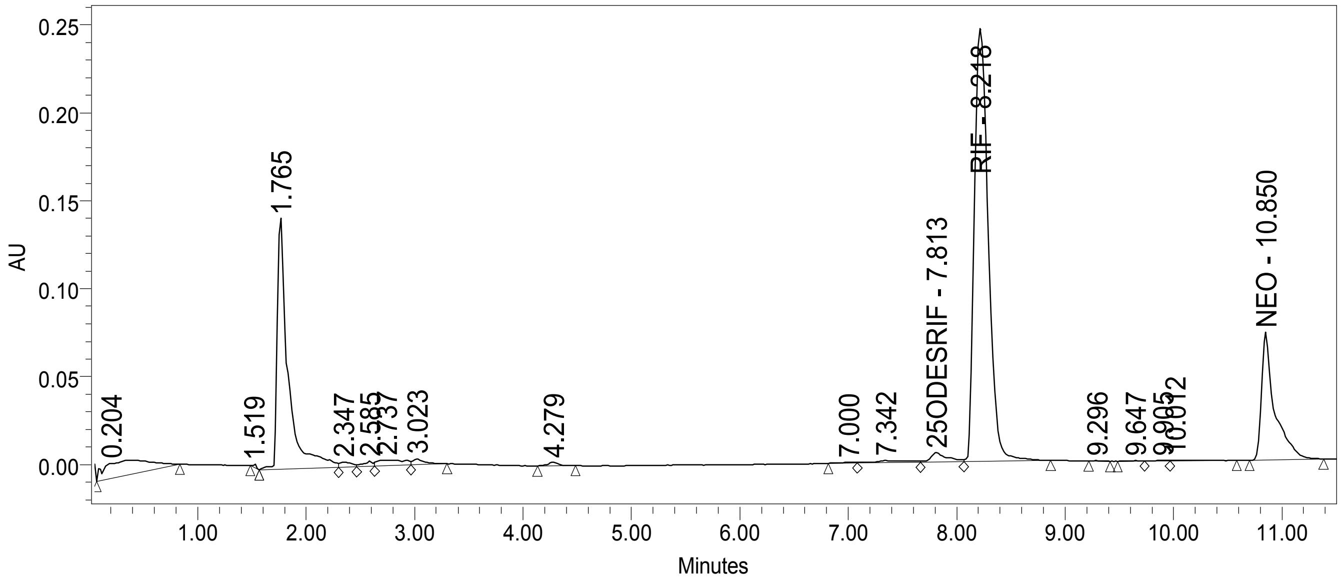

	Peak Name	RT	Area	% Area	Height
1		0.204	246578	5.76	8533
2		1.519	4280	0.10	1462
3		1.765	981483	22.91	139710
4		2.347	21867	0.51	2804
5		2.585	17161	0.40	2553
6		2.737	55985	1.31	3234
7		3.023	25324	0.59	3250
8		4.279	13185	0.31	2039
9		7.000	2404	0.06	259
10		7.342	20514	0.48	1024
11	25ODESRIF	7.813	52921	1.24	5152
12	RIF	8.218	2213104	51.66	245820

	Peak Name	RT	Area	% Area	Height
13		9.296	1725	0.04	308
14		9.647	2324	0.05	327
15		9.905	2873	0.07	317
16		10.012	8514	0.20	476
17	NEO	10.850	613506	14.32	72005

Multi Sample Summary

Reported by User: Saneesh (Saneesh) Project Name: HDI_Stellenbosch_2016

SAMPLE INFORMATION

Sample Name:	RIF Inc Basil_EtOH 10ug/mL 2	Acquired By:	Saneesh
Sample Type:	Unknown	Date Acquired:	2017/09/11 03:38:56 PM
Vial:	6	Acq. Method Set:	HDI_RIF_25ODESRIF_NEO
Injection #:	1	Date Processed:	2017/09/12 11:45:40 AM
Injection Volume:	15.00 ul	Processing Method:	HDI_RIF_ W2996 254.0nm-1.2
Run Time:	11.5 Minutes	Channel Name:	W2996 254.0nm-1.2
Sample Set Name:	RIF_Basil EtOH IC50 11_9_17	Proc. Chnl. Descr.:	W2996 PDA 254.0 nm at 1.2

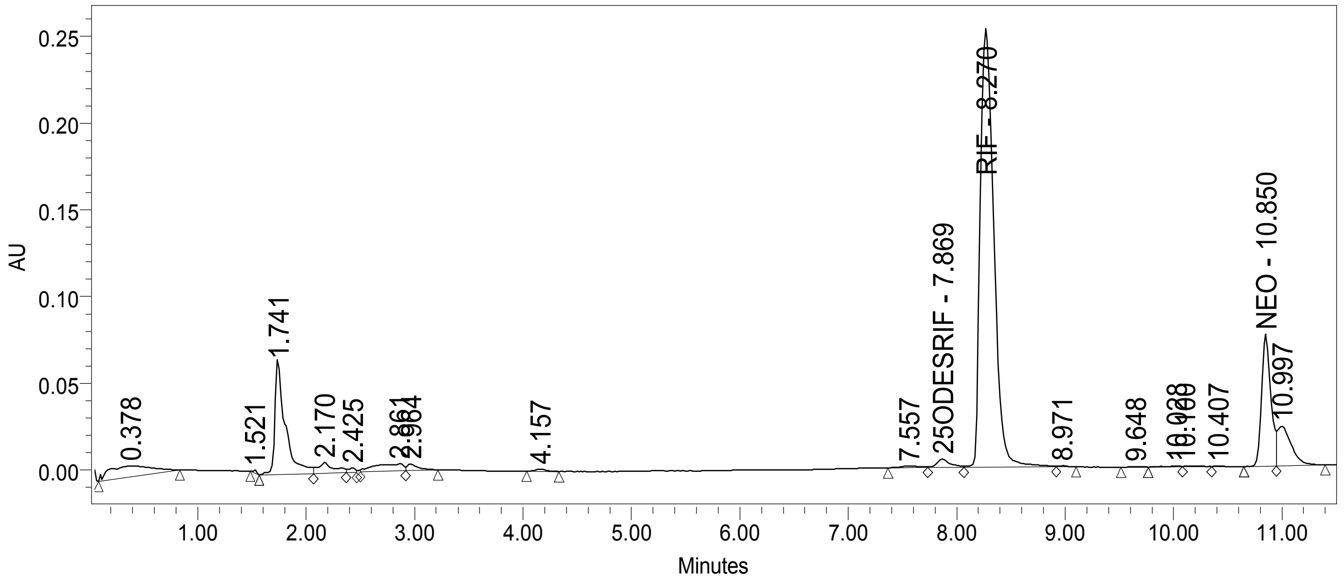

	Peak Name	RT	Area	% Area	Height
1		0.378	173877	4.63	6222
2		1.521	4736	0.13	1636
3		1.741	440433	11.72	64466
4		2.170	63376	1.69	6260
5		2.425	12345	0.33	2528
6		2.861	80416	2.14	4233
7		2.964	26577	0.71	3816
8		4.157	9473	0.25	1394
9		7.557	12397	0.33	939
10	25ODESRIF	7.869	42074	1.12	5034
11	RIF	8.270	2213622	58.90	253207
12		8.971	4309	0.11	640

Reported by User: Saneesh (Saneesh) Project Name: HDI_Stellenbosch_2016

	Peak Name	RT	Area	% Area	Height
13		9.648	1948	0.05	303
14		10.028	5469	0.15	464
15		10.100	5150	0.14	455
16		10.407	2760	0.07	282
17	NEO	10.850	454511	12.09	75496
18		10.997	205005	5.45	22916

Empower software

Multi Sample Summary

Reported by User: Saneesh (Saneesh) Project Name: HDI_Stellenbosch_2016

SAMPLE INFORMATION

Sample Name:	RIF Inc Basil_EtOH 10ug/mL 3	Acquired By:	Saneesh
Sample Type:	Unknown	Date Acquired:	2017/09/11 03:53:38 PM
Vial:	7	Acq. Method Set:	HDI_RIF_25ODESRIF_NEO
Injection #:	1	Date Processed:	2017/09/12 11:45:40 AM
Injection Volume:	15.00 ul	Processing Method:	HDI_RIF_ W2996 254.0nm-1.2
Run Time:	11.5 Minutes	Channel Name:	W2996 254.0nm-1.2
Sample Set Name:	RIF_Basil EtOH IC50 11_9_17	Proc. Chnl. Descr.:	W2996 PDA 254.0 nm at 1.2

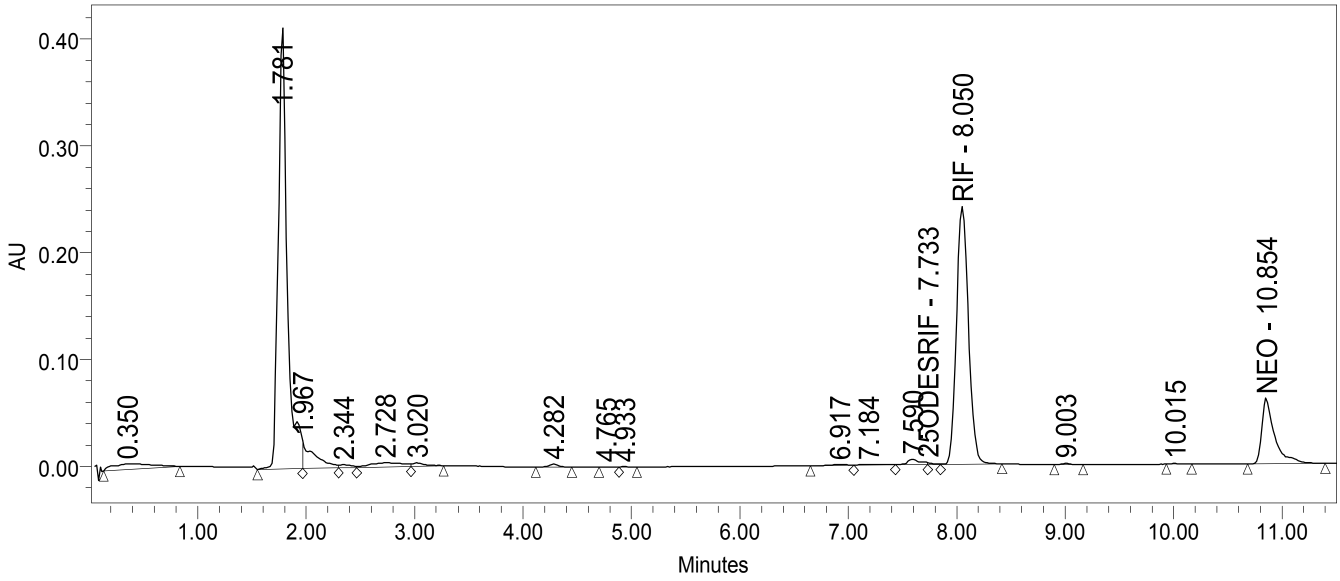

	Peak Name	RT	Area	% Area	Height
1		0.350	134113	2.55	5371
2		1.781	2362192	44.86	401222
3		1.967	192809	3.66	26116
4		2.344	20878	0.40	2671
5		2.728	79456	1.51	3753
6		3.020	23260	0.44	3176
7		4.282	16748	0.32	2671
8		4.765	1810	0.03	263
9		4.933	1660	0.03	372
10		6.917	8932	0.17	867
11		7.184	8886	0.17	566
12		7.590	41388	0.79	5219

	Peak Name	RT	Area	% Area	Height
13	25ODESRIF	7.733	5944	0.11	1766
14	RIF	8.050	1850785	35.15	240191
15		9.003	6588	0.13	1369
16		10.015	4543	0.09	782
17	NEO	10.854	505277	9.60	61517

Reported by User: Saneesh (Saneesh) Project Name: HDI_Stellenbosch_2016

SAMPLE INFORMATION

Sample Name:	RIF Inc Basil_EtOH 50ug/mL 1	Acquired By:	Saneesh
Sample Type:	Unknown	Date Acquired:	2017/09/11 04:08:21 PM
Vial:	8	Acq. Method Set:	HDI_RIF_25ODESRIF_NEO
Injection #:	1	Date Processed:	2017/09/12 11:45:40 AM
Injection Volume:	15.00 ul	Processing Method:	HDI_RIF_ W2996 254.0nm-1.2
Run Time:	11.5 Minutes	Channel Name:	W2996 254.0nm-1.2
Sample Set Name:	RIF_Basil EtOH IC50 11_9_17	Proc. Chnl. Descr.:	W2996 PDA 254.0 nm at 1.2

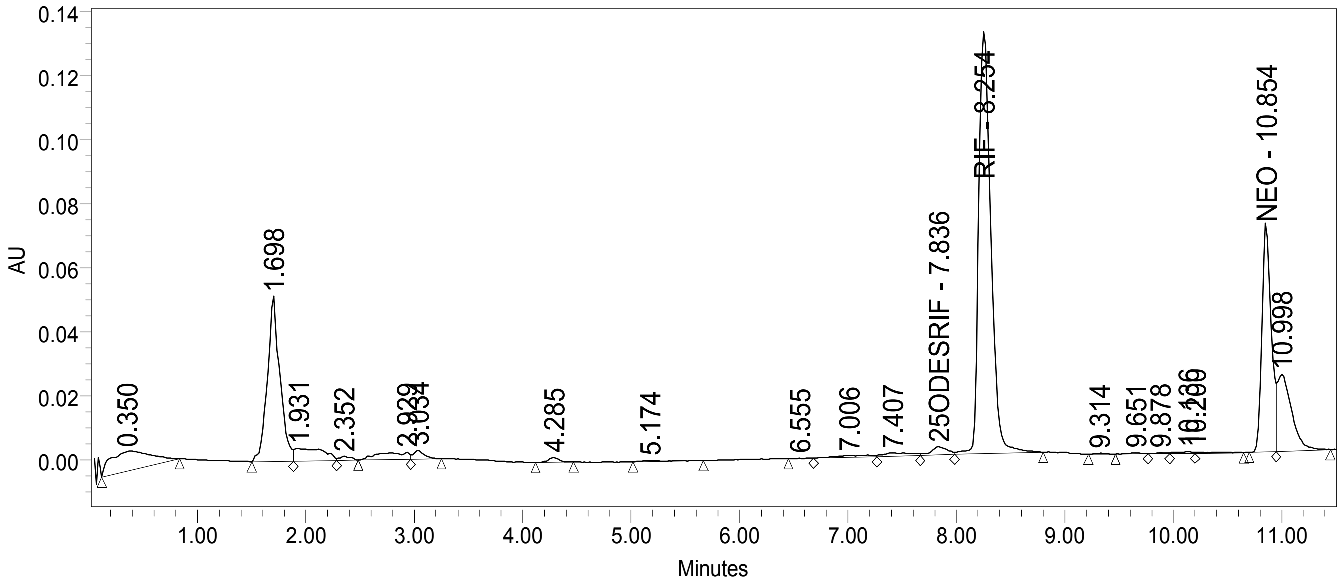

	Peak Name	RT	Area	% Area	Height
1		0.350	160370	6.41	6179
2		1.698	414048	16.54	50807
3		1.931	74080	2.96	3921
4		2.352	9834	0.39	1345
5		2.929	43838	1.75	2174
6		3.034	18992	0.76	2748
7		4.285	9592	0.38	1504
8		5.174	5607	0.22	386
9		6.555	958	0.04	143
10		7.006	12546	0.50	553
11		7.407	17763	0.71	992
12	25ODESRIF	7.836	26017	1.04	2578

	Peak Name	RT	Area	% Area	Height
13	RIF	8.254	1039187	41.52	132112
14		9.314	1977	0.08	308
15		9.651	3383	0.14	413
16		9.878	2693	0.11	347
17		10.136	5510	0.22	490
18		10.200	4593	0.18	324
19	NEO	10.854	427983	17.10	71214
20		10.998	223825	8.94	23996

SAMPLE INFORMATION

Sample Name:	RIF Inc Basil_EtOH 50ug/mL 2	Acquired By:	Saneesh
Sample Type:	Unknown	Date Acquired:	2017/09/11 04:23:05 PM
Vial:	9	Acq. Method Set:	HDI_RIF_25ODESRIF_NEO
Injection #:	1	Date Processed:	2017/09/12 11:45:40 AM
Injection Volume:	15.00 ul	Processing Method:	HDI_RIF_ W2996 254.0nm-1.2
Run Time:	11.5 Minutes	Channel Name:	W2996 254.0nm-1.2
Sample Set Name:	RIF_Basil EtOH IC50 11_9_17	Proc. Chnl. Descr.:	W2996 PDA 254.0 nm at 1.2

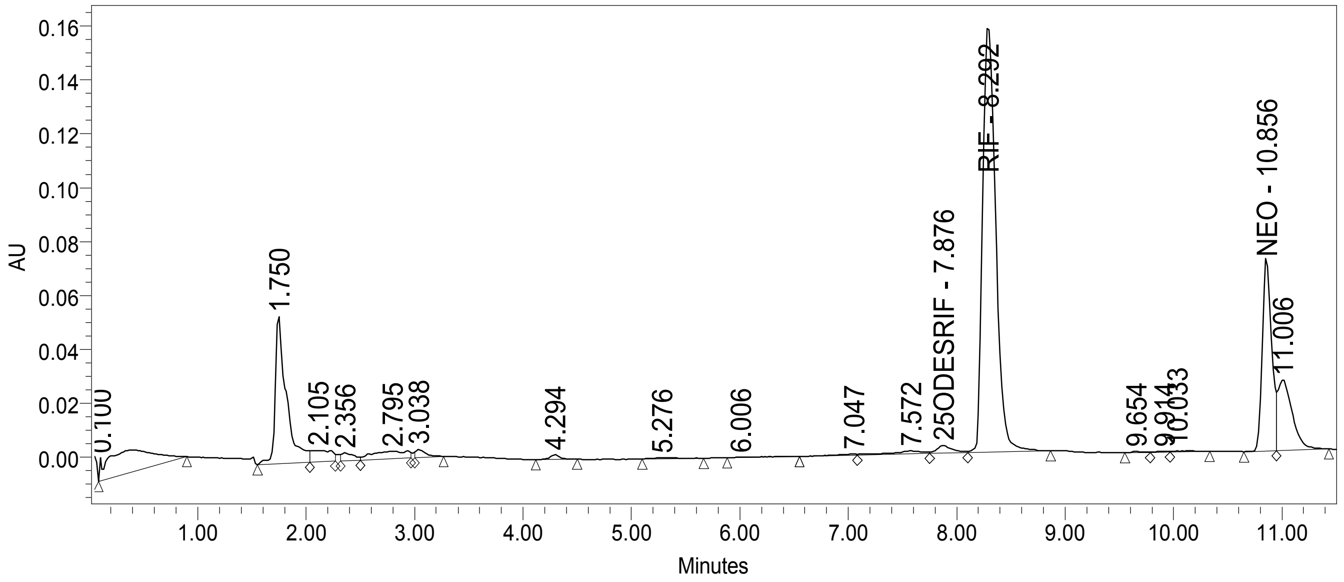

	Peak Name	RT	Area	% Area	Height
1		0.100	254561	8.75	8537
2		1.750	391606	13.45	53710
3		2.105	55785	1.92	4379
4		2.356	23634	0.81	2916
5		2.795	63606	2.19	2704
6		3.038	19273	0.66	3042
7		4.294	10910	0.37	1692
8		5.276	5854	0.20	363
9		6.006	4131	0.14	179
10		7.047	6991	0.24	480
11		7.572	24188	0.83	1099
12	25ODESRIF	7.876	28390	0.98	2924

Reported by User: Saneesh (Saneesh) Project Name: HDI_Stellenbosch_2016

	Peak Name	RT	Area	% Area	Height
13	RIF	8.292	1334123	45.83	158650
14		9.654	2451	0.08	373
15		9.914	2183	0.07	289
16		10.033	4763	0.16	325
17	NEO	10.856	432099	14.84	71535
18		11.006	246252	8.46	26136

SAMPLE INFORMATION

Sample Name:	RIF Inc Basil_EtOH 50ug/mL 3	Acquired By:	Saneesh
Sample Type:	Unknown	Date Acquired:	2017/09/11 04:37:47 PM
Vial:	10	Acq. Method Set:	HDI_RIF_25ODESRIF_NEO
Injection #:	1	Date Processed:	2017/09/12 11:45:40 AM
Injection Volume:	15.00 ul	Processing Method:	HDI_RIF_ W2996 254.0nm-1.2
Run Time:	11.5 Minutes	Channel Name:	W2996 254.0nm-1.2
Sample Set Name:	RIF_Basil EtOH IC50 11_9_17	Proc. Chnl. Descr.:	W2996 PDA 254.0 nm at 1.2

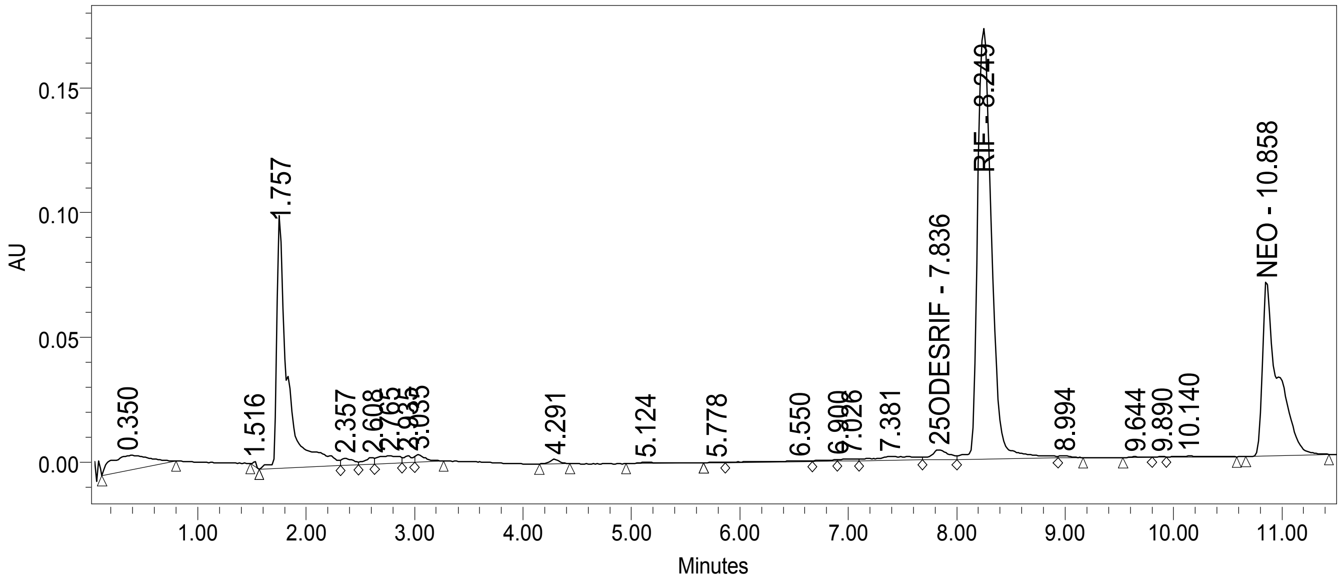

	Peak Name	RT	Area	% Area	Height
1		0.350	153423	4.55	6076
2		1.516	4026	0.12	1361
3		1.757	692172	20.54	98422
4		2.357	20673	0.61	2751
5		2.608	16045	0.48	2592
6		2.765	40354	1.20	2900
7		2.935	15343	0.46	2611
8		3.035	18760	0.56	3028
9		4.291	11336	0.34	1833
10		5.124	8537	0.25	447
11		5.778	1349	0.04	140
12		6.550	8838	0.26	258

Reported by User: Saneesh (Saneesh)   Project Name: HDI_Stellenbosch_2016

	Peak Name	RT	Area	% Area	Height
13		6.900	5260	0.16	583
14		7.026	8783	0.26	796
15		7.381	39287	1.17	1585
16	25ODESRIF	7.836	43842	1.30	3944
17	RIF	8.249	1556896	46.19	172673
18		8.994	5853	0.17	783
19		9.644	2140	0.06	318
20		9.890	1330	0.04	290
21		10.140	9818	0.29	441
22	NEO	10.858	706445	20.96	70426

Reported by User: Saneesh (Saneesh)   Project Name: HDI_Stellenbosch_2016

SAMPLE INFORMATION

Sample Name:	RIF Inc Basil_EtOH 100ug/mL 1	Acquired By:	Saneesh
Sample Type:	Unknown	Date Acquired:	2017/09/11 04:52:30 PM
Vial:	11	Acq. Method Set:	HDI_RIF_25ODESRIF_NEO
Injection #:	1	Date Processed:	2017/09/12 11:45:40 AM
Injection Volume:	15.00 ul	Processing Method:	HDI_RIF_ W2996 254.0nm-1.2
Run Time:	11.5 Minutes	Channel Name:	W2996 254.0nm-1.2
Sample Set Name:	RIF_Basil EtOH IC50 11_9_17	Proc. Chnl. Descr.:	W2996 PDA 254.0 nm at 1.2

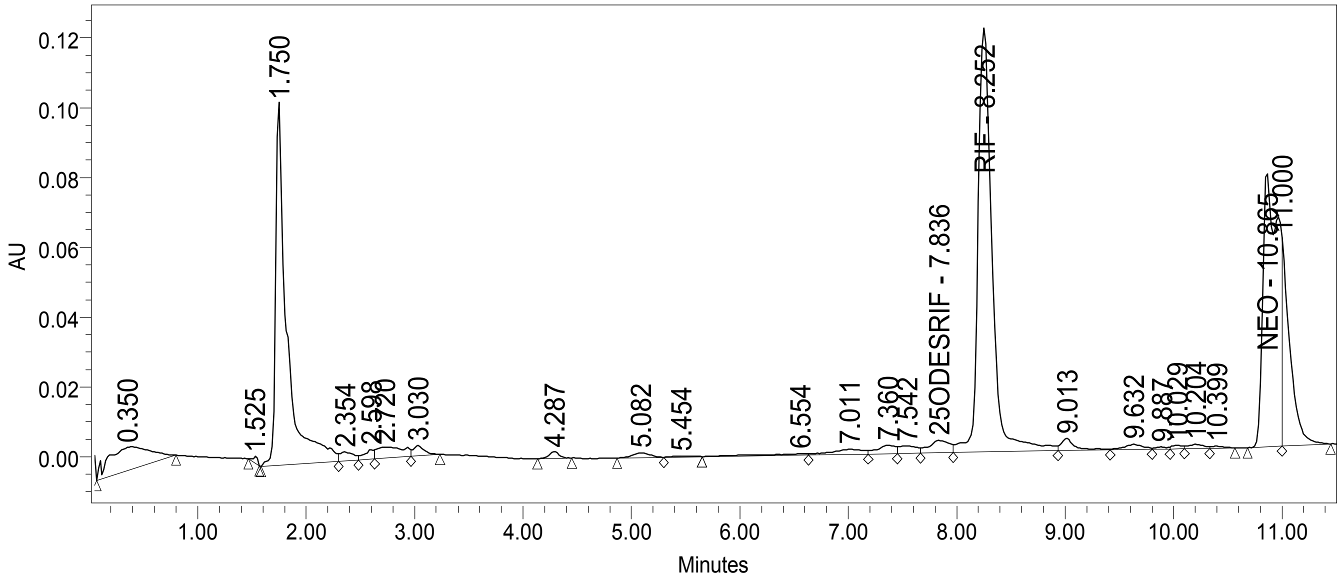

	Peak Name	RT	Area	% Area	Height
1		0.350	178801	5.18	6628
2		1.525	5982	0.17	1883
3		1.750	737324	21.35	101821
4		2.354	22102	0.64	2625
5		2.598	17099	0.50	2628
6		2.720	53439	1.55	3089
7		3.030	20892	0.60	2969
8		4.287	12787	0.37	1948
9		5.082	15761	0.46	1423
10		5.454	3568	0.10	280
11		6.554	13831	0.40	411
12		7.011	29373	0.85	1459

Reported by User: Saneesh (Saneesh) Project Name: HDI_Stellenbosch_2016

	Peak Name	RT	Area	% Area	Height
13		7.360	27732	0.80	2426
14		7.542	24757	0.72	2095
15	25ODESRIF	7.836	45998	1.33	3604
16	RIF	8.252	1102016	31.91	121471
17		9.013	31373	0.91	3510
18		9.632	17799	0.52	1553
19		9.887	4584	0.13	542
20		10.029	6449	0.19	935
21		10.204	12049	0.35	1211
22		10.399	4203	0.12	516
23	NEO	10.865	765988	22.18	77921
24		11.000	299551	8.67	59617

SAMPLE INFORMATION

Sample Name:	RIF Inc Basil_EtOH 100ug/mL 2	Acquired By:	Saneesh
Sample Type:	Unknown	Date Acquired:	2017/09/11 05:07:15 PM
Vial:	12	Acq. Method Set:	HDI_RIF_25ODESRIF_NEO
Injection #:	1	Date Processed:	2017/09/12 11:45:40 AM
Injection Volume:	15.00 ul	Processing Method:	HDI_RIF_ W2996 254.0nm-1.2
Run Time:	11.5 Minutes	Channel Name:	W2996 254.0nm-1.2
Sample Set Name:	RIF_Basil EtOH IC50 11_9_17	Proc. Chnl. Descr.:	W2996 PDA 254.0 nm at 1.2

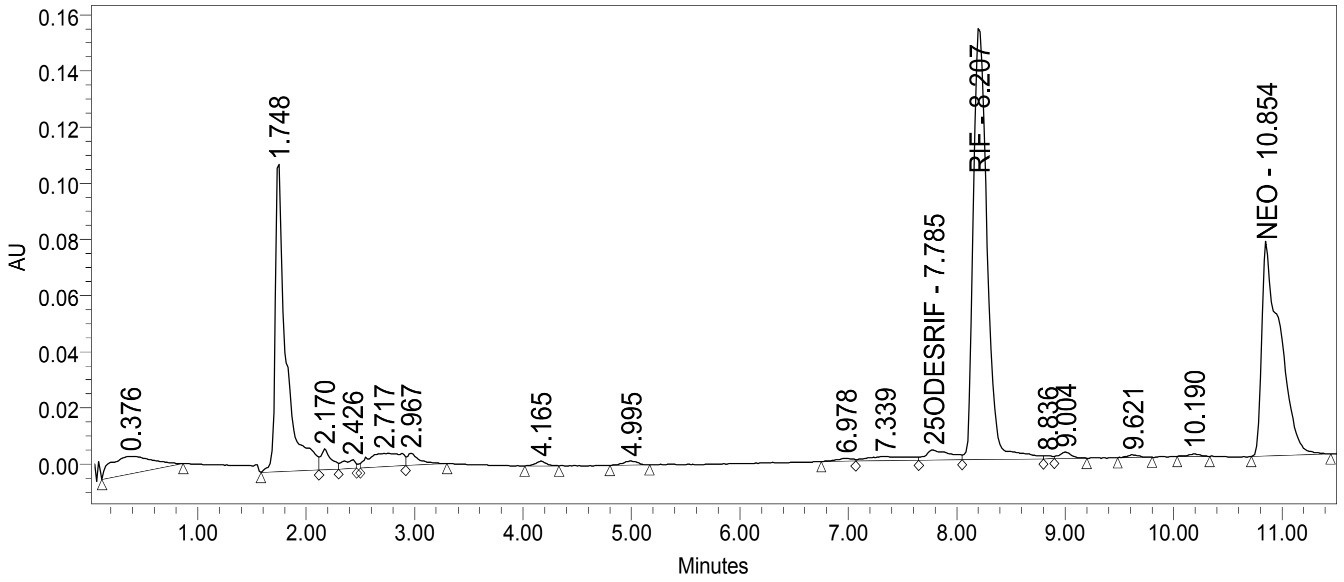

	Peak Name	RT	Area	% Area	Height
1		0.376	168335	4.71	6240
2		1.748	746076	20.88	108665
3		2.170	49157	1.38	7217
4		2.426	24118	0.67	2895
5		2.717	99785	2.79	4684
6		2.967	35588	1.00	4325
7		4.165	11615	0.32	1679
8		4.995	14110	0.39	1436
9		6.978	9969	0.28	1003
10		7.339	39507	1.11	1463
11	25ODESRIF	7.785	55102	1.54	3645
12	RIF	8.207	1380782	38.63	154575

Reported by User: Saneesh (Saneesh) Project Name: HDI_Stellenbosch_2016

	Peak Name	RT	Area	% Area	Height
13		8.836	5717	0.16	1066
14		9.004	17168	0.48	2240
15		9.621	7476	0.21	996
16		10.190	7257	0.20	932
17	NEO	10.854	902160	25.24	76421

Reported by User: Saneesh (Saneesh) Project Name: HDI_Stellenbosch_2016

SAMPLE INFORMATION

Sample Name:	RIF Inc Basil_EtOH 100ug/mL 3	Acquired By:	Saneesh
Sample Type:	Unknown	Date Acquired:	2017/09/11 05:22:08 PM
Vial:	13	Acq. Method Set:	HDI_RIF_25ODESRIF_NEO
Injection #:	1	Date Processed:	2017/09/12 11:45:40 AM
Injection Volume:	15.00 ul	Processing Method:	HDI_RIF_ W2996 254.0nm-1.2
Run Time:	11.5 Minutes	Channel Name:	W2996 254.0nm-1.2
Sample Set Name:	RIF_Basil EtOH IC50 11_9_17	Proc. Chnl. Descr.:	W2996 PDA 254.0 nm at 1.2

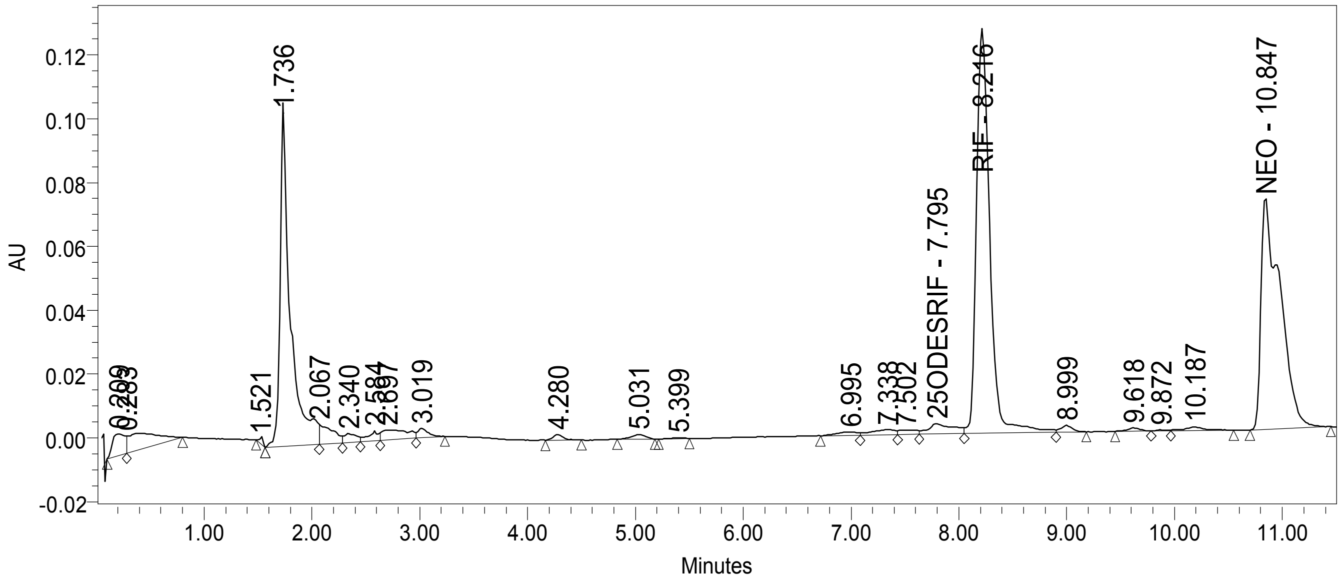

	Peak Name	RT	Area	% Area	Height
1		0.209	56500	1.78	6957
2		0.283	99374	3.12	5501
3		1.521	4750	0.15	1651
4		1.736	701461	22.05	104536
5		2.067	54255	1.71	6267
6		2.340	21987	0.69	2715
7		2.584	20641	0.65	2715
8		2.697	51820	1.63	3130
9		3.019	20051	0.63	2933
10		4.280	11835	0.37	1780
11		5.031	13466	0.42	1485
12		5.399	2414	0.08	277

Reported by User: Saneesh (Saneesh) Project Name: HDI_Stellenbosch_2016

	Peak Name	RT	Area	% Area	Height
13		6.995	13224	0.42	1035
14		7.338	24725	0.78	1603
15		7.502	15752	0.50	1381
16	25ODESRIF	7.795	52563	1.65	3209
17	RIF	8.216	1083792	34.07	126692
18		8.999	15534	0.49	2077
19		9.618	8055	0.25	998
20		9.872	1923	0.06	292
21		10.187	15080	0.47	1159
22	NEO	10.847	891532	28.03	72055

Multi Sample Summary

Reported by User: Saneesh (Saneesh) Project Name: HDI_Stellenbosch_2016

SAMPLE INFORMATION

Sample Name:	RIF Inc Basil_EtOH 150ug/mL 1	Acquired By:	Saneesh
Sample Type:	Unknown	Date Acquired:	2017/09/11 05:36:50 PM
Vial:	14	Acq. Method Set:	HDI_RIF_25ODESRIF_NEO
Injection #:	1	Date Processed:	2017/09/12 11:45:40 AM
Injection Volume:	15.00 ul	Processing Method:	HDI_RIF_ W2996 254.0nm-1.2
Run Time:	11.5 Minutes	Channel Name:	W2996 254.0nm-1.2
Sample Set Name:	RIF_Basil EtOH IC50 11_9_17	Proc. Chnl. Descr.:	W2996 PDA 254.0 nm at 1.2

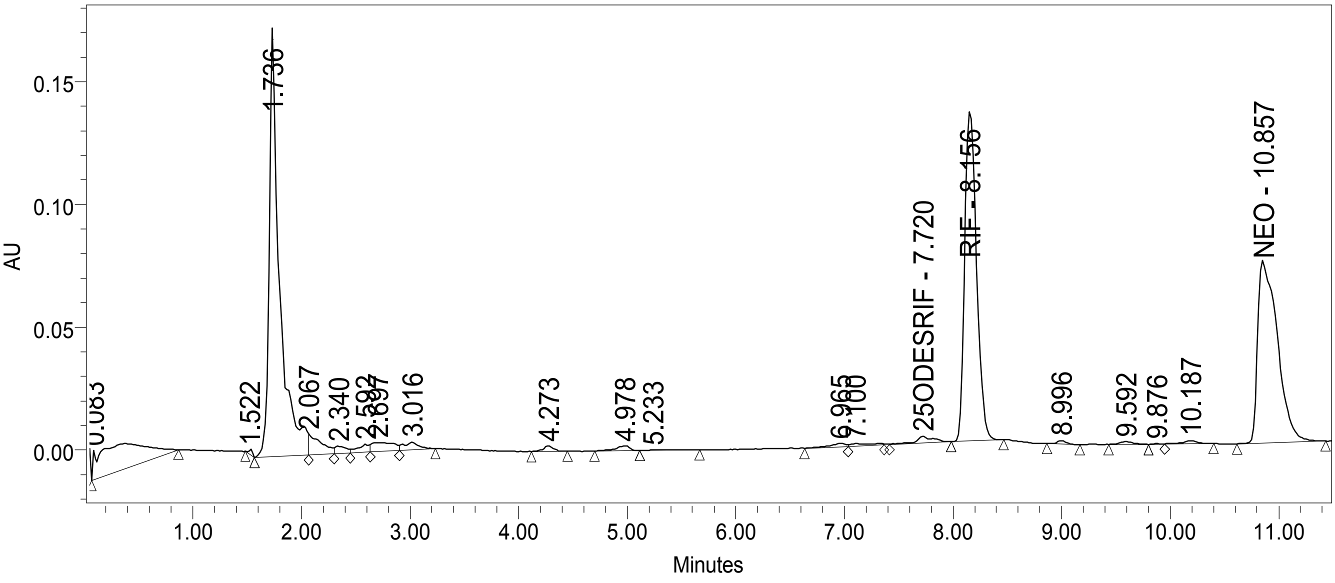

	Peak Name	RT	Area	% Area	Height
1		0.083	325705	8.66	11887
2		1.522	5064	0.13	1781
3		1.736	1163420	30.92	169676
4		2.067	67204	1.79	8578
5		2.340	21126	0.56	2875
6		2.592	24061	0.64	3048
7		2.697	50119	1.33	3670
8		3.016	30407	0.81	3090
9		4.273	14109	0.38	2165
10		4.978	19396	0.52	2019
11		5.233	4206	0.11	215
12		6.965	12956	0.34	1278

Reported by User: Saneesh (Saneesh) Project Name: HDI_Stellenbosch_2016

	Peak Name	RT	Area	% Area	Height
13		7.100	13217	0.35	977
14	25ODESRIF	7.720	31163	0.83	2789
15	RIF	8.156	1017832	27.05	134487
16		8.996	8254	0.22	1390
17		9.592	10019	0.27	1122
18		9.876	1436	0.04	232
19		10.187	13015	0.35	1323
20	NEO	10.857	929538	24.71	74693

SAMPLE INFORMATION

Sample Name:	RIF Inc Basil_EtOH 150ug/mL 2	Acquired By:	Saneesh
Sample Type:	Unknown	Date Acquired:	2017/09/11 05:51:33 PM
Vial:	15	Acq. Method Set:	HDI_RIF_25ODESRIF_NEO
Injection #:	1	Date Processed:	2017/09/12 11:45:40 AM
Injection Volume:	15.00 ul	Processing Method:	HDI_RIF_ W2996 254.0nm-1.2
Run Time:	11.5 Minutes	Channel Name:	W2996 254.0nm-1.2
Sample Set Name:	RIF_Basil EtOH IC50 11_9_17	Proc. Chnl. Descr.:	W2996 PDA 254.0 nm at 1.2

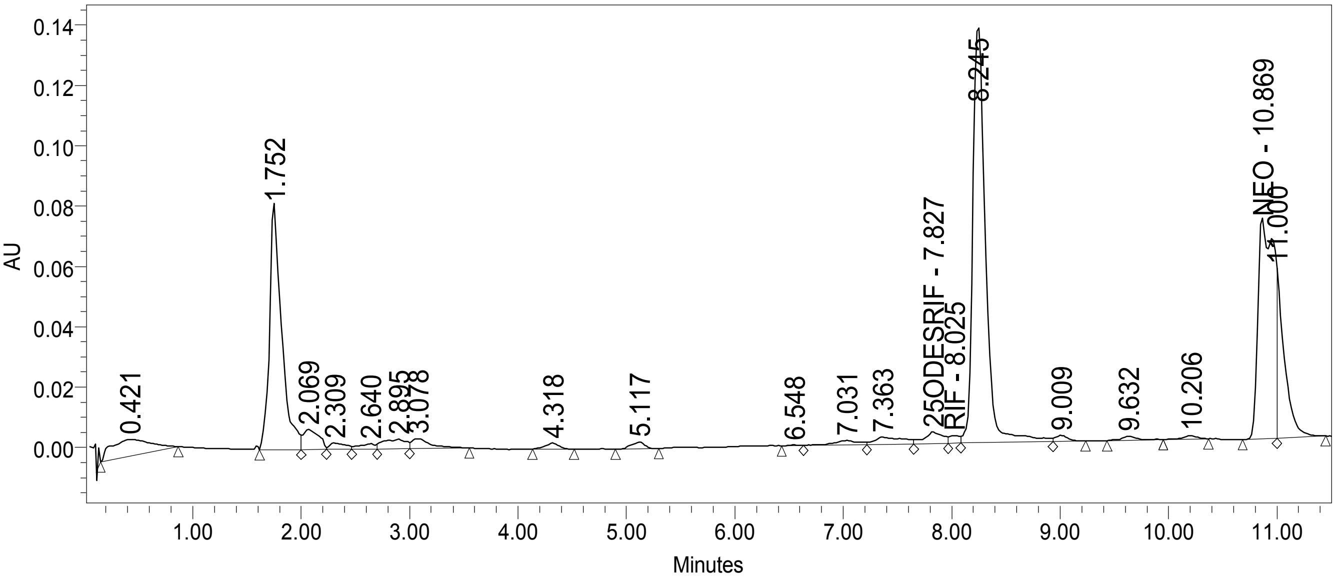

	Peak Name	RT	Area	% Area	Height
1		0.421	141486	4.27	5534
2		1.752	628389	18.95	80728
3		2.069	67465	2.03	6685
4		2.309	21070	0.64	2091
5		2.640	18092	0.55	1770
6		2.895	46660	1.41	3157
7		3.078	40713	1.23	3326
8		4.318	17250	0.52	1999
9		5.117	19120	0.58	2216
10		6.548	1506	0.05	259
11		7.031	24121	0.73	1509
12		7.363	43273	1.31	2340

Reported by User: Saneesh (Saneesh) Project Name: HDI_Stellenbosch_2016

	Peak Name	RT	Area	% Area	Height
13	25ODESRIF	7.827	45522	1.37	3774
14	RIF	8.025	16392	0.49	2490
15		8.245	1133482	34.18	137950
16		9.009	16025	0.48	2054
17		9.632	13320	0.40	1341
18		10.206	10300	0.31	1141
19	NEO	10.869	743174	22.41	72855
20		11.000	268536	8.10	56096

Reported by User: Saneesh (Saneesh)   Project Name: HDI_Stellenbosch_2016

SAMPLE INFORMATION

Sample Name:	RIF Inc Basil_EtOH 150ug/mL 3	Acquired By:	Saneesh
Sample Type:	Unknown	Date Acquired:	2017/09/11 06:06:17 PM
Vial:	16	Acq. Method Set:	HDI_RIF_25ODESRIF_NEO
Injection #:	1	Date Processed:	2017/09/12 11:45:40 AM
Injection Volume:	15.00 ul	Processing Method:	HDI_RIF_ W2996 254.0nm-1.2
Run Time:	11.5 Minutes	Channel Name:	W2996 254.0nm-1.2
Sample Set Name:	RIF_Basil EtOH IC50 11_9_17	Proc. Chnl. Descr.:	W2996 PDA 254.0 nm at 1.2

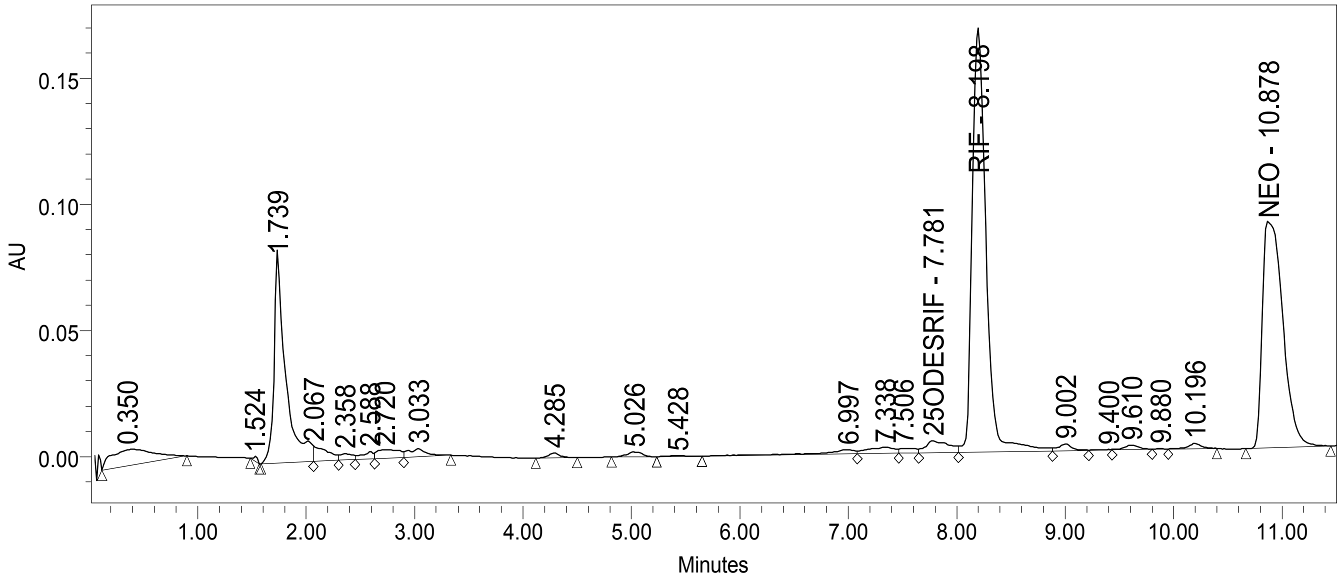

	Peak Name	RT	Area	% Area	Height
1		0.350	175667	4.45	6421
2		1.524	5192	0.13	1773
3		1.739	621889	15.76	81612
4		2.067	55664	1.41	6109
5		2.358	20218	0.51	2616
6		2.588	22175	0.56	2844
7		2.720	47746	1.21	3358
8		3.033	38219	0.97	3269
9		4.285	13858	0.35	1969
10		5.026	20046	0.51	2010
11		5.428	4767	0.12	332
12		6.997	36350	0.92	1681

Reported by User: Saneesh (Saneesh) Project Name: HDI_Stellenbosch_2016

	Peak Name	RT	Area	% Area	Height
13		7.338	43055	1.09	2369
14		7.506	19624	0.50	1851
15	25ODESRIF	7.781	70633	1.79	4760
16	RIF	8.198	1505750	38.16	168007
17		9.002	22274	0.56	2653
18		9.400	2066	0.05	273
19		9.610	16831	0.43	1843
20		9.880	1508	0.04	295
21		10.196	17579	0.45	2031
22	NEO	10.878	1185279	30.03	90922

SAMPLE INFORMATION

Sample Name:	RIF Inc Basil_EtOH 200ug/mL 1	Acquired By:	Saneesh
Sample Type:	Unknown	Date Acquired:	2017/09/11 06:21:00 PM
Vial:	17	Acq. Method Set:	HDI_RIF_25ODESRIF_NEO
Injection #:	1	Date Processed:	2017/09/12 11:45:40 AM
Injection Volume:	15.00 ul	Processing Method:	HDI_RIF_ W2996 254.0nm-1.2
Run Time:	11.5 Minutes	Channel Name:	W2996 254.0nm-1.2
Sample Set Name:	RIF_Basil EtOH IC50 11_9_17	Proc. Chnl. Descr.:	W2996 PDA 254.0 nm at 1.2

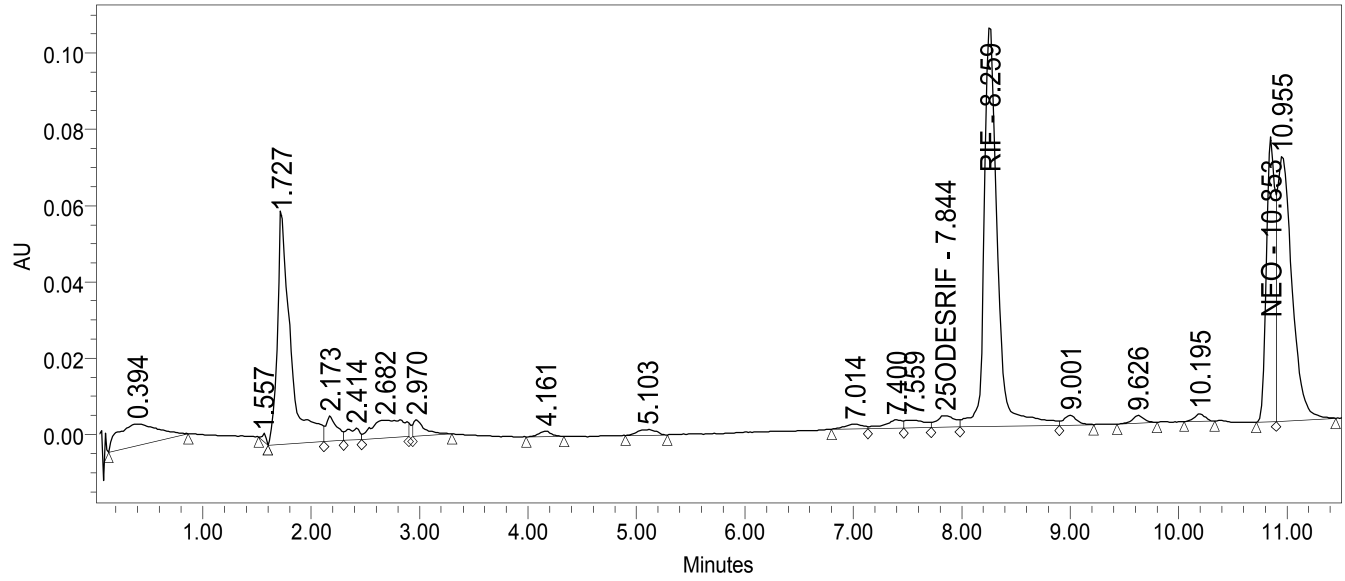

	Peak Name	RT	Area	% Area	Height
1		0.394	148180	4.91	5643
2		1.557	4735	0.16	1671
3		1.727	514881	17.06	60224
4		2.173	47579	1.58	6517
5		2.414	24679	0.82	2981
6		2.682	97722	3.24	4661
7		2.970	31588	1.05	4223
8		4.161	12266	0.41	1500
9		5.103	17339	0.57	1459
10		7.014	15564	0.52	1309
11		7.400	25350	0.84	2203
12		7.559	26851	0.89	2082

Reported by User: Saneesh (Saneesh) Project Name: HDI_Stellenbosch_2016

	Peak Name	RT	Area	% Area	Height
13	25ODESRIF	7.844	36749	1.22	3084
14	RIF	8.259	893420	29.61	105824
15		9.001	21501	0.71	2619
16		9.626	15700	0.52	2054
17		10.195	13006	0.43	1925
18	NEO	10.853	408805	13.55	74295
19		10.955	661754	21.93	69293

Reported by User: Saneesh (Saneesh) Project Name: HDI_Stellenbosch_2016

SAMPLE INFORMATION

Sample Name:	RIF Inc Basil_EtOH 200ug/mL 2	Acquired By:	Saneesh
Sample Type:	Unknown	Date Acquired:	2017/09/11 06:35:44 PM
Vial:	18	Acq. Method Set:	HDI_RIF_25ODESRIF_NEO
Injection #:	1	Date Processed:	2017/09/12 11:45:40 AM
Injection Volume:	15.00 ul	Processing Method:	HDI_RIF_ W2996 254.0nm-1.2
Run Time:	11.5 Minutes	Channel Name:	W2996 254.0nm-1.2
Sample Set Name:	RIF_Basil EtOH IC50 11_9_17	Proc. Chnl. Descr.:	W2996 PDA 254.0 nm at 1.2

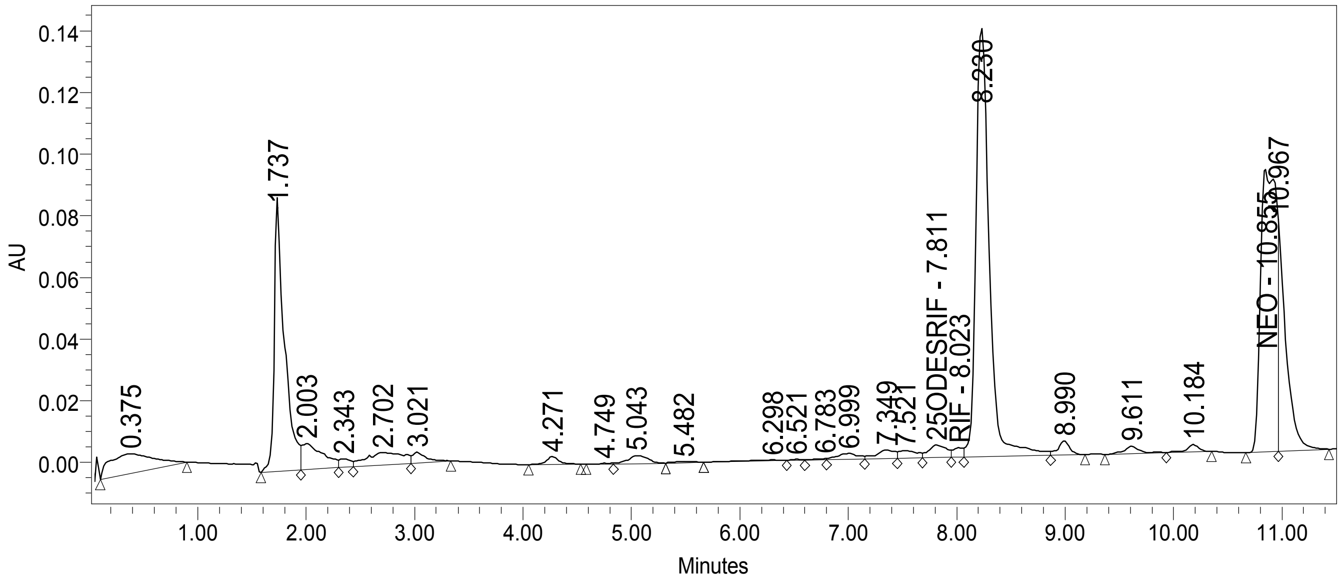

	Peak Name	RT	Area	% Area	Height
1		0.375	181713	4.76	6420
2		1.737	581446	15.22	86789
3		2.003	114791	3.00	8418
4		2.343	19661	0.51	2831
5		2.702	95713	2.50	3968
6		3.021	30392	0.80	3484
7		4.271	21374	0.56	2572
8		4.749	2001	0.05	267
9		5.043	32602	0.85	2560
10		5.482	5466	0.14	347
11		6.298	9073	0.24	384
12		6.521	3204	0.08	448

Reported by User: Saneesh (Saneesh) Project Name: HDI_Stellenbosch_2016

	Peak Name	RT	Area	% Area	Height
13		6.783	4105	0.11	477
14		6.999	26592	0.70	1950
15		7.349	33685	0.88	2938
16		7.521	28790	0.75	2366
17	25ODESRIF	7.811	46571	1.22	4115
18	RIF	8.023	19591	0.51	3045
19		8.230	1202739	31.47	138995
20		8.990	33791	0.88	4610
21		9.611	24037	0.63	2434
22		10.184	18733	0.49	2397
23	NEO	10.855	923419	24.16	91843
24		10.967	361932	9.47	75195

Multi Sample Summary

Reported by User: Saneesh (Saneesh)   Project Name: HDI_Stellenbosch_2016

SAMPLE INFORMATION

Sample Name:	RIF Inc Basil_EtOH 200ug/mL 3	Acquired By:	Saneesh
Sample Type:	Unknown	Date Acquired:	2017/09/11 06:50:35 PM
Vial:	19	Acq. Method Set:	HDI_RIF_25ODESRIF_NEO
Injection #:	1	Date Processed:	2017/09/12 11:45:40 AM
Injection Volume:	15.00 ul	Processing Method:	HDI_RIF_ W2996 254.0nm-1.2
Run Time:	11.5 Minutes	Channel Name:	W2996 254.0nm-1.2
Sample Set Name:	RIF_Basil EtOH IC50 11_9_17	Proc. Chnl. Descr.:	W2996 PDA 254.0 nm at 1.2

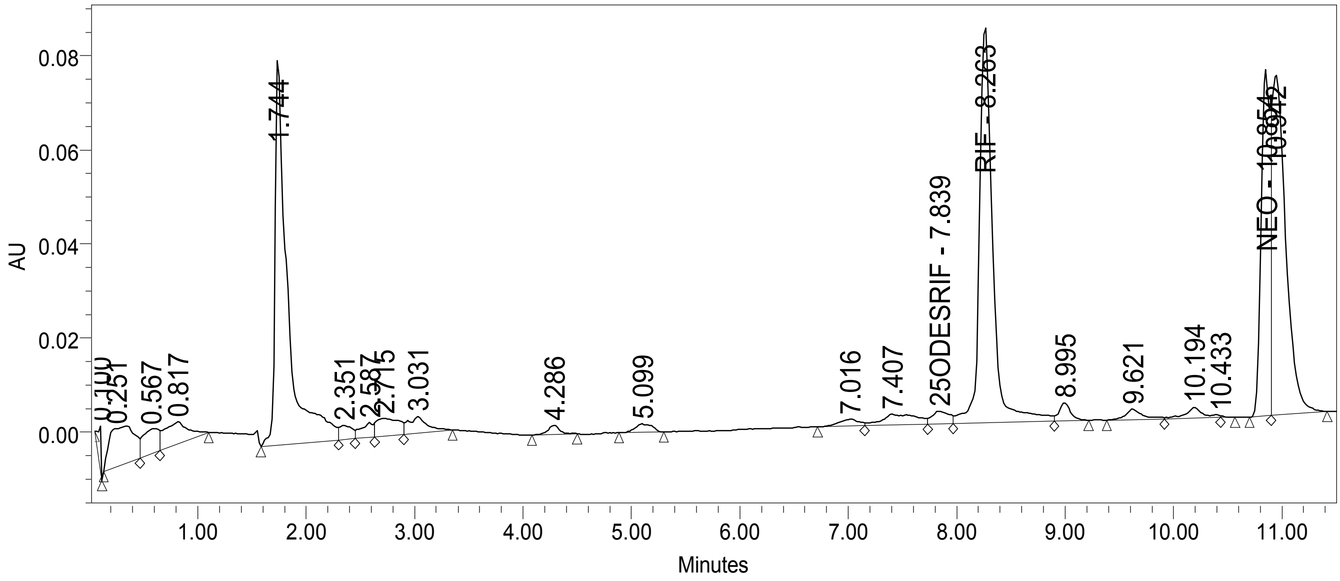

	Peak Name	RT	Area	% Area	Height
1		0.100	16658	0.54	8975
2		0.251	131465	4.27	8191
3		0.567	53289	1.73	5257
4		0.817	75089	2.44	4656
5		1.744	655469	21.28	80770
6		2.351	23182	0.75	2992
7		2.587	25921	0.84	3053
8		2.715	52870	1.72	3727
9		3.031	43457	1.41	3550
10		4.286	13834	0.45	1912
11		5.099	20514	0.67	1834
12		7.016	17981	0.58	1439

Reported by User: Saneesh (Saneesh) Project Name: HDI_Stellenbosch_2016

	Peak Name	RT	Area	% Area	Height
13		7.407	50955	1.65	2314
14	25ODESRIF	7.839	29392	0.95	2740
15	RIF	8.263	721514	23.42	83952
16		8.995	28340	0.92	3859
17		9.621	24133	0.78	2245
18		10.194	26414	0.86	2276
19		10.433	1187	0.04	425
20	NEO	10.854	422203	13.71	73498
21		10.942	646427	20.99	72468

Multi Sample Summary

Reported by User: Saneesh (Saneesh) | Project Name: HDI_Stellenbosch_2016

SAMPLE INFORMATION

Sample Name:	RIF Inc Basil_MeOH 1ug/mL 1	Acquired By:	Saneesh
Sample Type:	Unknown	Date Acquired:	2017/09/11 08:06:45 PM
Vial:	2	Acq. Method Set:	HDI_RIF_25ODESRIF_NEO
Injection #:	1	Date Processed:	2017/09/12 11:47:02 AM
Injection Volume:	15.00 ul	Processing Method:	HDI_RIF_ W2996 254.0nm-1.2
Run Time:	11.5 Minutes	Channel Name:	W2996 254.0nm-1.2
Sample Set Name:	RIF_Basil MeOH IC50 11_9_17	Proc. Chnl. Descr.:	W2996 PDA 254.0 nm at 1.2

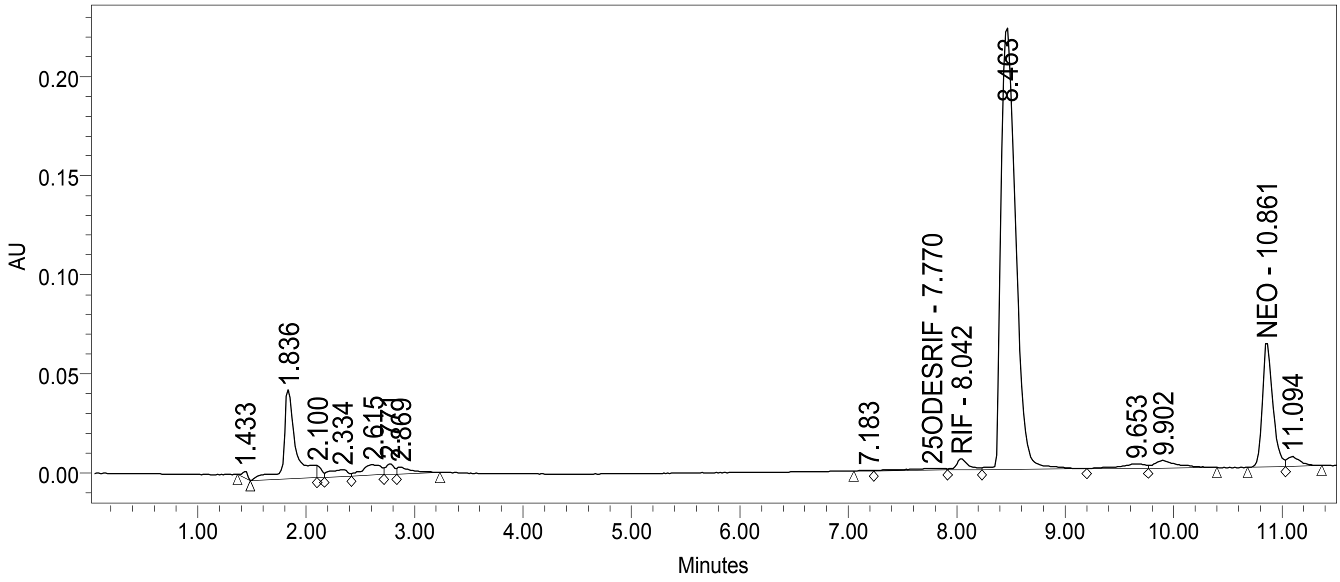

	Peak Name	RT	Area	% Area	Height
1		1.433	11031	0.34	3028
2		1.836	351423	10.83	45611
3		2.100	16747	0.52	5992
4		2.334	40251	1.24	3194
5		2.615	63485	1.96	5161
6		2.771	28640	0.88	5245
7		2.869	30874	0.95	3447
8		7.183	2099	0.06	299
9	25ODESRIF	7.770	21595	0.67	756
10	RIF	8.042	43410	1.34	5576
11		8.463	2081397	64.16	222941
12		9.653	37062	1.14	2320
13		9.902	55701	1.72	3830
14	NEO	10.861	420025	12.95	62408

Reported by User: Saneesh (Saneesh) Project Name: HDI_Stellenbosch_2016

	Peak Name	RT	Area	% Area	Height
15		11.094	40470	1.25	4859

Reported by User: Saneesh (Saneesh) Project Name: HDI_Stellenbosch_2016

SAMPLE INFORMATION

Sample Name:	RIF Inc Basil_MeOH 1ug/mL 2	Acquired By:	Saneesh
Sample Type:	Unknown	Date Acquired:	2017/09/11 08:21:30 PM
Vial:	3	Acq. Method Set:	HDI_RIF_25ODESRIF_NEO
Injection #:	1	Date Processed:	2017/09/12 11:47:02 AM
Injection Volume:	15.00 ul	Processing Method:	HDI_RIF_ W2996 254.0nm-1.2
Run Time:	11.5 Minutes	Channel Name:	W2996 254.0nm-1.2
Sample Set Name:	RIF_Basil MeOH IC50 11_9_17	Proc. Chnl. Descr.:	W2996 PDA 254.0 nm at 1.2

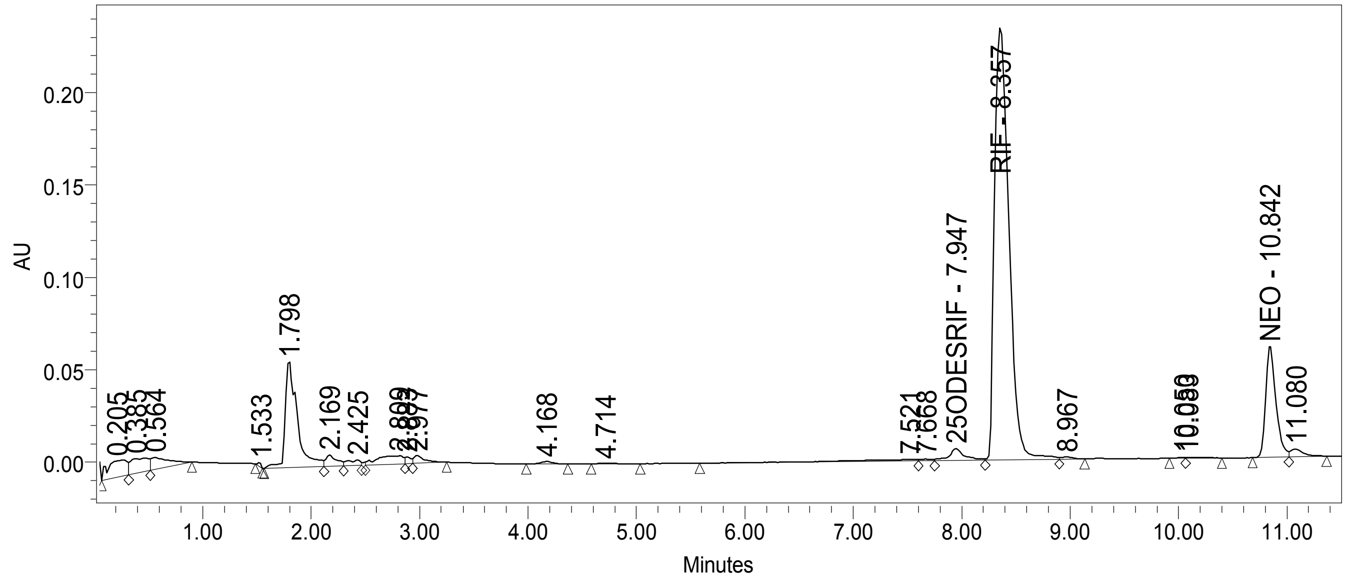

	Peak Name	RT	Area	% Area	Height
1		0.205	110553	3.05	8807
2		0.385	87919	2.43	8027
3		0.564	74220	2.05	6368
4		1.533	4650	0.13	2109
5		1.798	448937	12.41	56721
6		2.169	42968	1.19	6169
7		2.425	23662	0.65	2831
8		2.809	75296	2.08	4234
9		2.883	12480	0.34	3646
10		2.977	29474	0.81	4088
11		4.168	10287	0.28	1274
12		4.714	3787	0.10	292

	Peak Name	RT	Area	% Area	Height
13		7.521	34345	0.95	842
14		7.668	7121	0.20	848
15	25ODESRIF	7.947	60577	1.67	6454
16	RIF	8.357	2150103	59.41	234971
17		8.967	7737	0.21	1048
18		10.050	1649	0.05	305
19		10.083	4765	0.13	433
20	NEO	10.842	393651	10.88	60786
21		11.080	34795	0.96	4126

SAMPLE INFORMATION

Sample Name:	RIF Inc Basil_MeOH 1ug/mL 3	Acquired By:	Saneesh
Sample Type:	Unknown	Date Acquired:	2017/09/11 08:36:13 PM
Vial:	4	Acq. Method Set:	HDI_RIF_25ODESRIF_NEO
Injection #:	1	Date Processed:	2017/09/12 11:47:02 AM
Injection Volume:	15.00 ul	Processing Method:	HDI_RIF_ W2996 254.0nm-1.2
Run Time:	11.5 Minutes	Channel Name:	W2996 254.0nm-1.2
Sample Set Name:	RIF_Basil MeOH IC50 11_9_17	Proc. Chnl. Descr.:	W2996 PDA 254.0 nm at 1.2

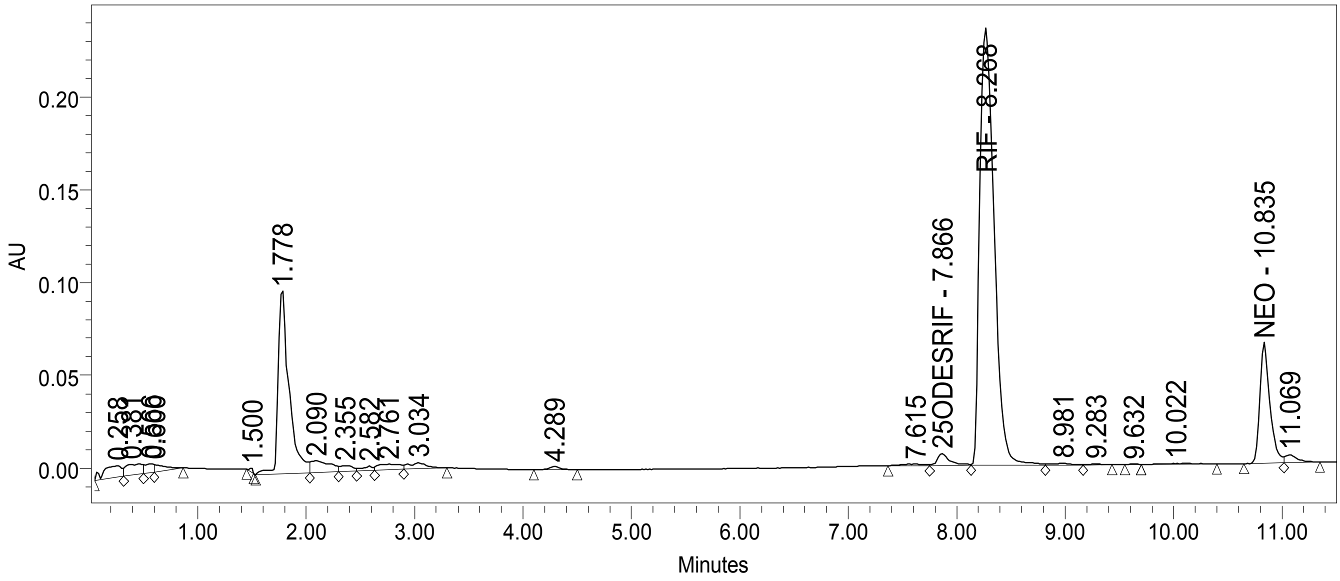

	Peak Name	RT	Area	% Area	Height
1		0.258	74285	1.95	6100
2		0.381	58557	1.53	5894
3		0.566	27045	0.71	4668
4		0.600	26075	0.68	4000
5		1.500	4271	0.11	2239
6		1.778	687132	18.00	97261
7		2.090	77123	2.02	6158
8		2.355	25554	0.67	3142
9		2.582	16608	0.44	2258
10		2.761	42990	1.13	2950
11		3.034	37604	0.99	3314
12		4.289	12672	0.33	1753

Reported by User: Saneesh (Saneesh) Project Name: HDI_Stellenbosch_2016

	Peak Name	RT	Area	% Area	Height
13		7.615	11608	0.30	764
14	25ODESRIF	7.866	50904	1.33	6434
15	RIF	8.268	2187092	57.31	235718
16		8.981	13213	0.35	1109
17		9.283	4386	0.11	632
18		9.632	1263	0.03	238
19		10.022	8868	0.23	413
20	NEO	10.835	416044	10.90	64642
21		11.069	33237	0.87	4193

Reported by User: Saneesh (Saneesh)

Project Name: HDI_Stellenbosch_2016

SAMPLE INFORMATION

Sample Name:	RIF Inc Basil_MeOH 10ug/mL 1	Acquired By:	Saneesh
Sample Type:	Unknown	Date Acquired:	2017/09/11 08:50:56 PM
Vial:	5	Acq. Method Set:	HDI_RIF_25ODESRIF_NEO
Injection #:	1	Date Processed:	2017/09/12 11:47:02 AM
Injection Volume:	15.00 ul	Processing Method:	HDI_RIF_ W2996 254.0nm-1.2
Run Time:	11.5 Minutes	Channel Name:	W2996 254.0nm-1.2
Sample Set Name:	RIF_Basil MeOH IC50 11_9_17	Proc. Chnl. Descr.:	W2996 PDA 254.0 nm at 1.2

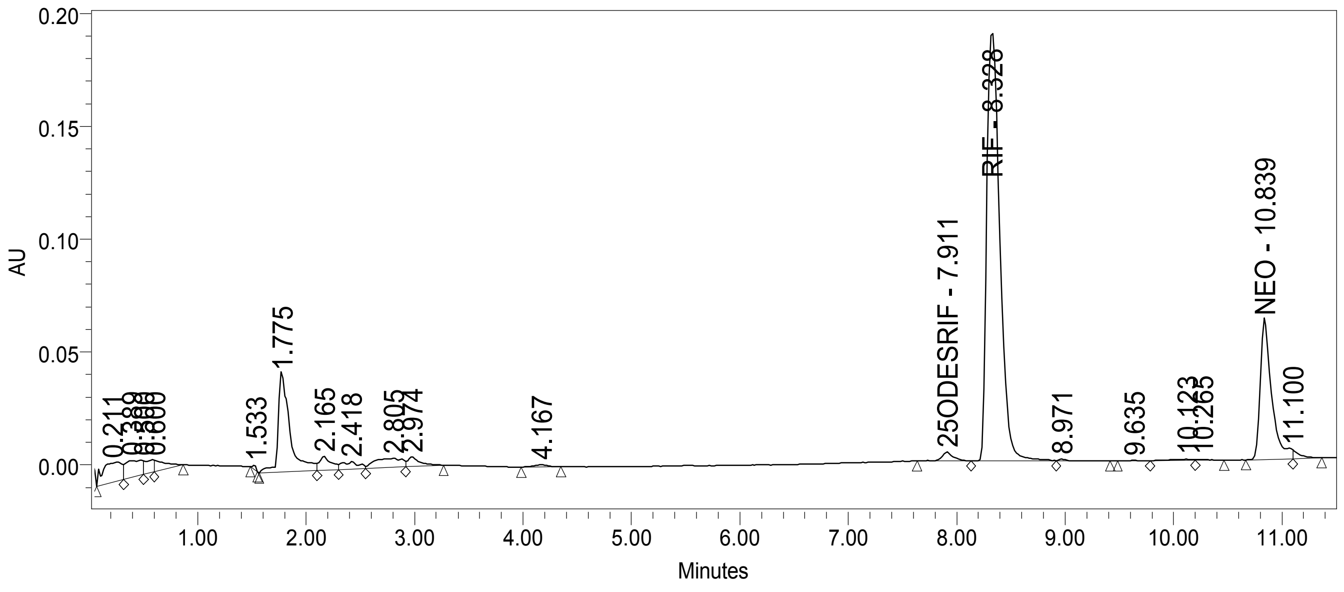

	Peak Name	RT	Area	% Area	Height
1		0.211	107360	3.69	8446
2		0.389	76212	2.62	7544
3		0.500	33463	1.15	6075
4		0.600	35086	1.21	5121
5		1.533	4572	0.16	2190
6		1.775	328731	11.31	44494
7		2.165	46467	1.60	6011
8		2.418	33449	1.15	3101
9		2.805	69934	2.41	3872
10		2.974	32547	1.12	4169
11		4.167	8754	0.30	1123
12	25ODESRIF	7.911	32491	1.12	4011

Reported by User: Saneesh (Saneesh) Project Name: HDI_Stellenbosch_2016

	Peak Name	RT	Area	% Area	Height
13	RIF	8.328	1602181	55.12	190221
14		8.971	8607	0.30	863
15		9.635	2421	0.08	291
16		10.123	8466	0.29	518
17		10.265	3194	0.11	356
18	NEO	10.839	455439	15.67	62520
19		11.100	17554	0.60	4019

SAMPLE INFORMATION

Sample Name:	RIF Inc Basil_MeOH 10ug/mL 2	Acquired By:	Saneesh
Sample Type:	Unknown	Date Acquired:	2017/09/11 09:05:38 PM
Vial:	6	Acq. Method Set:	HDI_RIF_25ODESRIF_NEO
Injection #:	1	Date Processed:	2017/09/12 11:47:02 AM
Injection Volume:	15.00 ul	Processing Method:	HDI_RIF_ W2996 254.0nm-1.2
Run Time:	11.5 Minutes	Channel Name:	W2996 254.0nm-1.2
Sample Set Name:	RIF_Basil MeOH IC50 11_9_17	Proc. Chnl. Descr.:	W2996 PDA 254.0 nm at 1.2

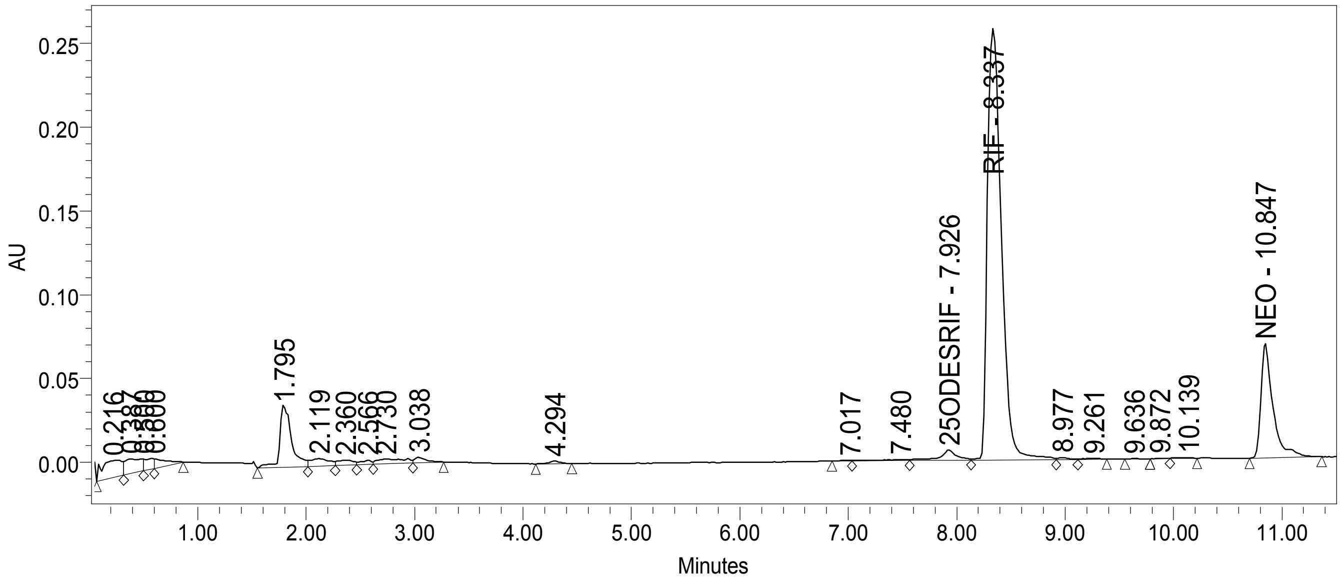

	Peak Name	RT	Area	% Area	Height
1		0.216	130569	3.54	9999
2		0.387	88640	2.40	8766
3		0.500	38418	1.04	7021
4		0.600	41150	1.11	5789
5		1.795	282259	7.64	37940
6		2.119	54658	1.48	4452
7		2.360	31163	0.84	3047
8		2.566	17373	0.47	2538
9		2.730	53677	1.45	2831
10		3.038	24478	0.66	3261
11		4.294	10429	0.28	1472
12		7.017	1729	0.05	263

	Peak Name	RT	Area	% Area	Height
13		7.480	13152	0.36	670
14	25ODESRIF	7.926	63909	1.73	5990
15	RIF	8.337	2294968	62.14	257835
16		8.977	7849	0.21	1085
17		9.261	4536	0.12	373
18		9.636	1410	0.04	247
19		9.872	1402	0.04	204
20		10.139	3618	0.10	305
21	NEO	10.847	527616	14.29	67956

SAMPLE INFORMATION

Sample Name:	RIF Inc Basil_MeOH 10ug/mL 3	Acquired By:	Saneesh
Sample Type:	Unknown	Date Acquired:	2017/09/11 09:20:20 PM
Vial:	7	Acq. Method Set:	HDI_RIF_25ODESRIF_NEO
Injection #:	1	Date Processed:	2017/09/12 11:47:02 AM
Injection Volume:	15.00 ul	Processing Method:	HDI_RIF_ W2996 254.0nm-1.2
Run Time:	11.5 Minutes	Channel Name:	W2996 254.0nm-1.2
Sample Set Name:	RIF_Basil MeOH IC50 11_9_17	Proc. Chnl. Descr.:	W2996 PDA 254.0 nm at 1.2

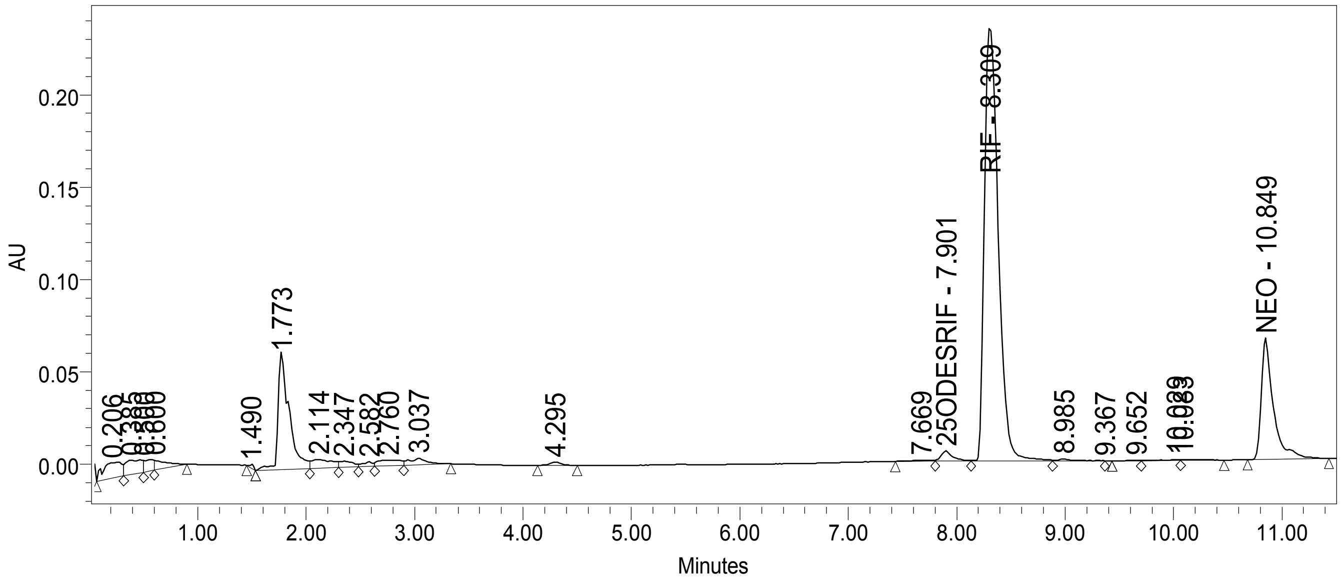

	Peak Name	RT	Area	% Area	Height
1		0.206	102635	2.84	8408
2		0.385	78306	2.16	7730
3		0.500	36090	1.00	6342
4		0.600	41392	1.14	5385
5		1.490	5842	0.16	1969
6		1.773	450761	12.45	63383
7		2.114	64629	1.79	4803
8		2.347	29475	0.81	3327
9		2.582	16953	0.47	2457
10		2.760	45113	1.25	3096
11		3.037	41043	1.13	3517
12		4.295	13861	0.38	1864

	Peak Name	RT	Area	% Area	Height
13		7.669	9634	0.27	709
14	25ODESRIF	7.901	43786	1.21	5548
15	RIF	8.309	2097789	57.96	236539
16		8.985	12716	0.35	1133
17		9.367	274	0.01	147
18		9.652	2872	0.08	377
19		10.029	7307	0.20	583
20		10.083	8679	0.24	635
21	NEO	10.849	510520	14.10	65426

SAMPLE INFORMATION

Sample Name:	RIF Inc Basil_MeOH 50ug/mL 1	Acquired By:	Saneesh
Sample Type:	Unknown	Date Acquired:	2017/09/11 09:35:03 PM
Vial:	8	Acq. Method Set:	HDI_RIF_25ODESRIF_NEO
Injection #:	1	Date Processed:	2017/09/12 11:47:02 AM
Injection Volume:	15.00 ul	Processing Method:	HDI_RIF_ W2996 254.0nm-1.2
Run Time:	11.5 Minutes	Channel Name:	W2996 254.0nm-1.2
Sample Set Name:	RIF_Basil MeOH IC50 11_9_17	Proc. Chnl. Descr.:	W2996 PDA 254.0 nm at 1.2

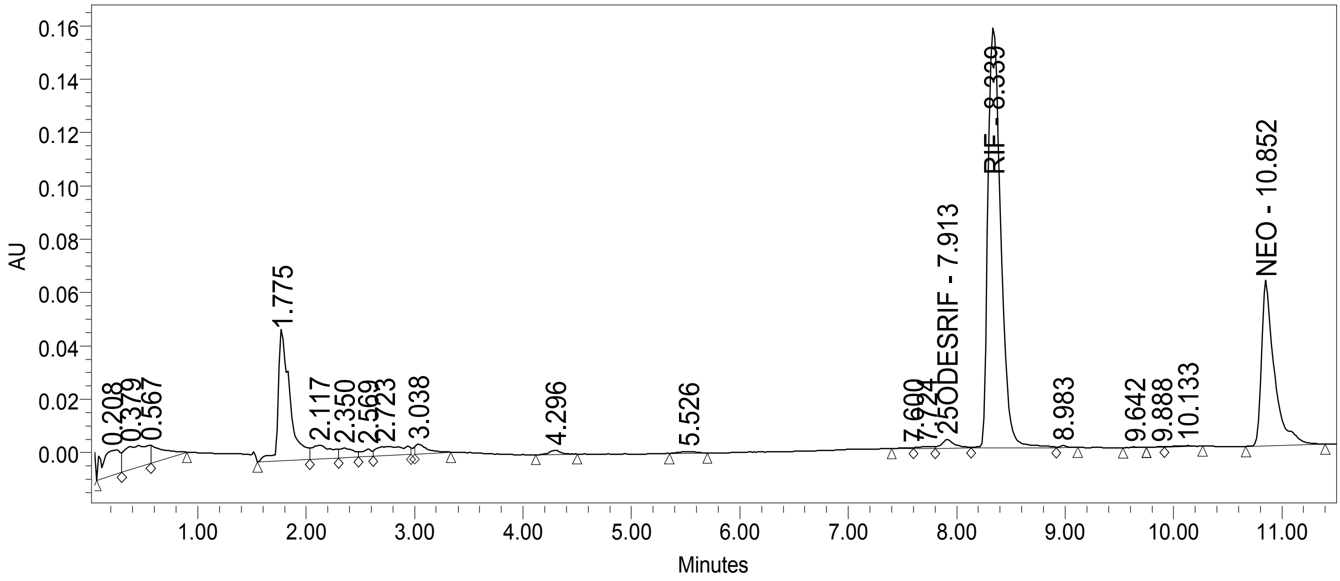

	Peak Name	RT	Area	% Area	Height
1		0.208	109882	4.03	9290
2		0.379	124282	4.56	8627
3		0.567	57528	2.11	6749
4		1.775	366063	13.42	49345
5		2.117	65088	2.39	4993
6		2.350	31851	1.17	3515
7		2.569	17313	0.63	2698
8		2.723	60789	2.23	3234
9		3.038	27971	1.03	3635
10		4.296	10884	0.40	1512
11		5.526	6816	0.25	620
12		7.600	1725	0.06	270

Reported by User: Saneesh (Saneesh)   Project Name: HDI_Stellenbosch_2016

	Peak Name	RT	Area	% Area	Height
13		7.724	5890	0.22	586
14	25ODESRIF	7.913	27851	1.02	3303
15	RIF	8.339	1292180	47.38	158062
16		8.983	3669	0.13	725
17		9.642	1546	0.06	229
18		9.888	1168	0.04	188
19		10.133	4560	0.17	356
20	NEO	10.852	510177	18.71	61803

Reported by User: Saneesh (Saneesh) Project Name: HDI_Stellenbosch_2016

SAMPLE INFORMATION

Sample Name:	RIF Inc Basil_MeOH 50ug/mL 2	Acquired By:	Saneesh
Sample Type:	Unknown	Date Acquired:	2017/09/11 09:49:48 PM
Vial:	9	Acq. Method Set:	HDI_RIF_25ODESRIF_NEO
Injection #:	1	Date Processed:	2017/09/12 11:47:02 AM
Injection Volume:	15.00 ul	Processing Method:	HDI_RIF_ W2996 254.0nm-1.2
Run Time:	11.5 Minutes	Channel Name:	W2996 254.0nm-1.2
Sample Set Name:	RIF_Basil MeOH IC50 11_9_17	Proc. Chnl. Descr.:	W2996 PDA 254.0 nm at 1.2

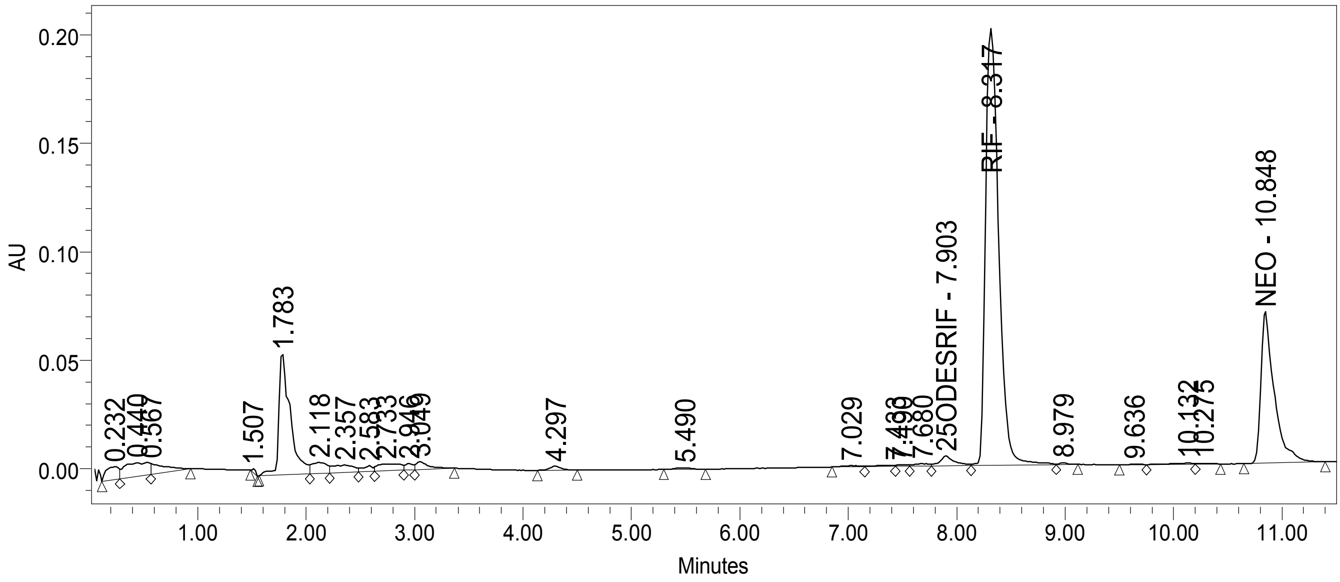

	Peak Name	RT	Area	% Area	Height
1		0.232	47968	1.49	5769
2		0.440	95495	2.97	6182
3		0.567	44294	1.38	5030
4		1.507	4731	0.15	1698
5		1.783	409835	12.75	55338
6		2.118	49593	1.54	5104
7		2.357	47374	1.47	3380
8		2.583	18259	0.57	2562
9		2.733	46329	1.44	3204
10		2.946	15383	0.48	2878
11		3.049	29034	0.90	3532
12		4.297	13203	0.41	1797

Reported by User: Saneesh (Saneesh)   Project Name: HDI_Stellenbosch_2016

	Peak Name	RT	Area	% Area	Height
13		5.490	7888	0.25	746
14		7.029	4699	0.15	419
15		7.433	5910	0.18	458
16		7.490	4366	0.14	605
17		7.680	10442	0.32	1011
18	25ODESRIF	7.903	41261	1.28	4464
19	RIF	8.317	1699817	52.87	201165
20		8.979	4209	0.13	787
21		9.636	3290	0.10	385
22		10.132	10283	0.32	602
23		10.275	4270	0.13	399
24	NEO	10.848	597116	18.57	69748

Reported by User: Saneesh (Saneesh) Project Name: HDI_Stellenbosch_2016

SAMPLE INFORMATION

Sample Name:	RIF Inc Basil_MeOH 50ug/mL 3	Acquired By:	Saneesh
Sample Type:	Unknown	Date Acquired:	2017/09/11 10:04:30 PM
Vial:	10	Acq. Method Set:	HDI_RIF_25ODESRIF_NEO
Injection #:	1	Date Processed:	2017/09/12 11:47:02 AM
Injection Volume:	15.00 ul	Processing Method:	HDI_RIF_ W2996 254.0nm-1.2
Run Time:	11.5 Minutes	Channel Name:	W2996 254.0nm-1.2
Sample Set Name:	RIF_Basil MeOH IC50 11_9_17	Proc. Chnl. Descr.:	W2996 PDA 254.0 nm at 1.2

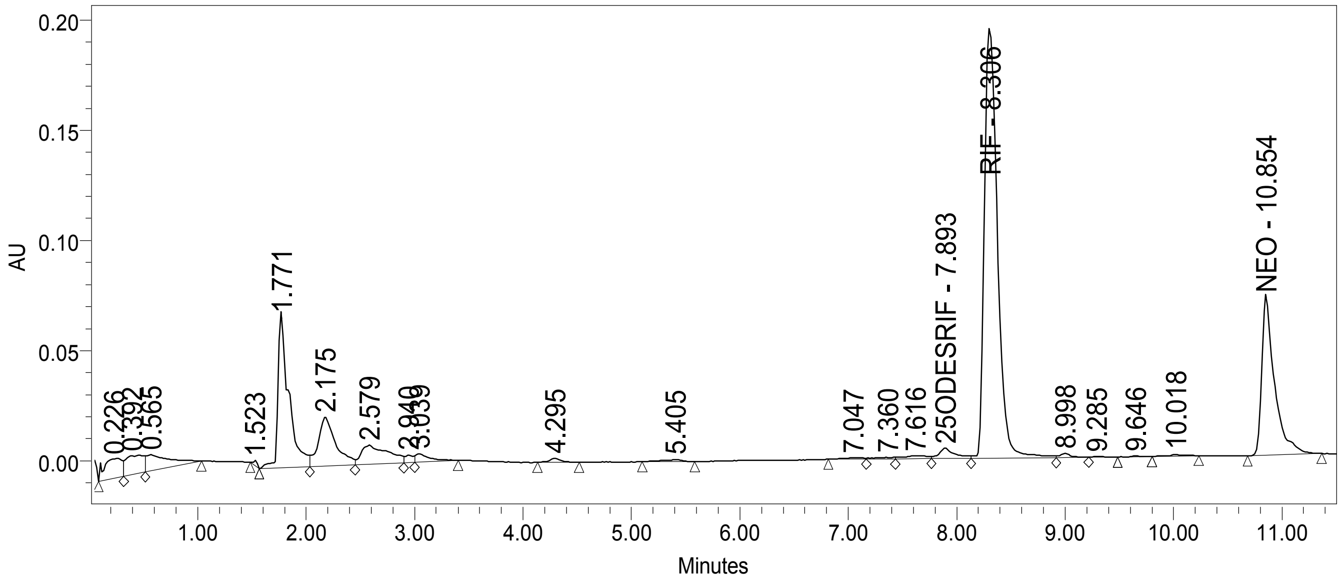

	Peak Name	RT	Area	% Area	Height
1		0.226	102243	2.84	8659
2		0.392	95750	2.66	8478
3		0.565	108419	3.01	7341
4		1.523	6130	0.17	2097
5		1.771	480154	13.35	69532
6		2.175	245272	6.82	22140
7		2.579	145610	4.05	8469
8		2.940	17507	0.49	3255
9		3.039	33618	0.93	3804
10		4.295	13888	0.39	1901
11		5.405	11646	0.32	1018
12		7.047	7159	0.20	559

	Peak Name	RT	Area	% Area	Height
13		7.360	8143	0.23	618
14		7.616	19624	0.55	1196
15	25ODESRIF	7.893	46957	1.31	4679
16	RIF	8.306	1635336	45.48	195730
17		8.998	12827	0.36	1807
18		9.285	2543	0.07	312
19		9.646	2384	0.07	353
20		10.018	7582	0.21	745
21	NEO	10.854	593254	16.50	72506

Multi Sample Summary

Reported by User: Saneesh (Saneesh)   Project Name: HDI_Stellenbosch_2016

SAMPLE INFORMATION

Sample Name:	RIF Inc Basil_MeOH 100ug/mL 1	Acquired By:	Saneesh
Sample Type:	Unknown	Date Acquired:	2017/09/11 10:19:14 PM
Vial:	11	Acq. Method Set:	HDI_RIF_25ODESRIF_NEO
Injection #:	1	Date Processed:	2017/09/12 11:47:02 AM
Injection Volume:	15.00 ul	Processing Method:	HDI_RIF_ W2996 254.0nm-1.2
Run Time:	11.5 Minutes	Channel Name:	W2996 254.0nm-1.2
Sample Set Name:	RIF_Basil MeOH IC50 11_9_17	Proc. Chnl. Descr.:	W2996 PDA 254.0 nm at 1.2

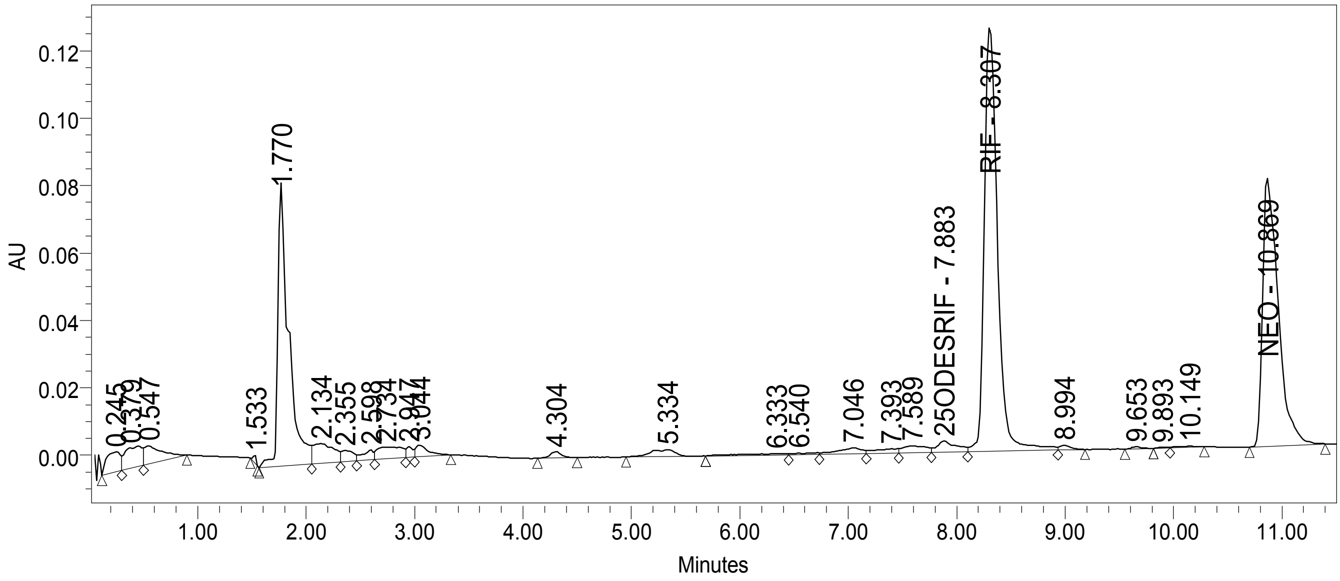

	Peak Name	RT	Area	% Area	Height
1		0.245	53501	1.75	5951
2		0.379	67243	2.21	6097
3		0.547	62745	2.06	5367
4		1.533	4695	0.15	2495
5		1.770	571364	18.74	82285
6		2.134	78226	2.57	5838
7		2.355	25814	0.85	3356
8		2.598	21835	0.72	2805
9		2.734	55337	1.82	3588
10		2.947	13897	0.46	3164
11		3.044	28181	0.92	3524
12		4.304	14204	0.47	1876

Reported by User: Saneesh (Saneesh) Project Name: HDI_Stellenbosch_2016

	Peak Name	RT	Area	% Area	Height
13		5.334	34347	1.13	2087
14		6.333	16119	0.53	454
15		6.540	6609	0.22	457
16		7.046	25632	0.84	1749
17		7.393	18813	0.62	1260
18		7.589	29408	0.96	1963
19	25ODESRIF	7.883	39731	1.30	3256
20	RIF	8.307	1064268	34.91	126226
21		8.994	9572	0.31	1377
22		9.653	3581	0.12	569
23		9.893	1630	0.05	253
24		10.149	4558	0.15	386
25	NEO	10.869	797239	26.15	79354

Reported by User: Saneesh (Saneesh)   Project Name: HDI_Stellenbosch_2016

SAMPLE INFORMATION

Sample Name:	RIF Inc Basil_MeOH 100ug/mL 2	Acquired By:	Saneesh
Sample Type:	Unknown	Date Acquired:	2017/09/11 10:33:57 PM
Vial:	12	Acq. Method Set:	HDI_RIF_25ODESRIF_NEO
Injection #:	1	Date Processed:	2017/09/12 11:47:02 AM
Injection Volume:	15.00 ul	Processing Method:	HDI_RIF_ W2996 254.0nm-1.2
Run Time:	11.5 Minutes	Channel Name:	W2996 254.0nm-1.2
Sample Set Name:	RIF_Basil MeOH IC50 11_9_17	Proc. Chnl. Descr.:	W2996 PDA 254.0 nm at 1.2

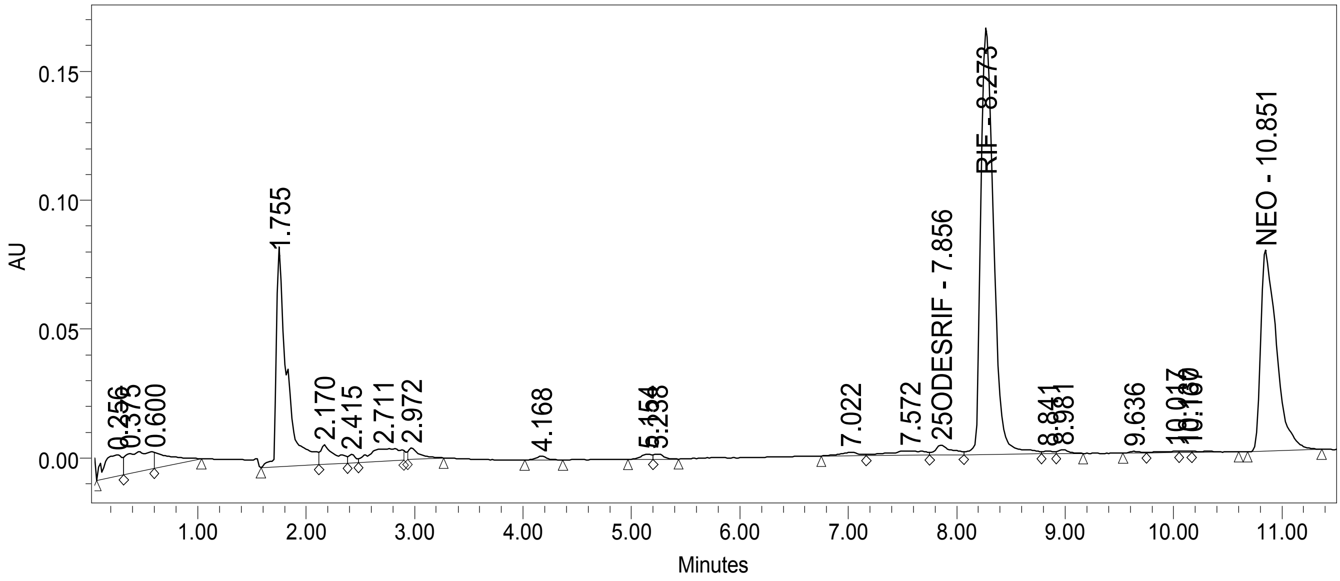

	Peak Name	RT	Area	% Area	Height
1		0.256	98409	2.90	8026
2		0.375	120175	3.54	7761
3		0.600	67185	1.98	6149
4		1.755	578868	17.06	83329
5		2.170	71938	2.12	7490
6		2.415	14692	0.43	3265
7		2.711	95736	2.82	4698
8		2.972	32096	0.95	4364
9		4.168	11160	0.33	1470
10		5.154	14649	0.43	1994
11		5.238	12618	0.37	1986
12		7.022	17962	0.53	1441

Reported by User: Saneesh (Saneesh) Project Name: HDI_Stellenbosch_2016

	Peak Name	RT	Area	% Area	Height
13		7.572	38787	1.14	1637
14	25ODESRIF	7.856	39112	1.15	3732
15	RIF	8.273	1360166	40.07	166109
16		8.841	6976	0.21	1002
17		8.981	9553	0.28	1407
18		9.636	3834	0.11	568
19		10.017	5667	0.17	482
20		10.130	3493	0.10	579
21		10.167	4966	0.15	451
22	NEO	10.851	786061	23.16	77921

Reported by User: Saneesh (Saneesh)   Project Name: HDI_Stellenbosch_2016

SAMPLE INFORMATION

Sample Name:	RIF Inc Basil_MeOH 100ug/mL 3	Acquired By:	Saneesh
Sample Type:	Unknown	Date Acquired:	2017/09/11 10:48:39 PM
Vial:	13	Acq. Method Set:	HDI_RIF_25ODESRIF_NEO
Injection #:	1	Date Processed:	2017/09/12 11:47:02 AM
Injection Volume:	15.00 ul	Processing Method:	HDI_RIF_ W2996 254.0nm-1.2
Run Time:	11.5 Minutes	Channel Name:	W2996 254.0nm-1.2
Sample Set Name:	RIF_Basil MeOH IC50 11_9_17	Proc. Chnl. Descr.:	W2996 PDA 254.0 nm at 1.2

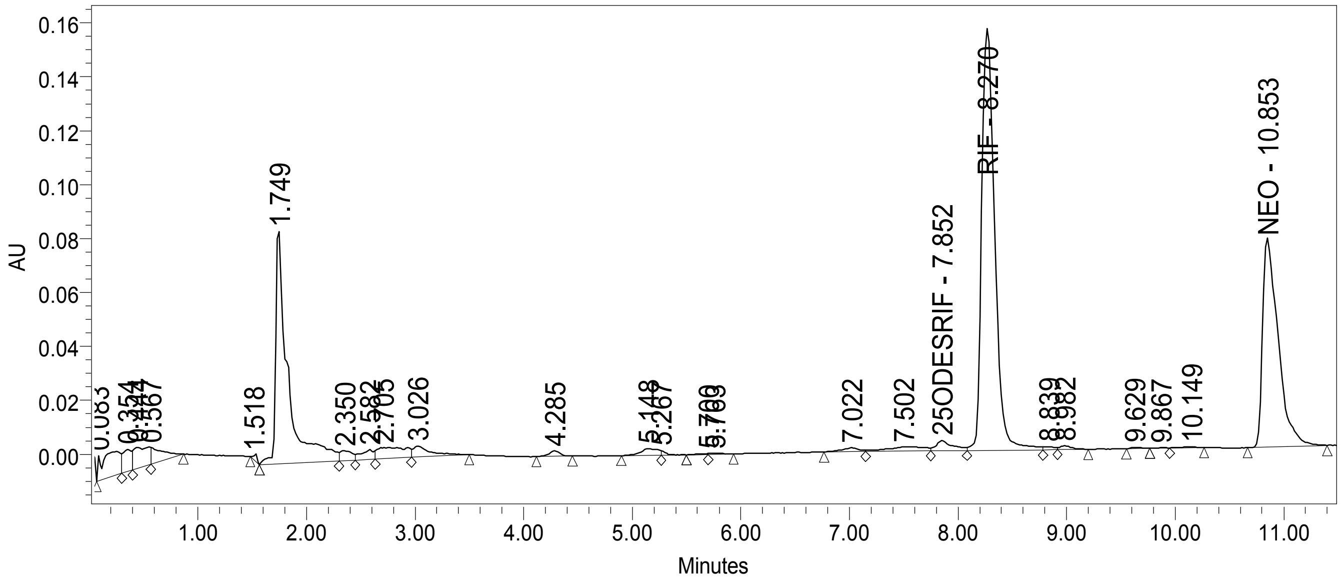

	Peak Name	RT	Area	% Area	Height
1		0.083	106226	3.14	9290
2		0.354	45935	1.36	8197
3		0.444	70189	2.07	7846
4		0.567	48660	1.44	6201
5		1.518	5319	0.16	1779
6		1.749	674313	19.91	85221
7		2.350	27699	0.82	3542
8		2.582	28984	0.86	3338
9		2.705	73776	2.18	4065
10		3.026	46965	1.39	4182
11		4.285	13772	0.41	1952
12		5.148	27649	0.82	2465

	Peak Name	RT	Area	% Area	Height
13		5.267	4829	0.14	1688
14		5.700	2029	0.06	340
15		5.763	3408	0.10	424
16		7.022	15328	0.45	1365
17		7.502	38684	1.14	1505
18	25ODESRIF	7.852	40495	1.20	3814
19	RIF	8.270	1318464	38.94	156545
20		8.839	7799	0.23	1089
21		8.982	10643	0.31	1434
22		9.629	3206	0.09	555
23		9.867	1181	0.03	181
24		10.149	3603	0.11	290
25	NEO	10.853	766884	22.65	77542

Reported by User: Saneesh (Saneesh) Project Name: HDI_Stellenbosch_2016

SAMPLE INFORMATION

Sample Name:	RIF Inc Basil_MeOH 150ug/mL 1	Acquired By:	Saneesh
Sample Type:	Unknown	Date Acquired:	2017/09/11 11:03:24 PM
Vial:	14	Acq. Method Set:	HDI_RIF_25ODESRIF_NEO
Injection #:	1	Date Processed:	2017/09/12 11:47:03 AM
Injection Volume:	15.00 ul	Processing Method:	HDI_RIF_ W2996 254.0nm-1.2
Run Time:	11.5 Minutes	Channel Name:	W2996 254.0nm-1.2
Sample Set Name:	RIF_Basil MeOH IC50 11_9_17	Proc. Chnl. Descr.:	W2996 PDA 254.0 nm at 1.2

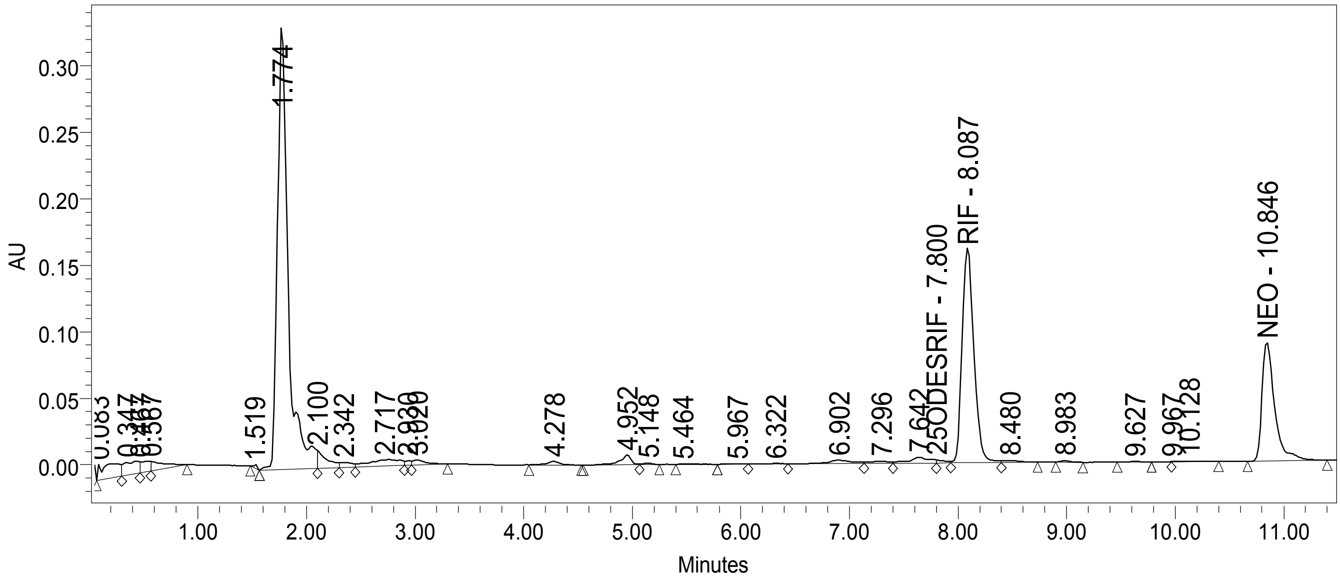

	Peak Name	RT	Area	% Area	Height
1		0.083	126450	2.51	11530
2		0.347	89516	1.77	9487
3		0.467	46233	0.92	8236
4		0.567	63123	1.25	7188
5		1.519	5662	0.11	1888
6		1.774	2269309	44.98	321044
7		2.100	80536	1.60	13368
8		2.342	29042	0.58	3647
9		2.717	95909	1.90	4573
10		2.930	12362	0.25	3179
11		3.020	30583	0.61	3698
12		4.278	22863	0.45	2806

	Peak Name	RT	Area	% Area	Height
13		4.952	54894	1.09	7218
14		5.148	7285	0.14	1245
15		5.464	3468	0.07	322
16		5.967	2019	0.04	179
17		6.322	5879	0.12	480
18		6.902	41549	0.82	2510
19		7.296	19952	0.40	1602
20		7.642	57252	1.13	4385
21	25ODESRIF	7.800	11967	0.24	2211
22	RIF	8.087	1214355	24.07	161478
23		8.480	14932	0.30	1400
24		8.983	6486	0.13	1130
25		9.627	4271	0.08	472
26		9.967	1831	0.04	257
27		10.128	7572	0.15	470
28	NEO	10.846	720242	14.27	88779

SAMPLE INFORMATION

Sample Name:	RIF Inc Basil_MeOH 150ug/mL 2	Acquired By:	Saneesh
Sample Type:	Unknown	Date Acquired:	2017/09/11 11:18:07 PM
Vial:	15	Acq. Method Set:	HDI_RIF_25ODESRIF_NEO
Injection #:	1	Date Processed:	2017/09/12 11:47:03 AM
Injection Volume:	15.00 ul	Processing Method:	HDI_RIF_ W2996 254.0nm-1.2
Run Time:	11.5 Minutes	Channel Name:	W2996 254.0nm-1.2
Sample Set Name:	RIF_Basil MeOH IC50 11_9_17	Proc. Chnl. Descr.:	W2996 PDA 254.0 nm at 1.2

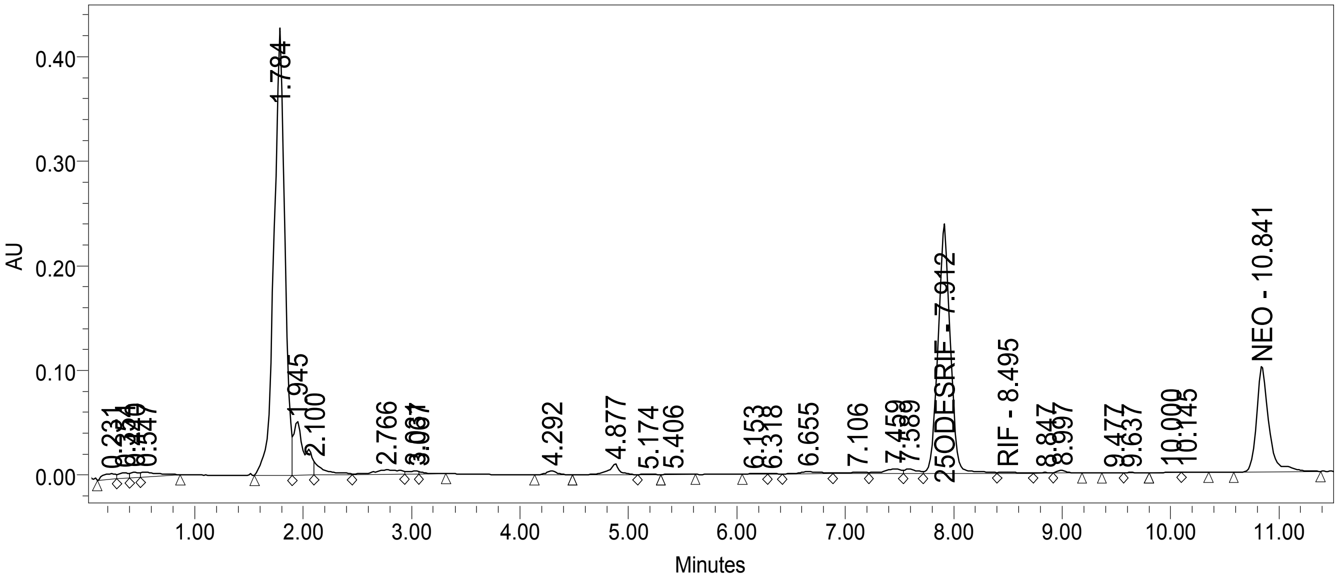

	Peak Name	RT	Area	% Area	Height
1		0.231	43953	0.66	5193
2		0.354	34191	0.52	5421
3		0.440	29483	0.45	5360
4		0.547	50685	0.77	4699
5		1.784	2946170	44.49	410769
6		1.945	366995	5.54	50389
7		2.100	78446	1.18	13030
8		2.766	73491	1.11	4160
9		3.031	21511	0.32	3118
10		3.067	9482	0.14	2346
11		4.292	27421	0.41	4005
12		4.877	75768	1.14	10145

Reported by User: Saneesh (Saneesh) Project Name: HDI_Stellenbosch_2016

	Peak Name	RT	Area	% Area	Height
13		5.174	3468	0.05	442
14		5.406	5726	0.09	611
15		6.153	2982	0.05	325
16		6.318	2257	0.03	467
17		6.655	31870	0.48	2185
18		7.106	24273	0.37	1586
19		7.459	50832	0.77	4458
20		7.589	35039	0.53	4435
21	25ODESRIF	7.912	1871851	28.26	236050
22	RIF	8.495	13488	0.20	1122
23		8.847	4601	0.07	633
24		8.997	15271	0.23	2624
25		9.477	1876	0.03	281
26		9.637	2091	0.03	354
27		10.000	4078	0.06	449
28		10.145	4698	0.07	503
29	NEO	10.841	790626	11.94	101403

SAMPLE INFORMATION

Sample Name:	RIF Inc Basil_MeOH 150ug/mL 3	Acquired By:	Saneesh
Sample Type:	Unknown	Date Acquired:	2017/09/11 11:32:50 PM
Vial:	16	Acq. Method Set:	HDI_RIF_25ODESRIF_NEO
Injection #:	1	Date Processed:	2017/09/12 11:47:03 AM
Injection Volume:	15.00 ul	Processing Method:	HDI_RIF_ W2996 254.0nm-1.2
Run Time:	11.5 Minutes	Channel Name:	W2996 254.0nm-1.2
Sample Set Name:	RIF_Basil MeOH IC50 11_9_17	Proc. Chnl. Descr.:	W2996 PDA 254.0 nm at 1.2

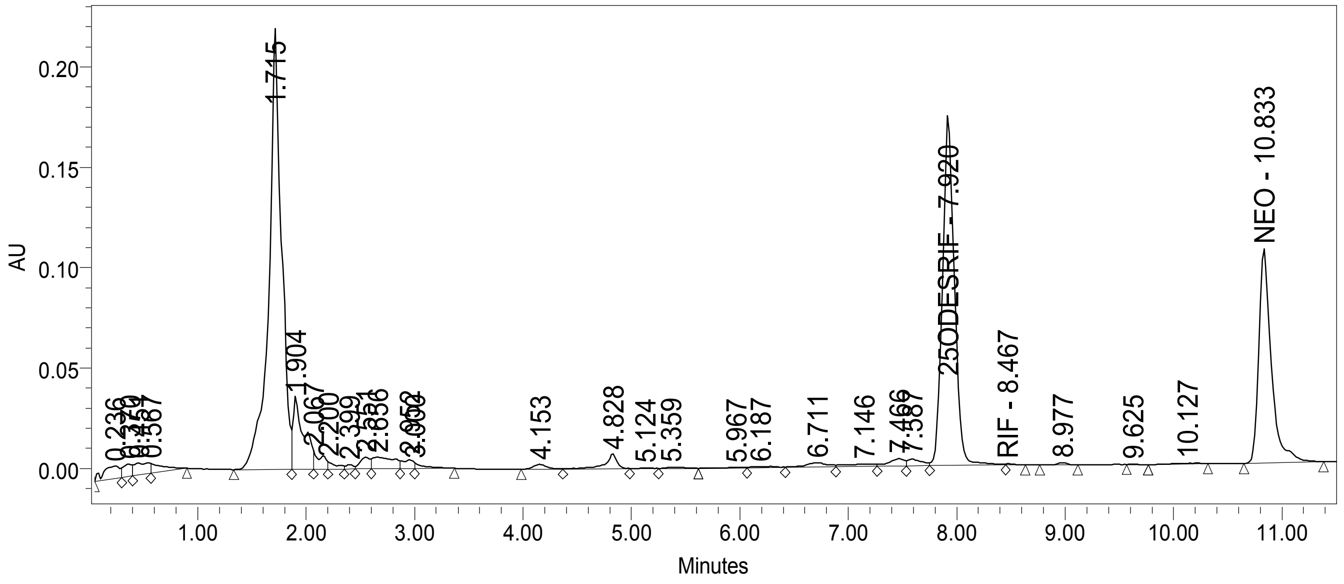

	Peak Name	RT	Area	% Area	Height
1		0.236	66144	1.35	6024
2		0.370	33923	0.69	6116
3		0.451	54279	1.11	5894
4		0.567	40321	0.83	4981
5		1.715	1777951	36.40	214539
6		1.904	249217	5.10	34364
7		2.067	46503	0.95	8947
8		2.200	19004	0.39	3734
9		2.399	9628	0.20	2098
10		2.551	36879	0.76	5721
11		2.656	79503	1.63	5622
12		2.952	30328	0.62	4385

Reported by User: Saneesh (Saneesh) Project Name: HDI_Stellenbosch_2016

	Peak Name	RT	Area	% Area	Height
13		3.000	16144	0.33	2831
14		4.153	19850	0.41	2361
15		4.828	57445	1.18	7262
16		5.124	3708	0.08	388
17		5.359	7726	0.16	700
18		5.967	4289	0.09	304
19		6.187	9158	0.19	593
20		6.711	30051	0.62	2164
21		7.146	23747	0.49	1296
22		7.466	37479	0.77	3796
23		7.587	32136	0.66	3476
24	25ODESRIF	7.920	1326784	27.16	172613
25	RIF	8.467	3115	0.06	654
26		8.977	8835	0.18	1196
27		9.625	1465	0.03	282
28		10.127	7843	0.16	406
29	NEO	10.833	850920	17.42	106238

Reported by User: Saneesh (Saneesh)

Project Name: HDI_Stellenbosch_2016

SAMPLE INFORMATION

Sample Name:	RIF Inc Basil_MeOH 200ug/mL 1	Acquired By:	Saneesh
Sample Type:	Unknown	Date Acquired:	2017/09/11 11:47:34 PM
Vial:	17	Acq. Method Set:	HDI_RIF_25ODESRIF_NEO
Injection #:	1	Date Processed:	2017/09/12 11:47:03 AM
Injection Volume:	15.00 ul	Processing Method:	HDI_RIF_ W2996 254.0nm-1.2
Run Time:	11.5 Minutes	Channel Name:	W2996 254.0nm-1.2
Sample Set Name:	RIF_Basil MeOH IC50 11_9_17	Proc. Chnl. Descr.:	W2996 PDA 254.0 nm at 1.2

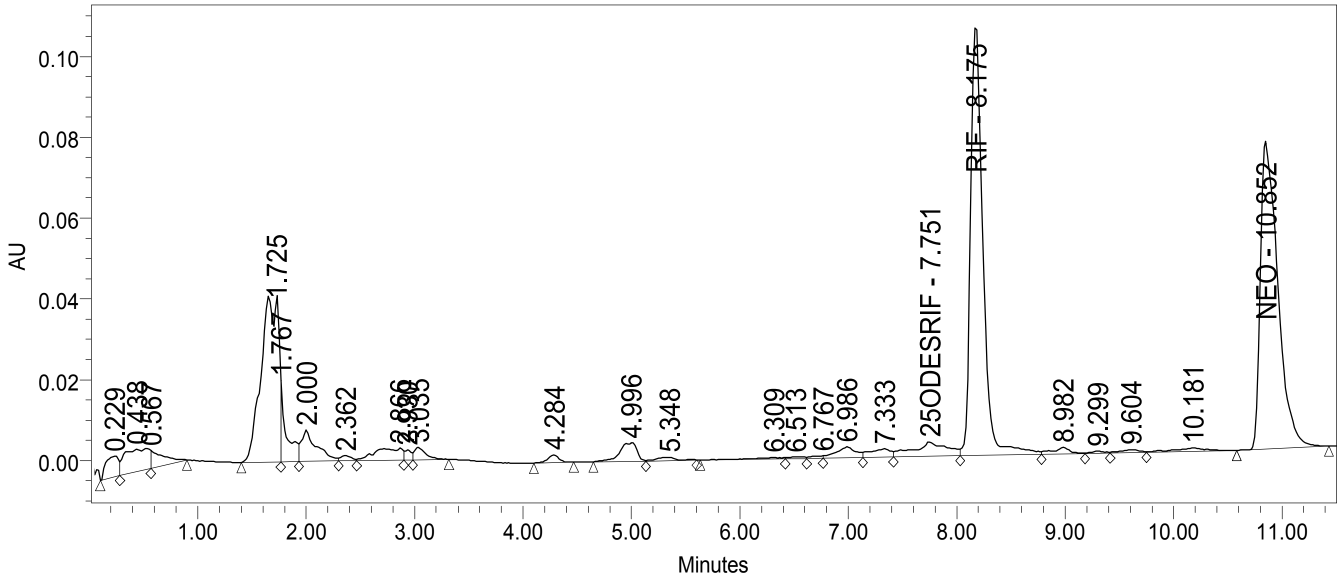

	Peak Name	RT	Area	% Area	Height
1		0.229	42312	1.49	5167
2		0.438	86102	3.04	5523
3		0.567	35749	1.26	4408
4		1.725	457605	16.14	39975
5		1.767	66961	2.36	20039
6		2.000	75733	2.67	7447
7		2.362	8935	0.32	1293
8		2.866	52128	1.84	2917
9		2.930	11056	0.39	2455
10		3.035	22908	0.81	3066
11		4.284	13721	0.48	1884
12		4.996	50373	1.78	4545

Reported by User: Saneesh (Saneesh) Project Name: HDI_Stellenbosch_2016

	Peak Name	RT	Area	% Area	Height
13		5.348	11717	0.41	854
14		6.309	8236	0.29	403
15		6.513	4356	0.15	435
16		6.767	4068	0.14	545
17		6.986	33921	1.20	2665
18		7.333	27107	0.96	2047
19	25ODESRIF	7.751	76038	2.68	3514
20	RIF	8.175	848431	29.93	107102
21		8.982	16886	0.60	1565
22		9.299	3862	0.14	499
23		9.604	8250	0.29	735
24		10.181	17413	0.61	819
25	NEO	10.852	850953	30.02	76247

SAMPLE INFORMATION

Sample Name:	RIF Inc Basil_MeOH 200ug/mL 2	Acquired By:	Saneesh
Sample Type:	Unknown	Date Acquired:	2017/09/12 12:02:16 AM
Vial:	18	Acq. Method Set:	HDI_RIF_25ODESRIF_NEO
Injection #:	1	Date Processed:	2017/09/12 11:47:03 AM
Injection Volume:	15.00 ul	Processing Method:	HDI_RIF_ W2996 254.0nm-1.2
Run Time:	11.5 Minutes	Channel Name:	W2996 254.0nm-1.2
Sample Set Name:	RIF_Basil MeOH IC50 11_9_17	Proc. Chnl. Descr.:	W2996 PDA 254.0 nm at 1.2

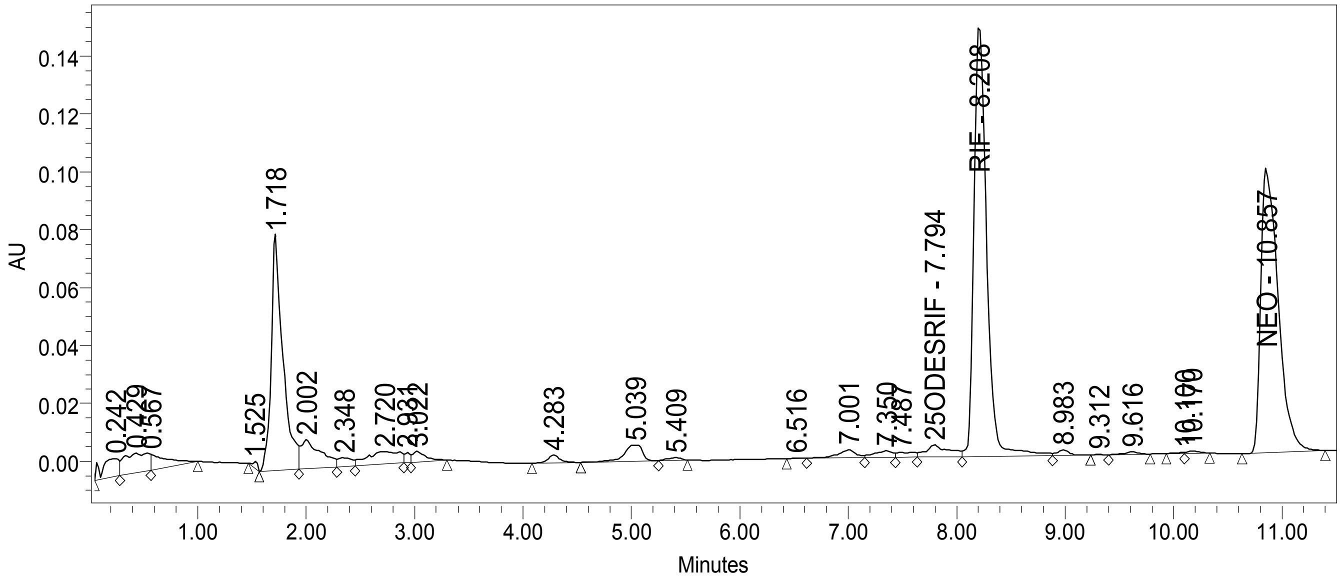

	Peak Name	RT	Area	% Area	Height
1		0.242	67915	1.82	6215
2		0.429	102821	2.76	6732
3		0.567	58375	1.57	5566
4		1.525	7864	0.21	2390
5		1.718	612250	16.43	81489
6		2.002	127744	3.43	9888
7		2.348	26554	0.71	3027
8		2.720	88979	2.39	4218
9		2.931	12484	0.34	3322
10		3.022	30830	0.83	3641
11		4.283	21185	0.57	2621
12		5.039	66023	1.77	5599

Reported by User: Saneesh (Saneesh) Project Name: HDI_Stellenbosch_2016

	Peak Name	RT	Area	% Area	Height
13		5.409	9356	0.25	979
14		6.516	1176	0.03	216
15		7.001	33688	0.90	2890
16		7.350	27778	0.75	2309
17		7.487	19379	0.52	1731
18	25ODESRIF	7.794	62387	1.67	4141
19	RIF	8.208	1223517	32.84	149570
20		8.983	14381	0.39	1879
21		9.312	1521	0.04	284
22		9.616	8296	0.22	866
23		10.100	2202	0.06	389
24		10.170	5443	0.15	727
25	NEO	10.857	1093833	29.36	98780

Reported by User: Saneesh (Saneesh) Project Name: HDI_Stellenbosch_2016

SAMPLE INFORMATION

Sample Name:	RIF Inc Basil_MeOH 200ug/mL 3	Acquired By:	Saneesh
Sample Type:	Unknown	Date Acquired:	2017/09/12 12:17:00 AM
Vial:	19	Acq. Method Set:	HDI_RIF_25ODESRIF_NEO
Injection #:	1	Date Processed:	2017/09/12 11:47:03 AM
Injection Volume:	15.00 ul	Processing Method:	HDI_RIF_ W2996 254.0nm-1.2
Run Time:	11.5 Minutes	Channel Name:	W2996 254.0nm-1.2
Sample Set Name:	RIF_Basil MeOH IC50 11_9_17	Proc. Chnl. Descr.:	W2996 PDA 254.0 nm at 1.2

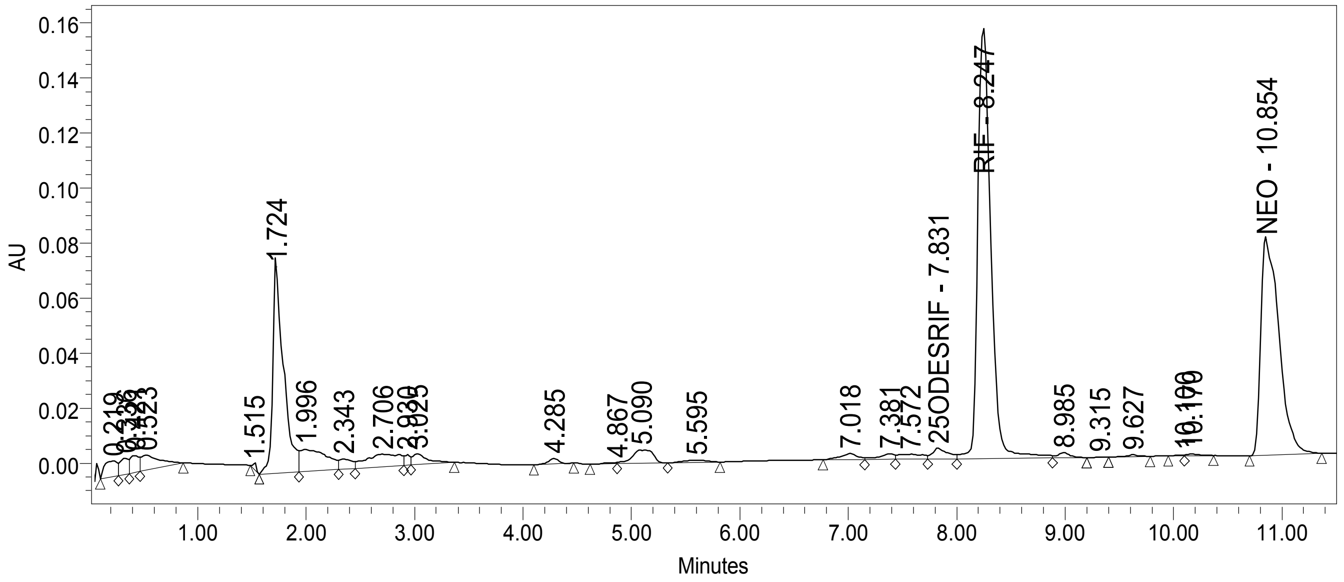

	Peak Name	RT	Area	% Area	Height
1		0.219	46277	1.32	5605
2		0.336	31589	0.90	5734
3		0.421	34038	0.97	6136
4		0.523	64346	1.84	5380
5		1.515	5217	0.15	1710
6		1.724	534859	15.26	76992
7		1.996	134808	3.85	7951
8		2.343	28522	0.81	3652
9		2.706	98657	2.81	4549
10		2.930	14049	0.40	3761
11		3.025	38488	1.10	4027
12		4.285	13864	0.40	2037

Reported by User: Saneesh (Saneesh) Project Name: HDI_Stellenbosch_2016

	Peak Name	RT	Area	% Area	Height
13		4.867	3350	0.10	495
14		5.090	65270	1.86	4884
15		5.595	12653	0.36	841
16		7.018	23973	0.68	2266
17		7.381	19391	0.55	1974
18		7.572	28434	0.81	1770
19	25ODESRIF	7.831	38502	1.10	3837
20	RIF	8.247	1317353	37.58	156383
21		8.985	16192	0.46	1939
22		9.315	1217	0.03	247
23		9.627	6806	0.19	825
24		10.100	1816	0.05	334
25		10.170	5282	0.15	651
26	NEO	10.854	920797	26.27	79590

Multi Sample Summary

Reported by User: Saneesh (Saneesh)   Project Name: HDI_Stellenbosch_2016

SAMPLE INFORMATION

Sample Name:	RIF Inc Basil_MeOH 1ug/mL 1	Acquired By:	Saneesh
Sample Type:	Unknown	Date Acquired:	2017/09/11 08:06:45 PM
Vial:	2	Acq. Method Set:	HDI_RIF_25ODESRIF_NEO
Injection #:	1	Date Processed:	2017/09/12 11:47:02 AM
Injection Volume:	15.00 ul	Processing Method:	HDI_RIF_ W2996 254.0nm-1.2
Run Time:	11.5 Minutes	Channel Name:	W2996 254.0nm-1.2
Sample Set Name:	RIF_Basil MeOH IC50 11_9_17	Proc. Chnl. Descr.:	W2996 PDA 254.0 nm at 1.2

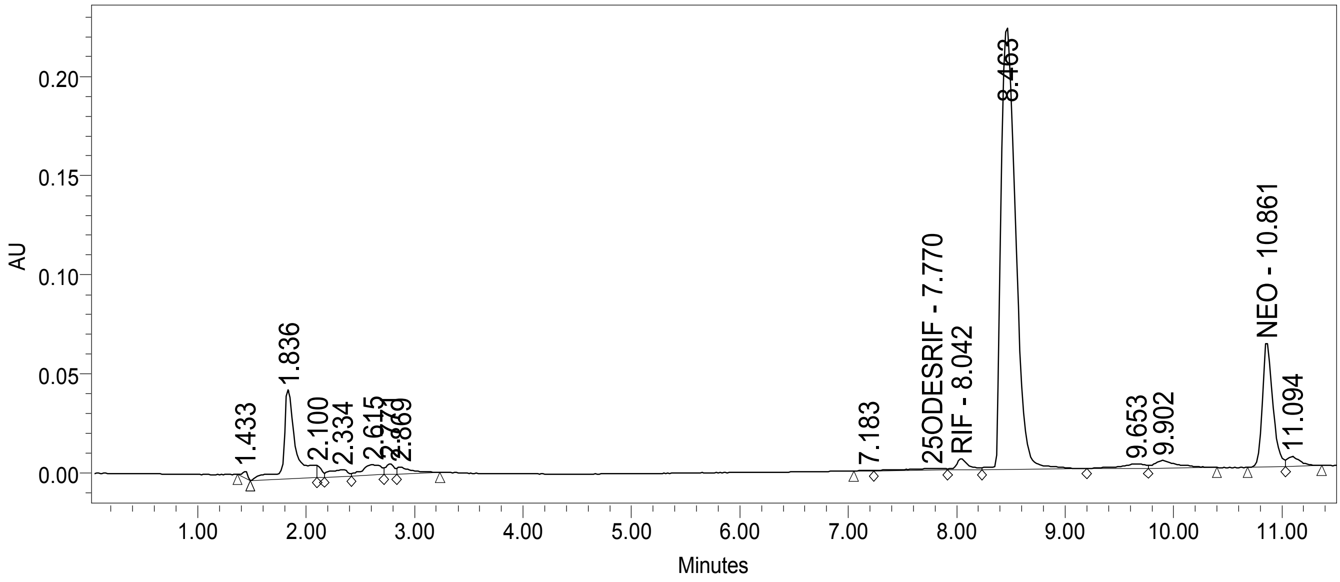

	Peak Name	RT	Area	% Area	Height
1		1.433	11031	0.34	3028
2		1.836	351423	10.83	45611
3		2.100	16747	0.52	5992
4		2.334	40251	1.24	3194
5		2.615	63485	1.96	5161
6		2.771	28640	0.88	5245
7		2.869	30874	0.95	3447
8		7.183	2099	0.06	299
9	25ODESRIF	7.770	21595	0.67	756
10	RIF	8.042	43410	1.34	5576
11		8.463	2081397	64.16	222941
12		9.653	37062	1.14	2320
13		9.902	55701	1.72	3830
14	NEO	10.861	420025	12.95	62408

Reported by User: Saneesh (Saneesh) Project Name: HDI_Stellenbosch_2016

	Peak Name	RT	Area	% Area	Height
15		11.094	40470	1.25	4859

Multi Sample Summary

Reported by User: Saneesh (Saneesh)   Project Name: HDI_Stellenbosch_2016

SAMPLE INFORMATION

Sample Name:	RIF Inc Basil_MeOH 1ug/mL 2	Acquired By:	Saneesh
Sample Type:	Unknown	Date Acquired:	2017/09/11 08:21:30 PM
Vial:	3	Acq. Method Set:	HDI_RIF_25ODESRIF_NEO
Injection #:	1	Date Processed:	2017/09/12 11:47:02 AM
Injection Volume:	15.00 ul	Processing Method:	HDI_RIF_ W2996 254.0nm-1.2
Run Time:	11.5 Minutes	Channel Name:	W2996 254.0nm-1.2
Sample Set Name:	RIF_Basil MeOH IC50 11_9_17	Proc. Chnl. Descr.:	W2996 PDA 254.0 nm at 1.2

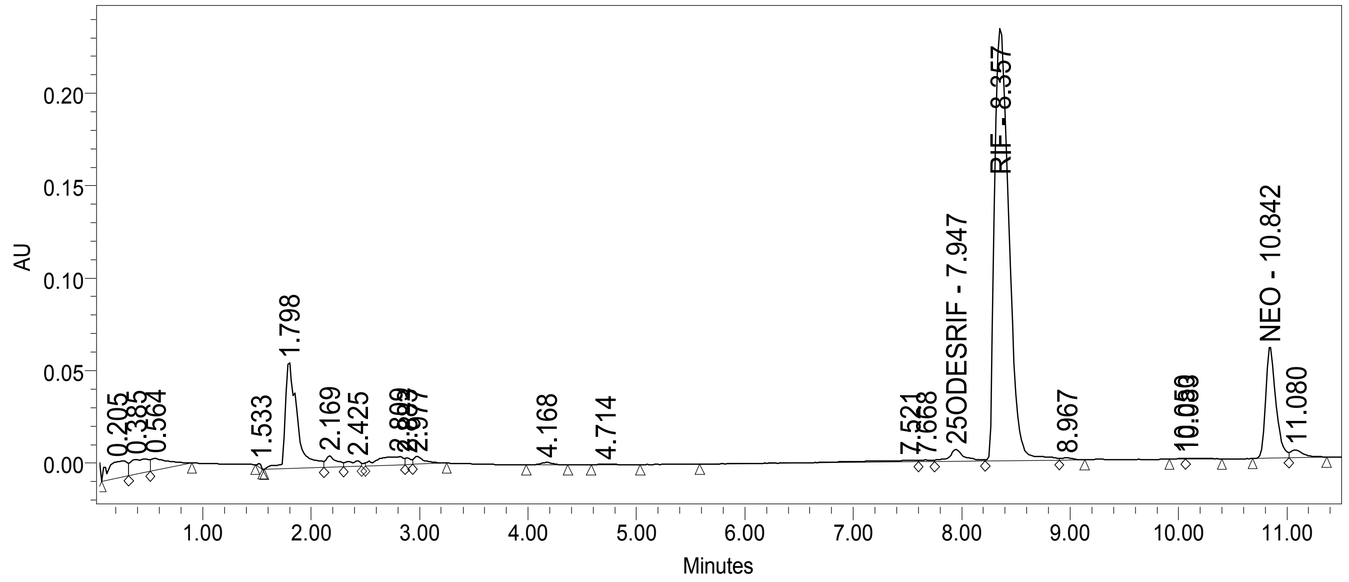

	Peak Name	RT	Area	% Area	Height
1		0.205	110553	3.05	8807
2		0.385	87919	2.43	8027
3		0.564	74220	2.05	6368
4		1.533	4650	0.13	2109
5		1.798	448937	12.41	56721
6		2.169	42968	1.19	6169
7		2.425	23662	0.65	2831
8		2.809	75296	2.08	4234
9		2.883	12480	0.34	3646
10		2.977	29474	0.81	4088
11		4.168	10287	0.28	1274
12		4.714	3787	0.10	292

	Peak Name	RT	Area	% Area	Height
13		7.521	34345	0.95	842
14		7.668	7121	0.20	848
15	25ODESRIF	7.947	60577	1.67	6454
16	RIF	8.357	2150103	59.41	234971
17		8.967	7737	0.21	1048
18		10.050	1649	0.05	305
19		10.083	4765	0.13	433
20	NEO	10.842	393651	10.88	60786
21		11.080	34795	0.96	4126

SAMPLE INFORMATION

Sample Name:	RIF Inc Basil_MeOH 1ug/mL 3	Acquired By:	Saneesh
Sample Type:	Unknown	Date Acquired:	2017/09/11 08:36:13 PM
Vial:	4	Acq. Method Set:	HDI_RIF_25ODESRIF_NEO
Injection #:	1	Date Processed:	2017/09/12 11:47:02 AM
Injection Volume:	15.00 ul	Processing Method:	HDI_RIF_ W2996 254.0nm-1.2
Run Time:	11.5 Minutes	Channel Name:	W2996 254.0nm-1.2
Sample Set Name:	RIF_Basil MeOH IC50 11_9_17	Proc. Chnl. Descr.:	W2996 PDA 254.0 nm at 1.2

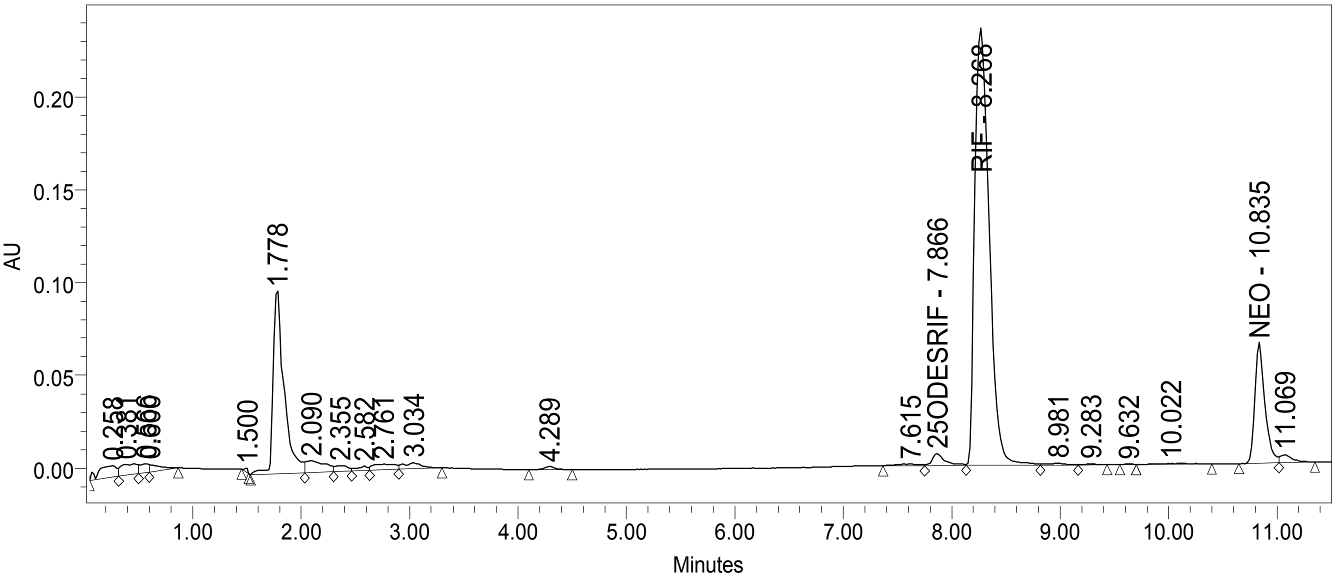

	Peak Name	RT	Area	% Area	Height
1		0.258	74285	1.95	6100
2		0.381	58557	1.53	5894
3		0.566	27045	0.71	4668
4		0.600	26075	0.68	4000
5		1.500	4271	0.11	2239
6		1.778	687132	18.00	97261
7		2.090	77123	2.02	6158
8		2.355	25554	0.67	3142
9		2.582	16608	0.44	2258
10		2.761	42990	1.13	2950
11		3.034	37604	0.99	3314
12		4.289	12672	0.33	1753

Reported by User: Saneesh (Saneesh)   Project Name: HDI_Stellenbosch_2016

	Peak Name	RT	Area	% Area	Height
13		7.615	11608	0.30	764
14	25ODESRIF	7.866	50904	1.33	6434
15	RIF	8.268	2187092	57.31	235718
16		8.981	13213	0.35	1109
17		9.283	4386	0.11	632
18		9.632	1263	0.03	238
19		10.022	8868	0.23	413
20	NEO	10.835	416044	10.90	64642
21		11.069	33237	0.87	4193

SAMPLE INFORMATION

Sample Name:	RIF Inc Basil_MeOH 10ug/mL 1	Acquired By:	Saneesh
Sample Type:	Unknown	Date Acquired:	2017/09/11 08:50:56 PM
Vial:	5	Acq. Method Set:	HDI_RIF_25ODESRIF_NEO
Injection #:	1	Date Processed:	2017/09/12 11:47:02 AM
Injection Volume:	15.00 ul	Processing Method:	HDI_RIF_ W2996 254.0nm-1.2
Run Time:	11.5 Minutes	Channel Name:	W2996 254.0nm-1.2
Sample Set Name:	RIF_Basil MeOH IC50 11_9_17	Proc. Chnl. Descr.:	W2996 PDA 254.0 nm at 1.2

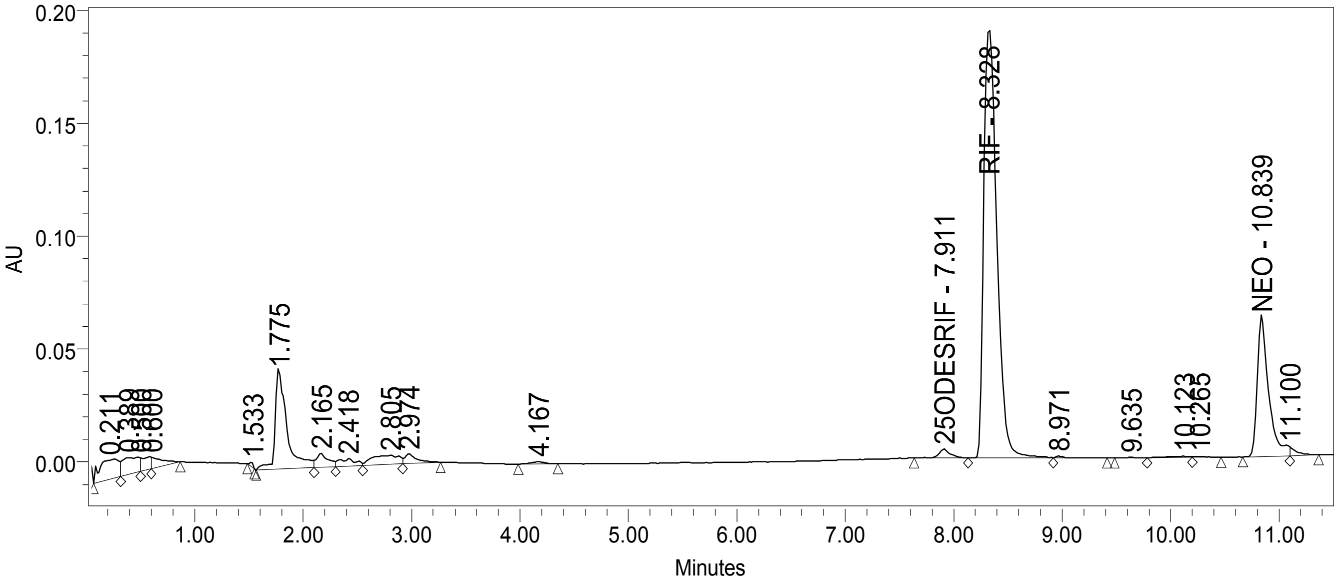

	Peak Name	RT	Area	% Area	Height
1		0.211	107360	3.69	8446
2		0.389	76212	2.62	7544
3		0.500	33463	1.15	6075
4		0.600	35086	1.21	5121
5		1.533	4572	0.16	2190
6		1.775	328731	11.31	44494
7		2.165	46467	1.60	6011
8		2.418	33449	1.15	3101
9		2.805	69934	2.41	3872
10		2.974	32547	1.12	4169
11		4.167	8754	0.30	1123
12	25ODESRIF	7.911	32491	1.12	4011

Reported by User: Saneesh (Saneesh) Project Name: HDI_Stellenbosch_2016

	Peak Name	RT	Area	% Area	Height
13	RIF	8.328	1602181	55.12	190221
14		8.971	8607	0.30	863
15		9.635	2421	0.08	291
16		10.123	8466	0.29	518
17		10.265	3194	0.11	356
18	NEO	10.839	455439	15.67	62520
19		11.100	17554	0.60	4019

Reported by User: Saneesh (Saneesh)

Project Name: HDI_Stellenbosch_2016

SAMPLE INFORMATION

Sample Name:	RIF Inc Basil_MeOH 10ug/mL 2	Acquired By:	Saneesh
Sample Type:	Unknown	Date Acquired:	2017/09/11 09:05:38 PM
Vial:	6	Acq. Method Set:	HDI_RIF_25ODESRIF_NEO
Injection #:	1	Date Processed:	2017/09/12 11:47:02 AM
Injection Volume:	15.00 ul	Processing Method:	HDI_RIF_ W2996 254.0nm-1.2
Run Time:	11.5 Minutes	Channel Name:	W2996 254.0nm-1.2
Sample Set Name:	RIF_Basil MeOH IC50 11_9_17	Proc. Chnl. Descr.:	W2996 PDA 254.0 nm at 1.2

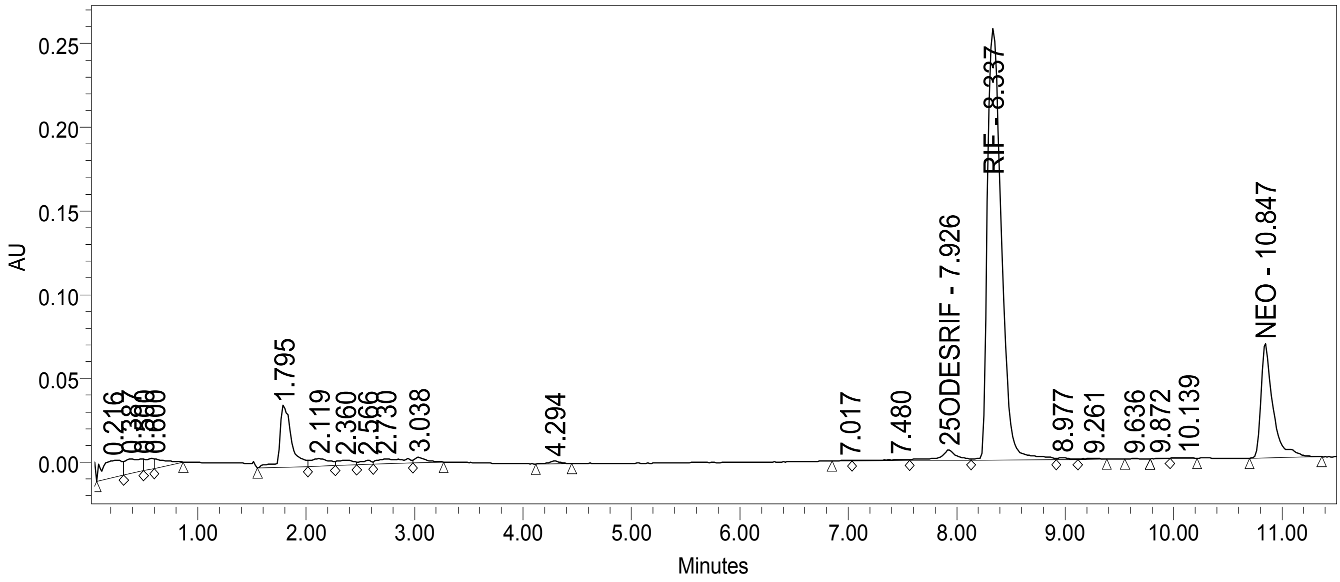

	Peak Name	RT	Area	% Area	Height
1		0.216	130569	3.54	9999
2		0.387	88640	2.40	8766
3		0.500	38418	1.04	7021
4		0.600	41150	1.11	5789
5		1.795	282259	7.64	37940
6		2.119	54658	1.48	4452
7		2.360	31163	0.84	3047
8		2.566	17373	0.47	2538
9		2.730	53677	1.45	2831
10		3.038	24478	0.66	3261
11		4.294	10429	0.28	1472
12		7.017	1729	0.05	263

	Peak Name	RT	Area	% Area	Height
13		7.480	13152	0.36	670
14	25ODESRIF	7.926	63909	1.73	5990
15	RIF	8.337	2294968	62.14	257835
16		8.977	7849	0.21	1085
17		9.261	4536	0.12	373
18		9.636	1410	0.04	247
19		9.872	1402	0.04	204
20		10.139	3618	0.10	305
21	NEO	10.847	527616	14.29	67956

Reported by User: Saneesh (Saneesh)   Project Name: HDI_Stellenbosch_2016

SAMPLE INFORMATION

Sample Name:	RIF Inc Basil_MeOH 10ug/mL 3	Acquired By:	Saneesh
Sample Type:	Unknown	Date Acquired:	2017/09/11 09:20:20 PM
Vial:	7	Acq. Method Set:	HDI_RIF_25ODESRIF_NEO
Injection #:	1	Date Processed:	2017/09/12 11:47:02 AM
Injection Volume:	15.00 ul	Processing Method:	HDI_RIF_ W2996 254.0nm-1.2
Run Time:	11.5 Minutes	Channel Name:	W2996 254.0nm-1.2
Sample Set Name:	RIF_Basil MeOH IC50 11_9_17	Proc. Chnl. Descr.:	W2996 PDA 254.0 nm at 1.2

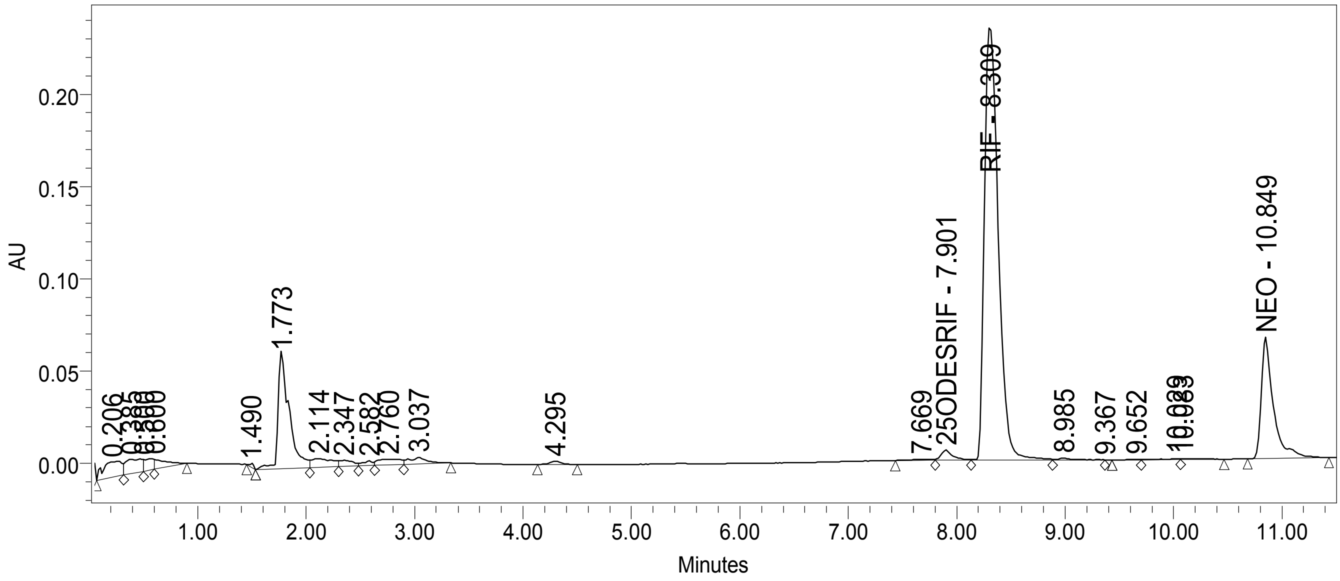

	Peak Name	RT	Area	% Area	Height
1		0.206	102635	2.84	8408
2		0.385	78306	2.16	7730
3		0.500	36090	1.00	6342
4		0.600	41392	1.14	5385
5		1.490	5842	0.16	1969
6		1.773	450761	12.45	63383
7		2.114	64629	1.79	4803
8		2.347	29475	0.81	3327
9		2.582	16953	0.47	2457
10		2.760	45113	1.25	3096
11		3.037	41043	1.13	3517
12		4.295	13861	0.38	1864

Reported by User: Saneesh (Saneesh) Project Name: HDI_Stellenbosch_2016

	Peak Name	RT	Area	% Area	Height
13		7.669	9634	0.27	709
14	25ODESRIF	7.901	43786	1.21	5548
15	RIF	8.309	2097789	57.96	236539
16		8.985	12716	0.35	1133
17		9.367	274	0.01	147
18		9.652	2872	0.08	377
19		10.029	7307	0.20	583
20		10.083	8679	0.24	635
21	NEO	10.849	510520	14.10	65426

SAMPLE INFORMATION

Sample Name:	RIF Inc Basil_MeOH 50ug/mL 1	Acquired By:	Saneesh
Sample Type:	Unknown	Date Acquired:	2017/09/11 09:35:03 PM
Vial:	8	Acq. Method Set:	HDI_RIF_25ODESRIF_NEO
Injection #:	1	Date Processed:	2017/09/12 11:47:02 AM
Injection Volume:	15.00 ul	Processing Method:	HDI_RIF_ W2996 254.0nm-1.2
Run Time:	11.5 Minutes	Channel Name:	W2996 254.0nm-1.2
Sample Set Name:	RIF_Basil MeOH IC50 11_9_17	Proc. Chnl. Descr.:	W2996 PDA 254.0 nm at 1.2

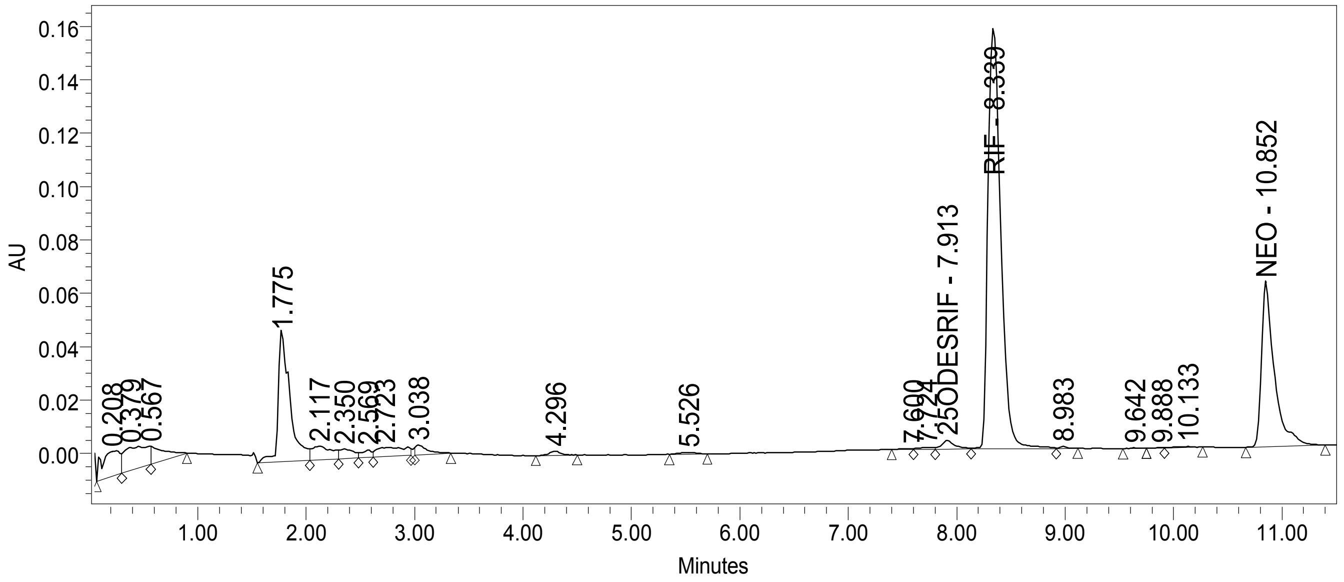

	Peak Name	RT	Area	% Area	Height
1		0.208	109882	4.03	9290
2		0.379	124282	4.56	8627
3		0.567	57528	2.11	6749
4		1.775	366063	13.42	49345
5		2.117	65088	2.39	4993
6		2.350	31851	1.17	3515
7		2.569	17313	0.63	2698
8		2.723	60789	2.23	3234
9		3.038	27971	1.03	3635
10		4.296	10884	0.40	1512
11		5.526	6816	0.25	620
12		7.600	1725	0.06	270

Reported by User: Saneesh (Saneesh) Project Name: HDI_Stellenbosch_2016

	Peak Name	RT	Area	% Area	Height
13		7.724	5890	0.22	586
14	25ODESRIF	7.913	27851	1.02	3303
15	RIF	8.339	1292180	47.38	158062
16		8.983	3669	0.13	725
17		9.642	1546	0.06	229
18		9.888	1168	0.04	188
19		10.133	4560	0.17	356
20	NEO	10.852	510177	18.71	61803

Reported by User: Saneesh (Saneesh)   Project Name: HDI_Stellenbosch_2016

SAMPLE INFORMATION

Sample Name:	RIF Inc Basil_MeOH 50ug/mL 2	Acquired By:	Saneesh
Sample Type:	Unknown	Date Acquired:	2017/09/11 09:49:48 PM
Vial:	9	Acq. Method Set:	HDI_RIF_25ODESRIF_NEO
Injection #:	1	Date Processed:	2017/09/12 11:47:02 AM
Injection Volume:	15.00 ul	Processing Method:	HDI_RIF_ W2996 254.0nm-1.2
Run Time:	11.5 Minutes	Channel Name:	W2996 254.0nm-1.2
Sample Set Name:	RIF_Basil MeOH IC50 11_9_17	Proc. Chnl. Descr.:	W2996 PDA 254.0 nm at 1.2

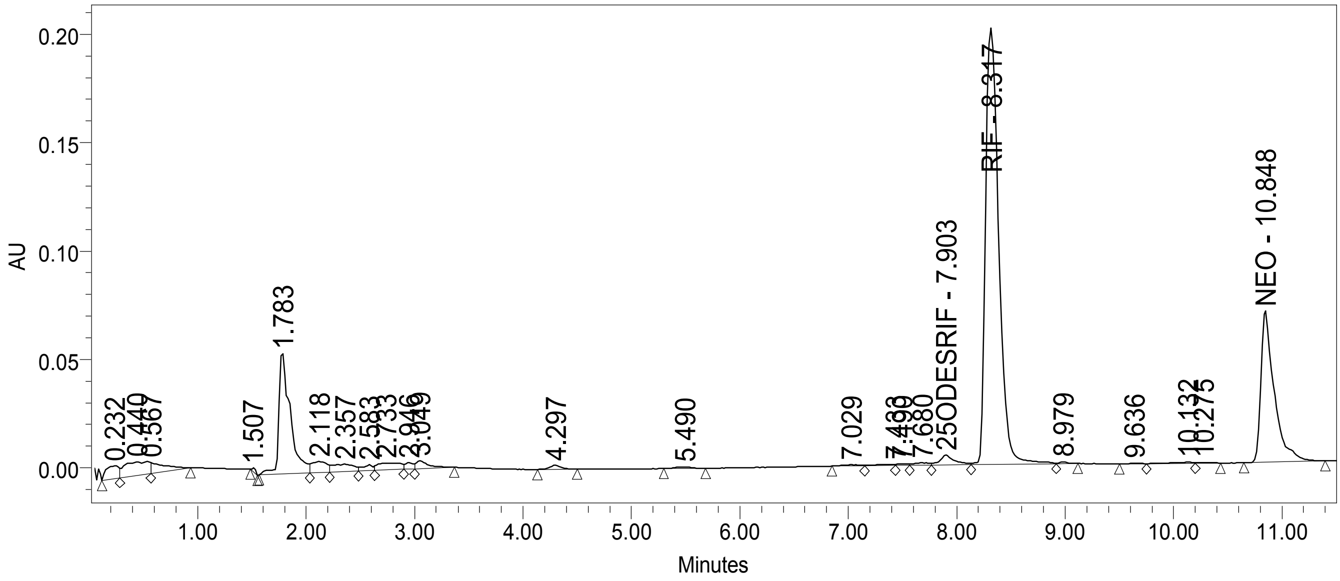

	Peak Name	RT	Area	% Area	Height
1		0.232	47968	1.49	5769
2		0.440	95495	2.97	6182
3		0.567	44294	1.38	5030
4		1.507	4731	0.15	1698
5		1.783	409835	12.75	55338
6		2.118	49593	1.54	5104
7		2.357	47374	1.47	3380
8		2.583	18259	0.57	2562
9		2.733	46329	1.44	3204
10		2.946	15383	0.48	2878
11		3.049	29034	0.90	3532
12		4.297	13203	0.41	1797

Reported by User: Saneesh (Saneesh) Project Name: HDI_Stellenbosch_2016

	Peak Name	RT	Area	% Area	Height
13		5.490	7888	0.25	746
14		7.029	4699	0.15	419
15		7.433	5910	0.18	458
16		7.490	4366	0.14	605
17		7.680	10442	0.32	1011
18	25ODESRIF	7.903	41261	1.28	4464
19	RIF	8.317	1699817	52.87	201165
20		8.979	4209	0.13	787
21		9.636	3290	0.10	385
22		10.132	10283	0.32	602
23		10.275	4270	0.13	399
24	NEO	10.848	597116	18.57	69748

SAMPLE INFORMATION

Sample Name:	RIF Inc Basil_MeOH 50ug/mL 3	Acquired By:	Saneesh
Sample Type:	Unknown	Date Acquired:	2017/09/11 10:04:30 PM
Vial:	10	Acq. Method Set:	HDI_RIF_25ODESRIF_NEO
Injection #:	1	Date Processed:	2017/09/12 11:47:02 AM
Injection Volume:	15.00 ul	Processing Method:	HDI_RIF_ W2996 254.0nm-1.2
Run Time:	11.5 Minutes	Channel Name:	W2996 254.0nm-1.2
Sample Set Name:	RIF_Basil MeOH IC50 11_9_17	Proc. Chnl. Descr.:	W2996 PDA 254.0 nm at 1.2

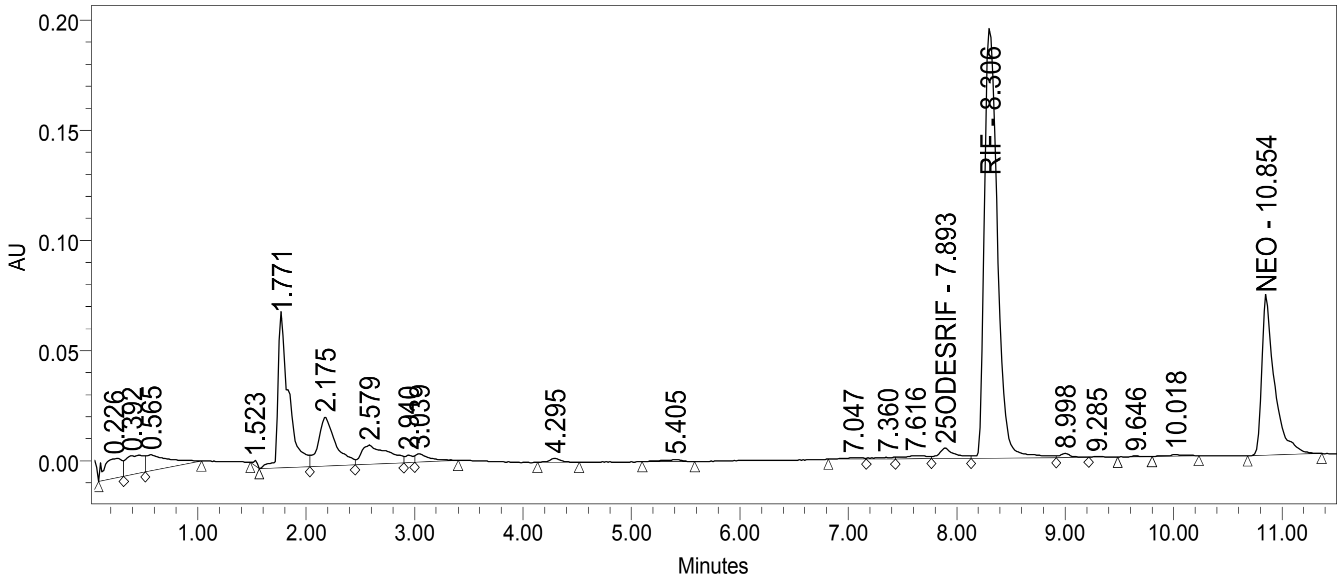

	Peak Name	RT	Area	% Area	Height
1		0.226	102243	2.84	8659
2		0.392	95750	2.66	8478
3		0.565	108419	3.01	7341
4		1.523	6130	0.17	2097
5		1.771	480154	13.35	69532
6		2.175	245272	6.82	22140
7		2.579	145610	4.05	8469
8		2.940	17507	0.49	3255
9		3.039	33618	0.93	3804
10		4.295	13888	0.39	1901
11		5.405	11646	0.32	1018
12		7.047	7159	0.20	559

	Peak Name	RT	Area	% Area	Height
13		7.360	8143	0.23	618
14		7.616	19624	0.55	1196
15	25ODESRIF	7.893	46957	1.31	4679
16	RIF	8.306	1635336	45.48	195730
17		8.998	12827	0.36	1807
18		9.285	2543	0.07	312
19		9.646	2384	0.07	353
20		10.018	7582	0.21	745
21	NEO	10.854	593254	16.50	72506

Reported by User: Saneesh (Saneesh) Project Name: HDI_Stellenbosch_2016

SAMPLE INFORMATION

Sample Name:	RIF Inc Basil_MeOH 100ug/mL 1	Acquired By:	Saneesh
Sample Type:	Unknown	Date Acquired:	2017/09/11 10:19:14 PM
Vial:	11	Acq. Method Set:	HDI_RIF_25ODESRIF_NEO
Injection #:	1	Date Processed:	2017/09/12 11:47:02 AM
Injection Volume:	15.00 ul	Processing Method:	HDI_RIF_ W2996 254.0nm-1.2
Run Time:	11.5 Minutes	Channel Name:	W2996 254.0nm-1.2
Sample Set Name:	RIF_Basil MeOH IC50 11_9_17	Proc. Chnl. Descr.:	W2996 PDA 254.0 nm at 1.2

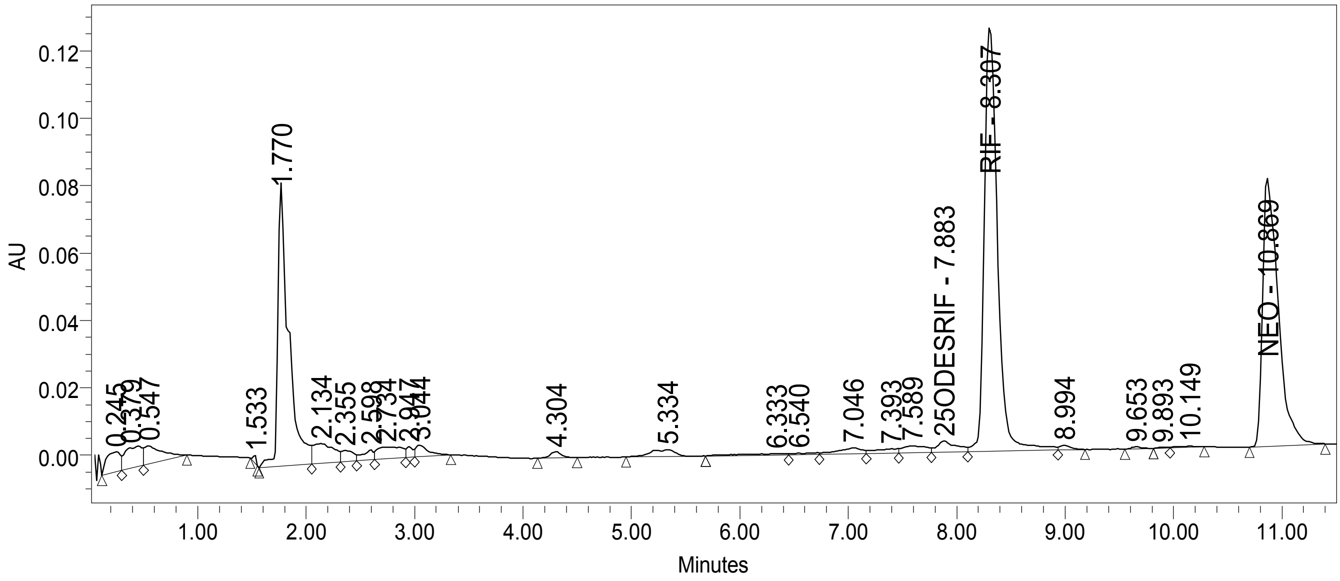

	Peak Name	RT	Area	% Area	Height
1		0.245	53501	1.75	5951
2		0.379	67243	2.21	6097
3		0.547	62745	2.06	5367
4		1.533	4695	0.15	2495
5		1.770	571364	18.74	82285
6		2.134	78226	2.57	5838
7		2.355	25814	0.85	3356
8		2.598	21835	0.72	2805
9		2.734	55337	1.82	3588
10		2.947	13897	0.46	3164
11		3.044	28181	0.92	3524
12		4.304	14204	0.47	1876

Reported by User: Saneesh (Saneesh) Project Name: HDI_Stellenbosch_2016

	Peak Name	RT	Area	% Area	Height
13		5.334	34347	1.13	2087
14		6.333	16119	0.53	454
15		6.540	6609	0.22	457
16		7.046	25632	0.84	1749
17		7.393	18813	0.62	1260
18		7.589	29408	0.96	1963
19	25ODESRIF	7.883	39731	1.30	3256
20	RIF	8.307	1064268	34.91	126226
21		8.994	9572	0.31	1377
22		9.653	3581	0.12	569
23		9.893	1630	0.05	253
24		10.149	4558	0.15	386
25	NEO	10.869	797239	26.15	79354

SAMPLE INFORMATION

Sample Name:	RIF Inc Basil_MeOH 100ug/mL 2	Acquired By:	Saneesh
Sample Type:	Unknown	Date Acquired:	2017/09/11 10:33:57 PM
Vial:	12	Acq. Method Set:	HDI_RIF_25ODESRIF_NEO
Injection #:	1	Date Processed:	2017/09/12 11:47:02 AM
Injection Volume:	15.00 ul	Processing Method:	HDI_RIF_ W2996 254.0nm-1.2
Run Time:	11.5 Minutes	Channel Name:	W2996 254.0nm-1.2
Sample Set Name:	RIF_Basil MeOH IC50 11_9_17	Proc. Chnl. Descr.:	W2996 PDA 254.0 nm at 1.2

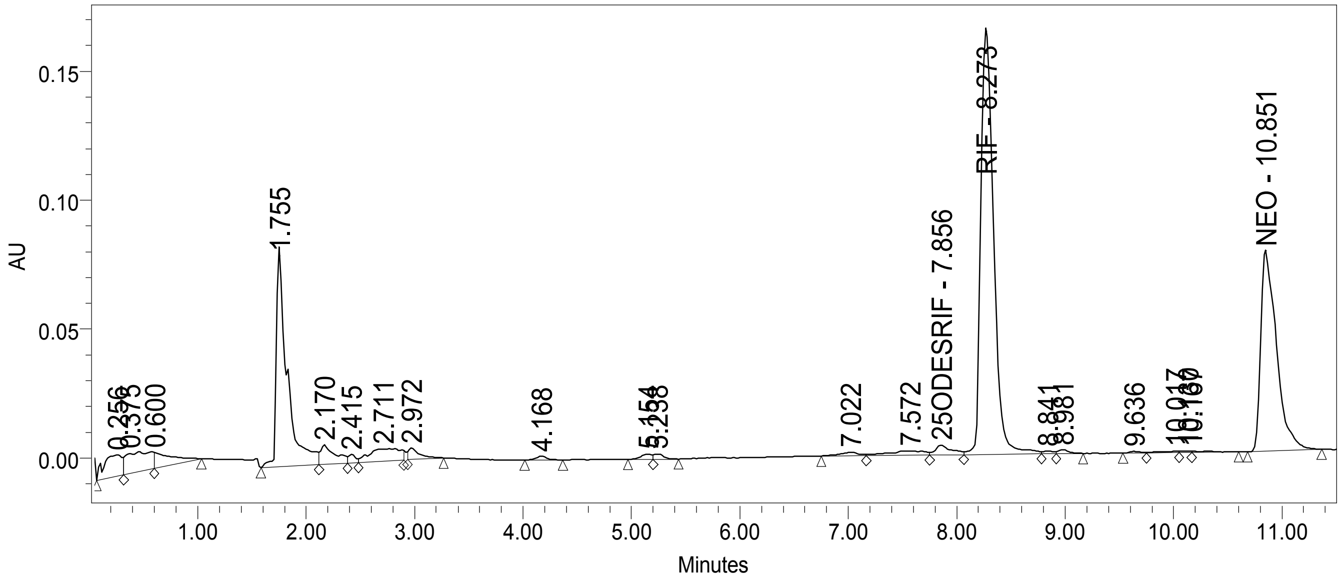

	Peak Name	RT	Area	% Area	Height
1		0.256	98409	2.90	8026
2		0.375	120175	3.54	7761
3		0.600	67185	1.98	6149
4		1.755	578868	17.06	83329
5		2.170	71938	2.12	7490
6		2.415	14692	0.43	3265
7		2.711	95736	2.82	4698
8		2.972	32096	0.95	4364
9		4.168	11160	0.33	1470
10		5.154	14649	0.43	1994
11		5.238	12618	0.37	1986
12		7.022	17962	0.53	1441

Reported by User: Saneesh (Saneesh) Project Name: HDI_Stellenbosch_2016

	Peak Name	RT	Area	% Area	Height
13		7.572	38787	1.14	1637
14	25ODESRIF	7.856	39112	1.15	3732
15	RIF	8.273	1360166	40.07	166109
16		8.841	6976	0.21	1002
17		8.981	9553	0.28	1407
18		9.636	3834	0.11	568
19		10.017	5667	0.17	482
20		10.130	3493	0.10	579
21		10.167	4966	0.15	451
22	NEO	10.851	786061	23.16	77921

SAMPLE INFORMATION

Sample Name:	RIF Inc Basil_MeOH 100ug/mL 3	Acquired By:	Saneesh
Sample Type:	Unknown	Date Acquired:	2017/09/11 10:48:39 PM
Vial:	13	Acq. Method Set:	HDI_RIF_25ODESRIF_NEO
Injection #:	1	Date Processed:	2017/09/12 11:47:02 AM
Injection Volume:	15.00 ul	Processing Method:	HDI_RIF_ W2996 254.0nm-1.2
Run Time:	11.5 Minutes	Channel Name:	W2996 254.0nm-1.2
Sample Set Name:	RIF_Basil MeOH IC50 11_9_17	Proc. Chnl. Descr.:	W2996 PDA 254.0 nm at 1.2

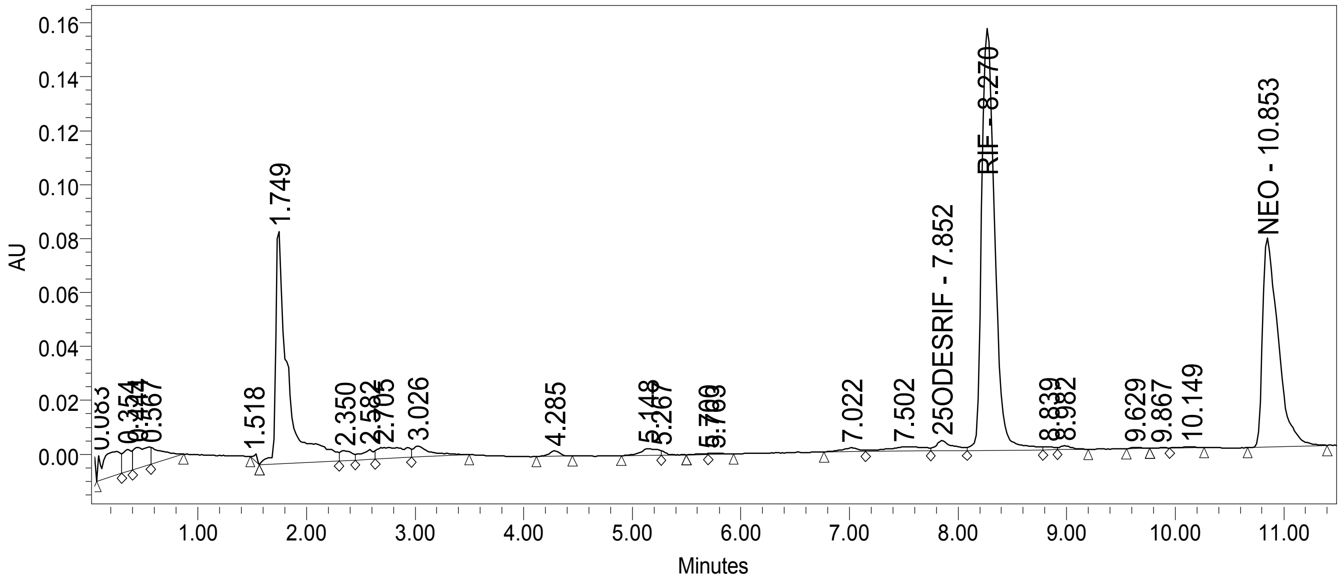

	Peak Name	RT	Area	% Area	Height
1		0.083	106226	3.14	9290
2		0.354	45935	1.36	8197
3		0.444	70189	2.07	7846
4		0.567	48660	1.44	6201
5		1.518	5319	0.16	1779
6		1.749	674313	19.91	85221
7		2.350	27699	0.82	3542
8		2.582	28984	0.86	3338
9		2.705	73776	2.18	4065
10		3.026	46965	1.39	4182
11		4.285	13772	0.41	1952
12		5.148	27649	0.82	2465

Reported by User: Saneesh (Saneesh) Project Name: HDI_Stellenbosch_2016

	Peak Name	RT	Area	% Area	Height
13		5.267	4829	0.14	1688
14		5.700	2029	0.06	340
15		5.763	3408	0.10	424
16		7.022	15328	0.45	1365
17		7.502	38684	1.14	1505
18	25ODESRIF	7.852	40495	1.20	3814
19	RIF	8.270	1318464	38.94	156545
20		8.839	7799	0.23	1089
21		8.982	10643	0.31	1434
22		9.629	3206	0.09	555
23		9.867	1181	0.03	181
24		10.149	3603	0.11	290
25	NEO	10.853	766884	22.65	77542

SAMPLE INFORMATION

Sample Name:	RIF Inc Basil_MeOH 150ug/mL 1	Acquired By:	Saneesh
Sample Type:	Unknown	Date Acquired:	2017/09/11 11:03:24 PM
Vial:	14	Acq. Method Set:	HDI_RIF_25ODESRIF_NEO
Injection #:	1	Date Processed:	2017/09/12 11:47:03 AM
Injection Volume:	15.00 ul	Processing Method:	HDI_RIF_ W2996 254.0nm-1.2
Run Time:	11.5 Minutes	Channel Name:	W2996 254.0nm-1.2
Sample Set Name:	RIF_Basil MeOH IC50 11_9_17	Proc. Chnl. Descr.:	W2996 PDA 254.0 nm at 1.2

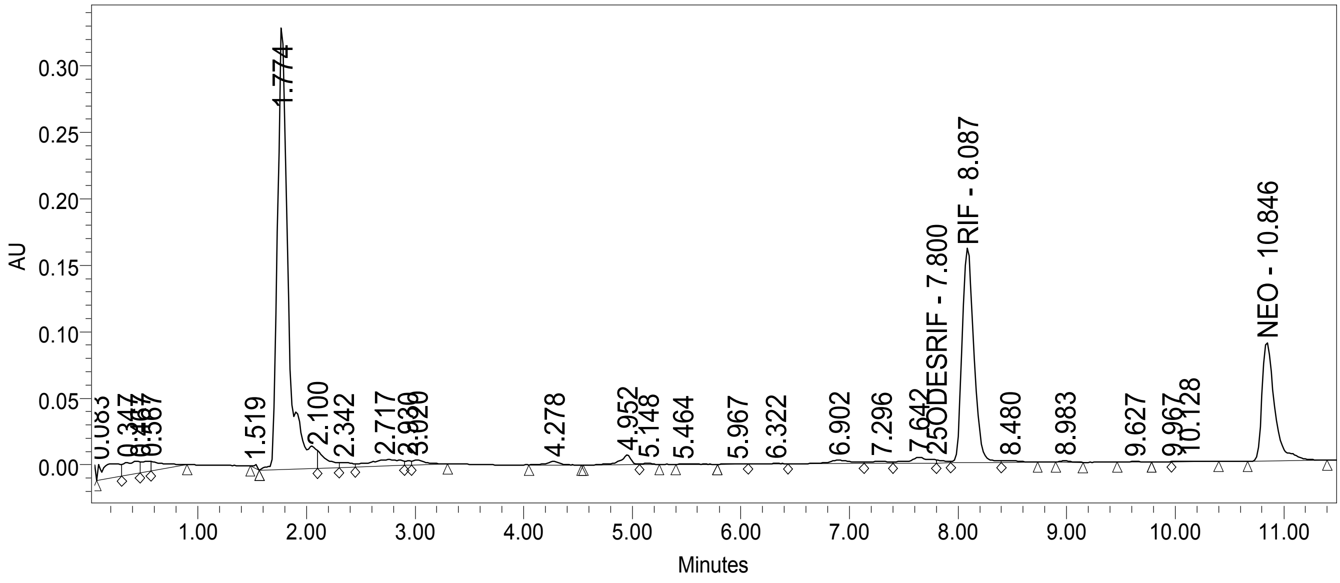

	Peak Name	RT	Area	% Area	Height
1		0.083	126450	2.51	11530
2		0.347	89516	1.77	9487
3		0.467	46233	0.92	8236
4		0.567	63123	1.25	7188
5		1.519	5662	0.11	1888
6		1.774	2269309	44.98	321044
7		2.100	80536	1.60	13368
8		2.342	29042	0.58	3647
9		2.717	95909	1.90	4573
10		2.930	12362	0.25	3179
11		3.020	30583	0.61	3698
12		4.278	22863	0.45	2806

Reported by User: Saneesh (Saneesh)   Project Name: HDI_Stellenbosch_2016

	Peak Name	RT	Area	% Area	Height
13		4.952	54894	1.09	7218
14		5.148	7285	0.14	1245
15		5.464	3468	0.07	322
16		5.967	2019	0.04	179
17		6.322	5879	0.12	480
18		6.902	41549	0.82	2510
19		7.296	19952	0.40	1602
20		7.642	57252	1.13	4385
21	25ODESRIF	7.800	11967	0.24	2211
22	RIF	8.087	1214355	24.07	161478
23		8.480	14932	0.30	1400
24		8.983	6486	0.13	1130
25		9.627	4271	0.08	472
26		9.967	1831	0.04	257
27		10.128	7572	0.15	470
28	NEO	10.846	720242	14.27	88779

SAMPLE INFORMATION

Sample Name:	RIF Inc Basil_MeOH 150ug/mL 2	Acquired By:	Saneesh
Sample Type:	Unknown	Date Acquired:	2017/09/11 11:18:07 PM
Vial:	15	Acq. Method Set:	HDI_RIF_25ODESRIF_NEO
Injection #:	1	Date Processed:	2017/09/12 11:47:03 AM
Injection Volume:	15.00 ul	Processing Method:	HDI_RIF_ W2996 254.0nm-1.2
Run Time:	11.5 Minutes	Channel Name:	W2996 254.0nm-1.2
Sample Set Name:	RIF_Basil MeOH IC50 11_9_17	Proc. Chnl. Descr.:	W2996 PDA 254.0 nm at 1.2

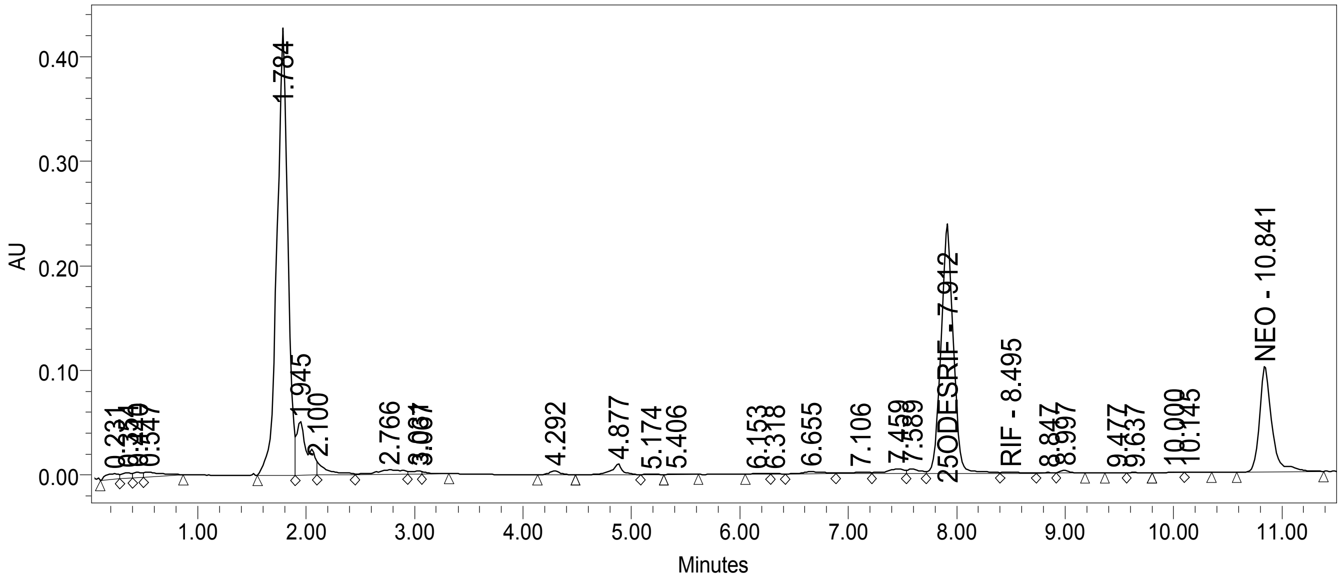

	Peak Name	RT	Area	% Area	Height
1		0.231	43953	0.66	5193
2		0.354	34191	0.52	5421
3		0.440	29483	0.45	5360
4		0.547	50685	0.77	4699
5		1.784	2946170	44.49	410769
6		1.945	366995	5.54	50389
7		2.100	78446	1.18	13030
8		2.766	73491	1.11	4160
9		3.031	21511	0.32	3118
10		3.067	9482	0.14	2346
11		4.292	27421	0.41	4005
12		4.877	75768	1.14	10145

	Peak Name	RT	Area	% Area	Height
13		5.174	3468	0.05	442
14		5.406	5726	0.09	611
15		6.153	2982	0.05	325
16		6.318	2257	0.03	467
17		6.655	31870	0.48	2185
18		7.106	24273	0.37	1586
19		7.459	50832	0.77	4458
20		7.589	35039	0.53	4435
21	25ODESRIF	7.912	1871851	28.26	236050
22	RIF	8.495	13488	0.20	1122
23		8.847	4601	0.07	633
24		8.997	15271	0.23	2624
25		9.477	1876	0.03	281
26		9.637	2091	0.03	354
27		10.000	4078	0.06	449
28		10.145	4698	0.07	503
29	NEO	10.841	790626	11.94	101403

Reported by User: Saneesh (Saneesh)   Project Name: HDI_Stellenbosch_2016

SAMPLE INFORMATION

Sample Name:	RIF Inc Basil_MeOH 150ug/mL 3	Acquired By:	Saneesh
Sample Type:	Unknown	Date Acquired:	2017/09/11 11:32:50 PM
Vial:	16	Acq. Method Set:	HDI_RIF_25ODESRIF_NEO
Injection #:	1	Date Processed:	2017/09/12 11:47:03 AM
Injection Volume:	15.00 ul	Processing Method:	HDI_RIF_ W2996 254.0nm-1.2
Run Time:	11.5 Minutes	Channel Name:	W2996 254.0nm-1.2
Sample Set Name:	RIF_Basil MeOH IC50 11_9_17	Proc. Chnl. Descr.:	W2996 PDA 254.0 nm at 1.2

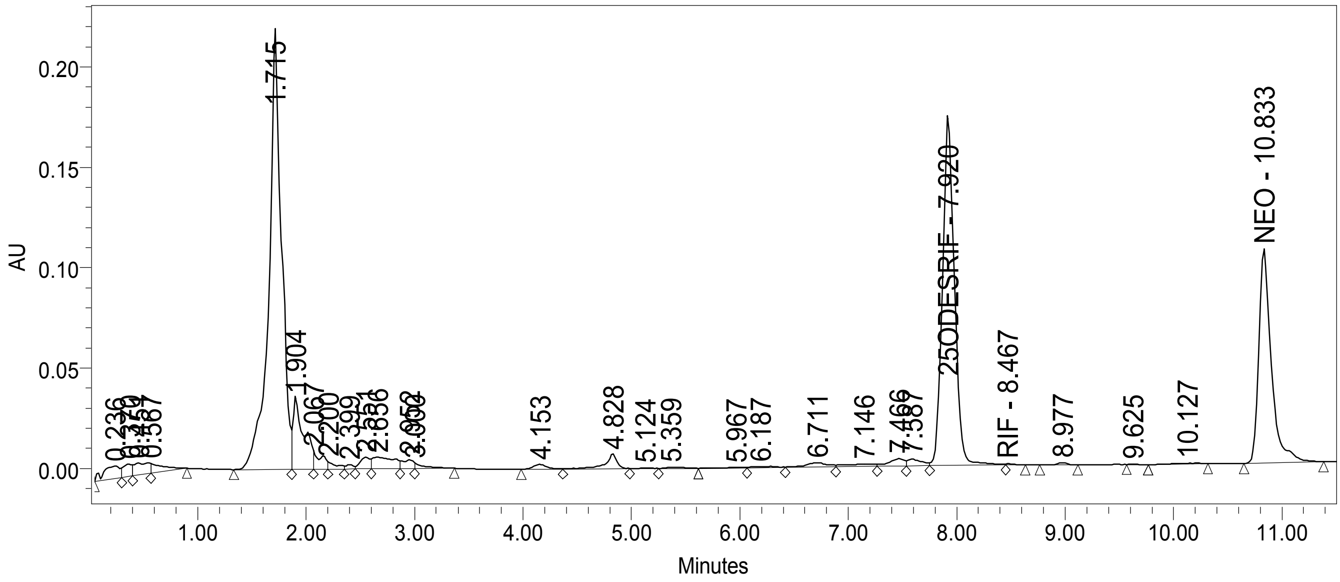

	Peak Name	RT	Area	% Area	Height
1		0.236	66144	1.35	6024
2		0.370	33923	0.69	6116
3		0.451	54279	1.11	5894
4		0.567	40321	0.83	4981
5		1.715	1777951	36.40	214539
6		1.904	249217	5.10	34364
7		2.067	46503	0.95	8947
8		2.200	19004	0.39	3734
9		2.399	9628	0.20	2098
10		2.551	36879	0.76	5721
11		2.656	79503	1.63	5622
12		2.952	30328	0.62	4385

Reported by User: Saneesh (Saneesh) Project Name: HDI_Stellenbosch_2016

	Peak Name	RT	Area	% Area	Height
13		3.000	16144	0.33	2831
14		4.153	19850	0.41	2361
15		4.828	57445	1.18	7262
16		5.124	3708	0.08	388
17		5.359	7726	0.16	700
18		5.967	4289	0.09	304
19		6.187	9158	0.19	593
20		6.711	30051	0.62	2164
21		7.146	23747	0.49	1296
22		7.466	37479	0.77	3796
23		7.587	32136	0.66	3476
24	25ODESRIF	7.920	1326784	27.16	172613
25	RIF	8.467	3115	0.06	654
26		8.977	8835	0.18	1196
27		9.625	1465	0.03	282
28		10.127	7843	0.16	406
29	NEO	10.833	850920	17.42	106238

SAMPLE INFORMATION

Sample Name:	RIF Inc Basil_MeOH 200ug/mL 1	Acquired By:	Saneesh
Sample Type:	Unknown	Date Acquired:	2017/09/11 11:47:34 PM
Vial:	17	Acq. Method Set:	HDI_RIF_25ODESRIF_NEO
Injection #:	1	Date Processed:	2017/09/12 11:47:03 AM
Injection Volume:	15.00 ul	Processing Method:	HDI_RIF_ W2996 254.0nm-1.2
Run Time:	11.5 Minutes	Channel Name:	W2996 254.0nm-1.2
Sample Set Name:	RIF_Basil MeOH IC50 11_9_17	Proc. Chnl. Descr.:	W2996 PDA 254.0 nm at 1.2

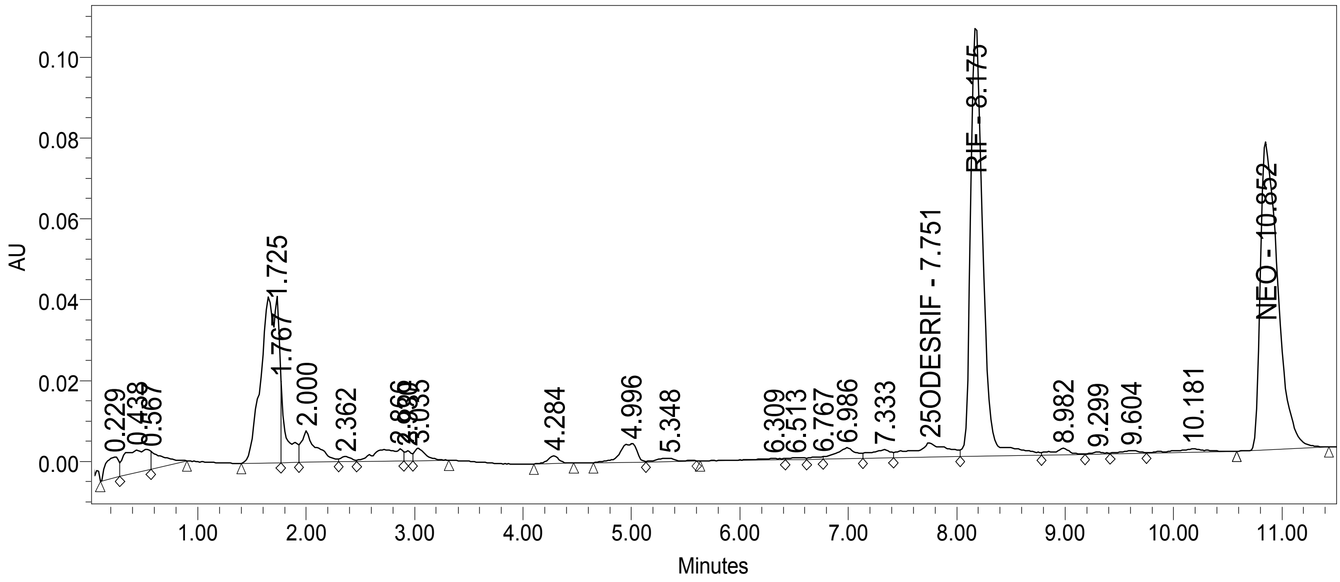

	Peak Name	RT	Area	% Area	Height
1		0.229	42312	1.49	5167
2		0.438	86102	3.04	5523
3		0.567	35749	1.26	4408
4		1.725	457605	16.14	39975
5		1.767	66961	2.36	20039
6		2.000	75733	2.67	7447
7		2.362	8935	0.32	1293
8		2.866	52128	1.84	2917
9		2.930	11056	0.39	2455
10		3.035	22908	0.81	3066
11		4.284	13721	0.48	1884
12		4.996	50373	1.78	4545

	Peak Name	RT	Area	% Area	Height
13		5.348	11717	0.41	854
14		6.309	8236	0.29	403
15		6.513	4356	0.15	435
16		6.767	4068	0.14	545
17		6.986	33921	1.20	2665
18		7.333	27107	0.96	2047
19	25ODESRIF	7.751	76038	2.68	3514
20	RIF	8.175	848431	29.93	107102
21		8.982	16886	0.60	1565
22		9.299	3862	0.14	499
23		9.604	8250	0.29	735
24		10.181	17413	0.61	819
25	NEO	10.852	850953	30.02	76247

Reported by User: Saneesh (Saneesh)   Project Name: HDI_Stellenbosch_2016

SAMPLE INFORMATION

Sample Name:	RIF Inc Basil_MeOH 200ug/mL 2	Acquired By:	Saneesh
Sample Type:	Unknown	Date Acquired:	2017/09/12 12:02:16 AM
Vial:	18	Acq. Method Set:	HDI_RIF_25ODESRIF_NEO
Injection #:	1	Date Processed:	2017/09/12 11:47:03 AM
Injection Volume:	15.00 ul	Processing Method:	HDI_RIF_ W2996 254.0nm-1.2
Run Time:	11.5 Minutes	Channel Name:	W2996 254.0nm-1.2
Sample Set Name:	RIF_Basil MeOH IC50 11_9_17	Proc. Chnl. Descr.:	W2996 PDA 254.0 nm at 1.2

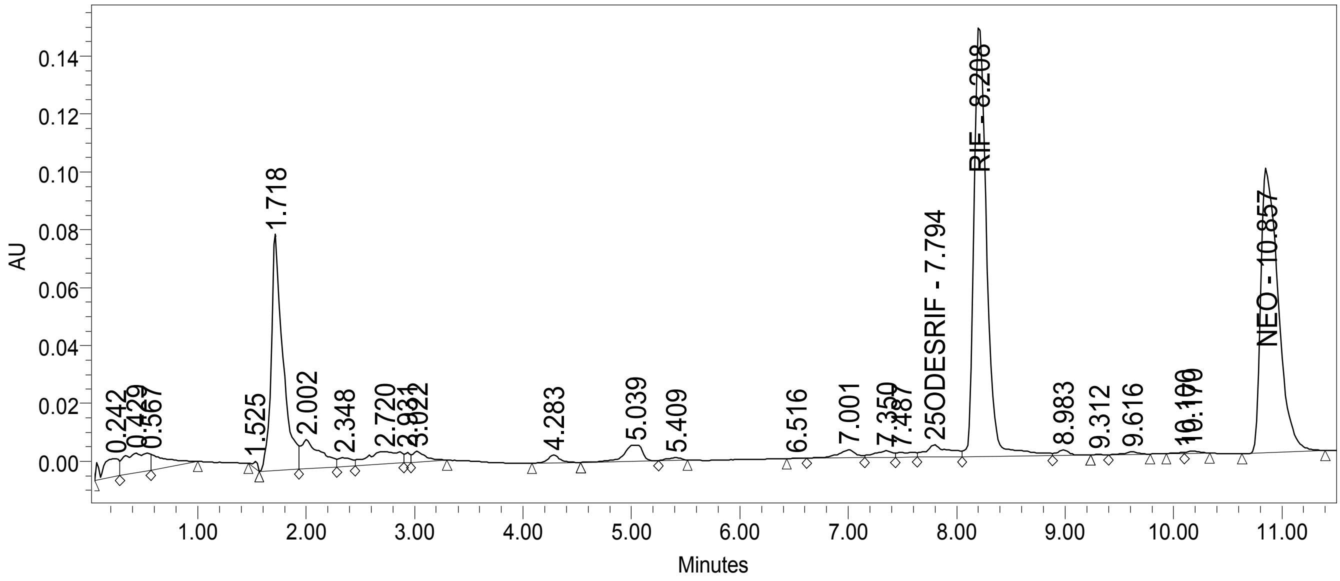

	Peak Name	RT	Area	% Area	Height
1		0.242	67915	1.82	6215
2		0.429	102821	2.76	6732
3		0.567	58375	1.57	5566
4		1.525	7864	0.21	2390
5		1.718	612250	16.43	81489
6		2.002	127744	3.43	9888
7		2.348	26554	0.71	3027
8		2.720	88979	2.39	4218
9		2.931	12484	0.34	3322
10		3.022	30830	0.83	3641
11		4.283	21185	0.57	2621
12		5.039	66023	1.77	5599

Reported by User: Saneesh (Saneesh) Project Name: HDI_Stellenbosch_2016

	Peak Name	RT	Area	% Area	Height
13		5.409	9356	0.25	979
14		6.516	1176	0.03	216
15		7.001	33688	0.90	2890
16		7.350	27778	0.75	2309
17		7.487	19379	0.52	1731
18	25ODESRIF	7.794	62387	1.67	4141
19	RIF	8.208	1223517	32.84	149570
20		8.983	14381	0.39	1879
21		9.312	1521	0.04	284
22		9.616	8296	0.22	866
23		10.100	2202	0.06	389
24		10.170	5443	0.15	727
25	NEO	10.857	1093833	29.36	98780

SAMPLE INFORMATION

Sample Name:	RIF Inc Basil_MeOH 200ug/mL 3	Acquired By:	Saneesh
Sample Type:	Unknown	Date Acquired:	2017/09/12 12:17:00 AM
Vial:	19	Acq. Method Set:	HDI_RIF_25ODESRIF_NEO
Injection #:	1	Date Processed:	2017/09/12 11:47:03 AM
Injection Volume:	15.00 ul	Processing Method:	HDI_RIF_ W2996 254.0nm-1.2
Run Time:	11.5 Minutes	Channel Name:	W2996 254.0nm-1.2
Sample Set Name:	RIF_Basil MeOH IC50 11_9_17	Proc. Chnl. Descr.:	W2996 PDA 254.0 nm at 1.2

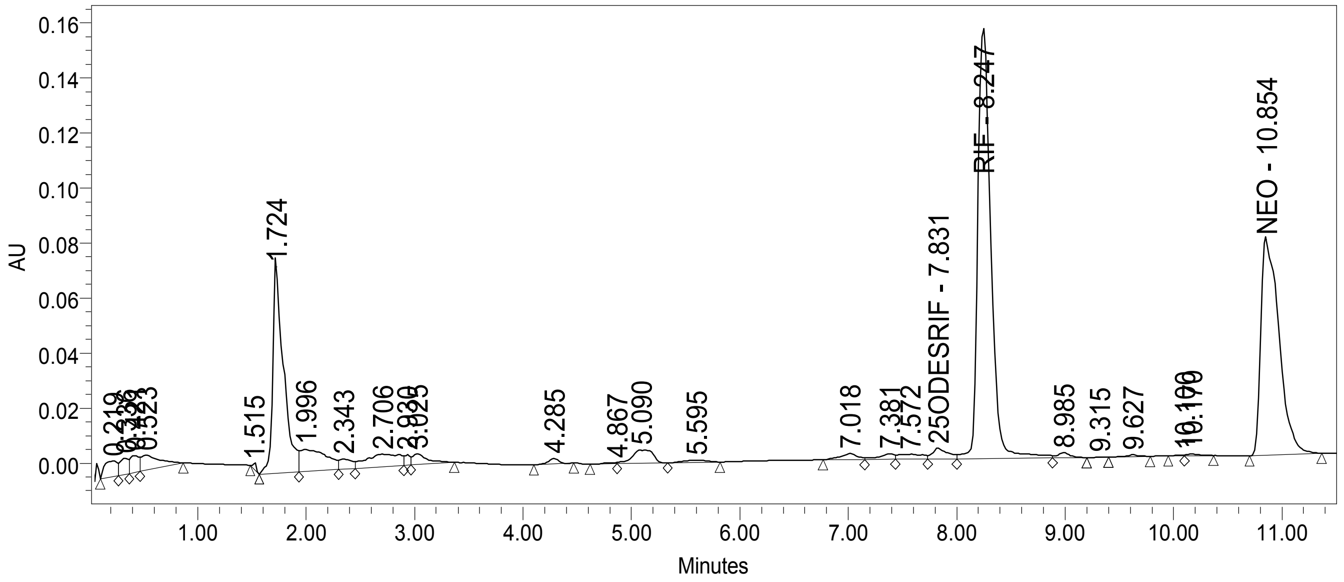

	Peak Name	RT	Area	% Area	Height
1		0.219	46277	1.32	5605
2		0.336	31589	0.90	5734
3		0.421	34038	0.97	6136
4		0.523	64346	1.84	5380
5		1.515	5217	0.15	1710
6		1.724	534859	15.26	76992
7		1.996	134808	3.85	7951
8		2.343	28522	0.81	3652
9		2.706	98657	2.81	4549
10		2.930	14049	0.40	3761
11		3.025	38488	1.10	4027
12		4.285	13864	0.40	2037

Reported by User: Saneesh (Saneesh) Project Name: HDI_Stellenbosch_2016

	Peak Name	RT	Area	% Area	Height
13		4.867	3350	0.10	495
14		5.090	65270	1.86	4884
15		5.595	12653	0.36	841
16		7.018	23973	0.68	2266
17		7.381	19391	0.55	1974
18		7.572	28434	0.81	1770
19	25ODESRIF	7.831	38502	1.10	3837
20	RIF	8.247	1317353	37.58	156383
21		8.985	16192	0.46	1939
22		9.315	1217	0.03	247
23		9.627	6806	0.19	825
24		10.100	1816	0.05	334
25		10.170	5282	0.15	651
26	NEO	10.854	920797	26.27	79590

Multi Sample Summary

Reported by User: Saneesh (Saneesh) Project Name: HDI_Stellenbosch_2016

SAMPLE INFORMATION

Sample Name:	RIF Inc Basil TDI Test Contrl K1	Acquired By:	Saneesh
Sample Type:	Unknown	Date Acquired:	2017/09/12 01:29:43 AM
Vial:	23	Acq. Method Set:	HDI_RIF_25ODESRIF_NEO
Injection #:	1	Date Processed:	2017/09/12 11:49:05 AM
Injection Volume:	15.00 ul	Processing Method:	HDI_RIF_ W2996 254.0nm-1.2
Run Time:	11.5 Minutes	Channel Name:	W2996 254.0nm-1.2
Sample Set Name:	RIF_BasilMeOH TDI IC50 12_9_17	Proc. Chnl. Descr.:	W2996 PDA 254.0 nm at 1.2

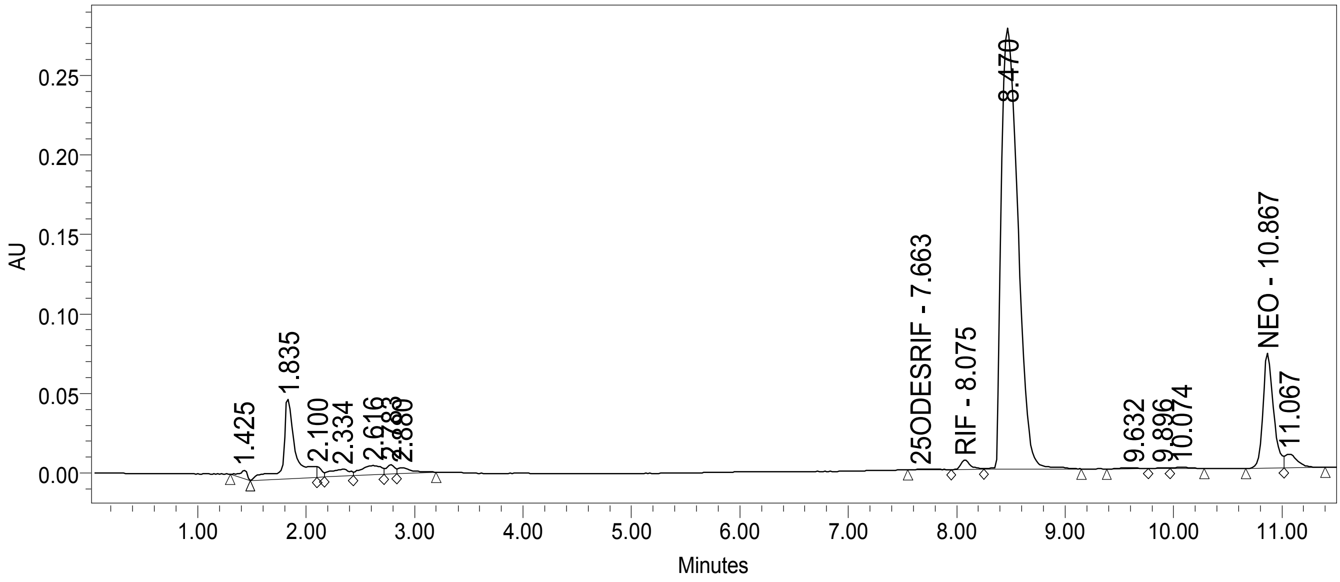

	Peak Name	RT	Area	% Area	Height
1		1.425	24118	0.57	5327
2		1.835	397807	9.47	50729
3		2.100	19583	0.47	6647
4		2.334	56733	1.35	4338
5		2.616	76149	1.81	5765
6		2.783	31786	0.76	5761
7		2.880	32977	0.78	3692
8	25ODESRIF	7.663	4852	0.12	331
9	RIF	8.075	37948	0.90	5910
10		8.470	2936387	69.88	277984
11		9.632	10247	0.24	812
12		9.896	6059	0.14	788
13		10.074	10008	0.24	834
14	NEO	10.867	485602	11.56	71752

Reported by User: Saneesh (Saneesh) Project Name: HDI_Stellenbosch_2016

	Peak Name	RT	Area	% Area	Height
15		11.067	71966	1.71	8627

SAMPLE INFORMATION

Sample Name:	RIF Inc Basil TDI Test Contrl K2	Acquired By:	Saneesh
Sample Type:	Unknown	Date Acquired:	2017/09/12 01:44:37 AM
Vial:	24	Acq. Method Set:	HDI_RIF_25ODESRIF_NEO
Injection #:	1	Date Processed:	2017/09/12 11:49:05 AM
Injection Volume:	15.00 ul	Processing Method:	HDI_RIF_ W2996 254.0nm-1.2
Run Time:	11.5 Minutes	Channel Name:	W2996 254.0nm-1.2
Sample Set Name:	RIF_BasilMeOH TDI IC50 12_9_17	Proc. Chnl. Descr.:	W2996 PDA 254.0 nm at 1.2

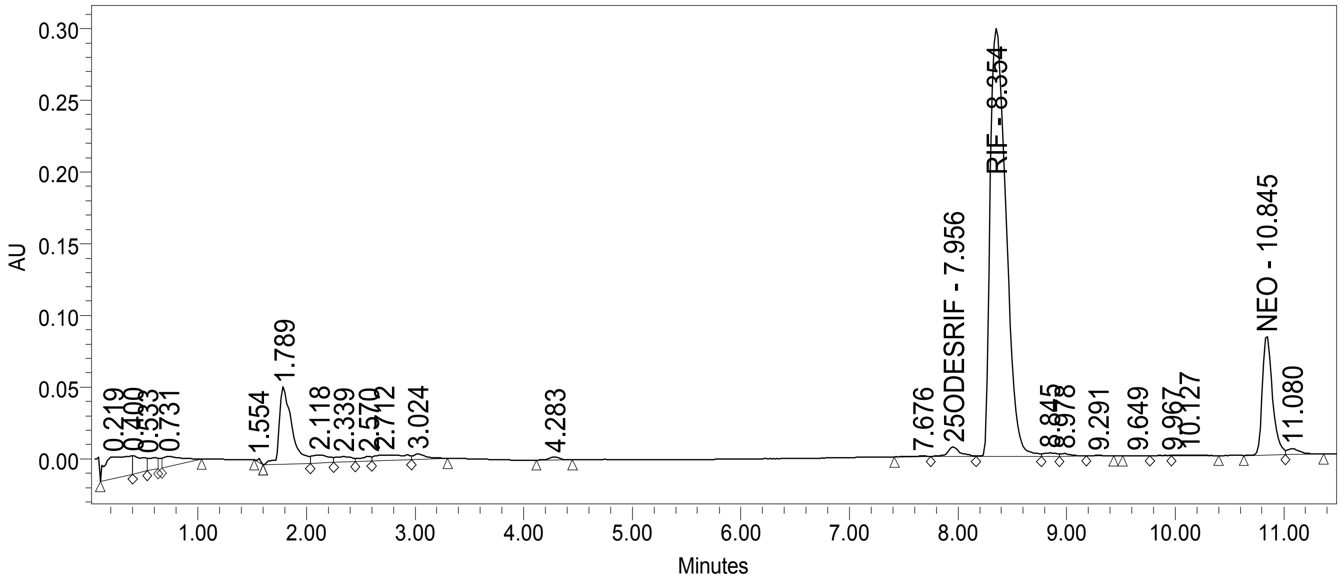

	Peak Name	RT	Area	% Area	Height
1		0.219	230177	4.74	15047
2		0.400	84033	1.73	12542
3		0.533	48696	1.00	8909
4		0.731	82814	1.70	6770
5		1.554	6402	0.13	2169
6		1.789	427174	8.79	53845
7		2.118	60941	1.25	5495
8		2.339	38736	0.80	3699
9		2.570	23146	0.48	3235
10		2.712	75849	1.56	3930
11		3.024	30930	0.64	3731
12		4.283	14416	0.30	1928

Reported by User: Saneesh (Saneesh) Project Name: HDI_Stellenbosch_2016

	Peak Name	RT	Area	% Area	Height
13		7.676	6426	0.13	497
14	25ODESRIF	7.956	57120	1.18	6842
15	RIF	8.354	3050317	62.75	298984
16		8.845	20709	0.43	2398
17		8.978	14860	0.31	1953
18		9.291	5120	0.11	609
19		9.649	2261	0.05	284
20		9.967	2934	0.06	363
21		10.127	8602	0.18	490
22	NEO	10.845	533793	10.98	82534
23		11.080	35260	0.73	4155

SAMPLE INFORMATION

Sample Name:	RIF Inc Basil_MeOH 1ug/mL TDI1	Acquired By:	Saneesh
Sample Type:	Unknown	Date Acquired:	2017/09/12 02:00:18 AM
Vial:	2	Acq. Method Set:	HDI_RIF_25ODESRIF_NEO
Injection #:	1	Date Processed:	2017/09/12 11:49:05 AM
Injection Volume:	15.00 ul	Processing Method:	HDI_RIF_ W2996 254.0nm-1.2
Run Time:	11.5 Minutes	Channel Name:	W2996 254.0nm-1.2
Sample Set Name:	RIF_BasilMeOH TDI IC50 12_9_17	Proc. Chnl. Descr.:	W2996 PDA 254.0 nm at 1.2

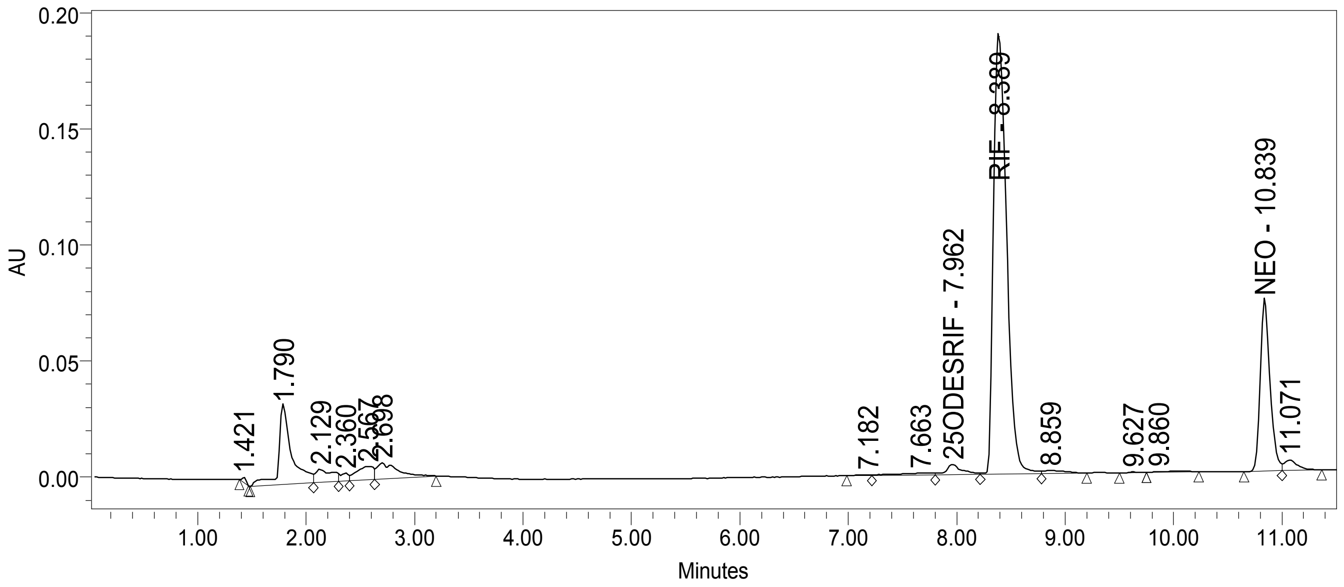

	Peak Name	RT	Area	% Area	Height
1		1.421	5972	0.22	1992
2		1.790	287819	10.62	34693
3		2.129	60723	2.24	5590
4		2.360	17981	0.66	3366
5		2.567	62561	2.31	5592
6		2.698	91208	3.37	6871
7		7.182	3335	0.12	358
8		7.663	19208	0.71	732
9	25ODESRIF	7.962	42480	1.57	4344
10	RIF	8.389	1591303	58.74	190518
11		8.859	15469	0.57	1181
12		9.627	1939	0.07	317

Reported by User: Saneesh (Saneesh) Project Name: HDI_Stellenbosch_2016

	Peak Name	RT	Area	% Area	Height
13		9.860	6313	0.23	313
14	NEO	10.839	463782	17.12	74432
15		11.071	39168	1.45	4520

Reported by User: Saneesh (Saneesh) Project Name: HDI_Stellenbosch_2016

SAMPLE INFORMATION

Sample Name:	RIF Inc Basil_MeOH 1ug/mL TDI2	Acquired By:	Saneesh
Sample Type:	Unknown	Date Acquired:	2017/09/12 02:15:00 AM
Vial:	3	Acq. Method Set:	HDI_RIF_25ODESRIF_NEO
Injection #:	1	Date Processed:	2017/09/12 11:49:05 AM
Injection Volume:	15.00 ul	Processing Method:	HDI_RIF_ W2996 254.0nm-1.2
Run Time:	11.5 Minutes	Channel Name:	W2996 254.0nm-1.2
Sample Set Name:	RIF_BasilMeOH TDI IC50 12_9_17	Proc. Chnl. Descr.:	W2996 PDA 254.0 nm at 1.2

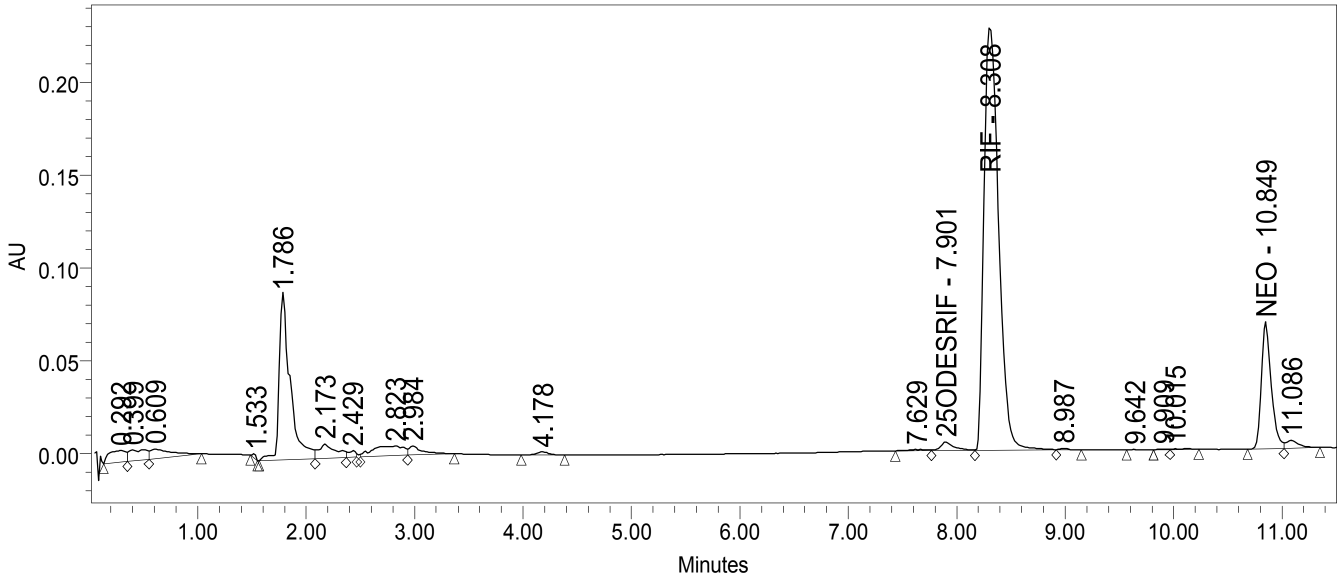

	Peak Name	RT	Area	% Area	Height
1		0.292	68441	1.80	6182
2		0.399	62348	1.64	5822
3		0.609	69668	1.84	5040
4		1.533	5524	0.15	2420
5		1.786	634466	16.72	88869
6		2.173	82882	2.18	7687
7		2.429	17161	0.45	3363
8		2.823	107268	2.83	5208
9		2.984	42931	1.13	4864
10		4.178	14289	0.38	1745
11		7.629	10463	0.28	771
12	25ODESRIF	7.901	41819	1.10	4734

Reported by User: Saneesh (Saneesh) Project Name: HDI_Stellenbosch_2016

	Peak Name	RT	Area	% Area	Height
13	RIF	8.308	2157621	56.86	229473
14		8.987	5965	0.16	992
15		9.642	1464	0.04	283
16		9.909	1755	0.05	245
17		10.015	3760	0.10	332
18	NEO	10.849	431310	11.37	68070
19		11.086	35275	0.93	4123

Reported by User: Saneesh (Saneesh)   Project Name: HDI_Stellenbosch_2016

SAMPLE INFORMATION

Sample Name:	RIF Inc Basil_MeOH 10ug/mL TDI1	Acquired By:	Saneesh
Sample Type:	Unknown	Date Acquired:	2017/09/12 02:29:44 AM
Vial:	4	Acq. Method Set:	HDI_RIF_25ODESRIF_NEO
Injection #:	1	Date Processed:	2017/09/12 11:49:05 AM
Injection Volume:	15.00 ul	Processing Method:	HDI_RIF_ W2996 254.0nm-1.2
Run Time:	11.5 Minutes	Channel Name:	W2996 254.0nm-1.2
Sample Set Name:	RIF_BasilMeOH TDI IC50 12_9_17	Proc. Chnl. Descr.:	W2996 PDA 254.0 nm at 1.2

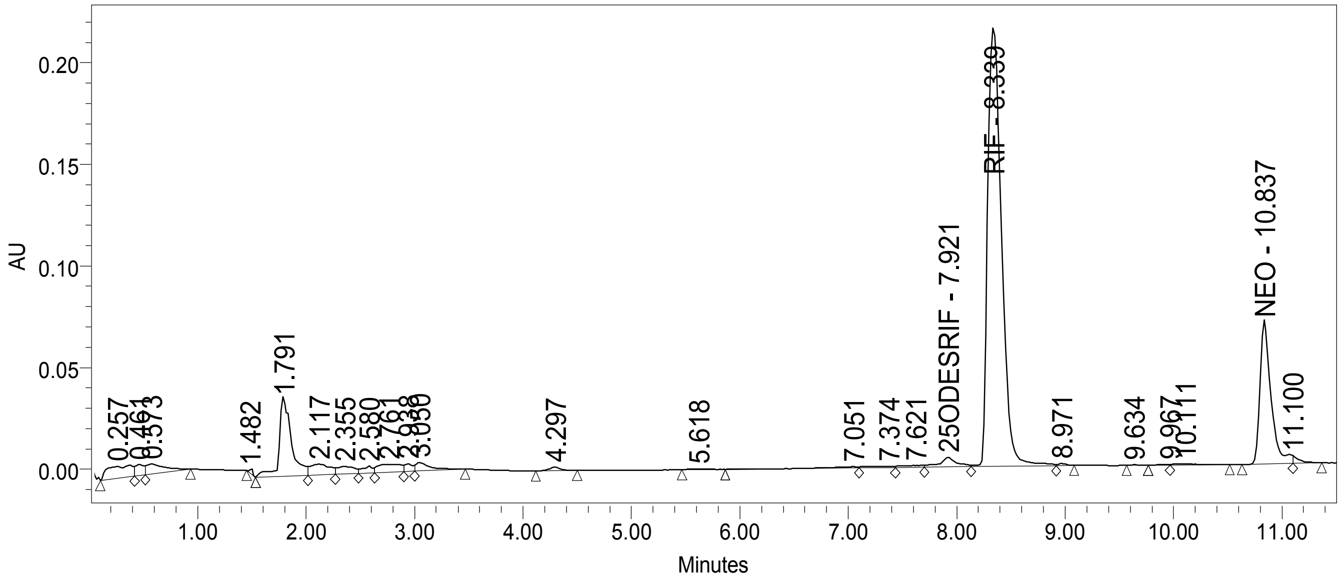

	Peak Name	RT	Area	% Area	Height
1		0.257	92709	2.82	5825
2		0.461	29586	0.90	5319
3		0.573	58201	1.77	4827
4		1.482	4937	0.15	1628
5		1.791	301729	9.18	39285
6		2.117	65670	2.00	5231
7		2.355	40849	1.24	3718
8		2.580	22271	0.68	3016
9		2.761	55596	1.69	3740
10		2.938	19357	0.59	3699
11		3.050	39791	1.21	4051
12		4.297	12764	0.39	1795

Reported by User: Saneesh (Saneesh) Project Name: HDI_Stellenbosch_2016

	Peak Name	RT	Area	% Area	Height
13		5.618	4279	0.13	334
14		7.051	17642	0.54	645
15		7.374	12185	0.37	657
16		7.621	13384	0.41	888
17	25ODESRIF	7.921	50441	1.53	4674
18	RIF	8.339	1911027	58.15	216613
19		8.971	5350	0.16	1007
20		9.634	1370	0.04	290
21		9.967	2365	0.07	347
22		10.111	9403	0.29	505
23	NEO	10.837	499553	15.20	70732
24		11.100	15816	0.48	3799

SAMPLE INFORMATION

Sample Name:	RIF Inc Basil_MeOH 10ug/mL TDI2	Acquired By:	Saneesh
Sample Type:	Unknown	Date Acquired:	2017/09/12 02:44:27 AM
Vial:	5	Acq. Method Set:	HDI_RIF_25ODESRIF_NEO
Injection #:	1	Date Processed:	2017/09/12 11:49:05 AM
Injection Volume:	15.00 ul	Processing Method:	HDI_RIF_ W2996 254.0nm-1.2
Run Time:	11.5 Minutes	Channel Name:	W2996 254.0nm-1.2
Sample Set Name:	RIF_BasilMeOH TDI IC50 12_9_17	Proc. Chnl. Descr.:	W2996 PDA 254.0 nm at 1.2

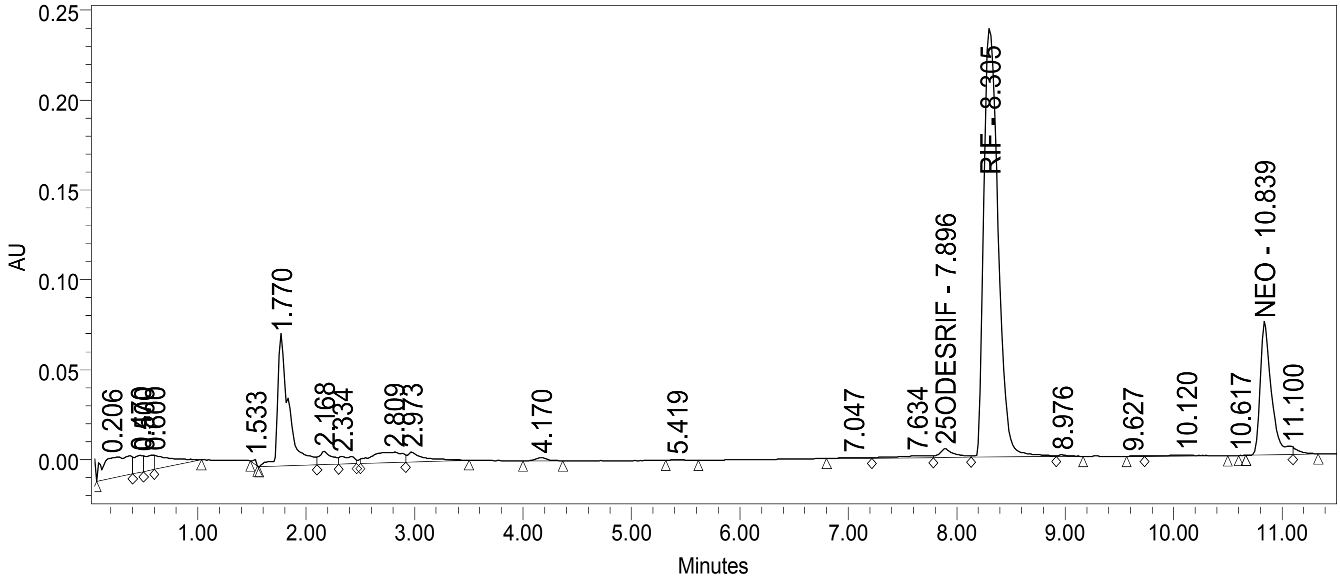

	Peak Name	RT	Area	% Area	Height
1		0.206	195366	4.82	11204
2		0.470	55151	1.36	9493
3		0.500	49288	1.22	8671
4		0.600	88481	2.18	7810
5		1.533	5187	0.13	2663
6		1.770	516327	12.75	72169
7		2.168	61262	1.51	7350
8		2.334	35649	0.88	4149
9		2.809	115097	2.84	5671
10		2.973	59666	1.47	5243
11		4.170	14135	0.35	1748
12		5.419	3089	0.08	325

	Peak Name	RT	Area	% Area	Height
13		7.047	6936	0.17	402
14		7.634	24234	0.60	1178
15	25ODESRIF	7.896	45117	1.11	4823
16	RIF	8.305	2210041	54.57	239284
17		8.976	5990	0.15	962
18		9.627	1156	0.03	213
19		10.120	10946	0.27	517
20		10.617	184	0.00	106
21	NEO	10.839	531657	13.13	74486
22		11.100	14896	0.37	3915

SAMPLE INFORMATION

Sample Name:	RIF Inc Basil_MeOH 50ug/mL TDI1	Acquired By:	Saneesh
Sample Type:	Unknown	Date Acquired:	2017/09/12 02:59:09 AM
Vial:	6	Acq. Method Set:	HDI_RIF_25ODESRIF_NEO
Injection #:	1	Date Processed:	2017/09/12 11:49:05 AM
Injection Volume:	15.00 ul	Processing Method:	HDI_RIF_ W2996 254.0nm-1.2
Run Time:	11.5 Minutes	Channel Name:	W2996 254.0nm-1.2
Sample Set Name:	RIF_BasilMeOH TDI IC50 12_9_17	Proc. Chnl. Descr.:	W2996 PDA 254.0 nm at 1.2

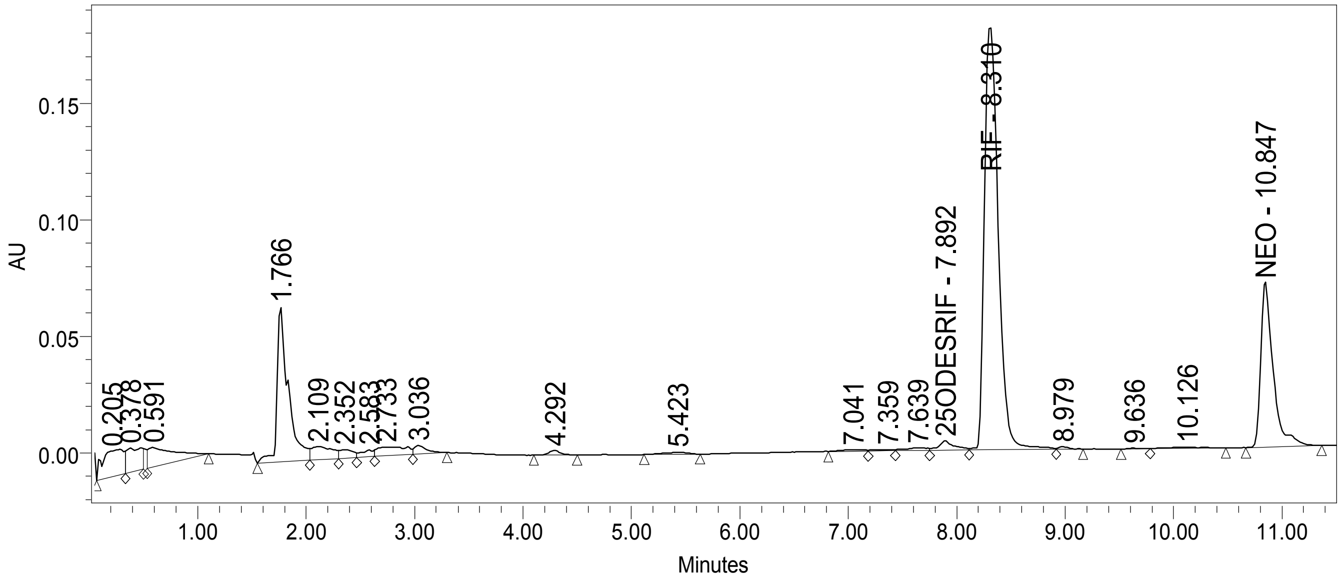

	Peak Name	RT	Area	% Area	Height
1		0.205	152106	4.50	11057
2		0.378	95224	2.82	10234
3		0.591	138560	4.10	8242
4		1.766	470946	13.93	65208
5		2.109	75111	2.22	5552
6		2.352	30998	0.92	3685
7		2.583	23281	0.69	2933
8		2.733	69241	2.05	3766
9		3.036	29292	0.87	3651
10		4.292	14733	0.44	1906
11		5.423	12937	0.38	882
12		7.041	8702	0.26	615

Reported by User: Saneesh (Saneesh) Project Name: HDI_Stellenbosch_2016

	Peak Name	RT	Area	% Area	Height
13		7.359	7132	0.21	596
14		7.639	17339	0.51	1176
15	25ODESRIF	7.892	40287	1.19	3947
16	RIF	8.310	1613955	47.73	181765
17		8.979	5827	0.17	1004
18		9.636	1784	0.05	297
19		10.126	10801	0.32	484
20	NEO	10.847	562911	16.65	70608

SAMPLE INFORMATION

Sample Name:	RIF Inc Basil_MeOH 50ug/mL TDI2	Acquired By:	Saneesh
Sample Type:	Unknown	Date Acquired:	2017/09/12 03:13:52 AM
Vial:	7	Acq. Method Set:	HDI_RIF_25ODESRIF_NEO
Injection #:	1	Date Processed:	2017/09/12 11:49:05 AM
Injection Volume:	15.00 ul	Processing Method:	HDI_RIF_ W2996 254.0nm-1.2
Run Time:	11.5 Minutes	Channel Name:	W2996 254.0nm-1.2
Sample Set Name:	RIF_BasilMeOH TDI IC50 12_9_17	Proc. Chnl. Descr.:	W2996 PDA 254.0 nm at 1.2

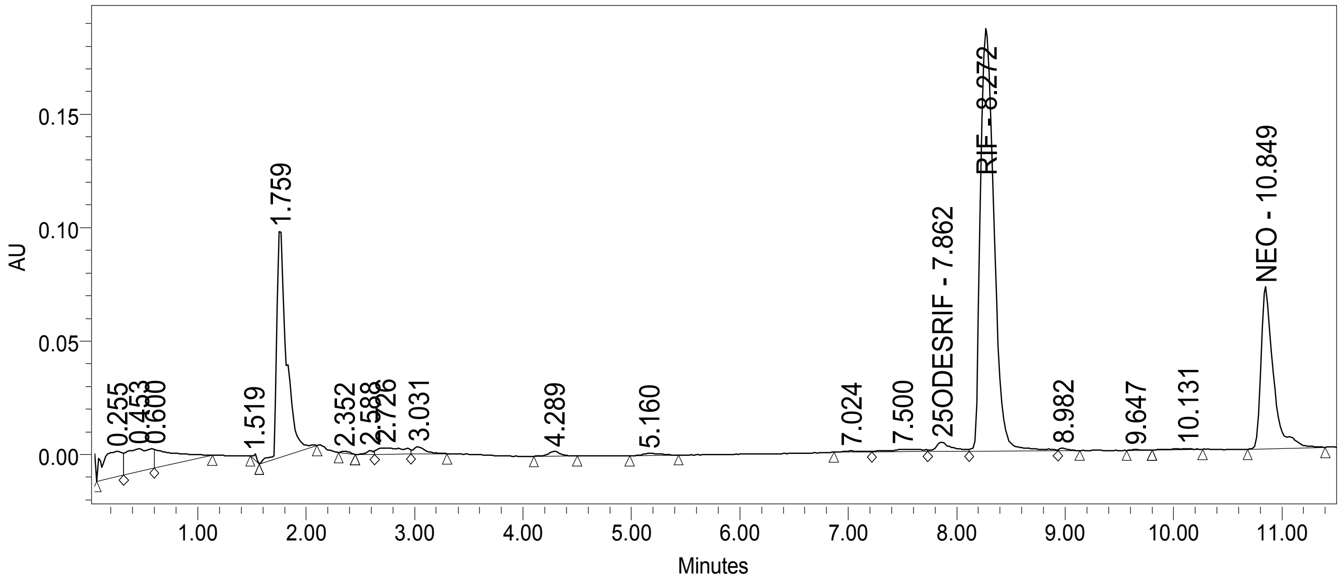

	Peak Name	RT	Area	% Area	Height
1		0.255	144290	4.21	11092
2		0.453	161343	4.71	10114
3		0.600	115544	3.37	8326
4		1.519	6238	0.18	2056
5		1.759	607856	17.73	100796
6		2.352	4476	0.13	768
7		2.588	7588	0.22	1627
8		2.726	48380	1.41	2761
9		3.031	25175	0.73	3166
10		4.289	15285	0.45	2056
11		5.160	11270	0.33	973
12		7.024	6695	0.20	495

Reported by User: Saneesh (Saneesh) Project Name: HDI_Stellenbosch_2016

	Peak Name	RT	Area	% Area	Height
13		7.500	23406	0.68	1085
14	25ODESRIF	7.862	41910	1.22	4070
15	RIF	8.272	1633870	47.67	186947
16		8.982	5415	0.16	1026
17		9.647	1443	0.04	275
18		10.131	4002	0.12	276
19	NEO	10.849	563449	16.44	71021

SAMPLE INFORMATION

Sample Name:	RIF Inc Basil_MeOH 100ug/mL TDI1	Acquired By:	Saneesh
Sample Type:	Unknown	Date Acquired:	2017/09/12 03:28:36 AM
Vial:	8	Acq. Method Set:	HDI_RIF_25ODESRIF_NEO
Injection #:	1	Date Processed:	2017/09/12 11:49:05 AM
Injection Volume:	15.00 ul	Processing Method:	HDI_RIF_ W2996 254.0nm-1.2
Run Time:	11.5 Minutes	Channel Name:	W2996 254.0nm-1.2
Sample Set Name:	RIF_BasilMeOH TDI IC50 12_9_17	Proc. Chnl. Descr.:	W2996 PDA 254.0 nm at 1.2

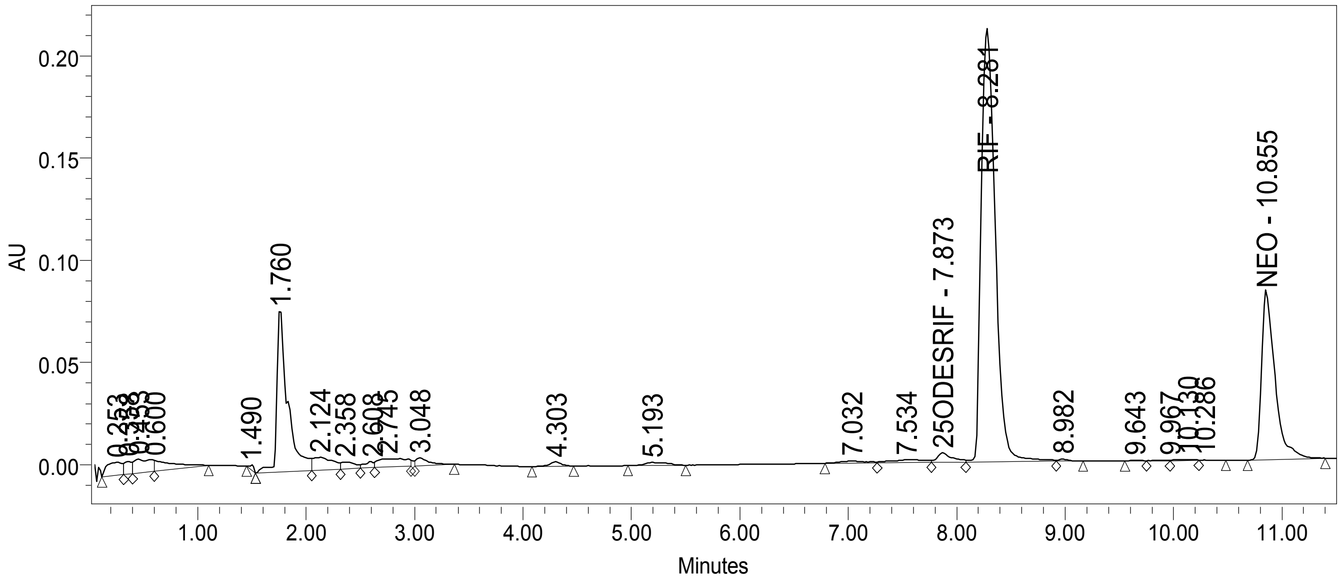

	Peak Name	RT	Area	% Area	Height
1		0.253	64618	1.70	6393
2		0.358	29697	0.78	6231
3		0.453	72409	1.91	6705
4		0.600	64884	1.71	5325
5		1.490	6768	0.18	2278
6		1.760	540971	14.27	79005
7		2.124	79205	2.09	6172
8		2.358	29898	0.79	3358
9		2.608	18721	0.49	2869
10		2.745	73486	1.94	4004
11		3.048	31794	0.84	3775
12		4.303	15996	0.42	2166

	Peak Name	RT	Area	% Area	Height
13		5.193	22891	0.60	1543
14		7.032	16362	0.43	1080
15		7.534	31240	0.82	1406
16	25ODESRIF	7.873	44148	1.16	4652
17	RIF	8.281	1915986	50.53	211883
18		8.982	6755	0.18	1086
19		9.643	2324	0.06	373
20		9.967	2558	0.07	317
21		10.130	7019	0.19	592
22		10.286	3050	0.08	326
23	NEO	10.855	710829	18.75	82995

Multi Sample Summary

Reported by User: Saneesh (Saneesh) Project Name: HDI_Stellenbosch_2016

SAMPLE INFORMATION

Sample Name:	RIF Inc Basil_MeOH 100ug/mL TDI2	Acquired By:	Saneesh
Sample Type:	Unknown	Date Acquired:	2017/09/12 03:43:18 AM
Vial:	9	Acq. Method Set:	HDI_RIF_25ODESRIF_NEO
Injection #:	1	Date Processed:	2017/09/12 11:49:05 AM
Injection Volume:	15.00 ul	Processing Method:	HDI_RIF_ W2996 254.0nm-1.2
Run Time:	11.5 Minutes	Channel Name:	W2996 254.0nm-1.2
Sample Set Name:	RIF_BasilMeOH TDI IC50 12_9_17	Proc. Chnl. Descr.:	W2996 PDA 254.0 nm at 1.2

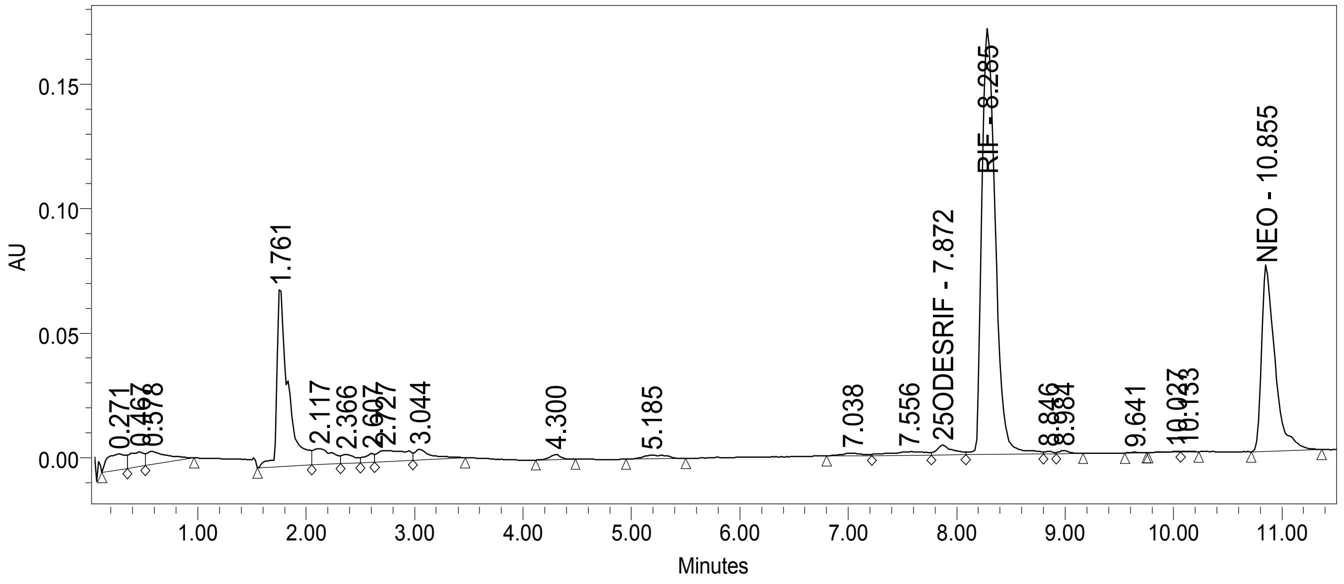

	Peak Name	RT	Area	% Area	Height
1		0.271	74911	2.32	6318
2		0.467	55374	1.71	5808
3		0.578	70143	2.17	5275
4		1.761	507264	15.71	71443
5		2.117	81259	2.52	6456
6		2.366	32003	0.99	3586
7		2.607	21675	0.67	3422
8		2.727	79660	2.47	4347
9		3.044	43991	1.36	4147
10		4.300	15423	0.48	2087
11		5.185	23869	0.74	1646
12		7.038	13868	0.43	1049

Reported by User: Saneesh (Saneesh) Project Name: HDI_Stellenbosch_2016

	Peak Name	RT	Area	% Area	Height
13		7.556	31189	0.97	1320
14	25ODESRIF	7.872	38364	1.19	3896
15	RIF	8.285	1482249	45.89	170784
16		8.846	4919	0.15	829
17		8.984	6486	0.20	1074
18		9.641	1763	0.05	319
19		10.027	2941	0.09	308
20		10.133	1988	0.06	330
21	NEO	10.855	640574	19.83	74792

Reported by User: Saneesh (Saneesh) Project Name: HDI_Stellenbosch_2016

SAMPLE INFORMATION

Sample Name:	RIF Inc Basil_MeOH 150ug/mL TDI1	Acquired By:	Saneesh
Sample Type:	Unknown	Date Acquired:	2017/09/12 03:58:01 AM
Vial:	10	Acq. Method Set:	HDI_RIF_25ODESRIF_NEO
Injection #:	1	Date Processed:	2017/09/12 11:49:05 AM
Injection Volume:	15.00 ul	Processing Method:	HDI_RIF_ W2996 254.0nm-1.2
Run Time:	11.5 Minutes	Channel Name:	W2996 254.0nm-1.2
Sample Set Name:	RIF_BasilMeOH TDI IC50 12_9_17	Proc. Chnl. Descr.:	W2996 PDA 254.0 nm at 1.2

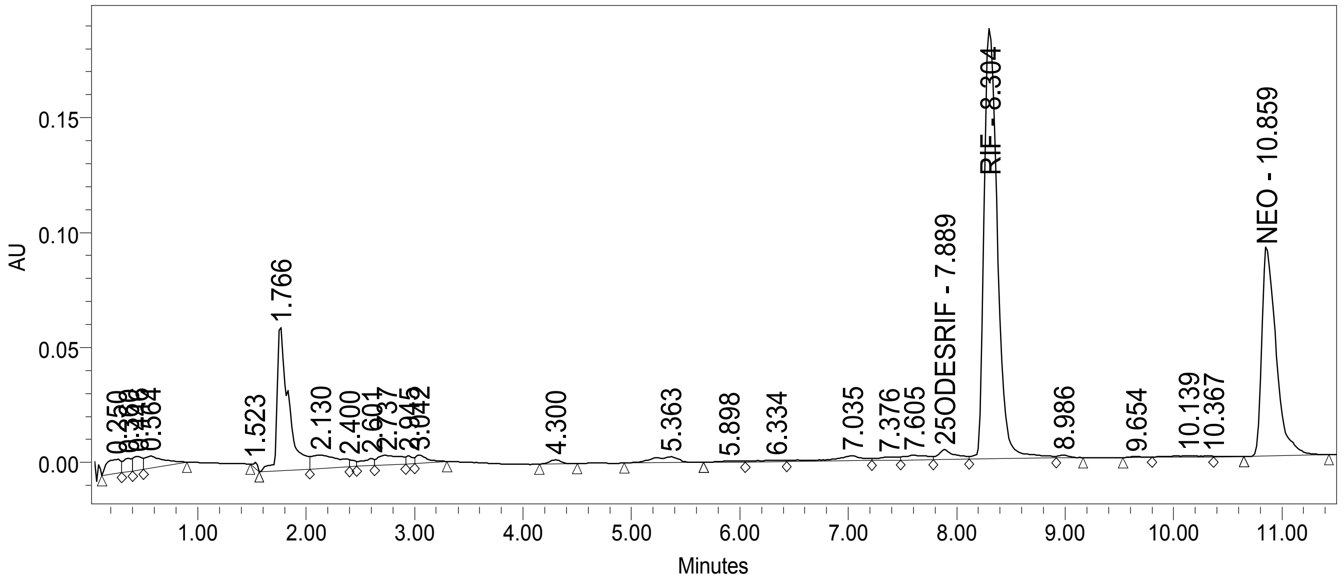

	Peak Name	RT	Area	% Area	Height
1		0.250	57100	1.60	6243
2		0.360	32824	0.92	5874
3		0.446	33386	0.94	5941
4		0.564	62212	1.74	5124
5		1.523	6890	0.19	2311
6		1.766	451722	12.65	61770
7		2.130	102634	2.87	5785
8		2.400	10538	0.30	3218
9		2.601	24864	0.70	3076
10		2.737	60293	1.69	4160
11		2.945	14671	0.41	3372
12		3.042	26909	0.75	3606

Reported by User: Saneesh (Saneesh) Project Name: HDI_Stellenbosch_2016

	Peak Name	RT	Area	% Area	Height
13		4.300	13262	0.37	1875
14		5.363	45567	1.28	2597
15		5.898	7332	0.21	452
16		6.334	10106	0.28	514
17		7.035	41025	1.15	2074
18		7.376	18016	0.50	1301
19		7.605	28975	0.81	1975
20	25ODESRIF	7.889	45137	1.26	4247
21	RIF	8.304	1613230	45.18	187661
22		8.986	8140	0.23	1269
23		9.654	3764	0.11	431
24		10.139	13850	0.39	604
25		10.367	1622	0.05	282
26	NEO	10.859	836403	23.43	91971

Reported by User: Saneesh (Saneesh)
Project Name: HDI_Stellenbosch_2016

SAMPLE INFORMATION

Sample Name:	RIF Inc Basil_MeOH 150ug/mL TDI2	Acquired By:	Saneesh
Sample Type:	Unknown	Date Acquired:	2017/09/12 04:12:45 AM
Vial:	11	Acq. Method Set:	HDI_RIF_25ODESRIF_NEO
Injection #:	1	Date Processed:	2017/09/12 11:49:05 AM
Injection Volume:	15.00 ul	Processing Method:	HDI_RIF_ W2996 254.0nm-1.2
Run Time:	11.5 Minutes	Channel Name:	W2996 254.0nm-1.2
Sample Set Name:	RIF_BasilMeOH TDI IC50 12_9_17	Proc. Chnl. Descr.:	W2996 PDA 254.0 nm at 1.2

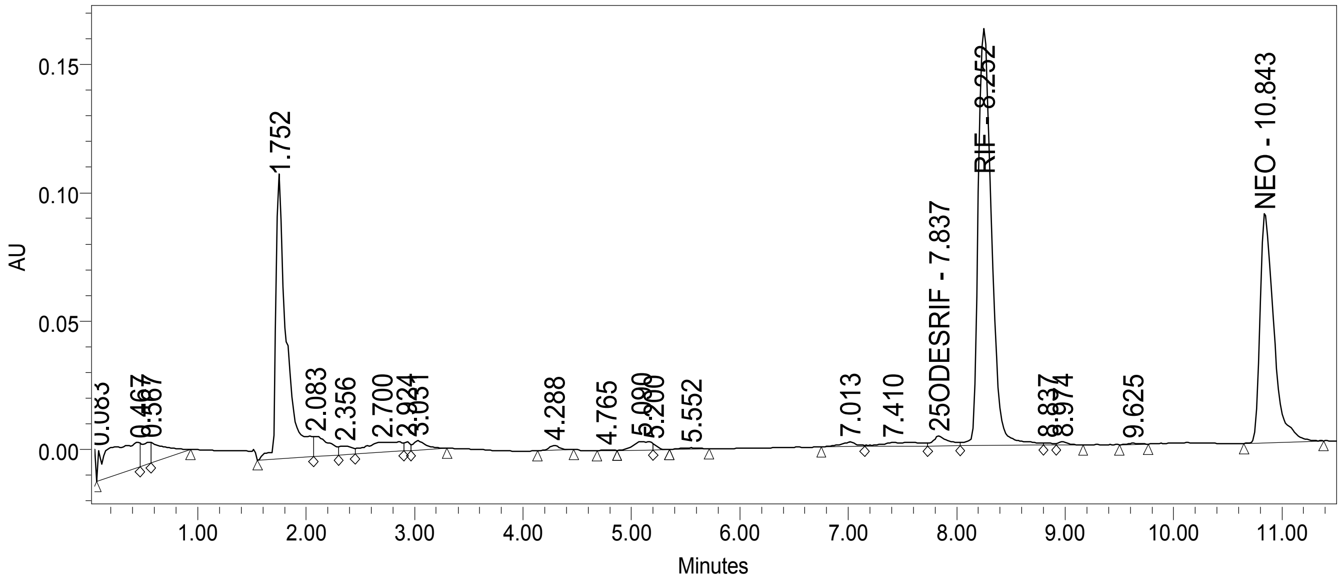

	Peak Name	RT	Area	% Area	Height
1		0.083	232375	6.26	11490
2		0.467	49845	1.34	9057
3		0.567	74743	2.01	7830
4		1.752	752345	20.26	108380
5		2.083	78563	2.12	7832
6		2.356	27486	0.74	3456
7		2.700	87500	2.36	3906
8		2.924	12333	0.33	3398
9		3.031	31595	0.85	3695
10		4.288	12714	0.34	1886
11		4.765	1400	0.04	203
12		5.090	37325	1.01	3358

	Peak Name	RT	Area	% Area	Height
13		5.200	5686	0.15	2412
14		5.552	5905	0.16	519
15		7.013	19506	0.53	1782
16		7.410	38758	1.04	1472
17	25ODESRIF	7.837	39073	1.05	3870
18	RIF	8.252	1400306	37.72	162438
19		8.837	5039	0.14	859
20		8.974	7231	0.19	1191
21		9.625	2883	0.08	434
22	NEO	10.843	789999	21.28	90495

Reported by User: Saneesh (Saneesh) Project Name: HDI_Stellenbosch_2016

SAMPLE INFORMATION

Sample Name:	RIF Inc Basil_MeOH 200ug/mL TDI1	Acquired By:	Saneesh
Sample Type:	Unknown	Date Acquired:	2017/09/12 04:27:29 AM
Vial:	12	Acq. Method Set:	HDI_RIF_25ODESRIF_NEO
Injection #:	1	Date Processed:	2017/09/12 11:49:05 AM
Injection Volume:	15.00 ul	Processing Method:	HDI_RIF_ W2996 254.0nm-1.2
Run Time:	11.5 Minutes	Channel Name:	W2996 254.0nm-1.2
Sample Set Name:	RIF_BasilMeOH TDI IC50 12_9_17	Proc. Chnl. Descr.:	W2996 PDA 254.0 nm at 1.2

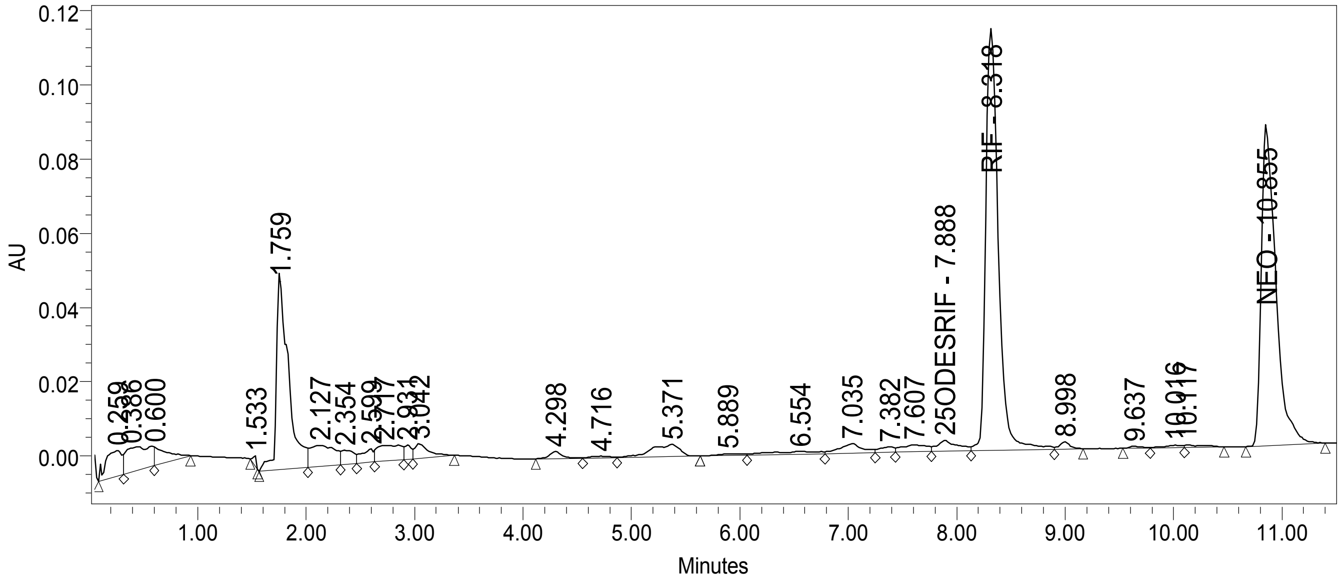

	Peak Name	RT	Area	% Area	Height
1		0.259	72637	2.52	6863
2		0.386	99197	3.44	6527
3		0.600	42031	1.46	4998
4		1.533	5397	0.19	2822
5		1.759	402287	13.96	52833
6		2.127	89428	3.10	5705
7		2.354	30236	1.05	3787
8		2.599	28195	0.98	3334
9		2.717	62872	2.18	4224
10		2.931	16636	0.58	3772
11		3.042	35567	1.23	3920
12		4.298	15975	0.55	1902

Reported by User: Saneesh (Saneesh) Project Name: HDI_Stellenbosch_2016

	Peak Name	RT	Area	% Area	Height
13		4.716	5548	0.19	442
14		5.371	61799	2.14	3364
15		5.889	8408	0.29	496
16		6.554	24552	0.85	731
17		7.035	38475	1.33	2511
18		7.382	13919	0.48	1505
19		7.607	31286	1.09	1933
20	25ODESRIF	7.888	38663	1.34	2930
21	RIF	8.318	918292	31.86	113595
22		8.998	11909	0.41	1878
23		9.637	3998	0.14	518
24		10.016	7361	0.26	639
25		10.117	8480	0.29	666
26	NEO	10.855	809288	28.08	86930

SAMPLE INFORMATION

Sample Name:	RIF Inc Basil_MeOH 200ug/mL TDI2	Acquired By:	Saneesh
Sample Type:	Unknown	Date Acquired:	2017/09/12 04:42:12 AM
Vial:	13	Acq. Method Set:	HDI_RIF_25ODESRIF_NEO
Injection #:	1	Date Processed:	2017/09/12 11:49:05 AM
Injection Volume:	15.00 ul	Processing Method:	HDI_RIF_ W2996 254.0nm-1.2
Run Time:	11.5 Minutes	Channel Name:	W2996 254.0nm-1.2
Sample Set Name:	RIF_BasilMeOH TDI IC50 12_9_17	Proc. Chnl. Descr.:	W2996 PDA 254.0 nm at 1.2

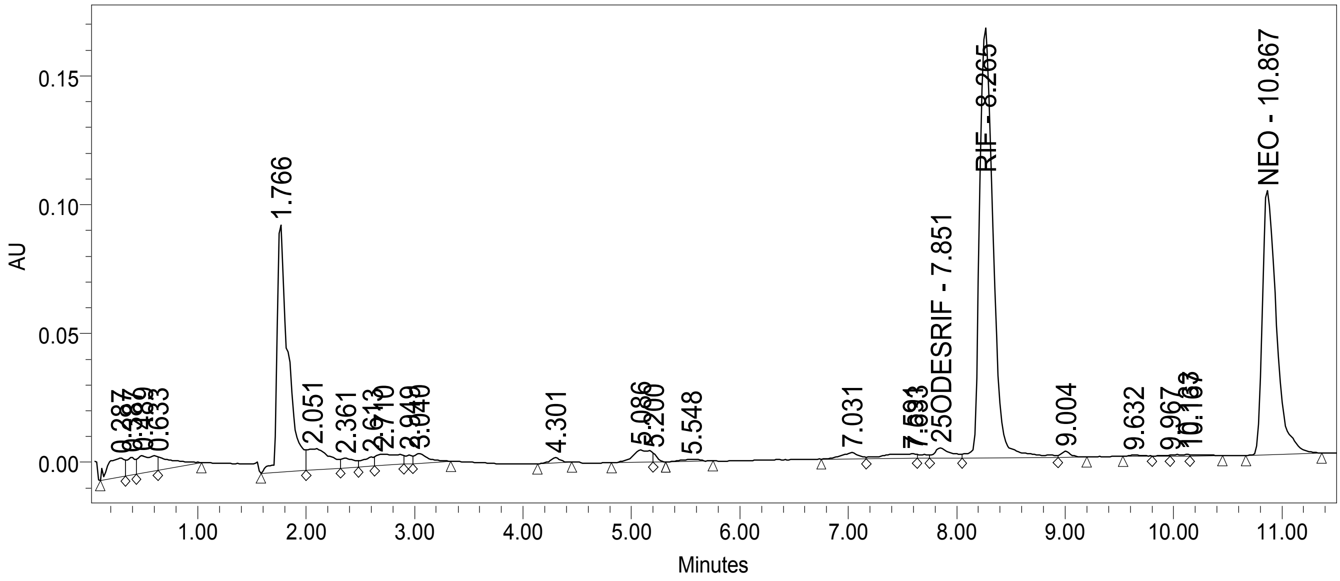

	Peak Name	RT	Area	% Area	Height
1		0.287	78380	2.06	7153
2		0.387	37642	0.99	6731
3		0.489	71309	1.87	6619
4		0.633	51964	1.36	5193
5		1.766	668948	17.57	95124
6		2.051	117301	3.08	8106
7		2.361	31153	0.82	3712
8		2.613	26664	0.70	3551
9		2.710	64097	1.68	4411
10		2.949	16106	0.42	3425
11		3.040	33312	0.87	3855
12		4.301	13134	0.34	1955

Reported by User: Saneesh (Saneesh)   Project Name: HDI_Stellenbosch_2016

	Peak Name	RT	Area	% Area	Height
13		5.086	54911	1.44	4817
14		5.200	8853	0.23	3604
15		5.548	10032	0.26	781
16		7.031	27229	0.72	2452
17		7.591	37293	0.98	1817
18		7.633	9944	0.26	1576
19	25ODESRIF	7.851	42475	1.12	4085
20	RIF	8.265	1451154	38.11	166772
21		9.004	14300	0.38	2307
22		9.632	3871	0.10	515
23		9.967	1993	0.05	347
24		10.133	5936	0.16	626
25		10.167	5212	0.14	537
26	NEO	10.867	924328	24.28	102600

Reported by User: Saneesh (Saneesh)   Project Name: HDI_Stellenbosch_2016

SAMPLE INFORMATION

Sample Name:	RIF Inc Basil TDI Test Contrl K1	Acquired By:	Saneesh
Sample Type:	Unknown	Date Acquired:	2017/09/12 01:29:43 AM
Vial:	23	Acq. Method Set:	HDI_RIF_25ODESRIF_NEO
Injection #:	1	Date Processed:	2017/09/12 11:49:05 AM
Injection Volume:	15.00 ul	Processing Method:	HDI_RIF_ W2996 254.0nm-1.2
Run Time:	11.5 Minutes	Channel Name:	W2996 254.0nm-1.2
Sample Set Name:	RIF_BasilMeOH TDI IC50 12_9_17	Proc. Chnl. Descr.:	W2996 PDA 254.0 nm at 1.2

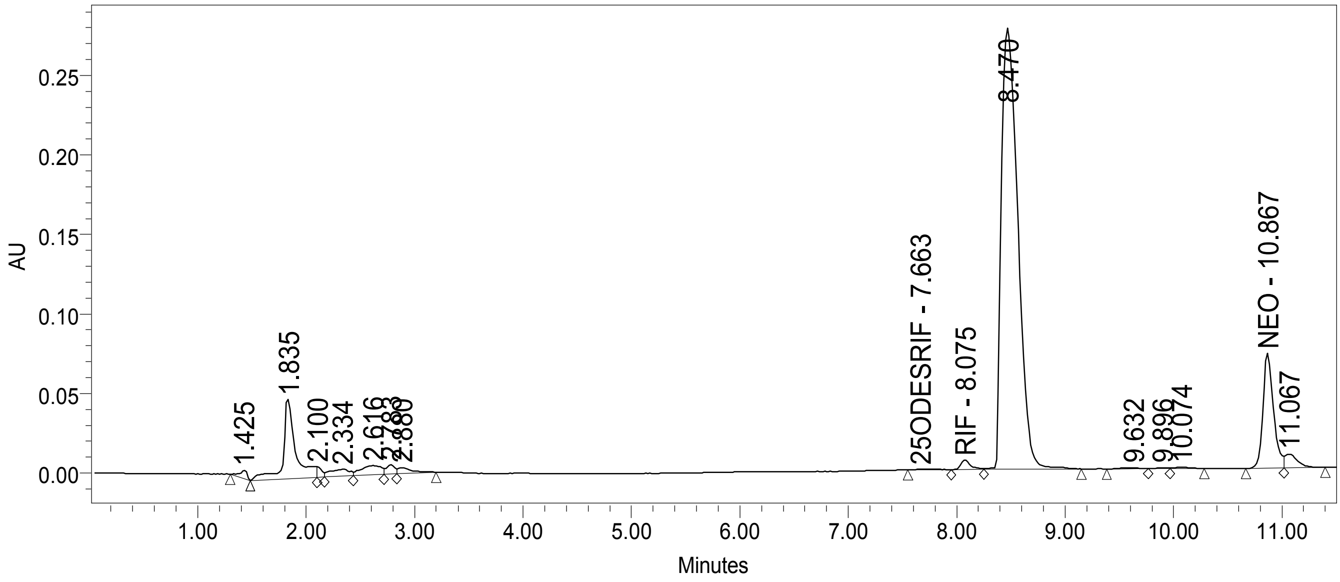

	Peak Name	RT	Area	% Area	Height
1		1.425	24118	0.57	5327
2		1.835	397807	9.47	50729
3		2.100	19583	0.47	6647
4		2.334	56733	1.35	4338
5		2.616	76149	1.81	5765
6		2.783	31786	0.76	5761
7		2.880	32977	0.78	3692
8	25ODESRIF	7.663	4852	0.12	331
9	RIF	8.075	37948	0.90	5910
10		8.470	2936387	69.88	277984
11		9.632	10247	0.24	812
12		9.896	6059	0.14	788
13		10.074	10008	0.24	834
14	NEO	10.867	485602	11.56	71752

Reported by User: Saneesh (Saneesh) Project Name: HDI_Stellenbosch_2016

	Peak Name	RT	Area	% Area	Height
15		11.067	71966	1.71	8627

SAMPLE INFORMATION

Sample Name:	RIF Inc Basil TDI Test Contrl K2	Acquired By:	Saneesh
Sample Type:	Unknown	Date Acquired:	2017/09/12 01:44:37 AM
Vial:	24	Acq. Method Set:	HDI_RIF_25ODESRIF_NEO
Injection #:	1	Date Processed:	2017/09/12 11:49:05 AM
Injection Volume:	15.00 ul	Processing Method:	HDI_RIF_ W2996 254.0nm-1.2
Run Time:	11.5 Minutes	Channel Name:	W2996 254.0nm-1.2
Sample Set Name:	RIF_BasilMeOH TDI IC50 12_9_17	Proc. Chnl. Descr.:	W2996 PDA 254.0 nm at 1.2

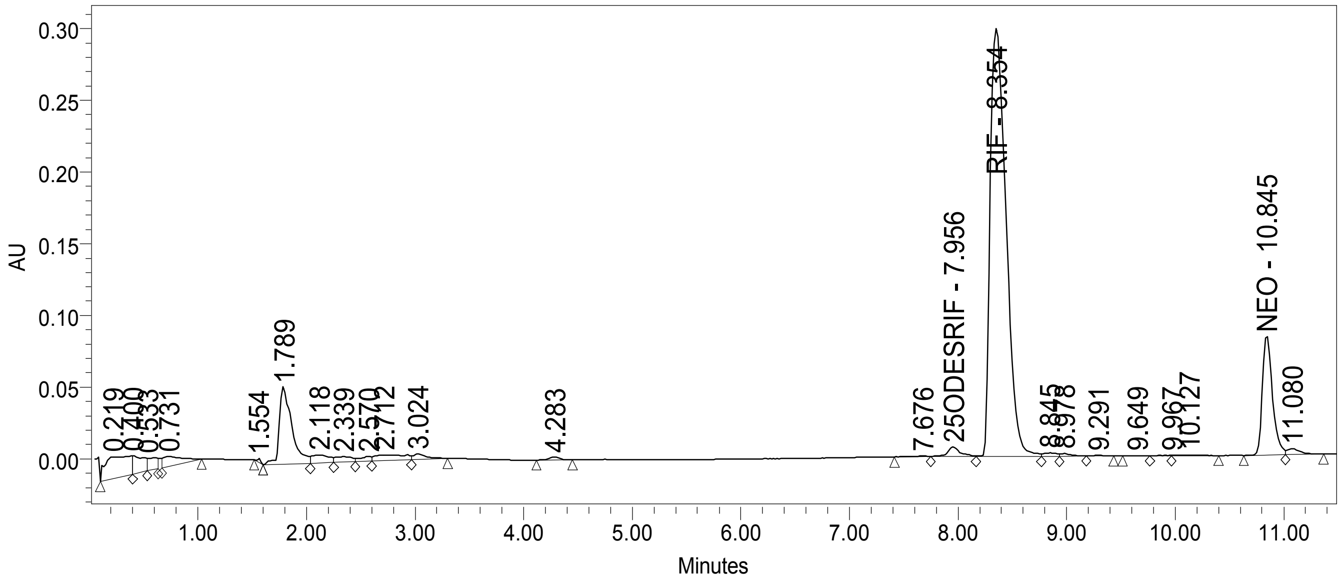

	Peak Name	RT	Area	% Area	Height
1		0.219	230177	4.74	15047
2		0.400	84033	1.73	12542
3		0.533	48696	1.00	8909
4		0.731	82814	1.70	6770
5		1.554	6402	0.13	2169
6		1.789	427174	8.79	53845
7		2.118	60941	1.25	5495
8		2.339	38736	0.80	3699
9		2.570	23146	0.48	3235
10		2.712	75849	1.56	3930
11		3.024	30930	0.64	3731
12		4.283	14416	0.30	1928

	Peak Name	RT	Area	% Area	Height
13		7.676	6426	0.13	497
14	25ODESRIF	7.956	57120	1.18	6842
15	RIF	8.354	3050317	62.75	298984
16		8.845	20709	0.43	2398
17		8.978	14860	0.31	1953
18		9.291	5120	0.11	609
19		9.649	2261	0.05	284
20		9.967	2934	0.06	363
21		10.127	8602	0.18	490
22	NEO	10.845	533793	10.98	82534
23		11.080	35260	0.73	4155

SAMPLE INFORMATION

Sample Name:	RIF Inc Basil_MeOH 1ug/mL TDI1	Acquired By:	Saneesh
Sample Type:	Unknown	Date Acquired:	2017/09/12 02:00:18 AM
Vial:	2	Acq. Method Set:	HDI_RIF_25ODESRIF_NEO
Injection #:	1	Date Processed:	2017/09/12 11:49:05 AM
Injection Volume:	15.00 ul	Processing Method:	HDI_RIF_ W2996 254.0nm-1.2
Run Time:	11.5 Minutes	Channel Name:	W2996 254.0nm-1.2
Sample Set Name:	RIF_BasilMeOH TDI IC50 12_9_17	Proc. Chnl. Descr.:	W2996 PDA 254.0 nm at 1.2

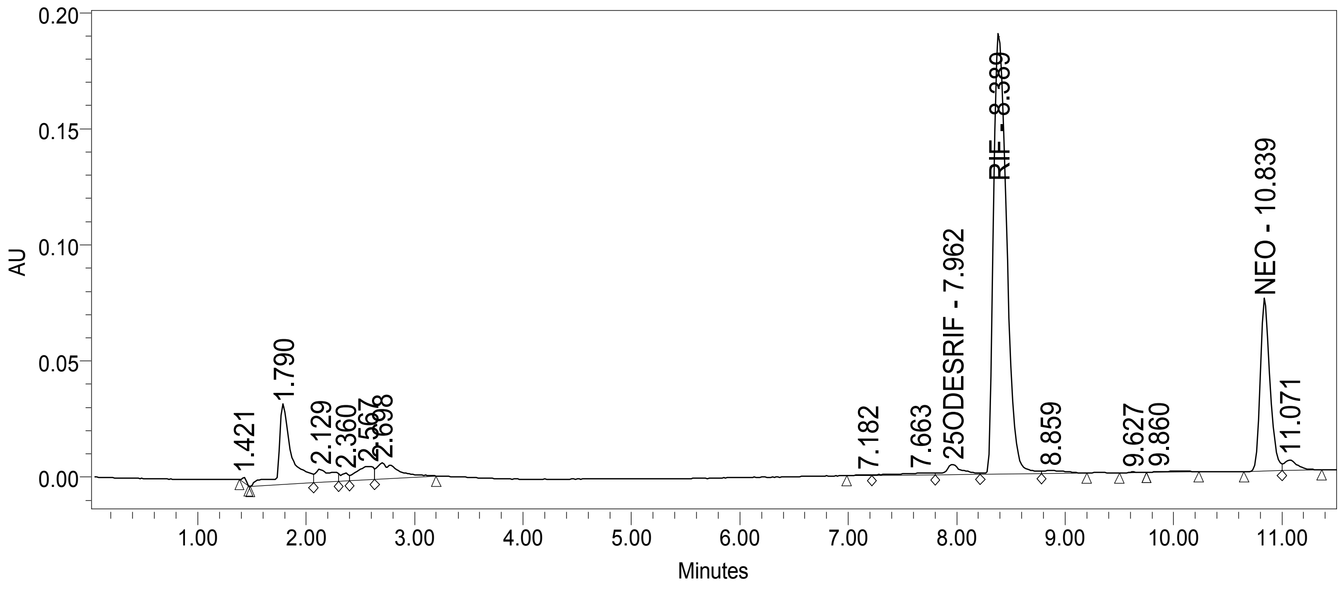

	Peak Name	RT	Area	% Area	Height
1		1.421	5972	0.22	1992
2		1.790	287819	10.62	34693
3		2.129	60723	2.24	5590
4		2.360	17981	0.66	3366
5		2.567	62561	2.31	5592
6		2.698	91208	3.37	6871
7		7.182	3335	0.12	358
8		7.663	19208	0.71	732
9	25ODESRIF	7.962	42480	1.57	4344
10	RIF	8.389	1591303	58.74	190518
11		8.859	15469	0.57	1181
12		9.627	1939	0.07	317

	Peak Name	RT	Area	% Area	Height
13		9.860	6313	0.23	313
14	NEO	10.839	463782	17.12	74432
15		11.071	39168	1.45	4520

Multi Sample Summary

Reported by User: Saneesh (Saneesh)   Project Name: HDI_Stellenbosch_2016

SAMPLE INFORMATION

Sample Name:	RIF Inc Basil_MeOH 1ug/mL TDI2	Acquired By:	Saneesh
Sample Type:	Unknown	Date Acquired:	2017/09/12 02:15:00 AM
Vial:	3	Acq. Method Set:	HDI_RIF_25ODESRIF_NEO
Injection #:	1	Date Processed:	2017/09/12 11:49:05 AM
Injection Volume:	15.00 ul	Processing Method:	HDI_RIF_ W2996 254.0nm-1.2
Run Time:	11.5 Minutes	Channel Name:	W2996 254.0nm-1.2
Sample Set Name:	RIF_BasilMeOH TDI IC50 12_9_17	Proc. Chnl. Descr.:	W2996 PDA 254.0 nm at 1.2

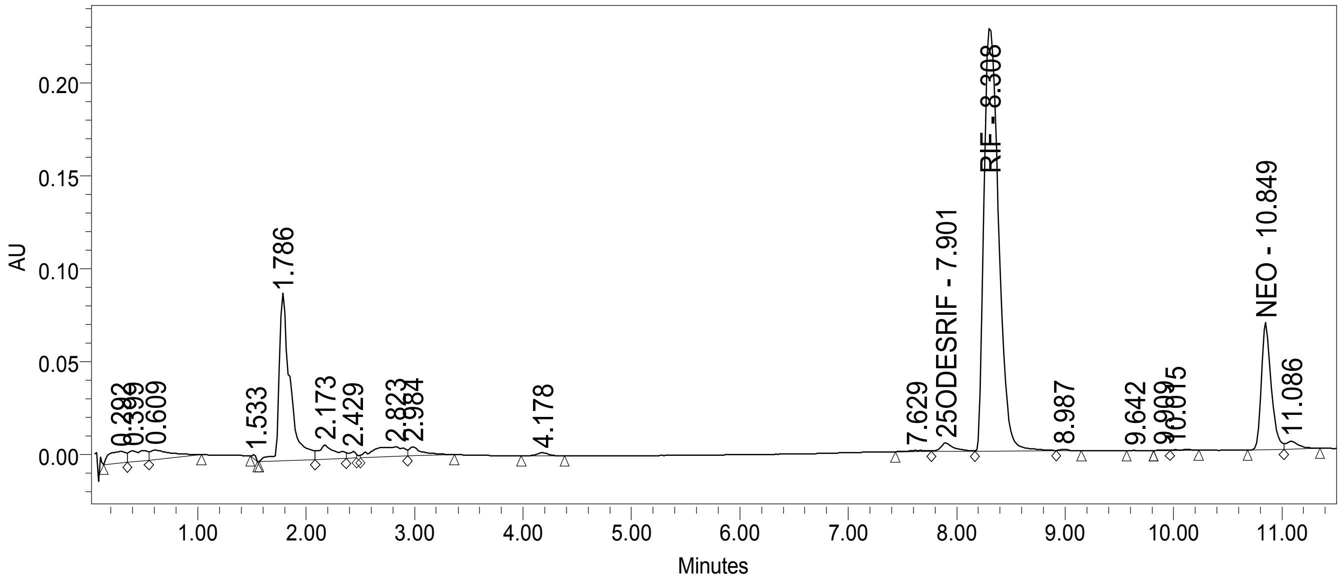

	Peak Name	RT	Area	% Area	Height
1		0.292	68441	1.80	6182
2		0.399	62348	1.64	5822
3		0.609	69668	1.84	5040
4		1.533	5524	0.15	2420
5		1.786	634466	16.72	88869
6		2.173	82882	2.18	7687
7		2.429	17161	0.45	3363
8		2.823	107268	2.83	5208
9		2.984	42931	1.13	4864
10		4.178	14289	0.38	1745
11		7.629	10463	0.28	771
12	25ODESRIF	7.901	41819	1.10	4734

Reported by User: Saneesh (Saneesh) Project Name: HDI_Stellenbosch_2016

	Peak Name	RT	Area	% Area	Height
13	RIF	8.308	2157621	56.86	229473
14		8.987	5965	0.16	992
15		9.642	1464	0.04	283
16		9.909	1755	0.05	245
17		10.015	3760	0.10	332
18	NEO	10.849	431310	11.37	68070
19		11.086	35275	0.93	4123

SAMPLE INFORMATION

Sample Name:	RIF Inc Basil_MeOH 10ug/mL TDI1	Acquired By:	Saneesh
Sample Type:	Unknown	Date Acquired:	2017/09/12 02:29:44 AM
Vial:	4	Acq. Method Set:	HDI_RIF_25ODESRIF_NEO
Injection #:	1	Date Processed:	2017/09/12 11:49:05 AM
Injection Volume:	15.00 ul	Processing Method:	HDI_RIF_ W2996 254.0nm-1.2
Run Time:	11.5 Minutes	Channel Name:	W2996 254.0nm-1.2
Sample Set Name:	RIF_BasilMeOH TDI IC50 12_9_17	Proc. Chnl. Descr.:	W2996 PDA 254.0 nm at 1.2

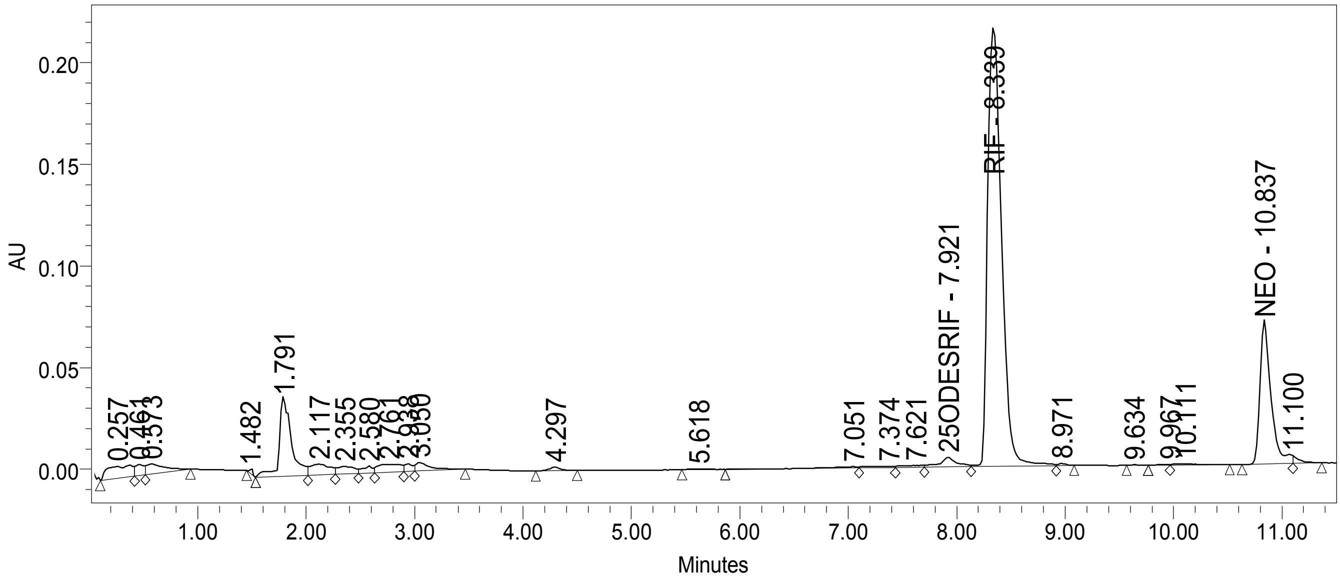

	Peak Name	RT	Area	% Area	Height
1		0.257	92709	2.82	5825
2		0.461	29586	0.90	5319
3		0.573	58201	1.77	4827
4		1.482	4937	0.15	1628
5		1.791	301729	9.18	39285
6		2.117	65670	2.00	5231
7		2.355	40849	1.24	3718
8		2.580	22271	0.68	3016
9		2.761	55596	1.69	3740
10		2.938	19357	0.59	3699
11		3.050	39791	1.21	4051
12		4.297	12764	0.39	1795

Reported by User: Saneesh (Saneesh)   Project Name: HDI_Stellenbosch_2016

	Peak Name	RT	Area	% Area	Height
13		5.618	4279	0.13	334
14		7.051	17642	0.54	645
15		7.374	12185	0.37	657
16		7.621	13384	0.41	888
17	25ODESRIF	7.921	50441	1.53	4674
18	RIF	8.339	1911027	58.15	216613
19		8.971	5350	0.16	1007
20		9.634	1370	0.04	290
21		9.967	2365	0.07	347
22		10.111	9403	0.29	505
23	NEO	10.837	499553	15.20	70732
24		11.100	15816	0.48	3799

Reported by User: Saneesh (Saneesh)   Project Name: HDI_Stellenbosch_2016

SAMPLE INFORMATION

Sample Name:	RIF Inc Basil_MeOH 10ug/mL TDI2	Acquired By:	Saneesh
Sample Type:	Unknown	Date Acquired:	2017/09/12 02:44:27 AM
Vial:	5	Acq. Method Set:	HDI_RIF_25ODESRIF_NEO
Injection #:	1	Date Processed:	2017/09/12 11:49:05 AM
Injection Volume:	15.00 ul	Processing Method:	HDI_RIF_ W2996 254.0nm-1.2
Run Time:	11.5 Minutes	Channel Name:	W2996 254.0nm-1.2
Sample Set Name:	RIF_BasilMeOH TDI IC50 12_9_17	Proc. Chnl. Descr.:	W2996 PDA 254.0 nm at 1.2

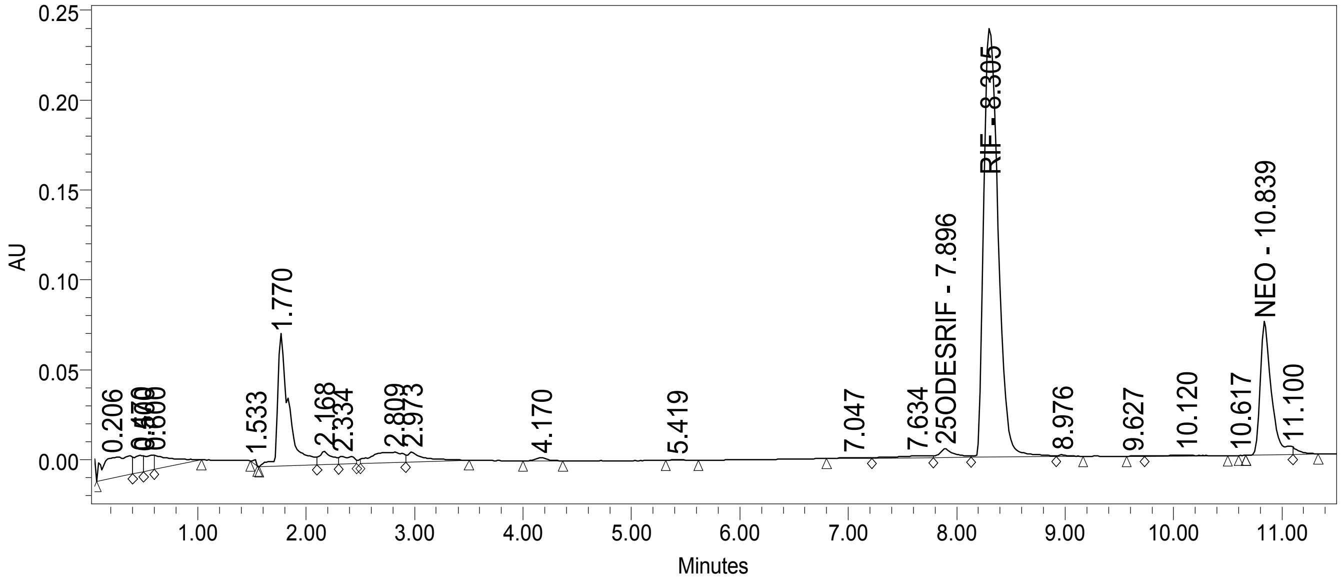

	Peak Name	RT	Area	% Area	Height
1		0.206	195366	4.82	11204
2		0.470	55151	1.36	9493
3		0.500	49288	1.22	8671
4		0.600	88481	2.18	7810
5		1.533	5187	0.13	2663
6		1.770	516327	12.75	72169
7		2.168	61262	1.51	7350
8		2.334	35649	0.88	4149
9		2.809	115097	2.84	5671
10		2.973	59666	1.47	5243
11		4.170	14135	0.35	1748
12		5.419	3089	0.08	325

Reported by User: Saneesh (Saneesh) Project Name: HDI_Stellenbosch_2016

	Peak Name	RT	Area	% Area	Height
13		7.047	6936	0.17	402
14		7.634	24234	0.60	1178
15	25ODESRIF	7.896	45117	1.11	4823
16	RIF	8.305	2210041	54.57	239284
17		8.976	5990	0.15	962
18		9.627	1156	0.03	213
19		10.120	10946	0.27	517
20		10.617	184	0.00	106
21	NEO	10.839	531657	13.13	74486
22		11.100	14896	0.37	3915

Reported by User: Saneesh (Saneesh) Project Name: HDI_Stellenbosch_2016

SAMPLE INFORMATION

Sample Name:	RIF Inc Basil_MeOH 50ug/mL TDI1	Acquired By:	Saneesh
Sample Type:	Unknown	Date Acquired:	2017/09/12 02:59:09 AM
Vial:	6	Acq. Method Set:	HDI_RIF_25ODESRIF_NEO
Injection #:	1	Date Processed:	2017/09/12 11:49:05 AM
Injection Volume:	15.00 ul	Processing Method:	HDI_RIF_ W2996 254.0nm-1.2
Run Time:	11.5 Minutes	Channel Name:	W2996 254.0nm-1.2
Sample Set Name:	RIF_BasilMeOH TDI IC50 12_9_17	Proc. Chnl. Descr.:	W2996 PDA 254.0 nm at 1.2

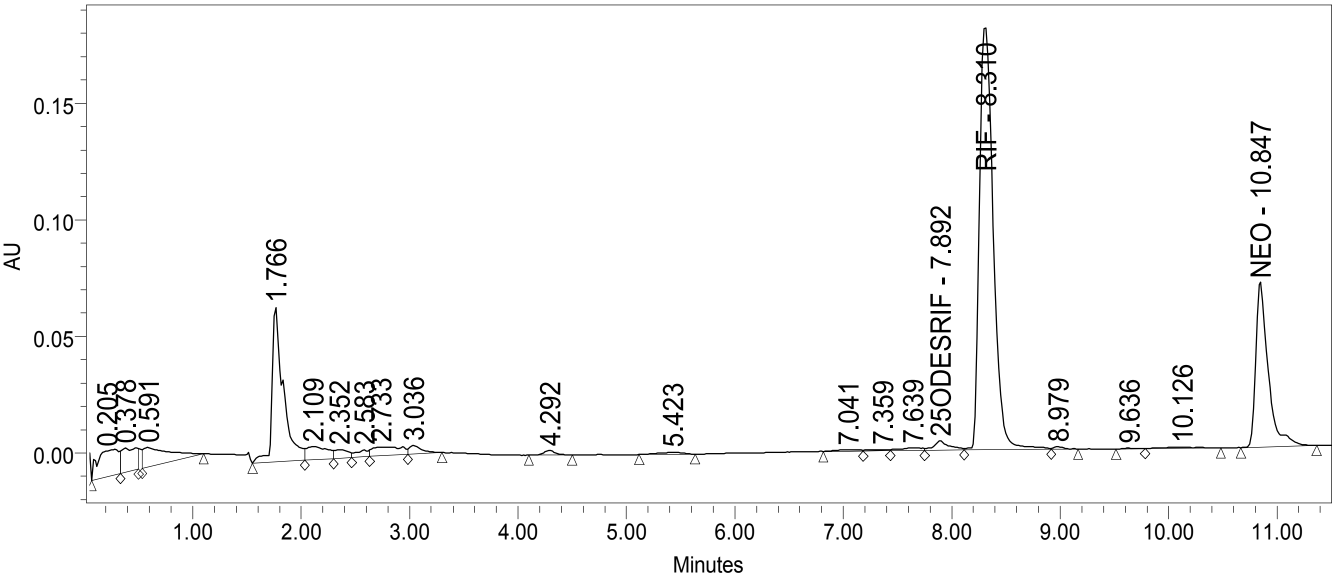

	Peak Name	RT	Area	% Area	Height
1		0.205	152106	4.50	11057
2		0.378	95224	2.82	10234
3		0.591	138560	4.10	8242
4		1.766	470946	13.93	65208
5		2.109	75111	2.22	5552
6		2.352	30998	0.92	3685
7		2.583	23281	0.69	2933
8		2.733	69241	2.05	3766
9		3.036	29292	0.87	3651
10		4.292	14733	0.44	1906
11		5.423	12937	0.38	882
12		7.041	8702	0.26	615

	Peak Name	RT	Area	% Area	Height
13		7.359	7132	0.21	596
14		7.639	17339	0.51	1176
15	25ODESRIF	7.892	40287	1.19	3947
16	RIF	8.310	1613955	47.73	181765
17		8.979	5827	0.17	1004
18		9.636	1784	0.05	297
19		10.126	10801	0.32	484
20	NEO	10.847	562911	16.65	70608

SAMPLE INFORMATION

Sample Name:	RIF Inc Basil_MeOH 50ug/mL TDI2	Acquired By:	Saneesh
Sample Type:	Unknown	Date Acquired:	2017/09/12 03:13:52 AM
Vial:	7	Acq. Method Set:	HDI_RIF_25ODESRIF_NEO
Injection #:	1	Date Processed:	2017/09/12 11:49:05 AM
Injection Volume:	15.00 ul	Processing Method:	HDI_RIF_ W2996 254.0nm-1.2
Run Time:	11.5 Minutes	Channel Name:	W2996 254.0nm-1.2
Sample Set Name:	RIF_BasilMeOH TDI IC50 12_9_17	Proc. Chnl. Descr.:	W2996 PDA 254.0 nm at 1.2

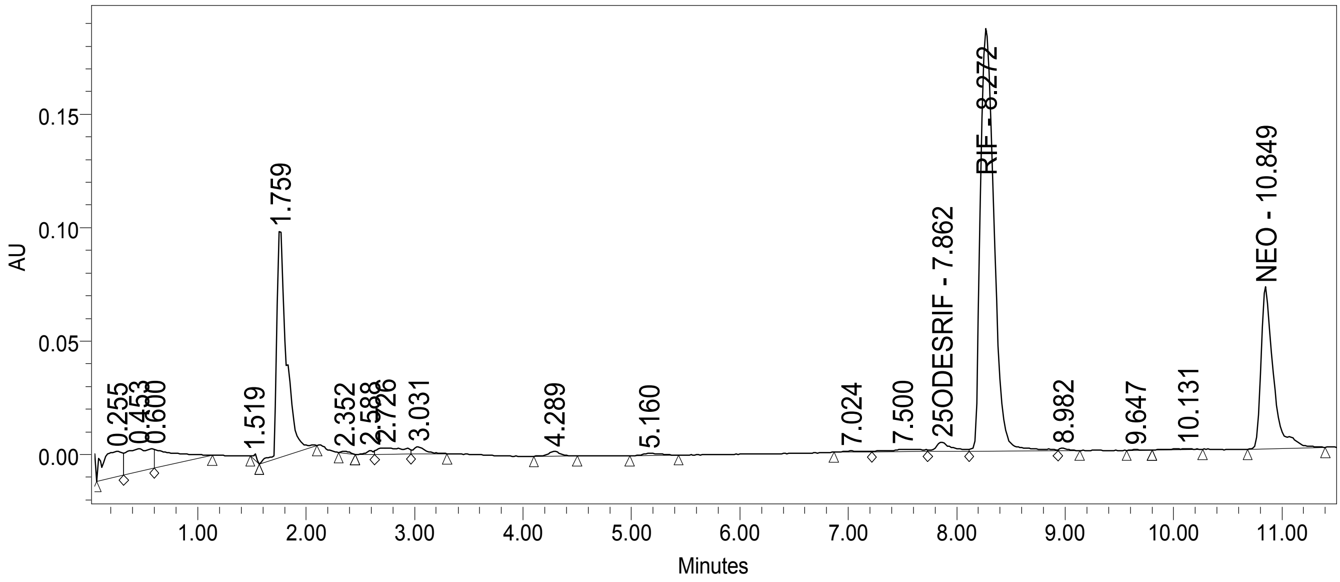

	Peak Name	RT	Area	% Area	Height
1		0.255	144290	4.21	11092
2		0.453	161343	4.71	10114
3		0.600	115544	3.37	8326
4		1.519	6238	0.18	2056
5		1.759	607856	17.73	100796
6		2.352	4476	0.13	768
7		2.588	7588	0.22	1627
8		2.726	48380	1.41	2761
9		3.031	25175	0.73	3166
10		4.289	15285	0.45	2056
11		5.160	11270	0.33	973
12		7.024	6695	0.20	495

Reported by User: Saneesh (Saneesh) Project Name: HDI_Stellenbosch_2016

	Peak Name	RT	Area	% Area	Height
13		7.500	23406	0.68	1085
14	25ODESRIF	7.862	41910	1.22	4070
15	RIF	8.272	1633870	47.67	186947
16		8.982	5415	0.16	1026
17		9.647	1443	0.04	275
18		10.131	4002	0.12	276
19	NEO	10.849	563449	16.44	71021

Reported by User: Saneesh (Saneesh) Project Name: HDI_Stellenbosch_2016

SAMPLE INFORMATION

Sample Name:	RIF Inc Basil_MeOH 100ug/mL TDI1	Acquired By:	Saneesh
Sample Type:	Unknown	Date Acquired:	2017/09/12 03:28:36 AM
Vial:	8	Acq. Method Set:	HDI_RIF_25ODESRIF_NEO
Injection #:	1	Date Processed:	2017/09/12 11:49:05 AM
Injection Volume:	15.00 ul	Processing Method:	HDI_RIF_ W2996 254.0nm-1.2
Run Time:	11.5 Minutes	Channel Name:	W2996 254.0nm-1.2
Sample Set Name:	RIF_BasilMeOH TDI IC50 12_9_17	Proc. Chnl. Descr.:	W2996 PDA 254.0 nm at 1.2

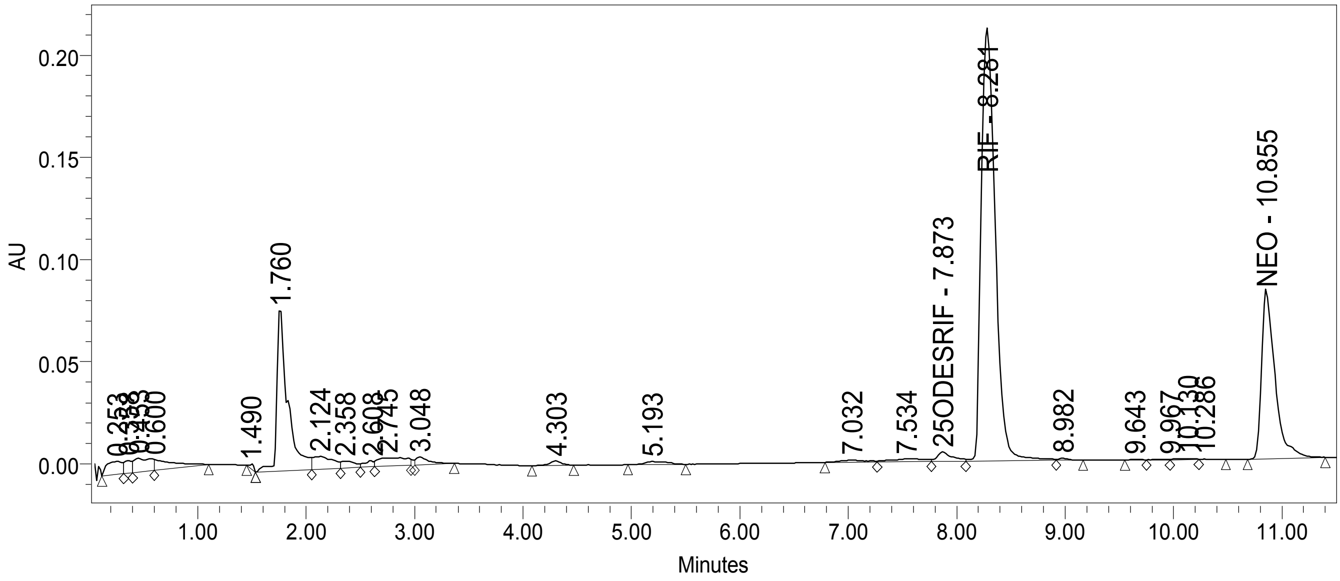

	Peak Name	RT	Area	% Area	Height
1		0.253	64618	1.70	6393
2		0.358	29697	0.78	6231
3		0.453	72409	1.91	6705
4		0.600	64884	1.71	5325
5		1.490	6768	0.18	2278
6		1.760	540971	14.27	79005
7		2.124	79205	2.09	6172
8		2.358	29898	0.79	3358
9		2.608	18721	0.49	2869
10		2.745	73486	1.94	4004
11		3.048	31794	0.84	3775
12		4.303	15996	0.42	2166

Reported by User: Saneesh (Saneesh) Project Name: HDI_Stellenbosch_2016

	Peak Name	RT	Area	% Area	Height
13		5.193	22891	0.60	1543
14		7.032	16362	0.43	1080
15		7.534	31240	0.82	1406
16	25ODESRIF	7.873	44148	1.16	4652
17	RIF	8.281	1915986	50.53	211883
18		8.982	6755	0.18	1086
19		9.643	2324	0.06	373
20		9.967	2558	0.07	317
21		10.130	7019	0.19	592
22		10.286	3050	0.08	326
23	NEO	10.855	710829	18.75	82995

Reported by User: Saneesh (Saneesh) Project Name: HDI_Stellenbosch_2016

SAMPLE INFORMATION

Sample Name:	RIF Inc Basil_MeOH 100ug/mL TDI2	Acquired By:	Saneesh
Sample Type:	Unknown	Date Acquired:	2017/09/12 03:43:18 AM
Vial:	9	Acq. Method Set:	HDI_RIF_25ODESRIF_NEO
Injection #:	1	Date Processed:	2017/09/12 11:49:05 AM
Injection Volume:	15.00 ul	Processing Method:	HDI_RIF_ W2996 254.0nm-1.2
Run Time:	11.5 Minutes	Channel Name:	W2996 254.0nm-1.2
Sample Set Name:	RIF_BasilMeOH TDI IC50 12_9_17	Proc. Chnl. Descr.:	W2996 PDA 254.0 nm at 1.2

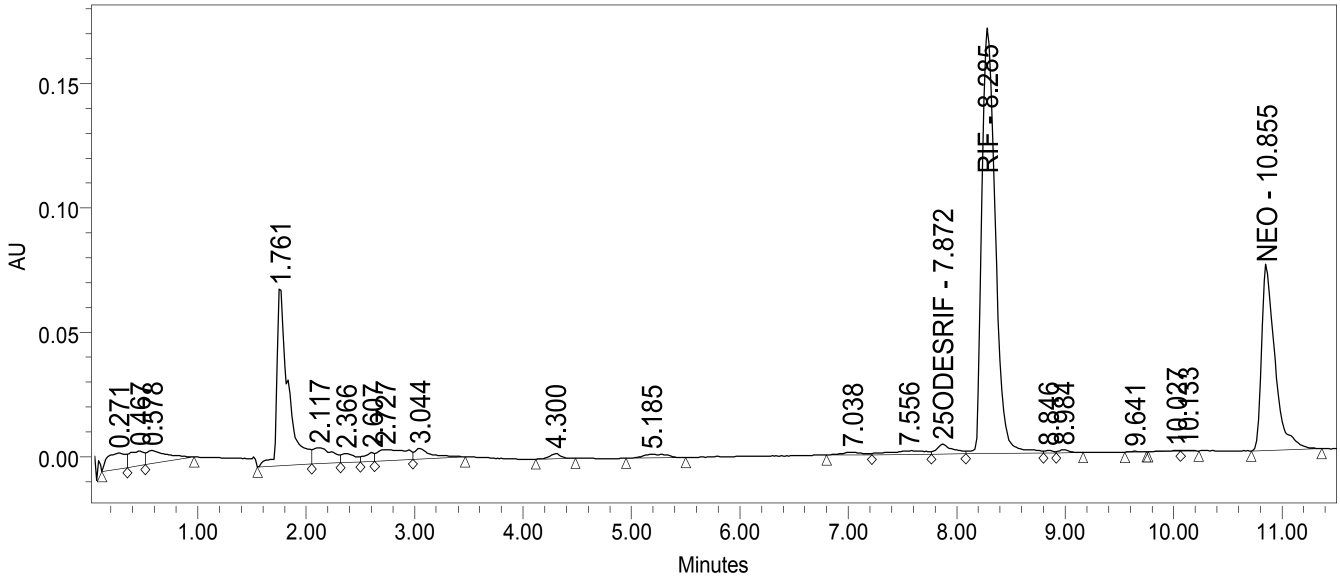

	Peak Name	RT	Area	% Area	Height
1		0.271	74911	2.32	6318
2		0.467	55374	1.71	5808
3		0.578	70143	2.17	5275
4		1.761	507264	15.71	71443
5		2.117	81259	2.52	6456
6		2.366	32003	0.99	3586
7		2.607	21675	0.67	3422
8		2.727	79660	2.47	4347
9		3.044	43991	1.36	4147
10		4.300	15423	0.48	2087
11		5.185	23869	0.74	1646
12		7.038	13868	0.43	1049

Reported by User: Saneesh (Saneesh) Project Name: HDI_Stellenbosch_2016

	Peak Name	RT	Area	% Area	Height
13		7.556	31189	0.97	1320
14	25ODESRIF	7.872	38364	1.19	3896
15	RIF	8.285	1482249	45.89	170784
16		8.846	4919	0.15	829
17		8.984	6486	0.20	1074
18		9.641	1763	0.05	319
19		10.027	2941	0.09	308
20		10.133	1988	0.06	330
21	NEO	10.855	640574	19.83	74792

Reported by User: Saneesh (Saneesh) Project Name: HDI_Stellenbosch_2016

SAMPLE INFORMATION

Sample Name:	RIF Inc Basil_MeOH 150ug/mL TDI1	Acquired By:	Saneesh
Sample Type:	Unknown	Date Acquired:	2017/09/12 03:58:01 AM
Vial:	10	Acq. Method Set:	HDI_RIF_25ODESRIF_NEO
Injection #:	1	Date Processed:	2017/09/12 11:49:05 AM
Injection Volume:	15.00 ul	Processing Method:	HDI_RIF_ W2996 254.0nm-1.2
Run Time:	11.5 Minutes	Channel Name:	W2996 254.0nm-1.2
Sample Set Name:	RIF_BasilMeOH TDI IC50 12_9_17	Proc. Chnl. Descr.:	W2996 PDA 254.0 nm at 1.2

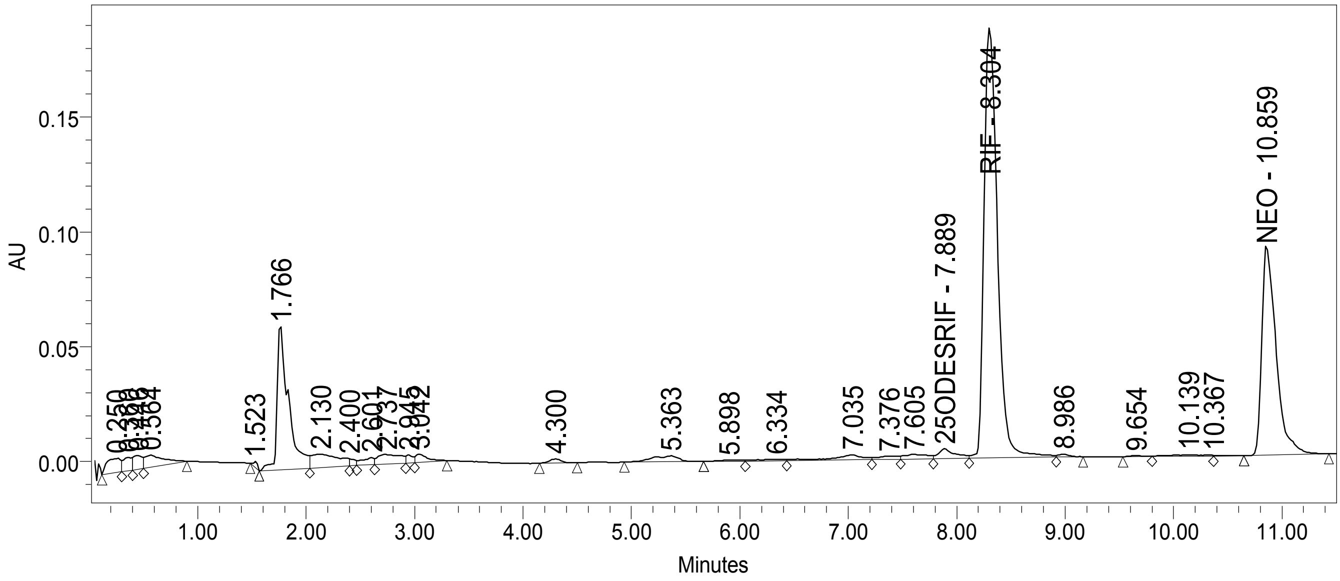

	Peak Name	RT	Area	% Area	Height
1		0.250	57100	1.60	6243
2		0.360	32824	0.92	5874
3		0.446	33386	0.94	5941
4		0.564	62212	1.74	5124
5		1.523	6890	0.19	2311
6		1.766	451722	12.65	61770
7		2.130	102634	2.87	5785
8		2.400	10538	0.30	3218
9		2.601	24864	0.70	3076
10		2.737	60293	1.69	4160
11		2.945	14671	0.41	3372
12		3.042	26909	0.75	3606

Reported by User: Saneesh (Saneesh) Project Name: HDI_Stellenbosch_2016

	Peak Name	RT	Area	% Area	Height
13		4.300	13262	0.37	1875
14		5.363	45567	1.28	2597
15		5.898	7332	0.21	452
16		6.334	10106	0.28	514
17		7.035	41025	1.15	2074
18		7.376	18016	0.50	1301
19		7.605	28975	0.81	1975
20	25ODESRIF	7.889	45137	1.26	4247
21	RIF	8.304	1613230	45.18	187661
22		8.986	8140	0.23	1269
23		9.654	3764	0.11	431
24		10.139	13850	0.39	604
25		10.367	1622	0.05	282
26	NEO	10.859	836403	23.43	91971

SAMPLE INFORMATION

Sample Name:	RIF Inc Basil_MeOH 150ug/mL TDI2	Acquired By:	Saneesh
Sample Type:	Unknown	Date Acquired:	2017/09/12 04:12:45 AM
Vial:	11	Acq. Method Set:	HDI_RIF_25ODESRIF_NEO
Injection #:	1	Date Processed:	2017/09/12 11:49:05 AM
Injection Volume:	15.00 ul	Processing Method:	HDI_RIF_ W2996 254.0nm-1.2
Run Time:	11.5 Minutes	Channel Name:	W2996 254.0nm-1.2
Sample Set Name:	RIF_BasilMeOH TDI IC50 12_9_17	Proc. Chnl. Descr.:	W2996 PDA 254.0 nm at 1.2

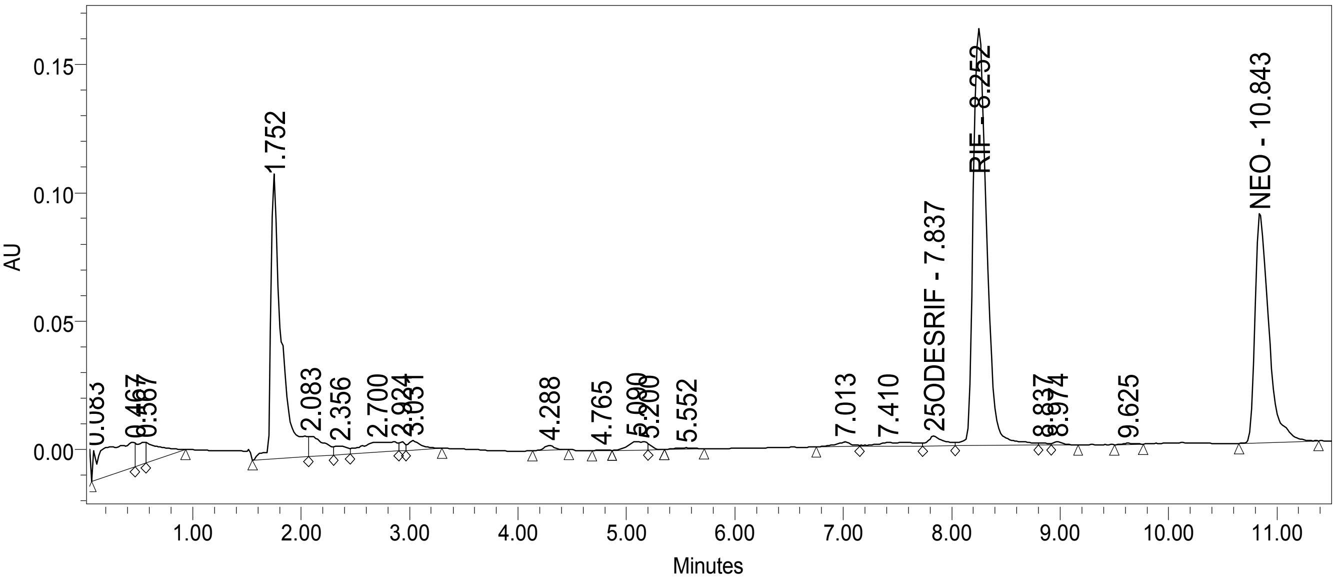

	Peak Name	RT	Area	% Area	Height
1		0.083	232375	6.26	11490
2		0.467	49845	1.34	9057
3		0.567	74743	2.01	7830
4		1.752	752345	20.26	108380
5		2.083	78563	2.12	7832
6		2.356	27486	0.74	3456
7		2.700	87500	2.36	3906
8		2.924	12333	0.33	3398
9		3.031	31595	0.85	3695
10		4.288	12714	0.34	1886
11		4.765	1400	0.04	203
12		5.090	37325	1.01	3358

Reported by User: Saneesh (Saneesh) Project Name: HDI_Stellenbosch_2016

	Peak Name	RT	Area	% Area	Height
13		5.200	5686	0.15	2412
14		5.552	5905	0.16	519
15		7.013	19506	0.53	1782
16		7.410	38758	1.04	1472
17	25ODESRIF	7.837	39073	1.05	3870
18	RIF	8.252	1400306	37.72	162438
19		8.837	5039	0.14	859
20		8.974	7231	0.19	1191
21		9.625	2883	0.08	434
22	NEO	10.843	789999	21.28	90495

SAMPLE INFORMATION

Sample Name:	RIF Inc Basil_MeOH 200ug/mL TDI1	Acquired By:	Saneesh
Sample Type:	Unknown	Date Acquired:	2017/09/12 04:27:29 AM
Vial:	12	Acq. Method Set:	HDI_RIF_25ODESRIF_NEO
Injection #:	1	Date Processed:	2017/09/12 11:49:05 AM
Injection Volume:	15.00 ul	Processing Method:	HDI_RIF_ W2996 254.0nm-1.2
Run Time:	11.5 Minutes	Channel Name:	W2996 254.0nm-1.2
Sample Set Name:	RIF_BasilMeOH TDI IC50 12_9_17	Proc. Chnl. Descr.:	W2996 PDA 254.0 nm at 1.2

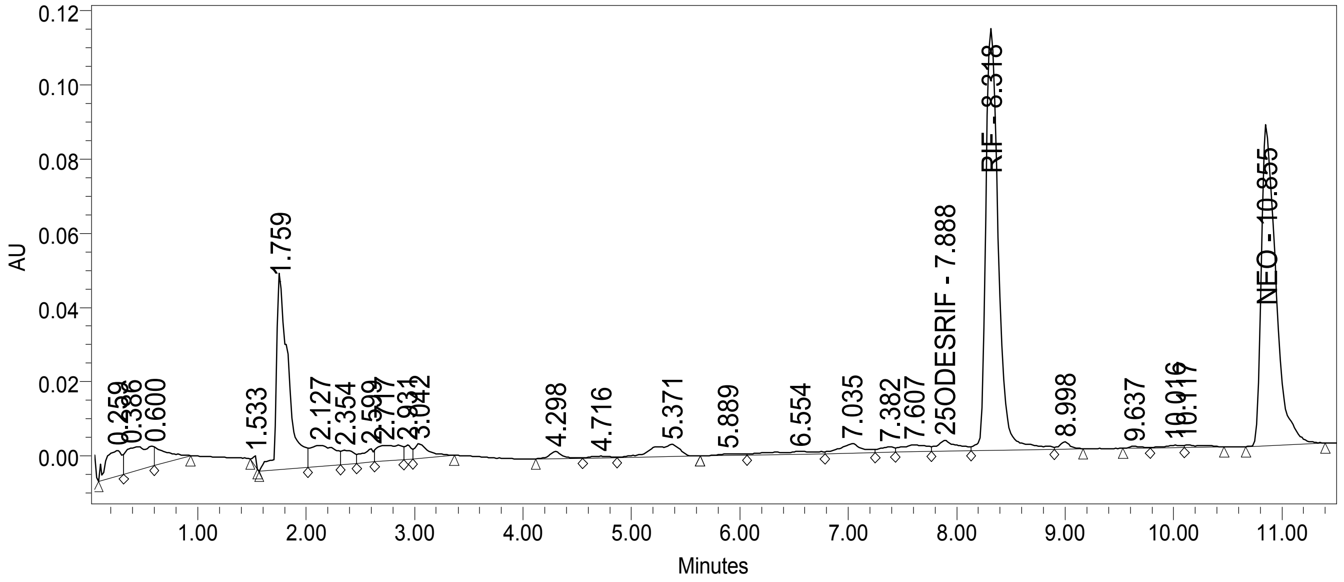

	Peak Name	RT	Area	% Area	Height
1		0.259	72637	2.52	6863
2		0.386	99197	3.44	6527
3		0.600	42031	1.46	4998
4		1.533	5397	0.19	2822
5		1.759	402287	13.96	52833
6		2.127	89428	3.10	5705
7		2.354	30236	1.05	3787
8		2.599	28195	0.98	3334
9		2.717	62872	2.18	4224
10		2.931	16636	0.58	3772
11		3.042	35567	1.23	3920
12		4.298	15975	0.55	1902

	Peak Name	RT	Area	% Area	Height
13		4.716	5548	0.19	442
14		5.371	61799	2.14	3364
15		5.889	8408	0.29	496
16		6.554	24552	0.85	731
17		7.035	38475	1.33	2511
18		7.382	13919	0.48	1505
19		7.607	31286	1.09	1933
20	25ODESRIF	7.888	38663	1.34	2930
21	RIF	8.318	918292	31.86	113595
22		8.998	11909	0.41	1878
23		9.637	3998	0.14	518
24		10.016	7361	0.26	639
25		10.117	8480	0.29	666
26	NEO	10.855	809288	28.08	86930

SAMPLE INFORMATION

Sample Name:	RIF Inc Basil_MeOH 200ug/mL TDI2	Acquired By:	Saneesh
Sample Type:	Unknown	Date Acquired:	2017/09/12 04:42:12 AM
Vial:	13	Acq. Method Set:	HDI_RIF_25ODESRIF_NEO
Injection #:	1	Date Processed:	2017/09/12 11:49:05 AM
Injection Volume:	15.00 ul	Processing Method:	HDI_RIF_ W2996 254.0nm-1.2
Run Time:	11.5 Minutes	Channel Name:	W2996 254.0nm-1.2
Sample Set Name:	RIF_BasilMeOH TDI IC50 12_9_17	Proc. Chnl. Descr.:	W2996 PDA 254.0 nm at 1.2

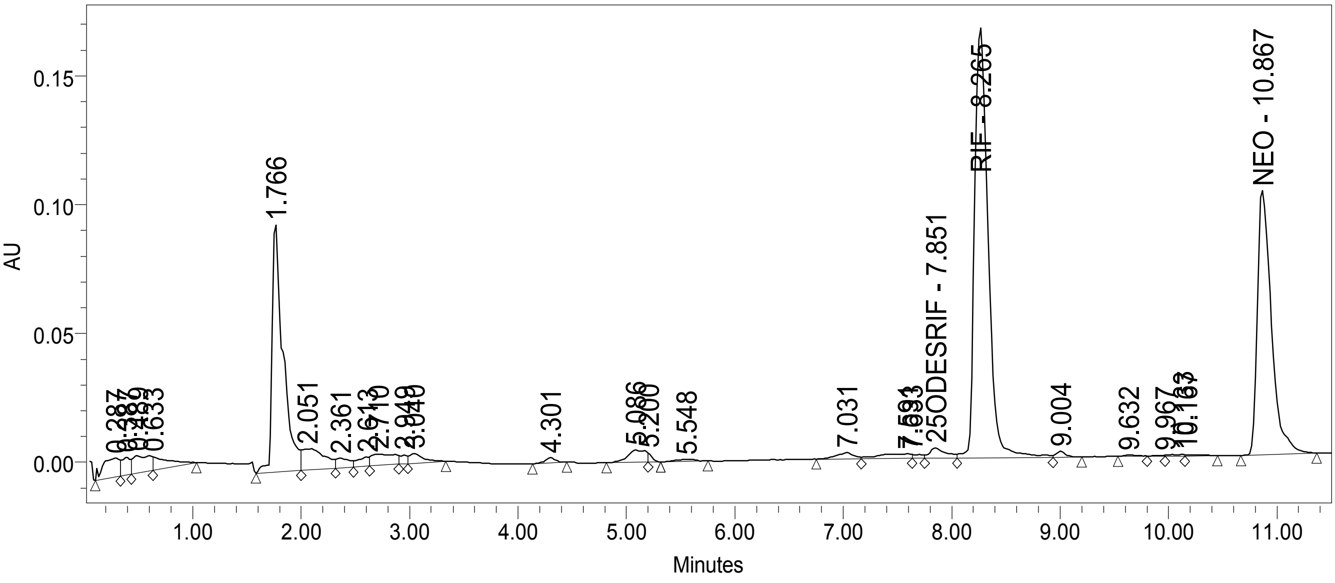

	Peak Name	RT	Area	% Area	Height
1		0.287	78380	2.06	7153
2		0.387	37642	0.99	6731
3		0.489	71309	1.87	6619
4		0.633	51964	1.36	5193
5		1.766	668948	17.57	95124
6		2.051	117301	3.08	8106
7		2.361	31153	0.82	3712
8		2.613	26664	0.70	3551
9		2.710	64097	1.68	4411
10		2.949	16106	0.42	3425
11		3.040	33312	0.87	3855
12		4.301	13134	0.34	1955

	Peak Name	RT	Area	% Area	Height
13		5.086	54911	1.44	4817
14		5.200	8853	0.23	3604
15		5.548	10032	0.26	781
16		7.031	27229	0.72	2452
17		7.591	37293	0.98	1817
18		7.633	9944	0.26	1576
19	25ODESRIF	7.851	42475	1.12	4085
20	RIF	8.265	1451154	38.11	166772
21		9.004	14300	0.38	2307
22		9.632	3871	0.10	515
23		9.967	1993	0.05	347
24		10.133	5936	0.16	626
25		10.167	5212	0.14	537
26	NEO	10.867	924328	24.28	102600

Reported by User: Saneesh (Saneesh)   Project Name: HDI_Stellenbosch_2016

SAMPLE INFORMATION

Sample Name:	RIF Inc Basil TDI Test Contrl K1	Acquired By:	Saneesh
Sample Type:	Unknown	Date Acquired:	2017/09/12 06:46:37 AM
Vial:	23	Acq. Method Set:	HDI_RIF_25ODESRIF_NEO
Injection #:	1	Date Processed:	2017/09/12 11:49:45 AM
Injection Volume:	15.00 ul	Processing Method:	HDI_RIF_ W2996 254.0nm-1.2
Run Time:	11.5 Minutes	Channel Name:	W2996 254.0nm-1.2
Sample Set Name:	RIF_Basil EOH TDI IC50 12_9_17	Proc. Chnl. Descr.:	W2996 PDA 254.0 nm at 1.2

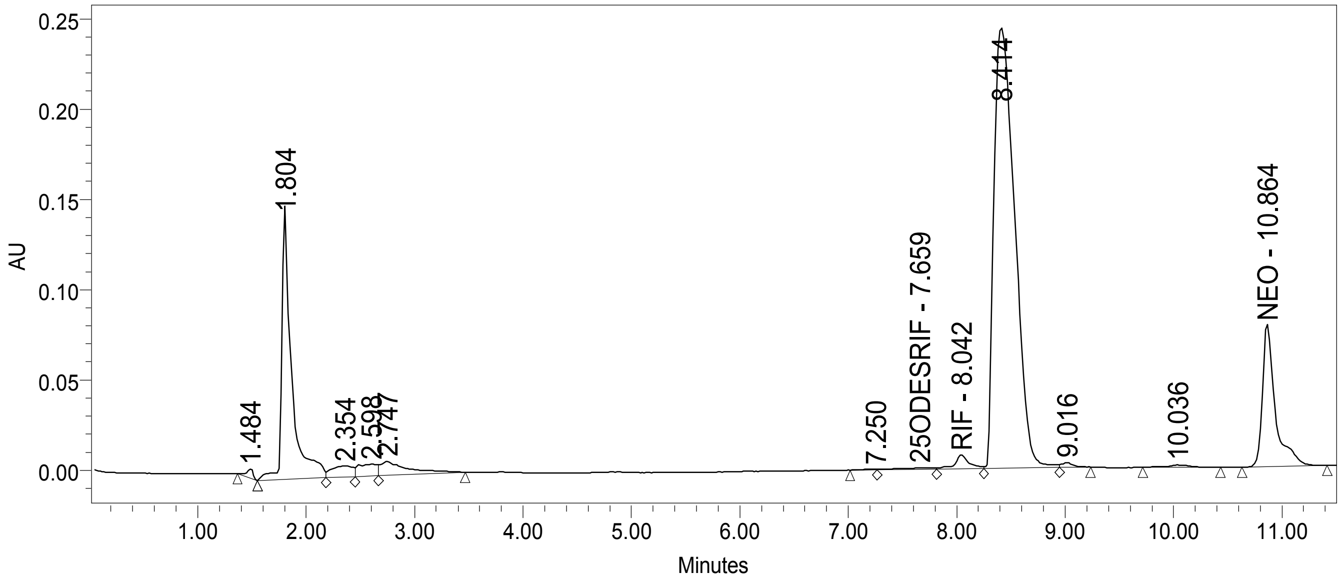

	Peak Name	RT	Area	% Area	Height
1		1.484	21528	0.41	4793
2		1.804	956229	18.04	147344
3		2.354	83136	1.57	6005
4		2.598	78727	1.49	6409
5		2.747	134306	2.53	7739
6		7.250	3343	0.06	332
7	25ODESRIF	7.659	18960	0.36	946
8	RIF	8.042	68024	1.28	7516
9		8.414	3247198	61.25	243914
10		9.016	17815	0.34	2582
11		10.036	20705	0.39	1342
12	NEO	10.864	651351	12.29	78387

Reported by User: Saneesh (Saneesh) Project Name: HDI_Stellenbosch_2016

SAMPLE INFORMATION

Sample Name:	RIF Inc Basil TDI Test Contrl K2	Acquired By:	Saneesh
Sample Type:	Unknown	Date Acquired:	2017/09/12 07:01:21 AM
Vial:	24	Acq. Method Set:	HDI_RIF_25ODESRIF_NEO
Injection #:	1	Date Processed:	2017/09/12 11:49:45 AM
Injection Volume:	15.00 ul	Processing Method:	HDI_RIF_ W2996 254.0nm-1.2
Run Time:	11.5 Minutes	Channel Name:	W2996 254.0nm-1.2
Sample Set Name:	RIF_Basil EOH TDI IC50 12_9_17	Proc. Chnl. Descr.:	W2996 PDA 254.0 nm at 1.2

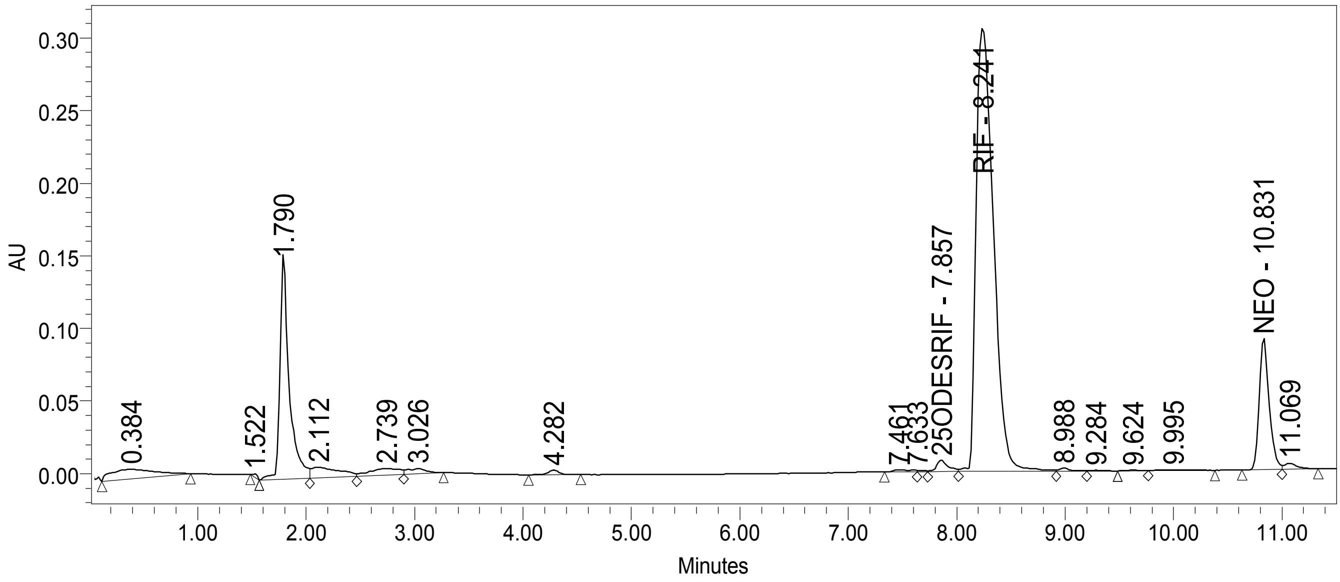

	Peak Name	RT	Area	% Area	Height
1		0.384	184157	3.34	6516
2		1.522	7356	0.13	2412
3		1.790	911488	16.54	149654
4		2.112	125145	2.27	7114
5		2.739	84442	1.53	4278
6		3.026	41627	0.76	3631
7		4.282	24006	0.44	3066
8		7.461	13723	0.25	1222
9		7.633	3632	0.07	856
10	25ODESRIF	7.857	57001	1.03	7746
11	RIF	8.241	3415540	61.98	306543
12		8.988	14674	0.27	2133

Reported by User: Saneesh (Saneesh) Project Name: HDI_Stellenbosch_2016

	Peak Name	RT	Area	% Area	Height
13		9.284	4246	0.08	542
14		9.624	3938	0.07	482
15		9.995	12841	0.23	673
16	NEO	10.831	572795	10.39	90182
17		11.069	33716	0.61	4026

SAMPLE INFORMATION

Sample Name:	RIF Inc Basil_EtOH 1ug/mL TDI1	Acquired By:	Saneesh
Sample Type:	Unknown	Date Acquired:	2017/09/12 07:17:01 AM
Vial:	2	Acq. Method Set:	HDI_RIF_25ODESRIF_NEO
Injection #:	1	Date Processed:	2017/09/12 11:49:45 AM
Injection Volume:	15.00 ul	Processing Method:	HDI_RIF_ W2996 254.0nm-1.2
Run Time:	11.5 Minutes	Channel Name:	W2996 254.0nm-1.2
Sample Set Name:	RIF_Basil EOH TDI IC50 12_9_17	Proc. Chnl. Descr.:	W2996 PDA 254.0 nm at 1.2

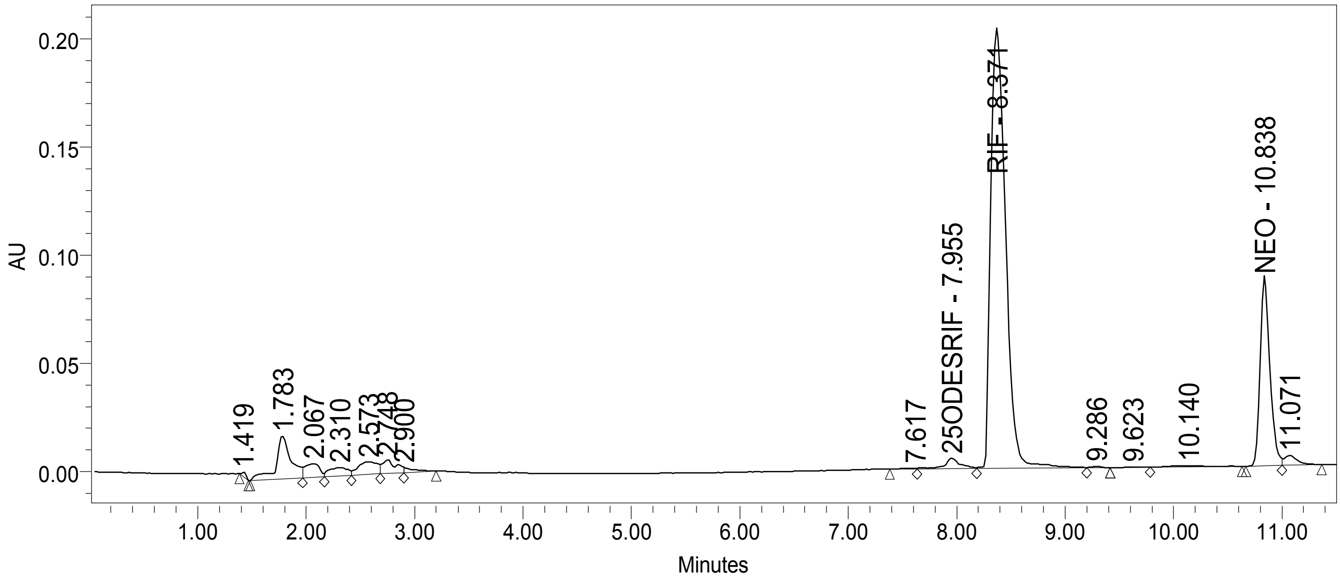

	Peak Name	RT	Area	% Area	Height
1		1.419	6312	0.21	2057
2		1.783	186782	6.16	19599
3		2.067	57700	1.90	6147
4		2.310	45548	1.50	3756
5		2.573	72105	2.38	5783
6		2.748	54763	1.81	6194
7		2.900	15201	0.50	2423
8		7.617	3255	0.11	415
9	25ODESRIF	7.955	47887	1.58	4813
10	RIF	8.371	1935891	63.87	203862
11		9.286	4602	0.15	693
12		9.623	2592	0.09	300

Reported by User: Saneesh (Saneesh) Project Name: HDI_Stellenbosch_2016

	Peak Name	RT	Area	% Area	Height
13		10.140	12790	0.42	505
14	NEO	10.838	545896	18.01	87568
15		11.071	39477	1.30	4514

Reported by User: Saneesh (Saneesh)

Project Name: HDI_Stellenbosch_2016

SAMPLE INFORMATION

Sample Name:	RIF Inc Basil_EtOH 1ug/mL TDI2	Acquired By:	Saneesh
Sample Type:	Unknown	Date Acquired:	2017/09/12 07:31:45 AM
Vial:	3	Acq. Method Set:	HDI_RIF_25ODESRIF_NEO
Injection #:	1	Date Processed:	2017/09/12 11:49:45 AM
Injection Volume:	15.00 ul	Processing Method:	HDI_RIF_ W2996 254.0nm-1.2
Run Time:	11.5 Minutes	Channel Name:	W2996 254.0nm-1.2
Sample Set Name:	RIF_Basil EOH TDI IC50 12_9_17	Proc. Chnl. Descr.:	W2996 PDA 254.0 nm at 1.2

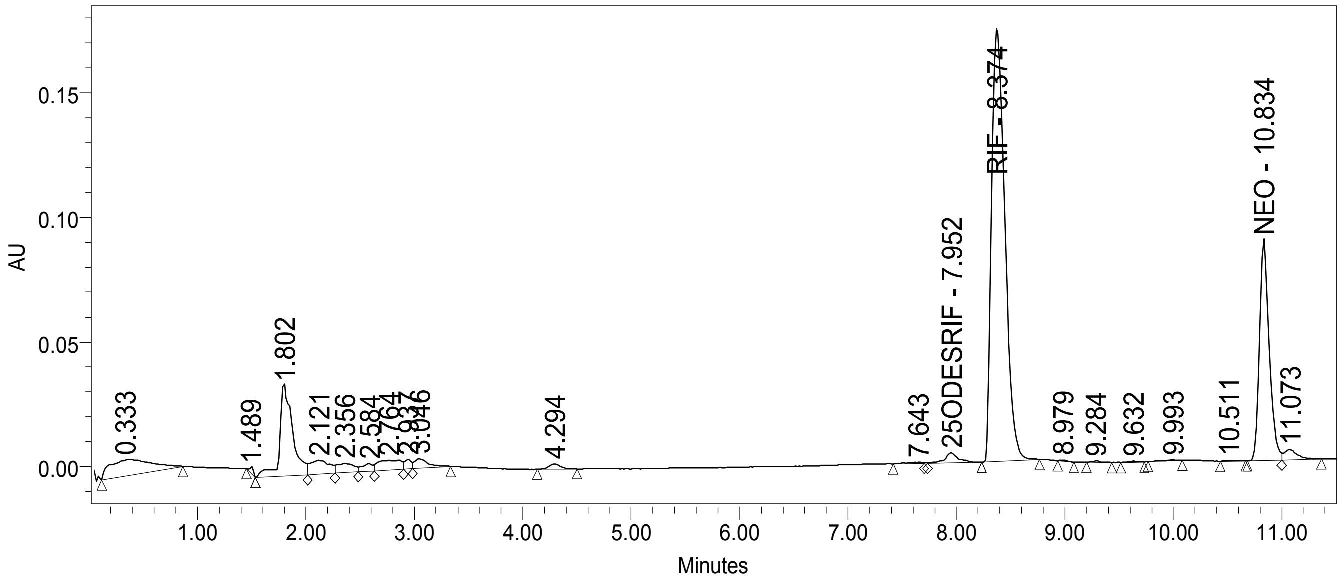

	Peak Name	RT	Area	% Area	Height
1		0.333	173774	6.07	6534
2		1.489	7036	0.25	2336
3		1.802	308540	10.78	36488
4		2.121	67992	2.38	5401
5		2.356	40030	1.40	3739
6		2.584	21925	0.77	2975
7		2.764	56186	1.96	3859
8		2.937	16299	0.57	3698
9		3.046	33980	1.19	3802
10		4.294	15667	0.55	2096
11		7.643	4909	0.17	420
12	25ODESRIF	7.952	45250	1.23	4090

Reported by User: Saneesh (Saneesh) Project Name: HDI_Stellenbosch_2016

	Peak Name	RT	Area	% Area	Height
13	RIF	8.374	1480193	51.74	174958
14		8.979	2009	0.07	448
15		9.284	3390	0.12	556
16		9.632	2660	0.09	371
17		9.993	3973	0.14	487
18		10.511	1423	0.05	215
19	NEO	10.834	550741	19.25	88253
20		11.073	35031	1.22	4075

Reported by User: Saneesh (Saneesh) Project Name: HDI_Stellenbosch_2016

SAMPLE INFORMATION

Sample Name:	RIF Inc Basil_EtOH 10ug/mL TDI1	Acquired By:	Saneesh
Sample Type:	Unknown	Date Acquired:	2017/09/12 07:46:27 AM
Vial:	4	Acq. Method Set:	HDI_RIF_25ODESRIF_NEO
Injection #:	1	Date Processed:	2017/09/12 11:49:45 AM
Injection Volume:	15.00 ul	Processing Method:	HDI_RIF_ W2996 254.0nm-1.2
Run Time:	11.5 Minutes	Channel Name:	W2996 254.0nm-1.2
Sample Set Name:	RIF_Basil EOH TDI IC50 12_9_17	Proc. Chnl. Descr.:	W2996 PDA 254.0 nm at 1.2

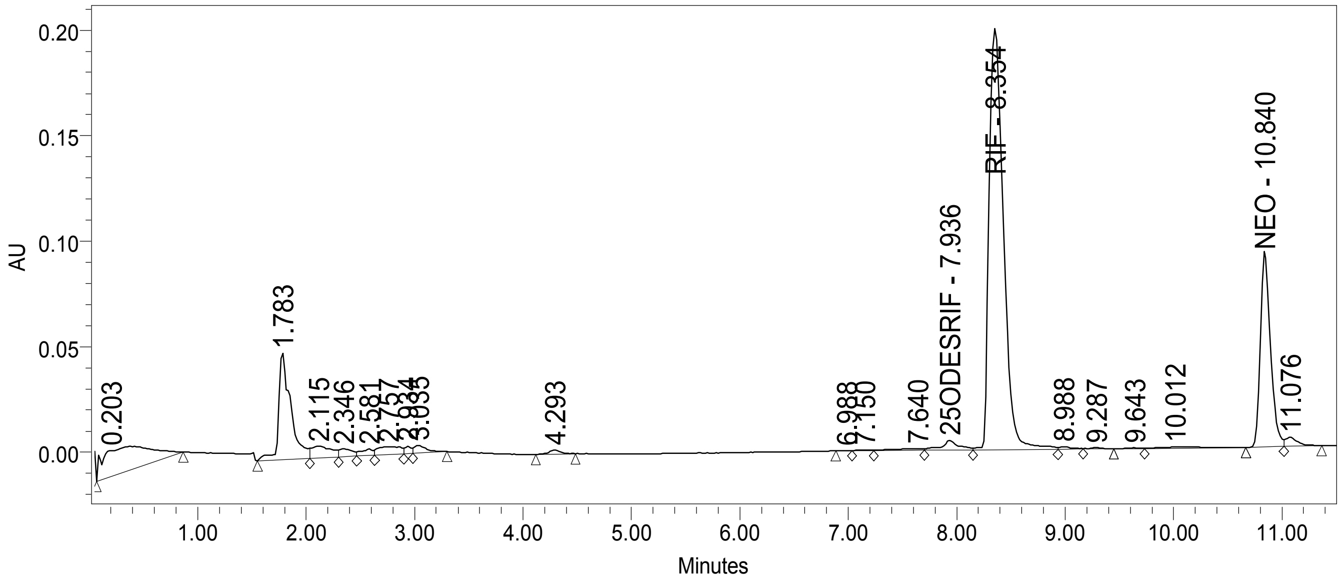

	Peak Name	RT	Area	% Area	Height
1		0.203	360673	10.13	12194
2		1.783	396164	11.13	49796
3		2.115	73714	2.07	5622
4		2.346	31425	0.88	3748
5		2.581	24162	0.68	3056
6		2.757	54156	1.52	3704
7		2.934	14943	0.42	3447
8		3.035	29307	0.82	3718
9		4.293	13774	0.39	1866
10		6.988	1609	0.05	230
11		7.150	3919	0.11	381
12		7.640	16201	0.46	836

Reported by User: Saneesh (Saneesh) Project Name: HDI_Stellenbosch_2016

	Peak Name	RT	Area	% Area	Height
13	25ODESRIF	7.936	49892	1.40	4600
14	RIF	8.354	1824009	51.24	199822
15		8.988	9074	0.25	1192
16		9.287	4580	0.13	589
17		9.643	3045	0.09	343
18		10.012	15096	0.42	628
19	NEO	10.840	598098	16.80	92872
20		11.076	35766	1.00	4299

SAMPLE INFORMATION

Sample Name:	RIF Inc Basil_EtOH 10ug/mL TDI2	Acquired By:	Saneesh
Sample Type:	Unknown	Date Acquired:	2017/09/12 08:01:12 AM
Vial:	5	Acq. Method Set:	HDI_RIF_25ODESRIF_NEO
Injection #:	1	Date Processed:	2017/09/12 11:49:45 AM
Injection Volume:	15.00 ul	Processing Method:	HDI_RIF_ W2996 254.0nm-1.2
Run Time:	11.5 Minutes	Channel Name:	W2996 254.0nm-1.2
Sample Set Name:	RIF_Basil EOH TDI IC50 12_9_17	Proc. Chnl. Descr.:	W2996 PDA 254.0 nm at 1.2

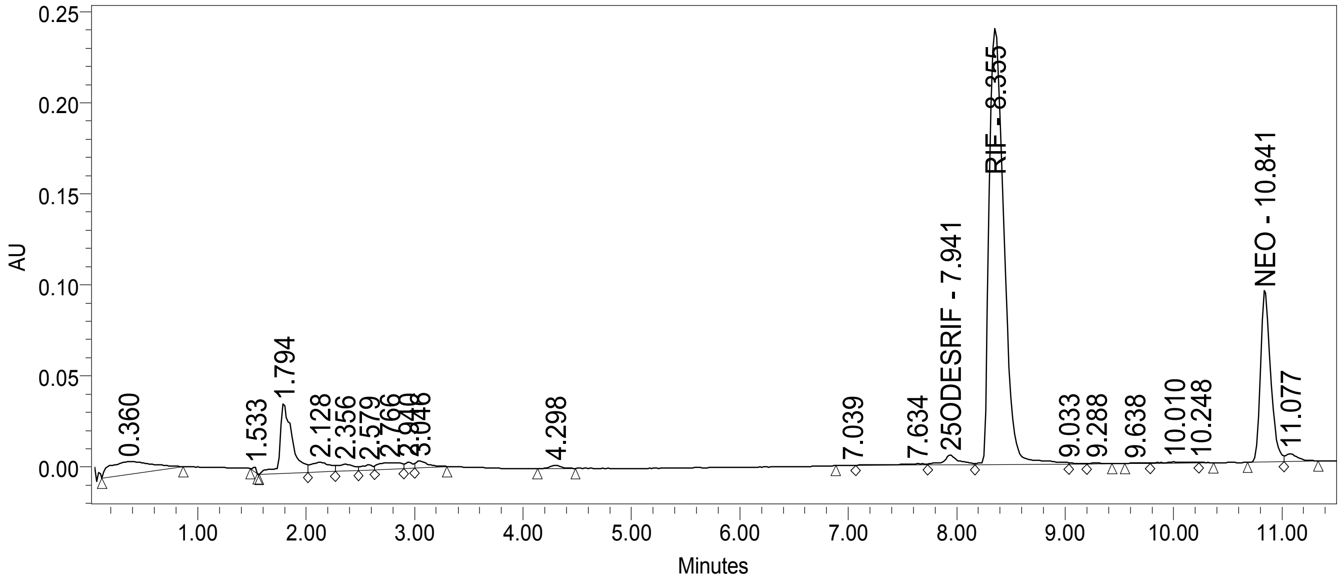

	Peak Name	RT	Area	% Area	Height
1		0.360	187045	5.02	6892
2		1.533	5518	0.15	2813
3		1.794	306928	8.23	38887
4		2.128	66109	1.77	5297
5		2.356	39703	1.06	3717
6		2.579	20344	0.55	2837
7		2.766	49594	1.33	3437
8		2.940	16917	0.45	3301
9		3.046	28081	0.75	3593
10		4.298	12643	0.34	1798
11		7.039	1662	0.04	219
12		7.634	18298	0.49	807

Reported by User: Saneesh (Saneesh) Project Name: HDI_Stellenbosch_2016

	Peak Name	RT	Area	% Area	Height
13	25ODESRIF	7.941	53341	1.43	5299
14	RIF	8.355	2258203	60.55	240186
15		9.033	3178	0.09	673
16		9.288	3462	0.09	542
17		9.638	1629	0.04	258
18		10.010	10067	0.27	605
19		10.248	1274	0.03	301
20	NEO	10.841	611191	16.39	94977
21		11.077	34339	0.92	4226

Reported by User: Saneesh (Saneesh)   Project Name: HDI_Stellenbosch_2016

SAMPLE INFORMATION

Sample Name:	RIF Inc Basil_EtOH 50ug/mL TDI1	Acquired By:	Saneesh
Sample Type:	Unknown	Date Acquired:	2017/09/12 08:15:53 AM
Vial:	6	Acq. Method Set:	HDI_RIF_25ODESRIF_NEO
Injection #:	1	Date Processed:	2017/09/12 11:49:45 AM
Injection Volume:	15.00 ul	Processing Method:	HDI_RIF_ W2996 254.0nm-1.2
Run Time:	11.5 Minutes	Channel Name:	W2996 254.0nm-1.2
Sample Set Name:	RIF_Basil EOH TDI IC50 12_9_17	Proc. Chnl. Descr.:	W2996 PDA 254.0 nm at 1.2

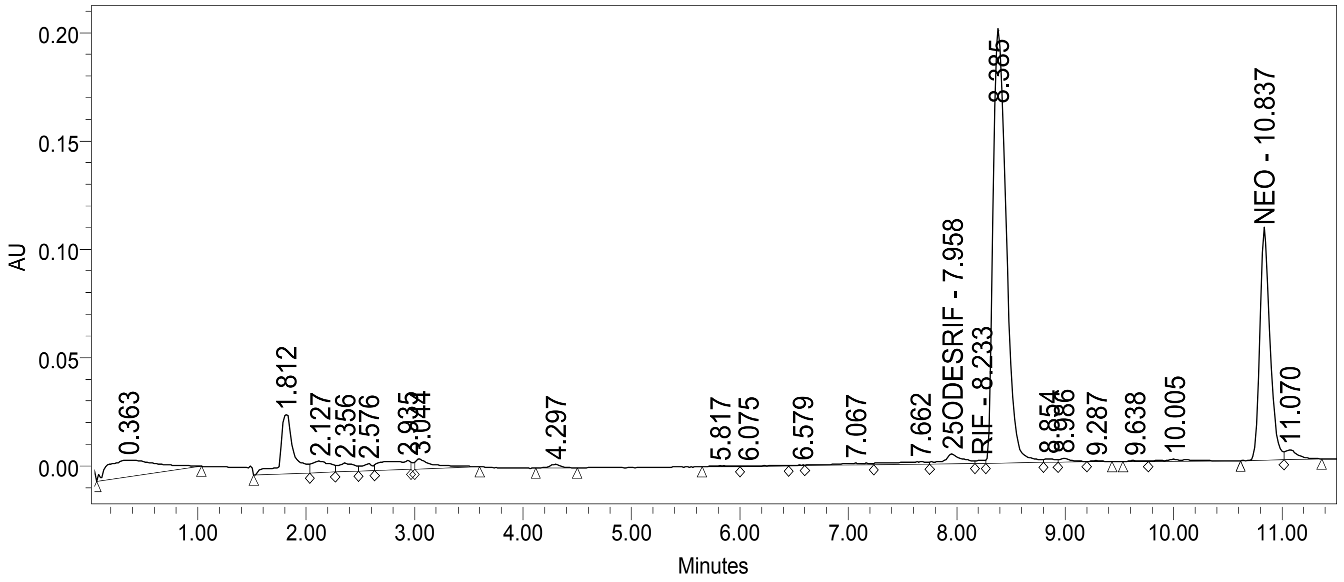

	Peak Name	RT	Area	% Area	Height
1		0.363	247628	7.39	7664
2		1.812	239013	7.13	28169
3		2.127	62386	1.86	5222
4		2.356	42092	1.26	3946
5		2.576	23850	0.71	3220
6		2.935	75016	2.24	4114
7		3.044	54847	1.64	4568
8		4.297	13006	0.39	1691
9		5.817	6171	0.18	446
10		6.075	4596	0.14	217
11		6.579	1545	0.05	228
12		7.067	20231	0.60	803

Reported by User: Saneesh (Saneesh) Project Name: HDI_Stellenbosch_2016

	Peak Name	RT	Area	% Area	Height
13		7.662	27849	0.83	1057
14	25ODESRIF	7.958	50582	1.51	4450
15	RIF	8.233	8416	0.25	1573
16		8.385	1687320	50.35	200609
17		8.854	12074	0.36	1692
18		8.986	14100	0.42	1761
19		9.287	3663	0.11	516
20		9.638	3135	0.09	375
21		10.005	17800	0.53	948
22	NEO	10.837	698905	20.86	107044
23		11.070	36953	1.10	4476

SAMPLE INFORMATION

Sample Name:	RIF Inc Basil_EtOH 50ug/mL TDI2	Acquired By:	Saneesh
Sample Type:	Unknown	Date Acquired:	2017/09/12 08:30:36 AM
Vial:	7	Acq. Method Set:	HDI_RIF_25ODESRIF_NEO
Injection #:	1	Date Processed:	2017/09/12 11:49:45 AM
Injection Volume:	15.00 ul	Processing Method:	HDI_RIF_ W2996 254.0nm-1.2
Run Time:	11.5 Minutes	Channel Name:	W2996 254.0nm-1.2
Sample Set Name:	RIF_Basil EOH TDI IC50 12_9_17	Proc. Chnl. Descr.:	W2996 PDA 254.0 nm at 1.2

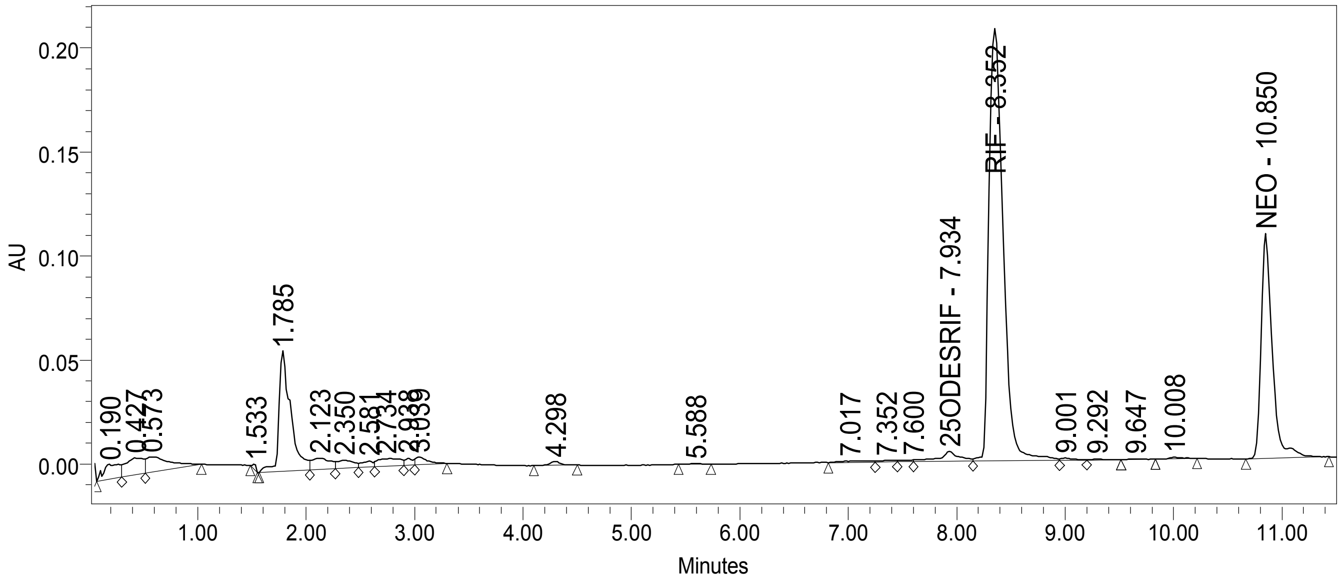

	Peak Name	RT	Area	% Area	Height
1		0.190	73031	1.96	6966
2		0.427	93203	2.50	8060
3		0.573	110088	2.95	7271
4		1.533	5779	0.15	2652
5		1.785	436692	11.69	57097
6		2.123	65127	1.74	5526
7		2.350	41574	1.11	3855
8		2.581	21675	0.58	2952
9		2.734	51955	1.39	3632
10		2.938	16900	0.45	3268
11		3.039	26767	0.72	3653
12		4.298	14058	0.38	1860

	Peak Name	RT	Area	% Area	Height
13		5.588	3067	0.08	325
14		7.017	12632	0.34	657
15		7.352	7874	0.21	705
16		7.600	7275	0.19	953
17	25ODESRIF	7.934	62868	1.68	4698
18	RIF	8.352	1887717	50.54	207991
19		9.001	9113	0.24	1107
20		9.292	4313	0.12	485
21		9.647	2193	0.06	269
22		10.008	6771	0.18	773
23	NEO	10.850	774298	20.73	107494

Reported by User: Saneesh (Saneesh) Project Name: HDI_Stellenbosch_2016

SAMPLE INFORMATION

Sample Name:	RIF Inc Basil_EtOH 100ug/mL TDI1	Acquired By:	Saneesh
Sample Type:	Unknown	Date Acquired:	2017/09/12 08:45:19 AM
Vial:	8	Acq. Method Set:	HDI_RIF_25ODESRIF_NEO
Injection #:	1	Date Processed:	2017/09/12 11:49:45 AM
Injection Volume:	15.00 ul	Processing Method:	HDI_RIF_ W2996 254.0nm-1.2
Run Time:	11.5 Minutes	Channel Name:	W2996 254.0nm-1.2
Sample Set Name:	RIF_Basil EOH TDI IC50 12_9_17	Proc. Chnl. Descr.:	W2996 PDA 254.0 nm at 1.2

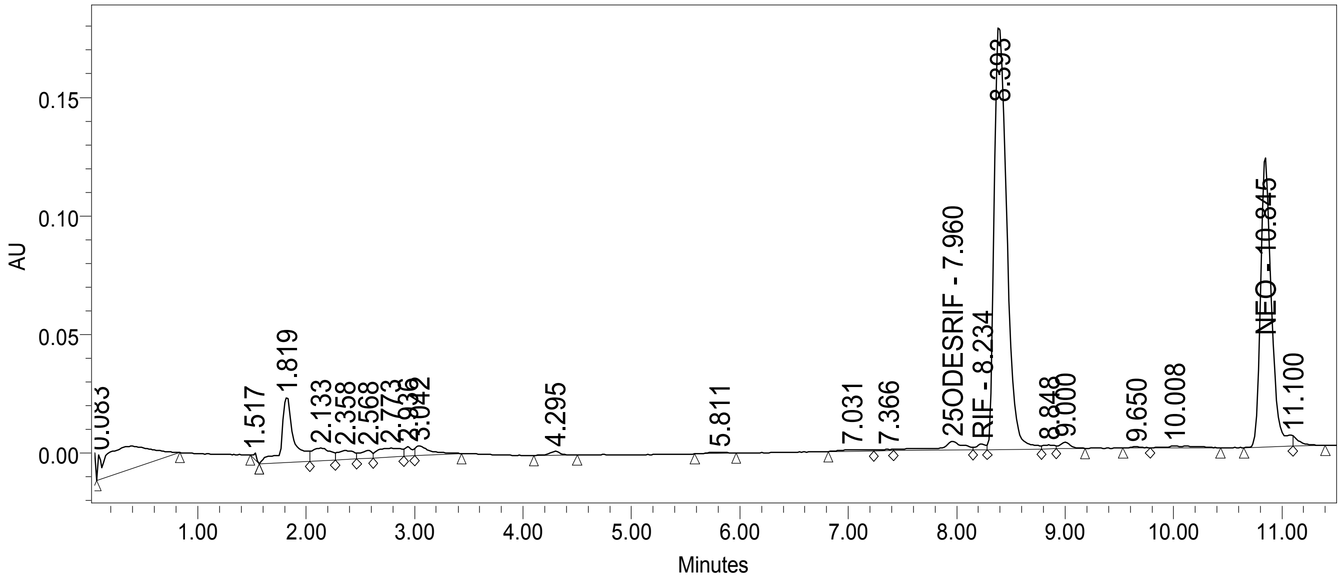

	Peak Name	RT	Area	% Area	Height
1		0.083	297357	9.19	10273
2		1.517	5891	0.18	1949
3		1.819	218519	6.75	27842
4		2.133	61311	1.89	5232
5		2.358	38952	1.20	3800
6		2.568	24140	0.75	3225
7		2.773	57124	1.77	3712
8		2.936	19620	0.61	3845
9		3.042	39191	1.21	4147
10		4.295	12917	0.40	1634
11		5.811	6908	0.21	588
12		7.031	10232	0.32	637

Reported by User: Saneesh (Saneesh) Project Name: HDI_Stellenbosch_2016

	Peak Name	RT	Area	% Area	Height
13		7.366	5671	0.18	567
14	25ODESRIF	7.960	57023	1.76	3653
15	RIF	8.234	14019	0.43	2326
16		8.393	1444789	44.64	180187
17		8.848	11664	0.36	1671
18		9.000	18566	0.57	2754
19		9.650	4093	0.13	562
20		10.008	16971	0.52	837
21	NEO	10.845	851184	26.30	121989
22		11.100	20235	0.63	4350

Reported by User: Saneesh (Saneesh)   Project Name: HDI_Stellenbosch_2016

SAMPLE INFORMATION

Sample Name:	RIF Inc Basil_EtOH 100ug/mL TDI2	Acquired By:	Saneesh
Sample Type:	Unknown	Date Acquired:	2017/09/12 09:00:03 AM
Vial:	9	Acq. Method Set:	HDI_RIF_25ODESRIF_NEO
Injection #:	1	Date Processed:	2017/09/12 11:49:45 AM
Injection Volume:	15.00 ul	Processing Method:	HDI_RIF_ W2996 254.0nm-1.2
Run Time:	11.5 Minutes	Channel Name:	W2996 254.0nm-1.2
Sample Set Name:	RIF_Basil EOH TDI IC50 12_9_17	Proc. Chnl. Descr.:	W2996 PDA 254.0 nm at 1.2

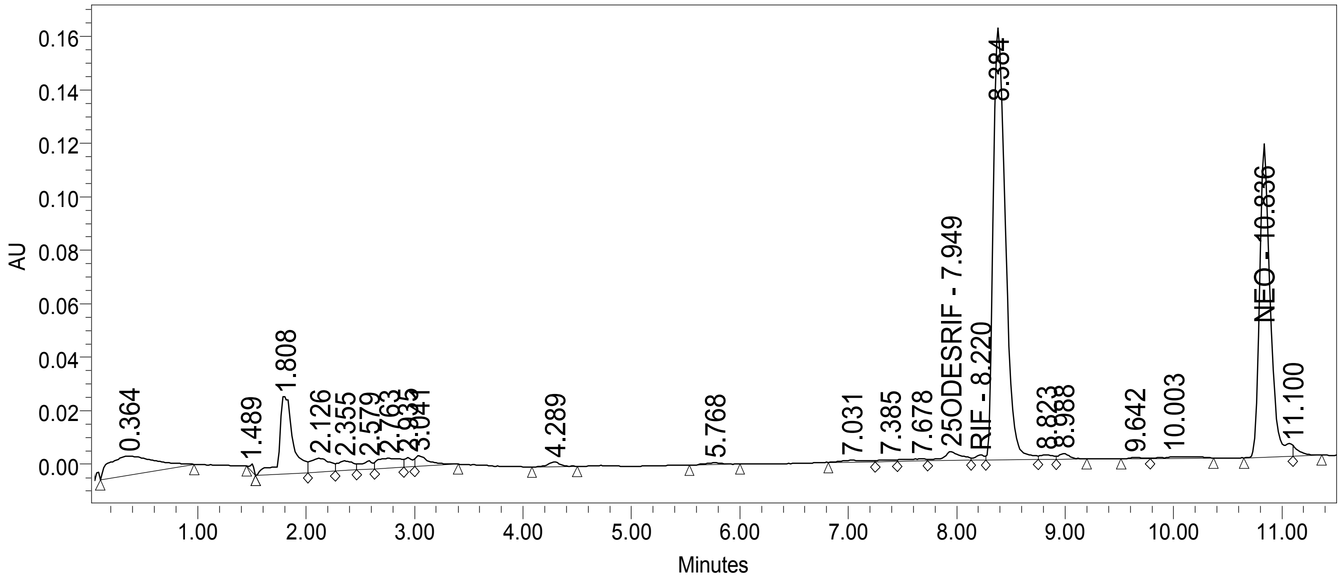

	Peak Name	RT	Area	% Area	Height
1		0.364	210643	7.15	7096
2		1.489	7342	0.25	2439
3		1.808	259246	8.80	29429
4		2.126	64080	2.18	5106
5		2.355	36893	1.25	3670
6		2.579	25078	0.85	3066
7		2.763	54037	1.84	3680
8		2.935	18547	0.63	3592
9		3.041	35603	1.21	3977
10		4.289	13300	0.45	1760
11		5.768	8712	0.30	735
12		7.031	10338	0.35	644

Reported by User: Saneesh (Saneesh) Project Name: HDI_Stellenbosch_2016

	Peak Name	RT	Area	% Area	Height
13		7.385	6304	0.21	586
14		7.678	12233	0.42	839
15	25ODESRIF	7.949	38857	1.32	3418
16	RIF	8.220	11705	0.40	1974
17		8.384	1268477	43.08	161516
18		8.823	14674	0.50	1714
19		8.988	14435	0.49	2065
20		9.642	3456	0.12	507
21		10.003	12772	0.43	600
22	NEO	10.836	801147	27.21	116431
23		11.100	16468	0.56	3997

SAMPLE INFORMATION

Sample Name:	RIF Inc Basil_EtOH 150ug/mL TDI1	Acquired By:	Saneesh
Sample Type:	Unknown	Date Acquired:	2017/09/12 09:14:45 AM
Vial:	10	Acq. Method Set:	HDI_RIF_25ODESRIF_NEO
Injection #:	1	Date Processed:	2017/09/12 11:49:45 AM
Injection Volume:	15.00 ul	Processing Method:	HDI_RIF_ W2996 254.0nm-1.2
Run Time:	11.5 Minutes	Channel Name:	W2996 254.0nm-1.2
Sample Set Name:	RIF_Basil EOH TDI IC50 12_9_17	Proc. Chnl. Descr.:	W2996 PDA 254.0 nm at 1.2

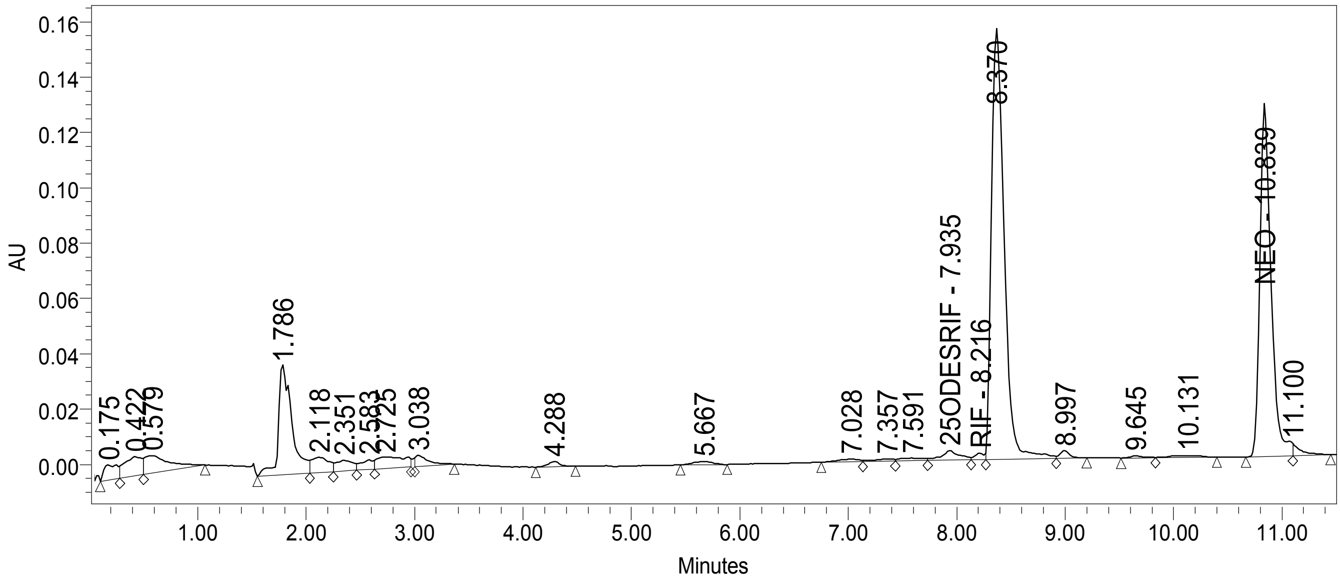

	Peak Name	RT	Area	% Area	Height
1		0.175	46619	1.48	5419
2		0.422	76997	2.44	6883
3		0.579	100344	3.18	6347
4		1.786	343944	10.89	39691
5		2.118	60964	1.93	5500
6		2.351	42743	1.35	3897
7		2.583	28919	0.92	3455
8		2.725	72469	2.30	4075
9		3.038	32358	1.02	3906
10		4.288	14914	0.47	1964
11		5.667	15140	0.48	1144
12		7.028	12937	0.41	971

Reported by User: Saneesh (Saneesh) Project Name: HDI_Stellenbosch_2016

	Peak Name	RT	Area	% Area	Height
13		7.357	10862	0.34	787
14		7.591	15095	0.48	961
15	25ODESRIF	7.935	40200	1.27	3495
16	RIF	8.216	13890	0.44	2345
17		8.370	1259493	39.90	155757
18		8.997	16471	0.52	2675
19		9.645	6144	0.19	801
20		10.131	14849	0.47	752
21	NEO	10.839	908426	28.77	127569
22		11.100	23226	0.74	4468

SAMPLE INFORMATION

Sample Name:	RIF Inc Basil_EtOH 150ug/mL TDI2	Acquired By:	Saneesh
Sample Type:	Unknown	Date Acquired:	2017/09/12 09:29:28 AM
Vial:	11	Acq. Method Set:	HDI_RIF_25ODESRIF_NEO
Injection #:	1	Date Processed:	2017/09/12 11:49:45 AM
Injection Volume:	15.00 ul	Processing Method:	HDI_RIF_ W2996 254.0nm-1.2
Run Time:	11.5 Minutes	Channel Name:	W2996 254.0nm-1.2
Sample Set Name:	RIF_Basil EOH TDI IC50 12_9_17	Proc. Chnl. Descr.:	W2996 PDA 254.0 nm at 1.2

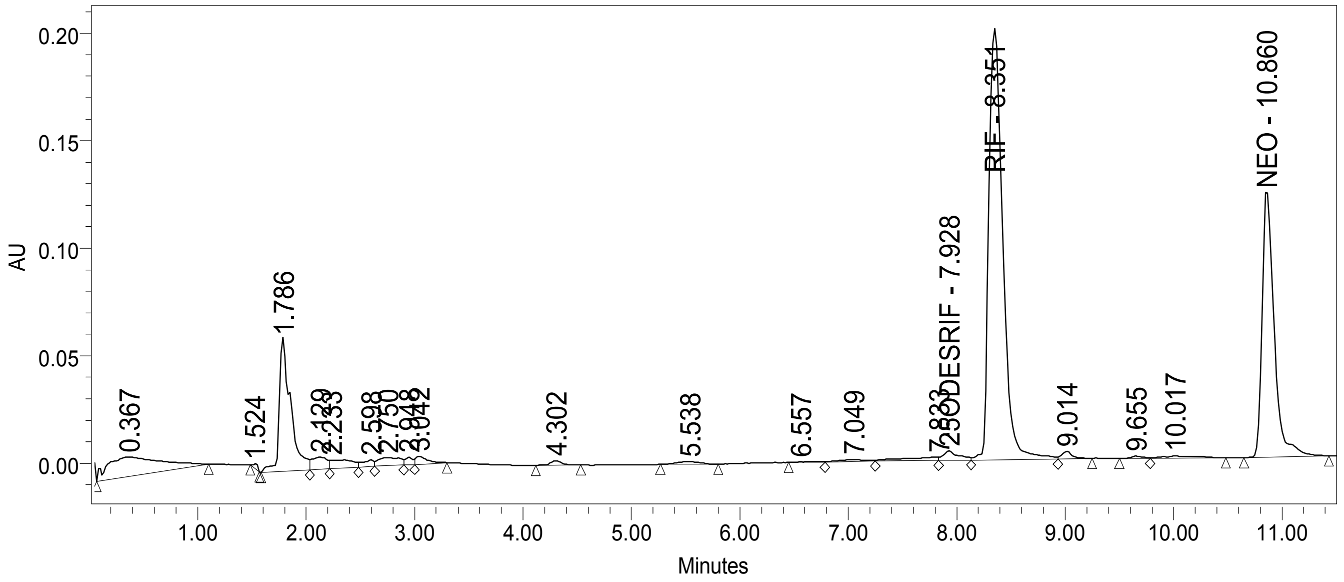

	Peak Name	RT	Area	% Area	Height
1		0.367	306965	7.83	8968
2		1.524	7084	0.18	2361
3		1.786	450631	11.49	61292
4		2.129	54865	1.40	5462
5		2.233	54092	1.38	3903
6		2.598	22625	0.58	3005
7		2.750	53067	1.35	3713
8		2.948	17176	0.44	3321
9		3.042	27834	0.71	3677
10		4.302	15780	0.40	2043
11		5.538	16421	0.42	1128
12		6.557	3198	0.08	232

Reported by User: Saneesh (Saneesh) Project Name: HDI_Stellenbosch_2016

	Peak Name	RT	Area	% Area	Height
13		7.049	17881	0.46	1028
14		7.833	37975	0.97	1451
15	25ODESRIF	7.928	42919	1.09	4251
16	RIF	8.351	1788696	45.60	200407
17		9.014	21368	0.54	3351
18		9.655	7047	0.18	1019
19		10.017	21995	0.56	959
20	NEO	10.860	955230	24.35	125045

SAMPLE INFORMATION

Sample Name:	RIF Inc Basil_EtOH 200ug/mL TDI1	Acquired By:	Saneesh
Sample Type:	Unknown	Date Acquired:	2017/09/12 09:44:12 AM
Vial:	12	Acq. Method Set:	HDI_RIF_25ODESRIF_NEO
Injection #:	1	Date Processed:	2017/09/12 11:49:45 AM
Injection Volume:	15.00 ul	Processing Method:	HDI_RIF_ W2996 254.0nm-1.2
Run Time:	11.5 Minutes	Channel Name:	W2996 254.0nm-1.2
Sample Set Name:	RIF_Basil EOH TDI IC50 12_9_17	Proc. Chnl. Descr.:	W2996 PDA 254.0 nm at 1.2

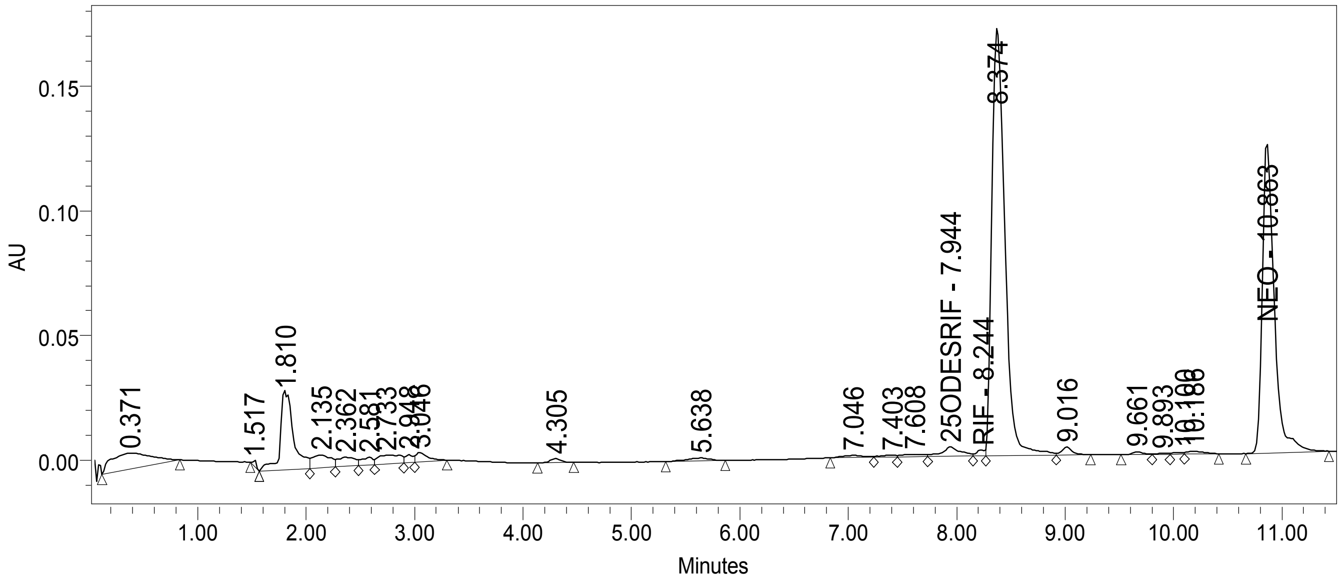

	Peak Name	RT	Area	% Area	Height
1		0.371	165931	5.18	6425
2		1.517	5881	0.18	1942
3		1.810	271967	8.49	32273
4		2.135	60523	1.89	5080
5		2.362	40241	1.26	3663
6		2.581	22421	0.70	3025
7		2.733	49090	1.53	3392
8		2.948	15863	0.50	3080
9		3.046	27930	0.87	3614
10		4.305	11786	0.37	1604
11		5.638	19416	0.61	1285
12		7.046	12894	0.40	968

Reported by User: Saneesh (Saneesh) Project Name: HDI_Stellenbosch_2016

	Peak Name	RT	Area	% Area	Height
13		7.403	8085	0.25	734
14		7.608	14716	0.46	974
15	25ODESRIF	7.944	44516	1.39	3708
16	RIF	8.244	12714	0.40	2343
17		8.374	1422557	44.43	172423
18		9.016	20151	0.63	3053
19		9.661	7412	0.23	1062
20		9.893	3096	0.10	392
21		10.100	4680	0.15	664
22		10.186	11730	0.37	1038
23	NEO	10.863	947914	29.61	123632

SAMPLE INFORMATION

Sample Name:	RIF Inc Basil_EtOH 200ug/mL TDI2	Acquired By:	Saneesh
Sample Type:	Unknown	Date Acquired:	2017/09/12 09:58:55 AM
Vial:	13	Acq. Method Set:	HDI_RIF_25ODESRIF_NEO
Injection #:	1	Date Processed:	2017/09/12 11:49:45 AM
Injection Volume:	15.00 ul	Processing Method:	HDI_RIF_ W2996 254.0nm-1.2
Run Time:	11.5 Minutes	Channel Name:	W2996 254.0nm-1.2
Sample Set Name:	RIF_Basil EOH TDI IC50 12_9_17	Proc. Chnl. Descr.:	W2996 PDA 254.0 nm at 1.2

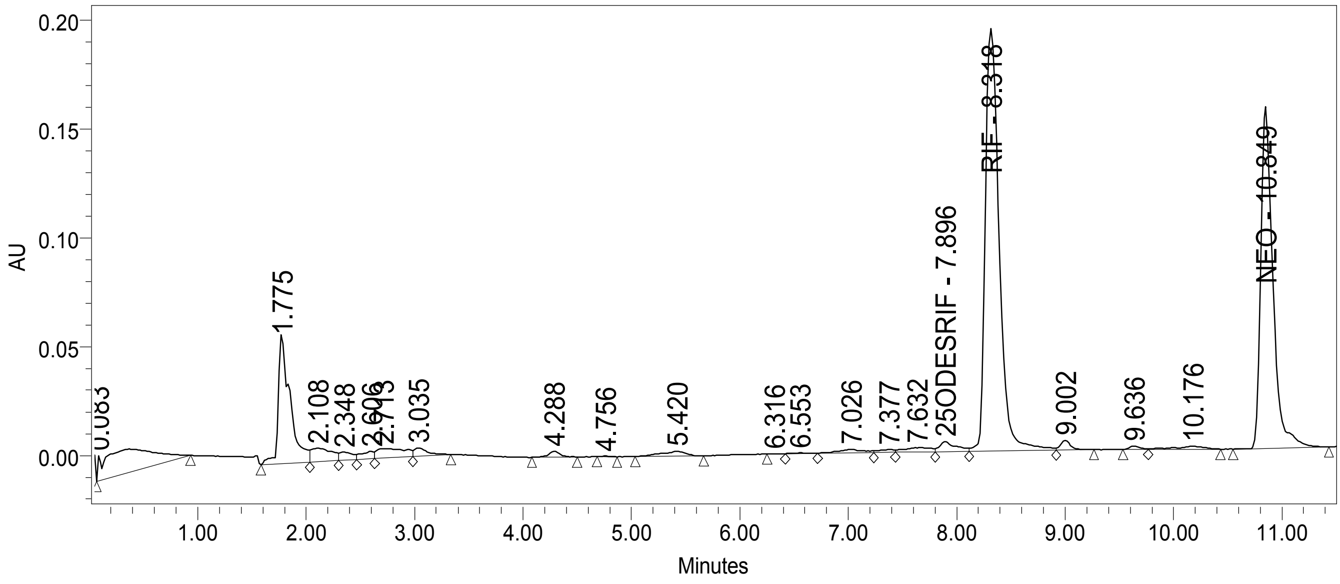

	Peak Name	RT	Area	% Area	Height
1		0.083	348757	8.23	11470
2		1.775	447350	10.55	59148
3		2.108	82474	1.95	6175
4		2.348	32163	0.76	3788
5		2.606	28323	0.67	3364
6		2.713	77106	1.82	4269
7		3.035	32489	0.77	3770
8		4.288	20260	0.48	2734
9		4.756	1904	0.04	322
10		5.420	35159	0.83	2254
11		6.316	1107	0.03	184
12		6.553	4412	0.10	356

Reported by User: Saneesh (Saneesh) Project Name: HDI_Stellenbosch_2016

	Peak Name	RT	Area	% Area	Height
13		7.026	26470	0.62	1655
14		7.377	13217	0.31	1302
15		7.632	35310	0.83	1947
16	25ODESRIF	7.896	49105	1.16	4689
17	RIF	8.318	1722903	40.63	193940
18		9.002	28329	0.67	4441
19		9.636	10271	0.24	1563
20		10.176	25914	0.61	1338
21	NEO	10.849	1217049	28.70	156606

Reported by User: Saneesh (Saneesh)   Project Name: HDI_Stellenbosch_2016

SAMPLE INFORMATION

Sample Name:	RIF Inc Basil TDI Test Contrl K1	Acquired By:	Saneesh
Sample Type:	Unknown	Date Acquired:	2017/09/12 06:46:37 AM
Vial:	23	Acq. Method Set:	HDI_RIF_25ODESRIF_NEO
Injection #:	1	Date Processed:	2017/09/12 11:49:45 AM
Injection Volume:	15.00 ul	Processing Method:	HDI_RIF_ W2996 254.0nm-1.2
Run Time:	11.5 Minutes	Channel Name:	W2996 254.0nm-1.2
Sample Set Name:	RIF_Basil EOH TDI IC50 12_9_17	Proc. Chnl. Descr.:	W2996 PDA 254.0 nm at 1.2

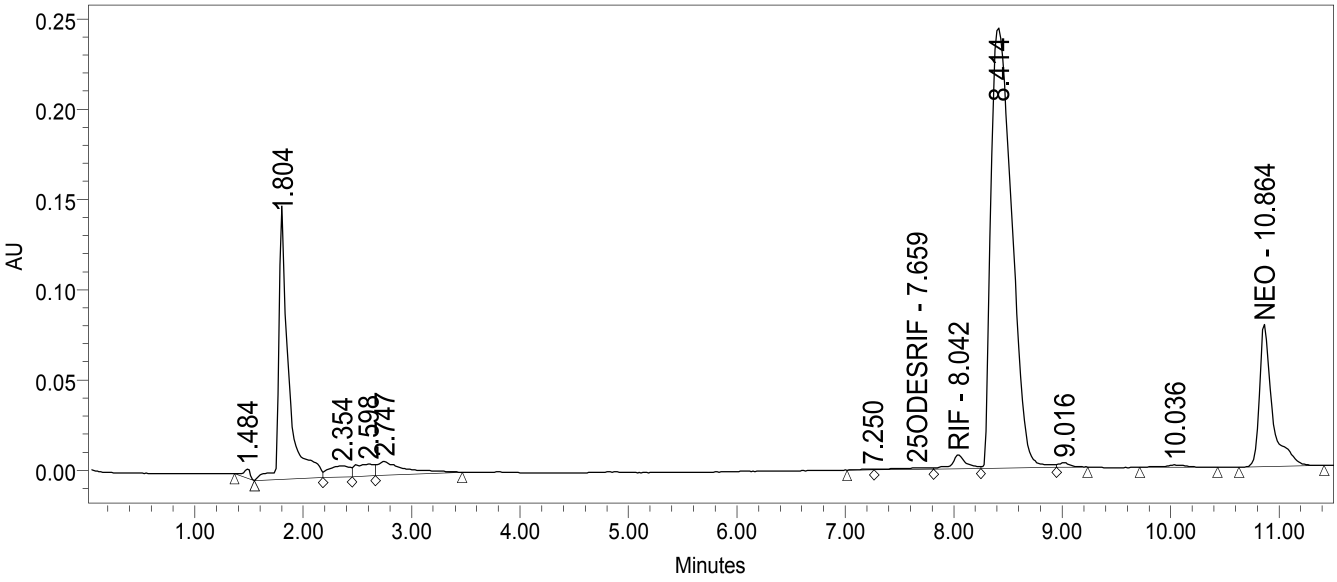

	Peak Name	RT	Area	% Area	Height
1		1.484	21528	0.41	4793
2		1.804	956229	18.04	147344
3		2.354	83136	1.57	6005
4		2.598	78727	1.49	6409
5		2.747	134306	2.53	7739
6		7.250	3343	0.06	332
7	25ODESRIF	7.659	18960	0.36	946
8	RIF	8.042	68024	1.28	7516
9		8.414	3247198	61.25	243914
10		9.016	17815	0.34	2582
11		10.036	20705	0.39	1342
12	NEO	10.864	651351	12.29	78387

SAMPLE INFORMATION

Sample Name:	RIF Inc Basil TDI Test Contrl K2	Acquired By:	Saneesh
Sample Type:	Unknown	Date Acquired:	2017/09/12 07:01:21 AM
Vial:	24	Acq. Method Set:	HDI_RIF_25ODESRIF_NEO
Injection #:	1	Date Processed:	2017/09/12 11:49:45 AM
Injection Volume:	15.00 ul	Processing Method:	HDI_RIF_ W2996 254.0nm-1.2
Run Time:	11.5 Minutes	Channel Name:	W2996 254.0nm-1.2
Sample Set Name:	RIF_Basil EOH TDI IC50 12_9_17	Proc. Chnl. Descr.:	W2996 PDA 254.0 nm at 1.2

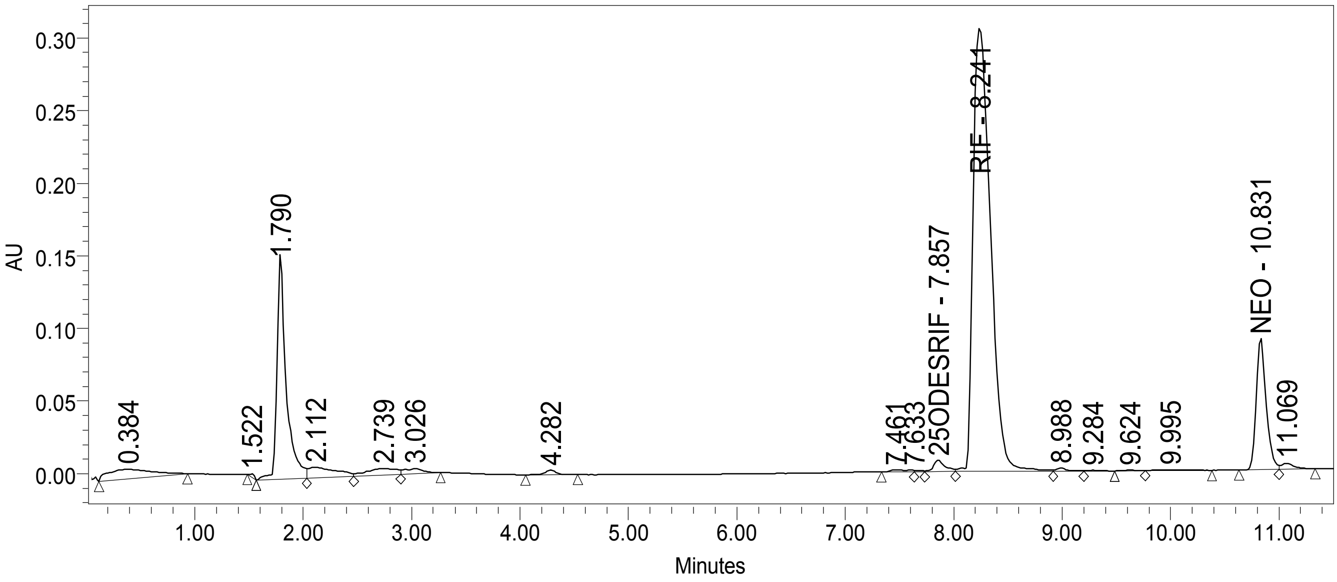

	Peak Name	RT	Area	% Area	Height
1		0.384	184157	3.34	6516
2		1.522	7356	0.13	2412
3		1.790	911488	16.54	149654
4		2.112	125145	2.27	7114
5		2.739	84442	1.53	4278
6		3.026	41627	0.76	3631
7		4.282	24006	0.44	3066
8		7.461	13723	0.25	1222
9		7.633	3632	0.07	856
10	25ODESRIF	7.857	57001	1.03	7746
11	RIF	8.241	3415540	61.98	306543
12		8.988	14674	0.27	2133

	Peak Name	RT	Area	% Area	Height
13		9.284	4246	0.08	542
14		9.624	3938	0.07	482
15		9.995	12841	0.23	673
16	NEO	10.831	572795	10.39	90182
17		11.069	33716	0.61	4026

SAMPLE INFORMATION

Sample Name:	RIF Inc Basil_EtOH 1ug/mL TDI1	Acquired By:	Saneesh
Sample Type:	Unknown	Date Acquired:	2017/09/12 07:17:01 AM
Vial:	2	Acq. Method Set:	HDI_RIF_25ODESRIF_NEO
Injection #:	1	Date Processed:	2017/09/12 11:49:45 AM
Injection Volume:	15.00 ul	Processing Method:	HDI_RIF_ W2996 254.0nm-1.2
Run Time:	11.5 Minutes	Channel Name:	W2996 254.0nm-1.2
Sample Set Name:	RIF_Basil EOH TDI IC50 12_9_17	Proc. Chnl. Descr.:	W2996 PDA 254.0 nm at 1.2

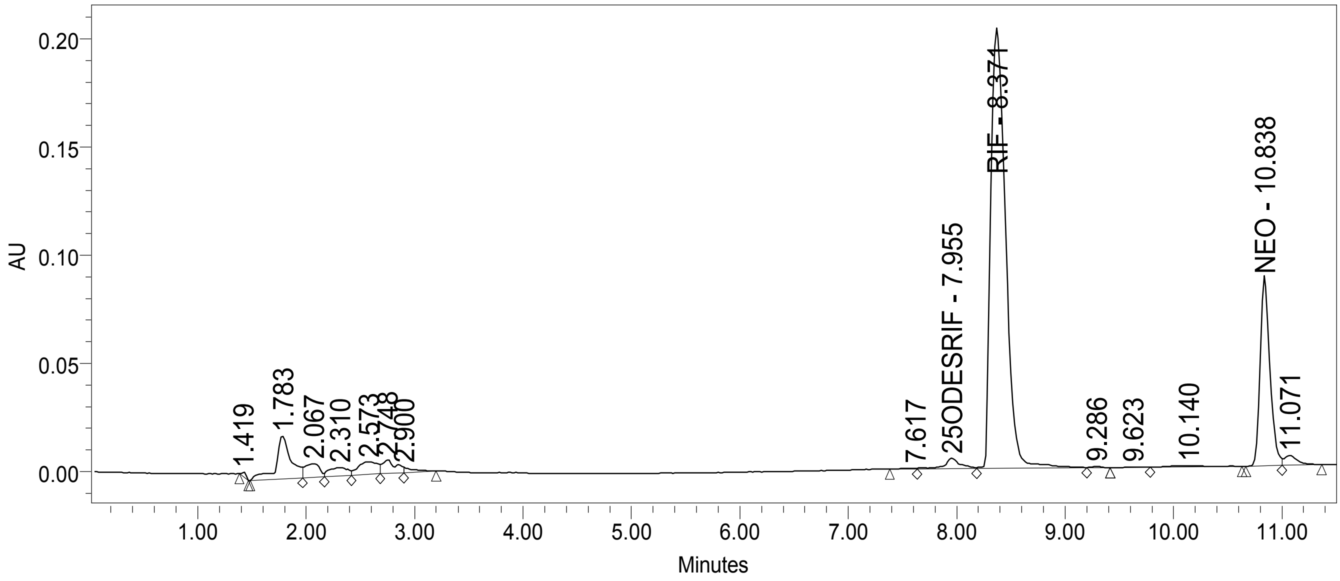

	Peak Name	RT	Area	% Area	Height
1		1.419	6312	0.21	2057
2		1.783	186782	6.16	19599
3		2.067	57700	1.90	6147
4		2.310	45548	1.50	3756
5		2.573	72105	2.38	5783
6		2.748	54763	1.81	6194
7		2.900	15201	0.50	2423
8		7.617	3255	0.11	415
9	25ODESRIF	7.955	47887	1.58	4813
10	RIF	8.371	1935891	63.87	203862
11		9.286	4602	0.15	693
12		9.623	2592	0.09	300

	Peak Name	RT	Area	% Area	Height
13		10.140	12790	0.42	505
14	NEO	10.838	545896	18.01	87568
15		11.071	39477	1.30	4514

SAMPLE INFORMATION

Sample Name:	RIF Inc Basil_EtOH 1ug/mL TDI2	Acquired By:	Saneesh
Sample Type:	Unknown	Date Acquired:	2017/09/12 07:31:45 AM
Vial:	3	Acq. Method Set:	HDI_RIF_25ODESRIF_NEO
Injection #:	1	Date Processed:	2017/09/12 11:49:45 AM
Injection Volume:	15.00 ul	Processing Method:	HDI_RIF_ W2996 254.0nm-1.2
Run Time:	11.5 Minutes	Channel Name:	W2996 254.0nm-1.2
Sample Set Name:	RIF_Basil EOH TDI IC50 12_9_17	Proc. Chnl. Descr.:	W2996 PDA 254.0 nm at 1.2

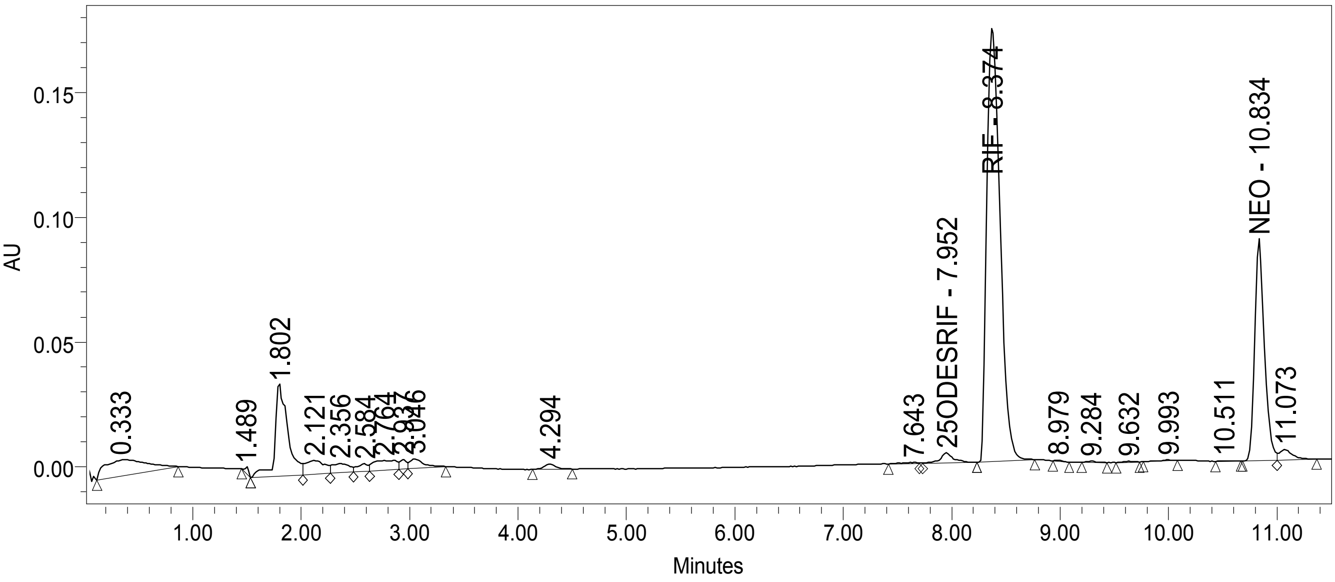

	Peak Name	RT	Area	% Area	Height
1		0.333	173774	6.07	6534
2		1.489	7036	0.25	2336
3		1.802	308540	10.78	36488
4		2.121	67992	2.38	5401
5		2.356	40030	1.40	3739
6		2.584	21925	0.77	2975
7		2.764	56186	1.96	3859
8		2.937	16299	0.57	3698
9		3.046	33980	1.19	3802
10		4.294	15667	0.55	2096
11		7.643	4909	0.17	420
12	25ODESRIF	7.952	35250	1.23	4090

Reported by User: Saneesh (Saneesh) Project Name: HDI_Stellenbosch_2016

	Peak Name	RT	Area	% Area	Height
13	RIF	8.374	1480193	51.74	174958
14		8.979	2009	0.07	448
15		9.284	3390	0.12	556
16		9.632	2660	0.09	371
17		9.993	3973	0.14	487
18		10.511	1423	0.05	215
19	NEO	10.834	550741	19.25	88253
20		11.073	35031	1.22	4075

SAMPLE INFORMATION

Sample Name:	RIF Inc Basil_EtOH 10ug/mL TDI1	Acquired By:	Saneesh
Sample Type:	Unknown	Date Acquired:	2017/09/12 07:46:27 AM
Vial:	4	Acq. Method Set:	HDI_RIF_25ODESRIF_NEO
Injection #:	1	Date Processed:	2017/09/12 11:49:45 AM
Injection Volume:	15.00 ul	Processing Method:	HDI_RIF_ W2996 254.0nm-1.2
Run Time:	11.5 Minutes	Channel Name:	W2996 254.0nm-1.2
Sample Set Name:	RIF_Basil EOH TDI IC50 12_9_17	Proc. Chnl. Descr.:	W2996 PDA 254.0 nm at 1.2

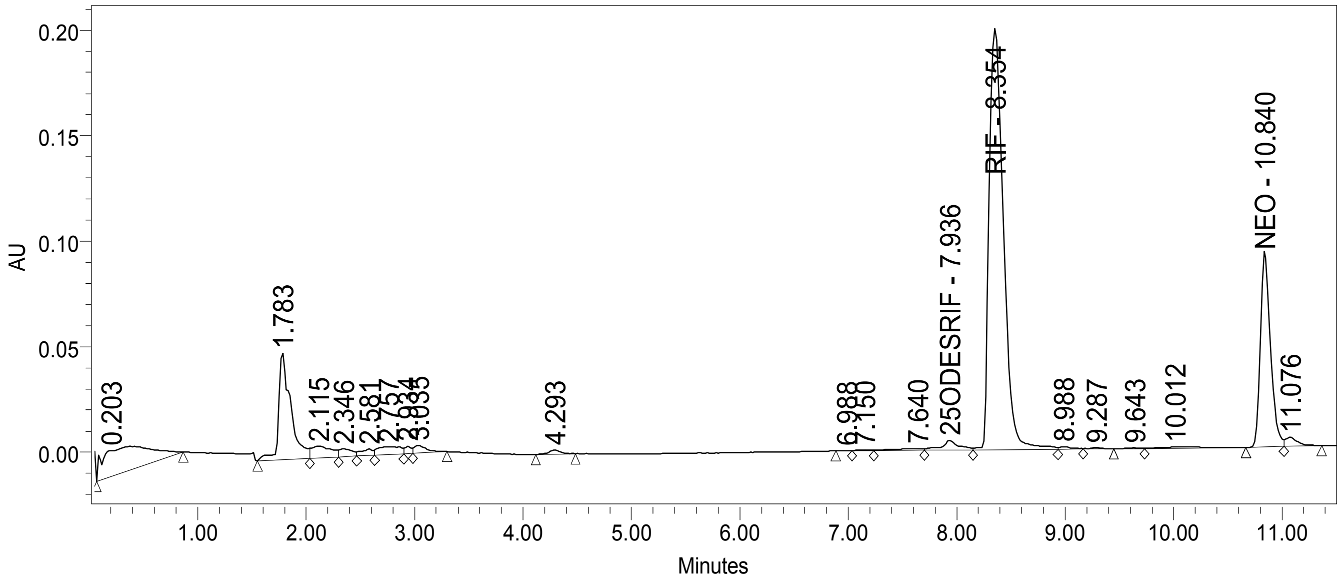

	Peak Name	RT	Area	% Area	Height
1		0.203	360673	10.13	12194
2		1.783	396164	11.13	49796
3		2.115	73714	2.07	5622
4		2.346	31425	0.88	3748
5		2.581	24162	0.68	3056
6		2.757	54156	1.52	3704
7		2.934	14943	0.42	3447
8		3.035	29307	0.82	3718
9		4.293	13774	0.39	1866
10		6.988	1609	0.05	230
11		7.150	3919	0.11	381
12		7.640	16201	0.46	836

Reported by User: Saneesh (Saneesh) Project Name: HDI_Stellenbosch_2016

	Peak Name	RT	Area	% Area	Height
13	25ODESRIF	7.936	49892	1.40	4600
14	RIF	8.354	1824009	51.24	199822
15		8.988	9074	0.25	1192
16		9.287	4580	0.13	589
17		9.643	3045	0.09	343
18		10.012	15096	0.42	628
19	NEO	10.840	598098	16.80	92872
20		11.076	35766	1.00	4299

Reported by User: Saneesh (Saneesh) Project Name: HDI_Stellenbosch_2016

SAMPLE INFORMATION

Sample Name:	RIF Inc Basil_EtOH 10ug/mL TDI2	Acquired By:	Saneesh
Sample Type:	Unknown	Date Acquired:	2017/09/12 08:01:12 AM
Vial:	5	Acq. Method Set:	HDI_RIF_25ODESRIF_NEO
Injection #:	1	Date Processed:	2017/09/12 11:49:45 AM
Injection Volume:	15.00 ul	Processing Method:	HDI_RIF_ W2996 254.0nm-1.2
Run Time:	11.5 Minutes	Channel Name:	W2996 254.0nm-1.2
Sample Set Name:	RIF_Basil EOH TDI IC50 12_9_17	Proc. Chnl. Descr.:	W2996 PDA 254.0 nm at 1.2

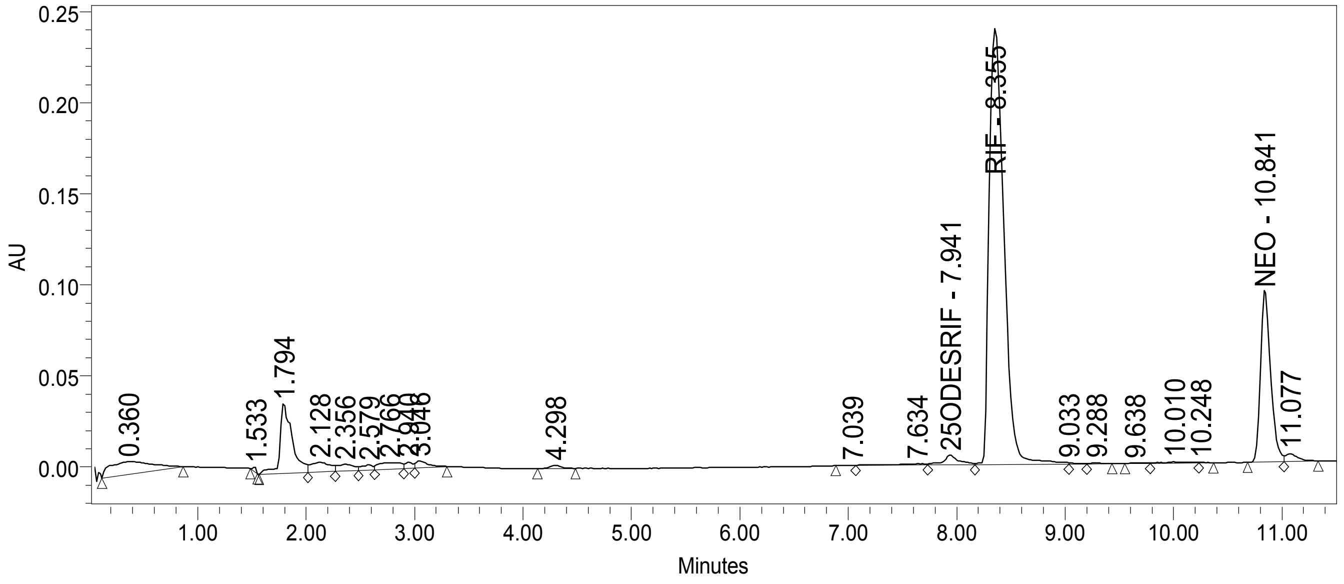

	Peak Name	RT	Area	% Area	Height
1		0.360	187045	5.02	6892
2		1.533	5518	0.15	2813
3		1.794	306928	8.23	38887
4		2.128	66109	1.77	5297
5		2.356	39703	1.06	3717
6		2.579	20344	0.55	2837
7		2.766	49594	1.33	3437
8		2.940	16917	0.45	3301
9		3.046	28081	0.75	3593
10		4.298	12643	0.34	1798
11		7.039	1662	0.04	219
12		7.634	18298	0.49	807

Reported by User: Saneesh (Saneesh) Project Name: HDI_Stellenbosch_2016

	Peak Name	RT	Area	% Area	Height
13	25ODESRIF	7.941	53341	1.43	5299
14	RIF	8.355	2258203	60.55	240186
15		9.033	3178	0.09	673
16		9.288	3462	0.09	542
17		9.638	1629	0.04	258
18		10.010	10067	0.27	605
19		10.248	1274	0.03	301
20	NEO	10.841	611191	16.39	94977
21		11.077	34339	0.92	4226

SAMPLE INFORMATION

Sample Name:	RIF Inc Basil_EtOH 50ug/mL TDI1	Acquired By:	Saneesh
Sample Type:	Unknown	Date Acquired:	2017/09/12 08:15:53 AM
Vial:	6	Acq. Method Set:	HDI_RIF_25ODESRIF_NEO
Injection #:	1	Date Processed:	2017/09/12 11:49:45 AM
Injection Volume:	15.00 ul	Processing Method:	HDI_RIF_ W2996 254.0nm-1.2
Run Time:	11.5 Minutes	Channel Name:	W2996 254.0nm-1.2
Sample Set Name:	RIF_Basil EOH TDI IC50 12_9_17	Proc. Chnl. Descr.:	W2996 PDA 254.0 nm at 1.2

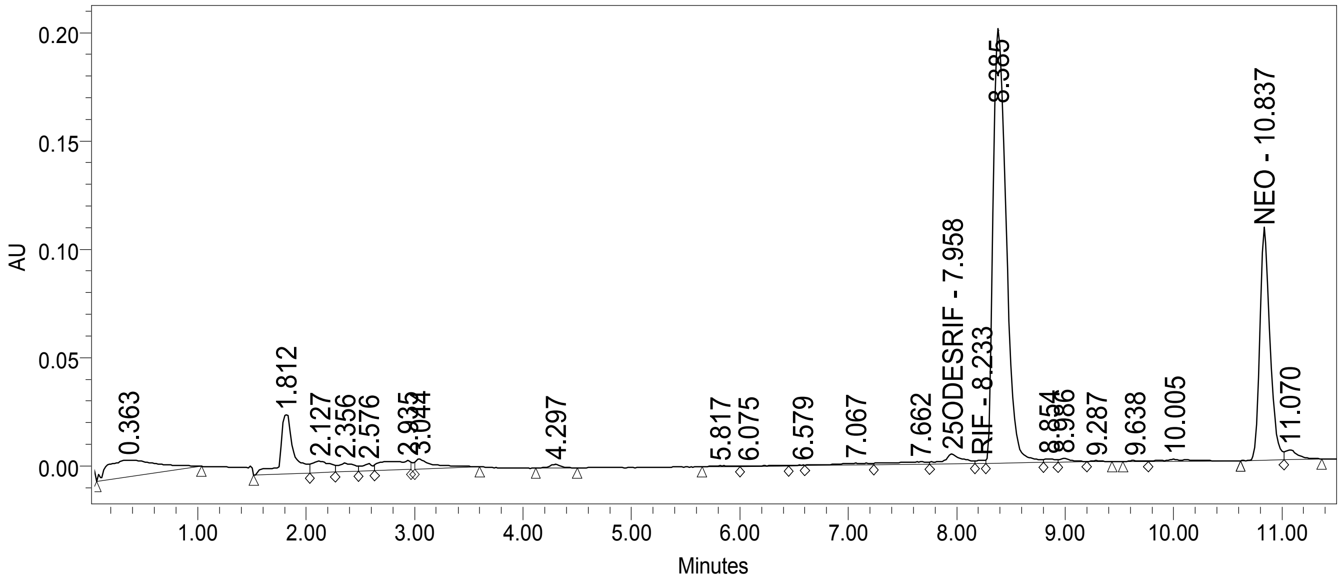

	Peak Name	RT	Area	% Area	Height
1		0.363	247628	7.39	7664
2		1.812	239013	7.13	28169
3		2.127	62386	1.86	5222
4		2.356	42092	1.26	3946
5		2.576	23850	0.71	3220
6		2.935	75016	2.24	4114
7		3.044	54847	1.64	4568
8		4.297	13006	0.39	1691
9		5.817	6171	0.18	446
10		6.075	4596	0.14	217
11		6.579	1545	0.05	228
12		7.067	20231	0.60	803

	Peak Name	RT	Area	% Area	Height
13		7.662	27849	0.83	1057
14	25ODESRIF	7.958	50582	1.51	4450
15	RIF	8.233	8416	0.25	1573
16		8.385	1687320	50.35	200609
17		8.854	12074	0.36	1692
18		8.986	14100	0.42	1761
19		9.287	3663	0.11	516
20		9.638	3135	0.09	375
21		10.005	17800	0.53	948
22	NEO	10.837	698905	20.86	107044
23		11.070	36953	1.10	4476

Multi Sample Summary

Reported by User: Saneesh (Saneesh)   Project Name: HDI_Stellenbosch_2016

SAMPLE INFORMATION

Sample Name:	RIF Inc Basil_EtOH 50ug/mL TDI2	Acquired By:	Saneesh
Sample Type:	Unknown	Date Acquired:	2017/09/12 08:30:36 AM
Vial:	7	Acq. Method Set:	HDI_RIF_25ODESRIF_NEO
Injection #:	1	Date Processed:	2017/09/12 11:49:45 AM
Injection Volume:	15.00 ul	Processing Method:	HDI_RIF_ W2996 254.0nm-1.2
Run Time:	11.5 Minutes	Channel Name:	W2996 254.0nm-1.2
Sample Set Name:	RIF_Basil EOH TDI IC50 12_9_17	Proc. Chnl. Descr.:	W2996 PDA 254.0 nm at 1.2

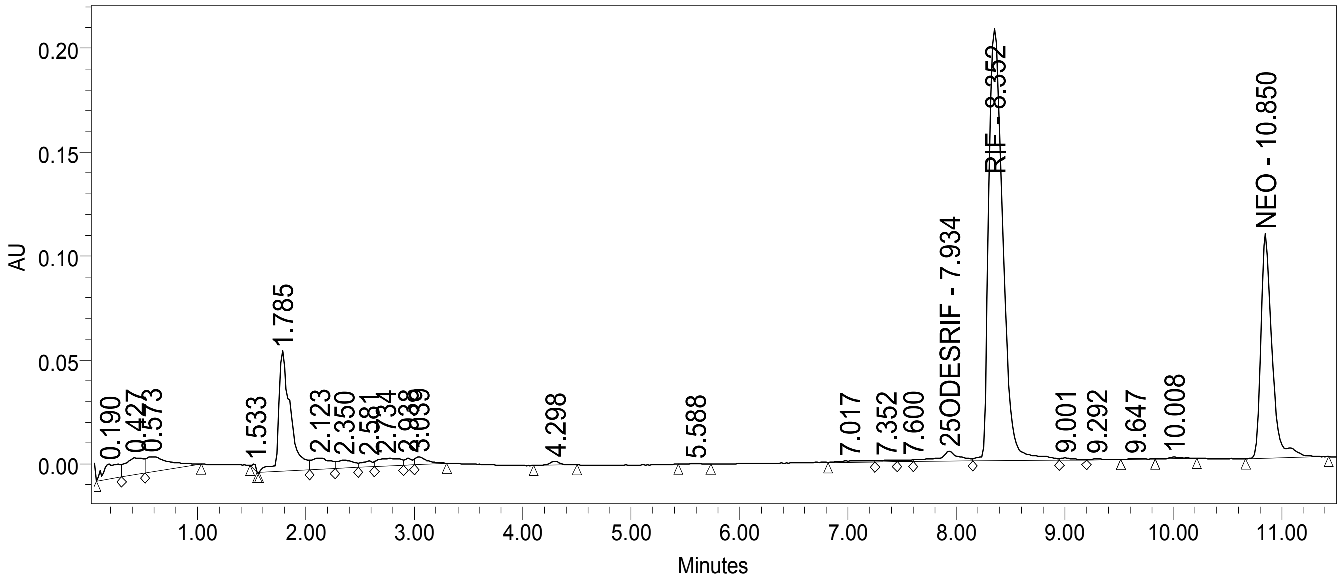

	Peak Name	RT	Area	% Area	Height
1		0.190	73031	1.96	6966
2		0.427	93203	2.50	8060
3		0.573	110088	2.95	7271
4		1.533	5779	0.15	2652
5		1.785	436692	11.69	57097
6		2.123	65127	1.74	5526
7		2.350	41574	1.11	3855
8		2.581	21675	0.58	2952
9		2.734	51955	1.39	3632
10		2.938	16900	0.45	3268
11		3.039	26767	0.72	3653
12		4.298	14058	0.38	1860

Reported by User: Saneesh (Saneesh) Project Name: HDI_Stellenbosch_2016

	Peak Name	RT	Area	% Area	Height
13		5.588	3067	0.08	325
14		7.017	12632	0.34	657
15		7.352	7874	0.21	705
16		7.600	7275	0.19	953
17	25ODESRIF	7.934	62868	1.68	4698
18	RIF	8.352	1887717	50.54	207991
19		9.001	9113	0.24	1107
20		9.292	4313	0.12	485
21		9.647	2193	0.06	269
22		10.008	6771	0.18	773
23	NEO	10.850	774298	20.73	107494

SAMPLE INFORMATION

Sample Name:	RIF Inc Basil_EtOH 100ug/mL TDI1	Acquired By:	Saneesh
Sample Type:	Unknown	Date Acquired:	2017/09/12 08:45:19 AM
Vial:	8	Acq. Method Set:	HDI_RIF_25ODESRIF_NEO
Injection #:	1	Date Processed:	2017/09/12 11:49:45 AM
Injection Volume:	15.00 ul	Processing Method:	HDI_RIF_ W2996 254.0nm-1.2
Run Time:	11.5 Minutes	Channel Name:	W2996 254.0nm-1.2
Sample Set Name:	RIF_Basil EOH TDI IC50 12_9_17	Proc. Chnl. Descr.:	W2996 PDA 254.0 nm at 1.2

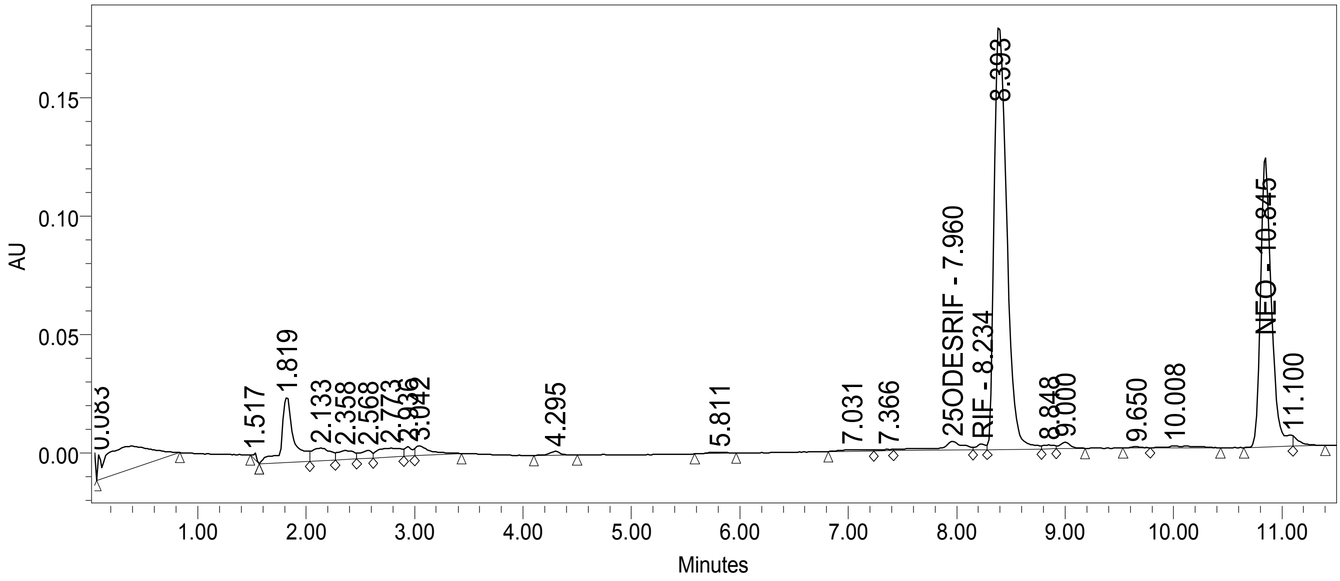

	Peak Name	RT	Area	% Area	Height
1		0.083	297357	9.19	10273
2		1.517	5891	0.18	1949
3		1.819	218519	6.75	27842
4		2.133	61311	1.89	5232
5		2.358	38952	1.20	3800
6		2.568	24140	0.75	3225
7		2.773	57124	1.77	3712
8		2.936	19620	0.61	3845
9		3.042	39191	1.21	4147
10		4.295	12917	0.40	1634
11		5.811	6908	0.21	588
12		7.031	10232	0.32	637

Reported by User: Saneesh (Saneesh) Project Name: HDI_Stellenbosch_2016

	Peak Name	RT	Area	% Area	Height
13		7.366	5671	0.18	567
14	25ODESRIF	7.960	57023	1.76	3653
15	RIF	8.234	14019	0.43	2326
16		8.393	1444789	44.64	180187
17		8.848	11664	0.36	1671
18		9.000	18566	0.57	2754
19		9.650	4093	0.13	562
20		10.008	16971	0.52	837
21	NEO	10.845	851184	26.30	121989
22		11.100	20235	0.63	4350

Reported by User: Saneesh (Saneesh)   Project Name: HDI_Stellenbosch_2016

SAMPLE INFORMATION

Sample Name:	RIF Inc Basil_EtOH 100ug/mL TDI2	Acquired By:	Saneesh
Sample Type:	Unknown	Date Acquired:	2017/09/12 09:00:03 AM
Vial:	9	Acq. Method Set:	HDI_RIF_25ODESRIF_NEO
Injection #:	1	Date Processed:	2017/09/12 11:49:45 AM
Injection Volume:	15.00 ul	Processing Method:	HDI_RIF_ W2996 254.0nm-1.2
Run Time:	11.5 Minutes	Channel Name:	W2996 254.0nm-1.2
Sample Set Name:	RIF_Basil EOH TDI IC50 12_9_17	Proc. Chnl. Descr.:	W2996 PDA 254.0 nm at 1.2

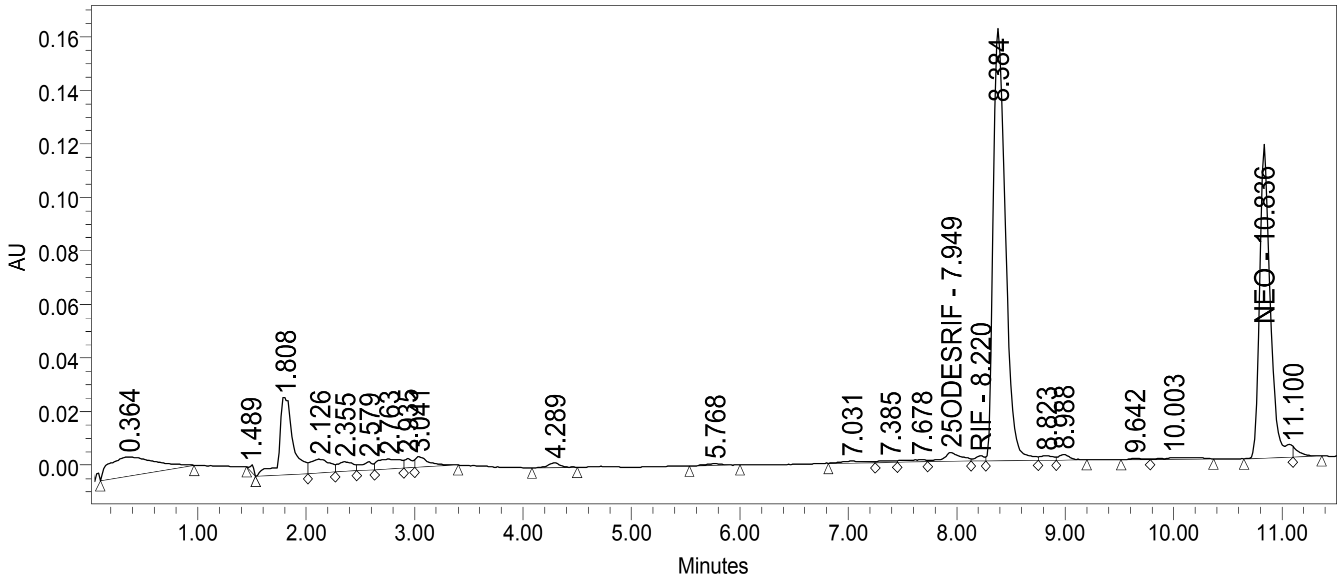

	Peak Name	RT	Area	% Area	Height
1		0.364	210643	7.15	7096
2		1.489	7342	0.25	2439
3		1.808	259246	8.80	29429
4		2.126	64080	2.18	5106
5		2.355	36893	1.25	3670
6		2.579	25078	0.85	3066
7		2.763	54037	1.84	3680
8		2.935	18547	0.63	3592
9		3.041	35603	1.21	3977
10		4.289	13300	0.45	1760
11		5.768	8712	0.30	735
12		7.031	10338	0.35	644

	Peak Name	RT	Area	% Area	Height
13		7.385	6304	0.21	586
14		7.678	12233	0.42	839
15	25ODESRIF	7.949	38857	1.32	3418
16	RIF	8.220	11705	0.40	1974
17		8.384	1268477	43.08	161516
18		8.823	14674	0.50	1714
19		8.988	14435	0.49	2065
20		9.642	3456	0.12	507
21		10.003	12772	0.43	600
22	NEO	10.836	801147	27.21	116431
23		11.100	16468	0.56	3997

Reported by User: Saneesh (Saneesh) Project Name: HDI_Stellenbosch_2016

SAMPLE INFORMATION

Sample Name:	RIF Inc Basil_EtOH 150ug/mL TDI1	Acquired By:	Saneesh
Sample Type:	Unknown	Date Acquired:	2017/09/12 09:14:45 AM
Vial:	10	Acq. Method Set:	HDI_RIF_25ODESRIF_NEO
Injection #:	1	Date Processed:	2017/09/12 11:49:45 AM
Injection Volume:	15.00 ul	Processing Method:	HDI_RIF_ W2996 254.0nm-1.2
Run Time:	11.5 Minutes	Channel Name:	W2996 254.0nm-1.2
Sample Set Name:	RIF_Basil EOH TDI IC50 12_9_17	Proc. Chnl. Descr.:	W2996 PDA 254.0 nm at 1.2

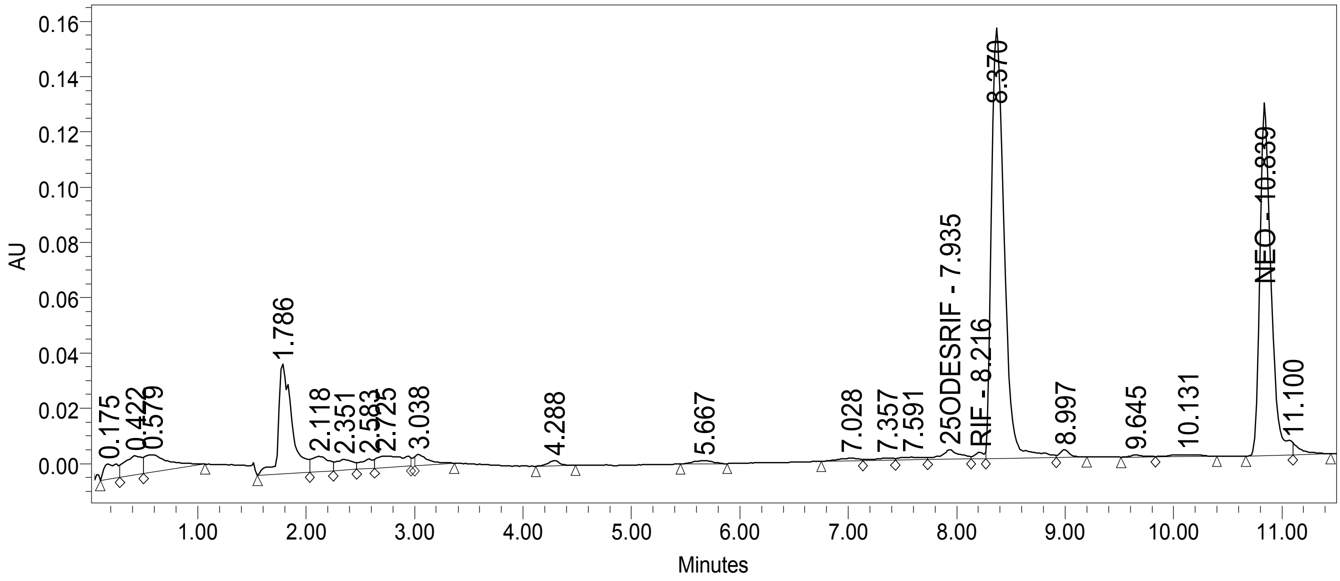

	Peak Name	RT	Area	% Area	Height
1		0.175	46619	1.48	5419
2		0.422	76997	2.44	6883
3		0.579	100344	3.18	6347
4		1.786	343944	10.89	39691
5		2.118	60964	1.93	5500
6		2.351	42743	1.35	3897
7		2.583	28919	0.92	3455
8		2.725	72469	2.30	4075
9		3.038	32358	1.02	3906
10		4.288	14914	0.47	1964
11		5.667	15140	0.48	1144
12		7.028	12937	0.41	971

Reported by User: Saneesh (Saneesh) Project Name: HDI_Stellenbosch_2016

	Peak Name	RT	Area	% Area	Height
13		7.357	10862	0.34	787
14		7.591	15095	0.48	961
15	25ODESRIF	7.935	40200	1.27	3495
16	RIF	8.216	13890	0.44	2345
17		8.370	1259493	39.90	155757
18		8.997	16471	0.52	2675
19		9.645	6144	0.19	801
20		10.131	14849	0.47	752
21	NEO	10.839	908426	28.77	127569
22		11.100	23226	0.74	4468

Reported by User: Saneesh (Saneesh)

Project Name: HDI_Stellenbosch_2016

SAMPLE INFORMATION

Sample Name:	RIF Inc Basil_EtOH 150ug/mL TDI2	Acquired By:	Saneesh
Sample Type:	Unknown	Date Acquired:	2017/09/12 09:29:28 AM
Vial:	11	Acq. Method Set:	HDI_RIF_25ODESRIF_NEO
Injection #:	1	Date Processed:	2017/09/12 11:49:45 AM
Injection Volume:	15.00 ul	Processing Method:	HDI_RIF_ W2996 254.0nm-1.2
Run Time:	11.5 Minutes	Channel Name:	W2996 254.0nm-1.2
Sample Set Name:	RIF_Basil EOH TDI IC50 12_9_17	Proc. Chnl. Descr.:	W2996 PDA 254.0 nm at 1.2

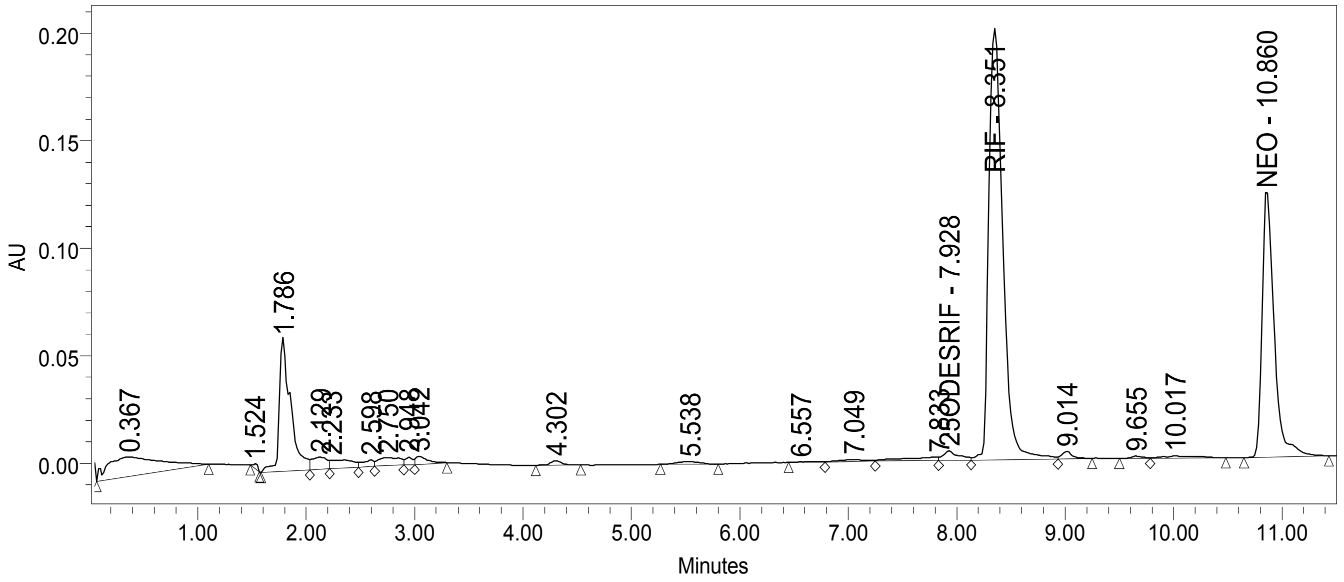

	Peak Name	RT	Area	% Area	Height
1		0.367	306965	7.83	8968
2		1.524	7084	0.18	2361
3		1.786	450631	11.49	61292
4		2.129	54865	1.40	5462
5		2.233	54092	1.38	3903
6		2.598	22625	0.58	3005
7		2.750	53067	1.35	3713
8		2.948	17176	0.44	3321
9		3.042	27834	0.71	3677
10		4.302	15780	0.40	2043
11		5.538	16421	0.42	1128
12		6.557	3198	0.08	232

Reported by User: Saneesh (Saneesh) Project Name: HDI_Stellenbosch_2016

	Peak Name	RT	Area	% Area	Height
13		7.049	17881	0.46	1028
14		7.833	37975	0.97	1451
15	25ODESRIF	7.928	42919	1.09	4251
16	RIF	8.351	1788696	45.60	200407
17		9.014	21368	0.54	3351
18		9.655	7047	0.18	1019
19		10.017	21995	0.56	959
20	NEO	10.860	955230	24.35	125045

SAMPLE INFORMATION

Sample Name:	RIF Inc Basil_EtOH 200ug/mL TDI1	Acquired By:	Saneesh
Sample Type:	Unknown	Date Acquired:	2017/09/12 09:44:12 AM
Vial:	12	Acq. Method Set:	HDI_RIF_25ODESRIF_NEO
Injection #:	1	Date Processed:	2017/09/12 11:49:45 AM
Injection Volume:	15.00 ul	Processing Method:	HDI_RIF_ W2996 254.0nm-1.2
Run Time:	11.5 Minutes	Channel Name:	W2996 254.0nm-1.2
Sample Set Name:	RIF_Basil EOH TDI IC50 12_9_17	Proc. Chnl. Descr.:	W2996 PDA 254.0 nm at 1.2

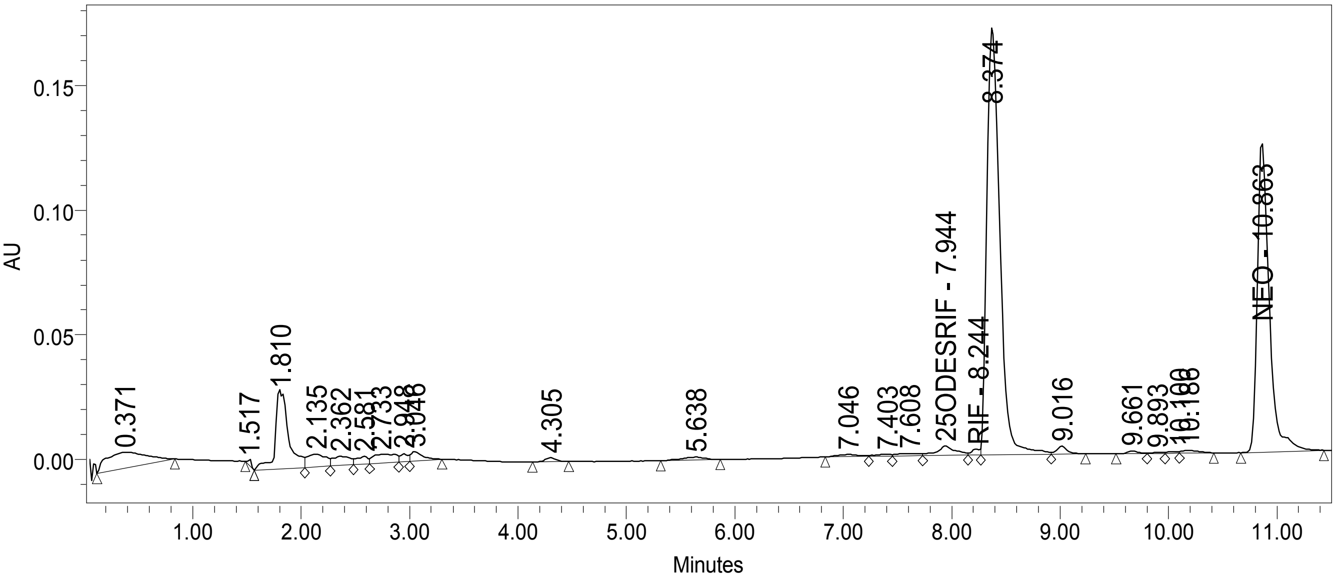

	Peak Name	RT	Area	% Area	Height
1		0.371	165931	5.18	6425
2		1.517	5881	0.18	1942
3		1.810	271967	8.49	32273
4		2.135	60523	1.89	5080
5		2.362	40241	1.26	3663
6		2.581	22421	0.70	3025
7		2.733	49090	1.53	3392
8		2.948	15863	0.50	3080
9		3.046	27930	0.87	3614
10		4.305	11786	0.37	1604
11		5.638	19416	0.61	1285
12		7.046	12894	0.40	968

Reported by User: Saneesh (Saneesh) Project Name: HDI_Stellenbosch_2016

	Peak Name	RT	Area	% Area	Height
13		7.403	8085	0.25	734
14		7.608	14716	0.46	974
15	25ODESRIF	7.944	44516	1.39	3708
16	RIF	8.244	12714	0.40	2343
17		8.374	1422557	44.43	172423
18		9.016	20151	0.63	3053
19		9.661	7412	0.23	1062
20		9.893	3096	0.10	392
21		10.100	4680	0.15	664
22		10.186	11730	0.37	1038
23	NEO	10.863	947914	29.61	123632

SAMPLE INFORMATION

Sample Name:	RIF Inc Basil_EtOH 200ug/mL TDI2	Acquired By:	Saneesh
Sample Type:	Unknown	Date Acquired:	2017/09/12 09:58:55 AM
Vial:	13	Acq. Method Set:	HDI_RIF_25ODESRIF_NEO
Injection #:	1	Date Processed:	2017/09/12 11:49:45 AM
Injection Volume:	15.00 ul	Processing Method:	HDI_RIF_ W2996 254.0nm-1.2
Run Time:	11.5 Minutes	Channel Name:	W2996 254.0nm-1.2
Sample Set Name:	RIF_Basil EOH TDI IC50 12_9_17	Proc. Chnl. Descr.:	W2996 PDA 254.0 nm at 1.2

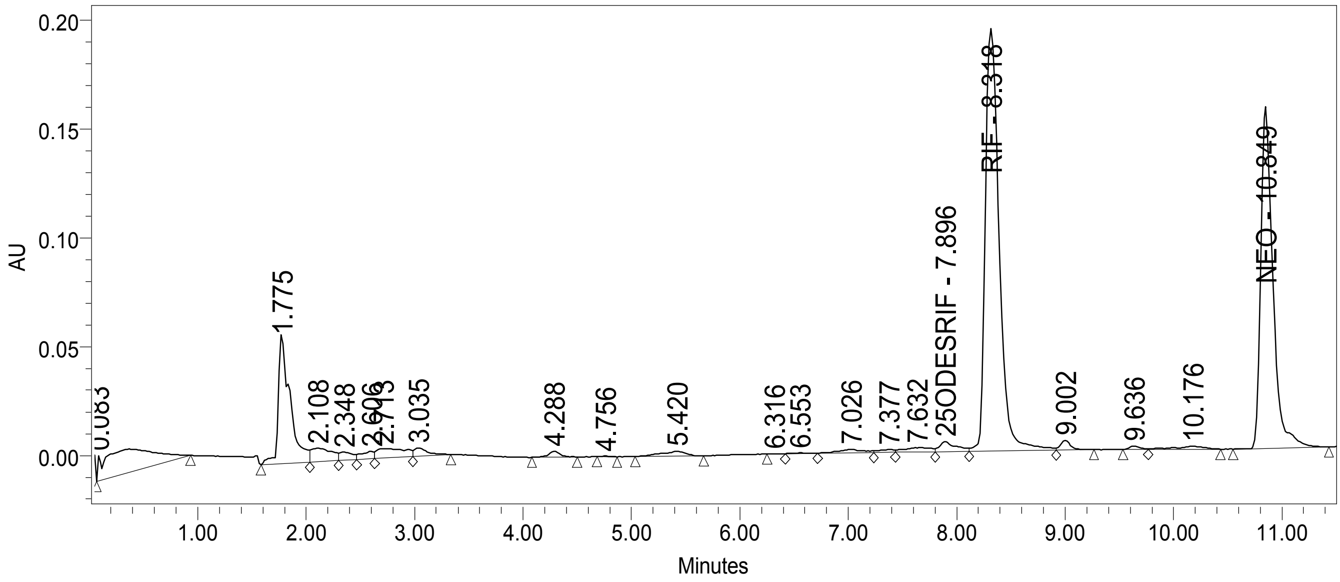

	Peak Name	RT	Area	% Area	Height
1		0.083	348757	8.23	11470
2		1.775	447350	10.55	59148
3		2.108	82474	1.95	6175
4		2.348	32163	0.76	3788
5		2.606	28323	0.67	3364
6		2.713	77106	1.82	4269
7		3.035	32489	0.77	3770
8		4.288	20260	0.48	2734
9		4.756	1904	0.04	322
10		5.420	35159	0.83	2254
11		6.316	1107	0.03	184
12		6.553	4412	0.10	356

Reported by User: Saneesh (Saneesh) Project Name: HDI_Stellenbosch_2016

	Peak Name	RT	Area	% Area	Height
13		7.026	26470	0.62	1655
14		7.377	13217	0.31	1302
15		7.632	35310	0.83	1947
16	25ODESRIF	7.896	49105	1.16	4689
17	RIF	8.318	1722903	40.63	193940
18		9.002	28329	0.67	4441
19		9.636	10271	0.24	1563
20		10.176	25914	0.61	1338
21	NEO	10.849	1217049	28.70	156606

4 CYP2B6/3A4 mRNA Expression by basil extracts – GEL Images

- Page 949: Gel image of the GAPDH house-keeping gene expression – blue marking represents the cell control CC and red markings represent 1. Basil$_{Aq}$, 2. Basil$_{MeOH}$, 3. Basil$_{EtOH}$.
- Page 950: Gel image of relative CYP3A4 mRNA expressions – blue marking represents the cell control CC and red markings represent 1. Basil$_{Aq}$, 2. Basil$_{MeOH}$, 3. Basil$_{EtOH}$.
- Page 951: Gel image of relative CYP2B6 mRNA expressions – blue marking represents the cell control CC and red markings represent 1. Basil$_{Aq}$, 2. Basil$_{MeOH}$, 3. Basil$_{EtOH}$.

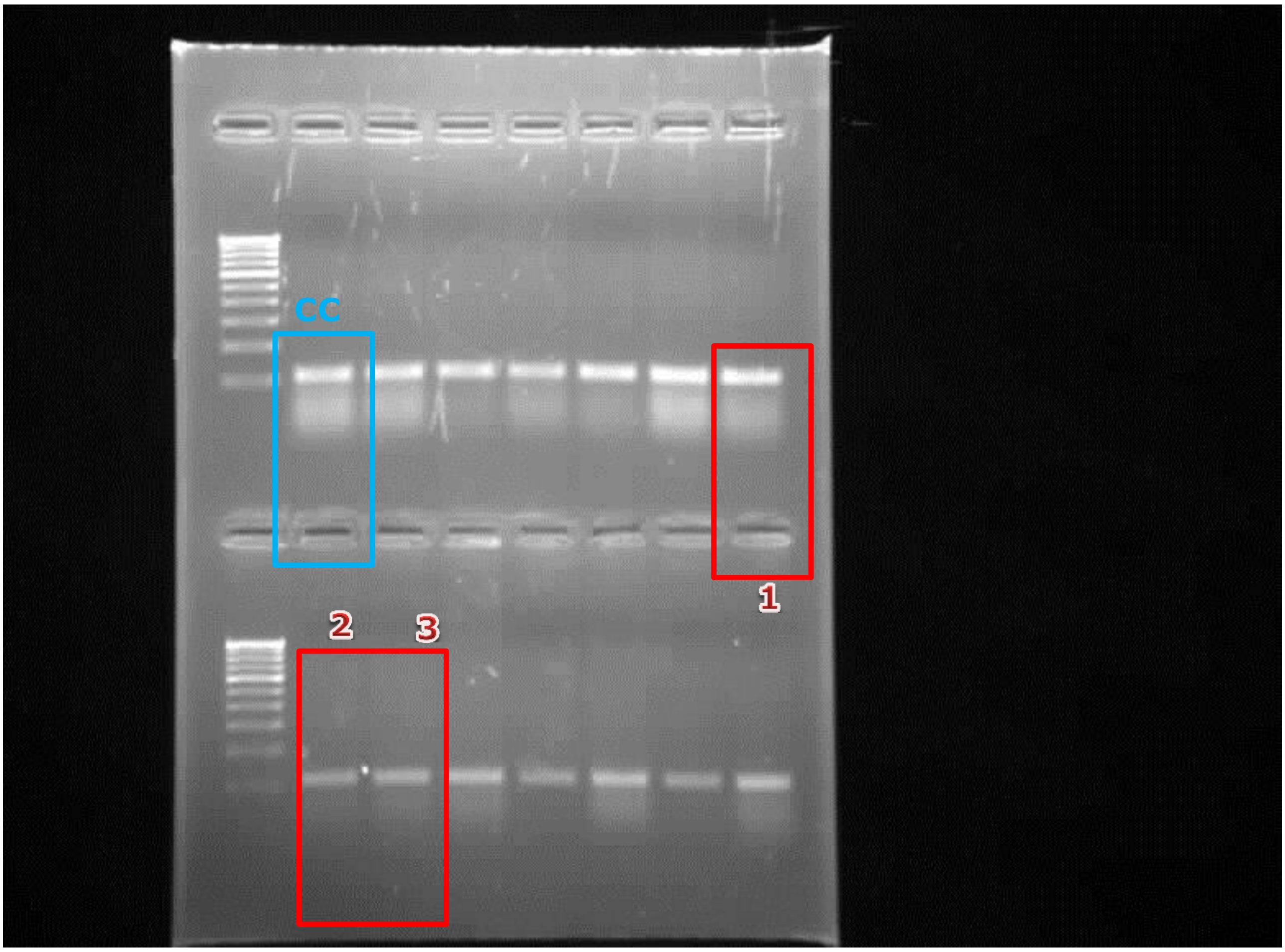

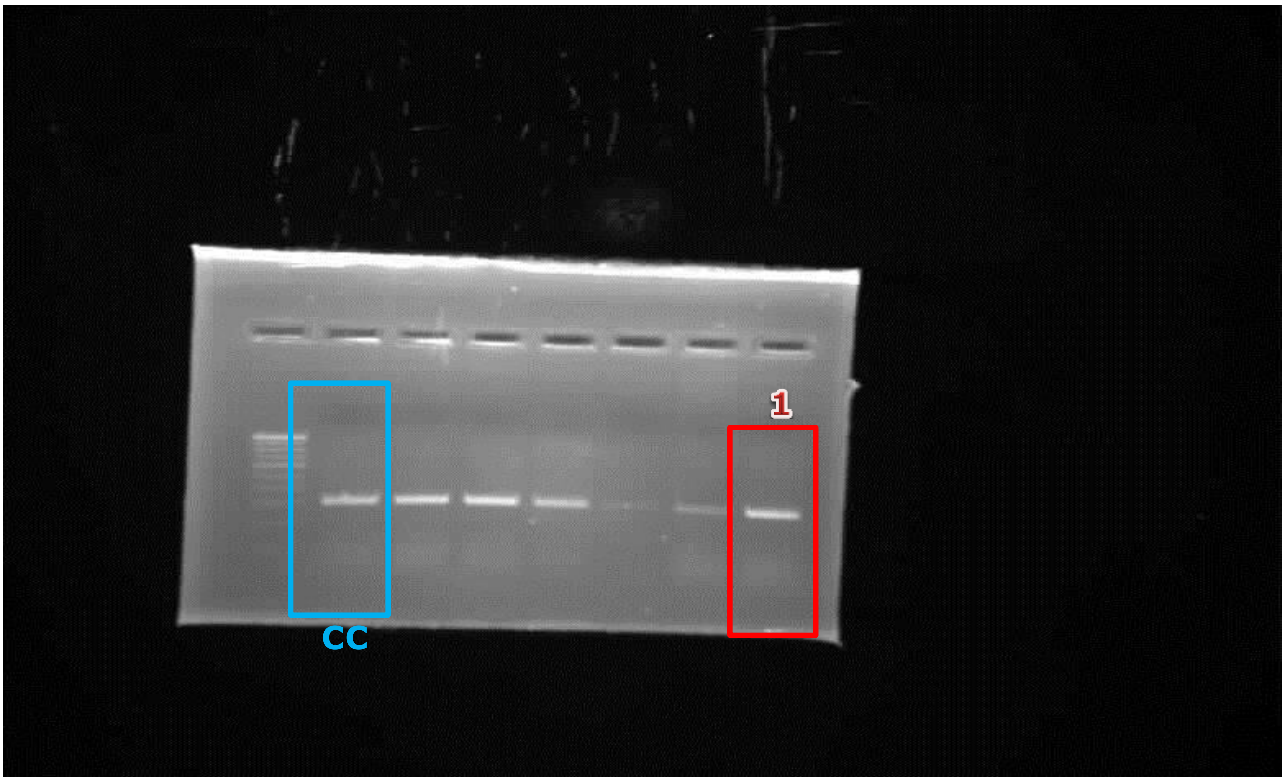
1
CC

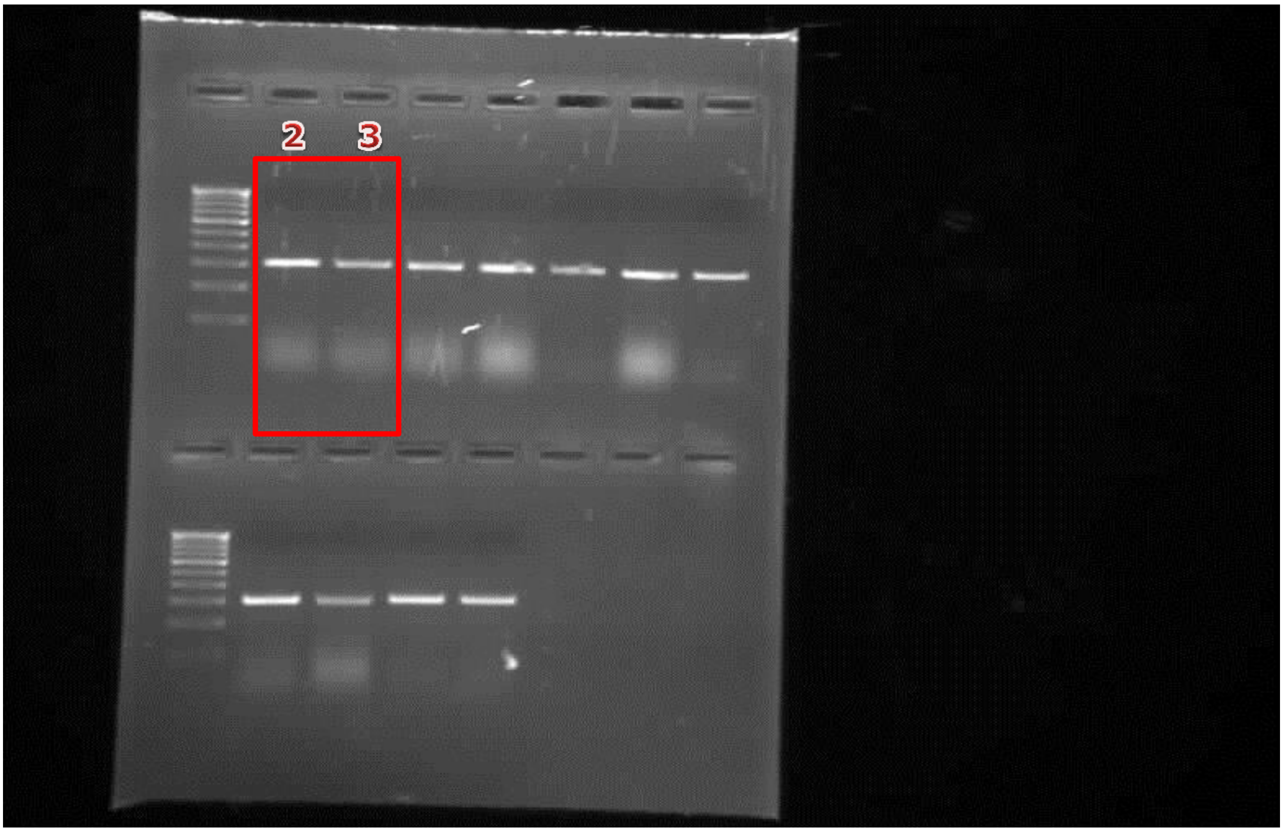
2
3

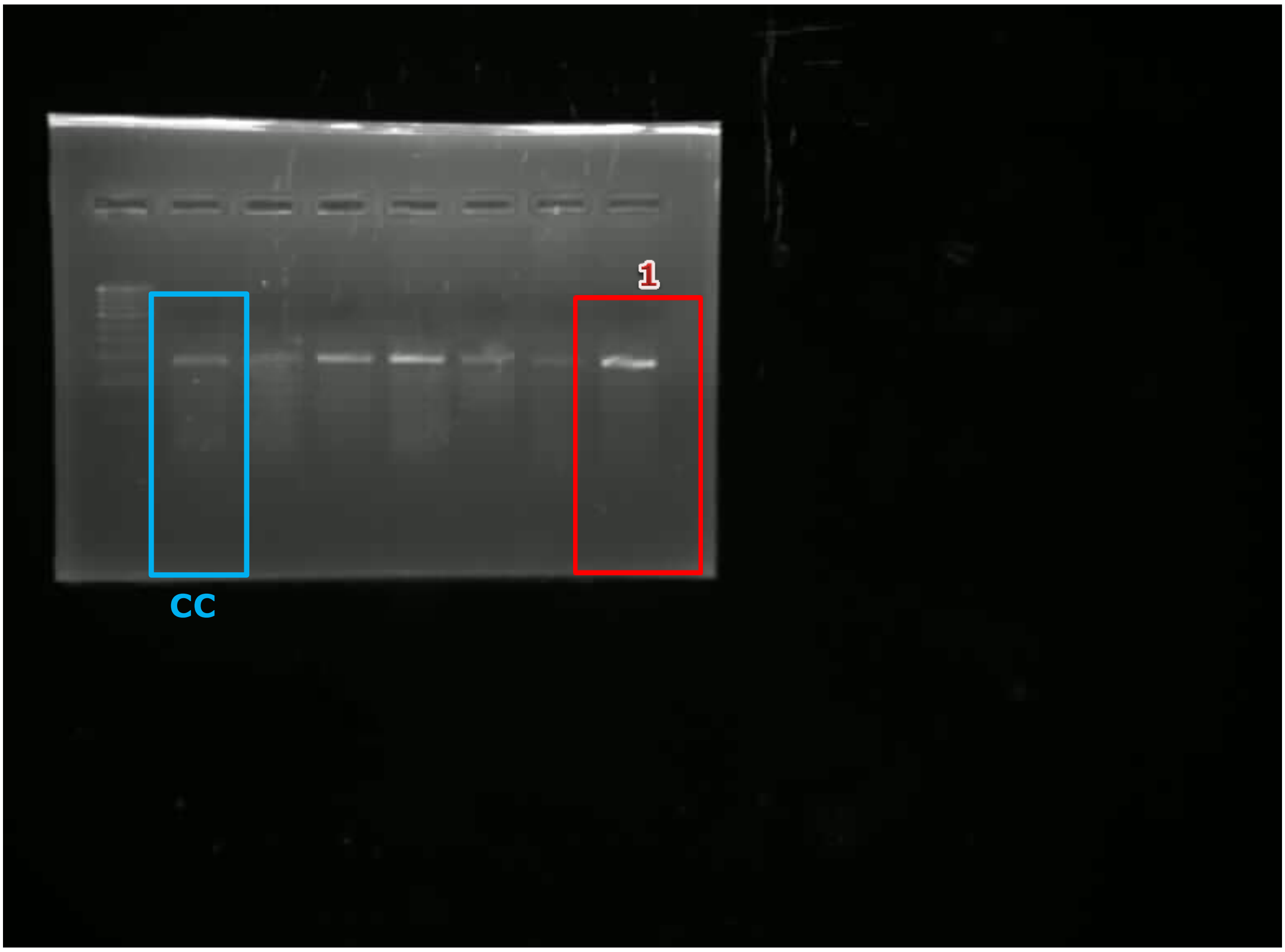
1
CC
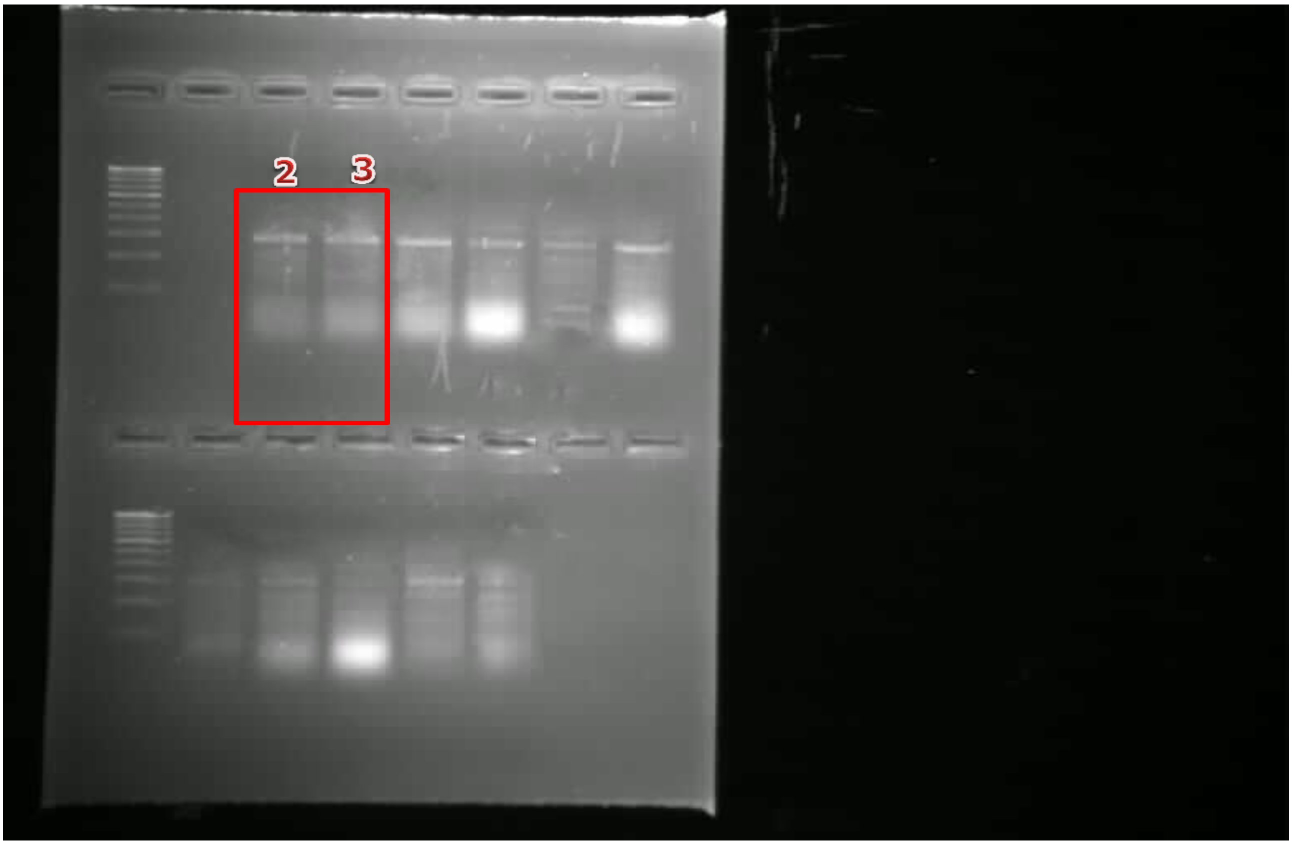
2
3

5 Certificate of analysis – Ocimum basilicum herb (Pharma Germania-Petrow Foods, RSA)

petrow
food ingredients (pty) ltd

CERTIFICATE OF ANALYSIS

Product: **Basil Whole**

Batch no	: PFI-2645
Production date	: 08/2014
Best before date	: 31/12/2016

IDENTITY

Botanical name	: *Ocimum basilicum L.*
Family name	: N.O. Labiatae
Part of Plant Used	: Milled dried leaves
Country of Origin	: Egypt

ORGANOLEPTIC	SPECIFICATION	RESULT
Appearance	: Flakes	: Complies
Colour	: Bright green	: Complies
Odour	: Fragrant, sweet, minty.	: Complies
Taste	: Sweet, camphorous with lemony-anise note.	: Complies

ANALYTICAL	SPECIFICATION	RESULT
Moisture	: 10 % max	8.5%

RECOMMENDED STORAGE CONDITIONS

On pallets in a cool, dry, insect and rodent proof room, in sealed bags, away from direct heat and sunlight.

ISSUED BY: **Melissa Roscotch** **DATEISSUED:** **12/09/2014**

Note: *The information contained in this certificate is to our knowledge true and correct and presented in good faith. This information is offered solely for your consideration and verification.*

GEMINI SPICES
Express Chef

REGISTRATION NO. 2000/009680/07 • VAT NO. 4400190890
Website: www.petrowfoods.co.za • E-mail: info@petrowfoods.co.za
JOHANNESBURG – 16 ENDEAN STREET, CITY DEEP, JOHANNESBURG • T +27 (11) 613 2702/5 • F +27 (11) 613 6227
CAPE TOWN – TREDOUX STREET, BEACONVILLE, PAROW • T +27 (21) 932 9142 • F +27 (21) 932 9142
DURBAN – PALM RIVER INDUSTRIAL PARK, UNIT 7, DEVON ROAD, PINETOWN • T +27 (31) 709 9211 • F +27 (31) 709 9212

DIRECTORS – M. SOSNOVIK (Group CEO), R. G. MOE (Managing), Jack M. GINSBERG (Group Financial Director), R. VORSTER (Financial), Dr. C. W. TAYLOR (Technical), W. DIRKER (Marketing), Y. Bhayat, N. Mudaly

6 LC-MS/MS Spectral data of basil extracts illustrating MS/MS fragmentation data for major molecules.

- **Basil$_{EtOH}$ (Negative Scan)**

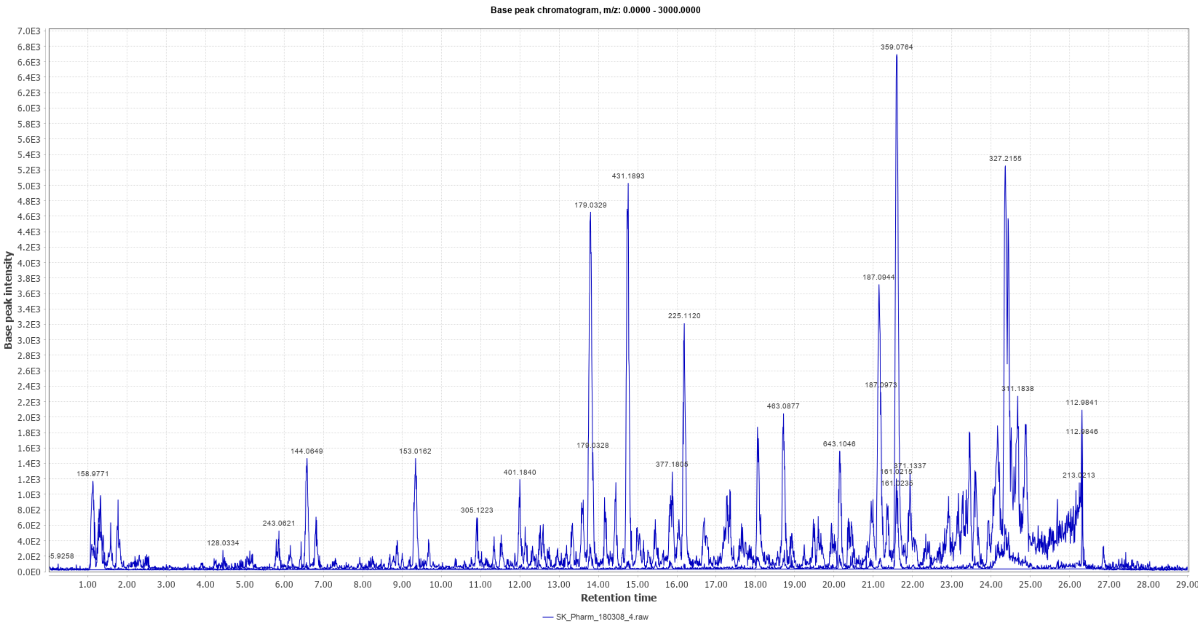

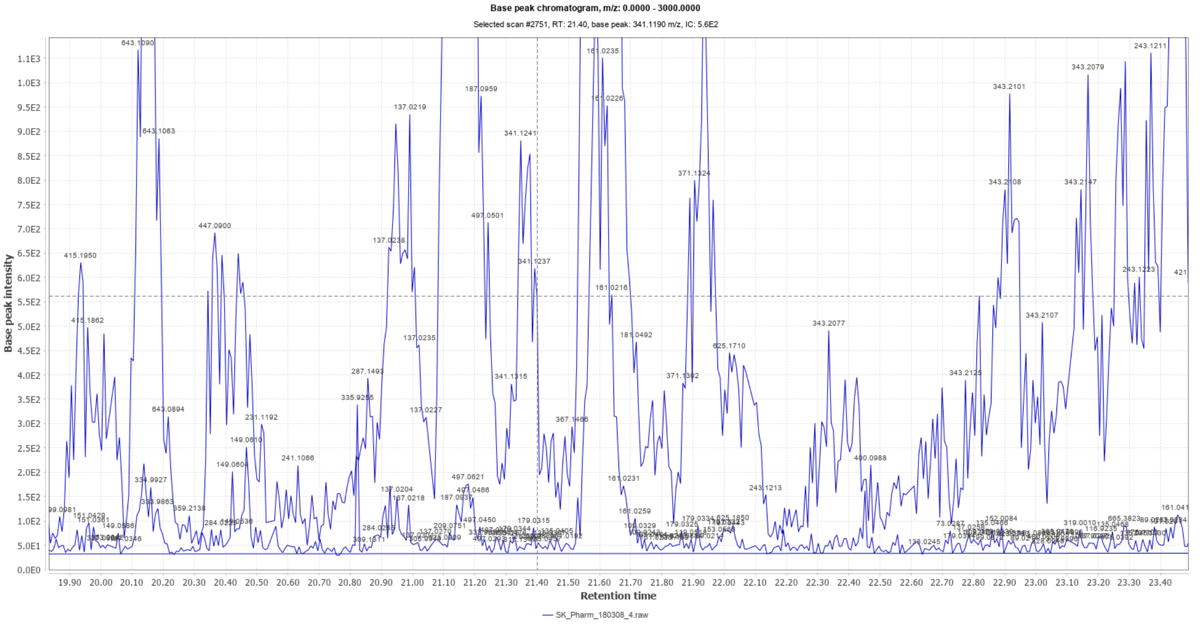

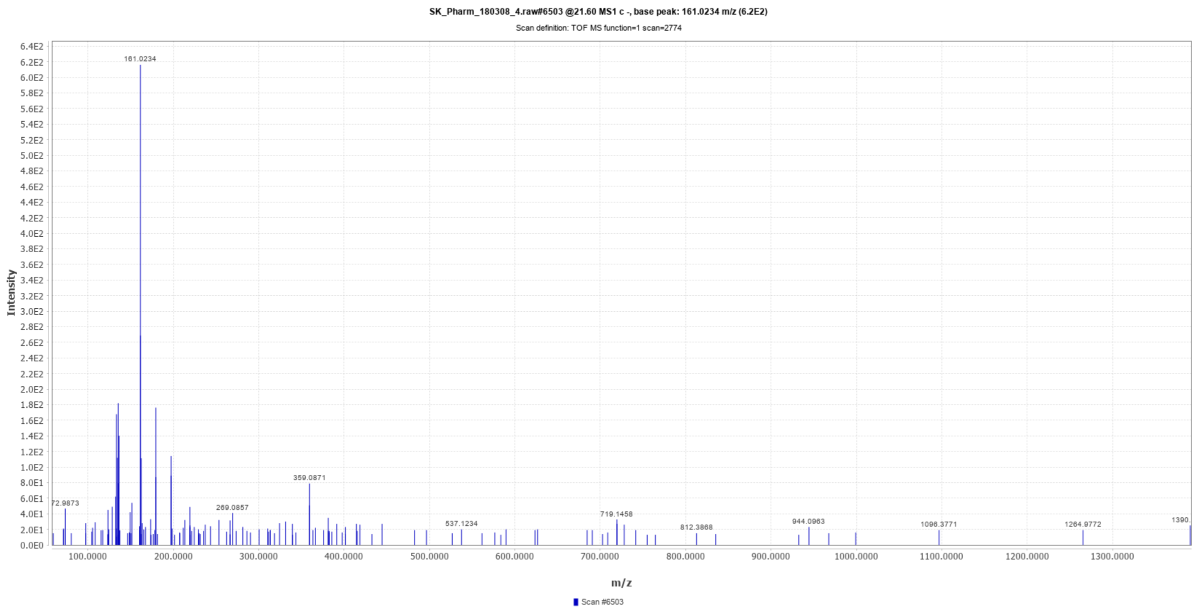
SK_Pharm_180308_4.raw#6503 @21.60 MS1 c -, base peak: 161.0234 m/z (6.2E2)
Scan definition: TOF MS function=1 scan=2774
Intensity
m/z
161.0234
72.9873
269.0857
359.0871
537.1234
719.1458
812.3868
944.0963
1096.3771
1264.9772
Scan #6503

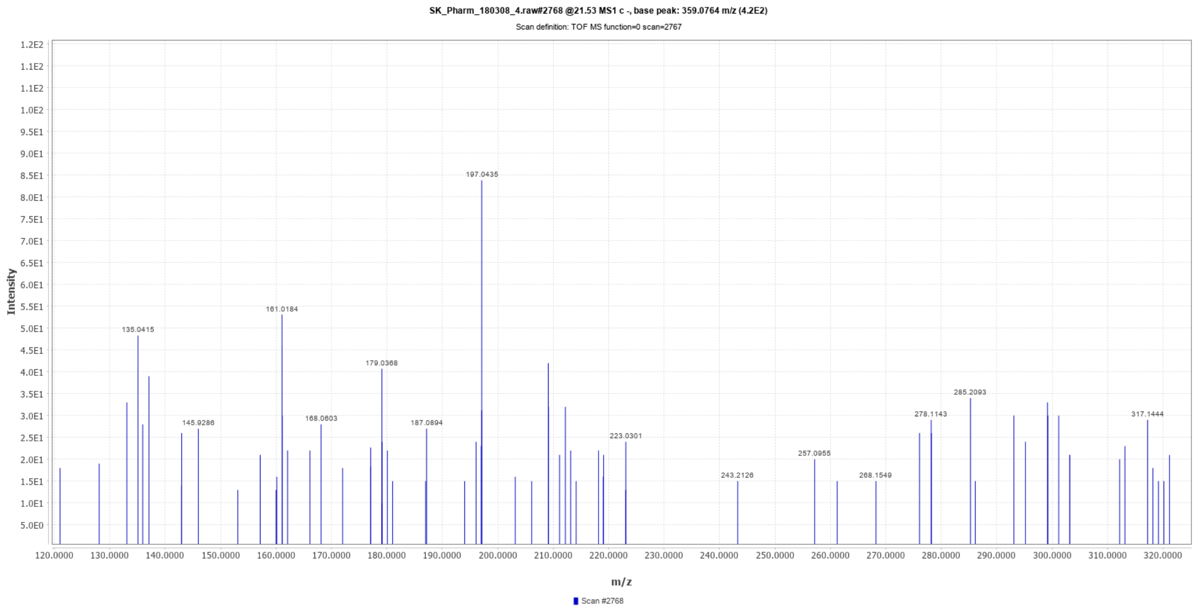
SK_Pharm_180308_4.raw#2768 @21.53 MS1 c -, base peak: 359.0764 m/z (4.2E2)
Scan definition: TOF MS function=0 scan=2767
Intensity
m/z
197.0435
161.0184
135.0415
179.0368
145.9286
168.0603
187.0894
223.0301
243.2126
257.0955
268.1549
278.1143
285.2093
317.1444
Scan #2768

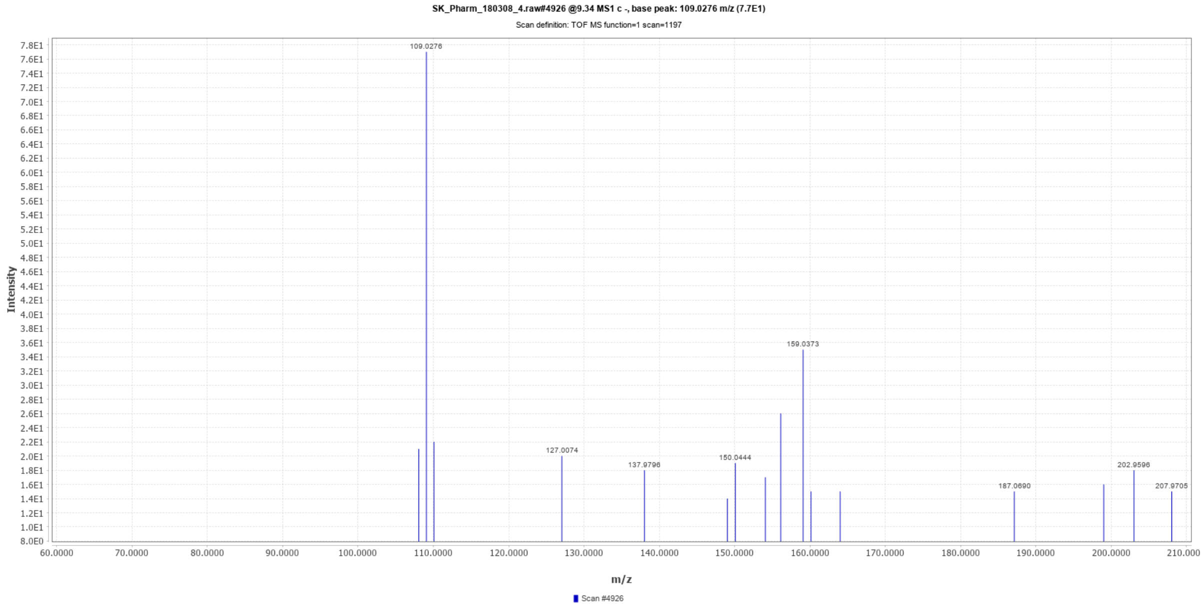
SK_Pharm_180308_4.raw#4926 @9.34 MS1 c -, base peak: 109.0276 m/z (7.7E1)
Scan definition: TOF MS function=1 scan=1197
Intensity
m/z
109.0276
159.0373
127.0074
137.9796
150.0444
187.0690
202.9596
207.9705
Scan #4926

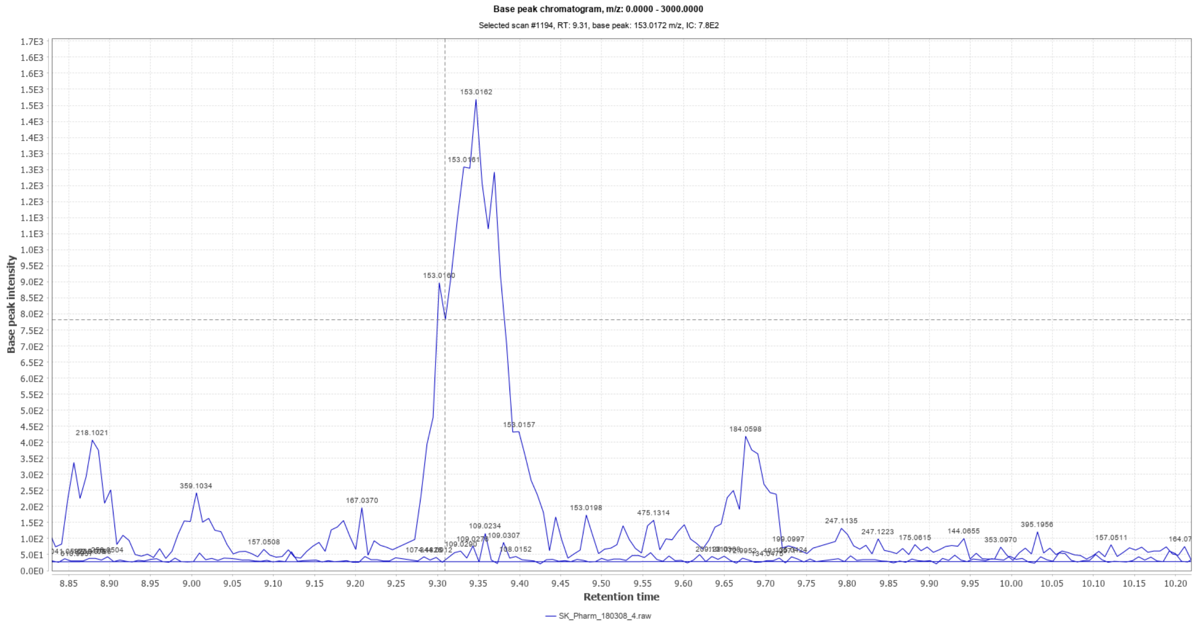
Base peak chromatogram, m/z: 0.0000 - 3000.0000
Selected scan #1194, RT: 9.31, base peak: 153.0172 m/z, IC: 7.8E2
Base peak intensity
Retention time
153.0162
153.0161
153.0160
153.0157
218.1021
359.1034
167.0370
184.0598
153.0198
475.1314
247.1135
247.1223
175.0615
144.0655
395.1956
353.0970
157.0511
157.0508
109.0234
109.0307
108.0152
199.0997
SK_Pharm_180308_4.raw

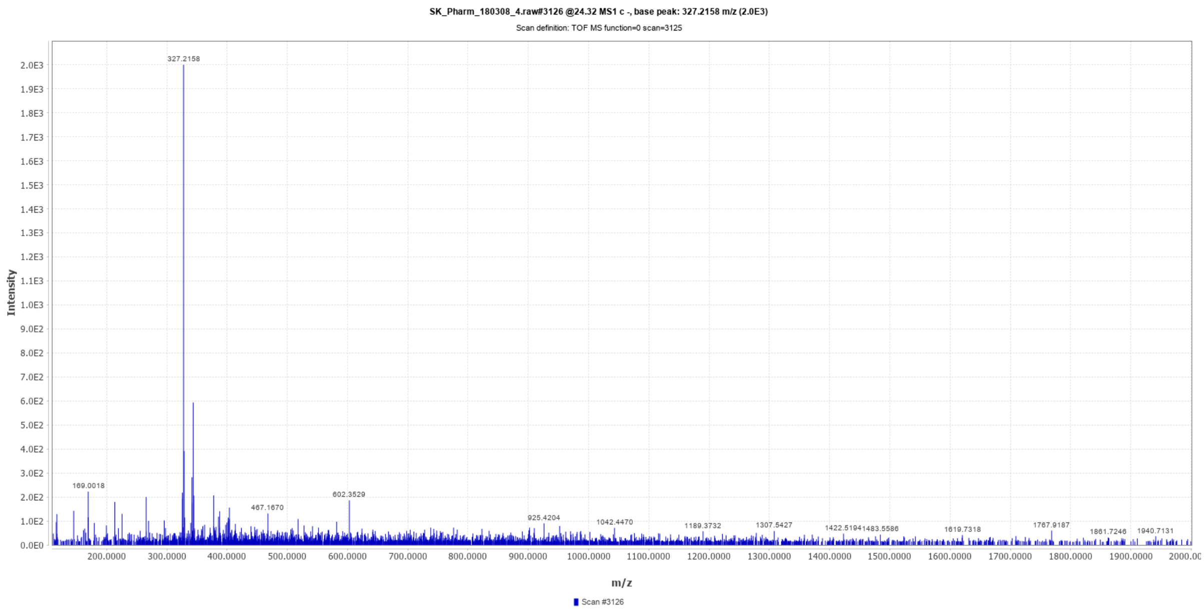
SK_Pharm_180308_4.raw#3126 @24.32 MS1 c -, base peak: 327.2158 m/z (2.0E3)
Scan definition: TOF MS function=0 scan=3125
Intensity
m/z
327.2158
169.0018
467.1670
602.3529
925.4204
1042.4470
1189.3732
1307.5427
1422.5194
1483.5586
1619.7318
1767.9187
1861.7246
1940.7131
Scan #3126

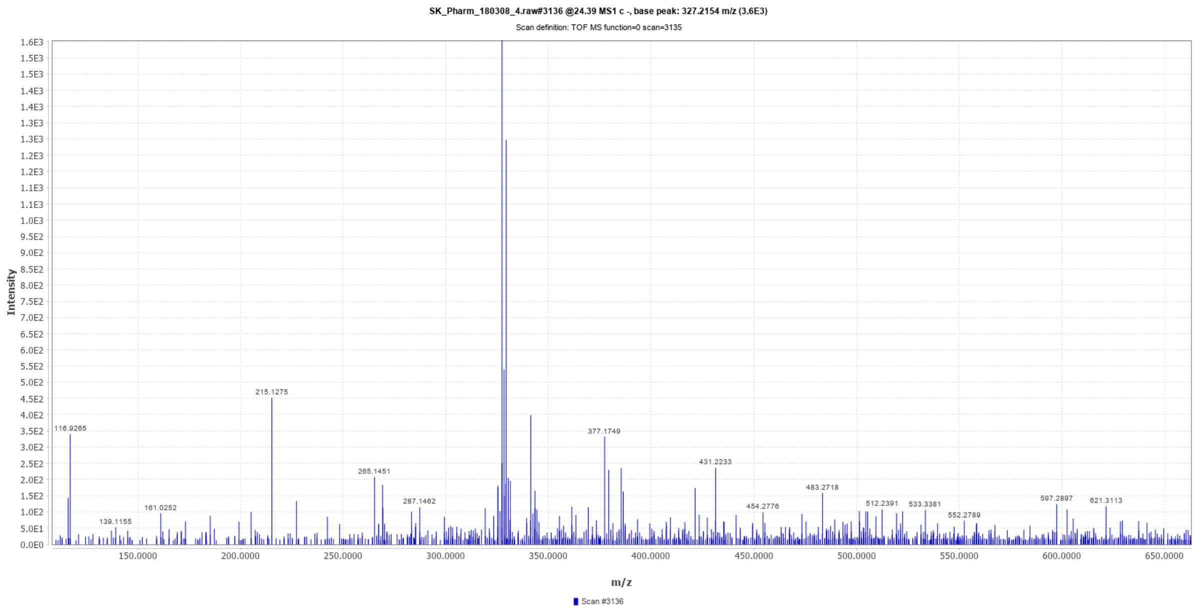
SK_Pharm_180308_4.raw#3136 @24.39 MS1 c -, base peak: 327.2154 m/z (3.6E3)
Scan definition: TOF MS function=0 scan=3135
Intensity
m/z
116.9265
139.1155
161.0252
215.1275
265.1451
287.1462
377.1749
431.2233
454.2776
483.2718
512.2391
533.3381
552.2789
597.2897
621.3113
Scan #3136

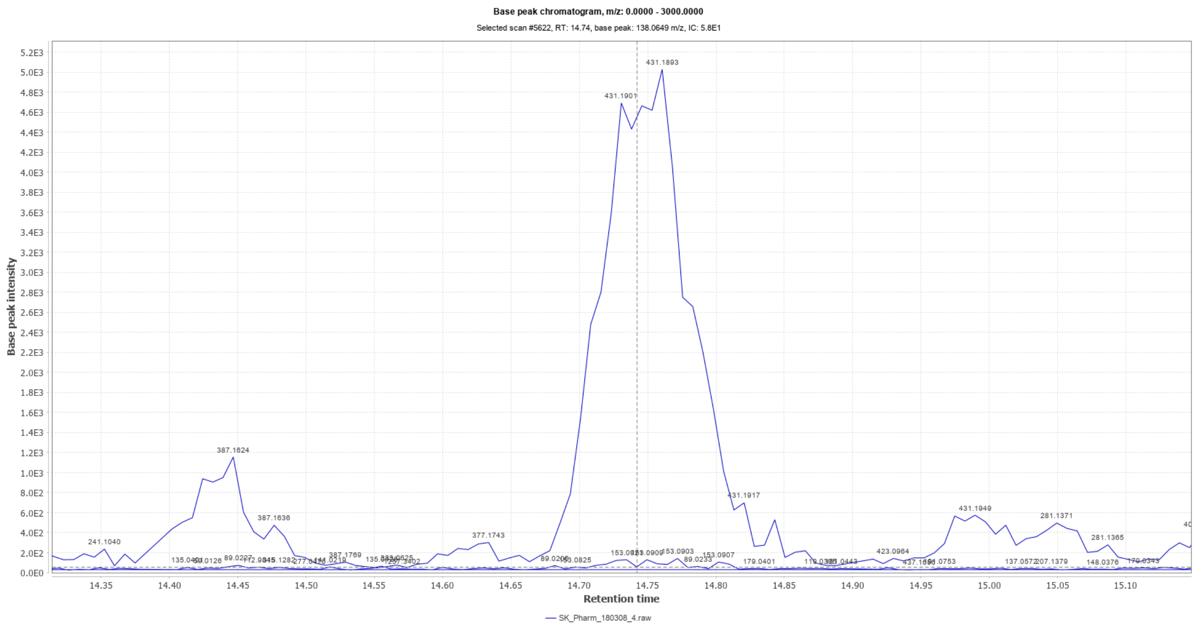
Base peak chromatogram, m/z: 0.0000 - 3000.0000
Selected scan #5622, RT: 14.74, base peak: 138.0649 m/z, IC: 5.8E1
Base peak intensity
Retention time
431.1893
431.1901
387.1624
387.1636
431.1917
431.1949
281.1371
281.1365
377.1743
241.1040
423.0964
SK_Pharm_180308_4.raw

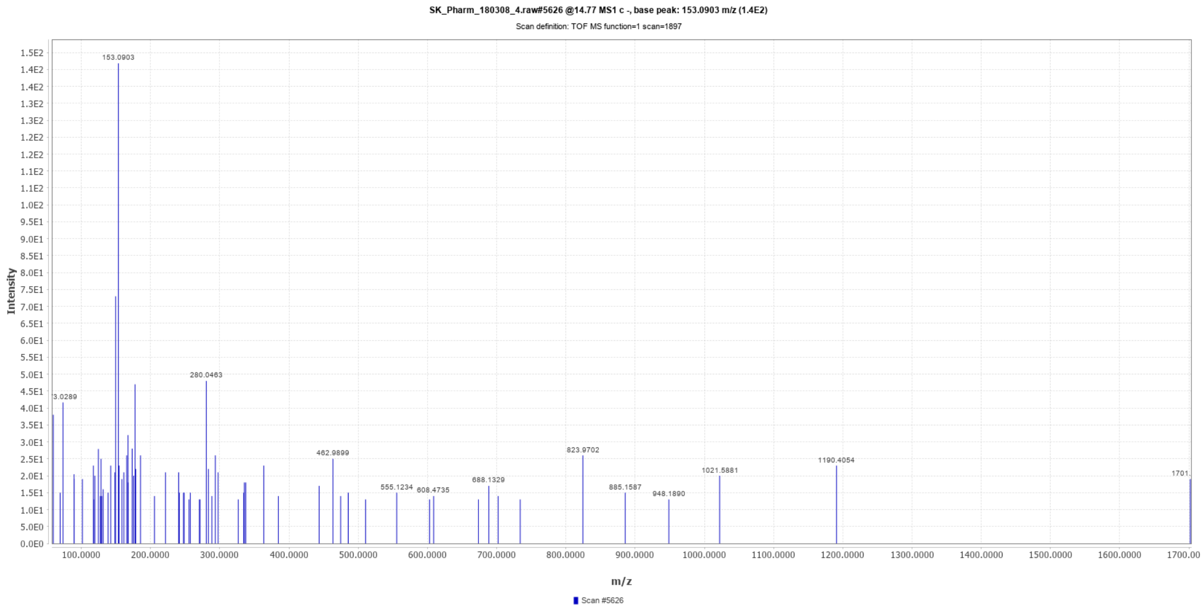
SK_Pharm_180308_4.raw#5626 @14.77 MS1 c -, base peak: 153.0903 m/z (1.4E2)
Scan definition: TOF MS function=1 scan=1897
Intensity
m/z
153.0903
280.0463
462.9899
555.1234
608.4735
688.1329
823.9702
885.1587
948.1890
1021.5881
1190.4054
Scan #5626

Base peak chromatogram, m/z: 0.0000 - 3000.0000

Selected scan #5501, RT: 13.80, base peak: 135.0448 m/z, IC: 2.4E2

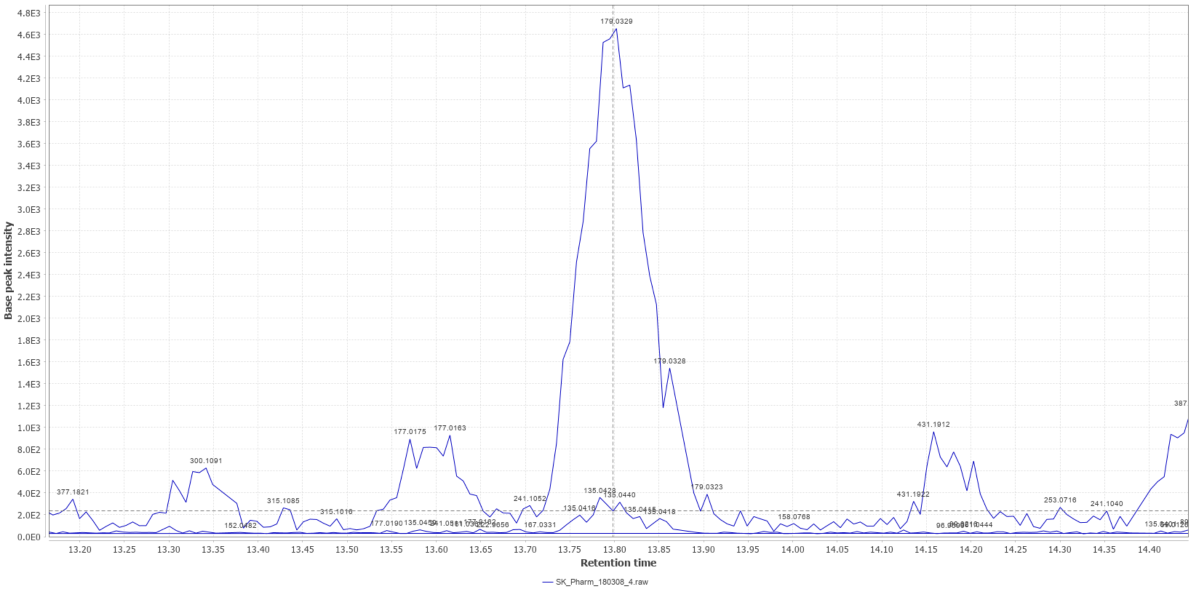

SK_Pharm_180308_4.raw#5501 @13.80 MS1 c -, base peak: 135.0448 m/z (2.4E2)

Scan definition: TOF MS function=1 scan=1772

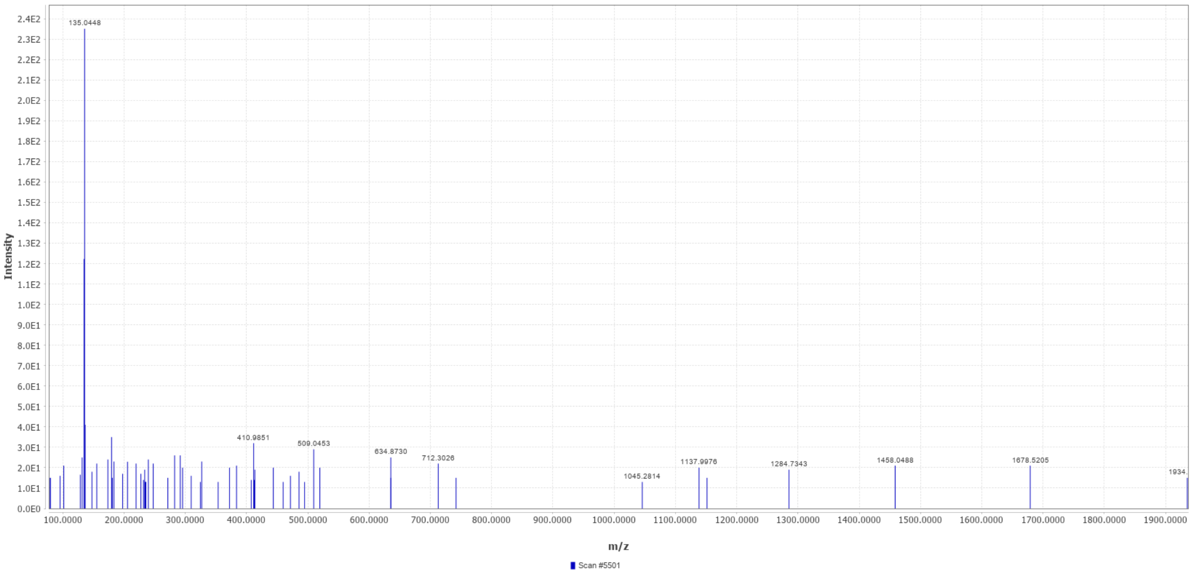

Base peak chromatogram, m/z: 0.0000 - 3000.0000

Selected scan #6870, RT: 24.44, base peak: 329.2326 m/z, IC: 5.1E2

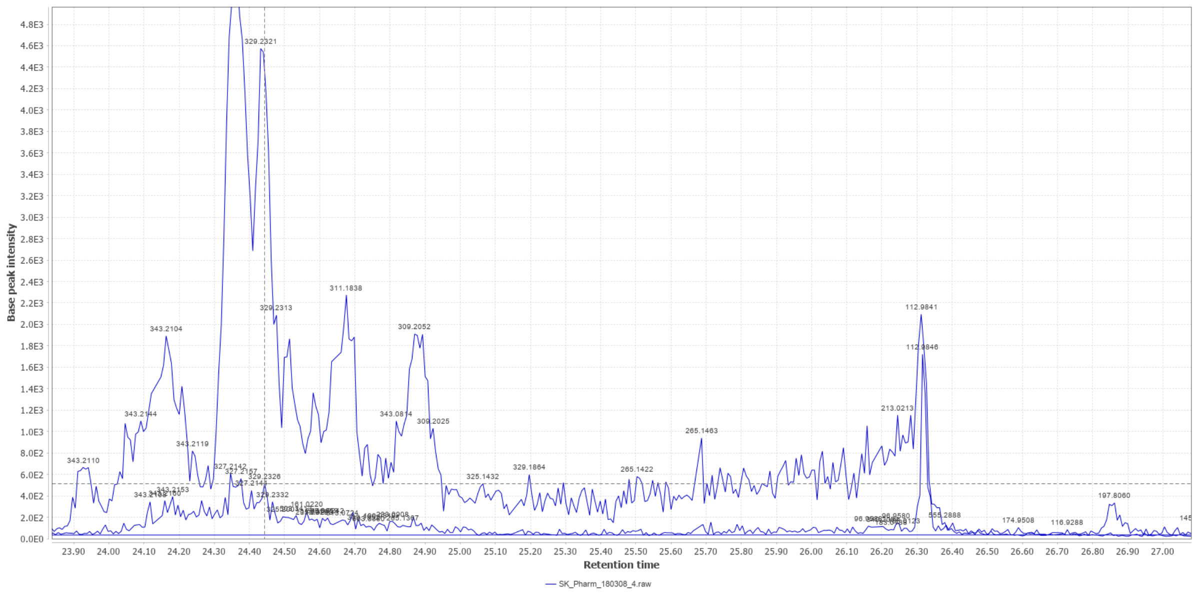

SK_Pharm_180308_4.raw#6870 @24.44 MS1 c -, base peak: 329.2326 m/z (5.1E2)

Scan definition: TOF MS function=1 scan=3141

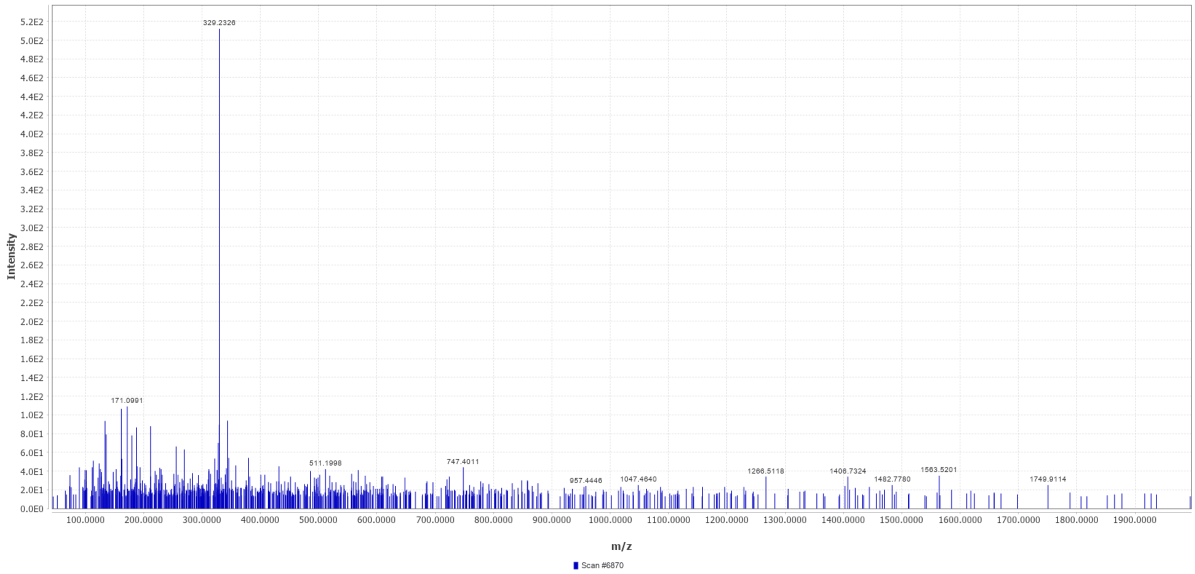

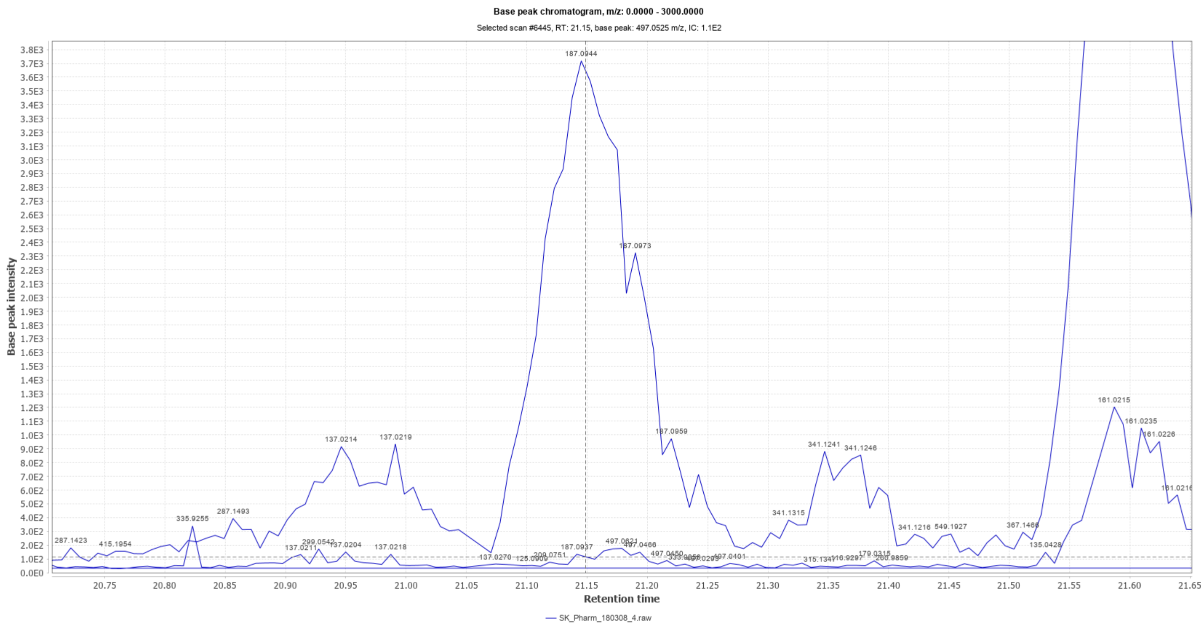

Base peak chromatogram, m/z: 0.0000 - 3000.0000
Selected scan #6445, RT: 21.15, base peak: 497.0525 m/z, IC: 1.1E2
Base peak intensity
Retention time
SK_Pharm_180308_4.raw

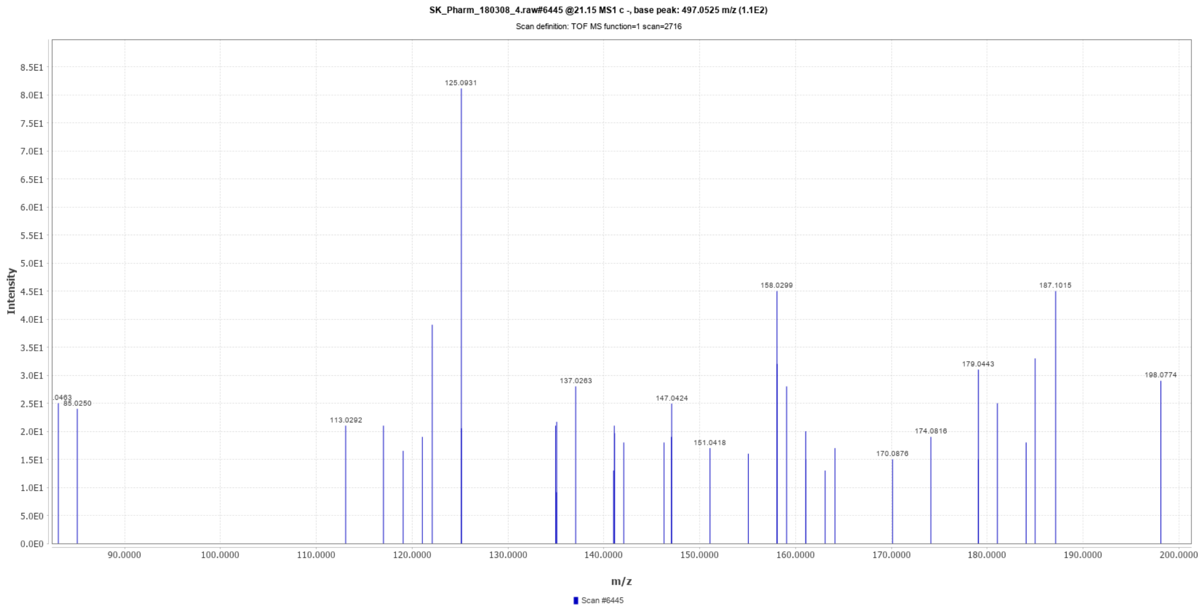

SK_Pharm_180308_4.raw#6445 @21.15 MS1 c -, base peak: 497.0525 m/z (1.1E2)
Scan definition: TOF MS function=1 scan=2716
Intensity
m/z
Scan #6445

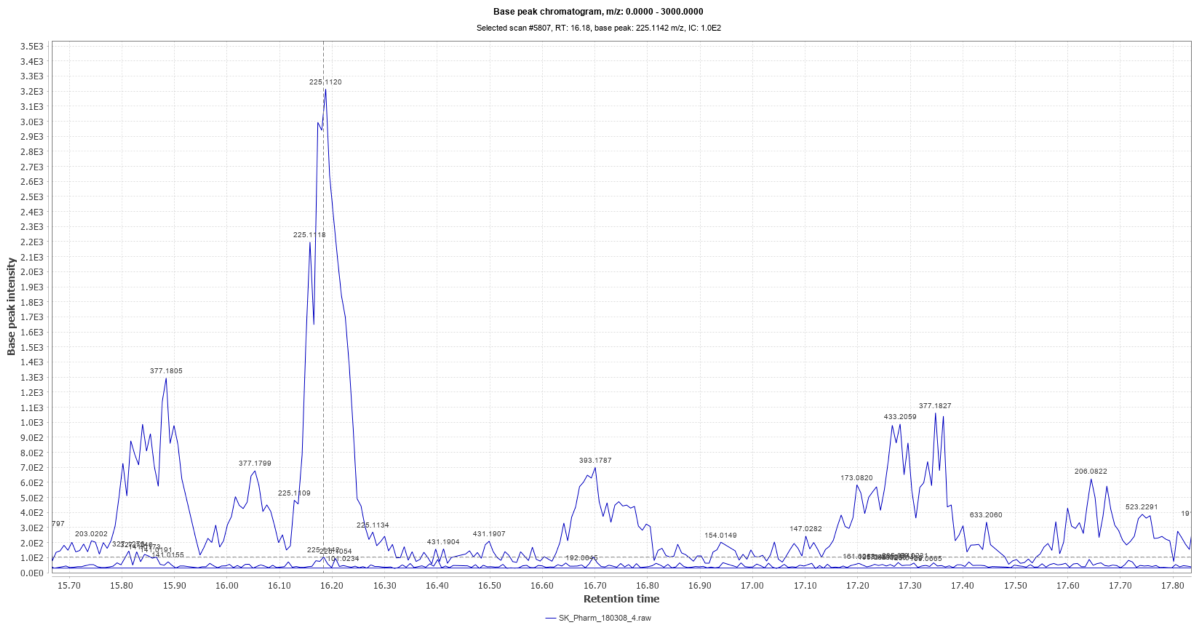

Base peak chromatogram, m/z: 0.0000 - 3000.0000
Selected scan #5807, RT: 16.18, base peak: 225.1142 m/z, IC: 1.0E2
Base peak intensity
Retention time
225.1120
225.1118
377.1805
377.1799
225.1109
225.1134
431.1904
431.1907
393.1787
154.0149
147.0282
173.0820
433.2059
377.1827
633.2060
206.0822
523.2291
203.0202
SK_Pharm_180308_4.raw

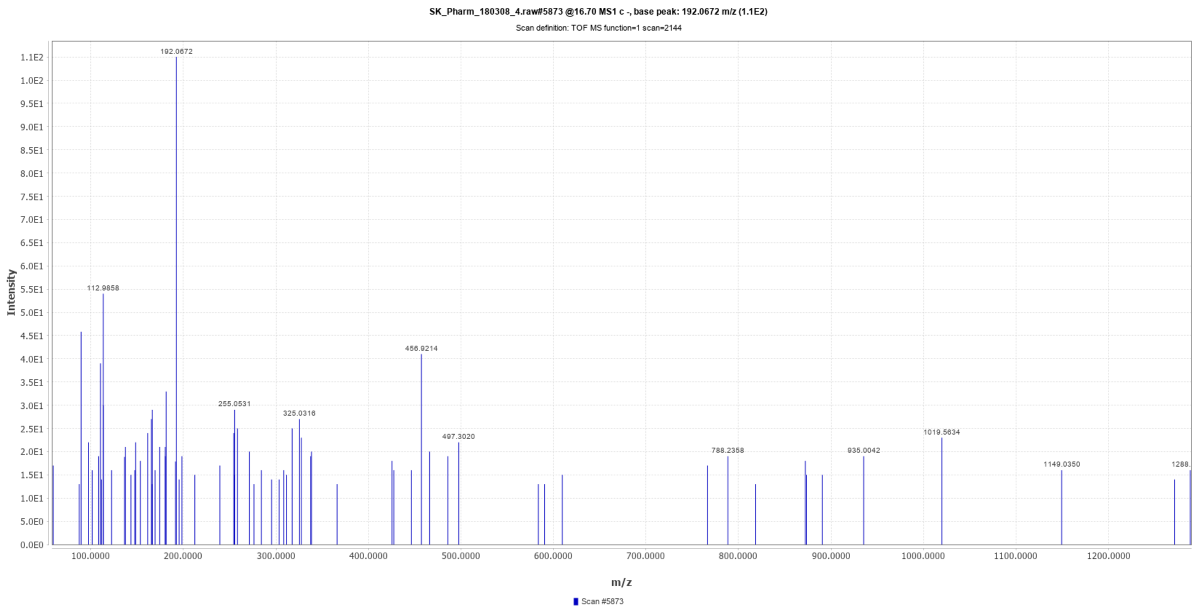
SK_Pharm_180308_4.raw#5873 @16.70 MS1 c -, base peak: 192.0672 m/z (1.1E2)
Scan definition: TOF MS function=1 scan=2144
Intensity
m/z
192.0672
112.9858
456.9214
255.0531
325.0316
497.3020
788.2358
935.0042
1019.5634
1149.0350
Scan #5873

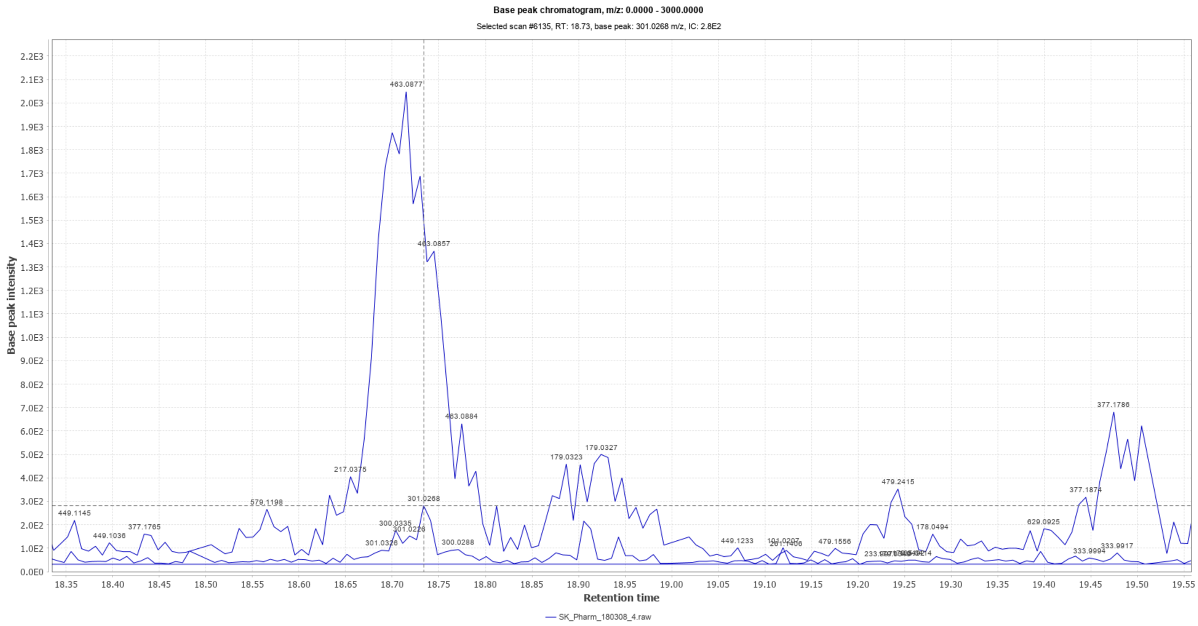
Base peak chromatogram, m/z: 0.0000 - 3000.0000
Selected scan #6135, RT: 18.73, base peak: 301.0268 m/z, IC: 2.8E2
Base peak intensity
Retention time
463.0877
463.0857
463.0884
217.0375
179.0323
179.0327
377.1786
479.2415
377.1874
449.1145
449.1036
377.1765
579.1198
301.0268
300.0335
301.0326
300.0288
178.0494
629.0925
333.9994
333.9917
449.1233
479.1556
SK_Pharm_180308_4.raw

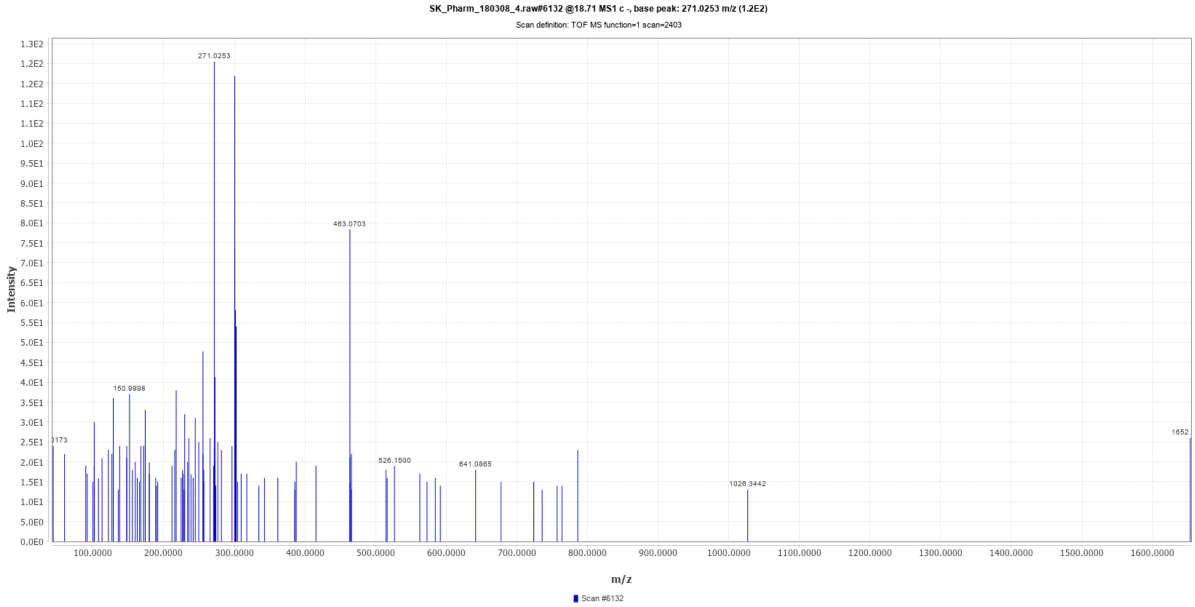

- **Basil$_{EtOH}$ (Positive Scan)**

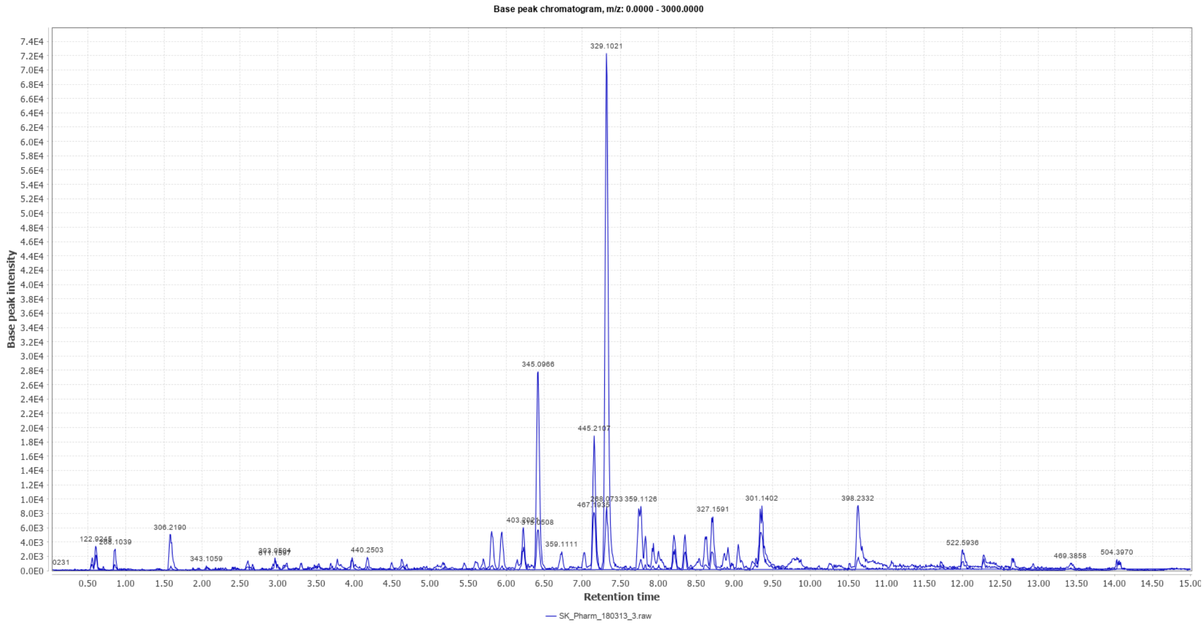

Base peak chromatogram, m/z: 0.0000 - 3000.0000

Selected scan #2862, RT: 7.31, base peak: 268.0736 m/z, IC: 6.1E3

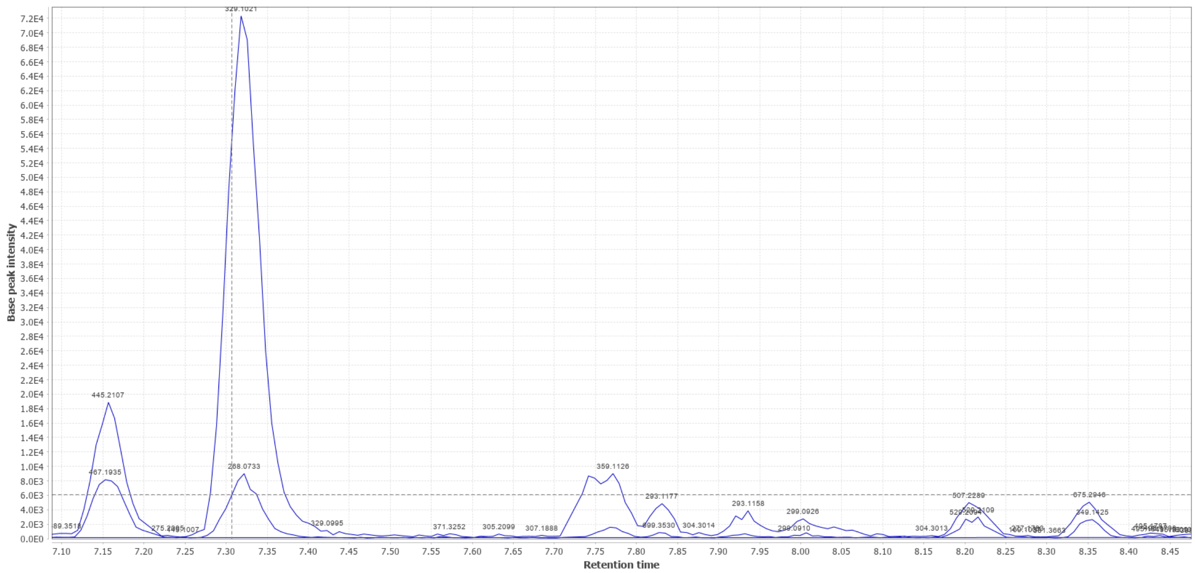

SK_Pharm_180313_3.raw#2862 @7.31 MS1 c +, base peak: 268.0736 m/z (6.1E3)

Scan definition: TOF MS function=1 scan=935

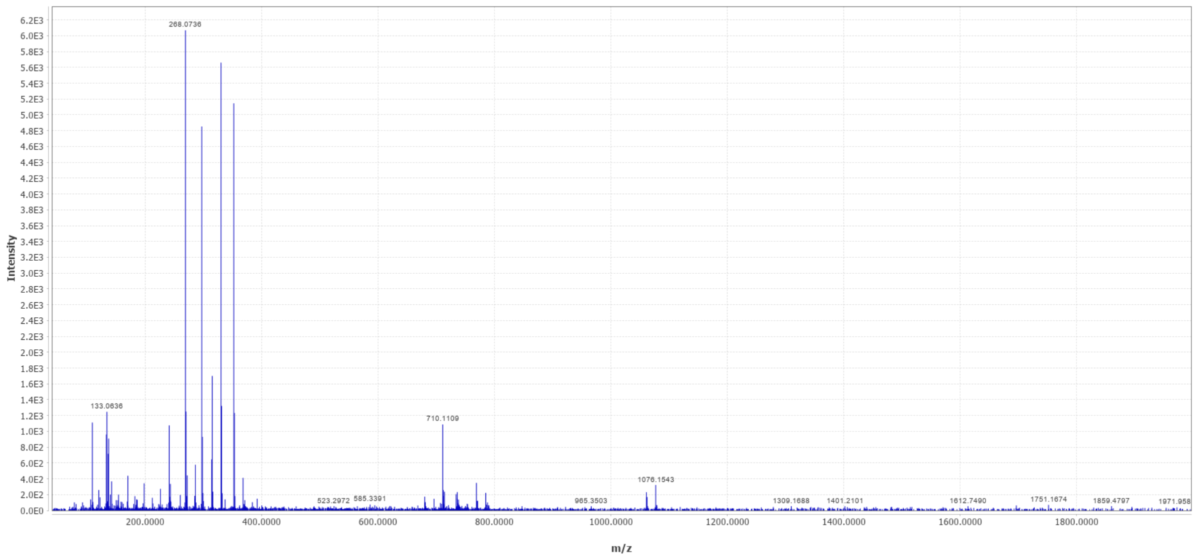

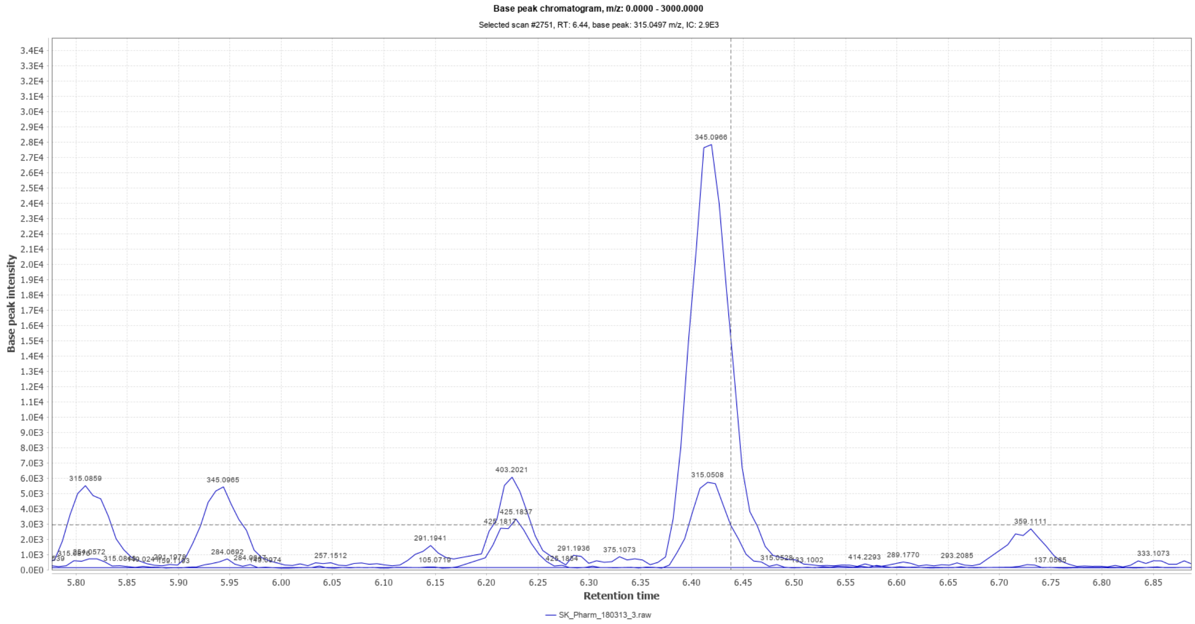
Base peak chromatogram, m/z: 0.0000 - 3000.0000
Selected scan #2751, RT: 6.44, base peak: 315.0497 m/z, IC: 2.9E3
Base peak intensity
Retention time
345.0966
315.0859
345.0965
403.2021
425.1837
315.0508
291.1941
359.1111
SK_Pharm_180313_3.raw

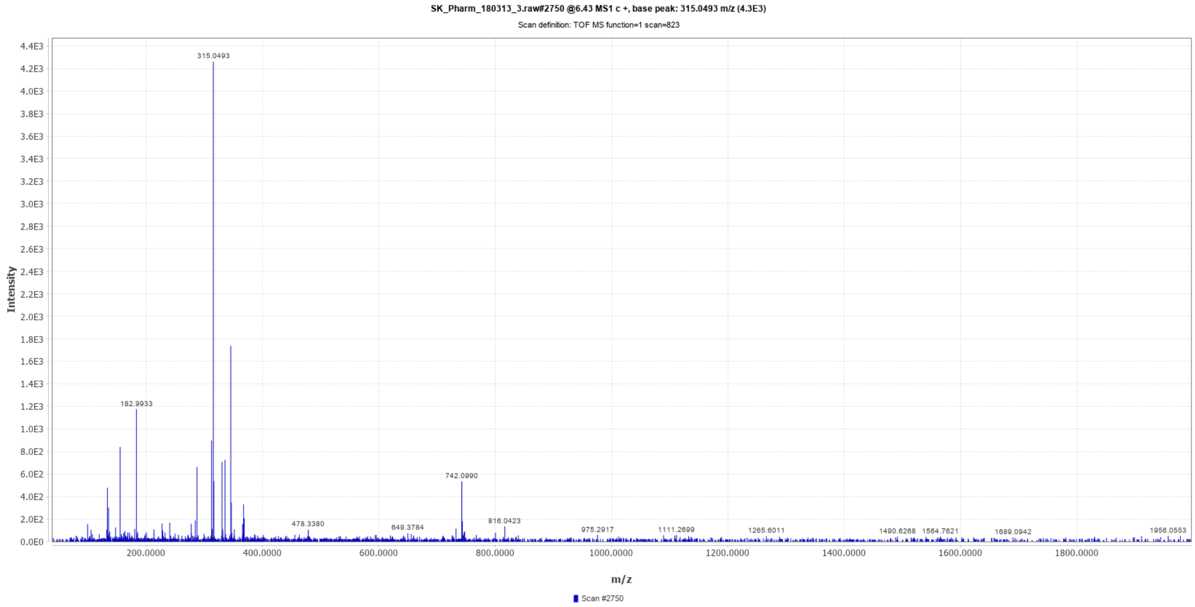
SK_Pharm_180313_3.raw#2750 @6.43 MS1 c +, base peak: 315.0493 m/z (4.3E3)
Scan definition: TOF MS function=1 scan=823
Intensity
315.0493
182.9933
742.0990
478.3380
649.3784
816.0423
975.2917
1111.2699
1265.6011
1490.6268
1564.7621
1689.0942
1956.0553
m/z
Scan #2750

Base peak chromatogram, m/z: 0.0000 - 3000.0000

Selected scan #2844, RT: 7.15, base peak: 467.1935 m/z, IC: 8.2E3

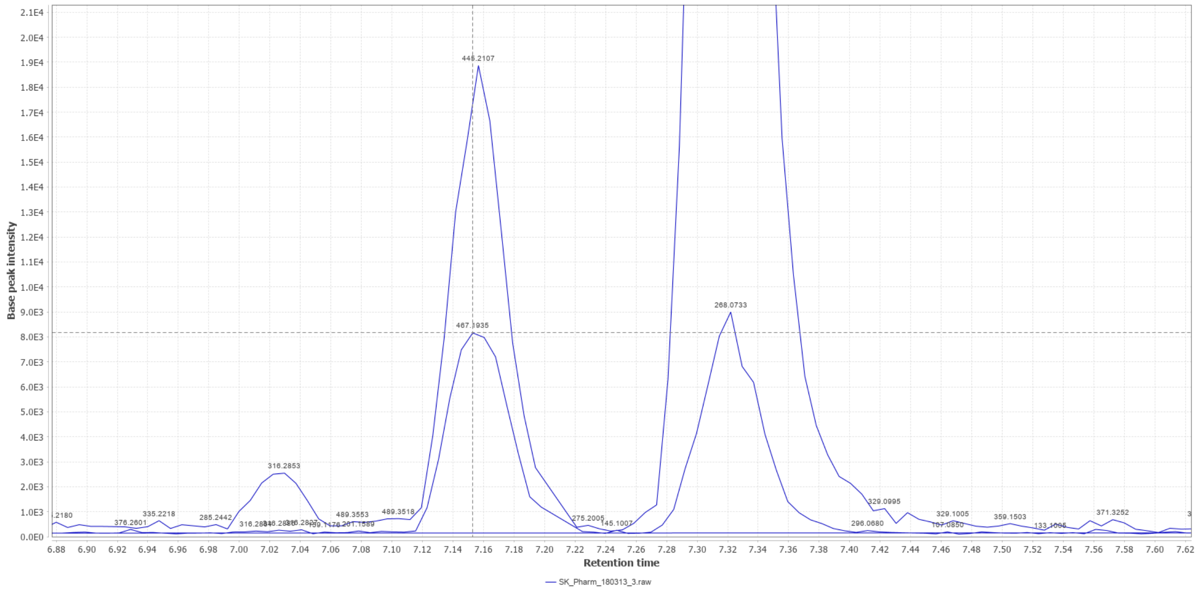

SK_Pharm_180313_3.raw#2844 @7.15 MS1 c +, base peak: 467.1935 m/z (8.2E3)

Scan definition: TOF MS function=1 scan=917

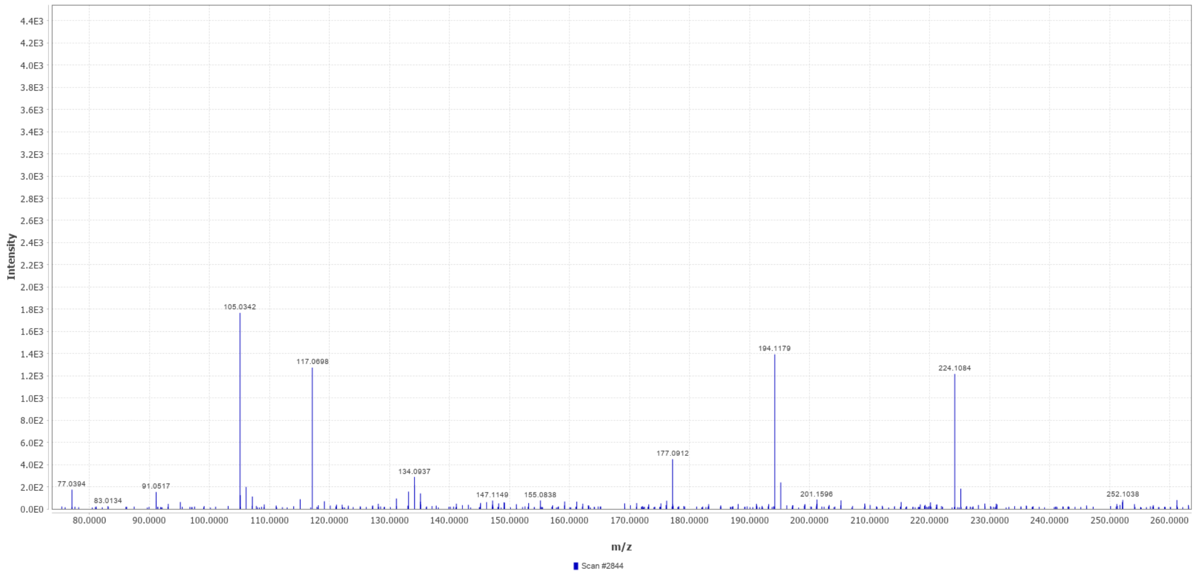

Base peak chromatogram, m/z: 0.0000 - 3000.0000

Selected scan #1366, RT: 10.64, base peak: 398.2327 m/z, IC: 8.2E3

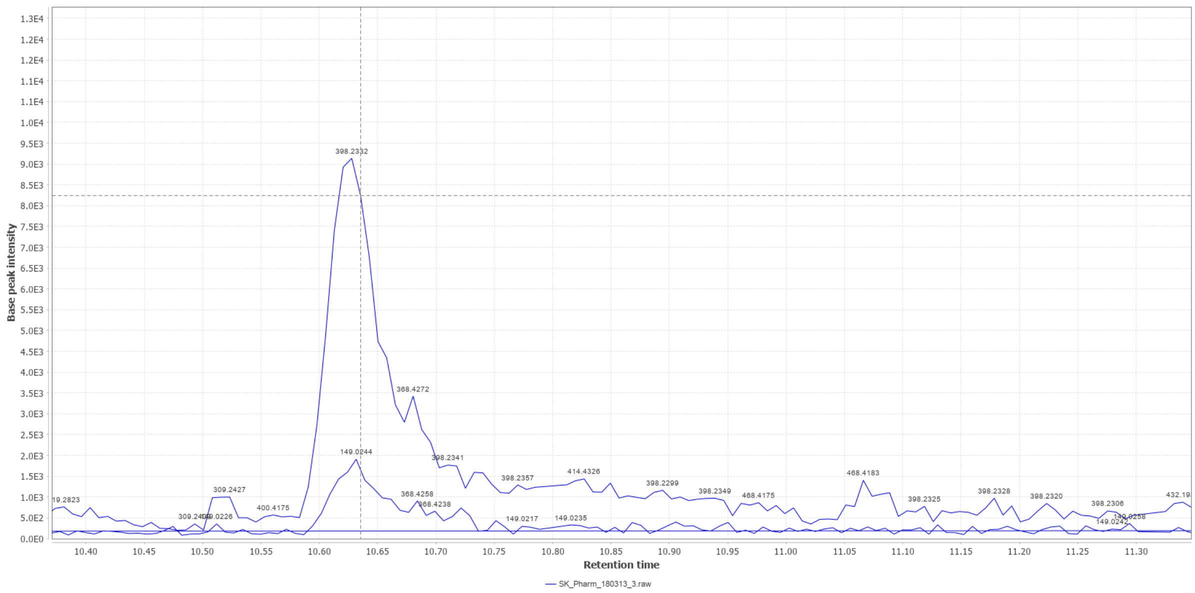

SK_Pharm_180313_3.raw#3287 @10.60 MS1 c +, base peak: 149.0243 m/z (6.1E2)

Scan definition: TOF MS function=1 scan=1360

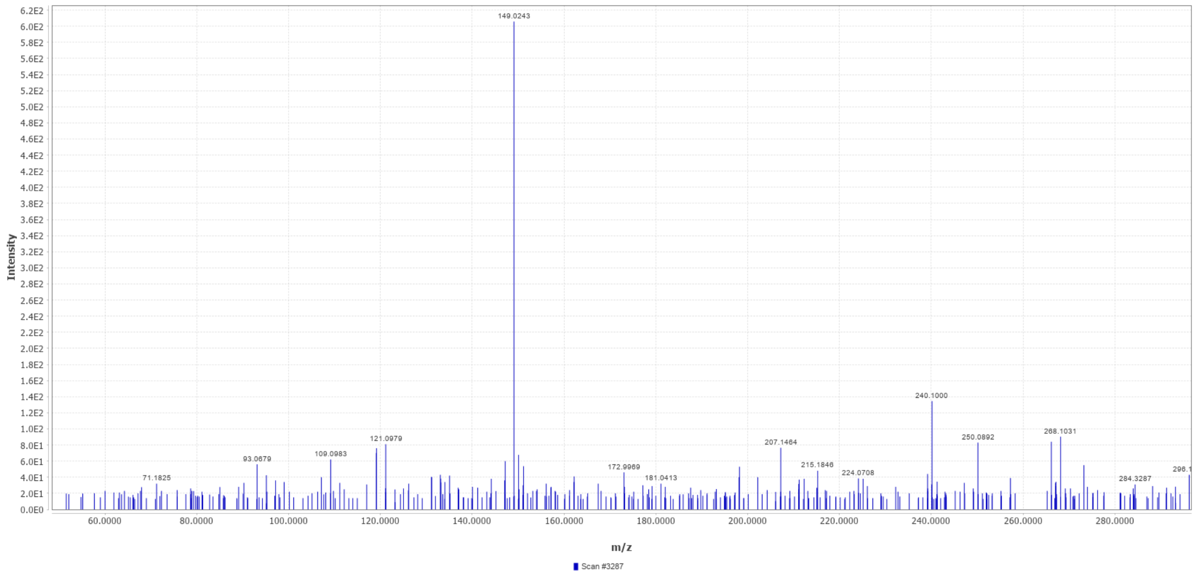

Supplement: Supplementary file 1 [file DataSheet_1.pdf]
